# A network-based, integrative study to identify core biological pathways that drive breast cancer clinical subtypes

**DOI:** 10.1038/bjc.2011.584

**Published:** 2012-02-16

**Authors:** B Dutta, L Pusztai, Y Qi, F André, V Lazar, G Bianchini, N Ueno, R Agarwal, B Wang, C Y Shiang, G N Hortobagyi, G B Mills, W F Symmans, G Balázsi

**Affiliations:** 1 Department of Systems Biology – Unit 950, The University of Texas MD Anderson Cancer Center, 7435 Fannin Street, Houston, TX 77054, USA; 2 Department of Breast Medical Oncology – Unit 1354, The University of Texas MD Anderson Cancer Center, PO Box 301439, Houston, TX 77230-1439, USA; 3 Department of Bioinformatics, The University of Texas MD Anderson Cancer Center, Houston, TX 77054, USA; 4 Institute Gustave Roussy, Villejuif, France; 5 Department of Medical Oncology, Imperial College, London, UK; 6 Department of Pathology, The University of Texas MD Anderson Cancer Center, Houston, TX 77054, USA

**Keywords:** driver-network, data integration, network analysis, breast cancer subtype, triple-negative breast cancer

## Abstract

**Background:**

The rapid collection of diverse genome-scale data raises the urgent need to integrate and utilise these resources for biological discovery or biomedical applications. For example, diverse transcriptomic and gene copy number variation data are currently collected for various cancers, but relatively few current methods are capable to utilise the emerging information.

**Methods:**

We developed and tested a data-integration method to identify gene networks that drive the biology of breast cancer clinical subtypes. The method simultaneously overlays gene expression and gene copy number data on protein–protein interaction, transcriptional-regulatory and signalling networks by identifying coincident genomic and transcriptional disturbances in local network neighborhoods.

**Results:**

We identified distinct driver-networks for each of the three common clinical breast cancer subtypes: oestrogen receptor (ER)+, human epidermal growth factor receptor 2 (HER2)+, and triple receptor-negative breast cancers (TNBC) from patient and cell line data sets. Driver-networks inferred from independent datasets were significantly reproducible. We also confirmed the functional relevance of a subset of randomly selected driver-network members for TNBC in gene knockdown experiments *in vitro*. We found that TNBC driver-network members genes have increased functional specificity to TNBC cell lines and higher functional sensitivity compared with genes selected by differential expression alone.

**Conclusion:**

Clinical subtype-specific driver-networks identified through data integration are reproducible and functionally important.

Over the past 2 decades, innovative genome-scale data collection approaches have revolutionised biological research (Hawkins *et al*, 2010). The possibility to sequence entire genomes or to monitor transcripts, proteins, or their interactions at genome-scale is opening new avenues of knowledge generation. However, the emergence and rapid accumulation of different genome-scale data types also poses a challenge: how to integrate and analyse various types of data to further accelerate the rate of biological discovery and the development of biomedical applications (Veiga *et al*, 2010)? Currently, few methods exist that address this problem, but recent attempts to integrate genome-scale data with known molecular interactions have already begun generating promising new insights into the biology of cancer (Chuang *et al*, 2007; Pujana *et al*, 2007).

A major systems biology challenge in the context of cancer research is how to extract and combine information from genome-wide DNA copy number alteration (CNA) and gene expression data into biologically meaningful and experimentally testable models of cancer. Although CNA is generally expected to cause corresponding expression changes, only a fraction of genes in amplified or deleted chromosomal regions show differential expression as detected by current methods (Hyman *et al*, 2002; Chin *et al*, 2006; Neve *et al*, 2006; The Cancer Genome Atlas Network, 2008). Likewise, few differentially expressed genes (based on high-throughput expression profiling) show significant CNA (Hyman *et al*, 2002; Chin *et al*, 2006; Neve *et al*, 2006; The Cancer Genome Atlas Network, 2008). This incongruity between different types of data sets has driven the development of various data integration methods (Hyman *et al*, 2002; Pollack *et al*, 2002; Garraway *et al*, 2005; Adler *et al*, 2006; Berger *et al*, 2006; Salari *et al*, 2010), aimed to differentiate between ‘driver’ and ‘passenger’ genes based on the assumption that functionally important driver genes must have simultaneous CNA and differential expression. However, this assumption might be neither necessary nor sufficient for defining driver genes. In spite of simultaneous CNA and differential expression, a gene might still be functionally unimportant if it does not affect the expression or biological activity of its interacting partner genes. Conversely, an amplified or deleted gene that is upregulated or downregulated only minimally, in a statistically non-significant manner in patient data can still function as a driver gene if it alters the expression of its network neighbourhood.

A recent study (Akavia *et al*, 2010) proposed a Bayesian approach for finding driver gene modules in melanoma and experimentally validated two predicted candidates. However, this method did not take advantage of the existing biological knowledge in the form of bio-molecular networks. Existing knowledge compiled as interactome networks can help to provide a validated basis in the search for biologically relevant driver genes. Moreover, network-based methods reveal links between biologically important genes, highlighting pathways and mechanisms that can become targets for therapy.

Here, we propose a method for integrating CNA and gene expression data in the context of large-scale signalling, protein–DNA (Matys *et al*, 2006), and protein–protein interaction (PPI) networks that have become recently available (Peri *et al*, 2004). Previous work has shown the utility of network-based gene expression data analysis in identifying disease signatures (Chuang *et al*, 2007; Cline *et al*, 2007; Pujana *et al*, 2007; Ideker and Sharan, 2008; Taylor *et al*, 2009; Nibbe *et al*, 2010). However, most network-based methods so far have focused on a single type of high-throughput data, instead of integrating multiple data types with network information. Most of these network-based approaches have either used differential expression or correlation between gene pairs. Although both of these characteristics are useful in elucidating disease networks in the context of breast cancer (Chuang *et al*, 2007; Taylor *et al*, 2009), no method has attempted to combine them.

Breast cancer patients can be clinically classified into three distinct subtypes based on oestrogen (ER)-, progesterone (PR)- and HER-2-receptor assessment (Onitilo *et al*, 2009) including: (i) ER-positive and human epidermal growth factor receptor 2 (HER2)-normal (ER+), (ii) HER2-positve regardless of ER (HER2+), and (iii) triple receptor-negative breast cancers (TNBC). These subsets have distinct clinical behaviours and require different treatment approaches. Gene expression analysis (Perou *et al*, 2000; Sotiriou and Pusztai, 2009) and comparative genomic hybridisation (CGH) studies (Bergamaschi *et al*, 2006; Andre *et al*, 2009) suggest that distinct biological pathways drive the biology of these different breast cancer subtypes. Although network-based methods have been applied to analyse breast cancer gene-expression data (Chuang *et al*, 2007; Pujana *et al*, 2007; Taylor *et al*, 2009), clinical subtype-specific driver-networks have never been defined. We applied our novel network-based data integration method to two independent breast cancer patient data sets (Chin *et al*, 2006; Andre *et al*, 2009) and one breast cancer cell line data set (Neve *et al*, 2006) to identify distinct driver-networks corresponding to each subtype. This network analysis revealed important differences in the biological processes that distinguish these 3 different types of breast cancers and also suggested potential new therapeutic strategies. Moreover, we experimentally validated the functional relevance of several novel genes that our analysis implicated as potentially important in the biology of TNBC using siRNA techniques in 13 different breast cancer cell lines.

## Materials and methods

### Data sets and sample classification

The network-based data integration method requires gene expression and DNA copy number data collected from the same breast cancer subtype. We obtained such data from two published papers that included 103 and 78 clinically annotated breast cancers, respectively (Chin *et al*, 2006; Andre *et al*, 2009). Patient samples were classified into three mutually exclusive groups of ER+ or HER2+ or TNBC based on routine clinical histopathology information. Numbers of samples in these three subtypes from each of the two data sets are included in Supplementary Table S1. Patients with HER2 protein overexpression by immunohistochemistry or HER2 gene amplification by fluorescent *in situ* hybridisation were assigned to the HER2+ group regardless of their ER/PR status. Patients with HER2-normal cancer with >10% ER or PR expression using immunohistochemistry were considered as ER+. The remaining patients with HER2-normal and also ER- and PR-negative disease were categorised as TNBC. In addition, we obtained cell line data from Neve *et al* (2006). We used similar criteria to assign breast cancer cell lines to three clinical subtypes based on information provided in Neve *et al* (2006). Human epidermal growth factor receptor 2 positive cell lines, irrespective of their ER/PR status were assigned to the HER2+ subtype. ER/PR positive and HER2-negative cell lines were assigned to the ER+ subtype. The remaining cell lines were assigned to the TNBC subtype. See the Supporting Information (SI) Appendix for the analysis of DNA copy number and gene expression data.

### Construction of the literature-based network space and network visualisation

The PPI network was downloaded from the HPRD database (www.hprd.org). The transcription factor (TF) network was assembled based on the ORegAnno and TRANSFAC databases (http://www.biobase-international.com/pages/index.php?id=transfac). Signalling networks were assembled from the KEGG database (www.kegg.com). All three types of networks (HPRD, TF, and KEGG) were assembled into a single combined network space, which was used for all analyses. In the combined network space, signalling and transcription-regulatory network connections were directed while PPIs were undirected. We used Cytoscape (www.cytoscape.org) for network visualisation.

### Seed gene selection

First, we calculated a discretised CNA score matrix, where rows and columns represent genes and samples, respectively. Elements of this matrix can have three different values, −1, 0, and 1, based on whether the gene is deleted, not changed, or amplified, respectively. Then, for each gene, we calculated the subtype-specific average CNA score.

Next, we calculated the correlation of this CNA score and gene expression for genes that had both expression and amplification data, pooling all subtypes together. For each subtype, we selected seed genes from the top 5% based on average CNA score and top 10% based on CNA score and expression correlation. Although we calculated a subtype-specific CNA score, for calculating CNA-expression correlation we used all the samples. We used this strategy because, if a gene is amplified in all samples of a single subtype, the CNA score across all these samples will be 1. Hence, correlation is not informative of subtype-specificity when one of the variables has a constant value. However, genes amplified as well as over-expressed in a subtype-specific manner are good candidates as seed genes. Expression-CNA correlation calculated for these genes should be higher if samples from all subtypes are pooled, as opposed to when samples from only a specific subtype are used. On the other hand, the reverse is not true: even if the correlation between CNA and expression is strong across all samples, subtype-specific correlation might still be quite low. Average CNA score and CNA-expression correlations are provided in Supplementary Table S2.

We compared the distribution of expression-CNA correlations for the three different data sets (Chin *et al*, 2006; Neve *et al*, 2006; Andre *et al*, 2009) (Supplementary Figure S1). Among the two patient data sets the correlation distribution was considerably higher for the Andre *et al* (2009) data set. One possible reason for this may be that the arrays used for measuring CNA had higher resolution, hence producing better data quality. Interestingly, for cell lines, the correlation was markedly improved compared with patient data sets. Hence, to select seed genes for patient and cell line driver-networks, we used the Andre *et al* (2009) and Neve *et al* (2006) data sets, respectively. In the Supplementary Information (SI) we have also included the driver-networks using seed genes from the Chin *et al* (2006) data set.

### Driver-network identification

Starting from a seed gene, we identified all of its direct neighbours in the network space (i.e., directly connected to the seed gene) and tested for (i) differential expression or (ii) differential co-expression. If a gene passed either one of these tests, it was included in the driver-network. The same process was carried out iteratively for all the new neighbours in the growing network until there was no new gene that met either inclusion criterion.

(i) For differential expression we used the following steps. First, a two-tailed *t*-test (combined with a Bonferroni correction for multiple testing) was carried out for each probe of the gene expression data to determine whether the expression of an immediate neighbour was significantly higher in the patient subset of interest compared with the rest of the patient groups. Probes were ranked in ascending order of *P*-values from the *t*-test and 2% of the probes with the lowest *P*-values were selected. To account for probes that did not pass the *t*-test due to high intra group standard deviation, we also selected probes based on median expression difference between the group of interest and the reference group. Probes were ranked in descending order based on median expression difference and the top 1% of the probes was selected. Finally, we calculated the union of the two aforementioned probe sets and converted them to corresponding gene IDs. We followed these steps for each of the data sets and for each clinical subtype.

(ii) The co-expression was calculated between each differentially expressed gene in the expanding network and each of its first neighbours. Therefore, correlations between a seed gene and its first neighbours were considered only if the seed gene was differentially expressed. For differential correlation, we calculated two Pearson correlations for each of the links, that is, the adjacent genes in the network space. One correlation was based on samples that belonged to the subtype under investigation. The other correlation was based on the rest of the samples (not belonging to the subtype). Finally, the latter was subtracted from the former and the top 0.2% of the links from the distribution of differential correlations were selected as significantly correlated. When multiple probes were present in the microarray corresponding to a gene, we selected the probe with the highest absolute correlation. To ensure comparability of the results, we used exactly the same criteria for network expansion for all data sets and for all clinical subtypes.

The driver-networks for each of the three different clinical subtypes identified through this process from all three data sets (two patient data sets and one cell line data set) are listed in Supplementary Table S3.

### Calculation of overlap

If A and B represent sets of driver-network members from different clinical data sets, we calculate the normalised overlap using the formula: 
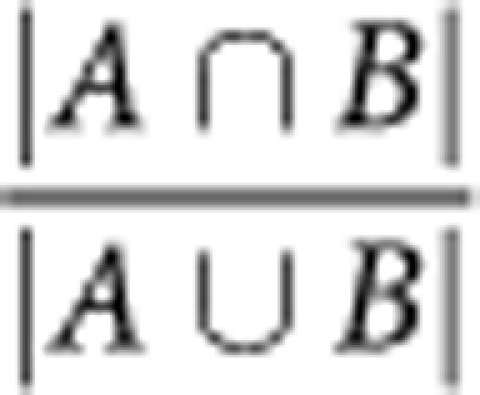
 where ∣*A*∣ denotes the cardinality (number of elements) of set *A*.

### siRNA validation

We selected 30 genes from the TNBC driver-networks for functional validation that were not previously studied extensively in the context of TNBC (Figure 2C, F and Supplementary Figure 3C). We also selected 10 genes differentially expressed in TNBC in all three data sets. Each of the 40 genes in the joint pool was targeted with four distinct siRNAs obtained from Dharmacon Inc. We performed siRNA screening for each gene in 12 different breast cancer cell lines (TNBC=9, non-TNBC=4). See the SI for the detailed description of the validation experiments. siRNA screen data for 40 genes (four siRNAs corresponding to each gene and three replicates for each siRNA) were provided in Supplementary Table S4.

## Results

### A computational method for network-based analysis of CNA and gene expression data

**Figure 1:**
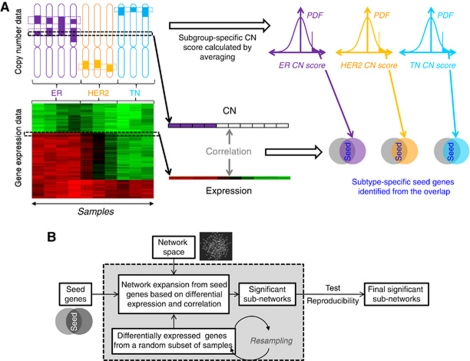
Overview of data analysis strategy and driver-network identification. (**A**) Clinical subtype-specific seed gene selection: Genes that were frequently amplified and showed strong correlation between amplification and mRNA expression were selected as seed genes. (**B**) Driver-network construction: starting with a seed gene, all of its nearest neighbours in an *a priori* assembled signalling, PPI and TF network space are considered for inclusion provided that at least one of two criteria (differential expression and co-expression) is satisfied (see Materials and Methods). Once the first neighbours of the seed gene are included, their neighbours are similarly tested in an iterative process to further expand the driver-network. We used extensive re-sampling, where a subset of the patients/cell lines were randomly selected to construct the driver-network samples. Genes that appeared in at least 50% of the driver-network samples were included in the final driver-network.

First, we assembled the biological network space for this analysis by combining PPI with transcription-regulatory and signalling interactions from curated databases including a total of 12 358 genes (Materials and Methods). Next, we developed an algorithm to identify breast cancer subtype-specific driver-networks by integrating two different genome-scale data types (mRNA expression and CGH data) on this *a priori* defined network space (Figure 1). These driver-networks were anchored to ‘seed’ genes (Figure 1A) defined by significant DNA copy number elevation in a given cancer subtype along with strong correlation between gene copy number and the corresponding mRNA expression (Materials and Methods). Starting from these seed genes, the algorithm searched their immediate network neighbourhood and tested if any of their neighbours were significantly (i) differentially overexpressed (i.e., expression in the subtype studied is significantly higher compared with the other subtypes) or (ii) differentially co-expressed (i.e., expression correlation between genes connected by an edge is significantly higher in the subtype compared with the other subtypes) in the gene expression data. We selected candidate genes to be tested for differential co-expression based on the criterion that they had to be nearest neighbours of a current network member that was differentially expressed. Thus, co-expression was calculated between each differentially expressed gene in the expanding network and each of its first neighbours. If differential expression or co-expression was observed, the seed and its neighbour(s) were included into the driver-network and the process was repeated within the new, expanding network neighbourhood (Figure 1B). This approach was based on two hypotheses: (i) that significant CNA of a potential seed gene should also be reflected in the expression of the corresponding mRNA; and (ii) that seed genes surrounded by genes with significantly increased expression or expression-correlation are more likely to represent biological driver functions than genes that do not affect their network neighbourhood.

To ensure that our results are not hijacked by a few outlier samples, we used extensive re-sampling analysis. Starting from the same seed genes, we randomly selected and used 80% of the samples for network expansion. This random sampling was repeated 1000 times and only those genes that appeared in at least 50% of the driver-network samples were included in the final driver-network (Figure 1B).

### Discovery of driver-networks associated with three major breast cancer subtypes

**Figure 2:**
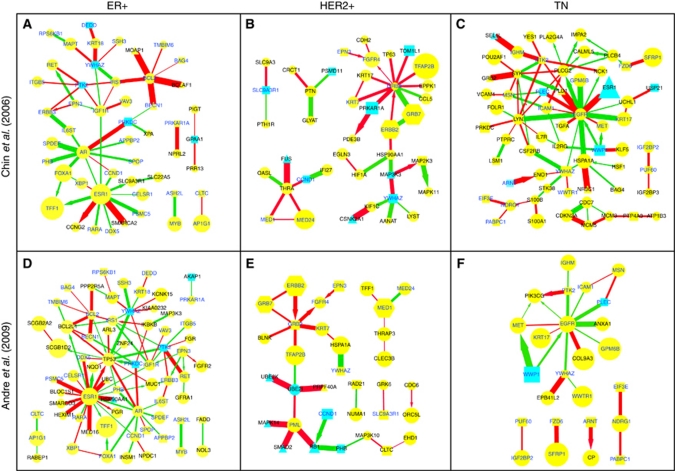
Breast cancer subtype-specific (ER+: **A** and **D**, HER2+: **B** and **E**, TNBC: **C** and **F**) driver-networks in two separate data sets (Chin *et al.* (2009): **A–C**, Andre *et al.* (2006): **D–F**). The size of each node is proportional to the differential expression level of the corresponding gene. Yellow and blue nodes represent upregulation and downregulation, respectively, from gene expression data. The shapes indicate the type of genomic change, squares representing the seed genes with copy number alterations, circles representing differential expression without copy number alteration, hexagons representing both copy number and mRNA expression changes, and triangles representing inclusion based on differential co-expression without differential expression. Downregulated genes were included in the network either as seed genes (square nodes) or based on differential positive or negative co-expression (triangle nodes), as only significant overexpression was used for network expansion. The width and colour of an edge connecting two nodes reflect the magnitude and sign of the correlation (red: positive; green: negative) between two genes within the driver-network. An arrow pointing from one member gene to another indicates a transcriptional or signalling relationship, whereas lines represent PPIs. Genes common between the two driver-networks have blue font colour.

We assessed the reproducibility of disease subtype-specific driver-networks by applying our method to two independent data sets (*n*=103 and *n*=78) of mRNA expression and DNA copy number data collected from each breast cancer patient from two different cohorts (Chin *et al*, 2006; Andre *et al*, 2009). The copy number data associated with the Chin *et al* (2006) data set was less recent and had lower resolution. We observed relatively poor correlation between gene expression and amplification in this data set, lowering the number of seed genes. Hence, here we present driver-networks expanded from seed genes from the Andre *et al* (2009) data set for both patient data sets, whereas driver-networks inferred using seed genes from Chin *et al* (2006) are shown in the Supplementary Figure S2. Figure 2 shows driver-networks identified for each breast cancer subtype in the two different patient cohorts. In general, a few highly connected ‘hub’ genes, some with known subtype-specific involvement appear to hold together components of these networks for both data sets, suggesting their central roles in these subtypes. Most of these subtype-specific hubs have no significant CNA, and are reached only through network expansion, suggesting that chromosomal aberrations can drive cancers by indirectly manipulating hub gene expression through various regulatory interactions, such as transcriptional regulation and protein modification. In addition to the main network components containing the hubs, we also observed a few satellite subnetworks without any links to the main component. Below, we discuss the membership of these driver-networks in some detail.

#### Genes from ER+ driver-networks

In ER+ breast cancers, for both cohorts the *ESR1* gene encoding ER alpha (ER*α*) was the most prominent hub of a large interconnected network, consistent with its central role in this cancer subtype. The ER*α* neighbourhood included many known transcriptional targets of ER*α* such as *TFF1* (Lacroix and Leclercq, 2004; Tozlu *et al*, 2006), *BCL2L1*, *NQO1*, *CCNG2, TP53*, *PGR*, and *SLC9A3R1*. Several other genes not directly transcriptionally regulated by ER*α*, but frequently overexpressed in ER+ breast cancer (Bhargava *et al*, 1994; Dressman *et al*, 2001; Lacroix and Leclercq, 2004; Laganiere *et al*, 2005; Chin *et al*, 2006; Maor *et al*, 2006; Tozlu *et al*, 2006; Hua *et al*, 2009) surrounded ER*α* in the driver-network, including androgen receptor (*AR)*, insulin-like growth factor 1 receptor (*IGF1R)* (Hartog *et al*, 2011), *RARA*, *XBP1*, *CCND1* (Dressman *et al*, 2001; Tozlu *et al*, 2006), and *CELSR1* (Figure 2A and D). Besides ER*α*, *AR*, *IGF1R* (Hartog *et al*, 2011), *IRS1* (Migliaccio *et al*, 2009), *YWHAZ,* and *BCL2* were additional hubs connecting multiple significantly over-expressed genes. These hub nodes appear to act as signal integrators and may have a key role besides *ESR1* in the developing ER+ breast cancer. *AR* and ER*α* are co-expressed in most of the breast tumours and *AR* is emerging as an independent prognostic factor in ER+ breast cancer (Peters *et al*, 2009). Previous studies have also indicated positive correlation between *IGF1R* and *ESR1* expression (Hartog *et al*, 2011). *IGF1R* phosphorylates *IRS1* (Insulin receptor substrate-1) and recruits its downstream effectors (Migliaccio *et al*, 2009). Moreover, *IRS1* can translocate to the nucleus and can modulate the transcriptional activity of ER*α* (Migliaccio *et al*, 2009). Finally, *IRS1* shows positive association with ER+ breast cancer in both data sets analysed here as well as in other studies (Migliaccio *et al*, 2009).

#### Genes involved in apoptosis and autophagy appear in ER+ driver-networks

We used both differential expression and differential co-expression for network expansion. In addition to the genes identified based on their differential expression, *BCLAF1*, *BECN1*, *MOAP1*, *SMARCA2*, *HSP90AA1*, *UBC*, and *MED16* were included in the driver-network based on differential co-expression only. These genes have significantly correlated expression with either *ESR1* or *BCL2* in the ER+ breast cancer subtype compared with other subtypes, but without differential expression. The protein Bcl-2 encoded by *BCL2* promotes tumourigenesis by inhibiting apoptosis, the basic mechanism of programmed cell death (Tabuchi *et al*, 2009). In breast cancer, Bcl-2 levels correlate with ER-positivity, as the expression of *BCL2* is transcriptionally regulated by ER*α* (Tabuchi *et al*, 2009; Subhawong *et al*, 2010). Two other members of the ER+ driver-network, *BCLAF1* and *MOAP1*, interact with several Bcl-2 family members and have important roles in regulating apoptosis. *BCN1* encodes Beclin1, which is differentially co-expressed with Bcl-2 in our ER+ driver-network and has a key role in autophagy, a mechanism used by cancer cells to survive under stressful conditions (John *et al*, 2008). The function of Beclin1 is defined by its interactions with Bcl-2 and its family members (John *et al*, 2008). Interestingly, these potentially important connections with apoptosis and autophagy in ER+ breast cancer are revealed by co-expression rather than differential expression.

#### Genes from HER2+ driver-networks

HER2+ cancers are frequently associated with amplification and over-expression of the *ERBB2* and *GRB7* genes, both from the 17q12–21 amplicon. These two genes were present in the driver-networks obtained from our analysis (Figure 2B and E). Multiple other genes from the driver subnetworks, including *GRB2*, *MED1*, *MED24*, and *EPN3* are also located in the proximity of the same amplicon (between 17q12–25), underscoring the significance of this chromosomal region in HER2+ breast cancer. Two other genes, *TFAP2B* and *FGFR4*, were present in driver-networks from both data sets. These two genes are differentially expressed without amplification and they are related to HER2+ breast cancer. *TFAP2B* is an AP-2 family TF that can regulate cell proliferation. AP-2 TFs can induce *HER2* protein expression even without gene amplification (Bosher *et al*, 1995). Although the *ERBB2* gene is frequently amplified in HER2+ breast cancer, upregulation of the *TFAP2B* gene provides an additional mechanism for over-expression of the ErbB2/HER2 protein. Regarding *FGFR4*, a previous study (Koziczak and Hynes, 2004) has shown that it is over-expressed in a significant fraction of HER2+ breast cancer samples and simultaneous inhibition of *FGFR4* and *ERBB2* using small molecule inhibitors have synergistic anti-proliferative effect. These observations provide important clues about pathway deregulation in HER2+ breast cancer and potential combinatorial therapies. However, as the number of samples in the HER2+ subtype (7 and 11 samples, see Supplementary Table S1) was much smaller compared with other subtypes (51 and 55 ER+ samples; 20 and 37 TNBC samples), our findings might be less statistically robust. This is also reflected in the lower overlap between the two independent data sets (Table 1). Larger data sets may enable the identification of more robust driver-networks.

#### Genes from TNBC driver-networks

TNBC is the focus of intense research because the key biological drivers behind these highly proliferative and poor-prognosis cancers remain unknown. Unlike the ER+ or HER2+ breast cancer subtypes, pathway deregulation is least understood in TNBC, hence no targeted therapies exist for these patients. Driver-networks from our analysis may shed light on the underlying biology of the disease and suggest new therapies. We identified *EGFR* (Corkery *et al*, 2009) as the central hub in both data sets, which was connected to *GPM6B*, *ICAM1*, *PTK2*, *MET*, *KRT17*, *ANXA1*, *COL9A3*, *NCK1*, *HSPA1A*, *TGF1*, *YWHAZ*, *LYN*, *PLEC*, *PLD1*, *PLCG2*, and *PIK3CG* (Figure 2C and F). In the Chin *et al* (2006) dataset, several other genes including *LYN*, *PLCG2*, *MSN*, *SYK*, and *ICAM1* were identified as hub genes. However, these other genes were not hubs in the Andre *et al* (2009) data set, possibly because the driver-network itself was much smaller. Multiple genes from our driver-networks, including *EGFR* (Hochgrafe *et al*, 2010), *MET* (Hochgrafe *et al*, 2010), *LYN* (Hochgrafe *et al*, 2010), *PTK2/FAK* (Hochgrafe *et al*, 2010), *KRT17* (Sharp *et al*, 2008), *ANXA1* (Sharp *et al*, 2008), and *NDRG1* (Sharp *et al*, 2008) were previously associated with TNBC. Although the *ESR1* gene is under-expressed in the TNBC subtype compared with other subtypes, expression of this gene is strongly correlated with *EGFR* in this subtype. As the *ESR1* gene is believed to have an important role in ER+ breast cancer, but not in TNBC, its strong co-expression with the central *EGFR* gene, specifically in TNBC compared with other clinical subtypes, is rather counter intuitive. We were able to identify this intriguing relationship, because we used differential co-expression as one of the criteria for network expansion.

#### Epithelial-mesenchymal transition (EMT)-related genes are enriched in TNBC driver-networks

Through EMT cancer cells lose their epithelial characteristic and transform to mesenchymal morphology, which causes the invasion of other tissues and later, distant metastasis (Mani *et al*, 2008). Recent studies have shown that EMT signatures are predominant in ‘basal like’ breast cancers, which is a subtype similar to TNBC (Sarrio *et al*, 2008). The TNBC driver-networks from both data sets are enriched in EMT-related genes, including *LYN*, *SYK*, *FAK/PTK2*, *MSN*, *WWTR1*, *YWHAZ*, *ICAM1*, *PLEC*, and *NDRG1* (Sharp *et al*, 2008; Hochgrafe *et al*, 2010). The concordant over-expression of *SFRP1* (a secreted protein that is antagonistic with Wnt) and amplification of *FZD6* (a Wnt receptor) suggests a functional role for Wnt signalling in TNBC that has not previously been appreciated. SFRP1 also has an important role in mediating TGF-*β* signalling (Gauger *et al*, 2011). Both Wnt and TGF-*β* signalling have important roles in EMT (Xu *et al*, 2009; Gauger *et al*, 2011). The enrichment of EMT genes in driver-networks appears specific to the TNBC clinical subtype. We could not estimate the statistical significance of this enrichment, as there is no database for a reliable and comprehensive gene set corresponding to breast cancer EMT, to the best of our knowledge.

Albeit *EGFR* is the major hub gene in the TNBC driver-networks, attempts to inhibit *EGFR* alone yielded disappointing results in the clinic (Corkery *et al*, 2009). Our results suggest that concordant inhibition of the EGFR and EMT pathways might be interesting to consider in the future as a new strategy for treating TNBC.

### Driver-networks are significantly reproducible

#### The overlap between driver-networks identified from the two data sets is statistically significant

As we are comparing two similar but independently acquired data sets, true and biologically relevant results should be reproducible across data sets. To test whether the driver-networks are significantly reproducible, we examined the overlap between subtype-specific driver-networks in the two data sets. As the overlap will depend on the sizes of both driver-networks, it should be properly normalised. We computed the normalised overlap by dividing the number of genes in the intersection of driver-networks with the number of genes in their union (Materials and Methods). Using the same formula, we also calculated the normalised overlap of those genes that were differentially expressed in at least 50% of the 1000 random samplings used to infer driver-networks (Supplementary Table S5), and applied it for control as follows.

To estimate the statistical significance of the reproducibility of driver-networks, we compared the observed results with the results obtained from the 1000 randomisations (background distribution). We used two different types of randomisation for calculating the background distribution, based on (i) seed randomisation and (ii) expression randomisation. In seed randomisation, we randomly selected the same number of seed genes as for constructing the observed driver-networks. Similar to driver-network construction, we used exactly the same random seeds for both data sets, whereas using the original gene expression data for network expansion. In expression randomisation, we used the original seed genes in both data sets, but the gene names were shuffled in both gene expression data sets. This ensures using the same numbers of differentially expressed genes as for the original network expansion.

For each of the 1000 seed and expression randomisations, we calculated the overlap between driver-networks obtained from the two data sets. As the number of overlapping genes between the networks will depend on the size of the individual networks, we again normalised the overlap by the union of the two networks. The distribution of this normalised overlap from randomisation was compared with the normalised overlap observed from our analysis to calculate the significance of the observed overlap. The significance levels (*P*-values) for each of the three subtypes are listed in Table 1. Driver-networks from all three subtypes show statistically significant overlap based on expression randomisation (*P*<0.05). From seed randomisation, the ER+ and TN subtypes show significant overlap (*P*<0.05). However, the *P*-value for the HER2+ subtype was relatively high (0.08). As mentioned before, the lack of robustness might have been caused by the fewer samples available for this subtype.

#### The advantage of data integration

Some of the most important members of the driver-networks were identifiable through the network expansion process involving data integration, and could not have been found by more simplistic approaches. For example, *ESR1* and many of its interacting partners in the driver-network (e.g., *TFF1*, *XBP1*, *IGF1R*, *AR*, *CELSR1*, and *RARA*) are significantly over-expressed without amplification. Hence, data integration algorithms requiring simultaneous CNA and differential expression as selection criteria would not have identified these genes as important. Likewise, several seed genes for the ER network, including *APPBP2*, *ASH2L*, *BAG4*, *CCND1*, *CLTC*, *DDX5*, *DEDD*, *EPN3*, *GPAA1*, *PHB*, *PRKAR1A*, *PRKDC*, *PSMC5*, *PTK2*, *RPS6KB1*, *SPOP*, and *YWHAZ* were not significantly over- or under-expressed in ER+ cancers relative to other subtypes but showed copy number abnormalities. These genes would have been missed by transcriptional analysis alone or without considering network interactions. Several of these genes were previously implicated in the biology of ER+ cancers, further supporting the validity of our method (Emberley *et al*, 2002; Song and Santen, 2006; Celebiler Cavusoglu *et al*, 2009; Chen *et al*, 2009). Also, most genes from TNBC driver-networks could not have been found without simultaneously projecting the expression and copy number data onto a comprehensive network space. In fact, none of the genes from driver-networks are simultaneously over-expressed and amplified in the TNBC driver-networks.

### Driver-networks identified using cancer cell line data

Cancer cell lines are widely used to study cancer biology and to test potential new drugs. Therefore, it is important to establish to what extent biological networks that drive human cancers are preserved in cell lines. We applied our network finding algorithm to gene expression and copy number data of 40 breast cancer cell lines (Neve *et al*, 2006) that we assigned to the ER+, HER2+, and TNBC groups (Supplementary Table S1). This is the largest cell line data set publicly available with simultaneously collected gene copy number and gene expression data.

#### Comparing the membership of driver-networks inferred from cell lines and patient data

For ER+ cells, *ESR1* was again in the centre of a network that included many members also identified in the patient data-derived networks (Supplementary Figure S2A). Overall, 11 member genes from the cell line driver-networks were also present in at least one of the two patient ER+ networks including *TFF1*, *XBP1*, *FOXA1*, *MUC1*, *ERBB3*, *PSMC5*, *SLC9A3R1*, *FADD*, *MYB*, and *ASH2L* (Supplementary Figure S3A). However, several potentially therapeutically important driver-network members in the patient data sets (e.g., *AR*, *IRS1*, *IGF1R*, *VAV3*, *ERBB3*, and *AP1G1*) were not present in the cell line-derived network. For the HER2+ cell lines, *ERBB2*, *GRB7*, *GRB2*, *MED1*, and *MED24* remained central nodes (Supplementary Figure S3B). Similar to patient cohorts, the number of cell lines from the HER2+ subtype was much smaller compared with other subtypes. For the TNBC cells, we also found considerable but not complete overlap of driver-network members between patient data and cell lines (Supplementary Figure S3C). Both *EGFR* (Hochgrafe *et al*, 2010) and *LYN* (Hochgrafe *et al*, 2010) were again identified as important hub genes and several other genes including *ANXA1* (Sharp *et al*, 2008), *MET* (Hochgrafe *et al*, 2010), and *MSN* were found significant in the TNBC cell line data set. *FN1* (Camara and Jarai, 2010) (fibronectin 1) and *CD44* (Brown *et al*, 2011) were cell line-specific hub genes. Although they were not present in any of the driver-networks from patient data sets, both of these genes are related to cell adhesion and migration (Camara and Jarai, 2010; Brown *et al*, 2011). These genes also have important roles in EMT, a process that was also predominant in TNBC patient driver-networks. Multiple members of the TGF-beta pathway (Xu *et al*, 2009) (*TGFB1*, *TGFBI*, and *TGFBR2*) and the Caveolin family (Elsheikh *et al*, 2008) (*CAV1* and *CAV2*) were present in TNBC cell line-specific driver-networks. These genes also have an important role in EMT, corroborating what we observed in patient data sets (Elsheikh *et al*, 2008; Xu *et al*, 2009). Another interesting and apparently counter-intuitive similarity with patient data sets was the strong correlation between *ESR1* and *EGFR* gene expressions.

Overall, these results suggest that some of the key biological processes are preserved but others are reconfigured in cancer cell lines, possibly due to cell culture conditions (Fidler, 2003). Alternatively, the variable network membership may reflect differences in statistical power (driven by the sample size) between the patient and cell line data sets. Also, the patient samples, particularly surgical data sets such as Chin *et al* (2006), contain variable amounts of stromal cells that may affect the mRNA expression and CNA data.

### Experimental validation of the functional importance of TNBC driver-network members in breast cancer cell lines

**Figure 3:**
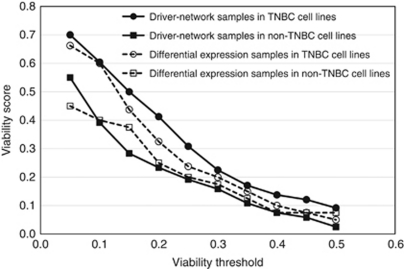
Functional relevance of the triple-negative breast cancer (TNBC) driver-networks. ‘Driver-network samples’ correspond to the thirty genes randomly selected from TNBC driver-networks and ‘differential expression samples’ correspond to 10 genes differentially expressed in all the data sets. Each of these genes was knocked down with four different siRNAs in eight TNBC and four non-TNBC cell lines. Viability score represents the average knockdown of all the genes in all the cell lines (see the Materials and Methods and the text for details). Functional validation showed that genes from driver-networks had subtype-specific effect on viability. The driver-network members were also functionally more important compared with the strongest candidates from differential gene expression analysis.

As no targeted therapy is currently available for TNBC patients, the identification of potential new therapeutic targets is of clinical importance. Therefore, we selected genes from our TNBC driver-networks for functional validation *in vitro*. Out of 92 genes that were present in any of the three driver-networks (union of the patient- and cell line-specific driver-networks) from TNBC subtype, we randomly selected 30 genes (‘driver-network members’) that were not previously linked to breast cancer biology. To test whether the integrated network analysis can functionally prioritise the candidates, we compared the performance of the ‘driver-network members’ with differentially expressed genes selected as follows. As the numbers of differentially expressed genes for each of the datasets are quite large (205, 230 and 191 from Neve *et al* (2006); Chin *et al* (2006) and Andre *et al* (2009), respectively), we had to select a small subset of these genes for functional validation. Genes that were significant in all three data sets should be the strongest candidate for validation. Hence, we identified 18 genes that were differentially expressed in all three data sets. Four genes from this list were also present in the ‘driver-network genes’ and were left out. Out of the remaining 14 genes, we randomly selected 10 for functional validation (‘differential expression members’). Each of the selected genes (30+10) were knocked down with four different siRNAs in 13 different breast cancer cell lines (*n*=9 TNBC, *n*=4 ER+ or HER2+) (Materials and methods). We considered a gene functionally validated if ⩾2 siRNAs reduced cell viability significantly compared with control siRNA (Materials and Methods). For each gene we calculated the fraction of cell lines where the gene significantly reduced cell viability in TNBC compared with non-TNBC cell lines. Finally, we averaged these numbers over all the genes to get two representative numbers from TNBC and non-TNBC groups (‘viability score’). The higher the ‘viability score’, the greater the functional importance of that gene. As the ‘viability score’ depends on the significance threshold, we calculated it for a wide range of thresholds. The ‘viability scores’ of ‘driver-network members’ were consistently higher in TNBC cell lines compared with non-TNBC cell lines (Figure 3). In TNBC cell lines, ‘driver-network members’ had higher ‘viability score’ compared with ‘differential expression members’ (Figure 3). Together, these observations imply that (i) driver-network genes have functional importance specific to the TNBC subtype and (ii) integrated analysis was able to reveal candidates that were functionally more important than what could be obtained using the strongest candidates from differential expression analysis alone.

## Discussion

### Conclusions

Data generated from different high-throughput genomic analysis platforms contain different types of noise and biases inherent to the individual technologies. Integrated approaches utilising multiple data types in combination with prior biological knowledge may help the separation of functionally important findings from noise and data set-specific biases. We present a new computational method for integrating mRNA expression and DNA copy number data in the context of comprehensive biological networks. Our method identifies local network neighborhoods with gene expression anomalies that are anchored around DNA copy number alterations. We consider these aberrantly expressed or amplified/deleted genes that are connected in the local network space as members of ‘driver-networks’. We applied this method to two human breast cancer data sets where both gene expression and DNA copy number data were available and found that different breast cancer clinical subtypes had different driver-networks. At the same time, the driver-networks from the same clinical subtype were significantly reproducible across independent data sets. The driver-networks were not only able to recapitulate existing biological knowledge, but also provided helpful insight that can pave the way to the development of novel therapeutic strategies. For example, we observed TNBC driver-networks predominantly associated with EMT, whereas ER+ driver-networks contained apoptosis-related genes responsible for tamoxifen resistance. Hence, driver-networks aid the development of rational combinatorial therapies in a subtype-specific manner. We validated the subtype specificity of the driver-networks and tested the value of the integrated analysis by knocking down 30 driver-network genes and comparing the results with knockdowns of 10 differentially expressed, non driver-network genes in a panel of breast cancer cell lines.

#### Novelty of the network expansion algorithm

Previous studies either use differential expression or differential co-expression for overlaying expression data on biological networks. Although differential expression-based analysis focuses on the properties of network nodes, differential co-expression interrogates the edges of a network. We defined differential expression based on the inter-subtype variability of expression, whereas for calculating differential co-expression we used correlation within a specific subtype. Hence, these two types of information are orthogonal to each other and could provide independent biological insight. Although most of the members of driver-networks were selected based on differential-expression, associations of Bcl-2 family members and Beclin1 with ER+ breast cancer were revealed by differential co-expression. Another interesting observation was the strong correlation of *EGFR* and *ESR1* expression in TNBC in both patient and cell line data sets (Chin *et al*, 2006; Neve *et al*, 2006).

#### Driver genes *vs* driver-networks

The objective of many previous studies was to identify driver genes, from a long list of amplified genes. Here, we shifted the focus to identifying driver-networks. The strategy of finding driver-networks instead of driver-genes has multiple advantages. First, driver-networks not only include driver genes, but also reveal the associated networks/pathways that are deregulated. Second, targeting individual driver genes is often difficult as not all driver genes are appropriate drug targets. Instead, we argue that finding a suitable drug target from members of a driver-network might be more feasible. Finally, based on the topology and membership of the driver-networks, one could propose rational combinatorial therapies, which might not be possible by only analysing a list of driver genes.

#### Validation of TNBC driver-networks using a high-throughput approach

Experimental validation of the driver-network genes has shown the functional importance of the results. We selected TNBC for functional validation as pathway deregulation is least understood in TNBC, and hence, no targeted therapy exists for this subtype. The objective of the validation was to show that driver-network genes, identified from our analysis, are functionally important and subtype specific. If only a few genes are cherry picked for detailed validation, the results will be heavily biased towards the genes that are selected. Hence, we adopted a high-throughput approach for validation involving a comparatively large set of 40 genes in 13 cell lines (four siRNAs and three replicates per gene), rather than cherry-picking a few genes and studying them in more detail. Still, extensive experimental studies are required for each of these genes to understand the mechanistic details of their action and the associated therapeutic potential, which is beyond the scope of this manuscript.

#### Satellite subnetworks

In all of the driver-networks, we found that most of the genes were either directly or indirectly connected to each other, creating a main component. However, we also observed a few satellite subnetworks, which were not connected to the main component. For example, the MYB-ASH2L and CLTC-AP1G1 satellite subnetworks were present in ER+ driver-networks from both patient data sets (Figure 2A and D). It is debatable whether these satellite subnetworks are biologically informative and whether we should retain them in the final driver-network. On several occasions, a satellite subnetwork from one patient data set was part of the main component in the other data set. For example, in the TNBC subtype, FZD6-SFRP1 and EIF3E-NDRG1-PABPC1 were satellite subnetworks in the Andre *et al* (2009) data set (Figure 2F), but became parts of the main component in the Chin *et al* data set (Figure 2C). They possibly appeared as satellite subnetworks in Figure 2F because the genes that could connect them to the main component were not differentially expressed in the Andre *et al* (2009) data set. Hence, even when the satellite networks are not linked to the main component, they could be biologically related. For some genes of the satellite subnetworks, there is earlier evidence indicating their association with the disease subtype. For example, *MYB* (Gonda *et al*, 2008) and NDRG1 (Sharp *et al*, 2008) are related to ER+ breast cancer and TNBC, respectively. Hence, we decided to retain the satellite subnetworks in the final driver-networks.

#### Clinical relevance of driver-network genes

Development of anti-oestrogen resistance is one of the impediments in the treatment of ER+ breast cancer. Tamoxifen, the most commonly used anti-oestrogen drug, competes with oestrogen to bind ER*α*, and thereby blocks the growth-promoting action of ER*α* in breast cancer cells. Yet, a subset of ER+ patients have intrinsic resistance to the therapy, and even those patients who respond, can eventually develop drug resistance (Schoenlein *et al*, 2009). The ER+ driver-networks have several members involved in anti-oestrogen resistance. For instance, *IGF1R* and *IRS1* were identified as predictive markers of tamoxifen response in patients with early breast cancer (Migliaccio *et al*, 2009). Second, autophagy and apoptosis-related genes from ER+ driver-networks provide further links to anti-oestrogen resistance. As autophagy provides a key cell survival mechanism during anti-oestrogen therapy, blocking autophagosome formation can help reduce the anti-oestrogen resistance in ER+ breast cancer (Schoenlein *et al*, 2009). Finally, several receptors emerged as dominant and consistent members of ER+ driver-networks including the *AR*, *IGF1R*, and *ERBB3*. Each of these targets is druggable and combined blockade of these receptors may be synergistic with anti-oestrogen therapy. *IGF1R* and *AR* inhibitors are currently tested separately in the clinic in this context, whereas *ERBB3* has not previously been linked to the biology of ER+ breast cancer. Hence, driver-networks identified from our analysis can provide clues on potential biomarkers that can suggest novel combinatorial therapies to circumvent anti-oestrogen resistance.

In TNBC, our analysis identified the *LYN* kinase as an important hub of the main driver-network in addition to *EGFR* in both data sets. Although *EGFR* was identified as the main hub gene and has been shown to inhibit the growth of TNBC cell lines, *EGFR* inhibitors alone or combined with carboplatin chemotherapy showed very little activity in the clinic (Corkery *et al*, 2009). Our network analysis suggests potential combination therapy approaches, such as inhibiting *LYN* and other network partners of *EGFR* in order to improve efficacy. Numerous small satellite networks that we describe implicate several biologically important genes as possible drivers of TNBC that were not previously linked to this disease. We also noted important driver-network differences between patient cancers and cell lines. This suggests that some network anomalies not consistently seen in cell lines, but observed in human data may not be readily studied in the current cell line-based experimental models and hence their therapeutic potential may be overlooked.

## Figures and Tables

**Table 1:** Reproducibility of the driver-networks from independent data sets and from seed gene- and expression randomisations (mean±s.d.)

	**ER+**	**HER2+**	**TN**
Normalised overlap of driver-networks	0.49	0.20	0.30
Normalised overlap from seed randomisation (*P*-value)	0.36±0.06 (0.01)	0.16±0.04 (0.08)	0.18±0.06 (0.01)
Normalised overlap from expression randomisation (*P*-value)	0.12±0.04 (0.00)	0.12±0.03 (0.00)	0.09±0.03 (0.00)

Abbreviations: ER=oestrogen receptor; HER2=human epidermal growth factor receptor 2; TN=triple receptor-negative.

Statistical significance of the reproducibility (*P*-value) was estimated by comparing the observed normalised overlap with the distribution of overlaps obtained from randomisation. The greatest and least normalised overlap of the driver-networks between the two data sets was observed for the ER+ and HER2+ subtypes, respectively.
