# Supplementary material for: A network-based, integrative study to identify core biological pathways that drive breast cancer clinical subtypes
Source: Br J Cancer. 2012 Feb 16;106(6):1107–16. doi: 10.1038/bjc.2011.584 (PMC3304402; doi:10.1038/bjc.2011.584)
Supplement: Supplementary Figure S1 [file bjc2011584x1.pdf]

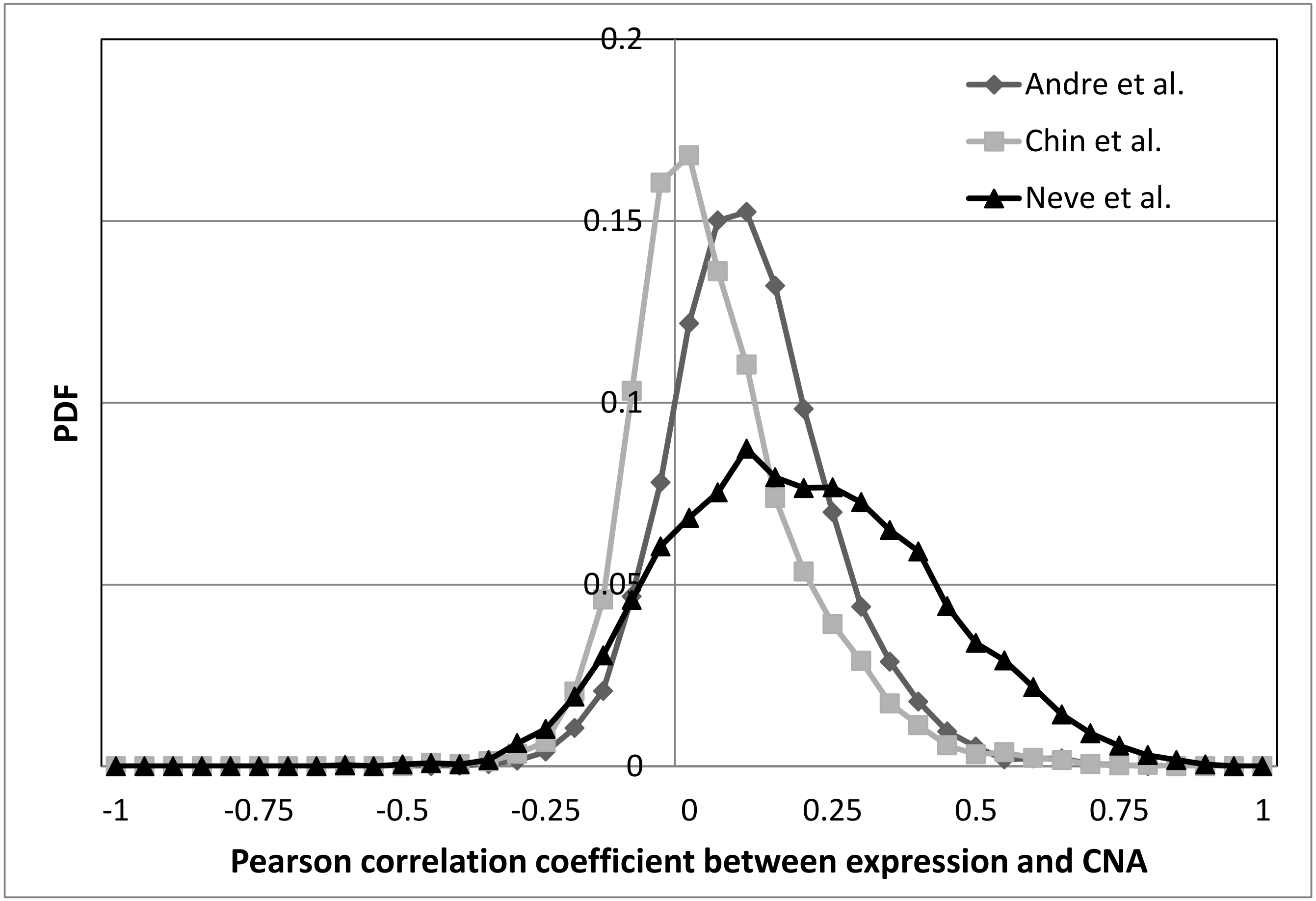

Supplemental figure 1: Distribution of Pearson correlation coefficients between CNA score and gene expression from three different datasets. Cell line dataset from Neve et al. have significantly higher correlation compared to patient datasets. Chin et al. data have shown lower correlation among the patient datasets.
