# Supplementary material for: A network-based, integrative study to identify core biological pathways that drive breast cancer clinical subtypes
Source: Br J Cancer. 2012 Feb 16;106(6):1107–16. doi: 10.1038/bjc.2011.584 (PMC3304402; doi:10.1038/bjc.2011.584)
Supplement: Supplementary Figure S3 [file bjc2011584x3.pdf]

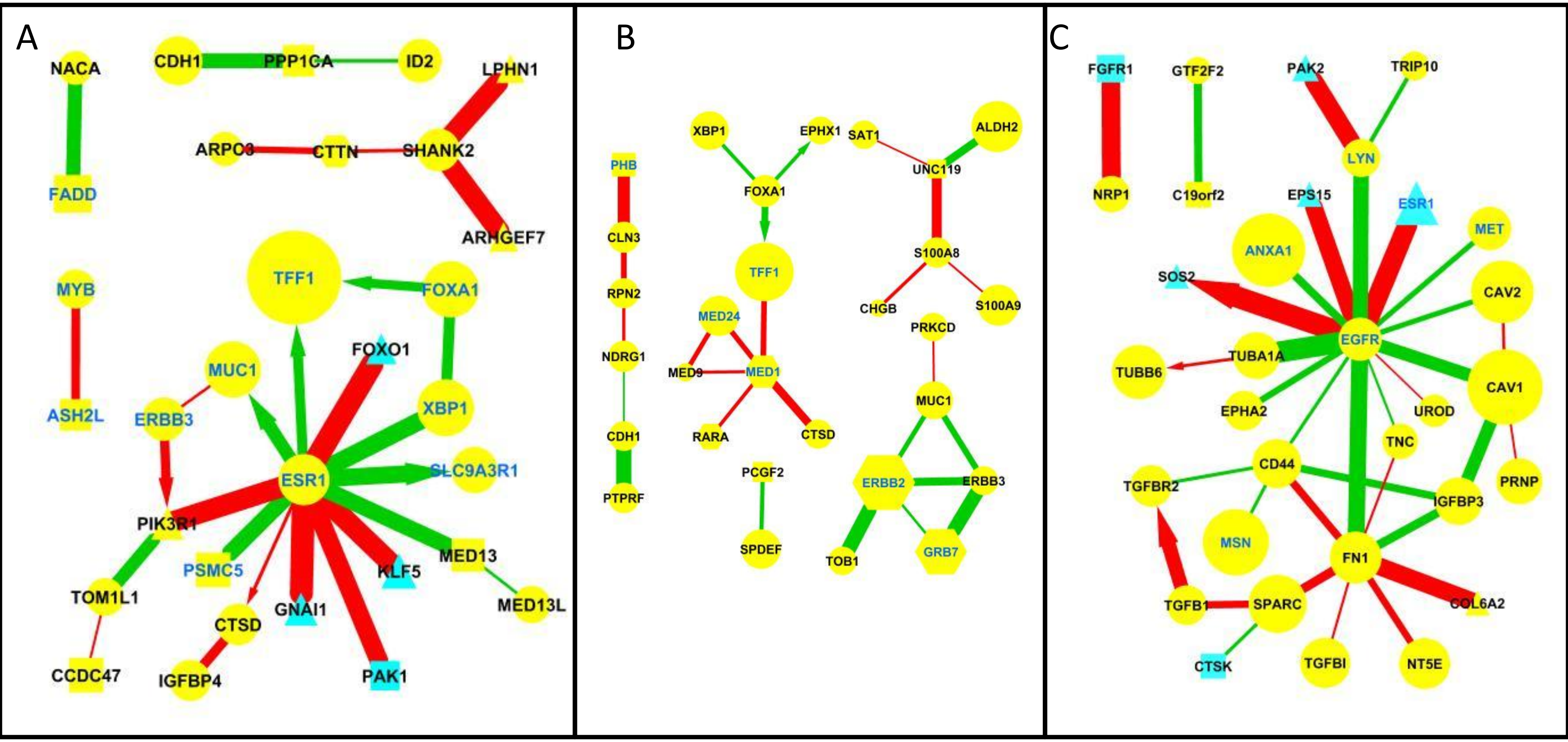

Supplemental figure 3: Driver network inferred from cell dataset (Neve et al.) for three breast cancer clinical subtypes (A) ER+, (B) HER2+, and (C)TN. Size of a node is proportional to the differential expression level of the corresponding gene. Yellow and blue nodes represent increased and decreased expression, respectively, from gene expression data. The shapes indicate the type of genomic change, squares representing the seed genes with copy number alterations, circles representing differential expression without copy number alteration, hexagons representing both copy number and gene expression changes, and triangles representing inclusion based on differential co-expression without differential expression. The width and color of an edge connecting two nodes reflect the magnitude and sign of the correlation between two genes within the driver-network. An arrow pointing from one member gene to another indicates a transcriptional or signaling relationship while lines without arrowheads represent protein-protein interactions. Genes that are also present in the corresponding subtype specific driver-networks from patient datasets (Fig 2A-F)have blue font color.
