# Supplementary material for: A network-based, integrative study to identify core biological pathways that drive breast cancer clinical subtypes
Source: Br J Cancer. 2012 Feb 16;106(6):1107–16. doi: 10.1038/bjc.2011.584 (PMC3304402; doi:10.1038/bjc.2011.584)
Supplement: Supplementary Table S1 [file bjc2011584x4.pdf]

Supplemental Table 1: Numbers of samples in 3 subtypes from each of the three datasets.

| | ER+ | HER2+ | TN |
|---|---|---|---|
| Chin *et al.* | 51 | 7 | 20 |
| André *et al.* | 55 | 11 | 37 |
| Neve *et al.* | 11 | 9 | 20 |
