# Supplementary material for: A network-based, integrative study to identify core biological pathways that drive breast cancer clinical subtypes
Source: Br J Cancer. 2012 Feb 16;106(6):1107–16. doi: 10.1038/bjc.2011.584 (PMC3304402; doi:10.1038/bjc.2011.584)
Supplement: Supplementary Table S2 [file bjc2011584x5.pdf]

Andre et al. dataset

Gene_no	gene_name	Average_CNA_Score_ER	Average_CNA_Score_HER2	Average_CNA_Score_TN	Overall_correlation
2	A2M	0.00	-0.18	0.05	0.24
9	NAT1	-0.16	-0.18	-0.22	0.04
10	NAT2	-0.16	-0.18	-0.22	0.02
12	SERPINA3	-0.02	0.00	-0.03	0.03
13	AADAC	-0.02	-0.09	0.03	0.09
14	AAMP	0.02	0.00	0.00	0.04
15	AANAT	0.04	0.09	0.03	0.01
16	AARS	-0.09	0.00	0.03	0.38
18	ABAT	0.04	0.00	0.00	0.16
19	ABCA1	-0.02	0.00	0.00	0.09
20	ABCA2	0.02	0.00	0.03	0.17
21	ABCA3	0.04	0.09	0.00	0.11
22	ABCB7	-0.04	0.00	-0.08	0.11
23	ABCF1	0.00	0.00	0.05	0.15
24	ABCA4	-0.02	0.00	0.00	0.01
25	ABL1	0.02	0.00	0.03	0.30
26	ABP1	0.02	0.09	0.00	0.01
27	ABL2	0.02	0.00	0.03	0.22
28	ABO	0.02	0.00	0.00	-0.06
29	ABR	-0.04	0.00	0.00	0.20
30	ACAA1	0.00	0.00	0.00	0.01
31	ACACA	0.00	0.09	0.05	0.26
32	ACACB	0.00	0.00	0.00	NaN
33	ACADL	0.00	0.00	0.00	NaN
34	ACADM	-0.02	0.00	-0.03	0.02
35	ACADS	0.00	0.00	0.00	NaN
36	ACADSB	-0.04	0.00	0.03	0.01
37	ACADVL	-0.05	0.00	0.00	0.00
38	ACAT1	-0.07	-0.09	0.00	0.03
39	ACAT2	-0.02	0.00	-0.05	0.03
40	ACCN1	0.00	0.09	0.00	-0.11
41	ACCN2	0.02	0.00	-0.03	0.01
43	ACHE	0.02	0.00	0.00	0.30
47	ACLY	-0.04	0.18	0.00	0.20
48	ACO1	-0.05	0.00	0.00	0.08
49	ACR	0.00	0.09	0.03	0.09
50	ACO2	-0.02	0.00	-0.03	0.36
51	ACOX1	0.04	0.27	0.03	0.44
52	ACP1	-0.02	0.00	0.00	0.13
53	ACP2	-0.02	0.00	-0.03	0.15
54	ACP5	0.00	-0.09	0.00	0.01
55	ACPP	0.00	0.00	0.00	NaN
56	ACRV1	-0.05	0.00	-0.03	0.11

58	ACTA1	0.07	0.00	0.03	-0.06
59	ACTA2	-0.04	0.00	-0.11	0.12
60	ACTB	0.00	0.00	0.00	NaN
71	ACTG1	0.02	0.09	0.08	0.18
72	ACTG2	0.00	0.00	0.00	NaN
81	ACTN4	0.02	0.09	0.05	0.42
87	ACTN1	-0.02	0.00	0.00	0.08
88	ACTN2	0.09	0.00	0.03	0.20
89	ACTN3	0.05	0.00	0.03	0.05
90	ACVR1	-0.02	0.00	0.03	0.17
91	ACVR1B	0.02	0.00	-0.03	0.09
93	ACVR2B	-0.04	0.00	0.00	0.02
94	ACVRL1	0.02	0.00	-0.03	-0.06
95	ACY1	0.00	0.00	0.00	NaN
97	ACYP1	-0.02	0.00	0.00	0.00
98	ACYP2	0.00	0.00	0.00	NaN
100	ADA	0.02	0.09	0.03	0.02
101	ADAM8	0.00	0.00	0.03	0.07
102	ADAM10	-0.02	0.00	-0.05	0.22
103	ADAR	0.11	0.00	0.14	0.29
104	ADARB1	0.00	0.00	0.00	0.13
107	ADCY1	-0.04	0.00	0.05	0.01
108	ADCY2	0.02	0.18	0.00	0.05
109	ADCY3	-0.02	0.00	0.03	0.17
112	ADCY6	0.00	0.00	0.00	NaN
113	ADCY7	-0.09	0.00	-0.03	0.14
114	ADCY8	0.20	0.09	0.38	0.16
115	ADCY9	0.04	0.00	0.00	0.25
116	ADCYAP1	0.04	-0.09	0.00	0.23
117	ADCYAP1R1	-0.02	0.09	-0.03	0.04
118	ADD1	-0.02	0.00	0.00	0.09
119	ADD2	0.02	0.00	0.00	0.01
120	ADD3	-0.04	0.00	0.00	0.22
123	ADFP	-0.04	0.00	0.00	0.16
124	ADH1A	-0.02	0.00	0.03	0.03
125	ADH1B	-0.02	0.00	0.03	0.00
126	ADH1C	-0.02	0.00	0.03	0.01
128	ADH5	-0.02	0.00	0.03	0.22
130	ADH6	-0.02	0.00	0.03	0.03
131	ADH7	-0.02	0.00	0.03	0.06
132	ADK	0.00	0.00	0.00	0.08
133	ADM	-0.04	-0.09	-0.03	-0.12
134	ADORA1	0.09	0.00	0.05	-0.01
135	ADORA2A	0.00	0.00	0.03	0.11
136	ADORA2B	-0.07	0.00	-0.03	0.23
140	ADORA3	0.02	0.00	-0.03	-0.02
141	ADPRH	0.00	0.00	-0.03	-0.12

146	ADRA1D	0.02	0.00	-0.03	0.03
147	ADRA1B	0.00	0.00	-0.03	-0.16
148	ADRA1A	-0.11	-0.18	-0.19	-0.06
150	ADRA2A	-0.04	0.00	0.00	0.01
151	ADRA2B	0.00	0.00	0.00	NaN
152	ADRA2C	-0.02	0.00	0.00	0.18
153	ADRB1	-0.04	0.00	0.00	-0.05
154	ADRB2	0.00	0.00	0.00	NaN
155	ADRB3	0.16	0.09	0.08	-0.13
156	ADRBK1	0.11	0.09	0.03	0.20
157	ADRBK2	-0.04	-0.09	0.03	0.18
158	ADSL	-0.04	0.00	-0.03	0.31
159	ADSS	0.07	0.00	0.05	0.25
161	AP2A2	0.00	0.00	0.00	NaN
162	AP1B1	0.00	0.00	0.03	0.04
163	AP2B1	-0.02	-0.09	0.03	0.17
164	AP1G1	-0.07	0.00	0.03	0.13
165	AEBP1	-0.02	0.00	0.05	0.02
166	AES	0.00	0.00	0.00	NaN
173	AFM	-0.02	0.00	0.00	-0.01
174	AFP	-0.02	0.00	0.00	0.00
175	AGA	-0.02	0.00	-0.05	0.06
176	AGC1	0.00	0.00	-0.05	0.02
177	AGER	0.00	0.00	0.05	0.01
178	AGL	-0.02	0.00	-0.03	0.20
181	AGRP	-0.04	0.00	0.00	-0.01
182	JAG1	0.00	0.00	-0.05	0.27
183	AGT	0.09	0.00	0.03	0.06
185	AGTR1	0.02	0.00	0.05	0.00
186	AGTR2	-0.05	0.09	-0.08	-0.02
187	AGTRL1	0.00	-0.09	0.03	-0.08
189	AGXT	0.00	0.00	0.00	NaN
190	NR0B1	-0.07	0.00	-0.11	-0.02
191	AHCY	0.00	0.00	0.00	NaN
195	AHNAK	-0.02	0.00	0.03	0.01
196	AHR	0.00	0.00	0.00	0.21
197	AHSG	0.04	0.00	0.03	0.07
199	AIF1	0.00	0.00	0.05	0.02
203	AK1	0.00	0.00	0.00	0.14
204	AK2	-0.02	0.00	0.00	0.23
205	AK3	-0.05	0.00	-0.05	0.18
207	AKT1	-0.05	-0.09	0.08	0.01
208	AKT2	0.00	0.00	0.05	0.03
210	ALAD	-0.02	0.00	0.00	0.07
211	ALAS1	0.00	0.00	0.00	NaN
214	ALCAM	0.00	0.00	-0.05	0.19
215	ABCD1	0.00	0.00	-0.03	-0.17

216	ALDH1A1	-0.02	0.00	-0.03	0.07
217	ALDH2	0.00	0.00	0.00	NaN
218	ALDH3A1	-0.04	0.09	0.03	0.13
219	ALDH1B1	-0.04	0.00	0.03	0.29
220	ALDH1A3	0.00	0.00	0.00	-0.12
221	ALDH3B1	0.07	0.09	0.03	0.17
222	ALDH3B2	0.05	0.09	0.03	0.09
223	ALDH9A1	0.04	0.00	0.08	0.16
224	ALDH3A2	-0.04	0.09	0.03	0.36
225	ABCD2	0.00	0.09	0.00	-0.06
229	ALDOB	-0.02	0.00	0.00	0.01
230	ALDOC	0.04	0.09	0.00	0.29
231	AKR1B1	0.02	0.00	0.00	-0.03
238	ALK	0.00	0.00	0.00	NaN
239	ALOX12	-0.05	0.00	0.00	-0.10
240	ALOX5	-0.02	0.09	0.03	0.18
241	ALOX5AP	-0.05	0.00	-0.05	0.18
242	ALOX12B	-0.05	0.00	0.00	-0.11
246	ALOX15	-0.04	0.00	0.00	0.00
247	ALOX15B	-0.05	0.00	0.00	-0.09
249	ALPL	0.00	0.00	0.00	NaN
251	ALPPL2	0.02	0.00	0.00	-0.03
257	ALX3	0.00	0.00	-0.03	-0.02
258	AMBN	-0.02	0.00	0.00	-0.02
259	AMBP	-0.02	0.00	0.00	0.07
262	AMD1	0.02	0.00	0.03	0.49
266	AMELY	0.00	0.00	-0.03	0.01
267	AMFR	-0.05	0.00	0.00	0.11
268	AMH	0.00	0.00	-0.03	-0.05
269	AMHR2	0.02	0.09	-0.03	-0.10
270	AMPD1	0.00	0.00	-0.03	0.01
271	AMPD2	0.00	0.00	-0.03	-0.04
272	AMPD3	0.00	0.00	-0.05	-0.05
273	AMPH	-0.02	0.00	0.00	0.15
274	BIN1	0.00	0.00	0.00	NaN
275	AMT	0.00	0.00	0.00	NaN
276	AMY1A	-0.16	-0.09	-0.08	0.11
284	ANGPT1	0.20	0.09	0.30	0.18
286	ANK1	0.05	0.09	0.14	0.18
287	ANK2	-0.02	0.00	0.00	0.13
288	ANK3	0.00	0.00	-0.05	0.20
290	ANPEP	0.02	0.00	-0.03	0.02
291	SLC25A4	-0.02	0.00	-0.05	0.14
292	SLC25A5	-0.05	0.00	-0.08	0.24
301	ANXA1	-0.02	0.00	0.00	0.07
302	ANXA2	-0.02	0.00	-0.05	0.11
306	ANXA3	-0.04	0.00	-0.03	0.04

307	ANXA4	0.02	0.00	0.00	0.08
308	ANXA5	0.00	0.00	-0.03	0.04
309	ANXA6	0.00	0.00	0.00	NaN
310	ANXA7	-0.02	0.00	0.00	0.21
311	ANXA11	0.00	0.00	0.00	0.22
312	ANXA13	0.18	0.09	0.38	-0.08
313	AOAH	-0.02	0.00	-0.03	0.00
314	AOC2	-0.04	0.00	0.00	0.07
316	AOX1	0.00	0.00	0.00	NaN
317	APAF1	0.00	0.00	0.00	NaN
318	NUDT2	0.00	0.00	0.00	0.04
319	APOF	0.00	0.00	0.00	NaN
320	APBA1	-0.02	0.00	-0.03	-0.02
321	APBA2	-0.04	0.09	-0.14	0.17
322	APBB1	-0.02	0.00	-0.03	0.16
323	APBB2	-0.05	0.00	-0.08	0.23
324	APC	0.00	0.00	-0.05	0.17
325	APCS	0.07	0.00	0.16	0.03
326	AIRE	0.00	0.00	0.00	-0.11
327	APEH	0.00	0.00	0.00	NaN
329	BIRC2	-0.05	-0.09	0.00	0.22
330	BIRC3	-0.05	-0.09	0.00	0.10
331	BIRC4	-0.05	0.00	-0.08	0.07
332	BIRC5	0.05	0.09	0.03	0.27
333	APLP1	0.00	0.09	0.05	0.05
334	APLP2	-0.05	0.00	-0.03	0.17
336	APOA2	0.11	0.00	0.14	0.12
338	APOB	0.00	0.00	0.00	NaN
339	APOBEC1	0.00	0.00	0.05	-0.05
341	APOC1	-0.02	0.00	-0.03	0.17
343	AQP8	0.02	0.00	0.00	-0.10
344	APOC2	-0.02	0.00	-0.03	0.09
346	APOC4	-0.02	0.00	-0.03	-0.14
348	APOE	-0.02	0.00	-0.03	0.31
350	APOH	0.07	0.18	0.03	0.16
351	APP	0.00	0.00	0.03	0.13
354	KLK3	0.00	0.00	-0.03	0.03
358	AQP1	-0.02	0.09	-0.03	-0.12
359	AQP2	0.02	0.00	-0.03	-0.05
360	AQP3	0.00	0.09	0.00	0.16
361	AQP4	0.02	-0.09	0.00	0.12
363	AQP6	0.02	0.00	-0.03	-0.08
366	AQP9	-0.02	0.00	-0.05	0.11
367	AR	-0.04	0.00	-0.05	0.00
368	ABCC6	0.02	0.09	0.00	0.13
372	ARCN1	-0.05	0.00	0.00	-0.01
375	ARF1	0.11	0.00	0.03	-0.11

377	ARF3	0.00	0.00	0.00	NaN
378	ARF4	-0.05	0.00	0.00	0.15
381	ARF5	0.02	0.00	-0.03	0.19
383	ARG1	0.00	0.00	0.08	0.12
384	ARG2	-0.02	0.00	0.00	0.03
392	ARHGAP1	-0.02	0.09	0.03	0.22
393	ARHGAP4	0.00	0.00	-0.03	0.08
394	ARHGAP5	-0.02	0.09	0.00	-0.13
395	ARHGAP6	-0.05	-0.09	-0.14	0.06
397	ARHGDIB	0.00	-0.09	0.05	-0.07
398	ARHGDIG	0.05	0.18	0.05	0.05
400	ARL1	0.00	0.00	0.03	0.16
402	ARL2	0.02	0.00	0.03	0.23
403	ARL3	0.00	0.00	-0.03	0.20
405	ARNT	0.07	0.09	0.16	0.32
406	ARNTL	0.00	0.00	-0.05	0.08
407	ARR3	-0.04	0.00	-0.08	0.16
408	ARRB1	0.07	0.09	0.00	0.02
409	ARRB2	-0.04	0.00	0.00	0.23
410	ARSA	0.00	0.09	0.03	0.03
411	ARSB	0.00	0.09	-0.14	-0.02
412	STS	-0.05	-0.09	-0.14	0.09
414	ARSD	-0.05	-0.09	-0.08	0.07
415	ARSE	-0.05	-0.09	-0.08	0.06
416	ARSF	-0.05	-0.09	-0.08	0.04
417	ART1	-0.02	0.00	-0.03	0.19
419	ART3	-0.02	0.00	0.00	0.00
421	ARVCF	-0.02	0.00	0.08	0.03
429	ASCL1	0.00	0.00	0.03	0.14
430	ASCL2	0.00	0.00	0.00	NaN
432	ASGR1	-0.05	0.00	0.00	0.15
433	ASGR2	-0.05	0.00	0.00	-0.05
434	ASIP	0.00	0.00	0.00	NaN
435	ASL	0.00	0.00	0.05	0.20
439	ASNA1	0.02	-0.09	0.00	0.36
440	ASNS	0.02	0.00	0.00	0.02
443	ASPA	-0.04	0.00	0.00	0.00
444	ASPH	0.13	0.00	0.14	0.28
462	SERPINC1	0.00	0.00	0.05	0.09
463	ATBF1	-0.07	0.00	0.00	0.10
466	ATF1	0.02	0.00	-0.03	0.17
467	ATF3	0.07	0.00	0.00	-0.01
468	ATF4	-0.04	0.00	-0.03	0.25
471	ATIC	0.00	0.00	0.00	NaN
472	ATM	-0.07	-0.09	0.00	0.16
473	RERE	-0.02	0.00	0.00	0.11
474	ATOH1	-0.04	0.00	-0.03	0.09

475	ATOX1	0.00	0.00	0.00	NaN
476	ATP1A1	0.02	0.00	-0.03	0.12
477	ATP1A2	0.05	0.00	0.14	0.06
478	ATP1A3	0.02	0.00	-0.03	0.13
479	ATP12A	-0.05	0.00	-0.08	-0.04
481	ATP1B1	0.02	0.00	0.05	0.19
482	ATP1B2	-0.07	0.00	0.00	-0.09
483	ATP1B3	0.02	0.00	0.03	0.17
486	FXYD2	-0.05	0.00	0.00	0.21
487	ATP2A1	0.05	0.00	0.00	-0.08
488	ATP2A2	0.00	0.00	0.00	NaN
489	ATP2A3	-0.04	0.00	0.00	0.07
490	ATP2B1	0.00	0.00	0.00	NaN
491	ATP2B2	-0.02	0.00	0.00	0.02
492	ATP2B3	0.00	0.00	0.00	0.11
493	ATP2B4	0.07	0.00	0.05	0.02
495	ATP4A	0.02	0.09	0.05	0.09
496	ATP4B	0.00	0.00	0.00	0.29
498	ATP5A1	0.00	-0.09	-0.03	0.12
501	ALDH7A1	0.00	0.00	-0.05	0.10
506	ATP5B	0.00	0.00	0.00	NaN
513	ATP5D	0.00	0.00	-0.03	0.26
514	ATP5E	0.05	0.09	0.03	0.28
515	ATP5F1	0.02	0.00	-0.03	-0.06
516	ATP5G1	0.05	0.18	0.00	0.44
517	ATP5G2	0.02	0.09	-0.03	0.24
518	ATP5G3	-0.02	0.00	0.00	0.10
521	ATP5I	-0.02	0.00	-0.03	0.08
522	ATP5J	0.00	0.00	0.03	0.08
525	ATP6V1B1	0.02	0.00	0.00	0.07
526	ATP6V1B2	-0.15	-0.18	-0.22	0.30
527	ATP6V0C	0.04	0.09	0.00	0.22
528	ATP6V1C1	0.22	0.09	0.32	0.40
533	ATP6V0B	0.00	0.00	0.03	0.29
534	ATP6V1G2	0.00	0.00	0.05	0.01
535	ATP6V0A1	-0.04	0.00	0.00	-0.14
539	ATP5O	0.00	0.00	0.00	0.07
540	ATP7B	-0.07	0.00	-0.08	0.13
545	ATR	0.02	0.00	0.03	0.39
546	ATRX	-0.04	0.00	-0.08	0.04
549	AUH	-0.02	0.00	0.00	-0.03
550	AUP1	0.00	0.00	0.00	NaN
551	AVP	0.00	0.00	0.00	0.10
552	AVPR1A	0.00	0.00	0.03	0.09
554	AVPR2	0.00	0.00	-0.03	-0.01
558	AXL	0.00	0.00	-0.03	-0.11
563	AZGP1	0.02	0.00	0.00	-0.10

566	AZU1	0.00	0.00	-0.03	-0.07
567	B2M	-0.04	0.00	-0.14	0.15
570	BAAT	-0.02	0.00	0.00	-0.17
571	BACH1	-0.02	0.00	0.05	0.22
572	BAD	0.00	0.00	0.03	0.13
573	BAG1	0.00	0.09	0.00	0.03
575	BAI1	0.16	0.09	0.41	0.03
576	BAI2	0.02	0.09	0.00	0.05
577	BAI3	-0.02	0.00	0.05	0.02
578	BAK1	0.00	0.00	0.05	0.06
579	BAPX1	-0.04	0.00	-0.05	-0.20
580	BARD1	0.00	0.00	0.00	NaN
581	BAX	0.00	0.00	-0.03	0.22
585	BBS4	0.00	0.00	-0.05	0.16
586	BCAT1	0.00	-0.09	0.08	0.02
587	BCAT2	0.00	0.00	-0.03	0.26
590	BCHE	0.02	0.09	0.00	0.08
593	BCKDHA	0.02	0.00	-0.03	0.28
594	BCKDHB	-0.02	0.00	-0.03	0.25
595	CCND1	0.22	0.18	0.05	0.36
596	BCL2	-0.02	-0.09	0.00	0.18
597	BCL2A1	0.00	0.00	-0.03	-0.12
598	BCL2L1	0.00	0.00	0.03	-0.22
599	BCL2L2	0.00	0.00	0.00	0.21
602	BCL3	-0.02	0.00	-0.03	0.14
604	BCL6	0.04	0.00	0.03	-0.05
605	BCL7A	0.02	0.00	0.00	0.19
607	BCL9	0.07	0.00	0.11	0.13
608	TNFRSF17	0.04	0.00	0.00	-0.10
610	HCN2	0.00	0.00	-0.03	0.09
611	OPN1SW	0.02	0.00	-0.03	0.00
613	BCR	-0.02	0.00	0.03	-0.37
617	BCS1L	0.00	0.00	0.00	NaN
623	BDKRB1	0.00	0.00	0.03	0.07
624	BDKRB2	0.00	0.00	0.03	0.41
627	BDNF	0.00	0.00	-0.03	0.13
631	BFSP1	0.00	0.00	-0.08	0.21
633	BGN	0.00	0.00	0.00	0.04
634	CEACAM1	0.02	0.00	-0.03	-0.10
635	BHMT	0.00	0.09	-0.14	-0.02
636	BICD1	0.00	-0.09	0.05	0.01
637	BID	-0.02	0.00	0.00	0.10
638	BIK	-0.02	0.00	-0.03	0.02
640	BLK	-0.16	-0.09	-0.22	0.06
641	BLM	0.02	0.00	0.00	0.15
642	BLMH	0.04	0.09	0.00	0.28
643	BLR1	-0.05	0.00	0.00	0.18

644	BLVRA	-0.02	0.00	0.05	-0.05
645	BLVRB	0.00	0.09	0.05	-0.17
648	BMI1	-0.02	0.00	0.05	0.03
649	BMP1	-0.13	-0.18	-0.22	0.11
650	BMP2	0.00	0.00	-0.05	-0.14
651	BMP3	-0.04	0.00	-0.03	-0.06
652	BMP4	0.00	0.00	0.00	0.22
653	BMP5	-0.04	0.00	0.08	-0.02
654	BMP6	-0.02	0.00	0.05	0.12
655	BMP7	0.07	0.09	0.05	0.11
657	BMPR1A	-0.02	0.00	-0.03	0.15
658	BMPR1B	-0.04	0.00	0.00	-0.09
659	BMPR2	0.00	0.00	0.00	NaN
660	BMX	-0.05	0.00	-0.14	0.13
662	BNIP1	0.00	0.00	-0.03	0.21
663	BNIP2	-0.02	0.00	-0.05	0.14
664	BNIP3	-0.04	0.00	0.00	0.10
665	BNIP3L	-0.11	-0.18	-0.19	0.13
666	BOK	0.00	0.00	0.00	NaN
668	FOXL2	0.02	0.00	0.00	-0.06
669	BPGM	0.02	0.00	0.00	0.10
670	BPHL	-0.02	0.00	0.00	0.03
671	BPI	0.02	0.00	0.03	0.04
672	BRCA1	-0.04	0.00	-0.03	0.19
673	BRAF	0.02	0.00	0.00	0.00
675	BRCA2	-0.05	0.00	-0.08	0.20
676	BRDT	-0.02	0.00	-0.03	-0.02
677	ZFP36L1	-0.02	0.00	0.00	0.15
682	BSG	0.00	0.00	-0.03	0.09
683	BST1	-0.04	-0.09	-0.05	0.10
684	BST2	0.05	0.00	0.00	0.03
685	BTC	-0.02	0.00	0.00	-0.04
686	BTD	-0.02	0.00	0.00	0.28
688	KLF5	-0.07	-0.09	-0.14	0.05
689	BTF3	0.00	0.00	-0.11	0.19
694	BTG1	0.00	0.09	0.00	-0.04
695	BTK	-0.05	0.00	-0.08	0.21
696	BTN1A1	0.00	0.00	0.03	-0.13
699	BUB1	0.00	0.00	0.00	NaN
701	BUB1B	-0.02	0.00	-0.14	-0.04
705	BYSL	0.00	0.00	0.08	0.24
708	C1QBP	-0.04	0.00	0.00	0.13
710	SERPING1	0.02	0.00	0.05	-0.02
712	C1QA	0.00	0.00	0.00	NaN
713	C1QB	0.00	0.00	0.00	NaN
715	C1R	-0.02	0.09	0.05	0.11
716	C1S	-0.02	0.09	0.05	0.16

717	C2	0.00	0.00	0.05	0.00
718	C3	0.00	-0.09	0.00	-0.01
719	C3AR1	0.00	-0.09	0.05	0.17
722	C4BPA	0.09	0.00	0.00	-0.02
725	C4BPB	0.09	0.00	0.00	-0.19
726	CAPN5	0.07	0.18	0.03	0.09
727	C5	-0.02	0.00	0.00	-0.02
729	C6	0.04	0.18	0.00	-0.05
730	C7	0.04	0.18	0.00	-0.01
731	C8A	0.00	0.00	0.00	-0.01
732	C8B	0.00	0.00	0.00	-0.07
733	C8G	0.02	0.00	0.03	-0.06
735	C9	0.04	0.09	0.00	0.07
738	C11orf2	0.02	0.00	0.03	0.32
740	MRPL49	0.02	0.00	0.03	0.14
745	C11orf9	-0.02	0.00	0.03	0.20
746	C11orf10	-0.02	0.00	0.03	0.13
747	C11orf11	-0.02	0.00	0.03	0.03
753	C18orf1	-0.02	-0.09	0.00	-0.08
754	PTTG1IP	0.00	0.00	0.00	0.29
755	C21orf2	0.00	0.00	0.00	0.33
759	CA1	0.15	0.00	0.14	0.09
760	CA2	0.15	0.00	0.14	0.03
761	CA3	0.15	0.00	0.14	-0.06
762	CA4	0.20	0.00	0.03	0.01
763	CA5A	-0.04	0.00	0.00	0.07
765	CA6	-0.02	0.00	0.00	0.01
766	CA7	-0.09	0.00	0.00	-0.04
767	CA8	0.16	0.00	0.14	0.34
768	CA9	0.00	0.00	0.00	0.15
770	CA11	0.00	0.00	-0.03	-0.08
771	CA12	0.02	0.09	-0.08	0.25
773	CACNA1A	0.00	-0.09	0.00	0.07
774	CACNA1B	0.02	0.00	0.05	0.05
775	CACNA1C	-0.02	0.09	0.08	0.17
776	CACNA1D	-0.02	0.00	0.00	0.28
777	CACNA1E	0.04	0.00	0.03	0.03
778	CACNA1F	-0.04	0.00	-0.05	0.12
779	CACNA1S	0.07	0.00	0.05	0.03
780	DDR1	0.02	0.00	0.05	0.07
781	CACNA2D1	0.02	0.00	0.00	0.02
782	CACNB1	0.00	0.55	0.05	0.18
783	CACNB2	-0.02	0.00	0.11	0.12
784	CACNB3	0.00	0.00	0.00	NaN
785	CACNB4	-0.02	0.00	0.03	0.00
786	CACNG1	0.13	0.27	0.03	0.05
788	SLC25A20	-0.04	0.00	0.00	0.12

790	CAD	0.00	0.00	0.00	NaN
793	CALB1	0.15	0.00	0.19	0.17
794	CALB2	-0.07	0.00	0.03	0.13
796	CALCA	-0.02	0.00	-0.05	0.10
797	CALCB	-0.02	0.00	-0.05	-0.04
799	CALCR	0.02	0.00	0.03	-0.02
800	CALD1	0.02	0.00	0.00	0.04
801	CALM1	-0.02	0.00	0.03	0.40
805	CALM2	0.02	0.00	0.00	0.12
808	CALM3	-0.02	0.00	-0.03	0.37
811	CALR	0.00	-0.09	0.00	0.13
813	CALU	0.02	0.00	-0.03	0.13
814	CAMK4	0.00	0.00	-0.08	-0.04
815	CAMK2A	0.00	0.00	0.00	NaN
816	CAMK2B	-0.02	0.00	0.05	0.01
818	CAMK2G	0.04	0.00	0.00	0.36
819	CAMLG	0.00	0.00	-0.03	0.15
820	CAMP	-0.04	0.00	0.00	0.07
821	CANX	0.02	0.00	-0.03	0.22
822	CAPG	0.00	0.00	0.00	NaN
823	CAPN1	0.02	0.00	0.03	0.10
824	CAPN2	0.07	0.00	0.00	0.06
825	CAPN3	-0.05	0.09	-0.14	0.08
826	CAPNS1	-0.02	0.09	0.08	-0.04
827	CAPN6	-0.05	0.00	-0.08	0.09
829	CAPZA1	0.02	0.00	-0.03	0.06
830	CAPZA2	0.00	0.00	-0.03	0.13
831	CAST	0.00	0.00	-0.14	0.12
832	CAPZB	0.00	0.00	0.00	NaN
833	CARS	-0.02	0.00	-0.03	0.11
834	CASP1	-0.07	0.00	0.00	0.31
835	CASP2	0.02	0.00	0.00	0.08
836	CASP3	-0.02	0.00	-0.05	0.19
837	CASP4	-0.07	0.00	0.00	0.03
838	CASP5	-0.07	0.00	0.00	0.10
839	CASP6	-0.02	0.00	0.00	-0.07
840	CASP7	-0.04	0.00	0.00	-0.07
841	CASP8	0.00	0.00	0.00	NaN
842	CASP9	-0.02	0.00	0.00	0.24
843	CASP10	0.00	0.00	0.00	NaN
844	CASQ1	0.05	0.00	0.14	-0.03
845	CASQ2	0.00	0.00	-0.03	0.00
846	CASR	0.00	0.00	-0.03	-0.19
847	CAT	0.04	0.00	-0.03	0.29
857	CAV1	0.00	0.00	-0.03	0.15
858	CAV2	0.00	0.00	-0.03	0.04
859	CAV3	0.00	0.00	0.00	0.09

860	RUNX2	0.00	0.00	0.05	0.01
861	RUNX1	0.02	0.00	0.00	0.18
863	CBFA2T3	-0.02	0.09	0.11	-0.09
864	RUNX3	0.00	0.00	0.00	NaN
865	CBFB	-0.05	0.00	0.00	0.26
866	SERPINA6	-0.02	0.00	0.00	-0.01
867	CBL	-0.05	0.00	0.00	0.03
868	CBLB	0.00	0.00	-0.05	0.01
869	CBLN1	-0.07	0.00	0.00	-0.02
871	SERPINH1	0.07	0.09	0.00	0.29
873	CBR1	0.00	0.00	0.00	0.16
874	CBR3	0.00	0.00	0.00	-0.06
875	CBS	0.00	0.00	0.00	0.08
881	CCIN	0.00	0.00	0.00	0.04
883	CCBL1	0.02	0.00	0.00	0.11
885	CCK	-0.04	0.00	0.00	0.02
886	CCKAR	-0.05	-0.09	-0.05	-0.10
887	CCKBR	-0.02	0.00	-0.03	0.09
890	CCNA2	0.00	0.00	-0.03	0.01
891	CCNB1	0.00	0.00	-0.08	0.01
894	CCND2	-0.02	0.09	0.08	0.11
896	CCND3	0.00	0.00	0.08	-0.04
898	CCNE1	0.00	0.09	0.05	-0.01
899	CCNF	0.04	0.09	0.00	0.18
900	CCNG1	0.00	0.00	-0.03	0.22
901	CCNG2	-0.02	0.00	-0.03	0.03
902	CCNH	0.00	0.00	-0.14	0.25
904	CCNT1	0.00	0.00	0.00	NaN
905	CCNT2	-0.02	0.00	0.00	0.07
908	CCT6A	0.00	0.00	0.05	0.40
909	CD1A	0.02	0.00	0.16	0.02
910	CD1B	0.02	0.00	0.16	-0.06
911	CD1C	0.02	0.00	0.16	-0.19
912	CD1D	0.02	0.00	0.16	-0.19
913	CD1E	0.02	0.00	0.16	-0.02
914	CD2	0.02	0.00	-0.03	-0.25
915	CD3D	-0.05	0.00	0.00	0.21
916	CD3E	-0.05	0.00	0.00	0.27
917	CD3G	-0.05	0.00	0.00	0.15
921	CD5	-0.02	0.00	0.05	0.09
922	CD5L	0.00	0.00	0.14	0.11
923	CD6	-0.02	0.00	0.05	0.24
924	CD7	-0.04	0.09	0.08	-0.10
925	CD8A	0.00	0.00	0.00	NaN
928	CD9	-0.02	0.09	0.05	0.29
929	CD14	0.00	0.00	0.00	NaN
930	CD19	0.05	0.00	0.00	-0.24

931	MS4A2	-0.04	0.00	0.00	0.18
933	CD22	0.02	0.00	0.05	0.20
934	CD24	0.02	0.00	0.00	0.17
940	CD28	0.02	0.00	0.00	0.05
941	CD80	0.00	0.00	-0.03	-0.33
942	CD86	0.00	0.00	-0.03	-0.11
943	TNFRSF8	-0.02	0.00	0.00	0.05
944	TNFSF8	-0.02	0.00	0.00	0.13
945	CD33	0.00	0.00	-0.03	-0.10
946	SIGLEC6	0.00	0.00	-0.03	-0.31
948	CD36	0.02	0.00	0.03	-0.01
951	CD37	0.02	0.00	-0.03	0.13
952	CD38	-0.04	-0.09	-0.05	-0.06
953	ENTPD1	-0.02	0.00	-0.05	0.17
955	ENTPD6	0.02	0.00	-0.03	0.11
956	ENTPD3	-0.04	0.00	0.00	-0.02
957	ENTPD5	-0.02	0.00	0.00	0.16
960	CD44	0.04	0.00	-0.03	0.30
961	CD47	0.00	0.00	-0.03	0.21
962	CD48	0.07	0.00	0.14	-0.18
963	CD53	0.00	0.00	-0.05	-0.16
965	CD58	0.02	0.00	-0.03	0.09
966	CD59	0.04	0.00	-0.05	0.25
967	CD63	0.02	0.00	0.00	0.14
968	CD68	-0.05	0.00	0.00	0.09
969	CD69	0.00	-0.18	0.05	0.04
972	CD74	0.00	0.00	0.00	NaN
973	CD79A	0.02	0.00	-0.03	-0.19
974	CD79B	0.16	0.18	0.03	-0.06
975	CD81	0.00	0.00	0.00	NaN
976	CD97	0.02	-0.09	0.00	0.19
977	CD151	0.00	0.00	0.00	NaN
978	CDA	0.00	0.00	0.00	NaN
983	CDC2	0.00	0.00	-0.05	0.05
984	CDC2L1	-0.02	0.00	0.03	0.24
987	LRBA	-0.02	0.00	0.00	0.17
988	CDC5L	0.00	0.00	0.08	0.33
990	CDC6	0.04	0.36	0.03	0.48
991	CDC20	0.02	0.00	0.00	0.14
993	CDC25A	-0.04	0.00	0.00	0.19
994	CDC25B	0.02	0.00	-0.03	-0.07
995	CDC25C	0.00	0.00	-0.03	-0.02
996	CDC27	-0.04	0.00	0.00	0.33
997	CDC34	0.00	0.00	-0.03	-0.02
998	CDC42	0.00	0.00	0.00	NaN
999	CDH1	-0.09	0.00	0.00	-0.03
1000	CDH2	0.02	-0.09	0.00	-0.04

1001	CDH3	-0.09	0.00	0.00	0.03
1002	CDH4	0.04	0.09	0.03	0.25
1003	CDH5	-0.09	0.00	0.00	0.04
1004	CDH6	0.02	0.18	0.00	0.16
1005	CDH7	-0.02	-0.09	0.00	-0.16
1006	CDH8	-0.11	0.00	0.00	-0.01
1007	CDH9	0.02	0.18	0.00	-0.01
1008	CDH10	0.02	0.18	0.00	-0.10
1009	CDH11	-0.11	0.00	0.00	0.09
1010	CDH12	0.02	0.18	0.00	-0.05
1012	CDH13	-0.09	0.00	0.00	0.02
1013	CDH15	-0.04	0.00	0.03	0.11
1014	CDH16	-0.05	0.00	0.00	-0.04
1015	CDH17	0.16	0.09	0.22	0.10
1016	CDH18	0.02	0.18	0.00	-0.26
1017	CDK2	0.04	0.00	0.00	0.26
1018	CDK3	0.04	0.27	0.03	0.22
1019	CDK4	0.02	0.09	0.00	0.21
1020	CDK5	0.04	0.00	0.08	-0.07
1021	CDK6	0.02	0.00	0.08	0.48
1022	CDK7	0.00	0.00	-0.08	0.06
1024	CDK8	-0.05	0.00	-0.05	0.15
1025	CDK9	0.00	0.00	0.00	0.11
1026	CDKN1A	0.02	0.00	0.05	0.11
1027	CDKN1B	0.00	-0.09	0.05	0.08
1028	CDKN1C	0.00	0.00	0.00	NaN
1029	CDKN2A	-0.13	-0.09	-0.05	0.19
1030	CDKN2B	-0.13	-0.09	-0.05	0.04
1031	CDKN2C	0.00	0.00	-0.03	0.22
1032	CDKN2D	0.02	0.00	0.00	0.18
1033	CDKN3	0.00	0.00	0.00	0.13
1036	CDO1	0.00	0.00	-0.05	-0.02
1038	CDR1	-0.05	0.09	-0.08	-0.16
1039	CDR2	0.02	-0.09	0.00	0.14
1040	CDS1	-0.04	0.00	-0.03	-0.02
1044	CDX1	0.00	0.00	0.00	NaN
1045	CDX2	-0.05	0.00	-0.05	-0.16
1046	CDX4	-0.04	0.00	-0.08	0.01
1047	CLGN	-0.02	0.00	0.00	-0.08
1048	CEACAM5	0.02	0.00	-0.03	0.08
1050	CEBPA	0.00	0.00	0.05	0.07
1051	CEBPB	0.05	0.00	0.05	0.16
1053	CEBPE	-0.02	0.00	0.00	0.20
1054	CEBPG	0.00	0.00	0.05	0.05
1056	CEL	0.02	0.00	0.00	0.00
1058	CENPA	-0.02	0.00	0.00	0.17
1060	CENPC1	-0.02	0.00	0.00	0.07

1062	CENPE	-0.04	0.00	0.00	0.04
1063	CENPF	0.07	0.00	0.00	0.04
1068	CETN1	0.02	-0.09	0.00	0.07
1069	CETN2	-0.02	0.00	-0.11	0.29
1070	CETN3	0.00	0.00	-0.14	0.12
1071	CETP	-0.05	0.00	0.00	0.07
1072	CFL1	0.02	0.00	0.05	-0.04
1075	CTSC	-0.05	0.00	0.00	0.14
1080	CFTR	0.00	0.00	-0.03	0.07
1081	CGA	-0.02	0.00	-0.05	-0.12
1084	CEACAM3	0.02	0.00	-0.03	-0.16
1087	CEACAM7	0.02	0.00	-0.03	0.06
1088	CEACAM8	0.00	0.00	-0.03	0.00
1089	CEACAM4	0.02	0.00	-0.03	-0.22
1103	CHAT	-0.02	0.00	-0.03	0.01
1105	CHD1	0.00	0.00	-0.14	0.20
1106	CHD2	0.02	0.00	-0.03	-0.10
1107	CHD3	-0.05	0.00	0.00	0.10
1108	CHD4	-0.02	0.09	0.05	0.26
1109	AKR1C4	0.00	0.00	0.08	0.01
1111	CHEK1	-0.05	0.00	-0.03	0.08
1112	CHES1	-0.02	0.00	0.03	0.14
1113	CHGA	-0.02	0.00	0.03	-0.01
1114	CHGB	0.00	0.00	-0.05	-0.11
1116	CHI3L1	0.09	0.00	0.05	0.17
1117	CHI3L2	0.00	0.00	-0.03	-0.13
1118	CHIT1	0.09	0.00	0.05	-0.06
1121	CHM	-0.07	0.00	-0.08	0.02
1123	CHN1	0.00	0.00	0.00	-0.09
1124	CHN2	-0.02	0.18	0.00	0.09
1129	CHRM2	0.02	0.00	0.03	0.05
1132	CHRM4	-0.02	0.00	-0.03	0.27
1134	CHRNA1	0.00	0.00	0.00	0.17
1135	CHRNA2	-0.09	-0.18	-0.19	0.04
1136	CHRNA3	0.00	0.00	-0.03	0.18
1137	CHRNA4	0.04	0.09	0.08	0.03
1138	CHRNA5	0.00	0.00	-0.03	-0.04
1139	CHRNA7	0.00	0.00	-0.16	-0.08
1140	CHRNB1	-0.05	0.00	0.00	-0.04
1141	CHRNB2	0.11	0.00	0.14	-0.01
1142	CHRNB3	0.02	0.09	0.11	0.07
1143	CHRNB4	0.00	0.00	-0.03	-0.09
1144	CHRND	0.02	0.00	0.00	0.12
1145	CHRNE	-0.04	0.00	0.00	0.24
1146	CHRNG	0.02	0.00	0.00	0.11
1147	CHUK	-0.02	0.00	-0.03	0.10
1149	CIDEA	-0.02	-0.09	0.00	0.07

1152	CKB	0.00	0.00	0.03	-0.03
1153	CIRBP	0.00	0.00	-0.03	0.07
1154	CISH	0.00	0.00	0.00	NaN
1158	CKM	-0.02	0.00	-0.03	0.02
1160	CKMT2	0.00	0.09	-0.14	-0.08
1164	CKS2	-0.02	0.00	0.00	0.09
1173	AP2M1	0.05	0.00	0.05	0.21
1174	AP1S1	0.02	0.00	0.00	0.18
1175	AP2S1	-0.04	0.00	-0.03	0.45
1176	AP3S1	0.00	0.00	-0.05	0.12
1178	CLC	0.02	0.09	0.08	0.01
1179	CLCA1	-0.02	0.00	-0.03	-0.12
1180	CLCN1	0.02	0.00	0.00	-0.04
1181	CLCN2	0.05	0.00	0.05	0.18
1182	CLCN3	-0.02	0.00	-0.05	0.10
1183	CLCN4	-0.05	-0.09	-0.14	0.01
1184	CLCN5	-0.04	0.00	-0.08	-0.15
1185	CLCN6	-0.02	0.00	0.00	-0.06
1186	CLCN7	0.04	0.18	0.08	0.20
1187	CLCNKA	0.02	0.18	0.00	0.17
1188	CLCNKB	0.02	0.18	0.00	0.02
1191	CLU	-0.09	-0.18	-0.19	0.30
1192	CLIC1	0.00	0.00	0.05	0.12
1193	CLIC2	-0.05	0.00	-0.11	0.23
1196	CLK2	0.09	0.00	0.14	0.41
1198	CLK3	0.00	0.00	0.00	NaN
1203	CLN5	-0.05	-0.09	-0.14	0.23
1207	CLNS1A	0.07	0.09	0.03	0.53
1209	CLPTM1	-0.04	0.00	-0.03	0.14
1211	CLTA	0.00	0.00	0.00	-0.14
1212	CLTB	0.00	0.00	-0.03	0.12
1213	CLTC	0.20	0.18	0.03	0.72
1215	CMA1	-0.02	0.00	0.00	-0.03
1230	CCR1	-0.04	0.00	0.00	0.25
1232	CCR3	-0.04	0.00	0.00	0.08
1233	CCR4	0.02	0.00	-0.03	-0.07
1234	CCR5	-0.04	0.00	0.00	0.18
1235	CCR6	-0.02	0.00	-0.03	0.15
1236	CCR7	0.02	0.09	0.00	0.24
1237	CCR8	-0.04	0.00	0.00	0.06
1238	CCBP2	-0.04	0.00	0.00	0.09
1240	CMKLR1	0.00	0.00	0.00	NaN
1241	LTB4R	0.00	0.00	0.00	0.08
1244	ABCC2	-0.02	0.00	-0.03	0.12
1258	CNGB1	-0.05	0.00	0.03	0.14
1259	CNGA1	-0.04	0.00	-0.03	-0.03
1261	CNGA3	0.00	0.00	0.00	NaN

1264	CNN1	0.00	0.00	0.00	NaN
1265	CNN2	0.00	0.00	-0.03	-0.03
1266	CNN3	-0.02	0.00	0.00	0.10
1267	CNP	-0.04	0.09	0.00	0.36
1268	CNR1	-0.02	0.00	-0.05	-0.10
1269	CNR2	0.00	0.00	0.00	NaN
1271	CNTFR	0.00	0.00	0.00	0.35
1272	CNTN1	0.00	0.09	0.03	0.00
1277	COL1A1	0.13	0.18	0.03	0.16
1280	COL2A1	0.00	0.00	0.00	NaN
1281	COL3A1	0.02	0.00	0.00	0.13
1282	COL4A1	0.00	0.00	-0.03	-0.03
1284	COL4A2	0.00	0.00	-0.05	0.04
1285	COL4A3	0.00	0.00	0.00	NaN
1286	COL4A4	0.00	0.00	0.00	NaN
1287	COL4A5	-0.05	0.00	-0.08	0.02
1288	COL4A6	-0.05	0.00	-0.08	-0.10
1289	COL5A1	0.02	0.00	0.03	0.13
1290	COL5A2	0.02	0.00	0.00	0.12
1291	COL6A1	0.00	0.09	0.00	0.17
1292	COL6A2	0.00	0.09	0.00	0.11
1293	COL6A3	0.02	0.00	0.00	0.08
1294	COL7A1	-0.04	0.00	0.00	-0.09
1295	COL8A1	0.00	0.00	0.00	0.11
1297	COL9A1	-0.02	0.00	0.00	0.02
1298	COL9A2	-0.02	0.00	0.00	0.11
1299	COL9A3	0.04	0.09	0.08	0.16
1301	COL11A1	-0.02	0.00	-0.03	0.01
1305	COL13A1	-0.02	0.00	0.03	0.07
1306	COL15A1	-0.02	0.00	0.00	0.05
1307	COL16A1	0.02	0.09	0.00	0.07
1308	COL17A1	-0.02	0.00	0.00	-0.01
1310	COL19A1	-0.02	0.00	0.00	0.03
1311	COMP	0.02	0.00	0.03	0.05
1312	COMT	-0.02	0.00	0.08	0.20
1314	COPA	0.05	0.00	0.14	0.43
1317	SLC31A1	-0.02	0.00	0.00	0.10
1318	SLC31A2	-0.02	0.00	0.00	0.11
1326	MAP3K8	-0.02	0.00	0.03	0.09
1327	COX4I1	-0.09	0.00	0.00	0.31
1329	COX5B	0.00	0.00	0.00	NaN
1337	COX6A1	0.02	0.00	0.00	0.13
1345	COX6C	0.16	0.18	0.27	0.06
1346	COX7A1	-0.02	0.09	0.08	-0.07
1347	COX7A2	-0.02	0.00	-0.03	0.21
1349	COX7B	-0.04	0.00	-0.08	0.33
1350	COX7C	0.00	0.00	-0.14	0.10

1352	COX10	-0.05	0.00	-0.03	0.23
1353	COX11	0.07	0.27	0.03	0.49
1355	COX15	-0.02	0.00	-0.03	0.36
1356	CP	0.04	0.00	0.03	0.20
1357	CPA1	0.02	0.00	0.00	0.08
1358	CPA2	0.02	0.00	0.00	0.06
1359	CPA3	0.02	0.00	0.03	0.14
1360	CPB1	0.02	0.00	0.05	0.03
1361	CPB2	-0.07	0.00	-0.11	0.18
1362	CPD	0.02	0.09	0.00	0.29
1363	CPE	0.00	0.00	-0.05	0.02
1364	CLDN4	0.02	0.00	0.05	0.03
1365	CLDN3	0.02	0.09	0.05	0.12
1366	CLDN7	-0.05	0.00	0.00	-0.05
1368	CPM	0.04	0.09	0.03	0.12
1369	CPN1	-0.02	0.00	-0.03	0.01
1371	CPO	0.00	0.00	0.00	NaN
1373	CPS1	0.00	0.00	0.00	NaN
1374	CPT1A	0.09	0.09	0.03	0.21
1376	CPT2	0.00	0.00	-0.03	0.04
1378	CR1	0.09	0.00	0.00	-0.03
1380	CR2	0.09	0.00	0.00	-0.13
1381	CRABP1	0.00	0.00	-0.03	0.03
1382	CRABP2	0.04	0.00	0.11	0.13
1384	CRAT	0.02	0.00	0.00	0.09
1385	CREB1	0.00	0.00	0.00	NaN
1386	ATF2	-0.02	0.00	0.00	-0.04
1387	CREBBP	0.04	0.00	-0.03	0.22
1388	CREBL1	0.00	0.00	0.05	0.17
1389	CREBL2	0.00	-0.09	0.05	0.13
1390	CREM	-0.02	0.00	0.00	0.22
1392	CRH	0.11	0.00	0.16	0.19
1393	CRHBP	0.00	0.00	-0.14	0.00
1394	CRHR1	-0.05	0.00	0.00	0.22
1395	CRHR2	-0.02	0.09	-0.03	-0.05
1397	CRIP2	0.00	0.00	0.08	-0.20
1398	CRK	-0.04	0.00	0.00	0.00
1399	CRKL	-0.02	0.00	0.03	0.22
1400	CRMP1	-0.02	0.00	0.00	-0.04
1401	CRP	0.07	0.00	0.16	-0.03
1406	CRX	-0.02	0.00	0.00	-0.06
1407	CRY1	0.00	0.00	0.00	NaN
1408	CRY2	-0.02	0.00	-0.03	-0.01
1409	CRYAA	0.00	0.00	-0.03	0.03
1410	CRYAB	-0.07	0.00	0.00	0.16
1411	CRYBA1	0.04	0.27	0.00	-0.06
1412	CRYBA2	0.00	0.00	0.00	NaN

1413	CRYBA4	-0.05	-0.09	0.03	0.09
1414	CRYBB1	-0.05	-0.09	0.03	0.04
1415	CRYBB2	0.00	0.00	0.03	0.20
1417	CRYBB3	0.00	0.00	0.03	0.02
1418	CRYGA	0.00	0.00	0.00	NaN
1419	CRYGB	0.00	0.00	0.00	NaN
1420	CRYGC	0.00	0.00	0.00	NaN
1421	CRYGD	0.00	0.00	0.00	NaN
1428	CRYM	0.02	0.00	0.00	-0.23
1429	CRYZ	-0.02	0.00	-0.03	0.02
1431	CS	0.00	0.00	0.00	NaN
1432	MAPK14	0.02	0.00	0.05	0.13
1434	CSE1L	0.07	0.00	0.03	0.44
1435	CSF1	0.00	0.00	-0.03	0.12
1436	CSF1R	0.00	0.00	0.00	NaN
1437	CSF2	0.00	0.00	-0.03	0.15
1439	CSF2RB	-0.04	0.00	0.03	0.20
1440	CSF3	0.04	0.55	0.05	0.02
1441	CSF3R	-0.02	0.00	0.00	-0.05
1445	CSK	0.00	0.00	0.00	NaN
1447	CSN2	-0.02	0.00	0.00	0.08
1452	CSNK1A1	0.00	0.00	0.00	NaN
1453	CSNK1D	-0.02	0.09	0.08	0.07
1454	CSNK1E	-0.04	0.00	-0.03	0.25
1455	CSNK1G2	-0.02	0.09	-0.03	0.23
1456	CSNK1G3	0.00	0.00	-0.03	0.07
1457	CSNK2A1	0.02	0.00	-0.03	0.23
1459	CSNK2A2	-0.07	0.00	0.00	0.24
1460	CSNK2B	0.00	0.00	0.05	0.14
1462	CSPG2	0.00	0.09	-0.14	0.00
1463	CSPG3	0.02	0.00	0.00	0.00
1464	CSPG4	0.00	0.00	0.00	NaN
1465	CSRP1	0.07	0.00	0.05	0.31
1466	CSRP2	-0.02	0.00	0.00	0.19
1468	SLC25A10	0.00	0.09	0.08	0.06
1471	CST3	0.02	0.00	-0.03	0.03
1472	CST4	0.02	0.00	-0.03	0.13
1473	CST5	0.02	0.00	-0.03	0.00
1474	CST6	0.02	0.00	0.03	-0.01
1475	CSTA	0.00	0.00	-0.03	0.33
1476	CSTB	0.00	0.00	0.00	0.26
1477	CSTF1	0.02	0.00	0.05	0.29
1478	CSTF2	-0.05	0.00	-0.08	-0.19
1479	CSTF3	0.04	0.00	-0.03	0.06
1486	CTBS	-0.02	0.00	-0.03	0.05
1487	CTBP1	0.02	0.00	0.00	0.06
1488	CTBP2	-0.04	0.00	0.03	0.12

1489	CTF1	0.07	0.09	0.00	0.05
1490	CTGF	-0.02	0.00	0.08	-0.07
1491	CTH	-0.02	0.00	-0.03	-0.03
1493	CTLA4	0.00	0.00	0.00	NaN
1495	CTNNA1	0.00	0.00	-0.03	0.10
1496	CTNNA2	0.00	0.00	0.00	NaN
1497	CTNS	-0.04	0.00	0.00	0.16
1499	CTNNB1	-0.04	0.00	0.00	0.12
1500	CTNND1	0.02	0.00	0.05	0.32
1501	CTNND2	0.02	0.18	0.00	0.06
1503	CTPS	0.00	0.00	0.00	NaN
1504	CTRB1	-0.09	0.00	0.03	0.10
1506	CTRL	-0.04	0.00	0.00	0.11
1508	CTSB	-0.15	-0.18	-0.22	0.32
1511	CTSG	-0.02	0.00	0.00	0.10
1512	CTSH	0.00	0.00	-0.03	-0.09
1513	CTSK	0.09	0.09	0.16	-0.08
1514	CTSL	-0.02	0.00	0.00	0.01
1515	CTSL2	-0.02	0.00	0.00	-0.03
1519	CTSO	-0.02	0.00	-0.03	0.13
1520	CTSS	0.09	0.09	0.16	-0.07
1521	CTSW	0.02	0.00	0.03	-0.11
1522	CTSZ	0.05	0.09	0.03	0.15
1523	CUTL1	0.02	0.00	0.00	0.14
1524	CX3CR1	-0.04	0.00	0.00	0.14
1525	CXADR	-0.04	-0.18	0.05	0.10
1527	CXorf2	0.02	0.00	-0.03	0.02
1534	CYB561	0.16	0.09	0.03	0.66
1535	CYBA	-0.04	0.00	0.03	0.12
1536	CYBB	-0.05	0.00	-0.11	0.12
1537	CYC1	0.16	0.00	0.35	0.28
1539	CYLC2	-0.02	0.00	0.00	-0.04
1540	CYLD	-0.05	0.00	-0.03	0.20
1543	CYP1A1	0.00	0.00	0.00	NaN
1544	CYP1A2	0.00	0.00	0.00	NaN
1545	CYP1B1	0.00	0.00	0.00	NaN
1548	CYP2A6	0.00	0.00	0.00	-0.11
1551	CYP3A7	0.02	0.00	0.00	0.12
1553	CYP2A13	0.00	0.00	0.00	-0.12
1555	CYP2B6	0.00	0.00	0.00	-0.06
1558	CYP2C8	-0.02	0.00	-0.05	0.17
1559	CYP2C9	-0.02	0.00	-0.05	0.09
1562	CYP2C18	-0.02	0.00	-0.05	0.19
1572	CYP2F1	0.00	0.00	0.00	-0.10
1573	CYP2J2	0.00	0.00	0.00	0.04
1576	CYP3A4	0.02	0.00	0.00	0.13
1577	CYP3A5	0.02	0.00	0.00	-0.10

1579	CYP4A11	0.00	0.00	0.00	0.11
1580	CYP4B1	0.00	0.00	0.00	-0.02
1581	CYP7A1	0.15	0.00	0.08	0.10
1584	CYP11B1	0.16	0.09	0.38	-0.05
1585	CYP11B2	0.16	0.00	0.35	-0.08
1592	CYP26A1	-0.02	0.00	-0.05	-0.09
1593	CYP27A1	0.00	0.00	0.00	NaN
1594	CYP27B1	0.02	0.09	0.00	0.17
1600	DAB1	0.00	0.00	0.00	0.06
1601	DAB2	0.04	0.09	0.00	0.13
1603	DAD1	-0.02	0.00	0.00	0.00
1605	DAG1	0.00	0.00	0.00	NaN
1606	DGKA	0.04	0.00	0.00	0.10
1607	DGKB	-0.02	0.00	0.00	-0.02
1608	DGKG	0.04	0.00	0.03	0.07
1609	DGKQ	0.02	0.00	0.05	-0.09
1610	DAO	0.00	0.00	0.00	NaN
1611	DAP	0.02	0.18	0.00	0.28
1612	DAPK1	-0.02	0.00	0.00	0.15
1613	DAPK3	0.00	0.00	0.00	NaN
1615	DARS	0.00	0.00	0.00	0.26
1616	DAXX	0.00	0.00	0.05	0.12
1618	DAZL	-0.02	0.00	-0.03	0.05
1621	DBH	0.02	0.00	0.00	-0.09
1622	DBI	0.00	0.00	0.00	NaN
1627	DBN1	0.00	0.00	0.00	NaN
1628	DBP	0.00	0.00	-0.03	-0.14
1629	DBT	-0.02	0.00	-0.03	0.16
1630	DCC	-0.02	-0.18	-0.05	0.13
1633	DCK	-0.02	0.00	0.00	0.11
1634	DCN	0.00	0.09	0.00	0.05
1635	DCTD	-0.02	0.00	-0.05	0.19
1636	ACE	0.16	0.09	0.03	0.18
1638	DCT	-0.02	0.00	-0.05	0.11
1639	DCTN1	0.00	0.00	0.00	NaN
1641	DCX	-0.05	0.00	-0.08	0.09
1642	DDB1	-0.02	0.09	0.05	0.29
1643	DDB2	-0.02	0.00	-0.03	0.35
1644	DDC	-0.05	0.00	0.05	0.19
1645	AKR1C1	0.02	0.00	0.08	0.18
1646	AKR1C2	0.00	0.00	0.08	0.20
1647	GADD45A	-0.02	0.00	0.00	0.00
1649	DDIT3	0.02	0.00	0.00	0.20
1650	DDOST	0.00	0.00	0.00	NaN
1652	DDT	0.00	0.00	0.05	-0.03
1653	DDX1	-0.02	0.00	0.00	0.03
1655	DDX5	0.16	0.18	0.03	0.42

1656	DDX6	-0.05	0.00	0.00	-0.01
1657	DMXL1	0.00	0.00	0.00	NaN
1662	DDX10	-0.07	-0.09	0.00	0.21
1663	DDX11	0.02	-0.09	0.08	0.15
1666	DECR1	0.15	0.00	0.19	0.01
1669	DEFA4	-0.18	-0.18	-0.19	0.07
1670	DEFA5	-0.18	-0.18	-0.19	0.01
1671	DEFA6	-0.18	-0.18	-0.19	0.11
1672	DEFB1	-0.18	-0.18	-0.19	0.12
1674	DES	0.00	0.00	0.00	NaN
1676	DFFA	0.00	0.00	0.00	NaN
1677	DFFB	-0.02	0.00	0.00	-0.11
1678	TIMM8A	-0.05	0.00	-0.08	-0.15
1687	DFNA5	0.00	0.18	0.03	-0.04
1690	COCH	-0.02	0.00	0.00	0.12
1716	DGUOK	0.00	0.00	0.00	NaN
1717	DHCR7	0.07	0.09	0.03	0.27
1718	DHCR24	0.00	0.00	0.00	0.16
1719	DHFR	0.00	0.00	-0.14	0.20
1723	DHODH	-0.07	0.00	0.00	0.09
1725	DHPS	0.02	-0.09	0.00	0.15
1728	NQO1	-0.09	0.00	0.03	0.01
1729	DIAPH1	0.00	0.00	0.00	NaN
1730	DIAPH2	-0.05	0.00	-0.08	0.10
1733	DIO1	0.00	0.00	0.00	0.04
1734	DIO2	-0.02	0.00	0.00	0.12
1735	DIO3	0.00	0.00	0.00	NaN
1736	DKC1	-0.04	0.00	-0.11	0.21
1737	DLAT	-0.07	0.00	0.00	0.07
1738	DLD	0.02	0.00	-0.03	0.08
1739	DLG1	0.02	0.09	-0.03	0.37
1740	DLG2	-0.02	0.00	0.00	-0.01
1741	DLG3	-0.04	0.00	-0.08	-0.13
1742	DLG4	-0.05	0.00	0.00	0.02
1743	DLST	-0.02	0.00	0.00	0.16
1746	DLX2	-0.02	0.00	0.00	0.03
1748	DLX4	0.13	0.18	0.03	0.21
1749	DLX5	0.02	0.00	-0.03	0.01
1750	DLX6	0.02	0.00	-0.03	0.03
1755	DMBT1	-0.04	0.00	-0.03	0.04
1756	DMD	-0.07	0.00	-0.11	0.16
1757	SARDH	0.02	0.00	0.00	0.08
1758	DMP1	-0.04	0.00	-0.03	-0.10
1759	DNM1	0.02	0.00	0.00	0.11
1760	DMPK	-0.02	0.00	0.00	0.10
1761	DMRT1	-0.04	0.00	-0.11	0.03
1762	DMWD	-0.02	0.00	0.00	0.13

1770	DNAH9	-0.09	0.00	-0.03	-0.01
1773	DNASE1	0.04	0.00	0.00	0.04
1774	DNASE1L1	0.00	0.00	-0.03	0.16
1776	DNASE1L3	-0.04	0.00	0.00	0.08
1777	DNASE2	0.02	-0.09	0.00	0.13
1785	DNM2	0.02	0.00	0.00	0.16
1786	DNMT1	0.00	0.00	0.00	NaN
1788	DNMT3A	-0.02	0.00	0.00	0.13
1789	DNMT3B	0.00	0.00	0.00	NaN
1791	DNTT	-0.02	0.00	-0.05	0.11
1793	DOCK1	-0.04	0.00	0.00	-0.02
1794	DOCK2	0.00	0.00	-0.03	0.04
1795	DOCK3	0.00	0.00	0.00	NaN
1796	DOK1	0.00	0.00	0.00	NaN
1797	DOM3Z	0.00	0.00	0.05	0.19
1798	DPAGT1	-0.05	0.00	0.00	0.09
1800	DPEP1	-0.04	0.00	0.03	0.03
1803	DPP4	-0.02	0.00	0.03	0.04
1804	DPP6	0.02	0.00	-0.03	0.01
1805	DPT	0.02	0.00	0.05	0.02
1806	DPYD	-0.02	0.00	-0.03	-0.03
1807	DPYS	0.20	0.09	0.30	-0.12
1808	DPYSL2	-0.11	-0.18	-0.19	0.09
1809	DPYSL3	0.00	0.00	0.00	NaN
1810	DR1	-0.02	0.00	0.00	0.12
1811	SLC26A3	0.02	0.00	-0.03	0.14
1812	DRD1	0.00	0.00	-0.03	0.04
1813	DRD2	-0.07	0.00	0.00	0.17
1814	DRD3	0.00	0.00	-0.03	-0.12
1815	DRD4	0.00	0.00	0.00	NaN
1819	DRG2	-0.05	0.00	0.00	0.06
1821	DRP2	-0.05	0.00	-0.08	-0.10
1823	DSC1	0.02	-0.09	0.00	-0.09
1824	DSC2	0.02	-0.09	0.00	0.06
1825	DSC3	0.02	-0.09	0.00	0.05
1826	DSCAM	0.00	0.00	0.00	0.15
1827	DSCR1	0.00	0.00	0.00	-0.03
1828	DSG1	0.02	-0.09	0.00	-0.03
1830	DSG3	0.02	-0.09	0.00	0.07
1832	DSP	-0.02	0.00	0.05	0.07
1836	SLC26A2	0.00	0.00	0.00	NaN
1837	DTNA	0.02	-0.09	-0.03	0.33
1838	DTNB	-0.02	0.00	0.00	0.04
1843	DUSP1	0.00	0.00	-0.03	0.11
1844	DUSP2	0.00	0.00	0.00	NaN
1845	DUSP3	-0.04	0.00	0.00	0.15
1846	DUSP4	-0.13	-0.18	-0.19	0.12

1847	DUSP5	-0.04	0.00	0.00	0.03
1848	DUSP6	0.00	0.00	0.00	NaN
1852	DUSP9	0.00	0.00	0.00	-0.08
1854	DUT	-0.05	0.00	-0.08	0.20
1856	DVL2	-0.05	0.00	0.00	0.08
1857	DVL3	0.05	0.00	0.05	0.36
1859	DYRK1A	0.02	0.00	0.00	0.29
1869	E2F1	0.00	0.00	0.00	NaN
1870	E2F2	0.00	0.00	0.00	NaN
1871	E2F3	0.00	0.00	0.08	0.39
1874	E2F4	-0.04	0.00	0.00	0.06
1875	E2F5	0.15	0.00	0.14	0.24
1876	E2F6	-0.02	0.00	0.00	-0.02
1877	E4F1	0.04	0.09	0.00	0.18
1889	ECE1	0.00	0.00	0.00	NaN
1890	ECGF1	0.00	0.09	0.03	0.12
1891	ECH1	0.02	0.09	0.05	0.19
1892	ECHS1	-0.02	0.00	0.00	0.04
1893	ECM1	0.07	0.18	0.14	0.10
1901	EDG1	-0.02	0.00	-0.03	-0.13
1902	EDG2	-0.02	0.00	0.00	0.03
1906	EDN1	0.00	0.00	0.11	-0.11
1907	EDN2	0.00	0.00	0.00	NaN
1908	EDN3	0.05	0.09	0.00	0.00
1909	EDNRA	-0.02	0.00	-0.03	0.31
1910	EDNRB	-0.04	-0.09	-0.14	0.19
1915	EEF1A1	0.00	0.00	-0.03	0.05
1933	EEF1B2	0.00	0.00	0.00	NaN
1936	EEF1D	0.16	0.00	0.35	0.22
1937	EEF1G	-0.02	0.00	0.03	0.16
1938	EEF2	0.00	0.00	0.00	NaN
1939	LGTN	0.07	0.00	0.00	0.30
1942	EFNA1	0.09	0.00	0.14	0.25
1943	EFNA2	0.00	0.00	-0.03	-0.04
1944	EFNA3	0.09	0.00	0.14	0.08
1945	EFNA4	0.09	0.00	0.14	0.24
1946	EFNA5	0.00	0.00	-0.11	-0.09
1947	EFNB1	-0.04	0.00	-0.05	-0.09
1948	EFNB2	-0.02	0.00	-0.05	0.09
1949	EFNB3	-0.07	0.00	0.00	0.01
1950	EGF	-0.02	0.00	0.00	-0.01
1951	CELSR3	-0.04	0.00	0.00	0.07
1952	CELSR2	0.00	0.00	-0.03	0.23
1956	EGFR	-0.02	0.00	0.11	0.63
1958	EGR1	0.00	0.00	-0.03	0.02
1959	EGR2	-0.02	0.00	-0.05	-0.05
1960	EGR3	-0.13	-0.18	-0.22	0.15

1961	EGR4	0.00	0.00	0.00	NaN
1962	EHHADH	0.04	0.00	0.00	0.18
1965	EIF2S1	-0.02	0.00	0.00	0.23
1967	EIF2B1	0.02	0.00	0.00	0.25
1968	EIF2S3	-0.05	0.00	-0.11	0.13
1969	EPHA2	-0.02	0.09	0.00	-0.02
1973	EIF4A1	-0.05	0.00	0.00	0.11
1974	EIF4A2	0.04	0.00	0.03	0.20
1975	EIF4B	0.02	0.00	-0.03	0.17
1977	EIF4E	-0.04	0.00	0.03	0.22
1978	EIF4EBP1	0.18	0.09	0.08	0.58
1979	EIF4EBP2	-0.02	0.00	-0.03	0.10
1981	EIF4G1	0.05	0.00	0.05	0.33
1982	EIF4G2	0.00	0.00	-0.05	0.02
1983	EIF5	0.00	0.00	0.00	NaN
1984	EIF5A	-0.05	0.00	0.00	0.17
1990	ELA1	0.02	0.00	-0.03	0.13
1991	ELA2	0.00	0.00	-0.03	0.07
1992	SERPINB1	-0.02	0.00	0.00	0.40
1993	ELAVL2	-0.11	0.00	-0.05	0.15
1994	ELAVL1	0.00	-0.09	0.00	0.04
1995	ELAVL3	0.00	0.00	0.00	NaN
1996	ELAVL4	0.00	0.00	-0.03	-0.05
1997	ELF1	-0.05	0.00	-0.05	0.14
1998	ELF2	0.00	0.00	0.00	NaN
2000	ELF4	-0.05	0.00	-0.08	0.20
2001	ELF5	0.04	0.00	-0.03	0.09
2002	ELK1	-0.05	0.00	-0.08	0.16
2004	ELK3	0.00	0.09	0.00	0.03
2005	ELK4	0.07	0.00	0.03	0.04
2006	ELN	0.02	0.00	0.05	0.17
2009	EML1	0.02	0.00	0.03	0.06
2010	EMD	0.00	0.00	-0.03	-0.07
2012	EMP1	0.00	-0.09	0.05	0.30
2013	EMP2	0.04	0.00	0.00	0.15
2014	EMP3	0.00	0.00	-0.03	0.36
2015	EMR1	0.00	-0.09	0.00	0.00
2016	EMX1	0.00	0.00	0.03	-0.03
2018	EMX2	-0.04	0.00	0.00	0.18
2019	EN1	0.00	0.00	0.00	NaN
2020	EN2	0.02	0.00	-0.03	0.01
2021	ENDOG	0.02	0.00	0.00	0.09
2022	ENG	0.00	0.00	0.00	0.11
2023	ENO1	-0.02	0.00	0.00	0.09
2026	ENO2	-0.02	0.09	0.05	0.17
2027	ENO3	-0.04	0.00	0.00	0.07
2028	ENPEP	-0.02	0.00	0.00	0.02

2029	ENSA	0.07	0.09	0.14	0.15
2030	SLC29A1	0.00	0.00	0.11	0.36
2033	EP300	-0.02	0.00	-0.03	0.08
2034	EPAS1	0.00	0.00	0.00	NaN
2035	EPB41	-0.02	0.09	0.00	0.01
2037	EPB41L2	0.00	0.00	0.08	0.13
2038	EPB42	-0.04	0.00	-0.14	0.18
2039	EPB49	-0.15	-0.09	-0.16	-0.03
2041	EPHA1	0.02	0.00	0.00	0.13
2042	EPHA3	-0.04	0.00	0.03	0.04
2043	EPHA4	0.00	0.00	0.00	NaN
2044	EPHA5	-0.02	0.00	0.00	0.09
2045	EPHA7	-0.02	0.00	-0.03	-0.03
2047	EPHB1	0.00	0.00	0.00	NaN
2048	EPHB2	0.00	0.00	0.00	NaN
2049	EPHB3	0.04	0.00	0.03	0.04
2050	EPHB4	0.02	0.00	0.00	0.01
2051	EPHB6	0.02	0.00	0.00	0.01
2052	EPHX1	0.07	0.00	0.03	0.08
2053	EPHX2	-0.09	-0.18	-0.19	0.19
2055	CLN8	-0.18	-0.18	-0.16	0.32
2056	EPO	0.02	0.00	0.00	-0.09
2057	EPOR	0.00	0.00	0.00	NaN
2058	EPRS	0.07	0.00	0.00	0.29
2059	EPS8	0.00	-0.09	0.05	-0.07
2060	EPS15	0.00	0.00	-0.03	-0.07
2063	NR2F6	0.04	0.00	0.00	0.30
2064	ERBB2	0.02	0.91	0.05	0.76
2065	ERBB3	0.04	0.00	0.00	0.26
2066	ERBB4	0.00	0.00	0.00	NaN
2067	ERCC1	-0.02	0.00	-0.03	0.18
2068	ERCC2	-0.02	0.00	-0.03	0.15
2069	EREG	-0.02	0.00	0.00	-0.13
2070	EYA4	-0.02	0.00	0.08	0.08
2071	ERCC3	-0.02	0.00	0.00	0.02
2072	ERCC4	0.02	0.00	0.00	-0.20
2073	ERCC5	0.00	0.00	-0.05	0.02
2074	ERCC6	-0.02	0.00	-0.03	-0.01
2077	ERF	0.02	0.00	-0.03	0.04
2078	ERG	0.00	0.00	0.00	0.08
2079	ERH	-0.02	0.00	0.00	0.15
2081	ERN1	0.15	0.18	0.03	-0.18
2098	ESD	-0.07	0.00	-0.14	0.35
2099	ESR1	0.02	0.00	0.00	0.21
2100	ESR2	-0.02	0.00	0.00	0.07
2101	ESRRA	0.00	0.00	0.03	0.16
2103	ESRRB	-0.02	0.00	0.00	-0.02

2104	ESRRG	0.07	0.00	0.00	0.11
2107	ETF1	0.00	0.00	-0.03	0.14
2108	ETFA	0.00	0.00	-0.05	0.02
2109	ETFB	0.00	0.00	-0.03	0.24
2110	ETFDH	-0.02	0.00	-0.05	0.20
2113	ETS1	-0.05	0.00	-0.03	0.05
2114	ETS2	0.00	0.00	0.00	0.00
2115	ETV1	-0.02	0.00	0.00	0.02
2117	ETV3	0.04	0.00	0.11	0.02
2119	ETV5	0.04	0.00	0.03	0.19
2120	ETV6	0.00	-0.09	0.08	0.34
2121	EVC	-0.02	0.00	0.00	0.00
2122	EVI1	0.02	0.00	0.03	-0.11
2125	EVPL	0.04	0.27	0.03	0.20
2128	EVX1	0.00	0.18	0.00	-0.03
2130	EWSR1	-0.02	0.00	0.03	0.07
2131	EXT1	0.22	0.18	0.35	0.33
2132	EXT2	-0.02	0.00	-0.08	0.40
2134	EXTL1	-0.02	0.00	0.00	0.05
2135	EXTL2	-0.02	0.00	-0.03	-0.02
2137	EXTL3	-0.11	-0.18	-0.19	0.07
2138	EYA1	0.13	0.00	0.14	0.07
2139	EYA2	0.04	0.00	0.03	0.07
2140	EYA3	-0.02	0.09	0.00	0.00
2145	EZH1	-0.04	0.00	0.00	0.23
2146	EZH2	0.02	0.00	0.00	0.08
2147	F2	-0.02	0.09	-0.03	-0.10
2149	F2R	0.00	0.00	-0.14	0.08
2150	F2RL1	0.00	0.00	-0.14	0.16
2152	F3	-0.02	0.00	0.00	-0.04
2153	F5	0.02	0.00	0.05	-0.07
2155	F7	0.00	0.00	0.00	0.25
2157	F8	-0.04	0.00	-0.11	0.11
2158	F9	-0.02	0.09	-0.11	0.00
2159	F10	0.00	0.00	0.00	0.20
2160	F11	-0.02	0.00	-0.08	0.15
2161	F12	0.00	0.00	0.00	NaN
2162	F13A1	-0.02	0.00	0.05	0.03
2165	F13B	0.07	0.00	0.05	-0.22
2166	FAAH	0.00	0.00	0.03	0.20
2167	FABP4	0.13	0.00	0.14	-0.16
2168	FABP1	0.00	0.00	0.00	NaN
2169	FABP2	0.00	0.00	-0.03	-0.15
2170	FABP3	0.02	0.09	0.00	0.07
2171	FABP5	0.13	0.00	0.14	-0.03
2172	FABP6	0.00	0.00	-0.03	0.06
2173	FABP7	-0.02	0.00	0.00	0.10

2175	FANCA	-0.04	0.00	0.00	0.14
2176	FANCC	-0.02	0.00	0.00	0.09
2178	FANCE	0.00	0.00	0.05	0.23
2184	FAH	0.00	0.00	-0.03	0.05
2185	PTK2B	-0.09	-0.18	-0.19	0.28
2188	FANCF	0.00	0.00	-0.05	0.11
2189	FANCG	0.00	0.00	0.00	-0.21
2191	FAP	-0.02	0.00	0.03	0.02
2192	FBLN1	-0.04	0.00	-0.03	0.21
2195	FAT	-0.02	0.00	-0.08	-0.02
2196	FAT2	0.00	0.00	0.00	NaN
2197	FAU	0.02	0.00	0.03	0.12
2199	FBLN2	0.02	0.00	0.00	0.01
2200	FBN1	-0.04	0.00	-0.08	0.05
2201	FBN2	0.00	0.00	-0.05	0.11
2202	EFEMP1	0.00	0.00	0.00	NaN
2203	FBP1	-0.02	0.00	0.00	-0.01
2204	FCAR	0.00	0.09	-0.03	-0.03
2205	FCER1A	0.04	0.00	0.16	-0.17
2206	MS4A1	-0.02	0.00	0.03	0.11
2207	FCER1G	0.11	0.00	0.14	-0.14
2208	FCER2	0.00	-0.09	0.00	0.04
2209	FCGR1A	0.05	0.00	0.14	0.10
2212	FCGR2A	0.07	0.00	0.14	-0.08
2213	FCGR2B	0.07	0.00	0.14	-0.05
2214	FCGR3A	0.07	0.00	0.14	-0.06
2217	FCGRT	0.02	0.00	-0.03	0.11
2218	FCMD	-0.04	0.00	0.00	0.04
2219	FCN1	0.02	0.00	0.03	-0.04
2220	FCN2	0.02	0.00	0.03	-0.05
2222	FDFT1	-0.15	-0.09	-0.22	0.39
2224	FDPS	0.09	0.00	0.14	0.39
2230	FDX1	-0.07	-0.09	0.00	0.10
2232	FDXR	0.05	0.27	0.03	0.27
2235	FECH	-0.02	0.00	-0.03	0.14
2237	FEN1	-0.02	0.00	0.03	0.17
2239	GPC4	-0.04	0.00	-0.08	0.09
2241	FER	0.00	0.00	-0.11	0.04
2242	FES	0.02	0.00	0.03	0.12
2243	FGA	-0.02	0.00	-0.03	0.20
2244	FGB	-0.02	0.00	-0.03	0.03
2245	FGD1	-0.04	0.00	-0.05	-0.17
2247	FGF2	0.00	0.00	-0.03	0.02
2248	FGF3	0.18	0.18	0.03	-0.13
2250	FGF5	-0.04	0.00	-0.03	0.13
2251	FGF6	-0.02	0.09	0.08	-0.03
2253	FGF8	-0.02	0.00	-0.03	-0.02

2254	FGF9	-0.07	0.00	-0.11	0.14
2258	FGF13	-0.02	0.00	-0.08	0.05
2259	FGF14	-0.02	0.00	-0.05	-0.05
2260	FGFR1	0.18	0.09	0.11	0.51
2261	FGFR3	-0.02	0.00	0.00	0.10
2262	GPC5	-0.02	0.00	-0.08	-0.09
2263	FGFR2	-0.04	0.00	0.03	0.25
2264	FGFR4	0.00	0.00	0.00	NaN
2266	FGG	-0.02	0.00	-0.03	0.07
2267	FGL1	-0.16	-0.18	-0.22	0.02
2268	FGR	-0.02	0.00	0.00	-0.03
2271	FH	0.07	0.00	0.05	0.29
2272	FHIT	-0.04	0.00	0.00	0.09
2273	FHL1	-0.04	0.00	-0.08	0.08
2274	FHL2	0.00	0.00	0.00	NaN
2275	FHL3	0.00	0.00	0.00	NaN
2277	FIGF	-0.05	0.00	-0.14	-0.12
2280	FKBP1A	0.02	0.00	-0.03	0.12
2281	FKBP1B	-0.02	0.00	0.00	0.08
2286	FKBP2	0.00	0.00	0.03	0.12
2287	FKBP3	0.00	0.00	-0.05	0.31
2288	FKBP4	-0.02	0.09	0.08	0.15
2289	FKBP5	0.00	0.00	0.05	0.09
2290	FOXG1B	-0.02	0.00	-0.03	0.07
2294	FOXF1	-0.07	0.00	0.00	0.04
2295	FOXF2	-0.04	0.00	0.05	0.19
2297	FOXD1	0.00	0.00	-0.11	0.13
2299	FOXI1	0.00	0.00	-0.03	0.11
2301	FOXE3	0.00	0.00	0.00	0.17
2302	FOXJ1	0.04	0.18	0.03	0.12
2304	FOXE1	-0.02	0.00	0.00	-0.08
2305	FOXM1	-0.02	0.09	0.08	0.23
2306	FOXD2	0.00	0.00	0.00	0.15
2308	FOXO1A	-0.05	0.00	-0.08	0.31
2309	FOXO3A	0.02	0.00	0.00	0.33
2313	FLI1	-0.05	0.00	-0.03	0.26
2314	FLII	-0.05	0.00	0.00	0.23
2315	MLANA	-0.05	0.00	-0.05	0.18
2316	FLNA	0.00	0.00	-0.03	0.37
2317	FLNB	-0.04	0.00	0.00	-0.08
2318	FLNC	0.02	0.00	-0.03	-0.02
2319	FLOT2	0.02	0.09	0.00	0.36
2322	FLT3	-0.05	0.00	-0.05	-0.08
2323	FLT3LG	0.02	0.00	-0.03	0.13
2324	FLT4	0.00	0.00	-0.03	-0.07
2326	FMO1	0.02	0.00	0.05	-0.16
2327	FMO2	0.02	0.00	0.05	0.21

2328	FMO3	0.02	0.00	0.05	0.01
2329	FMO4	0.02	0.00	0.05	0.06
2330	FMO5	0.07	0.00	0.14	0.02
2331	FMOD	0.07	0.00	0.05	0.03
2332	FMR1	-0.05	0.00	-0.11	0.24
2335	FN1	0.00	0.00	0.00	NaN
2339	FNTA	0.02	0.09	0.11	0.31
2342	FNTB	-0.02	0.00	0.00	0.00
2346	FOLH1	-0.02	0.00	0.00	0.07
2348	FOLR1	-0.02	0.09	0.00	0.14
2350	FOLR2	-0.02	0.09	0.00	0.05
2352	FOLR3	-0.02	0.09	0.00	0.02
2353	FOS	-0.02	0.00	0.00	-0.09
2354	FOSB	-0.02	0.00	-0.03	0.08
2355	FOSL2	0.00	0.00	0.00	NaN
2356	FPGS	0.00	0.00	0.00	0.10
2357	FPR1	0.00	-0.09	-0.03	0.12
2358	FPRL1	0.00	-0.09	-0.03	0.15
2359	FPRL2	0.00	-0.09	-0.03	0.09
2444	FRK	0.00	0.00	0.00	0.31
2475	FRAP1	0.00	0.00	0.00	NaN
2483	FRG1	-0.05	-0.09	-0.03	-0.05
2487	FRZB	0.02	0.00	-0.03	0.04
2488	FSHB	0.02	0.00	0.03	0.05
2492	FSHR	0.02	0.00	0.00	-0.01
2494	NR5A2	0.09	0.00	0.03	0.06
2495	FTH1	-0.02	0.09	0.03	0.13
2512	FTL	0.00	0.00	-0.03	0.38
2515	ADAM2	0.09	0.09	0.05	0.09
2516	NR5A1	-0.02	0.00	0.00	-0.02
2517	FUCA1	0.00	0.00	0.00	NaN
2521	FUS	0.04	0.09	0.03	0.12
2523	FUT1	0.00	0.00	-0.03	-0.29
2524	FUT2	0.00	0.00	-0.03	0.07
2525	FUT3	-0.02	-0.09	0.00	0.14
2526	FUT4	-0.07	0.09	0.03	0.21
2527	FUT5	-0.02	-0.09	0.00	-0.01
2528	FUT6	-0.02	-0.09	0.00	0.18
2530	FUT8	-0.02	0.00	0.00	0.06
2531	FVT1	-0.02	-0.09	0.00	0.21
2533	FYB	0.04	0.09	0.00	0.09
2534	FYN	0.02	0.00	0.05	-0.02
2538	G6PC	-0.04	0.00	0.00	-0.05
2539	G6PD	-0.02	0.00	-0.03	0.18
2548	GAA	0.00	0.00	0.00	NaN
2549	GAB1	0.00	0.00	0.00	NaN
2550	GABBR1	0.00	0.00	0.05	0.03

2551	GABPA	0.00	0.00	0.03	0.00
2553	GABPB2	-0.04	0.00	-0.08	-0.01
2554	GABRA1	0.00	0.00	-0.03	0.02
2555	GABRA2	-0.04	0.00	-0.05	-0.09
2556	GABRA3	-0.02	0.00	-0.11	-0.06
2557	GABRA4	-0.04	0.00	0.00	0.03
2558	GABRA5	-0.02	0.09	-0.08	0.23
2559	GABRA6	0.00	0.00	-0.03	0.04
2560	GABRB1	-0.04	0.00	0.00	0.11
2561	GABRB2	0.00	0.00	-0.03	-0.09
2562	GABRB3	-0.02	0.09	-0.08	0.01
2564	GABRE	-0.02	0.00	-0.11	0.14
2566	GABRG2	0.00	0.00	-0.03	-0.07
2567	GABRG3	-0.04	0.09	-0.08	0.11
2568	GABRP	0.00	0.00	-0.03	-0.16
2569	GABRR1	0.02	0.00	0.03	0.03
2570	GABRR2	-0.02	0.00	0.00	0.06
2571	GAD1	0.00	0.00	0.03	0.10
2572	GAD2	-0.02	0.00	0.00	0.21
2580	GAK	-0.09	0.00	-0.03	0.07
2581	GALC	-0.04	0.00	0.00	0.43
2582	GALE	0.00	0.00	0.00	NaN
2584	GALK1	0.05	0.27	0.03	0.40
2585	GALK2	-0.05	0.00	-0.08	0.06
2586	GAL	0.09	0.09	0.03	-0.08
2587	GALR1	-0.02	-0.09	0.00	-0.10
2588	GALNS	-0.04	0.00	0.11	0.14
2589	GALNT1	0.00	-0.09	-0.03	0.07
2590	GALNT2	0.09	0.00	0.03	0.04
2591	GALNT3	-0.02	0.00	0.03	0.11
2592	GALT	0.00	0.00	0.00	0.22
2593	GAMT	0.00	0.00	-0.03	0.17
2596	GAP43	0.00	0.00	-0.03	0.05
2617	GARS	-0.02	0.09	-0.03	0.36
2618	GART	0.00	0.00	0.00	0.17
2619	GAS1	-0.02	0.00	0.00	0.03
2620	GAS2	0.00	0.00	-0.05	-0.05
2621	GAS6	0.00	0.00	0.00	0.17
2623	GATA1	-0.04	0.00	-0.05	-0.08
2624	GATA2	0.00	0.00	-0.03	-0.38
2625	GATA3	0.00	0.00	0.16	-0.14
2626	GATA4	-0.15	-0.09	-0.22	-0.02
2627	GATA6	0.00	-0.09	0.03	0.02
2628	GATM	-0.04	0.00	-0.11	0.08
2629	GBA	0.09	0.00	0.14	0.25
2631	GBAS	0.00	0.00	0.05	0.41
2632	GBE1	-0.05	0.00	-0.03	0.18

2633	GBP1	0.00	0.00	-0.03	0.01
2634	GBP2	0.00	0.00	-0.03	0.01
2637	GBX2	0.00	0.00	0.00	NaN
2638	GC	-0.02	0.00	0.00	-0.10
2639	GCDH	0.00	-0.09	0.00	0.05
2641	GCG	-0.02	0.00	0.03	-0.22
2643	GCH1	0.00	0.00	0.00	0.24
2644	GCHFR	-0.02	0.00	-0.14	0.10
2645	GCK	-0.02	0.00	0.08	0.15
2646	GCKR	0.00	0.00	0.00	NaN
2648	GCN5L2	-0.04	0.09	0.00	0.28
2649	NR6A1	-0.02	0.00	0.00	0.06
2650	GCNT1	-0.02	0.00	0.00	0.01
2651	GCNT2	-0.02	0.00	0.11	0.23
2653	GCSH	-0.09	0.00	0.00	0.15
2657	GDF1	0.02	0.00	0.00	0.10
2658	GDF2	-0.02	0.00	0.00	0.08
2660	GDF8	0.00	0.00	0.00	NaN
2661	GDF9	0.00	0.00	-0.03	0.05
2662	GDF10	-0.02	0.00	0.00	-0.01
2664	GDI1	0.00	0.00	-0.03	0.09
2665	GDI2	0.00	0.00	0.11	0.25
2668	GDNF	0.04	0.18	0.00	0.03
2669	GEM	0.16	0.09	0.22	0.19
2670	GFAP	-0.05	0.00	0.00	0.14
2671	GFER	0.04	0.18	0.00	0.22
2672	GFI1	-0.02	0.00	-0.03	0.06
2673	GFPT1	0.00	0.00	0.00	NaN
2674	GFRA1	-0.04	0.00	0.03	-0.13
2675	GFRA2	-0.13	-0.18	-0.22	-0.04
2676	GFRA3	0.00	0.00	-0.03	0.02
2677	GGCX	0.02	0.00	0.00	0.04
2678	GGT1	0.00	0.00	0.03	0.12
2683	B4GALT1	-0.04	0.09	0.00	0.21
2687	GGTLA1	0.00	0.00	0.05	0.11
2690	GHR	0.02	0.09	0.00	0.21
2691	GHRH	0.02	0.00	0.03	-0.20
2692	GHRHR	-0.02	0.09	-0.03	-0.11
2693	GHSR	0.04	0.00	0.00	-0.10
2694	GIF	-0.04	0.00	0.00	0.04
2695	GIP	0.07	0.18	0.00	0.19
2696	GIPR	-0.02	0.00	0.00	-0.01
2697	GJA1	-0.02	0.00	0.03	0.04
2700	GJA3	-0.09	0.00	-0.08	0.00
2702	GJA5	0.07	0.00	0.11	0.02
2705	GJB1	-0.04	0.00	-0.08	-0.01
2710	GK	-0.07	0.00	-0.11	0.30

2717	GLA	-0.05	0.00	-0.08	0.16
2719	GPC3	-0.04	0.00	-0.08	0.00
2720	GLB1	0.02	0.00	-0.03	0.29
2729	GCLC	-0.02	0.00	0.08	0.14
2730	GCLM	-0.02	0.00	0.00	0.16
2731	GLDC	-0.05	0.00	-0.05	0.09
2733	GLE1L	0.02	0.00	0.00	0.13
2734	GLG1	-0.09	0.00	0.00	0.23
2736	GLI2	0.00	0.00	0.00	NaN
2737	GLI3	-0.02	0.00	0.03	0.02
2739	GLO1	0.02	0.00	0.05	0.08
2740	GLP1R	0.02	0.00	0.05	0.09
2741	GLRA1	0.00	0.00	0.00	NaN
2742	GLRA2	-0.05	0.00	-0.14	-0.13
2743	GLRB	-0.02	0.00	-0.05	0.15
2744	GLS	0.00	0.00	0.00	NaN
2745	GLRX	0.00	0.00	-0.14	0.09
2746	GLUD1	-0.02	0.00	-0.03	0.26
2752	GLUL	0.04	0.00	0.03	0.21
2760	GM2A	0.00	0.00	0.00	NaN
2762	GMDS	-0.02	0.00	0.00	0.46
2764	GMFB	0.00	0.00	0.00	0.26
2765	GML	0.16	0.09	0.38	0.06
2766	GMPR	0.00	0.00	0.05	0.04
2767	GNA11	0.00	0.00	0.00	NaN
2769	GNA15	0.00	0.00	0.00	NaN
2770	GNAI1	0.02	0.00	0.03	0.25
2771	GNAI2	0.00	0.00	0.03	0.11
2773	GNAI3	0.00	0.00	-0.03	0.07
2774	GNAL	-0.02	-0.09	0.00	0.12
2775	GNAO1	-0.05	0.00	0.00	0.12
2776	GNAQ	-0.02	0.00	0.00	0.08
2778	GNAS	0.05	0.09	0.03	0.19
2779	GNAT1	0.00	0.00	0.03	-0.04
2780	GNAT2	0.00	0.00	-0.03	0.00
2782	GNB1	-0.02	0.00	0.03	0.10
2783	GNB2	0.09	0.00	0.08	0.11
2784	GNB3	-0.02	0.09	0.05	0.00
2785	GNG3	0.00	0.00	0.03	0.03
2786	GNG4	0.09	0.00	0.03	0.05
2787	GNG5	-0.02	0.00	-0.03	0.08
2788	GNG7	0.00	0.00	0.00	NaN
2791	GNG11	0.02	0.00	0.00	0.11
2792	GNGT1	0.02	0.00	0.00	0.24
2794	GNL1	0.00	0.00	0.05	0.15
2796	GNRH1	-0.11	-0.18	-0.19	0.23
2797	GNRH2	0.02	0.00	-0.03	0.00

2798	GNRHR	-0.02	0.00	0.00	0.05
2799	GNS	0.00	0.00	0.00	NaN
2800	GOLGA1	-0.02	0.00	0.00	-0.01
2801	GOLGA2	0.02	0.00	0.00	0.11
2802	GOLGA3	0.00	0.00	0.00	NaN
2803	GOLGA4	0.02	0.00	0.00	0.35
2804	GOLGB1	0.00	0.00	-0.03	0.06
2805	GOT1	-0.02	0.00	-0.03	0.18
2806	GOT2	-0.07	0.00	0.00	0.32
2810	SFN	-0.02	0.00	0.00	0.03
2811	GP1BA	-0.04	0.00	0.00	-0.02
2813	GP2	0.02	-0.09	0.00	0.28
2814	GP5	0.02	0.09	0.00	0.02
2815	GP9	0.00	0.00	-0.03	-0.22
2817	GPC1	0.00	0.00	0.00	NaN
2819	GPD1	0.02	0.00	-0.03	0.04
2820	GPD2	-0.02	0.00	0.03	0.08
2821	GPI	0.00	0.00	0.05	0.15
2822	GPLD1	0.00	0.00	0.03	0.01
2823	GPM6A	-0.02	0.00	-0.05	-0.07
2824	GPM6B	-0.05	0.00	-0.14	-0.05
2825	GPR1	0.00	0.00	0.00	NaN
2827	GPR3	-0.02	0.00	0.00	0.02
2828	GPR4	-0.02	0.00	0.00	0.18
2835	GPR12	-0.05	0.00	-0.08	-0.04
2838	GPR15	0.00	0.00	-0.03	-0.08
2842	GPR19	0.00	-0.09	0.05	0.07
2843	GPR20	0.16	0.00	0.41	0.16
2846	GPR23	-0.04	0.00	-0.08	-0.19
2850	GPR27	-0.05	0.00	0.00	0.02
2854	GPR32	0.00	0.00	-0.03	-0.25
2859	GPR35	0.00	0.00	0.00	NaN
2861	GPR37	0.00	0.00	-0.03	0.17
2863	GPR39	-0.02	0.00	0.00	0.03
2874	GPS2	-0.05	0.00	0.00	0.10
2876	GPX1	0.00	0.00	0.00	NaN
2877	GPX2	-0.02	0.00	0.00	-0.04
2878	GPX3	0.00	0.00	0.00	NaN
2879	GPX4	0.00	0.00	-0.03	0.15
2880	GPX5	0.00	0.00	0.05	0.02
2885	GRB2	0.05	0.18	0.05	0.37
2886	GRB7	0.02	0.82	0.05	0.66
2887	GRB10	-0.05	0.00	0.03	0.23
2888	GRB14	-0.02	0.00	0.03	0.10
2890	GRIA1	0.00	0.00	0.00	NaN
2891	GRIA2	-0.02	0.00	-0.05	0.02
2892	GRIA3	-0.05	0.00	-0.08	0.03

2893	GRIA4	-0.07	-0.09	0.00	-0.03
2895	GRID2	-0.04	0.00	-0.03	0.08
2896	GRN	-0.05	0.00	0.00	0.29
2897	GRIK1	0.00	0.00	0.00	-0.02
2899	GRIK3	-0.02	0.00	0.00	0.09
2900	GRIK4	-0.07	0.00	0.00	-0.05
2901	GRIK5	0.02	0.00	-0.03	0.06
2902	GRIN1	0.02	0.00	0.03	0.16
2903	GRIN2A	0.04	0.00	0.00	-0.12
2904	GRIN2B	0.00	-0.09	0.05	0.08
2905	GRIN2C	0.05	0.27	0.03	-0.04
2906	GRIN2D	0.00	0.00	-0.03	-0.11
2908	NR3C1	0.00	0.00	0.00	NaN
2909	GRLF1	-0.04	0.00	-0.03	0.09
2911	GRM1	0.02	0.00	0.00	0.14
2912	GRM2	0.00	0.00	0.00	NaN
2913	GRM3	0.02	0.00	0.00	0.11
2914	GRM4	0.00	0.00	0.05	0.01
2915	GRM5	-0.05	0.00	0.00	-0.03
2916	GRM6	0.00	0.00	-0.03	-0.01
2917	GRM7	0.00	0.00	0.00	0.23
2918	GRM8	0.02	0.00	-0.03	0.14
2922	GRP	-0.02	-0.09	-0.03	-0.02
2925	GRPR	-0.05	0.00	-0.11	0.17
2926	GRSF1	-0.02	0.00	0.00	0.23
2931	GSK3A	0.02	0.00	-0.03	-0.20
2932	GSK3B	0.00	0.00	-0.03	-0.12
2934	GSN	-0.02	0.00	0.00	0.10
2935	GSPT1	0.04	0.00	0.00	0.14
2936	GSR	-0.09	-0.18	-0.19	0.30
2937	GSS	0.05	0.00	0.00	0.34
2940	GSTA3	-0.02	0.00	0.08	0.01
2941	GSTA4	-0.02	0.00	0.08	0.27
2944	GSTM1	0.00	0.00	-0.03	0.06
2946	GSTM2	0.00	0.00	-0.03	0.05
2947	GSTM3	0.00	0.00	-0.03	-0.03
2948	GSTM4	0.00	0.00	-0.03	0.08
2949	GSTM5	0.00	0.00	-0.03	-0.07
2950	GSTP1	0.07	0.09	0.03	-0.03
2952	GSTT1	0.00	0.00	0.05	-0.04
2954	GSTZ1	-0.02	0.00	-0.03	0.17
2956	MSH6	0.02	0.00	0.00	0.21
2957	GTF2A1	0.00	0.00	0.00	0.01
2958	GTF2A2	-0.02	0.00	-0.05	0.34
2960	GTF2E1	0.00	0.00	-0.03	-0.11
2961	GTF2E2	-0.09	-0.18	-0.19	0.17
2962	GTF2F1	0.00	-0.09	0.00	0.13

2963	GTF2F2	-0.05	0.00	-0.11	0.04
2965	GTF2H1	0.00	0.00	-0.05	0.05
2967	GTF2H3	0.02	0.00	0.00	0.27
2968	GTF2H4	0.02	0.00	0.05	0.25
2972	BRF1	-0.02	0.00	0.08	0.05
2975	GTF3C1	0.02	0.00	0.00	0.18
2976	GTF3C2	0.00	0.00	0.00	NaN
2977	GUCY1A2	-0.07	-0.09	0.00	-0.03
2979	GUCA1B	0.00	0.00	0.08	-0.19
2981	GUCA2B	0.00	0.00	0.00	NaN
2982	GUCY1A3	-0.02	0.00	-0.03	0.09
2983	GUCY1B3	-0.02	0.00	-0.03	0.18
2984	GUCY2C	0.00	-0.09	0.05	0.09
2986	GUCY2F	-0.05	0.00	-0.08	-0.04
2987	GUK1	0.09	0.00	0.03	0.04
2990	GUSB	0.00	0.00	0.05	0.13
2993	GYPA	0.00	0.00	0.00	NaN
2995	GYPC	0.00	0.00	0.00	NaN
2997	GYS1	0.00	0.00	-0.03	-0.27
2998	GYS2	0.00	-0.09	0.05	-0.12
3000	GUCY2D	-0.05	0.00	0.00	0.06
3001	GZMA	0.00	0.00	-0.11	0.02
3003	GZMK	0.00	0.00	-0.11	-0.02
3004	GZMM	0.00	0.00	-0.03	0.01
3005	H1F0	-0.04	0.00	0.03	0.06
3014	H2AFX	-0.05	0.00	0.00	0.23
3015	H2AFZ	-0.02	0.00	0.03	0.11
3021	H3F3B	0.05	0.27	0.03	0.43
3026	HABP2	-0.04	0.00	0.00	0.02
3029	HAGH	0.04	0.18	0.00	0.37
3030	HADHA	-0.02	0.00	0.00	0.09
3032	HADHB	-0.02	0.00	0.00	0.04
3034	HAL	0.00	0.09	0.00	0.07
3035	HARS	0.00	0.00	0.00	NaN
3036	HAS1	0.00	-0.09	-0.03	-0.15
3037	HAS2	0.18	0.18	0.35	0.07
3043	HBB	-0.02	-0.09	-0.03	0.07
3045	HBD	-0.02	0.00	-0.03	0.08
3046	HBE1	-0.02	0.00	-0.03	0.13
3052	HCCS	-0.05	-0.09	-0.14	0.15
3054	HCFC1	0.00	0.00	-0.03	0.15
3055	HCK	0.00	0.00	0.00	NaN
3059	HCLS1	0.00	0.00	-0.03	0.03
3060	HCRT	-0.04	0.00	0.00	0.08
3061	HCRTR1	0.02	0.09	0.00	-0.05
3062	HCRTR2	-0.04	0.00	0.08	-0.13
3064	HD	-0.02	0.00	0.00	0.28

3065	HDAC1	0.00	0.00	0.00	NaN
3066	HDAC2	0.02	0.00	0.03	0.33
3067	HDC	-0.04	0.00	-0.08	0.15
3068	HDGF	0.04	0.00	0.11	0.30
3069	HDLBP	0.00	0.00	0.00	NaN
3073	HEXA	0.00	0.00	-0.05	-0.01
3074	HEXB	0.00	0.09	-0.14	0.28
3077	HFE	0.00	0.00	0.03	-0.01
3082	HGF	0.02	0.00	0.00	0.14
3083	HGFAC	-0.02	0.00	0.00	-0.16
3084	NRG1	-0.11	0.09	-0.14	0.08
3087	HHEX	-0.02	0.00	-0.05	0.15
3091	HIF1A	0.00	0.00	0.00	0.27
3092	HIP1	0.02	0.00	0.08	0.01
3093	HIP2	-0.05	0.00	-0.03	0.22
3094	HINT1	0.00	0.00	-0.03	0.04
3096	HIVEP1	0.00	0.00	0.11	-0.02
3098	HK1	0.00	0.00	0.03	0.15
3099	HK2	0.00	0.00	0.00	NaN
3101	HK3	0.00	0.00	-0.03	0.06
3109	HLA-DMB	0.00	0.00	0.05	0.07
3110	HLXB9	0.02	0.00	-0.03	0.23
3111	HLA-DOA	0.00	0.00	0.05	0.12
3112	HLA-DOB	0.00	0.00	0.05	0.18
3113	HLA-DPA1	0.00	0.00	0.05	0.10
3115	HLA-DPB1	0.00	0.00	0.05	0.02
3122	HLA-DRA	0.00	0.00	0.05	0.12
3123	HLA-DRB1	-0.02	0.00	0.05	0.15
3127	HLA-DRB5	-0.02	0.00	0.05	0.14
3131	HLF	0.11	0.27	0.03	0.05
3134	HLA-F	0.00	0.00	0.08	0.18
3141	HLCS	0.00	0.00	0.00	0.11
3145	HMBS	-0.05	0.00	0.00	0.21
3155	HMGCL	0.00	0.00	0.00	NaN
3156	HMGCR	0.00	0.09	-0.14	0.16
3157	HMGCS1	0.04	0.09	0.00	0.26
3158	HMGCS2	0.00	0.00	0.00	-0.05
3161	HMMR	0.00	0.00	-0.03	0.05
3162	HMOX1	-0.02	0.00	0.03	0.13
3163	HMOX2	0.04	0.00	0.00	0.15
3164	NR4A1	0.02	0.00	0.00	0.11
3172	HNF4A	0.02	0.09	0.03	0.02
3174	HNF4G	0.09	0.00	0.08	0.26
3175	ONECUT1	-0.02	0.00	-0.05	-0.05
3176	HNMT	-0.02	0.00	0.00	0.06
3177	SLC29A2	0.07	0.00	0.03	-0.01
3178	HNRPA1	0.02	0.00	-0.03	0.27

3181	HNRPA2B1	0.00	0.18	0.03	0.15
3182	HNRPAB	0.00	0.00	0.00	NaN
3183	HNRPC	-0.02	0.00	0.00	0.05
3184	HNRPD	-0.04	0.00	-0.03	0.19
3185	HNRPF	0.00	0.09	-0.03	0.06
3187	HNRPH1	0.00	0.00	-0.03	0.15
3188	HNRPH2	-0.05	0.00	-0.08	0.29
3189	HNRPH3	-0.02	0.00	-0.03	0.15
3190	HNRPK	-0.02	0.00	0.00	0.03
3191	HNRPL	0.02	0.09	0.05	0.22
3192	HNRPU	0.07	0.00	0.03	-0.03
3198	HOXA1	0.00	0.09	0.03	0.03
3199	HOXA2	0.00	0.18	0.03	0.22
3200	HOXA3	0.00	0.18	0.03	-0.14
3201	HOXA4	0.00	0.18	0.03	0.01
3202	HOXA5	0.00	0.18	0.00	0.03
3203	HOXA6	0.00	0.27	0.00	0.02
3204	HOXA7	0.00	0.27	0.00	-0.04
3205	HOXA9	0.00	0.18	0.00	0.18
3206	HOXA10	0.00	0.18	0.00	0.17
3207	HOXA11	0.00	0.18	0.00	0.29
3208	HPCA	0.00	0.00	0.00	NaN
3211	HOXB1	0.02	0.09	0.00	0.15
3212	HOXB2	0.02	0.09	0.00	0.11
3213	HOXB3	0.02	0.09	0.00	0.12
3215	HOXB5	0.04	0.09	0.00	0.13
3216	HOXB6	0.05	0.09	0.00	-0.03
3217	HOXB7	0.05	0.09	0.00	0.19
3218	HOXB8	0.05	0.09	0.00	-0.14
3221	HOXC4	0.02	0.00	-0.03	0.07
3224	HOXC8	0.02	0.00	-0.03	0.03
3226	HOXC10	0.02	0.00	-0.03	-0.13
3227	HOXC11	0.02	0.00	-0.03	-0.19
3229	HOXC13	0.02	0.00	-0.03	0.01
3231	HOXD1	0.02	0.00	0.00	-0.01
3232	HOXD3	0.02	0.00	0.00	0.03
3233	HOXD4	0.02	0.00	0.00	0.07
3235	HOXD9	0.02	0.00	0.00	0.05
3236	HOXD10	0.02	0.00	0.00	0.05
3237	HOXD11	0.00	0.18	0.05	-0.05
3238	HOXD12	-0.04	0.18	0.05	0.01
3239	HOXD13	0.00	0.00	0.00	0.17
3240	HP	-0.07	0.00	0.00	0.02
3241	HPCAL1	-0.02	0.00	0.00	0.13
3242	HPD	0.02	0.00	0.00	0.02
3248	HPGD	-0.02	0.00	-0.05	0.16
3249	HPN	0.00	0.00	0.08	0.00

3250	HPR	-0.07	0.00	0.00	0.09
3251	HPRT1	-0.04	0.00	-0.08	-0.02
3257	HPS1	-0.02	0.00	-0.03	0.19
3263	HPX	-0.02	0.00	-0.03	0.12
3265	HRAS	0.04	0.00	0.08	0.06
3267	HRB	0.00	0.00	0.00	NaN
3269	HRH1	-0.02	0.00	0.00	0.09
3270	HRC	0.00	0.00	-0.03	-0.10
3273	HRG	0.04	0.00	0.03	0.02
3274	HRH2	0.00	0.00	-0.03	0.01
3281	HSBP1	-0.09	0.00	0.00	0.05
3283	HSD3B1	0.00	0.00	0.00	0.04
3284	HSD3B2	0.00	0.00	0.00	-0.05
3290	HSD11B1	0.07	0.00	0.00	-0.03
3291	HSD11B2	-0.04	0.00	0.00	-0.09
3293	HSD17B3	-0.02	0.00	0.00	-0.14
3294	HSD17B2	-0.09	0.00	0.00	0.12
3295	HSD17B4	0.00	0.00	0.00	NaN
3297	HSF1	0.16	0.00	0.35	0.34
3298	HSF2	-0.02	0.00	0.00	0.02
3299	HSF4	-0.04	0.00	0.00	0.01
3300	DNAJB2	0.00	0.00	0.00	NaN
3301	DNAJA1	-0.04	0.09	0.00	-0.09
3303	HSPA1A	-0.04	0.00	0.05	0.12
3305	HSPA1L	0.00	0.00	0.05	-0.03
3306	HSPA2	-0.02	0.00	0.00	-0.17
3308	HSPA4	0.00	0.00	-0.03	0.02
3309	HSPA5	-0.02	0.00	0.00	0.04
3312	HSPA8	-0.05	0.00	-0.03	0.12
3315	HSPB1	0.02	0.00	0.05	0.06
3316	HSPB2	-0.07	0.00	0.00	0.15
3321	IGSF3	0.02	0.00	-0.03	0.28
3329	HSPD1	0.02	0.00	0.00	0.21
3336	HSPE1	0.02	0.00	0.00	0.20
3337	DNAJB1	0.02	-0.09	0.00	0.38
3338	DNAJC4	0.00	0.00	0.03	0.09
3339	HSPG2	0.00	0.00	0.00	NaN
3340	NDST1	0.00	0.00	0.00	NaN
3346	HTN1	-0.02	0.00	0.00	0.00
3347	HTN3	-0.02	0.00	0.00	0.06
3350	HTR1A	0.00	0.00	-0.11	0.10
3351	HTR1B	-0.02	0.00	-0.03	-0.08
3352	HTR1D	0.02	0.00	0.00	0.00
3354	HTR1E	-0.02	0.00	-0.08	0.03
3355	HTR1F	-0.05	0.00	0.03	0.15
3356	HTR2A	-0.07	0.00	-0.14	0.00
3358	HTR2C	-0.05	0.00	-0.05	0.12

3359	HTR3A	-0.07	0.00	0.00	0.11
3360	HTR4	0.00	0.00	0.00	NaN
3361	HTR5A	0.02	0.00	-0.03	0.02
3363	HTR7	-0.02	0.00	-0.05	0.23
3364	HUS1	-0.04	0.00	0.03	0.23
3373	HYAL1	0.00	0.00	0.00	NaN
3376	IARS	-0.02	0.00	0.00	0.02
3381	IBSP	-0.04	0.00	-0.03	-0.12
3382	ICA1	0.00	0.00	0.03	0.10
3383	ICAM1	0.00	0.00	0.00	NaN
3384	ICAM2	0.15	0.18	0.03	-0.12
3385	ICAM3	0.00	0.00	0.00	NaN
3386	ICAM4	0.00	0.00	0.00	NaN
3396	ICT1	0.05	0.27	0.05	0.37
3397	ID1	0.00	0.00	0.03	0.18
3398	ID2	-0.02	0.00	0.00	0.07
3399	ID3	0.05	0.00	0.05	-0.02
3400	ID4	0.00	0.00	0.08	0.35
3416	IDE	-0.02	0.00	-0.05	0.34
3417	IDH1	0.00	0.00	0.00	NaN
3418	IDH2	0.02	0.00	-0.03	0.18
3419	IDH3A	0.00	0.00	-0.05	-0.01
3420	IDH3B	0.02	0.00	-0.03	0.08
3421	IDH3G	0.00	0.00	-0.03	0.17
3422	IDI1	0.00	0.00	0.14	0.26
3423	IDS	-0.05	0.00	-0.08	0.06
3425	IDUA	0.02	0.09	0.03	0.06
3428	IFI16	0.04	0.00	0.16	0.06
3429	IFI27	-0.02	0.00	0.00	0.04
3430	IFI35	-0.04	0.00	0.00	0.24
3431	SP110	-0.02	0.00	0.00	0.12
3434	IFIT1	-0.04	0.00	-0.11	0.17
3440	IFNA2	-0.13	0.00	-0.05	0.07
3445	IFNA8	-0.13	0.00	-0.05	-0.06
3454	IFNAR1	0.00	0.00	-0.03	0.10
3455	IFNAR2	0.00	0.00	-0.03	0.13
3456	IFNB1	-0.13	0.00	-0.05	-0.05
3458	IFNG	0.02	0.00	0.00	-0.02
3459	IFNGR1	-0.02	0.00	0.05	0.15
3460	IFNGR2	0.00	0.00	0.00	0.28
3467	IFNW1	-0.13	0.00	-0.05	0.03
3475	IFRD1	0.00	0.00	-0.03	0.30
3476	IGBP1	-0.04	0.00	-0.08	0.27
3479	IGF1	0.00	0.00	0.03	0.03
3480	IGF1R	0.05	0.00	0.00	0.31
3481	IGF2	0.00	0.00	0.00	NaN
3482	IGF2R	-0.02	0.00	-0.05	0.41

3483	IGFALS	0.04	0.18	0.00	0.06
3484	IGFBP1	-0.04	0.00	0.03	0.09
3485	IGFBP2	0.02	0.00	0.00	0.10
3486	IGFBP3	-0.04	0.00	0.03	-0.06
3487	IGFBP4	0.02	0.18	0.00	0.09
3488	IGFBP5	0.02	0.00	0.00	0.26
3489	IGFBP6	0.02	0.00	-0.03	-0.07
3490	IGFBP7	-0.02	0.00	0.00	0.11
3491	CYR61	-0.02	0.00	-0.03	0.04
3508	IGHMBP2	0.15	0.09	0.03	-0.02
3543	IGLL1	-0.02	0.00	0.03	-0.01
3547	IGSF1	-0.04	0.00	-0.08	-0.04
3550	IK	0.00	0.00	0.00	NaN
3551	IKBKB	0.02	0.09	0.11	0.28
3552	IL1A	0.00	0.00	0.00	NaN
3553	IL1B	0.00	0.00	0.00	NaN
3554	IL1R1	0.02	0.00	0.00	-0.08
3556	IL1RAP	0.02	0.00	0.00	0.10
3557	IL1RN	0.00	0.00	0.00	NaN
3558	IL2	0.00	0.00	-0.03	0.03
3559	IL2RA	0.00	0.00	0.11	0.10
3560	IL2RB	-0.04	0.00	0.03	0.20
3561	IL2RG	-0.04	0.00	-0.08	0.18
3562	IL3	0.00	0.00	-0.03	-0.02
3565	IL4	0.00	0.00	-0.03	0.09
3566	IL4R	0.02	0.00	0.00	0.09
3567	IL5	0.00	0.00	-0.03	0.12
3568	IL5RA	-0.02	0.00	0.00	0.04
3569	IL6	-0.02	0.09	0.03	0.05
3570	IL6R	0.05	0.00	0.14	-0.01
3572	IL6ST	0.00	0.00	-0.11	0.17
3574	IL7	0.11	0.09	0.11	0.18
3575	IL7R	0.02	0.27	0.00	-0.15
3576	IL8	-0.02	0.00	0.00	0.04
3577	IL8RA	0.02	0.00	0.00	0.02
3578	IL9	0.00	0.00	-0.03	-0.04
3579	IL8RB	0.02	0.00	0.00	0.09
3586	IL10	0.09	0.00	0.00	-0.15
3587	IL10RA	-0.05	0.00	0.00	0.24
3588	IL10RB	0.00	0.00	-0.03	0.10
3589	IL11	0.00	0.09	-0.03	0.09
3590	IL11RA	0.00	0.00	0.00	0.11
3592	IL12A	0.02	0.00	0.03	0.01
3593	IL12B	0.00	0.00	-0.03	-0.07
3594	IL12RB1	0.02	0.00	0.00	0.04
3595	IL12RB2	-0.02	0.00	0.00	0.12
3596	IL13	0.00	0.00	-0.03	0.02

3597	IL13RA1	-0.05	0.00	-0.08	0.08
3598	IL13RA2	-0.05	0.00	-0.05	-0.04
3600	IL15	0.00	0.00	0.00	NaN
3601	IL15RA	0.00	0.00	0.11	-0.04
3603	IL16	0.00	0.00	-0.03	-0.20
3604	TNFRSF9	-0.02	0.00	0.00	0.20
3606	IL18	-0.07	0.00	0.00	0.19
3608	ILF2	0.07	0.00	0.16	0.33
3609	ILF3	0.02	0.00	0.00	0.26
3611	ILK	-0.02	0.00	-0.03	0.05
3612	IMPA1	0.13	0.00	0.14	0.06
3613	IMPA2	-0.02	-0.09	0.00	-0.02
3614	IMPDH1	0.02	0.00	0.00	0.11
3615	IMPDH2	0.00	0.00	0.00	NaN
3617	IMPG1	-0.02	0.00	-0.03	-0.03
3619	INCENP	-0.02	0.00	0.03	0.13
3620	INDO	0.09	0.18	0.08	0.15
3621	ING1	0.00	0.00	-0.03	0.29
3623	INHA	0.00	0.00	0.00	NaN
3624	INHBA	-0.02	0.00	0.03	0.01
3625	INHBB	0.02	0.00	0.00	0.14
3628	INPP1	0.00	0.00	0.00	NaN
3631	INPP4A	0.00	0.00	0.00	NaN
3632	INPP5A	-0.02	0.00	0.03	0.06
3635	INPP5D	0.00	0.00	0.00	NaN
3636	INPPL1	-0.02	0.09	0.00	0.25
3638	INSIG1	0.02	0.00	-0.03	0.06
3640	INSL3	0.02	0.00	0.00	-0.01
3641	INSL4	-0.05	0.00	-0.05	0.05
3642	INSM1	0.00	0.00	-0.08	-0.04
3643	INSR	0.00	-0.09	0.00	0.13
3646	EIF3S6	0.18	0.09	0.27	0.34
3652	IPP	0.00	0.00	0.00	NaN
3654	IRAK1	0.00	0.00	-0.03	0.38
3655	ITGA6	0.00	0.00	0.00	0.04
3659	IRF1	0.00	0.00	-0.03	0.05
3660	IRF2	-0.02	0.00	-0.05	0.02
3661	IRF3	0.07	0.09	-0.03	0.26
3662	IRF4	-0.04	0.00	-0.03	0.09
3663	IRF5	0.02	0.00	0.00	0.28
3667	IRS1	0.00	0.00	0.00	NaN
3669	ISG20	0.00	0.00	-0.05	-0.02
3670	ISL1	0.00	0.00	-0.03	-0.04
3671	ISLR	0.00	0.00	0.08	0.10
3673	ITGA2	0.00	0.00	-0.08	0.05
3674	ITGA2B	-0.05	0.00	0.00	0.25
3675	ITGA3	0.13	0.18	0.03	0.26

3676	ITGA4	0.00	0.00	0.00	NaN
3678	ITGA5	0.02	0.00	-0.03	-0.07
3679	ITGA7	0.02	0.00	0.00	0.00
3680	ITGA9	0.00	0.00	0.00	0.06
3682	ITGAE	-0.04	0.00	0.00	0.32
3684	ITGAM	0.04	0.09	0.03	0.01
3685	ITGAV	0.02	0.00	0.00	0.23
3687	ITGAX	0.04	0.00	0.03	-0.04
3688	ITGB1	-0.02	0.00	0.03	0.34
3689	ITGB2	0.00	0.00	0.00	0.20
3690	ITGB3	-0.04	0.00	0.00	0.20
3691	ITGB4	0.05	0.27	0.03	0.31
3692	ITGB4BP	0.05	0.00	0.00	0.57
3693	ITGB5	0.00	0.00	-0.03	0.26
3694	ITGB6	0.00	0.00	0.03	0.12
3695	ITGB7	0.02	0.00	-0.03	-0.12
3696	ITGB8	-0.02	0.00	0.03	0.04
3698	ITIH2	0.00	0.00	0.14	0.03
3699	ITIH3	-0.02	0.00	-0.03	-0.09
3700	ITIH4	-0.02	0.00	-0.03	0.09
3702	ITK	0.00	0.00	0.00	NaN
3704	ITPA	0.02	0.00	-0.03	0.06
3705	ITPK1	-0.02	0.00	0.03	0.17
3706	ITPKA	-0.02	0.09	-0.14	0.08
3707	ITPKB	0.07	0.00	0.03	0.04
3708	ITPR1	-0.02	0.00	0.00	-0.02
3709	ITPR2	0.00	-0.09	0.05	0.05
3710	ITPR3	0.00	0.00	0.05	0.11
3712	IVD	0.00	0.00	-0.14	0.24
3713	IVL	0.07	0.00	0.16	-0.02
3714	JAG2	0.00	0.00	0.08	0.03
3716	JAK1	-0.02	0.00	0.00	0.07
3717	JAK2	-0.05	0.00	-0.05	0.26
3718	JAK3	0.02	0.00	0.00	-0.03
3725	JUN	0.00	0.00	0.03	0.29
3726	JUNB	0.02	-0.09	0.00	0.20
3727	JUND	0.00	0.00	0.00	0.19
3728	JUP	0.02	0.18	0.00	0.22
3730	KAL1	-0.05	-0.09	-0.14	0.05
3735	KARS	-0.09	0.00	0.03	0.33
3736	KCNA1	-0.02	0.09	0.05	-0.03
3737	KCNA2	0.00	0.00	-0.03	0.11
3738	KCNA3	0.00	0.00	-0.03	-0.05
3739	KCNA4	0.02	0.00	0.03	-0.06
3741	KCNA5	-0.02	0.09	0.05	-0.06
3742	KCNA6	-0.02	0.09	0.08	0.04
3744	KCNA10	0.00	0.00	-0.03	0.02

3745	KCNB1	0.05	0.00	0.05	0.00
3746	KCNC1	0.00	0.00	-0.05	0.06
3748	KCNC3	0.00	0.00	-0.03	-0.20
3749	KCNC4	0.00	0.00	-0.03	0.09
3750	KCND1	-0.04	0.00	-0.05	0.03
3751	KCND2	0.00	0.00	-0.03	0.01
3752	KCND3	0.02	0.00	-0.03	0.01
3753	KCNE1	0.00	0.00	0.00	0.13
3755	KCNG1	0.07	0.09	0.05	-0.05
3756	KCNH1	0.07	0.00	0.00	-0.03
3757	KCNH2	0.02	0.09	0.00	0.17
3758	KCNJ1	-0.05	0.00	-0.03	0.01
3759	KCNJ2	0.02	0.09	0.03	-0.01
3760	KCNJ3	-0.02	0.00	0.03	0.03
3761	KCNJ4	-0.04	0.00	-0.03	0.13
3762	KCNJ5	-0.05	0.00	-0.03	0.20
3763	KCNJ6	0.00	0.00	0.00	-0.06
3764	KCNJ8	0.00	-0.09	0.05	0.01
3765	KCNJ9	0.05	0.00	0.14	0.01
3766	KCNJ10	0.07	0.00	0.14	0.00
3768	KCNJ12	-0.04	0.18	0.03	0.25
3770	KCNJ14	0.00	0.00	-0.03	-0.22
3772	KCNJ15	0.00	0.00	0.00	0.07
3773	KCNJ16	0.02	0.18	0.03	0.10
3775	KCNK1	0.11	0.00	0.03	0.24
3776	KCNK2	0.09	0.00	0.00	-0.11
3777	KCNK3	-0.02	0.00	0.00	0.11
3778	KCNMA1	-0.02	0.00	0.03	0.05
3780	KCNN1	0.02	0.00	0.00	0.07
3781	KCNN2	0.00	0.00	-0.05	0.13
3782	KCNN3	0.11	0.00	0.14	-0.06
3783	KCNN4	-0.02	0.00	-0.03	0.07
3784	KCNQ1	0.00	0.00	0.00	NaN
3785	KCNQ2	0.04	0.09	0.08	0.14
3786	KCNQ3	0.20	0.09	0.38	0.03
3787	KCNS1	0.02	0.09	0.03	0.02
3790	KCNS3	-0.02	0.00	0.00	-0.08
3791	KDR	-0.02	0.00	0.00	0.02
3792	KEL	0.02	0.00	0.00	-0.05
3795	KHK	0.00	0.00	0.00	NaN
3797	KIF3C	-0.02	0.00	0.00	-0.03
3798	KIF5A	0.02	0.00	0.00	-0.28
3799	KIF5B	-0.02	0.00	0.03	0.35
3800	KIF5C	-0.02	0.00	0.00	0.18
3801	KIFC3	-0.05	0.00	0.03	0.17
3812	KIR3DL2	0.00	0.09	-0.03	0.05
3814	KISS1	0.07	0.00	0.05	-0.13

3815	KIT	-0.02	0.00	0.00	0.05
3817	KLK2	0.00	0.00	-0.03	0.13
3818	KLKB1	-0.02	0.00	-0.08	0.00
3820	KLRB1	0.00	-0.18	0.05	0.07
3823	KLRC3	0.00	-0.18	0.08	0.09
3824	KLRD1	0.00	-0.18	0.08	0.22
3831	KNS2	0.00	0.00	0.03	-0.03
3836	KPNA1	0.00	0.00	-0.03	0.35
3837	KPNB1	0.00	0.18	0.00	0.19
3839	KPNA3	-0.07	0.00	-0.11	0.36
3840	KPNA4	0.02	0.00	0.03	0.12
3841	KPNA5	-0.02	0.00	0.00	0.01
3848	KRT1	0.02	0.00	-0.03	-0.07
3851	KRT4	0.02	0.00	-0.03	0.01
3852	KRT5	0.02	0.00	-0.03	-0.06
3853	KRT6A	0.02	0.00	-0.03	-0.02
3854	KRT6B	0.02	0.00	0.00	0.00
3855	KRT7	0.02	0.00	0.00	0.17
3856	KRT8	0.02	0.00	-0.03	0.19
3857	KRT9	0.02	0.18	0.00	-0.05
3858	KRT10	0.00	0.09	0.00	0.33
3859	KRT12	0.00	0.09	0.00	0.03
3860	KRT13	0.02	0.18	0.00	-0.12
3866	KRT15	0.02	0.18	0.00	0.06
3875	KRT18	0.02	0.00	-0.03	0.25
3880	KRT19	0.02	0.18	0.00	0.13
3895	KTN1	0.00	0.00	0.00	0.15
3897	L1CAM	0.00	0.00	-0.03	-0.04
3898	LAD1	0.07	0.00	0.05	0.27
3902	LAG3	-0.02	0.09	0.05	-0.12
3903	LAIR1	0.00	0.09	-0.03	-0.05
3904	LAIR2	0.00	0.09	-0.03	-0.17
3906	LALBA	0.00	0.00	0.00	NaN
3908	LAMA2	-0.02	0.00	0.05	-0.09
3909	LAMA3	0.00	-0.09	0.03	0.07
3910	LAMA4	0.02	0.00	0.03	0.03
3911	LAMA5	0.05	0.09	0.08	0.40
3912	LAMB1	0.02	0.00	-0.03	-0.02
3913	LAMB2	0.00	0.00	0.00	NaN
3914	LAMB3	0.07	0.00	0.00	0.22
3915	LAMC1	0.07	0.00	0.03	0.12
3916	LAMP1	0.00	0.00	0.00	-0.15
3918	LAMC2	0.07	0.00	0.03	0.06
3920	LAMP2	-0.05	0.00	-0.08	0.18
3925	STMN1	0.00	0.00	0.00	NaN
3927	LASP1	0.00	0.45	0.05	0.58
3929	LBP	0.02	0.00	0.03	-0.07

3930	LBR	0.07	0.00	0.03	-0.21
3931	LCAT	-0.04	0.00	0.00	0.12
3932	LCK	0.00	0.00	0.00	NaN
3933	LCN1	0.02	0.00	0.03	0.04
3934	LCN2	0.02	0.00	0.00	-0.02
3937	LCP2	0.00	0.00	-0.03	0.10
3938	LCT	-0.02	0.00	0.00	0.28
3939	LDHA	0.00	0.00	-0.05	-0.05
3945	LDHB	0.00	-0.09	0.05	0.21
3948	LDHC	0.00	0.00	-0.05	-0.05
3949	LDLR	0.02	0.00	0.00	0.16
3950	LECT2	0.00	0.00	-0.03	0.08
3952	LEP	0.02	0.00	0.00	0.04
3953	LEPR	-0.02	0.00	0.00	0.11
3954	LETM1	-0.02	0.00	0.00	0.23
3956	LGALS1	-0.04	0.00	0.03	-0.32
3957	LGALS2	-0.04	0.00	0.03	0.09
3958	LGALS3	0.00	0.00	0.00	0.17
3959	LGALS3BP	0.02	0.18	0.03	0.32
3960	LGALS4	0.02	0.09	0.05	0.03
3964	LGALS8	0.09	0.00	0.03	0.30
3973	LHCGR	0.02	0.00	0.00	-0.08
3975	LHX1	0.00	0.09	0.03	0.03
3976	LIF	0.00	0.00	0.03	0.12
3977	LIFR	0.04	0.09	0.00	0.13
3978	LIG1	0.00	0.00	-0.03	0.22
3980	LIG3	-0.02	0.09	0.03	0.15
3981	LIG4	-0.02	0.00	-0.05	0.13
3982	LIM2	0.00	0.00	-0.03	-0.13
3984	LIMK1	0.02	0.00	0.05	0.04
3985	LIMK2	0.00	0.00	0.05	0.34
3987	LIMS1	0.00	0.00	0.00	NaN
3988	LIPA	-0.04	0.00	-0.11	0.26
3990	LIPC	-0.02	0.00	-0.05	-0.04
3991	LIPE	0.02	0.00	-0.03	0.14
3992	FADS1	-0.02	0.00	0.05	0.16
3993	LLGL2	0.05	0.27	0.03	0.50
3995	FADS3	-0.04	0.00	0.03	0.14
3996	LLGL1	-0.05	0.00	0.00	-0.02
3998	LMAN1	-0.02	-0.09	-0.03	0.15
4000	LMNA	0.09	0.00	0.14	0.27
4001	LMNB1	0.00	0.00	-0.05	-0.09
4004	LMO1	0.00	0.00	-0.08	-0.02
4005	LMO2	0.04	0.00	-0.05	0.03
4008	LMO7	-0.05	-0.09	-0.14	0.00
4010	LMX1B	-0.02	0.00	0.03	0.22
4012	LNPEP	0.00	0.00	-0.14	0.00

4013	LOH11CR2A	-0.05	0.00	-0.03	0.09
4014	LOR	0.05	0.00	0.16	-0.17
4015	LOX	0.00	0.00	-0.03	0.12
4016	LOXL1	0.00	0.00	0.00	NaN
4017	LOXL2	-0.13	-0.18	-0.22	-0.04
4018	LPA	-0.02	0.00	-0.05	-0.18
4023	LPL	-0.15	-0.18	-0.22	0.08
4025	LPO	0.16	0.00	0.03	0.18
4026	LPP	0.04	0.00	0.00	0.13
4033	LRMP	0.00	-0.09	0.08	0.14
4034	LRRN1	-0.02	0.00	0.00	0.11
4035	LRP1	0.02	0.09	-0.03	-0.07
4036	LRP2	0.00	0.00	0.03	-0.07
4037	LRP3	0.00	0.00	0.05	-0.12
4038	LRP4	0.00	0.00	-0.03	-0.02
4040	LRP6	0.00	-0.09	0.05	0.18
4041	LRP5	0.07	0.09	0.03	0.21
4043	LRPAP1	0.00	0.00	0.00	-0.04
4045	LSAMP	0.00	0.00	-0.03	-0.07
4046	LSP1	0.00	0.00	0.00	NaN
4047	LSS	0.00	0.09	0.00	0.30
4048	LTA4H	0.00	0.09	0.00	0.16
4049	LTA	0.00	0.00	0.05	-0.03
4050	LTB	0.00	0.00	0.05	-0.06
4051	CYP4F3	0.00	0.00	0.00	NaN
4052	LTBP1	0.00	0.00	0.00	NaN
4053	LTBP2	-0.02	0.00	0.00	-0.07
4054	LTBP3	0.05	0.09	0.14	0.08
4055	LTBR	-0.02	0.09	0.05	0.36
4056	LTC4S	0.02	0.00	-0.03	0.03
4057	LTF	-0.04	0.00	0.00	-0.04
4058	LTK	-0.02	0.09	-0.14	0.05
4060	LUM	0.00	0.09	0.00	0.00
4061	LY6E	0.16	0.00	0.35	0.26
4062	LY6H	0.16	0.00	0.35	-0.03
4063	LY9	0.07	0.00	0.14	-0.10
4065	LY75	-0.02	0.00	0.03	0.27
4066	LYL1	0.00	-0.09	0.00	0.13
4067	LYN	0.11	0.00	0.14	0.03
4068	SH2D1A	-0.05	0.00	-0.08	0.17
4070	TACSTD2	0.00	0.00	0.00	0.16
4071	TM4SF1	0.04	0.00	0.03	0.28
4072	TACSTD1	0.02	0.00	0.00	0.08
4074	M6PR	0.00	-0.18	0.05	0.18
4082	MARCKS	0.02	0.00	0.03	0.24
4085	MAD2L1	0.00	0.00	-0.03	-0.04
4094	MAF	-0.09	0.00	0.00	0.16

4099	MAG	0.02	0.00	0.05	0.19
4100	MAGEA1	0.00	0.00	-0.03	0.01
4103	MAGEA4	-0.02	0.00	-0.11	-0.25
4107	MAGEA8	-0.05	0.00	-0.08	-0.24
4109	MAGEA10	-0.02	0.00	-0.11	0.15
4112	MAGEB1	-0.07	0.00	-0.11	0.04
4113	MAGEB2	-0.07	0.00	-0.11	0.15
4114	MAGEB3	-0.07	0.00	-0.11	-0.08
4115	MAGEB4	-0.07	0.00	-0.11	0.04
4117	MAK	-0.02	0.00	0.11	0.12
4118	MAL	0.00	0.00	0.00	NaN
4121	MAN1A1	-0.04	0.00	0.03	-0.01
4122	MAN2A2	0.02	0.00	0.03	0.18
4123	MAN2C1	0.00	0.00	0.00	NaN
4124	MAN2A1	0.00	0.00	-0.08	0.20
4125	MAN2B1	0.02	-0.09	0.00	0.10
4126	MANBA	-0.04	0.00	0.00	0.14
4128	MAOA	-0.05	0.00	-0.11	0.16
4129	MAOB	-0.05	0.00	-0.11	-0.04
4130	MAP1A	-0.04	0.00	-0.14	0.01
4131	MAP1B	0.00	0.00	-0.11	-0.17
4133	MAP2	0.00	0.00	0.00	NaN
4134	MAP4	-0.04	0.00	0.00	0.05
4137	MAPT	-0.04	0.00	0.00	-0.08
4139	MARK1	0.07	0.00	0.00	0.02
4140	MARK3	0.00	0.00	0.00	NaN
4141	MARS	0.02	0.00	0.00	0.40
4142	MAS1	-0.02	0.00	-0.05	-0.22
4143	MAT1A	0.00	0.00	0.00	-0.10
4144	MAT2A	0.00	0.00	0.00	NaN
4145	MATK	0.00	0.00	0.00	NaN
4146	MATN1	-0.02	0.09	0.00	0.03
4147	MATN2	0.16	0.18	0.27	0.19
4148	MATN3	-0.02	0.00	0.00	-0.03
4149	MAX	-0.02	0.00	0.00	0.17
4151	MB	-0.02	0.00	0.03	0.10
4152	MBD1	-0.02	-0.09	-0.05	0.06
4153	MBL2	-0.04	0.00	-0.03	-0.02
4155	MBP	-0.02	-0.09	0.00	0.09
4157	MC1R	-0.05	0.00	0.00	-0.02
4158	MC2R	-0.02	-0.09	0.00	0.10
4159	MC3R	0.02	0.00	0.05	-0.09
4160	MC4R	-0.02	-0.09	0.03	0.08
4161	MC5R	-0.02	-0.09	0.00	0.02
4162	MCAM	-0.05	0.00	0.00	0.28
4163	MCC	0.00	0.00	-0.05	0.11
4166	CHST6	-0.09	0.00	0.03	0.22

4168	MCF2	-0.04	0.09	-0.11	-0.04
4170	MCL1	0.07	0.09	0.14	0.18
4171	MCM2	0.00	0.00	-0.03	-0.01
4172	MCM3	-0.02	0.00	0.08	0.34
4173	MCM4	0.13	0.00	0.16	0.25
4174	MCM5	-0.02	0.00	0.03	0.01
4175	MCM6	-0.02	0.00	0.00	0.03
4176	MCM7	0.02	0.00	0.00	0.23
4185	ADAM11	-0.05	0.00	0.00	0.01
4188	MDFI	0.00	0.09	0.11	0.00
4189	DNAJB9	0.02	0.00	-0.03	0.00
4190	MDH1	0.02	0.00	0.00	0.14
4191	MDH2	0.02	0.00	0.08	0.32
4192	MDK	-0.02	0.00	-0.03	0.10
4193	MDM2	0.04	0.09	0.00	0.13
4194	MDM4	0.09	0.00	0.05	0.24
4197	MDS1	0.02	0.00	0.03	0.30
4199	ME1	-0.02	0.00	-0.05	0.12
4200	ME2	-0.02	-0.09	-0.05	0.15
4204	MECP2	0.00	0.00	-0.03	0.11
4205	MEF2A	-0.02	0.00	0.00	0.01
4207	MEF2B	0.02	0.00	0.00	0.21
4208	MEF2C	0.00	0.00	-0.14	-0.13
4209	MEF2D	0.04	0.00	0.11	0.03
4210	MEFV	0.04	0.00	0.00	-0.12
4211	MEIS1	0.02	0.00	0.00	0.14
4212	MEIS2	-0.02	0.00	-0.19	0.11
4215	MAP3K3	0.16	0.18	0.03	0.48
4216	MAP3K4	-0.02	0.00	-0.05	0.23
4217	MAP3K5	-0.02	0.00	0.08	0.40
4221	MEN1	0.00	-0.09	0.03	0.39
4222	MEOX1	-0.04	0.00	0.00	0.33
4223	MEOX2	-0.02	0.00	0.00	-0.02
4224	MEP1A	0.00	0.00	0.05	0.18
4225	MEP1B	0.02	-0.09	0.00	-0.12
4232	MEST	0.02	0.00	0.00	0.10
4233	MET	0.00	0.00	-0.03	0.01
4234	METTL1	0.02	0.09	0.00	0.02
4236	MFAP1	-0.04	0.00	-0.14	0.35
4237	MFAP2	0.00	0.00	0.00	NaN
4238	MFAP3	0.00	0.00	0.00	NaN
4239	MFAP4	-0.04	0.09	0.03	0.14
4240	MFGE8	0.00	0.00	-0.05	-0.03
4241	MFI2	0.02	0.09	-0.03	-0.09
4242	MFNG	-0.04	0.00	0.03	0.22
4245	MGAT1	0.00	0.00	-0.03	0.02
4246	SCGB2A1	-0.02	0.00	0.03	-0.01

4247	MGAT2	0.02	0.00	0.00	0.17
4248	MGAT3	-0.04	0.00	-0.03	-0.12
4249	MGAT5	-0.02	0.00	0.00	-0.05
4250	SCGB2A2	-0.02	0.00	0.03	-0.12
4254	KITLG	0.00	0.00	0.03	0.03
4255	MGMT	-0.04	0.00	0.00	0.06
4256	MGP	0.00	-0.09	0.05	0.02
4258	MGST2	-0.02	0.00	0.00	-0.02
4259	MGST3	0.04	0.00	0.08	0.27
4277	MICB	0.00	0.00	0.05	0.00
4281	MID1	-0.05	-0.09	-0.14	0.04
4284	MIP	0.00	0.00	0.00	NaN
4285	MIPEP	-0.07	0.00	-0.11	0.14
4286	MITF	-0.05	0.00	0.00	0.03
4288	MKI67	-0.04	0.00	0.00	0.11
4289	MKLN1	0.02	0.00	0.00	-0.22
4291	MLF1	0.02	0.00	0.03	0.25
4292	MLH1	0.02	0.00	0.00	0.33
4294	MAP3K10	0.00	0.00	0.05	0.11
4295	MLN	0.00	0.00	0.05	-0.08
4296	MAP3K11	0.02	0.00	0.05	-0.01
4297	MLL	-0.05	0.00	0.00	0.31
4298	MLLT1	0.00	-0.09	0.00	0.09
4300	MLLT3	-0.07	0.00	-0.03	0.34
4301	MLLT4	-0.02	0.00	-0.03	0.19
4303	MLLT7	-0.04	0.00	-0.08	-0.08
4306	NR3C2	-0.02	0.00	-0.03	0.18
4308	TRPM1	-0.04	0.00	-0.14	0.07
4311	MME	0.02	0.00	0.03	-0.05
4312	MMP1	-0.07	-0.09	0.00	-0.07
4313	MMP2	-0.07	0.00	0.00	0.13
4314	MMP3	-0.07	-0.09	0.00	0.06
4316	MMP7	-0.07	-0.09	0.00	0.15
4317	MMP8	-0.07	-0.09	0.00	0.08
4318	MMP9	0.02	0.00	0.03	0.03
4319	MMP10	-0.07	-0.09	0.00	-0.09
4320	MMP11	-0.02	0.00	0.03	0.10
4321	MMP12	-0.07	-0.09	0.00	0.03
4322	MMP13	-0.07	-0.09	0.00	-0.02
4323	MMP14	-0.02	0.00	0.00	0.14
4324	MMP15	-0.05	0.00	0.00	0.15
4325	MMP16	0.15	0.00	0.16	0.27
4326	MMP17	0.00	-0.09	0.00	-0.05
4327	MMP19	0.04	0.00	0.00	0.04
4329	ALDH6A1	-0.02	0.00	0.00	0.22
4330	MN1	-0.02	-0.09	0.03	0.19
4331	MNAT1	0.00	0.00	0.00	0.09

4332	MNDA	0.02	0.00	0.16	-0.08
4335	MNT	-0.05	0.00	0.00	0.12
4336	MOBP	-0.04	0.00	0.00	0.04
4338	MOCS2	0.00	0.00	-0.08	0.15
4340	MOG	0.00	0.00	0.08	-0.02
4342	MOS	0.11	0.00	0.14	0.07
4350	MPG	0.05	0.09	0.00	0.23
4351	MPI	0.00	0.00	0.00	NaN
4352	MPL	0.02	0.00	0.00	0.05
4353	MPO	0.16	0.00	0.03	0.09
4354	MPP1	-0.04	0.00	-0.11	0.20
4355	MPP2	-0.04	0.09	0.00	0.15
4356	MPP3	-0.04	0.00	0.00	0.14
4357	MPST	-0.04	0.00	0.03	0.16
4358	MPV17	0.00	0.00	0.00	NaN
4359	MPZ	0.11	0.00	0.14	0.09
4360	MRC1	0.00	0.00	0.08	-0.09
4361	MRE11A	-0.07	0.09	-0.03	0.10
4363	ABCC1	0.02	0.00	0.00	0.16
4435	CITED1	-0.04	0.00	-0.08	0.01
4436	MSH2	0.02	0.00	0.00	0.15
4437	MSH3	0.00	0.00	-0.14	0.14
4438	MSH4	-0.02	0.00	-0.03	-0.03
4439	MSH5	0.00	0.00	0.05	0.26
4440	MSI1	0.02	0.00	0.00	0.05
4477	MSMB	-0.02	0.00	0.00	-0.17
4478	MSN	-0.04	0.00	-0.05	0.18
4481	MSR1	-0.18	-0.18	-0.22	0.16
4482	MSRA	-0.11	-0.09	-0.14	0.15
4486	MST1R	0.00	0.00	0.00	NaN
4487	MSX1	-0.02	0.00	0.00	-0.06
4488	MSX2	0.00	0.00	-0.03	0.15
4495	MT1G	-0.05	0.00	0.00	0.15
4496	MT1H	-0.05	0.00	0.00	0.14
4501	MT1X	-0.05	0.00	0.00	0.18
4502	MT2A	-0.05	0.00	0.00	0.23
4504	MT3	-0.05	0.00	0.00	0.06
4507	MTAP	-0.13	-0.09	-0.05	0.42
4515	MTCP1	-0.05	0.00	-0.11	0.15
4520	MTF1	0.00	0.00	0.00	NaN
4521	NUDT1	0.00	0.00	0.05	0.18
4522	MTHFD1	-0.02	0.00	0.00	0.05
4524	MTHFR	-0.02	0.00	0.00	-0.14
4528	MTIF2	0.00	0.00	0.00	NaN
4534	MTM1	-0.04	0.00	-0.11	0.08
4542	MYO1F	0.02	-0.09	0.03	0.13
4544	MTNR1B	-0.09	0.09	-0.03	-0.05

4548	MTR	0.09	0.00	0.03	0.14
4552	MTRR	0.02	0.18	0.00	0.21
4580	MTX1	0.09	0.00	0.14	0.27
4582	MUC1	0.09	0.00	0.14	0.11
4583	MUC2	0.00	0.00	0.00	NaN
4585	MUC4	0.04	0.09	0.00	0.16
4593	MUSK	-0.02	0.00	0.00	-0.02
4594	MUT	0.00	0.00	0.08	0.19
4595	MUTYH	0.00	0.00	0.00	NaN
4597	MVD	-0.04	0.00	0.03	0.11
4598	MVK	0.00	0.00	0.00	NaN
4599	MX1	0.00	0.00	0.00	0.26
4600	MX2	0.00	0.00	0.00	0.28
4601	MXI1	-0.04	0.00	0.00	0.08
4602	MYB	-0.02	0.00	0.16	0.22
4605	MYBL2	0.02	0.09	0.03	0.09
4606	MYBPC2	0.00	0.00	-0.03	-0.03
4608	MYBPH	0.09	0.00	0.05	0.02
4609	MYC	0.24	0.27	0.38	0.17
4610	MYCL1	-0.02	0.09	0.00	0.00
4613	MYCN	-0.02	0.00	0.00	0.05
4615	MYD88	-0.04	0.00	0.00	0.26
4616	GADD45B	0.00	0.00	0.00	NaN
4617	MYF5	0.00	0.00	0.00	NaN
4618	MYF6	0.00	0.00	0.00	NaN
4619	MYH1	-0.07	0.00	0.00	-0.09
4620	MYH2	-0.07	0.00	0.00	0.03
4621	MYH3	-0.07	0.00	0.00	0.02
4622	MYH4	-0.07	0.00	0.00	-0.11
4624	MYH6	0.00	0.00	0.00	0.07
4625	MYH7	0.00	0.00	0.00	0.05
4626	MYH8	-0.07	0.00	0.00	0.18
4627	MYH9	-0.02	0.00	0.03	-0.08
4629	MYH11	0.02	0.00	0.00	0.17
4632	MYL1	0.00	0.00	0.00	NaN
4633	MYL2	0.00	0.00	0.00	NaN
4634	MYL3	-0.04	0.00	0.00	0.05
4635	MYL4	-0.04	0.00	0.00	0.13
4637	MYL6	0.00	0.00	0.00	NaN
4638	MYLK	0.00	0.00	-0.03	0.14
4640	MYO1A	0.02	0.00	0.00	-0.14
4641	MYO1C	-0.05	0.00	0.00	0.12
4642	MYO1D	0.00	0.18	0.00	-0.01
4643	MYO1E	-0.02	0.00	-0.05	0.00
4644	MYO5A	-0.02	0.00	-0.05	0.18
4646	MYO6	-0.02	0.00	-0.03	0.17
4647	MYO7A	0.07	0.09	0.03	0.29

4649	MYO9A	0.00	0.00	-0.05	-0.02
4650	MYO9B	0.04	0.00	0.00	0.14
4651	MYO10	0.04	0.18	0.00	0.20
4653	MYOC	0.02	0.00	0.05	0.02
4654	MYOD1	0.00	0.00	-0.05	0.01
4656	MYOG	0.09	0.00	0.05	0.06
4659	PPP1R12A	0.00	0.00	0.00	NaN
4660	PPP1R12B	0.09	0.00	0.05	0.19
4661	MYT1	0.07	0.09	0.14	0.27
4664	NAB1	0.00	0.00	0.00	NaN
4665	NAB2	0.02	0.00	0.00	-0.01
4666	NACA	0.00	0.00	0.00	NaN
4668	NAGA	-0.02	0.00	-0.03	-0.08
4669	NAGLU	-0.04	0.00	0.00	-0.03
4670	HNRPM	0.00	-0.09	0.00	0.02
4673	NAP1L1	-0.02	0.00	0.00	0.26
4674	NAP1L2	-0.04	0.00	-0.08	0.04
4675	NAP1L3	-0.07	0.00	-0.08	0.08
4676	NAP1L4	-0.02	0.00	-0.03	0.06
4677	NARS	-0.02	0.00	-0.03	0.29
4678	NASP	0.00	0.00	0.00	NaN
4680	CEACAM6	0.02	0.00	-0.03	0.11
4681	NBL1	0.00	0.00	0.00	NaN
4682	NUBP1	0.04	0.00	0.00	0.17
4684	NCAM1	-0.07	0.00	0.00	0.09
4685	NCAM2	0.00	0.00	-0.03	-0.03
4686	NCBP1	-0.02	0.00	0.00	0.21
4688	NCF2	0.07	0.00	0.03	-0.26
4689	NCF4	-0.04	0.00	0.03	0.08
4690	NCK1	0.02	0.00	-0.03	0.36
4691	NCL	0.00	0.00	0.00	NaN
4692	NDN	0.00	0.09	-0.08	-0.09
4693	NDP	-0.05	0.00	-0.11	-0.12
4694	NDUFA1	-0.05	0.00	-0.08	0.21
4695	NDUFA2	0.00	0.00	0.00	NaN
4696	NDUFA3	0.00	0.09	0.00	0.53
4697	NDUFA4	-0.02	0.00	0.00	0.07
4698	NDUFA5	0.00	0.00	-0.03	0.12
4700	NDUFA6	-0.02	0.00	-0.03	0.20
4701	NDUFA7	0.00	-0.09	0.00	-0.01
4702	NDUFA8	-0.02	0.00	0.00	0.12
4703	NEB	-0.02	0.00	0.03	-0.02
4704	NDUFA9	-0.02	0.09	0.08	0.45
4705	NDUFA10	0.00	0.00	0.00	NaN
4706	NDUFAB1	0.02	0.00	0.00	0.13
4707	NDUFB1	-0.02	0.00	0.03	0.30
4708	NDUFB2	0.02	0.00	0.00	0.11

4709	NDUFB3	0.00	0.00	0.00	NaN
4711	NDUFB5	0.04	0.00	0.03	0.44
4712	NDUFB6	-0.05	0.09	0.00	-0.11
4714	NDUFB8	-0.02	0.00	-0.03	0.28
4717	NDUFC1	0.00	0.00	0.00	NaN
4718	NDUFC2	0.11	0.09	0.03	0.55
4719	NDUFS1	0.00	0.00	0.00	NaN
4720	NDUFS2	0.11	0.00	0.14	0.47
4722	NDUFS3	-0.02	0.00	-0.03	0.31
4724	NDUFS4	0.00	0.00	-0.08	0.30
4725	NDUFS5	0.00	0.09	0.00	0.17
4726	NDUFS6	0.02	0.18	0.00	0.33
4728	NDUFS8	0.07	0.09	0.03	0.34
4729	NDUFV2	-0.02	-0.09	0.00	0.06
4733	DRG1	0.00	0.00	0.03	-0.12
4734	NEDD4	-0.02	0.00	-0.05	0.16
4736	RPL10A	0.00	0.00	0.05	0.04
4738	NEDD8	-0.02	0.00	0.00	-0.04
4744	NEFH	0.00	0.00	0.03	0.02
4745	NELL1	0.00	0.00	-0.05	0.01
4747	NEFL	-0.11	-0.18	-0.19	0.06
4750	NEK1	-0.02	0.00	-0.05	0.21
4751	NEK2	0.07	0.00	0.00	0.17
4752	NEK3	-0.07	0.00	-0.08	0.27
4753	NELL2	0.00	0.00	0.05	0.03
4756	NEO1	0.00	0.00	-0.03	0.04
4758	NEU1	0.00	0.00	0.05	0.11
4759	NEU2	0.00	0.00	0.00	NaN
4760	NEUROD1	0.00	0.00	0.00	NaN
4761	NEUROD2	0.02	0.82	0.05	0.07
4762	NEUROG1	0.00	0.00	-0.03	-0.08
4763	NF1	0.02	0.09	0.00	0.24
4771	NF2	0.00	0.00	0.03	0.22
4772	NFATC1	-0.02	-0.09	0.00	0.20
4775	NFATC3	-0.05	0.00	0.00	0.13
4776	NFATC4	-0.02	0.00	0.00	-0.12
4778	NFE2	0.02	0.00	-0.03	-0.20
4779	NFE2L1	0.05	0.18	0.00	0.55
4780	NFE2L2	0.02	0.00	0.00	0.15
4781	NFIB	-0.05	0.00	-0.03	0.06
4782	NFIC	0.00	0.00	0.00	NaN
4783	NFIL3	-0.02	0.00	0.00	-0.16
4784	NFIX	0.00	-0.09	0.00	-0.01
4790	NFKB1	-0.04	0.00	0.00	0.11
4791	NFKB2	0.00	0.00	-0.03	0.18
4792	NFKBIA	0.02	0.09	0.00	0.11
4793	NFKBIB	0.02	0.09	0.05	0.13

4794	NFKBIE	0.00	0.00	0.11	-0.01
4795	NFKBIL1	0.00	0.00	0.05	0.04
4798	NFRKB	-0.05	0.00	-0.03	0.19
4799	NFX1	0.00	0.09	0.00	0.24
4800	NFYA	0.00	0.00	0.08	0.18
4801	NFYB	0.00	0.00	0.00	NaN
4802	NFYC	0.00	0.00	0.00	NaN
4803	NGFB	-0.02	0.00	-0.03	-0.16
4804	NGFR	0.15	0.27	0.03	0.00
4809	NHP2L1	-0.02	0.00	-0.03	0.40
4814	NINJ1	-0.02	0.00	0.00	0.01
4815	NINJ2	0.00	0.09	0.08	0.18
4817	NIT1	0.11	0.00	0.14	0.41
4818	NKG7	0.00	0.00	-0.03	0.21
4820	NKTR	-0.04	0.00	0.00	0.08
4828	NMB	0.02	0.00	-0.03	0.09
4829	NMBR	0.00	0.00	0.03	-0.09
4832	NME3	0.04	0.18	0.00	0.23
4833	NME4	0.05	0.18	0.05	0.28
4835	NQO2	-0.02	0.00	0.00	0.39
4836	NMT1	-0.05	0.00	0.00	0.14
4837	NNMT	-0.09	0.00	0.00	0.12
4839	NOL1	-0.02	0.09	0.05	0.37
4841	NONO	-0.04	0.00	-0.08	0.32
4842	NOS1	0.02	0.00	-0.03	0.13
4843	NOS2A	0.00	0.18	0.00	-0.01
4846	NOS3	0.02	0.00	0.00	0.03
4848	CNOT2	0.04	0.00	0.03	0.35
4849	CNOT3	0.00	0.09	0.00	0.49
4850	CNOT4	0.02	0.00	0.03	0.16
4852	NPY	-0.02	0.18	0.03	-0.02
4853	NOTCH2	0.00	0.00	0.03	0.12
4854	NOTCH3	0.02	0.00	0.00	0.24
4855	NOTCH4	0.00	0.00	0.05	0.06
4856	NOV	0.20	0.18	0.38	0.16
4857	NOVA1	-0.02	0.00	-0.03	0.04
4858	NOVA2	-0.02	0.00	0.00	0.29
4860	NP	-0.02	0.00	-0.03	0.05
4861	NPAS1	-0.05	0.00	-0.03	0.06
4862	NPAS2	0.02	0.00	0.00	0.06
4863	NPAT	-0.07	-0.09	0.00	0.24
4864	NPC1	0.00	-0.09	0.03	0.40
4867	NPHP1	0.00	0.00	0.00	NaN
4868	NPHS1	0.00	0.09	0.05	0.04
4869	NPM1	0.00	0.00	-0.03	0.08
4879	NPPB	-0.02	0.00	0.00	0.16
4881	NPR1	0.07	0.00	0.16	0.23

4882	NPR2	0.00	0.00	0.00	0.20
4883	NPR3	0.02	0.18	0.00	-0.05
4884	NPTX1	0.00	0.00	0.05	0.08
4885	NPTX2	0.02	0.00	0.00	-0.07
4886	NPY1R	0.02	0.00	-0.05	0.10
4887	NPY2R	-0.02	0.00	-0.03	0.28
4889	NPY5R	0.02	0.00	-0.05	-0.04
4891	SLC11A2	0.02	0.00	-0.03	0.18
4893	NRAS	0.00	-0.09	0.00	0.07
4897	NRCAM	0.02	0.00	-0.03	-0.01
4898	NRD1	0.00	0.00	-0.03	0.16
4899	NRF1	0.02	0.00	0.00	0.16
4900	NRGN	-0.05	0.00	-0.03	0.07
4901	NRL	-0.02	0.00	0.00	0.08
4902	NRTN	-0.02	-0.09	0.00	0.12
4905	NSF	-0.04	0.00	0.00	-0.02
4907	NT5E	-0.02	0.00	-0.05	0.18
4908	NTF3	-0.02	0.18	0.05	-0.05
4913	NTHL1	0.04	0.18	0.00	0.27
4914	NTRK1	0.04	0.00	0.11	-0.08
4915	NTRK2	-0.02	0.00	0.00	0.03
4916	NTRK3	0.00	0.00	-0.05	0.07
4917	NTN2L	0.04	0.09	0.00	0.10
4919	ROR1	-0.02	0.00	0.00	0.17
4920	ROR2	-0.02	0.00	0.00	0.07
4921	DDR2	0.05	0.00	0.14	0.27
4922	NTS	0.00	0.00	0.00	NaN
4923	NTSR1	0.04	0.09	0.08	-0.06
4924	NUCB1	0.00	0.00	-0.03	0.41
4925	NUCB2	0.00	0.00	-0.05	0.14
4926	NUMA1	-0.02	0.09	0.00	0.22
4927	NUP88	-0.04	0.00	0.00	0.12
4928	NUP98	-0.02	0.00	-0.03	0.00
4929	NR4A2	0.00	0.00	0.03	0.01
4931	NVL	0.09	0.00	0.00	0.21
4938	OAS1	0.02	0.00	0.00	-0.14
4939	OAS2	0.02	0.00	0.00	-0.10
4940	OAS3	0.02	0.00	0.00	-0.13
4942	OAT	-0.04	0.00	0.03	0.32
4947	OAZ2	0.00	0.09	-0.05	0.14
4948	OCA2	-0.04	0.09	-0.08	0.11
4952	OCRL	-0.05	0.00	-0.08	0.18
4953	ODC1	-0.02	0.00	0.00	0.15
4956	ODF1	0.22	0.09	0.32	0.05
4957	ODF2	0.02	0.00	0.00	0.12
4967	OGDH	-0.02	0.00	0.08	0.20
4968	OGG1	-0.02	0.00	0.00	0.07

4973	OLR1	0.00	-0.18	0.08	0.05
4976	OPA1	0.02	0.09	0.00	0.35
4978	OPCML	-0.07	0.00	-0.05	0.14
4982	TNFRSF11B	0.22	0.18	0.38	0.16
4983	OPHN1	-0.04	0.00	-0.05	-0.10
4985	OPRD1	-0.02	0.09	0.00	-0.11
4986	OPRK1	0.13	0.00	0.14	0.00
4987	OPRL1	0.07	0.09	0.11	0.01
4988	OPRM1	0.00	0.00	-0.03	0.02
4990	SIX6	0.00	0.00	-0.03	0.01
4991	OR1D2	-0.05	0.00	0.00	0.06
4992	OR1F1	0.04	0.00	0.00	-0.04
4993	OR2C1	0.04	0.00	0.00	-0.06
4994	OR3A1	-0.05	0.00	0.00	-0.01
4998	ORC1L	0.00	0.00	-0.03	-0.08
4999	ORC2L	0.00	0.00	0.00	NaN
5000	ORC4L	-0.02	0.00	0.00	0.01
5001	ORC5L	0.02	0.00	0.00	0.09
5005	ORM2	-0.02	0.00	0.00	-0.03
5007	OSBP	-0.04	0.00	0.00	0.09
5008	OSM	0.00	0.00	0.05	0.02
5009	OTC	-0.05	0.00	-0.11	0.16
5010	CLDN11	0.05	0.00	0.05	-0.10
5016	OVGP1	0.00	0.00	-0.03	-0.04
5017	OVOL1	0.02	0.00	0.05	0.07
5018	OXA1L	-0.02	0.00	0.00	0.02
5020	OXT	0.00	0.00	0.00	-0.08
5021	OXTR	0.00	0.00	0.00	0.16
5023	P2RX1	-0.04	0.00	0.00	0.12
5024	P2RX3	0.00	0.00	0.05	-0.03
5025	P2RX4	0.02	0.09	0.00	0.05
5026	P2RX5	-0.04	0.00	0.00	0.13
5027	P2RX7	0.02	0.00	0.00	-0.19
5028	P2RY1	0.02	0.00	0.03	0.02
5029	P2RY2	0.05	0.09	-0.03	0.40
5030	P2RY4	-0.04	0.00	-0.08	-0.09
5031	P2RY6	0.05	0.09	-0.03	0.14
5033	P4HA1	-0.02	0.00	0.00	0.12
5034	P4HB	0.00	0.09	0.08	0.14
5047	PAEP	0.02	0.00	0.03	0.06
5048	PAFAH1B1	-0.05	0.00	-0.03	0.15
5049	PAFAH1B2	-0.07	0.00	0.00	0.09
5050	PAFAH1B3	0.02	0.00	-0.03	0.24
5051	PAFAH2	-0.02	0.00	0.00	0.17
5052	PRDX1	0.00	0.00	0.00	NaN
5053	PAH	0.00	0.00	0.03	0.08
5054	SERPINE1	0.02	0.00	0.00	-0.04

5055	SERPINB2	-0.02	-0.09	0.00	-0.07
5058	PAK1	0.07	0.09	0.03	0.27
5062	PAK2	0.02	0.09	-0.03	0.26
5063	PAK3	-0.05	0.00	-0.08	-0.11
5064	PALM	0.00	0.00	-0.03	0.02
5066	PAM	0.00	0.00	-0.14	-0.01
5069	PAPPA	-0.04	0.00	0.00	0.25
5071	PARK2	-0.02	0.00	-0.08	-0.11
5073	PARN	0.02	0.00	0.00	0.15
5074	PAWR	-0.02	0.00	0.00	0.23
5076	PAX2	-0.02	0.00	-0.03	-0.08
5077	PAX3	0.00	0.00	0.00	NaN
5078	PAX4	0.02	0.00	-0.03	0.17
5079	PAX5	-0.02	0.00	0.03	0.06
5080	PAX6	0.04	0.00	0.00	0.05
5081	PAX7	0.00	0.00	0.00	NaN
5082	PDCL	-0.02	0.00	0.00	0.05
5083	PAX9	0.04	0.09	0.00	0.06
5087	PBX1	0.05	0.00	0.11	0.19
5089	PBX2	0.00	0.00	0.05	0.14
5090	PBX3	-0.02	0.00	0.03	0.04
5091	PC	0.07	0.09	0.03	0.21
5093	PCBP1	0.02	0.00	0.00	0.06
5094	PCBP2	0.02	0.09	-0.03	0.36
5095	PCCA	-0.02	0.00	-0.05	0.01
5096	PCCB	0.00	0.00	0.00	NaN
5097	PCDH1	0.00	0.00	0.00	NaN
5099	PCDH7	-0.05	-0.09	-0.05	0.08
5100	PCDH8	-0.07	0.00	-0.08	0.17
5101	PCDH9	-0.07	-0.09	-0.14	0.06
5104	SERPINA5	-0.02	0.00	-0.03	0.06
5105	PCK1	0.05	0.00	0.05	0.45
5108	PCM1	-0.16	-0.18	-0.22	0.36
5110	PCMT1	0.00	0.00	-0.03	0.48
5111	PCNA	0.02	0.00	-0.05	0.12
5118	PCOLCE	0.02	0.00	0.00	0.13
5119	PCOLN3	-0.04	0.00	0.03	0.20
5121	PCP4	0.00	0.00	-0.03	0.18
5122	PCSK1	0.00	0.00	-0.14	0.03
5125	PCSK5	-0.02	0.00	0.00	0.04
5126	PCSK2	0.00	0.00	-0.08	-0.10
5127	PCTK1	-0.05	0.00	-0.08	0.10
5128	PCTK2	0.00	0.09	0.00	0.20
5130	PCYT1A	0.02	0.09	0.00	0.23
5132	PDC	0.04	0.09	0.03	-0.13
5133	PDCD1	0.00	0.00	0.00	NaN
5134	PDCD2	-0.02	0.00	-0.03	0.22

5136	PDE1A	0.00	0.00	0.00	NaN
5137	PDE1C	-0.02	0.09	-0.03	-0.12
5138	PDE2A	0.00	0.09	0.00	0.01
5139	PDE3A	0.00	0.00	0.08	0.07
5140	PDE3B	-0.02	0.00	-0.05	0.02
5141	PDE4A	0.02	0.00	0.00	0.19
5142	PDE4B	-0.02	0.00	0.00	0.07
5143	PDE4C	0.02	0.00	0.00	-0.02
5144	PDE4D	0.00	0.09	-0.11	0.21
5145	PDE6A	0.00	0.00	0.00	NaN
5146	PDE6C	-0.02	0.00	-0.05	-0.06
5147	PDE6D	0.00	0.00	0.00	NaN
5148	PDE6G	0.00	0.09	0.08	0.16
5149	PDE6H	0.00	-0.09	0.05	-0.02
5151	PDE8A	0.00	0.00	-0.03	-0.03
5152	PDE9A	0.00	0.00	0.00	0.01
5153	PDE1B	0.02	0.00	-0.03	-0.13
5155	PDGFB	-0.04	0.00	-0.03	-0.12
5156	PDGFRA	-0.02	0.00	0.00	0.12
5157	PDGFRL	-0.16	-0.18	-0.22	0.03
5158	PDE6B	-0.02	0.00	-0.03	0.07
5159	PDGFRB	0.00	0.00	0.00	NaN
5160	PDHA1	-0.05	0.00	-0.11	0.22
5161	PDHA2	-0.04	0.00	0.00	0.07
5162	PDHB	-0.04	0.00	0.00	0.06
5163	PDK1	0.00	0.00	0.00	-0.03
5164	PDK2	0.13	0.18	0.03	0.32
5165	PDK3	-0.05	0.00	-0.11	0.06
5166	PDK4	0.02	0.00	-0.03	0.03
5167	ENPP1	-0.02	0.00	0.08	0.14
5168	ENPP2	0.20	0.18	0.38	0.12
5169	ENPP3	0.00	0.00	0.05	0.01
5170	PDPK1	0.05	0.09	0.03	0.22
5172	SLC26A4	0.02	0.00	-0.03	0.25
5173	PDYN	0.02	0.00	-0.03	-0.06
5174	PDZK1	0.04	0.00	0.11	-0.14
5175	PECAM1	0.15	0.18	0.03	-0.14
5176	SERPINF1	-0.05	0.00	0.00	0.02
5179	PENK	0.11	0.00	0.11	0.03
5184	PEPD	0.00	0.00	0.05	-0.17
5187	PER1	-0.05	0.00	0.00	0.08
5188	PET112L	-0.02	0.00	0.00	-0.11
5189	PEX1	0.02	0.00	0.08	0.38
5190	PEX6	0.00	0.00	0.08	0.15
5191	PEX7	-0.02	0.00	0.08	0.38
5192	PEX10	-0.02	0.00	0.03	0.11
5193	PEX12	-0.02	-0.09	0.03	0.12

5194	PEX13	0.00	0.00	0.00	NaN
5195	PEX14	0.00	0.00	0.00	NaN
5196	PF4	-0.02	0.00	0.00	0.04
5198	PFAS	-0.05	0.00	0.00	0.08
5201	PFDN1	0.00	0.00	0.00	NaN
5202	PFDN2	0.11	0.00	0.14	0.42
5203	PFDN4	0.07	0.09	0.05	0.34
5204	PFDN5	0.02	0.00	-0.03	0.08
5205	ATP8B1	-0.02	0.00	-0.03	0.05
5207	PFKFB1	-0.04	0.00	-0.05	-0.07
5208	PFKFB2	0.09	0.00	0.00	0.10
5209	PFKFB3	0.00	0.00	0.11	0.06
5210	PFKFB4	-0.04	0.00	0.00	0.05
5211	PFKL	0.00	0.00	0.00	0.41
5213	PFKM	0.00	0.00	0.00	NaN
5214	PFKP	0.02	0.00	0.11	0.14
5216	PFN1	-0.04	0.00	0.00	0.11
5217	PFN2	0.02	0.00	0.03	0.19
5218	PFTK1	0.02	0.00	0.05	0.35
5223	PGAM1	-0.02	-0.09	-0.03	0.08
5224	PGAM2	-0.02	0.00	0.05	0.06
5225	PGC	0.00	0.00	0.08	-0.05
5226	PGD	0.00	0.00	0.00	NaN
5228	PGF	-0.02	0.00	0.00	0.11
5229	PGGT1B	0.00	0.00	-0.05	0.14
5230	PGK1	-0.04	0.00	-0.08	0.21
5236	PGM1	-0.02	0.00	0.00	0.21
5239	PGM5	-0.02	0.00	-0.03	0.10
5241	PGR	-0.04	-0.09	0.00	0.07
5243	ABCB1	0.02	0.00	0.03	0.22
5244	ABCB4	0.02	0.00	0.00	0.03
5245	PHB	0.13	0.27	0.00	0.65
5250	SLC25A3	0.00	0.00	0.00	NaN
5251	PHEX	-0.05	0.00	-0.11	0.32
5252	PHF1	0.00	0.00	0.05	0.04
5253	PHF2	-0.02	0.00	0.00	0.07
5255	PHKA1	-0.04	0.00	-0.08	0.09
5256	PHKA2	-0.05	-0.09	-0.11	0.20
5257	PHKB	-0.11	0.09	0.03	0.20
5260	PHKG1	0.00	0.00	0.05	0.11
5261	PHKG2	0.07	0.00	0.00	0.22
5264	PHYH	0.02	0.00	0.14	0.32
5265	SERPINA1	-0.02	0.00	0.00	0.07
5266	PI3	0.02	0.09	0.03	0.00
5267	SERPINA4	-0.02	0.00	-0.03	0.03
5268	SERPINB5	-0.02	-0.09	0.00	-0.07
5271	SERPINB8	-0.02	-0.09	0.00	0.12

5272	SERPINB9	-0.02	0.00	0.00	0.13
5273	SERPINB10	-0.02	-0.09	0.00	0.11
5274	SERPINI1	0.02	0.00	0.00	0.07
5275	SERPINB13	-0.02	-0.09	0.00	0.02
5276	SERPINI2	0.02	0.00	0.00	0.02
5277	PIGA	-0.05	0.00	-0.14	0.07
5281	PIGF	0.00	0.00	0.00	NaN
5283	PIGH	-0.02	0.00	0.00	0.01
5284	PIGR	0.09	0.00	0.00	0.06
5287	PIK3C2B	0.07	0.00	0.05	-0.05
5288	PIK3C2G	0.00	-0.09	0.05	-0.03
5289	PIK3C3	0.00	-0.09	-0.03	0.01
5290	PIK3CA	0.04	0.00	0.03	0.30
5291	PIK3CB	0.02	0.00	0.00	0.20
5292	PIM1	0.02	0.00	0.08	0.17
5293	PIK3CD	-0.02	0.00	0.00	0.14
5294	PIK3CG	0.02	0.00	-0.03	-0.02
5295	PIK3R1	0.00	0.00	-0.08	0.14
5296	PIK3R2	0.02	0.00	0.00	0.14
5297	PIK4CA	-0.02	0.00	0.05	-0.29
5298	PIK4CB	0.07	0.09	0.19	0.19
5300	PIN1	0.00	0.00	0.00	NaN
5303	PIN4	-0.04	0.00	-0.08	0.07
5304	PIP	0.02	0.00	0.00	-0.15
5305	PIP5K2A	-0.02	0.00	0.05	-0.02
5307	PITX1	0.00	0.00	-0.03	-0.09
5308	PITX2	-0.02	0.00	0.00	0.00
5309	PITX3	0.00	0.00	-0.03	0.18
5310	PKD1	0.04	0.18	0.00	0.12
5311	PKD2	-0.04	0.00	-0.03	0.17
5313	PKLR	0.09	0.00	0.14	-0.08
5315	PKM2	0.00	0.00	-0.05	-0.05
5316	PKNOX1	0.00	0.00	0.03	0.04
5317	PKP1	0.07	0.00	0.05	0.08
5318	PKP2	0.00	0.09	0.05	0.16
5319	PLA2G1B	0.02	0.00	0.00	0.14
5320	PLA2G2A	0.00	0.00	0.00	NaN
5321	PLA2G4A	0.09	0.09	0.03	-0.08
5322	PLA2G5	0.00	0.00	0.00	NaN
5324	PLAG1	0.11	0.00	0.14	0.12
5325	PLAGL1	0.02	0.00	0.00	0.08
5326	PLAGL2	0.00	0.00	0.00	NaN
5327	PLAT	0.02	0.09	0.11	-0.04
5328	PLAU	0.04	0.00	0.00	0.20
5329	PLAUR	-0.02	0.00	-0.03	0.11
5330	PLCB2	-0.02	0.00	-0.14	0.08
5332	PLCB4	0.00	0.00	-0.05	0.24

5333	PLCD1	0.00	0.00	0.00	-0.09
5334	PLCL1	0.00	0.00	0.00	NaN
5335	PLCG1	0.04	0.00	0.00	0.00
5336	PLCG2	-0.09	0.00	0.00	0.14
5337	PLD1	0.05	0.00	0.03	0.00
5338	PLD2	-0.04	0.00	0.00	0.01
5339	PLEC1	0.16	0.00	0.35	0.30
5340	PLG	-0.02	0.00	-0.05	-0.18
5341	PLEK	0.00	0.00	0.00	NaN
5345	SERPINF2	-0.05	0.00	0.00	0.08
5346	PLIN	0.02	0.00	-0.05	0.04
5348	FXYD1	0.00	0.00	0.08	0.07
5349	FXYD3	0.00	0.00	0.08	0.13
5352	PLOD2	0.02	0.00	0.00	0.18
5355	PLP2	-0.04	0.00	-0.05	0.39
5357	PLS1	0.02	0.00	0.03	0.25
5358	PLS3	-0.05	0.00	-0.05	0.11
5359	PLSCR1	0.02	0.00	0.00	0.30
5360	PLTP	0.04	0.00	0.03	0.07
5361	PLXNA1	0.00	0.00	-0.03	0.06
5362	PLXNA2	0.07	0.00	0.00	0.02
5364	PLXNB1	-0.04	0.00	0.00	-0.07
5365	PLXNB3	0.00	0.00	-0.03	0.03
5366	PMAIP1	-0.02	-0.09	-0.03	0.03
5367	PMCH	0.00	0.09	0.03	-0.04
5368	PNOC	-0.13	0.00	-0.19	-0.03
5371	PML	0.00	0.00	0.00	NaN
5372	PMM1	-0.02	0.00	-0.03	0.15
5373	PMM2	0.04	0.00	0.00	0.10
5375	PMP2	0.13	0.00	0.14	0.07
5376	PMP22	-0.07	0.00	-0.03	-0.12
5378	PMS1	0.00	0.00	0.00	NaN
5395	PMS2	0.00	0.00	0.00	NaN
5406	PNLIP	-0.04	0.00	0.03	0.09
5407	PNLIPRP1	-0.04	0.00	0.03	0.09
5408	PNLIPRP2	-0.04	0.00	0.00	0.08
5409	PNMT	0.02	0.91	0.05	0.52
5411	PNN	0.00	0.00	-0.03	0.20
5412	UBL3	-0.05	0.00	-0.05	0.19
5420	PODXL	0.02	0.00	0.00	0.08
5423	POLB	0.02	0.09	0.11	0.27
5424	POLD1	0.00	0.00	-0.03	-0.12
5425	POLD2	-0.02	0.00	0.05	0.14
5426	POLE	0.00	0.00	0.00	NaN
5427	POLE2	0.02	0.00	0.00	0.14
5428	POLG	0.02	0.00	-0.05	0.16
5429	POLH	0.00	0.00	0.11	0.21

5430	POLR2A	-0.05	0.00	0.00	0.11
5431	POLR2B	-0.02	0.00	0.00	0.06
5432	POLR2C	-0.05	0.00	0.00	0.35
5433	POLR2D	-0.02	0.00	0.00	0.34
5434	POLR2E	0.00	0.00	-0.03	0.08
5435	POLR2F	-0.04	0.00	0.03	-0.07
5436	POLR2G	0.00	0.00	0.03	0.26
5437	POLR2H	0.05	0.00	0.05	0.17
5438	POLR2I	-0.02	0.09	0.08	0.12
5439	POLR2J	0.02	0.00	0.00	0.13
5440	POLR2K	0.18	0.18	0.27	0.22
5441	POLR2L	0.00	0.00	0.00	NaN
5442	POLRMT	0.00	0.00	-0.03	0.06
5443	POMC	-0.02	0.00	0.00	0.04
5444	PON1	0.02	0.00	-0.03	0.03
5445	PON2	0.02	0.00	-0.03	0.07
5446	PON3	0.02	0.00	-0.03	0.01
5447	POR	0.00	0.09	0.11	0.11
5449	POU1F1	-0.05	0.00	0.00	-0.02
5450	POU2AF1	-0.07	0.00	0.00	0.23
5451	POU2F1	0.02	0.00	0.05	0.07
5452	POU2F2	0.02	0.00	-0.03	0.13
5454	POU3F2	-0.02	0.00	0.00	0.07
5456	POU3F4	-0.05	0.00	-0.08	-0.24
5457	POU4F1	-0.04	-0.09	-0.14	0.17
5458	POU4F2	0.00	0.00	-0.03	-0.01
5459	POU4F3	0.00	0.00	0.00	NaN
5460	POU5F1	0.00	0.00	0.05	0.25
5463	POU6F1	0.02	0.00	-0.03	0.05
5465	PPARA	-0.02	0.00	-0.03	0.14
5467	PPARD	0.00	0.00	0.05	0.14
5468	PPARG	0.02	0.00	0.00	0.12
5469	PPARBP	0.00	0.55	0.05	0.64
5470	PPEF2	-0.02	0.00	0.00	-0.10
5471	PPAT	-0.02	0.00	0.00	0.11
5473	PPBP	-0.02	0.00	0.00	0.06
5475	PPEF1	-0.05	-0.09	-0.11	0.03
5478	PPIA	-0.02	0.00	0.08	0.33
5479	PPIB	0.00	0.09	-0.08	0.16
5480	PPIC	0.00	0.00	-0.03	0.21
5481	PPID	-0.02	0.00	-0.05	0.31
5493	PPL	0.04	0.00	0.00	0.09
5494	PPM1A	0.00	0.00	-0.03	0.17
5495	PPM1B	0.00	0.00	0.00	NaN
5496	PPM1G	0.00	0.00	0.00	NaN
5498	PPOX	0.11	0.00	0.14	0.47
5500	PPP1CB	0.00	0.00	0.00	NaN

5501	PPP1CC	0.00	0.00	0.00	NaN
5502	PPP1R1A	0.00	0.00	-0.03	0.08
5504	PPP1R2	0.02	0.09	0.00	0.29
5506	PPP1R3A	0.00	0.00	-0.03	0.15
5507	PPP1R3C	-0.02	0.00	-0.05	0.15
5509	PPP1R3D	0.05	0.09	0.00	0.30
5510	PPP1R7	0.00	0.00	0.00	NaN
5511	PPP1R8	-0.02	0.00	0.00	-0.01
5514	PPP1R10	0.00	0.00	0.05	0.11
5516	PPP2CB	-0.09	-0.18	-0.19	0.24
5518	PPP2R1A	0.00	-0.09	-0.03	0.01
5519	PPP2R1B	-0.07	0.00	0.00	0.24
5520	PPP2R2A	-0.11	-0.18	-0.19	0.22
5521	PPP2R2B	0.00	0.00	0.00	NaN
5523	PPP2R3A	0.00	0.00	0.00	NaN
5524	PPP2R4	0.02	0.00	0.00	0.21
5525	PPP2R5A	0.07	0.00	0.00	0.19
5526	PPP2R5B	0.02	0.00	0.03	0.11
5527	PPP2R5C	0.00	0.00	-0.03	0.02
5528	PPP2R5D	0.00	0.00	0.08	0.28
5529	PPP2R5E	-0.02	0.00	0.00	0.07
5530	PPP3CA	-0.04	0.00	0.00	0.08
5532	PPP3CB	-0.02	0.00	0.00	0.29
5533	PPP3CC	-0.13	-0.18	-0.22	0.30
5534	PPP3R1	0.02	0.00	0.00	0.12
5536	PPP5C	-0.02	0.00	-0.03	0.04
5537	PPP6C	-0.02	0.00	0.00	0.06
5538	PPT1	-0.02	0.00	0.00	-0.11
5544	PRB3	0.00	-0.18	0.05	0.11
5546	PRCC	0.04	0.00	0.11	0.26
5547	PRCP	0.00	0.00	0.00	0.26
5550	PREP	0.04	0.09	-0.03	0.34
5551	PRF1	-0.02	0.00	-0.03	0.13
5552	PRG1	-0.02	0.00	0.03	0.09
5553	PRG2	0.02	0.00	0.05	0.10
5554	PRH1	0.00	-0.18	0.08	0.12
5557	PRIM1	0.00	0.00	0.00	NaN
5558	PRIM2A	-0.04	0.00	0.11	0.19
5563	PRKAA2	0.00	0.00	0.00	-0.16
5564	PRKAB1	0.00	0.00	0.00	NaN
5565	PRKAB2	0.07	0.00	0.14	0.16
5566	PRKACA	0.02	-0.09	0.00	0.15
5567	PRKACB	-0.02	0.00	-0.03	-0.02
5568	PRKACG	-0.02	0.00	-0.03	0.07
5569	PKIA	0.11	0.09	0.11	0.19
5571	PRKAG1	0.00	0.00	0.00	NaN
5573	PRKAR1A	0.11	0.18	0.03	0.45

5576	PRKAR2A	-0.04	0.00	0.00	0.15
5577	PRKAR2B	0.02	0.00	-0.03	0.03
5578	PRKCA	0.11	0.18	0.03	0.06
5579	PRKCB1	0.02	0.00	0.00	-0.13
5580	PRKCD	-0.02	0.00	0.00	0.06
5581	PRKCE	0.00	0.00	0.00	NaN
5582	PRKCG	0.00	0.09	0.00	-0.13
5583	PRKCH	0.00	0.00	0.00	0.04
5584	PRKCI	0.05	0.00	0.05	0.51
5588	PRKCQ	0.00	0.00	0.11	0.14
5589	PRKCSH	0.00	0.00	0.00	NaN
5590	PRKCZ	-0.02	0.00	0.03	-0.20
5591	PRKDC	0.11	0.00	0.16	0.36
5592	PRKG1	-0.04	0.00	-0.03	0.15
5593	PRKG2	-0.04	0.00	-0.03	0.12
5594	MAPK1	0.00	0.00	0.03	0.08
5596	MAPK4	-0.02	-0.09	-0.05	0.13
5597	MAPK6	-0.02	0.00	-0.05	0.18
5598	MAPK7	-0.04	0.09	0.03	0.43
5599	MAPK8	-0.02	0.00	-0.03	0.12
5600	MAPK11	0.00	0.09	0.03	0.06
5601	MAPK9	0.00	0.00	-0.03	0.42
5602	MAPK10	-0.04	0.00	-0.03	0.02
5603	MAPK13	0.02	0.00	0.05	-0.04
5604	MAP2K1	0.00	0.09	-0.05	-0.02
5605	MAP2K2	0.00	0.00	0.00	NaN
5606	MAP2K3	-0.04	0.18	0.00	0.47
5607	MAP2K5	0.00	0.00	-0.05	0.08
5608	MAP2K6	0.04	0.27	0.03	0.03
5609	MAP2K7	0.00	-0.09	0.00	0.11
5611	DNAJC3	-0.02	0.00	-0.05	-0.04
5612	PRKRIR	0.04	0.09	-0.03	0.49
5613	PRKX	-0.05	-0.09	-0.08	0.14
5616	PRKY	0.00	0.00	-0.03	0.04
5617	PRL	0.00	0.00	0.05	0.17
5618	PRLR	0.02	0.27	0.00	0.26
5619	PRM1	0.04	0.00	0.00	-0.07
5620	PRM2	0.04	0.00	0.00	-0.10
5621	PRNP	0.02	0.00	-0.03	0.17
5624	PROC	-0.02	0.00	0.00	0.02
5625	PRODH	-0.02	0.00	0.03	0.21
5626	PROP1	0.00	0.00	0.00	NaN
5627	PROS1	-0.02	0.00	-0.05	-0.17
5629	PROX1	0.07	0.00	0.00	0.01
5630	PRPH	0.00	0.00	-0.03	-0.08
5631	PRPS1	-0.05	0.09	-0.08	0.24
5634	PRPS2	-0.05	-0.09	-0.14	-0.06

5635	PRPSAP1	0.04	0.27	0.03	0.20
5636	PRPSAP2	-0.05	0.00	0.00	0.10
5638	PRRG1	-0.05	0.00	-0.11	-0.09
5639	PRRG2	0.02	0.00	-0.03	-0.23
5641	LGMN	-0.02	0.00	0.03	0.16
5648	MASP1	0.04	0.00	0.03	0.05
5649	RELN	0.02	0.00	0.00	0.09
5650	KLK7	0.00	0.00	-0.03	0.09
5651	PRSS7	-0.04	-0.18	0.05	-0.06
5652	PRSS8	0.04	0.09	0.00	0.34
5653	KLK6	0.00	0.00	-0.03	-0.17
5655	KLK10	0.00	0.00	-0.03	-0.04
5657	PRTN3	0.00	0.00	-0.03	0.18
5660	PSAP	-0.02	0.00	-0.03	0.14
5662	PSD	0.00	0.00	-0.03	0.00
5663	PSEN1	-0.02	0.00	-0.03	0.41
5664	PSEN2	0.07	0.00	0.03	0.28
5682	PSMA1	-0.02	0.00	-0.05	0.02
5683	PSMA2	-0.02	0.00	0.05	0.29
5684	PSMA3	0.00	0.00	-0.03	0.23
5685	PSMA4	0.00	0.00	-0.03	-0.04
5686	PSMA5	0.00	0.00	-0.03	-0.07
5687	PSMA6	0.02	0.09	0.00	0.41
5688	PSMA7	0.04	0.09	0.05	0.24
5689	PSMB1	-0.02	0.00	-0.03	0.19
5690	PSMB2	-0.02	0.00	0.00	0.09
5691	PSMB3	0.00	0.45	0.05	0.63
5692	PSMB4	0.07	0.09	0.19	0.19
5693	PSMB5	-0.02	0.00	0.00	0.03
5694	PSMB6	-0.04	0.00	0.00	0.20
5695	PSMB7	-0.02	0.00	0.00	0.06
5696	PSMB8	0.00	0.00	0.05	0.09
5698	PSMB9	0.00	0.00	0.05	0.07
5699	PSMB10	-0.04	0.00	0.00	0.03
5700	PSMC1	-0.02	0.00	0.03	0.61
5702	PSMC3	-0.02	0.00	-0.03	0.18
5704	PSMC4	0.02	0.00	0.05	0.11
5705	PSMC5	0.16	0.27	0.03	0.48
5706	PSMC6	0.00	0.00	0.00	0.15
5707	PSMD1	0.00	0.00	0.00	NaN
5708	PSMD2	0.05	0.00	0.05	0.17
5709	PSMD3	0.04	0.55	0.05	0.70
5710	PSMD4	0.07	0.09	0.19	0.25
5711	PSMD5	-0.02	0.00	0.00	0.02
5713	PSMD7	-0.09	0.00	0.00	0.11
5714	PSMD8	0.02	0.09	0.05	0.21
5715	PSMD9	0.02	0.00	0.00	0.09

5716	PSMD10	-0.05	0.00	-0.08	0.11
5717	PSMD11	0.00	0.36	0.00	0.53
5718	PSMD12	0.11	0.27	0.03	0.65
5719	PSMD13	0.00	0.00	0.00	NaN
5720	PSME1	-0.02	0.00	0.00	0.08
5721	PSME2	-0.02	0.00	0.00	0.14
5723	PSPH	0.00	0.00	0.05	0.43
5724	PTAFR	-0.02	0.09	0.00	-0.05
5725	PTBP1	0.00	0.00	-0.03	0.03
5728	PTEN	-0.04	0.00	-0.11	0.42
5729	PTGDR	0.02	0.00	0.00	0.03
5730	PTGDS	0.02	0.00	0.03	0.01
5731	PTGER1	0.02	-0.09	0.00	0.10
5732	PTGER2	0.02	0.00	0.00	0.15
5733	PTGER3	-0.02	0.00	-0.03	0.11
5734	PTGER4	0.04	0.18	0.00	-0.04
5737	PTGFR	-0.02	0.00	-0.03	0.07
5739	PTGIR	-0.02	0.00	-0.03	0.06
5740	PTGIS	0.05	0.00	0.05	0.13
5741	PTH	-0.02	0.00	-0.05	0.13
5742	PTGS1	-0.02	0.00	0.00	0.06
5743	PTGS2	0.04	0.09	0.03	-0.01
5744	PTHLH	-0.02	-0.09	0.05	0.08
5745	PTHR1	-0.04	0.00	0.00	0.09
5746	PTHR2	0.00	0.00	0.00	NaN
5747	PTK2	0.16	0.00	0.38	0.37
5753	PTK6	0.05	0.09	0.08	0.11
5754	PTK7	0.00	0.00	0.08	0.26
5757	PTMA	0.00	0.00	0.00	NaN
5763	PTMS	-0.02	0.09	0.05	0.10
5764	PTN	0.02	0.00	0.03	0.11
5768	QSCN6	0.04	0.00	0.03	0.11
5770	PTPN1	0.07	0.09	0.05	0.37
5771	PTPN2	-0.02	-0.09	0.00	0.03
5774	PTPN3	-0.02	0.00	0.00	0.05
5775	PTPN4	0.00	0.00	0.00	NaN
5777	PTPN6	-0.02	0.09	0.05	0.08
5778	PTPN7	0.09	0.00	0.05	-0.08
5780	PTPN9	0.00	0.00	0.00	NaN
5781	PTPN11	0.00	0.00	0.00	NaN
5782	PTPN12	0.02	0.00	0.03	0.15
5783	PTPN13	-0.04	0.00	-0.03	-0.10
5784	PTPN14	0.07	0.00	0.00	0.03
5786	PTPRA	0.02	0.00	-0.03	0.17
5787	PTPRB	0.02	0.00	0.03	0.15
5788	PTPRC	0.09	0.00	0.03	-0.14
5789	PTPRD	-0.07	0.00	-0.05	0.02

5790	PTPRCAP	0.07	0.18	0.05	0.14
5791	PTPRE	-0.04	0.00	0.00	0.22
5792	PTPRF	0.00	0.00	0.03	0.19
5793	PTPRG	-0.05	0.00	0.00	-0.01
5794	PTPRH	0.00	0.09	-0.03	-0.18
5795	PTPRJ	-0.02	0.00	0.00	0.13
5796	PTPRK	-0.02	0.00	0.03	0.11
5797	PTPRM	0.00	-0.09	0.00	0.01
5798	PTPRN	0.00	0.00	0.00	NaN
5799	PTPRN2	0.02	0.09	-0.03	0.23
5801	PTPRR	0.02	0.00	0.03	0.15
5802	PTPRS	-0.02	0.00	0.00	-0.09
5803	PTPRZ1	0.00	0.00	-0.03	0.00
5805	PTS	-0.07	0.00	0.00	0.08
5810	RAD1	0.02	0.27	0.00	0.52
5813	PURA	0.00	0.00	0.00	NaN
5816	PVALB	-0.04	0.00	0.03	0.08
5817	PVR	-0.02	0.00	-0.03	0.19
5818	PVRL1	-0.07	0.00	0.00	0.11
5819	PVRL2	-0.02	0.00	-0.03	-0.08
5825	ABCD3	-0.02	0.00	0.00	0.15
5826	ABCD4	-0.02	0.00	0.00	0.08
5827	PXMP2	0.00	0.00	0.00	NaN
5828	PXMP3	0.09	0.09	0.08	0.16
5829	PXN	0.02	0.00	0.00	0.41
5833	PCYT2	0.00	0.09	0.08	-0.09
5834	PYGB	0.02	0.00	-0.03	0.00
5836	PYGL	0.02	0.00	0.00	0.01
5837	PYGM	0.00	-0.09	0.03	0.05
5858	PZP	0.00	-0.18	0.05	0.11
5859	QARS	0.00	0.00	0.00	NaN
5860	QDPR	-0.04	-0.09	-0.05	0.20
5861	RAB1A	0.00	0.00	0.00	NaN
5862	RAB2	0.16	0.00	0.14	0.41
5864	RAB3A	0.02	0.00	0.00	-0.01
5865	RAB3B	0.00	0.00	-0.03	-0.06
5866	RAB3IL1	-0.04	0.00	0.03	0.07
5867	RAB4A	0.07	0.00	0.03	0.29
5868	RAB5A	-0.05	0.00	-0.05	0.32
5869	RAB5B	0.04	0.00	0.00	0.16
5870	RAB6A	0.05	0.09	-0.03	0.40
5871	MAP4K2	0.00	-0.09	0.03	0.07
5872	RAB13	0.05	0.00	0.14	0.06
5873	RAB27A	-0.02	0.00	-0.05	0.20
5874	RAB27B	-0.02	-0.09	-0.03	0.25
5875	RABGGTA	-0.02	0.00	0.00	0.03
5876	RABGGTB	-0.02	0.00	-0.03	0.16

5877	RABIF	0.13	0.00	0.05	0.29
5878	RAB5C	-0.04	0.09	0.00	0.21
5879	RAC1	0.00	0.00	0.00	NaN
5880	RAC2	-0.04	0.00	0.03	-0.07
5881	RAC3	0.00	0.09	0.08	-0.09
5884	RAD17	0.00	0.00	-0.08	0.17
5885	RAD21	0.22	0.27	0.35	0.42
5886	RAD23A	0.00	-0.09	0.00	0.10
5887	RAD23B	-0.02	0.00	0.00	0.03
5888	RAD51	-0.02	0.00	-0.14	0.13
5889	RAD51C	0.18	0.00	0.03	0.38
5890	RAD51L1	-0.02	0.00	0.00	0.10
5891	RAGE	0.00	0.00	-0.03	0.07
5892	RAD51L3	-0.02	0.09	0.03	0.35
5893	RAD52	0.00	0.09	0.08	0.24
5894	RAF1	0.02	0.00	0.00	0.26
5896	RAG1	0.04	0.00	-0.03	0.23
5897	RAG2	0.04	0.00	-0.03	0.09
5898	RALA	-0.02	0.00	0.03	0.25
5899	RALB	0.02	0.00	0.00	0.08
5900	RALGDS	0.02	0.00	0.00	0.17
5901	RAN	0.00	0.00	0.00	NaN
5902	RANBP1	-0.02	0.00	0.08	0.21
5903	RANBP2	0.00	0.00	0.00	NaN
5905	RANGAP1	-0.02	0.00	-0.03	0.21
5906	RAP1A	0.02	0.00	-0.03	-0.01
5908	RAP1B	0.02	0.09	0.00	0.18
5910	RAP1GDS1	-0.04	0.00	0.03	0.06
5911	RAP2A	-0.02	0.09	-0.05	0.16
5912	RAP2B	0.02	0.00	0.03	0.16
5913	RAPSN	-0.02	0.00	-0.03	-0.22
5914	RARA	0.02	0.09	0.03	0.35
5915	RARB	-0.04	0.00	-0.05	0.05
5917	RARS	0.00	0.00	0.00	0.25
5918	RARRES1	0.02	0.00	0.03	0.13
5919	RARRES2	0.02	0.00	0.00	-0.20
5920	RARRES3	0.00	0.00	0.03	-0.13
5921	RASA1	0.00	0.00	-0.14	0.18
5922	RASA2	0.02	0.00	0.03	0.14
5923	RASGRF1	0.00	0.00	-0.03	-0.11
5925	RB1	-0.09	0.00	-0.16	0.13
5928	RBBP4	0.00	0.00	0.00	NaN
5929	RBBP5	0.07	0.00	0.03	0.21
5930	RBBP6	0.02	0.00	0.00	0.19
5931	RBBP7	-0.05	0.00	-0.11	0.28
5932	RBBP8	0.00	-0.09	0.03	0.20
5934	RBL2	-0.05	0.00	0.00	0.19

5935	RBM3	-0.04	0.00	-0.05	0.27
5936	RBM4	0.07	0.00	0.03	0.17
5937	RBMS1	0.00	0.00	0.03	0.24
5939	RBMS2	0.00	0.00	0.00	NaN
5947	RBP1	0.02	0.00	0.00	0.34
5949	RBP3	-0.02	0.00	0.00	0.05
5950	RBP4	-0.02	0.00	-0.05	0.18
5954	RCN1	0.04	0.00	-0.03	0.28
5955	RCN2	0.00	0.00	-0.11	0.25
5959	RDH5	0.02	0.00	0.00	0.14
5962	RDX	-0.07	-0.09	0.00	0.30
5965	RECQL	0.00	-0.09	0.05	0.25
5966	REL	0.02	0.00	0.00	0.02
5967	REG1A	0.00	0.00	0.00	NaN
5968	REG1B	0.00	0.00	0.00	NaN
5970	RELA	0.02	0.00	0.05	0.17
5971	RELB	-0.02	0.00	-0.03	0.18
5972	REN	0.07	0.00	0.05	-0.06
5973	RENBP	0.00	0.00	-0.03	-0.11
5978	REST	-0.02	0.00	0.00	0.09
5979	RET	0.00	0.09	-0.03	0.12
5980	REV3L	0.02	0.00	0.05	0.24
5981	RFC1	-0.05	0.00	-0.03	0.10
5982	RFC2	0.02	0.00	0.05	0.08
5983	RFC3	-0.05	0.00	-0.08	0.14
5984	RFC4	0.04	0.00	0.03	0.32
5985	RFC5	0.00	0.00	-0.03	0.06
5988	RFPL1	0.00	0.00	0.03	0.17
5989	RFX1	0.02	-0.09	0.00	0.00
5990	RFX2	0.00	-0.09	0.00	-0.01
5991	RFX3	-0.05	0.00	-0.08	0.08
5992	RFX4	0.00	0.00	0.00	NaN
5993	RFX5	0.07	0.09	0.19	0.07
5994	RFXAP	-0.05	0.00	-0.11	-0.01
5995	RGR	-0.02	0.00	-0.05	-0.11
5996	RGS1	0.07	0.00	0.03	0.06
5997	RGS2	0.07	0.00	0.03	-0.16
5998	RGS3	-0.02	0.00	0.00	0.04
5999	RGS4	0.05	0.00	0.14	0.20
6000	RGS7	0.07	0.00	0.05	0.12
6001	RGS10	-0.04	0.00	-0.03	0.05
6002	RGS12	-0.02	0.00	0.00	0.13
6003	RGS13	0.07	0.00	0.03	-0.06
6004	RGS16	0.04	0.00	0.03	0.01
6005	RHAG	0.00	0.00	0.08	-0.02
6010	RHO	0.00	0.00	-0.03	0.22
6013	RLN1	-0.05	0.00	-0.05	0.00

6015	RING1	0.00	0.00	0.05	0.20
6017	RLBP1	0.02	0.00	-0.05	0.05
6018	RLF	-0.02	0.00	0.00	0.10
6019	RLN2	-0.05	0.00	-0.05	0.04
6036	RNASE2	-0.02	0.00	-0.03	0.12
6037	RNASE3	-0.02	0.00	-0.03	-0.03
6038	RNASE4	-0.02	0.00	-0.03	0.11
6039	RNASE6	-0.02	0.00	-0.03	0.29
6040	RNASEH1	-0.02	0.00	0.00	0.10
6041	RNASEL	0.04	0.00	0.03	0.11
6045	RNF2	0.07	0.00	0.03	0.04
6046	BRD2	0.00	0.00	0.05	0.24
6047	RNF4	-0.02	0.00	0.00	0.12
6048	RNF5	0.00	0.00	0.05	0.15
6049	RNF6	-0.05	0.00	-0.08	0.23
6051	RNPEP	0.09	0.00	0.05	0.18
6059	ABCE1	0.00	0.00	0.00	NaN
6091	ROBO1	-0.05	0.00	-0.03	-0.07
6093	ROCK1	0.00	-0.09	0.00	0.14
6094	ROM1	-0.02	0.00	0.03	0.05
6095	RORA	0.02	0.00	-0.05	0.07
6096	RORB	-0.02	0.00	0.00	-0.05
6097	RORC	0.07	0.00	0.19	0.05
6098	ROS1	0.02	0.00	0.00	0.04
6102	RP2	-0.05	0.00	-0.11	0.22
6103	RPGR	-0.05	0.00	-0.11	0.08
6117	RPA1	-0.05	0.00	0.00	0.15
6118	RPA2	-0.02	0.09	0.00	0.27
6119	RPA3	0.00	0.00	0.03	0.17
6120	RPE	0.00	0.00	0.00	NaN
6121	RPE65	-0.02	0.00	0.00	0.14
6122	RPL3	-0.04	0.00	-0.03	0.35
6123	RPL3L	0.04	0.18	0.00	0.01
6124	RPL4	0.00	0.09	-0.05	0.02
6125	RPL5	-0.02	0.00	-0.03	0.02
6128	RPL6	0.00	0.00	0.00	NaN
6129	RPL7	0.15	0.09	0.11	0.17
6132	RPL8	0.15	0.00	0.30	0.23
6133	RPL9	-0.05	0.00	-0.03	0.08
6134	RPL10	0.00	0.00	-0.03	0.65
6135	RPL11	0.00	0.00	0.00	NaN
6136	RPL12	0.00	0.00	0.00	0.02
6137	RPL13	-0.04	0.00	0.03	-0.02
6138	RPL15	-0.04	0.00	-0.05	0.11
6139	RPL17	-0.02	-0.09	-0.03	0.16
6141	RPL18	0.00	0.00	-0.03	0.58
6142	RPL18A	0.02	0.00	0.00	0.18

6143	RPL19	0.00	0.55	0.05	0.49
6144	RPL21	-0.05	0.00	-0.08	0.02
6146	RPL22	-0.02	0.00	0.00	0.08
6147	RPL23A	0.04	0.09	0.00	0.26
6150	MRPL23	0.00	0.00	0.00	NaN
6152	RPL24	0.00	0.00	-0.03	0.73
6155	RPL27	-0.04	0.00	0.00	0.12
6156	RPL30	0.16	0.18	0.27	0.22
6157	RPL27A	0.00	0.00	-0.08	-0.07
6158	RPL28	0.00	0.09	-0.03	0.49
6159	RPL29	0.00	0.00	0.00	NaN
6160	RPL31	0.02	0.00	0.00	0.19
6161	RPL32	0.04	0.00	0.00	0.17
6164	RPL34	-0.02	0.00	0.00	0.03
6166	RPL36AL	0.02	0.00	0.00	0.15
6167	RPL37	0.04	0.18	0.00	0.26
6168	RPL37A	0.02	0.00	0.00	0.25
6169	RPL38	0.04	0.18	0.08	0.37
6170	RPL39	-0.05	0.00	-0.08	0.28
6171	RPL41	0.04	0.00	0.00	0.15
6173	RPL36A	-0.05	0.00	-0.08	0.25
6175	RPLP0	0.02	0.00	0.00	0.21
6176	RPLP1	0.00	0.00	-0.05	0.02
6181	RPLP2	0.00	0.00	0.00	NaN
6182	MRPL12	0.00	0.09	0.08	0.04
6183	MRPS12	0.02	0.09	0.05	0.21
6184	RPN1	0.00	0.00	-0.03	0.45
6185	RPN2	0.02	0.00	0.03	0.25
6187	RPS2	0.04	0.18	0.00	0.02
6188	RPS3	0.07	0.09	0.00	0.31
6189	RPS3A	-0.02	0.00	0.00	0.11
6191	RPS4X	-0.04	0.00	-0.08	0.35
6193	RPS5	0.00	0.18	0.00	0.44
6194	RPS6	-0.04	0.00	0.00	0.16
6195	RPS6KA1	-0.02	0.09	0.00	0.19
6196	RPS6KA2	-0.02	0.00	-0.03	0.02
6197	RPS6KA3	-0.05	0.00	-0.11	0.00
6198	RPS6KB1	0.22	0.18	0.03	0.62
6199	RPS6KB2	0.07	0.18	0.05	0.26
6201	RPS7	-0.02	0.00	0.00	0.07
6202	RPS8	0.00	0.00	0.05	0.21
6203	RPS9	0.00	0.09	0.00	0.25
6204	RPS10	0.00	0.00	0.05	0.01
6205	RPS11	0.02	0.00	-0.03	0.28
6206	RPS12	-0.02	0.00	0.11	0.06
6207	RPS13	0.00	0.00	-0.05	-0.07
6208	RPS14	0.00	0.00	0.00	NaN

6209	RPS15	0.04	0.09	-0.03	0.08
6210	RPS15A	0.02	0.09	0.00	0.01
6217	RPS16	0.02	0.00	0.08	0.16
6222	RPS18	0.00	0.00	0.05	-0.02
6223	RPS19	0.02	0.00	-0.03	0.43
6224	RPS20	0.11	0.00	0.14	0.11
6227	RPS21	0.05	0.09	0.08	0.20
6228	RPS23	0.00	0.09	-0.14	0.07
6229	RPS24	-0.02	0.00	0.03	0.16
6230	RPS25	-0.05	0.00	0.00	0.06
6231	RPS26	0.04	0.00	0.00	0.14
6232	RPS27	0.05	0.00	0.14	0.07
6233	RPS27A	0.00	0.00	0.00	NaN
6234	RPS28	0.00	-0.09	0.00	0.06
6235	RPS29	0.02	0.00	0.00	0.11
6236	RRAD	-0.05	0.00	0.00	-0.01
6237	RRAS	0.07	0.09	-0.03	0.20
6238	RRBP1	0.00	0.00	-0.08	0.38
6239	RREB1	-0.02	0.00	0.05	0.19
6240	RRM1	-0.02	0.00	-0.03	0.05
6241	RRM2	-0.02	0.00	0.00	0.09
6247	RS1	-0.05	-0.09	-0.11	-0.04
6248	RSC1A1	-0.02	0.00	0.00	0.09
6251	RSU1	-0.02	0.00	0.08	0.18
6252	RTN1	0.02	0.00	-0.03	0.14
6253	RTN2	-0.02	0.00	-0.03	0.10
6256	RXRA	0.13	0.00	0.05	-0.02
6257	RXRB	0.00	0.00	0.05	0.28
6258	RXRG	0.04	0.00	0.08	-0.05
6259	RYK	0.00	0.00	0.00	NaN
6261	RYR1	0.02	0.09	0.05	0.04
6262	RYR2	0.07	0.00	0.03	0.03
6263	RYR3	-0.02	0.00	-0.16	-0.10
6272	SORT1	0.00	0.00	-0.03	0.15
6278	S100A7	0.05	0.00	0.16	0.12
6279	S100A8	0.05	0.00	0.19	0.06
6280	S100A9	0.05	0.00	0.19	0.06
6281	S100A10	0.07	0.00	0.19	0.16
6282	S100A11	0.07	0.00	0.19	0.01
6283	S100A12	0.05	0.00	0.19	-0.06
6284	S100A13	0.07	0.00	0.16	0.24
6285	S100B	0.00	0.09	0.00	0.00
6286	S100P	-0.02	0.00	0.00	-0.14
6289	SAA2	0.00	0.00	-0.05	0.19
6291	SAA4	0.00	0.00	-0.05	-0.01
6294	SAFB	-0.02	0.00	0.00	0.08
6295	SAG	0.00	0.00	0.00	NaN

6299	SALL1	-0.09	0.00	-0.03	0.24
6301	SARS	0.00	0.00	-0.03	-0.07
6304	SATB1	-0.02	0.00	-0.03	0.18
6305	SBF1	0.00	0.09	0.03	0.16
6307	SC4MOL	0.00	0.00	-0.05	0.07
6309	SC5DL	-0.07	0.00	0.00	-0.03
6317	SERPINB3	-0.02	-0.09	0.00	0.19
6318	SERPINB4	-0.02	-0.09	0.00	0.09
6319	SCD	-0.02	0.00	-0.03	0.02
6322	SCML1	-0.05	-0.09	-0.11	0.01
6323	SCN1A	-0.02	0.00	0.03	0.11
6324	SCN1B	-0.02	0.09	0.08	-0.09
6326	SCN2A2	-0.02	0.00	0.03	0.00
6327	SCN2B	-0.05	0.00	0.00	0.01
6328	SCN3A	-0.02	0.00	0.03	-0.02
6329	SCN4A	0.15	0.18	0.03	0.14
6331	SCN5A	-0.04	0.00	0.00	0.00
6334	SCN8A	0.02	0.00	-0.03	0.12
6335	SCN9A	-0.02	0.00	0.03	-0.08
6336	SCN10A	-0.04	0.00	0.00	0.02
6337	SCNN1A	-0.02	0.09	0.05	0.03
6338	SCNN1B	0.02	0.00	0.00	0.05
6339	SCNN1D	0.00	0.00	0.05	-0.05
6340	SCNN1G	0.02	0.00	0.00	-0.18
6342	SCP2	0.00	0.00	-0.03	0.02
6344	SCTR	0.00	0.00	0.00	NaN
6382	SDC1	0.00	0.00	0.00	NaN
6383	SDC2	0.18	0.18	0.27	0.09
6385	SDC4	0.04	0.09	0.03	0.39
6386	SDCBP	0.15	0.00	0.14	0.06
6388	SDF2	0.04	0.09	0.00	0.50
6389	SDHA	0.02	0.18	0.00	0.26
6390	SDHB	0.00	0.00	0.00	NaN
6391	SDHC	0.11	0.00	0.14	0.51
6392	SDHD	-0.07	0.00	0.00	0.03
6397	SEC14L1	0.04	0.00	0.00	0.19
6398	SECTM1	-0.04	0.09	0.08	0.01
6400	SEL1L	0.00	0.00	0.00	0.28
6401	SELE	0.02	0.00	0.05	0.20
6402	SELL	0.02	0.00	0.05	-0.04
6403	SELP	0.02	0.00	0.05	-0.02
6404	SELPLG	0.00	0.00	0.00	NaN
6405	SEMA3F	0.00	0.00	0.03	0.15
6406	SEMG1	0.04	0.09	0.03	0.05
6414	SEPP1	0.02	0.09	0.00	0.18
6415	SEPW1	-0.02	0.00	0.00	0.28
6416	MAP2K4	-0.09	0.00	-0.03	0.15

6418	SET	0.02	0.00	0.00	0.10
6419	SETMAR	-0.02	0.00	0.00	0.15
6421	SFPQ	-0.02	0.00	0.00	0.03
6422	SFRP1	0.05	0.09	0.03	-0.03
6424	SFRP4	-0.02	0.00	-0.03	0.02
6425	SFRP5	-0.02	0.00	-0.03	0.05
6426	SFRS1	0.09	0.00	0.03	0.18
6427	SFRS2	0.04	0.00	0.03	0.12
6428	SFRS3	0.02	0.00	0.05	0.14
6429	SFRS4	-0.02	0.09	0.00	0.13
6430	SFRS5	-0.02	0.00	0.00	0.17
6431	SFRS6	0.02	0.09	0.03	0.14
6432	SFRS7	0.00	0.00	0.00	NaN
6433	SFRS8	0.00	0.00	0.00	NaN
6434	SFRS10	0.05	0.00	0.03	0.19
6439	SFTPB	0.02	0.00	0.00	0.09
6440	SFTPC	-0.13	-0.18	-0.22	0.05
6441	SFTPD	-0.02	0.00	0.03	0.14
6442	SGCA	0.15	0.27	0.11	0.17
6443	SGCB	-0.02	0.00	0.00	0.02
6444	SGCD	0.00	0.00	0.00	NaN
6445	SGCG	-0.07	0.00	-0.11	0.05
6446	SGK	-0.02	0.00	0.08	0.22
6448	SGSH	0.00	0.00	-0.03	-0.01
6450	SH3BGR	0.00	0.00	0.00	0.21
6451	SH3BGRL	-0.04	0.00	-0.08	0.26
6452	SH3BP2	-0.02	0.00	0.00	0.03
6453	ITSN1	0.00	0.00	0.00	0.17
6455	SH3GL1	0.00	0.00	0.00	NaN
6456	SH3GL2	-0.04	0.00	0.03	0.11
6457	SH3GL3	0.00	0.00	-0.03	0.06
6461	SHB	-0.05	0.00	0.03	-0.15
6462	SHBG	-0.07	0.00	0.00	0.03
6464	SHC1	0.09	0.00	0.14	0.24
6469	SHH	0.02	0.00	-0.03	0.12
6470	SHMT1	-0.05	0.00	0.00	-0.12
6472	SHMT2	0.02	0.18	0.00	0.12
6474	SHOX2	0.02	0.00	0.03	0.11
6476	SI	0.02	0.00	0.00	-0.11
6477	SIAH1	-0.09	0.09	0.03	0.15
6478	SIAH2	0.02	0.00	0.05	0.01
6490	SILV	0.04	0.00	0.00	-0.25
6493	SIM2	0.00	0.00	0.00	0.15
6494	SIPA1	0.02	0.00	0.05	0.13
6495	SIX1	0.00	0.00	0.00	0.00
6496	SIX3	0.00	0.00	0.00	NaN
6497	SKI	-0.02	0.00	0.03	0.25

6498	SKIL	0.05	0.00	0.05	0.11
6499	SKIV2L	0.00	0.00	0.05	0.01
6502	SKP2	0.02	0.27	0.00	0.12
6505	SLC1A1	-0.05	0.00	-0.05	0.16
6506	SLC1A2	0.04	0.00	-0.03	0.17
6507	SLC1A3	0.02	0.27	0.00	0.16
6508	SLC4A3	0.00	0.00	0.00	NaN
6509	SLC1A4	0.00	0.00	0.00	NaN
6510	SLC1A5	-0.04	0.00	-0.03	0.13
6511	SLC1A6	0.02	0.00	0.00	-0.01
6512	SLC1A7	0.00	0.00	-0.03	0.10
6513	SLC2A1	0.02	0.00	0.00	0.21
6514	SLC2A2	0.05	0.00	0.05	0.00
6515	SLC2A3	0.00	-0.09	0.05	0.17
6517	SLC2A4	-0.05	0.00	0.00	0.04
6518	SLC2A5	-0.02	0.00	0.00	0.05
6519	SLC3A1	0.00	0.00	0.00	NaN
6520	SLC3A2	0.00	0.00	0.03	0.11
6521	SLC4A1	-0.02	0.00	0.00	0.08
6522	SLC4A2	0.04	0.00	0.08	-0.10
6523	SLC5A1	0.00	0.00	0.03	0.17
6524	SLC5A2	0.04	0.09	0.03	0.06
6525	SMTN	0.00	0.00	0.05	0.00
6527	SLC5A4	-0.02	0.00	0.03	0.18
6528	SLC5A5	0.02	0.00	0.00	-0.12
6529	SLC6A1	-0.02	0.00	0.00	-0.12
6530	SLC6A2	-0.07	0.00	0.00	0.20
6531	SLC6A3	0.02	0.18	0.00	-0.04
6532	SLC6A4	0.04	0.09	0.00	0.05
6533	SLC6A6	0.02	0.00	0.00	0.03
6534	SLC6A7	0.00	0.00	0.00	NaN
6536	SLC6A9	0.00	0.00	0.03	0.11
6538	SLC6A11	-0.02	0.00	0.00	0.06
6539	SLC6A12	0.00	0.09	0.05	0.10
6540	SLC6A13	0.00	0.09	0.05	0.07
6541	SLC7A1	-0.05	0.00	-0.05	-0.06
6542	SLC7A2	-0.16	-0.18	-0.22	0.06
6546	SLC8A1	0.00	0.00	0.00	NaN
6548	SLC9A1	-0.02	0.00	0.00	0.10
6549	SLC9A2	0.00	0.00	0.00	NaN
6550	SLC9A3	0.04	0.27	0.00	0.09
6553	SLC9A5	-0.04	0.00	0.00	0.13
6554	SLC10A1	-0.02	0.00	0.00	-0.05
6555	SLC10A2	0.00	0.00	-0.05	0.11
6556	SLC11A1	0.02	0.00	0.00	0.02
6557	SLC12A1	-0.05	0.00	-0.08	0.00
6558	SLC12A2	0.00	0.00	-0.05	0.13

6559	SLC12A3	-0.05	0.00	0.00	0.24
6560	SLC12A4	-0.04	0.00	0.00	0.20
6561	SLC13A1	0.00	0.00	-0.03	0.19
6563	SLC14A1	0.00	-0.09	-0.03	-0.05
6564	SLC15A1	-0.02	0.00	-0.05	0.21
6565	SLC15A2	0.00	0.00	-0.03	-0.12
6566	SLC16A1	0.02	0.00	-0.03	0.01
6567	SLC16A2	-0.04	0.00	-0.08	-0.19
6568	SLC17A1	0.00	0.00	0.03	-0.15
6569	SLC34A1	0.00	0.00	0.00	NaN
6570	SLC18A1	-0.15	-0.18	-0.22	-0.04
6571	SLC18A2	-0.04	0.00	0.00	0.05
6573	SLC19A1	0.02	0.09	0.00	0.15
6574	SLC20A1	0.00	0.00	0.00	NaN
6575	SLC20A2	0.02	0.09	0.11	0.19
6576	SLC25A1	-0.02	0.00	0.03	0.01
6580	SLC22A1	-0.02	0.00	-0.05	-0.16
6581	SLC22A3	-0.02	0.00	-0.05	-0.13
6582	SLC22A2	-0.02	0.00	-0.05	0.03
6583	SLC22A4	0.00	0.00	-0.03	0.05
6584	SLC22A5	0.00	0.00	-0.03	0.04
6585	SLIT1	-0.02	0.00	-0.03	0.12
6586	SLIT3	0.00	0.00	-0.03	0.16
6588	SLN	-0.07	-0.09	0.00	0.10
6590	SLPI	0.04	0.09	0.03	-0.03
6591	SNAI2	0.13	0.00	0.11	0.12
6594	SMARCA1	-0.05	0.00	-0.08	0.09
6595	SMARCA2	-0.04	0.00	-0.11	0.31
6597	SMARCA4	0.02	0.00	0.00	0.18
6598	SMARCB1	-0.02	0.00	0.03	0.23
6599	SMARCC1	-0.04	0.00	0.00	0.14
6601	SMARCC2	0.00	0.00	0.00	NaN
6602	SMARCD1	0.02	0.00	-0.03	0.11
6604	SMARCD3	0.02	0.00	-0.03	0.02
6605	SMARCE1	0.02	0.09	0.00	0.35
6609	SMPD1	-0.02	0.00	-0.03	0.33
6610	SMPD2	0.00	0.00	0.00	0.19
6611	SMS	-0.05	0.00	-0.11	0.19
6615	SNAI1	0.07	0.00	0.05	-0.06
6616	SNAP25	0.00	0.00	-0.05	-0.11
6617	SNAPC1	0.00	0.00	0.00	0.06
6618	SNAPC2	0.00	-0.09	0.00	-0.02
6619	SNAPC3	-0.04	0.00	-0.03	0.36
6620	SNCB	0.00	0.00	-0.03	-0.02
6621	SNAPC4	0.02	0.00	0.03	0.17
6622	SNCA	-0.04	0.00	-0.03	0.20
6623	SNCG	-0.02	0.00	-0.03	0.12

6625	SNRP70	0.00	0.00	-0.03	0.69
6626	SNRPA	0.00	0.00	0.00	0.51
6627	SNRPA1	-0.02	0.00	0.00	0.13
6628	SNRPB	0.02	0.00	-0.03	0.12
6629	SNRPB2	0.00	0.00	-0.08	0.18
6631	SNRPC	0.00	0.00	0.05	0.14
6632	SNRPD1	0.00	-0.09	0.03	0.17
6633	SNRPD2	-0.02	0.00	0.00	0.03
6634	SNRPD3	0.00	0.00	0.03	-0.03
6635	SNRPE	0.07	0.00	0.05	0.31
6636	SNRPF	0.00	0.09	0.00	0.34
6637	SNRPG	0.02	0.00	0.00	0.15
6638	SNRPN	0.00	0.09	-0.08	0.01
6640	SNTA1	0.00	0.00	0.00	NaN
6641	SNTB1	0.18	0.09	0.35	0.16
6642	SNX1	0.00	0.09	-0.08	0.31
6643	SNX2	0.00	0.00	-0.03	0.17
6645	SNTB2	-0.09	0.00	0.03	0.43
6646	SOAT1	0.02	0.00	0.03	0.14
6647	SOD1	-0.02	0.00	-0.03	0.09
6648	SOD2	-0.02	0.00	-0.05	0.16
6649	SOD3	-0.05	-0.09	-0.05	0.04
6650	SOLH	0.07	0.18	0.14	0.02
6651	SON	0.00	0.00	0.00	0.26
6652	SORD	-0.04	0.00	-0.11	0.26
6653	SORL1	-0.07	0.00	0.00	0.22
6654	SOS1	0.00	0.00	0.00	NaN
6655	SOS2	0.02	0.00	0.00	0.18
6656	SOX1	0.00	0.00	-0.03	-0.03
6657	SOX2	0.04	0.00	0.00	0.08
6659	SOX4	0.00	0.00	0.05	0.09
6660	SOX5	0.00	0.00	0.08	-0.13
6662	SOX9	0.05	0.00	0.03	-0.11
6663	SOX10	-0.04	0.00	0.03	0.18
6664	SOX11	-0.02	0.00	0.00	0.12
6667	SP1	0.02	0.00	-0.03	0.14
6668	SP2	0.02	0.18	0.00	0.31
6670	SP3	-0.02	0.00	0.00	0.01
6671	SP4	-0.02	0.09	0.00	0.02
6672	SP100	-0.02	0.00	0.00	0.13
6674	SPAG1	0.18	0.18	0.27	0.06
6675	UAP1	0.05	0.00	0.14	0.28
6676	SPAG4	0.04	0.00	0.00	0.16
6677	SPAM1	0.00	0.00	-0.03	0.10
6678	SPARC	0.00	0.00	0.00	NaN
6687	SPG7	-0.04	0.00	0.03	0.04
6688	SPI1	-0.02	0.00	-0.03	0.23

6689	SPIB	0.00	0.00	-0.03	-0.17
6690	SPINK1	0.00	0.00	0.00	NaN
6691	SPINK2	-0.02	0.00	0.00	0.02
6692	SPINT1	0.00	0.00	-0.11	0.04
6694	SPP2	0.00	0.00	0.00	NaN
6696	SPP1	-0.04	0.00	-0.03	-0.02
6697	SPR	0.00	0.00	0.03	-0.04
6698	SPRR1A	0.07	0.00	0.16	-0.07
6699	SPRR1B	0.07	0.00	0.16	0.11
6701	SPRR2B	0.05	0.00	0.16	-0.12
6707	SPRR3	0.07	0.00	0.16	0.08
6708	SPTA1	0.02	0.00	0.16	0.12
6709	SPTAN1	0.02	0.00	0.00	0.23
6710	SPTB	-0.02	0.00	0.00	0.13
6711	SPTBN1	0.00	0.00	0.00	NaN
6712	SPTBN2	0.07	0.09	0.03	-0.03
6713	SQLE	0.20	0.09	0.38	0.39
6714	SRC	0.02	0.09	0.03	0.30
6715	SRD5A1	0.02	0.18	0.00	0.27
6716	SRD5A2	0.00	0.00	0.00	NaN
6717	SRI	0.02	0.00	0.00	0.12
6718	AKR1D1	0.02	0.00	0.03	0.09
6720	SREBF1	-0.05	0.00	0.00	-0.01
6721	SREBF2	-0.02	0.00	-0.03	0.29
6722	SRF	0.00	0.00	0.11	0.26
6723	SRM	0.00	0.00	0.00	NaN
6726	SRP9	0.07	0.00	0.03	-0.08
6727	SRP14	-0.02	0.00	-0.16	0.41
6728	SRP19	0.00	0.00	-0.05	0.11
6729	SRP54	0.02	0.09	0.00	0.43
6731	SRP72	-0.02	0.00	0.00	0.11
6732	SRPK1	0.00	0.00	0.05	0.21
6733	SRPK2	0.02	0.00	0.00	0.07
6734	SRPR	-0.05	0.00	-0.03	0.15
6741	SSB	0.00	0.00	0.03	0.15
6742	SSBP1	0.02	0.00	0.00	0.05
6744	SSFA2	0.00	0.00	0.00	NaN
6745	SSR1	-0.02	0.00	0.05	0.28
6746	SSR2	0.09	0.00	0.14	0.34
6747	SSR3	0.02	0.00	0.03	0.13
6748	SSR4	0.00	0.00	-0.03	0.40
6749	SSRP1	0.00	0.00	0.05	0.30
6750	SST	0.04	0.00	0.03	-0.10
6751	SSTR1	0.00	0.00	-0.03	0.04
6752	SSTR2	0.05	0.00	0.03	0.05
6753	SSTR3	-0.04	0.00	0.03	0.18
6756	SSX1	-0.04	0.00	-0.08	0.01

6758	SSX5	-0.04	0.00	-0.08	-0.02
6760	SS18	0.00	-0.09	0.03	0.09
6764	ST5	0.00	0.00	-0.08	-0.07
6767	ST13	-0.02	0.00	-0.03	0.33
6768	ST14	-0.05	0.00	-0.03	0.15
6769	STAC	0.02	0.00	0.00	-0.09
6770	STAR	0.16	0.09	0.11	-0.09
6772	STAT1	0.02	0.00	0.00	0.02
6773	STAT2	0.00	0.00	0.00	NaN
6774	STAT3	-0.04	0.00	0.00	0.11
6775	STAT4	0.02	0.00	0.00	-0.03
6776	STAT5A	-0.04	0.00	0.00	0.27
6777	STAT5B	-0.04	0.00	0.00	0.04
6778	STAT6	0.02	0.00	0.00	0.07
6779	STATH	-0.02	0.00	0.00	0.10
6781	STC1	-0.13	-0.18	-0.19	0.13
6782	STCH	-0.05	-0.18	0.05	0.24
6785	ELOVL4	-0.04	0.00	-0.03	-0.03
6786	STIM1	-0.02	0.00	-0.03	0.07
6787	NEK4	-0.02	0.00	0.00	-0.03
6788	STK3	0.16	0.09	0.30	0.19
6789	STK4	0.02	0.09	0.03	0.05
6793	STK10	0.00	0.00	-0.03	0.18
6794	STK11	0.00	0.00	-0.03	0.15
6801	STRN	0.00	0.00	0.00	NaN
6804	STX1A	0.02	0.00	0.05	0.20
6812	STXBP1	0.00	0.00	0.00	0.18
6813	STXBP2	0.00	-0.09	0.00	0.06
6814	STXBP3	-0.02	0.00	-0.03	0.13
6819	SULT1C1	0.00	0.00	0.00	NaN
6820	SULT2B1	0.00	0.00	-0.03	-0.29
6821	SUOX	0.04	0.00	0.00	0.18
6822	SULT2A1	-0.02	0.00	0.00	-0.22
6827	SUPT4H1	0.16	0.00	0.03	0.56
6829	SUPT5H	0.02	0.00	0.08	0.20
6830	SUPT6H	0.04	0.09	0.00	0.30
6832	SUPV3L1	0.00	0.00	0.03	0.30
6833	ABCC8	0.00	0.00	-0.05	0.05
6839	SUV39H1	-0.04	0.00	-0.05	-0.10
6840	SVIL	0.00	0.00	0.03	0.12
6843	VAMP1	-0.02	0.09	0.05	-0.03
6844	VAMP2	-0.05	0.00	0.00	0.19
6847	SYCP1	-0.02	0.00	-0.03	0.08
6850	SYK	-0.02	0.00	0.00	-0.05
6853	SYN1	-0.05	0.00	-0.08	0.06
6854	SYN2	0.00	0.00	0.00	0.03
6857	SYT1	-0.02	0.00	0.00	0.12

6861	SYT5	0.00	0.09	-0.03	-0.10
6862	T	-0.02	0.00	-0.03	-0.01
6863	TAC1	0.02	0.00	0.00	0.07
6865	TACR2	0.00	0.00	0.03	-0.03
6866	TAC3	0.02	0.00	0.00	-0.09
6867	TACC1	0.13	0.09	0.08	0.24
6868	ADAM17	-0.02	0.00	0.00	0.07
6869	TACR1	0.00	0.00	0.00	NaN
6870	TACR3	-0.04	0.00	0.00	0.23
6871	TADA2L	0.00	0.09	0.05	0.43
6872	TAF1	-0.04	0.00	-0.08	0.04
6874	TAF4	0.04	0.09	0.05	0.39
6876	TAGLN	-0.07	0.00	0.00	0.14
6877	TAF5	0.00	0.00	-0.03	0.05
6878	TAF6	0.02	0.00	0.00	0.28
6879	TAF7	0.00	0.00	0.00	NaN
6880	TAF9	0.00	0.00	-0.08	0.09
6881	TAF10	-0.02	0.00	-0.03	0.06
6883	TAF12	-0.02	0.09	0.00	0.06
6885	MAP3K7	-0.05	0.00	0.00	0.37
6886	TAL1	0.00	0.00	0.00	0.04
6888	TALDO1	0.00	0.00	0.00	NaN
6890	TAP1	0.00	0.00	0.05	0.14
6891	TAP2	0.00	0.00	0.05	0.16
6894	TARBP1	0.09	0.00	0.03	0.20
6895	TARBP2	0.02	0.09	-0.03	0.44
6897	TARS	0.02	0.27	0.00	0.42
6898	TAT	-0.07	0.00	0.03	0.05
6899	TBX1	-0.02	0.00	0.08	0.14
6900	CNTN2	0.07	0.00	0.03	-0.18
6902	TBCA	0.00	0.09	-0.14	0.30
6903	TBCC	0.00	0.00	0.08	0.17
6904	TBCD	-0.02	0.09	0.08	0.18
6905	TBCE	0.09	0.00	0.03	0.11
6906	SERPINA7	-0.05	0.00	-0.08	-0.01
6908	TBP	-0.02	0.00	-0.03	0.20
6909	TBX2	0.18	0.18	0.03	0.12
6910	TBX5	0.02	0.00	0.00	0.07
6915	TBXA2R	0.00	0.00	0.00	NaN
6916	TBXAS1	0.02	0.00	0.00	0.10
6917	TCEA1	0.13	0.00	0.14	0.24
6919	TCEA2	0.09	0.09	0.08	0.06
6921	TCEB1	0.11	0.00	0.08	0.29
6923	TCEB2	0.04	0.09	0.00	0.10
6924	TCEB3	0.00	0.00	0.00	NaN
6925	TCF4	-0.02	-0.09	-0.03	0.07
6926	TBX3	0.02	0.00	0.00	0.12

6927	TCF1	0.00	0.00	0.00	NaN
6928	TCF2	0.00	0.18	0.05	0.28
6929	TCF3	0.00	0.00	-0.03	0.14
6934	TCF7L2	-0.04	0.00	0.00	0.12
6935	TCF8	-0.02	0.00	0.08	-0.03
6936	C2orf3	0.00	0.00	0.00	NaN
6938	TCF12	-0.02	0.00	-0.05	0.09
6939	TCF15	0.02	0.00	-0.03	0.05
6940	ZNF354A	0.00	0.00	-0.03	0.02
6942	TCF20	-0.02	0.00	-0.03	0.10
6943	TCF21	-0.02	0.00	0.08	-0.06
6947	TCN1	-0.04	0.00	0.00	0.07
6948	TCN2	0.00	0.00	0.05	0.20
6949	TCOF1	0.00	0.00	0.00	NaN
6950	TCP1	-0.02	0.00	-0.05	0.38
6954	TCP11	0.00	0.00	0.05	0.04
6988	TCTA	0.00	0.00	0.00	NaN
6992	PPP1R11	0.00	0.00	0.05	0.03
6996	TDG	0.00	0.00	0.00	NaN
6997	TDGF1	-0.04	0.00	0.00	0.09
6999	TDO2	-0.02	0.00	-0.03	-0.14
7001	PRDX2	0.02	-0.09	0.00	0.16
7003	TEAD1	0.00	0.00	-0.05	0.05
7004	TEAD4	-0.02	0.09	0.08	0.13
7005	TEAD3	0.00	0.00	0.05	0.06
7006	TEC	-0.04	0.00	0.00	-0.05
7007	TECTA	-0.07	0.00	0.00	0.19
7008	TEF	-0.02	0.00	-0.03	0.04
7009	TEGT	0.02	0.00	-0.03	0.16
7010	TEK	-0.11	0.00	-0.05	0.10
7011	TEP1	-0.02	0.00	-0.03	0.01
7013	TERF1	0.13	0.09	0.11	0.36
7014	TERF2	-0.09	0.00	0.03	0.25
7015	TERT	0.00	0.36	0.00	0.27
7016	TESK1	0.00	0.00	0.00	-0.04
7018	TF	0.00	0.00	0.00	NaN
7019	TFAM	-0.02	0.00	-0.03	0.02
7020	TFAP2A	-0.02	0.00	0.11	0.10
7021	TFAP2B	-0.02	0.00	0.08	-0.07
7022	TFAP2C	0.04	0.09	0.05	0.25
7023	TFAP4	0.04	0.00	0.00	-0.06
7024	TFCP2	0.02	0.00	-0.03	0.17
7025	NR2F1	0.00	0.00	-0.14	0.04
7026	NR2F2	0.04	0.00	0.00	0.29
7027	TFDP1	0.00	0.00	0.00	0.06
7029	TFDP2	0.02	0.00	0.03	0.17
7031	TFF1	0.00	0.00	0.00	-0.06

7032	TFF2	0.00	0.00	0.00	0.06
7033	TFF3	0.00	0.00	0.00	0.09
7035	TFPI	0.02	0.00	0.00	0.10
7036	TFR2	0.02	0.00	0.00	0.12
7037	TFRC	0.04	0.09	0.00	0.18
7038	TG	0.20	0.09	0.35	0.09
7039	TGFA	0.02	0.00	0.00	0.02
7040	TGFB1	0.02	0.00	-0.03	0.01
7042	TGFB2	0.07	0.00	0.00	0.09
7043	TGFB3	-0.02	0.00	-0.03	0.07
7045	TGFBI	0.00	0.00	-0.03	-0.17
7046	TGFBR1	-0.02	0.00	0.00	0.03
7047	TGM4	-0.04	0.00	0.00	0.18
7048	TGFBR2	0.02	0.00	-0.05	0.08
7049	TGFBR3	-0.02	0.00	-0.03	0.04
7050	TGIF	0.00	-0.09	0.00	0.02
7051	TGM1	-0.02	0.00	0.00	0.04
7052	TGM2	0.00	0.00	0.03	0.13
7053	TGM3	0.02	0.00	-0.03	-0.02
7054	TH	0.00	0.00	0.00	NaN
7056	THBD	0.00	0.00	-0.05	0.17
7057	THBS1	-0.02	0.00	-0.16	0.24
7058	THBS2	-0.02	0.00	-0.03	0.00
7059	THBS3	0.09	0.00	0.14	0.31
7060	THBS4	0.00	0.00	-0.14	-0.02
7064	THOP1	0.00	0.00	0.00	NaN
7066	THPO	0.05	0.00	0.05	0.09
7067	THRA	0.04	0.45	0.05	0.23
7068	THRB	-0.04	0.00	-0.05	-0.01
7070	THY1	-0.07	0.09	0.03	0.22
7072	TIA1	0.02	0.00	0.00	0.28
7073	TIAL1	-0.04	0.00	-0.03	0.10
7074	TIAM1	-0.02	0.00	0.00	-0.01
7077	TIMP2	0.02	0.18	0.03	0.13
7080	TITF1	0.02	0.09	0.00	0.12
7082	TJP1	-0.04	0.09	-0.16	0.21
7083	TK1	0.05	0.09	0.03	0.18
7084	TK2	-0.09	0.00	0.00	0.06
7086	TKT	-0.02	0.00	0.00	0.25
7087	ICAM5	0.00	0.00	0.00	NaN
7088	TLE1	-0.02	0.00	0.00	0.20
7089	TLE2	0.00	0.00	0.00	NaN
7090	TLE3	0.02	0.00	-0.05	0.13
7091	TLE4	-0.02	0.00	0.00	0.01
7092	TLL1	0.00	0.00	-0.05	0.21
7093	TLL2	-0.02	0.00	-0.05	0.03
7095	TLOC1	0.05	0.00	0.05	0.44

7096	TLR1	-0.05	0.00	-0.03	0.12
7097	TLR2	-0.02	0.00	-0.03	0.06
7098	TLR3	-0.02	0.00	-0.08	0.11
7099	TLR4	-0.02	0.00	0.00	0.13
7100	TLR5	0.07	0.00	0.00	0.18
7101	NR2E1	0.04	0.00	0.00	0.09
7104	TM4SF4	0.02	0.00	0.03	0.18
7108	TM7SF2	0.02	0.00	0.03	0.16
7109	TMEM1	0.00	0.00	0.00	0.24
7110	TMF1	-0.05	0.00	0.00	0.06
7112	TMPO	0.00	0.00	0.00	NaN
7113	TMPRSS2	0.00	0.00	0.00	0.26
7122	CLDN5	-0.02	0.00	0.08	-0.09
7124	TNF	0.00	0.00	0.05	0.00
7125	TNNC2	0.04	0.00	0.03	0.06
7126	TNFAIP1	0.04	0.09	0.00	0.26
7127	TNFAIP2	0.00	0.00	0.00	NaN
7128	TNFAIP3	-0.02	0.00	0.03	0.14
7130	TNFAIP6	-0.02	0.00	0.03	0.04
7132	TNFRSF1A	-0.02	0.09	0.05	0.21
7133	TNFRSF1B	-0.02	0.00	0.00	0.10
7134	TNNC1	0.00	0.00	0.00	NaN
7135	TNNI1	0.07	0.00	0.05	0.02
7137	TNNI3	0.00	0.09	-0.03	-0.23
7139	TNNT2	0.07	0.00	0.05	-0.06
7140	TNNT3	0.00	0.00	0.00	NaN
7141	TNP1	0.02	0.00	0.00	0.09
7142	TNP2	0.04	0.00	0.00	0.01
7143	TNR	0.02	0.00	0.03	-0.11
7148	TNXB	0.00	0.00	0.05	0.16
7150	TOP1	0.04	0.00	0.00	0.13
7153	TOP2A	0.02	0.09	0.00	0.24
7155	TOP2B	-0.04	0.00	-0.05	0.24
7156	TOP3A	-0.05	0.00	0.00	0.04
7157	TP53	-0.07	0.00	0.00	0.17
7158	TP53BP1	-0.04	0.00	-0.14	0.23
7159	TP53BP2	0.07	0.00	0.00	0.10
7161	TP73	-0.02	0.00	0.00	0.01
7162	TPBG	0.00	0.00	-0.05	0.12
7163	TPD52	0.15	0.09	0.14	0.23
7164	TPD52L1	-0.02	0.00	0.03	0.10
7165	TPD52L2	0.09	0.00	0.08	0.23
7167	TPI1	-0.02	0.09	0.05	0.31
7168	TPM1	0.02	0.00	-0.05	0.12
7169	TPM2	0.00	0.00	0.00	0.05
7171	TPM4	0.00	0.00	0.00	NaN
7172	TPMT	0.00	0.00	0.08	0.26

7173	TPO	-0.02	0.00	0.00	-0.05
7174	TPP2	-0.02	0.00	-0.05	0.17
7175	TPR	0.04	0.09	0.03	0.42
7178	TPT1	-0.05	0.00	-0.11	0.09
7181	NR2C1	0.00	0.09	0.00	0.21
7182	NR2C2	0.02	0.00	0.00	-0.17
7185	TRAF1	-0.02	0.00	0.00	-0.01
7186	TRAF2	0.02	0.00	0.03	-0.20
7187	TRAF3	0.00	0.00	-0.03	0.33
7188	TRAF5	0.07	0.00	0.00	0.10
7189	TRAF6	0.04	0.00	-0.03	0.27
7200	TRH	0.00	0.00	-0.03	-0.12
7201	TRHR	0.20	0.09	0.27	0.03
7203	CCT3	0.04	0.00	0.11	0.19
7204	TRIO	0.05	0.27	0.03	0.25
7205	TRIP6	0.02	0.00	0.00	-0.14
7216	TRO	-0.04	0.00	-0.05	0.12
7220	TRPC1	0.02	0.00	0.03	0.13
7222	TRPC3	0.00	0.09	0.08	0.03
7223	TRPC4	-0.05	0.00	-0.11	0.18
7224	TRPC5	-0.05	0.00	-0.08	-0.10
7225	TRPC6	-0.04	-0.09	0.00	0.06
7226	TRPM2	0.00	0.00	0.00	0.21
7227	TRPS1	0.22	0.27	0.41	0.11
7247	TSN	0.00	0.00	0.00	NaN
7248	TSC1	0.02	0.00	0.00	0.04
7251	TSG101	0.00	0.00	-0.05	0.08
7252	TSHB	-0.02	0.00	-0.03	-0.06
7253	TSHR	0.00	0.00	0.00	0.11
7257	TSNAX	0.09	0.00	0.03	0.18
7260	TSSC1	-0.02	0.00	0.00	0.23
7263	TST	-0.04	0.00	0.03	-0.18
7264	TSTA3	0.16	0.00	0.35	0.17
7265	TTC1	0.00	0.00	-0.03	0.10
7266	DNAJC7	-0.04	0.09	0.00	0.21
7267	TTC3	0.00	0.00	0.00	0.19
7268	TTC4	0.00	0.00	0.00	0.22
7270	TTF1	0.02	0.00	0.03	0.24
7272	TTK	-0.04	0.00	-0.03	0.06
7273	TTN	0.00	0.00	0.00	NaN
7274	TTPA	0.13	0.00	0.14	0.19
7275	TUB	0.00	0.00	-0.08	-0.09
7276	TTR	0.02	-0.09	0.00	0.14
7277	TUBA1	0.00	0.00	0.00	NaN
7278	TUBA2	-0.05	0.00	-0.11	-0.14
7280	TUBB	0.00	0.00	0.05	0.14
7283	TUBG1	-0.04	0.00	0.00	-0.01

7284	TUFM	0.05	0.00	0.00	0.18
7286	TUFT1	0.07	0.09	0.19	0.34
7287	TULP1	0.00	0.00	0.05	-0.16
7288	TULP2	0.00	0.00	-0.03	-0.24
7289	TULP3	-0.02	0.09	0.08	0.34
7290	HIRA	-0.02	0.00	0.05	-0.10
7292	TNFSF4	0.02	0.00	0.05	0.00
7294	TXK	-0.04	0.00	0.00	0.24
7295	TXN	-0.02	0.00	0.00	0.04
7296	TXNRD1	0.00	0.00	0.00	NaN
7297	TYK2	0.00	0.00	0.00	NaN
7298	TYMS	0.02	-0.09	0.00	0.13
7299	TYR	-0.05	0.00	0.03	0.10
7301	TYRO3	-0.02	0.09	-0.14	0.04
7305	TYROBP	0.00	0.09	0.05	-0.10
7306	TYRP1	-0.05	0.00	-0.03	0.11
7307	U2AF1	0.00	0.00	-0.03	0.11
7311	UBA52	0.02	0.00	0.00	0.11
7316	UBC	0.02	0.00	0.00	0.05
7317	UBE1	-0.05	0.00	-0.08	0.21
7318	UBE1L	0.00	0.00	0.00	NaN
7319	UBE2A	-0.05	0.00	-0.08	0.33
7321	UBE2D1	-0.02	0.00	-0.03	0.08
7322	UBE2D2	0.00	0.00	0.00	NaN
7323	UBE2D3	-0.04	0.00	0.00	0.22
7324	UBE2E1	-0.04	0.00	-0.05	0.19
7326	UBE2G1	-0.04	0.00	0.00	0.27
7327	UBE2G2	0.00	0.00	0.00	0.23
7328	UBE2H	0.02	0.00	0.00	0.07
7329	UBE2I	0.04	0.18	0.03	0.26
7332	UBE2L3	0.00	0.00	0.03	0.19
7334	UBE2N	0.00	0.09	0.00	0.28
7336	UBE2V2	0.13	0.00	0.16	0.14
7337	UBE3A	0.00	0.09	-0.08	0.39
7342	UBP1	0.02	0.00	-0.03	0.32
7343	UBTF	-0.02	0.00	0.00	-0.01
7345	UCHL1	-0.05	0.00	-0.08	0.17
7347	UCHL3	-0.05	-0.09	-0.14	0.15
7348	UPK1B	0.00	0.00	-0.03	-0.25
7350	UCP1	-0.02	0.00	0.00	0.01
7351	UCP2	0.05	0.09	-0.03	0.26
7352	UCP3	0.05	0.09	-0.03	0.10
7353	UFD1L	-0.02	0.00	0.05	-0.15
7355	SLC35A2	-0.04	0.00	-0.05	0.06
7356	SCGB1A1	-0.02	0.00	0.03	0.11
7357	UGCG	-0.02	0.00	0.00	0.03
7358	UGDH	-0.05	0.00	-0.03	0.02

7360	UGP2	0.02	0.00	0.00	0.19
7363	UGT2B4	-0.02	0.00	0.00	0.03
7365	UGT2B10	-0.02	0.00	0.00	-0.07
7367	UGT2B17	-0.02	0.00	0.00	0.03
7368	UGT8	-0.02	0.00	0.03	0.20
7369	UMOD	0.02	-0.09	0.00	0.02
7372	UMPS	0.00	0.00	-0.03	0.22
7374	UNG	0.00	0.00	0.00	NaN
7375	USP4	0.00	0.00	0.00	NaN
7379	UPK2	-0.05	0.00	0.00	0.19
7381	UQCRB	0.18	0.18	0.24	0.42
7384	UQCRC1	-0.04	0.00	0.00	0.00
7385	UQCRC2	0.02	-0.09	0.00	0.26
7386	UQCRFS1	0.00	0.09	0.03	0.24
7388	UQCRH	0.00	0.00	0.03	0.21
7389	UROD	0.00	0.00	0.03	0.46
7390	UROS	-0.04	0.00	0.03	0.20
7392	USF2	0.02	0.00	0.05	-0.05
7398	USP1	-0.02	0.00	0.03	0.30
7399	USH2A	0.07	0.00	0.00	-0.01
7402	UTRN	0.02	0.00	0.03	0.10
7403	UTX	-0.05	0.00	-0.11	0.15
7404	UTY	0.02	0.00	-0.03	0.10
7405	UVRAG	0.07	0.09	-0.03	0.38
7408	VASP	-0.02	0.00	-0.03	0.33
7409	VAV1	0.00	-0.09	0.00	0.05
7410	VAV2	0.02	0.00	0.00	0.25
7411	VBP1	-0.05	0.00	-0.11	0.12
7412	VCAM1	-0.02	0.00	-0.03	-0.18
7414	VCL	0.04	0.00	0.00	0.49
7415	VCP	0.00	0.00	0.00	0.12
7416	VDAC1	0.00	0.00	-0.03	0.02
7419	VDAC3	0.02	0.09	0.11	0.44
7421	VDR	0.00	0.00	0.00	NaN
7423	VEGFB	0.00	0.00	0.03	0.08
7424	VEGFC	-0.02	0.00	-0.05	0.00
7425	VGF	0.02	0.00	0.00	0.09
7428	VHL	-0.02	0.00	0.00	-0.04
7429	VIL1	0.00	0.00	0.14	-0.05
7430	VIL2	-0.02	0.00	-0.03	0.42
7431	VIM	-0.02	0.00	0.08	0.12
7432	VIP	0.00	0.00	0.00	-0.04
7433	VIPR1	-0.04	0.00	0.00	-0.08
7434	VIPR2	0.02	0.00	-0.03	0.15
7436	VLDLR	-0.05	0.00	-0.08	0.22
7441	VPREB1	-0.02	0.00	0.03	-0.04
7442	TRPV1	-0.04	0.00	0.00	0.04

7443	VRK1	0.00	0.00	-0.03	0.36
7444	VRK2	0.00	0.00	0.00	NaN
7447	VSNL1	-0.02	0.00	0.00	0.06
7448	VTN	0.04	0.09	0.00	0.05
7450	VWF	-0.02	0.18	0.05	0.15
7453	WARS	0.00	0.00	0.03	0.15
7454	WAS	-0.04	0.00	-0.05	0.13
7464	CORO2A	-0.02	0.00	0.00	0.09
7465	WEE1	0.00	0.00	-0.05	0.05
7466	WFS1	-0.02	0.00	0.00	0.06
7468	WHSC1	-0.02	0.00	0.00	0.24
7469	WHSC2	-0.02	0.00	0.00	0.08
7471	WNT1	0.00	0.09	0.00	0.10
7472	WNT2	0.00	0.00	-0.03	-0.11
7473	WNT3	-0.04	0.00	0.00	0.15
7474	WNT5A	-0.04	0.00	-0.03	-0.04
7475	WNT6	0.00	0.00	0.00	NaN
7476	WNT7A	0.02	0.00	0.00	0.07
7479	WNT8B	-0.02	0.00	-0.03	0.16
7480	WNT10B	0.00	0.00	0.00	NaN
7481	WNT11	0.04	0.09	-0.03	-0.07
7482	WNT2B	0.02	0.00	-0.03	0.04
7485	WRB	0.00	0.00	0.00	0.18
7486	WRN	-0.11	-0.18	-0.14	0.31
7490	WT1	0.04	0.00	-0.03	0.23
7494	XBP1	-0.02	-0.09	0.03	-0.14
7498	XDH	0.00	0.00	0.00	NaN
7504	XK	-0.05	0.00	-0.11	-0.07
7507	XPA	-0.02	0.00	0.00	-0.03
7508	XPC	0.02	0.00	0.00	0.24
7511	XPNPEP1	-0.04	0.00	0.00	0.14
7512	XPNPEP2	-0.05	0.00	-0.08	-0.13
7514	XPO1	0.00	0.00	0.00	NaN
7515	XRCC1	-0.02	0.00	-0.03	0.22
7516	XRCC2	0.02	0.00	-0.03	0.14
7518	XRCC4	0.00	0.09	-0.14	0.23
7520	XRCC5	0.02	0.00	0.00	0.20
7525	YES1	0.02	-0.09	0.00	0.11
7528	YY1	0.00	0.00	0.03	0.41
7529	YWHAB	0.02	0.09	0.03	0.19
7531	YWHAE	-0.04	0.00	0.00	0.12
7533	YWHAH	0.00	0.00	0.03	0.14
7534	YWHAZ	0.20	0.18	0.27	0.44
7535	ZAP70	0.00	0.00	0.00	NaN
7536	SF1	0.00	-0.09	0.03	0.12
7538	ZFP36	0.02	0.00	0.08	0.11
7539	ZFP37	-0.02	0.00	0.00	0.15

7541	ZFP161	0.00	-0.09	-0.03	0.23
7542	ZFPL1	0.02	0.00	0.03	0.20
7543	ZFX	-0.05	0.00	-0.11	0.03
7544	ZFY	0.00	0.00	-0.05	0.01
7545	ZIC1	0.02	0.00	-0.03	0.07
7547	ZIC3	-0.02	0.00	-0.08	-0.02
7553	ZNF7	0.15	0.00	0.30	0.35
7554	ZNF8	0.00	0.18	0.00	0.01
7556	ZNF10	0.00	0.00	0.00	NaN
7561	ZNF14	0.02	0.00	0.00	0.17
7564	ZNF16	0.15	0.00	0.27	0.15
7568	ZNF20	0.00	-0.09	0.00	-0.04
7570	ZNF22	-0.02	0.09	0.03	0.13
7571	ZNF23	-0.07	0.00	0.03	0.10
7572	ZNF24	0.00	-0.09	-0.03	0.10
7574	ZNF26	0.00	0.00	0.00	NaN
7580	ZNF32	0.00	0.09	-0.03	0.12
7584	ZNF35	-0.04	0.00	0.00	0.15
7592	ZNF41	-0.05	0.00	-0.08	-0.15
7594	ZNF43	0.00	0.00	0.03	0.10
7595	ZNF44	0.00	-0.09	0.00	0.12
7596	ZNF45	-0.02	0.00	-0.03	-0.11
7625	ZNF74	-0.02	0.00	0.08	-0.14
7626	ZNF75	-0.04	0.00	-0.08	0.19
7629	ZNF76	0.00	0.00	0.05	0.24
7633	ZNF79	-0.02	0.00	0.00	0.05
7634	ZNF80	0.00	0.00	-0.03	-0.23
7637	ZNF84	0.00	0.00	0.00	NaN
7638	ZNF221	-0.02	0.00	-0.03	-0.06
7639	ZNF85	0.02	0.00	0.00	0.16
7644	ZNF91	0.00	0.00	0.00	NaN
7673	ZNF222	-0.02	0.00	-0.03	0.14
7678	ZNF124	0.07	0.00	0.03	0.17
7681	MKRN3	0.00	0.09	-0.08	0.15
7690	ZNF131	0.04	0.09	0.00	0.46
7691	ZNF132	0.00	0.18	0.00	-0.01
7692	ZNF133	0.00	0.00	-0.05	0.14
7693	ZNF134	0.00	0.18	0.00	0.11
7695	ZNF136	0.00	-0.09	0.00	0.07
7699	ZNF140	0.00	0.00	0.00	NaN
7700	ZNF141	-0.02	0.00	-0.05	0.14
7701	ZNF142	0.00	0.00	0.00	NaN
7702	ZNF143	0.00	0.00	-0.08	0.00
7705	ZNF146	-0.02	0.09	0.08	0.29
7707	ZNF148	0.00	0.00	-0.03	0.14
7711	ZNF155	-0.02	0.00	-0.03	0.21
7712	ZNF157	-0.05	0.00	-0.08	-0.19

7718	ZNF165	0.00	0.00	0.05	0.02
7726	TRIM26	0.00	0.00	0.05	0.12
7727	ZNF174	0.04	0.00	0.00	0.24
7728	ZNF175	0.00	0.00	-0.03	-0.23
7730	ZNF177	0.00	0.00	0.00	NaN
7733	ZNF180	-0.02	0.00	-0.03	-0.10
7738	ZNF184	0.00	0.00	0.05	0.20
7741	ZNF187	0.00	0.00	0.05	0.13
7743	ZNF189	-0.02	0.00	0.00	0.09
7745	ZNF192	0.00	0.00	0.05	-0.03
7746	ZNF193	0.00	0.00	0.05	0.10
7748	ZNF195	-0.02	0.00	-0.03	0.12
7752	ZNF200	0.04	0.00	0.00	0.10
7753	ZNF202	-0.05	0.00	-0.03	0.14
7755	ZNF205	0.04	0.00	0.00	0.15
7756	ZNF207	0.00	0.27	0.00	0.22
7757	ZNF208	0.00	0.00	0.00	0.12
7761	ZNF214	-0.02	0.00	-0.05	-0.03
7762	ZNF215	-0.02	0.00	-0.05	-0.04
7764	ZNF217	0.11	0.00	0.05	0.32
7766	ZNF223	-0.02	0.00	-0.03	0.37
7768	ZNF225	-0.02	0.00	-0.03	-0.14
7769	ZNF226	-0.02	0.00	-0.03	-0.10
7773	ZNF230	-0.02	0.00	-0.03	0.09
7775	ZNF232	-0.04	0.00	0.00	0.20
7776	ZNF236	-0.02	-0.09	0.00	0.12
7781	SLC30A3	0.00	0.00	0.00	NaN
7782	SLC30A4	-0.04	0.00	-0.11	-0.08
7783	ZP2	0.02	0.00	0.00	0.22
7786	MAP3K12	0.02	0.09	-0.03	0.40
7791	ZYX	0.02	0.00	0.00	0.06
7799	PRDM2	-0.02	0.00	0.00	0.20
7803	PTP4A1	0.00	0.00	0.05	0.48
7804	LRP8	0.00	0.00	0.00	0.16
7805	LAPTM5	-0.02	0.09	0.00	0.05
7813	EVI5	-0.02	0.00	-0.03	0.19
7818	DAP3	0.09	0.00	0.14	0.20
7827	NPHS2	0.04	0.00	0.03	-0.28
7832	BTG2	0.09	0.00	0.05	0.11
7840	ALMS1	0.00	0.00	0.00	NaN
7841	GCS1	0.00	0.00	0.00	NaN
7846	TUBA3	0.00	0.00	-0.03	0.03
7849	PAX8	0.00	0.00	0.00	NaN
7850	IL1R2	0.02	0.00	0.00	0.07
7852	CXCR4	0.00	0.00	0.00	0.29
7855	FZD5	0.00	0.00	0.00	NaN
7857	SCG2	0.00	0.00	0.00	NaN

7862	BRPF1	-0.02	0.00	0.00	0.07
7866	IFRD2	0.00	0.00	0.00	NaN
7867	MAPKAPK3	0.00	0.00	0.00	NaN
7869	SEMA3B	0.00	0.00	0.00	NaN
7873	ARMET	0.00	0.00	0.00	NaN
7874	USP7	0.04	0.00	0.00	0.23
7879	RAB7	0.00	0.00	-0.03	0.58
7881	KCNAB1	0.02	0.00	0.03	0.24
7884	SLBP	-0.02	0.00	0.00	0.23
7913	DEK	0.00	0.00	0.08	0.19
7915	ALDH5A1	0.00	0.00	0.03	0.16
7916	BAT2	0.00	0.00	0.05	0.09
7917	BAT3	0.00	0.00	0.05	0.20
7919	BAT1	0.00	0.00	0.05	0.13
7936	RDBP	0.00	0.00	0.05	0.14
7941	PLA2G7	0.00	0.00	0.05	0.06
7957	EPM2A	0.02	0.00	0.00	0.14
7965	JTV1	0.00	0.00	0.00	NaN
7975	MAFK	0.11	0.00	0.24	0.08
7976	FZD3	-0.13	0.00	-0.19	0.40
7978	MTERF	0.02	0.00	0.05	0.24
7980	TFPI2	0.02	0.00	0.00	0.01
7982	ST7	0.00	0.00	-0.03	0.10
7984	ARHGEF5	0.00	0.00	-0.05	0.09
7988	ZNF212	0.02	0.00	0.00	0.20
8000	PSCA	0.16	0.09	0.38	-0.02
8001	GLRA3	-0.02	0.00	-0.05	0.12
8013	NR4A3	-0.02	0.00	0.00	0.14
8019	BRD3	0.02	0.00	0.00	0.10
8021	NUP214	0.02	0.00	0.03	0.12
8022	LHX3	0.02	0.00	0.03	-0.11
8027	STAM	-0.02	0.00	0.08	0.17
8028	MLLT10	-0.02	0.00	0.03	0.31
8029	CUBN	-0.02	0.00	0.08	0.09
8031	NCOA4	-0.02	0.00	0.00	0.00
8034	SLC25A16	-0.02	0.00	-0.03	0.03
8036	SHOC2	-0.04	0.00	0.00	0.22
8038	ADAM12	-0.04	0.00	0.00	0.01
8048	CSRP3	0.00	0.00	-0.05	0.01
8050	PDX1	-0.05	0.09	-0.08	-0.18
8061	FOSL1	0.02	0.00	0.03	0.00
8065	CUL5	-0.07	-0.09	0.00	0.12
8073	PTP4A2	0.02	0.09	0.00	0.21
8074	FGF23	-0.02	0.09	0.08	-0.24
8078	USP5	-0.02	0.09	0.05	0.07
8079	MLF2	-0.02	0.09	0.05	0.30
8082	SSPN	0.00	-0.09	0.08	0.35

8085	MLL2	0.00	0.00	0.00	NaN
8087	FXR1	0.04	0.00	0.00	0.36
8092	CART1	0.00	0.00	0.00	NaN
8099	CDK2AP1	0.02	0.00	0.00	0.05
8106	PABPN1	0.00	0.00	0.00	0.14
8111	GPR68	-0.02	0.00	0.03	0.03
8115	TCL1A	-0.04	0.00	-0.03	0.12
8120	AP3B2	0.00	0.00	-0.03	0.00
8125	ANP32A	0.00	0.00	-0.05	0.02
8139	GAN	-0.09	0.00	0.00	0.10
8140	SLC7A5	-0.07	0.00	0.00	0.18
8148	TAF15	-0.02	0.00	0.03	-0.02
8161	COIL	0.15	0.00	0.03	0.47
8165	AKAP1	0.15	0.09	0.03	0.41
8170	SLC14A2	0.00	-0.09	-0.03	0.13
8175	SF3A2	0.00	0.00	-0.03	0.21
8178	ELL	0.02	0.00	0.00	0.13
8187	ZNF239	0.00	0.09	-0.03	-0.02
8190	MIA	0.00	0.00	0.00	0.20
8192	CLPP	0.00	-0.09	0.00	0.09
8195	MKKS	0.00	0.00	-0.05	0.15
8200	GDF5	0.05	0.00	0.00	-0.02
8202	NCOA3	0.04	0.00	0.03	0.32
8204	NRIP1	-0.05	-0.18	0.05	0.14
8208	CHAF1B	0.00	0.00	0.00	-0.03
8209	C21orf33	0.00	0.00	0.00	0.40
8214	DGCR6	-0.02	0.00	0.03	0.01
8216	LZTR1	-0.02	0.00	0.03	0.07
8218	CLTCL1	-0.02	0.00	0.03	0.06
8224	SYN3	-0.02	0.00	0.03	0.22
8237	USP11	-0.05	0.00	-0.08	0.18
8239	USP9X	-0.05	0.00	-0.11	0.32
8241	RBM10	-0.05	0.00	-0.08	0.08
8269	CXorf12	0.00	0.00	-0.03	0.29
8277	TKTL1	0.00	0.00	-0.03	-0.02
8287	USP9Y	0.02	0.00	-0.03	0.10
8288	EPX	0.15	0.00	0.03	-0.08
8291	DYSF	0.02	0.00	0.00	-0.01
8292	COLQ	-0.02	0.00	0.00	0.18
8295	TRRAP	0.02	0.00	0.00	0.16
8301	PICALM	-0.02	0.00	0.00	0.25
8302	KLRC4	0.00	-0.18	0.08	-0.09
8303	SNN	0.04	0.00	0.00	0.10
8309	ACOX2	-0.04	0.00	0.00	0.03
8310	ACOX3	-0.02	0.00	0.00	0.01
8312	AXIN1	0.05	0.18	0.05	0.27
8314	BAP1	0.00	0.00	0.00	NaN

8315	BRAP	0.00	0.00	0.00	NaN
8318	CDC45L	-0.02	0.00	0.05	-0.02
8321	FZD1	0.02	0.00	0.05	0.38
8322	FZD4	-0.02	0.00	0.00	0.30
8323	FZD6	0.22	0.09	0.32	0.34
8324	FZD7	0.00	0.00	0.00	NaN
8328	GFI1B	0.02	0.00	0.00	0.06
8372	HYAL3	0.00	0.00	0.00	NaN
8379	MAD1L1	0.04	0.09	0.19	0.12
8382	NME5	0.00	0.00	-0.03	0.08
8387	OR1E1	-0.05	0.00	0.00	-0.04
8388	OR1E2	-0.05	0.00	0.00	-0.01
8390	OR1G1	-0.05	0.00	0.00	0.12
8394	PIP5K1A	0.07	0.09	0.19	0.17
8395	PIP5K1B	-0.02	0.00	-0.03	0.09
8396	PIP5K2B	0.00	0.45	0.05	0.66
8398	PLA2G6	-0.04	0.00	0.03	0.30
8399	PLA2G10	0.02	0.00	0.00	0.08
8402	SLC25A11	-0.04	0.00	0.00	0.18
8403	SOX14	0.02	0.00	0.00	0.09
8404	SPARCL1	-0.04	0.00	-0.03	-0.01
8405	SPOP	0.11	0.09	0.03	0.43
8406	SRPX	-0.05	0.00	-0.11	-0.02
8408	ULK1	0.00	-0.09	0.00	-0.11
8409	UXT	-0.05	0.00	-0.08	0.27
8411	EEA1	0.00	0.09	0.00	0.08
8412	BCAR3	-0.02	0.00	0.00	0.05
8416	ANXA9	0.07	0.09	0.16	-0.06
8417	STX7	-0.02	0.00	0.08	0.36
8419	BFSP2	0.00	0.00	0.00	NaN
8424	BBOX1	0.00	0.00	-0.03	0.02
8425	LTBP4	0.00	0.09	0.11	0.22
8427	ZNF282	0.02	0.00	0.00	0.02
8428	STK24	-0.02	0.09	-0.05	0.27
8431	NR0B2	-0.04	0.00	0.00	-0.05
8433	UTF1	0.00	0.00	0.03	0.12
8434	RECK	0.00	0.00	0.00	0.19
8435	SOAT2	0.02	0.00	-0.03	0.10
8436	SDPR	0.00	0.00	0.00	NaN
8437	RASAL1	0.02	0.00	0.00	0.05
8438	RAD54L	0.00	0.00	0.03	0.17
8439	NSMAF	0.15	0.00	0.14	0.35
8440	NCK2	0.00	0.00	0.00	NaN
8443	GNPAT	0.09	0.00	0.03	-0.01
8444	DYRK3	0.07	0.00	0.00	0.12
8445	DYRK2	0.02	0.00	0.00	0.20
8446	DUSP11	0.00	0.00	0.00	NaN

8450	CUL4B	-0.05	0.00	-0.08	0.13
8451	CUL4A	0.00	0.00	0.00	0.31
8452	CUL3	0.00	0.00	0.00	NaN
8453	CUL2	-0.02	0.00	0.03	0.19
8454	CUL1	0.02	0.00	0.00	0.03
8455	ATRN	0.02	0.00	-0.03	0.11
8458	TTF2	0.02	0.00	-0.03	0.07
8459	TPST2	-0.05	-0.09	0.03	0.03
8460	TPST1	0.00	0.00	0.05	-0.17
8464	SUPT3H	0.00	0.00	0.05	0.14
8467	SMARCA5	0.00	0.00	0.00	NaN
8468	FKBP6	0.02	0.00	0.03	-0.18
8473	OGT	-0.04	0.00	-0.08	0.27
8477	GPR65	-0.04	0.00	0.00	0.25
8480	RAE1	0.05	0.00	0.05	0.58
8481	OFD1	-0.05	-0.09	-0.14	0.28
8482	SEMA7A	0.00	0.00	0.00	NaN
8483	CILP	0.00	0.09	-0.05	-0.01
8487	SIP1	0.00	0.00	-0.03	0.26
8490	RGS5	0.05	0.00	0.14	0.18
8491	MAP4K3	0.00	0.00	0.00	NaN
8492	PRSS12	0.00	0.00	-0.03	0.05
8493	PPM1D	0.20	0.18	0.03	0.60
8495	PPFIBP2	-0.02	0.00	-0.05	0.14
8496	PPFIBP1	-0.02	-0.09	0.03	0.19
8498	RANBP3	0.00	-0.09	0.00	0.12
8499	PPFIA2	0.00	0.00	0.00	NaN
8500	PPFIA1	0.18	0.09	0.03	0.41
8502	PKP4	-0.02	0.00	0.03	0.16
8503	PIK3R3	0.00	0.00	0.03	0.02
8504	PEX3	0.00	0.00	0.08	0.56
8505	PARG	-0.02	0.00	-0.03	0.04
8506	CNTNAP1	-0.04	0.00	0.00	0.26
8507	ENC1	0.00	0.09	-0.14	0.22
8508	NIPSNAP1	0.00	0.00	0.03	0.28
8509	NDST2	0.04	0.00	0.00	0.30
8513	LIPF	-0.04	0.00	-0.11	0.02
8514	KCNAB2	-0.02	0.00	0.00	0.21
8515	ITGA10	0.05	0.00	0.08	-0.01
8516	ITGA8	-0.02	0.00	0.11	0.11
8517	IKBKG	-0.04	0.00	-0.08	0.18
8518	IKBKAP	-0.02	0.00	0.00	0.08
8519	IFITM1	0.00	0.09	0.00	0.06
8520	HAT1	-0.02	0.00	0.00	0.00
8522	GAS7	-0.07	0.00	0.00	0.14
8525	DGKZ	-0.02	0.00	-0.03	0.43
8526	DGKE	0.15	0.00	0.03	0.10

8527	DGKD	0.00	0.00	0.00	NaN
8528	DDO	0.00	0.00	0.00	0.10
8529	CYP4F2	0.00	0.00	0.00	NaN
8530	CST7	0.02	0.00	-0.03	0.00
8531	CSDA	0.00	-0.18	0.08	0.27
8532	CPZ	-0.02	0.00	0.00	-0.07
8533	COPS3	-0.07	0.00	0.00	0.08
8534	CHST1	-0.02	0.00	-0.03	-0.08
8535	CBX4	0.04	0.00	0.00	0.19
8536	CAMK1	-0.02	0.00	0.00	0.15
8537	BCAS1	0.07	0.09	0.05	0.15
8538	BARX2	-0.05	0.00	-0.03	0.09
8539	API5	0.00	0.00	-0.08	0.24
8540	AGPS	0.02	0.00	0.00	-0.10
8541	PPFIA3	0.00	0.00	-0.03	-0.07
8542	APOL1	-0.02	0.00	0.03	-0.11
8543	LMO4	-0.02	0.00	-0.03	0.10
8544	PIR	-0.05	0.00	-0.14	-0.04
8545	CGGBP1	-0.05	0.00	0.03	0.29
8546	AP3B1	0.00	0.09	-0.14	0.33
8547	FCN3	-0.02	0.00	0.00	-0.09
8548	BLZF1	0.02	0.00	0.05	0.11
8550	MAPKAPK5	0.00	0.00	0.00	NaN
8553	BHLHB2	-0.02	0.00	0.00	-0.02
8554	PIAS1	0.00	0.00	-0.05	0.20
8555	CDC14B	-0.02	0.00	0.00	0.39
8556	CDC14A	-0.02	0.00	-0.03	0.00
8557	TCAP	0.02	0.91	0.05	0.23
8558	CDK10	-0.04	0.00	0.00	0.04
8562	DENR	0.02	0.00	0.00	0.28
8564	KMO	0.07	0.00	0.05	0.19
8565	YARS	0.00	0.00	0.00	NaN
8566	PDXK	0.00	0.00	0.00	0.19
8567	MADD	-0.02	0.00	-0.03	0.17
8568	D21S2056E	0.00	0.00	0.00	0.07
8569	MKNK1	0.00	0.00	0.00	0.00
8570	KHSRP	0.00	-0.09	0.00	0.08
8573	CASK	-0.05	0.00	-0.11	0.03
8574	AKR7A2	0.00	0.00	0.00	NaN
8575	PRKRA	0.00	0.00	0.00	NaN
8576	STK16	0.00	0.00	0.00	NaN
8577	TMEFF1	-0.02	0.00	0.00	0.06
8600	TNFSF11	-0.05	0.00	-0.05	0.11
8601	RGS20	0.13	0.00	0.14	0.00
8604	SLC25A12	-0.02	0.00	0.00	0.26
8605	PLA2G4C	0.00	0.00	-0.03	-0.06
8607	RUVBL1	0.00	0.00	-0.03	0.35

8609	KLF7	0.00	0.00	0.00	NaN
8611	PPAP2A	0.00	0.00	-0.11	0.15
8612	PPAP2C	0.00	0.00	-0.03	-0.05
8613	PPAP2B	0.00	0.00	0.00	0.26
8614	STC2	0.00	0.00	-0.03	0.08
8618	CADPS	-0.05	0.00	0.00	0.06
8620	NPFF	0.02	0.09	-0.03	0.13
8621	CDC2L5	-0.02	0.00	0.03	0.25
8624	DSCR2	0.00	0.00	0.00	0.14
8625	RFXANK	0.02	0.00	0.00	0.15
8629	JRK	0.16	0.09	0.38	0.30
8633	UNC5C	-0.04	0.00	0.00	-0.01
8634	RTCD1	-0.02	0.00	-0.03	0.07
8636	SSNA1	0.02	0.00	0.03	0.03
8638	OASL	0.00	0.00	0.00	NaN
8639	AOC3	-0.04	0.00	0.00	0.30
8643	PTCH2	0.00	0.00	0.03	0.18
8644	AKR1C3	0.00	0.00	0.08	0.11
8645	KCNK5	0.02	0.00	0.05	0.19
8646	CHRD	0.04	0.00	0.05	0.07
8647	ABCB11	0.00	0.00	0.03	-0.08
8648	NCOA1	-0.02	0.00	0.00	0.26
8649	MAP2K1IP1	-0.02	0.00	0.03	0.10
8650	NUMB	-0.02	0.00	-0.03	0.30
8654	PDE5A	0.00	0.00	-0.03	-0.03
8658	TNKS	-0.18	-0.18	-0.19	0.22
8660	IRS2	0.00	0.00	-0.03	0.41
8661	EIF3S10	-0.04	0.00	0.00	0.10
8662	EIF3S9	0.00	0.00	0.05	0.33
8664	EIF3S7	-0.04	0.00	0.03	-0.02
8665	EIF3S5	0.00	0.00	-0.08	0.00
8666	EIF3S4	0.00	0.00	0.00	NaN
8667	EIF3S3	0.22	0.18	0.35	0.39
8668	EIF3S2	0.02	0.00	0.00	0.21
8669	EIF3S1	-0.04	0.00	-0.14	0.26
8671	SLC4A4	-0.02	0.00	0.00	0.03
8672	EIF4G3	0.00	0.00	0.00	NaN
8673	VAMP8	0.02	0.00	0.00	0.05
8674	VAMP4	0.00	0.00	0.05	0.20
8676	STX11	0.02	0.00	0.00	0.05
8677	STX10	0.00	-0.09	0.00	0.19
8678	BECN1	-0.04	0.00	0.00	0.00
8681	PLA2G4B	-0.04	0.09	-0.14	0.13
8682	PEA15	0.05	0.00	0.14	0.28
8683	SFRS9	0.02	0.00	0.00	0.13
8685	MARCO	0.00	0.00	0.00	NaN
8690	JRKL	-0.09	-0.09	0.00	0.13

8692	HYAL2	0.00	0.00	0.00	NaN
8697	CDC23	0.00	0.00	-0.03	0.16
8702	B4GALT4	0.00	0.00	-0.03	-0.03
8703	B4GALT3	0.11	0.00	0.14	0.49
8704	B4GALT2	0.00	0.00	0.03	0.22
8705	B3GALT4	0.00	0.00	0.05	0.07
8708	B3GALT1	0.00	0.00	0.03	-0.18
8710	SERPINB7	-0.02	-0.09	0.00	0.11
8711	TNK1	-0.05	0.00	0.00	-0.05
8714	ABCC3	0.15	0.18	0.03	-0.04
8715	NOL4	0.02	-0.09	-0.03	-0.03
8717	TRADD	-0.04	0.00	0.00	0.06
8720	MBTPS1	-0.09	0.00	0.00	0.00
8721	EDF1	0.02	0.00	0.03	0.11
8722	CTSF	0.05	0.00	0.03	0.17
8723	SNX4	0.00	0.00	-0.03	0.33
8724	SNX3	0.04	0.00	0.00	0.41
8726	EED	-0.02	0.09	0.00	0.24
8727	CTNNAL1	-0.02	0.00	0.00	0.12
8728	ADAM19	0.00	0.00	0.00	NaN
8729	GBF1	0.00	0.00	-0.03	0.18
8731	RNMT	-0.02	-0.09	0.00	0.10
8732	RNGTT	-0.02	0.00	-0.05	0.25
8733	GPAA1	0.16	0.00	0.35	0.52
8735	MYH13	-0.07	0.00	0.00	-0.04
8736	MYOM1	0.00	-0.09	0.00	0.01
8737	RIPK1	-0.02	0.00	0.00	0.04
8738	CRADD	0.00	0.09	0.00	0.15
8739	HRK	0.02	0.00	-0.03	0.06
8740	TNFSF14	0.00	-0.09	0.00	0.08
8742	TNFSF12	-0.05	0.00	0.00	-0.03
8743	TNFSF10	0.04	0.00	0.00	0.10
8744	TNFSF9	0.00	-0.09	0.00	0.04
8745	ADAM23	0.00	0.00	0.00	NaN
8747	ADAM21	-0.02	0.00	0.00	0.06
8748	ADAM20	-0.02	0.00	0.00	0.03
8749	ADAM18	0.04	0.09	0.05	0.01
8754	ADAM9	0.11	0.09	0.11	0.24
8756	ADAM7	-0.13	-0.18	-0.19	0.20
8760	CDS2	0.02	0.00	-0.05	0.25
8761	PABPC4	-0.02	0.09	0.00	0.14
8763	CD164	0.00	0.00	0.00	0.35
8764	TNFRSF14	-0.02	0.00	0.03	0.07
8766	RAB11A	0.00	0.09	-0.05	0.21
8767	RIPK2	0.15	0.00	0.16	0.25
8772	FADD	0.18	0.09	0.03	0.45
8773	SNAP23	-0.05	0.09	-0.14	0.34

8774	NAPG	-0.02	-0.09	0.00	0.02
8775	NAPA	-0.04	0.00	-0.03	0.44
8776	MTMR1	-0.04	0.00	-0.11	0.19
8777	MPDZ	-0.05	0.00	-0.03	0.23
8778	SIGLEC5	0.00	-0.09	-0.03	0.00
8785	MATN4	0.04	0.09	0.03	0.08
8786	RGS11	0.05	0.18	0.05	0.21
8787	RGS9	0.15	0.27	0.03	-0.02
8788	DLK1	0.02	0.00	0.03	-0.17
8789	FBP2	-0.02	0.00	0.00	0.03
8790	FPGT	-0.02	0.00	-0.03	0.11
8792	TNFRSF11A	-0.02	-0.09	0.03	0.06
8793	TNFRSF10D	-0.13	-0.18	-0.22	-0.13
8794	TNFRSF10C	-0.13	-0.18	-0.22	0.13
8795	TNFRSF10B	-0.13	-0.18	-0.22	0.24
8796	SCEL	-0.04	-0.09	-0.14	-0.04
8798	DYRK4	-0.02	0.09	0.08	0.22
8799	PEX11B	0.05	0.00	0.08	0.38
8800	PEX11A	0.02	0.00	-0.05	0.05
8801	SUCLG2	-0.05	0.00	0.00	0.25
8802	SUCLG1	-0.02	0.00	0.00	0.06
8803	SUCLA2	-0.07	0.00	-0.14	0.16
8807	IL18RAP	0.00	0.00	0.00	NaN
8808	IL1RL2	0.02	0.00	0.00	-0.05
8809	IL18R1	0.00	0.00	0.00	NaN
8811	GALR2	0.04	0.18	0.03	0.06
8813	DPM1	0.07	0.09	0.05	0.36
8814	CDKL1	0.02	0.00	0.00	0.03
8817	FGF18	0.00	0.00	-0.03	0.08
8818	DPM2	0.02	0.00	0.00	0.22
8819	SAP30	-0.02	0.00	-0.05	0.10
8820	HESX1	-0.05	0.00	-0.03	0.07
8821	INPP4B	0.00	0.00	0.00	NaN
8822	FGF17	-0.15	-0.09	-0.16	-0.05
8824	CES2	-0.05	0.00	0.00	0.17
8826	IQGAP1	0.04	0.00	0.00	0.33
8828	NRP2	0.00	0.00	0.00	NaN
8829	NRP1	-0.02	0.00	0.03	0.18
8832	CD84	0.07	0.00	0.14	-0.07
8833	GMPS	0.02	0.00	0.03	0.28
8836	GGH	0.13	0.00	0.14	0.37
8837	CFLAR	0.00	0.00	0.00	NaN
8838	WISP3	0.02	0.00	0.03	-0.04
8839	WISP2	0.02	0.09	0.03	0.00
8840	WISP1	0.20	0.09	0.35	0.12
8850	PCAF	-0.05	0.00	-0.05	0.10
8851	CDK5R1	0.00	0.27	0.00	0.26

8852	AKAP4	-0.04	0.00	-0.08	0.06
8853	DDEF2	-0.02	0.00	0.00	-0.01
8854	ALDH1A2	-0.02	0.00	-0.05	0.00
8856	NR1I2	0.00	0.00	-0.03	-0.16
8857	FCGBP	0.02	0.09	0.05	0.22
8858	PROZ	0.00	0.00	0.00	0.21
8861	LDB1	0.00	0.00	-0.03	0.11
8863	PER3	-0.02	0.00	0.00	0.07
8864	PER2	0.00	0.00	0.00	NaN
8867	SYNJ1	0.00	0.00	-0.03	0.23
8870	IER3	0.02	0.00	0.05	0.06
8871	SYNJ2	-0.02	0.00	-0.03	0.23
8874	ARHGEF7	0.00	0.00	-0.03	0.32
8875	VNN2	-0.02	0.00	0.11	-0.04
8876	VNN1	-0.02	0.00	0.11	-0.02
8877	SPHK1	0.04	0.27	0.03	-0.03
8878	SQSTM1	0.02	0.00	-0.03	0.24
8879	SGPL1	-0.02	0.00	-0.03	0.15
8880	FUBP1	-0.02	0.00	-0.03	-0.04
8881	CDC16	0.00	0.00	0.00	0.33
8882	ZNF259	-0.09	0.00	0.00	0.10
8883	APPBP1	-0.09	0.00	0.00	0.08
8884	SLC5A6	0.00	0.00	0.00	NaN
8886	DDX18	0.00	0.00	0.00	NaN
8887	TAX1BP1	-0.02	0.18	0.00	0.43
8888	MCM3AP	0.00	0.09	0.00	0.21
8891	EIF2B3	0.00	0.00	0.03	0.39
8892	EIF2B2	-0.02	0.00	0.00	0.25
8893	EIF2B5	0.05	0.00	0.00	0.25
8894	EIF2S2	0.00	0.00	0.00	NaN
8895	CPNE3	0.15	0.00	0.14	0.35
8897	MTMR3	0.00	0.00	0.03	0.26
8898	MTMR2	-0.09	-0.09	0.00	0.30
8900	CCNA1	-0.05	0.00	-0.11	0.03
8904	CPNE1	0.04	0.00	0.00	0.41
8905	AP1S2	-0.05	0.00	-0.11	0.15
8906	AP1G2	-0.02	0.00	0.00	-0.01
8908	GYG2	-0.05	-0.09	-0.08	0.26
8909	P11	0.00	0.00	0.00	NaN
8910	SGCE	0.02	0.00	-0.03	0.05
8911	CACNA1I	-0.04	0.00	-0.03	-0.02
8912	CACNA1H	0.04	0.18	0.03	0.11
8913	CACNA1G	0.15	0.18	0.03	0.06
8914	TIMELESS	0.00	0.00	0.00	NaN
8915	BCL10	-0.02	0.00	-0.03	-0.08
8916	HERC3	-0.04	0.00	-0.03	0.03
8924	HERC2	-0.04	0.09	-0.08	0.29

8925	HERC1	0.00	0.09	-0.08	0.19
8927	BSN	0.00	0.00	0.00	NaN
8930	MBD4	0.00	0.00	-0.03	0.61
8932	MBD2	-0.02	-0.18	-0.03	0.28
8934	RAB7L1	0.07	0.00	0.03	0.25
8936	WASF1	0.00	0.00	-0.03	0.08
8938	BAIAP3	0.04	0.18	0.03	0.21
8939	FUBP3	0.02	0.00	0.00	0.09
8940	TOP3B	-0.02	0.00	0.03	0.10
8941	CDK5R2	0.00	0.00	0.00	NaN
8942	KYNU	0.00	0.00	0.00	NaN
8943	AP3D1	0.00	0.00	-0.03	0.10
8945	BTRC	-0.02	0.00	-0.03	0.08
8971	H1FX	0.00	0.00	-0.03	0.50
8972	MGAM	0.02	0.00	0.00	0.13
8973	CHRNA6	0.02	0.09	0.11	0.08
8974	P4HA2	0.00	0.00	-0.03	0.00
8975	USP13	0.04	0.00	0.03	0.38
8976	WASL	0.00	0.00	-0.03	0.05
8985	PLOD3	0.02	0.00	0.00	0.24
8986	RPS6KA4	0.00	0.00	0.03	0.11
8987	GENX-3414	-0.02	0.00	0.00	-0.04
8988	HSPB3	0.02	0.00	-0.08	-0.02
8991	SELENBP1	0.07	0.09	0.19	-0.05
8994	LIMD1	-0.04	0.00	0.00	0.07
8995	TNFSF18	0.02	0.00	0.05	-0.01
8996	NOL3	-0.04	0.00	0.00	-0.04
8999	CDKL2	-0.02	0.00	0.00	0.04
9001	HAP1	0.00	0.18	0.00	0.03
9013	TAF1C	-0.09	0.00	0.00	0.11
9014	TAF1B	-0.02	0.00	0.00	0.03
9015	TAF1A	0.07	0.00	0.00	0.15
9016	SLC25A14	-0.05	0.00	-0.08	0.15
9019	MPZL1	0.02	0.00	0.05	0.16
9020	MAP3K14	-0.05	0.00	0.00	0.30
9023	CH25H	-0.04	0.00	-0.11	0.00
9025	RNF8	0.02	0.00	0.08	0.11
9027	NAT8	0.00	0.00	0.00	NaN
9031	BAZ1B	0.02	0.00	0.05	0.22
9032	TM4SF5	-0.04	0.00	0.00	0.18
9033	PKD2L1	-0.02	0.00	-0.03	0.14
9037	SEMA5A	0.02	0.18	0.00	-0.16
9039	UBE1C	-0.05	0.00	0.00	0.16
9040	UBE2M	0.00	0.09	-0.03	0.43
9043	SPAG9	0.15	0.09	0.03	0.47
9044	BTAF1	-0.02	0.00	-0.05	0.13
9045	RPL14	-0.04	0.00	0.00	0.08

9046	DOK2	-0.13	-0.18	-0.22	0.07
9047	SH2D2A	0.04	0.00	0.11	0.07
9048	ARTN	0.00	0.00	0.03	-0.05
9049	AIP	0.07	0.09	0.03	0.25
9050	PSTPIP2	0.00	-0.09	-0.03	0.03
9051	PSTPIP1	0.00	0.00	-0.08	0.01
9053	MAP7	-0.02	0.00	0.05	0.33
9054	NFS1	0.04	0.00	0.00	0.37
9055	PRC1	0.02	0.00	0.03	0.09
9056	SLC7A7	-0.02	0.00	0.00	0.05
9057	SLC7A6	-0.05	0.00	0.00	0.14
9058	SLC13A2	0.04	0.09	0.00	0.04
9060	PAPSS2	-0.04	0.00	-0.11	0.29
9061	PAPSS1	-0.02	0.00	0.00	0.04
9064	MAP3K6	-0.02	0.00	0.00	-0.07
9070	ASH2L	0.16	0.09	0.11	0.69
9071	CLDN10	-0.02	0.00	-0.05	0.02
9074	CLDN6	0.04	0.00	0.00	-0.05
9076	CLDN1	0.02	0.00	0.00	0.03
9079	LDB2	-0.04	-0.09	-0.05	-0.03
9086	EIF1AY	0.02	0.00	0.00	0.31
9087	TMSB4Y	0.02	0.00	-0.03	-0.07
9088	PKMYT1	0.04	0.00	0.00	-0.03
9090	PSIP1	-0.05	0.00	-0.03	0.15
9091	PIGQ	0.04	0.18	0.05	0.07
9092	SART1	0.02	0.00	0.03	0.18
9093	DNAJA3	0.04	0.00	0.00	0.13
9094	UNC119	0.04	0.09	0.00	0.15
9095	TBX19	0.02	0.00	0.05	0.14
9097	USP14	0.02	-0.09	0.00	0.19
9098	USP6	-0.02	0.00	0.00	0.00
9099	USP2	-0.07	0.18	0.05	-0.01
9100	USP10	-0.09	0.00	0.00	0.24
9101	USP8	-0.02	0.00	-0.05	0.28
9104	RGN	-0.05	0.00	-0.08	0.12
9107	MTMR6	-0.05	0.00	-0.08	0.18
9108	MTMR7	-0.16	-0.18	-0.22	-0.02
9110	MTMR4	0.16	0.00	0.03	0.60
9111	NMI	-0.02	0.00	0.03	0.32
9112	MTA1	-0.02	0.00	0.08	0.11
9113	LATS1	0.00	0.00	-0.03	-0.07
9114	ATP6V0D1	-0.04	0.00	0.00	0.07
9120	SLC16A6	0.13	0.18	0.03	0.30
9121	SLC16A5	0.05	0.27	0.05	0.19
9122	SLC16A4	0.00	0.00	-0.03	0.16
9123	SLC16A3	-0.02	0.09	0.08	0.15
9124	PDLIM1	-0.02	0.00	-0.05	0.07

9125	RQCD1	0.00	0.00	0.00	NaN
9127	P2RXL1	-0.02	0.00	0.03	0.28
9128	PRPF4	-0.02	0.00	0.00	-0.04
9132	KCNQ4	0.00	0.00	0.00	NaN
9133	CCNB2	-0.02	0.00	-0.05	0.05
9134	CCNE2	0.20	0.09	0.24	0.41
9138	ARHGEF1	0.02	0.00	-0.03	0.08
9139	CBFA2T2	0.00	0.00	0.00	NaN
9141	PDCD5	0.00	0.00	0.05	0.00
9142	CXorf1	-0.05	0.00	-0.08	0.13
9143	SYNGR3	0.04	0.18	0.00	0.09
9144	SYNGR2	0.05	0.09	0.03	0.25
9145	SYNGR1	-0.04	0.00	0.00	0.10
9146	HGS	0.00	0.09	0.08	0.16
9147	SDCCAG1	0.02	0.00	0.00	0.34
9148	NEURL	0.00	0.00	0.00	0.16
9149	DYRK1B	0.02	0.09	0.08	0.07
9150	CTDP1	-0.02	-0.09	0.00	-0.04
9152	SLC6A5	0.00	0.00	-0.05	-0.10
9153	SLC28A2	-0.04	0.00	-0.11	0.10
9154	SLC28A1	0.02	0.00	-0.03	0.00
9156	EXO1	0.07	0.00	0.05	-0.02
9158	FIBP	0.02	0.00	0.03	0.16
9159	PCSK7	-0.07	0.00	0.00	0.03
9162	DGKI	0.02	0.00	0.03	-0.06
9166	EBAG9	0.18	0.09	0.27	0.31
9167	COX7A2L	0.00	0.00	0.00	NaN
9168	TMSB10	0.00	0.00	0.00	NaN
9169	SFRS2IP	0.00	0.00	0.00	NaN
9170	EDG4	0.02	0.00	0.00	0.24
9172	MYOM2	-0.18	-0.18	-0.19	0.05
9173	IL1RL1	0.00	0.00	0.00	NaN
9175	MAP3K13	0.04	0.00	0.00	0.21
9177	HTR3B	-0.07	0.00	0.00	0.19
9179	AP4M1	0.02	0.00	0.00	0.01
9180	OSMR	0.04	0.09	0.00	0.07
9181	ARHGEF2	0.09	0.00	0.14	0.08
9182	PAMCI	0.00	0.00	0.00	NaN
9183	ZW10	-0.07	0.00	0.00	0.15
9184	BUB3	-0.04	0.00	0.03	0.09
9185	REPS2	-0.05	0.00	-0.11	0.20
9187	SLC24A1	0.02	0.09	-0.05	0.32
9188	DDX21	-0.04	0.00	0.00	0.14
9191	DEDD	0.11	0.00	0.14	0.38
9194	SLC16A7	0.00	0.00	0.00	NaN
9196	KCNAB3	-0.05	0.00	0.00	0.05
9200	PTPLA	-0.02	0.00	0.08	0.00

9201	DCAMKL1	-0.05	0.00	-0.11	0.12
9208	LRRFIP1	0.04	0.00	0.00	0.42
9209	LRRFIP2	0.02	0.00	0.00	0.46
9210	BMP15	-0.04	0.00	-0.08	-0.06
9211	LGI1	-0.02	0.00	-0.05	-0.05
9215	LARGE	-0.02	0.00	0.03	0.15
9217	VAPB	0.07	0.09	0.05	0.43
9218	VAPA	-0.02	-0.09	0.00	0.19
9221	NOLC1	0.00	0.00	-0.03	0.22
9227	LRAT	-0.02	0.00	-0.03	0.03
9228	DLGAP2	-0.18	-0.18	-0.16	0.04
9229	DLGAP1	0.00	-0.09	0.00	0.13
9230	RAB11B	0.00	-0.09	0.00	-0.03
9231	DLG5	-0.02	0.00	0.03	0.16
9232	PTTG1	0.00	0.00	-0.03	-0.03
9240	PNMA1	-0.02	0.00	0.00	0.10
9242	MSC	0.15	0.00	0.08	-0.03
9244	CRLF1	0.02	0.00	0.00	0.06
9245	GCNT3	-0.02	0.00	-0.05	0.16
9246	UBE2L6	0.02	0.00	0.05	0.08
9248	GPR50	-0.04	0.00	-0.11	0.04
9252	RPS6KA5	-0.02	0.00	0.03	0.18
9254	CACNA2D2	0.00	0.00	0.00	NaN
9255	SCYE1	-0.02	0.00	0.00	0.00
9258	MFHAS1	-0.16	-0.18	-0.19	0.05
9261	MAPKAPK2	0.09	0.00	0.00	0.16
9262	STK17B	0.02	0.00	0.00	-0.17
9263	STK17A	-0.02	0.00	0.05	0.07
9265	PSCD3	0.00	0.00	0.03	-0.08
9266	PSCD2	0.00	0.00	-0.03	0.43
9267	PSCD1	0.02	0.18	0.03	0.27
9271	PIWIL1	0.00	0.00	0.00	NaN
9274	BCL7C	0.07	0.09	0.00	0.26
9275	BCL7B	0.02	0.00	0.05	0.19
9276	COPB2	0.02	0.00	0.00	0.27
9282	CRSP2	-0.05	0.00	-0.11	0.24
9289	GPR56	-0.05	0.00	0.03	0.19
9294	EDG5	0.00	0.00	0.00	NaN
9295	SFRS11	-0.02	0.00	-0.03	0.01
9296	ATP6V1F	0.02	0.00	-0.03	0.08
9308	CD83	0.00	0.00	0.05	0.11
9311	ACCN3	0.02	0.00	0.00	0.14
9312	KCNB2	0.13	0.09	0.11	0.08
9313	MMP20	-0.07	-0.09	0.00	-0.05
9314	KLF4	-0.02	0.00	0.00	0.01
9317	PTER	-0.02	0.00	0.08	0.11
9319	TRIP13	0.02	0.27	0.00	0.25

9320	TRIP12	0.00	0.00	0.00	NaN
9321	TRIP11	-0.02	0.00	0.03	0.33
9322	TRIP10	0.00	-0.09	0.00	0.20
9325	TRIP4	0.00	0.09	-0.08	0.17
9328	GTF3C5	0.02	0.00	0.00	-0.05
9329	GTF3C4	0.02	0.00	0.00	0.10
9330	GTF3C3	0.00	0.00	0.00	NaN
9331	B4GALT6	0.02	-0.09	0.00	0.11
9332	CD163	0.00	0.09	0.05	0.06
9333	TGM5	-0.04	0.00	-0.14	-0.15
9334	B4GALT5	0.05	0.00	0.05	0.26
9337	CNOT8	0.00	0.00	0.00	NaN
9338	TCEAL1	-0.05	0.00	-0.08	-0.09
9340	GLP2R	-0.07	0.00	0.00	-0.07
9341	VAMP3	-0.02	0.00	0.00	0.06
9342	SNAP29	-0.02	0.00	0.03	-0.37
9348	NDST3	-0.02	0.00	-0.03	-0.07
9349	RPL23	0.00	0.45	0.05	0.34
9350	CER1	-0.04	0.00	-0.03	0.02
9351	SLC9A3R2	0.04	0.18	0.00	0.20
9353	SLIT2	-0.05	-0.09	-0.05	0.00
9354	UBE4A	-0.05	0.00	0.00	0.01
9355	LHX2	-0.02	0.00	0.00	0.09
9356	SLC22A6	0.00	0.00	0.03	0.08
9358	ITGBL1	-0.02	0.00	-0.05	0.08
9360	PPIG	0.00	0.00	0.03	0.22
9362	CPNE6	-0.02	0.00	0.00	0.16
9363	RAB33A	-0.05	0.00	-0.08	0.07
9364	RAB28	-0.04	0.00	-0.05	0.02
9365	KL	-0.05	0.00	-0.08	-0.03
9368	SLC9A3R1	0.05	0.27	0.03	0.49
9369	NRXN3	-0.02	0.00	0.00	-0.19
9371	KIF3B	0.00	0.00	0.00	NaN
9373	PLAA	-0.11	0.00	-0.05	0.31
9374	PPT2	-0.02	0.00	0.05	0.02
9375	TM9SF2	-0.02	0.00	-0.05	0.15
9376	SLC22A8	0.00	0.00	0.03	-0.04
9377	COX5A	0.00	0.00	0.00	NaN
9378	NRXN1	0.02	0.00	0.00	-0.05
9379	NRXN2	0.02	-0.09	0.03	0.00
9380	GRHPR	-0.04	0.00	0.03	0.24
9381	OTOF	-0.02	0.00	0.00	-0.04
9388	LIPG	-0.02	-0.09	-0.05	0.04
9391	CIAO1	0.00	0.00	0.00	NaN
9397	NMT2	-0.02	0.00	0.11	0.27
9398	IGSF2	0.02	0.00	-0.03	-0.02
9399	STOML1	0.00	0.00	0.00	NaN

9400	RECQL5	0.05	0.27	0.03	0.30
9401	RECQL4	0.15	0.00	0.30	0.26
9402	GRAP2	-0.04	0.00	-0.03	-0.01
9409	PEX16	-0.02	0.00	-0.03	0.13
9412	SURB7	0.00	-0.09	0.05	0.27
9414	TJP2	-0.02	0.00	-0.03	0.04
9415	FADS2	-0.02	0.00	0.05	0.03
9419	CRIPT	0.00	0.00	0.00	NaN
9420	CYP7B1	0.13	0.00	0.14	0.17
9421	HAND1	0.00	0.00	0.00	NaN
9422	ZNF264	0.00	0.09	0.00	0.05
9423	NTN1	-0.07	0.00	0.00	-0.02
9425	CDYL	-0.02	0.00	0.05	0.16
9427	ECEL1	0.02	0.00	0.00	-0.16
9429	ABCG2	-0.04	0.00	-0.03	0.18
9435	CHST2	0.02	0.00	0.03	-0.02
9439	CRSP3	0.00	0.00	0.05	0.07
9440	CRSP6	-0.07	0.09	-0.03	0.20
9442	CRSP8	0.02	0.00	0.03	0.26
9443	CRSP9	0.00	0.00	0.00	NaN
9444	QKI	-0.02	0.00	-0.08	0.25
9445	ITM2B	-0.07	0.00	-0.14	0.19
9447	AIM2	0.04	0.00	0.16	0.08
9448	MAP4K4	0.02	0.00	0.00	0.07
9451	EIF2AK3	0.00	0.00	0.00	NaN
9452	ITM2A	-0.04	0.00	-0.08	0.14
9453	GGPS1	0.09	0.00	0.03	0.35
9459	ARHGEF6	-0.02	0.00	-0.08	0.05
9462	RASAL2	0.04	0.00	0.03	0.15
9464	HAND2	-0.02	0.00	-0.05	0.06
9465	AKAP7	0.00	0.00	0.08	0.23
9467	SH3BP5	-0.02	0.00	0.00	0.37
9468	PCYT1B	-0.05	0.00	-0.11	0.04
9469	CHST3	-0.02	0.00	-0.03	-0.07
9472	AKAP6	-0.02	0.09	0.00	-0.03
9475	ROCK2	-0.02	0.00	0.00	0.07
9477	TRFP	0.00	0.00	0.08	0.21
9478	CABP1	0.02	0.00	0.00	0.32
9479	MAPK8IP1	-0.02	0.00	-0.03	0.09
9480	ONECUT2	-0.02	0.00	-0.03	0.05
9482	STX8	-0.07	0.00	0.00	0.15
9487	PIGL	-0.07	0.00	-0.03	0.15
9488	PIGB	-0.02	0.00	-0.05	0.32
9489	PGS1	0.02	0.09	0.03	0.19
9491	PSMF1	0.02	0.00	-0.03	0.14
9495	AKAP5	-0.02	0.00	0.00	0.07
9496	TBX4	0.16	0.18	0.03	-0.08

9497	SLC4A7	0.00	0.00	-0.05	0.26
9498	SLC4A8	0.02	0.00	-0.03	0.21
9500	MAGED1	-0.04	0.00	-0.08	0.00
9501	RPH3AL	-0.09	0.00	0.00	-0.07
9508	ADAMTS3	-0.02	0.00	0.00	-0.01
9509	ADAMTS2	0.00	0.00	-0.03	0.01
9512	PMPCB	0.02	0.00	0.00	0.03
9513	FXR2	-0.05	0.00	0.00	0.00
9517	SPTLC2	-0.02	0.00	-0.03	0.24
9519	TBPL1	-0.02	0.00	0.08	0.19
9520	NPEPPS	0.00	0.00	0.00	0.19
9521	EEF1E1	-0.02	0.00	0.05	0.21
9522	SCAMP1	0.00	0.09	-0.14	0.32
9524	GPSN2	0.02	-0.09	0.00	0.11
9526	MPDU1	-0.05	0.00	0.00	-0.06
9527	GOSR1	0.02	0.09	0.00	0.19
9529	BAG5	0.00	0.00	0.03	0.01
9530	BAG4	0.16	0.09	0.14	0.41
9531	BAG3	-0.04	0.00	-0.03	0.04
9532	BAG2	-0.04	0.00	0.08	0.14
9534	ZNF254	0.00	0.00	0.00	NaN
9535	GMFG	0.02	0.00	0.08	0.14
9536	PTGES	0.02	0.00	0.00	-0.03
9541	CIR	0.00	0.00	0.00	0.05
9542	NRG2	0.00	0.00	0.00	NaN
9545	RAB3D	0.00	0.00	0.00	NaN
9546	APBA3	0.00	0.00	0.00	NaN
9550	ATP6V1G1	-0.02	0.00	0.00	0.00
9551	ATP5J2	0.02	0.00	0.00	0.12
9552	SPAG7	-0.04	0.00	0.00	0.24
9553	MRPL33	0.00	0.00	0.00	NaN
9555	H2AFY	0.00	0.00	-0.03	0.12
9556	C14orf2	0.00	0.00	0.03	0.04
9557	CHD1L	0.07	0.00	0.14	0.23
9562	MINPP1	-0.04	0.00	-0.05	0.22
9563	H6PD	-0.02	0.00	0.00	-0.06
9567	GTPBP1	-0.04	0.00	-0.03	0.03
9570	GOSR2	-0.04	0.00	0.00	0.09
9572	NR1D1	0.04	0.45	0.03	0.30
9573	GDF3	0.00	-0.09	0.05	0.09
9575	CLOCK	-0.02	0.00	0.00	0.09
9576	SPAG6	-0.02	0.00	0.05	0.04
9577	BRE	0.00	0.00	0.00	NaN
9578	CDC42BPB	0.00	0.00	-0.03	0.31
9580	SOX13	0.07	0.00	0.05	0.21
9585	MPHOSPH1	-0.04	0.00	-0.11	0.19
9589	WTAP	-0.02	0.00	-0.05	0.45

9590	AKAP12	0.00	0.00	0.00	0.04
9595	PSCDBP	-0.02	0.00	0.03	0.12
9603	NFE2L3	0.00	0.18	0.03	0.06
9604	RNF14	0.00	0.00	0.00	NaN
9605	C16orf7	-0.04	0.00	0.00	0.08
9609	RAB36	-0.02	0.00	0.03	0.19
9610	RIN1	0.07	0.00	0.03	0.00
9611	NCOR1	-0.07	0.00	-0.03	0.14
9612	NCOR2	0.02	0.09	0.03	0.14
9616	RNF7	0.02	0.00	0.03	0.32
9617	MTRF1	-0.05	0.00	-0.05	0.16
9618	TRAF4	0.02	0.09	0.00	0.24
9619	ABCG1	0.00	0.00	0.00	0.17
9620	CELSR1	-0.02	0.00	-0.03	0.07
9623	TCL1B	-0.04	0.00	-0.03	0.00
9627	SNCAIP	0.00	0.00	-0.03	0.01
9628	RGS6	-0.02	0.00	0.00	0.14
9629	CLCA3	-0.02	0.00	-0.03	-0.05
9630	GNA14	-0.02	0.00	0.00	-0.01
9631	NUP155	0.04	0.27	0.00	0.40
9632	SEC24C	0.04	0.09	0.00	0.35
9633	MTL5	0.09	0.09	0.03	0.40
9635	CLCA2	-0.02	0.00	-0.03	0.12
9637	FEZ2	0.00	0.00	0.00	NaN
9638	FEZ1	-0.05	0.00	-0.03	0.23
9639	ARHGEF10	-0.18	-0.18	-0.16	0.13
9652	KIAA0372	0.00	0.00	-0.14	0.15
9653	HS2ST1	-0.02	0.00	-0.03	0.12
9655	SOCS5	0.00	0.00	0.00	NaN
9659	PDE4DIP	0.04	0.00	0.00	0.20
9663	LPIN2	0.00	-0.09	0.00	0.02
9672	SDC3	-0.02	0.09	0.00	0.23
9674	KIAA0040	0.02	0.00	0.03	0.14
9675	KIAA0406	0.02	0.00	0.03	0.22
9683	N4BP1	-0.09	0.09	-0.03	0.09
9692	KIAA0391	0.02	0.09	0.00	0.44
9698	PUM1	0.00	0.09	0.00	0.22
9700	ESPL1	0.02	0.00	-0.03	0.23
9703	KIAA0100	0.04	0.09	0.00	0.44
9706	ULK2	-0.04	0.09	0.03	0.28
9709	HERPUD1	-0.05	0.00	0.00	0.06
9710	KIAA0355	0.00	0.00	0.05	0.35
9715	KIAA0773	0.02	0.00	0.00	0.14
9718	ECE2	0.05	0.00	0.05	-0.01
9723	SEMA3E	0.02	0.00	0.00	0.08
9729	KIAA0408	-0.02	0.00	0.00	0.02
9731	KIAA0562	-0.02	0.00	0.00	0.12

9733	SART3	0.00	0.00	0.00	NaN
9735	KNTC1	0.02	0.00	0.00	0.17
9741	LAPTM4A	-0.02	0.00	0.00	0.02
9744	CENTB1	-0.05	0.00	0.00	0.07
9746	CLSTN3	0.00	0.09	0.05	0.09
9747	KIAA0738	0.02	0.00	0.00	0.11
9748	SLK	-0.02	0.00	0.00	0.15
9750	C6orf32	0.00	0.00	0.03	-0.07
9751	SNPH	0.02	0.00	-0.03	0.07
9757	MLL4	0.00	0.09	0.03	0.15
9759	HDAC4	0.00	0.00	0.00	NaN
9760	TOX	0.15	0.00	0.14	-0.01
9761	KIAA0152	0.00	0.00	0.00	NaN
9764	KIAA0513	-0.09	0.00	0.00	0.11
9766	KIAA0247	-0.02	0.00	0.00	0.08
9768	KIAA0101	0.00	0.09	-0.08	0.07
9770	RASSF2	0.02	0.00	-0.03	-0.12
9772	KIAA0195	0.05	0.27	0.03	0.18
9776	KIAA0652	-0.02	0.00	-0.03	0.04
9778	KIAA0232	-0.02	0.00	0.00	0.09
9782	MATR3	0.00	0.00	0.00	NaN
9784	SNX17	0.00	0.00	0.00	NaN
9786	KIAA0586	0.02	0.00	-0.03	0.15
9791	PTDSS1	0.18	0.18	0.27	0.32
9794	MAML1	0.02	0.00	-0.03	0.14
9796	PHYHIP	-0.13	-0.18	-0.22	-0.03
9798	KIAA0174	-0.07	0.00	0.03	0.13
9801	MRPL19	0.00	0.00	0.00	NaN
9802	DAZAP2	0.02	0.00	-0.03	0.15
9807	IHPK1	0.00	0.00	0.00	NaN
9810	RNF40	0.07	0.09	0.00	0.27
9811	KIAA0427	-0.02	-0.09	-0.03	0.21
9812	KIAA0141	0.00	0.00	0.00	NaN
9813	KIAA0494	0.00	0.00	0.00	-0.02
9815	GIT2	0.00	0.00	0.00	NaN
9816	KIAA0133	0.07	0.00	0.03	0.10
9817	KEAP1	0.02	0.00	0.00	0.20
9821	RB1CC1	0.13	0.00	0.14	0.34
9824	ARHGAP11A	0.02	0.00	-0.16	-0.10
9825	SPATA2	0.05	0.00	0.05	0.31
9826	ARHGEF11	0.04	0.00	0.11	0.23
9829	DNAJC6	-0.02	0.00	0.00	0.14
9830	TRIM14	-0.02	0.00	0.00	-0.03
9833	MELK	-0.02	0.00	0.03	0.00
9839	ZFHX1B	-0.02	0.00	0.00	-0.02
9843	HEPH	-0.04	0.00	-0.05	-0.09
9844	ELMO1	-0.02	0.00	-0.03	0.09

9846	GAB2	0.11	0.09	0.03	0.30
9847	KIAA0528	0.00	-0.09	0.05	0.17
9851	KIAA0753	-0.05	0.00	0.00	0.04
9856	KIAA0319	0.00	0.00	0.03	0.10
9858	KIAA0649	0.02	0.00	0.03	0.10
9865	KIAA0644	-0.02	0.18	0.00	0.30
9868	TOMM70A	0.00	0.00	0.00	0.22
9869	SETDB1	0.07	0.09	0.16	0.27
9870	KIAA0317	-0.02	0.00	0.00	0.09
9871	SEC24D	-0.02	0.00	-0.03	0.25
9874	TLK1	0.00	0.00	0.03	0.16
9878	KIAA0737	-0.02	0.00	0.00	0.07
9885	OSBPL2	0.05	0.09	0.14	0.19
9892	SNAP91	-0.02	0.00	-0.05	-0.23
9895	KIAA0329	0.00	0.00	-0.03	0.19
9897	KIAA0196	0.20	0.09	0.38	0.29
9899	SV2B	0.02	0.00	0.03	-0.08
9901	SRGAP3	0.00	0.00	0.00	0.05
9908	G3BP2	-0.02	0.00	0.00	0.22
9912	KIAA0672	-0.07	0.00	-0.03	0.08
9915	ARNT2	0.00	0.00	-0.03	0.21
9921	RNF10	0.02	0.00	0.00	0.22
9927	MFN2	-0.02	0.00	0.00	0.15
9931	HELZ	0.13	0.27	0.03	0.35
9933	KIAA0020	-0.05	0.00	-0.08	0.34
9935	MAFB	0.04	0.00	0.00	0.11
9937	DCLRE1A	-0.04	0.00	0.00	0.11
9939	RBM8A	0.05	0.00	0.08	0.26
9940	DLEC1	0.00	0.00	0.00	0.08
9941	ENDOGL1	-0.04	0.00	0.00	-0.08
9942	XYLB	-0.04	0.00	0.00	0.14
9943	OSR1	-0.02	0.00	0.00	-0.06
9945	GFPT2	0.00	0.00	-0.03	-0.03
9946	CRYZL1	0.00	0.00	0.00	0.03
9947	MAGEC1	-0.05	0.00	-0.08	-0.14
9948	WDR1	-0.04	0.00	-0.05	0.24
9950	GOLGA5	-0.02	0.00	0.03	0.41
9953	HS3ST3B1	-0.05	0.00	-0.03	0.04
9955	HS3ST3A1	-0.05	0.00	-0.03	0.11
9956	HS3ST2	0.02	-0.09	0.00	-0.01
9957	HS3ST1	-0.04	0.00	-0.05	0.15
9958	USP15	0.00	0.00	0.00	NaN
9962	SLC23A1	0.00	0.00	-0.03	-0.04
9966	TNFSF15	-0.02	0.00	0.00	-0.08
9970	NR1I3	0.11	0.00	0.14	0.06
9971	NR1H4	0.00	0.00	0.03	0.06
9972	NUP153	0.00	0.00	0.08	0.19

9973	CCS	0.07	0.00	0.03	0.22
9978	RBX1	-0.02	0.00	-0.03	0.16
9986	RCE1	0.07	0.09	0.03	0.24
9987	HNRPDL	-0.04	0.00	-0.03	0.12
9988	DMTF1	0.02	0.00	0.00	0.16
9989	PPP4R1	-0.02	-0.09	0.00	0.07
9990	SLC12A6	-0.02	0.00	-0.16	-0.02
9991	ROD1	-0.02	0.00	0.00	0.25
9992	KCNE2	0.00	0.00	0.00	0.09
9993	DGCR2	-0.02	0.00	0.03	0.03
9994	CASP8AP2	-0.04	0.00	0.00	0.29
10000	AKT3	0.09	0.00	0.05	0.12
10001	MED6	-0.02	0.00	0.00	0.11
10002	NR2E3	0.00	0.00	-0.05	0.12
10003	NAALAD2	-0.11	0.00	0.03	-0.07
10010	TANK	0.00	0.00	0.03	0.29
10013	HDAC6	-0.04	0.00	-0.05	-0.09
10014	HDAC5	-0.02	0.00	0.00	0.16
10015	PDCD6IP	0.02	0.00	-0.03	0.39
10016	PDCD6	0.02	0.18	0.00	0.40
10017	BCL2L10	-0.02	0.00	-0.05	0.09
10018	BCL2L11	0.00	0.00	0.00	NaN
10020	GNE	0.00	0.00	0.00	0.16
10021	HCN4	0.00	0.00	0.00	NaN
10022	INSL5	-0.02	0.00	0.00	-0.01
10023	FRAT1	-0.02	0.00	-0.03	0.04
10024	TROAP	0.00	0.00	-0.03	0.00
10026	PIGK	-0.02	0.00	-0.03	0.08
10036	CHAF1A	0.00	0.00	0.00	NaN
10040	TOM1L1	0.07	0.27	0.03	0.49
10042	HMG2L1	-0.02	0.00	0.03	0.07
10043	TOM1	-0.02	0.00	0.03	-0.09
10045	SH2D3A	0.00	-0.09	0.00	0.02
10046	CXorf6	-0.04	0.00	-0.08	-0.10
10047	CST8	0.02	0.00	-0.03	0.04
10048	RANBP9	0.00	0.00	0.08	0.16
10049	DNAJB6	0.02	0.00	-0.03	0.05
10050	SLC17A4	0.00	0.00	0.03	-0.04
10052	GJA7	-0.05	0.00	0.00	0.08
10053	AP1M2	0.02	0.00	0.00	0.17
10057	ABCC5	0.04	0.00	0.00	0.04
10058	ABCB6	0.00	0.00	0.00	NaN
10059	DNM1L	0.00	0.00	0.05	0.12
10060	ABCC9	0.00	-0.09	0.05	0.02
10061	ABCF2	0.02	0.00	-0.03	0.17
10062	NR1H3	-0.02	0.00	-0.03	0.01
10063	COX17	0.00	0.00	-0.03	0.44

10066	SCAMP2	0.00	0.00	0.00	NaN
10067	SCAMP3	0.09	0.00	0.14	0.28
10069	C21orf6	-0.02	0.00	0.05	0.17
10072	DPP3	0.05	0.00	0.03	0.26
10076	PTPRU	-0.02	0.09	0.00	0.03
10078	TSSC4	0.00	0.00	0.00	NaN
10079	ATP9A	0.09	0.00	0.05	0.39
10084	PQBP1	-0.04	0.00	-0.05	0.21
10087	COL4A3BP	0.00	0.09	-0.14	0.26
10089	KCNK7	0.02	0.00	0.05	0.01
10090	UST	0.00	0.00	0.00	0.25
10092	ARPC5	0.07	0.00	0.03	0.14
10093	ARPC4	-0.02	0.00	0.00	0.12
10094	ARPC3	0.00	0.00	0.00	NaN
10095	ARPC1B	0.02	0.00	0.00	0.13
10096	ACTR3	0.00	0.00	0.00	NaN
10097	ACTR2	0.00	0.00	0.00	NaN
10101	NUBP2	0.04	0.18	0.00	0.22
10102	TSFM	0.02	0.09	0.00	0.18
10105	PPIF	0.00	0.00	0.05	0.33
10109	ARPC2	0.02	0.00	0.00	0.28
10110	SGK2	0.02	0.09	0.03	0.08
10111	RAD50	0.00	0.00	-0.03	0.01
10113	PREB	0.00	0.00	0.00	NaN
10114	HIPK3	0.04	0.00	-0.05	0.11
10116	FEM1B	0.00	0.00	-0.05	0.16
10120	ACTR1B	0.00	0.00	0.00	NaN
10121	ACTR1A	0.00	0.00	-0.03	0.25
10125	RASGRP1	-0.02	0.00	-0.19	0.12
10126	DNAL4	-0.04	0.00	-0.03	0.31
10127	ZNF263	0.04	0.00	0.00	0.31
10128	LRPPRC	0.00	0.00	0.00	NaN
10131	TRAP1	0.04	0.00	0.00	-0.03
10133	OPTN	0.02	0.00	0.14	0.30
10136	ELA3A	0.00	0.00	0.00	NaN
10138	YAF2	0.00	0.09	0.03	0.14
10140	TOB1	0.13	0.09	0.03	0.45
10141	C4orf6	-0.02	0.00	0.00	-0.14
10142	AKAP9	0.02	0.00	0.05	0.33
10148	EBI3	0.00	0.00	0.00	NaN
10149	GPR64	-0.05	-0.09	-0.11	-0.02
10151	HNRPA3	0.02	0.00	0.00	0.21
10154	PLXNC1	0.00	0.09	0.00	0.07
10155	TRIM28	0.00	0.18	-0.03	0.40
10157	AASS	0.00	0.00	-0.03	-0.10
10160	FARP1	-0.02	0.09	-0.05	0.22
10164	CHST4	-0.07	0.00	0.03	0.03

10165	SLC25A13	0.02	0.00	-0.03	0.21
10166	SLC25A15	-0.02	0.00	-0.03	0.11
10168	ZNF197	-0.04	0.00	0.00	0.03
10179	RBM7	-0.09	0.00	0.00	0.15
10180	RBM6	-0.02	0.00	0.00	0.33
10181	RBM5	-0.02	0.00	0.03	0.29
10184	LHFPL2	0.00	0.09	-0.14	0.04
10186	LHFP	-0.05	0.00	-0.08	0.22
10196	PRMT3	0.00	0.00	-0.05	-0.03
10197	PSME3	-0.04	0.00	0.00	0.16
10198	MPHOSPH9	0.02	0.00	0.00	0.16
10199	MPHOSPH10	0.02	0.00	0.00	0.10
10200	MPHOSPH6	-0.09	0.00	0.00	0.27
10202	DHRS2	-0.02	0.00	0.00	0.10
10203	CALCRL	0.02	0.00	0.00	0.20
10205	EVA1	-0.05	0.00	0.00	0.08
10207	INADL	0.00	0.00	0.00	0.16
10211	FLOT1	0.02	0.00	0.05	-0.03
10212	DDX39	0.02	-0.09	0.00	0.36
10215	OLIG2	0.00	0.00	-0.03	0.05
10216	PRG4	0.04	0.09	0.03	0.04
10219	KLRG1	0.00	-0.18	0.05	0.08
10220	GDF11	0.02	0.00	0.00	0.22
10223	GPA33	0.00	0.00	0.05	-0.06
10227	TETRAN	-0.02	0.00	0.00	0.14
10228	STX6	0.04	0.00	0.03	0.12
10229	COQ7	0.02	0.09	0.00	0.23
10231	DSCR1L1	0.00	0.00	0.05	0.08
10232	MSLN	0.07	0.18	0.05	-0.03
10235	RASGRP2	0.00	-0.09	0.03	0.11
10236	HNRPR	0.02	0.00	0.00	0.15
10239	AP3S2	0.02	0.00	-0.03	0.27
10240	MRPS31	-0.02	0.00	-0.05	0.30
10242	KCNMB2	0.04	0.00	0.03	0.01
10243	GPHN	-0.02	0.00	0.00	-0.04
10245	TIMM17B	-0.04	0.00	-0.05	0.05
10246	SLC17A2	0.00	0.00	0.03	0.14
10249	GLYAT	-0.04	0.00	0.00	0.02
10250	SRRM1	0.00	0.00	0.00	NaN
10253	SPRY2	-0.04	-0.09	-0.14	0.06
10254	STAM2	-0.02	0.00	0.03	0.12
10257	ABCC4	-0.02	0.00	-0.05	0.07
10262	SF3B4	0.07	0.00	0.14	0.48
10265	IRX5	-0.07	0.00	0.00	-0.02
10266	RAMP2	-0.04	0.00	0.00	0.07
10267	RAMP1	0.04	0.00	0.00	0.05
10268	RAMP3	-0.04	0.00	0.05	-0.07

10269	ZMPSTE24	-0.02	0.00	0.00	-0.08
10270	AKAP8	0.02	0.00	0.00	0.08
10272	FSTL3	0.00	0.00	-0.03	0.06
10273	STUB1	0.04	0.18	0.05	0.23
10274	STAG1	0.00	0.00	-0.03	0.14
10276	NET1	0.00	0.00	0.11	0.12
10277	UBE4B	-0.02	0.00	0.00	0.21
10279	PRSS16	0.00	-0.09	0.05	0.10
10281	DSCR4	0.00	0.00	0.00	0.05
10282	BET1	0.02	0.00	0.00	0.39
10284	SAP18	-0.07	0.00	-0.11	0.27
10286	BCAS2	0.00	0.00	-0.03	-0.01
10287	RGS19	0.07	0.09	0.08	0.33
10288	LILRB2	0.00	0.09	-0.03	-0.04
10291	SF3A1	0.00	0.00	0.05	0.28
10294	DNAJA2	-0.11	0.09	0.03	0.18
10295	BCKDK	0.04	0.09	0.00	0.27
10296	MAEA	-0.02	0.00	0.00	0.12
10298	PAK4	0.02	0.09	0.05	0.25
10300	KATNB1	-0.05	0.00	0.03	0.40
10302	SNAPC5	0.00	0.09	-0.05	0.10
10308	ZNF267	0.02	0.09	0.00	0.09
10309	UNG2	0.00	0.00	-0.11	0.11
10311	DSCR3	0.00	0.00	0.00	0.12
10312	TCIRG1	0.07	0.09	0.03	0.24
10313	RTN3	0.02	0.00	0.03	0.23
10314	LANCL1	0.00	0.00	0.00	NaN
10317	B3GALT5	0.00	0.00	-0.03	0.01
10319	LAMC3	0.02	0.00	0.03	0.04
10326	SIRPB1	0.02	0.00	-0.03	-0.08
10327	AKR1A1	0.00	0.00	0.00	NaN
10329	TMEM5	0.00	0.00	0.00	NaN
10330	TMEM4	0.00	0.00	0.00	NaN
10331	B3GNT3	0.02	0.00	0.00	-0.14
10333	TLR6	-0.05	0.00	-0.03	0.07
10342	TFG	0.00	0.00	0.00	0.33
10343	PKDREJ	-0.02	0.00	-0.03	-0.10
10346	TRIM22	-0.02	0.00	-0.03	0.06
10347	ABCA7	0.00	0.00	-0.03	0.06
10351	ABCA8	0.07	0.18	0.03	-0.06
10352	WARS2	0.00	0.00	0.00	0.16
10360	NPM3	0.00	0.00	-0.03	0.16
10362	HMG20B	0.00	0.00	0.00	NaN
10363	HMG20A	0.00	0.00	-0.08	0.01
10365	KLF2	0.00	0.00	0.00	NaN
10367	CBARA1	-0.02	0.00	0.00	0.20
10368	CACNG3	0.02	0.00	0.00	0.05

10369	CACNG2	-0.04	0.00	0.03	-0.05
10370	CITED2	0.00	0.00	0.03	0.03
10371	SEMA3A	0.02	0.00	0.00	0.09
10376	K-ALPHA-1	0.00	0.00	-0.03	0.02
10379	ISGF3G	-0.02	0.00	0.00	-0.01
10380	BPNT1	0.07	0.00	0.00	-0.02
10381	TUBB4	0.00	-0.09	0.00	-0.05
10384	BTN3A3	0.00	0.00	0.03	0.24
10385	BTN2A2	0.00	0.00	0.03	0.11
10388	SYCP2	0.05	0.09	0.00	0.11
10389	SCML2	-0.05	-0.09	-0.11	-0.06
10390	CEPT1	0.00	0.00	-0.05	0.09
10391	CORO2B	0.00	0.00	-0.05	0.06
10394	PRG3	0.00	0.00	0.05	-0.11
10395	DLC1	-0.16	-0.18	-0.22	0.13
10396	ATP8A1	-0.04	0.00	-0.11	-0.02
10397	NDRG1	0.20	0.27	0.32	0.24
10398	MYL9	0.02	0.00	0.00	0.08
10399	GNB2L1	0.00	0.00	-0.03	0.12
10400	PEMT	-0.07	0.00	0.00	0.00
10401	PIAS3	0.05	0.00	0.11	0.08
10404	PGCP	0.16	0.18	0.24	0.12
10406	WFDC2	0.04	0.00	0.03	0.08
10409	BASP1	0.04	0.18	0.00	-0.07
10413	YAP1	-0.05	-0.09	0.00	0.07
10417	SPON2	0.02	0.00	0.00	0.07
10418	SPON1	-0.02	0.00	-0.05	0.11
10420	TESK2	0.00	0.00	0.00	NaN
10421	CD2BP2	0.05	0.00	0.00	0.24
10424	PGRMC2	0.00	0.00	-0.03	0.05
10425	ARIH2	-0.04	0.00	0.00	0.25
10427	SEC24B	-0.02	0.00	0.00	0.05
10428	CFDP1	-0.09	0.00	0.03	0.21
10431	TIMM23	-0.02	0.00	0.00	0.16
10432	RBM14	0.07	0.00	0.03	0.07
10434	LYPLA1	0.11	0.00	0.14	0.01
10437	IFI30	0.02	0.00	0.00	-0.09
10438	C1D	0.02	0.00	0.00	0.04
10439	OLFM1	0.02	0.00	0.03	0.03
10440	TIMM17A	0.11	0.00	0.05	0.24
10445	MCRS1	0.00	0.00	-0.03	0.15
10449	ACAA2	-0.02	-0.09	-0.05	0.03
10450	PPIE	-0.02	0.09	0.00	0.24
10451	VAV3	-0.02	0.00	-0.03	0.06
10452	TOMM40	-0.02	0.00	-0.03	-0.09
10454	MAP3K7IP1	-0.04	0.00	0.00	0.15
10455	PECI	-0.02	0.00	0.05	0.06

10456	HAX1	0.05	0.00	0.14	0.35
10457	GPNMB	-0.02	0.09	0.03	0.15
10458	BAIAP2	0.07	0.18	0.19	0.08
10460	TACC3	-0.02	0.00	0.00	0.12
10461	MERTK	0.00	0.00	0.00	NaN
10465	PPIH	0.02	0.00	0.00	0.14
10466	COG5	0.02	0.00	-0.03	0.19
10468	FST	0.00	0.00	-0.08	0.04
10469	TIMM44	0.00	-0.09	0.00	0.15
10472	ZNF238	0.09	0.00	0.05	0.13
10474	TADA3L	-0.02	0.00	0.00	0.03
10477	UBE2E3	0.00	0.00	0.00	NaN
10478	SLC25A17	-0.02	0.00	-0.03	0.01
10479	SLC9A6	-0.04	0.00	-0.08	0.02
10481	HOXB13	0.05	0.18	0.00	0.05
10482	NXF1	0.00	0.00	0.03	0.10
10483	SEC23B	0.00	0.00	-0.08	0.34
10484	SEC23A	0.00	0.00	-0.03	0.31
10486	CAP2	0.00	0.00	0.05	0.00
10488	CREB3	0.00	0.00	0.00	-0.17
10491	CRTAP	0.02	0.00	-0.03	0.23
10494	STK25	0.00	0.00	0.00	NaN
10495	COVA1	-0.05	0.00	-0.08	0.10
10498	CARM1	0.02	0.00	0.00	0.25
10499	NCOA2	0.15	0.00	0.14	0.34
10500	SEMA6C	0.07	0.09	0.19	0.08
10501	SEMA6B	0.00	0.00	0.00	NaN
10505	SEMA4F	0.00	0.00	0.00	NaN
10507	SEMA4D	-0.02	0.00	0.00	0.02
10512	SEMA3C	0.02	0.00	0.03	0.21
10513	APPBP2	0.20	0.18	0.03	0.64
10514	MYBBP1A	0.04	0.00	0.03	0.03
10516	FBLN5	-0.02	0.00	0.03	0.20
10519	CIB1	0.02	0.00	-0.03	0.40
10520	ZNF211	0.00	0.18	0.00	0.29
10521	DDX17	-0.04	0.00	-0.03	0.24
10522	DEAF1	0.00	0.00	0.00	NaN
10523	CHERP	0.00	0.00	0.00	NaN
10524	HTATIP	0.02	0.00	0.05	0.27
10528	NOL5A	0.02	0.00	-0.03	0.15
10529	NEBL	-0.02	0.00	0.05	0.02
10534	SSSCA1	0.05	0.09	0.14	0.09
10538	BATF	-0.02	0.00	0.00	0.36
10539	TXNL2	-0.04	0.00	-0.03	0.14
10540	DCTN2	0.02	0.00	0.00	0.20
10541	ANP32B	-0.02	0.00	0.00	0.17
10542	HBXIP	0.00	0.00	-0.03	0.01

10544	PROCR	0.05	0.00	0.00	0.24
10548	TM9SF1	-0.02	0.00	0.00	0.06
10549	PRDX4	-0.05	0.00	-0.11	0.14
10551	AGR2	0.00	0.00	0.00	0.05
10552	ARPC1A	0.02	0.00	0.00	0.16
10553	HTATIP2	0.00	0.00	-0.05	-0.02
10554	AGPAT1	0.00	0.00	0.05	0.13
10555	AGPAT2	0.04	0.00	0.03	0.08
10556	RPP30	-0.02	0.00	-0.05	0.15
10557	RPP38	-0.02	0.00	0.14	0.24
10558	SPTLC1	-0.02	0.00	0.00	0.09
10559	SLC35A1	-0.02	0.00	-0.05	0.29
10560	SLC19A2	0.02	0.00	0.05	0.02
10561	IFI44	-0.02	0.00	-0.03	0.03
10564	ARFGEF2	0.07	0.00	0.03	0.17
10566	AKAP3	-0.02	0.09	0.08	0.01
10567	RABAC1	0.02	0.00	-0.03	0.08
10568	SLC34A2	-0.05	-0.09	-0.05	-0.07
10570	DPYSL4	-0.04	0.00	0.00	0.17
10574	CCT7	0.00	0.00	0.00	NaN
10575	CCT4	0.00	0.00	0.00	NaN
10576	CCT2	0.04	0.09	0.05	0.48
10577	NPC2	-0.02	0.00	0.00	0.06
10578	GNLY	0.00	0.00	0.00	NaN
10579	TACC2	-0.04	0.00	0.00	0.09
10584	COLEC10	0.22	0.18	0.38	0.05
10585	POMT1	0.02	0.00	0.03	0.11
10587	TXNRD2	-0.02	0.00	0.08	0.19
10588	MTHFS	0.00	0.00	-0.03	0.01
10589	DRAP1	0.02	0.00	0.03	0.15
10590	SCGN	0.00	0.00	0.03	-0.09
10594	PRPF8	-0.05	0.00	0.00	0.06
10595	ERN2	0.02	0.00	0.00	0.07
10600	USP16	-0.02	0.00	0.05	0.22
10605	PAIP1	0.02	0.09	0.00	0.29
10606	PAICS	-0.02	0.00	0.00	0.03
10609	SC65	0.00	0.18	0.00	0.02
10612	TRIM3	-0.02	0.00	-0.03	0.20
10618	TGOLN2	0.00	0.00	0.00	NaN
10621	POLR3F	0.00	0.00	-0.08	0.17
10626	TRIM16	-0.07	0.00	-0.03	0.18
10628	TXNIP	0.05	0.00	0.08	-0.10
10629	TAF6L	0.00	0.00	0.03	0.16
10632	ATP5L	-0.05	0.00	0.00	0.13
10633	RRP22	0.00	0.00	0.03	0.03
10634	GAS2L1	0.00	0.00	0.03	0.02
10636	RGS14	0.00	0.00	0.00	NaN

10638	SPHAR	0.07	0.00	0.03	0.26
10645	CAMKK2	0.02	0.09	0.00	0.17
10647	SCGB1D2	-0.02	0.00	0.03	-0.10
10648	SCGB1D1	-0.02	0.00	0.03	0.14
10651	MTX2	0.02	0.00	0.00	0.18
10652	YKT6	-0.02	0.00	0.05	0.22
10653	SPINT2	0.02	0.09	0.08	0.25
10654	PMVK	0.11	0.00	0.14	0.36
10656	KHDRBS3	0.16	0.09	0.32	0.27
10657	KHDRBS1	0.02	0.09	0.00	0.20
10658	CUGBP1	-0.02	0.00	-0.03	0.00
10659	CUGBP2	0.02	0.00	0.14	0.10
10660	LBX1	-0.02	0.00	-0.03	0.15
10661	KLF1	0.00	-0.09	0.00	0.08
10664	CTCF	-0.04	0.00	0.00	0.06
10665	C6orf10	0.00	0.00	0.05	-0.06
10672	GNA13	0.15	0.27	0.03	0.15
10675	CSPG5	-0.04	0.00	0.00	-0.03
10677	AVIL	0.02	0.09	0.00	0.22
10678	B3GNT1	0.07	0.00	0.03	0.04
10681	GNB5	-0.02	0.00	-0.05	0.14
10682	EBP	-0.04	0.00	-0.05	0.20
10683	DLL3	0.02	0.00	0.08	0.18
10686	CLDN16	0.02	0.00	0.00	0.09
10687	PNMA2	-0.11	-0.18	-0.19	0.23
10690	FUT9	-0.02	0.00	-0.03	0.02
10691	GMEB1	-0.02	0.09	0.00	0.04
10692	RRH	-0.02	0.00	0.00	0.09
10693	CCT6B	-0.02	0.09	0.03	0.31
10694	CCT8	-0.02	0.00	0.05	0.09
10695	TNRC5	0.00	0.00	0.08	0.12
10712	C1orf2	0.09	0.00	0.14	0.28
10714	POLD3	0.05	0.09	-0.03	0.36
10716	TBR1	-0.02	0.00	0.03	-0.02
10721	POLQ	0.00	0.00	-0.03	-0.21
10723	SLC12A7	0.02	0.27	0.00	0.36
10724	MGEA5	0.00	0.00	-0.03	0.15
10725	NFAT5	-0.09	0.00	0.03	0.27
10726	NUDC	-0.04	-0.09	0.00	0.14
10730	YME1L1	-0.02	0.00	0.00	0.17
10732	TCFL5	0.04	0.09	0.08	0.18
10734	STAG3	0.02	0.00	0.00	0.06
10735	STAG2	-0.05	0.00	-0.08	0.25
10736	SIX2	0.00	0.00	0.00	NaN
10738	RFPL3	-0.02	0.00	0.03	0.27
10741	RBBP9	0.00	0.00	-0.08	0.22
10742	RAI2	-0.05	-0.09	-0.11	0.10

10743	RAI1	-0.05	0.00	0.00	-0.07
10745	PHTF1	0.00	0.00	-0.03	0.00
10747	MASP2	0.00	0.00	0.00	NaN
10748	KLRA1	0.00	-0.18	0.08	0.17
10749	KIF1C	-0.04	0.00	0.00	0.09
10750	GRAP	-0.05	0.00	0.00	0.01
10752	CHL1	-0.02	0.00	0.00	-0.04
10753	CAPN9	0.09	0.00	0.03	0.20
10761	PLAC1	-0.04	0.00	-0.08	0.01
10762	NUP50	-0.04	0.00	-0.03	0.18
10766	TOB2	-0.02	0.00	-0.03	0.08
10767	HBS1L	-0.02	0.00	0.16	0.40
10768	AHCYL1	0.00	0.00	-0.03	0.11
10775	POP4	0.00	0.09	0.05	0.21
10776	ARPP-19	-0.02	0.00	-0.05	0.34
10777	ARPP-21	0.02	0.00	-0.03	0.14
10781	ZNF266	0.00	0.00	0.00	NaN
10782	ZNF274	0.00	0.18	0.00	0.16
10785	WDR4	0.00	0.00	0.00	0.13
10786	SLC17A3	0.00	0.00	0.03	-0.15
10787	NCKAP1	0.02	0.00	-0.03	0.23
10788	IQGAP2	0.00	0.00	-0.14	0.22
10791	VAMP5	0.02	0.00	0.00	0.04
10795	ZNF268	0.00	0.00	0.00	NaN
10797	MTHFD2	0.00	0.00	0.00	NaN
10798	OR5I1	-0.07	-0.09	0.03	0.21
10799	RPP40	-0.02	0.00	0.08	0.32
10803	CCR9	-0.04	0.00	0.00	0.05
10807	SDCCAG3	0.02	0.00	0.03	0.01
10810	WASF3	-0.05	0.00	-0.08	0.04
10814	CPLX2	0.00	0.00	-0.03	0.04
10825	NEU3	0.07	0.09	0.00	-0.06
10826	C5orf4	0.00	0.00	0.00	NaN
10827	C5orf3	0.00	0.00	0.00	NaN
10841	FTCD	0.00	0.09	0.00	0.09
10845	CLPX	0.00	0.09	-0.05	0.10
10846	PDE10A	-0.02	0.00	-0.03	0.23
10847	SRCAP	0.05	0.00	0.00	0.25
10855	HPSE	-0.04	0.00	-0.03	0.07
10856	RUVBL2	0.00	0.00	-0.03	0.31
10857	PGRMC1	-0.05	0.00	-0.08	0.08
10859	LILRB1	0.00	0.09	-0.03	0.08
10861	SLC26A1	0.02	0.09	0.03	-0.05
10863	ADAM28	-0.13	-0.18	-0.19	0.13
10864	SLC22A7	0.00	0.00	0.11	0.00
10868	USP20	0.02	0.00	0.00	0.17
10873	ME3	-0.02	0.09	0.00	0.18

10874	NMU	-0.02	0.00	0.00	0.11
10880	ACTL7B	-0.02	0.00	0.00	-0.04
10881	ACTL7A	-0.02	0.00	0.00	0.24
10884	MRPS30	0.04	0.18	0.00	0.21
10885	WDR3	0.00	0.00	-0.03	-0.03
10892	MALT1	-0.02	-0.09	-0.03	0.06
10893	MMP24	0.05	0.00	0.00	0.17
10894	XLKD1	0.00	0.00	-0.05	-0.08
10898	CPSF4	0.02	0.00	0.00	0.26
10899	JTB	0.05	0.00	0.14	0.17
10900	RPIP8	-0.05	0.00	0.00	0.13
10904	BLCAP	0.02	0.09	0.03	0.12
10905	MAN1A2	0.02	0.00	-0.03	-0.03
10911	UTS2	-0.02	0.00	0.00	0.08
10912	GADD45G	-0.02	0.00	0.00	-0.07
10913	EDAR	0.00	0.00	0.00	NaN
10915	TCERG1	0.00	0.00	0.00	NaN
10916	MAGED2	-0.04	0.00	-0.05	0.10
10921	RNPS1	0.04	0.09	0.00	0.13
10922	FASTK	0.00	0.00	0.03	-0.06
10928	RALBP1	-0.02	-0.09	0.00	0.05
10930	APOBEC2	0.00	0.00	0.08	0.03
10935	PRDX3	-0.04	0.00	0.00	0.05
10936	GPR75	0.00	0.00	0.00	NaN
10938	EHD1	0.02	0.00	0.03	0.11
10939	AFG3L2	-0.02	-0.09	0.00	0.08
10941	UGT2A1	-0.02	0.00	0.00	-0.08
10942	PRSS21	0.04	0.09	0.03	-0.04
10943	MSL3L1	-0.05	-0.09	-0.14	0.08
10945	KDELR1	0.00	0.00	-0.03	0.40
10946	SF3A3	0.00	0.00	0.00	NaN
10947	AP3M2	0.04	0.09	0.11	0.46
10949	HNRPA0	0.00	0.00	-0.03	0.09
10950	BTG3	-0.04	-0.18	0.05	0.26
10951	CBX1	0.04	0.18	0.00	0.47
10952	SEC61B	-0.02	0.00	0.00	0.11
10953	TOMM34	0.02	0.09	0.03	0.18
10963	STIP1	0.00	0.00	0.03	0.13
10966	RAB40B	-0.02	0.09	0.08	0.15
10969	EBNA1BP2	0.02	0.00	0.00	0.27
10970	CKAP4	0.00	0.00	0.00	NaN
10971	YWHAQ	-0.02	0.00	0.00	0.22
10975	UQCR	0.00	0.00	-0.03	0.10
10981	RAB32	0.00	0.00	0.03	0.09
10982	MAPRE2	0.00	-0.09	-0.03	0.05
10983	CCNI	-0.02	0.00	-0.03	0.16
10987	COPS5	0.13	0.00	0.16	0.24

10988	METAP2	0.00	0.09	0.00	0.13
10989	IMMT	0.00	0.00	0.00	NaN
10990	LILRB5	0.00	0.09	-0.03	-0.10
10991	SLC38A3	0.00	0.00	0.03	0.24
10992	SF3B2	0.02	0.00	0.03	0.14
10993	SDS	0.02	0.00	0.00	-0.03
10994	ILVBL	0.02	0.00	0.00	0.25
11005	SPINK5	0.00	0.00	0.00	NaN
11006	LILRB4	0.00	0.09	-0.03	-0.04
11009	IL24	0.09	0.00	0.00	0.10
11011	TLK2	0.20	0.09	0.03	0.48
11012	KLK11	0.00	0.00	-0.03	-0.01
11014	KDELR2	0.00	0.09	0.03	-0.03
11015	KDELR3	-0.04	0.00	-0.03	-0.04
11016	ATF7	0.02	0.09	-0.03	-0.18
11017	RY1	0.02	0.00	0.00	0.18
11020	RABL4	-0.04	0.00	0.03	0.12
11021	RAB35	0.02	0.00	0.00	0.06
11022	TDRKH	0.07	0.00	0.19	0.26
11024	LILRA1	0.00	0.09	-0.03	0.06
11026	LILRA3	0.00	0.09	-0.03	-0.05
11027	LILRA2	0.00	0.09	-0.03	-0.01
11030	RBPMS	-0.09	-0.18	-0.19	0.21
11031	RAB31	-0.02	-0.09	0.00	0.23
11033	CENTA1	0.02	0.00	0.05	-0.06
11034	DSTN	0.00	0.00	-0.08	0.24
11037	SALF	0.02	0.00	0.00	0.01
11040	PIM2	-0.04	0.00	-0.05	-0.01
11041	B3GNT6	0.07	0.09	0.03	0.03
11043	MID2	-0.05	0.00	-0.08	-0.11
11044	POLS	0.02	0.18	0.00	0.33
11045	UPK1A	0.00	0.09	0.05	0.08
11047	ADRM1	0.05	0.09	0.11	0.43
11052	CPSF6	0.04	0.09	0.05	0.22
11054	OGFR	0.04	0.09	0.08	0.23
11055	ZPBP	-0.05	0.00	0.05	-0.04
11059	WWP1	0.15	0.09	0.14	0.35
11060	WWP2	-0.09	0.00	0.03	0.11
11063	SOX30	0.00	0.00	0.00	NaN
11065	UBE2C	0.04	0.00	0.03	0.18
11071	ATP9B	-0.02	-0.09	0.00	0.13
11072	DUSP14	0.00	0.09	0.05	0.26
11073	TOPBP1	0.00	0.00	0.00	NaN
11074	TRIM31	0.00	0.00	0.05	-0.02
11075	STMN2	0.11	0.09	0.14	-0.05
11077	HSF2BP	0.00	0.00	0.00	0.22
11079	RER1	-0.02	0.00	0.03	0.12

11080	DNAJB4	-0.02	0.00	-0.03	0.11
11081	KERA	0.00	0.09	0.00	0.01
11082	ESM1	0.00	0.00	-0.11	-0.10
11085	ADAM30	0.00	0.00	0.00	0.01
11086	ADAM29	-0.02	0.00	-0.05	0.11
11092	C9orf9	0.02	0.00	0.00	0.13
11095	ADAMTS8	-0.05	0.00	-0.03	0.06
11096	ADAMTS5	0.00	0.00	0.03	0.10
11099	PTPN21	-0.04	0.00	0.00	0.11
11102	RPP14	-0.04	0.00	0.00	0.02
11104	KATNA1	0.00	0.00	-0.03	0.17
11107	PRDM5	0.00	0.00	-0.03	-0.07
11108	PRDM4	0.00	0.00	0.00	NaN
11113	CIT	0.00	0.00	0.00	NaN
11118	BTN3A2	0.00	0.00	0.03	0.20
11119	BTN3A1	0.00	0.00	0.03	0.18
11120	BTN2A1	0.00	0.00	0.03	0.06
11122	PTPRT	0.02	0.00	0.03	0.11
11123	DSCR1L2	0.00	0.00	0.00	NaN
11124	FAF1	0.00	0.00	-0.03	-0.01
11127	KIF3A	0.00	0.00	-0.03	0.20
11130	ZWINT	-0.02	0.00	-0.03	0.05
11131	CAPN11	0.00	0.00	0.11	-0.13
11132	CAPN10	0.00	0.00	0.00	NaN
11133	KPTN	-0.04	0.00	-0.03	0.09
11136	SLC7A9	0.00	0.00	0.05	0.18
11137	PWP1	0.00	0.00	0.00	NaN
11140	CDC37	0.00	0.00	0.00	NaN
11141	IL1RAPL1	-0.07	0.00	-0.11	-0.10
11142	PKIG	0.02	0.09	0.03	0.29
11144	DMC1	-0.04	0.00	-0.03	-0.12
11145	HRASLS3	0.00	0.00	0.03	-0.19
11147	HHLA3	-0.02	0.00	-0.03	0.01
11148	HHLA2	0.00	0.00	-0.03	-0.19
11153	HYPE	0.00	0.00	0.00	NaN
11154	AP4S1	-0.02	0.00	0.00	0.10
11156	PTP4A3	0.16	0.00	0.41	0.33
11157	LSM6	0.02	0.00	0.00	0.15
11161	C14orf1	-0.02	0.00	0.00	0.05
11162	NUDT6	0.00	0.00	-0.03	0.10
11163	NUDT4	0.00	0.09	0.00	0.13
11165	NUDT3	0.00	0.00	0.05	0.23
11166	SOX21	-0.02	0.00	-0.05	0.10
11167	FSTL1	0.00	0.00	-0.03	0.46
11173	ADAMTS7	0.00	0.00	-0.03	-0.02
11174	ADAMTS6	0.00	0.00	-0.11	-0.07
11176	BAZ2A	0.00	0.00	0.00	NaN

11177	BAZ1A	0.02	0.09	0.00	0.54
11178	LZTS1	-0.13	-0.18	-0.22	0.19
11180	WDR6	-0.04	0.00	0.00	0.18
11181	TREH	-0.05	0.00	0.00	0.15
11183	MAP4K5	0.02	0.00	0.00	0.18
11184	MAP4K1	0.02	0.09	0.05	0.19
11186	RASSF1	0.00	0.00	0.00	NaN
11187	PKP3	0.02	0.09	0.00	-0.02
11188	NISCH	0.00	0.00	0.00	NaN
11189	TNRC4	0.07	0.00	0.19	-0.13
11193	WBP4	-0.05	0.00	-0.05	0.26
11194	ABCB8	0.02	0.00	0.00	0.01
11197	WIF1	0.00	0.00	0.00	NaN
11199	ANXA10	-0.02	0.00	-0.08	-0.10
11200	CHEK2	-0.02	-0.09	0.03	0.26
11201	POLI	-0.02	-0.18	-0.03	0.02
11202	KLK8	0.00	0.00	-0.03	-0.21
11211	FZD10	0.00	0.00	0.00	NaN
11212	PROSC	0.18	0.09	0.08	0.74
11215	AKAP11	-0.05	0.00	-0.05	0.31
11216	AKAP10	-0.04	0.09	0.03	0.53
11221	DUSP10	0.07	0.00	0.00	0.15
11222	MRPL3	0.00	0.00	0.00	NaN
11224	RPL35	-0.02	0.00	0.00	-0.01
11226	GALNT6	0.02	0.00	-0.03	0.20
11232	POLG2	0.16	0.18	0.03	0.40
11235	PDCD10	0.02	0.00	0.00	0.17
11237	RNF24	0.02	0.00	-0.03	0.20
11238	CA5B	-0.05	0.00	-0.11	-0.01
11243	PMF1	0.07	0.00	0.14	0.37
11248	NXPH3	0.11	0.18	0.03	0.04
11250	GPR45	0.00	0.00	0.00	NaN
11251	GPR44	-0.02	0.00	0.05	0.19
11252	PACSIN2	-0.02	0.00	-0.03	0.16
11253	MAN1B1	0.02	0.00	0.03	0.12
11254	SLC6A14	-0.05	0.00	-0.08	-0.15
11255	HRH3	0.04	0.09	0.14	0.12
11258	DCTN3	0.00	0.00	0.00	0.22
11260	XPOT	0.00	0.00	0.00	NaN
11261	CHP	-0.02	0.09	-0.14	0.07
11262	SP140	-0.02	0.00	0.00	0.02
11264	PXMP4	0.00	0.00	0.00	NaN
11266	DUSP12	0.07	0.00	0.11	0.35
11274	USP18	-0.02	0.00	0.00	-0.07
11275	KLHL2	0.00	0.00	-0.05	-0.01
11277	TREX1	-0.04	0.00	0.00	0.10
11278	KLF12	-0.07	-0.09	-0.14	0.15

11279	KLF8	-0.05	0.00	-0.05	-0.06
11282	MGAT4B	0.02	0.00	-0.03	0.28
11283	CYP4F8	0.00	0.00	0.00	NaN
11284	PNKP	0.00	0.00	-0.03	0.32
11285	B4GALT7	0.00	0.00	0.00	NaN
11313	LYPLA2	0.00	0.00	0.00	NaN
11316	COPE	0.00	0.00	0.00	0.21
11317	RBPSUHL	0.04	0.09	0.03	0.09
11318	ADMR	0.02	0.00	0.00	-0.05
11320	MGAT4A	0.02	0.00	0.00	0.05
11329	STK38	0.02	0.00	0.05	0.26
11330	CTRC	-0.02	0.00	0.00	0.10
11333	PDAP1	0.02	0.00	0.00	0.14
11335	CBX3	0.00	0.18	0.03	0.30
11337	GABARAP	-0.05	0.00	0.00	0.09
11339	OIP5	-0.02	0.09	-0.14	0.09
11341	SCRG1	-0.02	0.00	-0.05	-0.05
11342	RNF13	0.02	0.00	0.03	0.19
11343	MGLL	0.00	0.00	-0.03	-0.14
11345	GABARAPL2	-0.09	0.00	0.03	0.18
22797	TFEC	0.00	0.00	-0.03	0.03
22800	RRAS2	-0.02	0.00	-0.03	0.19
22802	CLCA4	-0.02	0.00	-0.03	-0.06
22808	MRAS	0.02	0.00	0.00	-0.09
22809	ATF5	0.00	0.00	-0.03	0.19
22822	PHLDA1	-0.02	0.00	0.00	0.12
22826	DNAJC8	-0.02	0.09	0.00	0.20
22827	SIAHBP1	0.16	0.00	0.35	0.53
22832	KIAA1009	-0.02	0.00	-0.05	-0.05
22856	CHSY1	-0.02	0.00	0.00	0.02
22858	ICK	-0.02	0.00	0.08	0.10
22863	KIAA0831	0.00	0.00	0.00	0.11
22871	NLGN1	0.04	0.00	0.03	-0.03
22873	DZIP1	-0.02	0.00	-0.05	0.21
22875	ENPP4	0.00	0.00	0.05	0.04
22878	KIAA1012	0.02	-0.09	0.00	0.34
22881	ANKRD6	-0.04	0.00	0.00	0.05
22883	CLSTN1	-0.02	0.00	0.00	0.11
22889	KIAA0907	0.09	0.00	0.14	0.52
22895	RPH3A	0.02	0.00	0.00	-0.03
22899	ARHGEF15	-0.05	0.00	0.00	0.21
22903	BTBD3	0.00	0.00	-0.05	0.20
22905	EPN2	-0.04	0.09	0.03	0.29
22908	SACM1L	-0.04	0.00	0.00	0.09
22913	RALY	0.00	0.00	0.00	NaN
22916	NCBP2	0.02	0.09	-0.03	0.36
22919	MAPRE1	0.00	0.00	0.00	NaN

22920	KIFAP3	0.02	0.00	0.05	0.22
22924	MAPRE3	0.00	0.00	0.00	NaN
22925	PLA2R1	-0.02	0.00	0.03	0.09
22926	ATF6	0.07	0.00	0.08	0.27
22932	POMZP3	0.02	0.00	0.03	0.11
22933	SIRT2	0.02	0.09	0.05	0.24
22934	RPIA	0.00	0.00	0.00	NaN
22936	ELL2	0.00	0.00	-0.14	0.18
22937	SCAP	-0.04	0.00	0.00	0.03
22938	SNW1	-0.02	0.00	-0.03	0.21
22939	PTRF	-0.04	0.00	0.00	0.05
22941	SHANK2	0.11	0.09	0.03	0.30
22943	DKK1	-0.04	0.00	-0.03	0.15
22944	KIN	0.00	0.00	0.14	0.20
22948	CCT5	0.02	0.18	0.00	0.30
22950	SLC4A1AP	0.00	0.00	0.00	NaN
22953	P2RX2	0.00	0.00	0.00	NaN
22955	SCMH1	0.00	0.00	0.00	NaN
22977	AKR7A3	0.00	0.00	0.00	NaN
22978	NT5C2	0.00	0.00	-0.03	0.13
22981	KIAA0980	0.02	0.00	-0.03	0.15
22986	SORCS3	-0.02	0.00	-0.03	-0.11
22992	FBXL11	0.11	0.09	0.03	0.34
23008	KIAA0265	0.02	0.00	0.00	0.11
23011	RAB21	0.04	0.00	0.03	0.60
23012	STK38L	0.00	-0.09	0.05	0.18
23014	FBXO21	0.02	0.00	-0.03	0.41
23037	PDZD2	0.02	0.18	0.00	0.28
23040	MYT1L	-0.02	0.00	0.00	0.00
23049	SMG1	0.02	0.09	0.00	0.15
23054	NCOA6	0.04	0.00	0.00	0.33
23062	GGA2	0.02	0.00	0.00	0.14
23065	KIAA0090	0.00	0.00	0.00	NaN
23070	KIAA0082	0.02	0.00	0.05	0.12
23074	KIAA0701	0.00	0.00	0.03	0.14
23075	SWAP70	0.00	0.00	-0.05	-0.04
23076	KIAA0179	0.00	0.00	0.00	0.25
23078	KIAA0564	-0.05	0.00	-0.05	0.08
23080	KIAA0241	-0.02	0.09	-0.03	0.44
23089	PEG10	0.02	0.00	-0.03	-0.11
23095	KIF1B	0.00	0.00	0.00	NaN
23107	MRPS27	0.00	0.00	-0.11	0.14
23114	NFASC	0.07	0.00	0.03	0.06
23116	KIAA0423	0.00	0.00	-0.03	0.26
23118	MAP3K7IP2	0.00	0.00	-0.03	0.56
23119	HIC2	0.00	0.00	0.05	0.16
23122	CLASP2	0.02	0.00	-0.03	0.39

23136	EPB41L3	0.00	-0.09	-0.03	0.25
23152	CIC	0.02	0.00	-0.03	-0.04
23161	SNX13	0.00	0.00	0.00	0.32
23162	MAPK8IP3	0.04	0.18	0.00	0.26
23163	GGA3	0.04	0.18	0.05	0.44
23166	STAB1	0.00	0.00	0.00	NaN
23172	KIAA0157	-0.04	0.00	0.03	0.30
23173	METAP1	-0.02	0.00	0.03	0.26
23175	LPIN1	-0.02	0.00	0.00	0.29
23178	PASK	0.00	0.00	0.00	NaN
23190	UBXD2	-0.02	0.00	0.00	0.13
23191	CYFIP1	-0.02	0.09	-0.08	0.38
23194	FBXL7	0.02	0.18	0.00	0.05
23195	MDN1	-0.04	0.00	0.00	0.02
23199	KIAA0182	-0.09	0.00	0.00	0.09
23200	ATP11B	0.04	0.00	0.00	0.05
23203	INPP5E	0.02	0.00	0.03	0.03
23212	RRS1	0.11	0.00	0.16	0.24
23216	TBC1D1	-0.05	0.00	-0.03	0.17
23228	PLCL2	-0.02	0.00	-0.03	0.08
23229	ARHGEF9	-0.04	0.00	-0.03	0.10
23236	PLCB1	0.00	0.00	-0.05	0.17
23237	ARC	0.16	0.09	0.38	-0.21
23240	KIAA0922	-0.02	0.00	-0.03	0.07
23245	ASTN2	-0.02	0.00	0.00	0.20
23246	BOP1	0.16	0.00	0.35	0.35
23247	KIAA0556	0.02	0.00	0.00	0.04
23255	KIAA0802	0.00	-0.09	0.00	0.07
23263	MCF2L	0.00	0.00	0.00	0.33
23273	KIAA0367	-0.02	0.00	0.00	0.05
23274	KIAA0350	0.04	0.00	0.00	0.10
23277	KIAA0664	-0.05	0.00	-0.03	0.05
23279	NUP160	-0.02	0.00	-0.03	0.39
23281	KIAA0774	-0.05	0.00	-0.05	-0.06
23287	AGTPBP1	-0.02	0.00	0.00	0.04
23299	BICD2	-0.02	0.00	0.00	0.05
23302	KIAA0523	-0.05	0.00	0.00	0.07
23303	KIF13B	-0.13	-0.18	-0.19	0.27
23321	TRIM2	-0.02	0.00	-0.03	0.09
23322	KIAA1005	-0.05	0.00	0.00	0.20
23327	NEDD4L	-0.02	0.00	-0.03	-0.02
23332	CLASP1	0.00	0.00	0.00	NaN
23334	KIAA0467	0.00	0.00	0.00	NaN
23335	WDR7	-0.02	-0.09	-0.03	0.12
23336	DMN	0.05	0.00	0.00	-0.01
23339	VPS39	-0.04	0.09	-0.14	0.15
23351	KIAA0323	-0.02	0.00	0.00	0.02

23365	ARHGEF12	-0.07	0.00	0.00	0.07
23366	KIAA0895	-0.02	0.00	0.00	0.35
23369	PUM2	0.00	0.00	0.00	NaN
23376	KIAA0776	0.00	0.09	-0.03	0.35
23378	KIAA0409	-0.02	0.00	-0.03	0.08
23380	SRGAP2	0.07	0.00	0.00	0.16
23382	KIAA0828	0.02	0.00	0.00	-0.01
23385	NCSTN	0.05	0.00	0.14	0.29
23387	KIAA0999	-0.07	0.00	0.00	0.16
23394	ADNP	0.07	0.09	0.05	0.25
23395	LARS2	-0.04	0.00	0.00	0.09
23396	PIP5K1C	0.00	0.00	0.00	NaN
23401	FRAT2	-0.02	0.00	-0.03	0.12
23405	DICER1	-0.02	0.00	-0.03	0.12
23408	SIRT5	0.00	0.00	0.11	0.30
23409	SIRT4	0.02	0.00	0.00	0.06
23410	SIRT3	0.00	0.00	0.00	NaN
23411	SIRT1	-0.04	0.00	-0.05	0.24
23413	FREQ	0.02	0.00	0.00	0.11
23415	KCNH4	-0.04	0.00	0.00	0.11
23417	MLYCD	-0.09	0.00	0.00	0.12
23418	CRB1	0.07	0.00	0.03	-0.04
23421	ITGB3BP	-0.02	0.00	0.00	0.08
23426	GRIP1	0.00	0.00	0.00	NaN
23428	SLC7A8	-0.02	0.00	0.00	-0.07
23429	RYBP	-0.05	0.00	0.00	0.16
23430	TPSD1	0.04	0.18	0.03	-0.26
23431	AP4E1	-0.02	0.00	-0.05	0.17
23435	TARDBP	0.00	0.00	0.00	NaN
23436	ELA3B	0.00	0.00	0.00	NaN
23438	HARSL	0.00	0.00	0.00	NaN
23439	ATP1B4	-0.05	0.00	-0.08	-0.13
23443	SLC35A3	-0.02	0.00	-0.03	0.11
23450	SF3B3	-0.09	0.00	0.03	0.34
23451	SF3B1	0.02	0.00	0.00	0.29
23457	ABCB9	0.02	0.00	0.00	0.03
23460	ABCA6	0.05	0.09	0.03	-0.07
23461	ABCA5	0.05	0.09	0.03	0.33
23462	HEY1	0.13	0.09	0.14	0.20
23463	ICMT	-0.02	0.00	0.00	0.11
23464	GCAT	-0.04	0.00	0.03	0.11
23466	CBX6	-0.04	0.00	-0.03	0.13
23468	CBX5	0.02	0.00	-0.03	0.11
23469	PHF3	-0.02	0.00	0.05	0.44
23473	CAPN7	-0.02	0.00	0.00	0.20
23476	BRD4	0.02	0.00	0.00	0.08
23480	SEC61G	-0.02	0.00	0.00	0.19

23481	PES1	0.00	0.00	0.05	-0.11
23484	LEPROTL1	-0.09	0.00	-0.19	0.39
23493	HEY2	-0.02	0.00	0.00	-0.05
23498	HAAO	0.00	0.00	0.00	NaN
23499	MACF1	0.00	0.09	0.00	0.15
23500	DAAM2	0.00	0.00	0.08	0.10
23506	KIAA0240	0.00	0.00	0.08	0.14
23509	POFUT1	0.00	0.00	0.00	NaN
23513	SCRIB	0.16	0.00	0.35	0.30
23521	RPL13A	0.02	0.00	-0.03	0.45
23523	CABIN1	0.00	0.00	0.05	-0.05
23524	SRRM2	0.04	0.09	0.00	0.16
23527	CENTB2	0.02	0.09	0.00	0.23
23528	ZNF281	0.09	0.00	0.03	0.25
23530	NNT	0.02	0.09	0.00	-0.01
23531	MMD	0.13	0.27	0.03	0.34
23532	PRAME	-0.02	0.00	0.03	0.18
23536	ADAT1	-0.09	0.00	0.03	0.13
23538	OR52A1	-0.02	-0.09	-0.03	-0.02
23539	SLC16A8	-0.04	0.00	0.03	0.17
23541	SEC14L2	0.00	0.00	0.05	0.08
23542	MAPK8IP2	0.00	0.09	0.03	0.00
23543	RBM9	-0.02	0.00	0.03	0.17
23544	SEZ6L	-0.05	-0.09	0.03	0.11
23545	ATP6V0A2	0.02	0.00	0.00	0.21
23546	SYNGR4	0.00	0.00	-0.03	-0.19
23549	DNPEP	0.00	0.00	0.00	NaN
23552	CCRK	-0.02	0.00	0.00	0.03
23553	HYAL4	0.00	0.00	-0.03	0.12
23556	PIGN	-0.02	-0.09	0.03	0.19
23558	WBP2	0.04	0.27	0.03	0.24
23562	CLDN14	0.00	0.00	0.00	0.04
23563	CHST5	-0.09	0.00	0.03	0.06
23564	DDAH2	0.00	0.00	0.05	0.07
23566	EDG7	-0.02	0.00	-0.03	0.00
23583	SMUG1	0.02	0.00	-0.03	0.44
23594	ORC6L	-0.11	0.09	0.03	0.26
23595	ORC3L	-0.02	0.00	-0.05	0.33
23596	OPN3	0.07	0.00	0.05	0.11
23600	AMACR	0.02	0.27	0.00	0.42
23603	CORO1C	0.00	0.00	0.00	NaN
23604	DAPK2	0.00	0.09	-0.08	0.08
23607	CD2AP	0.00	0.00	0.05	0.13
23608	MKRN1	0.02	0.00	0.00	0.03
23609	MKRN2	0.02	0.00	0.00	0.15
23612	PHLDA3	0.07	0.00	0.05	0.18
23613	PRKCBP1	0.05	0.00	0.03	0.38

23616	SH3BP1	-0.04	0.00	0.03	0.13
23619	ZIM2	0.02	0.09	-0.03	-0.07
23620	NTSR2	-0.02	0.00	0.00	-0.02
23624	CBLC	-0.02	0.00	-0.03	0.22
23630	KCNE1L	-0.05	0.00	-0.08	0.13
23632	CA14	0.07	0.09	0.14	-0.09
23633	KPNA6	0.02	0.09	0.00	0.31
23635	SSBP2	0.00	0.09	-0.14	0.15
23640	HSPBP1	0.00	0.09	-0.03	0.04
23641	LDOC1	-0.05	0.00	-0.08	-0.08
23645	PPP1R15A	0.00	0.00	-0.03	0.12
23649	POLA2	0.02	0.00	0.03	0.19
23650	TRIM29	-0.07	0.00	0.00	0.18
23657	SLC7A11	-0.02	0.00	-0.03	0.04
23658	LSM5	-0.02	0.09	-0.03	0.42
23659	LYPLA3	-0.05	0.00	0.00	0.15
23660	ZFP95	0.02	0.00	0.00	0.08
23670	TMEM2	-0.02	0.00	-0.03	0.02
23673	STX12	-0.02	0.00	0.00	0.31
23676	SMPX	-0.05	0.00	-0.11	0.07
23677	SH3BP4	0.00	0.00	0.00	NaN
23682	RAB38	-0.05	0.00	0.00	0.13
23705	IGSF4	-0.07	0.00	0.00	0.20
23708	GSPT2	-0.04	0.00	-0.08	0.04
23710	GABARAPL1	0.00	-0.18	0.08	0.21
23729	CARKL	-0.04	0.00	0.00	0.28
23733	C9orf3	-0.02	0.00	0.00	-0.04
23742	C15orf2	0.00	0.09	-0.08	-0.03
23743	BHMT2	0.00	0.09	-0.14	-0.15
23746	AIPL1	-0.05	0.00	0.00	0.08
23753	SDF2L1	0.00	0.00	0.03	0.10
23759	PPIL2	0.00	0.00	0.03	0.17
23761	PISD	0.00	0.00	0.03	-0.04
23762	OSBP2	0.00	0.00	0.05	0.09
23764	MAFF	-0.04	0.00	0.03	0.23
23768	FLRT2	-0.04	0.00	0.00	0.01
23770	FKBP8	0.02	0.00	0.00	0.14
23774	BRD1	-0.02	0.00	-0.03	0.00
23780	APOL2	-0.02	0.00	0.03	0.08
23787	MTCH1	0.02	0.00	0.08	0.20
23788	MTCH2	-0.02	0.00	-0.03	0.43
24137	KIF4A	-0.04	0.00	-0.08	-0.14
24139	EML2	-0.02	0.00	0.00	0.12
24140	FTSJ1	-0.04	0.00	-0.08	0.35
24141	C20orf103	0.00	0.00	-0.05	0.00
24144	TIP39	0.02	0.00	-0.03	0.35
24145	PANX1	-0.07	0.09	-0.03	0.16

24146	CLDN15	0.02	0.00	0.00	0.08
24147	FJX1	0.04	0.00	-0.03	0.13
25764	HYPK	-0.04	0.00	-0.14	0.18
25769	SLC24A2	-0.05	0.00	-0.03	0.04
25788	RAD54B	0.16	0.09	0.24	0.30
25792	CIZ1	0.02	0.00	0.00	0.17
25793	FBXO7	-0.02	0.00	0.03	0.06
25796	PGLS	0.02	0.00	0.00	0.27
25797	QPCT	0.00	0.00	0.00	NaN
25801	GCA	-0.02	0.00	0.03	0.08
25802	LMOD1	0.11	0.00	0.05	0.05
25804	LSM4	0.02	0.00	0.00	0.28
25806	VAX2	0.02	0.00	0.00	0.00
25809	TTLL1	-0.02	0.00	-0.03	0.24
25819	CCRN4L	0.00	0.00	0.00	NaN
25820	ARIH1	0.00	0.00	-0.05	0.09
25821	MTO1	0.00	0.00	-0.03	0.31
25822	DNAJB5	0.00	0.00	0.00	-0.05
25825	BACE2	0.00	0.00	0.00	0.33
25827	FBXL2	0.02	0.00	-0.03	0.07
25828	TXN2	-0.02	0.00	0.03	-0.05
25829	C22orf5	-0.04	0.00	0.00	-0.01
25830	SULT4A1	-0.02	0.00	-0.03	-0.23
25837	RAB26	0.04	0.09	0.00	0.20
25839	COG4	-0.09	0.00	0.03	0.12
25843	PREI3	0.02	0.00	0.00	0.25
25849	DKFZP564O08	-0.02	0.00	0.00	0.13
25850	ZNF345	0.02	0.09	0.03	0.23
25855	BRMS1	0.07	0.00	0.03	0.39
25891	DKFZP586H21	0.04	0.00	-0.03	0.03
25913	POT1	0.00	0.00	-0.03	0.16
25920	COBRA1	0.02	0.00	0.03	0.05
25932	CLIC4	0.00	0.00	0.00	NaN
25937	TAZ	0.00	0.00	-0.03	0.00
25939	SAMHD1	0.02	0.00	0.00	0.10
25975	EGFL6	-0.05	-0.09	-0.14	-0.07
25980	C20orf4	0.04	0.00	0.00	0.24
25988	MIZF	-0.05	0.00	0.00	-0.05
25998	IBTK	-0.02	0.00	-0.05	0.29
26031	OSBPL3	0.00	0.18	0.03	0.17
26039	SS18L1	0.04	0.09	0.05	0.36
26040	SETBP1	0.00	-0.09	-0.03	-0.02
26047	CNTNAP2	0.02	0.00	0.00	0.13
26051	PPP1R16B	0.02	0.00	0.03	-0.06
26058	TNRC15	0.02	0.00	0.00	-0.10
26060	APPL	-0.05	0.00	-0.03	0.14
26063	DECR2	0.05	0.18	0.05	0.19

26064	RAI14	0.02	0.27	0.00	0.26
26085	KLK13	0.00	0.00	-0.03	-0.16
26088	GGA1	-0.04	0.00	0.03	0.32
26108	PYGO1	-0.02	0.00	-0.05	0.05
26118	WSB1	0.00	0.09	0.00	0.39
26121	PRPF31	0.00	0.09	0.00	0.65
26136	TES	0.00	0.00	-0.03	0.13
26152	ZNF337	0.00	0.00	-0.03	0.13
26154	ABCA12	0.00	0.00	0.00	NaN
26168	SENP3	-0.05	0.00	0.00	0.10
26190	FBXW2	-0.02	0.00	0.00	0.14
26191	PTPN22	0.00	0.00	-0.03	-0.03
26205	GMEB2	0.07	0.09	0.08	0.35
26206	SPAG8	0.00	0.00	0.00	0.35
26227	PHGDH	0.00	0.00	0.00	0.23
26228	BRDG1	-0.02	0.00	0.00	0.03
26229	B3GAT3	-0.02	0.00	0.03	0.12
26230	TIAM2	-0.02	0.00	-0.05	-0.18
26232	FBXO2	-0.02	0.00	0.00	0.04
26233	FBXL6	0.16	0.00	0.35	0.26
26234	FBXL5	-0.04	-0.09	-0.05	0.08
26235	FBXL4	-0.02	0.00	0.00	0.08
26249	KLHL3	0.00	0.00	-0.03	0.15
26251	KCNG2	-0.02	-0.09	0.00	0.03
26256	CABYR	0.00	-0.09	0.03	0.10
26261	FBXO24	0.02	0.00	0.00	0.17
26263	FBXO22	0.00	0.00	-0.05	0.14
26266	SLC13A4	0.02	0.00	0.03	-0.15
26268	FBXO9	-0.02	0.00	0.08	0.23
26271	FBXO5	0.00	0.00	0.00	0.25
26273	FBXO3	0.04	0.00	-0.05	0.28
26275	HIBCH	0.00	0.00	0.00	NaN
26276	VPS33B	0.02	0.00	0.03	0.15
26277	TINF2	-0.02	0.00	0.00	0.04
26278	SACS	-0.07	0.00	-0.11	0.09
26279	PLA2G2D	0.00	0.00	0.00	NaN
26280	IL1RAPL2	-0.05	0.00	-0.08	-0.10
26281	FGF20	-0.18	-0.18	-0.22	0.03
26284	ERAL1	0.02	0.09	0.00	0.33
26285	CLDN17	0.00	0.00	0.00	0.13
26286	ARFGAP1	0.04	0.09	0.08	-0.15
26287	ANKRD2	-0.02	0.00	-0.03	-0.01
26289	AK5	-0.02	0.00	-0.03	-0.07
26290	GALNT8	-0.02	0.09	0.08	-0.11
26291	FGF21	0.00	0.00	-0.03	-0.11
26292	MYCBP	0.00	0.09	0.00	0.41
26298	EHF	0.04	0.00	0.03	0.13

26468	LHX6	-0.02	0.00	0.00	0.02
26469	PTPN18	-0.02	0.00	0.00	0.05
26472	PPP1R14B	0.02	0.09	0.05	-0.02
26476	OR10J1	0.04	0.00	0.16	0.02
26499	PLEK2	-0.02	0.00	0.00	0.16
26502	NARF	-0.02	0.09	0.08	0.10
26503	SLC17A5	0.00	0.00	-0.03	0.15
26504	CNNM4	0.00	0.00	0.00	NaN
26505	CNNM3	0.00	0.00	0.00	NaN
26507	CNNM1	-0.02	0.00	-0.03	-0.08
26508	HEYL	-0.02	0.09	0.00	0.03
26509	FER1L3	-0.02	0.00	-0.05	0.13
26511	CHIC2	-0.02	0.00	0.00	0.18
26515	FXC1	-0.02	0.00	-0.03	0.32
26517	TIMM13	0.00	0.00	0.00	NaN
26519	TIMM10	0.02	0.00	0.05	0.38
26520	TIMM9	0.00	0.00	-0.03	0.18
26521	TIMM8B	-0.07	0.00	0.00	0.26
26523	EIF2C1	-0.02	0.00	0.00	0.14
26528	DAZAP1	0.02	0.09	-0.03	-0.01
26529	OR12D2	0.00	0.00	0.05	-0.10
26531	OR11A1	0.00	0.00	0.05	-0.03
26539	OR10H1	0.00	0.00	0.00	NaN
26548	ITGB1BP2	-0.04	0.00	-0.08	0.02
26575	RGS17	0.00	0.00	0.00	-0.10
26577	PCOLCE2	0.02	0.00	0.03	0.16
26578	OSTF1	-0.02	0.00	0.00	0.10
26586	CKAP2	-0.07	0.00	-0.08	0.12
26589	MRPL46	0.00	0.00	-0.05	0.22
26608	TBL2	0.02	0.00	0.05	0.14
26658	OR7C2	0.02	0.00	0.00	-0.08
26692	OR2W1	0.00	0.00	0.05	0.00
26747	NUFIP1	-0.05	0.00	-0.11	0.06
26750	RPS6KC1	0.07	0.00	0.00	0.33
26959	HBP1	0.02	0.00	-0.03	0.04
26960	NBEA	-0.05	0.00	-0.11	-0.02
26973	CHORDC1	-0.11	0.00	0.03	0.29
26984	SEC22A	0.00	0.00	-0.03	-0.32
26986	PABPC1	0.20	0.18	0.27	0.31
26994	RNF11	0.00	0.00	-0.03	0.05
26998	FETUB	0.04	0.00	0.03	0.01
26999	CYFIP2	0.00	0.00	0.00	NaN
27004	TCL6	-0.02	0.00	-0.03	0.12
27005	USP21	0.11	0.00	0.14	0.34
27010	TPK1	0.02	0.00	0.00	0.02
27019	DNAI1	0.00	0.00	0.00	0.05
27023	FOXB1	-0.02	0.00	-0.05	0.10

27030	MLH3	-0.02	0.00	0.00	-0.01
27032	ATP2C1	0.00	0.00	-0.03	0.49
27034	ACAD8	-0.07	0.00	-0.05	0.13
27035	NOX1	-0.05	0.00	-0.08	-0.01
27036	SIGLEC7	0.00	0.00	-0.03	-0.09
27037	HTF9C	-0.02	0.00	0.08	-0.14
27039	PKD2L2	0.00	0.00	-0.03	-0.03
27040	LAT	0.05	0.00	0.00	0.09
27043	PELP1	-0.04	0.00	0.00	0.16
27065	D4S234E	-0.02	0.00	0.00	0.07
27067	STAU2	0.11	0.09	0.08	0.25
27069	GHITM	-0.02	0.00	-0.05	0.21
27071	DAPP1	-0.02	0.00	0.03	0.17
27072	VPS41	-0.02	0.00	-0.03	0.16
27074	LAMP3	0.04	0.00	0.00	-0.02
27087	B3GAT1	-0.07	0.00	-0.05	0.10
27091	CACNG5	0.13	0.27	0.03	-0.08
27094	KCNMB3	0.04	0.00	0.03	0.16
27097	TAF5L	0.07	0.00	0.03	0.20
27098	CLUL1	0.02	-0.09	0.00	0.01
27113	BBC3	-0.04	0.00	-0.03	-0.08
27115	PDE7B	-0.02	0.00	0.03	0.04
27121	DKK4	0.02	0.09	0.11	0.15
27122	DKK3	0.00	0.00	-0.05	0.04
27123	DKK2	-0.02	0.00	0.00	-0.02
27124	PIB5PA	0.00	0.00	0.05	0.07
27128	PSCD4	-0.04	0.00	0.03	0.29
27129	HSPB7	0.02	0.09	0.00	-0.03
27130	INVS	-0.02	0.00	0.00	-0.05
27131	SNX5	0.00	0.00	-0.08	0.13
27132	CPNE7	-0.04	0.00	0.03	-0.07
27134	TJP3	0.00	0.00	0.00	NaN
27141	CIDEB	0.00	0.00	0.00	0.11
27156	RTDR1	-0.02	0.00	0.03	0.17
27173	SLC39A1	0.05	0.00	0.14	0.10
27175	TUBG2	-0.04	0.00	0.00	0.11
27180	SIGLEC9	0.00	0.00	-0.03	-0.04
27181	SIGLEC8	0.00	0.00	-0.03	0.02
27183	VPS4A	-0.09	0.00	0.03	0.11
27185	DISC1	0.09	0.00	0.03	-0.06
27190	IL17B	0.00	0.00	0.00	NaN
27229	76P	-0.04	0.00	-0.14	0.10
27230	SERP1	0.02	0.00	0.03	0.09
27232	GNMT	0.00	0.00	0.08	-0.14
27233	SULT1C2	0.00	0.00	0.00	NaN
27237	ARHGEF16	0.05	-0.09	0.08	0.01
27242	TNFRSF21	0.00	0.00	0.05	0.26

27246	ZNF364	0.05	0.00	0.11	0.32
27250	PDCD4	-0.04	0.00	0.00	0.22
27253	PCDH17	-0.07	0.00	-0.08	-0.01
27255	CNTN6	-0.02	0.00	0.00	-0.05
27257	LSM1	0.16	0.09	0.14	0.59
27258	LSM3	0.02	0.00	0.00	0.28
27284	SULT1B1	-0.02	0.00	0.00	0.10
27285	TEKT2	-0.02	0.00	0.00	0.07
27288	HNRNPG-T	-0.02	0.00	-0.05	0.01
27290	SPINK4	-0.02	0.09	0.00	0.17
27296	C20orf10	0.04	0.09	0.03	0.07
27299	ADAMDEC1	-0.13	-0.18	-0.19	0.09
27302	BMP10	0.00	0.00	0.00	NaN
27303	RBMS3	-0.02	0.00	-0.05	0.00
27304	MOCS3	0.07	0.09	0.05	0.24
27306	PGDS	-0.04	0.00	-0.03	0.35
27314	RAB30	0.00	0.00	0.00	0.24
27315	FRAG1	-0.02	0.00	-0.03	0.03
27316	RBMX	-0.02	0.00	-0.08	0.06
27330	RPS6KA6	-0.04	0.00	-0.08	0.21
27333	GOLPH4	0.02	0.00	0.03	0.16
27336	HTATSF1	-0.02	0.00	-0.08	0.28
27343	POLL	-0.02	0.00	-0.03	0.19
27344	PCSK1N	-0.04	0.00	-0.05	-0.07
27345	KCNMB4	0.02	0.00	0.03	0.06
27347	STK39	0.00	0.00	0.03	0.05
27430	MAT2B	0.00	0.00	-0.03	0.24
27434	POLM	-0.02	0.00	0.05	0.04
27436	EML4	0.00	0.00	0.00	NaN
27440	CECR5	-0.04	0.00	0.00	0.09
28316	CDH20	-0.02	-0.09	0.03	0.06
28513	CDH19	0.00	-0.09	0.00	0.19
28957	MRPS28	0.13	0.09	0.14	0.13
28964	GIT1	0.04	0.18	0.00	0.23
28973	MRPS18B	0.00	0.00	0.05	0.15
28977	MRPL42	0.00	0.09	0.00	0.15
28984	RGC32	-0.05	0.00	-0.05	0.14
28986	MAGEH1	-0.04	0.00	-0.05	0.13
28992	LRP16	0.00	0.00	0.03	0.04
28996	HIPK2	0.02	0.00	0.00	-0.03
28998	MRPL13	0.18	0.09	0.35	0.30
28999	KLF15	0.00	0.00	-0.03	-0.14
29058	C20orf30	0.02	0.00	-0.05	0.07
29062	HSPC049	0.02	0.00	0.03	0.08
29074	MRPL18	-0.02	0.00	-0.05	0.24
29086	HSPC142	0.04	0.00	0.00	0.32
29088	MRPL15	0.11	0.00	0.14	0.28

29093	MRPL22	0.00	0.00	0.00	NaN
29094	HSPC159	0.00	0.00	0.00	NaN
29104	N6AMT1	-0.02	0.00	0.05	-0.10
29106	SCG3	-0.02	0.00	-0.05	0.15
29107	NXT1	0.02	0.00	-0.03	0.16
29109	FHOD1	-0.04	0.00	0.00	0.00
29110	TBK1	0.00	0.00	0.00	NaN
29117	BRD7	-0.09	0.00	-0.03	0.25
29122	TSP50	-0.04	0.00	0.00	0.05
29127	RACGAP1	0.02	0.00	-0.03	0.26
29760	BLNK	-0.02	0.00	-0.05	0.22
29761	USP25	-0.05	-0.09	0.05	0.46
29763	PACSIN3	-0.02	0.00	-0.03	0.06
29766	TMOD3	-0.02	0.00	-0.05	0.09
29767	TMOD2	-0.02	0.00	-0.05	0.23
29775	CARD10	-0.04	0.00	0.03	0.08
29777	ABT1	0.00	0.00	0.03	0.09
29780	PARVB	-0.02	0.00	-0.03	0.30
29802	VPREB3	-0.02	0.00	0.03	-0.07
29844	TFPT	0.00	0.09	0.00	0.31
29851	ICOS	0.00	0.00	0.00	NaN
29855	UBN1	0.04	0.00	0.00	0.10
29880	ALG5	-0.05	0.00	-0.11	0.29
29881	NPC1L1	-0.02	0.00	0.05	0.05
29882	APC2	0.00	0.00	-0.03	0.20
29883	CNOT7	-0.18	-0.18	-0.22	0.34
29887	SNX10	0.00	0.18	0.03	-0.03
29894	CPSF1	0.15	0.00	0.30	0.27
29907	SNX15	0.02	0.00	0.03	0.20
29911	HOOK2	0.02	-0.09	0.00	0.13
29916	SNX11	0.04	0.18	0.00	0.47
29922	NME7	0.02	0.00	0.05	0.26
29923	HIG2	0.02	0.00	0.00	0.07
29925	GMPPB	0.00	0.00	0.00	NaN
29926	GMPPA	0.00	0.00	0.00	NaN
29927	SEC61A1	0.00	0.00	-0.03	0.39
29928	TIMM22	-0.05	0.00	0.00	0.02
29929	ALG6	-0.02	0.00	0.00	0.21
29930	PCDHB1	0.00	0.00	0.00	NaN
29942	PURG	-0.11	-0.18	-0.19	-0.04
29943	PADI1	0.00	0.00	0.00	NaN
29947	DNMT3L	0.00	0.00	0.00	-0.02
29949	IL19	0.09	0.00	0.00	0.05
29953	TRHDE	0.04	0.00	0.03	-0.02
29954	POMT2	-0.02	0.00	-0.03	-0.03
29956	LASS2	0.07	0.09	0.16	0.10
29960	FTSJ2	0.00	0.00	0.05	0.21

29965	C16orf5	0.04	0.00	0.00	0.06
29970	SCHIP1	0.02	0.00	0.03	0.20
29974	ACF	-0.02	0.00	-0.03	-0.01
29980	DONSON	0.00	0.00	0.00	0.01
29986	SLC39A2	-0.02	0.00	0.00	0.14
29988	SLC2A8	-0.02	0.00	0.00	0.01
29994	BAZ2B	-0.02	0.00	0.03	0.15
29995	LMCD1	0.00	0.00	0.00	0.14
29997	GLTSCR2	-0.02	0.00	0.00	0.47
29998	GLTSCR1	-0.04	0.00	0.00	0.00
29999	FSCN3	0.02	0.00	-0.03	0.00
30001	ERO1L	0.02	0.00	0.00	0.31
30008	EFEMP2	0.02	0.00	0.03	0.09
30009	TBX21	0.02	0.18	0.00	0.02
30013	S100A14	0.07	0.00	0.16	0.20
30811	HUNK	0.00	0.00	-0.03	-0.04
30813	VSX1	0.02	0.00	-0.03	-0.19
30814	PLA2G2E	0.00	0.00	0.00	NaN
30817	EMR2	0.02	0.00	0.00	-0.09
30819	KCNIP2	0.00	0.00	-0.03	-0.08
30820	KCNIP1	0.00	0.00	-0.03	0.00
30835	CD209	0.00	-0.09	0.00	-0.05
30844	EHD4	-0.04	0.09	-0.14	0.40
30845	EHD3	0.00	0.00	0.00	NaN
30846	EHD2	-0.04	0.00	0.00	0.17
30849	PIK3R4	0.00	0.00	-0.03	0.27
43847	KLK14	0.00	0.00	-0.03	-0.05
49856	WDR8	-0.02	0.00	0.00	0.00
50485	SMARCAL1	0.02	0.00	0.00	0.10
50486	G0S2	0.07	0.00	0.00	0.03
50487	PLA2G3	0.00	0.00	0.05	0.18
50506	DUOX2	-0.04	0.00	-0.11	0.16
50507	NOX4	-0.05	0.00	0.03	0.11
50508	NOX3	-0.02	0.00	-0.03	0.03
50509	COL5A3	0.00	0.00	0.00	NaN
50613	UBQLN3	-0.02	0.00	-0.03	0.01
50615	IL21R	0.02	0.00	0.00	-0.02
50616	IL22	0.02	0.00	0.00	0.09
50617	ATP6V0A4	0.02	0.00	0.03	0.22
50618	ITSN2	-0.02	0.00	0.00	0.10
50619	DEF6	0.00	0.00	0.05	0.11
50626	CYHR1	0.15	0.00	0.30	0.10
50649	ARHGEF4	0.00	0.00	0.00	NaN
50650	ARHGEF3	-0.05	0.00	-0.03	0.10
50674	NEUROG3	-0.02	0.00	0.03	0.03
50700	RDH8	0.00	0.00	0.00	NaN
50805	IRX4	0.02	0.18	0.00	0.11

50807	DDEF1	0.20	0.09	0.38	0.10
50813	COPS7A	-0.02	0.09	0.05	0.40
50831	TAS2R3	0.02	0.00	0.00	-0.11
50832	TAS2R4	0.02	0.00	0.00	0.12
50833	TAS2R16	0.00	0.00	-0.03	-0.02
50834	TAS2R1	0.02	0.18	0.00	-0.18
50835	TAS2R9	0.00	-0.18	0.08	-0.01
50836	TAS2R8	0.00	-0.18	0.08	0.08
50837	TAS2R7	0.00	-0.18	0.08	-0.09
50838	TAS2R13	0.00	-0.18	0.08	0.18
50839	TAS2R10	0.00	-0.18	0.08	0.08
50840	TAS2R14	0.00	-0.18	0.11	0.00
50853	VILL	0.00	0.00	0.00	-0.15
50855	PARD6A	-0.04	0.00	0.00	0.12
50861	STMN3	0.07	0.09	0.08	0.04
50863	HNT	-0.07	0.00	-0.05	-0.01
50865	HEBP1	0.00	-0.09	0.05	0.08
50937	CDON	-0.05	0.00	-0.03	0.02
50939	IMPG2	0.00	0.00	-0.03	-0.16
50940	PDE11A	0.00	0.00	0.00	NaN
50944	SHANK1	0.00	0.00	-0.03	-0.12
51012	C20orf45	0.05	0.09	0.03	0.27
51021	MRPS16	-0.02	0.00	0.00	0.12
51022	GLRX2	0.07	0.00	0.03	0.25
51023	MRPS18C	-0.04	0.00	-0.03	0.05
51025	Magmas	0.04	0.00	0.00	0.22
51050	PI15	0.09	0.00	0.08	-0.05
51056	LAP3	-0.04	-0.09	-0.05	0.12
51065	RPS27L	0.02	0.00	-0.05	0.14
51069	MRPL2	0.00	0.00	0.08	0.15
51070	NOSIP	0.02	0.00	-0.03	0.46
51073	MRPL4	0.00	0.00	0.00	NaN
51081	MRPS7	0.04	0.18	0.05	0.44
51084	CRYL1	-0.07	0.00	-0.08	0.02
51100	SH3GLB1	-0.02	0.00	-0.03	0.13
51106	TFB1M	-0.02	0.00	-0.03	0.12
51116	MRPS2	0.02	0.00	0.03	0.03
51117	COQ4	0.02	0.00	0.00	0.12
51121	RPL26L1	0.00	0.00	-0.03	0.00
51127	TRIM17	0.09	0.09	0.03	0.26
51129	ANGPTL4	0.00	-0.09	0.00	-0.13
51135	IRAK4	0.00	0.00	0.03	0.09
51138	COPS4	-0.04	0.00	-0.03	0.17
51144	HSD17B12	-0.02	0.00	-0.08	0.15
51147	ING4	-0.02	0.09	0.05	0.35
51155	HN1	0.05	0.18	0.05	0.33
51156	SERPINA10	-0.02	0.00	0.00	-0.08

51160	VPS28	0.15	0.00	0.30	0.27
51163	DBR1	0.02	0.00	0.00	0.15
51164	DCTN4	0.00	0.00	0.00	NaN
51168	MYO15A	-0.05	0.00	0.00	0.17
51176	LEF1	-0.02	0.00	0.00	-0.02
51179	HAO2	0.00	0.00	0.00	-0.06
51187	C15orf15	-0.02	0.00	-0.05	0.14
51188	SS18L2	-0.04	0.00	0.00	0.09
51196	PLCE1	-0.02	0.00	-0.05	-0.04
51199	NIN	0.02	0.00	0.00	0.10
51200	CPA4	0.02	0.00	0.00	0.16
51206	GP6	0.00	0.09	-0.03	-0.16
51208	CLDN18	0.02	0.00	0.00	0.02
51222	ZNF219	-0.02	0.00	0.00	-0.09
51232	CRIM1	0.00	0.00	0.00	NaN
51268	PIPOX	0.02	0.18	0.00	0.21
51274	KLF3	-0.05	0.00	-0.03	0.24
51278	IER5	0.04	0.00	0.03	0.29
51280	GOLPH2	-0.02	0.00	0.00	-0.05
51282	SCAND1	0.04	0.00	0.03	0.39
51284	TLR7	-0.05	-0.09	-0.14	0.17
51294	PCDH12	0.00	0.00	0.00	NaN
51297	PLUNC	0.00	0.00	0.00	NaN
51298	THEG	0.00	0.00	-0.03	-0.04
51302	CYP39A1	0.00	0.00	0.05	0.19
51304	ZDHHC3	-0.04	0.00	0.00	0.17
51306	C5orf5	0.00	0.00	-0.03	0.12
51311	TLR8	-0.05	-0.09	-0.14	-0.06
51318	MRPL35	0.00	0.00	0.00	NaN
51322	WAC	-0.02	0.00	0.03	0.23
51327	ERAF	0.04	0.09	0.03	-0.14
51332	SPTBN5	-0.04	0.09	-0.14	0.01
51338	MS4A4A	-0.04	0.00	0.03	0.24
51340	CRNKL1	0.00	0.00	-0.08	0.34
51343	FZR1	0.00	0.00	0.00	NaN
51348	KLRF1	0.00	-0.18	0.05	0.12
51360	MBTPS2	-0.05	0.00	-0.11	-0.01
51361	HOOK1	0.00	0.00	0.00	0.17
51367	POP5	0.02	0.00	0.00	0.06
51373	MRPS17	0.00	0.00	0.05	0.59
51375	SNX7	-0.02	0.00	-0.03	0.09
51378	ANGPT4	0.02	0.00	-0.03	-0.05
51379	CRLF3	0.00	0.00	0.00	0.23
51380	CSAD	0.02	0.00	-0.03	0.09
51382	ATP6V1D	-0.02	0.00	0.00	0.14
51384	WNT16	0.00	0.00	-0.03	-0.08
51386	EIF3S6IP	-0.04	0.00	0.03	-0.06

51411	BIN2	0.02	0.00	-0.03	-0.17
51421	AMOTL2	0.00	0.00	0.00	NaN
51422	PRKAG2	0.02	0.00	0.00	0.09
51430	C1orf9	0.02	0.00	0.05	0.27
51433	ANAPC5	0.02	0.00	0.00	0.30
51438	MAGEE1	-0.04	0.00	-0.08	-0.15
51439	FAM8A1	0.00	0.00	0.05	0.19
51440	HPCAL4	-0.02	0.09	0.00	0.11
51447	IHPK2	-0.04	0.00	0.00	0.05
51458	RHCG	0.02	0.00	-0.05	-0.01
51475	CABP2	0.07	0.09	0.03	0.06
51478	HSD17B7	0.05	0.00	0.14	0.25
51491	HSPC111	0.00	0.00	-0.03	0.06
51497	TH1L	0.05	0.09	0.03	0.49
51507	C20orf43	0.04	0.00	0.05	0.41
51512	GTSE1	-0.02	0.00	-0.03	0.01
51526	C20orf111	0.02	0.09	0.03	0.27
51540	SCLY	0.02	0.00	0.00	0.06
51547	SIRT7	0.00	0.09	0.08	-0.01
51548	SIRT6	0.00	0.00	0.00	NaN
51550	CINP	0.00	0.00	-0.03	0.17
51552	RAB14	-0.02	0.00	0.00	0.06
51560	RAB6B	0.00	0.00	0.00	NaN
51561	IL23A	0.00	0.00	0.00	NaN
51562	MBIP	0.02	0.09	0.00	0.11
51564	HDAC7A	0.00	0.00	0.00	NaN
51573	MIR16	0.02	-0.09	0.00	0.11
51585	PCF11	0.00	0.00	0.00	-0.08
51586	PCQAP	-0.02	0.00	0.05	-0.21
51592	TRIM33	0.00	0.00	-0.03	0.07
51593	ARS2	0.02	0.00	0.00	0.25
51594	NAG	-0.02	0.00	0.00	0.11
51606	ATP6V1H	0.13	0.00	0.14	0.10
51621	KLF13	-0.04	0.00	-0.14	0.20
51642	MRPL48	0.05	0.09	-0.03	0.35
51650	MRPS33	0.02	0.00	0.00	0.04
51663	ZFR	0.02	0.18	0.00	0.29
51666	ASB4	0.02	0.00	-0.03	0.07
51678	MPP6	0.00	0.18	0.03	0.17
51701	NLK	0.04	0.18	0.00	0.05
51702	PADI3	0.00	0.00	0.00	NaN
51704	GPRC5B	0.02	-0.09	0.00	0.22
51715	RAB23	-0.04	0.00	0.08	0.25
51728	POLR3K	0.05	0.09	0.00	0.24
51729	WBP11	0.00	-0.09	0.05	0.09
51733	UPB1	0.00	0.00	0.03	0.15
51734	SEPX1	0.04	0.18	0.00	0.10

51741	WWOX	-0.09	0.00	0.00	0.15
51744	CD244	0.07	0.00	0.14	-0.05
51752	ARTS-1	0.00	0.00	-0.14	0.11
51761	ATP8A2	-0.05	0.00	-0.08	-0.08
51763	SKIP	-0.05	0.00	0.00	0.35
51765	MST4	-0.04	0.00	-0.08	0.08
51768	TM7SF3	0.00	-0.09	0.05	0.05
51776	ZAK	-0.02	0.00	0.00	-0.08
51778	MYOZ2	0.00	0.00	-0.03	0.07
51807	TUBA8	-0.02	0.00	0.00	-0.08
51809	GALNT7	-0.02	0.00	-0.05	0.13
51816	CECR1	-0.04	0.00	0.00	0.08
53335	BCL11A	0.00	0.00	0.00	NaN
53339	BTBD1	0.00	0.00	-0.03	0.03
53340	SPA17	-0.05	0.00	-0.03	-0.05
53343	NUDT9	-0.04	0.00	-0.03	0.14
53346	TM6SF1	0.00	0.00	-0.03	-0.04
53347	UBASH3A	0.00	0.00	0.00	0.02
53353	LRP1B	0.00	0.00	0.00	NaN
53358	SHC3	-0.02	0.00	0.00	-0.06
53371	NUP54	-0.02	0.00	0.00	0.06
53405	CLIC5	0.00	0.00	0.05	-0.08
53407	STX18	-0.02	0.00	0.00	0.28
53615	MBD3	0.00	0.00	-0.03	0.14
53616	ADAM22	0.02	0.00	0.00	0.21
53635	PTOV1	0.02	0.00	-0.03	0.20
53637	EDG8	0.02	0.00	0.00	0.16
53820	DSCR6	0.00	0.00	0.00	-0.03
53822	FXYD7	0.00	0.00	0.08	0.25
53826	FXYD6	-0.05	0.00	0.00	0.24
53827	FXYD5	0.00	0.00	0.08	0.09
53832	IL20RA	-0.02	0.00	0.05	0.08
53838	C11orf24	0.07	0.09	0.03	0.32
53841	MUCDHL	0.02	0.00	0.00	0.05
53904	MYO3A	-0.02	0.00	0.00	-0.18
53905	DUOX1	-0.04	0.00	-0.11	0.12
53916	RAB4B	0.00	0.00	0.00	0.22
53918	PELO	0.00	0.00	-0.08	0.21
53942	CNTN5	-0.04	-0.09	0.00	-0.02
53944	CSNK1G1	0.00	0.09	-0.08	0.23
53947	A4GALT	0.00	0.00	-0.03	-0.25
54020	SLC37A1	0.00	0.00	0.00	0.12
54039	PCBP3	0.00	0.09	0.00	0.15
54069	C21orf45	0.00	0.00	-0.03	0.09
54107	POLE3	-0.02	0.00	0.00	0.14
54112	GPR88	-0.02	0.00	-0.03	-0.07
54148	MRPL39	0.00	0.00	0.03	0.20

54149	C21orf91	-0.04	-0.18	0.05	0.26
54207	KCNK10	-0.04	0.00	0.00	0.05
54209	TREM2	0.00	0.00	0.08	0.05
54210	TREM1	0.00	0.00	0.08	0.01
54212	SNTG1	0.13	0.00	0.11	0.08
54221	SNTG2	-0.02	0.00	0.00	-0.06
54329	GPR85	0.00	0.00	-0.03	-0.02
54332	GDAP1	0.09	0.00	0.08	0.22
54344	DPM3	0.09	0.00	0.14	0.21
54345	SOX18	0.09	0.00	0.08	0.04
54361	WNT4	0.00	0.00	0.00	NaN
54363	HAO1	0.00	0.00	-0.05	-0.03
54407	SLC38A2	0.00	0.00	0.00	NaN
54413	NLGN3	-0.04	0.00	-0.08	0.03
54433	NOLA1	-0.02	0.00	0.00	0.01
54434	SSH1	0.00	0.00	0.00	NaN
54453	RIN2	0.00	0.00	-0.08	0.26
54457	TAF7L	-0.05	0.00	-0.08	0.03
54462	KIAA1128	-0.02	0.00	-0.05	0.27
54463	FLJ20152	0.02	0.18	0.00	0.12
54468	FLJ20323	0.00	0.00	0.03	0.29
54472	TOLLIP	0.00	0.00	0.00	NaN
54474	KRT20	0.00	0.09	0.00	0.05
54476	TRIAD3	0.00	0.00	0.00	NaN
54487	DGCR8	-0.02	0.00	0.08	0.03
54502	FLJ20273	-0.04	0.00	-0.08	0.30
54504	CPVL	-0.02	0.18	-0.03	0.02
54530	FLJ20054	0.07	0.00	0.03	0.14
54542	MNAB	-0.02	0.00	0.00	0.48
54554	WDR5B	0.00	0.00	-0.03	-0.30
54556	ING3	0.00	0.00	-0.03	0.13
54558	SPATA6	0.00	0.00	-0.03	0.04
54576	UGT1A8	0.00	0.00	0.00	NaN
54583	EGLN1	0.09	0.00	0.03	0.06
54585	LZTFL1	-0.04	0.00	0.00	-0.05
54621	FLJ20674	0.00	0.00	-0.03	0.07
54676	GTPBP2	0.00	0.00	0.11	0.18
54677	CROT	0.02	0.00	0.00	-0.01
54714	CNGB3	0.15	0.00	0.14	-0.02
54715	A2BP1	0.02	0.00	0.00	0.03
54718	BTN2A3	0.00	0.00	0.03	0.03
54766	BTG4	-0.07	0.00	0.00	-0.04
54788	DNAJB12	-0.02	0.00	0.00	0.14
54795	TRPM4	0.00	0.00	-0.03	0.01
54805	CNNM2	0.00	0.00	-0.03	0.01
54810	GIPC2	-0.02	0.00	-0.03	-0.12
54821	FLJ20105	-0.04	0.00	-0.08	-0.01

54834	GDAP2	0.00	0.00	-0.03	-0.10
54840	APTX	-0.04	0.09	0.00	0.41
54842	FLJ20160	0.00	0.00	0.00	NaN
54848	FLJ20184	-0.02	0.00	0.00	0.06
54849	FLJ20186	-0.04	0.00	0.00	0.12
54860	MS4A12	-0.02	0.00	0.03	0.18
54866	PPP1R14D	0.00	0.00	-0.14	-0.02
54867	FLJ20254	0.00	0.00	0.00	NaN
54873	PALMD	-0.02	0.00	-0.03	0.12
54878	DPP8	0.00	0.09	-0.05	0.18
54879	ST7L	0.02	0.00	-0.03	0.17
54891	FLJ20309	0.00	0.00	0.00	NaN
54898	ELOVL2	-0.02	0.00	0.11	0.03
54904	WHSC1L1	0.16	0.09	0.11	0.51
54910	SEMA4C	0.00	0.00	0.00	NaN
54932	FLJ20433	0.02	0.00	0.03	-0.15
54943	C21orf55	0.00	0.00	0.00	0.00
54948	MRPL16	-0.04	0.00	0.00	0.13
54953	C1orf27	0.04	0.09	0.03	0.24
54971	BANP	-0.04	0.00	0.00	0.18
54976	C20orf27	0.02	0.00	-0.03	0.07
54979	HRASLS2	0.00	0.00	0.03	0.01
54982	CLN6	0.00	0.00	-0.05	0.01
54988	FLJ20581	0.02	-0.09	0.00	0.09
54994	C20orf11	0.04	0.09	0.08	0.31
55020	FLJ20699	-0.02	0.00	-0.03	-0.01
55022	FLJ20701	0.00	0.00	0.00	NaN
55023	PHIP	-0.04	0.00	-0.03	0.07
55038	CDCA4	0.00	0.00	0.08	-0.06
55040	EPN3	0.15	0.18	0.03	0.38
55049	FLJ20850	0.02	0.00	0.00	0.30
55074	OXR1	0.20	0.09	0.30	0.19
55082	FLJ10154	-0.02	0.00	-0.05	0.27
55084	FLJ10159	0.07	0.00	0.03	-0.06
55089	SLC38A4	0.00	0.00	0.00	NaN
55101	FLJ10241	0.02	0.00	-0.03	0.29
55110	FLJ10292	0.00	-0.18	0.08	0.15
55118	CRTAC1	-0.02	0.00	-0.03	-0.05
55124	PIWIL2	-0.13	-0.18	-0.22	-0.04
55146	ZDHHC4	0.00	0.00	0.00	NaN
55168	MRPS18A	0.00	0.00	0.11	0.20
55173	MRPS10	0.00	0.00	0.08	0.28
55184	C20orf12	0.00	0.00	-0.08	0.15
55193	PB1	0.00	0.00	0.00	NaN
55217	TMLHE	-0.05	0.09	-0.11	0.07
55228	FLJ10781	-0.02	0.00	-0.03	0.04
55238	FLJ10815	-0.07	0.00	0.00	0.23

55243	KIRREL	0.02	0.00	0.16	0.31
55244	FLJ10847	-0.04	0.09	0.03	0.06
55245	C20orf44	0.05	0.00	0.00	0.12
55257	C20orf20	0.04	0.09	0.08	0.32
55258	FLJ10916	0.00	0.00	0.00	NaN
55277	FLJ10986	0.00	0.00	0.00	-0.07
55283	MCOLN3	-0.02	0.00	-0.03	0.02
55290	BRF2	0.16	0.09	0.08	0.66
55294	FBXW7	-0.02	0.00	0.00	0.04
55313	FLJ11151	0.02	0.00	0.00	0.04
55317	C20orf29	0.02	0.00	-0.03	0.02
55319	FLJ11184	0.02	0.00	-0.05	0.09
55321	C20orf46	0.02	0.00	-0.03	0.09
55336	FBXL8	-0.04	0.00	0.00	0.07
55337	FLJ11286	0.00	0.00	0.00	NaN
55350	VNN3	-0.02	0.00	0.11	0.11
55355	DKFZp762E13	0.00	0.00	0.00	NaN
55357	TBC1D2	-0.02	0.00	0.00	0.07
55361	PI4KII	-0.02	0.00	-0.03	0.06
55364	IMPACT	0.00	-0.09	0.03	0.05
55367	LRDD	0.00	0.09	0.00	0.17
55388	MCM10	0.02	0.00	0.14	0.27
55466	DNAJA4	0.00	0.00	-0.03	-0.02
55471	PRO1853	0.00	0.00	0.00	NaN
55486	PARL	0.04	0.00	0.00	0.32
55505	NOLA3	-0.02	0.00	-0.16	0.20
55506	H2AFY2	-0.02	0.00	0.00	0.07
55507	GPRC5D	0.00	-0.09	0.05	0.04
55509	SNFT	0.07	0.00	0.00	0.11
55520	ELAC1	-0.02	-0.09	-0.05	0.09
55521	TRIM36	0.00	0.00	-0.05	-0.01
55554	KLK15	0.00	0.00	-0.03	0.07
55576	STAB2	0.00	0.00	0.00	NaN
55577	NAGK	0.02	0.00	0.00	0.05
55584	CHRNA9	-0.04	0.00	-0.08	0.09
55601	FLJ20035	-0.02	0.00	-0.05	0.16
55607	PPP1R9A	0.02	0.00	-0.03	0.03
55612	C20orf42	0.00	0.00	-0.05	-0.02
55625	ZDHHC7	-0.09	0.00	0.00	0.28
55626	FLJ20294	-0.02	0.00	-0.03	0.29
55630	SLC39A4	0.15	0.00	0.30	0.14
55638	FLJ20366	0.18	0.09	0.27	-0.01
55643	BTBD2	0.00	0.00	-0.03	0.09
55644	OSGEP	-0.02	0.00	-0.03	0.15
55647	RAB20	0.00	0.00	-0.03	0.10
55651	NOLA2	0.00	0.00	0.00	NaN
55652	FLJ20489	0.00	0.00	0.00	NaN

55653	BCAS4	0.07	0.09	0.05	0.40
55661	DDX27	0.07	0.00	0.03	0.22
55662	HIF1AN	-0.02	0.00	-0.03	0.05
55665	URG4	-0.02	0.00	0.05	0.14
55669	MFN1	0.04	0.00	0.03	0.38
55683	FLJ10081	0.00	0.00	0.00	NaN
55692	LUC7L	0.05	0.09	0.03	0.22
55701	FLJ10357	-0.02	0.00	0.00	0.12
55713	ZNF334	0.02	0.00	0.03	0.00
55715	DOK4	-0.05	0.00	0.00	0.16
55734	ZFP64	0.09	0.00	0.05	0.34
55737	VPS35	-0.11	0.09	0.03	0.37
55739	FLJ10769	0.00	0.00	-0.03	0.31
55742	PARVA	0.00	0.00	-0.05	0.06
55743	CHFR	0.00	0.00	0.00	NaN
55746	NUP133	0.07	0.00	0.03	0.12
55757	UGCGL2	-0.02	0.00	-0.05	0.00
55759	WDR12	0.02	0.00	0.00	0.19
55766	H2AFJ	0.00	-0.09	0.05	-0.07
55769	ZNF83	0.00	0.00	-0.03	0.27
55775	TDP1	-0.02	0.00	0.03	0.39
55790	ChGn	-0.15	-0.18	-0.22	0.08
55794	DDX28	-0.05	0.00	0.00	0.17
55799	CACNA2D3	-0.04	0.00	-0.03	0.00
55801	IL26	0.02	0.00	0.00	0.11
55803	CENTA2	0.02	0.00	0.00	0.07
55806	HR	-0.13	-0.18	-0.22	-0.06
55811	SAC	0.04	0.00	0.05	0.04
55821	ALLC	-0.02	0.00	0.00	0.14
55823	VPS11	-0.05	0.00	0.00	0.08
55835	CENPJ	-0.05	0.00	-0.08	-0.08
55850	MDS032	0.04	0.00	0.00	0.16
55857	C20orf19	0.00	0.00	-0.05	0.08
55859	BEX1	-0.05	0.00	-0.08	0.01
55867	SLC22A11	0.00	-0.09	0.03	-0.06
55879	GABRQ	-0.02	0.00	-0.11	0.04
55890	GPRC5C	0.04	0.27	0.08	0.31
55891	LENEP	0.09	0.00	0.14	0.08
55892	MYNN	0.04	0.00	0.05	0.25
55900	ZNF302	0.00	0.00	0.05	-0.04
55905	ZNF313	0.05	0.00	0.05	0.39
55907	CMAS	0.00	-0.09	0.05	0.03
55909	BIN3	-0.13	-0.18	-0.22	0.23
55914	ERBB2IP	0.00	0.00	-0.11	0.15
55915	LANCL2	0.00	0.00	0.08	0.61
55930	MYO5C	-0.02	0.00	-0.05	0.35
55957	F25965	0.02	0.09	0.05	-0.04

55968	NSFL1C	0.02	0.00	-0.03	0.12
55969	C20orf24	0.02	0.00	0.00	0.21
56000	NXF3	-0.05	0.00	-0.08	-0.05
56006	FLJ12886	-0.02	0.00	-0.03	-0.19
56033	BARX1	-0.02	0.00	0.00	-0.02
56034	PDGFC	-0.02	0.00	-0.03	0.03
56061	UBPH	0.02	0.00	0.00	0.09
56062	KLHL4	-0.07	0.00	-0.08	-0.09
56114	PCDHGA1	0.00	0.00	0.00	NaN
56123	PCDHB13	0.00	0.00	0.00	NaN
56124	PCDHB12	0.00	0.00	0.00	NaN
56125	PCDHB11	0.00	0.00	0.00	NaN
56130	PCDHB6	0.00	0.00	0.00	NaN
56132	PCDHB3	0.00	0.00	0.00	NaN
56154	TEX15	-0.09	-0.18	-0.19	0.04
56155	TEX14	0.18	0.00	0.03	0.06
56156	TEX13B	-0.05	0.00	-0.08	-0.11
56158	TEX12	-0.07	0.00	0.00	-0.07
56159	TEX11	-0.04	0.00	-0.08	0.03
56163	RNF17	-0.05	0.00	-0.08	0.03
56165	TDRD1	-0.04	0.00	0.03	-0.01
56171	DNAH7	0.02	0.00	0.00	0.06
56172	ANKH	0.02	0.18	0.00	0.12
56242	ZNF253	0.02	0.00	0.00	0.09
56244	BTNL2	0.00	0.00	0.05	-0.28
56245	C21orf62	0.00	0.00	-0.03	-0.10
56253	CRTAM	-0.04	0.00	-0.03	0.18
56288	PARD3	-0.02	0.00	0.03	0.27
56302	TRPV5	0.02	0.00	0.00	0.04
56340	PPP4R2	-0.05	0.00	-0.03	0.26
56342	PPAN	0.00	0.00	0.00	NaN
56344	CABP5	-0.02	0.00	-0.03	-0.18
56474	CTPS2	-0.05	0.00	-0.11	0.04
56547	MMP26	-0.02	-0.09	-0.03	0.05
56548	CHST7	-0.05	0.00	-0.11	-0.06
56606	SLC2A9	-0.02	0.00	0.00	0.02
56647	BCCIP	-0.04	0.00	0.03	0.19
56648	EIF5A2	0.05	0.00	0.05	-0.04
56659	KCNK13	-0.02	0.00	0.03	0.05
56660	KCNK12	0.02	0.00	0.00	-0.09
56672	C11orf17	0.00	0.00	-0.08	0.16
56673	C11orf16	0.00	0.00	-0.08	-0.08
56676	ASCL3	0.00	0.00	-0.08	-0.14
56683	C21orf59	0.00	0.00	-0.03	0.07
56731	SLC2A4RG	0.05	0.00	0.08	0.28
56848	SPHK2	0.00	0.00	-0.03	0.10
56886	UGCGL1	-0.02	0.00	0.00	0.17

56890	MDM1	0.02	0.09	0.00	0.24
56892	C8orf4	0.09	0.18	0.08	0.02
56894	AGPAT3	0.00	0.00	0.00	0.24
56904	SH3GLB2	0.02	0.00	0.03	0.21
56910	STARD7	0.00	0.00	0.00	NaN
56911	C21orf7	-0.02	0.00	0.05	0.00
56913	C1GALT1	0.00	0.00	0.03	0.11
56914	OTOR	0.00	0.00	-0.08	-0.09
56922	MCCC1	0.04	0.00	0.00	0.14
56924	PAK6	-0.02	0.00	-0.14	0.02
56929	FEM1A	0.00	0.00	0.00	NaN
56937	TMEPAI	0.09	0.00	0.05	0.23
56938	ARNTL2	0.00	-0.09	0.03	0.15
56945	MRPS22	0.02	0.00	0.00	0.34
56953	NT5M	-0.07	0.00	0.00	0.13
56954	NIT2	0.00	0.00	0.00	0.36
56955	MEPE	-0.04	0.00	-0.03	0.11
56978	PRDM8	-0.04	0.00	-0.03	-0.13
56979	PRDM9	0.02	0.18	0.00	-0.04
56980	PRDM10	-0.05	0.00	-0.03	0.19
56981	PRDM11	-0.02	0.00	-0.03	0.00
56993	TOMM22	-0.04	0.00	-0.03	0.20
56994	CHPT1	0.00	0.00	0.03	0.27
56997	CABC1	0.07	0.00	0.03	0.16
56998	CTNNBIP1	-0.02	0.00	0.00	0.25
56999	ADAMTS9	-0.05	0.00	0.00	0.07
57016	AKR1B10	0.02	0.00	0.00	0.01
57020	MGC16824	0.02	-0.09	0.00	0.14
57030	SLC17A7	0.02	0.00	-0.03	-0.22
57047	PLSCR2	0.02	0.00	0.00	-0.23
57048	PLSCR3	-0.05	0.00	0.00	0.19
57050	SAS10	-0.02	0.00	0.00	0.06
57053	CHRNA10	-0.02	0.00	-0.03	-0.04
57060	PCBP4	0.00	0.00	0.00	NaN
57062	DDX24	-0.02	0.00	0.00	0.18
57084	SLC17A6	0.00	0.00	-0.05	-0.01
57088	PLSCR4	0.02	0.00	0.00	0.04
57091	C20orf32	0.04	0.00	0.05	0.13
57096	RPGRIP1	-0.02	0.00	0.00	0.07
57102	C12orf4	-0.02	0.09	0.08	0.07
57103	C12orf5	-0.02	0.09	0.08	0.27
57110	HRASLS	0.02	0.09	0.00	0.26
57111	RAB25	0.09	0.00	0.14	0.18
57119	SPINLW1	0.02	0.00	0.03	0.04
57122	NUP107	0.02	0.09	0.00	0.64
57127	RHBG	0.04	0.00	0.11	0.00
57139	RGL3	0.00	0.00	0.00	NaN

57140	RNPEPL1	0.00	0.00	0.00	NaN
57142	RTN4	0.00	0.00	0.00	NaN
57144	PAK7	0.00	0.00	-0.05	0.12
57148	KIAA1219	0.02	0.00	0.03	0.24
57154	SMURF1	0.02	0.00	0.00	0.08
57158	JPH2	0.02	0.09	0.03	0.01
57161	PELI2	0.00	0.00	0.00	-0.09
57162	PELI1	0.02	0.00	0.00	0.01
57172	CAMK1G	0.07	0.00	0.00	-0.01
57192	MCOLN1	0.00	-0.09	0.00	-0.09
57198	ATP8B2	0.05	0.00	0.14	-0.08
57205	ATP10D	-0.04	0.00	-0.03	0.39
57213	C13orf1	-0.07	0.00	-0.11	0.00
57214	KIAA1199	0.00	0.00	-0.03	-0.08
57231	SNX14	-0.02	0.00	-0.05	-0.15
57282	SLC4A10	-0.02	0.00	0.03	0.13
57332	CBX8	0.04	0.09	0.00	0.32
57336	ZNF287	-0.07	0.00	-0.03	0.01
57337	SENP7	0.00	0.00	-0.03	0.18
57338	JPH3	-0.07	0.00	0.00	0.03
57343	ZNF304	0.00	0.09	0.00	0.30
57348	TTYH1	0.00	0.09	-0.03	-0.05
57369	CX36	-0.02	0.00	-0.16	-0.19
57379	AICDA	0.00	-0.09	0.05	-0.13
57380	MRS2L	0.00	0.00	0.03	0.18
57403	RAB22A	0.07	0.09	0.05	0.33
57418	WDR18	0.00	0.00	-0.03	-0.01
57419	SLC24A3	0.00	0.00	-0.08	0.17
57446	NDRG3	0.04	0.00	0.00	0.18
57447	NDRG2	-0.02	0.00	0.00	-0.07
57468	SLC12A5	0.02	0.00	0.03	0.06
57498	KIDINS220	-0.02	0.00	0.00	0.00
57513	CASKIN2	0.05	0.27	0.03	0.36
57535	KIAA1324	0.00	0.00	-0.03	0.12
57556	SEMA6A	0.00	0.00	-0.03	0.11
57579	KIAA1411	0.00	0.00	0.00	0.00
57586	SYT13	-0.02	0.00	-0.03	-0.03
57591	MKL1	-0.02	0.00	-0.03	0.17
57596	KIAA1446	0.02	0.00	0.03	-0.03
57604	KIAA1456	-0.16	-0.18	-0.22	0.17
57634	EP400	0.00	0.00	0.00	NaN
57663	USP29	0.02	0.09	0.00	-0.11
57664	PLEKHA4	0.00	0.00	-0.03	0.06
57698	KIAA1598	-0.04	0.00	0.00	-0.01
57707	KIAA1609	-0.09	0.00	0.00	0.21
57712	TRPM3	-0.02	0.00	-0.03	0.03
57715	SEMA4G	-0.02	0.00	-0.03	0.01

57716	PRX	0.00	0.09	0.03	0.08
57718	KIAA1622	-0.02	0.00	0.00	0.06
57720	GPR107	0.02	0.00	0.00	0.12
57731	SPTBN4	0.00	0.09	0.03	0.05
57763	ANKRA2	0.00	0.00	-0.11	0.21
57786	RBAK	0.00	0.00	0.03	-0.10
57799	RAB40C	0.04	0.18	0.05	0.07
57804	POLD4	0.09	0.09	0.03	0.18
57817	HAMP	0.02	0.00	0.05	0.23
57834	CYP4F11	0.00	0.00	0.00	NaN
57835	SLC4A5	0.00	0.00	0.00	NaN
58155	PTBP2	-0.02	0.00	-0.03	0.12
58157	NGB	-0.02	0.00	-0.03	0.15
58158	NEUROD4	-0.02	0.00	-0.05	-0.03
58189	WFDC1	-0.09	0.00	0.00	0.10
58472	SQRDL	-0.04	0.00	-0.11	0.23
58478	MASA	-0.04	0.00	-0.03	0.12
58488	PCTP	0.13	0.09	0.03	0.44
58494	JAM2	0.00	0.00	0.03	0.04
58503	PROL1	-0.02	0.00	0.00	-0.02
58529	MYOZ1	0.04	0.00	0.00	-0.08
58533	SNX6	0.02	0.09	0.00	0.31
58986	TMEM8	0.11	0.18	0.11	0.16
59067	IL21	0.00	0.00	-0.03	-0.06
59269	HIVEP3	0.00	0.00	0.00	NaN
59272	ACE2	-0.05	0.00	-0.14	-0.01
59286	UBL5	0.00	0.00	0.00	NaN
59307	SIGIRR	0.00	0.09	0.05	-0.10
59335	PRDM12	0.02	0.00	0.03	-0.07
59336	PRDM13	0.00	0.00	0.00	-0.08
59338	PLEKHA1	-0.04	0.00	-0.03	0.02
59340	HRH4	0.00	-0.09	0.03	0.06
59341	TRPV4	0.00	0.00	0.00	NaN
59343	SENP2	0.04	0.00	0.00	0.28
59344	ALOXE3	-0.05	0.00	0.00	0.05
59347	FKSG2	0.13	0.18	0.05	0.07
60312	AFAP	-0.02	0.00	0.00	-0.09
60314	C12orf10	0.02	0.00	-0.03	0.20
60385	TSKS	0.02	0.00	-0.03	-0.12
60436	TGIF2	0.02	0.00	0.00	0.12
60468	BACH2	-0.04	0.00	0.00	0.07
60482	SLC5A7	0.00	0.00	0.00	NaN
60488	MRPS35	-0.02	-0.09	0.03	0.16
60491	NIF3L1	0.00	0.00	0.00	NaN
60496	AASDHPPT	-0.07	-0.09	0.00	0.13
60506	NYX	-0.05	0.00	-0.11	-0.12
60509	FLJ21839	0.00	0.00	0.00	NaN

60528	ELAC2	-0.07	0.00	-0.03	0.10
60529	ALX4	-0.02	0.00	-0.03	-0.20
60598	KCNK15	0.02	0.09	0.03	0.25
60681	FKBP10	-0.04	0.18	0.00	0.10
60682	SMAP1	0.00	0.00	0.00	0.36
60684	FLJ12716	-0.02	0.00	-0.05	0.33
63826	SRR	-0.05	0.00	0.00	0.05
63827	BCAN	0.04	0.00	0.11	0.02
63875	MRPL17	-0.02	0.00	-0.05	0.11
63876	PKNOX2	-0.05	0.00	-0.03	0.11
63910	C20orf59	0.04	0.09	0.08	0.06
63916	ELMO2	0.02	0.00	0.03	0.19
63925	ZNF335	0.04	0.00	0.03	0.13
63926	ANKRD5	0.00	0.00	-0.05	0.16
63931	MRPS14	0.00	0.00	0.03	0.36
63935	C20orf67	0.04	0.00	0.03	0.05
63941	APBA2BP	0.00	0.00	0.00	NaN
63943	FKBPL	0.00	0.00	0.05	0.14
63970	P53AIP1	-0.05	0.00	-0.03	-0.03
63971	KIF13A	0.00	0.00	0.08	0.13
63974	NEUROD6	-0.02	0.09	-0.03	-0.13
63976	PRDM16	-0.02	0.00	0.05	0.10
63978	PRDM14	0.15	0.00	0.14	0.13
64063	PRSS22	0.04	0.09	0.00	0.08
64066	MMP27	-0.07	-0.09	0.00	0.23
64067	NPAS3	0.02	0.09	0.03	-0.04
64077	LHPP	-0.04	0.00	0.03	0.18
64078	SLC28A3	-0.02	0.00	0.00	0.05
64083	GOLPH3	0.02	0.18	0.03	0.37
64084	CLSTN2	0.02	0.00	0.03	0.16
64087	MCCC2	0.00	0.00	-0.11	0.28
64089	SNX16	0.13	0.00	0.14	-0.02
64092	SAMSN1	-0.05	-0.18	0.05	0.26
64096	GFRA4	0.02	0.00	-0.03	-0.03
64100	ELSPBP1	-0.02	0.00	-0.03	-0.19
64110	MAGEF1	0.04	0.00	0.00	0.19
64122	FN3K	-0.02	0.09	0.08	0.03
64132	XYLT2	0.13	0.18	0.03	0.39
64137	ABCG4	-0.05	0.00	0.00	0.27
64170	CARD9	0.02	0.00	0.03	-0.03
64173	SPATA1	-0.02	0.00	-0.03	0.00
64210	MMS19L	-0.02	0.00	-0.03	-0.04
64211	LHX5	0.02	0.00	0.00	-0.03
64216	TFB2M	0.07	0.00	0.03	0.11
64219	PJA1	-0.04	0.00	-0.05	0.17
64223	GBL	0.04	0.09	0.00	0.15
64231	MS4A6A	-0.04	0.00	0.00	0.09

64232	MS4A5	-0.04	0.00	0.03	0.03
64240	ABCG5	0.00	0.00	0.00	NaN
64284	RAB17	0.02	0.00	0.00	0.15
64320	RNF25	0.00	0.00	0.00	NaN
64321	SOX17	0.09	0.00	0.14	0.13
64324	NSD1	0.00	0.00	0.00	NaN
64328	XPO4	-0.07	0.00	-0.11	0.04
64374	SIL1	0.00	0.00	-0.03	-0.02
64377	CHST8	0.00	0.00	0.05	-0.26
64386	MMP25	0.04	0.00	0.00	-0.03
64397	ZFP106	-0.05	0.09	-0.14	0.33
64398	MPP5	-0.02	0.00	0.00	0.07
64400	FTS	-0.05	0.00	0.00	0.23
64405	CDH22	0.02	0.00	0.03	-0.05
64446	DNAI2	0.04	0.27	0.08	0.10
64506	CPEB1	0.00	0.00	-0.03	-0.05
64577	ALDH8A1	-0.02	0.00	0.16	0.26
64579	NDST4	-0.02	0.00	0.00	0.25
64600	PLA2G2F	0.00	0.00	0.00	NaN
64601	VPS16	0.02	0.00	-0.03	0.29
64699	TMPRSS3	0.00	0.00	0.00	0.06
64708	COPS7B	0.00	0.00	0.00	NaN
64750	SMURF2	0.16	0.09	0.03	0.35
64760	RAI16	-0.13	-0.18	-0.22	0.12
64772	FLJ21865	0.02	0.18	0.03	0.20
64783	RBM15	0.00	0.00	-0.03	-0.04
64794	DDX31	0.02	0.00	0.00	-0.08
64800	FLJ23588	-0.02	0.00	-0.03	0.05
64816	CYP3A43	0.02	0.00	0.00	0.10
64834	ELOVL1	0.02	0.00	0.00	0.27
64849	SLC13A3	0.02	0.00	0.03	0.16
64850	AGXT2L1	-0.02	0.00	0.00	-0.02
64858	DCLRE1B	0.00	0.00	-0.03	-0.03
64895	PAPOLG	0.02	0.00	0.00	0.00
64901	RANBP17	0.00	0.00	-0.03	0.04
64919	BCL11B	-0.02	0.00	-0.03	0.08
64960	MRPS15	-0.02	0.00	0.00	0.01
64963	MRPS11	0.00	0.00	-0.05	0.02
64981	MRPL34	0.04	0.00	0.00	0.24
65003	MRPL11	0.07	0.00	0.03	0.24
65005	MRPL9	0.07	0.00	0.19	0.30
65009	NDRG4	-0.07	0.00	0.00	0.16
65010	SLC26A6	-0.04	0.00	0.00	0.05
65012	SLC26A10	0.02	0.00	0.00	0.06
65018	PINK1	0.00	0.00	0.00	NaN
65080	MRPL44	0.00	0.00	0.00	NaN
65082	VPS33A	0.02	0.00	0.00	-0.02

65095	FLJ12949	0.02	0.00	0.00	0.24
65109	UPF3B	-0.05	0.00	-0.08	0.15
65110	UPF3A	0.00	0.00	0.00	0.22
65117	FLJ11021	0.02	0.00	0.00	0.27
65250	FLJ13231	0.04	0.18	0.00	0.21
65258	MPPE1	-0.02	-0.09	0.00	0.19
65992	C20orf116	0.02	0.00	-0.03	0.14
65993	MRPS34	0.04	0.18	0.00	0.27
66002	CYP4F12	0.00	0.00	0.00	NaN
66035	SLC2A11	-0.02	0.00	0.03	0.22
66036	MTMR8	-0.04	0.00	-0.05	-0.09
78988	MRP63	-0.07	0.00	-0.11	0.19
79033	PRNPIP	0.00	0.00	0.03	0.29
79054	TRPM8	0.00	0.00	0.00	NaN
79058	ASPSCR1	0.00	0.09	0.08	0.00
79083	MLPH	0.02	0.00	0.00	0.14
79092	CARD14	0.00	0.00	-0.03	0.05
79095	C9orf16	0.02	0.00	0.00	0.24
79132	LGP2	-0.04	0.09	0.00	0.09
79133	C20orf7	0.00	0.00	-0.05	-0.08
79143	LENG4	0.00	0.09	0.00	0.43
79147	FKRP	-0.04	0.00	-0.03	-0.07
79148	MMP28	-0.02	0.00	0.03	0.04
79154	MGC4172	0.00	0.09	0.03	0.20
79157	ET	0.04	0.00	0.03	0.08
79171	MGC10433	0.00	0.09	0.05	-0.23
79183	C20orf121	0.02	0.09	0.03	0.18
79187	FSD1	0.00	0.00	0.00	NaN
79365	BHLHB3	0.00	-0.09	0.08	0.20
79366	NSBP1	-0.04	0.00	-0.08	0.02
79369	B3GNT4	0.02	0.00	0.00	-0.37
79400	NOX5	0.02	0.00	-0.05	0.06
79442	LRRC2	-0.04	0.00	0.00	0.16
79443	FYCO1	-0.04	0.00	0.00	0.01
79584	FLJ12684	-0.02	0.00	0.00	0.02
79587	FLJ12118	0.00	0.00	-0.03	-0.04
79590	MRPL24	0.04	0.00	0.11	0.16
79600	FLJ21127	0.00	0.00	0.00	NaN
79611	FLJ21963	0.00	0.00	0.00	NaN
79640	FLJ23584	-0.02	0.00	-0.03	0.16
79657	FLJ21908	0.00	0.00	0.00	NaN
79661	NEIL1	0.00	0.00	0.00	NaN
79701	FLJ22222	-0.02	0.09	0.08	0.09
79703	FLJ22531	0.07	0.09	0.03	0.21
79716	NPEPL1	0.05	0.09	0.03	0.36
79719	FLJ11506	0.00	0.00	-0.05	0.09
79723	SUV39H2	-0.02	0.00	0.14	0.09

79740	FLJ23049	0.02	0.00	0.00	0.12
79753	SNIP1	0.00	0.00	0.00	NaN
79754	ASB13	0.00	0.00	0.14	0.17
79767	ELMO3	-0.04	0.00	0.00	-0.05
79783	C7orf10	-0.02	0.00	0.03	0.07
79785	FLJ22655	0.00	-0.09	0.05	-0.09
79786	C16orf44	-0.09	0.00	0.00	0.28
79800	ALS2CR8	0.02	0.00	0.00	0.10
79814	AGMAT	-0.02	0.00	0.00	0.29
79833	GEMIN6	0.00	0.00	0.00	NaN
79846	FLJ21062	0.02	0.00	0.00	0.13
79858	NEK11	0.00	0.00	0.00	NaN
79869	FLJ12529	-0.02	0.09	0.05	0.31
79870	BAALC	0.22	0.09	0.32	0.12
79887	FLJ22662	0.00	-0.09	0.05	0.03
79890	RIN3	-0.02	0.00	0.03	0.29
79899	FLJ14213	0.04	0.00	-0.03	-0.10
79903	FLJ14154	0.04	0.00	0.00	0.19
79912	FLJ22028	0.00	-0.09	0.05	0.30
79917	FLJ21687	-0.04	0.00	-0.05	0.02
79919	FLJ22671	0.00	0.00	0.00	NaN
79953	C20orf39	0.02	0.00	-0.03	0.19
79962	FLJ13236	0.00	0.00	-0.03	0.05
79974	FLJ21986	0.00	0.00	-0.03	-0.02
79984	FLJ21736	-0.05	0.00	0.00	0.05
80006	FLJ13611	0.00	0.00	-0.11	0.03
80011	NIP30	-0.05	0.00	0.00	0.14
80146	UXS1	0.00	0.00	0.00	NaN
80157	FLJ21511	-0.05	0.00	0.00	0.08
80196	RNF34	0.02	0.00	0.00	0.34
80198	MUS81	0.02	0.00	0.05	0.17
80207	OPA3	-0.02	0.00	0.00	-0.03
80221	FLJ20920	0.13	0.18	0.03	0.33
80271	ITPKC	0.00	0.00	0.00	-0.21
80324	PUS1	0.00	0.00	0.00	NaN
80328	ULBP2	0.00	0.00	-0.03	0.18
80329	ULBP1	0.00	0.00	-0.03	-0.26
80351	TNKS2	-0.02	0.00	-0.05	0.22
80700	UBXD1	0.00	0.00	0.00	NaN
80704	SLC19A3	0.00	0.00	0.00	NaN
80705	TSGA10	0.02	0.00	0.00	-0.02
80737	C6orf27	0.00	0.00	0.05	0.07
80765	STARD5	0.00	0.00	-0.03	-0.08
80776	MGC4093	0.02	0.00	-0.03	-0.07
80781	COL18A1	0.02	0.00	-0.03	0.22
80830	APOL6	-0.02	0.00	0.03	0.03
80831	APOL5	-0.02	0.00	0.03	0.04

80833	APOL3	-0.02	0.00	0.03	-0.09
80853	KIAA1718	0.02	0.00	0.00	0.07
80895	ILKAP	0.00	0.00	0.00	NaN
80975	TMPRSS5	-0.07	0.00	0.00	0.26
81025	GJA10	0.00	0.09	0.00	0.07
81027	TUBB1	0.05	0.09	0.03	0.04
81029	WNT5B	-0.02	0.09	0.08	0.14
81031	SLC2A10	0.02	0.00	0.03	0.13
81035	COLEC12	0.02	-0.09	0.00	0.11
81285	OR51E2	-0.02	0.00	-0.03	0.04
81490	PTDSS2	0.00	0.09	0.05	-0.06
81491	GPR63	0.00	0.09	-0.03	-0.02
81492	RSHL1	-0.02	0.00	0.00	-0.07
81539	SLC38A1	0.00	0.00	0.00	NaN
81543	LRRC3	0.00	0.00	0.00	0.04
81551	STMN4	-0.09	-0.18	-0.19	-0.05
81563	C1orf21	0.07	0.00	0.03	0.28
81566	C12orf22	0.02	0.00	-0.03	0.19
81578	COL21A1	-0.04	0.00	0.08	0.05
81620	CDT1	0.00	0.09	0.11	0.12
81622	UNC93B1	0.07	0.09	0.03	0.21
81623	DEFB126	0.02	0.00	-0.03	-0.07
81624	DIAPH3	-0.07	0.00	-0.08	0.01
81626	C1orf14	0.07	0.00	0.03	-0.12
81627	C1orf25	0.05	0.00	0.03	0.09
81691	LOC81691	0.02	0.00	0.00	0.13
81693	AMN	0.00	0.00	-0.03	0.00
81696	OR5V1	0.00	0.00	0.05	0.00
81792	ADAMTS12	0.02	0.27	0.00	-0.18
81797	OR12D3	0.00	0.00	0.05	-0.13
81831	NETO2	-0.11	0.09	0.03	0.18
81833	SPACA1	-0.02	0.00	-0.05	-0.01
81876	RAB1B	0.09	0.00	0.03	0.30
83442	SH3BGRL3	0.00	0.09	0.00	0.15
83452	RAB33B	0.00	0.00	0.00	NaN
83481	EPPK1	0.16	0.00	0.35	0.17
83483	PLVAP	0.04	0.00	0.00	0.06
83660	TLN2	0.02	0.00	-0.05	0.09
83737	ITCH	0.00	0.00	0.00	NaN
83988	NCALD	0.22	0.09	0.32	0.25
83990	BRIP1	0.16	0.18	0.03	0.12
84060	DKFZP564O05	0.02	0.00	0.08	0.50
84107	ZIC4	0.02	0.00	-0.03	-0.12
84148	MYST1	0.04	0.09	0.00	0.30
84561	SLC12A8	0.00	0.00	-0.03	-0.12
84612	PARD6B	0.07	0.09	0.05	0.25
84658	EMR3	0.02	0.00	0.00	0.05

84752	MGC4655	-0.04	0.00	0.00	0.11
84790	TUBA6	0.00	0.00	-0.03	0.02
84928	FLJ14803	0.02	0.00	0.00	0.08
85359	DGCR6L	-0.02	0.00	0.05	0.15
85363	TRIM5	-0.02	0.00	-0.03	-0.01
85477	SCIN	-0.02	0.00	0.00	-0.03
86722	GREB1	-0.02	0.00	0.00	-0.07
89781	HPS4	-0.05	-0.09	0.03	0.07
89870	TRIM15	0.00	0.00	0.05	-0.05
89874	SLC25A21	0.02	0.00	0.00	-0.02
89910	UBE3B	0.00	0.00	0.00	NaN
90634	CG018	-0.05	0.00	-0.08	0.15
90665	TBL1Y	0.00	0.00	-0.03	-0.16
91754	NEK9	-0.02	0.00	0.00	0.04
91949	COG7	0.02	0.00	0.00	0.16
91977	MYOZ3	0.00	0.00	0.00	NaN
94009	SERHL	0.00	0.00	-0.03	0.19
94104	C21orf66	0.00	0.00	-0.03	0.14
112399	EGLN3	0.04	0.09	0.00	0.04
113791	MGC17330	0.00	0.00	0.05	0.15
114049	WBSCR22	0.02	0.00	0.05	0.22
114088	TRIM9	0.02	0.00	0.00	-0.03
114625	ERMAP	0.02	0.00	0.00	0.04
114876	OSBPL1A	0.00	-0.09	0.03	0.10
114881	OSBPL7	0.02	0.18	0.00	0.32
114882	OSBPL8	-0.02	0.00	0.00	0.12
114883	OSBPL9	0.00	0.00	-0.03	-0.04
114884	OSBPL10	0.02	0.00	-0.05	0.23
114885	OSBPL11	0.00	0.00	-0.03	0.19
114897	C1QTNF1	0.02	0.18	0.03	0.21
114899	C1QTNF3	0.02	0.27	0.00	0.13
116039	OSR2	0.16	0.18	0.27	-0.01
116832	RPL39L	0.04	0.00	0.03	0.28
116984	CENTD1	-0.05	-0.09	-0.05	0.09
116985	CENTD2	0.00	0.09	0.00	0.34
116986	CENTG1	0.02	0.09	0.00	-0.14
116987	CENTG2	0.00	0.00	0.00	NaN
117246	FTSJ3	0.16	0.27	0.03	0.64
117247	SLC16A10	0.02	0.00	0.05	0.07
140545	RNF32	0.02	0.00	-0.03	0.01
140803	TRPM6	-0.02	0.00	0.00	0.00
142684	RAB40A	-0.05	0.00	-0.08	-0.12
154796	AMOT	-0.05	0.00	-0.08	0.06
171558	PTCRA	0.00	0.00	0.08	0.00
192683	SCAMP5	0.00	0.00	0.00	NaN

Chin et al. dataset

Gene_no	Gene_name	Average_CNA_Score_ER	Average_CNA_Score_HER2	Average_CNA_Score_TN	Overall_Correlation
2	A2M	0.02	0.00	0.15	-0.05
9	NAT1	0.00	0.00	0.00	NaN
10	NAT2	0.00	0.00	0.00	NaN
12	SERPINA3	0.00	0.00	0.00	NaN
13	AADAC	0.00	0.00	0.00	NaN
14	AAMP	0.04	0.00	0.00	0.06
15	AANAT	0.00	0.14	0.05	0.47
16	AARS	0.02	0.00	0.00	0.05
18	ABAT	0.00	0.00	0.00	NaN
19	ABCA1	0.02	0.00	0.10	0.35
21	ABCA3	0.18	0.00	0.10	0.12
23	ABCF1	0.04	0.14	0.05	0.09
24	ABCA4	0.00	0.00	0.00	NaN
25	ABL1	0.04	0.00	0.00	-0.06
26	ABP1	0.02	0.00	0.00	0.29
27	ABL2	0.04	0.00	0.10	0.05
28	ABO	0.04	0.00	0.00	-0.08
29	ABR	0.02	0.00	0.00	0.11
30	ACAA1	0.00	0.00	0.15	0.22
31	ACACA	0.06	0.00	0.00	-0.01
32	ACACB	0.02	0.00	0.00	-0.18
33	ACADL	0.02	0.00	0.05	0.00
34	ACADM	0.02	0.00	0.05	-0.20
35	ACADS	0.02	0.00	0.10	0.10
36	ACADSB	0.04	0.00	0.05	-0.07
37	ACADVL	0.00	0.00	0.00	NaN
38	ACAT1	0.02	0.00	0.00	0.08
39	ACAT2	0.04	0.00	0.05	-0.02
40	ACCN1	0.04	0.00	0.05	-0.08
41	ACCN2	0.02	0.00	0.00	0.08
43	ACHE	0.00	0.00	0.00	NaN
47	ACLY	0.02	0.86	0.05	0.24
48	ACO1	0.00	0.00	0.15	0.11
50	ACO2	0.00	0.14	0.00	-0.09
51	ACOX1	0.02	0.14	0.05	0.32
52	ACP1	0.20	0.14	0.35	0.13
53	ACP2	0.02	0.00	0.00	-0.15
54	ACP5	0.02	0.00	0.00	0.05
55	ACPP	0.00	0.00	0.10	-0.12
56	ACRV1	0.02	0.00	0.00	0.02
58	ACTA1	0.00	0.00	0.05	0.00
59	ACTA2	0.02	0.00	0.05	0.04
60	ACTB	0.00	0.00	0.00	NaN

71	ACTG1	0.00	0.00	0.00	NaN
72	ACTG2	0.00	0.00	0.05	-0.06
81	ACTN4	0.00	0.00	0.00	NaN
87	ACTN1	0.02	0.00	0.00	0.12
88	ACTN2	0.00	0.00	0.00	NaN
89	ACTN3	0.04	0.00	0.05	0.07
90	ACVR1	0.00	0.00	0.00	NaN
91	ACVR1B	0.00	0.00	0.00	NaN
93	ACVR2B	0.02	0.00	0.05	-0.07
94	ACVRL1	0.00	0.00	0.00	NaN
95	ACY1	0.00	0.00	0.10	-0.03
97	ACYP1	0.00	0.00	0.00	NaN
98	ACYP2	0.02	0.00	0.05	0.08
100	ADA	0.02	0.00	0.00	-0.12
101	ADAM8	0.00	0.00	0.00	NaN
102	ADAM10	0.00	0.00	0.00	NaN
103	ADAR	0.04	0.00	0.15	0.12
104	ADARB1	0.00	0.00	0.05	0.10
107	ADCY1	0.00	0.00	0.00	NaN
108	ADCY2	0.00	0.00	0.00	NaN
109	ADCY3	0.00	0.00	0.00	NaN
112	ADCY6	0.02	0.00	0.00	0.06
113	ADCY7	0.08	0.00	0.05	0.02
114	ADCY8	0.10	0.14	0.00	-0.06
115	ADCY9	0.00	0.00	0.00	NaN
116	ADCYAP1	0.00	0.00	0.00	NaN
117	ADCYAP1R1	0.00	0.00	0.00	NaN
118	ADD1	0.00	0.29	0.00	0.08
119	ADD2	0.00	0.00	0.05	0.00
120	ADD3	0.00	0.14	0.00	0.02
123	ADFP	0.02	0.00	0.05	-0.05
124	ADH1A	0.00	0.00	0.00	NaN
125	ADH1B	0.00	0.00	0.00	NaN
126	ADH1C	0.00	0.00	0.00	NaN
128	ADH5	0.00	0.00	0.00	NaN
130	ADH6	0.00	0.00	0.00	NaN
131	ADH7	0.00	0.00	0.00	NaN
132	ADK	0.02	0.00	0.00	0.23
133	ADM	0.00	0.00	0.00	NaN
134	ADORA1	0.00	0.00	0.00	NaN
135	ADORA2A	0.00	0.14	0.05	-0.12
136	ADORA2B	0.00	0.00	0.00	NaN
140	ADORA3	0.00	0.00	0.00	NaN
141	ADPRH	0.00	0.00	0.10	0.10
146	ADRA1D	0.00	0.00	0.05	-0.13
147	ADRA1B	0.00	0.00	0.00	NaN
148	ADRA1A	0.02	0.00	0.00	0.10

150	ADRA2A	0.00	0.14	0.00	0.07
151	ADRA2B	0.04	0.00	0.05	-0.13
152	ADRA2C	0.00	0.00	0.00	NaN
153	ADRB1	0.04	0.00	0.05	-0.02
154	ADRB2	0.02	0.14	0.00	-0.07
155	ADRB3	0.12	0.14	0.05	-0.12
156	ADRBK1	0.04	0.00	0.00	0.58
157	ADRBK2	0.00	0.14	0.05	0.03
158	ADSL	0.00	0.14	0.00	0.07
161	AP2A2	0.02	0.00	0.05	-0.03
162	AP1B1	0.00	0.14	0.05	-0.01
163	AP2B1	0.06	0.00	0.00	0.25
164	AP1G1	0.00	0.00	0.05	0.03
165	AEBP1	0.00	0.00	0.00	NaN
166	AES	0.00	0.00	0.00	NaN
173	AFM	0.02	0.00	0.00	0.01
174	AFP	0.02	0.00	0.00	0.04
175	AGA	0.00	0.00	0.00	NaN
177	AGER	0.02	0.00	0.05	0.14
178	AGL	0.02	0.00	0.00	-0.09
181	AGRP	0.02	0.00	0.00	0.02
182	JAG1	0.00	0.00	0.05	0.63
183	AGT	0.00	0.00	0.05	-0.07
185	AGTR1	0.00	0.00	0.00	NaN
189	AGXT	0.08	0.43	0.10	0.03
191	AHCY	0.02	0.00	0.05	-0.23
195	AHNAK	0.00	0.00	0.05	-0.11
196	AHR	0.02	0.00	0.05	0.01
197	AHSG	0.02	0.14	0.00	-0.04
199	AIF1	0.02	0.00	0.05	0.12
202	AIM1	0.04	0.00	0.00	0.20
203	AK1	0.02	0.00	0.00	-0.05
204	AK2	0.00	0.00	0.00	NaN
205	AK3	0.00	0.00	0.00	NaN
207	AKT1	0.02	0.00	0.10	-0.02
208	AKT2	0.06	0.00	0.05	0.03
210	ALAD	0.00	0.00	0.00	NaN
211	ALAS1	0.00	0.00	0.10	-0.10
214	ALCAM	0.02	0.14	0.05	-0.20
216	ALDH1A1	0.00	0.00	0.00	NaN
217	ALDH2	0.00	0.00	0.00	NaN
218	ALDH3A1	0.10	0.00	0.05	0.15
219	ALDH1B1	0.14	0.00	0.15	-0.15
220	ALDH1A3	0.16	0.00	0.10	-0.10
221	ALDH3B1	0.04	0.00	0.05	0.22
222	ALDH3B2	0.04	0.00	0.05	0.29
223	ALDH9A1	0.02	0.00	0.00	-0.24

224	ALDH3A2	0.02	0.00	0.00	0.11
225	ABCD2	0.00	0.00	0.35	-0.10
226	ALDOA	0.02	0.00	0.00	-0.07
229	ALDOB	0.04	0.00	0.00	0.22
230	ALDOC	0.10	0.00	0.05	0.16
231	AKR1B1	0.00	0.00	0.05	0.03
238	ALK	0.00	0.00	0.00	NaN
239	ALOX12	0.00	0.00	0.00	NaN
240	ALOX5	0.02	0.00	0.10	-0.03
241	ALOX5AP	0.04	0.14	0.00	-0.20
242	ALOX12B	0.00	0.00	0.00	NaN
244	ANXA8	0.02	0.00	0.10	0.04
246	ALOX15	0.04	0.00	0.10	-0.05
247	ALOX15B	0.00	0.00	0.00	NaN
248	ALPI	0.00	0.00	0.05	-0.01
249	ALPL	0.02	0.00	0.00	-0.05
250	ALPP	0.00	0.00	0.05	0.01
251	ALPPL2	0.00	0.00	0.05	-0.02
257	ALX3	0.00	0.00	0.00	NaN
258	AMBN	0.00	0.14	0.00	0.14
259	AMBP	0.00	0.00	0.00	NaN
262	AMD1	0.02	0.00	0.10	0.53
267	AMFR	0.02	0.00	0.00	-0.06
268	AMH	0.02	0.00	0.05	-0.16
269	AMHR2	0.00	0.00	0.00	NaN
270	AMPD1	0.08	0.00	0.10	0.02
271	AMPD2	0.00	0.00	0.00	NaN
272	AMPD3	0.00	0.00	0.00	NaN
273	AMPH	0.00	0.00	0.00	NaN
274	BIN1	0.00	0.00	0.10	0.12
275	AMT	0.06	0.00	0.05	0.21
276	AMY1A	0.06	0.00	0.20	-0.01
283	ANG	0.02	0.00	0.00	-0.03
284	ANGPT1	0.12	0.00	0.25	0.07
285	ANGPT2	0.02	0.14	0.10	0.01
286	ANK1	0.04	0.00	0.00	0.68
287	ANK2	0.00	0.00	0.05	-0.11
288	ANK3	0.06	0.00	0.05	0.01
290	ANPEP	0.00	0.00	0.00	NaN
291	SLC25A4	0.00	0.00	0.00	NaN
301	ANXA1	0.00	0.00	0.00	NaN
302	ANXA2	0.00	0.00	0.05	-0.19
306	ANXA3	0.00	0.00	0.00	NaN
307	ANXA4	0.00	0.14	0.00	0.10
308	ANXA5	0.00	0.14	0.05	0.05
309	ANXA6	0.00	0.14	0.00	0.22
310	ANXA7	0.00	0.00	0.00	NaN

311	ANXA11	0.06	0.14	0.05	0.23
312	ANXA13	0.08	0.00	0.00	-0.18
313	AOAH	0.02	0.00	0.00	-0.16
314	AOC2	0.02	0.86	0.05	0.09
316	AOX1	0.00	0.00	0.05	-0.02
317	APAF1	0.04	0.14	0.05	0.19
318	NUDT2	0.04	0.00	0.00	0.09
319	APOF	0.00	0.00	0.00	NaN
320	APBA1	0.02	0.00	0.00	0.02
321	APBA2	0.00	0.00	0.00	NaN
322	APBB1	0.00	0.00	0.00	NaN
323	APBB2	0.00	0.00	0.05	-0.13
324	APC	0.00	0.00	0.00	NaN
325	APCS	0.00	0.00	0.00	NaN
326	AIRE	0.02	0.00	0.00	-0.04
327	APEH	0.06	0.00	0.05	0.00
329	BIRC2	0.00	0.00	0.00	NaN
330	BIRC3	0.00	0.00	0.00	NaN
332	BIRC5	0.02	0.14	0.05	0.09
333	APLP1	0.00	0.00	0.00	NaN
334	APLP2	0.02	0.00	0.00	0.04
335	APOA1	0.02	0.00	0.00	-0.06
336	APOA2	0.00	0.00	0.00	NaN
337	APOA4	0.02	0.00	0.00	-0.06
338	APOB	0.00	0.00	0.00	NaN
339	APOBEC1	0.02	0.00	0.15	0.01
341	APOC1	0.00	0.00	0.00	NaN
343	AQP8	0.06	0.14	0.00	0.01
344	APOC2	0.00	0.00	0.00	NaN
345	APOC3	0.02	0.00	0.00	-0.08
346	APOC4	0.00	0.00	0.00	NaN
347	APOD	0.02	0.00	0.00	0.05
348	APOE	0.00	0.00	0.00	NaN
350	APOH	0.12	0.00	0.20	-0.08
351	APP	0.00	0.00	0.00	NaN
353	APRT	0.00	0.00	0.10	0.10
354	KLK3	0.00	0.00	0.00	NaN
358	AQP1	0.00	0.00	0.00	NaN
359	AQP2	0.02	0.00	0.00	-0.12
360	AQP3	0.04	0.00	0.00	-0.07
361	AQP4	0.00	0.00	0.10	-0.01
362	AQP5	0.02	0.00	0.00	-0.04
363	AQP6	0.02	0.00	0.00	0.06
364	AQP7	0.04	0.00	0.00	0.17
366	AQP9	0.00	0.00	0.00	NaN
368	ABCC6	0.00	0.00	0.00	NaN
372	ARCN1	0.00	0.00	0.00	NaN

374	AREG	0.00	0.00	0.05	-0.12
375	ARF1	0.00	0.00	0.05	0.14
377	ARF3	0.02	0.00	0.00	0.05
378	ARF4	0.02	0.00	0.00	-0.07
381	ARF5	0.02	0.00	0.00	0.03
382	ARF6	0.00	0.00	0.00	NaN
383	ARG1	0.02	0.00	0.00	-0.06
384	ARG2	0.00	0.00	0.05	0.33
392	ARHGAP1	0.02	0.00	0.00	0.33
394	ARHGAP5	0.02	0.00	0.00	0.25
396	ARHGDIA	0.00	0.00	0.00	NaN
397	ARHGDIB	0.00	0.00	0.00	NaN
398	ARHGDIG	0.18	0.00	0.10	0.04
400	ARL1	0.00	0.00	0.00	NaN
402	ARL2	0.02	0.00	0.00	-0.05
403	ARL3	0.02	0.00	0.00	-0.06
405	ARNT	0.02	0.00	0.10	0.10
406	ARNTL	0.00	0.00	0.00	NaN
408	ARRB1	0.02	0.14	0.00	0.04
409	ARRB2	0.04	0.00	0.10	0.12
411	ARSB	0.00	0.00	0.00	NaN
417	ART1	0.00	0.00	0.05	-0.09
419	ART3	0.00	0.00	0.00	NaN
421	ARVCF	0.06	0.14	0.15	-0.01
429	ASCL1	0.00	0.00	0.00	NaN
430	ASCL2	0.00	0.00	0.00	NaN
432	ASGR1	0.00	0.00	0.00	NaN
433	ASGR2	0.00	0.00	0.00	NaN
434	ASIP	0.02	0.00	0.05	-0.03
435	ASL	0.04	0.00	0.00	-0.01
439	ASNA1	0.06	0.14	0.00	0.12
440	ASNS	0.00	0.14	0.05	0.11
443	ASPA	0.04	0.00	0.10	-0.11
444	ASPH	0.02	0.00	0.00	-0.03
462	SERPINC1	0.04	0.00	0.00	0.04
466	ATF1	0.02	0.00	0.00	0.03
467	ATF3	0.02	0.00	0.15	-0.05
468	ATF4	0.00	0.14	0.00	0.05
471	ATIC	0.02	0.00	0.00	0.02
472	ATM	0.02	0.00	0.00	0.12
473	RERE	0.00	0.00	0.00	NaN
474	ATOH1	0.04	0.00	0.05	-0.06
475	ATOX1	0.00	0.14	0.00	0.08
476	ATP1A1	0.08	0.00	0.10	-0.02
477	ATP1A2	0.00	0.00	0.00	NaN
478	ATP1A3	0.00	0.00	0.05	0.11
479	ATP12A	0.06	0.00	0.00	0.14

481	ATP1B1	0.00	0.00	0.10	0.13
482	ATP1B2	0.00	0.00	0.00	NaN
483	ATP1B3	0.02	0.00	0.00	-0.20
486	FXYD2	0.00	0.00	0.00	NaN
487	ATP2A1	0.06	0.14	0.00	0.26
488	ATP2A2	0.02	0.00	0.00	-0.19
489	ATP2A3	0.04	0.00	0.10	0.00
490	ATP2B1	0.00	0.00	0.00	NaN
491	ATP2B2	0.02	0.00	0.05	0.29
493	ATP2B4	0.00	0.00	0.00	NaN
495	ATP4A	0.00	0.00	0.00	NaN
498	ATP5A1	0.02	0.00	0.00	0.00
501	ALDH7A1	0.00	0.00	0.00	NaN
506	ATP5B	0.00	0.00	0.00	NaN
513	ATP5D	0.02	0.00	0.05	0.04
514	ATP5E	0.12	0.00	0.00	0.27
515	ATP5F1	0.06	0.14	0.00	-0.07
516	ATP5G1	0.04	0.00	0.05	0.01
517	ATP5G2	0.00	0.00	0.00	NaN
518	ATP5G3	0.00	0.00	0.05	0.10
522	ATP5J	0.02	0.00	0.05	0.07
525	ATP6V1B1	0.00	0.00	0.05	-0.18
526	ATP6V1B2	0.00	0.00	0.00	NaN
527	ATP6V0C	0.02	0.00	0.00	0.13
528	ATP6V1C1	0.12	0.00	0.25	0.36
533	ATP6V0B	0.02	0.00	0.05	0.23
534	ATP6V1G2	0.04	0.14	0.05	-0.13
535	ATP6V0A1	0.02	0.86	0.05	-0.19
539	ATP5O	0.00	0.00	0.00	NaN
540	ATP7B	0.04	0.00	0.10	-0.16
545	ATR	0.02	0.00	0.00	-0.03
549	AUH	0.00	0.00	0.00	NaN
550	AUP1	0.00	0.00	0.05	-0.13
551	AVP	0.00	0.00	0.00	NaN
552	AVPR1A	0.04	0.00	0.00	-0.01
553	AVPR1B	0.02	0.00	0.05	-0.02
558	AXL	0.00	0.00	0.05	0.15
563	AZGP1	0.02	0.00	0.00	0.01
566	AZU1	0.06	0.00	0.15	0.02
567	B2M	0.02	0.00	0.05	0.25
570	BAAT	0.04	0.00	0.00	-0.09
571	BACH1	0.02	0.00	0.00	0.09
572	BAD	0.02	0.00	0.00	0.07
573	BAG1	0.04	0.00	0.00	0.03
575	BAI1	0.10	0.00	0.15	-0.14
576	BAI2	0.00	0.14	0.00	-0.06
577	BAI3	0.12	0.00	0.05	0.08

578	BAK1	0.02	0.00	0.05	0.15
580	BARD1	0.02	0.00	0.00	0.11
581	BAX	0.06	0.00	0.05	-0.01
585	BBS4	0.00	0.00	0.05	-0.04
586	BCAT1	0.06	0.00	0.00	0.10
587	BCAT2	0.06	0.00	0.05	0.14
590	BCHE	0.00	0.00	0.00	NaN
593	BCKDHA	0.00	0.00	0.05	0.27
594	BCKDHB	0.10	0.00	0.20	0.02
595	CCND1	0.12	0.00	0.10	0.51
596	BCL2	0.02	0.00	0.00	0.35
597	BCL2A1	0.02	0.00	0.00	-0.05
598	BCL2L1	0.04	0.00	0.15	0.16
599	BCL2L2	0.02	0.14	0.10	0.00
602	BCL3	0.00	0.00	0.00	NaN
604	BCL6	0.00	0.14	0.00	-0.05
605	BCL7A	0.02	0.00	0.10	0.07
607	BCL9	0.02	0.00	0.00	0.09
608	TNFRSF17	0.00	0.00	0.00	NaN
610	HCN2	0.06	0.00	0.15	-0.02
611	OPN1SW	0.00	0.00	0.05	-0.04
613	BCR	0.00	0.14	0.00	0.04
617	BCS1L	0.04	0.00	0.00	0.14
623	BDKRB1	0.00	0.00	0.05	-0.09
624	BDKRB2	0.00	0.00	0.05	-0.09
627	BDNF	0.02	0.00	0.00	-0.03
631	BFSP1	0.00	0.00	0.00	NaN
632	BGLAP	0.04	0.14	0.05	-0.07
634	CEACAM1	0.00	0.00	0.00	NaN
635	BHMT	0.00	0.00	0.00	NaN
636	BICD1	0.02	0.00	0.00	0.00
637	BID	0.02	0.14	0.20	0.13
638	BIK	0.00	0.14	0.05	0.02
640	BLK	0.00	0.00	0.00	NaN
641	BLM	0.02	0.00	0.00	0.38
642	BLMH	0.10	0.00	0.05	0.03
644	BLVRA	0.00	0.00	0.00	NaN
645	BLVRB	0.00	0.00	0.05	0.15
648	BMI1	0.00	0.00	0.00	NaN
649	BMP1	0.00	0.00	0.05	0.25
650	BMP2	0.00	0.00	0.00	NaN
651	BMP3	0.02	0.00	0.00	-0.13
652	BMP4	0.00	0.00	0.00	NaN
653	BMP5	0.06	0.14	0.05	-0.11
654	BMP6	0.00	0.00	0.05	0.02
655	BMP7	0.08	0.00	0.05	0.10
657	BMPR1A	0.00	0.00	0.05	0.31

658	BMPR1B	0.04	0.00	0.05	0.07
659	BMPR2	0.00	0.00	0.00	NaN
662	BNIP1	0.00	0.00	0.10	-0.03
663	BNIP2	0.00	0.00	0.00	NaN
664	BNIP3	0.00	0.00	0.05	-0.01
665	BNIP3L	0.00	0.00	0.05	-0.12
666	BOK	0.08	0.43	0.10	-0.03
668	FOXL2	0.00	0.00	0.00	NaN
669	BPGM	0.00	0.00	0.05	-0.03
671	BPI	0.02	0.00	0.00	0.11
672	BRCA1	0.00	0.86	0.00	-0.04
673	BRAF	0.00	0.14	0.05	0.00
675	BRCA2	0.00	0.00	0.05	0.18
676	BRDT	0.00	0.00	0.00	NaN
677	ZFP36L1	0.02	0.00	0.00	0.04
678	ZFP36L2	0.02	0.00	0.05	-0.02
682	BSG	0.06	0.00	0.15	0.06
683	BST1	0.00	0.00	0.00	NaN
684	BST2	0.04	0.00	0.00	0.34
685	BTC	0.00	0.00	0.05	-0.15
686	BTD	0.00	0.00	0.05	-0.01
688	KLF5	0.02	0.00	0.00	-0.04
689	BTF3	0.06	0.00	0.00	0.10
690	BTF3L1	0.02	0.00	0.00	-0.06
694	BTG1	0.00	0.00	0.00	NaN
696	BTN1A1	0.00	0.14	0.00	0.07
699	BUB1	0.02	0.00	0.00	0.02
701	BUB1B	0.00	0.00	0.00	NaN
705	BYSL	0.06	0.00	0.05	0.08
708	C1QBP	0.04	0.00	0.10	0.11
710	SERPING1	0.00	0.00	0.00	NaN
712	C1QA	0.02	0.00	0.00	0.00
713	C1QB	0.02	0.00	0.00	0.03
715	C1R	0.02	0.00	0.15	0.04
716	C1S	0.02	0.00	0.15	-0.02
717	C2	0.02	0.00	0.05	-0.04
718	C3	0.02	0.00	0.15	0.25
719	C3AR1	0.02	0.00	0.15	0.10
720	C4A	0.02	0.00	0.05	0.12
721	C4B	0.02	0.00	0.05	0.11
722	C4BPA	0.00	0.00	0.05	-0.03
725	C4BPB	0.00	0.00	0.05	0.02
726	CAPN5	0.02	0.00	0.05	0.60
727	C5	0.00	0.00	0.00	NaN
729	C6	0.04	0.00	0.00	0.32
730	C7	0.04	0.00	0.00	0.28
731	C8A	0.02	0.00	0.00	-0.13

732	C8B	0.02	0.00	0.00	-0.06
735	C9	0.04	0.00	0.00	0.26
738	C11orf2	0.02	0.00	0.00	0.02
740	MRPL49	0.02	0.00	0.00	0.05
745	C11orf9	0.00	0.00	0.05	-0.06
746	C11orf10	0.00	0.00	0.05	0.07
750	C16orf3	0.00	0.00	0.10	0.01
753	C18orf1	0.08	0.14	0.10	0.12
754	PTTG1IP	0.00	0.00	0.05	-0.28
755	C21orf2	0.02	0.00	0.00	0.05
759	CA1	0.06	0.00	0.15	-0.05
760	CA2	0.06	0.00	0.15	0.16
761	CA3	0.06	0.00	0.15	-0.11
762	CA4	0.10	0.00	0.05	0.14
763	CA5A	0.00	0.00	0.10	-0.05
765	CA6	0.00	0.00	0.00	NaN
766	CA7	0.02	0.00	0.00	-0.08
767	CA8	0.02	0.00	0.10	-0.01
768	CA9	0.00	0.00	0.00	NaN
770	CA11	0.06	0.00	0.05	0.02
771	CA12	0.02	0.00	0.00	0.16
773	CACNA1A	0.00	0.14	0.05	-0.03
775	CACNA1C	0.08	0.14	0.10	0.11
776	CACNA1D	0.00	0.00	0.00	NaN
777	CACNA1E	0.02	0.00	0.00	0.29
779	CACNA1S	0.00	0.00	0.10	0.27
780	DDR1	0.04	0.14	0.05	0.06
781	CACNA2D1	0.02	0.00	0.00	-0.12
782	CACNB1	0.02	0.86	0.05	0.17
783	CACNB2	0.00	0.00	0.05	0.00
784	CACNB3	0.02	0.00	0.00	0.07
785	CACNB4	0.00	0.00	0.00	NaN
786	CACNG1	0.12	0.00	0.20	-0.10
788	SLC25A20	0.00	0.00	0.05	-0.11
790	CAD	0.00	0.00	0.00	NaN
793	CALB1	0.06	0.00	0.15	0.06
794	CALB2	0.00	0.00	0.05	-0.05
796	CALCA	0.00	0.00	0.10	-0.02
797	CALCB	0.00	0.00	0.10	0.01
799	CALCR	0.00	0.00	0.00	NaN
800	CALD1	0.00	0.00	0.05	0.13
801	CALM1	0.00	0.00	0.00	NaN
805	CALM2	0.00	0.00	0.00	NaN
808	CALM3	0.00	0.00	0.05	0.06
811	CALR	0.00	0.14	0.05	0.06
813	CALU	0.00	0.00	0.05	0.15
814	CAMK4	0.02	0.00	0.00	-0.09

815	CAMK2A	0.00	0.00	0.00	NaN
816	CAMK2B	0.00	0.00	0.00	NaN
818	CAMK2G	0.02	0.00	0.00	0.21
819	CAMLG	0.00	0.00	0.00	NaN
820	CAMP	0.00	0.00	0.05	-0.01
821	CANX	0.08	0.43	0.10	0.20
822	CAPG	0.00	0.00	0.00	NaN
823	CAPN1	0.02	0.00	0.00	0.04
824	CAPN2	0.02	0.00	0.05	0.08
825	CAPN3	0.04	0.00	0.00	-0.01
826	CAPNS1	0.02	0.00	0.05	0.18
829	CAPZA1	0.02	0.00	0.05	-0.04
830	CAPZA2	0.00	0.00	0.00	NaN
831	CAST	0.02	0.00	0.00	0.07
832	CAPZB	0.00	0.00	0.00	NaN
833	CARS	0.02	0.00	0.05	-0.02
834	CASP1	0.02	0.00	0.05	-0.11
835	CASP2	0.00	0.14	0.05	-0.04
836	CASP3	0.02	0.00	0.00	-0.01
837	CASP4	0.06	0.00	0.05	-0.09
838	CASP5	0.02	0.00	0.05	0.17
839	CASP6	0.08	0.00	0.00	0.00
840	CASP7	0.00	0.14	0.05	0.04
841	CASP8	0.00	0.00	0.00	NaN
842	CASP9	0.02	0.00	0.00	0.03
843	CASP10	0.00	0.00	0.05	0.43
844	CASQ1	0.00	0.00	0.00	NaN
845	CASQ2	0.08	0.00	0.10	0.20
846	CASR	0.00	0.00	0.10	-0.01
847	CAT	0.02	0.00	0.00	-0.14
857	CAV1	0.00	0.00	0.00	NaN
858	CAV2	0.00	0.00	0.00	NaN
859	CAV3	0.00	0.00	0.10	0.08
860	RUNX2	0.04	0.14	0.20	0.15
861	RUNX1	0.00	0.00	0.05	-0.08
863	CBFA2T3	0.00	0.00	0.10	-0.04
864	RUNX3	0.02	0.00	0.05	0.03
865	CBFB	0.02	0.00	0.00	-0.04
866	SERPINA6	0.00	0.00	0.00	NaN
867	CBL	0.00	0.00	0.05	-0.06
868	CBLB	0.02	0.14	0.05	0.32
869	CBLN1	0.43	0.29	0.15	-0.17
871	SERPINH1	0.00	0.00	0.00	NaN
873	CBR1	0.00	0.00	0.05	0.02
874	CBR3	0.00	0.00	0.05	0.04
875	CBS	0.00	0.00	0.00	NaN
881	CCIN	0.02	0.00	0.00	-0.02

883	CCBL1	0.00	0.00	0.00	NaN
885	CCK	0.00	0.00	0.05	-0.04
886	CCKAR	0.00	0.00	0.00	NaN
887	CCKBR	0.00	0.00	0.00	NaN
890	CCNA2	0.00	0.14	0.05	-0.15
891	CCNB1	0.06	0.00	0.00	-0.13
894	CCND2	0.02	0.00	0.00	-0.02
896	CCND3	0.06	0.00	0.05	-0.21
898	CCNE1	0.04	0.00	0.10	0.41
899	CCNF	0.02	0.00	0.00	0.09
900	CCNG1	0.00	0.00	0.00	NaN
901	CCNG2	0.00	0.00	0.00	NaN
902	CCNH	0.00	0.00	0.00	NaN
904	CCNT1	0.04	0.00	0.05	-0.06
905	CCNT2	0.00	0.00	0.00	NaN
908	CCT6A	0.04	0.14	0.20	-0.05
909	CD1A	0.00	0.00	0.00	NaN
910	CD1B	0.00	0.00	0.00	NaN
911	CD1C	0.00	0.00	0.00	NaN
912	CD1D	0.00	0.00	0.00	NaN
913	CD1E	0.00	0.00	0.00	NaN
914	CD2	0.08	0.00	0.10	0.22
915	CD3D	0.00	0.00	0.00	NaN
916	CD3E	0.00	0.00	0.00	NaN
917	CD3G	0.00	0.00	0.00	NaN
921	CD5	0.00	0.00	0.05	-0.08
922	CD5L	0.04	0.14	0.05	-0.10
923	CD6	0.00	0.00	0.05	0.02
924	CD7	0.00	0.00	0.00	NaN
925	CD8A	0.04	0.00	0.05	-0.05
928	CD9	0.02	0.00	0.15	0.04
929	CD14	0.00	0.00	0.00	NaN
930	CD19	0.06	0.14	0.00	0.01
931	MS4A2	0.00	0.00	0.00	NaN
933	CD22	0.00	0.00	0.00	NaN
940	CD28	0.00	0.00	0.00	NaN
941	CD80	0.00	0.00	0.00	NaN
942	CD86	0.00	0.00	0.10	0.17
943	TNFRSF8	0.00	0.00	0.00	NaN
944	TNFSF8	0.02	0.00	0.00	-0.08
945	CD33	0.02	0.00	0.05	-0.19
946	SIGLEC6	0.02	0.00	0.05	-0.07
948	CD36	0.00	0.00	0.00	NaN
951	CD37	0.02	0.00	0.05	-0.01
952	CD38	0.00	0.00	0.00	NaN
953	ENTPD1	0.00	0.00	0.00	NaN
955	ENTPD6	0.04	0.00	0.15	-0.20

956	ENTPD3	0.02	0.00	0.05	-0.13
957	ENTPD5	0.02	0.00	0.05	0.01
960	CD44	0.02	0.00	0.00	0.16
961	CD47	0.02	0.00	0.00	-0.06
962	CD48	0.00	0.00	0.00	NaN
963	CD53	0.06	0.14	0.00	-0.09
965	CD58	0.08	0.00	0.10	-0.05
966	CD59	0.00	0.00	0.00	NaN
967	CD63	0.02	0.00	0.05	-0.04
968	CD68	0.02	0.00	0.00	0.00
969	CD69	0.02	0.00	0.15	0.10
972	CD74	0.00	0.00	0.00	NaN
973	CD79A	0.00	0.00	0.05	0.29
974	CD79B	0.12	0.00	0.20	-0.06
975	CD81	0.00	0.00	0.00	NaN
976	CD97	0.00	0.00	0.05	-0.03
977	CD151	0.02	0.00	0.05	0.08
978	CDA	0.02	0.00	0.05	0.24
983	CDC2	0.00	0.00	0.05	0.24
987	LRBA	0.02	0.14	0.10	-0.15
988	CDC5L	0.04	0.14	0.20	0.18
990	CDC6	0.02	0.86	0.05	0.17
991	CDC20	0.02	0.00	0.05	0.03
993	CDC25A	0.00	0.00	0.05	0.20
994	CDC25B	0.02	0.00	0.00	0.11
995	CDC25C	0.02	0.00	0.00	0.03
996	CDC27	0.00	0.00	0.00	NaN
997	CDC34	0.06	0.00	0.15	0.16
998	CDC42	0.00	0.00	0.05	0.08
999	CDH1	0.00	0.00	0.05	0.20
1000	CDH2	0.00	0.00	0.05	0.11
1001	CDH3	0.00	0.00	0.05	0.27
1002	CDH4	0.12	0.00	0.00	0.00
1003	CDH5	0.02	0.00	0.00	-0.14
1004	CDH6	0.02	0.14	0.05	-0.07
1005	CDH7	0.02	0.00	0.00	-0.03
1006	CDH8	0.00	0.00	0.00	NaN
1007	CDH9	0.02	0.14	0.05	-0.02
1008	CDH10	0.02	0.14	0.05	-0.03
1009	CDH11	0.00	0.14	0.05	0.06
1010	CDH12	0.02	0.14	0.05	0.24
1012	CDH13	0.02	0.00	0.00	0.19
1013	CDH15	0.00	0.00	0.10	-0.06
1014	CDH16	0.02	0.00	0.00	0.04
1015	CDH17	0.08	0.00	0.05	-0.06
1016	CDH18	0.02	0.14	0.05	-0.03
1017	CDK2	0.02	0.00	0.05	0.02

1018	CDK3	0.02	0.14	0.05	0.07
1019	CDK4	0.02	0.00	0.00	0.07
1020	CDK5	0.02	0.00	0.00	-0.05
1021	CDK6	0.00	0.00	0.00	NaN
1022	CDK7	0.06	0.00	0.00	-0.14
1024	CDK8	0.00	0.00	0.05	0.19
1025	CDK9	0.02	0.00	0.00	-0.02
1026	CDKN1A	0.04	0.00	0.00	0.00
1027	CDKN1B	0.04	0.00	0.05	-0.28
1028	CDKN1C	0.00	0.00	0.00	NaN
1029	CDKN2A	-0.02	0.00	0.00	0.12
1030	CDKN2B	-0.02	0.00	0.00	0.03
1031	CDKN2C	0.02	0.00	0.00	-0.05
1032	CDKN2D	0.02	0.00	0.15	-0.13
1033	CDKN3	0.00	0.00	0.05	-0.12
1036	CDO1	0.02	0.00	0.00	-0.01
1039	CDR2	0.02	0.14	0.00	-0.05
1040	CDS1	0.00	0.00	0.05	-0.10
1041	CDSN	0.04	0.14	0.05	0.06
1044	CDX1	0.00	0.00	0.00	NaN
1045	CDX2	0.00	0.00	0.05	0.03
1047	CLGN	0.00	0.00	0.05	-0.11
1048	CEACAM5	0.00	0.00	0.05	-0.09
1050	CEBPA	0.00	0.00	0.00	NaN
1051	CEBPB	0.06	0.00	0.05	0.36
1052	CEBPD	0.04	0.00	0.00	-0.09
1053	CEBPE	0.02	0.14	0.10	0.26
1054	CEBPG	0.00	0.00	0.00	NaN
1056	CEL	0.04	0.00	0.00	-0.12
1058	CENPA	0.00	0.00	0.00	NaN
1060	CENPC1	0.00	0.00	0.05	0.21
1062	CENPE	0.08	0.00	0.00	0.24
1063	CENPF	0.00	0.00	0.05	0.19
1066	CES1	0.02	0.00	0.00	0.13
1068	CETN1	0.02	0.00	0.05	-0.06
1070	CETN3	0.00	0.00	0.00	NaN
1071	CETP	0.00	0.14	0.00	-0.02
1072	CFL1	0.04	0.00	0.05	0.31
1075	CTSC	0.00	0.00	0.00	NaN
1080	CFTR	0.00	0.00	0.15	-0.09
1081	CGA	0.00	0.00	0.05	-0.03
1082	CGB	0.06	0.00	0.05	0.29
1084	CEACAM3	0.00	0.00	0.05	0.63
1087	CEACAM7	0.00	0.00	0.05	0.59
1088	CEACAM8	0.00	0.00	0.00	NaN
1089	CEACAM4	0.00	0.00	0.05	0.19
1101	CHAD	0.10	0.00	0.10	0.00

1103	CHAT	0.02	0.00	0.05	-0.10
1105	CHD1	0.02	0.00	0.00	-0.08
1106	CHD2	0.02	0.00	0.10	-0.05
1107	CHD3	0.00	0.00	0.00	NaN
1108	CHD4	0.02	0.00	0.15	0.05
1109	AKR1C4	0.00	0.00	0.00	NaN
1111	CHEK1	0.02	0.00	0.00	-0.07
1113	CHGA	0.02	0.00	0.00	0.01
1114	CHGB	0.00	0.00	0.00	NaN
1116	CHI3L1	0.00	0.00	0.00	NaN
1117	CHI3L2	0.06	0.14	0.00	-0.21
1118	CHIT1	0.00	0.00	0.00	NaN
1122	CHML	0.02	0.00	0.10	0.09
1123	CHN1	0.00	0.00	0.00	NaN
1124	CHN2	0.00	0.00	0.00	NaN
1129	CHRM2	0.00	0.00	0.00	NaN
1132	CHRM4	0.00	0.00	0.05	0.02
1133	CHRM5	0.04	0.00	0.05	0.07
1134	CHRNA1	0.00	0.00	0.00	NaN
1135	CHRNA2	0.04	0.00	0.10	-0.06
1136	CHRNA3	0.00	0.00	0.05	0.02
1137	CHRNA4	0.08	0.00	0.05	0.07
1138	CHRNA5	0.00	0.00	0.05	-0.03
1139	CHRNA7	0.00	0.00	0.00	NaN
1140	CHRNB1	0.02	0.00	0.00	-0.03
1141	CHRNB2	0.04	0.00	0.15	-0.14
1142	CHRNB3	0.04	0.14	0.05	0.14
1143	CHRNB4	0.00	0.00	0.05	0.06
1144	CHRND	0.00	0.00	0.05	0.17
1145	CHRNE	0.04	0.00	0.10	0.18
1146	CHRNG	0.00	0.00	0.05	-0.08
1147	CHUK	0.00	0.00	0.05	-0.16
1149	CIDEA	0.04	0.00	0.10	-0.05
1152	CKB	0.00	0.00	0.05	-0.13
1153	CIRBP	0.02	0.00	0.05	-0.11
1154	CISH	0.00	0.00	0.10	-0.10
1158	CKM	0.00	0.00	0.00	NaN
1160	CKMT2	0.00	0.00	0.00	NaN
1164	CKS2	0.00	0.00	0.00	NaN
1173	AP2M1	0.00	0.00	0.00	NaN
1174	AP1S1	0.00	0.14	0.00	0.22
1175	AP2S1	0.00	0.00	0.05	0.16
1176	AP3S1	0.02	0.00	0.00	0.02
1178	CLC	0.06	0.00	0.05	0.03
1179	CLCA1	0.04	0.00	0.05	0.03
1180	CLCN1	0.00	0.14	0.05	-0.05
1181	CLCN2	0.02	0.14	0.00	-0.07

1182	CLCN3	0.02	0.00	0.00	0.29
1185	CLCN6	0.02	0.00	0.00	0.03
1186	CLCN7	0.18	0.00	0.10	-0.08
1187	CLCNKA	0.02	0.00	0.00	-0.06
1188	CLCNKB	0.02	0.00	0.00	-0.02
1191	CLU	0.04	0.00	0.10	-0.12
1192	CLIC1	0.02	0.00	0.05	0.15
1196	CLK2	0.04	0.00	0.15	0.11
1198	CLK3	0.00	0.00	0.00	NaN
1201	CLN3	0.06	0.14	0.00	0.23
1203	CLN5	0.02	0.00	0.00	-0.06
1207	CLNS1A	0.06	0.00	0.05	0.22
1208	CLPS	0.04	0.00	0.00	-0.05
1209	CLPTM1	0.00	0.00	0.00	NaN
1211	CLTA	0.02	0.00	0.00	-0.19
1212	CLTB	0.00	0.00	0.00	NaN
1213	CLTC	0.06	0.00	0.05	0.49
1215	CMA1	0.02	0.00	0.05	-0.08
1230	CCR1	0.02	0.14	0.15	-0.11
1231	CCR2	0.02	0.14	0.15	-0.04
1232	CCR3	0.02	0.14	0.15	-0.16
1233	CCR4	0.06	0.00	0.05	0.02
1235	CCR6	0.02	0.00	0.05	-0.02
1236	CCR7	0.02	0.86	0.05	-0.02
1237	CCR8	0.02	0.00	0.05	0.02
1238	CCBP2	0.00	0.00	0.05	0.16
1240	CMKLR1	0.02	0.00	0.00	-0.03
1241	LTB4R	0.02	0.14	0.10	-0.06
1244	ABCC2	0.00	0.00	0.00	NaN
1258	CNGB1	0.02	0.00	0.00	0.07
1259	CNGA1	0.00	0.29	0.10	0.29
1261	CNGA3	0.02	0.00	0.10	-0.11
1264	CNN1	0.02	0.00	0.00	-0.08
1265	CNN2	0.02	0.00	0.05	-0.26
1266	CNN3	0.00	0.00	0.00	NaN
1267	CNP	0.02	0.86	0.05	0.35
1268	CNR1	0.00	0.00	0.05	0.07
1269	CNR2	0.02	0.00	0.05	-0.05
1270	CNTF	0.02	0.00	0.00	0.37
1271	CNTFR	0.00	0.00	0.00	NaN
1272	CNTN1	0.00	0.00	0.35	-0.03
1277	COL1A1	0.06	0.00	0.00	0.07
1280	COL2A1	0.04	0.00	0.05	0.03
1281	COL3A1	0.00	0.00	0.05	0.09
1282	COL4A1	0.00	0.00	0.05	0.14
1284	COL4A2	0.00	0.00	0.05	0.10
1285	COL4A3	0.02	0.00	0.15	0.05

1286	COL4A4	0.02	0.00	0.15	0.03
1290	COL5A2	0.00	0.00	0.05	0.07
1291	COL6A1	0.00	0.00	0.05	0.03
1292	COL6A2	0.00	0.00	0.05	-0.04
1293	COL6A3	0.00	0.00	0.00	NaN
1294	COL7A1	0.00	0.00	0.05	0.08
1295	COL8A1	0.00	0.00	0.15	-0.09
1297	COL9A1	0.12	0.00	0.05	0.22
1298	COL9A2	0.02	0.00	0.05	-0.11
1299	COL9A3	0.08	0.00	0.05	-0.06
1300	COL10A1	0.06	0.00	0.05	-0.09
1301	COL11A1	0.00	0.14	0.00	0.10
1305	COL13A1	0.00	0.00	0.00	NaN
1306	COL15A1	0.04	0.00	0.00	0.05
1307	COL16A1	0.00	0.14	0.00	-0.12
1308	COL17A1	0.02	0.00	0.00	0.20
1310	COL19A1	0.12	0.00	0.05	0.12
1311	COMP	0.02	0.00	0.00	0.10
1312	COMT	0.06	0.14	0.15	0.17
1314	COPA	0.00	0.00	0.00	NaN
1317	SLC31A1	0.00	0.00	0.00	NaN
1318	SLC31A2	0.02	0.00	0.00	0.11
1325	CORT	0.02	0.00	0.00	0.20
1326	MAP3K8	0.02	0.14	0.00	0.25
1327	COX4I1	0.02	0.00	0.05	0.05
1329	COX5B	0.00	0.00	0.10	-0.03
1337	COX6A1	0.02	0.00	0.10	0.02
1339	COX6A2	0.02	0.00	0.00	-0.09
1345	COX6C	0.08	0.00	0.05	0.25
1346	COX7A1	0.02	0.00	0.05	0.03
1347	COX7A2	0.10	0.00	0.20	0.20
1350	COX7C	0.00	0.00	0.00	NaN
1352	COX10	0.04	0.00	0.00	0.18
1353	COX11	0.02	0.00	0.10	0.22
1355	COX15	0.00	0.00	0.00	NaN
1356	CP	0.00	0.00	0.00	NaN
1357	CPA1	0.00	0.00	0.00	NaN
1358	CPA2	0.00	0.00	0.00	NaN
1359	CPA3	0.00	0.00	0.00	NaN
1360	CPB1	0.00	0.00	0.00	NaN
1361	CPB2	0.00	0.00	0.00	NaN
1362	CPD	0.10	0.00	0.05	0.18
1363	CPE	0.02	0.00	0.00	0.05
1364	CLDN4	0.00	0.00	0.00	NaN
1365	CLDN3	0.00	0.00	0.00	NaN
1366	CLDN7	0.00	0.00	0.00	NaN
1368	CPM	0.08	0.00	0.00	0.12

1369	CPN1	0.00	0.00	0.05	-0.04
1370	CPN2	0.02	0.00	0.00	-0.02
1371	CPO	0.00	0.00	0.00	NaN
1373	CPS1	0.02	0.00	0.05	-0.04
1374	CPT1A	0.02	0.00	0.05	0.58
1376	CPT2	0.02	0.00	0.00	0.15
1378	CR1	0.00	0.00	0.05	0.12
1380	CR2	0.00	0.00	0.05	-0.04
1381	CRABP1	0.00	0.00	0.05	0.00
1382	CRABP2	0.04	0.14	0.05	0.03
1384	CRAT	0.00	0.00	0.00	NaN
1385	CREB1	0.02	0.00	0.05	0.04
1386	ATF2	0.00	0.00	0.00	NaN
1387	CREBBP	0.02	0.00	0.00	0.07
1389	CREBL2	0.04	0.00	0.05	-0.13
1390	CREM	0.04	0.29	0.25	0.26
1392	CRH	0.00	0.00	0.00	NaN
1393	CRHBP	0.04	0.00	0.05	-0.07
1394	CRHR1	0.00	0.00	0.05	0.02
1395	CRHR2	0.00	0.00	0.00	NaN
1396	CRIP1	0.02	0.00	0.10	-0.11
1397	CRIP2	0.02	0.00	0.10	-0.05
1398	CRK	0.04	0.00	0.05	0.01
1399	CRKL	0.06	0.14	0.15	0.12
1400	CRMP1	0.00	0.00	0.00	NaN
1401	CRP	0.00	0.00	0.00	NaN
1406	CRX	0.00	0.00	0.00	NaN
1407	CRY1	0.02	0.00	0.00	0.00
1408	CRY2	0.04	0.00	0.05	-0.22
1409	CRYAA	0.00	0.00	0.00	NaN
1410	CRYAB	0.02	0.00	0.00	-0.11
1411	CRYBA1	0.10	0.00	0.05	0.17
1412	CRYBA2	0.04	0.00	0.00	-0.07
1413	CRYBA4	0.00	0.14	0.05	0.19
1414	CRYBB1	0.00	0.14	0.05	-0.12
1415	CRYBB2	0.00	0.14	0.05	0.17
1417	CRYBB3	0.00	0.14	0.05	0.18
1418	CRYGA	0.02	0.00	0.05	-0.10
1419	CRYGB	0.02	0.00	0.05	-0.13
1420	CRYGC	0.02	0.00	0.05	0.04
1421	CRYGD	0.02	0.00	0.05	-0.01
1428	CRYM	0.02	0.14	0.00	-0.12
1429	CRYZ	0.04	0.14	0.00	-0.08
1431	CS	0.00	0.00	0.00	NaN
1432	MAPK14	0.04	0.00	0.00	0.18
1434	CSE1L	0.04	0.00	0.00	0.78
1435	CSF1	0.02	0.00	0.05	0.08

1436	CSF1R	0.00	0.14	0.05	-0.09
1437	CSF2	0.02	0.00	0.00	0.06
1439	CSF2RB	0.00	0.14	0.00	0.12
1440	CSF3	0.02	0.86	0.05	0.00
1441	CSF3R	0.04	0.00	0.05	0.04
1442	CSH1	0.12	0.00	0.20	0.08
1443	CSH2	0.12	0.00	0.20	0.07
1444	CSHL1	0.12	0.00	0.20	0.05
1445	CSK	0.00	0.00	0.05	-0.01
1447	CSN2	0.00	0.14	0.00	-0.01
1452	CSNK1A1	0.00	0.14	0.05	-0.13
1453	CSNK1D	0.00	0.00	0.00	NaN
1454	CSNK1E	0.00	0.14	0.00	0.11
1455	CSNK1G2	0.02	0.00	0.05	0.12
1456	CSNK1G3	0.00	0.00	0.00	NaN
1457	CSNK2A1	0.00	0.00	0.00	NaN
1459	CSNK2A2	0.04	0.00	0.05	-0.03
1460	CSNK2B	0.02	0.00	0.05	0.10
1464	CSPG4	0.00	0.00	0.05	0.07
1465	CSRP1	0.00	0.00	0.05	-0.07
1466	CSRP2	0.00	0.00	0.00	NaN
1468	SLC25A10	0.00	0.00	0.00	NaN
1469	CST1	0.02	0.00	0.05	-0.10
1470	CST2	0.02	0.00	0.05	-0.08
1471	CST3	0.00	0.00	0.00	NaN
1472	CST4	0.02	0.00	0.05	-0.08
1473	CST5	0.02	0.00	0.05	-0.06
1474	CST6	0.04	0.00	0.05	0.14
1475	CSTA	0.00	0.00	0.10	0.30
1476	CSTB	0.02	0.00	0.00	-0.03
1477	CSTF1	0.12	0.00	0.10	-0.11
1479	CSTF3	0.02	0.00	0.00	-0.07
1486	CTBS	0.04	0.00	0.05	0.08
1487	CTBP1	0.02	0.00	0.00	0.09
1488	CTBP2	0.04	0.00	0.05	0.04
1489	CTF1	0.02	0.00	0.00	-0.01
1490	CTGF	0.02	0.00	0.00	-0.09
1491	CTH	0.04	0.14	0.00	0.03
1493	CTLA4	0.00	0.00	0.00	NaN
1495	CTNNA1	0.00	0.00	0.00	NaN
1496	CTNNA2	0.00	0.00	0.00	NaN
1497	CTNS	0.04	0.00	0.10	0.06
1499	CTNNB1	0.00	0.00	0.10	-0.01
1500	CTNND1	0.02	0.00	0.00	0.16
1501	CTNND2	0.02	0.00	0.00	0.25
1503	CTPS	0.02	0.00	0.05	0.08
1504	CTRB1	0.00	0.00	0.00	NaN

1506	CTRL	0.02	0.00	0.00	0.00
1508	CTSB	0.02	0.00	0.05	0.24
1509	CTSD	0.02	0.00	0.05	0.10
1510	CTSE	0.02	0.00	0.05	-0.02
1511	CTSG	0.02	0.00	0.05	-0.18
1512	CTSH	0.02	0.00	0.00	0.10
1513	CTSK	0.02	0.00	0.10	-0.31
1515	CTSL2	0.04	0.00	0.00	0.02
1519	CTSO	0.00	0.14	0.00	-0.16
1520	CTSS	0.02	0.00	0.10	0.14
1521	CTSW	0.04	0.00	0.05	0.06
1522	CTSZ	0.12	0.00	0.00	0.09
1524	CX3CR1	0.02	0.00	0.05	-0.16
1525	CXADR	0.00	0.00	0.00	NaN
1534	CYB561	0.12	0.00	0.20	0.39
1535	CYBA	0.00	0.00	0.10	0.18
1537	CYC1	0.10	0.00	0.15	0.55
1539	CYLC2	0.04	0.00	0.00	-0.08
1540	CYLD	0.08	0.00	0.05	0.04
1543	CYP1A1	0.00	0.00	0.05	-0.10
1544	CYP1A2	0.00	0.00	0.05	0.00
1545	CYP1B1	0.02	0.00	0.05	0.08
1548	CYP2A6	0.00	0.00	0.05	0.11
1549	CYP2A7	0.00	0.00	0.05	0.09
1551	CYP3A7	0.02	0.00	0.00	-0.01
1553	CYP2A13	0.00	0.00	0.05	0.48
1555	CYP2B6	0.00	0.00	0.05	0.09
1558	CYP2C8	0.00	0.00	0.00	NaN
1559	CYP2C9	0.00	0.00	0.00	NaN
1562	CYP2C18	0.02	0.00	0.05	-0.01
1565	CYP2D6	0.00	0.14	0.05	0.07
1572	CYP2F1	0.00	0.00	0.05	0.06
1573	CYP2J2	0.06	0.00	0.00	0.26
1576	CYP3A4	0.02	0.00	0.00	0.11
1577	CYP3A5	0.02	0.00	0.00	-0.13
1579	CYP4A11	0.02	0.00	0.05	-0.09
1580	CYP4B1	0.02	0.00	0.05	-0.09
1581	CYP7A1	0.04	0.00	0.00	-0.05
1584	CYP11B1	0.10	0.00	0.15	-0.01
1585	CYP11B2	0.10	0.00	0.15	-0.03
1589	CYP21A2	0.02	0.00	0.05	0.26
1592	CYP26A1	0.00	0.00	0.15	-0.11
1593	CYP27A1	0.04	0.00	0.00	-0.05
1594	CYP27B1	0.02	0.00	0.00	-0.08
1600	DAB1	0.02	0.00	0.00	-0.02
1601	DAB2	0.04	0.00	0.00	0.18
1603	DAD1	0.02	0.00	0.00	-0.03

1605	DAG1	0.06	0.00	0.05	-0.07
1606	DGKA	0.02	0.00	0.05	0.02
1607	DGKB	0.00	0.00	0.00	NaN
1608	DGKG	0.02	0.14	0.00	0.01
1609	DGKQ	0.06	0.00	0.05	-0.01
1610	DAO	0.02	0.00	0.00	-0.03
1611	DAP	0.00	0.00	0.00	NaN
1612	DAPK1	0.04	0.00	0.00	0.07
1613	DAPK3	0.02	0.00	0.15	0.01
1615	DARS	0.00	0.00	0.00	NaN
1616	DAXX	0.02	0.00	0.05	0.27
1618	DAZL	0.06	0.00	0.05	-0.09
1621	DBH	0.04	0.00	0.00	-0.03
1622	DBI	0.00	0.00	0.10	0.07
1627	DBN1	0.00	0.29	0.00	0.09
1628	DBP	0.06	0.00	0.05	0.23
1629	DBT	0.02	0.00	0.00	0.04
1630	DCC	0.00	0.00	0.15	0.00
1633	DCK	0.02	0.00	0.05	0.12
1634	DCN	0.00	0.00	0.00	NaN
1635	DCTD	0.00	0.14	0.05	-0.05
1636	ACE	0.12	0.00	0.20	0.01
1638	DCT	0.04	0.00	0.00	-0.01
1639	DCTN1	0.00	0.00	0.05	-0.02
1642	DDB1	0.00	0.00	0.05	0.01
1643	DDB2	0.02	0.00	0.00	0.04
1644	DDC	0.00	0.00	0.00	NaN
1645	AKR1C1	0.00	0.00	0.00	NaN
1646	AKR1C2	0.00	0.00	0.00	NaN
1647	GADD45A	0.02	0.00	0.00	0.07
1649	DDIT3	0.02	0.14	0.00	-0.01
1650	DDOST	0.02	0.00	0.05	-0.03
1652	DDT	0.00	0.14	0.05	-0.02
1653	DDX1	0.00	0.00	0.00	NaN
1655	DDX5	0.12	0.00	0.20	0.19
1656	DDX6	0.00	0.00	0.05	0.18
1657	DMXL1	0.00	0.00	0.00	NaN
1662	DDX10	0.02	0.00	0.00	-0.06
1663	DDX11	0.02	0.00	0.00	0.01
1666	DECR1	0.06	0.00	0.15	0.21
1667	DEFA1	0.02	0.14	0.10	-0.07
1669	DEFA4	0.02	0.14	0.10	-0.15
1670	DEFA5	0.02	0.14	0.10	-0.05
1671	DEFA6	0.02	0.14	0.10	0.11
1672	DEFB1	0.02	0.14	0.10	0.09
1673	DEFB4	0.02	0.14	0.10	-0.10
1674	DES	0.04	0.00	0.00	0.21

1676	DFFA	0.02	0.00	0.00	0.07
1687	DFNA5	0.00	0.00	0.05	0.08
1690	COCH	0.02	0.00	0.00	0.06
1716	DGUOK	0.00	0.00	0.05	-0.05
1717	DHCR7	0.06	0.00	0.15	0.60
1718	DHCR24	0.02	0.00	0.00	-0.02
1719	DHFR	0.00	0.00	0.00	NaN
1723	DHODH	0.00	0.00	0.05	-0.07
1725	DHPS	0.06	0.14	0.00	0.10
1728	NQO1	0.00	0.00	0.00	NaN
1729	DIAPH1	0.00	0.00	0.00	NaN
1733	DIO1	0.04	0.00	0.00	-0.12
1734	DIO2	0.02	0.00	0.00	0.13
1735	DIO3	0.02	0.00	0.00	-0.09
1737	DLAT	0.02	0.00	0.00	-0.04
1738	DLD	0.00	0.00	0.00	NaN
1739	DLG1	0.02	0.00	0.00	0.24
1740	DLG2	0.00	0.00	0.00	NaN
1742	DLG4	0.00	0.00	0.00	NaN
1743	DLST	0.02	0.00	0.05	-0.07
1746	DLX2	0.00	0.00	0.00	NaN
1748	DLX4	0.06	0.00	0.00	-0.05
1749	DLX5	0.04	0.14	0.05	0.08
1750	DLX6	0.04	0.14	0.05	0.03
1755	DMBT1	0.04	0.00	0.00	0.02
1757	SARDH	0.04	0.00	0.00	0.08
1758	DMP1	0.02	0.00	0.00	0.05
1759	DNM1	0.00	0.00	0.00	NaN
1760	DMPK	0.00	0.00	0.05	-0.12
1761	DMRT1	0.04	0.00	0.10	-0.04
1762	DMWD	0.00	0.00	0.05	0.15
1770	DNAH9	0.04	0.00	0.00	0.18
1773	DNASE1	0.02	0.00	0.00	0.07
1775	DNASE1L2	0.18	0.00	0.10	0.06
1776	DNASE1L3	0.02	0.00	0.00	0.36
1777	DNASE2	0.00	0.14	0.05	-0.14
1785	DNM2	0.02	0.00	0.15	0.23
1786	DNMT1	0.02	0.00	0.15	-0.04
1788	DNMT3A	0.00	0.00	0.00	NaN
1789	DNMT3B	0.04	0.00	0.15	0.00
1791	DNTT	0.00	0.00	0.00	NaN
1793	DOCK1	0.02	0.00	0.00	-0.04
1794	DOCK2	0.00	0.00	0.00	NaN
1795	DOCK3	0.00	0.00	0.10	0.01
1796	DOK1	0.00	0.00	0.05	0.00
1797	DOM3Z	0.02	0.00	0.05	0.27
1798	DPAGT1	0.00	0.00	0.05	0.00

1800	DPEP1	0.00	0.00	0.10	-0.12
1803	DPP4	0.02	0.00	0.00	0.02
1804	DPP6	0.00	0.00	0.00	NaN
1805	DPT	0.00	0.00	0.10	-0.01
1806	DPYD	0.02	0.00	0.00	-0.13
1807	DPYS	0.12	0.00	0.25	0.06
1808	DPYSL2	0.00	0.00	0.05	0.17
1809	DPYSL3	0.00	0.00	0.00	NaN
1810	DR1	0.00	0.00	0.00	NaN
1811	SLC26A3	0.00	0.00	0.00	NaN
1812	DRD1	0.00	0.00	0.00	NaN
1813	DRD2	0.00	0.00	0.00	NaN
1814	DRD3	0.02	0.00	0.00	0.14
1815	DRD4	0.02	0.00	0.05	0.09
1819	DRG2	0.02	0.00	0.00	-0.02
1823	DSC1	0.02	0.00	0.10	-0.14
1824	DSC2	0.02	0.00	0.10	0.13
1825	DSC3	0.02	0.00	0.10	-0.04
1826	DSCAM	0.00	0.00	0.05	0.10
1828	DSG1	0.02	0.00	0.10	-0.04
1830	DSG3	0.02	0.00	0.10	0.01
1832	DSP	0.00	0.00	0.05	0.06
1836	SLC26A2	0.00	0.14	0.05	-0.01
1837	DTNA	0.04	0.00	0.10	-0.03
1838	DTNB	0.00	0.00	0.00	NaN
1841	DTYMK	0.08	0.43	0.10	-0.11
1842	ECM2	0.04	0.00	0.05	-0.19
1843	DUSP1	0.00	0.00	0.10	-0.04
1844	DUSP2	0.04	0.00	0.05	0.03
1845	DUSP3	0.00	0.14	0.05	0.09
1846	DUSP4	0.04	0.00	0.00	0.08
1847	DUSP5	0.00	0.14	0.00	-0.16
1848	DUSP6	0.00	0.00	0.00	NaN
1849	DUSP7	0.00	0.00	0.10	-0.10
1850	DUSP8	0.02	0.00	0.05	0.08
1854	DUT	0.02	0.00	0.05	-0.28
1856	DVL2	0.00	0.00	0.00	NaN
1857	DVL3	0.00	0.00	0.00	NaN
1859	DYRK1A	0.00	0.00	0.00	NaN
1869	E2F1	0.02	0.00	0.05	-0.06
1870	E2F2	0.02	0.00	0.05	0.19
1871	E2F3	0.00	0.00	0.00	NaN
1874	E2F4	0.02	0.00	0.00	-0.02
1875	E2F5	0.06	0.00	0.15	0.29
1876	E2F6	0.00	0.00	0.00	NaN
1877	E4F1	0.18	0.00	0.10	-0.07
1889	ECE1	0.02	0.00	0.00	0.04

1891	ECH1	0.06	0.00	0.05	0.16
1892	ECHS1	0.00	0.00	0.00	NaN
1893	ECM1	0.02	0.00	0.10	-0.17
1906	EDN1	0.04	0.00	0.00	-0.04
1907	EDN2	0.02	0.00	0.05	-0.05
1908	EDN3	0.12	0.00	0.00	0.04
1909	EDNRA	0.02	0.14	0.10	-0.03
1910	EDNRB	0.02	0.00	0.00	0.05
1915	EEF1A1	0.02	0.00	0.00	-0.14
1917	EEF1A2	0.08	0.00	0.05	0.10
1933	EEF1B2	0.00	0.00	0.00	NaN
1936	EEF1D	0.10	0.00	0.15	0.19
1937	EEF1G	0.00	0.00	0.05	0.07
1938	EEF2	0.02	0.00	0.15	-0.04
1939	LGTN	0.00	0.00	0.05	-0.22
1942	EFNA1	0.04	0.00	0.15	0.07
1943	EFNA2	0.02	0.00	0.05	-0.02
1944	EFNA3	0.04	0.00	0.15	0.00
1945	EFNA4	0.04	0.00	0.15	0.19
1946	EFNA5	0.02	0.00	0.00	-0.11
1948	EFNB2	0.02	0.00	0.05	0.14
1949	EFNB3	0.00	0.00	0.00	NaN
1950	EGF	0.08	0.00	0.00	-0.01
1951	CELSR3	0.00	0.00	0.05	0.09
1952	CELSR2	0.00	0.00	0.00	NaN
1956	EGFR	0.02	0.00	0.05	-0.02
1958	EGR1	0.00	0.00	0.00	NaN
1959	EGR2	0.00	0.00	0.00	NaN
1960	EGR3	0.00	0.00	0.05	-0.09
1961	EGR4	0.00	0.00	0.05	-0.07
1962	EHHADH	0.02	0.14	0.00	-0.15
1965	EIF2S1	0.00	0.00	0.05	0.15
1967	EIF2B1	0.02	0.00	0.10	0.04
1969	EPHA2	0.02	0.00	0.00	-0.06
1973	EIF4A1	0.02	0.00	0.00	-0.10
1974	EIF4A2	0.02	0.14	0.00	-0.07
1975	EIF4B	0.00	0.00	0.00	NaN
1977	EIF4E	0.00	0.00	0.00	NaN
1978	EIF4EBP1	0.12	0.14	0.05	0.31
1979	EIF4EBP2	0.00	0.00	0.00	NaN
1981	EIF4G1	0.02	0.14	0.00	-0.06
1982	EIF4G2	0.02	0.00	0.00	0.04
1983	EIF5	0.00	0.00	0.05	0.03
1984	EIF5A	0.02	0.00	0.00	0.03
1990	ELA1	0.00	0.00	0.00	NaN
1991	ELA2	0.06	0.00	0.15	0.02
1993	ELAVL2	0.02	0.14	0.10	-0.02

1994	ELAVL1	0.02	0.00	0.15	0.25
1995	ELAVL3	0.02	0.00	0.00	0.03
1996	ELAVL4	0.02	0.00	0.00	-0.04
1997	ELF1	0.00	0.00	0.00	NaN
1998	ELF2	0.00	0.00	0.05	0.10
1999	ELF3	0.02	0.00	0.00	0.08
2001	ELF5	0.02	0.00	0.00	0.03
2004	ELK3	0.02	0.00	0.00	0.00
2005	ELK4	0.02	0.00	0.05	0.02
2006	ELN	0.00	0.00	0.00	NaN
2009	EML1	0.04	0.00	0.00	-0.11
2012	EMP1	0.04	0.00	0.00	-0.09
2013	EMP2	0.00	0.00	0.00	NaN
2014	EMP3	0.02	0.00	0.00	-0.11
2015	EMR1	0.02	0.00	0.15	0.30
2016	EMX1	0.00	0.00	0.05	-0.13
2018	EMX2	0.04	0.14	0.05	-0.02
2019	EN1	0.00	0.00	0.10	0.15
2020	EN2	0.00	0.00	0.05	0.00
2021	ENDOG	0.00	0.00	0.00	NaN
2022	ENG	0.02	0.00	0.00	0.14
2023	ENO1	0.00	0.00	0.00	NaN
2026	ENO2	0.02	0.00	0.15	0.23
2027	ENO3	0.04	0.00	0.10	0.10
2028	ENPEP	0.08	0.00	0.00	0.18
2029	ENSA	0.02	0.00	0.10	-0.01
2030	SLC29A1	0.04	0.14	0.20	-0.10
2033	EP300	0.00	0.14	0.00	-0.06
2034	EPAS1	0.00	0.00	0.00	NaN
2035	EPB41	0.04	0.00	0.00	0.28
2037	EPB41L2	0.02	0.00	0.10	0.16
2038	EPB42	0.04	0.00	0.00	-0.14
2039	EPB49	0.00	0.00	0.05	0.00
2041	EPHA1	0.00	0.14	0.05	0.12
2042	EPHA3	0.16	0.14	0.30	0.18
2043	EPHA4	0.04	0.00	0.00	0.00
2044	EPHA5	0.06	0.00	0.00	0.04
2045	EPHA7	0.08	0.00	0.00	-0.04
2047	EPHB1	0.00	0.00	0.10	0.32
2048	EPHB2	0.02	0.00	0.00	0.08
2049	EPHB3	0.02	0.14	0.00	-0.13
2050	EPHB4	0.00	0.00	0.00	NaN
2051	EPHB6	0.00	0.14	0.05	0.07
2052	EPHX1	0.00	0.00	0.05	0.14
2053	EPHX2	0.04	0.00	0.10	-0.12
2055	CLN8	0.00	0.00	0.00	NaN
2056	EPO	0.00	0.00	0.00	NaN

2057	EPOR	0.02	0.00	0.00	0.01
2058	EPRS	0.02	0.00	0.00	0.07
2059	EPS8	0.02	0.00	0.00	0.11
2060	EPS15	0.02	0.00	0.00	0.19
2063	NR2F6	0.04	0.00	0.00	0.45
2064	ERBB2	0.02	0.86	0.05	0.71
2065	ERBB3	0.02	0.00	0.05	0.04
2066	ERBB4	0.02	0.00	0.05	0.06
2067	ERCC1	0.00	0.00	0.00	NaN
2068	ERCC2	0.00	0.00	0.00	NaN
2069	EREG	0.00	0.00	0.05	-0.02
2070	EYA4	0.00	0.00	0.00	NaN
2071	ERCC3	0.00	0.00	0.10	-0.08
2072	ERCC4	0.02	0.00	0.05	-0.01
2073	ERCC5	0.02	0.00	0.00	0.07
2074	ERCC6	0.02	0.00	0.05	0.10
2077	ERF	0.00	0.00	0.05	-0.14
2078	ERG	0.00	0.00	0.00	NaN
2079	ERH	0.02	0.00	0.05	-0.07
2081	ERN1	0.12	0.00	0.20	0.09
2098	ESD	0.00	0.00	0.00	NaN
2099	ESR1	0.02	0.00	0.05	0.00
2100	ESR2	0.06	0.00	0.00	0.17
2101	ESRRA	0.02	0.00	0.00	0.03
2103	ESRRB	0.00	0.00	0.00	NaN
2104	ESRRG	0.00	0.00	0.00	NaN
2107	ETF1	0.00	0.00	0.00	NaN
2108	ETFA	0.00	0.00	0.05	-0.07
2109	ETFB	0.02	0.00	0.05	0.00
2110	ETFDH	0.02	0.00	0.00	-0.09
2113	ETS1	0.02	0.00	0.00	0.03
2114	ETS2	0.02	0.00	0.00	0.08
2115	ETV1	0.00	0.00	0.00	NaN
2116	ETV2	0.00	0.00	0.00	NaN
2117	ETV3	0.04	0.14	0.05	-0.09
2119	ETV5	0.02	0.14	0.00	-0.04
2120	ETV6	0.02	0.00	0.15	0.00
2121	EVC	0.00	0.00	0.00	NaN
2122	EVI1	0.00	0.00	0.00	NaN
2123	EVI2A	0.10	0.00	0.05	-0.12
2124	EVI2B	0.10	0.00	0.05	-0.16
2125	EVPL	0.02	0.14	0.05	0.13
2128	EVX1	0.00	0.00	0.00	NaN
2130	EWSR1	0.00	0.14	0.05	0.28
2131	EXT1	0.10	0.00	0.15	-0.03
2132	EXT2	0.00	0.00	0.00	NaN
2134	EXTL1	0.02	0.00	0.05	0.02

2135	EXTL2	0.02	0.00	0.00	0.06
2137	EXTL3	0.00	0.00	0.00	NaN
2138	EYA1	0.04	0.14	0.00	0.01
2139	EYA2	0.02	0.00	0.00	-0.03
2140	EYA3	0.04	0.00	0.00	0.45
2145	EZH1	0.02	0.86	0.05	-0.02
2146	EZH2	0.06	0.00	0.15	0.28
2147	F2	0.02	0.00	0.00	-0.06
2149	F2R	0.04	0.00	0.05	0.18
2150	F2RL1	0.04	0.00	0.05	0.04
2151	F2RL2	0.04	0.00	0.05	-0.08
2152	F3	0.00	0.00	0.00	NaN
2153	F5	0.02	0.00	0.00	0.07
2155	F7	0.06	0.00	0.00	0.12
2159	F10	0.06	0.00	0.00	0.08
2160	F11	0.00	0.00	0.00	NaN
2161	F12	0.00	0.29	0.00	0.35
2162	F13A1	0.02	0.00	0.00	-0.18
2165	F13B	0.00	0.00	0.00	NaN
2166	FAAH	0.02	0.00	0.05	0.22
2167	FABP4	0.06	0.00	0.15	-0.19
2168	FABP1	0.04	0.00	0.05	-0.04
2169	FABP2	0.00	0.00	0.00	NaN
2170	FABP3	0.00	0.14	0.00	0.01
2171	FABP5	0.06	0.00	0.00	-0.02
2172	FABP6	0.00	0.00	0.00	NaN
2173	FABP7	0.08	0.00	0.00	-0.03
2175	FANCA	0.00	0.00	0.10	0.27
2176	FANCC	0.00	0.00	0.00	NaN
2178	FANCE	0.04	0.00	0.00	0.01
2184	FAH	0.02	0.00	0.00	0.01
2185	PTK2B	0.04	0.00	0.10	0.05
2188	FANCF	0.02	0.00	0.00	0.12
2189	FANCG	0.00	0.00	0.00	NaN
2191	FAP	0.02	0.00	0.00	0.03
2192	FBLN1	0.02	0.14	0.05	-0.05
2196	FAT2	0.00	0.14	0.00	0.12
2197	FAU	0.02	0.00	0.00	0.02
2199	FBLN2	0.00	0.00	0.05	0.01
2200	FBN1	0.02	0.00	0.00	-0.14
2201	FBN2	0.00	0.00	0.00	NaN
2202	EFEMP1	0.02	0.00	0.05	-0.18
2203	FBP1	0.00	0.00	0.00	NaN
2204	FCAR	0.02	0.00	0.05	0.12
2205	FCER1A	0.00	0.00	0.00	NaN
2206	MS4A1	0.00	0.00	0.05	-0.06
2207	FCER1G	0.00	0.00	0.00	NaN

2208	FCER2	0.02	0.00	0.15	0.02
2209	FCGR1A	0.02	0.00	0.00	0.04
2212	FCGR2A	0.00	0.00	0.00	NaN
2213	FCGR2B	0.00	0.00	0.00	NaN
2214	FCGR3A	0.00	0.00	0.00	NaN
2215	FCGR3B	0.00	0.00	0.00	NaN
2217	FCGRT	0.02	0.00	0.05	0.03
2222	FDFT1	0.00	0.00	0.00	NaN
2224	FDPS	0.04	0.00	0.15	0.20
2230	FDX1	0.02	0.00	0.05	0.27
2232	FDXR	0.04	0.00	0.00	0.03
2235	FECH	0.00	0.00	0.15	0.15
2237	FEN1	0.00	0.00	0.05	0.32
2241	FER	0.02	0.00	0.00	-0.03
2242	FES	0.02	0.00	0.10	-0.05
2243	FGA	0.00	0.00	0.00	NaN
2244	FGB	0.00	0.00	0.00	NaN
2247	FGF2	0.00	0.14	0.05	0.08
2248	FGF3	0.12	0.00	0.05	0.33
2250	FGF5	0.02	0.00	0.00	0.08
2251	FGF6	0.02	0.00	0.00	0.13
2252	FGF7	0.00	0.14	0.00	-0.10
2253	FGF8	0.02	0.00	0.00	0.31
2254	FGF9	0.18	0.00	0.20	0.00
2259	FGF14	0.00	0.00	0.00	NaN
2260	FGFR1	0.12	0.14	0.15	0.42
2261	FGFR3	0.02	0.00	0.00	0.09
2262	GPC5	0.02	0.00	0.00	-0.05
2263	FGFR2	0.02	0.00	0.00	0.05
2264	FGFR4	0.00	0.00	0.00	NaN
2266	FGG	0.00	0.00	0.00	NaN
2267	FGL1	0.00	0.00	0.00	NaN
2268	FGR	0.04	0.00	0.00	0.09
2271	FH	0.06	0.00	0.00	0.01
2272	FHIT	0.02	0.00	0.05	0.32
2274	FHL2	0.02	0.00	0.10	-0.02
2275	FHL3	0.04	0.00	0.05	0.03
2280	FKBP1A	0.00	0.00	0.00	NaN
2281	FKBP1B	0.00	0.00	0.00	NaN
2286	FKBP2	0.02	0.00	0.00	0.05
2287	FKBP3	0.00	0.00	0.10	0.07
2288	FKBP4	0.08	0.14	0.10	-0.04
2289	FKBP5	0.04	0.00	0.00	-0.08
2294	FOXF1	0.02	0.00	0.05	0.06
2297	FOXD1	0.06	0.00	0.00	-0.07
2299	FOXI1	0.02	0.00	0.00	-0.12
2301	FOXE3	0.02	0.00	0.05	-0.06

2302	FOXJ1	0.02	0.14	0.05	-0.09
2303	FOXC2	0.00	0.00	0.05	0.04
2304	FOXE1	0.04	0.00	0.00	-0.07
2305	FOXM1	0.08	0.14	0.10	0.02
2306	FOXD2	0.02	0.00	0.05	-0.11
2313	FLI1	0.02	0.00	0.00	0.05
2314	FLII	0.02	0.00	0.00	-0.08
2315	MLANA	0.02	0.00	0.00	-0.05
2317	FLNB	0.02	0.00	0.00	-0.04
2318	FLNC	0.00	0.00	0.05	0.00
2319	FLOT2	0.10	0.00	0.05	0.39
2322	FLT3	0.00	0.00	0.05	-0.08
2323	FLT3LG	0.02	0.00	0.05	-0.03
2326	FMO1	0.02	0.00	0.00	0.05
2327	FMO2	0.02	0.00	0.00	-0.01
2328	FMO3	0.02	0.00	0.00	0.16
2329	FMO4	0.02	0.00	0.00	0.15
2330	FMO5	0.02	0.00	0.00	0.00
2331	FMOD	0.00	0.00	0.00	NaN
2335	FN1	0.02	0.00	0.00	-0.05
2339	FNTA	0.14	0.14	0.10	-0.04
2342	FNTB	0.06	0.00	0.00	0.13
2346	FOLH1	0.00	0.00	0.00	NaN
2348	FOLR1	0.06	0.00	0.15	0.03
2350	FOLR2	0.06	0.00	0.15	0.01
2352	FOLR3	0.06	0.00	0.15	0.12
2353	FOS	0.00	0.00	0.00	NaN
2354	FOSB	0.00	0.00	0.00	NaN
2355	FOSL2	0.00	0.00	0.00	NaN
2356	FPGS	0.02	0.00	0.00	0.00
2357	FPR1	0.02	0.00	0.05	0.06
2444	FRK	0.06	0.00	0.05	0.03
2475	FRAP1	0.02	0.00	0.00	0.20
2483	FRG1	0.02	0.00	0.05	-0.09
2487	FRZB	0.00	0.00	0.00	NaN
2488	FSHB	0.04	0.00	0.00	-0.04
2492	FSHR	0.02	0.00	0.05	0.02
2494	NR5A2	0.06	0.14	0.15	-0.03
2495	FTH1	0.00	0.00	0.05	0.02
2512	FTL	0.06	0.00	0.05	0.23
2515	ADAM2	0.12	0.14	0.15	-0.04
2516	NR5A1	0.06	0.00	0.00	-0.16
2517	FUCA1	0.02	0.00	0.05	-0.01
2521	FUS	0.02	0.00	0.00	0.01
2523	FUT1	0.06	0.00	0.05	0.28
2524	FUT2	0.06	0.00	0.05	0.14
2525	FUT3	0.02	0.00	0.15	-0.05

2526	FUT4	0.00	0.00	0.00	NaN
2527	FUT5	0.02	0.00	0.15	-0.08
2528	FUT6	0.02	0.00	0.15	-0.01
2530	FUT8	0.06	0.00	0.00	-0.08
2533	FYB	0.04	0.00	0.00	0.27
2534	FYN	0.02	0.00	0.00	0.07
2535	FZD2	0.00	0.00	0.05	0.11
2538	G6PC	0.00	0.86	0.00	0.22
2548	GAA	0.00	0.14	0.00	-0.16
2549	GAB1	0.00	0.00	0.00	NaN
2550	GABBR1	0.04	0.14	0.05	-0.03
2551	GABPA	0.02	0.00	0.05	-0.05
2552	GABPB1	0.00	0.00	0.00	NaN
2553	GABPB2	0.02	0.00	0.10	-0.14
2554	GABRA1	0.00	0.00	0.00	NaN
2555	GABRA2	0.00	0.29	0.10	-0.06
2557	GABRA4	0.00	0.29	0.10	-0.09
2558	GABRA5	0.02	0.00	0.10	0.33
2559	GABRA6	0.00	0.00	0.00	NaN
2560	GABRB1	0.00	0.29	0.10	-0.04
2561	GABRB2	0.00	0.00	0.00	NaN
2562	GABRB3	0.02	0.00	0.10	0.01
2566	GABRG2	0.00	0.00	0.00	NaN
2567	GABRG3	0.02	0.00	0.10	0.07
2568	GABRP	0.02	0.00	0.00	-0.06
2569	GABRR1	0.00	0.00	0.05	-0.04
2570	GABRR2	0.00	0.00	0.05	0.03
2571	GAD1	0.00	0.00	0.00	NaN
2572	GAD2	0.00	0.00	0.00	NaN
2580	GAK	0.06	0.00	0.05	-0.01
2581	GALC	0.00	0.00	0.00	NaN
2582	GALE	0.02	0.00	0.05	0.03
2584	GALK1	0.02	0.14	0.05	-0.02
2585	GALK2	0.02	0.00	0.00	0.10
2586	GAL	0.02	0.00	0.05	0.35
2587	GALR1	0.02	0.00	0.00	-0.09
2588	GALNS	0.00	0.00	0.10	0.14
2589	GALNT1	0.04	0.00	0.05	0.17
2590	GALNT2	0.00	0.00	0.05	-0.22
2591	GALNT3	0.02	0.00	0.00	0.13
2592	GALT	0.00	0.00	0.00	NaN
2593	GAMT	0.02	0.00	0.05	-0.12
2596	GAP43	0.02	0.00	0.00	-0.05
2617	GARS	0.00	0.00	0.00	NaN
2618	GART	0.00	0.00	0.00	NaN
2619	GAS1	0.00	0.00	0.00	NaN
2620	GAS2	0.02	0.00	0.00	-0.04

2624	GATA2	0.04	0.00	0.00	0.13
2625	GATA3	0.00	0.00	0.20	-0.36
2626	GATA4	0.00	0.00	0.00	NaN
2627	GATA6	0.00	0.14	0.00	-0.01
2628	GATM	0.02	0.00	0.05	0.10
2629	GBA	0.04	0.00	0.15	0.01
2631	GBAS	0.04	0.14	0.20	-0.07
2632	GBE1	0.16	0.14	0.30	-0.15
2633	GBP1	0.04	0.00	0.05	0.12
2634	GBP2	0.04	0.00	0.05	0.13
2637	GBX2	0.00	0.00	0.00	NaN
2638	GC	0.02	0.00	0.05	-0.04
2639	GCDH	0.00	0.14	0.05	-0.05
2641	GCG	0.02	0.00	0.00	0.10
2643	GCH1	0.00	0.00	0.10	0.21
2644	GCHFR	0.00	0.00	0.00	NaN
2645	GCK	0.00	0.00	0.00	NaN
2646	GCKR	0.00	0.00	0.00	NaN
2649	NR6A1	0.06	0.00	0.00	0.24
2650	GCNT1	0.00	0.00	0.05	0.01
2651	GCNT2	0.04	0.00	0.00	0.00
2653	GCSH	0.02	0.00	0.00	-0.04
2657	GDF1	0.02	0.00	0.00	0.07
2658	GDF2	0.02	0.00	0.10	0.07
2661	GDF9	0.00	0.00	0.00	NaN
2662	GDF10	0.02	0.00	0.10	-0.10
2665	GDI2	0.00	0.00	0.00	NaN
2668	GDNF	0.04	0.00	0.15	-0.04
2669	GEM	0.08	0.00	0.05	-0.11
2670	GFAP	0.00	0.00	0.05	0.23
2671	GFER	0.18	0.00	0.10	-0.06
2672	GFI1	0.00	0.00	0.00	NaN
2673	GFPT1	0.00	0.14	0.00	0.10
2674	GFRA1	0.04	0.14	0.05	-0.14
2675	GFRA2	0.00	0.00	0.05	0.07
2676	GFRA3	0.02	0.00	0.00	0.01
2677	GGCX	0.00	0.00	0.00	NaN
2678	GGT1	0.00	0.14	0.05	0.12
2683	B4GALT1	0.00	0.00	0.05	0.03
2688	GH1	0.12	0.00	0.20	0.07
2689	GH2	0.12	0.00	0.20	0.06
2690	GHR	0.18	0.00	0.30	0.08
2691	GHRH	0.04	0.00	0.00	-0.03
2692	GHRHR	0.00	0.00	0.00	NaN
2693	GHSR	0.06	0.00	0.00	-0.01
2694	GIF	0.00	0.00	0.00	NaN
2695	GIP	0.04	0.00	0.05	-0.04

2696	GIPR	0.00	0.00	0.05	-0.07
2697	GJA1	0.08	0.00	0.00	-0.26
2701	GJA4	0.00	0.00	0.00	NaN
2702	GJA5	0.02	0.00	0.00	0.01
2703	GJA8	0.02	0.00	0.00	-0.06
2707	GJB3	0.00	0.00	0.00	NaN
2709	GJB5	0.02	0.00	0.00	-0.08
2720	GLB1	0.06	0.00	0.05	0.09
2729	GCLC	0.02	0.00	0.00	-0.04
2730	GCLM	0.00	0.00	0.00	NaN
2731	GLDC	0.02	0.00	0.00	0.23
2734	GLG1	0.00	0.00	0.00	NaN
2736	GLI2	0.00	0.00	0.10	0.04
2737	GLI3	0.00	0.00	0.00	NaN
2739	GLO1	0.00	0.00	0.00	NaN
2740	GLP1R	0.00	0.00	0.00	NaN
2741	GLRA1	0.00	0.14	0.00	0.38
2743	GLRB	0.00	0.14	0.00	-0.05
2744	GLS	0.00	0.00	0.05	-0.04
2745	GLRX	0.00	0.00	0.00	NaN
2746	GLUD1	0.00	0.00	0.05	0.10
2752	GLUL	0.02	0.00	0.00	0.02
2760	GM2A	0.00	0.14	0.00	0.08
2764	GMFB	0.00	0.00	0.05	-0.03
2765	GML	0.10	0.00	0.15	0.04
2766	GMPR	0.00	0.00	0.00	NaN
2767	GNA11	0.02	0.00	0.15	-0.09
2769	GNA15	0.02	0.00	0.15	0.01
2770	GNAI1	0.00	0.00	0.00	NaN
2771	GNAI2	0.00	0.00	0.05	-0.08
2773	GNAI3	0.00	0.00	0.00	NaN
2774	GNAL	0.04	0.00	0.10	0.07
2775	GNAO1	0.02	0.00	0.00	-0.05
2776	GNAQ	0.00	0.00	0.05	-0.04
2778	GNAS	0.12	0.00	0.00	0.43
2779	GNAT1	0.00	0.00	0.05	-0.05
2780	GNAT2	0.00	0.00	0.00	NaN
2781	GNAZ	0.06	0.14	0.15	0.22
2783	GNB2	0.00	0.00	0.00	NaN
2784	GNB3	0.02	0.00	0.15	0.07
2785	GNG3	0.00	0.00	0.05	-0.02
2786	GNG4	0.00	0.00	0.00	NaN
2787	GNG5	0.04	0.00	0.05	0.00
2788	GNG7	0.00	0.00	0.00	NaN
2790	GNG10	0.02	0.00	0.00	0.02
2791	GNG11	0.02	0.00	0.00	0.02
2792	GNGT1	0.02	0.00	0.00	-0.10

2794	GNL1	0.04	0.14	0.05	0.07
2796	GNRH1	0.02	0.00	0.00	-0.01
2797	GNRH2	0.00	0.00	0.00	NaN
2798	GNRHR	0.00	0.00	0.05	0.22
2799	GNS	0.06	0.00	0.00	0.26
2800	GOLGA1	0.06	0.00	0.00	0.06
2801	GOLGA2	0.00	0.00	0.00	NaN
2802	GOLGA3	0.08	0.14	0.05	-0.09
2803	GOLGA4	0.02	0.14	0.10	0.02
2804	GOLGB1	0.00	0.00	0.10	0.14
2805	GOT1	0.00	0.00	0.00	NaN
2806	GOT2	0.04	0.00	0.05	-0.06
2810	SFN	0.06	0.00	0.05	-0.08
2811	GP1BA	0.04	0.00	0.10	0.02
2812	GP1BB	0.06	0.14	0.15	0.06
2813	GP2	0.00	0.00	0.00	NaN
2814	GP5	0.02	0.00	0.00	-0.12
2815	GP9	0.02	0.00	0.00	0.06
2817	GPC1	0.08	0.43	0.10	0.01
2819	GPD1	0.02	0.00	0.00	-0.04
2820	GPD2	0.00	0.00	0.00	NaN
2821	GPI	0.00	0.00	0.00	NaN
2822	GPLD1	0.00	0.00	0.00	NaN
2823	GPM6A	0.00	0.14	0.00	-0.03
2825	GPR1	0.00	0.00	0.00	NaN
2827	GPR3	0.04	0.00	0.00	0.26
2828	GPR4	0.00	0.00	0.05	-0.10
2835	GPR12	0.00	0.00	0.05	-0.10
2838	GPR15	0.16	0.14	0.30	-0.02
2840	GPR17	0.00	0.00	0.10	0.23
2841	GPR18	0.00	0.00	0.05	-0.07
2842	GPR19	0.04	0.00	0.05	-0.04
2843	GPR20	0.10	0.00	0.15	-0.03
2844	GPR21	0.06	0.00	0.00	0.08
2845	GPR22	0.00	0.00	0.00	NaN
2848	GPR25	0.00	0.00	0.05	-0.12
2850	GPR27	0.02	0.00	0.00	-0.06
2853	GPR31	0.02	0.00	0.05	-0.21
2854	GPR32	0.00	0.00	0.00	NaN
2859	GPR35	0.08	0.43	0.10	-0.09
2861	GPR37	0.00	0.00	0.00	NaN
2863	GPR39	0.00	0.00	0.10	0.34
2873	GPS1	0.00	0.00	0.00	NaN
2874	GPS2	0.02	0.00	0.00	-0.16
2875	GPT	0.10	0.00	0.15	0.31
2876	GPX1	0.06	0.00	0.05	0.15
2877	GPX2	0.06	0.00	0.00	0.39

2878	GPX3	0.00	0.14	0.00	0.03
2879	GPX4	0.02	0.00	0.05	0.07
2880	GPX5	0.04	0.14	0.05	-0.13
2885	GRB2	0.02	0.14	0.00	0.36
2886	GRB7	0.02	0.86	0.05	0.70
2887	GRB10	0.00	0.00	0.00	NaN
2888	GRB14	0.02	0.00	0.00	-0.06
2890	GRIA1	0.02	0.00	0.00	0.06
2891	GRIA2	0.04	0.00	0.00	0.32
2893	GRIA4	0.02	0.00	0.05	-0.11
2895	GRID2	0.02	0.00	0.00	0.00
2896	GRN	0.00	0.00	0.00	NaN
2897	GRIK1	0.18	0.14	0.00	0.05
2899	GRIK3	0.04	0.00	0.05	-0.09
2900	GRIK4	0.00	0.00	0.00	NaN
2901	GRIK5	0.00	0.00	0.05	0.36
2903	GRIN2A	0.00	0.00	0.00	NaN
2904	GRIN2B	0.04	0.00	0.00	0.05
2905	GRIN2C	0.04	0.00	0.00	-0.01
2906	GRIN2D	0.02	0.00	0.00	0.12
2908	NR3C1	0.00	0.00	0.00	NaN
2909	GRLF1	0.00	0.00	0.05	0.08
2911	GRM1	0.02	0.00	0.05	0.02
2912	GRM2	0.00	0.00	0.10	-0.11
2913	GRM3	0.00	0.00	0.00	NaN
2914	GRM4	0.00	0.00	0.00	NaN
2915	GRM5	0.00	0.00	0.00	NaN
2916	GRM6	0.08	0.43	0.10	0.12
2917	GRM7	0.00	0.00	0.05	0.43
2918	GRM8	0.02	0.00	0.00	-0.04
2922	GRP	0.00	0.00	0.05	-0.05
2926	GRSF1	0.02	0.00	0.05	0.08
2931	GSK3A	0.00	0.00	0.05	0.32
2932	GSK3B	0.00	0.00	0.10	-0.04
2934	GSN	0.06	0.00	0.00	0.07
2935	GSPT1	0.00	0.00	0.00	NaN
2936	GSR	0.04	0.00	0.00	0.10
2937	GSS	0.00	0.00	0.10	-0.18
2939	GSTA2	0.02	0.00	0.00	0.01
2940	GSTA3	0.02	0.00	0.00	-0.02
2941	GSTA4	0.02	0.00	0.00	0.12
2944	GSTM1	0.00	0.00	0.00	NaN
2946	GSTM2	0.00	0.00	0.00	NaN
2947	GSTM3	0.00	0.00	0.00	NaN
2948	GSTM4	0.00	0.00	0.00	NaN
2949	GSTM5	0.00	0.00	0.00	NaN
2950	GSTP1	0.04	0.00	0.05	0.20

2952	GSTT1	0.00	0.14	0.05	-0.08
2953	GSTT2	0.00	0.14	0.05	-0.04
2954	GSTZ1	0.00	0.00	0.00	NaN
2956	MSH6	0.02	0.00	0.05	0.34
2957	GTF2A1	0.02	0.00	0.00	-0.13
2958	GTF2A2	0.00	0.00	0.00	NaN
2960	GTF2E1	0.00	0.00	0.10	0.01
2961	GTF2E2	0.04	0.00	0.00	-0.08
2962	GTF2F1	0.02	0.00	0.15	0.12
2963	GTF2F2	0.00	0.00	0.00	NaN
2965	GTF2H1	0.00	0.00	0.05	0.02
2967	GTF2H3	0.02	0.00	0.10	0.12
2968	GTF2H4	0.04	0.14	0.05	-0.10
2969	GTF2I	0.00	0.00	0.00	NaN
2971	GTF3A	0.00	0.00	0.05	0.06
2972	BRF1	0.02	0.00	0.10	0.02
2974	GUCY1B2	0.04	0.00	0.00	0.38
2975	GTF3C1	0.06	0.14	0.00	-0.01
2976	GTF3C2	0.00	0.00	0.00	NaN
2977	GUCY1A2	0.04	0.00	0.00	-0.14
2979	GUCA1B	0.06	0.00	0.05	0.08
2981	GUCA2B	0.02	0.00	0.05	0.02
2982	GUCY1A3	0.00	0.14	0.00	0.21
2983	GUCY1B3	0.00	0.14	0.00	0.11
2984	GUCY2C	0.04	0.00	0.05	0.01
2987	GUK1	0.00	0.00	0.05	-0.01
2990	GUSB	0.00	0.00	0.00	NaN
2993	GYPA	0.00	0.00	0.00	NaN
2994	GYPB	0.00	0.00	0.00	NaN
2995	GYPC	0.00	0.00	0.10	0.14
2996	GYPE	0.00	0.00	0.00	NaN
2997	GYS1	0.06	0.00	0.05	-0.05
2998	GYS2	0.02	0.00	0.05	-0.14
3000	GUCY2D	0.00	0.00	0.00	NaN
3001	GZMA	0.00	0.14	0.05	-0.01
3003	GZMK	0.00	0.14	0.05	-0.03
3004	GZMM	0.06	0.00	0.15	0.01
3005	H1F0	0.00	0.14	0.00	0.02
3014	H2AFX	0.00	0.00	0.05	0.20
3015	H2AFZ	0.00	0.00	0.00	NaN
3021	H3F3B	0.02	0.14	0.05	0.18
3026	HABP2	0.00	0.14	0.05	0.21
3029	HAGH	0.18	0.00	0.10	0.07
3030	HADHA	0.00	0.00	0.00	NaN
3032	HADHB	0.00	0.00	0.00	NaN
3034	HAL	0.02	0.00	0.00	-0.01
3035	HARS	0.00	0.00	0.00	NaN

3036	HAS1	0.02	0.00	0.05	0.00
3037	HAS2	0.10	0.00	0.15	0.22
3039	HBA1	0.18	0.00	0.10	0.08
3040	HBA2	0.18	0.00	0.10	0.06
3043	HBB	0.00	0.00	0.05	-0.12
3045	HBD	0.00	0.00	0.05	0.01
3046	HBE1	0.00	0.00	0.00	NaN
3047	HBG1	0.00	0.00	0.05	-0.05
3049	HBQ1	0.18	0.00	0.10	-0.03
3050	HBZ	0.18	0.00	0.10	-0.09
3053	SERPIND1	0.06	0.14	0.15	-0.16
3055	HCK	0.04	0.00	0.15	-0.04
3059	HCLS1	0.00	0.00	0.10	0.23
3060	HCRT	0.02	0.86	0.05	0.21
3061	HCRTR1	0.00	0.14	0.00	-0.15
3062	HCRTR2	0.02	0.00	0.00	-0.08
3065	HDAC1	0.00	0.14	0.05	0.06
3066	HDAC2	0.04	0.00	0.00	0.21
3067	HDC	0.00	0.00	0.00	NaN
3068	HDGF	0.04	0.14	0.05	-0.02
3069	HDLBP	0.08	0.43	0.10	0.08
3073	HEXA	0.04	0.00	0.05	0.30
3074	HEXB	0.06	0.00	0.00	-0.29
3077	HFE	0.00	0.14	0.00	0.40
3081	HGD	0.00	0.00	0.10	-0.08
3082	HGF	0.02	0.00	0.00	-0.02
3083	HGFAC	0.00	0.00	0.00	NaN
3084	NRG1	0.04	0.00	0.05	0.78
3087	HHEX	0.00	0.00	0.15	-0.11
3090	HIC1	0.04	0.00	0.05	-0.06
3091	HIF1A	0.00	0.14	0.00	0.19
3092	HIP1	0.00	0.00	0.00	NaN
3094	HINT1	0.00	0.00	0.00	NaN
3096	HIVEP1	0.04	0.00	0.00	-0.15
3098	HK1	0.00	0.00	0.00	NaN
3099	HK2	0.00	0.00	0.05	-0.11
3101	HK3	0.00	0.00	0.00	NaN
3105	HLA-A	0.04	0.14	0.05	0.04
3106	HLA-B	0.04	0.14	0.05	0.03
3107	HLA-C	0.04	0.14	0.05	0.05
3109	HLA-DMB	0.02	0.00	0.05	-0.04
3111	HLA-DOA	0.02	0.00	0.05	0.08
3112	HLA-DOB	0.02	0.00	0.05	-0.07
3113	HLA-DPA1	0.02	0.00	0.05	0.07
3115	HLA-DPB1	0.02	0.00	0.05	0.02
3117	HLA-DQA1	0.02	0.00	0.05	0.17
3119	HLA-DQB1	0.02	0.00	0.05	0.06

3122	HLA-DRA	0.02	0.00	0.05	-0.06
3123	HLA-DRB1	0.02	0.00	0.05	0.01
3127	HLA-DRB5	0.02	0.00	0.05	0.04
3131	HLF	0.02	0.00	0.10	-0.06
3134	HLA-F	0.04	0.14	0.05	0.04
3135	HLA-G	0.04	0.14	0.05	0.03
3141	HLCS	0.00	0.00	0.00	NaN
3145	HMBS	0.00	0.00	0.05	0.26
3155	HMGCL	0.02	0.00	0.05	-0.04
3156	HMGCR	0.04	0.00	0.05	0.19
3157	HMGCS1	0.18	0.00	0.30	0.25
3158	HMGCS2	0.41	0.43	0.10	0.12
3161	HMMR	0.00	0.00	0.00	NaN
3162	HMOX1	0.00	0.29	0.05	-0.11
3163	HMOX2	0.00	0.00	0.00	NaN
3164	NR4A1	0.00	0.00	0.00	NaN
3172	HNF4A	0.02	0.00	0.00	0.45
3174	HNF4G	0.06	0.00	0.00	0.02
3175	ONECUT1	0.00	0.00	0.00	NaN
3176	HNMT	0.02	0.00	0.00	0.03
3177	SLC29A2	0.04	0.00	0.05	-0.16
3198	HOXA1	0.00	0.00	0.00	NaN
3199	HOXA2	0.00	0.00	0.00	NaN
3200	HOXA3	0.00	0.00	0.00	NaN
3201	HOXA4	0.00	0.00	0.00	NaN
3202	HOXA5	0.00	0.00	0.00	NaN
3203	HOXA6	0.00	0.00	0.00	NaN
3204	HOXA7	0.00	0.00	0.00	NaN
3205	HOXA9	0.00	0.00	0.00	NaN
3206	HOXA10	0.00	0.00	0.00	NaN
3207	HOXA11	0.00	0.00	0.00	NaN
3208	HPCA	0.00	0.00	0.00	NaN
3211	HOXB1	0.04	0.00	0.05	0.05
3212	HOXB2	0.04	0.00	0.05	0.07
3213	HOXB3	0.04	0.00	0.05	0.01
3215	HOXB5	0.04	0.00	0.05	-0.03
3216	HOXB6	0.04	0.00	0.05	-0.09
3217	HOXB7	0.04	0.00	0.05	-0.09
3218	HOXB8	0.04	0.00	0.05	-0.07
3221	HOXC4	0.02	0.00	0.05	-0.17
3222	HOXC5	0.02	0.00	0.05	-0.11
3223	HOXC6	0.02	0.00	0.05	-0.17
3224	HOXC8	0.02	0.00	0.05	-0.14
3226	HOXC10	0.02	0.00	0.05	-0.18
3227	HOXC11	0.02	0.00	0.05	-0.10
3229	HOXC13	0.02	0.00	0.05	-0.12
3231	HOXD1	0.02	0.00	0.00	0.23

3232	HOXD3	0.02	0.00	0.00	-0.06
3233	HOXD4	0.02	0.00	0.00	0.14
3235	HOXD9	0.02	0.00	0.00	0.22
3236	HOXD10	0.02	0.00	0.00	-0.07
3237	HOXD11	0.02	0.00	0.00	-0.11
3238	HOXD12	0.02	0.00	0.00	-0.13
3239	HOXD13	0.02	0.00	0.00	0.10
3240	HP	0.00	0.00	0.05	-0.09
3241	HPCAL1	0.00	0.00	0.00	NaN
3242	HPD	0.02	0.00	0.10	0.04
3248	HPGD	0.00	0.00	0.00	NaN
3249	HPN	0.00	0.00	0.00	NaN
3250	HPR	0.00	0.00	0.05	0.03
3257	HPS1	0.00	0.00	0.05	0.37
3263	HPX	0.00	0.00	0.00	NaN
3265	HRAS	0.00	0.00	0.05	-0.03
3269	HRH1	0.02	0.00	0.05	0.38
3270	HRC	0.06	0.00	0.05	0.16
3273	HRG	0.02	0.14	0.00	0.07
3274	HRH2	0.00	0.00	0.00	NaN
3281	HSBP1	0.00	0.00	0.00	NaN
3283	HSD3B1	0.41	0.43	0.10	0.08
3284	HSD3B2	0.41	0.43	0.10	0.08
3290	HSD11B1	0.02	0.00	0.00	-0.04
3291	HSD11B2	0.02	0.00	0.00	-0.04
3292	HSD17B1	0.02	0.86	0.05	0.27
3293	HSD17B3	0.00	0.00	0.00	NaN
3294	HSD17B2	0.02	0.00	0.00	-0.03
3295	HSD17B4	0.00	0.00	0.00	NaN
3297	HSF1	0.10	0.00	0.15	0.40
3298	HSF2	0.08	0.00	0.00	0.05
3299	HSF4	0.02	0.00	0.00	0.06
3300	DNAJB2	0.04	0.00	0.00	-0.11
3301	DNAJA1	0.00	0.00	0.05	-0.05
3303	HSPA1A	0.02	0.00	0.05	0.02
3304	HSPA1B	0.02	0.00	0.05	-0.04
3305	HSPA1L	0.02	0.00	0.05	-0.02
3306	HSPA2	0.06	0.00	0.00	0.09
3308	HSPA4	0.00	0.00	0.00	NaN
3309	HSPA5	0.06	0.00	0.00	-0.12
3310	HSPA6	0.00	0.00	0.00	NaN
3312	HSPA8	0.00	0.00	0.00	NaN
3315	HSPB1	0.00	0.00	0.00	NaN
3316	HSPB2	0.02	0.00	0.00	-0.08
3321	IGSF3	0.08	0.00	0.10	-0.08
3329	HSPD1	0.00	0.00	0.05	-0.04
3336	HSPE1	0.00	0.00	0.05	-0.07

3337	DNAJB1	0.00	0.00	0.05	0.14
3338	DNAJC4	0.02	0.00	0.00	0.08
3339	HSPG2	0.00	0.00	0.05	0.04
3340	NDST1	0.00	0.14	0.00	0.03
3346	HTN1	0.00	0.14	0.00	0.08
3347	HTN3	0.00	0.14	0.00	-0.04
3350	HTR1A	0.04	0.00	0.05	0.08
3351	HTR1B	0.10	0.00	0.20	0.11
3352	HTR1D	0.02	0.00	0.05	-0.05
3354	HTR1E	0.00	0.00	0.05	0.29
3355	HTR1F	0.16	0.14	0.30	-0.12
3356	HTR2A	0.00	0.00	0.00	NaN
3357	HTR2B	0.02	0.00	0.15	0.05
3359	HTR3A	0.00	0.00	0.00	NaN
3360	HTR4	0.02	0.14	0.00	0.27
3361	HTR5A	0.00	0.14	0.00	-0.09
3363	HTR7	0.02	0.00	0.05	0.00
3364	HUS1	0.00	0.14	0.00	0.07
3373	HYAL1	0.00	0.00	0.10	0.17
3375	IAPP	0.02	0.00	0.05	-0.07
3376	IARS	0.04	0.00	0.05	0.29
3381	IBSP	0.02	0.00	0.00	0.00
3382	ICA1	0.00	0.00	0.00	NaN
3383	ICAM1	0.02	0.00	0.15	0.28
3384	ICAM2	0.12	0.00	0.20	0.22
3385	ICAM3	0.02	0.00	0.15	0.12
3386	ICAM4	0.02	0.00	0.15	0.08
3396	ICT1	0.04	0.00	0.00	-0.03
3397	ID1	0.04	0.00	0.15	0.03
3398	ID2	0.00	0.00	0.00	NaN
3399	ID3	0.02	0.00	0.05	-0.08
3400	ID4	0.04	0.00	0.10	0.12
3416	IDE	0.00	0.00	0.15	0.21
3417	IDH1	0.02	0.00	0.05	0.03
3418	IDH2	0.00	0.00	0.00	NaN
3419	IDH3A	0.00	0.00	0.05	0.13
3420	IDH3B	0.00	0.00	0.00	NaN
3422	IDI1	0.00	0.00	0.10	0.39
3425	IDUA	0.06	0.00	0.05	-0.03
3428	IFI16	0.00	0.00	0.00	NaN
3429	IFI27	0.00	0.00	0.00	NaN
3430	IFI35	0.00	0.86	0.00	-0.03
3431	SP110	0.02	0.00	0.15	0.04
3434	IFIT1	0.02	0.00	0.05	0.00
3439	IFNA1	0.02	0.00	0.05	-0.08
3440	IFNA2	0.02	0.00	0.05	0.07
3441	IFNA4	0.02	0.00	0.05	-0.11

3442	IFNA5	0.02	0.00	0.05	-0.01
3443	IFNA6	0.02	0.00	0.05	-0.01
3444	IFNA7	0.02	0.00	0.05	-0.10
3445	IFNA8	0.02	0.00	0.05	0.03
3446	IFNA10	0.02	0.00	0.05	-0.10
3447	IFNA13	0.02	0.00	0.05	-0.10
3448	IFNA14	0.02	0.00	0.05	-0.01
3449	IFNA16	0.02	0.00	0.05	-0.06
3451	IFNA17	0.02	0.00	0.05	-0.14
3452	IFNA21	0.02	0.00	0.05	-0.03
3454	IFNAR1	0.00	0.00	0.00	NaN
3455	IFNAR2	0.00	0.00	0.00	NaN
3456	IFNB1	0.02	0.00	0.05	-0.16
3458	IFNG	0.08	0.14	0.05	-0.02
3459	IFNGR1	0.02	0.00	0.05	-0.07
3460	IFNGR2	0.00	0.00	0.00	NaN
3467	IFNW1	0.02	0.00	0.05	-0.12
3475	IFRD1	0.00	0.00	0.00	NaN
3479	IGF1	0.00	0.00	0.00	NaN
3480	IGF1R	0.16	0.00	0.10	0.18
3481	IGF2	0.02	0.00	0.05	0.27
3482	IGF2R	0.02	0.00	0.05	0.24
3483	IGFALS	0.18	0.00	0.10	0.07
3484	IGFBP1	0.02	0.00	0.00	-0.01
3485	IGFBP2	0.02	0.00	0.00	0.04
3486	IGFBP3	0.00	0.00	0.00	NaN
3487	IGFBP4	0.02	0.86	0.05	0.00
3488	IGFBP5	0.02	0.00	0.00	0.36
3489	IGFBP6	0.00	0.00	0.00	NaN
3490	IGFBP7	0.06	0.00	0.00	0.22
3491	CYR61	0.04	0.00	0.05	0.02
3508	IGHMBP2	0.02	0.00	0.05	0.28
3543	IGLL1	0.00	0.14	0.00	0.08
3549	IHH	0.04	0.00	0.00	0.01
3550	IK	0.00	0.00	0.00	NaN
3551	IKBKB	0.04	0.14	0.05	0.31
3552	IL1A	0.00	0.00	0.00	NaN
3553	IL1B	0.00	0.00	0.00	NaN
3554	IL1R1	0.02	0.00	0.10	0.14
3556	IL1RAP	0.00	0.00	0.05	-0.08
3557	IL1RN	0.00	0.00	0.00	NaN
3558	IL2	0.00	0.14	0.05	0.09
3559	IL2RA	0.00	0.00	0.00	NaN
3560	IL2RB	0.00	0.14	0.00	0.11
3562	IL3	0.02	0.00	0.00	0.09
3565	IL4	0.00	0.00	0.00	NaN
3566	IL4R	0.06	0.14	0.00	0.12

3567	IL5	0.02	0.14	0.00	0.05
3568	IL5RA	0.00	0.00	0.05	0.06
3569	IL6	0.00	0.00	0.10	0.04
3570	IL6R	0.04	0.00	0.15	0.13
3572	IL6ST	0.00	0.14	0.05	-0.07
3574	IL7	0.02	0.00	0.00	-0.01
3575	IL7R	0.04	0.00	0.15	0.14
3576	IL8	0.00	0.00	0.00	NaN
3577	IL8RA	0.04	0.00	0.00	-0.19
3578	IL9	0.02	0.00	0.00	0.32
3579	IL8RB	0.04	0.00	0.00	0.04
3586	IL10	0.00	0.00	0.05	0.12
3587	IL10RA	0.00	0.00	0.00	NaN
3588	IL10RB	0.00	0.00	0.00	NaN
3589	IL11	0.02	0.00	0.05	0.07
3590	IL11RA	0.00	0.00	0.00	NaN
3592	IL12A	0.00	0.00	0.00	NaN
3593	IL12B	0.00	0.00	0.00	NaN
3594	IL12RB1	0.04	0.00	0.00	-0.12
3595	IL12RB2	0.00	0.00	0.05	0.29
3596	IL13	0.00	0.00	0.00	NaN
3600	IL15	0.00	0.00	0.00	NaN
3601	IL15RA	0.00	0.00	0.00	NaN
3603	IL16	0.02	0.00	0.00	-0.02
3604	TNFRSF9	0.00	0.00	0.00	NaN
3606	IL18	0.02	0.00	0.00	-0.08
3608	ILF2	0.02	0.00	0.10	0.10
3609	ILF3	0.02	0.00	0.15	0.06
3611	ILK	0.00	0.00	0.00	NaN
3612	IMPA1	0.06	0.00	0.15	-0.02
3613	IMPA2	0.04	0.00	0.10	0.22
3614	IMPDH1	0.02	0.00	0.00	-0.03
3615	IMPDH2	0.00	0.00	0.05	-0.13
3617	IMPG1	0.10	0.00	0.20	-0.04
3619	INCENP	0.00	0.00	0.05	-0.06
3621	ING1	0.00	0.00	0.05	0.18
3623	INHA	0.04	0.00	0.00	-0.04
3624	INHBA	0.00	0.00	0.00	NaN
3625	INHBB	0.00	0.00	0.10	0.23
3628	INPP1	0.00	0.00	0.05	-0.06
3630	INS	0.02	0.00	0.05	0.52
3631	INPP4A	0.02	0.00	0.10	0.34
3632	INPP5A	0.00	0.00	0.00	NaN
3633	INPP5B	0.04	0.00	0.05	-0.04
3635	INPP5D	0.00	0.00	0.05	0.02
3636	INPPL1	0.06	0.00	0.15	0.03
3638	INSIG1	0.00	0.14	0.00	0.10

3640	INSL3	0.04	0.00	0.00	0.04
3641	INSL4	0.02	0.00	0.00	-0.13
3642	INSM1	0.02	0.00	0.00	-0.03
3643	INSR	0.02	0.00	0.15	0.13
3645	INSRR	0.04	0.14	0.05	0.18
3652	IPP	0.02	0.00	0.05	0.21
3653	IPW	0.02	0.00	0.05	-0.11
3655	ITGA6	0.00	0.00	0.00	NaN
3658	IREB2	0.00	0.00	0.05	-0.19
3659	IRF1	0.02	0.00	0.00	0.08
3660	IRF2	0.00	0.00	0.00	NaN
3661	IRF3	0.02	0.00	0.05	0.04
3663	IRF5	0.00	0.00	0.05	-0.06
3665	IRF7	0.02	0.00	0.05	-0.01
3667	IRS1	0.02	0.00	0.15	-0.16
3669	ISG20	0.00	0.00	0.00	NaN
3670	ISL1	0.18	0.00	0.30	-0.09
3671	ISLR	0.00	0.00	0.00	NaN
3673	ITGA2	0.18	0.00	0.30	-0.29
3674	ITGA2B	0.00	0.00	0.00	NaN
3675	ITGA3	0.06	0.00	0.00	0.05
3676	ITGA4	0.00	0.00	0.00	NaN
3678	ITGA5	0.02	0.00	0.05	0.10
3679	ITGA7	0.02	0.00	0.05	0.09
3680	ITGA9	0.02	0.14	0.10	-0.01
3682	ITGAE	0.04	0.00	0.10	0.08
3684	ITGAM	0.02	0.00	0.00	-0.02
3685	ITGAV	0.00	0.00	0.05	0.02
3687	ITGAX	0.02	0.00	0.00	-0.08
3688	ITGB1	0.02	0.14	0.00	0.35
3689	ITGB2	0.00	0.00	0.05	-0.03
3690	ITGB3	0.00	0.00	0.00	NaN
3691	ITGB4	0.02	0.14	0.00	0.24
3693	ITGB5	0.00	0.00	0.10	-0.06
3694	ITGB6	0.00	0.00	0.05	-0.09
3695	ITGB7	0.00	0.00	0.00	NaN
3696	ITGB8	0.00	0.00	0.00	NaN
3698	ITIH2	0.00	0.00	0.00	NaN
3699	ITIH3	0.00	0.00	0.10	0.12
3700	ITIH4	0.00	0.00	0.10	0.03
3702	ITK	0.00	0.00	0.00	NaN
3704	ITPA	0.02	0.00	0.00	-0.12
3705	ITPK1	0.02	0.00	0.00	0.29
3706	ITPKA	0.00	0.00	0.00	NaN
3707	ITPKB	0.00	0.00	0.05	0.18
3708	ITPR1	0.00	0.00	0.05	-0.08
3709	ITPR2	0.00	0.00	0.00	NaN

3710	ITPR3	0.02	0.00	0.05	0.18
3712	IVD	0.00	0.00	0.00	NaN
3713	IVL	0.02	0.00	0.10	0.00
3714	JAG2	0.02	0.00	0.10	0.18
3716	JAK1	0.02	0.00	0.15	-0.19
3717	JAK2	0.00	0.00	0.00	NaN
3718	JAK3	0.04	0.00	0.00	0.06
3725	JUN	0.06	0.00	0.00	0.32
3726	JUNB	0.00	0.14	0.05	0.00
3727	JUND	0.02	0.00	0.00	0.06
3728	JUP	0.02	0.86	0.05	0.24
3735	KARS	0.00	0.00	0.00	NaN
3736	KCNA1	0.02	0.00	0.00	-0.08
3737	KCNA2	0.06	0.14	0.00	0.01
3738	KCNA3	0.06	0.14	0.00	-0.01
3739	KCNA4	0.04	0.00	0.00	-0.05
3741	KCNA5	0.02	0.00	0.00	0.08
3742	KCNA6	0.02	0.00	0.00	0.01
3744	KCNA10	0.06	0.14	0.00	-0.02
3745	KCNB1	0.06	0.00	0.00	0.17
3746	KCNC1	0.00	0.00	0.00	NaN
3748	KCNC3	0.00	0.00	0.00	NaN
3749	KCNC4	0.00	0.00	0.00	NaN
3751	KCND2	0.00	0.00	0.15	0.01
3752	KCND3	0.00	0.00	0.00	NaN
3753	KCNE1	0.02	0.00	0.05	-0.03
3754	KCNF1	0.00	0.00	0.00	NaN
3755	KCNG1	0.10	0.00	0.00	0.03
3756	KCNH1	0.02	0.00	0.00	0.03
3757	KCNH2	0.02	0.00	0.00	-0.05
3758	KCNJ1	0.02	0.00	0.00	0.08
3759	KCNJ2	0.10	0.00	0.15	-0.12
3760	KCNJ3	0.00	0.00	0.00	NaN
3761	KCNJ4	0.00	0.14	0.00	0.09
3762	KCNJ5	0.02	0.00	0.00	-0.01
3763	KCNJ6	0.00	0.00	0.00	NaN
3764	KCNJ8	0.02	0.00	0.05	0.13
3765	KCNJ9	0.00	0.00	0.00	NaN
3766	KCNJ10	0.00	0.00	0.00	NaN
3768	KCNJ12	0.10	0.00	0.05	-0.03
3769	KCNJ13	0.00	0.00	0.05	-0.01
3770	KCNJ14	0.02	0.00	0.00	-0.11
3772	KCNJ15	0.00	0.00	0.00	NaN
3773	KCNJ16	0.10	0.00	0.15	-0.09
3775	KCNK1	0.00	0.00	0.00	NaN
3776	KCNK2	0.00	0.00	0.00	NaN
3777	KCNK3	0.00	0.00	0.00	NaN

3778	KCNMA1	0.00	0.00	0.05	-0.07
3779	KCNMB1	0.02	0.00	0.00	-0.10
3780	KCNN1	0.04	0.00	0.00	0.03
3781	KCNN2	0.02	0.00	0.00	-0.03
3782	KCNN3	0.04	0.00	0.15	-0.05
3783	KCNN4	0.00	0.00	0.00	NaN
3784	KCNQ1	0.00	0.00	0.00	NaN
3785	KCNQ2	0.08	0.00	0.05	0.06
3786	KCNQ3	0.06	0.00	0.00	-0.07
3787	KCNS1	0.04	0.00	0.00	0.19
3790	KCNS3	0.00	0.00	0.05	0.05
3791	KDR	0.00	0.00	0.10	-0.23
3792	KEL	0.00	0.14	0.05	-0.04
3795	KHK	0.00	0.00	0.00	NaN
3797	KIF3C	0.00	0.00	0.00	NaN
3798	KIF5A	0.02	0.14	0.00	0.08
3799	KIF5B	0.02	0.14	0.00	0.26
3800	KIF5C	0.00	0.00	0.00	NaN
3801	KIFC3	0.02	0.00	0.00	-0.03
3802	KIR2DL1	0.02	0.00	0.05	-0.04
3805	KIR2DL4	0.02	0.00	0.05	0.01
3809	KIR2DS4	0.02	0.00	0.05	0.15
3811	KIR3DL1	0.02	0.00	0.05	-0.08
3812	KIR3DL2	0.02	0.00	0.05	0.00
3814	KISS1	0.00	0.00	0.15	-0.06
3815	KIT	0.00	0.00	0.10	0.24
3817	KLK2	0.00	0.00	0.00	NaN
3818	KLKB1	0.00	0.00	0.00	NaN
3820	KLRB1	0.02	0.00	0.15	0.05
3822	KLRC2	0.02	0.00	0.15	-0.01
3823	KLRC3	0.02	0.00	0.15	0.06
3824	KLRD1	0.02	0.00	0.15	0.10
3836	KPNA1	0.00	0.00	0.10	0.17
3837	KPNB1	0.00	0.00	0.00	NaN
3838	KPNA2	0.12	0.00	0.20	0.18
3839	KPNA3	0.04	0.00	0.00	-0.18
3840	KPNA4	0.00	0.00	0.00	NaN
3841	KPNA5	0.06	0.00	0.05	0.37
3848	KRT1	0.00	0.00	0.00	NaN
3851	KRT4	0.00	0.00	0.00	NaN
3852	KRT5	0.00	0.00	0.00	NaN
3853	KRT6A	0.00	0.00	0.00	NaN
3854	KRT6B	0.00	0.00	0.00	NaN
3855	KRT7	0.00	0.00	0.00	NaN
3856	KRT8	0.00	0.00	0.00	NaN
3857	KRT9	0.02	0.86	0.05	0.39
3858	KRT10	0.02	0.86	0.05	-0.06

3859	KRT12	0.02	0.86	0.05	0.26
3860	KRT13	0.02	0.86	0.05	0.00
3861	KRT14	0.02	0.86	0.05	0.11
3866	KRT15	0.02	0.86	0.05	0.17
3868	KRT16	0.02	0.86	0.05	0.15
3872	KRT17	0.02	0.86	0.05	0.25
3875	KRT18	0.00	0.00	0.00	NaN
3880	KRT19	0.02	0.86	0.05	-0.08
3895	KTN1	0.04	0.00	0.10	0.11
3898	LAD1	0.00	0.00	0.05	0.17
3902	LAG3	0.02	0.00	0.15	-0.01
3903	LAIR1	0.02	0.00	0.05	0.08
3904	LAIR2	0.02	0.00	0.05	-0.11
3906	LALBA	0.04	0.00	0.05	-0.06
3908	LAMA2	0.02	0.00	0.10	-0.05
3909	LAMA3	0.00	0.00	0.00	NaN
3910	LAMA4	0.02	0.00	0.05	-0.02
3911	LAMA5	0.08	0.00	0.05	0.03
3912	LAMB1	0.00	0.00	0.00	NaN
3913	LAMB2	0.00	0.00	0.05	-0.07
3914	LAMB3	0.02	0.00	0.00	0.02
3915	LAMC1	0.02	0.00	0.00	0.00
3916	LAMP1	0.06	0.00	0.00	-0.01
3918	LAMC2	0.02	0.00	0.00	-0.04
3925	STMN1	0.02	0.00	0.05	0.02
3927	LASP1	0.02	0.86	0.05	0.06
3929	LBP	0.02	0.00	0.00	-0.04
3930	LBR	0.00	0.00	0.05	0.12
3931	LCAT	0.02	0.00	0.00	0.18
3932	LCK	0.00	0.14	0.05	0.17
3934	LCN2	0.00	0.00	0.00	NaN
3937	LCP2	0.02	0.00	0.00	0.09
3938	LCT	0.00	0.00	0.00	NaN
3939	LDHA	0.00	0.00	0.05	-0.08
3945	LDHB	0.02	0.00	0.05	-0.10
3948	LDHC	0.00	0.00	0.05	-0.03
3949	LDLR	0.02	0.00	0.00	0.07
3950	LECT2	0.02	0.00	0.00	-0.08
3952	LEP	0.02	0.00	0.00	0.08
3953	LEPR	0.02	0.00	0.15	0.01
3954	LETM1	0.02	0.00	0.00	-0.03
3955	LFNG	0.00	0.00	0.00	NaN
3956	LGALS1	0.00	0.14	0.00	-0.04
3957	LGALS2	0.00	0.14	0.00	0.17
3958	LGALS3	0.00	0.00	0.10	0.16
3959	LGALS3BP	0.02	0.14	0.00	0.19
3960	LGALS4	0.06	0.00	0.05	0.15

3963	LGALS7	0.06	0.00	0.05	-0.06
3964	LGALS8	0.00	0.00	0.00	NaN
3965	LGALS9	0.10	0.00	0.05	-0.08
3972	LHB	0.06	0.00	0.05	0.22
3973	LHCGR	0.02	0.00	0.05	0.03
3975	LHX1	0.06	0.00	0.00	0.09
3976	LIF	0.00	0.29	0.05	0.46
3977	LIFR	0.02	0.00	0.00	0.06
3978	LIG1	0.02	0.00	0.00	0.00
3980	LIG3	0.06	0.00	0.00	0.28
3981	LIG4	0.00	0.00	0.05	-0.11
3982	LIM2	0.02	0.00	0.05	-0.13
3984	LIMK1	0.00	0.00	0.00	NaN
3985	LIMK2	0.00	0.29	0.05	0.29
3987	LIMS1	0.02	0.00	0.00	-0.06
3988	LIPA	0.02	0.00	0.05	0.03
3990	LIPC	0.00	0.00	0.00	NaN
3991	LIPE	0.00	0.00	0.05	0.12
3992	FADS1	0.00	0.00	0.05	0.17
3993	LLGL2	0.02	0.14	0.00	0.38
3995	FADS3	0.00	0.00	0.05	0.01
3996	LLGL1	0.02	0.00	0.00	-0.01
3998	LMAN1	0.00	0.00	0.05	0.11
4000	LMNA	0.04	0.14	0.05	-0.04
4001	LMNB1	0.00	0.00	0.00	NaN
4004	LMO1	0.00	0.00	0.00	NaN
4005	LMO2	0.02	0.00	0.00	-0.18
4008	LMO7	0.00	0.00	0.00	NaN
4010	LMX1B	0.02	0.00	0.00	-0.01
4012	LNPEP	0.02	0.00	0.00	-0.07
4014	LOR	0.02	0.00	0.10	0.08
4015	LOX	0.00	0.00	0.00	NaN
4016	LOXL1	0.00	0.00	0.05	0.00
4017	LOXL2	0.02	0.00	0.00	0.56
4018	LPA	0.02	0.00	0.05	-0.01
4023	LPL	0.00	0.00	0.00	NaN
4025	LPO	0.04	0.00	0.05	0.02
4026	LPP	0.00	0.14	0.00	0.04
4033	LRMP	0.06	0.00	0.00	-0.07
4034	LRRN1	0.00	0.00	0.05	0.41
4035	LRP1	0.00	0.00	0.00	NaN
4036	LRP2	0.00	0.00	0.00	NaN
4037	LRP3	0.00	0.00	0.00	NaN
4038	LRP4	0.02	0.00	0.00	0.00
4040	LRP6	0.04	0.00	0.05	-0.06
4041	LRP5	0.02	0.00	0.05	0.17
4043	LRPAP1	0.00	0.00	0.00	NaN

4045	LSAMP	0.02	0.00	0.00	-0.03
4046	LSP1	0.02	0.00	0.05	-0.03
4047	LSS	0.00	0.00	0.05	-0.02
4048	LTA4H	0.02	0.00	0.00	-0.01
4049	LTA	0.04	0.14	0.05	0.09
4050	LTB	0.02	0.00	0.05	0.00
4051	CYP4F3	0.04	0.00	0.00	-0.02
4052	LTBP1	0.00	0.00	0.00	NaN
4053	LTBP2	0.02	0.00	0.05	0.18
4054	LTBP3	0.04	0.00	0.05	-0.23
4055	LTBR	0.02	0.00	0.15	0.18
4056	LTC4S	0.08	0.43	0.10	0.29
4057	LTF	0.02	0.14	0.15	-0.06
4058	LTK	0.00	0.00	0.00	NaN
4060	LUM	0.00	0.00	0.00	NaN
4061	LY6E	0.10	0.00	0.15	0.10
4062	LY6H	0.10	0.00	0.15	0.01
4063	LY9	0.00	0.00	0.00	NaN
4065	LY75	0.00	0.00	0.05	0.08
4066	LYL1	0.00	0.14	0.05	-0.02
4067	LYN	0.04	0.00	0.00	-0.05
4070	TACSTD2	0.06	0.00	0.00	0.28
4071	TM4SF1	0.00	0.00	0.00	NaN
4074	M6PR	0.02	0.00	0.15	0.11
4081	MAB21L1	0.02	0.00	0.00	-0.03
4082	MARCKS	0.04	0.00	0.00	0.04
4085	MAD2L1	0.02	0.00	0.00	0.13
4094	MAF	0.00	0.00	0.00	NaN
4097	MAFG	0.00	0.00	0.00	NaN
4099	MAG	0.00	0.00	0.00	NaN
4117	MAK	0.04	0.00	0.00	-0.08
4118	MAL	0.04	0.00	0.05	-0.11
4121	MAN1A1	0.04	0.00	0.00	0.25
4122	MAN2A2	0.02	0.00	0.10	0.03
4123	MAN2C1	0.00	0.00	0.05	-0.09
4124	MAN2A1	0.02	0.00	0.00	0.10
4125	MAN2B1	0.06	0.14	0.00	-0.05
4126	MANBA	0.08	0.00	0.00	0.12
4130	MAP1A	0.04	0.00	0.00	0.18
4131	MAP1B	0.06	0.00	0.00	0.12
4133	MAP2	0.02	0.00	0.05	0.06
4134	MAP4	0.02	0.14	0.15	0.24
4137	MAPT	0.00	0.00	0.00	NaN
4139	MARK1	0.02	0.00	0.00	-0.09
4140	MARK3	0.00	0.00	0.05	-0.11
4141	MARS	0.02	0.14	0.00	0.01
4142	MAS1	0.02	0.00	0.05	-0.09

4143	MAT1A	0.06	0.14	0.05	0.02
4144	MAT2A	0.00	0.00	0.00	NaN
4145	MATK	0.02	0.00	0.15	0.00
4146	MATN1	0.00	0.14	0.00	0.02
4147	MATN2	0.06	0.00	0.00	-0.11
4148	MATN3	0.00	0.00	0.00	NaN
4149	MAX	0.06	0.00	0.00	0.22
4150	MAZ	0.06	0.14	0.00	0.15
4151	MB	0.00	0.29	0.05	-0.17
4152	MBD1	0.00	0.00	0.05	0.13
4153	MBL2	0.02	0.14	0.10	-0.25
4155	MBP	0.00	0.00	0.05	0.09
4157	MC1R	0.00	0.00	0.10	0.07
4158	MC2R	0.08	0.14	0.10	0.02
4159	MC3R	0.12	0.00	0.10	-0.08
4160	MC4R	0.00	0.00	0.05	-0.08
4161	MC5R	0.08	0.14	0.10	0.04
4162	MCAM	0.00	0.00	0.05	0.17
4163	MCC	0.00	0.00	0.00	NaN
4166	CHST6	0.00	0.00	0.00	NaN
4170	MCL1	0.02	0.00	0.10	0.20
4171	MCM2	0.04	0.00	0.00	0.08
4172	MCM3	0.02	0.00	0.00	-0.13
4173	MCM4	0.04	0.00	0.00	-0.10
4174	MCM5	0.00	0.29	0.05	0.11
4175	MCM6	0.00	0.00	0.00	NaN
4176	MCM7	0.02	0.00	0.00	0.13
4185	ADAM11	0.00	0.00	0.05	0.02
4188	MDFI	0.06	0.00	0.05	0.10
4189	DNAJB9	0.00	0.00	0.00	NaN
4190	MDH1	0.00	0.00	0.00	NaN
4191	MDH2	0.00	0.00	0.00	NaN
4192	MDK	0.00	0.00	0.05	0.04
4193	MDM2	0.08	0.00	0.00	0.55
4194	MDM4	0.00	0.00	0.00	NaN
4197	MDS1	0.00	0.00	0.00	NaN
4199	ME1	0.10	0.00	0.20	0.10
4200	ME2	0.00	0.00	0.05	0.11
4205	MEF2A	0.16	0.00	0.10	0.01
4207	MEF2B	0.12	0.00	0.15	0.00
4208	MEF2C	0.00	0.00	0.00	NaN
4209	MEF2D	0.04	0.14	0.05	-0.02
4210	MEFV	0.02	0.00	0.00	0.07
4211	MEIS1	0.00	0.00	0.00	NaN
4212	MEIS2	0.04	0.00	0.05	0.09
4214	MAP3K1	0.00	0.00	0.05	-0.09
4215	MAP3K3	0.12	0.00	0.20	0.31

4216	MAP3K4	0.02	0.00	0.05	0.09
4217	MAP3K5	0.00	0.00	0.10	0.07
4221	MEN1	0.00	0.00	0.00	NaN
4222	MEOX1	0.00	0.14	0.05	-0.11
4223	MEOX2	0.00	0.00	0.00	NaN
4224	MEP1A	0.04	0.14	0.20	-0.02
4225	MEP1B	0.02	0.00	0.10	-0.09
4232	MEST	0.00	0.00	0.00	NaN
4233	MET	0.00	0.00	0.00	NaN
4234	METTL1	0.02	0.00	0.00	-0.02
4236	MFAP1	0.04	0.00	0.00	0.07
4237	MFAP2	0.02	0.00	0.00	0.05
4238	MFAP3	0.02	0.00	0.00	0.17
4239	MFAP4	0.02	0.00	0.00	-0.02
4240	MFGE8	0.00	0.00	0.00	NaN
4241	MFI2	0.02	0.00	0.00	-0.04
4242	MFNG	0.00	0.14	0.00	0.05
4246	SCGB2A1	0.00	0.00	0.05	-0.12
4247	MGAT2	0.00	0.00	0.00	NaN
4248	MGAT3	0.00	0.14	0.00	0.11
4249	MGAT5	0.00	0.00	0.00	NaN
4250	SCGB2A2	0.00	0.00	0.05	-0.18
4254	KITLG	0.02	0.14	0.00	0.03
4255	MGMT	0.04	0.00	0.00	0.11
4256	MGP	0.00	0.00	0.00	NaN
4258	MGST2	0.00	0.00	0.05	-0.10
4259	MGST3	0.02	0.00	0.00	-0.16
4276	MICA	0.04	0.14	0.05	0.13
4277	MICB	0.04	0.14	0.05	0.22
4282	MIF	0.00	0.14	0.05	0.04
4284	MIP	0.00	0.00	0.00	NaN
4285	MIPEP	0.02	0.00	0.00	0.14
4286	MITF	0.02	0.00	0.00	-0.02
4288	MKI67	0.04	0.00	0.00	0.09
4289	MKLN1	0.00	0.00	0.00	NaN
4291	MLF1	0.02	0.00	0.10	0.11
4292	MLH1	0.02	0.14	0.10	-0.03
4293	MAP3K9	0.02	0.00	0.05	-0.11
4294	MAP3K10	0.06	0.00	0.05	-0.02
4295	MLN	0.00	0.00	0.00	NaN
4296	MAP3K11	0.04	0.00	0.05	0.12
4297	MLL	0.00	0.00	0.00	NaN
4298	MLLT1	0.02	0.00	0.15	-0.11
4300	MLLT3	0.02	0.00	0.05	-0.07
4301	MLLT4	0.02	0.00	0.05	-0.01
4306	NR3C2	0.02	0.14	0.10	0.11
4308	TRPM1	0.00	0.00	0.00	NaN

4311	MME	0.02	0.00	0.10	-0.03
4312	MMP1	0.00	0.00	0.00	NaN
4313	MMP2	0.02	0.00	0.00	0.09
4314	MMP3	0.02	0.00	0.10	-0.02
4316	MMP7	0.00	0.00	0.00	NaN
4317	MMP8	0.00	0.00	0.00	NaN
4318	MMP9	0.02	0.00	0.00	0.01
4319	MMP10	0.00	0.00	0.00	NaN
4320	MMP11	0.00	0.14	0.05	0.26
4321	MMP12	0.02	0.00	0.10	0.28
4322	MMP13	0.02	0.00	0.10	-0.05
4323	MMP14	0.02	0.00	0.00	0.10
4324	MMP15	0.04	0.00	0.05	0.22
4325	MMP16	0.06	0.00	0.15	0.19
4326	MMP17	0.08	0.14	0.05	-0.01
4327	MMP19	0.02	0.00	0.05	0.15
4329	ALDH6A1	0.02	0.00	0.05	0.05
4330	MN1	0.00	0.14	0.05	-0.15
4331	MNAT1	0.00	0.14	0.00	0.01
4332	MNDA	0.00	0.00	0.00	NaN
4335	MNT	0.04	0.00	0.05	0.12
4336	MOBP	0.02	0.00	0.05	0.10
4338	MOCS2	0.18	0.00	0.30	-0.20
4340	MOG	0.04	0.14	0.05	-0.05
4342	MOS	0.04	0.00	0.00	-0.03
4350	MPG	0.18	0.00	0.10	0.01
4351	MPI	0.00	0.00	0.05	-0.08
4352	MPL	0.02	0.00	0.05	-0.02
4353	MPO	0.04	0.00	0.05	0.13
4355	MPP2	0.00	0.14	0.05	0.08
4356	MPP3	0.00	0.14	0.05	0.06
4357	MPST	0.00	0.14	0.00	0.03
4358	MPV17	0.00	0.00	0.00	NaN
4359	MPZ	0.00	0.00	0.00	NaN
4361	MRE11A	0.00	0.00	0.00	NaN
4363	ABCC1	0.02	0.00	0.05	0.26
4430	MYO1B	0.00	0.00	0.05	0.00
4436	MSH2	0.00	0.00	0.00	NaN
4437	MSH3	0.00	0.00	0.00	NaN
4438	MSH4	0.02	0.00	0.05	-0.08
4439	MSH5	0.02	0.00	0.05	0.02
4440	MSI1	0.02	0.00	0.10	-0.15
4477	MSMB	0.02	0.00	0.05	0.06
4481	MSR1	0.00	0.00	0.10	0.00
4482	MSRA	0.00	0.00	0.00	NaN
4485	MST1	0.06	0.00	0.05	-0.10
4486	MST1R	0.06	0.00	0.05	0.09

4487	MSX1	0.00	0.00	0.00	NaN
4488	MSX2	0.00	0.00	0.00	NaN
4495	MT1G	0.00	0.14	0.00	-0.06
4496	MT1H	0.00	0.14	0.00	-0.09
4501	MT1X	0.00	0.14	0.00	-0.04
4502	MT2A	0.00	0.14	0.00	-0.06
4504	MT3	0.00	0.14	0.00	0.01
4507	MTAP	0.02	0.00	0.05	-0.08
4520	MTF1	0.04	0.00	0.05	0.11
4521	NUDT1	0.00	0.00	0.00	NaN
4522	MTHFD1	0.06	0.00	0.00	0.37
4524	MTHFR	0.02	0.00	0.00	-0.12
4528	MTIF2	0.02	0.00	0.05	0.01
4542	MYO1F	0.02	0.00	0.15	0.06
4543	MTNR1A	0.00	0.00	0.00	NaN
4544	MTNR1B	0.00	0.00	0.00	NaN
4548	MTR	0.00	0.00	0.00	NaN
4552	MTRR	0.00	0.00	0.00	NaN
4580	MTX1	0.04	0.00	0.15	-0.16
4582	MUC1	0.04	0.00	0.15	-0.07
4583	MUC2	0.02	0.00	0.05	-0.02
4585	MUC4	0.02	0.00	0.00	0.05
4586	MUC5AC	0.02	0.00	0.05	0.03
4587	MUC5B	0.02	0.00	0.05	-0.04
4588	MUC6	0.02	0.00	0.05	0.15
4593	MUSK	0.00	0.00	0.00	NaN
4594	MUT	0.00	0.00	0.00	NaN
4595	MUTYH	0.02	0.00	0.05	0.11
4597	MVD	0.00	0.00	0.10	0.24
4598	MVK	0.02	0.00	0.00	0.01
4599	MX1	0.00	0.00	0.00	NaN
4600	MX2	0.00	0.00	0.05	0.10
4601	MXI1	0.00	0.14	0.00	-0.02
4602	MYB	0.00	0.00	0.00	NaN
4603	MYBL1	0.00	0.00	0.00	NaN
4605	MYBL2	0.02	0.00	0.10	0.13
4606	MYBPC2	0.00	0.00	0.00	NaN
4608	MYBPH	0.00	0.00	0.00	NaN
4609	MYC	0.12	0.00	0.05	0.28
4610	MYCL1	0.02	0.00	0.05	0.00
4613	MYCN	0.00	0.00	0.00	NaN
4615	MYD88	0.00	0.00	0.15	0.20
4616	GADD45B	0.00	0.00	0.00	NaN
4617	MYF5	0.02	0.00	0.00	-0.02
4618	MYF6	0.02	0.00	0.00	-0.17
4619	MYH1	0.04	0.00	0.00	-0.04
4620	MYH2	0.04	0.00	0.00	0.06

4621	MYH3	0.04	0.00	0.00	0.02
4622	MYH4	0.04	0.00	0.00	-0.07
4624	MYH6	0.02	0.14	0.10	0.11
4625	MYH7	0.02	0.14	0.10	0.22
4626	MYH8	0.04	0.00	0.00	0.03
4627	MYH9	0.00	0.14	0.00	-0.25
4629	MYH11	0.02	0.00	0.05	-0.03
4632	MYL1	0.02	0.00	0.05	-0.20
4633	MYL2	0.02	0.00	0.00	-0.11
4634	MYL3	0.02	0.14	0.15	0.02
4635	MYL4	0.00	0.00	0.00	NaN
4637	MYL6	0.00	0.00	0.00	NaN
4638	MYLK	0.00	0.00	0.10	0.28
4640	MYO1A	0.00	0.00	0.00	NaN
4641	MYO1C	0.04	0.00	0.05	-0.16
4642	MYO1D	0.04	0.00	0.05	0.03
4643	MYO1E	0.00	0.00	0.00	NaN
4644	MYO5A	0.00	0.00	0.00	NaN
4646	MYO6	0.10	0.00	0.20	-0.02
4647	MYO7A	0.02	0.00	0.05	0.17
4649	MYO9A	0.00	0.00	0.00	NaN
4650	MYO9B	0.04	0.00	0.00	0.36
4651	MYO10	0.02	0.00	0.00	-0.05
4653	MYOC	0.04	0.00	0.05	-0.15
4654	MYOD1	0.00	0.00	0.00	NaN
4656	MYOG	0.00	0.00	0.00	NaN
4659	PPP1R12A	0.02	0.00	0.00	0.21
4660	PPP1R12B	0.02	0.00	0.00	0.05
4661	MYT1	0.08	0.00	0.05	0.15
4664	NAB1	0.00	0.00	0.05	-0.06
4665	NAB2	0.00	0.00	0.00	NaN
4666	NACA	0.00	0.00	0.00	NaN
4668	NAGA	0.00	0.14	0.05	0.04
4669	NAGLU	0.02	0.86	0.05	0.09
4673	NAP1L1	0.02	0.00	0.00	0.08
4676	NAP1L4	0.02	0.00	0.05	-0.07
4677	NARS	0.00	0.00	0.15	0.15
4678	NASP	0.02	0.00	0.05	0.17
4680	CEACAM6	0.00	0.00	0.05	-0.10
4681	NBL1	0.00	0.00	0.00	NaN
4682	NUBP1	0.00	0.00	0.00	NaN
4684	NCAM1	0.00	0.00	0.00	NaN
4685	NCAM2	0.00	0.00	0.05	-0.03
4686	NCBP1	0.04	0.00	0.00	0.03
4687	NCF1	0.00	0.00	0.00	NaN
4688	NCF2	0.02	0.00	0.00	0.15
4689	NCF4	0.00	0.14	0.00	0.06

4690	NCK1	0.02	0.14	0.00	-0.01
4691	NCL	0.02	0.00	0.15	0.09
4692	NDN	0.14	0.14	0.25	-0.15
4695	NDUFA2	0.00	0.00	0.00	NaN
4696	NDUFA3	0.02	0.00	0.05	-0.10
4697	NDUFA4	0.00	0.00	0.00	NaN
4698	NDUFA5	0.00	0.00	0.00	NaN
4700	NDUFA6	0.00	0.14	0.05	0.18
4701	NDUFA7	0.02	0.00	0.15	0.07
4702	NDUFA8	0.06	0.00	0.00	-0.13
4703	NEB	0.00	0.00	0.00	NaN
4704	NDUFA9	0.02	0.00	0.00	-0.33
4705	NDUFA10	0.08	0.43	0.10	-0.18
4706	NDUFAB1	0.02	0.14	0.00	-0.15
4707	NDUFB1	0.00	0.00	0.05	-0.06
4708	NDUFB2	0.00	0.14	0.05	0.06
4709	NDUFB3	0.00	0.00	0.05	-0.09
4710	NDUFB4	0.00	0.00	0.10	-0.07
4711	NDUFB5	0.00	0.00	0.10	0.26
4712	NDUFB6	0.00	0.00	0.15	0.10
4713	NDUFB7	0.00	0.00	0.05	0.33
4714	NDUFB8	0.00	0.00	0.05	-0.03
4717	NDUFC1	0.00	0.00	0.05	0.01
4718	NDUFC2	0.02	0.00	0.00	0.36
4719	NDUFS1	0.00	0.00	0.00	NaN
4720	NDUFS2	0.00	0.00	0.00	NaN
4722	NDUFS3	0.04	0.00	0.00	0.10
4724	NDUFS4	0.18	0.00	0.30	-0.34
4725	NDUFS5	0.04	0.00	0.05	0.01
4726	NDUFS6	0.00	0.00	0.00	NaN
4727	NDUFS7	0.02	0.00	0.05	-0.04
4728	NDUFS8	0.04	0.00	0.05	0.23
4729	NDUFV2	0.02	0.00	0.00	0.08
4733	DRG1	0.00	0.29	0.05	0.19
4734	NEDD4	0.00	0.00	0.00	NaN
4736	RPL10A	0.04	0.00	0.00	0.10
4738	NEDD8	0.02	0.14	0.10	0.10
4744	NEFH	0.00	0.14	0.00	-0.04
4745	NELL1	0.02	0.00	0.00	-0.01
4747	NEFL	0.00	0.14	0.00	-0.02
4750	NEK1	0.02	0.00	0.00	0.20
4751	NEK2	0.02	0.00	0.15	0.35
4752	NEK3	0.04	0.00	0.10	-0.01
4753	NELL2	0.00	0.00	0.35	0.00
4756	NEO1	0.00	0.00	0.05	-0.20
4758	NEU1	0.02	0.00	0.05	0.07
4759	NEU2	0.00	0.00	0.05	-0.02

4760	NEUROD1	0.00	0.00	0.00	NaN
4761	NEUROD2	0.02	0.86	0.05	0.33
4762	NEUROG1	0.02	0.00	0.00	-0.01
4763	NF1	0.10	0.00	0.05	0.23
4771	NF2	0.00	0.29	0.05	0.39
4772	NFATC1	0.02	0.00	0.05	0.16
4775	NFATC3	0.02	0.00	0.00	-0.09
4776	NFATC4	0.02	0.00	0.05	-0.10
4778	NFE2	0.02	0.00	0.05	0.00
4779	NFE2L1	0.04	0.00	0.05	-0.02
4780	NFE2L2	0.02	0.00	0.00	0.16
4781	NFIB	0.00	0.00	0.00	NaN
4782	NFIC	0.02	0.00	0.15	-0.15
4783	NFIL3	0.00	0.00	0.05	0.21
4784	NFIX	0.00	0.14	0.05	0.12
4790	NFKB1	0.08	0.00	0.00	0.02
4791	NFKB2	0.02	0.00	0.00	-0.02
4792	NFKBIA	0.04	0.00	0.00	-0.04
4793	NFKBIB	0.06	0.00	0.05	0.09
4794	NFKBIE	0.04	0.14	0.20	0.31
4795	NFKBIL1	0.04	0.14	0.05	0.09
4798	NFRKB	0.02	0.00	0.00	-0.05
4799	NFX1	0.04	0.00	0.00	0.05
4800	NFYA	0.06	0.00	0.05	0.11
4801	NFYB	0.00	0.00	0.00	NaN
4802	NFYC	0.02	0.00	0.05	-0.04
4804	NGFR	0.04	0.00	0.05	-0.02
4808	NHLH2	0.08	0.00	0.10	0.03
4809	NHP2L1	0.00	0.14	0.00	-0.02
4814	NINJ1	0.04	0.00	0.05	-0.03
4815	NINJ2	0.08	0.14	0.10	0.11
4817	NIT1	0.00	0.00	0.00	NaN
4818	NKG7	0.02	0.00	0.05	0.00
4820	NKTR	0.00	0.00	0.05	0.22
4826	NNAT	0.04	0.00	0.00	0.02
4828	NMB	0.00	0.00	0.00	NaN
4829	NMBR	0.02	0.00	0.05	0.04
4830	NME1	0.10	0.00	0.10	0.09
4831	NME2	0.10	0.00	0.10	0.13
4832	NME3	0.18	0.00	0.10	0.00
4833	NME4	0.18	0.00	0.10	-0.03
4836	NMT1	0.00	0.00	0.05	-0.04
4837	NNMT	0.00	0.00	0.00	NaN
4842	NOS1	0.02	0.00	0.10	0.25
4846	NOS3	0.02	0.00	0.00	0.11
4848	CNOT2	0.12	0.00	0.05	0.44
4849	CNOT3	0.02	0.00	0.05	0.04

4850	CNOT4	0.00	0.00	0.10	0.08
4852	NPY	0.00	0.00	0.10	0.00
4853	NOTCH2	0.41	0.43	0.10	-0.16
4854	NOTCH3	0.04	0.00	0.00	0.05
4855	NOTCH4	0.02	0.00	0.05	0.10
4856	NOV	0.10	0.00	0.15	-0.02
4857	NOVA1	0.04	0.00	0.00	-0.09
4858	NOVA2	0.00	0.00	0.05	0.09
4860	NP	0.02	0.00	0.00	-0.02
4861	NPAS1	0.00	0.00	0.05	0.24
4862	NPAS2	0.02	0.00	0.10	0.29
4863	NPAT	0.02	0.00	0.00	0.08
4864	NPC1	0.00	0.14	0.00	-0.04
4867	NPHP1	0.02	0.00	0.00	0.27
4868	NPHS1	0.00	0.00	0.00	NaN
4869	NPM1	0.02	0.00	0.00	0.13
4879	NPPB	0.02	0.00	0.00	-0.07
4880	NPPC	0.00	0.00	0.05	0.25
4881	NPR1	0.02	0.00	0.10	0.20
4882	NPR2	0.00	0.00	0.00	NaN
4883	NPR3	0.00	0.00	0.10	0.23
4884	NPTX1	0.00	0.14	0.00	-0.01
4885	NPTX2	0.02	0.00	0.00	-0.08
4886	NPY1R	0.00	0.00	0.05	-0.15
4887	NPY2R	0.00	0.14	0.00	0.19
4888	NPY6R	0.02	0.00	0.00	-0.09
4889	NPY5R	0.00	0.00	0.05	-0.04
4891	SLC11A2	0.02	0.00	0.00	0.22
4893	NRAS	0.08	0.00	0.10	-0.13
4897	NRCAM	0.00	0.00	0.00	NaN
4898	NRD1	0.02	0.00	0.00	0.03
4899	NRF1	0.00	0.00	0.00	NaN
4900	NRGN	0.02	0.00	0.00	0.07
4901	NRL	0.02	0.14	0.10	0.03
4902	NRTN	0.02	0.00	0.15	0.29
4905	NSF	0.00	0.00	0.00	NaN
4907	NT5E	0.00	0.00	0.05	-0.11
4908	NTF3	0.02	0.00	0.15	0.03
4913	NTHL1	0.18	0.00	0.10	0.12
4914	NTRK1	0.04	0.14	0.05	-0.01
4915	NTRK2	0.00	0.00	0.00	NaN
4916	NTRK3	0.00	0.00	0.00	NaN
4919	ROR1	0.00	0.00	0.00	NaN
4920	ROR2	0.00	0.00	0.05	-0.11
4921	DDR2	0.00	0.00	0.00	NaN
4922	NTS	0.02	0.14	0.00	-0.02
4923	NTSR1	0.08	0.00	0.05	-0.06

4924	NUCB1	0.06	0.00	0.05	0.18
4925	NUCB2	0.00	0.00	0.00	NaN
4926	NUMA1	0.06	0.00	0.15	-0.09
4927	NUP88	0.04	0.00	0.10	0.13
4928	NUP98	0.00	0.00	0.05	0.25
4929	NR4A2	0.00	0.00	0.00	NaN
4931	NVL	0.02	0.00	0.05	-0.04
4938	OAS1	0.02	0.00	0.00	-0.09
4939	OAS2	0.02	0.00	0.00	-0.10
4940	OAS3	0.02	0.00	0.00	-0.09
4942	OAT	0.04	0.00	0.05	-0.01
4947	OAZ2	0.02	0.00	0.00	-0.35
4948	OCA2	0.00	0.00	0.00	NaN
4950	OCLN	0.06	0.00	0.00	0.02
4951	OCM	0.02	0.00	0.00	-0.08
4953	ODC1	0.00	0.00	0.00	NaN
4956	ODF1	0.12	0.00	0.25	-0.15
4957	ODF2	0.00	0.00	0.00	NaN
4958	OMD	0.04	0.00	0.05	-0.03
4967	OGDH	0.02	0.00	0.00	0.02
4968	OGG1	0.00	0.00	0.10	0.00
4969	OGN	0.04	0.00	0.05	-0.01
4973	OLR1	0.02	0.00	0.15	-0.04
4974	OMG	0.10	0.00	0.05	0.02
4975	OMP	0.02	0.00	0.05	-0.08
4976	OPA1	0.02	0.00	0.00	0.26
4978	OPCML	0.00	0.00	0.00	NaN
4982	TNFRSF11B	0.10	0.00	0.15	-0.04
4985	OPRD1	0.04	0.00	0.00	0.25
4986	OPRK1	0.00	0.14	0.00	-0.05
4987	OPRL1	0.08	0.00	0.05	-0.10
4988	OPRM1	0.02	0.00	0.05	0.13
4990	SIX6	0.00	0.14	0.00	0.10
4991	OR1D2	0.04	0.00	0.10	-0.04
4992	OR1F1	0.02	0.00	0.00	-0.07
4993	OR2C1	0.02	0.00	0.00	0.03
4994	OR3A1	0.04	0.00	0.10	-0.02
4995	OR3A2	0.04	0.00	0.10	-0.05
4998	ORC1L	0.02	0.00	0.00	-0.03
4999	ORC2L	0.00	0.00	0.05	0.00
5000	ORC4L	0.00	0.00	0.00	NaN
5001	ORC5L	0.00	0.00	0.00	NaN
5004	ORM1	0.00	0.00	0.00	NaN
5005	ORM2	0.00	0.00	0.00	NaN
5007	OSBP	0.00	0.00	0.00	NaN
5008	OSM	0.00	0.29	0.05	0.12
5010	CLDN11	0.00	0.00	0.00	NaN

5016	OVGP1	0.06	0.14	0.00	0.05
5017	OVOL1	0.04	0.00	0.05	-0.02
5018	OXA1L	0.02	0.00	0.00	0.21
5020	OXT	0.00	0.00	0.00	NaN
5021	OXTR	0.00	0.00	0.10	0.02
5023	P2RX1	0.04	0.00	0.10	-0.17
5024	P2RX3	0.00	0.00	0.00	NaN
5025	P2RX4	0.02	0.00	0.10	-0.04
5026	P2RX5	0.04	0.00	0.10	-0.05
5027	P2RX7	0.02	0.00	0.10	0.04
5028	P2RY1	0.02	0.00	0.10	0.14
5029	P2RY2	0.02	0.00	0.00	0.54
5031	P2RY6	0.02	0.00	0.00	-0.01
5032	P2RY11	0.02	0.00	0.15	0.03
5033	P4HA1	0.00	0.00	0.00	NaN
5034	P4HB	0.00	0.00	0.00	NaN
5042	PABPC3	0.06	0.00	0.00	-0.02
5048	PAFAH1B1	0.04	0.00	0.05	0.21
5049	PAFAH1B2	0.02	0.00	0.00	0.04
5050	PAFAH1B3	0.00	0.00	0.05	-0.03
5051	PAFAH2	0.02	0.00	0.05	0.02
5052	PRDX1	0.02	0.00	0.05	0.29
5053	PAH	0.00	0.00	0.00	NaN
5054	SERPINE1	0.00	0.14	0.00	-0.06
5055	SERPINB2	0.02	0.00	0.00	-0.02
5058	PAK1	0.02	0.00	0.05	0.46
5062	PAK2	0.02	0.00	0.00	0.38
5064	PALM	0.06	0.00	0.15	-0.21
5066	PAM	0.02	0.00	0.00	-0.02
5069	PAPPA	0.02	0.00	0.00	0.00
5071	PARK2	0.02	0.00	0.05	-0.03
5073	PARN	0.02	0.00	0.05	0.02
5074	PAWR	0.02	0.00	0.00	0.20
5076	PAX2	0.00	0.00	0.00	NaN
5077	PAX3	0.00	0.00	0.00	NaN
5078	PAX4	0.02	0.00	0.00	0.02
5079	PAX5	0.04	0.00	0.00	-0.05
5080	PAX6	0.00	0.00	0.00	NaN
5081	PAX7	0.00	0.00	0.00	NaN
5082	PDCL	0.06	0.00	0.00	0.37
5083	PAX9	0.04	0.00	0.00	-0.02
5087	PBX1	0.00	0.00	0.00	NaN
5089	PBX2	0.02	0.00	0.05	0.21
5090	PBX3	0.00	0.00	0.00	NaN
5091	PC	0.04	0.00	0.05	0.08
5093	PCBP1	0.00	0.14	0.00	-0.10
5094	PCBP2	0.00	0.00	0.00	NaN

5095	PCCA	0.00	0.00	0.05	-0.21
5096	PCCB	0.02	0.14	0.00	0.16
5097	PCDH1	0.00	0.00	0.00	NaN
5098	PCDHGC3	0.00	0.00	0.00	NaN
5099	PCDH7	0.00	0.00	0.00	NaN
5100	PCDH8	0.04	0.00	0.10	0.34
5101	PCDH9	0.08	0.14	0.00	0.42
5104	SERPINA5	0.00	0.00	0.00	NaN
5105	PCK1	0.08	0.00	0.05	0.30
5106	PCK2	0.02	0.14	0.10	-0.01
5108	PCM1	0.00	0.00	0.00	NaN
5110	PCMT1	0.02	0.00	0.05	0.05
5111	PCNA	0.00	0.00	0.10	-0.04
5118	PCOLCE	0.00	0.00	0.00	NaN
5121	PCP4	0.00	0.00	0.00	NaN
5122	PCSK1	0.00	0.00	0.00	NaN
5125	PCSK5	0.00	0.00	0.00	NaN
5126	PCSK2	0.02	0.00	0.00	-0.04
5128	PCTK2	0.02	0.00	0.00	0.18
5129	PCTK3	0.02	0.00	0.05	0.00
5130	PCYT1A	0.02	0.00	0.00	0.46
5132	PDC	0.00	0.00	0.05	-0.03
5133	PDCD1	0.08	0.43	0.10	-0.11
5134	PDCD2	0.04	0.00	0.05	0.01
5136	PDE1A	0.00	0.00	0.00	NaN
5137	PDE1C	0.02	0.00	0.00	-0.09
5138	PDE2A	0.02	0.00	0.00	-0.02
5139	PDE3A	0.02	0.00	0.05	0.00
5140	PDE3B	0.00	0.00	0.15	-0.04
5141	PDE4A	0.02	0.00	0.15	-0.02
5142	PDE4B	0.02	0.00	0.15	-0.04
5143	PDE4C	0.02	0.00	0.00	0.03
5144	PDE4D	0.02	0.00	0.00	0.05
5145	PDE6A	0.00	0.14	0.05	0.00
5146	PDE6C	0.02	0.00	0.05	-0.15
5147	PDE6D	0.02	0.00	0.15	0.07
5148	PDE6G	0.00	0.00	0.00	NaN
5149	PDE6H	0.00	0.00	0.00	NaN
5151	PDE8A	0.00	0.00	0.00	NaN
5152	PDE9A	0.00	0.00	0.00	NaN
5153	PDE1B	0.02	0.00	0.05	-0.03
5154	PDGFA	0.06	0.00	0.05	-0.07
5155	PDGFB	0.00	0.14	0.05	-0.10
5156	PDGFRA	0.00	0.00	0.00	NaN
5157	PDGFRL	0.00	0.00	0.00	NaN
5159	PDGFRB	0.00	0.00	0.00	NaN
5161	PDHA2	0.04	0.00	0.05	0.03

5162	PDHB	0.02	0.00	0.00	-0.12
5163	PDK1	0.00	0.00	0.00	NaN
5164	PDK2	0.06	0.00	0.00	-0.03
5166	PDK4	0.00	0.00	0.00	NaN
5167	ENPP1	0.02	0.00	0.00	0.12
5168	ENPP2	0.10	0.00	0.15	-0.05
5169	ENPP3	0.02	0.00	0.00	0.06
5170	PDPK1	0.02	0.00	0.00	0.03
5172	SLC26A4	0.00	0.00	0.00	NaN
5173	PDYN	0.00	0.00	0.00	NaN
5174	PDZK1	0.02	0.00	0.00	-0.06
5175	PECAM1	0.12	0.00	0.20	0.04
5176	SERPINF1	0.04	0.00	0.05	-0.15
5178	PEG3	0.00	0.00	0.05	0.17
5179	PENK	0.04	0.00	0.00	0.01
5184	PEPD	0.00	0.00	0.00	NaN
5187	PER1	0.00	0.00	0.00	NaN
5188	PET112L	0.00	0.00	0.00	NaN
5189	PEX1	0.00	0.00	0.00	NaN
5190	PEX6	0.06	0.00	0.05	-0.09
5191	PEX7	0.00	0.00	0.10	-0.10
5193	PEX12	0.06	0.00	0.00	0.13
5194	PEX13	0.00	0.00	0.05	0.08
5195	PEX14	0.02	0.00	0.00	0.06
5196	PF4	0.00	0.00	0.00	NaN
5197	PF4V1	0.00	0.00	0.00	NaN
5198	PFAS	0.00	0.00	0.00	NaN
5201	PFDN1	0.00	0.00	0.00	NaN
5202	PFDN2	0.00	0.00	0.00	NaN
5203	PFDN4	0.14	0.00	0.05	0.36
5204	PFDN5	0.00	0.00	0.00	NaN
5205	ATP8B1	0.00	0.00	0.15	-0.09
5208	PFKFB2	0.00	0.00	0.05	0.18
5209	PFKFB3	0.00	0.00	0.00	NaN
5210	PFKFB4	0.00	0.00	0.05	0.00
5211	PFKL	0.02	0.00	0.00	0.02
5213	PFKM	0.04	0.00	0.05	0.17
5214	PFKP	0.02	0.00	0.00	-0.03
5216	PFN1	0.04	0.00	0.10	0.16
5217	PFN2	0.00	0.00	0.00	NaN
5218	PFTK1	0.00	0.00	0.00	NaN
5223	PGAM1	0.00	0.00	0.05	0.02
5224	PGAM2	0.00	0.00	0.00	NaN
5225	PGC	0.06	0.00	0.05	0.07
5226	PGD	0.02	0.00	0.00	0.16
5228	PGF	0.02	0.00	0.05	0.09
5229	PGGT1B	0.02	0.00	0.00	-0.02

5236	PGM1	0.00	0.00	0.00	NaN
5239	PGM5	0.00	0.00	0.00	NaN
5241	PGR	0.00	0.00	0.00	NaN
5243	ABCB1	0.00	0.00	0.00	NaN
5244	ABCB4	0.00	0.00	0.00	NaN
5245	PHB	0.04	0.00	0.05	0.07
5250	SLC25A3	0.04	0.14	0.05	-0.14
5252	PHF1	0.02	0.00	0.05	0.18
5253	PHF2	0.04	0.00	0.05	-0.11
5257	PHKB	0.43	0.29	0.15	-0.32
5260	PHKG1	0.04	0.14	0.20	0.05
5261	PHKG2	0.02	0.00	0.00	-0.04
5264	PHYH	0.00	0.14	0.00	0.21
5265	SERPINA1	0.00	0.00	0.00	NaN
5266	PI3	0.04	0.00	0.00	0.01
5267	SERPINA4	0.00	0.00	0.00	NaN
5268	SERPINB5	0.02	0.00	0.00	-0.05
5271	SERPINB8	0.02	0.00	0.00	-0.08
5273	SERPINB10	0.02	0.00	0.00	0.00
5274	SERPINI1	0.00	0.00	0.00	NaN
5275	SERPINB13	0.02	0.00	0.00	-0.02
5276	SERPINI2	0.00	0.00	0.00	NaN
5279	PIGC	0.04	0.00	0.05	0.23
5281	PIGF	0.00	0.00	0.00	NaN
5283	PIGH	0.00	0.00	0.05	-0.06
5284	PIGR	0.00	0.00	0.05	-0.01
5287	PIK3C2B	0.00	0.00	0.00	NaN
5288	PIK3C2G	0.02	0.00	0.00	-0.01
5289	PIK3C3	0.02	0.00	0.00	0.06
5290	PIK3CA	0.00	0.00	0.00	NaN
5291	PIK3CB	0.00	0.00	0.00	NaN
5292	PIM1	0.00	0.00	0.00	NaN
5293	PIK3CD	0.00	0.00	0.00	NaN
5294	PIK3CG	0.00	0.00	0.10	-0.04
5295	PIK3R1	0.00	0.00	0.00	NaN
5296	PIK3R2	0.02	0.00	0.00	0.14
5300	PIN1	0.02	0.00	0.15	0.02
5301	PIN1L	0.04	0.14	0.00	-0.03
5304	PIP	0.00	0.14	0.05	0.22
5307	PITX1	0.00	0.00	0.00	NaN
5308	PITX2	0.00	0.00	0.00	NaN
5309	PITX3	0.02	0.00	0.00	0.05
5310	PKD1	0.18	0.00	0.10	0.06
5311	PKD2	0.02	0.00	0.00	-0.02
5313	PKLR	0.04	0.00	0.15	-0.10
5315	PKM2	0.04	0.00	0.05	0.15
5316	PKNOX1	0.00	0.00	0.00	NaN

5317	PKP1	0.00	0.00	0.05	-0.05
5318	PKP2	0.02	0.00	0.00	0.02
5319	PLA2G1B	0.02	0.00	0.10	-0.03
5320	PLA2G2A	0.02	0.00	0.05	-0.03
5321	PLA2G4A	0.00	0.00	0.05	0.31
5322	PLA2G5	0.02	0.00	0.05	0.07
5324	PLAG1	0.04	0.00	0.00	-0.02
5325	PLAGL1	0.02	0.00	0.05	-0.02
5326	PLAGL2	0.04	0.00	0.15	0.02
5327	PLAT	0.04	0.14	0.05	-0.10
5328	PLAU	0.02	0.00	0.00	0.04
5329	PLAUR	0.00	0.00	0.00	NaN
5330	PLCB2	0.00	0.00	0.00	NaN
5332	PLCB4	0.00	0.00	0.05	0.01
5333	PLCD1	0.00	0.00	0.15	0.00
5334	PLCL1	0.00	0.00	0.05	-0.05
5335	PLCG1	0.04	0.00	0.00	0.32
5336	PLCG2	0.02	0.00	0.00	-0.02
5337	PLD1	0.02	0.00	0.00	0.08
5338	PLD2	0.04	0.00	0.10	0.06
5339	PLEC1	0.10	0.00	0.15	0.28
5340	PLG	0.02	0.00	0.05	-0.04
5341	PLEK	0.00	0.00	0.00	NaN
5345	SERPINF2	0.04	0.00	0.05	0.08
5346	PLIN	0.00	0.00	0.00	NaN
5348	FXYD1	0.00	0.00	0.00	NaN
5349	FXYD3	0.00	0.00	0.00	NaN
5350	PLN	0.06	0.00	0.05	-0.04
5352	PLOD2	0.04	0.00	0.00	-0.03
5357	PLS1	0.02	0.00	0.00	-0.13
5359	PLSCR1	0.04	0.00	0.00	0.03
5360	PLTP	0.02	0.00	0.00	-0.21
5361	PLXNA1	0.02	0.00	0.00	0.26
5362	PLXNA2	0.00	0.00	0.05	0.01
5364	PLXNB1	0.00	0.00	0.05	-0.03
5366	PMAIP1	0.00	0.00	0.05	-0.04
5367	PMCH	0.00	0.00	0.00	NaN
5368	PNOC	0.00	0.00	0.00	NaN
5371	PML	0.02	0.00	0.00	0.22
5372	PMM1	0.00	0.14	0.00	-0.06
5373	PMM2	0.00	0.00	0.00	NaN
5375	PMP2	0.06	0.00	0.15	0.03
5376	PMP22	0.00	0.00	0.00	NaN
5378	PMS1	0.00	0.00	0.05	-0.26
5379	PMS2L1	0.02	0.00	0.00	0.08
5380	PMS2L2	0.00	0.00	0.00	NaN
5381	PMS2L3	0.00	0.00	0.00	NaN

5382	PMS2L4	0.04	0.00	0.00	0.00
5389	PMS2L11	0.00	0.00	0.00	NaN
5395	PMS2	0.02	0.00	0.00	-0.25
5406	PNLIP	0.04	0.14	0.05	-0.04
5407	PNLIPRP1	0.04	0.14	0.05	0.07
5408	PNLIPRP2	0.04	0.14	0.05	-0.12
5409	PNMT	0.02	0.86	0.05	0.29
5411	PNN	0.00	0.00	0.10	0.16
5412	UBL3	0.04	0.14	0.00	0.11
5420	PODXL	0.00	0.00	0.00	NaN
5423	POLB	0.04	0.14	0.05	0.19
5424	POLD1	0.00	0.00	0.00	NaN
5425	POLD2	0.00	0.00	0.00	NaN
5426	POLE	0.08	0.14	0.05	-0.10
5427	POLE2	0.00	0.00	0.00	NaN
5428	POLG	0.00	0.00	0.00	NaN
5429	POLH	0.00	0.00	0.00	NaN
5430	POLR2A	0.02	0.00	0.00	0.08
5431	POLR2B	0.06	0.00	0.00	0.31
5432	POLR2C	0.02	0.00	0.00	0.17
5433	POLR2D	0.00	0.00	0.10	0.07
5434	POLR2E	0.02	0.00	0.05	0.10
5435	POLR2F	0.00	0.14	0.00	-0.12
5436	POLR2G	0.00	0.00	0.05	-0.09
5437	POLR2H	0.02	0.14	0.00	-0.08
5438	POLR2I	0.02	0.00	0.05	0.11
5439	POLR2J	0.00	0.00	0.00	NaN
5440	POLR2K	0.12	0.00	0.25	0.34
5441	POLR2L	0.02	0.00	0.05	0.05
5442	POLRMT	0.06	0.00	0.15	0.15
5443	POMC	0.00	0.00	0.00	NaN
5444	PON1	0.00	0.00	0.00	NaN
5445	PON2	0.00	0.00	0.00	NaN
5446	PON3	0.00	0.00	0.00	NaN
5447	POR	0.00	0.00	0.00	NaN
5449	POU1F1	0.16	0.14	0.30	-0.06
5450	POU2AF1	0.02	0.00	0.05	0.13
5451	POU2F1	0.00	0.00	0.10	-0.02
5452	POU2F2	0.00	0.00	0.05	0.28
5453	POU3F1	0.04	0.00	0.05	-0.01
5454	POU3F2	0.08	0.00	0.00	0.16
5455	POU3F3	0.02	0.00	0.10	-0.01
5457	POU4F1	0.02	0.00	0.00	0.01
5458	POU4F2	0.02	0.00	0.05	-0.04
5459	POU4F3	0.00	0.00	0.00	NaN
5460	POU5F1	0.04	0.14	0.05	-0.02
5463	POU6F1	0.00	0.00	0.00	NaN

5465	PPARA	0.02	0.14	0.05	0.09
5467	PPARD	0.04	0.00	0.00	0.00
5468	PPARG	0.00	0.00	0.05	-0.03
5470	PPEF2	0.00	0.00	0.00	NaN
5471	PPAT	0.04	0.00	0.00	0.04
5473	PPBP	0.00	0.00	0.00	NaN
5478	PPIA	0.02	0.00	0.00	0.27
5479	PPIB	0.02	0.00	0.00	0.28
5480	PPIC	0.00	0.00	0.00	NaN
5481	PPID	0.02	0.00	0.00	-0.15
5493	PPL	0.00	0.00	0.00	NaN
5494	PPM1A	0.02	0.14	0.00	0.06
5495	PPM1B	0.02	0.00	0.05	-0.22
5496	PPM1G	0.00	0.00	0.00	NaN
5498	PPOX	0.00	0.00	0.00	NaN
5499	PPP1CA	0.04	0.00	0.00	0.58
5500	PPP1CB	0.00	0.00	0.00	NaN
5501	PPP1CC	0.02	0.00	0.00	-0.09
5502	PPP1R1A	0.02	0.00	0.05	0.10
5504	PPP1R2	0.02	0.00	0.00	0.13
5506	PPP1R3A	0.00	0.00	0.00	NaN
5507	PPP1R3C	0.02	0.00	0.05	-0.08
5509	PPP1R3D	0.12	0.00	0.00	0.16
5510	PPP1R7	0.08	0.43	0.10	0.04
5511	PPP1R8	0.04	0.00	0.00	-0.47
5514	PPP1R10	0.04	0.14	0.05	0.07
5515	PPP2CA	0.00	0.00	0.00	NaN
5516	PPP2CB	0.04	0.00	0.00	0.02
5518	PPP2R1A	0.02	0.00	0.05	-0.05
5519	PPP2R1B	0.00	0.00	0.05	-0.06
5520	PPP2R2A	0.02	0.00	0.00	-0.30
5521	PPP2R2B	0.00	0.00	0.00	NaN
5523	PPP2R3A	0.00	0.00	0.00	NaN
5524	PPP2R4	0.00	0.00	0.00	NaN
5525	PPP2R5A	0.02	0.00	0.15	-0.05
5526	PPP2R5B	0.02	0.00	0.00	-0.03
5527	PPP2R5C	0.02	0.00	0.00	-0.05
5528	PPP2R5D	0.06	0.00	0.05	0.00
5529	PPP2R5E	0.00	0.00	0.00	NaN
5530	PPP3CA	0.08	0.00	0.00	0.24
5531	PPP4C	0.02	0.00	0.00	0.00
5532	PPP3CB	0.00	0.00	0.00	NaN
5533	PPP3CC	0.00	0.00	0.05	-0.03
5534	PPP3R1	0.00	0.00	0.00	NaN
5536	PPP5C	0.00	0.00	0.05	0.31
5537	PPP6C	0.06	0.00	0.00	-0.05
5538	PPT1	0.02	0.00	0.05	-0.20

5539	PPY	0.00	0.14	0.05	0.00
5540	PPYR1	0.02	0.00	0.10	0.03
5542	PRB1	0.02	0.00	0.15	-0.01
5543	PRB2	0.02	0.00	0.15	-0.02
5544	PRB3	0.02	0.00	0.15	-0.05
5546	PRCC	0.04	0.14	0.05	-0.08
5547	PRCP	0.02	0.00	0.00	-0.10
5549	PRELP	0.00	0.00	0.00	NaN
5550	PREP	0.04	0.00	0.00	0.36
5551	PRF1	0.00	0.00	0.00	NaN
5552	PRG1	0.00	0.00	0.00	NaN
5553	PRG2	0.00	0.00	0.00	NaN
5554	PRH1	0.02	0.00	0.15	0.04
5557	PRIM1	0.00	0.00	0.00	NaN
5563	PRKAA2	0.02	0.00	0.00	-0.05
5564	PRKAB1	0.02	0.00	0.10	0.01
5565	PRKAB2	0.02	0.00	0.00	-0.08
5566	PRKACA	0.00	0.00	0.00	NaN
5567	PRKACB	0.04	0.00	0.05	-0.02
5568	PRKACG	0.00	0.00	0.00	NaN
5569	PKIA	0.02	0.00	0.00	0.21
5571	PRKAG1	0.00	0.00	0.00	NaN
5573	PRKAR1A	0.12	0.00	0.20	0.13
5575	PRKAR1B	0.06	0.00	0.05	0.01
5576	PRKAR2A	0.00	0.00	0.05	0.30
5577	PRKAR2B	0.00	0.00	0.05	-0.13
5578	PRKCA	0.12	0.00	0.20	0.09
5580	PRKCD	0.00	0.00	0.10	-0.14
5581	PRKCE	0.02	0.00	0.05	0.26
5582	PRKCG	0.02	0.00	0.05	-0.12
5583	PRKCH	0.00	0.14	0.00	0.18
5584	PRKCI	0.00	0.00	0.05	0.13
5588	PRKCQ	0.00	0.00	0.00	NaN
5589	PRKCSH	0.02	0.00	0.00	0.07
5591	PRKDC	0.04	0.00	0.00	-0.06
5592	PRKG1	0.00	0.00	0.05	0.04
5593	PRKG2	0.02	0.00	0.00	-0.09
5594	MAPK1	0.06	0.14	0.15	0.11
5595	MAPK3	0.02	0.00	0.00	-0.08
5596	MAPK4	0.00	0.00	0.05	0.17
5597	MAPK6	0.00	0.00	0.00	NaN
5598	MAPK7	0.02	0.00	0.00	-0.01
5599	MAPK8	0.02	0.00	0.10	-0.13
5600	MAPK11	0.02	0.14	0.05	0.23
5602	MAPK10	0.00	0.00	0.00	NaN
5603	MAPK13	0.04	0.00	0.00	-0.09
5604	MAP2K1	0.04	0.00	0.00	-0.08

5605	MAP2K2	0.02	0.00	0.15	0.21
5606	MAP2K3	0.10	0.00	0.05	-0.26
5607	MAP2K5	0.02	0.00	0.00	0.31
5608	MAP2K6	0.12	0.00	0.20	0.11
5609	MAP2K7	0.02	0.00	0.15	0.00
5611	DNAJC3	0.04	0.00	0.00	-0.03
5612	PRKRIR	0.04	0.00	0.05	0.12
5617	PRL	0.02	0.14	0.00	0.09
5618	PRLR	0.02	0.14	0.05	-0.06
5619	PRM1	0.02	0.00	0.00	0.16
5620	PRM2	0.02	0.00	0.00	0.10
5621	PRNP	0.00	0.00	0.10	0.33
5623	PSPN	0.02	0.00	0.15	-0.13
5624	PROC	0.00	0.00	0.10	0.08
5625	PRODH	0.02	0.14	0.20	0.02
5626	PROP1	0.00	0.29	0.00	0.00
5627	PROS1	0.16	0.14	0.30	0.19
5629	PROX1	0.02	0.00	0.15	-0.06
5630	PRPH	0.00	0.00	0.00	NaN
5635	PRPSAP1	0.00	0.14	0.05	0.20
5636	PRPSAP2	0.02	0.00	0.00	0.17
5639	PRRG2	0.02	0.00	0.05	0.02
5641	LGMN	0.02	0.00	0.00	-0.26
5644	PRSS1	0.00	0.14	0.05	-0.08
5645	PRSS2	0.00	0.14	0.05	-0.05
5646	PRSS3	0.04	0.00	0.00	0.07
5648	MASP1	0.02	0.14	0.00	0.15
5649	RELN	0.00	0.00	0.00	NaN
5650	KLK7	0.00	0.00	0.00	NaN
5651	PRSS7	0.02	0.00	0.00	-0.08
5652	PRSS8	0.02	0.00	0.00	-0.01
5653	KLK6	0.00	0.00	0.00	NaN
5655	KLK10	0.02	0.00	0.05	0.17
5657	PRTN3	0.06	0.00	0.15	-0.03
5660	PSAP	0.04	0.00	0.05	0.02
5662	PSD	0.02	0.00	0.00	-0.01
5663	PSEN1	0.02	0.00	0.05	0.09
5664	PSEN2	0.00	0.00	0.05	0.10
5669	PSG1	0.00	0.00	0.00	NaN
5670	PSG2	0.00	0.00	0.00	NaN
5671	PSG3	0.00	0.00	0.00	NaN
5672	PSG4	0.00	0.00	0.00	NaN
5673	PSG5	0.00	0.00	0.00	NaN
5675	PSG6	0.00	0.00	0.00	NaN
5676	PSG7	0.00	0.00	0.00	NaN
5678	PSG9	0.00	0.00	0.00	NaN
5680	PSG11	0.00	0.00	0.00	NaN

5682	PSMA1	0.00	0.00	0.15	0.27
5683	PSMA2	0.00	0.00	0.00	NaN
5684	PSMA3	0.02	0.14	0.00	0.03
5685	PSMA4	0.00	0.00	0.05	0.08
5686	PSMA5	0.00	0.00	0.00	NaN
5687	PSMA6	0.04	0.00	0.00	0.10
5688	PSMA7	0.08	0.00	0.05	0.16
5689	PSMB1	0.02	0.00	0.05	0.00
5690	PSMB2	0.00	0.00	0.00	NaN
5691	PSMB3	0.02	0.86	0.05	0.42
5692	PSMB4	0.02	0.00	0.10	0.23
5693	PSMB5	0.00	0.00	0.00	NaN
5694	PSMB6	0.04	0.00	0.10	0.16
5695	PSMB7	0.06	0.00	0.00	-0.13
5696	PSMB8	0.02	0.00	0.05	0.03
5697	PYY	0.00	0.14	0.05	0.16
5698	PSMB9	0.02	0.00	0.05	0.02
5699	PSMB10	0.02	0.00	0.00	0.03
5700	PSMC1	0.00	0.00	0.00	NaN
5702	PSMC3	0.04	0.00	0.00	0.06
5704	PSMC4	0.06	0.00	0.05	0.16
5705	PSMC5	0.12	0.00	0.20	0.38
5706	PSMC6	0.02	0.00	0.00	0.40
5707	PSMD1	0.02	0.00	0.15	0.09
5708	PSMD2	0.02	0.14	0.00	-0.05
5709	PSMD3	0.02	0.86	0.05	0.55
5710	PSMD4	0.02	0.00	0.10	0.28
5711	PSMD5	0.00	0.00	0.00	NaN
5713	PSMD7	0.00	0.00	0.00	NaN
5714	PSMD8	0.00	0.00	0.00	NaN
5715	PSMD9	0.02	0.00	0.10	0.09
5717	PSMD11	0.04	0.00	0.05	0.30
5718	PSMD12	0.12	0.00	0.20	0.16
5719	PSMD13	0.06	0.29	0.10	0.12
5720	PSME1	0.02	0.14	0.10	0.01
5721	PSME2	0.02	0.14	0.10	0.06
5723	PSPH	0.04	0.14	0.20	-0.10
5724	PTAFR	0.04	0.00	0.00	0.34
5725	PTBP1	0.06	0.00	0.15	0.09
5728	PTEN	0.00	0.00	0.00	NaN
5729	PTGDR	0.02	0.00	0.00	0.03
5731	PTGER1	0.00	0.00	0.05	0.16
5732	PTGER2	0.02	0.00	0.00	0.09
5733	PTGER3	0.04	0.14	0.00	0.11
5734	PTGER4	0.04	0.00	0.00	0.01
5737	PTGFR	0.00	0.00	0.00	NaN
5739	PTGIR	0.00	0.00	0.05	0.05

5740	PTGIS	0.06	0.00	0.00	0.14
5741	PTH	0.00	0.00	0.00	NaN
5742	PTGS1	0.06	0.00	0.00	0.16
5743	PTGS2	0.00	0.00	0.05	0.06
5744	PTHLH	0.00	0.00	0.00	NaN
5747	PTK2	0.10	0.00	0.15	0.38
5753	PTK6	0.08	0.00	0.05	0.23
5754	PTK7	0.00	0.00	0.00	NaN
5757	PTMA	0.02	0.00	0.15	-0.05
5763	PTMS	0.02	0.00	0.15	0.10
5764	PTN	0.00	0.00	0.00	NaN
5770	PTPN1	0.10	0.00	0.00	0.23
5771	PTPN2	0.00	0.00	0.05	0.15
5774	PTPN3	0.00	0.00	0.00	NaN
5775	PTPN4	0.00	0.00	0.10	-0.17
5777	PTPN6	0.02	0.00	0.15	0.05
5778	PTPN7	0.02	0.00	0.00	-0.03
5780	PTPN9	0.00	0.00	0.05	-0.05
5781	PTPN11	0.02	0.00	0.00	0.14
5782	PTPN12	0.00	0.00	0.00	NaN
5783	PTPN13	0.00	0.00	0.00	NaN
5784	PTPN14	0.02	0.00	0.15	0.22
5786	PTPRA	0.00	0.00	0.00	NaN
5787	PTPRB	0.12	0.00	0.05	-0.01
5788	PTPRC	0.02	0.00	0.00	-0.02
5789	PTPRD	0.02	0.00	0.00	-0.11
5790	PTPRCAP	0.02	0.00	0.00	0.00
5791	PTPRE	0.04	0.00	0.00	-0.10
5792	PTPRF	0.02	0.00	0.05	0.36
5793	PTPRG	0.04	0.00	0.00	-0.08
5794	PTPRH	0.02	0.00	0.05	-0.04
5795	PTPRJ	0.04	0.00	0.00	0.25
5796	PTPRK	0.00	0.00	0.10	0.04
5797	PTPRM	0.02	0.00	0.05	-0.12
5798	PTPRN	0.04	0.00	0.00	-0.01
5799	PTPRN2	0.20	0.14	0.10	0.00
5801	PTPRR	0.12	0.00	0.05	0.19
5802	PTPRS	0.02	0.00	0.15	0.07
5803	PTPRZ1	0.00	0.00	0.00	NaN
5805	PTS	0.02	0.00	0.00	-0.11
5806	PTX3	0.02	0.00	0.10	-0.06
5810	RAD1	0.02	0.14	0.05	0.15
5813	PURA	0.00	0.00	0.00	NaN
5816	PVALB	0.00	0.14	0.00	0.17
5817	PVR	0.00	0.00	0.00	NaN
5818	PVRL1	0.00	0.00	0.00	NaN
5819	PVRL2	0.00	0.00	0.00	NaN

5825	ABCD3	0.00	0.00	0.00	NaN
5826	ABCD4	0.02	0.00	0.05	0.16
5827	PXMP2	0.08	0.14	0.05	-0.06
5828	PXMP3	0.06	0.00	0.00	0.15
5829	PXN	0.02	0.00	0.10	0.07
5831	PYCR1	0.00	0.00	0.00	NaN
5833	PCYT2	0.00	0.00	0.00	NaN
5834	PYGB	0.04	0.00	0.15	0.09
5836	PYGL	0.02	0.00	0.00	0.30
5837	PYGM	0.02	0.00	0.00	-0.04
5858	PZP	0.02	0.00	0.15	-0.06
5859	QARS	0.00	0.00	0.05	0.26
5860	QDPR	0.00	0.00	0.00	NaN
5861	RAB1A	0.00	0.00	0.00	NaN
5864	RAB3A	0.02	0.00	0.00	0.05
5865	RAB3B	0.02	0.00	0.00	0.08
5866	RAB3IL1	0.00	0.00	0.05	0.01
5867	RAB4A	0.00	0.00	0.05	0.06
5868	RAB5A	0.06	0.00	0.05	0.05
5869	RAB5B	0.02	0.00	0.05	0.05
5870	RAB6A	0.02	0.00	0.00	0.32
5871	MAP4K2	0.02	0.00	0.00	0.13
5872	RAB13	0.02	0.00	0.10	0.25
5873	RAB27A	0.00	0.00	0.00	NaN
5874	RAB27B	0.00	0.00	0.05	-0.04
5875	RABGGTA	0.02	0.14	0.10	0.00
5876	RABGGTB	0.02	0.00	0.05	-0.07
5877	RABIF	0.02	0.00	0.00	-0.12
5878	RAB5C	0.02	0.86	0.05	0.14
5879	RAC1	0.02	0.00	0.00	-0.05
5880	RAC2	0.00	0.14	0.00	-0.01
5881	RAC3	0.00	0.00	0.00	NaN
5884	RAD17	0.06	0.00	0.00	-0.09
5885	RAD21	0.08	0.14	0.00	0.10
5886	RAD23A	0.00	0.14	0.05	-0.09
5887	RAD23B	0.00	0.00	0.00	NaN
5888	RAD51	0.00	0.00	0.00	NaN
5889	RAD51C	0.06	0.00	0.05	0.22
5890	RAD51L1	0.00	0.00	0.00	NaN
5891	RAGE	0.02	0.00	0.00	-0.14
5892	RAD51L3	0.06	0.00	0.00	0.39
5893	RAD52	0.08	0.14	0.10	0.20
5894	RAF1	0.00	0.00	0.05	-0.32
5896	RAG1	0.02	0.00	0.00	0.01
5897	RAG2	0.02	0.00	0.00	0.05
5898	RALA	0.00	0.00	0.00	NaN
5899	RALB	0.00	0.00	0.10	-0.11

5900	RALGDS	0.04	0.00	0.00	-0.01
5901	RAN	0.08	0.14	0.05	0.03
5902	RANBP1	0.06	0.14	0.15	0.14
5903	RANBP2	0.02	0.00	0.00	-0.07
5905	RANGAP1	0.00	0.14	0.00	0.12
5906	RAP1A	0.00	0.00	0.00	NaN
5910	RAP1GDS1	0.02	0.00	0.00	-0.11
5911	RAP2A	0.00	0.00	0.00	NaN
5912	RAP2B	0.02	0.00	0.10	0.08
5913	RAPSN	0.04	0.00	0.00	-0.25
5914	RARA	0.02	0.86	0.05	0.13
5915	RARB	0.02	0.00	0.05	0.11
5916	RARG	0.00	0.00	0.00	NaN
5917	RARS	0.00	0.00	0.00	NaN
5918	RARRES1	0.02	0.00	0.10	0.06
5919	RARRES2	0.06	0.00	0.15	-0.03
5920	RARRES3	0.02	0.00	0.00	-0.05
5921	RASA1	0.00	0.00	0.00	NaN
5922	RASA2	0.02	0.00	0.00	0.05
5923	RASGRF1	0.02	0.00	0.00	0.00
5925	RB1	0.00	0.00	0.00	NaN
5928	RBBP4	0.00	0.00	0.00	NaN
5929	RBBP5	0.02	0.00	0.05	0.03
5930	RBBP6	0.06	0.14	0.00	0.05
5932	RBBP8	0.00	0.14	0.00	-0.13
5934	RBL2	0.02	0.00	0.00	0.11
5936	RBM4	0.04	0.00	0.05	-0.19
5937	RBMS1	0.00	0.00	0.05	0.07
5939	RBMS2	0.00	0.00	0.00	NaN
5947	RBP1	0.00	0.00	0.05	0.11
5949	RBP3	0.02	0.00	0.10	0.05
5950	RBP4	0.02	0.00	0.05	0.10
5954	RCN1	0.00	0.00	0.00	NaN
5955	RCN2	0.00	0.00	0.05	0.06
5959	RDH5	0.02	0.00	0.05	0.04
5962	RDX	0.02	0.00	0.05	0.18
5965	RECQL	0.02	0.00	0.05	-0.13
5966	REL	0.00	0.00	0.05	0.08
5967	REG1A	0.00	0.00	0.00	NaN
5968	REG1B	0.00	0.00	0.00	NaN
5970	RELA	0.04	0.00	0.05	0.05
5971	RELB	0.00	0.00	0.00	NaN
5972	REN	0.00	0.00	0.15	-0.17
5978	REST	0.06	0.00	0.00	0.03
5979	RET	0.02	0.00	0.05	0.06
5980	REV3L	0.02	0.00	0.10	0.34
5981	RFC1	0.04	0.00	0.10	-0.03

5982	RFC2	0.00	0.00	0.00	NaN
5983	RFC3	0.02	0.00	0.00	0.08
5984	RFC4	0.02	0.14	0.00	-0.10
5985	RFC5	0.02	0.00	0.10	-0.03
5986	RFNG	0.00	0.00	0.00	NaN
5988	RFPL1	0.00	0.14	0.00	-0.11
5989	RFX1	0.00	0.00	0.00	NaN
5990	RFX2	0.02	0.00	0.15	-0.16
5991	RFX3	0.02	0.00	0.00	-0.14
5992	RFX4	0.00	0.00	0.00	NaN
5993	RFX5	0.02	0.00	0.10	0.45
5994	RFXAP	0.02	0.00	0.00	-0.09
5995	RGR	0.06	0.14	0.05	-0.08
5996	RGS1	0.00	0.00	0.00	NaN
5997	RGS2	0.00	0.00	0.00	NaN
5998	RGS3	0.00	0.00	0.00	NaN
5999	RGS4	0.00	0.00	0.00	NaN
6000	RGS7	0.06	0.00	0.00	0.13
6001	RGS10	0.04	0.00	0.10	0.14
6002	RGS12	0.00	0.00	0.00	NaN
6003	RGS13	0.00	0.00	0.00	NaN
6004	RGS16	0.02	0.00	0.00	-0.02
6005	RHAG	0.00	0.00	0.00	NaN
6006	RHCE	0.02	0.00	0.05	-0.05
6007	RHD	0.02	0.00	0.05	-0.07
6010	RHO	0.02	0.00	0.00	0.00
6013	RLN1	0.02	0.00	0.00	0.17
6015	RING1	0.02	0.00	0.05	0.29
6017	RLBP1	0.00	0.00	0.00	NaN
6018	RLF	0.02	0.00	0.05	-0.05
6019	RLN2	0.02	0.00	0.00	0.03
6036	RNASE2	0.02	0.00	0.00	-0.06
6037	RNASE3	0.02	0.00	0.00	-0.08
6038	RNASE4	0.02	0.00	0.00	-0.02
6039	RNASE6	0.02	0.00	0.00	-0.02
6040	RNASEH1	0.20	0.14	0.35	0.07
6041	RNASEL	0.02	0.00	0.00	-0.19
6045	RNF2	0.00	0.00	0.05	-0.02
6046	BRD2	0.02	0.00	0.05	0.20
6047	RNF4	0.00	0.29	0.00	-0.21
6048	RNF5	0.02	0.00	0.05	0.05
6049	RNF6	0.00	0.00	0.05	0.07
6051	RNPEP	0.02	0.00	0.00	-0.11
6059	ABCE1	0.00	0.00	0.00	NaN
6091	ROBO1	0.16	0.14	0.30	-0.07
6093	ROCK1	0.08	0.14	0.10	0.03
6094	ROM1	0.00	0.00	0.05	0.06

6095	RORA	0.00	0.00	0.00	NaN
6096	RORB	0.00	0.00	0.00	NaN
6097	RORC	0.02	0.00	0.10	-0.01
6098	ROS1	0.06	0.00	0.05	-0.08
6117	RPA1	0.04	0.00	0.05	0.08
6118	RPA2	0.04	0.00	0.00	-0.46
6119	RPA3	0.00	0.00	0.00	NaN
6120	RPE	0.02	0.00	0.05	0.16
6121	RPE65	0.02	0.00	0.00	-0.07
6122	RPL3	0.00	0.14	0.05	0.03
6123	RPL3L	0.18	0.00	0.10	-0.02
6124	RPL4	0.04	0.00	0.00	0.04
6125	RPL5	0.00	0.00	0.00	NaN
6128	RPL6	0.02	0.00	0.00	-0.07
6129	RPL7	0.02	0.00	0.00	-0.04
6130	RPL7A	0.04	0.00	0.00	-0.21
6132	RPL8	0.10	0.00	0.15	0.33
6133	RPL9	0.04	0.00	0.10	-0.26
6135	RPL11	0.02	0.00	0.05	-0.07
6136	RPL12	0.02	0.00	0.00	-0.05
6137	RPL13	0.00	0.00	0.10	0.04
6138	RPL15	0.02	0.00	0.05	0.10
6141	RPL18	0.06	0.00	0.05	0.27
6142	RPL18A	0.04	0.00	0.00	0.17
6143	RPL19	0.02	0.86	0.05	0.32
6144	RPL21	0.00	0.00	0.05	0.08
6146	RPL22	0.02	0.00	0.00	0.11
6147	RPL23A	0.10	0.00	0.05	0.09
6150	MRPL23	0.02	0.00	0.05	-0.12
6152	RPL24	0.00	0.00	0.00	NaN
6155	RPL27	0.00	0.86	0.00	0.14
6156	RPL30	0.06	0.14	0.10	0.15
6157	RPL27A	0.00	0.00	0.00	NaN
6158	RPL28	0.02	0.00	0.05	0.26
6159	RPL29	0.00	0.00	0.10	-0.08
6160	RPL31	0.02	0.00	0.10	0.15
6161	RPL32	0.00	0.00	0.05	0.10
6164	RPL34	0.08	0.00	0.00	-0.22
6165	RPL35A	0.20	0.14	0.10	0.21
6166	RPL36AL	0.00	0.00	0.00	NaN
6167	RPL37	0.04	0.00	0.00	0.39
6168	RPL37A	0.02	0.00	0.00	-0.06
6169	RPL38	0.04	0.00	0.00	0.09
6171	RPL41	0.00	0.00	0.00	NaN
6175	RPLP0	0.02	0.00	0.10	0.04
6176	RPLP1	0.00	0.00	0.00	NaN
6181	RPLP2	0.02	0.00	0.05	0.25

6182	MRPL12	0.00	0.00	0.00	NaN
6183	MRPS12	0.06	0.00	0.05	0.16
6184	RPN1	0.04	0.00	0.00	0.37
6185	RPN2	0.04	0.00	0.00	0.14
6187	RPS2	0.18	0.00	0.10	0.12
6188	RPS3	0.02	0.14	0.00	-0.20
6189	RPS3A	0.02	0.14	0.10	0.10
6193	RPS5	0.00	0.00	0.00	NaN
6194	RPS6	0.02	0.00	0.05	-0.02
6195	RPS6KA1	0.02	0.00	0.05	-0.06
6196	RPS6KA2	0.02	0.00	0.05	-0.03
6198	RPS6KB1	0.06	0.00	0.05	0.44
6199	RPS6KB2	0.02	0.00	0.00	0.36
6201	RPS7	0.20	0.14	0.35	0.01
6202	RPS8	0.02	0.00	0.05	0.31
6203	RPS9	0.02	0.00	0.05	-0.01
6204	RPS10	0.00	0.00	0.00	NaN
6205	RPS11	0.02	0.00	0.05	-0.01
6206	RPS12	0.02	0.00	0.00	0.00
6207	RPS13	0.00	0.00	0.10	0.08
6208	RPS14	0.00	0.14	0.00	0.04
6209	RPS15	0.02	0.00	0.05	0.02
6210	RPS15A	0.00	0.00	0.00	NaN
6217	RPS16	0.06	0.00	0.05	-0.01
6218	RPS17	0.02	0.00	0.00	0.03
6222	RPS18	0.02	0.00	0.05	0.06
6223	RPS19	0.00	0.00	0.05	0.44
6224	RPS20	0.04	0.00	0.00	-0.09
6227	RPS21	0.08	0.00	0.05	0.31
6228	RPS23	0.04	0.00	0.00	0.06
6229	RPS24	0.02	0.00	0.10	-0.04
6230	RPS25	0.00	0.00	0.05	0.00
6231	RPS26	0.02	0.00	0.05	-0.08
6232	RPS27	0.02	0.00	0.10	0.20
6233	RPS27A	0.02	0.00	0.05	-0.02
6234	RPS28	0.02	0.00	0.15	0.12
6235	RPS29	0.00	0.00	0.00	NaN
6236	RRAD	0.02	0.00	0.00	0.05
6237	RRAS	0.02	0.00	0.05	0.02
6238	RRBP1	0.00	0.00	0.00	NaN
6239	RREB1	0.02	0.00	0.05	0.12
6240	RRM1	0.00	0.00	0.00	NaN
6241	RRM2	0.00	0.00	0.00	NaN
6248	RSC1A1	0.02	0.00	0.00	-0.04
6251	RSU1	0.02	0.14	0.05	0.05
6252	RTN1	0.02	0.14	0.00	0.01
6253	RTN2	0.00	0.00	0.00	NaN

6256	RXRA	0.04	0.00	0.00	-0.13
6257	RXRB	0.02	0.00	0.05	0.07
6258	RXRG	0.02	0.00	0.00	-0.06
6259	RYK	0.00	0.00	0.10	0.25
6261	RYR1	0.00	0.00	0.00	NaN
6262	RYR2	0.00	0.00	0.00	NaN
6263	RYR3	0.04	0.00	0.05	-0.05
6271	S100A1	0.02	0.00	0.10	0.11
6272	SORT1	0.00	0.00	0.00	NaN
6273	S100A2	0.02	0.00	0.10	-0.13
6274	S100A3	0.02	0.00	0.10	-0.03
6275	S100A4	0.02	0.00	0.10	0.16
6276	S100A5	0.02	0.00	0.10	0.15
6277	S100A6	0.02	0.00	0.10	0.11
6278	S100A7	0.02	0.00	0.10	0.18
6279	S100A8	0.02	0.00	0.10	0.16
6280	S100A9	0.02	0.00	0.10	0.17
6281	S100A10	0.02	0.00	0.10	-0.02
6282	S100A11	0.02	0.00	0.10	0.02
6283	S100A12	0.02	0.00	0.10	0.46
6284	S100A13	0.02	0.00	0.10	-0.13
6285	S100B	0.04	0.00	0.05	-0.09
6286	S100P	0.00	0.00	0.00	NaN
6289	SAA2	0.00	0.00	0.05	0.00
6291	SAA4	0.00	0.00	0.05	0.00
6294	SAFB	0.02	0.00	0.15	0.13
6295	SAG	0.02	0.00	0.00	-0.08
6297	SALL2	0.02	0.00	0.00	-0.15
6299	SALL1	0.02	0.00	0.00	-0.03
6301	SARS	0.06	0.00	0.20	0.21
6304	SATB1	0.06	0.00	0.05	-0.04
6307	SC4MOL	0.02	0.00	0.00	0.03
6309	SC5DL	0.00	0.00	0.00	NaN
6317	SERPINB3	0.02	0.00	0.00	-0.02
6318	SERPINB4	0.02	0.00	0.00	-0.03
6319	SCD	0.00	0.00	0.05	-0.07
6323	SCN1A	0.02	0.00	0.00	-0.04
6324	SCN1B	0.00	0.00	0.00	NaN
6327	SCN2B	0.00	0.00	0.00	NaN
6328	SCN3A	0.02	0.00	0.00	-0.09
6329	SCN4A	0.12	0.00	0.20	0.01
6331	SCN5A	0.02	0.00	0.05	0.06
6334	SCN8A	0.00	0.00	0.00	NaN
6335	SCN9A	0.02	0.00	0.00	0.08
6336	SCN10A	0.02	0.00	0.05	-0.02
6337	SCNN1A	0.02	0.00	0.15	-0.14
6338	SCNN1B	0.02	0.14	0.00	0.01

6340	SCNN1G	0.02	0.14	0.00	-0.06
6342	SCP2	0.02	0.00	0.00	0.19
6343	SCT	0.02	0.00	0.05	0.37
6344	SCTR	0.00	0.00	0.10	-0.06
6382	SDC1	0.00	0.00	0.00	NaN
6383	SDC2	0.08	0.00	0.05	0.05
6385	SDC4	0.04	0.00	0.05	0.23
6386	SDCBP	0.04	0.00	0.00	-0.10
6388	SDF2	0.10	0.00	0.05	0.15
6389	SDHA	0.00	0.00	0.00	NaN
6390	SDHB	0.02	0.00	0.00	-0.10
6391	SDHC	0.00	0.00	0.00	NaN
6392	SDHD	0.02	0.00	0.00	-0.13
6397	SEC14L1	0.00	0.14	0.05	0.07
6398	SECTM1	0.00	0.00	0.00	NaN
6400	SEL1L	0.02	0.00	0.00	0.02
6401	SELE	0.02	0.00	0.00	0.00
6402	SELL	0.02	0.00	0.00	-0.06
6403	SELP	0.02	0.00	0.00	0.00
6404	SELPLG	0.02	0.00	0.00	-0.09
6405	SEMA3F	0.00	0.00	0.05	-0.08
6406	SEMG1	0.04	0.00	0.00	-0.12
6414	SEPP1	0.18	0.00	0.30	0.10
6415	SEPW1	0.00	0.00	0.00	NaN
6416	MAP2K4	0.04	0.00	0.00	0.07
6418	SET	0.00	0.00	0.00	NaN
6419	SETMAR	0.00	0.00	0.05	0.04
6421	SFPQ	0.00	0.00	0.00	NaN
6422	SFRP1	0.10	0.29	0.00	-0.16
6424	SFRP4	0.00	0.00	0.00	NaN
6425	SFRP5	0.00	0.14	0.00	-0.02
6426	SFRS1	0.04	0.00	0.05	0.27
6427	SFRS2	0.00	0.14	0.05	0.04
6428	SFRS3	0.00	0.00	0.00	NaN
6429	SFRS4	0.04	0.00	0.00	-0.18
6430	SFRS5	0.02	0.00	0.05	0.17
6431	SFRS6	0.02	0.00	0.00	0.08
6432	SFRS7	0.02	0.00	0.05	0.48
6433	SFRS8	0.08	0.14	0.05	-0.05
6436	SFTPA2	0.02	0.00	0.10	-0.02
6439	SFTPB	0.00	0.00	0.00	NaN
6440	SFTPC	0.00	0.00	0.05	0.21
6441	SFTPD	0.02	0.00	0.10	0.25
6442	SGCA	0.06	0.00	0.00	0.10
6443	SGCB	0.02	0.00	0.05	0.23
6444	SGCD	0.00	0.00	0.00	NaN
6445	SGCG	0.02	0.00	0.00	-0.02

6448	SGSH	0.00	0.14	0.00	0.10
6450	SH3BGR	0.00	0.00	0.00	NaN
6452	SH3BP2	0.00	0.29	0.00	0.00
6453	ITSN1	0.00	0.00	0.00	NaN
6455	SH3GL1	0.02	0.00	0.15	-0.13
6456	SH3GL2	0.02	0.00	0.05	-0.02
6457	SH3GL3	0.00	0.00	0.00	NaN
6461	SHB	0.02	0.00	0.00	-0.09
6462	SHBG	0.00	0.00	0.00	NaN
6464	SHC1	0.04	0.00	0.15	0.09
6469	SHH	0.00	0.00	0.05	-0.23
6470	SHMT1	0.02	0.00	0.00	-0.06
6472	SHMT2	0.00	0.00	0.00	NaN
6474	SHOX2	0.02	0.00	0.10	0.07
6476	SI	0.00	0.00	0.00	NaN
6477	SIAH1	0.43	0.29	0.15	-0.19
6478	SIAH2	0.02	0.00	0.05	-0.22
6490	SILV	0.02	0.00	0.05	0.03
6493	SIM2	0.00	0.00	0.00	NaN
6494	SIPA1	0.04	0.00	0.05	0.07
6495	SIX1	0.00	0.14	0.00	-0.12
6496	SIX3	0.02	0.00	0.05	-0.04
6498	SKIL	0.00	0.00	0.00	NaN
6499	SKIV2L	0.02	0.00	0.05	0.31
6502	SKP2	0.04	0.00	0.15	0.06
6503	SLA	0.06	0.00	0.00	0.12
6505	SLC1A1	0.00	0.00	0.00	NaN
6506	SLC1A2	0.02	0.00	0.00	0.00
6507	SLC1A3	0.04	0.00	0.15	0.26
6508	SLC4A3	0.04	0.00	0.00	0.01
6509	SLC1A4	0.00	0.00	0.00	NaN
6510	SLC1A5	0.00	0.00	0.05	0.18
6511	SLC1A6	0.04	0.00	0.00	0.05
6512	SLC1A7	0.02	0.00	0.00	-0.12
6513	SLC2A1	0.02	0.00	0.05	0.35
6514	SLC2A2	0.02	0.00	0.00	0.08
6515	SLC2A3	0.02	0.00	0.15	0.17
6517	SLC2A4	0.00	0.00	0.00	NaN
6518	SLC2A5	0.00	0.00	0.00	NaN
6519	SLC3A1	0.02	0.00	0.05	0.01
6520	SLC3A2	0.00	0.00	0.00	NaN
6521	SLC4A1	0.00	0.00	0.00	NaN
6522	SLC4A2	0.02	0.00	0.00	-0.12
6523	SLC5A1	0.00	0.29	0.05	0.02
6524	SLC5A2	0.02	0.00	0.00	0.00
6525	SMTN	0.00	0.29	0.05	0.37
6527	SLC5A4	0.00	0.29	0.05	-0.01

6528	SLC5A5	0.04	0.00	0.00	0.02
6529	SLC6A1	0.02	0.00	0.05	0.05
6530	SLC6A2	0.02	0.00	0.00	-0.03
6531	SLC6A3	0.00	0.00	0.00	NaN
6532	SLC6A4	0.10	0.00	0.05	-0.18
6533	SLC6A6	0.00	0.00	0.05	0.03
6534	SLC6A7	0.00	0.00	0.00	NaN
6536	SLC6A9	0.02	0.00	0.05	-0.11
6538	SLC6A11	0.02	0.00	0.05	0.04
6540	SLC6A13	0.08	0.14	0.10	0.02
6541	SLC7A1	0.00	0.00	0.05	0.13
6542	SLC7A2	0.00	0.00	0.00	NaN
6543	SLC8A2	0.00	0.00	0.05	-0.06
6545	SLC7A4	0.06	0.14	0.15	-0.15
6546	SLC8A1	0.02	0.00	0.05	0.14
6548	SLC9A1	0.06	0.00	0.05	-0.10
6549	SLC9A2	0.02	0.00	0.10	0.03
6550	SLC9A3	0.00	0.00	0.00	NaN
6553	SLC9A5	0.02	0.00	0.00	0.08
6554	SLC10A1	0.02	0.00	0.05	0.06
6555	SLC10A2	0.02	0.00	0.00	-0.03
6556	SLC11A1	0.04	0.00	0.00	0.01
6557	SLC12A1	0.02	0.00	0.05	-0.10
6558	SLC12A2	0.00	0.00	0.00	NaN
6559	SLC12A3	0.00	0.14	0.00	0.09
6560	SLC12A4	0.02	0.00	0.00	0.15
6561	SLC13A1	0.00	0.00	0.00	NaN
6563	SLC14A1	0.02	0.00	0.00	-0.09
6564	SLC15A1	0.00	0.00	0.05	0.03
6565	SLC15A2	0.00	0.00	0.10	0.25
6566	SLC16A1	0.02	0.00	0.05	-0.06
6568	SLC17A1	0.02	0.00	0.05	-0.07
6569	SLC34A1	0.00	0.29	0.00	-0.18
6570	SLC18A1	0.00	0.00	0.00	NaN
6571	SLC18A2	0.04	0.14	0.05	-0.04
6572	SLC18A3	0.02	0.00	0.05	0.02
6573	SLC19A1	0.00	0.00	0.05	0.02
6574	SLC20A1	0.00	0.00	0.00	NaN
6575	SLC20A2	0.04	0.14	0.05	0.34
6576	SLC25A1	0.02	0.14	0.20	0.25
6580	SLC22A1	0.02	0.00	0.05	0.18
6581	SLC22A3	0.02	0.00	0.05	-0.15
6582	SLC22A2	0.02	0.00	0.05	-0.03
6583	SLC22A4	0.02	0.00	0.00	-0.02
6584	SLC22A5	0.02	0.00	0.00	-0.03
6585	SLIT1	0.00	0.00	0.00	NaN
6586	SLIT3	0.00	0.00	0.00	NaN

6588	SLN	0.04	0.00	0.00	-0.07
6590	SLPI	0.04	0.00	0.05	0.04
6591	SNAI2	0.02	0.00	0.00	-0.02
6595	SMARCA2	0.04	0.00	0.10	0.19
6597	SMARCA4	0.02	0.00	0.15	0.28
6598	SMARCB1	0.00	0.14	0.05	-0.09
6599	SMARCC1	0.02	0.14	0.15	0.35
6601	SMARCC2	0.00	0.00	0.00	NaN
6602	SMARCD1	0.02	0.00	0.00	0.00
6604	SMARCD3	0.02	0.00	0.00	-0.13
6605	SMARCE1	0.02	0.86	0.05	0.16
6606	SMN1	0.06	0.00	0.00	0.08
6609	SMPD1	0.00	0.00	0.00	NaN
6610	SMPD2	0.02	0.00	0.10	0.23
6615	SNAI1	0.06	0.00	0.05	0.09
6616	SNAP25	0.00	0.00	0.05	0.13
6617	SNAPC1	0.00	0.14	0.00	0.43
6618	SNAPC2	0.02	0.00	0.15	0.04
6619	SNAPC3	0.04	0.00	0.05	0.39
6620	SNCB	0.00	0.00	0.00	NaN
6622	SNCA	0.00	0.00	0.00	NaN
6623	SNCG	0.00	0.00	0.05	-0.08
6626	SNRPA	0.00	0.00	0.05	0.43
6627	SNRPA1	0.16	0.00	0.10	-0.07
6628	SNRPB	0.00	0.00	0.00	NaN
6629	SNRPB2	0.00	0.00	0.10	-0.11
6631	SNRPC	0.00	0.00	0.00	NaN
6632	SNRPD1	0.00	0.14	0.00	0.05
6633	SNRPD2	0.00	0.00	0.05	0.29
6634	SNRPD3	0.00	0.14	0.05	0.08
6635	SNRPE	0.00	0.00	0.15	0.11
6636	SNRPF	0.02	0.00	0.00	0.24
6637	SNRPG	0.00	0.14	0.00	-0.02
6638	SNRPN	0.00	0.14	0.00	0.10
6640	SNTA1	0.00	0.00	0.00	NaN
6641	SNTB1	0.10	0.00	0.15	0.27
6642	SNX1	0.02	0.00	0.00	-0.15
6643	SNX2	0.00	0.00	0.00	NaN
6645	SNTB2	0.00	0.00	0.00	NaN
6646	SOAT1	0.04	0.00	0.10	0.01
6647	SOD1	0.00	0.00	0.00	NaN
6648	SOD2	0.04	0.00	0.05	0.02
6649	SOD3	0.00	0.00	0.00	NaN
6650	SOLH	0.18	0.00	0.10	-0.11
6651	SON	0.00	0.00	0.00	NaN
6652	SORD	0.02	0.00	0.05	-0.05
6653	SORL1	0.00	0.00	0.00	NaN

6654	SOS1	0.02	0.00	0.05	0.16
6655	SOS2	0.00	0.00	0.00	NaN
6656	SOX1	0.06	0.00	0.00	-0.02
6657	SOX2	0.00	0.00	0.10	-0.03
6659	SOX4	0.00	0.00	0.00	NaN
6660	SOX5	0.06	0.00	0.00	0.00
6662	SOX9	0.02	0.00	0.05	0.17
6663	SOX10	0.00	0.14	0.00	-0.08
6664	SOX11	0.20	0.14	0.35	-0.04
6667	SP1	0.00	0.00	0.00	NaN
6668	SP2	0.04	0.00	0.05	0.13
6670	SP3	0.00	0.00	0.00	NaN
6671	SP4	0.00	0.00	0.00	NaN
6672	SP100	0.02	0.00	0.15	0.09
6674	SPAG1	0.12	0.00	0.25	0.14
6675	UAP1	0.00	0.00	0.00	NaN
6676	SPAG4	0.06	0.14	0.05	0.30
6677	SPAM1	0.00	0.00	0.00	NaN
6678	SPARC	0.00	0.14	0.00	0.23
6687	SPG7	0.00	0.00	0.10	0.10
6688	SPI1	0.04	0.00	0.00	-0.04
6689	SPIB	0.00	0.00	0.00	NaN
6690	SPINK1	0.00	0.00	0.00	NaN
6691	SPINK2	0.06	0.00	0.00	-0.25
6692	SPINT1	0.00	0.00	0.00	NaN
6693	SPN	0.06	0.14	0.00	0.00
6694	SPP2	0.02	0.00	0.00	0.26
6696	SPP1	0.02	0.00	0.00	0.10
6697	SPR	0.00	0.00	0.05	-0.09
6698	SPRR1A	0.02	0.00	0.10	-0.04
6699	SPRR1B	0.02	0.00	0.10	-0.01
6701	SPRR2B	0.02	0.00	0.10	-0.08
6702	SPRR2C	0.02	0.00	0.10	0.12
6707	SPRR3	0.02	0.00	0.10	-0.09
6708	SPTA1	0.00	0.00	0.00	NaN
6709	SPTAN1	0.00	0.00	0.00	NaN
6710	SPTB	0.06	0.00	0.00	0.01
6711	SPTBN1	0.02	0.00	0.05	0.20
6712	SPTBN2	0.04	0.00	0.05	0.22
6713	SQLE	0.10	0.00	0.05	0.44
6714	SRC	0.04	0.00	0.00	0.14
6715	SRD5A1	0.00	0.00	0.00	NaN
6716	SRD5A2	0.00	0.00	0.00	NaN
6717	SRI	0.00	0.00	0.00	NaN
6718	AKR1D1	0.00	0.00	0.00	NaN
6720	SREBF1	0.02	0.00	0.00	0.08
6721	SREBF2	0.00	0.14	0.05	-0.05

6722	SRF	0.00	0.00	0.00	NaN
6723	SRM	0.02	0.00	0.00	0.06
6726	SRP9	0.00	0.00	0.05	0.09
6727	SRP14	0.00	0.00	0.00	NaN
6728	SRP19	0.00	0.00	0.00	NaN
6729	SRP54	0.04	0.00	0.00	0.06
6731	SRP72	0.04	0.00	0.00	0.07
6732	SRPK1	0.04	0.00	0.00	0.05
6733	SRPK2	0.00	0.00	0.10	-0.28
6734	SRPR	0.00	0.00	0.00	NaN
6741	SSB	0.00	0.00	0.00	NaN
6742	SSBP1	0.00	0.14	0.05	-0.03
6744	SSFA2	0.00	0.00	0.00	NaN
6745	SSR1	0.02	0.00	0.05	0.06
6746	SSR2	0.04	0.14	0.05	-0.13
6747	SSR3	0.02	0.00	0.10	-0.08
6749	SSRP1	0.00	0.00	0.00	NaN
6750	SST	0.00	0.14	0.00	0.06
6751	SSTR1	0.04	0.00	0.00	-0.02
6752	SSTR2	0.02	0.00	0.05	0.03
6753	SSTR3	0.00	0.14	0.00	0.12
6754	SSTR4	0.02	0.00	0.00	-0.05
6755	SSTR5	0.18	0.00	0.10	-0.17
6760	SS18	0.00	0.00	0.10	0.42
6764	ST5	0.00	0.00	0.00	NaN
6767	ST13	0.00	0.14	0.00	0.01
6768	ST14	0.02	0.00	0.00	-0.03
6769	STAC	0.02	0.14	0.10	0.08
6770	STAR	0.12	0.14	0.05	-0.16
6772	STAT1	0.00	0.00	0.05	-0.10
6773	STAT2	0.00	0.00	0.00	NaN
6774	STAT3	0.02	0.86	0.05	0.06
6775	STAT4	0.00	0.00	0.05	-0.13
6776	STAT5A	0.02	0.86	0.05	-0.06
6777	STAT5B	0.02	0.86	0.05	0.01
6778	STAT6	0.00	0.00	0.00	NaN
6779	STATH	0.00	0.14	0.00	0.02
6781	STC1	0.00	0.00	0.00	NaN
6785	ELOVL4	0.10	0.00	0.20	-0.03
6786	STIM1	0.00	0.00	0.00	NaN
6787	NEK4	0.00	0.00	0.10	0.08
6788	STK3	0.06	0.14	0.05	0.32
6789	STK4	0.02	0.00	0.00	0.43
6793	STK10	0.00	0.00	0.10	0.05
6794	STK11	0.02	0.00	0.05	0.08
6799	SULT1A2	0.06	0.14	0.00	0.23
6801	STRN	0.00	0.00	0.00	NaN

6804	STX1A	0.00	0.00	0.00	NaN
6812	STXBP1	0.02	0.00	0.00	0.06
6813	STXBP2	0.02	0.00	0.15	-0.08
6814	STXBP3	0.06	0.00	0.20	0.11
6817	SULT1A1	0.06	0.14	0.00	0.18
6818	SULT1A3	0.00	0.00	0.00	NaN
6820	SULT2B1	0.02	0.00	0.00	0.27
6821	SUOX	0.02	0.00	0.05	-0.18
6822	SULT2A1	0.00	0.00	0.00	NaN
6827	SUPT4H1	0.04	0.00	0.05	0.17
6829	SUPT5H	0.06	0.00	0.05	0.09
6830	SUPT6H	0.10	0.00	0.05	0.18
6832	SUPV3L1	0.00	0.00	0.00	NaN
6833	ABCC8	0.00	0.00	0.00	NaN
6834	SURF1	0.04	0.00	0.00	-0.08
6835	SURF2	0.04	0.00	0.00	-0.20
6840	SVIL	0.00	0.00	0.00	NaN
6843	VAMP1	0.02	0.00	0.15	0.07
6844	VAMP2	0.00	0.00	0.00	NaN
6847	SYCP1	0.08	0.00	0.10	-0.06
6850	SYK	0.00	0.00	0.00	NaN
6854	SYN2	0.00	0.00	0.05	0.15
6857	SYT1	0.00	0.00	0.00	NaN
6861	SYT5	0.02	0.00	0.05	0.12
6862	T	0.02	0.00	0.05	-0.06
6863	TAC1	0.00	0.14	0.05	-0.04
6865	TACR2	0.00	0.00	0.00	NaN
6866	TAC3	0.00	0.00	0.00	NaN
6867	TACC1	0.12	0.14	0.15	0.41
6868	ADAM17	0.00	0.00	0.00	NaN
6869	TACR1	0.00	0.00	0.05	-0.07
6870	TACR3	0.08	0.00	0.00	-0.04
6871	TADA2L	0.06	0.00	0.00	0.04
6874	TAF4	0.08	0.00	0.05	0.25
6875	TAF4B	0.00	0.00	0.10	0.43
6876	TAGLN	0.02	0.00	0.00	0.05
6877	TAF5	0.02	0.00	0.00	-0.11
6878	TAF6	0.02	0.00	0.00	0.18
6879	TAF7	0.00	0.00	0.00	NaN
6880	TAF9	0.06	0.00	0.00	-0.39
6881	TAF10	0.00	0.00	0.00	NaN
6883	TAF12	0.04	0.00	0.00	-0.26
6884	TAF13	0.06	0.00	0.20	-0.17
6885	MAP3K7	0.08	0.00	0.00	-0.05
6886	TAL1	0.02	0.00	0.05	-0.07
6888	TALDO1	0.02	0.00	0.05	0.04
6890	TAP1	0.02	0.00	0.05	0.00

6891	TAP2	0.02	0.00	0.05	-0.05
6894	TARBP1	0.00	0.00	0.00	NaN
6895	TARBP2	0.00	0.00	0.00	NaN
6897	TARS	0.00	0.00	0.10	0.37
6898	TAT	0.00	0.00	0.05	-0.02
6899	TBX1	0.06	0.14	0.15	0.09
6900	CNTN2	0.02	0.00	0.05	-0.11
6902	TBCA	0.00	0.00	0.00	NaN
6903	TBCC	0.06	0.00	0.05	0.06
6904	TBCD	0.00	0.00	0.00	NaN
6905	TBCE	0.02	0.00	0.00	0.06
6908	TBP	0.04	0.00	0.05	-0.03
6909	TBX2	0.10	0.00	0.05	0.42
6910	TBX5	0.02	0.00	0.00	0.08
6911	TBX6	0.02	0.00	0.00	-0.06
6915	TBXA2R	0.02	0.00	0.15	0.05
6916	TBXAS1	0.00	0.00	0.05	-0.10
6917	TCEA1	0.00	0.00	0.00	NaN
6919	TCEA2	0.08	0.00	0.05	0.16
6921	TCEB1	0.06	0.00	0.00	0.23
6923	TCEB2	0.02	0.00	0.00	0.17
6924	TCEB3	0.02	0.00	0.05	0.06
6925	TCF4	0.00	0.00	0.05	-0.07
6926	TBX3	0.02	0.00	0.00	0.18
6929	TCF3	0.02	0.00	0.05	0.23
6932	TCF7	0.00	0.00	0.00	NaN
6934	TCF7L2	0.00	0.14	0.05	0.18
6936	C2orf3	0.00	0.00	0.00	NaN
6938	TCF12	0.00	0.00	0.00	NaN
6939	TCF15	0.00	0.00	0.00	NaN
6940	ZNF354A	0.08	0.43	0.10	-0.10
6942	TCF20	0.00	0.14	0.05	0.01
6943	TCF21	0.00	0.00	0.00	NaN
6947	TCN1	0.00	0.00	0.00	NaN
6948	TCN2	0.00	0.29	0.05	-0.13
6949	TCOF1	0.00	0.00	0.00	NaN
6950	TCP1	0.04	0.00	0.05	0.05
6953	TCP10	0.02	0.00	0.05	-0.06
6954	TCP11	0.00	0.00	0.00	NaN
6988	TCTA	0.06	0.00	0.05	0.03
6992	PPP1R11	0.04	0.14	0.05	0.15
6996	TDG	0.00	0.00	0.00	NaN
6997	TDGF1	0.02	0.14	0.15	0.01
6999	TDO2	0.00	0.14	0.00	-0.09
7001	PRDX2	0.00	0.14	0.05	-0.08
7003	TEAD1	0.00	0.00	0.00	NaN
7004	TEAD4	0.02	0.00	0.00	0.14

7005	TEAD3	0.04	0.00	0.00	-0.03
7006	TEC	0.00	0.00	0.00	NaN
7007	TECTA	0.00	0.00	0.00	NaN
7008	TEF	0.00	0.14	0.00	0.04
7010	TEK	0.02	0.00	0.00	-0.03
7011	TEP1	0.02	0.00	0.00	-0.08
7013	TERF1	0.04	0.14	0.00	0.15
7014	TERF2	0.00	0.00	0.00	NaN
7015	TERT	0.00	0.00	0.00	NaN
7016	TESK1	0.00	0.00	0.00	NaN
7018	TF	0.00	0.00	0.10	-0.05
7019	TFAM	0.02	0.00	0.05	0.14
7020	TFAP2A	0.04	0.00	0.00	-0.02
7021	TFAP2B	0.00	0.00	0.00	NaN
7022	TFAP2C	0.06	0.00	0.00	0.22
7023	TFAP4	0.00	0.00	0.00	NaN
7024	TFCP2	0.02	0.00	0.00	0.16
7025	NR2F1	0.00	0.00	0.00	NaN
7026	NR2F2	0.02	0.00	0.05	-0.14
7029	TFDP2	0.02	0.00	0.00	-0.13
7031	TFF1	0.00	0.00	0.00	NaN
7032	TFF2	0.00	0.00	0.00	NaN
7033	TFF3	0.00	0.00	0.00	NaN
7035	TFPI	0.00	0.00	0.05	0.01
7036	TFR2	0.00	0.00	0.00	NaN
7037	TFRC	0.02	0.00	0.00	0.20
7038	TG	0.12	0.00	0.00	-0.04
7039	TGFA	0.00	0.14	0.00	0.00
7040	TGFB1	0.00	0.00	0.05	0.60
7042	TGFB2	0.02	0.00	0.00	0.22
7043	TGFB3	0.00	0.00	0.00	NaN
7045	TGFBI	0.02	0.00	0.00	0.01
7046	TGFBR1	0.04	0.00	0.00	0.08
7047	TGM4	0.04	0.00	0.10	0.32
7048	TGFBR2	0.02	0.00	0.05	-0.01
7049	TGFBR3	0.00	0.00	0.00	NaN
7051	TGM1	0.02	0.14	0.10	0.02
7052	TGM2	0.02	0.00	0.00	0.39
7053	TGM3	0.00	0.00	0.00	NaN
7054	TH	0.02	0.00	0.05	0.10
7056	THBD	0.02	0.00	0.00	-0.04
7057	THBS1	0.02	0.00	0.00	0.38
7058	THBS2	0.02	0.00	0.05	0.06
7059	THBS3	0.04	0.00	0.15	-0.02
7060	THBS4	0.00	0.00	0.00	NaN
7064	THOP1	0.00	0.00	0.00	NaN
7066	THPO	0.02	0.14	0.00	0.03

7067	THRA	0.02	0.86	0.05	0.40
7068	THRB	0.02	0.00	0.05	0.24
7070	THY1	0.00	0.00	0.05	-0.07
7072	TIA1	0.00	0.14	0.00	0.14
7073	TIAL1	0.04	0.00	0.10	0.14
7074	TIAM1	0.00	0.00	0.00	NaN
7077	TIMP2	0.02	0.14	0.00	-0.04
7078	TIMP3	0.00	0.29	0.05	-0.13
7079	TIMP4	0.00	0.00	0.05	0.04
7082	TJP1	0.00	0.00	0.00	NaN
7083	TK1	0.02	0.14	0.05	-0.11
7084	TK2	0.02	0.00	0.00	0.23
7086	TKT	0.02	0.00	0.00	-0.04
7087	ICAM5	0.02	0.00	0.15	0.27
7088	TLE1	0.00	0.00	0.00	NaN
7089	TLE2	0.00	0.00	0.00	NaN
7090	TLE3	0.00	0.00	0.00	NaN
7091	TLE4	0.00	0.00	0.00	NaN
7092	TLL1	0.02	0.00	0.00	-0.02
7093	TLL2	0.00	0.00	0.00	NaN
7096	TLR1	0.04	0.00	0.10	0.09
7097	TLR2	0.02	0.14	0.00	0.00
7098	TLR3	0.00	0.00	0.00	NaN
7099	TLR4	0.02	0.00	0.00	-0.07
7100	TLR5	0.02	0.00	0.05	-0.09
7101	NR2E1	0.02	0.00	0.10	0.20
7104	TM4SF4	0.00	0.00	0.00	NaN
7108	TM7SF2	0.02	0.00	0.00	-0.04
7110	TMF1	0.02	0.00	0.00	-0.15
7112	TMPO	0.04	0.14	0.05	0.00
7113	TMPRSS2	0.00	0.00	0.00	NaN
7122	CLDN5	0.06	0.14	0.15	-0.09
7124	TNF	0.02	0.00	0.05	-0.05
7125	TNNC2	0.02	0.00	0.00	0.18
7126	TNFAIP1	0.10	0.00	0.05	0.12
7127	TNFAIP2	0.00	0.00	0.05	0.39
7128	TNFAIP3	0.02	0.00	0.05	-0.08
7130	TNFAIP6	0.00	0.00	0.00	NaN
7132	TNFRSF1A	0.02	0.00	0.15	0.05
7133	TNFRSF1B	0.00	0.00	0.00	NaN
7134	TNNC1	0.00	0.00	0.10	-0.11
7135	TNNI1	0.00	0.00	0.05	-0.09
7136	TNNI2	0.02	0.00	0.05	0.10
7137	TNNI3	0.02	0.00	0.05	0.07
7139	TNNT2	0.00	0.00	0.05	0.26
7140	TNNT3	0.02	0.00	0.05	0.15
7141	TNP1	0.02	0.00	0.00	-0.01

7142	TNP2	0.02	0.00	0.00	0.02
7143	TNR	0.02	0.00	0.10	-0.09
7148	TNXB	0.02	0.00	0.05	0.16
7150	TOP1	0.04	0.00	0.00	0.38
7153	TOP2A	0.02	0.86	0.05	0.22
7155	TOP2B	0.02	0.00	0.05	0.06
7156	TOP3A	0.02	0.00	0.00	0.18
7157	TP53	0.00	0.00	0.00	NaN
7158	TP53BP1	0.04	0.00	0.00	-0.02
7159	TP53BP2	0.02	0.00	0.05	0.03
7162	TPBG	0.10	0.00	0.20	-0.10
7163	TPD52	0.04	0.00	0.00	0.46
7164	TPD52L1	0.00	0.00	0.10	0.03
7165	TPD52L2	0.08	0.00	0.05	0.26
7167	TPI1	0.02	0.00	0.15	0.27
7168	TPM1	0.00	0.00	0.00	NaN
7169	TPM2	0.00	0.00	0.00	NaN
7171	TPM4	0.04	0.00	0.00	-0.07
7172	TPMT	0.04	0.00	0.10	0.26
7173	TPO	0.20	0.14	0.35	-0.09
7174	TPP2	0.02	0.00	0.00	0.24
7175	TPR	0.00	0.00	0.05	-0.04
7178	TPT1	0.00	0.00	0.00	NaN
7181	NR2C1	0.00	0.00	0.00	NaN
7182	NR2C2	0.00	0.00	0.05	0.05
7185	TRAF1	0.00	0.00	0.00	NaN
7187	TRAF3	0.00	0.00	0.05	0.06
7188	TRAF5	0.02	0.00	0.15	-0.12
7189	TRAF6	0.02	0.00	0.00	-0.04
7200	TRH	0.02	0.00	0.00	-0.01
7201	TRHR	0.12	0.00	0.25	-0.08
7203	CCT3	0.04	0.14	0.05	0.02
7204	TRIO	0.00	0.00	0.00	NaN
7205	TRIP6	0.00	0.00	0.00	NaN
7220	TRPC1	0.02	0.00	0.00	-0.02
7221	TRPC2	0.00	0.00	0.05	-0.04
7222	TRPC3	0.00	0.14	0.05	0.09
7223	TRPC4	0.02	0.00	0.00	0.23
7225	TRPC6	0.00	0.00	0.00	NaN
7226	TRPM2	0.02	0.00	0.00	-0.03
7227	TRPS1	0.08	0.14	0.00	-0.02
7247	TSN	0.00	0.00	0.10	0.00
7248	TSC1	0.04	0.00	0.00	-0.06
7251	TSG101	0.00	0.00	0.05	-0.15
7252	TSHB	0.08	0.00	0.10	0.14
7253	TSHR	0.02	0.00	0.00	0.29
7257	TSNAX	0.00	0.00	0.00	NaN

7260	TSSC1	0.20	0.14	0.35	-0.17
7263	TST	0.00	0.14	0.00	0.00
7264	TSTA3	0.10	0.00	0.15	0.28
7265	TTC1	0.00	0.00	0.00	NaN
7266	DNAJC7	0.02	0.86	0.05	0.21
7267	TTC3	0.00	0.00	0.00	NaN
7268	TTC4	0.02	0.00	0.00	0.43
7270	TTF1	0.04	0.00	0.00	-0.06
7272	TTK	0.10	0.00	0.20	0.23
7273	TTN	0.02	0.00	0.00	-0.04
7274	TTPA	0.02	0.00	0.00	0.05
7275	TUB	0.00	0.00	0.00	NaN
7276	TTR	0.02	0.00	0.10	-0.10
7280	TUBB	0.04	0.14	0.05	0.17
7283	TUBG1	0.02	0.86	0.05	0.05
7284	TUFM	0.06	0.14	0.00	-0.03
7286	TUFT1	0.02	0.00	0.10	-0.44
7287	TULP1	0.04	0.00	0.00	-0.03
7288	TULP2	0.06	0.00	0.05	0.11
7289	TULP3	0.08	0.14	0.10	0.07
7290	HIRA	0.06	0.14	0.15	0.12
7292	TNFSF4	0.04	0.00	0.05	-0.07
7294	TXK	0.00	0.00	0.00	NaN
7295	TXN	0.02	0.00	0.05	0.17
7296	TXNRD1	0.00	0.00	0.00	NaN
7297	TYK2	0.02	0.00	0.15	0.24
7298	TYMS	0.02	0.00	0.05	0.08
7299	TYR	0.00	0.00	0.00	NaN
7301	TYRO3	0.00	0.00	0.00	NaN
7305	TYROBP	0.00	0.00	0.00	NaN
7306	TYRP1	0.00	0.00	0.00	NaN
7307	U2AF1	0.00	0.00	0.00	NaN
7311	UBA52	0.02	0.00	0.00	-0.02
7314	UBB	0.00	0.00	0.00	NaN
7316	UBC	0.02	0.00	0.10	-0.04
7320	UBE2B	0.00	0.00	0.00	NaN
7321	UBE2D1	0.02	0.00	0.05	-0.03
7322	UBE2D2	0.00	0.00	0.00	NaN
7323	UBE2D3	0.08	0.00	0.00	0.28
7324	UBE2E1	0.02	0.00	0.05	-0.08
7326	UBE2G1	0.04	0.00	0.10	0.14
7327	UBE2G2	0.02	0.00	0.00	-0.13
7328	UBE2H	0.00	0.00	0.00	NaN
7329	UBE2I	0.18	0.00	0.10	0.04
7332	UBE2L3	0.06	0.14	0.15	0.16
7334	UBE2N	0.00	0.00	0.00	NaN
7336	UBE2V2	0.04	0.00	0.00	-0.05

7337	UBE3A	0.02	0.00	0.05	0.09
7342	UBP1	0.06	0.00	0.05	-0.09
7343	UBTF	0.00	0.00	0.00	NaN
7345	UCHL1	0.00	0.00	0.05	-0.07
7347	UCHL3	0.00	0.00	0.00	NaN
7348	UPK1B	0.00	0.00	0.00	NaN
7349	UCN	0.00	0.00	0.00	NaN
7350	UCP1	0.00	0.00	0.05	-0.02
7351	UCP2	0.02	0.14	0.00	0.32
7352	UCP3	0.02	0.14	0.00	0.10
7353	UFD1L	0.06	0.14	0.15	0.11
7356	SCGB1A1	0.00	0.00	0.05	-0.03
7357	UGCG	0.02	0.00	0.00	-0.01
7358	UGDH	0.04	0.00	0.10	-0.13
7360	UGP2	0.00	0.00	0.00	NaN
7363	UGT2B4	0.00	0.14	0.00	0.10
7365	UGT2B10	0.00	0.14	0.00	-0.05
7366	UGT2B15	0.00	0.14	0.00	0.07
7367	UGT2B17	0.00	0.14	0.00	0.03
7368	UGT8	0.02	0.00	0.05	0.00
7369	UMOD	0.00	0.00	0.00	NaN
7372	UMPS	0.00	0.00	0.10	0.19
7373	COL14A1	0.10	0.00	0.15	0.01
7374	UNG	0.02	0.00	0.00	0.18
7375	USP4	0.00	0.00	0.05	0.27
7376	NR1H2	0.00	0.00	0.00	NaN
7379	UPK2	0.00	0.00	0.05	0.01
7381	UQCRB	0.08	0.00	0.05	0.30
7384	UQCRC1	0.00	0.00	0.05	0.28
7385	UQCRC2	0.02	0.14	0.00	-0.11
7386	UQCRFS1	0.00	0.14	0.05	0.36
7388	UQCRH	0.02	0.00	0.05	0.22
7389	UROD	0.02	0.00	0.05	0.38
7390	UROS	0.02	0.00	0.00	0.15
7392	USF2	0.00	0.00	0.00	NaN
7398	USP1	0.04	0.00	0.00	0.19
7399	USH2A	0.00	0.00	0.00	NaN
7402	UTRN	0.02	0.00	0.05	0.03
7405	UVRAG	0.00	0.00	0.00	NaN
7407	VARS2	0.04	0.14	0.05	0.09
7408	VASP	0.00	0.00	0.05	0.22
7409	VAV1	0.02	0.00	0.15	-0.04
7410	VAV2	0.04	0.00	0.00	-0.07
7412	VCAM1	0.02	0.00	0.00	-0.13
7414	VCL	0.02	0.00	0.00	0.13
7415	VCP	0.00	0.00	0.00	NaN
7416	VDAC1	0.00	0.00	0.00	NaN

7419	VDAC3	0.04	0.14	0.05	0.27
7421	VDR	0.04	0.00	0.05	0.17
7423	VEGFB	0.02	0.00	0.00	0.07
7424	VEGFC	0.00	0.14	0.00	0.04
7425	VGF	0.00	0.14	0.00	0.02
7428	VHL	0.02	0.00	0.05	0.01
7429	VIL1	0.04	0.00	0.00	-0.02
7431	VIM	0.02	0.14	0.05	0.00
7432	VIP	0.02	0.00	0.05	0.01
7433	VIPR1	0.00	0.00	0.05	0.41
7436	VLDLR	0.04	0.00	0.10	0.12
7441	VPREB1	0.06	0.14	0.15	0.13
7442	TRPV1	0.04	0.00	0.10	-0.16
7443	VRK1	0.00	0.00	0.05	0.30
7444	VRK2	0.00	0.00	0.05	0.15
7447	VSNL1	0.00	0.00	0.05	-0.05
7448	VTN	0.10	0.00	0.05	-0.04
7450	VWF	0.02	0.00	0.15	-0.15
7453	WARS	0.04	0.00	0.00	-0.01
7464	CORO2A	0.04	0.00	0.00	-0.08
7465	WEE1	0.00	0.00	0.00	NaN
7466	WFS1	0.00	0.00	0.00	NaN
7468	WHSC1	0.00	0.29	0.00	0.30
7469	WHSC2	0.00	0.29	0.00	0.30
7471	WNT1	0.02	0.00	0.00	0.04
7472	WNT2	0.00	0.00	0.15	-0.16
7473	WNT3	0.00	0.00	0.00	NaN
7474	WNT5A	0.02	0.00	0.00	0.09
7475	WNT6	0.04	0.00	0.00	0.06
7476	WNT7A	0.00	0.00	0.05	0.28
7477	WNT7B	0.02	0.14	0.05	-0.19
7479	WNT8B	0.00	0.00	0.05	-0.03
7480	WNT10B	0.02	0.00	0.00	-0.08
7481	WNT11	0.00	0.00	0.00	NaN
7482	WNT2B	0.02	0.00	0.05	0.04
7485	WRB	0.00	0.00	0.00	NaN
7486	WRN	0.04	0.00	0.00	-0.07
7490	WT1	0.00	0.00	0.00	NaN
7494	XBP1	0.00	0.14	0.05	-0.16
7498	XDH	0.00	0.00	0.00	NaN
7507	XPA	0.04	0.00	0.00	-0.05
7508	XPC	0.00	0.00	0.05	-0.07
7511	XPNPEP1	0.00	0.14	0.00	-0.04
7514	XPO1	0.00	0.00	0.00	NaN
7515	XRCC1	0.00	0.00	0.00	NaN
7516	XRCC2	0.02	0.00	0.00	-0.01
7518	XRCC4	0.02	0.00	0.00	0.12

7520	XRCC5	0.02	0.00	0.00	0.10
7525	YES1	0.00	0.00	0.00	NaN
7528	YY1	0.04	0.00	0.00	-0.18
7529	YWHAB	0.02	0.00	0.00	0.50
7531	YWHAE	0.04	0.00	0.05	0.13
7533	YWHAH	0.00	0.29	0.05	0.08
7534	YWHAZ	0.12	0.00	0.25	0.34
7535	ZAP70	0.00	0.00	0.10	-0.09
7536	SF1	0.02	0.00	0.00	0.11
7538	ZFP36	0.06	0.00	0.05	-0.13
7539	ZFP37	0.02	0.00	0.00	0.06
7541	ZFP161	0.02	0.00	0.00	0.12
7542	ZFPL1	0.02	0.00	0.00	0.15
7545	ZIC1	0.04	0.00	0.00	-0.08
7553	ZNF7	0.10	0.00	0.15	0.31
7554	ZNF8	0.00	0.00	0.00	NaN
7561	ZNF14	0.12	0.00	0.15	-0.03
7564	ZNF16	0.10	0.00	0.15	0.25
7568	ZNF20	0.06	0.14	0.00	0.02
7570	ZNF22	0.02	0.00	0.10	0.07
7571	ZNF23	0.00	0.00	0.05	-0.14
7572	ZNF24	0.04	0.00	0.05	0.26
7580	ZNF32	0.00	0.00	0.00	NaN
7584	ZNF35	0.04	0.00	0.10	-0.04
7587	ZNF37A	0.04	0.29	0.25	0.09
7594	ZNF43	0.12	0.00	0.15	-0.06
7595	ZNF44	0.06	0.14	0.00	-0.04
7596	ZNF45	0.00	0.00	0.00	NaN
7625	ZNF74	0.06	0.14	0.15	0.28
7629	ZNF76	0.04	0.00	0.00	-0.11
7633	ZNF79	0.02	0.00	0.00	0.04
7634	ZNF80	0.02	0.00	0.00	-0.10
7638	ZNF221	0.00	0.00	0.00	NaN
7639	ZNF85	0.12	0.00	0.15	0.03
7644	ZNF91	0.12	0.00	0.15	-0.06
7670	ZNF117	0.00	0.00	0.00	NaN
7673	ZNF222	0.00	0.00	0.00	NaN
7681	MKRN3	0.14	0.14	0.25	-0.15
7690	ZNF131	0.18	0.00	0.30	0.45
7691	ZNF132	0.00	0.00	0.00	NaN
7692	ZNF133	0.02	0.00	0.00	0.00
7693	ZNF134	0.00	0.00	0.00	NaN
7695	ZNF136	0.06	0.14	0.00	0.08
7696	ZNF137	0.02	0.00	0.05	-0.10
7701	ZNF142	0.04	0.00	0.00	0.21
7702	ZNF143	0.00	0.00	0.00	NaN
7705	ZNF146	0.02	0.00	0.05	0.10

7707	ZNF148	0.00	0.00	0.10	0.19
7710	ZNF154	0.00	0.00	0.00	NaN
7711	ZNF155	0.00	0.00	0.00	NaN
7718	ZNF165	0.04	0.14	0.05	-0.09
7726	TRIM26	0.04	0.14	0.05	0.08
7727	ZNF174	0.02	0.00	0.00	0.09
7728	ZNF175	0.02	0.00	0.05	0.01
7730	ZNF177	0.02	0.00	0.15	-0.08
7733	ZNF180	0.00	0.00	0.00	NaN
7738	ZNF184	0.00	0.14	0.00	-0.05
7741	ZNF187	0.04	0.14	0.05	-0.16
7743	ZNF189	0.04	0.00	0.00	-0.19
7745	ZNF192	0.04	0.14	0.05	0.07
7746	ZNF193	0.04	0.14	0.05	-0.05
7748	ZNF195	0.02	0.00	0.05	0.05
7752	ZNF200	0.02	0.00	0.00	0.06
7753	ZNF202	0.02	0.00	0.00	0.04
7755	ZNF205	0.02	0.00	0.00	0.00
7756	ZNF207	0.04	0.00	0.05	0.23
7757	ZNF208	0.12	0.00	0.15	0.03
7761	ZNF214	0.00	0.00	0.00	NaN
7762	ZNF215	0.00	0.00	0.00	NaN
7764	ZNF217	0.12	0.14	0.00	0.33
7766	ZNF223	0.00	0.00	0.00	NaN
7768	ZNF225	0.00	0.00	0.00	NaN
7769	ZNF226	0.00	0.00	0.00	NaN
7773	ZNF230	0.00	0.00	0.00	NaN
7775	ZNF232	0.04	0.00	0.10	0.06
7776	ZNF236	0.00	0.00	0.05	0.10
7781	SLC30A3	0.00	0.00	0.00	NaN
7782	SLC30A4	0.02	0.00	0.05	-0.05
7783	ZP2	0.02	0.14	0.00	-0.05
7786	MAP3K12	0.00	0.00	0.00	NaN
7791	ZYX	0.00	0.14	0.05	0.02
7799	PRDM2	0.00	0.00	0.00	NaN
7803	PTP4A1	0.12	0.00	0.05	0.02
7804	LRP8	0.02	0.00	0.00	0.03
7805	LAPTM5	0.00	0.14	0.00	0.10
7813	EVI5	0.00	0.00	0.00	NaN
7818	DAP3	0.04	0.00	0.15	0.19
7827	NPHS2	0.04	0.00	0.10	-0.07
7832	BTG2	0.00	0.00	0.00	NaN
7840	ALMS1	0.00	0.00	0.05	-0.02
7849	PAX8	0.00	0.00	0.10	0.16
7850	IL1R2	0.02	0.00	0.10	0.07
7852	CXCR4	0.00	0.00	0.00	NaN
7855	FZD5	0.02	0.00	0.05	-0.05

7857	SCG2	0.00	0.00	0.00	NaN
7862	BRPF1	0.00	0.00	0.10	0.19
7866	IFRD2	0.00	0.00	0.10	0.08
7867	MAPKAPK3	0.00	0.00	0.10	0.02
7869	SEMA3B	0.00	0.00	0.05	-0.01
7873	ARMET	0.00	0.00	0.10	-0.18
7874	USP7	0.00	0.00	0.00	NaN
7881	KCNAB1	0.02	0.00	0.10	0.05
7884	SLBP	0.02	0.00	0.00	-0.10
7913	DEK	0.04	0.00	0.10	0.01
7915	ALDH5A1	0.00	0.00	0.00	NaN
7916	BAT2	0.02	0.00	0.05	0.18
7917	BAT3	0.02	0.00	0.05	-0.03
7919	BAT1	0.04	0.14	0.05	0.02
7936	RDBP	0.02	0.00	0.05	0.26
7941	PLA2G7	0.04	0.14	0.20	0.20
7957	EPM2A	0.02	0.00	0.05	0.02
7965	JTV1	0.02	0.00	0.00	0.06
7975	MAFK	0.04	0.00	0.05	-0.08
7976	FZD3	0.00	0.00	0.00	NaN
7978	MTERF	0.00	0.00	0.00	NaN
7980	TFPI2	0.02	0.00	0.00	-0.04
7982	ST7	0.00	0.00	0.00	NaN
7984	ARHGEF5	0.02	0.00	0.05	0.08
7988	ZNF212	0.06	0.00	0.15	0.06
8000	PSCA	0.10	0.00	0.15	0.27
8001	GLRA3	0.00	0.00	0.00	NaN
8013	NR4A3	0.04	0.00	0.00	0.12
8019	BRD3	0.04	0.00	0.00	-0.12
8021	NUP214	0.04	0.00	0.00	-0.15
8027	STAM	0.02	0.14	0.05	0.14
8028	MLLT10	0.00	0.00	0.00	NaN
8029	CUBN	0.02	0.14	0.05	-0.06
8031	NCOA4	0.02	0.00	0.05	0.15
8034	SLC25A16	0.04	0.14	0.20	0.02
8036	SHOC2	0.00	0.14	0.00	-0.05
8038	ADAM12	0.02	0.00	0.00	0.22
8048	CSRP3	0.00	0.00	0.05	-0.22
8050	PDX1	0.00	0.00	0.05	0.03
8061	FOSL1	0.04	0.00	0.05	0.12
8065	CUL5	0.04	0.00	0.00	0.06
8073	PTP4A2	0.00	0.14	0.00	0.02
8074	FGF23	0.02	0.00	0.00	-0.02
8078	USP5	0.02	0.00	0.15	0.13
8079	MLF2	0.02	0.00	0.15	0.09
8082	SSPN	0.00	0.00	0.00	NaN
8085	MLL2	0.00	0.00	0.00	NaN

8086	AAAS	0.00	0.00	0.00	NaN
8087	FXR1	0.00	0.00	0.10	0.37
8099	CDK2AP1	0.02	0.00	0.10	0.00
8106	PABPN1	0.02	0.14	0.10	-0.01
8111	GPR68	0.02	0.00	0.00	-0.07
8115	TCL1A	0.00	0.00	0.05	-0.03
8120	AP3B2	0.02	0.00	0.00	-0.08
8125	ANP32A	0.00	0.00	0.15	-0.01
8139	GAN	0.02	0.00	0.00	-0.02
8140	SLC7A5	0.00	0.00	0.10	0.13
8148	TAF15	0.06	0.00	0.00	0.31
8161	COIL	0.04	0.00	0.05	0.42
8165	AKAP1	0.04	0.00	0.05	0.33
8170	SLC14A2	0.02	0.00	0.00	-0.10
8174	MADCAM1	0.06	0.00	0.15	0.18
8175	SF3A2	0.02	0.00	0.05	0.15
8178	ELL	0.02	0.00	0.00	0.07
8187	ZNF239	0.02	0.00	0.05	-0.07
8190	MIA	0.00	0.00	0.05	0.03
8192	CLPP	0.02	0.00	0.15	-0.05
8195	MKKS	0.00	0.00	0.05	-0.15
8200	GDF5	0.06	0.14	0.05	0.14
8202	NCOA3	0.02	0.00	0.00	0.24
8204	NRIP1	0.00	0.00	0.10	-0.06
8208	CHAF1B	0.00	0.00	0.05	0.10
8209	C21orf33	0.02	0.00	0.00	0.10
8214	DGCR6	0.02	0.14	0.20	0.09
8216	LZTR1	0.06	0.14	0.15	0.21
8218	CLTCL1	0.02	0.14	0.20	0.18
8224	SYN3	0.00	0.29	0.05	0.02
8288	EPX	0.04	0.00	0.05	-0.05
8291	DYSF	0.00	0.00	0.05	-0.10
8292	COLQ	0.00	0.00	0.05	-0.08
8295	TRRAP	0.02	0.00	0.00	0.17
8301	PICALM	0.00	0.00	0.05	0.12
8302	KLRC4	0.02	0.00	0.15	0.01
8303	SNN	0.00	0.00	0.00	NaN
8309	ACOX2	0.02	0.00	0.00	-0.04
8310	ACOX3	0.02	0.00	0.00	0.17
8312	AXIN1	0.18	0.00	0.10	-0.16
8314	BAP1	0.00	0.00	0.10	0.03
8315	BRAP	0.00	0.00	0.00	NaN
8318	CDC45L	0.06	0.14	0.15	0.29
8321	FZD1	0.00	0.00	0.00	NaN
8322	FZD4	0.02	0.00	0.00	0.10
8323	FZD6	0.12	0.00	0.25	0.38
8324	FZD7	0.00	0.00	0.00	NaN

8326	FZD9	0.00	0.00	0.00	NaN
8328	GFI1B	0.04	0.00	0.00	0.06
8372	HYAL3	0.00	0.00	0.10	-0.08
8379	MAD1L1	0.04	0.00	0.05	0.00
8382	NME5	0.02	0.00	0.00	-0.11
8383	OR1A1	0.04	0.00	0.10	-0.05
8385	OR1D4	0.04	0.00	0.10	-0.03
8387	OR1E1	0.04	0.00	0.10	0.06
8388	OR1E2	0.04	0.00	0.10	0.07
8390	OR1G1	0.04	0.00	0.10	-0.18
8392	OR3A3	0.04	0.00	0.10	-0.06
8394	PIP5K1A	0.02	0.00	0.10	0.22
8395	PIP5K1B	0.00	0.00	0.00	NaN
8398	PLA2G6	0.00	0.14	0.00	0.11
8399	PLA2G10	0.02	0.00	0.05	0.10
8402	SLC25A11	0.04	0.00	0.10	0.13
8403	SOX14	0.00	0.00	0.00	NaN
8404	SPARCL1	0.02	0.00	0.00	-0.14
8405	SPOP	0.04	0.00	0.05	0.05
8408	ULK1	0.08	0.14	0.05	-0.04
8411	EEA1	0.00	0.00	0.00	NaN
8412	BCAR3	0.00	0.00	0.00	NaN
8416	ANXA9	0.02	0.00	0.10	-0.05
8417	STX7	0.02	0.00	0.00	-0.05
8418	CMAH	0.02	0.00	0.05	0.00
8419	BFSP2	0.00	0.00	0.10	0.09
8424	BBOX1	0.00	0.00	0.00	NaN
8425	LTBP4	0.00	0.00	0.05	0.07
8427	ZNF282	0.06	0.00	0.15	0.11
8428	STK24	0.00	0.00	0.05	0.18
8431	NR0B2	0.06	0.00	0.05	0.05
8433	UTF1	0.00	0.00	0.00	NaN
8434	RECK	0.02	0.00	0.00	0.10
8435	SOAT2	0.00	0.00	0.00	NaN
8436	SDPR	0.00	0.00	0.05	0.08
8437	RASAL1	0.02	0.00	0.00	-0.06
8438	RAD54L	0.02	0.00	0.05	0.15
8439	NSMAF	0.04	0.00	0.00	0.06
8440	NCK2	0.02	0.00	0.10	0.07
8443	GNPAT	0.00	0.00	0.05	0.12
8444	DYRK3	0.00	0.00	0.05	0.09
8445	DYRK2	0.08	0.14	0.00	0.38
8446	DUSP11	0.00	0.00	0.05	0.09
8448	DOC2A	0.06	0.14	0.00	0.07
8451	CUL4A	0.06	0.00	0.00	0.14
8452	CUL3	0.00	0.00	0.00	NaN
8453	CUL2	0.04	0.29	0.25	0.35

8454	CUL1	0.06	0.00	0.15	0.29
8455	ATRN	0.02	0.00	0.00	-0.11
8458	TTF2	0.08	0.00	0.10	-0.18
8459	TPST2	0.00	0.14	0.05	0.02
8460	TPST1	0.04	0.00	0.00	-0.02
8464	SUPT3H	0.04	0.14	0.20	0.09
8467	SMARCA5	0.00	0.00	0.00	NaN
8468	FKBP6	0.00	0.00	0.00	NaN
8477	GPR65	0.00	0.00	0.00	NaN
8479	HIRIP3	0.06	0.14	0.00	0.18
8480	RAE1	0.08	0.00	0.05	0.63
8482	SEMA7A	0.00	0.00	0.00	NaN
8483	CILP	0.00	0.00	0.10	0.01
8484	GALR3	0.00	0.14	0.00	0.29
8487	SIP1	0.04	0.00	0.00	0.13
8490	RGS5	0.00	0.00	0.00	NaN
8491	MAP4K3	0.02	0.00	0.05	-0.33
8492	PRSS12	0.02	0.00	0.05	0.12
8493	PPM1D	0.10	0.00	0.05	0.37
8495	PPFIBP2	0.00	0.00	0.00	NaN
8496	PPFIBP1	0.00	0.00	0.00	NaN
8498	RANBP3	0.02	0.00	0.15	0.11
8499	PPFIA2	0.02	0.00	0.00	0.00
8500	PPFIA1	0.16	0.00	0.10	0.50
8502	PKP4	0.00	0.00	0.05	0.04
8503	PIK3R3	0.02	0.00	0.05	0.16
8504	PEX3	0.02	0.00	0.05	-0.07
8505	PARG	0.02	0.00	0.05	0.08
8506	CNTNAP1	0.02	0.86	0.05	0.06
8507	ENC1	0.06	0.00	0.00	-0.17
8508	NIPSNAP1	0.00	0.14	0.00	-0.05
8509	NDST2	0.02	0.00	0.00	0.03
8513	LIPF	0.02	0.00	0.05	-0.09
8514	KCNAB2	0.22	0.29	0.15	-0.06
8515	ITGA10	0.02	0.00	0.00	0.02
8516	ITGA8	0.02	0.14	0.05	-0.09
8518	IKBKAP	0.02	0.00	0.05	0.03
8519	IFITM1	0.06	0.29	0.10	-0.06
8520	HAT1	0.00	0.00	0.00	NaN
8522	GAS7	0.02	0.00	0.00	0.07
8525	DGKZ	0.00	0.00	0.05	0.11
8526	DGKE	0.04	0.00	0.05	0.05
8527	DGKD	0.00	0.00	0.00	NaN
8528	DDO	0.02	0.00	0.10	-0.10
8529	CYP4F2	0.04	0.00	0.00	0.09
8530	CST7	0.04	0.00	0.15	-0.05
8531	CSDA	0.02	0.00	0.15	0.04

8532	CPZ	0.02	0.00	0.00	0.03
8533	COPS3	0.02	0.00	0.00	0.00
8534	CHST1	0.04	0.00	0.05	-0.01
8535	CBX4	0.00	0.14	0.00	-0.03
8536	CAMK1	0.00	0.00	0.10	-0.04
8537	BCAS1	0.14	0.00	0.05	0.23
8538	BARX2	0.02	0.00	0.00	-0.06
8539	API5	0.00	0.00	0.00	NaN
8540	AGPS	0.02	0.00	0.00	0.01
8541	PPFIA3	0.06	0.00	0.05	0.22
8542	APOL1	0.00	0.14	0.00	0.12
8543	LMO4	0.04	0.00	0.05	0.17
8545	CGGBP1	0.16	0.14	0.30	0.02
8546	AP3B1	0.00	0.00	0.00	NaN
8547	FCN3	0.04	0.00	0.00	0.06
8548	BLZF1	0.02	0.00	0.00	0.08
8550	MAPKAPK5	0.00	0.00	0.00	NaN
8554	PIAS1	0.00	0.00	0.15	0.22
8555	CDC14B	0.00	0.00	0.00	NaN
8556	CDC14A	0.02	0.00	0.00	0.17
8557	TCAP	0.02	0.86	0.05	0.39
8558	CDK10	0.00	0.00	0.10	0.04
8562	DENR	0.02	0.00	0.10	-0.04
8564	KMO	0.06	0.00	0.00	0.01
8565	YARS	0.00	0.00	0.00	NaN
8566	PDXK	0.02	0.00	0.00	-0.06
8567	MADD	0.04	0.00	0.00	0.09
8569	MKNK1	0.02	0.00	0.05	0.05
8570	KHSRP	0.02	0.00	0.15	0.06
8574	AKR7A2	0.00	0.00	0.00	NaN
8575	PRKRA	0.02	0.00	0.00	0.07
8576	STK16	0.04	0.00	0.00	0.01
8577	TMEFF1	0.04	0.00	0.00	0.11
8600	TNFSF11	0.02	0.00	0.00	0.01
8601	RGS20	0.00	0.00	0.00	NaN
8604	SLC25A12	0.00	0.00	0.00	NaN
8605	PLA2G4C	0.00	0.00	0.00	NaN
8607	RUVBL1	0.04	0.00	0.00	0.25
8609	KLF7	0.02	0.00	0.05	0.18
8611	PPAP2A	0.00	0.14	0.05	-0.17
8612	PPAP2C	0.06	0.00	0.15	0.08
8613	PPAP2B	0.02	0.00	0.00	0.20
8614	STC2	0.00	0.00	0.05	-0.04
8618	CADPS	0.04	0.00	0.00	-0.24
8620	NPFF	0.00	0.00	0.00	NaN
8621	CDC2L5	0.00	0.00	0.00	NaN
8625	RFXANK	0.12	0.00	0.15	-0.07

8626	TP63	0.00	0.00	0.00	NaN
8629	JRK	0.10	0.00	0.15	0.23
8633	UNC5C	0.04	0.00	0.05	-0.17
8634	RTCD1	0.02	0.00	0.00	-0.03
8638	OASL	0.02	0.00	0.10	0.15
8639	AOC3	0.02	0.86	0.05	-0.12
8643	PTCH2	0.02	0.00	0.05	0.16
8644	AKR1C3	0.00	0.00	0.00	NaN
8645	KCNK5	0.02	0.00	0.05	0.11
8646	CHRD	0.02	0.14	0.00	0.18
8647	ABCB11	0.00	0.00	0.00	NaN
8648	NCOA1	0.00	0.00	0.00	NaN
8650	NUMB	0.02	0.00	0.05	0.12
8654	PDE5A	0.00	0.00	0.00	NaN
8658	TNKS	0.02	0.14	0.10	0.00
8659	ALDH4A1	0.00	0.00	0.00	NaN
8660	IRS2	0.00	0.00	0.05	-0.01
8671	SLC4A4	0.02	0.00	0.05	-0.02
8672	EIF4G3	0.02	0.00	0.05	-0.05
8673	VAMP8	0.00	0.00	0.00	NaN
8674	VAMP4	0.04	0.00	0.05	0.16
8676	STX11	0.02	0.00	0.05	-0.11
8677	STX10	0.00	0.14	0.05	-0.08
8678	BECN1	0.02	0.86	0.05	-0.08
8681	PLA2G4B	0.00	0.00	0.00	NaN
8682	PEA15	0.00	0.00	0.00	NaN
8683	SFRS9	0.02	0.00	0.10	0.10
8685	MARCO	0.00	0.00	0.10	-0.01
8690	JRKL	0.00	0.00	0.00	NaN
8692	HYAL2	0.00	0.00	0.10	-0.06
8693	GALNT4	0.00	0.00	0.00	NaN
8694	DGAT1	0.10	0.00	0.15	0.41
8697	CDC23	0.02	0.00	0.00	0.09
8702	B4GALT4	0.00	0.00	0.00	NaN
8703	B4GALT3	0.00	0.00	0.00	NaN
8704	B4GALT2	0.02	0.00	0.05	0.13
8705	B3GALT4	0.02	0.00	0.05	0.30
8707	B3GALT2	0.00	0.00	0.00	NaN
8708	B3GALT1	0.00	0.00	0.00	NaN
8710	SERPINB7	0.02	0.00	0.00	0.00
8711	TNK1	0.02	0.00	0.00	-0.02
8714	ABCC3	0.10	0.00	0.10	0.02
8715	NOL4	0.04	0.00	0.10	-0.07
8717	TRADD	0.02	0.00	0.00	0.06
8720	MBTPS1	0.00	0.00	0.00	NaN
8722	CTSF	0.04	0.00	0.05	0.06
8723	SNX4	0.00	0.00	0.10	0.27

8724	SNX3	0.02	0.00	0.10	0.35
8726	EED	0.02	0.00	0.00	0.06
8727	CTNNAL1	0.02	0.00	0.05	0.17
8728	ADAM19	0.00	0.00	0.00	NaN
8729	GBF1	0.02	0.00	0.00	-0.18
8731	RNMT	0.08	0.14	0.10	0.10
8732	RNGTT	0.00	0.00	0.05	0.02
8733	GPAA1	0.10	0.00	0.15	0.35
8735	MYH13	0.04	0.00	0.00	0.12
8736	MYOM1	0.02	0.00	0.00	0.01
8738	CRADD	0.00	0.00	0.05	-0.07
8739	HRK	0.02	0.00	0.10	-0.02
8740	TNFSF14	0.02	0.00	0.15	0.05
8741	TNFSF13	0.02	0.00	0.00	0.04
8743	TNFSF10	0.06	0.00	0.00	-0.13
8744	TNFSF9	0.02	0.00	0.15	0.00
8745	ADAM23	0.00	0.00	0.00	NaN
8747	ADAM21	0.02	0.00	0.05	0.00
8748	ADAM20	0.02	0.00	0.05	-0.07
8749	ADAM18	0.12	0.14	0.15	-0.17
8754	ADAM9	0.12	0.14	0.15	0.35
8756	ADAM7	0.00	0.00	0.00	NaN
8760	CDS2	0.00	0.00	0.10	0.13
8761	PABPC4	0.02	0.00	0.05	-0.03
8763	CD164	0.02	0.00	0.10	0.25
8766	RAB11A	0.00	0.00	0.00	NaN
8767	RIPK2	0.06	0.00	0.15	0.30
8771	TNFRSF6B	0.08	0.00	0.05	0.17
8772	FADD	0.16	0.00	0.10	0.38
8773	SNAP23	0.04	0.00	0.00	0.13
8774	NAPG	0.00	0.00	0.00	NaN
8775	NAPA	0.00	0.00	0.00	NaN
8777	MPDZ	0.00	0.00	0.00	NaN
8778	SIGLEC5	0.02	0.00	0.05	0.11
8785	MATN4	0.04	0.00	0.05	0.06
8786	RGS11	0.18	0.00	0.10	-0.09
8787	RGS9	0.12	0.00	0.20	0.38
8788	DLK1	0.04	0.00	0.00	-0.03
8789	FBP2	0.00	0.00	0.00	NaN
8790	FPGT	0.04	0.14	0.00	-0.12
8792	TNFRSF11A	0.02	0.00	0.00	-0.04
8793	TNFRSF10D	0.02	0.00	0.00	0.29
8794	TNFRSF10C	0.02	0.00	0.00	0.13
8795	TNFRSF10B	0.02	0.00	0.00	0.07
8796	SCEL	0.02	0.00	0.00	-0.07
8798	DYRK4	0.02	0.00	0.00	-0.26
8799	PEX11B	0.02	0.00	0.00	-0.02

8800	PEX11A	0.00	0.00	0.00	NaN
8801	SUCLG2	0.04	0.00	0.00	0.22
8802	SUCLG1	0.00	0.00	0.00	NaN
8803	SUCLA2	0.00	0.00	0.00	NaN
8807	IL18RAP	0.02	0.00	0.10	0.14
8808	IL1RL2	0.02	0.00	0.10	0.19
8809	IL18R1	0.02	0.00	0.10	0.06
8811	GALR2	0.02	0.14	0.05	-0.10
8813	DPM1	0.10	0.00	0.00	0.17
8814	CDKL1	0.00	0.00	0.00	NaN
8817	FGF18	0.02	0.00	0.00	-0.10
8818	DPM2	0.00	0.00	0.00	NaN
8819	SAP30	0.00	0.00	0.00	NaN
8820	HESX1	0.00	0.00	0.05	-0.09
8821	INPP4B	0.00	0.00	0.00	NaN
8822	FGF17	0.00	0.00	0.05	0.16
8824	CES2	0.02	0.00	0.00	0.03
8826	IQGAP1	0.00	0.00	0.00	NaN
8828	NRP2	0.00	0.00	0.00	NaN
8829	NRP1	0.02	0.14	0.00	0.09
8832	CD84	0.00	0.00	0.00	NaN
8833	GMPS	0.02	0.00	0.10	-0.01
8836	GGH	0.02	0.00	0.00	0.06
8837	CFLAR	0.00	0.00	0.05	0.25
8838	WISP3	0.02	0.00	0.05	-0.02
8839	WISP2	0.02	0.00	0.00	-0.04
8840	WISP1	0.06	0.00	0.00	0.05
8851	CDK5R1	0.04	0.00	0.05	0.06
8854	ALDH1A2	0.00	0.00	0.00	NaN
8856	NR1I2	0.00	0.00	0.10	0.09
8857	FCGBP	0.06	0.00	0.05	-0.09
8858	PROZ	0.06	0.00	0.00	0.00
8859	STK19	0.02	0.00	0.05	0.34
8861	LDB1	0.02	0.00	0.00	-0.08
8863	PER3	0.00	0.00	0.00	NaN
8864	PER2	0.08	0.43	0.10	0.27
8867	SYNJ1	0.00	0.00	0.00	NaN
8870	IER3	0.04	0.14	0.05	0.05
8871	SYNJ2	0.04	0.00	0.05	-0.01
8874	ARHGEF7	0.00	0.00	0.00	NaN
8875	VNN2	0.02	0.00	0.00	-0.02
8876	VNN1	0.02	0.00	0.00	-0.03
8877	SPHK1	0.00	0.14	0.05	0.12
8878	SQSTM1	0.08	0.43	0.10	-0.05
8879	SGPL1	0.00	0.00	0.00	NaN
8880	FUBP1	0.06	0.00	0.00	-0.12
8882	ZNF259	0.02	0.00	0.00	-0.07

8884	SLC5A6	0.00	0.00	0.00	NaN
8886	DDX18	0.00	0.00	0.10	0.11
8887	TAX1BP1	0.00	0.00	0.05	0.04
8888	MCM3AP	0.00	0.00	0.05	0.02
8891	EIF2B3	0.02	0.00	0.05	0.23
8892	EIF2B2	0.02	0.00	0.05	0.06
8893	EIF2B5	0.00	0.00	0.00	NaN
8894	EIF2S2	0.02	0.00	0.05	-0.28
8895	CPNE3	0.06	0.00	0.15	0.06
8897	MTMR3	0.00	0.29	0.05	0.25
8898	MTMR2	0.00	0.00	0.00	NaN
8900	CCNA1	0.02	0.00	0.00	-0.06
8904	CPNE1	0.06	0.14	0.05	0.38
8906	AP1G2	0.02	0.14	0.10	-0.06
8909	P11	0.04	0.00	0.05	-0.09
8910	SGCE	0.02	0.00	0.00	0.06
8911	CACNA1I	0.00	0.14	0.00	0.32
8912	CACNA1H	0.18	0.00	0.10	0.02
8913	CACNA1G	0.10	0.00	0.10	0.05
8914	TIMELESS	0.00	0.00	0.00	NaN
8915	BCL10	0.04	0.00	0.05	0.10
8916	HERC3	0.00	0.00	0.00	NaN
8924	HERC2	0.00	0.00	0.00	NaN
8925	HERC1	0.02	0.00	0.00	0.07
8926	SNURF	0.00	0.14	0.00	-0.05
8927	BSN	0.06	0.00	0.05	0.01
8928	FOXH1	0.10	0.00	0.15	0.06
8930	MBD4	0.02	0.00	0.00	0.28
8932	MBD2	0.00	0.00	0.05	0.04
8934	RAB7L1	0.02	0.00	0.05	0.07
8936	WASF1	0.02	0.00	0.10	0.29
8938	BAIAP3	0.18	0.00	0.10	0.06
8939	FUBP3	0.04	0.00	0.00	-0.03
8940	TOP3B	0.06	0.14	0.15	0.32
8941	CDK5R2	0.04	0.00	0.00	-0.11
8942	KYNU	0.02	0.00	0.00	0.06
8943	AP3D1	0.02	0.00	0.05	0.04
8945	BTRC	0.02	0.00	0.00	0.14
8971	H1FX	0.02	0.00	0.00	0.20
8972	MGAM	0.00	0.14	0.05	-0.04
8973	CHRNA6	0.04	0.14	0.05	-0.05
8974	P4HA2	0.02	0.00	0.00	0.08
8975	USP13	0.00	0.00	0.10	0.24
8976	WASL	0.00	0.00	0.00	NaN
8985	PLOD3	0.00	0.14	0.00	0.06
8986	RPS6KA4	0.02	0.00	0.00	-0.01
8988	HSPB3	0.04	0.00	0.00	0.29

8991	SELENBP1	0.02	0.00	0.10	-0.24
8994	LIMD1	0.02	0.14	0.15	-0.20
8995	TNFSF18	0.04	0.00	0.05	-0.09
8996	NOL3	0.02	0.00	0.00	0.13
8999	CDKL2	0.00	0.00	0.00	NaN
9001	HAP1	0.02	0.86	0.05	0.17
9002	F2RL3	0.04	0.00	0.00	-0.05
9013	TAF1C	0.00	0.00	0.00	NaN
9014	TAF1B	0.00	0.00	0.00	NaN
9015	TAF1A	0.02	0.00	0.00	0.00
9019	MPZL1	0.00	0.00	0.10	0.23
9020	MAP3K14	0.00	0.00	0.05	-0.08
9023	CH25H	0.02	0.00	0.05	0.09
9025	RNF8	0.00	0.00	0.00	NaN
9027	NAT8	0.00	0.00	0.05	-0.16
9031	BAZ1B	0.00	0.00	0.00	NaN
9032	TM4SF5	0.04	0.00	0.10	0.01
9033	PKD2L1	0.00	0.00	0.05	0.12
9034	CCRL2	0.02	0.14	0.15	0.40
9037	SEMA5A	0.00	0.00	0.00	NaN
9040	UBE2M	0.00	0.00	0.00	NaN
9043	SPAG9	0.10	0.00	0.10	0.22
9044	BTAF1	0.02	0.00	0.05	0.08
9045	RPL14	0.02	0.00	0.05	-0.01
9046	DOK2	0.00	0.00	0.05	0.09
9047	SH2D2A	0.04	0.14	0.05	0.02
9048	ARTN	0.02	0.00	0.05	-0.04
9049	AIP	0.02	0.00	0.00	0.57
9050	PSTPIP2	0.02	0.00	0.00	0.03
9051	PSTPIP1	0.00	0.00	0.05	-0.07
9053	MAP7	0.00	0.00	0.10	0.04
9054	NFS1	0.06	0.14	0.05	0.36
9055	PRC1	0.02	0.00	0.10	0.25
9056	SLC7A7	0.02	0.00	0.00	0.02
9057	SLC7A6	0.02	0.00	0.00	0.05
9058	SLC13A2	0.10	0.00	0.05	0.05
9060	PAPSS2	0.00	0.00	0.00	NaN
9061	PAPSS1	0.08	0.00	0.00	-0.05
9064	MAP3K6	0.00	0.00	0.00	NaN
9070	ASH2L	0.12	0.14	0.05	0.39
9071	CLDN10	0.04	0.00	0.00	-0.13
9074	CLDN6	0.02	0.00	0.00	-0.09
9076	CLDN1	0.00	0.00	0.05	-0.06
9079	LDB2	0.00	0.00	0.00	NaN
9080	CLDN9	0.02	0.00	0.00	-0.02
9088	PKMYT1	0.02	0.00	0.00	-0.16
9090	PSIP1	0.04	0.00	0.05	0.33

9091	PIGQ	0.18	0.00	0.10	-0.02
9092	SART1	0.04	0.00	0.05	0.05
9093	DNAJA3	0.00	0.00	0.00	NaN
9094	UNC119	0.10	0.00	0.05	0.10
9095	TBX19	0.00	0.00	0.10	0.24
9097	USP14	0.02	0.00	0.05	0.16
9098	USP6	0.04	0.00	0.10	0.01
9099	USP2	0.00	0.00	0.05	0.14
9100	USP10	0.00	0.00	0.00	NaN
9101	USP8	0.00	0.00	0.00	NaN
9107	MTMR6	0.06	0.00	0.00	0.12
9108	MTMR7	0.00	0.00	0.10	0.05
9110	MTMR4	0.04	0.00	0.05	0.07
9111	NMI	0.00	0.00	0.00	NaN
9112	MTA1	0.02	0.00	0.10	-0.01
9113	LATS1	0.02	0.00	0.05	0.00
9114	ATP6V0D1	0.02	0.00	0.00	0.00
9120	SLC16A6	0.12	0.00	0.20	-0.04
9121	SLC16A5	0.02	0.14	0.00	0.57
9122	SLC16A4	0.06	0.14	0.00	-0.13
9123	SLC16A3	0.00	0.00	0.00	NaN
9124	PDLIM1	0.00	0.00	0.00	NaN
9125	RQCD1	0.04	0.00	0.00	0.01
9128	PRPF4	0.00	0.00	0.00	NaN
9132	KCNQ4	0.02	0.00	0.05	-0.07
9133	CCNB2	0.00	0.00	0.00	NaN
9134	CCNE2	0.08	0.00	0.05	0.41
9138	ARHGEF1	0.00	0.00	0.05	0.36
9139	CBFA2T2	0.00	0.00	0.00	NaN
9141	PDCD5	0.00	0.00	0.00	NaN
9143	SYNGR3	0.18	0.00	0.10	-0.01
9144	SYNGR2	0.02	0.14	0.05	-0.40
9145	SYNGR1	0.00	0.14	0.05	0.08
9146	HGS	0.00	0.00	0.00	NaN
9147	SDCCAG1	0.00	0.00	0.00	NaN
9148	NEURL	0.02	0.00	0.00	0.02
9149	DYRK1B	0.06	0.00	0.05	0.11
9150	CTDP1	0.02	0.00	0.05	0.15
9152	SLC6A5	0.02	0.00	0.00	0.01
9153	SLC28A2	0.02	0.00	0.05	0.02
9154	SLC28A1	0.00	0.00	0.00	NaN
9158	FIBP	0.04	0.00	0.05	-0.44
9159	PCSK7	0.02	0.00	0.00	-0.16
9162	DGKI	0.00	0.00	0.05	0.22
9166	EBAG9	0.08	0.14	0.00	0.13
9167	COX7A2L	0.02	0.00	0.05	-0.18
9168	TMSB10	0.00	0.00	0.00	NaN

9169	SFRS2IP	0.04	0.00	0.05	0.06
9172	MYOM2	0.00	0.00	0.00	NaN
9173	IL1RL1	0.02	0.00	0.10	0.00
9175	MAP3K13	0.02	0.14	0.00	-0.13
9177	HTR3B	0.00	0.00	0.00	NaN
9179	AP4M1	0.02	0.00	0.00	0.31
9180	OSMR	0.04	0.00	0.00	-0.03
9181	ARHGEF2	0.04	0.14	0.05	0.00
9183	ZW10	0.00	0.00	0.00	NaN
9184	BUB3	0.04	0.00	0.05	0.08
9187	SLC24A1	0.00	0.00	0.00	NaN
9188	DDX21	0.04	0.14	0.20	0.05
9191	DEDD	0.00	0.00	0.00	NaN
9194	SLC16A7	0.02	0.00	0.05	0.00
9196	KCNAB3	0.00	0.00	0.00	NaN
9200	PTPLA	0.02	0.14	0.05	-0.04
9208	LRRFIP1	0.00	0.00	0.00	NaN
9209	LRRFIP2	0.02	0.14	0.10	0.08
9211	LGI1	0.02	0.00	0.05	-0.10
9215	LARGE	0.00	0.29	0.05	0.12
9217	VAPB	0.08	0.00	0.00	0.66
9218	VAPA	0.00	0.00	0.05	0.10
9220	TIAF1	0.10	0.00	0.05	-0.10
9221	NOLC1	0.02	0.00	0.00	0.04
9227	LRAT	0.00	0.00	0.00	NaN
9228	DLGAP2	0.00	0.00	0.00	NaN
9229	DLGAP1	0.00	0.00	0.00	NaN
9230	RAB11B	0.02	0.00	0.15	0.04
9231	DLG5	0.02	0.00	0.10	0.00
9232	PTTG1	0.00	0.00	0.00	NaN
9240	PNMA1	0.02	0.00	0.05	0.02
9242	MSC	0.04	0.14	0.00	-0.09
9244	CRLF1	0.02	0.00	0.00	0.00
9245	GCNT3	0.00	0.00	0.00	NaN
9246	UBE2L6	0.00	0.00	0.00	NaN
9252	RPS6KA5	0.00	0.00	0.00	NaN
9254	CACNA2D2	0.00	0.00	0.10	-0.17
9255	SCYE1	0.08	0.00	0.00	0.17
9258	MFHAS1	0.02	0.14	0.10	0.19
9261	MAPKAPK2	0.00	0.00	0.05	0.07
9262	STK17B	0.00	0.00	0.05	0.24
9263	STK17A	0.00	0.00	0.00	NaN
9271	PIWIL1	0.02	0.00	0.10	-0.02
9274	BCL7C	0.02	0.00	0.00	-0.24
9275	BCL7B	0.00	0.00	0.00	NaN
9276	COPB2	0.00	0.00	0.05	0.04
9284	NPIP	0.02	0.00	0.05	-0.03

9289	GPR56	0.02	0.00	0.00	0.11
9293	GPR52	0.02	0.00	0.10	0.00
9295	SFRS11	0.04	0.14	0.00	0.15
9296	ATP6V1F	0.00	0.00	0.05	-0.05
9308	CD83	0.02	0.00	0.00	-0.13
9311	ACCN3	0.02	0.00	0.00	-0.01
9312	KCNB2	0.04	0.14	0.00	0.25
9313	MMP20	0.00	0.00	0.00	NaN
9314	KLF4	0.00	0.00	0.00	NaN
9317	PTER	0.02	0.14	0.05	-0.10
9319	TRIP13	0.00	0.00	0.00	NaN
9320	TRIP12	0.02	0.00	0.15	0.11
9321	TRIP11	0.00	0.00	0.05	-0.08
9322	TRIP10	0.02	0.00	0.15	0.01
9325	TRIP4	0.02	0.00	0.00	-0.26
9328	GTF3C5	0.04	0.00	0.00	-0.11
9329	GTF3C4	0.04	0.00	0.00	-0.05
9330	GTF3C3	0.00	0.00	0.05	-0.03
9331	B4GALT6	0.02	0.00	0.10	0.06
9332	CD163	0.02	0.00	0.15	0.37
9333	TGM5	0.04	0.00	0.00	-0.06
9334	B4GALT5	0.06	0.00	0.00	0.26
9337	CNOT8	0.02	0.00	0.00	0.34
9340	GLP2R	0.02	0.00	0.00	0.07
9341	VAMP3	0.00	0.00	0.00	NaN
9342	SNAP29	0.06	0.14	0.15	0.09
9348	NDST3	0.02	0.00	0.05	-0.13
9349	RPL23	0.02	0.86	0.05	0.19
9350	CER1	0.04	0.00	0.05	-0.10
9351	SLC9A3R2	0.18	0.00	0.10	0.17
9353	SLIT2	0.02	0.00	0.00	0.31
9354	UBE4A	0.00	0.00	0.00	NaN
9355	LHX2	0.06	0.00	0.00	0.39
9356	SLC22A6	0.00	0.00	0.00	NaN
9358	ITGBL1	0.00	0.00	0.00	NaN
9360	PPIG	0.00	0.00	0.00	NaN
9362	CPNE6	0.02	0.14	0.10	0.01
9364	RAB28	0.00	0.00	0.05	-0.13
9365	KL	0.00	0.00	0.05	-0.02
9368	SLC9A3R1	0.04	0.00	0.00	0.09
9369	NRXN3	0.02	0.00	0.00	0.08
9371	KIF3B	0.04	0.00	0.15	-0.03
9373	PLAA	0.02	0.00	0.00	0.05
9374	PPT2	0.02	0.00	0.05	0.02
9375	TM9SF2	0.00	0.00	0.05	0.00
9376	SLC22A8	0.00	0.00	0.00	NaN
9377	COX5A	0.00	0.00	0.05	0.07

9378	NRXN1	0.02	0.00	0.05	-0.08
9379	NRXN2	0.02	0.00	0.00	-0.08
9380	GRHPR	0.04	0.00	0.00	0.06
9381	OTOF	0.00	0.00	0.00	NaN
9388	LIPG	0.00	0.00	0.00	NaN
9391	CIAO1	0.04	0.00	0.05	0.21
9397	NMT2	0.02	0.14	0.05	0.01
9398	IGSF2	0.08	0.00	0.10	0.17
9399	STOML1	0.02	0.00	0.00	-0.02
9400	RECQL5	0.02	0.14	0.00	0.55
9401	RECQL4	0.10	0.00	0.15	0.32
9402	GRAP2	0.00	0.14	0.00	-0.08
9403	9/15/2010	0.04	0.00	0.05	0.12
9409	PEX16	0.04	0.00	0.05	-0.03
9414	TJP2	0.00	0.00	0.00	NaN
9415	FADS2	0.00	0.00	0.05	0.12
9419	CRIPT	0.00	0.00	0.00	NaN
9420	CYP7B1	0.00	0.00	0.05	-0.08
9421	HAND1	0.02	0.00	0.00	0.01
9422	ZNF264	0.00	0.00	0.05	-0.07
9423	NTN1	0.00	0.00	0.00	NaN
9425	CDYL	0.08	0.14	0.10	-0.06
9427	ECEL1	0.00	0.00	0.05	0.07
9429	ABCG2	0.02	0.00	0.00	0.00
9435	CHST2	0.00	0.14	0.00	-0.08
9444	QKI	0.02	0.00	0.05	0.05
9445	ITM2B	0.00	0.00	0.00	NaN
9447	AIM2	0.00	0.00	0.00	NaN
9448	MAP4K4	0.02	0.00	0.10	0.31
9451	EIF2AK3	0.04	0.00	0.05	0.08
9453	GGPS1	0.02	0.00	0.00	0.15
9462	RASAL2	0.04	0.00	0.10	0.07
9464	HAND2	0.00	0.00	0.00	NaN
9465	AKAP7	0.00	0.00	0.00	NaN
9467	SH3BP5	0.00	0.00	0.05	-0.06
9469	CHST3	0.04	0.00	0.05	0.07
9472	AKAP6	0.02	0.00	0.00	0.21
9475	ROCK2	0.00	0.00	0.00	NaN
9478	CABP1	0.02	0.00	0.10	0.19
9479	MAPK8IP1	0.04	0.00	0.05	-0.08
9480	ONECUT2	0.00	0.00	0.15	0.08
9482	STX8	0.02	0.00	0.00	0.32
9487	PIGL	0.00	0.00	0.00	NaN
9488	PIGB	0.00	0.00	0.00	NaN
9489	PGS1	0.02	0.14	0.00	-0.05
9491	PSMF1	0.00	0.00	0.00	NaN
9495	AKAP5	0.06	0.00	0.00	0.01

9496	TBX4	0.10	0.00	0.05	0.24
9497	SLC4A7	0.02	0.00	0.05	0.13
9498	SLC4A8	0.00	0.00	0.00	NaN
9501	RPH3AL	0.18	0.00	0.10	0.22
9508	ADAMTS3	0.02	0.00	0.05	-0.07
9509	ADAMTS2	0.08	0.43	0.10	0.31
9512	PMPCB	0.00	0.00	0.00	NaN
9513	FXR2	0.02	0.00	0.00	-0.07
9517	SPTLC2	0.00	0.00	0.00	NaN
9519	TBPL1	0.00	0.00	0.00	NaN
9520	NPEPPS	0.00	0.00	0.00	NaN
9521	EEF1E1	0.00	0.00	0.00	NaN
9522	SCAMP1	0.00	0.00	0.00	NaN
9524	GPSN2	0.00	0.00	0.05	-0.19
9526	MPDU1	0.02	0.00	0.00	-0.15
9527	GOSR1	0.10	0.00	0.05	0.06
9529	BAG5	0.00	0.00	0.05	0.03
9530	BAG4	0.08	0.00	0.00	0.67
9531	BAG3	0.04	0.00	0.10	-0.06
9532	BAG2	0.06	0.14	0.05	0.02
9534	ZNF254	0.12	0.00	0.15	-0.15
9535	GMFG	0.06	0.00	0.05	0.16
9536	PTGES	0.00	0.00	0.00	NaN
9541	CIR	0.00	0.00	0.00	NaN
9542	NRG2	0.00	0.00	0.00	NaN
9545	RAB3D	0.02	0.00	0.00	-0.12
9546	APBA3	0.02	0.00	0.15	-0.12
9550	ATP6V1G1	0.02	0.00	0.00	0.04
9551	ATP5J2	0.02	0.00	0.00	0.02
9552	SPAG7	0.04	0.00	0.10	-0.04
9553	MRPL33	0.00	0.00	0.00	NaN
9555	H2AFY	0.02	0.00	0.00	0.15
9556	C14orf2	0.02	0.00	0.10	0.05
9557	CHD1L	0.02	0.00	0.00	-0.01
9562	MINPP1	0.00	0.00	0.05	0.03
9563	H6PD	0.00	0.00	0.00	NaN
9567	GTPBP1	0.00	0.14	0.00	0.15
9569	GTF2IRD1	0.00	0.00	0.00	NaN
9570	GOSR2	0.00	0.00	0.00	NaN
9572	NR1D1	0.02	0.86	0.05	0.29
9573	GDF3	0.02	0.00	0.15	-0.15
9575	CLOCK	0.00	0.00	0.10	-0.10
9576	SPAG6	0.00	0.00	0.00	NaN
9577	BRE	0.00	0.00	0.00	NaN
9578	CDC42BPB	0.00	0.00	0.05	-0.08
9580	SOX13	0.00	0.00	0.15	-0.12
9589	WTAP	0.04	0.00	0.05	0.04

9590	AKAP12	0.02	0.00	0.05	0.05
9603	NFE2L3	0.00	0.00	0.00	NaN
9604	RNF14	0.00	0.00	0.00	NaN
9605	C16orf7	0.00	0.00	0.10	0.36
9609	RAB36	0.06	0.14	0.15	-0.07
9610	RIN1	0.04	0.00	0.05	0.17
9611	NCOR1	0.00	0.00	0.00	NaN
9612	NCOR2	0.02	0.00	0.10	0.10
9616	RNF7	0.02	0.00	0.00	0.04
9617	MTRF1	0.00	0.00	0.00	NaN
9618	TRAF4	0.10	0.00	0.05	0.28
9619	ABCG1	0.00	0.00	0.00	NaN
9620	CELSR1	0.02	0.14	0.05	0.17
9623	TCL1B	0.00	0.00	0.05	-0.10
9625	AATK	0.00	0.14	0.00	0.10
9627	SNCAIP	0.00	0.00	0.00	NaN
9628	RGS6	0.02	0.00	0.05	0.23
9630	GNA14	0.00	0.00	0.05	-0.08
9631	NUP155	0.04	0.00	0.15	0.04
9632	SEC24C	0.02	0.00	0.00	0.16
9633	MTL5	0.02	0.00	0.05	0.19
9635	CLCA2	0.04	0.00	0.05	-0.07
9637	FEZ2	0.00	0.00	0.00	NaN
9638	FEZ1	0.02	0.00	0.00	0.14
9639	ARHGEF10	0.00	0.00	0.00	NaN
9653	HS2ST1	0.04	0.00	0.05	0.13
9655	SOCS5	0.00	0.00	0.00	NaN
9659	PDE4DIP	0.02	0.00	0.00	-0.02
9663	LPIN2	0.02	0.00	0.00	0.06
9672	SDC3	0.00	0.14	0.00	-0.05
9674	KIAA0040	0.02	0.00	0.10	0.08
9675	KIAA0406	0.02	0.00	0.00	0.20
9683	N4BP1	0.43	0.29	0.15	-0.29
9692	KIAA0391	0.04	0.00	0.00	0.01
9698	PUM1	0.00	0.14	0.00	-0.03
9700	ESPL1	0.00	0.00	0.00	NaN
9703	KIAA0100	0.10	0.00	0.05	0.16
9706	ULK2	0.10	0.00	0.05	-0.08
9708	PCDHGA8	0.00	0.00	0.00	NaN
9709	HERPUD1	0.00	0.14	0.00	-0.19
9710	KIAA0355	0.00	0.00	0.00	NaN
9711	KIAA0226	0.20	0.14	0.10	0.01
9718	ECE2	0.02	0.14	0.00	-0.07
9723	SEMA3E	0.04	0.00	0.05	0.00
9729	KIAA0408	0.00	0.00	0.10	-0.12
9733	SART3	0.02	0.00	0.00	0.15
9735	KNTC1	0.02	0.00	0.10	0.29

9741	LAPTM4A	0.00	0.00	0.00	NaN
9746	CLSTN3	0.02	0.00	0.15	-0.03
9748	SLK	0.02	0.00	0.00	0.01
9751	SNPH	0.00	0.00	0.00	NaN
9752	PCDHA9	0.00	0.00	0.00	NaN
9757	MLL4	0.00	0.00	0.00	NaN
9759	HDAC4	0.08	0.43	0.10	-0.13
9760	TOX	0.04	0.00	0.00	0.03
9764	KIAA0513	0.00	0.00	0.00	NaN
9766	KIAA0247	0.02	0.00	0.05	-0.04
9768	KIAA0101	0.02	0.00	0.00	0.27
9770	RASSF2	0.00	0.00	0.10	0.08
9772	KIAA0195	0.02	0.14	0.00	0.53
9776	KIAA0652	0.02	0.00	0.00	0.26
9778	KIAA0232	0.00	0.00	0.00	NaN
9782	MATR3	0.00	0.00	0.00	NaN
9784	SNX17	0.00	0.00	0.00	NaN
9786	KIAA0586	0.02	0.14	0.00	0.14
9791	PTDSS1	0.08	0.00	0.05	0.32
9794	MAML1	0.08	0.43	0.10	0.15
9796	PHYHIP	0.00	0.00	0.05	0.16
9798	KIAA0174	0.00	0.00	0.05	-0.14
9801	MRPL19	0.00	0.00	0.00	NaN
9802	DAZAP2	0.00	0.00	0.00	NaN
9808	KIAA0087	0.00	0.00	0.00	NaN
9810	RNF40	0.02	0.00	0.00	-0.07
9811	KIAA0427	0.00	0.00	0.00	NaN
9812	KIAA0141	0.00	0.00	0.00	NaN
9813	KIAA0494	0.02	0.00	0.05	0.00
9815	GIT2	0.02	0.00	0.00	-0.14
9817	KEAP1	0.02	0.00	0.15	0.06
9821	RB1CC1	0.00	0.14	0.00	0.18
9824	ARHGAP11A	0.00	0.00	0.00	NaN
9825	SPATA2	0.06	0.00	0.00	0.66
9826	ARHGEF11	0.04	0.14	0.05	0.10
9829	DNAJC6	0.02	0.00	0.15	0.14
9830	TRIM14	0.04	0.00	0.00	-0.11
9833	MELK	0.04	0.00	0.00	0.03
9840	KIAA0748	0.02	0.00	0.05	0.08
9844	ELMO1	0.02	0.00	0.00	0.06
9846	GAB2	0.02	0.00	0.00	0.51
9847	KIAA0528	0.06	0.00	0.00	-0.25
9851	KIAA0753	0.04	0.00	0.10	-0.19
9856	KIAA0319	0.00	0.00	0.00	NaN
9865	KIAA0644	0.00	0.00	0.00	NaN
9868	TOMM70A	0.00	0.00	0.15	0.12
9869	SETDB1	0.02	0.00	0.10	0.11

9870	KIAA0317	0.02	0.00	0.05	0.06
9871	SEC24D	0.02	0.00	0.05	0.29
9874	TLK1	0.00	0.00	0.00	NaN
9885	OSBPL2	0.08	0.00	0.05	0.22
9892	SNAP91	0.10	0.00	0.20	-0.05
9897	KIAA0196	0.10	0.00	0.05	0.32
9899	SV2B	0.02	0.00	0.10	0.36
9901	SRGAP3	0.00	0.00	0.10	0.07
9907	KIAA0415	0.00	0.00	0.00	NaN
9908	G3BP2	0.00	0.00	0.00	NaN
9915	ARNT2	0.02	0.00	0.00	0.00
9921	RNF10	0.02	0.00	0.10	0.06
9927	MFN2	0.02	0.00	0.00	0.07
9931	HELZ	0.12	0.00	0.20	0.16
9933	KIAA0020	0.02	0.00	0.00	-0.11
9935	MAFB	0.04	0.00	0.00	-0.09
9937	DCLRE1A	0.00	0.00	0.00	NaN
9939	RBM8A	0.02	0.00	0.00	0.16
9940	DLEC1	0.00	0.00	0.15	0.22
9942	XYLB	0.02	0.00	0.05	0.01
9943	OSR1	0.00	0.00	0.00	NaN
9946	CRYZL1	0.00	0.00	0.00	NaN
9948	WDR1	0.02	0.00	0.00	-0.04
9950	GOLGA5	0.02	0.00	0.00	-0.21
9953	HS3ST3B1	0.04	0.00	0.00	-0.02
9955	HS3ST3A1	0.04	0.00	0.00	-0.01
9956	HS3ST2	0.02	0.14	0.00	-0.08
9957	HS3ST1	0.04	0.14	0.10	0.19
9958	USP15	0.02	0.00	0.05	0.00
9961	MVP	0.06	0.14	0.00	0.08
9962	SLC23A1	0.00	0.00	0.00	NaN
9966	TNFSF15	0.02	0.00	0.00	-0.06
9970	NR1I3	0.00	0.00	0.00	NaN
9971	NR1H4	0.00	0.00	0.00	NaN
9972	NUP153	0.04	0.00	0.10	-0.05
9973	CCS	0.04	0.00	0.05	0.09
9978	RBX1	0.00	0.14	0.00	0.07
9985	REC8	0.02	0.14	0.10	-0.09
9986	RCE1	0.04	0.00	0.05	0.48
9987	HNRPDL	0.00	0.00	0.10	0.13
9988	DMTF1	0.00	0.00	0.00	NaN
9989	PPP4R1	0.02	0.00	0.00	0.09
9990	SLC12A6	0.04	0.00	0.05	0.21
9991	ROD1	0.02	0.00	0.00	0.01
9992	KCNE2	0.02	0.00	0.05	-0.04
9993	DGCR2	0.02	0.14	0.20	0.10
9994	CASP8AP2	0.00	0.00	0.05	0.23

10001	MED6	0.02	0.00	0.05	-0.04
10002	NR2E3	0.00	0.00	0.00	NaN
10003	NAALAD2	0.00	0.00	0.00	NaN
10010	TANK	0.02	0.00	0.00	-0.01
10014	HDAC5	0.00	0.00	0.00	NaN
10015	PDCD6IP	0.06	0.00	0.05	0.04
10016	PDCD6	0.00	0.00	0.00	NaN
10017	BCL2L10	0.00	0.00	0.00	NaN
10018	BCL2L11	0.02	0.00	0.00	0.19
10020	GNE	0.04	0.00	0.00	-0.04
10021	HCN4	0.00	0.00	0.05	-0.05
10022	INSL5	0.02	0.00	0.15	-0.02
10023	FRAT1	0.00	0.00	0.05	-0.14
10024	TROAP	0.00	0.00	0.00	NaN
10026	PIGK	0.06	0.00	0.00	0.00
10036	CHAF1A	0.02	0.00	0.15	0.08
10040	TOM1L1	0.02	0.00	0.10	0.07
10043	TOM1	0.00	0.29	0.05	-0.06
10045	SH2D3A	0.02	0.00	0.15	0.07
10047	CST8	0.00	0.00	0.00	NaN
10048	RANBP9	0.02	0.00	0.00	0.08
10049	DNAJB6	0.00	0.00	0.05	-0.01
10050	SLC17A4	0.02	0.00	0.05	-0.05
10053	AP1M2	0.02	0.00	0.15	0.05
10054	UBA2	0.00	0.00	0.00	NaN
10057	ABCC5	0.00	0.00	0.10	-0.04
10058	ABCB6	0.04	0.00	0.00	0.15
10059	DNM1L	0.02	0.00	0.00	0.09
10060	ABCC9	0.02	0.00	0.05	0.06
10061	ABCF2	0.02	0.00	0.00	0.08
10062	NR1H3	0.02	0.00	0.00	-0.08
10063	COX17	0.00	0.00	0.10	-0.13
10066	SCAMP2	0.00	0.00	0.05	-0.12
10067	SCAMP3	0.04	0.00	0.15	0.09
10068	IL18BP	0.06	0.00	0.15	-0.11
10072	DPP3	0.04	0.00	0.05	-0.10
10076	PTPRU	0.04	0.00	0.00	0.22
10078	TSSC4	0.00	0.00	0.00	NaN
10079	ATP9A	0.08	0.00	0.00	0.08
10086	HHLA1	0.06	0.00	0.00	0.00
10087	COL4A3BP	0.04	0.00	0.05	0.10
10089	KCNK7	0.04	0.00	0.05	-0.04
10090	UST	0.02	0.00	0.05	-0.03
10092	ARPC5	0.02	0.00	0.00	0.02
10093	ARPC4	0.00	0.00	0.10	0.11
10094	ARPC3	0.02	0.00	0.00	-0.08
10095	ARPC1B	0.02	0.00	0.00	-0.04

10096	ACTR3	0.00	0.00	0.10	0.05
10097	ACTR2	0.00	0.00	0.00	NaN
10101	NUBP2	0.18	0.00	0.10	-0.06
10102	TSFM	0.02	0.00	0.00	0.00
10105	PPIF	0.02	0.00	0.10	0.22
10107	TRIM10	0.04	0.14	0.05	0.20
10109	ARPC2	0.04	0.00	0.00	0.01
10110	SGK2	0.02	0.00	0.00	0.21
10111	RAD50	0.02	0.14	0.00	-0.02
10113	PREB	0.00	0.00	0.00	NaN
10114	HIPK3	0.02	0.00	0.00	0.22
10116	FEM1B	0.00	0.00	0.15	0.15
10120	ACTR1B	0.00	0.00	0.10	0.28
10121	ACTR1A	0.02	0.00	0.00	-0.18
10125	RASGRP1	0.04	0.00	0.05	0.10
10126	DNAL4	0.00	0.14	0.00	-0.22
10127	ZNF263	0.02	0.00	0.00	0.05
10128	LRPPRC	0.02	0.00	0.05	-0.23
10131	TRAP1	0.02	0.00	0.00	-0.07
10133	OPTN	0.00	0.14	0.00	0.19
10136	ELA3A	0.00	0.00	0.05	0.09
10137	RBM12	0.06	0.14	0.05	0.04
10138	YAF2	0.00	0.00	0.35	-0.21
10140	TOB1	0.10	0.00	0.10	0.07
10141	C4orf6	0.00	0.00	0.00	NaN
10142	AKAP9	0.00	0.00	0.00	NaN
10148	EBI3	0.02	0.00	0.15	-0.07
10154	PLXNC1	0.00	0.00	0.00	NaN
10155	TRIM28	0.00	0.00	0.00	NaN
10157	AASS	0.00	0.00	0.00	NaN
10160	FARP1	0.00	0.00	0.05	0.10
10164	CHST4	0.00	0.00	0.05	-0.05
10165	SLC25A13	0.04	0.14	0.05	0.09
10166	SLC25A15	0.00	0.00	0.00	NaN
10168	ZNF197	0.04	0.00	0.10	-0.01
10169	SERF2	0.04	0.00	0.00	-0.15
10179	RBM7	0.00	0.00	0.00	NaN
10180	RBM6	0.06	0.00	0.05	-0.07
10181	RBM5	0.00	0.00	0.05	0.05
10184	LHFPL2	0.00	0.00	0.00	NaN
10186	LHFP	0.00	0.00	0.00	NaN
10196	PRMT3	0.02	0.00	0.00	0.01
10197	PSME3	0.02	0.86	0.05	0.01
10198	MPHOSPH9	0.02	0.00	0.10	0.29
10199	MPHOSPH10	0.00	0.00	0.05	-0.12
10200	MPHOSPH6	0.02	0.00	0.00	-0.20
10202	DHRS2	0.02	0.14	0.10	-0.15

10203	CALCRL	0.00	0.00	0.05	0.01
10207	INADL	0.04	0.00	0.00	0.60
10211	FLOT1	0.04	0.14	0.05	0.15
10212	DDX39	0.00	0.00	0.05	0.03
10215	OLIG2	0.00	0.00	0.00	NaN
10216	PRG4	0.00	0.00	0.05	-0.06
10219	KLRG1	0.02	0.00	0.15	0.00
10220	GDF11	0.02	0.00	0.05	0.37
10223	GPA33	0.00	0.00	0.00	NaN
10228	STX6	0.04	0.00	0.10	0.03
10229	COQ7	0.00	0.00	0.00	NaN
10230	NBR2	0.00	0.86	0.00	-0.04
10232	MSLN	0.18	0.00	0.10	-0.05
10235	RASGRP2	0.02	0.00	0.00	-0.02
10239	AP3S2	0.00	0.00	0.00	NaN
10240	MRPS31	0.00	0.00	0.00	NaN
10242	KCNMB2	0.06	0.00	0.00	-0.09
10243	GPHN	0.00	0.00	0.05	0.00
10246	SLC17A2	0.02	0.00	0.05	-0.11
10249	GLYAT	0.02	0.00	0.00	0.19
10250	SRRM1	0.02	0.00	0.05	0.04
10252	SPRY1	0.02	0.00	0.00	0.29
10253	SPRY2	0.02	0.00	0.00	-0.11
10254	STAM2	0.00	0.00	0.00	NaN
10257	ABCC4	0.04	0.00	0.00	-0.06
10261	IGSF6	0.02	0.14	0.00	-0.13
10262	SF3B4	0.02	0.00	0.00	-0.04
10265	IRX5	0.02	0.00	0.00	0.07
10266	RAMP2	0.02	0.86	0.05	-0.09
10267	RAMP1	0.08	0.43	0.10	0.16
10268	RAMP3	0.02	0.00	0.00	0.04
10269	ZMPSTE24	0.02	0.00	0.05	-0.29
10270	AKAP8	0.04	0.00	0.00	-0.18
10272	FSTL3	0.06	0.00	0.15	-0.20
10273	STUB1	0.18	0.00	0.10	-0.02
10274	STAG1	0.02	0.14	0.00	0.17
10276	NET1	0.00	0.00	0.00	NaN
10277	UBE4B	0.00	0.00	0.00	NaN
10279	PRSS16	0.00	0.14	0.00	-0.01
10281	DSCR4	0.00	0.00	0.00	NaN
10282	BET1	0.02	0.00	0.00	-0.05
10284	SAP18	0.18	0.00	0.20	0.11
10286	BCAS2	0.04	0.00	0.05	0.40
10287	RGS19	0.08	0.00	0.05	0.07
10288	LILRB2	0.02	0.00	0.05	0.11
10291	SF3A1	0.00	0.29	0.05	-0.05
10294	DNAJA2	0.43	0.29	0.15	-0.41

10295	BCKDK	0.02	0.00	0.00	-0.09
10296	MAEA	0.02	0.00	0.00	0.09
10298	PAK4	0.06	0.00	0.05	0.06
10300	KATNB1	0.02	0.00	0.00	0.29
10301	DLEU1	0.04	0.00	0.00	-0.15
10302	SNAPC5	0.04	0.00	0.00	0.07
10308	ZNF267	0.43	0.29	0.15	0.06
10311	DSCR3	0.00	0.00	0.00	NaN
10312	TCIRG1	0.04	0.00	0.05	-0.04
10313	RTN3	0.02	0.00	0.00	-0.23
10314	LANCL1	0.02	0.00	0.05	-0.20
10317	B3GALT5	0.00	0.00	0.00	NaN
10318	NAF1	0.00	0.00	0.05	0.00
10319	LAMC3	0.04	0.00	0.00	-0.10
10326	SIRPB1	0.00	0.00	0.00	NaN
10327	AKR1A1	0.02	0.00	0.05	0.18
10329	TMEM5	0.04	0.00	0.00	0.26
10331	B3GNT3	0.04	0.00	0.00	0.22
10333	TLR6	0.04	0.00	0.10	-0.04
10342	TFG	0.00	0.00	0.15	0.14
10343	PKDREJ	0.02	0.14	0.05	-0.06
10346	TRIM22	0.02	0.00	0.00	-0.02
10347	ABCA7	0.02	0.00	0.05	-0.14
10351	ABCA8	0.12	0.00	0.20	-0.03
10352	WARS2	0.12	0.00	0.05	-0.01
10360	NPM3	0.02	0.00	0.00	-0.12
10362	HMG20B	0.02	0.00	0.15	-0.10
10363	HMG20A	0.00	0.00	0.05	0.06
10365	KLF2	0.04	0.00	0.00	-0.01
10367	CBARA1	0.04	0.00	0.05	0.11
10368	CACNG3	0.06	0.14	0.00	-0.02
10369	CACNG2	0.00	0.14	0.00	-0.01
10370	CITED2	0.02	0.00	0.05	-0.03
10371	SEMA3A	0.04	0.00	0.05	-0.03
10380	BPNT1	0.02	0.00	0.00	-0.03
10381	TUBB4	0.02	0.00	0.15	-0.14
10384	BTN3A3	0.00	0.14	0.00	0.07
10385	BTN2A2	0.00	0.14	0.00	-0.07
10388	SYCP2	0.12	0.00	0.00	0.08
10390	CEPT1	0.06	0.14	0.00	-0.14
10391	CORO2B	0.00	0.00	0.15	0.00
10394	PRG3	0.00	0.00	0.00	NaN
10395	DLC1	0.00	0.00	0.00	NaN
10396	ATP8A1	0.02	0.00	0.10	-0.04
10397	NDRG1	0.06	0.00	0.00	0.13
10398	MYL9	0.04	0.00	0.00	0.00
10400	PEMT	0.02	0.00	0.00	-0.02

10401	PIAS3	0.02	0.00	0.00	0.02
10404	PGCP	0.08	0.00	0.05	0.19
10406	WFDC2	0.04	0.00	0.05	0.17
10409	BASP1	0.02	0.00	0.00	0.08
10410	IFITM3	0.06	0.29	0.10	-0.07
10413	YAP1	0.00	0.00	0.00	NaN
10417	SPON2	0.02	0.00	0.00	0.09
10418	SPON1	0.00	0.00	0.00	NaN
10420	TESK2	0.02	0.00	0.05	0.27
10421	CD2BP2	0.02	0.00	0.00	-0.01
10423	CDIPT	0.06	0.14	0.00	-0.07
10424	PGRMC2	0.00	0.00	0.05	0.11
10425	ARIH2	0.00	0.00	0.05	0.32
10427	SEC24B	0.08	0.00	0.00	0.11
10428	CFDP1	0.00	0.00	0.00	NaN
10431	TIMM23	0.02	0.00	0.05	0.08
10432	RBM14	0.04	0.00	0.05	-0.03
10434	LYPLA1	0.00	0.00	0.00	NaN
10437	IFI30	0.02	0.00	0.00	0.09
10438	C1D	0.00	0.00	0.00	NaN
10440	TIMM17A	0.02	0.00	0.00	0.10
10445	MCRS1	0.02	0.00	0.00	0.28
10449	ACAA2	0.00	0.00	0.00	NaN
10450	PPIE	0.02	0.00	0.05	0.05
10451	VAV3	0.06	0.00	0.20	-0.10
10452	TOMM40	0.00	0.00	0.00	NaN
10454	MAP3K7IP1	0.00	0.14	0.05	0.05
10456	HAX1	0.04	0.00	0.15	0.07
10457	GPNMB	0.00	0.00	0.10	0.09
10458	BAIAP2	0.00	0.14	0.00	-0.01
10460	TACC3	0.02	0.00	0.00	-0.09
10461	MERTK	0.02	0.00	0.00	-0.06
10464	PIBF1	0.00	0.00	0.00	NaN
10465	PPIH	0.02	0.00	0.05	0.34
10466	COG5	0.00	0.00	0.00	NaN
10468	FST	0.18	0.00	0.30	0.01
10469	TIMM44	0.02	0.00	0.15	0.06
10474	TADA3L	0.00	0.00	0.10	0.23
10477	UBE2E3	0.00	0.00	0.00	NaN
10478	SLC25A17	0.00	0.14	0.00	-0.05
10481	HOXB13	0.04	0.00	0.05	-0.09
10482	NXF1	0.00	0.00	0.00	NaN
10483	SEC23B	0.02	0.00	0.00	0.20
10484	SEC23A	0.04	0.00	0.00	0.10
10486	CAP2	0.04	0.00	0.10	-0.17
10488	CREB3	0.00	0.00	0.00	NaN
10491	CRTAP	0.06	0.00	0.05	0.04

10494	STK25	0.08	0.43	0.10	-0.20
10498	CARM1	0.02	0.00	0.15	0.16
10499	NCOA2	0.04	0.14	0.00	0.20
10500	SEMA6C	0.02	0.00	0.10	0.35
10501	SEMA6B	0.02	0.00	0.15	-0.22
10505	SEMA4F	0.00	0.00	0.05	-0.01
10507	SEMA4D	0.00	0.00	0.00	NaN
10512	SEMA3C	0.02	0.00	0.00	0.15
10513	APPBP2	0.10	0.00	0.05	0.61
10514	MYBBP1A	0.04	0.00	0.10	0.14
10516	FBLN5	0.00	0.00	0.05	-0.17
10519	CIB1	0.00	0.00	0.00	NaN
10520	ZNF211	0.00	0.00	0.00	NaN
10521	DDX17	0.00	0.14	0.00	0.13
10522	DEAF1	0.02	0.00	0.05	0.02
10523	CHERP	0.04	0.00	0.00	0.12
10529	NEBL	0.02	0.00	0.00	-0.06
10534	SSSCA1	0.04	0.00	0.05	-0.13
10537	UBD	0.04	0.14	0.05	-0.06
10538	BATF	0.00	0.00	0.00	NaN
10540	DCTN2	0.02	0.14	0.00	0.01
10541	ANP32B	0.04	0.00	0.00	-0.10
10542	HBXIP	0.06	0.14	0.00	-0.12
10544	PROCR	0.00	0.00	0.10	-0.11
10548	TM9SF1	0.02	0.14	0.10	-0.06
10551	AGR2	0.00	0.00	0.00	NaN
10552	ARPC1A	0.02	0.00	0.00	0.00
10553	HTATIP2	0.02	0.00	0.00	0.22
10554	AGPAT1	0.02	0.00	0.05	0.03
10556	RPP30	0.02	0.00	0.05	-0.27
10557	RPP38	0.02	0.14	0.05	0.15
10558	SPTLC1	0.04	0.00	0.05	0.30
10559	SLC35A1	0.00	0.00	0.05	-0.05
10560	SLC19A2	0.02	0.00	0.00	0.09
10561	IFI44	0.00	0.00	0.00	NaN
10564	ARFGEF2	0.04	0.00	0.00	0.62
10566	AKAP3	0.02	0.00	0.00	0.06
10567	RABAC1	0.00	0.00	0.05	-0.21
10568	SLC34A2	0.00	0.00	0.00	NaN
10570	DPYSL4	0.00	0.00	0.05	0.18
10574	CCT7	0.00	0.00	0.05	-0.12
10575	CCT4	0.00	0.00	0.00	NaN
10576	CCT2	0.12	0.00	0.05	0.54
10577	NPC2	0.02	0.00	0.05	0.14
10578	GNLY	0.00	0.00	0.00	NaN
10579	TACC2	0.04	0.00	0.00	0.12
10581	IFITM2	0.06	0.29	0.10	-0.02

10584	COLEC10	0.10	0.00	0.15	0.01
10585	POMT1	0.04	0.00	0.00	-0.13
10586	MAB21L2	0.02	0.14	0.10	0.03
10587	TXNRD2	0.06	0.14	0.15	0.08
10588	MTHFS	0.02	0.00	0.00	0.13
10589	DRAP1	0.04	0.00	0.05	0.01
10590	SCGN	0.02	0.00	0.05	-0.04
10594	PRPF8	0.04	0.00	0.05	-0.11
10595	ERN2	0.00	0.00	0.00	NaN
10600	USP16	0.02	0.00	0.00	-0.07
10605	PAIP1	0.18	0.00	0.30	0.35
10606	PAICS	0.04	0.00	0.00	0.00
10607	TBL3	0.18	0.00	0.10	-0.17
10609	SC65	0.02	0.86	0.05	0.19
10612	TRIM3	0.00	0.00	0.00	NaN
10618	TGOLN2	0.00	0.00	0.00	NaN
10621	POLR3F	0.02	0.00	0.00	0.43
10626	TRIM16	0.00	0.00	0.00	NaN
10628	TXNIP	0.02	0.00	0.00	0.24
10629	TAF6L	0.00	0.00	0.00	NaN
10632	ATP5L	0.00	0.00	0.00	NaN
10634	GAS2L1	0.00	0.14	0.05	0.08
10636	RGS14	0.00	0.29	0.00	-0.04
10638	SPHAR	0.00	0.00	0.05	0.03
10645	CAMKK2	0.02	0.00	0.10	0.17
10647	SCGB1D2	0.00	0.00	0.05	-0.14
10648	SCGB1D1	0.00	0.00	0.05	0.03
10651	MTX2	0.02	0.00	0.00	0.22
10652	YKT6	0.00	0.00	0.00	NaN
10653	SPINT2	0.00	0.00	0.00	NaN
10654	PMVK	0.04	0.00	0.15	-0.05
10656	KHDRBS3	0.06	0.00	0.00	-0.06
10657	KHDRBS1	0.00	0.14	0.05	0.00
10658	CUGBP1	0.04	0.00	0.00	0.16
10659	CUGBP2	0.02	0.00	0.00	-0.05
10660	LBX1	0.02	0.00	0.00	0.17
10661	KLF1	0.00	0.14	0.05	-0.03
10664	CTCF	0.00	0.00	0.00	NaN
10665	C6orf10	0.02	0.00	0.05	0.16
10672	GNA13	0.12	0.00	0.20	0.29
10675	CSPG5	0.02	0.14	0.15	0.49
10677	AVIL	0.02	0.00	0.00	0.11
10678	B3GNT1	0.04	0.00	0.05	0.20
10681	GNB5	0.00	0.00	0.00	NaN
10683	DLL3	0.06	0.00	0.05	0.02
10686	CLDN16	0.00	0.00	0.05	-0.05
10687	PNMA2	0.00	0.00	0.05	0.46

10690	FUT9	0.08	0.00	0.00	0.02
10691	GMEB1	0.04	0.00	0.00	0.12
10692	RRH	0.08	0.00	0.00	0.05
10693	CCT6B	0.06	0.00	0.00	0.28
10694	CCT8	0.02	0.00	0.00	0.04
10712	C1orf2	0.04	0.00	0.15	0.20
10714	POLD3	0.02	0.14	0.00	0.11
10716	TBR1	0.02	0.00	0.00	-0.07
10721	POLQ	0.00	0.00	0.10	0.26
10723	SLC12A7	0.00	0.00	0.00	NaN
10724	MGEA5	0.02	0.00	0.00	0.05
10725	NFAT5	0.00	0.00	0.00	NaN
10726	NUDC	0.06	0.00	0.05	-0.11
10730	YME1L1	0.00	0.00	0.00	NaN
10732	TCFL5	0.08	0.00	0.05	-0.13
10734	STAG3	0.02	0.00	0.00	-0.01
10736	SIX2	0.02	0.00	0.05	0.01
10738	RFPL3	0.00	0.29	0.05	-0.17
10739	RFPL2	0.00	0.29	0.05	-0.11
10740	RFPL1S	0.00	0.14	0.00	-0.03
10741	RBBP9	0.02	0.00	0.00	0.10
10743	RAI1	0.02	0.00	0.00	-0.04
10744	PTTG2	0.06	0.14	0.10	-0.01
10745	PHTF1	0.02	0.00	0.05	0.18
10747	MASP2	0.02	0.00	0.00	0.08
10748	KLRA1	0.02	0.00	0.15	-0.21
10749	KIF1C	0.04	0.00	0.10	-0.10
10750	GRAP	0.02	0.00	0.00	-0.18
10752	CHL1	0.20	0.29	0.30	-0.05
10753	CAPN9	0.00	0.00	0.05	-0.07
10762	NUP50	0.00	0.14	0.05	0.01
10766	TOB2	0.00	0.14	0.00	0.14
10767	HBS1L	0.00	0.00	0.00	NaN
10768	AHCYL1	0.02	0.00	0.05	0.18
10772	FUSIP1	0.02	0.00	0.05	0.13
10775	POP4	0.00	0.14	0.05	0.40
10777	ARPP-21	0.06	0.00	0.05	-0.05
10781	ZNF266	0.02	0.00	0.15	0.08
10782	ZNF274	0.00	0.00	0.00	NaN
10785	WDR4	0.00	0.00	0.00	NaN
10786	SLC17A3	0.02	0.00	0.05	-0.08
10787	NCKAP1	0.00	0.00	0.00	NaN
10788	IQGAP2	0.04	0.00	0.05	0.02
10791	VAMP5	0.00	0.00	0.00	NaN
10793	ZNF273	0.00	0.00	0.00	NaN
10797	MTHFD2	0.00	0.00	0.05	0.03
10798	OR5I1	0.00	0.00	0.00	NaN

10799	RPP40	0.08	0.14	0.10	0.11
10802	SEC24A	0.00	0.00	0.00	NaN
10803	CCR9	0.02	0.14	0.15	0.20
10810	WASF3	0.00	0.00	0.05	0.16
10814	CPLX2	0.00	0.00	0.00	NaN
10825	NEU3	0.02	0.14	0.00	-0.03
10826	C5orf4	0.02	0.00	0.00	0.00
10841	FTCD	0.00	0.00	0.05	-0.11
10845	CLPX	0.00	0.00	0.10	-0.07
10846	PDE10A	0.02	0.00	0.05	0.06
10847	SRCAP	0.02	0.00	0.00	0.01
10855	HPSE	0.00	0.00	0.10	0.14
10856	RUVBL2	0.06	0.00	0.05	0.01
10859	LILRB1	0.02	0.00	0.05	0.10
10861	SLC26A1	0.06	0.00	0.05	-0.04
10863	ADAM28	0.00	0.00	0.00	NaN
10864	SLC22A7	0.00	0.00	0.00	NaN
10868	USP20	0.00	0.00	0.00	NaN
10869	USP19	0.00	0.00	0.05	0.02
10873	ME3	0.02	0.00	0.00	0.15
10874	NMU	0.00	0.00	0.10	0.20
10875	FGL2	0.00	0.00	0.00	NaN
10880	ACTL7B	0.02	0.00	0.05	0.04
10881	ACTL7A	0.02	0.00	0.05	-0.03
10884	MRPS30	0.18	0.00	0.30	-0.07
10885	WDR3	0.12	0.00	0.05	-0.05
10892	MALT1	0.00	0.00	0.15	0.26
10893	MMP24	0.00	0.00	0.10	0.17
10896	OCLM	0.00	0.00	0.05	-0.02
10898	CPSF4	0.02	0.00	0.00	0.17
10899	JTB	0.02	0.00	0.10	0.15
10904	BLCAP	0.04	0.00	0.00	0.35
10905	MAN1A2	0.08	0.00	0.10	0.14
10911	UTS2	0.00	0.00	0.00	NaN
10912	GADD45G	0.00	0.00	0.00	NaN
10913	EDAR	0.02	0.00	0.00	-0.03
10915	TCERG1	0.00	0.00	0.00	NaN
10921	RNPS1	0.18	0.00	0.10	-0.12
10922	FASTK	0.02	0.00	0.00	-0.02
10928	RALBP1	0.02	0.00	0.00	0.12
10930	APOBEC2	0.06	0.00	0.05	0.28
10934	MORF4	0.00	0.00	0.00	NaN
10935	PRDX3	0.04	0.00	0.05	-0.01
10936	GPR75	0.02	0.00	0.05	0.06
10938	EHD1	0.02	0.00	0.00	0.02
10939	AFG3L2	0.04	0.00	0.10	0.18
10942	PRSS21	0.02	0.00	0.00	-0.06

10945	KDELR1	0.02	0.00	0.00	0.18
10946	SF3A3	0.04	0.00	0.05	0.17
10947	AP3M2	0.04	0.14	0.05	0.18
10950	BTG3	0.00	0.00	0.00	NaN
10951	CBX1	0.04	0.00	0.05	0.16
10952	SEC61B	0.04	0.00	0.00	-0.13
10953	TOMM34	0.02	0.00	0.00	0.66
10963	STIP1	0.02	0.00	0.00	-0.03
10966	RAB40B	0.00	0.00	0.00	NaN
10969	EBNA1BP2	0.02	0.00	0.05	0.22
10970	CKAP4	0.00	0.29	0.00	0.27
10971	YWHAQ	0.00	0.00	0.00	NaN
10975	UQCR	0.02	0.00	0.05	0.05
10981	RAB32	0.02	0.00	0.05	0.03
10982	MAPRE2	0.02	0.00	0.05	0.06
10983	CCNI	0.00	0.00	0.00	NaN
10985	GCN1L1	0.02	0.00	0.10	0.26
10987	COPS5	0.00	0.00	0.00	NaN
10988	METAP2	0.00	0.00	0.00	NaN
10989	IMMT	0.04	0.00	0.05	0.06
10990	LILRB5	0.02	0.00	0.05	0.08
10991	SLC38A3	0.00	0.00	0.05	0.08
10992	SF3B2	0.04	0.00	0.05	-0.06
10993	SDS	0.02	0.00	0.00	-0.03
10994	ILVBL	0.04	0.00	0.00	-0.05
11005	SPINK5	0.02	0.14	0.00	0.10
11006	LILRB4	0.02	0.00	0.05	0.10
11009	IL24	0.00	0.00	0.05	0.00
11011	TLK2	0.10	0.00	0.05	0.51
11012	KLK11	0.02	0.00	0.05	0.02
11014	KDELR2	0.02	0.00	0.00	0.15
11015	KDELR3	0.00	0.14	0.00	0.12
11016	ATF7	0.00	0.00	0.00	NaN
11020	RABL4	0.00	0.14	0.00	0.21
11021	RAB35	0.02	0.00	0.10	0.17
11022	TDRKH	0.02	0.00	0.10	0.23
11024	LILRA1	0.02	0.00	0.05	0.00
11025	LILRB3	0.02	0.00	0.05	0.07
11026	LILRA3	0.02	0.00	0.05	0.01
11027	LILRA2	0.02	0.00	0.05	0.08
11030	RBPMS	0.06	0.00	0.05	0.07
11031	RAB31	0.00	0.00	0.05	0.07
11034	DSTN	0.00	0.00	0.00	NaN
11041	B3GNT6	0.02	0.00	0.05	-0.08
11044	POLS	0.00	0.00	0.00	NaN
11045	UPK1A	0.00	0.00	0.00	NaN
11047	ADRM1	0.08	0.00	0.05	0.19

11052	CPSF6	0.08	0.00	0.00	0.25
11054	OGFR	0.08	0.00	0.05	0.06
11055	ZPBP	0.00	0.00	0.00	NaN
11059	WWP1	0.06	0.00	0.15	-0.04
11060	WWP2	0.00	0.00	0.00	NaN
11063	SOX30	0.00	0.00	0.00	NaN
11065	UBE2C	0.02	0.00	0.00	0.17
11071	ATP9B	0.02	0.00	0.05	0.00
11072	DUSP14	0.06	0.00	0.00	0.21
11073	TOPBP1	0.00	0.00	0.10	-0.01
11074	TRIM31	0.04	0.14	0.05	0.17
11075	STMN2	0.10	0.00	0.05	-0.02
11077	HSF2BP	0.02	0.00	0.00	-0.05
11080	DNAJB4	0.06	0.00	0.00	-0.04
11081	KERA	0.00	0.00	0.00	NaN
11082	ESM1	0.00	0.14	0.05	0.00
11085	ADAM30	0.41	0.43	0.10	-0.02
11086	ADAM29	0.00	0.00	0.00	NaN
11092	C9orf9	0.04	0.00	0.00	-0.17
11094	C9orf7	0.04	0.00	0.00	-0.02
11095	ADAMTS8	0.02	0.00	0.00	-0.05
11096	ADAMTS5	0.04	0.00	0.00	0.02
11099	PTPN21	0.04	0.00	0.05	0.09
11102	RPP14	0.02	0.00	0.00	-0.27
11104	KATNA1	0.02	0.00	0.05	-0.01
11107	PRDM5	0.02	0.00	0.00	0.02
11108	PRDM4	0.02	0.00	0.00	0.23
11113	CIT	0.02	0.00	0.10	0.01
11118	BTN3A2	0.00	0.14	0.00	-0.02
11119	BTN3A1	0.00	0.14	0.00	0.08
11120	BTN2A1	0.00	0.14	0.00	-0.01
11122	PTPRT	0.02	0.00	0.00	-0.11
11124	FAF1	0.02	0.00	0.00	0.27
11127	KIF3A	0.00	0.00	0.00	NaN
11130	ZWINT	0.02	0.14	0.10	-0.05
11131	CAPN11	0.04	0.14	0.20	-0.21
11132	CAPN10	0.08	0.43	0.10	-0.09
11133	KPTN	0.00	0.00	0.00	NaN
11136	SLC7A9	0.00	0.00	0.00	NaN
11137	PWP1	0.02	0.00	0.00	0.10
11140	CDC37	0.02	0.00	0.15	0.20
11142	PKIG	0.02	0.00	0.00	0.56
11144	DMC1	0.00	0.14	0.00	0.11
11147	HHLA3	0.04	0.14	0.00	0.08
11148	HHLA2	0.02	0.00	0.00	0.08
11151	CORO1A	0.02	0.00	0.00	0.01
11154	AP4S1	0.02	0.00	0.00	0.55

11155	LDB3	0.00	0.00	0.05	0.00
11156	PTP4A3	0.10	0.00	0.15	0.19
11157	LSM6	0.00	0.14	0.00	-0.15
11159	RABL2A	0.00	0.00	0.10	0.01
11161	C14orf1	0.00	0.00	0.00	NaN
11162	NUDT6	0.00	0.14	0.05	-0.10
11163	NUDT4	0.00	0.00	0.00	NaN
11165	NUDT3	0.00	0.00	0.00	NaN
11166	SOX21	0.04	0.00	0.00	0.08
11167	FSTL1	0.00	0.00	0.10	0.04
11173	ADAMTS7	0.00	0.00	0.05	0.01
11174	ADAMTS6	0.04	0.00	0.05	-0.09
11176	BAZ2A	0.00	0.00	0.00	NaN
11177	BAZ1A	0.04	0.00	0.00	0.10
11178	LZTS1	0.00	0.00	0.00	NaN
11179	ZNF277	0.00	0.00	0.00	NaN
11180	WDR6	0.00	0.00	0.05	0.17
11181	TREH	0.00	0.00	0.05	-0.03
11182	SLC2A6	0.04	0.00	0.00	-0.11
11183	MAP4K5	0.02	0.00	0.00	0.20
11184	MAP4K1	0.00	0.00	0.00	NaN
11186	RASSF1	0.00	0.00	0.10	0.31
11187	PKP3	0.06	0.29	0.10	-0.10
11188	NISCH	0.00	0.00	0.10	-0.05
11189	TNRC4	0.02	0.00	0.10	0.05
11193	WBP4	0.00	0.00	0.00	NaN
11194	ABCB8	0.02	0.00	0.00	0.02
11197	WIF1	0.06	0.00	0.00	-0.08
11199	ANXA10	0.00	0.00	0.00	NaN
11200	CHEK2	0.00	0.14	0.05	0.26
11201	POLI	0.00	0.00	0.05	0.01
11202	KLK8	0.00	0.00	0.00	NaN
11211	FZD10	0.02	0.00	0.10	-0.10
11212	PROSC	0.12	0.14	0.05	0.45
11215	AKAP11	0.02	0.00	0.00	0.12
11216	AKAP10	0.10	0.00	0.05	-0.02
11217	AKAP2	0.02	0.00	0.05	0.10
11221	DUSP10	0.02	0.00	0.00	0.03
11222	MRPL3	0.02	0.00	0.00	-0.06
11223	MSTP9	0.02	0.00	0.00	0.03
11224	RPL35	0.06	0.00	0.00	0.11
11226	GALNT6	0.00	0.00	0.00	NaN
11232	POLG2	0.12	0.00	0.20	0.34
11235	PDCD10	0.00	0.00	0.00	NaN
11237	RNF24	0.00	0.00	0.05	0.18
11243	PMF1	0.04	0.14	0.05	-0.02
11247	NXPH4	0.00	0.00	0.00	NaN

11248	NXPH3	0.04	0.00	0.05	0.20
11250	GPR45	0.02	0.00	0.10	-0.08
11251	GPR44	0.00	0.00	0.05	-0.05
11252	PACSIN2	0.00	0.14	0.05	-0.08
11255	HRH3	0.08	0.00	0.05	0.02
11257	TP53TG1	0.00	0.00	0.00	NaN
11258	DCTN3	0.00	0.00	0.00	NaN
11260	XPOT	0.04	0.14	0.00	0.24
11261	CHP	0.00	0.00	0.00	NaN
11262	SP140	0.02	0.00	0.15	0.23
11264	PXMP4	0.02	0.00	0.05	-0.17
11266	DUSP12	0.00	0.00	0.00	NaN
11274	USP18	0.02	0.14	0.20	-0.12
11275	KLHL2	0.02	0.00	0.00	0.05
11277	TREX1	0.00	0.00	0.05	0.22
11278	KLF12	0.02	0.00	0.00	-0.09
11282	MGAT4B	0.08	0.43	0.10	-0.19
11283	CYP4F8	0.04	0.00	0.00	-0.06
11284	PNKP	0.02	0.00	0.05	0.09
11285	B4GALT7	0.00	0.29	0.00	0.09
11313	LYPLA2	0.02	0.00	0.05	-0.07
11316	COPE	0.02	0.00	0.00	0.19
11320	MGAT4A	0.02	0.00	0.10	-0.02
11329	STK38	0.00	0.00	0.00	NaN
11330	CTRC	0.02	0.00	0.00	-0.07
11333	PDAP1	0.02	0.00	0.00	0.01
11335	CBX3	0.00	0.00	0.00	NaN
11337	GABARAP	0.00	0.00	0.00	NaN
11339	OIP5	0.00	0.00	0.00	NaN
11341	SCRG1	0.00	0.00	0.00	NaN
11342	RNF13	0.00	0.00	0.00	NaN
11343	MGLL	0.04	0.00	0.00	0.18
11345	GABARAPL2	0.00	0.00	0.00	NaN
22795	NID2	0.02	0.00	0.00	0.23
22797	TFEC	0.00	0.00	0.05	0.14
22798	LAMB4	0.00	0.00	0.00	NaN
22800	RRAS2	0.00	0.00	0.00	NaN
22802	CLCA4	0.04	0.00	0.05	0.03
22808	MRAS	0.00	0.00	0.00	NaN
22809	ATF5	0.02	0.00	0.05	0.37
22822	PHLDA1	0.02	0.00	0.00	0.12
22826	DNAJC8	0.04	0.00	0.00	0.13
22832	KIAA1009	0.10	0.00	0.20	-0.16
22856	CHSY1	0.16	0.00	0.10	-0.10
22858	ICK	0.02	0.00	0.00	-0.02
22863	KIAA0831	0.00	0.00	0.10	0.11
22871	NLGN1	0.06	0.00	0.00	-0.06

22873	DZIP1	0.04	0.00	0.00	-0.02
22875	ENPP4	0.04	0.14	0.20	-0.11
22878	KIAA1012	0.02	0.00	0.10	-0.05
22881	ANKRD6	0.00	0.00	0.05	0.09
22883	CLSTN1	0.00	0.00	0.00	NaN
22889	KIAA0907	0.04	0.14	0.05	0.01
22894	DIS3	0.00	0.00	0.00	NaN
22895	RPH3A	0.02	0.00	0.00	-0.05
22899	ARHGEF15	0.00	0.00	0.00	NaN
22903	BTBD3	0.00	0.00	0.00	NaN
22905	EPN2	0.02	0.00	0.00	0.02
22908	SACM1L	0.02	0.14	0.15	-0.02
22913	RALY	0.02	0.00	0.05	-0.07
22916	NCBP2	0.02	0.00	0.00	0.42
22919	MAPRE1	0.04	0.00	0.15	-0.07
22920	KIFAP3	0.02	0.00	0.00	-0.03
22924	MAPRE3	0.00	0.00	0.00	NaN
22925	PLA2R1	0.00	0.00	0.05	-0.01
22926	ATF6	0.00	0.00	0.00	NaN
22932	POMZP3	0.00	0.00	0.00	NaN
22933	SIRT2	0.06	0.00	0.05	0.00
22934	RPIA	0.04	0.00	0.05	0.16
22936	ELL2	0.00	0.00	0.00	NaN
22937	SCAP	0.02	0.14	0.15	0.27
22938	SNW1	0.00	0.00	0.00	NaN
22939	PTRF	0.02	0.86	0.05	0.23
22941	SHANK2	0.06	0.00	0.10	0.31
22943	DKK1	0.02	0.14	0.10	0.15
22944	KIN	0.00	0.00	0.00	NaN
22948	CCT5	0.00	0.00	0.00	NaN
22950	SLC4A1AP	0.00	0.00	0.00	NaN
22953	P2RX2	0.08	0.14	0.05	0.05
22954	TRIM32	0.02	0.00	0.00	0.16
22955	SCMH1	0.02	0.00	0.05	0.11
22977	AKR7A3	0.00	0.00	0.00	NaN
22978	NT5C2	0.02	0.00	0.00	-0.23
22986	SORCS3	0.02	0.00	0.05	-0.12
23011	RAB21	0.12	0.00	0.05	0.33
23012	STK38L	0.00	0.00	0.00	NaN
23014	FBXO21	0.02	0.00	0.10	0.03
23018	KIAA0284	0.02	0.00	0.10	-0.05
23037	PDZD2	0.00	0.00	0.00	NaN
23040	MYT1L	0.20	0.14	0.35	-0.14
23049	SMG1	0.00	0.00	0.00	NaN
23053	KIAA0913	0.02	0.00	0.00	0.15
23054	NCOA6	0.02	0.00	0.05	-0.09
23056	KIAA0754	0.04	0.00	0.05	0.19

23062	GGA2	0.02	0.14	0.00	0.02
23065	KIAA0090	0.00	0.00	0.00	NaN
23075	SWAP70	0.00	0.00	0.00	NaN
23078	KIAA0564	0.00	0.00	0.00	NaN
23089	PEG10	0.02	0.00	0.00	0.02
23095	KIF1B	0.00	0.00	0.00	NaN
23107	MRPS27	0.06	0.00	0.00	-0.32
23114	NFASC	0.02	0.00	0.05	-0.01
23118	MAP3K7IP2	0.02	0.00	0.05	-0.03
23119	HIC2	0.06	0.14	0.15	0.33
23120	ATP10B	0.00	0.00	0.00	NaN
23122	CLASP2	0.06	0.00	0.05	0.11
23136	EPB41L3	0.02	0.00	0.00	-0.07
23138	N4BP3	0.00	0.29	0.00	-0.10
23152	CIC	0.00	0.00	0.05	0.35
23157	9/2/2010	0.08	0.43	0.10	-0.05
23161	SNX13	0.00	0.00	0.05	0.30
23162	MAPK8IP3	0.18	0.00	0.10	-0.09
23163	GGA3	0.02	0.14	0.00	0.37
23166	STAB1	0.00	0.00	0.10	0.06
23173	METAP1	0.00	0.00	0.00	NaN
23175	LPIN1	0.00	0.00	0.00	NaN
23178	PASK	0.08	0.43	0.10	0.09
23194	FBXL7	0.00	0.00	0.00	NaN
23195	MDN1	0.00	0.00	0.05	0.25
23199	KIAA0182	0.00	0.00	0.00	NaN
23200	ATP11B	0.00	0.00	0.10	0.29
23212	RRS1	0.00	0.00	0.00	NaN
23216	TBC1D1	0.06	0.14	0.10	-0.04
23228	PLCL2	0.06	0.00	0.05	0.12
23231	KIAA0746	0.00	0.00	0.00	NaN
23236	PLCB1	0.00	0.00	0.00	NaN
23237	ARC	0.10	0.00	0.15	0.04
23240	KIAA0922	0.02	0.14	0.00	-0.03
23245	ASTN2	0.02	0.00	0.00	0.07
23246	BOP1	0.10	0.00	0.15	0.28
23247	KIAA0556	0.06	0.14	0.00	0.03
23250	ATP11A	0.06	0.00	0.00	0.00
23255	KIAA0802	0.02	0.00	0.00	0.02
23263	MCF2L	0.06	0.00	0.00	0.16
23269	MGA	0.00	0.00	0.00	NaN
23277	KIAA0664	0.00	0.00	0.00	NaN
23279	NUP160	0.04	0.00	0.00	0.01
23281	KIAA0774	0.00	0.00	0.05	-0.11
23283	CSTF2T	0.00	0.00	0.05	-0.11
23285	KIAA1107	0.00	0.00	0.00	NaN
23287	AGTPBP1	0.00	0.00	0.00	NaN

23299	BICD2	0.04	0.00	0.05	0.12
23303	KIF13B	0.02	0.00	0.00	-0.05
23321	TRIM2	0.02	0.14	0.00	0.00
23325	KIAA1033	0.00	0.00	0.00	NaN
23326	USP22	0.10	0.00	0.05	0.25
23327	NEDD4L	0.00	0.00	0.15	0.37
23332	CLASP1	0.00	0.00	0.10	-0.14
23334	KIAA0467	0.02	0.00	0.05	0.26
23335	WDR7	0.00	0.00	0.00	NaN
23339	VPS39	0.04	0.00	0.00	-0.12
23349	KIAA1045	0.00	0.00	0.00	NaN
23351	KIAA0323	0.02	0.00	0.05	0.07
23358	USP24	0.02	0.00	0.00	0.32
23359	KIAA0574	0.00	0.00	0.00	NaN
23364	KIAA1109	0.00	0.14	0.05	0.29
23365	ARHGEF12	0.00	0.00	0.00	NaN
23366	KIAA0895	0.00	0.00	0.00	NaN
23369	PUM2	0.00	0.00	0.00	NaN
23376	KIAA0776	0.08	0.00	0.00	-0.01
23379	KIAA0947	0.00	0.00	0.00	NaN
23380	SRGAP2	0.02	0.00	0.05	0.12
23383	KIAA0892	0.12	0.00	0.15	0.02
23385	NCSTN	0.00	0.00	0.00	NaN
23387	KIAA0999	0.02	0.00	0.00	-0.14
23392	KIAA0368	0.02	0.00	0.00	0.52
23394	ADNP	0.10	0.00	0.00	0.06
23395	LARS2	0.02	0.14	0.15	0.20
23396	PIP5K1C	0.02	0.00	0.15	-0.27
23401	FRAT2	0.00	0.00	0.05	-0.11
23405	DICER1	0.00	0.00	0.05	0.03
23408	SIRT5	0.02	0.00	0.00	0.04
23409	SIRT4	0.02	0.00	0.10	-0.20
23410	SIRT3	0.06	0.29	0.10	0.01
23411	SIRT1	0.00	0.00	0.00	NaN
23413	FREQ	0.04	0.00	0.00	-0.18
23415	KCNH4	0.02	0.86	0.05	0.13
23417	MLYCD	0.00	0.00	0.00	NaN
23418	CRB1	0.00	0.00	0.00	NaN
23421	ITGB3BP	0.04	0.00	0.00	0.32
23426	GRIP1	0.08	0.00	0.00	0.00
23428	SLC7A8	0.02	0.14	0.10	-0.10
23429	RYBP	0.16	0.14	0.30	-0.19
23430	TPSD1	0.18	0.00	0.10	0.00
23431	AP4E1	0.00	0.00	0.00	NaN
23435	TARDBP	0.02	0.00	0.00	0.04
23436	ELA3B	0.00	0.00	0.05	-0.10
23443	SLC35A3	0.02	0.00	0.00	-0.11

23450	SF3B3	0.00	0.00	0.00	NaN
23451	SF3B1	0.00	0.00	0.05	0.01
23452	ANGPTL2	0.02	0.00	0.00	-0.06
23457	ABCB9	0.02	0.00	0.10	0.06
23460	ABCA6	0.12	0.00	0.20	0.09
23461	ABCA5	0.12	0.00	0.20	0.29
23462	HEY1	0.10	0.00	0.05	0.02
23463	ICMT	0.02	0.00	0.00	0.25
23464	GCAT	0.00	0.14	0.00	0.22
23466	CBX6	0.00	0.14	0.00	-0.01
23468	CBX5	0.02	0.00	0.05	-0.06
23469	PHF3	0.12	0.00	0.05	0.04
23473	CAPN7	0.00	0.00	0.05	-0.08
23475	QPRT	0.06	0.14	0.00	-0.05
23476	BRD4	0.04	0.00	0.00	-0.09
23480	SEC61G	0.02	0.00	0.05	-0.33
23481	PES1	0.00	0.29	0.05	0.16
23484	LEPROTL1	0.06	0.00	0.05	-0.17
23491	CES3	0.02	0.00	0.00	-0.08
23493	HEY2	0.00	0.00	0.10	-0.01
23498	HAAO	0.02	0.00	0.05	0.07
23499	MACF1	0.04	0.00	0.05	0.17
23500	DAAM2	0.02	0.00	0.05	-0.10
23506	KIAA0240	0.06	0.00	0.05	-0.02
23509	POFUT1	0.04	0.00	0.15	0.10
23513	SCRIB	0.10	0.00	0.15	0.36
23514	KIAA0146	0.04	0.00	0.00	-0.03
23519	ANP32D	0.04	0.00	0.05	0.16
23520	ANP32C	0.00	0.00	0.05	-0.02
23521	RPL13A	0.02	0.00	0.05	0.05
23523	CABIN1	0.00	0.14	0.05	0.08
23524	SRRM2	0.02	0.00	0.00	-0.06
23528	ZNF281	0.00	0.00	0.05	0.06
23530	NNT	0.18	0.00	0.30	0.19
23531	MMD	0.02	0.00	0.05	0.15
23532	PRAME	0.06	0.14	0.15	0.03
23536	ADAT1	0.00	0.00	0.00	NaN
23538	OR52A1	0.00	0.00	0.05	-0.04
23539	SLC16A8	0.00	0.14	0.00	0.12
23541	SEC14L2	0.00	0.29	0.05	-0.03
23543	RBM9	0.00	0.29	0.05	0.04
23544	SEZ6L	0.00	0.14	0.05	-0.04
23545	ATP6V0A2	0.02	0.00	0.10	0.05
23546	SYNGR4	0.02	0.00	0.00	0.04
23549	DNPEP	0.04	0.00	0.00	0.17
23552	CCRK	0.00	0.00	0.00	NaN
23553	HYAL4	0.00	0.00	0.00	NaN

23556	PIGN	0.02	0.00	0.00	-0.06
23558	WBP2	0.02	0.14	0.05	0.06
23562	CLDN14	0.00	0.00	0.00	NaN
23563	CHST5	0.00	0.00	0.00	NaN
23564	DDAH2	0.02	0.00	0.05	0.35
23583	SMUG1	0.02	0.00	0.05	-0.18
23594	ORC6L	0.43	0.29	0.15	-0.24
23595	ORC3L	0.00	0.00	0.05	-0.13
23596	OPN3	0.02	0.00	0.10	0.01
23600	AMACR	0.02	0.14	0.05	0.06
23603	CORO1C	0.02	0.00	0.00	0.02
23604	DAPK2	0.02	0.00	0.00	0.03
23607	CD2AP	0.00	0.00	0.00	NaN
23608	MKRN1	0.00	0.00	0.00	NaN
23609	MKRN2	0.00	0.00	0.05	0.11
23612	PHLDA3	0.00	0.00	0.05	0.02
23614	PPY2	0.10	0.00	0.05	0.12
23615	PYY2	0.10	0.00	0.05	-0.05
23616	SH3BP1	0.00	0.14	0.00	0.10
23619	ZIM2	0.00	0.00	0.05	0.03
23620	NTSR2	0.00	0.00	0.00	NaN
23624	CBLC	0.00	0.00	0.00	NaN
23632	CA14	0.02	0.00	0.10	-0.07
23633	KPNA6	0.00	0.14	0.05	0.00
23635	SSBP2	0.00	0.00	0.00	NaN
23636	NUP62	0.02	0.00	0.05	0.38
23640	HSPBP1	0.02	0.00	0.05	0.26
23645	PPP1R15A	0.06	0.00	0.05	0.29
23649	POLA2	0.02	0.00	0.00	-0.06
23650	TRIM29	0.00	0.00	0.00	NaN
23654	PLXNB2	0.02	0.14	0.05	-0.02
23657	SLC7A11	0.02	0.00	0.00	-0.03
23658	LSM5	0.02	0.00	0.00	0.18
23670	TMEM2	0.00	0.00	0.00	NaN
23673	STX12	0.04	0.00	0.00	0.00
23677	SH3BP4	0.02	0.00	0.00	-0.01
23682	RAB38	0.00	0.00	0.00	NaN
23710	GABARAPL1	0.02	0.00	0.15	0.14
23733	C9orf3	0.00	0.00	0.00	NaN
23742	C15orf2	0.00	0.14	0.00	0.03
23743	BHMT2	0.00	0.00	0.00	NaN
23746	AIPL1	0.04	0.00	0.10	-0.04
23753	SDF2L1	0.06	0.14	0.15	0.10
23759	PPIL2	0.06	0.14	0.15	0.15
23761	PISD	0.00	0.29	0.05	-0.01
23762	OSBP2	0.00	0.29	0.05	0.01
23764	MAFF	0.00	0.14	0.00	0.02

23767	FLRT3	0.00	0.14	0.00	-0.06
23768	FLRT2	0.02	0.00	0.00	-0.02
23769	FLRT1	0.02	0.00	0.00	-0.10
23770	FKBP8	0.02	0.00	0.00	0.24
23774	BRD1	0.02	0.14	0.05	-0.01
23779	ARHGAP8	0.00	0.14	0.05	0.08
23780	APOL2	0.00	0.14	0.00	0.26
23787	MTCH1	0.04	0.00	0.00	-0.20
23788	MTCH2	0.04	0.00	0.00	-0.29
24139	EML2	0.00	0.00	0.05	0.02
24141	C20orf103	0.00	0.00	0.05	0.00
24145	PANX1	0.00	0.00	0.00	NaN
24146	CLDN15	0.00	0.14	0.00	0.04
24147	FJX1	0.00	0.00	0.10	0.14
24150	TP53TG3	0.43	0.29	0.15	0.07
25764	HYPK	0.04	0.00	0.00	-0.01
25769	SLC24A2	0.02	0.00	0.05	-0.01
25788	RAD54B	0.08	0.00	0.05	0.37
25792	CIZ1	0.00	0.00	0.00	NaN
25793	FBXO7	0.00	0.29	0.05	0.05
25794	FSCN2	0.00	0.00	0.00	NaN
25796	PGLS	0.04	0.00	0.00	0.55
25797	QPCT	0.00	0.00	0.00	NaN
25801	GCA	0.02	0.00	0.00	0.02
25802	LMOD1	0.02	0.00	0.00	0.08
25804	LSM4	0.02	0.00	0.00	0.16
25806	VAX2	0.00	0.00	0.05	-0.13
25809	TTLL1	0.00	0.14	0.05	-0.05
25819	CCRN4L	0.00	0.00	0.05	-0.04
25820	ARIH1	0.04	0.00	0.05	0.20
25821	MTO1	0.02	0.00	0.00	-0.01
25822	DNAJB5	0.00	0.00	0.00	NaN
25823	TPSG1	0.18	0.00	0.10	0.04
25825	BACE2	0.00	0.00	0.05	0.03
25827	FBXL2	0.06	0.00	0.05	0.01
25828	TXN2	0.00	0.14	0.00	0.00
25830	SULT4A1	0.00	0.14	0.05	-0.02
25837	RAB26	0.18	0.00	0.10	0.09
25839	COG4	0.00	0.00	0.00	NaN
25849	DKFZP564O08	0.00	0.00	0.00	NaN
25850	ZNF345	0.02	0.00	0.05	0.08
25855	BRMS1	0.04	0.00	0.05	0.12
25859	PART1	0.00	0.00	0.05	0.03
25873	RPL36	0.02	0.00	0.15	0.00
25913	POT1	0.00	0.00	0.00	NaN
25932	CLIC4	0.02	0.00	0.05	0.09
25939	SAMHD1	0.04	0.00	0.00	0.03

25980	C20orf4	0.04	0.00	0.00	0.39
25998	IBTK	0.10	0.00	0.20	-0.06
26031	OSBPL3	0.00	0.00	0.05	0.20
26039	SS18L1	0.08	0.00	0.05	0.21
26040	SETBP1	0.02	0.00	0.00	-0.21
26047	CNTNAP2	0.02	0.00	0.00	0.02
26051	PPP1R16B	0.06	0.00	0.05	0.14
26063	DECR2	0.18	0.00	0.10	-0.10
26064	RAI14	0.02	0.14	0.05	0.12
26082	DKFZP434L18	0.00	0.00	0.00	NaN
26085	KLK13	0.02	0.00	0.05	0.03
26088	GGA1	0.00	0.14	0.00	0.21
26108	PYGO1	0.00	0.00	0.00	NaN
26118	WSB1	0.10	0.00	0.05	0.11
26121	PRPF31	0.02	0.00	0.05	-0.01
26136	TES	0.00	0.00	0.00	NaN
26152	ZNF337	0.04	0.00	0.15	-0.04
26154	ABCA12	0.02	0.00	0.00	0.02
26168	SENP3	0.02	0.00	0.00	-0.07
26189	OR1A2	0.04	0.00	0.10	-0.07
26190	FBXW2	0.02	0.00	0.00	0.06
26191	PTPN22	0.02	0.00	0.05	0.19
26205	GMEB2	0.08	0.00	0.05	0.30
26206	SPAG8	0.00	0.00	0.00	NaN
26211	OR2F1	0.04	0.00	0.05	-0.03
26220	DGCR5	0.02	0.14	0.20	0.18
26227	PHGDH	0.41	0.43	0.10	-0.25
26229	B3GAT3	0.00	0.00	0.05	0.03
26230	TIAM2	0.04	0.00	0.05	0.12
26232	FBXO2	0.02	0.00	0.00	0.31
26233	FBXL6	0.10	0.00	0.15	0.32
26234	FBXL5	0.00	0.00	0.00	NaN
26235	FBXL4	0.08	0.00	0.00	0.06
26249	KLHL3	0.02	0.00	0.00	-0.08
26251	KCNG2	0.02	0.00	0.05	-0.08
26255	PTTG3	0.00	0.00	0.00	NaN
26256	CABYR	0.00	0.00	0.10	0.02
26261	FBXO24	0.00	0.00	0.00	NaN
26263	FBXO22	0.00	0.00	0.05	-0.06
26266	SLC13A4	0.00	0.00	0.10	-0.07
26268	FBXO9	0.02	0.00	0.00	0.25
26271	FBXO5	0.02	0.00	0.05	0.03
26273	FBXO3	0.02	0.00	0.00	-0.12
26275	HIBCH	0.00	0.00	0.05	-0.22
26276	VPS33B	0.02	0.00	0.10	-0.06
26277	TINF2	0.02	0.14	0.10	0.14
26278	SACS	0.02	0.00	0.00	-0.03

26279	PLA2G2D	0.02	0.00	0.05	-0.01
26281	FGF20	0.00	0.00	0.10	-0.07
26284	ERAL1	0.10	0.00	0.05	0.10
26285	CLDN17	0.18	0.14	0.00	0.24
26286	ARFGAP1	0.08	0.00	0.05	-0.16
26287	ANKRD2	0.00	0.14	0.00	0.09
26289	AK5	0.06	0.00	0.00	-0.06
26290	GALNT8	0.02	0.00	0.00	-0.10
26291	FGF21	0.06	0.00	0.05	0.23
26292	MYCBP	0.04	0.00	0.05	0.03
26298	EHF	0.02	0.00	0.00	-0.06
26333	OR7A17	0.00	0.00	0.05	-0.07
26468	LHX6	0.06	0.00	0.00	-0.04
26469	PTPN18	0.00	0.00	0.10	-0.23
26472	PPP1R14B	0.02	0.00	0.00	-0.03
26476	OR10J1	0.00	0.00	0.00	NaN
26499	PLEK2	0.00	0.00	0.05	-0.03
26502	NARF	0.00	0.00	0.00	NaN
26503	SLC17A5	0.02	0.00	0.00	-0.09
26504	CNNM4	0.00	0.00	0.10	-0.04
26505	CNNM3	0.00	0.00	0.10	-0.20
26507	CNNM1	0.00	0.00	0.00	NaN
26508	HEYL	0.02	0.00	0.05	-0.03
26511	CHIC2	0.00	0.00	0.00	NaN
26515	FXC1	0.00	0.00	0.00	NaN
26517	TIMM13	0.02	0.00	0.05	0.07
26519	TIMM10	0.00	0.00	0.00	NaN
26520	TIMM9	0.02	0.14	0.00	-0.02
26521	TIMM8B	0.02	0.00	0.00	0.03
26523	EIF2C1	0.00	0.00	0.00	NaN
26528	DAZAP1	0.02	0.00	0.05	0.10
26529	OR12D2	0.04	0.14	0.05	-0.08
26531	OR11A1	0.04	0.14	0.05	0.04
26532	OR10H3	0.04	0.00	0.00	-0.03
26538	OR10H2	0.04	0.00	0.00	0.05
26539	OR10H1	0.04	0.00	0.00	-0.01
26542	OR10C1	0.04	0.14	0.05	0.06
26575	RGS17	0.02	0.00	0.05	-0.01
26577	PCOLCE2	0.02	0.00	0.00	-0.05
26578	OSTF1	0.00	0.00	0.00	NaN
26586	CKAP2	0.04	0.00	0.10	0.06
26589	MRPL46	0.00	0.00	0.00	NaN
26608	TBL2	0.00	0.00	0.00	NaN
26658	OR7C2	0.04	0.00	0.00	-0.06
26659	OR7C1	0.00	0.00	0.05	-0.11
26692	OR2W1	0.04	0.14	0.05	0.11
26707	OR2J2	0.04	0.14	0.05	-0.16

26747	NUFIP1	0.00	0.00	0.00	NaN
26750	RPS6KC1	0.02	0.00	0.15	0.17
26873	OPLAH	0.10	0.00	0.15	0.37
26953	RANBP6	0.02	0.00	0.00	0.20
26959	HBP1	0.00	0.00	0.00	NaN
26960	NBEA	0.02	0.00	0.00	0.03
26973	CHORDC1	0.00	0.00	0.00	NaN
26984	SEC22A	0.00	0.00	0.10	0.18
26986	PABPC1	0.12	0.00	0.25	0.17
26994	RNF11	0.02	0.00	0.00	0.12
26998	FETUB	0.02	0.14	0.00	0.00
26999	CYFIP2	0.00	0.00	0.00	NaN
27004	TCL6	0.00	0.00	0.05	0.10
27005	USP21	0.00	0.00	0.00	NaN
27006	FGF22	0.06	0.00	0.15	0.01
27010	TPK1	0.02	0.00	0.05	0.03
27019	DNAI1	0.04	0.00	0.00	0.10
27022	FOXD3	0.04	0.00	0.00	0.00
27023	FOXB1	0.00	0.00	0.00	NaN
27030	MLH3	0.00	0.00	0.00	NaN
27032	ATP2C1	0.02	0.00	0.00	0.06
27034	ACAD8	0.00	0.00	0.00	NaN
27036	SIGLEC7	0.02	0.00	0.05	0.02
27039	PKD2L2	0.02	0.00	0.00	-0.13
27040	LAT	0.06	0.14	0.00	0.10
27043	PELP1	0.04	0.00	0.10	0.08
27065	D4S234E	0.00	0.00	0.00	NaN
27067	STAU2	0.02	0.00	0.00	0.03
27069	GHITM	0.06	0.14	0.05	0.10
27071	DAPP1	0.00	0.00	0.00	NaN
27072	VPS41	0.00	0.00	0.00	NaN
27074	LAMP3	0.00	0.00	0.10	0.17
27087	B3GAT1	0.00	0.00	0.00	NaN
27091	CACNG5	0.12	0.00	0.20	0.10
27094	KCNMB3	0.00	0.00	0.00	NaN
27097	TAF5L	0.00	0.00	0.05	0.08
27098	CLUL1	0.02	0.00	0.05	0.17
27113	BBC3	0.00	0.00	0.05	-0.06
27115	PDE7B	0.00	0.00	0.00	NaN
27121	DKK4	0.04	0.14	0.05	-0.04
27122	DKK3	0.04	0.00	0.00	0.19
27123	DKK2	0.08	0.00	0.00	0.05
27129	HSPB7	0.02	0.00	0.00	-0.02
27130	INVS	0.04	0.00	0.00	0.17
27131	SNX5	0.00	0.00	0.00	NaN
27132	CPNE7	0.00	0.00	0.10	-0.07
27134	TJP3	0.02	0.00	0.15	-0.25

27141	CIDEB	0.02	0.14	0.10	-0.02
27156	RTDR1	0.06	0.14	0.15	0.08
27173	SLC39A1	0.02	0.00	0.10	-0.14
27175	TUBG2	0.02	0.86	0.05	0.01
27180	SIGLEC9	0.02	0.00	0.05	-0.03
27181	SIGLEC8	0.02	0.00	0.05	-0.09
27183	VPS4A	0.00	0.00	0.00	NaN
27185	DISC1	0.00	0.00	0.00	NaN
27190	IL17B	0.00	0.14	0.05	-0.15
27230	SERP1	0.02	0.00	0.05	-0.11
27232	GNMT	0.06	0.00	0.05	0.02
27233	SULT1C2	0.02	0.00	0.00	0.16
27242	TNFRSF21	0.02	0.00	0.00	0.03
27250	PDCD4	0.00	0.14	0.00	-0.01
27253	PCDH17	0.04	0.00	0.10	0.10
27255	CNTN6	0.20	0.29	0.30	-0.05
27257	LSM1	0.08	0.00	0.00	0.57
27258	LSM3	0.00	0.00	0.05	-0.29
27284	SULT1B1	0.00	0.14	0.00	0.13
27285	TEKT2	0.00	0.00	0.00	NaN
27290	SPINK4	0.04	0.00	0.00	-0.12
27299	ADAMDEC1	0.00	0.00	0.00	NaN
27302	BMP10	0.00	0.14	0.00	-0.17
27303	RBMS3	0.00	0.00	0.05	-0.10
27304	MOCS3	0.10	0.00	0.00	0.40
27306	PGDS	0.04	0.00	0.05	-0.05
27314	RAB30	0.00	0.00	0.00	NaN
27315	FRAG1	0.00	0.00	0.05	0.18
27329	ANGPTL3	0.04	0.00	0.00	-0.12
27343	POLL	0.02	0.00	0.00	0.17
27345	KCNMB4	0.12	0.00	0.05	0.15
27347	STK39	0.00	0.00	0.00	NaN
27430	MAT2B	0.00	0.00	0.00	NaN
27434	POLM	0.00	0.00	0.00	NaN
27436	EML4	0.02	0.00	0.05	0.01
27440	CECR5	0.02	0.14	0.20	0.20
27445	PCLO	0.02	0.00	0.00	-0.06
28316	CDH20	0.02	0.00	0.00	-0.06
28513	CDH19	0.02	0.00	0.00	-0.01
28957	MRPS28	0.10	0.00	0.05	0.23
28964	GIT1	0.10	0.00	0.05	-0.01
28973	MRPS18B	0.04	0.14	0.05	0.04
28977	MRPL42	0.00	0.00	0.00	NaN
28996	HIPK2	0.00	0.00	0.05	0.03
28997	PRO0611	0.00	0.14	0.00	0.10
28998	MRPL13	0.10	0.00	0.15	0.19
28999	KLF15	0.02	0.00	0.00	0.05

29018	PRO1768	0.00	0.00	0.00	NaN
29053	PRO0628	0.04	0.00	0.00	0.03
29058	C20orf30	0.00	0.00	0.10	-0.03
29074	MRPL18	0.04	0.00	0.05	-0.05
29075	HSPC072	0.04	0.00	0.05	-0.05
29088	MRPL15	0.00	0.00	0.00	NaN
29092	HSPC157	0.00	0.00	0.05	0.15
29093	MRPL22	0.02	0.00	0.00	0.14
29094	HSPC159	0.00	0.00	0.00	NaN
29104	N6AMT1	0.02	0.00	0.00	0.19
29106	SCG3	0.00	0.00	0.00	NaN
29107	NXT1	0.00	0.00	0.00	NaN
29109	FHOD1	0.02	0.00	0.00	0.06
29110	TBK1	0.04	0.14	0.00	0.40
29117	BRD7	0.08	0.00	0.05	-0.08
29122	TSP50	0.02	0.14	0.15	0.12
29125	C11orf21	0.00	0.00	0.00	NaN
29127	RACGAP1	0.02	0.00	0.00	-0.09
29760	BLNK	0.00	0.00	0.00	NaN
29761	USP25	0.00	0.00	0.10	0.21
29763	PACSIN3	0.02	0.00	0.00	-0.11
29766	TMOD3	0.00	0.00	0.00	NaN
29767	TMOD2	0.00	0.00	0.00	NaN
29775	CARD10	0.00	0.14	0.00	0.00
29777	ABT1	0.00	0.14	0.00	0.07
29780	PARVB	0.00	0.14	0.05	0.05
29802	VPREB3	0.00	0.14	0.05	0.04
29844	TFPT	0.02	0.00	0.05	-0.03
29851	ICOS	0.00	0.00	0.00	NaN
29855	UBN1	0.00	0.00	0.00	NaN
29880	ALG5	0.02	0.00	0.00	-0.13
29881	NPC1L1	0.02	0.00	0.00	-0.05
29882	APC2	0.02	0.00	0.05	-0.14
29883	CNOT7	0.00	0.00	0.10	0.12
29887	SNX10	0.00	0.00	0.00	NaN
29894	CPSF1	0.10	0.00	0.15	0.28
29907	SNX15	0.02	0.00	0.00	-0.04
29911	HOOK2	0.06	0.14	0.00	0.01
29916	SNX11	0.04	0.00	0.05	0.05
29922	NME7	0.02	0.00	0.00	-0.10
29925	GMPPB	0.06	0.00	0.05	0.07
29926	GMPPA	0.04	0.00	0.00	0.02
29927	SEC61A1	0.04	0.00	0.00	0.36
29928	TIMM22	0.00	0.00	0.00	NaN
29929	ALG6	0.04	0.00	0.00	0.15
29930	PCDHB1	0.00	0.00	0.00	NaN
29942	PURG	0.04	0.00	0.00	-0.03

29943	PADI1	0.02	0.00	0.00	-0.02
29947	DNMT3L	0.02	0.00	0.00	-0.12
29949	IL19	0.00	0.00	0.05	-0.01
29953	TRHDE	0.12	0.00	0.05	-0.08
29954	POMT2	0.00	0.00	0.00	NaN
29956	LASS2	0.02	0.00	0.10	-0.30
29960	FTSJ2	0.00	0.00	0.00	NaN
29965	C16orf5	0.00	0.00	0.00	NaN
29970	SCHIP1	0.02	0.00	0.10	0.03
29980	DONSON	0.00	0.00	0.00	NaN
29986	SLC39A2	0.02	0.00	0.00	-0.04
29988	SLC2A8	0.02	0.00	0.00	-0.04
29989	OBP2B	0.04	0.00	0.00	-0.07
29994	BAZ2B	0.00	0.00	0.05	-0.17
29995	LMCD1	0.00	0.00	0.05	-0.08
29997	GLTSCR2	0.00	0.00	0.00	NaN
29998	GLTSCR1	0.00	0.00	0.00	NaN
29999	FSCN3	0.02	0.00	0.00	-0.13
30001	ERO1L	0.02	0.00	0.00	0.28
30008	EFEMP2	0.04	0.00	0.05	-0.05
30009	TBX21	0.00	0.00	0.00	NaN
30013	S100A14	0.02	0.00	0.10	0.00
30811	HUNK	0.00	0.00	0.00	NaN
30813	VSX1	0.04	0.00	0.15	-0.13
30814	PLA2G2E	0.02	0.00	0.05	0.56
30817	EMR2	0.00	0.00	0.05	0.00
30819	KCNIP2	0.02	0.00	0.00	-0.07
30820	KCNIP1	0.02	0.00	0.00	-0.08
30835	CD209	0.02	0.00	0.15	0.33
30844	EHD4	0.00	0.00	0.00	NaN
30845	EHD3	0.00	0.00	0.00	NaN
30846	EHD2	0.00	0.00	0.00	NaN
30849	PIK3R4	0.02	0.00	0.00	0.01
43847	KLK14	0.02	0.00	0.05	0.01
50485	SMARCAL1	0.02	0.00	0.00	-0.09
50486	G0S2	0.02	0.00	0.00	-0.03
50487	PLA2G3	0.00	0.29	0.05	0.03
50506	DUOX2	0.02	0.00	0.05	0.05
50507	NOX4	0.00	0.00	0.00	NaN
50508	NOX3	0.04	0.00	0.05	0.01
50509	COL5A3	0.02	0.00	0.15	-0.09
50514	12/1/2010	0.02	0.00	0.00	-0.01
50613	UBQLN3	0.02	0.00	0.00	-0.03
50615	IL21R	0.06	0.14	0.00	-0.06
50616	IL22	0.08	0.14	0.05	0.14
50617	ATP6V0A4	0.00	0.00	0.00	NaN
50618	ITSN2	0.00	0.00	0.00	NaN

50619	DEF6	0.04	0.00	0.00	-0.09
50626	CYHR1	0.10	0.00	0.15	0.18
50649	ARHGEF4	0.00	0.00	0.10	0.11
50650	ARHGEF3	0.00	0.00	0.05	0.26
50674	NEUROG3	0.00	0.00	0.00	NaN
50700	RDH8	0.02	0.00	0.15	0.21
50801	KCNK4	0.02	0.00	0.00	-0.03
50805	IRX4	0.00	0.00	0.00	NaN
50813	COPS7A	0.02	0.00	0.15	0.03
50831	TAS2R3	0.00	0.14	0.05	-0.11
50832	TAS2R4	0.00	0.14	0.05	0.09
50833	TAS2R16	0.00	0.00	0.00	NaN
50834	TAS2R1	0.00	0.00	0.00	NaN
50835	TAS2R9	0.02	0.00	0.15	-0.16
50836	TAS2R8	0.02	0.00	0.15	-0.11
50837	TAS2R7	0.02	0.00	0.15	-0.04
50838	TAS2R13	0.02	0.00	0.15	-0.02
50839	TAS2R10	0.02	0.00	0.15	0.16
50840	TAS2R14	0.02	0.00	0.15	-0.09
50853	VILL	0.00	0.00	0.15	0.17
50855	PARD6A	0.00	0.00	0.00	NaN
50861	STMN3	0.08	0.00	0.05	-0.16
50865	HEBP1	0.04	0.00	0.00	-0.12
50937	CDON	0.02	0.00	0.00	0.01
50939	IMPG2	0.00	0.00	0.00	NaN
50940	PDE11A	0.02	0.00	0.00	0.04
50944	SHANK1	0.00	0.00	0.00	NaN
51021	MRPS16	0.00	0.00	0.00	NaN
51022	GLRX2	0.00	0.00	0.00	NaN
51023	MRPS18C	0.00	0.00	0.10	-0.36
51025	Magmas	0.00	0.00	0.00	NaN
51050	PI15	0.06	0.00	0.00	-0.08
51056	LAP3	0.00	0.00	0.00	NaN
51065	RPS27L	0.00	0.00	0.00	NaN
51069	MRPL2	0.06	0.00	0.05	0.25
51070	NOSIP	0.02	0.00	0.05	0.17
51073	MRPL4	0.02	0.00	0.15	0.08
51081	MRPS7	0.02	0.14	0.00	0.27
51100	SH3GLB1	0.04	0.00	0.05	-0.01
51106	TFB1M	0.04	0.00	0.05	0.07
51117	COQ4	0.00	0.00	0.00	NaN
51121	RPL26L1	0.00	0.00	0.10	-0.16
51127	TRIM17	0.00	0.00	0.05	-0.10
51129	ANGPTL4	0.02	0.00	0.15	-0.02
51135	IRAK4	0.00	0.00	0.35	-0.16
51138	COPS4	0.00	0.00	0.10	0.16
51144	HSD17B12	0.00	0.00	0.00	NaN

51147	ING4	0.02	0.00	0.15	-0.03
51155	HN1	0.02	0.14	0.00	0.31
51156	SERPINA10	0.00	0.00	0.00	NaN
51160	VPS28	0.10	0.00	0.15	0.33
51163	DBR1	0.00	0.00	0.00	NaN
51164	DCTN4	0.00	0.14	0.00	-0.11
51168	MYO15A	0.02	0.00	0.00	0.19
51176	LEF1	0.08	0.00	0.00	-0.05
51179	HAO2	0.41	0.43	0.10	0.12
51181	DCXR	0.00	0.00	0.00	NaN
51188	SS18L2	0.00	0.00	0.05	-0.04
51196	PLCE1	0.02	0.00	0.05	0.05
51199	NIN	0.02	0.00	0.00	-0.07
51200	CPA4	0.00	0.00	0.00	NaN
51206	GP6	0.02	0.00	0.05	0.03
51208	CLDN18	0.00	0.00	0.00	NaN
51214	IGF2AS	0.02	0.00	0.05	0.14
51222	ZNF219	0.02	0.00	0.00	-0.03
51232	CRIM1	0.00	0.00	0.00	NaN
51265	CDKL3	0.00	0.00	0.00	NaN
51268	PIPOX	0.10	0.00	0.05	-0.23
51274	KLF3	0.04	0.00	0.10	0.06
51278	IER5	0.04	0.00	0.10	-0.05
51282	SCAND1	0.06	0.14	0.05	0.37
51294	PCDH12	0.00	0.00	0.00	NaN
51297	PLUNC	0.02	0.00	0.00	-0.04
51298	THEG	0.06	0.00	0.15	0.10
51302	CYP39A1	0.04	0.14	0.20	0.20
51304	ZDHHC3	0.04	0.00	0.10	0.14
51318	MRPL35	0.04	0.00	0.05	0.16
51322	WAC	0.00	0.00	0.00	NaN
51327	ERAF	0.02	0.00	0.00	-0.06
51332	SPTBN5	0.00	0.00	0.00	NaN
51338	MS4A4A	0.00	0.00	0.00	NaN
51340	CRNKL1	0.02	0.00	0.00	-0.05
51343	FZR1	0.02	0.00	0.15	-0.14
51348	KLRF1	0.02	0.00	0.15	0.05
51361	HOOK1	0.06	0.00	0.00	0.29
51363	GALNAC4S-6S	0.04	0.00	0.05	-0.04
51367	POP5	0.02	0.00	0.10	0.13
51373	MRPS17	0.04	0.14	0.20	0.12
51375	SNX7	0.02	0.00	0.00	-0.05
51378	ANGPT4	0.00	0.00	0.00	NaN
51379	CRLF3	0.10	0.00	0.05	-0.12
51380	CSAD	0.00	0.00	0.00	NaN
51382	ATP6V1D	0.00	0.00	0.05	-0.03
51384	WNT16	0.02	0.00	0.10	-0.18

51411	BIN2	0.00	0.00	0.00	NaN
51421	AMOTL2	0.00	0.00	0.10	0.26
51422	PRKAG2	0.02	0.00	0.00	-0.08
51430	C1orf9	0.04	0.00	0.05	0.27
51433	ANAPC5	0.02	0.00	0.10	0.14
51439	FAM8A1	0.04	0.00	0.10	-0.05
51440	HPCAL4	0.02	0.00	0.05	-0.02
51458	RHCG	0.00	0.00	0.00	NaN
51475	CABP2	0.02	0.00	0.00	-0.03
51477	ISYNA1	0.02	0.00	0.00	0.05
51478	HSD17B7	0.00	0.00	0.00	NaN
51497	TH1L	0.12	0.00	0.00	0.51
51504	HSPC152	0.02	0.00	0.00	0.18
51507	C20orf43	0.06	0.00	0.00	0.21
51512	GTSE1	0.02	0.14	0.05	0.11
51526	C20orf111	0.02	0.00	0.10	0.32
51540	SCLY	0.08	0.43	0.10	0.19
51547	SIRT7	0.00	0.00	0.00	NaN
51548	SIRT6	0.02	0.00	0.15	-0.11
51550	CINP	0.02	0.00	0.00	-0.03
51552	RAB14	0.06	0.00	0.00	0.11
51554	CCRL1	0.00	0.00	0.10	-0.09
51560	RAB6B	0.00	0.00	0.10	0.12
51561	IL23A	0.00	0.00	0.00	NaN
51562	MBIP	0.04	0.00	0.00	0.07
51585	PCF11	0.00	0.00	0.00	NaN
51592	TRIM33	0.04	0.00	0.05	0.34
51606	ATP6V1H	0.00	0.00	0.00	NaN
51621	KLF13	0.00	0.00	0.00	NaN
51642	MRPL48	0.02	0.00	0.00	0.22
51650	MRPS33	0.00	0.14	0.05	0.15
51663	ZFR	0.00	0.00	0.10	0.24
51666	ASB4	0.00	0.00	0.00	NaN
51678	MPP6	0.00	0.00	0.05	0.05
51701	NLK	0.10	0.00	0.05	0.02
51702	PADI3	0.02	0.00	0.00	-0.01
51704	GPRC5B	0.00	0.00	0.00	NaN
51715	RAB23	0.06	0.14	0.05	0.06
51729	WBP11	0.02	0.00	0.00	-0.01
51733	UPB1	0.00	0.14	0.05	0.01
51734	SEPX1	0.18	0.00	0.10	-0.10
51741	WWOX	0.00	0.00	0.00	NaN
51744	CD244	0.00	0.00	0.00	NaN
51761	ATP8A2	0.06	0.00	0.00	-0.01
51764	GNG13	0.18	0.00	0.10	-0.10
51768	TM7SF3	0.00	0.00	0.00	NaN
51776	ZAK	0.00	0.00	0.00	NaN

51778	MYOZ2	0.02	0.00	0.05	0.01
51807	TUBA8	0.02	0.14	0.20	-0.10
51809	GALNT7	0.00	0.00	0.00	NaN
51816	CECR1	0.02	0.14	0.20	0.21
53335	BCL11A	0.00	0.00	0.05	-0.01
53339	BTBD1	0.02	0.00	0.00	-0.14
53340	SPA17	0.02	0.00	0.00	0.07
53343	NUDT9	0.02	0.00	0.00	0.09
53346	TM6SF1	0.02	0.00	0.00	-0.08
53347	UBASH3A	0.00	0.00	0.00	NaN
53353	LRP1B	0.02	0.00	0.00	-0.03
53358	SHC3	0.00	0.00	0.00	NaN
53371	NUP54	0.00	0.00	0.00	NaN
53405	CLIC5	0.04	0.14	0.20	-0.15
53407	STX18	0.00	0.00	0.00	NaN
53615	MBD3	0.02	0.00	0.05	0.13
53616	ADAM22	0.00	0.00	0.00	NaN
53635	PTOV1	0.02	0.00	0.05	0.17
53820	DSCR6	0.00	0.00	0.00	NaN
53822	FXYD7	0.00	0.00	0.00	NaN
53826	FXYD6	0.00	0.00	0.00	NaN
53827	FXYD5	0.00	0.00	0.00	NaN
53832	IL20RA	0.00	0.00	0.10	-0.11
53836	GPR87	0.00	0.00	0.00	NaN
53838	C11orf24	0.04	0.00	0.05	0.06
53840	TRIM34	0.02	0.00	0.00	-0.10
53904	MYO3A	0.02	0.00	0.00	-0.08
53905	DUOX1	0.02	0.00	0.05	0.11
53916	RAB4B	0.00	0.00	0.05	0.03
53918	PELO	0.18	0.00	0.30	-0.12
53942	CNTN5	0.04	0.00	0.00	0.32
53944	CSNK1G1	0.02	0.00	0.00	-0.26
53947	A4GALT	0.00	0.14	0.05	-0.01
54020	SLC37A1	0.00	0.00	0.00	NaN
54039	PCBP3	0.00	0.00	0.05	0.18
54069	C21orf45	0.02	0.00	0.00	0.06
54107	POLE3	0.00	0.00	0.00	NaN
54112	GPR88	0.02	0.00	0.00	-0.04
54148	MRPL39	0.02	0.00	0.05	0.15
54149	C21orf91	0.00	0.00	0.00	NaN
54207	KCNK10	0.00	0.00	0.00	NaN
54209	TREM2	0.06	0.00	0.05	-0.22
54210	TREM1	0.06	0.00	0.05	0.10
54212	SNTG1	0.02	0.00	0.00	-0.16
54221	SNTG2	0.20	0.14	0.35	-0.11
54329	GPR85	0.00	0.00	0.00	NaN
54332	GDAP1	0.06	0.00	0.00	0.13

54344	DPM3	0.04	0.00	0.15	0.23
54345	SOX18	0.08	0.00	0.05	-0.20
54361	WNT4	0.00	0.00	0.05	0.23
54363	HAO1	0.00	0.00	0.00	NaN
54407	SLC38A2	0.04	0.00	0.05	0.12
54434	SSH1	0.02	0.00	0.00	-0.11
54453	RIN2	0.02	0.00	0.00	0.04
54462	KIAA1128	0.06	0.14	0.05	0.32
54472	TOLLIP	0.02	0.00	0.05	0.00
54474	KRT20	0.02	0.86	0.05	0.14
54487	DGCR8	0.06	0.14	0.15	0.18
54490	UGT2B28	0.00	0.14	0.00	0.01
54504	CPVL	0.00	0.00	0.00	NaN
54540	FLJ10404	0.00	0.29	0.00	0.06
54551	MAGEL2	0.14	0.14	0.25	-0.03
54554	WDR5B	0.00	0.00	0.10	0.03
54556	ING3	0.00	0.00	0.15	0.17
54558	SPATA6	0.02	0.00	0.05	-0.06
54576	UGT1A8	0.00	0.00	0.00	NaN
54578	UGT1A6	0.00	0.00	0.00	NaN
54581	SCAND2	0.00	0.00	0.00	NaN
54583	EGLN1	0.00	0.00	0.05	0.24
54585	LZTFL1	0.02	0.14	0.15	-0.09
54600	UGT1A9	0.00	0.00	0.00	NaN
54621	FLJ20674	0.02	0.00	0.10	0.05
54626	HES2	0.02	0.00	0.00	0.11
54657	UGT1A4	0.00	0.00	0.00	NaN
54658	UGT1A1	0.00	0.00	0.00	NaN
54659	UGT1A3	0.00	0.00	0.00	NaN
54674	LRRN3	0.02	0.00	0.10	-0.02
54676	GTPBP2	0.00	0.00	0.00	NaN
54677	CROT	0.00	0.00	0.00	NaN
54714	CNGB3	0.06	0.00	0.15	0.02
54715	A2BP1	0.04	0.00	0.00	-0.10
54718	BTN2A3	0.00	0.14	0.00	0.18
54766	BTG4	0.02	0.00	0.05	-0.07
54788	DNAJB12	0.04	0.00	0.05	0.24
54795	TRPM4	0.06	0.00	0.05	0.36
54805	CNNM2	0.02	0.00	0.00	-0.02
54810	GIPC2	0.06	0.00	0.00	-0.05
54829	ASPN	0.04	0.00	0.05	-0.21
54834	GDAP2	0.12	0.00	0.05	-0.14
54840	APTX	0.00	0.00	0.15	0.24
54848	FLJ20184	0.08	0.00	0.00	-0.05
54860	MS4A12	0.00	0.00	0.05	-0.07
54866	PPP1R14D	0.00	0.00	0.00	NaN
54873	PALMD	0.02	0.00	0.00	-0.19

54878	DPP8	0.00	0.00	0.10	0.02
54879	ST7L	0.02	0.00	0.05	0.23
54898	ELOVL2	0.04	0.00	0.00	-0.09
54904	WHSC1L1	0.08	0.00	0.00	0.77
54910	SEMA4C	0.00	0.00	0.10	-0.01
54948	MRPL16	0.00	0.00	0.00	NaN
54953	C1orf27	0.00	0.00	0.05	-0.04
54971	BANP	0.00	0.00	0.10	0.02
54976	C20orf27	0.02	0.00	0.00	0.01
54979	HRASLS2	0.02	0.00	0.00	-0.03
54982	CLN6	0.00	0.00	0.15	0.23
54994	C20orf11	0.08	0.00	0.05	0.00
55023	PHIP	0.10	0.00	0.20	0.05
55038	CDCA4	0.02	0.00	0.10	0.03
55040	EPN3	0.10	0.00	0.10	0.20
55056	FLJ10038	0.00	0.00	0.00	NaN
55074	OXR1	0.12	0.00	0.25	0.30
55089	SLC38A4	0.04	0.00	0.05	0.26
55096	FLJ10213	0.16	0.14	0.30	-0.30
55118	CRTAC1	0.00	0.14	0.00	-0.05
55124	PIWIL2	0.00	0.00	0.05	0.01
55146	ZDHHC4	0.02	0.00	0.00	0.17
55168	MRPS18A	0.00	0.00	0.00	NaN
55173	MRPS10	0.06	0.00	0.05	0.23
55184	C20orf12	0.02	0.00	0.00	0.11
55243	KIRREL	0.04	0.14	0.05	-0.08
55257	C20orf20	0.08	0.00	0.05	0.11
55283	MCOLN3	0.04	0.00	0.05	0.06
55290	BRF2	0.12	0.14	0.05	0.47
55294	FBXW7	0.02	0.14	0.00	-0.15
55317	C20orf29	0.02	0.00	0.00	0.19
55321	C20orf46	0.00	0.00	0.00	NaN
55336	FBXL8	0.02	0.00	0.00	0.08
55350	VNN3	0.02	0.00	0.00	0.00
55357	TBC1D2	0.04	0.00	0.00	0.08
55364	IMPACT	0.00	0.00	0.10	0.08
55367	LRDD	0.02	0.00	0.05	0.01
55384	MEG3	0.02	0.00	0.00	-0.05
55388	MCM10	0.00	0.14	0.00	-0.06
55423	SIRPB2	0.00	0.00	0.00	NaN
55466	DNAJA4	0.00	0.00	0.05	0.17
55486	PARL	0.00	0.00	0.10	-0.01
55506	H2AFY2	0.02	0.00	0.00	0.20
55507	GPRC5D	0.04	0.00	0.00	0.08
55520	ELAC1	0.00	0.00	0.05	0.15
55521	TRIM36	0.02	0.00	0.00	0.21
55554	KLK15	0.00	0.00	0.00	NaN

55576	STAB2	0.00	0.00	0.00	NaN
55577	NAGK	0.00	0.00	0.05	0.15
55584	CHRNA9	0.04	0.00	0.10	-0.07
55607	PPP1R9A	0.02	0.00	0.00	-0.18
55625	ZDHHC7	0.00	0.00	0.00	NaN
55630	SLC39A4	0.10	0.00	0.15	0.28
55643	BTBD2	0.02	0.00	0.05	0.06
55644	OSGEP	0.02	0.00	0.00	0.09
55647	RAB20	0.00	0.00	0.05	-0.12
55653	BCAS4	0.10	0.00	0.00	0.16
55661	DDX27	0.06	0.00	0.00	0.56
55662	HIF1AN	0.00	0.00	0.05	-0.02
55665	URG4	0.00	0.00	0.00	NaN
55669	MFN1	0.00	0.00	0.00	NaN
55692	LUC7L	0.18	0.00	0.10	-0.14
55701	FLJ10357	0.02	0.00	0.00	0.13
55713	ZNF334	0.02	0.00	0.00	0.02
55715	DOK4	0.02	0.00	0.00	0.12
55734	ZFP64	0.08	0.00	0.05	0.38
55737	VPS35	0.43	0.29	0.15	-0.46
55742	PARVA	0.04	0.00	0.00	0.13
55743	CHFR	0.08	0.14	0.05	0.09
55746	NUP133	0.00	0.00	0.05	0.05
55757	UGCGL2	0.04	0.00	0.00	0.02
55759	WDR12	0.00	0.00	0.00	NaN
55766	H2AFJ	0.02	0.00	0.00	0.01
55769	ZNF83	0.02	0.00	0.05	0.01
55775	TDP1	0.00	0.00	0.00	NaN
55794	DDX28	0.02	0.00	0.00	0.08
55799	CACNA2D3	0.02	0.00	0.00	-0.02
55801	IL26	0.08	0.14	0.05	-0.12
55806	HR	0.00	0.00	0.05	0.00
55821	ALLC	0.20	0.14	0.35	-0.19
55823	VPS11	0.00	0.00	0.05	-0.09
55835	CENPJ	0.06	0.00	0.00	-0.09
55867	SLC22A11	0.02	0.00	0.00	-0.07
55890	GPRC5C	0.04	0.00	0.00	-0.05
55891	LENEP	0.04	0.00	0.15	-0.14
55892	MYNN	0.00	0.00	0.05	0.04
55900	ZNF302	0.00	0.00	0.00	NaN
55907	CMAS	0.02	0.00	0.05	-0.08
55908	LOC55908	0.02	0.00	0.00	-0.03
55909	BIN3	0.00	0.00	0.05	-0.07
55911	APOB48R	0.06	0.14	0.00	0.08
55914	ERBB2IP	0.00	0.00	0.00	NaN
55915	LANCL2	0.04	0.14	0.20	0.07
55930	MYO5C	0.00	0.00	0.00	NaN

55968	NSFL1C	0.00	0.00	0.00	NaN
55969	C20orf24	0.04	0.00	0.00	0.23
56005	IL27	0.06	0.14	0.00	0.07
56033	BARX1	0.04	0.00	0.05	-0.03
56034	PDGFC	0.00	0.14	0.00	-0.04
56100	PCDHGB6	0.00	0.00	0.00	NaN
56101	PCDHGB5	0.00	0.00	0.00	NaN
56105	PCDHGA11	0.00	0.00	0.00	NaN
56106	PCDHGA10	0.00	0.00	0.00	NaN
56107	PCDHGA9	0.00	0.00	0.00	NaN
56112	PCDHGA3	0.00	0.00	0.00	NaN
56114	PCDHGA1	0.00	0.00	0.00	NaN
56123	PCDHB13	0.00	0.00	0.00	NaN
56124	PCDHB12	0.00	0.00	0.00	NaN
56125	PCDHB11	0.00	0.00	0.00	NaN
56128	PCDHB8	0.00	0.00	0.00	NaN
56130	PCDHB6	0.00	0.00	0.00	NaN
56132	PCDHB3	0.00	0.00	0.00	NaN
56137	PCDHA12	0.00	0.00	0.00	NaN
56139	PCDHA10	0.00	0.00	0.00	NaN
56142	PCDHA6	0.00	0.00	0.00	NaN
56143	PCDHA5	0.00	0.00	0.00	NaN
56145	PCDHA3	0.00	0.00	0.00	NaN
56146	PCDHA2	0.00	0.00	0.00	NaN
56154	TEX15	0.04	0.00	0.00	-0.05
56155	TEX14	0.04	0.00	0.05	0.26
56158	TEX12	0.02	0.00	0.00	0.16
56163	RNF17	0.06	0.00	0.00	0.14
56165	TDRD1	0.04	0.14	0.05	-0.11
56171	DNAH7	0.00	0.00	0.05	-0.09
56172	ANKH	0.00	0.00	0.05	0.01
56242	ZNF253	0.12	0.00	0.15	-0.01
56244	BTNL2	0.02	0.00	0.05	-0.02
56245	C21orf62	0.00	0.00	0.00	NaN
56253	CRTAM	0.00	0.00	0.00	NaN
56288	PARD3	0.04	0.29	0.25	0.19
56302	TRPV5	0.00	0.14	0.05	0.13
56340	PPP4R2	0.16	0.14	0.30	0.04
56344	CABP5	0.00	0.00	0.00	NaN
56547	MMP26	0.00	0.00	0.05	0.00
56604	TUBB4Q	0.02	0.00	0.05	-0.05
56606	SLC2A9	0.02	0.00	0.00	-0.01
56617	SERF1B	0.06	0.00	0.00	0.15
56647	BCCIP	0.02	0.00	0.00	-0.05
56648	EIF5A2	0.02	0.00	0.00	-0.05
56652	C10orf2	0.00	0.00	0.00	NaN
56656	OR2S2	0.02	0.00	0.00	0.06

56659	KCNK13	0.00	0.00	0.00	NaN
56660	KCNK12	0.00	0.00	0.00	NaN
56672	C11orf17	0.00	0.00	0.00	NaN
56673	C11orf16	0.00	0.00	0.00	NaN
56676	ASCL3	0.00	0.00	0.00	NaN
56683	C21orf59	0.00	0.00	0.00	NaN
56731	SLC2A4RG	0.08	0.00	0.05	0.16
56848	SPHK2	0.06	0.00	0.05	-0.03
56886	UGCGL1	0.00	0.00	0.10	0.17
56890	MDM1	0.08	0.14	0.05	0.49
56892	C8orf4	0.14	0.00	0.10	-0.01
56894	AGPAT3	0.02	0.00	0.00	0.06
56903	PAPOLB	0.00	0.00	0.00	NaN
56904	SH3GLB2	0.00	0.00	0.00	NaN
56910	STARD7	0.04	0.00	0.05	-0.19
56911	C21orf7	0.02	0.00	0.00	-0.02
56913	C1GALT1	0.00	0.00	0.00	NaN
56914	OTOR	0.00	0.00	0.10	0.05
56922	MCCC1	0.00	0.00	0.10	0.33
56924	PAK6	0.00	0.00	0.00	NaN
56925	LXN	0.02	0.00	0.10	0.12
56929	FEM1A	0.02	0.00	0.15	-0.12
56938	ARNTL2	0.00	0.00	0.00	NaN
56945	MRPS22	0.00	0.00	0.05	0.27
56953	NT5M	0.02	0.00	0.00	0.13
56954	NIT2	0.00	0.00	0.15	-0.05
56955	MEPE	0.02	0.00	0.00	-0.07
56978	PRDM8	0.02	0.00	0.00	-0.10
56979	PRDM9	0.02	0.14	0.05	-0.06
56980	PRDM10	0.02	0.00	0.00	0.02
56981	PRDM11	0.04	0.00	0.05	-0.07
56993	TOMM22	0.00	0.14	0.00	-0.12
56994	CHPT1	0.00	0.00	0.00	NaN
56997	CABC1	0.00	0.00	0.05	0.04
56998	CTNNBIP1	0.00	0.00	0.00	NaN
56999	ADAMTS9	0.04	0.00	0.00	-0.11
57016	AKR1B10	0.00	0.00	0.05	-0.06
57030	SLC17A7	0.02	0.00	0.05	-0.03
57047	PLSCR2	0.04	0.00	0.00	-0.08
57048	PLSCR3	0.02	0.00	0.00	-0.09
57053	CHRNA10	0.00	0.00	0.05	0.16
57060	PCBP4	0.00	0.00	0.10	-0.02
57062	DDX24	0.00	0.00	0.00	NaN
57084	SLC17A6	0.02	0.00	0.00	0.00
57088	PLSCR4	0.04	0.00	0.00	0.18
57096	RPGRIP1	0.02	0.00	0.00	-0.07
57102	C12orf4	0.02	0.00	0.00	-0.11

57103	C12orf5	0.02	0.00	0.00	-0.08
57110	HRASLS	0.02	0.00	0.00	0.04
57111	RAB25	0.04	0.14	0.05	-0.17
57119	SPINLW1	0.04	0.00	0.05	0.25
57122	NUP107	0.08	0.00	0.00	0.56
57127	RHBG	0.04	0.14	0.05	0.14
57139	RGL3	0.02	0.00	0.00	-0.09
57140	RNPEPL1	0.08	0.43	0.10	0.04
57142	RTN4	0.02	0.00	0.05	0.03
57144	PAK7	0.00	0.00	0.05	0.57
57148	KIAA1219	0.02	0.00	0.00	0.22
57154	SMURF1	0.02	0.00	0.00	0.11
57158	JPH2	0.02	0.00	0.10	0.11
57161	PELI2	0.04	0.00	0.10	-0.12
57162	PELI1	0.00	0.00	0.00	NaN
57172	CAMK1G	0.02	0.00	0.00	0.05
57192	MCOLN1	0.02	0.00	0.15	-0.09
57198	ATP8B2	0.04	0.00	0.15	-0.22
57205	ATP10D	0.00	0.29	0.10	0.07
57213	C13orf1	0.04	0.00	0.00	0.38
57214	KIAA1199	0.02	0.00	0.00	-0.04
57231	SNX14	0.00	0.00	0.05	-0.03
57282	SLC4A10	0.02	0.00	0.00	-0.09
57332	CBX8	0.00	0.14	0.00	-0.03
57336	ZNF287	0.04	0.00	0.05	-0.06
57337	SENP7	0.00	0.00	0.00	NaN
57338	JPH3	0.00	0.00	0.10	0.00
57343	ZNF304	0.00	0.00	0.00	NaN
57348	TTYH1	0.02	0.00	0.05	-0.05
57379	AICDA	0.02	0.00	0.15	-0.14
57403	RAB22A	0.08	0.00	0.00	0.60
57418	WDR18	0.02	0.00	0.05	0.10
57419	SLC24A3	0.04	0.00	0.05	0.11
57446	NDRG3	0.04	0.00	0.00	-0.01
57447	NDRG2	0.02	0.00	0.00	-0.04
57468	SLC12A5	0.02	0.00	0.00	0.50
57498	KIDINS220	0.00	0.00	0.00	NaN
57513	CASKIN2	0.02	0.14	0.00	0.49
57523	KIAA1305	0.02	0.00	0.05	-0.03
57535	KIAA1324	0.06	0.00	0.20	-0.02
57556	SEMA6A	0.02	0.00	0.00	-0.03
57586	SYT13	0.04	0.00	0.05	0.00
57591	MKL1	0.00	0.14	0.00	-0.18
57613	KIAA1467	0.04	0.00	0.00	0.07
57634	EP400	0.08	0.14	0.05	-0.07
57663	USP29	0.00	0.00	0.05	-0.01
57664	PLEKHA4	0.06	0.00	0.05	0.14

57698	KIAA1598	0.04	0.14	0.05	-0.13
57707	KIAA1609	0.00	0.00	0.00	NaN
57710	KIAA1614	0.04	0.00	0.10	0.05
57712	TRPM3	0.02	0.00	0.00	0.22
57715	SEMA4G	0.00	0.00	0.00	NaN
57716	PRX	0.00	0.00	0.05	0.14
57720	GPR107	0.04	0.00	0.00	-0.06
57731	SPTBN4	0.00	0.00	0.05	0.66
57763	ANKRA2	0.06	0.00	0.00	0.04
57786	RBAK	0.00	0.00	0.00	NaN
57799	RAB40C	0.18	0.00	0.10	-0.13
57804	POLD4	0.04	0.00	0.00	0.56
57817	HAMP	0.00	0.00	0.00	NaN
57834	CYP4F11	0.04	0.00	0.00	0.00
57835	SLC4A5	0.00	0.00	0.05	0.03
58155	PTBP2	0.02	0.00	0.00	-0.13
58157	NGB	0.00	0.00	0.00	NaN
58158	NEUROD4	0.02	0.00	0.05	-0.05
58189	WFDC1	0.00	0.00	0.00	NaN
58472	SQRDL	0.02	0.00	0.05	0.11
58488	PCTP	0.04	0.00	0.05	0.16
58494	JAM2	0.02	0.00	0.05	0.01
58503	PROL1	0.00	0.14	0.00	0.09
58529	MYOZ1	0.00	0.00	0.00	NaN
58533	SNX6	0.04	0.00	0.00	-0.03
58986	TMEM8	0.18	0.00	0.10	0.02
59067	IL21	0.00	0.14	0.05	-0.17
59269	HIVEP3	0.02	0.00	0.05	0.15
59286	UBL5	0.02	0.00	0.15	0.17
59307	SIGIRR	0.06	0.29	0.10	0.01
59335	PRDM12	0.04	0.00	0.00	-0.05
59336	PRDM13	0.02	0.00	0.05	-0.02
59338	PLEKHA1	0.04	0.00	0.00	-0.08
59340	HRH4	0.00	0.00	0.10	-0.09
59341	TRPV4	0.02	0.00	0.00	-0.11
59343	SENP2	0.02	0.14	0.00	-0.10
59344	ALOXE3	0.00	0.00	0.00	NaN
59347	FKSG2	0.12	0.14	0.05	-0.07
60314	C12orf10	0.00	0.00	0.00	NaN
60385	TSKS	0.02	0.00	0.05	0.03
60436	TGIF2	0.04	0.00	0.00	0.41
60467	BPESC1	0.00	0.00	0.00	NaN
60468	BACH2	0.00	0.00	0.05	-0.01
60482	SLC5A7	0.02	0.00	0.00	0.05
60488	MRPS35	0.00	0.00	0.00	NaN
60491	NIF3L1	0.00	0.00	0.05	-0.03
60496	AASDHPPT	0.04	0.00	0.00	0.05

60528	ELAC2	0.04	0.00	0.00	-0.04
60529	ALX4	0.00	0.00	0.05	0.02
60598	KCNK15	0.02	0.00	0.00	0.65
60681	FKBP10	0.02	0.86	0.05	0.23
60682	SMAP1	0.12	0.00	0.05	-0.12
63826	SRR	0.04	0.00	0.05	0.17
63827	BCAN	0.04	0.14	0.05	0.04
63875	MRPL17	0.00	0.00	0.00	NaN
63876	PKNOX2	0.02	0.00	0.00	-0.07
63916	ELMO2	0.02	0.00	0.10	0.04
63925	ZNF335	0.02	0.00	0.00	0.16
63926	ANKRD5	0.00	0.00	0.05	-0.05
63931	MRPS14	0.02	0.00	0.10	0.16
63943	FKBPL	0.02	0.00	0.05	0.50
63971	KIF13A	0.04	0.00	0.10	-0.02
63974	NEUROD6	0.00	0.00	0.00	NaN
63978	PRDM14	0.04	0.14	0.00	0.08
64063	PRSS22	0.02	0.00	0.00	-0.03
64064	OXCT2	0.02	0.00	0.05	0.01
64066	MMP27	0.00	0.00	0.00	NaN
64067	NPAS3	0.02	0.00	0.05	-0.02
64077	LHPP	0.04	0.00	0.05	-0.18
64078	SLC28A3	0.00	0.00	0.00	NaN
64083	GOLPH3	0.00	0.00	0.10	0.29
64084	CLSTN2	0.02	0.00	0.00	0.19
64087	MCCC2	0.06	0.00	0.00	0.07
64089	SNX16	0.06	0.00	0.15	0.11
64096	GFRA4	0.02	0.00	0.00	0.00
64100	ELSPBP1	0.00	0.00	0.00	NaN
64110	MAGEF1	0.02	0.14	0.00	-0.16
64122	FN3K	0.00	0.00	0.00	NaN
64132	XYLT2	0.10	0.00	0.10	0.19
64137	ABCG4	0.00	0.00	0.05	0.02
64173	SPATA1	0.04	0.00	0.05	-0.07
64211	LHX5	0.02	0.00	0.00	0.18
64223	GBL	0.18	0.00	0.10	-0.04
64231	MS4A6A	0.00	0.00	0.00	NaN
64232	MS4A5	0.00	0.00	0.00	NaN
64240	ABCG5	0.02	0.00	0.05	-0.15
64284	RAB17	0.00	0.00	0.00	NaN
64320	RNF25	0.04	0.00	0.00	0.14
64321	SOX17	0.00	0.00	0.00	NaN
64324	NSD1	0.00	0.00	0.00	NaN
64328	XPO4	0.18	0.00	0.20	-0.02
64374	SIL1	0.00	0.00	0.00	NaN
64377	CHST8	0.00	0.00	0.00	NaN
64386	MMP25	0.02	0.00	0.00	0.01

64397	ZFP106	0.04	0.00	0.00	-0.03
64398	MPP5	0.00	0.00	0.05	-0.14
64405	CDH22	0.02	0.00	0.00	0.06
64411	ARAP3	0.00	0.00	0.00	NaN
64446	DNAI2	0.04	0.00	0.00	0.19
64499	TPSB2	0.18	0.00	0.10	-0.09
64506	CPEB1	0.02	0.00	0.00	0.02
64577	ALDH8A1	0.00	0.00	0.00	NaN
64579	NDST4	0.02	0.00	0.05	-0.06
64600	PLA2G2F	0.02	0.00	0.05	0.02
64699	TMPRSS3	0.00	0.00	0.00	NaN
64708	COPS7B	0.02	0.00	0.15	0.16
64750	SMURF2	0.12	0.00	0.20	0.31
64783	RBM15	0.06	0.14	0.00	-0.14
64794	DDX31	0.04	0.00	0.00	-0.16
64816	CYP3A43	0.02	0.00	0.00	0.00
64834	ELOVL1	0.02	0.00	0.05	0.11
64849	SLC13A3	0.02	0.00	0.00	0.04
64850	AGXT2L1	0.08	0.00	0.00	-0.09
64858	DCLRE1B	0.02	0.00	0.05	0.34
64895	PAPOLG	0.00	0.00	0.05	0.03
64901	RANBP17	0.02	0.00	0.00	0.08
64919	BCL11B	0.00	0.00	0.00	NaN
64960	MRPS15	0.04	0.00	0.05	0.00
64963	MRPS11	0.00	0.00	0.00	NaN
64981	MRPL34	0.04	0.00	0.00	0.46
65003	MRPL11	0.04	0.00	0.05	0.10
65005	MRPL9	0.02	0.00	0.10	0.16
65009	NDRG4	0.04	0.00	0.05	-0.02
65010	SLC26A6	0.00	0.00	0.05	0.39
65012	SLC26A10	0.02	0.14	0.00	0.26
65018	PINK1	0.02	0.00	0.05	0.12
65080	MRPL44	0.00	0.00	0.00	NaN
65082	VPS33A	0.02	0.00	0.10	0.00
65258	MPPE1	0.04	0.00	0.10	0.08
65993	MRPS34	0.18	0.00	0.10	-0.05
66002	CYP4F12	0.04	0.00	0.00	-0.09
66035	SLC2A11	0.00	0.14	0.05	-0.20
78988	MRP63	0.18	0.00	0.20	-0.02
79054	TRPM8	0.02	0.00	0.00	-0.14
79058	ASPSCR1	0.00	0.00	0.00	NaN
79083	MLPH	0.00	0.00	0.00	NaN
79092	CARD14	0.00	0.14	0.00	0.11
79095	C9orf16	0.00	0.00	0.00	NaN
79133	C20orf7	0.00	0.14	0.00	-0.02
79144	C20orf149	0.08	0.00	0.05	0.20
79147	FKRP	0.00	0.00	0.05	-0.10

79148	MMP28	0.06	0.00	0.00	0.01
79187	FSD1	0.02	0.00	0.15	0.05
79369	B3GNT4	0.02	0.00	0.10	-0.09
79400	NOX5	0.00	0.00	0.15	0.15
79442	LRRC2	0.02	0.14	0.15	-0.06
79443	FYCO1	0.02	0.14	0.15	0.12
79444	BIRC7	0.08	0.00	0.05	0.02
79583	FLJ22167	0.00	0.00	0.00	NaN
79590	MRPL24	0.04	0.14	0.05	-0.04
79640	FLJ23584	0.00	0.14	0.00	-0.04
79661	NEIL1	0.00	0.00	0.05	-0.18
79667	FLJ13197	0.04	0.00	0.10	0.09
79716	NPEPL1	0.10	0.00	0.00	0.58
79719	FLJ11506	0.02	0.00	0.00	0.32
79723	SUV39H2	0.02	0.14	0.05	-0.08
79753	SNIP1	0.04	0.00	0.05	0.02
79754	ASB13	0.00	0.00	0.00	NaN
79767	ELMO3	0.02	0.00	0.00	0.07
79783	C7orf10	0.00	0.00	0.00	NaN
79800	ALS2CR8	0.00	0.00	0.00	NaN
79814	AGMAT	0.02	0.00	0.00	-0.02
79833	GEMIN6	0.02	0.00	0.05	0.05
79857	FLJ13224	0.02	0.00	0.00	0.01
79858	NEK11	0.02	0.00	0.00	0.14
79870	BAALC	0.12	0.00	0.25	0.31
79890	RIN3	0.00	0.00	0.05	0.03
79899	FLJ14213	0.02	0.00	0.00	0.02
79953	C20orf39	0.04	0.00	0.15	-0.06
80011	NIP30	0.00	0.00	0.00	NaN
80146	UXS1	0.02	0.00	0.10	-0.03
80157	FLJ21511	0.04	0.00	0.05	0.04
80196	RNF34	0.02	0.00	0.10	0.18
80198	MUS81	0.04	0.00	0.05	-0.09
80207	OPA3	0.00	0.00	0.05	0.36
80271	ITPKC	0.00	0.00	0.05	0.22
80307	FER1L4	0.06	0.14	0.05	0.32
80324	PUS1	0.08	0.14	0.05	-0.01
80328	ULBP2	0.02	0.00	0.05	-0.03
80329	ULBP1	0.02	0.00	0.05	-0.03
80351	TNKS2	0.02	0.00	0.05	0.03
80704	SLC19A3	0.02	0.00	0.15	-0.01
80705	TSGA10	0.02	0.00	0.10	-0.07
80737	C6orf27	0.02	0.00	0.05	0.12
80738	C6orf26	0.02	0.00	0.05	0.02
80739	C6orf25	0.02	0.00	0.05	-0.19
80765	STARD5	0.02	0.00	0.00	-0.05
80781	COL18A1	0.00	0.00	0.05	-0.20

80830	APOL6	0.00	0.29	0.05	-0.12
80831	APOL5	0.00	0.29	0.05	-0.13
80833	APOL3	0.00	0.14	0.00	0.14
80895	ILKAP	0.08	0.43	0.10	-0.15
80975	TMPRSS5	0.00	0.00	0.00	NaN
81025	GJA10	0.00	0.00	0.05	0.01
81027	TUBB1	0.12	0.00	0.00	-0.08
81029	WNT5B	0.08	0.14	0.10	0.07
81031	SLC2A10	0.02	0.00	0.00	0.03
81035	COLEC12	0.02	0.00	0.05	-0.14
81285	OR51E2	0.00	0.00	0.05	-0.06
81490	PTDSS2	0.06	0.29	0.10	0.05
81491	GPR63	0.08	0.00	0.00	0.03
81492	RSHL1	0.00	0.00	0.05	-0.09
81539	SLC38A1	0.04	0.00	0.05	0.05
81543	LRRC3	0.02	0.00	0.00	-0.17
81551	STMN4	0.02	0.00	0.00	0.08
81563	C1orf21	0.00	0.00	0.00	NaN
81578	COL21A1	0.06	0.14	0.05	-0.09
81620	CDT1	0.00	0.00	0.10	0.02
81622	UNC93B1	0.04	0.00	0.05	0.19
81624	DIAPH3	0.08	0.14	0.00	-0.14
81626	C1orf14	0.02	0.00	0.00	0.00
81627	C1orf25	0.00	0.00	0.05	-0.18
81691	LOC81691	0.02	0.14	0.00	0.23
81693	AMN	0.00	0.00	0.05	0.05
81696	OR5V1	0.04	0.14	0.05	-0.05
81698	C15orf5	0.00	0.00	0.05	0.12
81792	ADAMTS12	0.00	0.00	0.10	-0.12
81797	OR12D3	0.04	0.14	0.05	0.08
81831	NETO2	0.43	0.29	0.15	-0.12
81833	SPACA1	0.00	0.00	0.05	0.19
81854	MGC3771	0.02	0.00	0.00	-0.04
81876	RAB1B	0.04	0.00	0.05	0.23
83442	SH3BGRL3	0.02	0.00	0.05	-0.02
83452	RAB33B	0.00	0.00	0.05	0.04
83481	EPPK1	0.10	0.00	0.15	0.18
83482	SCRT1	0.10	0.00	0.15	-0.02
83483	PLVAP	0.04	0.00	0.00	-0.01
83660	TLN2	0.00	0.00	0.00	NaN
83737	ITCH	0.02	0.00	0.05	-0.10
83988	NCALD	0.12	0.00	0.25	0.18
83990	BRIP1	0.10	0.00	0.05	0.32
84084	RAB6C	0.00	0.00	0.10	0.04
84099	ID2B	0.04	0.00	0.00	-0.14
84107	ZIC4	0.04	0.00	0.00	-0.04
84148	MYST1	0.02	0.00	0.00	-0.12

84561	SLC12A8	0.00	0.00	0.10	0.12
84612	PARD6B	0.10	0.00	0.00	0.44
84658	EMR3	0.00	0.00	0.05	-0.22
84789	MGC2889	0.02	0.00	0.00	-0.01
85352	KIAA1644	0.00	0.14	0.05	-0.15
85359	DGCR6L	0.06	0.14	0.15	0.16
85363	TRIM5	0.02	0.00	0.00	0.03
85459	KIAA1731	0.00	0.00	0.00	NaN
85477	SCIN	0.00	0.00	0.00	NaN
86722	GREB1	0.00	0.00	0.00	NaN
89781	HPS4	0.00	0.14	0.05	-0.08
89870	TRIM15	0.04	0.14	0.05	0.21
89874	SLC25A21	0.04	0.00	0.00	-0.02
89910	UBE3B	0.02	0.00	0.00	-0.09
91754	NEK9	0.00	0.00	0.00	NaN
91949	COG7	0.02	0.14	0.00	-0.13
91977	MYOZ3	0.00	0.14	0.00	0.00
94009	SERHL	0.00	0.14	0.05	0.12
94025	MUC16	0.02	0.00	0.15	0.23
94104	C21orf66	0.00	0.00	0.00	NaN
112399	EGLN3	0.04	0.00	0.00	-0.08
113878	DTX2	0.00	0.00	0.00	NaN
114049	WBSCR22	0.00	0.00	0.00	NaN
114088	TRIM9	0.02	0.00	0.00	0.00
114625	ERMAP	0.02	0.00	0.05	0.44
114876	OSBPL1A	0.00	0.00	0.10	0.18
114881	OSBPL7	0.00	0.00	0.00	NaN
114882	OSBPL8	0.02	0.00	0.00	-0.06
114883	OSBPL9	0.02	0.00	0.00	0.11
114884	OSBPL10	0.06	0.00	0.05	0.17
114885	OSBPL11	0.00	0.00	0.10	0.23
114897	C1QTNF1	0.02	0.14	0.00	-0.04
114899	C1QTNF3	0.02	0.14	0.05	-0.07
116039	OSR2	0.06	0.14	0.05	-0.03
116832	RPL39L	0.02	0.14	0.00	0.00
117246	FTSJ3	0.12	0.00	0.20	0.39
117247	SLC16A10	0.02	0.00	0.10	-0.02
118433	RPL23AP7	0.00	0.00	0.10	0.16
140467	ZNF358	0.02	0.00	0.15	0.05
140545	RNF32	0.00	0.00	0.05	-0.05
140801	RPL10L	0.00	0.00	0.10	-0.16
140803	TRPM6	0.00	0.00	0.00	NaN
171558	PTCRA	0.06	0.00	0.05	0.32
192683	SCAMP5	0.00	0.00	0.05	-0.13

Neve et al. dataset

Gene_no	Gene_name	Average_CNA_Score_ER	Average_CNA_Score_HER2	Average_CNA_Score_TN	Overall_Correlation
2	A2M	-0.09	0.00	0.00	0.05
9	NAT1	0.00	-0.13	-0.05	0.18
10	NAT2	0.00	-0.13	-0.05	0.02
12	SERPINA3	0.00	0.00	0.00	NaN
13	AADAC	0.00	0.13	0.00	-0.06
14	AAMP	0.00	0.00	0.00	NaN
15	AANAT	0.00	0.13	0.00	-0.04
16	AARS	-0.09	-0.13	0.05	0.53
18	ABAT	0.00	0.00	0.00	NaN
19	ABCA1	0.00	0.00	0.00	NaN
20	ABCA2	0.00	0.00	0.00	NaN
21	ABCA3	0.00	0.13	0.00	0.03
23	ABCF1	0.00	0.00	0.00	NaN
24	ABCA4	0.09	0.13	0.00	0.38
25	ABL1	0.00	0.00	0.00	NaN
26	ABP1	0.00	0.00	0.00	0.09
27	ABL2	0.00	0.00	0.00	NaN
28	ABO	0.00	0.00	0.00	NaN
29	ABR	0.00	-0.13	0.00	-0.11
30	ACAA1	0.00	0.25	0.00	0.70
31	ACACA	0.00	0.00	0.00	0.49
32	ACACB	0.00	0.00	0.00	NaN
33	ACADL	0.00	0.00	0.00	NaN
34	ACADM	0.00	0.00	0.00	NaN
35	ACADS	0.00	-0.13	0.00	-0.05
36	ACADSB	0.09	0.00	-0.05	0.66
37	ACADVL	0.00	0.00	0.00	NaN
38	ACAT1	0.00	0.00	0.00	NaN
39	ACAT2	0.00	0.00	0.05	0.17
40	ACCN1	0.00	0.00	0.00	0.12
41	ACCN2	0.00	0.00	0.00	NaN
43	ACHE	0.18	0.00	0.00	0.13
47	ACLY	0.00	0.13	0.00	0.32
48	ACO1	-0.09	0.00	0.00	0.44
49	ACR	0.00	-0.25	-0.05	-0.29
50	ACO2	0.00	0.00	0.05	0.29
51	ACOX1	0.00	0.13	0.05	0.37
52	ACP1	0.00	0.00	0.00	NaN
53	ACP2	0.00	0.00	0.00	NaN
54	ACP5	0.00	0.00	0.00	NaN
55	ACPP	0.00	0.00	0.00	NaN
56	ACRV1	-0.09	0.00	-0.10	-0.06
58	ACTA1	0.09	0.00	0.00	-0.06

59	ACTA2	0.09	0.00	0.00	-0.06
60	ACTB	0.00	0.00	0.10	-0.09
70	ACTC	0.00	0.00	0.00	NaN
71	ACTG1	0.00	0.00	0.00	NaN
72	ACTG2	0.00	0.00	0.00	NaN
81	ACTN4	0.00	0.00	0.00	NaN
86	BAF53A	0.09	0.00	0.00	0.16
87	ACTN1	0.00	0.00	0.00	NaN
88	ACTN2	0.18	0.13	0.00	0.27
89	ACTN3	0.00	0.00	0.05	-0.01
90	ACVR1	0.00	0.00	-0.05	0.27
91	ACVR1B	0.00	0.00	0.00	NaN
92	ACVR2	0.00	0.00	0.00	NaN
93	ACVR2B	0.00	0.00	0.00	NaN
94	ACVRL1	0.00	0.00	0.00	NaN
95	ACY1	-0.09	0.00	-0.10	0.41
97	ACYP1	0.00	0.00	0.00	NaN
98	ACYP2	0.09	0.00	-0.05	0.08
100	ADA	0.09	0.13	0.00	0.02
101	ADAM8	0.00	0.00	-0.10	0.23
102	ADAM10	0.09	0.00	0.05	0.37
103	ADAR	0.09	0.25	0.00	0.39
104	ADARB1	0.00	0.00	0.00	NaN
107	ADCY1	0.00	0.00	0.00	NaN
108	ADCY2	0.00	0.00	0.05	0.00
109	ADCY3	0.09	0.00	0.00	-0.16
112	ADCY6	0.00	0.00	0.00	NaN
113	ADCY7	-0.09	0.00	-0.05	0.19
114	ADCY8	0.00	-0.13	0.15	0.11
115	ADCY9	0.00	0.00	0.00	NaN
116	ADCYAP1	0.00	0.00	0.00	NaN
117	ADCYAP1R1	0.00	0.13	0.00	-0.02
118	ADD1	0.00	-0.13	-0.15	0.56
119	ADD2	0.00	0.00	0.00	NaN
120	ADD3	0.00	0.00	-0.05	0.24
123	ADFP	0.00	-0.25	-0.10	0.31
124	ADH1A	0.00	0.00	0.00	NaN
125	ADH1B	0.00	0.00	0.00	NaN
126	ADH1C	0.00	0.00	0.00	NaN
128	ADH5	0.00	0.00	0.00	NaN
130	ADH6	0.00	0.00	0.00	NaN
131	ADH7	0.00	0.00	0.00	NaN
132	ADK	0.09	0.00	0.00	0.42
133	ADM	0.00	0.00	0.00	NaN
134	ADORA1	0.00	0.13	0.00	0.08
135	ADORA2A	0.00	0.00	0.00	NaN
136	ADORA2B	0.09	0.00	0.00	-0.14

140	ADORA3	0.09	0.00	0.00	0.11
141	ADPRH	0.00	0.00	0.00	NaN
142	ADPRT	0.09	0.13	0.00	0.08
143	ADPRTL1	0.00	-0.13	-0.15	0.44
146	ADRA1D	0.00	0.00	0.00	0.09
147	ADRA1B	0.00	0.00	0.00	NaN
150	ADRA2A	0.00	0.00	0.00	0.01
151	ADRA2B	0.00	0.00	0.00	NaN
152	ADRA2C	0.00	-0.13	-0.15	0.00
153	ADRB1	0.09	0.00	-0.05	-0.10
154	ADRB2	0.00	0.00	0.00	NaN
155	ADRB3	0.55	-0.13	-0.05	-0.09
156	ADRBK1	0.45	0.00	0.05	0.59
157	ADRBK2	0.00	0.00	-0.05	-0.12
158	ADSL	0.00	0.00	0.50	0.43
159	ADSS	0.00	0.13	0.00	0.31
161	AP2A2	-0.18	-0.13	-0.05	0.50
162	AP1B1	-0.09	0.00	0.05	0.15
163	AP2B1	0.00	0.00	0.00	0.48
164	AP1G1	0.00	0.00	0.05	0.49
165	AEBP1	0.00	0.00	0.00	NaN
166	AES	0.00	0.00	0.00	NaN
167	AEGL1	0.00	0.00	0.00	NaN
173	AFM	0.00	0.00	0.00	NaN
174	AFP	0.00	0.00	0.00	NaN
175	AGA	0.00	0.00	-0.10	0.37
176	AGC1	0.00	0.00	-0.05	0.11
177	AGER	0.00	0.00	0.00	-0.01
178	AGL	0.00	0.00	0.00	NaN
181	AGRP	-0.09	0.00	0.05	-0.16
182	JAG1	0.00	0.00	0.05	0.10
183	AGT	0.09	0.00	0.00	-0.04
185	AGTR1	0.00	0.00	0.00	NaN
187	AGTRL1	0.00	0.00	0.00	NaN
189	AGXT	0.00	0.00	-0.05	0.18
191	AHCY	0.00	-0.13	0.00	0.37
196	AHR	0.00	0.13	0.00	-0.09
197	AHSG	0.09	0.00	0.05	0.10
199	AIF1	0.00	0.00	0.00	NaN
202	AIM1	0.00	0.00	0.10	0.47
203	AK1	-0.09	0.00	0.00	0.19
204	AK2	0.00	0.00	0.00	NaN
205	AK3	0.00	0.50	0.00	0.18
207	AKT1	0.00	0.00	0.00	NaN
208	AKT2	0.00	0.00	0.00	NaN
210	ALAD	0.00	0.00	0.00	NaN
211	ALAS1	-0.09	0.00	-0.10	0.19

214	ALCAM	0.09	0.00	0.00	0.20
215	ABCD1	0.00	0.00	0.00	NaN
216	ALDH1A1	0.00	0.00	-0.05	-0.21
217	ALDH2	0.00	0.00	0.00	NaN
218	ALDH3A1	0.00	-0.13	0.00	0.03
219	ALDH1B1	-0.09	0.00	0.00	0.20
220	ALDH1A3	0.00	0.00	0.00	NaN
221	ALDH3B1	0.18	0.00	0.05	0.33
222	ALDH3B2	0.27	0.00	0.05	0.28
223	ALDH9A1	0.00	0.00	0.00	NaN
224	ALDH3A2	0.00	-0.13	0.00	0.24
225	ABCD2	0.00	0.00	-0.05	0.16
226	ALDOA	0.00	0.00	0.00	NaN
229	ALDOB	0.00	0.00	0.00	NaN
230	ALDOC	0.00	0.38	0.00	0.64
231	AKR1B1	0.00	0.00	0.00	NaN
238	ALK	0.00	0.00	0.00	NaN
239	ALOX12	0.00	0.00	0.00	NaN
240	ALOX5	0.00	0.00	0.00	NaN
241	ALOX5AP	0.00	0.00	0.10	-0.10
242	ALOX12B	0.00	0.00	0.00	NaN
244	ANXA8	0.00	0.00	0.00	NaN
246	ALOX15	0.00	0.00	0.00	NaN
247	ALOX15B	0.00	0.00	0.00	NaN
248	ALPI	0.00	0.00	0.00	NaN
249	ALPL	-0.09	0.00	0.00	0.05
250	ALPP	0.00	0.00	0.00	NaN
251	ALPPL2	0.00	0.00	0.00	NaN
257	ALX3	0.09	0.00	0.00	0.05
258	AMBN	0.00	0.00	0.05	-0.08
259	AMBP	0.00	0.00	0.00	NaN
262	AMD1	0.00	0.00	0.05	0.19
267	AMFR	0.00	0.00	0.00	NaN
268	AMH	0.00	0.00	0.50	0.33
269	AMHR2	0.00	0.00	0.00	NaN
270	AMPD1	0.18	0.00	0.10	0.24
271	AMPD2	0.09	0.00	0.05	0.52
272	AMPD3	0.00	0.00	0.00	NaN
273	AMPH	0.00	0.00	0.00	NaN
274	BIN1	0.00	0.00	-0.05	0.12
275	AMT	-0.09	0.00	-0.05	0.13
276	AMY1A	0.00	0.13	0.00	-0.05
283	ANG	0.00	0.00	0.00	NaN
284	ANGPT1	0.18	0.13	0.00	0.12
285	ANGPT2	-0.09	-0.25	-0.10	0.23
286	ANK1	0.45	-0.13	-0.05	0.34
287	ANK2	0.00	0.00	0.00	NaN

288	ANK3	0.00	0.00	0.05	0.00
290	ANPEP	0.00	0.00	0.00	NaN
291	SLC25A4	0.00	0.00	-0.05	0.16
301	ANXA1	0.00	0.00	-0.05	-0.14
302	ANXA2	0.00	0.00	0.00	NaN
306	ANXA3	0.00	0.00	0.00	NaN
307	ANXA4	0.00	0.00	0.00	NaN
308	ANXA5	0.00	0.00	0.00	NaN
309	ANXA6	0.00	0.00	0.00	NaN
310	ANXA7	0.09	0.00	0.00	0.38
311	ANXA11	0.09	0.00	0.00	0.23
312	ANXA13	0.18	0.25	0.00	0.33
313	AOAH	0.00	0.00	0.00	NaN
314	AOC2	0.00	-0.13	0.00	0.06
316	AOX1	0.00	0.00	0.00	NaN
317	APAF1	0.00	0.13	0.00	0.19
318	NUDT2	-0.09	0.13	0.10	0.65
319	APOF	0.00	0.00	0.00	NaN
320	APBA1	0.00	0.00	0.00	NaN
321	APBA2	0.00	-0.13	0.00	0.15
322	APBB1	0.00	-0.13	0.00	0.15
323	APBB2	0.00	0.00	0.05	0.03
324	APC	-0.09	0.00	0.05	0.14
325	APCS	0.00	0.00	0.00	NaN
326	AIRE	0.00	0.00	0.00	NaN
327	APEH	-0.09	0.00	-0.05	0.41
328	APEX	0.00	0.50	0.00	0.04
329	BIRC2	0.00	0.13	0.00	0.17
330	BIRC3	0.00	0.13	0.00	-0.03
332	BIRC5	0.00	0.13	0.00	0.19
333	APLP1	0.00	0.00	0.00	NaN
334	APLP2	0.00	0.00	0.00	0.35
335	APOA1	-0.09	0.00	0.00	-0.02
336	APOA2	0.00	0.00	0.00	NaN
337	APOA4	-0.09	0.00	0.00	-0.05
338	APOB	0.09	0.00	-0.05	0.15
339	APOBEC1	-0.09	0.00	0.00	-0.10
341	APOC1	0.09	0.00	0.00	-0.08
343	AQP8	0.00	0.00	0.00	NaN
344	APOC2	0.09	0.00	0.00	0.15
345	APOC3	-0.09	0.00	0.00	-0.06
346	APOC4	0.09	0.00	0.00	-0.17
347	APOD	0.18	0.13	0.20	-0.11
348	APOE	0.09	0.00	0.00	0.28
350	APOH	0.18	0.13	0.00	-0.42
351	APP	0.00	0.00	0.00	NaN
353	APRT	0.09	-0.13	-0.05	0.09

354	KLK3	0.00	0.00	0.00	NaN
355	TNFRSF6	0.09	0.00	0.00	0.11
356	TNFSF6	0.00	0.00	0.00	NaN
358	AQP1	0.00	0.13	0.00	0.45
359	AQP2	0.00	0.00	0.00	NaN
360	AQP3	-0.09	0.00	0.10	-0.14
361	AQP4	0.00	0.00	-0.15	0.26
362	AQP5	0.00	0.00	0.00	NaN
363	AQP6	0.00	0.00	0.00	NaN
364	AQP7	0.00	0.00	0.50	0.03
368	ABCC6	0.00	0.00	0.00	NaN
372	ARCN1	0.00	0.00	0.00	NaN
373	ARFD1	0.00	0.00	0.00	NaN
374	AREG	0.00	0.00	0.00	NaN
375	ARF1	0.09	0.00	0.00	0.40
377	ARF3	0.00	0.00	0.00	NaN
378	ARF4	0.00	0.00	-0.10	0.18
379	ARF4L	0.00	0.00	0.00	-0.06
381	ARF5	0.00	0.25	0.00	0.36
382	ARF6	0.00	0.00	0.00	NaN
383	ARG1	0.00	0.00	0.00	NaN
384	ARG2	0.00	0.00	0.00	NaN
387	ARHA	-0.09	0.00	-0.05	0.36
388	ARHB	0.09	0.00	-0.05	0.36
389	ARHC	0.09	0.00	0.05	0.49
390	ARHE	0.00	0.00	0.00	NaN
391	ARHG	0.00	-0.13	0.00	0.27
392	ARHGAP1	0.00	0.00	0.00	NaN
394	ARHGAP5	0.00	0.00	0.00	NaN
396	ARHGDIA	0.00	0.00	0.00	NaN
397	ARHGDIB	-0.09	0.00	0.00	0.09
398	ARHGDIG	0.00	0.00	-0.05	0.16
399	ARHH	0.00	0.00	0.00	NaN
400	ARL1	0.00	0.00	0.00	NaN
401	ARIX	0.00	0.00	0.00	-0.05
402	ARL2	0.00	0.00	0.00	NaN
403	ARL3	0.00	-0.13	-0.05	0.42
405	ARNT	0.00	0.00	0.05	0.32
406	ARNTL	0.00	0.00	0.00	NaN
408	ARRB1	0.09	0.00	0.00	0.57
409	ARRB2	0.00	0.00	0.00	NaN
410	ARSA	0.00	-0.25	0.00	-0.14
411	ARSB	0.00	0.00	0.00	NaN
417	ART1	0.00	-0.13	0.00	0.00
419	ART3	0.00	0.00	0.00	NaN
420	DO	0.00	0.00	0.00	-0.16
421	ARVCF	-0.09	0.00	0.00	-0.19

427	ASAH	0.00	-0.13	-0.05	0.26
429	ASCL1	0.00	0.00	0.00	NaN
430	ASCL2	0.00	-0.13	0.00	-0.11
432	ASGR1	0.00	0.00	0.00	NaN
433	ASGR2	0.00	0.00	0.00	NaN
434	ASIP	0.00	-0.13	0.00	-0.03
439	ASNA1	0.00	0.00	0.00	NaN
440	ASNS	0.18	0.00	0.00	0.37
443	ASPA	0.00	0.00	0.00	NaN
444	ASPH	0.00	0.00	0.00	NaN
445	ASS	0.00	0.00	0.00	NaN
460	ASTN	0.00	0.00	0.00	NaN
462	SERPINC1	0.00	0.00	0.05	-0.26
463	ATBF1	0.00	0.00	0.05	0.21
466	ATF1	0.00	0.00	0.00	NaN
467	ATF3	0.00	0.00	0.00	NaN
468	ATF4	-0.09	0.00	0.05	0.32
471	ATIC	0.00	0.00	0.00	NaN
472	ATM	0.00	0.00	0.00	NaN
473	RERE	0.00	0.00	0.00	NaN
474	ATOH1	0.00	0.00	0.00	NaN
475	ATOX1	0.00	0.00	0.00	NaN
476	ATP1A1	0.09	0.00	0.10	0.15
477	ATP1A2	0.00	0.00	0.00	NaN
478	ATP1A3	0.00	0.00	0.00	-0.09
479	ATP12A	0.00	-0.13	-0.15	0.08
481	ATP1B1	0.00	0.00	-0.05	0.50
482	ATP1B2	0.00	0.00	0.05	-0.04
483	ATP1B3	0.00	0.00	0.00	NaN
486	FXYD2	-0.09	0.00	0.00	0.06
487	ATP2A1	0.00	0.00	0.00	NaN
488	ATP2A2	0.00	0.00	0.00	NaN
489	ATP2A3	0.00	0.00	0.00	NaN
490	ATP2B1	0.09	0.00	-0.05	0.13
491	ATP2B2	0.00	0.00	0.00	NaN
493	ATP2B4	0.00	0.13	0.00	-0.25
495	ATP4A	0.00	0.00	0.00	NaN
496	ATP4B	-0.09	-0.13	-0.05	0.13
498	ATP5A1	-0.09	-0.13	-0.20	0.71
501	ALDH7A1	0.00	0.00	0.00	NaN
506	ATP5B	0.00	0.00	0.00	NaN
513	ATP5D	0.00	0.00	0.00	NaN
514	ATP5E	0.18	0.25	0.00	0.49
515	ATP5F1	0.09	0.00	0.00	0.42
516	ATP5G1	0.00	0.38	0.00	0.44
517	ATP5G2	0.00	0.00	0.00	NaN
518	ATP5G3	0.00	0.00	0.00	NaN

521	ATP5I	0.00	-0.13	-0.10	0.11
522	ATP5J	0.00	0.00	0.00	NaN
523	ATP6V1A1	0.00	0.00	0.00	NaN
525	ATP6V1B1	0.00	0.00	0.05	-0.11
526	ATP6V1B2	-0.09	-0.38	-0.10	0.44
527	ATP6V0C	0.00	0.00	0.00	NaN
528	ATP6V1C1	0.18	0.00	0.00	0.09
529	ATP6V1E	0.00	0.00	0.00	0.55
533	ATP6V0B	0.00	0.00	0.00	NaN
534	ATP6V1G2	0.00	0.00	0.00	NaN
535	ATP6V0A1	0.00	-0.13	0.00	0.13
539	ATP5O	0.00	0.00	0.00	NaN
540	ATP7B	0.00	0.00	0.00	NaN
545	ATR	0.00	0.00	-0.05	0.33
546	ATRX	0.00	0.00	0.00	NaN
547	ATSV	0.00	0.00	-0.05	0.12
549	AUH	0.00	0.00	0.00	NaN
550	AUP1	0.00	0.00	0.00	NaN
551	AVP	0.00	0.00	0.00	NaN
552	AVPR1A	0.09	0.00	0.00	0.39
553	AVPR1B	0.00	0.13	0.00	-0.02
558	AXL	0.00	0.00	-0.05	-0.02
563	AZGP1	0.18	0.00	0.00	-0.17
566	AZU1	0.00	0.00	0.00	NaN
567	B2M	0.00	0.00	-0.05	0.87
570	BAAT	0.00	0.00	0.00	NaN
571	BACH1	0.00	0.00	0.00	NaN
572	BAD	0.00	0.00	0.00	NaN
573	BAG1	-0.09	0.00	0.05	0.10
574	BAGE	0.00	0.00	-0.10	0.00
575	BAI1	0.18	0.13	0.10	0.13
576	BAI2	0.00	0.00	0.00	NaN
577	BAI3	0.00	0.00	0.00	NaN
578	BAK1	0.00	0.00	0.00	NaN
579	BAPX1	0.00	0.00	-0.05	-0.03
580	BARD1	0.00	0.00	0.00	NaN
581	BAX	0.09	0.00	0.00	-0.05
585	BBS4	0.00	0.00	0.00	NaN
586	BCAT1	0.00	0.00	0.00	NaN
587	BCAT2	1.00	0.00	0.00	-0.03
590	BCHE	0.00	0.00	0.00	NaN
593	BCKDHA	0.00	0.00	-0.05	0.15
594	BCKDHB	0.00	0.00	0.00	NaN
595	CCND1	0.55	0.25	0.00	0.53
596	BCL2	-0.09	0.00	-0.15	0.19
597	BCL2A1	0.00	0.00	0.00	NaN
598	BCL2L1	0.00	0.00	0.05	0.00

599	BCL2L2	0.00	0.00	0.00	NaN
602	BCL3	0.09	0.00	0.00	0.18
604	BCL6	0.00	0.00	0.10	0.01
605	BCL7A	0.00	-0.13	0.00	0.05
607	BCL9	0.00	0.00	0.05	0.32
608	TNFRSF17	0.00	0.00	0.00	NaN
610	HCN2	0.00	0.00	0.00	-0.03
611	OPN1SW	0.00	0.25	0.00	0.02
613	BCR	0.00	0.00	0.00	NaN
617	BCS1L	0.00	0.00	0.00	NaN
622	BDH	0.18	0.00	0.00	0.57
623	BDKRB1	0.00	0.00	0.00	NaN
624	BDKRB2	0.00	0.00	0.00	NaN
627	BDNF	0.00	0.00	0.00	NaN
629	BF	0.00	0.00	0.00	NaN
631	BFSP1	0.00	0.00	0.00	NaN
632	BGLAP	0.00	1.00	0.00	0.10
634	CEACAM1	0.09	0.00	-0.05	0.18
635	BHMT	0.00	0.00	0.00	NaN
636	BICD1	0.00	0.00	0.00	NaN
637	BID	0.00	0.00	0.00	0.29
638	BIK	0.00	0.00	0.00	NaN
640	BLK	-0.09	-0.50	-0.15	-0.15
641	BLM	0.00	0.00	0.00	NaN
642	BLMH	0.00	0.38	0.00	0.16
643	BLR1	0.00	0.00	0.00	NaN
644	BLVRA	0.00	0.00	0.00	NaN
645	BLVRB	0.00	0.00	0.00	NaN
648	BMI1	-0.09	0.00	0.05	0.05
649	BMP1	0.00	0.50	0.50	0.27
650	BMP2	0.00	0.00	0.00	NaN
651	BMP3	0.00	0.00	0.00	NaN
652	BMP4	0.00	0.00	0.05	0.26
653	BMP5	0.00	0.00	0.00	NaN
654	BMP6	0.00	0.00	0.00	NaN
655	BMP7	0.27	0.50	0.00	0.65
656	BMP8	0.00	0.00	0.00	NaN
657	BMPR1A	0.09	0.00	-0.05	0.61
658	BMPR1B	0.00	0.00	0.00	NaN
659	BMPR2	0.00	0.00	0.00	NaN
661	BN51T	-0.09	-0.25	-0.05	0.34
662	BNIP1	0.00	0.00	0.00	NaN
663	BNIP2	0.00	0.00	0.05	0.11
664	BNIP3	0.00	0.00	-0.05	0.21
665	BNIP3L	-0.09	-0.25	-0.05	0.18
666	BOK	0.00	0.00	-0.05	0.02
667	BPAG1	0.00	0.13	0.00	0.00

668	FOXL2	0.00	0.00	0.00	NaN
669	BPGM	0.00	0.00	0.00	NaN
670	BPHL	0.00	0.00	-0.05	0.27
671	BPI	0.00	-0.13	0.00	-0.14
672	BRCA1	0.00	-0.13	0.00	0.08
673	BRAF	0.00	0.00	0.00	NaN
675	BRCA2	0.00	0.00	0.00	NaN
676	BRDT	0.00	0.00	0.00	NaN
677	ZFP36L1	0.00	0.00	0.00	NaN
678	ZFP36L2	0.50	0.00	0.50	-0.17
682	BSG	0.00	0.00	-0.05	0.18
683	BST1	0.00	0.00	-0.10	-0.07
684	BST2	-0.09	0.00	0.00	0.08
685	BTC	0.00	0.00	0.00	NaN
686	BTD	0.00	0.00	0.05	0.51
687	BTEB1	0.00	0.00	0.00	NaN
688	KLF5	0.00	0.50	0.00	0.13
689	BTF3	0.00	0.00	0.00	NaN
690	BTF3L1	0.00	0.50	0.50	0.22
694	BTG1	0.00	0.00	0.00	NaN
696	BTN1A1	0.00	0.00	0.00	NaN
699	BUB1	0.00	0.00	0.00	NaN
701	BUB1B	0.00	0.00	0.00	NaN
705	BYSL	0.00	0.00	0.05	0.30
706	BZRP	0.00	0.00	0.50	0.31
708	C1QBP	0.00	-0.13	0.00	0.08
710	SERPING1	0.00	0.00	0.00	NaN
711	C1orf1	0.00	0.00	-0.10	-0.04
712	C1QA	-0.09	0.00	0.00	-0.21
713	C1QB	-0.09	0.00	0.00	0.19
715	C1R	0.00	0.00	0.00	0.03
716	C1S	0.00	0.00	0.00	0.02
717	C2	0.00	0.00	0.00	NaN
718	C3	0.00	0.00	0.00	NaN
719	C3AR1	-0.09	0.00	0.00	-0.07
720	C4A	0.00	0.00	0.00	NaN
721	C4B	0.00	0.00	0.00	NaN
722	C4BPA	0.00	0.13	0.00	-0.16
725	C4BPB	0.00	0.13	0.00	-0.05
726	CAPN5	0.50	0.00	0.00	0.27
727	C5	0.00	0.00	-0.05	0.21
728	C5R1	0.09	0.00	-0.05	0.14
729	C6	0.00	0.00	0.10	-0.11
730	C7	0.00	0.00	0.10	0.15
731	C8A	0.00	0.00	-0.05	-0.09
732	C8B	0.00	0.00	-0.05	0.00
733	C8G	0.00	0.00	0.00	NaN

734	C8orf1	0.18	0.25	0.05	0.59
735	C9	0.09	0.00	0.05	-0.11
738	C11orf2	-0.09	0.00	0.00	0.18
740	MRPL49	-0.09	0.00	0.00	0.30
741	C11orf5	-0.09	0.00	0.00	0.07
744	C11orf8	0.00	0.00	0.00	NaN
745	C11orf9	-0.09	0.00	0.00	-0.04
746	C11orf10	-0.09	0.00	0.00	0.40
747	C11orf11	-0.09	0.00	0.00	-0.29
750	C16orf3	-0.09	-0.13	-0.05	-0.25
752	FMNL	0.00	-0.13	0.00	0.02
753	C18orf1	0.00	0.00	0.00	NaN
754	PTTG1IP	0.00	0.00	0.00	NaN
755	C21orf2	0.00	0.00	0.00	NaN
757	C21orf4	0.00	0.00	0.00	NaN
758	C22orf1	0.00	0.00	0.05	-0.26
759	CA1	0.00	0.00	0.00	NaN
760	CA2	0.18	0.00	0.00	0.22
761	CA3	0.18	0.00	0.00	-0.12
762	CA4	0.18	0.13	0.00	0.19
763	CA5A	-0.09	-0.13	-0.05	-0.17
765	CA6	0.00	0.00	0.00	NaN
766	CA7	-0.09	0.00	0.00	-0.26
767	CA8	0.00	0.00	0.00	NaN
768	CA9	-0.09	0.00	0.10	0.28
770	CA11	0.18	0.00	0.00	-0.14
771	CA12	0.00	0.00	0.00	NaN
773	CACNA1A	0.00	0.00	0.00	NaN
774	CACNA1B	0.00	0.00	0.05	-0.08
775	CACNA1C	0.00	0.00	0.05	0.15
776	CACNA1D	0.00	0.00	-0.10	0.32
777	CACNA1E	0.00	0.00	0.00	NaN
779	CACNA1S	0.00	0.00	0.00	NaN
780	DDR1	0.00	0.00	0.00	NaN
781	CACNA2D1	0.09	0.00	0.00	-0.14
782	CACNB1	0.00	0.75	0.00	0.47
783	CACNB2	0.00	0.00	0.05	-0.05
784	CACNB3	0.00	0.00	0.00	NaN
785	CACNB4	0.00	0.00	0.00	NaN
786	CACNG1	0.18	0.13	0.00	0.24
788	SLC25A20	-0.09	0.00	-0.05	0.25
790	CAD	0.09	0.00	0.05	0.45
793	CALB1	0.18	0.25	0.05	0.00
794	CALB2	-0.09	0.13	0.05	0.25
795	CALB3	0.00	-0.13	0.00	-0.21
796	CALCA	0.00	0.00	0.00	NaN
797	CALCB	0.00	0.00	0.00	NaN

799	CALCR	0.18	0.00	0.00	0.19
800	CALD1	0.00	0.00	0.00	NaN
801	CALM1	0.33	0.33	0.00	0.25
811	CALR	0.00	0.00	0.00	NaN
813	CALU	0.00	0.25	0.00	0.30
814	CAMK4	0.00	0.13	0.00	-0.08
815	CAMK2A	0.00	0.00	0.00	NaN
816	CAMK2B	0.00	0.00	0.00	NaN
818	CAMK2G	0.09	0.00	0.00	0.29
819	CAMLG	0.00	-0.13	0.00	0.22
820	CAMP	-0.09	0.00	-0.05	0.05
821	CANX	0.00	0.00	0.05	0.11
822	CAPG	0.00	0.00	-0.05	0.07
823	CAPN1	-0.09	0.00	0.00	0.07
824	CAPN2	0.09	0.00	0.00	0.13
825	CAPN3	0.00	0.00	0.00	NaN
826	CAPNS1	0.00	0.00	0.00	NaN
829	CAPZA1	0.09	0.00	0.05	0.42
830	CAPZA2	0.00	0.25	0.00	0.50
831	CAST	0.00	0.00	0.00	NaN
832	CAPZB	-0.09	0.00	0.00	0.19
833	CARS	0.00	-0.13	0.00	0.08
834	CASP1	0.00	0.00	0.00	NaN
835	CASP2	0.00	0.00	-0.05	0.30
836	CASP3	0.00	0.00	-0.05	0.47
837	CASP4	0.00	0.00	0.00	NaN
838	CASP5	0.00	0.00	0.00	NaN
839	CASP6	0.00	0.00	0.00	NaN
840	CASP7	0.09	-0.13	-0.05	0.58
841	CASP8	0.00	0.00	0.00	NaN
842	CASP9	0.00	0.00	0.00	NaN
843	CASP10	0.00	0.00	0.00	NaN
844	CASQ1	0.00	0.00	0.00	NaN
845	CASQ2	0.09	0.00	0.10	0.00
846	CASR	0.00	0.00	0.00	NaN
847	CAT	0.00	0.13	0.05	0.65
857	CAV1	0.00	0.25	0.00	-0.18
858	CAV2	0.00	0.25	0.00	-0.18
859	CAV3	0.00	0.00	0.00	NaN
860	RUNX2	0.00	0.00	0.00	NaN
861	RUNX1	0.00	0.00	-0.05	0.38
862	CBFA2T1	0.00	-0.13	0.05	-0.05
863	CBFA2T3	-0.09	-0.13	-0.05	0.11
864	RUNX3	-0.09	0.00	0.00	0.12
865	CBFB	-0.09	0.00	0.00	0.48
866	SERPINA6	0.00	0.00	0.00	NaN
867	CBL	-0.09	0.00	-0.10	0.01

868	CBLB	0.00	0.00	0.00	NaN
869	CBLN1	-0.09	0.00	-0.05	0.20
871	SERPINH1	0.18	0.00	0.00	0.33
873	CBR1	0.00	0.00	0.00	NaN
874	CBR3	0.00	0.00	0.00	NaN
875	CBS	0.00	0.13	0.00	0.17
881	CCIN	-0.09	0.00	0.10	-0.26
883	CCBL1	0.00	0.00	0.00	NaN
885	CCK	0.00	0.00	-0.05	-0.06
886	CCKAR	0.00	0.00	-0.05	-0.29
887	CCKBR	0.00	-0.13	0.00	0.15
889	CCM1	0.18	0.00	0.00	0.52
890	CCNA2	0.00	0.00	0.00	NaN
891	CCNB1	0.00	0.00	0.05	0.02
894	CCND2	-0.09	0.00	0.05	0.11
896	CCND3	0.00	0.00	0.05	-0.06
898	CCNE1	0.00	0.13	0.15	0.73
899	CCNF	0.00	0.00	0.00	NaN
900	CCNG1	0.00	0.00	0.05	0.03
901	CCNG2	0.00	0.00	0.00	NaN
902	CCNH	0.00	-0.13	0.00	0.39
904	CCNT1	0.00	0.00	0.00	NaN
905	CCNT2	0.00	0.00	-0.05	0.23
908	CCT6A	0.00	0.00	0.20	0.53
909	CD1A	0.00	0.00	0.00	NaN
910	CD1B	0.00	0.00	0.00	NaN
911	CD1C	0.00	0.00	0.00	NaN
912	CD1D	0.00	0.00	0.00	NaN
913	CD1E	0.00	0.00	0.00	NaN
914	CD2	0.09	0.00	0.10	0.08
915	CD3D	0.00	0.00	0.00	NaN
916	CD3E	0.00	0.00	0.00	NaN
917	CD3G	0.00	0.00	0.00	NaN
919	CD3Z	0.00	0.00	0.00	NaN
921	CD5	0.00	0.00	0.00	NaN
922	CD5L	0.00	0.00	0.00	NaN
923	CD6	0.00	0.00	0.00	NaN
924	CD7	0.00	0.00	0.00	NaN
925	CD8A	0.00	0.00	0.00	0.19
926	CD8B1	0.00	0.00	-0.05	0.28
928	CD9	-0.09	0.00	0.00	0.20
929	CD14	0.00	0.00	0.00	NaN
930	CD19	0.00	0.00	0.00	NaN
931	MS4A2	0.00	0.00	0.00	NaN
933	CD22	0.00	0.00	0.05	-0.01
939	TNFRSF7	-0.09	0.00	0.00	-0.14
940	CD28	0.00	0.00	0.00	NaN

941	CD80	0.00	0.00	0.00	NaN
942	CD86	0.00	0.00	0.00	NaN
943	TNFRSF8	0.00	0.00	0.00	NaN
944	TNFSF8	0.00	0.00	0.00	NaN
945	CD33	0.00	0.13	-0.05	0.28
946	SIGLEC6	0.00	0.13	-0.05	0.25
948	CD36	0.09	0.00	0.00	0.04
949	CD36L1	-0.09	0.00	0.00	0.29
950	CD36L2	0.00	0.00	0.00	NaN
951	CD37	0.00	0.00	0.00	NaN
952	CD38	0.00	0.00	-0.10	-0.15
953	ENTPD1	0.00	0.00	-0.05	0.25
954	ENTPD2	0.00	0.00	0.00	NaN
955	ENTPD6	0.00	0.00	-0.05	0.26
956	ENTPD3	0.00	0.00	-0.05	0.26
957	ENTPD5	0.00	0.00	0.00	NaN
958	TNFRSF5	0.00	0.00	0.00	NaN
960	CD44	0.00	0.13	0.05	0.51
961	CD47	0.00	0.00	0.00	NaN
962	CD48	0.00	0.00	0.00	NaN
963	CD53	0.09	0.00	0.00	0.17
965	CD58	0.09	0.00	0.10	0.37
966	CD59	0.00	0.00	0.05	0.27
967	CD63	0.00	0.00	0.00	NaN
968	CD68	0.00	0.00	0.00	NaN
969	CD69	-0.09	0.00	0.00	0.10
970	TNFSF7	0.00	0.00	0.00	NaN
972	CD74	0.00	0.00	0.00	NaN
973	CD79A	0.00	0.00	-0.05	0.07
974	CD79B	0.27	0.13	0.00	0.10
975	CD81	0.00	-0.13	0.00	0.08
976	CD97	0.00	0.00	0.00	NaN
977	CD151	-0.18	-0.13	-0.05	0.42
978	CDA	-0.09	0.00	0.00	0.00
983	CDC2	0.00	0.00	0.00	NaN
984	CDC2L1	0.00	0.00	0.50	0.57
987	LRBA	0.00	0.00	0.00	0.28
988	CDC5L	0.00	0.00	0.00	NaN
989	CDC10	0.00	0.00	0.00	NaN
990	CDC6	0.00	0.38	0.00	0.69
991	CDC20	0.00	0.00	0.00	NaN
993	CDC25A	-0.09	0.00	-0.05	0.38
994	CDC25B	0.00	0.00	-0.05	0.29
995	CDC25C	0.00	0.00	0.00	NaN
996	CDC27	0.00	-0.13	0.00	0.25
997	CDC34	0.00	0.00	-0.05	0.18
998	CDC42	-0.09	0.00	0.00	0.08

999	CDH1	-0.09	-0.25	0.00	0.33
1000	CDH2	0.00	0.00	-0.10	0.26
1001	CDH3	-0.09	-0.25	0.00	0.43
1002	CDH4	0.18	0.13	0.00	-0.20
1003	CDH5	-0.09	-0.13	0.00	-0.01
1004	CDH6	0.00	0.00	0.15	0.00
1005	CDH7	0.00	0.00	-0.10	0.02
1006	CDH8	0.00	-0.13	0.00	-0.01
1007	CDH9	0.00	0.00	0.15	0.11
1008	CDH10	0.00	0.00	0.10	0.30
1009	CDH11	0.00	0.00	0.00	NaN
1010	CDH12	0.00	0.00	0.10	0.11
1012	CDH13	-0.09	0.00	-0.05	0.08
1013	CDH15	-0.09	-0.13	-0.05	-0.24
1014	CDH16	-0.09	-0.13	0.00	0.16
1015	CDH17	0.00	-0.13	0.00	-0.15
1016	CDH18	0.00	0.00	0.50	0.03
1017	CDK2	0.00	0.00	0.00	NaN
1018	CDK3	0.00	0.13	0.00	0.14
1019	CDK4	0.00	0.00	0.00	NaN
1020	CDK5	0.00	0.00	-0.05	0.26
1021	CDK6	0.18	0.00	0.00	0.34
1022	CDK7	0.00	0.00	0.00	NaN
1024	CDK8	0.00	-0.13	-0.05	0.22
1025	CDK9	-0.09	0.00	-0.05	0.21
1026	CDKN1A	0.00	0.00	0.00	NaN
1027	CDKN1B	-0.09	0.00	0.00	0.15
1028	CDKN1C	0.00	-0.13	-0.05	0.20
1029	CDKN2A	-0.18	-0.13	-0.25	0.62
1030	CDKN2B	-0.18	-0.13	-0.35	0.21
1031	CDKN2C	0.00	0.00	0.00	NaN
1032	CDKN2D	0.00	0.13	0.05	0.30
1033	CDKN3	0.00	0.00	0.05	0.33
1036	CDO1	0.00	0.13	0.00	-0.08
1039	CDR2	0.00	0.00	0.00	NaN
1040	CDS1	0.00	0.00	0.00	NaN
1041	CDSN	0.00	0.00	0.00	NaN
1043	CDW52	-0.09	0.00	0.00	0.21
1044	CDX1	0.00	0.00	0.00	NaN
1045	CDX2	0.00	0.00	-0.05	-0.01
1047	CLGN	0.00	0.00	0.00	NaN
1048	CEACAM5	0.00	0.00	-0.05	0.11
1050	CEBPA	0.00	0.00	0.05	-0.04
1051	CEBPB	0.27	0.25	0.05	0.34
1052	CEBPD	0.00	0.13	0.00	0.43
1053	CEBPE	0.00	0.00	0.00	NaN
1054	CEBPG	0.00	0.00	0.05	0.28

1056	CEL	0.33	0.00	0.33	-0.02
1057	CELL	0.00	0.00	0.00	NaN
1058	CENPA	0.09	0.00	0.05	0.36
1060	CENPC1	0.00	0.00	0.00	NaN
1062	CENPE	0.00	0.00	0.00	NaN
1063	CENPF	0.09	0.00	0.00	0.28
1066	CES1	0.50	0.00	0.00	-0.01
1068	CETN1	0.00	0.00	0.00	NaN
1070	CETN3	0.00	0.00	0.00	NaN
1071	CETP	-0.09	0.00	0.00	-0.08
1072	CFL1	-0.09	0.00	0.05	0.42
1075	CTSC	0.00	0.13	0.05	0.41
1080	CFTR	0.00	-0.13	0.00	-0.19
1081	CGA	0.00	0.00	0.00	NaN
1082	CGB	1.00	0.00	0.00	0.36
1084	CEACAM3	0.00	0.00	-0.05	0.10
1087	CEACAM7	0.00	0.00	-0.05	0.09
1088	CEACAM8	0.09	0.00	-0.05	0.10
1089	CEACAM4	0.00	0.00	-0.05	0.16
1101	CHAD	0.00	0.25	0.00	-0.04
1102	CHC1L	0.00	0.13	0.05	-0.02
1103	CHAT	0.00	0.00	0.00	NaN
1104	CHC1	0.00	0.00	0.00	NaN
1105	CHD1	0.00	0.00	0.00	NaN
1106	CHD2	0.00	0.00	0.00	NaN
1107	CHD3	0.00	0.00	0.00	NaN
1108	CHD4	-0.09	0.00	0.00	0.28
1109	AKR1C4	0.00	0.00	0.05	-0.01
1111	CHEK1	-0.09	0.00	-0.10	0.34
1112	CHES1	0.00	0.13	0.00	0.07
1113	CHGA	0.00	0.00	0.00	NaN
1114	CHGB	0.00	0.00	0.00	NaN
1116	CHI3L1	0.00	0.13	0.00	-0.07
1117	CHI3L2	0.09	0.00	0.00	-0.06
1118	CHIT1	0.00	0.13	0.00	-0.34
1119	CHK	0.18	0.00	0.05	0.32
1120	CHKL	0.00	-0.25	0.05	0.10
1122	CHML	0.00	0.00	0.00	NaN
1123	CHN1	0.00	0.00	0.00	NaN
1124	CHN2	0.00	0.13	0.00	0.16
1129	CHRM2	0.00	0.00	0.00	NaN
1130	CHS1	0.09	0.00	0.00	0.19
1132	CHRM4	0.00	0.00	0.00	NaN
1133	CHRM5	0.00	0.00	0.00	NaN
1134	CHRNA1	0.00	0.00	0.00	NaN
1135	CHRNA2	-0.09	-0.25	-0.10	-0.05
1136	CHRNA3	0.00	0.00	0.05	0.07

1137	CHRNA4	0.09	0.00	0.00	0.21
1138	CHRNA5	0.00	0.00	0.00	NaN
1139	CHRNA7	0.00	0.00	0.00	NaN
1140	CHRNB1	0.00	0.00	0.00	NaN
1141	CHRNB2	0.09	0.25	0.00	-0.16
1142	CHRNB3	0.27	0.00	-0.05	0.41
1143	CHRNB4	0.00	0.00	0.00	NaN
1144	CHRND	0.00	0.00	0.00	NaN
1145	CHRNE	0.00	0.00	0.00	NaN
1146	CHRNG	0.00	0.00	0.00	NaN
1147	CHUK	0.00	-0.13	-0.05	0.31
1149	CIDEA	0.00	0.00	0.00	NaN
1152	CKB	0.09	0.00	0.00	0.05
1153	CIRBP	0.00	0.00	0.00	NaN
1154	CISH	-0.09	0.00	-0.05	0.12
1155	CKAP1	0.00	0.00	0.00	NaN
1158	CKM	0.09	0.00	0.00	-0.15
1159	CKMT1	0.00	0.00	0.50	0.10
1160	CKMT2	0.00	0.00	0.00	NaN
1161	CKN1	0.00	0.00	0.00	NaN
1163	CKS1	0.09	0.13	0.00	0.21
1164	CKS2	0.00	0.00	0.00	NaN
1173	AP2M1	0.09	0.00	0.00	0.33
1174	AP1S1	0.18	0.00	0.00	0.32
1175	AP2S1	0.09	0.00	0.00	0.48
1176	AP3S1	0.00	0.13	0.00	0.16
1178	CLC	0.00	0.00	0.00	NaN
1179	CLCA1	0.00	0.00	0.10	0.14
1180	CLCN1	0.00	0.00	-0.05	0.26
1181	CLCN2	0.09	0.00	0.00	0.12
1182	CLCN3	0.00	0.00	-0.05	0.25
1185	CLCN6	0.00	0.00	0.00	NaN
1186	CLCN7	0.00	0.00	0.00	NaN
1187	CLCNKA	0.00	0.00	0.00	NaN
1188	CLCNKB	0.00	0.00	0.00	NaN
1191	CLU	0.00	0.00	0.00	0.18
1192	CLIC1	0.00	0.00	0.00	NaN
1196	CLK2	0.09	0.13	0.00	0.46
1198	CLK3	0.00	0.00	0.00	NaN
1200	CLN2	0.00	-0.13	0.00	0.27
1201	CLN3	0.00	0.00	0.00	NaN
1203	CLN5	-0.09	0.13	-0.10	0.64
1207	CLNS1A	0.27	0.00	0.00	0.62
1208	CLPS	0.00	0.00	0.00	NaN
1209	CLPTM1	0.09	0.00	0.00	0.53
1211	CLTA	-0.09	0.00	0.10	0.55
1212	CLTB	0.00	0.00	0.00	NaN

1213	CLTC	0.18	0.13	0.00	0.64
1215	CMA1	0.00	0.00	0.00	NaN
1230	CCR1	-0.09	0.00	-0.05	0.10
1232	CCR3	-0.09	0.00	-0.05	0.03
1233	CCR4	0.00	-0.13	0.00	-0.07
1235	CCR6	0.00	0.00	0.00	NaN
1236	CCR7	0.00	0.38	0.00	0.37
1237	CCR8	0.00	0.00	-0.05	0.10
1238	CCBP2	0.00	0.00	0.00	0.16
1240	CMKLR1	0.00	-0.13	0.00	-0.03
1241	LTB4R	0.00	0.00	0.00	NaN
1244	ABCC2	0.00	-0.13	-0.05	-0.13
1258	CNGB1	0.00	0.00	0.00	-0.05
1259	CNGA1	0.00	0.00	0.00	NaN
1261	CNGA3	0.00	0.00	0.00	NaN
1263	CNK	0.00	0.00	0.00	NaN
1264	CNN1	0.00	0.00	0.00	NaN
1265	CNN2	0.00	0.13	0.00	0.08
1266	CNN3	0.09	0.00	0.05	0.47
1267	CNP	0.00	0.13	0.00	0.32
1268	CNR1	0.00	0.00	0.00	NaN
1269	CNR2	-0.09	0.00	0.00	-0.17
1270	CNTF	0.00	0.00	0.00	NaN
1271	CNTFR	-0.09	0.13	0.10	-0.23
1272	CNTN1	0.50	0.50	0.50	-0.14
1277	COL1A1	0.00	0.25	0.00	0.17
1280	COL2A1	0.00	0.00	0.00	NaN
1281	COL3A1	0.00	0.00	0.00	NaN
1282	COL4A1	-0.09	-0.13	0.00	0.20
1284	COL4A2	-0.09	-0.13	0.00	0.21
1285	COL4A3	0.00	0.00	0.00	NaN
1286	COL4A4	0.00	0.00	0.00	NaN
1289	COL5A1	0.00	0.00	0.00	NaN
1290	COL5A2	0.00	0.00	0.00	NaN
1291	COL6A1	0.00	0.00	0.00	NaN
1292	COL6A2	0.00	0.00	0.00	NaN
1293	COL6A3	0.00	0.00	-0.05	0.07
1294	COL7A1	-0.09	0.00	-0.05	-0.05
1295	COL8A1	0.00	0.00	0.00	NaN
1297	COL9A1	0.00	0.00	0.05	0.46
1298	COL9A2	0.00	0.00	0.00	NaN
1299	COL9A3	0.09	0.13	0.00	-0.07
1300	COL10A1	-0.09	0.00	0.10	-0.28
1301	COL11A1	0.00	0.00	0.00	NaN
1305	COL13A1	0.00	0.00	0.00	NaN
1306	COL15A1	0.00	0.00	0.00	NaN
1307	COL16A1	0.00	0.00	0.00	NaN

1308	COL17A1	0.09	-0.13	-0.05	0.01
1310	COL19A1	-0.09	0.00	0.05	0.27
1311	COMP	0.00	0.00	0.00	NaN
1312	COMT	-0.09	0.00	0.00	0.11
1314	COPA	0.00	0.00	0.00	NaN
1315	COPB	0.00	0.00	0.00	NaN
1316	COPEB	0.00	0.00	0.00	NaN
1317	SLC31A1	0.00	0.00	0.00	NaN
1318	SLC31A2	0.00	0.00	0.00	NaN
1325	CORT	0.00	0.00	0.00	NaN
1326	MAP3K8	-0.09	-0.13	0.00	0.47
1327	COX4I1	-0.09	0.00	-0.05	0.51
1329	COX5B	0.00	0.00	0.00	NaN
1337	COX6A1	0.00	-0.13	0.00	0.04
1339	COX6A2	0.00	0.00	0.00	NaN
1340	COX6B	0.00	0.00	0.00	NaN
1345	COX6C	0.09	0.00	0.05	0.33
1346	COX7A1	0.00	0.00	0.00	NaN
1347	COX7A2	0.00	0.00	0.05	0.27
1350	COX7C	0.00	-0.13	0.00	0.56
1352	COX10	0.00	0.00	0.00	NaN
1353	COX11	0.00	0.00	0.00	0.44
1355	COX15	0.00	-0.13	-0.05	0.35
1356	CP	0.00	0.00	0.05	-0.04
1357	CPA1	0.00	0.25	0.05	0.14
1358	CPA2	0.00	0.25	0.05	0.12
1359	CPA3	0.00	0.00	0.00	NaN
1360	CPB1	0.00	0.00	0.00	NaN
1361	CPB2	0.00	0.00	-0.05	-0.11
1362	CPD	0.00	0.38	0.00	0.62
1363	CPE	0.00	0.00	0.00	NaN
1364	CLDN4	-0.09	0.00	0.00	0.06
1365	CLDN3	-0.09	0.00	0.00	0.04
1366	CLDN7	0.00	0.00	0.00	NaN
1368	CPM	0.09	0.13	0.00	-0.02
1369	CPN1	0.00	-0.13	-0.05	-0.08
1370	CPN2	0.09	0.00	0.00	-0.15
1371	CPO	0.00	0.00	0.00	NaN
1373	CPS1	0.00	0.00	0.00	NaN
1374	CPT1A	0.45	0.00	0.00	0.64
1375	CPT1B	0.00	-0.25	0.00	0.22
1376	CPT2	0.00	0.00	0.00	NaN
1378	CR1	0.09	0.13	0.00	-0.03
1380	CR2	0.09	0.13	0.00	-0.08
1381	CRABP1	0.50	0.00	0.00	0.10
1382	CRABP2	0.00	0.13	0.00	0.27
1384	CRAT	0.00	0.00	0.00	NaN

1385	CREB1	0.00	0.00	0.00	NaN
1386	ATF2	0.00	0.00	0.00	NaN
1387	CREBBP	0.00	0.00	0.00	NaN
1388	CREBL1	0.18	0.00	0.00	0.07
1389	CREBL2	-0.09	0.00	0.00	0.35
1390	CREM	-0.09	0.00	0.05	0.50
1392	CRH	0.00	0.00	0.05	0.18
1393	CRHBP	0.00	0.00	0.00	NaN
1394	CRHR1	0.00	-0.13	0.00	-0.19
1395	CRHR2	0.00	0.25	0.00	0.53
1396	CRIP1	0.00	0.00	0.00	NaN
1397	CRIP2	0.00	0.00	0.00	NaN
1398	CRK	0.00	-0.13	0.00	0.05
1399	CRKL	0.00	0.13	0.00	0.25
1400	CRMP1	0.00	-0.13	-0.10	0.10
1401	CRP	0.00	0.00	0.05	-0.03
1404	CRTL1	0.00	-0.13	0.00	0.10
1406	CRX	0.09	0.00	0.00	-0.06
1407	CRY1	0.00	0.00	0.00	NaN
1408	CRY2	0.00	0.00	0.00	NaN
1409	CRYAA	0.00	0.13	0.00	0.41
1410	CRYAB	0.00	0.00	0.00	NaN
1411	CRYBA1	0.00	0.38	0.00	0.11
1412	CRYBA2	0.00	0.00	0.00	NaN
1413	CRYBA4	0.00	0.13	0.00	0.26
1414	CRYBB1	0.00	0.13	0.00	0.37
1415	CRYBB2	0.00	0.00	0.00	NaN
1417	CRYBB3	0.00	0.00	0.00	NaN
1418	CRYGA	0.00	0.00	0.00	NaN
1419	CRYGB	0.00	0.00	0.00	NaN
1420	CRYGC	0.00	0.00	0.00	NaN
1421	CRYGD	0.00	0.00	0.00	NaN
1428	CRYM	0.00	0.00	0.00	NaN
1429	CRYZ	0.00	0.00	0.00	NaN
1431	CS	0.00	0.00	0.00	NaN
1432	MAPK14	0.00	0.00	0.00	NaN
1434	CSE1L	0.09	0.00	0.00	0.17
1435	CSF1	0.09	0.00	0.05	0.23
1436	CSF1R	0.00	0.00	0.00	NaN
1437	CSF2	0.00	-0.13	0.00	-0.15
1439	CSF2RB	-0.09	0.00	0.00	0.07
1440	CSF3	0.00	0.50	0.00	0.34
1441	CSF3R	0.00	0.00	0.00	NaN
1442	CSH1	1.00	1.00	0.00	0.23
1444	CSHL1	0.27	0.13	0.00	0.17
1445	CSK	0.00	0.00	0.00	NaN
1446	CSN1	0.00	0.00	0.00	NaN

1447	CSN2	0.00	0.00	0.00	NaN
1448	CSN10	0.00	0.00	0.00	NaN
1452	CSNK1A1	0.00	0.00	0.00	NaN
1453	CSNK1D	0.00	0.00	0.00	NaN
1454	CSNK1E	-0.09	0.00	0.05	0.40
1455	CSNK1G2	0.00	0.00	0.00	NaN
1456	CSNK1G3	0.00	0.00	0.00	NaN
1457	CSNK2A1	0.00	0.00	0.00	0.54
1459	CSNK2A2	-0.09	0.00	0.00	0.34
1460	CSNK2B	0.00	0.00	0.00	NaN
1462	CSPG2	0.00	-0.13	0.00	0.13
1463	CSPG3	0.00	0.00	0.00	NaN
1464	CSPG4	0.00	0.00	0.00	NaN
1465	CSRP1	0.00	0.00	0.00	NaN
1466	CSRP2	0.00	0.00	0.00	NaN
1468	SLC25A10	0.00	0.00	0.00	NaN
1469	CST1	0.00	0.00	0.00	NaN
1470	CST2	0.00	0.00	0.00	NaN
1471	CST3	0.00	0.00	0.00	NaN
1472	CST4	0.00	0.00	0.00	NaN
1473	CST5	0.00	0.00	0.00	NaN
1474	CST6	-0.09	0.00	0.05	-0.05
1475	CSTA	0.00	0.00	0.00	NaN
1476	CSTB	0.00	0.00	0.00	NaN
1477	CSTF1	0.09	0.50	0.00	0.55
1479	CSTF3	0.00	0.00	0.05	0.08
1482	CSX	0.00	0.00	0.00	NaN
1486	CTBS	0.00	0.13	0.05	0.54
1487	CTBP1	0.00	-0.13	-0.10	0.64
1488	CTBP2	0.00	0.00	-0.05	0.25
1489	CTF1	0.00	0.00	0.00	NaN
1490	CTGF	0.00	0.00	0.00	NaN
1491	CTH	0.00	0.00	0.00	NaN
1493	CTLA4	0.00	0.00	0.00	NaN
1495	CTNNA1	0.00	0.00	-0.05	0.61
1496	CTNNA2	0.00	0.00	0.00	NaN
1497	CTNS	0.00	0.00	0.00	NaN
1499	CTNNB1	0.00	0.00	-0.05	0.09
1500	CTNND1	0.00	0.00	0.00	NaN
1501	CTNND2	0.00	0.00	0.10	-0.23
1503	CTPS	0.00	0.00	0.00	NaN
1504	CTRB1	0.00	0.00	0.05	0.07
1506	CTRL	-0.09	-0.13	0.00	0.32
1508	CTSB	-0.09	-0.50	-0.15	0.39
1509	CTSD	0.00	-0.13	-0.05	-0.08
1510	CTSE	0.00	0.13	0.00	-0.09
1511	CTSG	0.00	0.00	0.00	NaN

1512	CTSH	0.00	0.00	0.00	NaN
1513	CTSK	0.00	0.00	0.50	0.57
1514	CTSL	0.00	0.00	0.00	NaN
1515	CTSL2	0.00	0.00	0.00	NaN
1520	CTSS	0.00	0.13	0.05	0.42
1521	CTSW	-0.09	0.00	0.05	0.08
1522	CTSZ	0.18	0.25	0.00	0.38
1523	CUTL1	0.18	0.00	0.00	0.20
1524	CX3CR1	0.00	0.00	0.00	0.19
1525	CXADR	0.00	-0.13	0.00	0.17
1528	CYB5	-0.09	0.00	-0.25	0.49
1534	CYB561	0.27	0.13	0.00	0.60
1535	CYBA	-0.09	-0.13	-0.05	-0.01
1537	CYC1	0.18	0.00	0.10	0.27
1539	CYLC2	0.00	0.00	0.00	NaN
1540	CYLD	0.00	0.00	-0.05	0.13
1543	CYP1A1	0.00	0.50	0.50	0.08
1544	CYP1A2	0.00	0.00	0.00	NaN
1545	CYP1B1	0.00	0.25	0.00	0.45
1548	CYP2A6	0.00	0.00	0.00	NaN
1549	CYP2A7	0.00	0.00	0.00	NaN
1551	CYP3A7	0.18	0.00	0.00	0.59
1553	CYP2A13	0.00	0.00	-0.05	0.07
1555	CYP2B6	0.00	0.00	-0.05	0.07
1556	CYP2B7	0.00	0.00	-0.05	0.12
1558	CYP2C8	0.00	0.00	0.00	NaN
1559	CYP2C9	0.00	0.00	0.00	NaN
1562	CYP2C18	0.00	0.00	0.00	NaN
1565	CYP2D6	0.00	0.00	0.05	0.04
1571	CYP2E	0.00	0.00	-0.10	0.14
1572	CYP2F1	0.00	0.00	-0.05	-0.02
1573	CYP2J2	0.00	0.00	-0.05	0.18
1576	CYP3A4	0.18	0.00	0.00	0.24
1577	CYP3A5	0.18	0.00	0.00	-0.04
1579	CYP4A11	0.00	0.00	0.00	NaN
1580	CYP4B1	0.00	0.00	0.00	NaN
1581	CYP7A1	0.00	0.00	0.00	NaN
1583	CYP11A	0.00	0.00	0.00	NaN
1584	CYP11B1	0.18	0.13	0.05	-0.14
1585	CYP11B2	0.18	0.13	0.05	-0.10
1586	CYP17	0.00	-0.13	-0.05	-0.18
1588	CYP19	0.18	0.00	0.00	-0.07
1589	CYP21A2	0.00	0.00	0.00	NaN
1591	CYP24	0.18	0.38	0.00	0.09
1592	CYP26A1	0.00	0.00	0.00	NaN
1593	CYP27A1	0.00	0.00	0.00	NaN
1594	CYP27B1	0.00	0.00	0.00	NaN

1595	CYP51	0.18	0.00	0.00	0.41
1600	DAB1	0.00	0.00	-0.05	-0.07
1601	DAB2	0.00	0.00	0.05	0.04
1602	DACH	0.00	0.00	-0.10	0.13
1603	DAD1	0.00	0.00	0.00	NaN
1604	DAF	0.00	0.13	0.00	0.27
1605	DAG1	-0.09	0.00	-0.05	0.41
1606	DGKA	0.00	0.00	0.00	NaN
1607	DGKB	0.00	0.13	0.05	-0.27
1608	DGKG	0.09	0.00	0.05	-0.18
1609	DGKQ	0.00	-0.13	-0.05	0.05
1611	DAP	0.00	0.00	0.10	0.41
1612	DAPK1	0.00	0.00	0.00	NaN
1613	DAPK3	0.09	0.00	0.00	-0.12
1615	DARS	0.00	0.00	-0.05	0.49
1616	DAXX	0.09	0.00	0.00	-0.05
1618	DAZL	0.00	0.00	0.00	NaN
1620	DBCCR1	0.00	0.00	0.00	NaN
1621	DBH	0.00	0.00	0.00	NaN
1627	DBN1	0.00	0.13	0.00	-0.13
1628	DBP	0.09	0.00	0.00	0.17
1629	DBT	0.00	0.00	0.00	NaN
1630	DCC	-0.09	-0.25	-0.20	-0.17
1633	DCK	0.00	0.00	0.00	NaN
1634	DCN	0.00	0.00	0.00	NaN
1635	DCTD	0.00	0.00	-0.05	0.30
1636	ACE	0.27	0.13	0.00	0.09
1638	DCT	0.00	0.13	-0.05	0.08
1639	DCTN1	0.00	0.00	0.00	NaN
1642	DDB1	-0.09	0.00	0.00	0.20
1643	DDB2	0.00	0.00	0.00	NaN
1644	DDC	0.00	0.00	0.00	NaN
1645	AKR1C1	0.00	0.00	0.05	0.01
1646	AKR1C2	0.00	0.00	0.05	0.03
1647	GADD45A	0.00	0.00	0.00	NaN
1649	DDIT3	0.00	0.00	0.00	NaN
1650	DDOST	0.00	0.00	0.00	0.38
1652	DDT	0.00	0.13	0.00	0.21
1653	DDX1	0.09	0.00	-0.10	0.57
1655	DDX5	0.27	0.13	0.00	0.57
1656	DDX6	0.00	0.00	0.00	NaN
1657	DMXL1	0.00	0.00	0.00	NaN
1659	DDX8	0.00	0.00	0.00	0.24
1660	DDX9	0.00	0.00	0.00	NaN
1662	DDX10	0.00	0.00	0.00	NaN
1663	DDX11	0.00	0.00	0.00	0.03
1665	DDX15	0.00	0.00	0.00	0.55

1666	DECR1	0.18	0.25	0.05	0.42
1667	DEFA1	-0.09	-0.38	-0.05	0.11
1669	DEFA4	-0.09	-0.38	-0.10	-0.10
1670	DEFA5	-0.09	-0.38	-0.10	-0.36
1671	DEFA6	0.00	0.00	0.00	0.02
1672	DEFB1	-0.09	-0.38	-0.10	0.14
1673	DEFB4	-0.27	-0.25	-0.15	0.15
1674	DES	0.00	0.00	0.00	NaN
1675	DF	0.00	0.00	0.05	-0.13
1676	DFFA	0.00	0.00	0.00	NaN
1677	DFFB	0.00	0.00	0.00	NaN
1687	DFNA5	0.00	0.13	0.00	-0.06
1690	COCH	0.00	0.00	0.00	NaN
1716	DGUOK	0.00	0.00	0.00	NaN
1717	DHCR7	0.00	0.00	0.00	0.38
1718	DHCR24	0.00	0.00	0.00	NaN
1719	DHFR	0.00	0.00	0.00	NaN
1723	DHODH	0.00	0.00	0.05	0.39
1725	DHPS	0.00	0.00	0.00	NaN
1727	DIA1	0.00	0.00	0.00	NaN
1728	NQO1	0.00	-0.13	0.05	0.45
1729	DIAPH1	0.00	0.00	0.00	NaN
1733	DIO1	0.00	0.00	0.00	NaN
1734	DIO2	0.00	0.00	0.00	NaN
1735	DIO3	0.00	0.00	0.00	NaN
1737	DLAT	0.00	0.00	0.00	NaN
1738	DLD	0.09	0.00	0.00	0.20
1739	DLG1	0.18	0.00	0.00	0.52
1740	DLG2	0.00	0.00	0.05	0.07
1742	DLG4	0.00	0.00	0.00	NaN
1743	DLST	0.00	0.00	0.00	NaN
1746	DLX2	0.00	0.00	0.00	NaN
1748	DLX4	0.00	0.25	0.00	0.37
1749	DLX5	0.18	0.00	0.00	0.00
1750	DLX6	0.18	0.00	0.00	-0.05
1755	DMBT1	0.00	0.00	-0.05	-0.08
1757	SARDH	0.00	0.00	0.00	NaN
1758	DMP1	0.00	0.00	0.00	NaN
1759	DNM1	-0.09	0.00	0.00	0.15
1760	DMPK	0.09	0.00	0.00	0.34
1761	DMRT1	0.00	-0.25	-0.15	-0.02
1762	DMWD	0.09	0.00	0.00	0.04
1763	DNA2L	0.00	0.00	0.00	NaN
1770	DNAH9	0.00	0.00	0.00	NaN
1773	DNASE1	0.00	0.00	0.00	NaN
1775	DNASE1L2	0.00	0.00	0.00	NaN
1776	DNASE1L3	0.00	-0.13	-0.10	0.24

1777	DNASE2	0.00	0.00	0.00	NaN
1778	DNCH1	0.00	0.00	0.00	NaN
1780	DNCI1	0.18	0.00	0.00	0.24
1781	DNCI2	0.00	0.00	0.00	NaN
1783	DNCLI2	-0.09	-0.13	0.00	0.35
1785	DNM2	0.00	-0.13	-0.05	-0.14
1786	DNMT1	0.00	0.00	0.05	0.40
1787	DNMT2	0.00	0.00	0.05	0.47
1788	DNMT3A	0.09	0.00	0.00	0.38
1789	DNMT3B	0.00	0.00	0.00	NaN
1791	DNTT	0.00	0.00	-0.05	0.08
1793	DOCK1	0.00	0.00	-0.05	0.28
1794	DOCK2	0.00	0.00	0.00	NaN
1795	DOCK3	-0.09	0.00	-0.05	-0.04
1797	DOM3Z	0.00	0.00	0.00	NaN
1798	DPAGT1	0.50	0.00	0.50	0.36
1800	DPEP1	0.00	0.00	0.50	-0.03
1801	DPH2L1	0.00	-0.13	0.00	-0.02
1802	DPH2L2	0.00	0.00	0.00	NaN
1803	DPP4	0.00	0.00	-0.05	0.10
1804	DPP6	0.00	0.00	0.00	NaN
1805	DPT	0.00	0.00	0.00	NaN
1806	DPYD	0.09	0.00	0.05	0.23
1807	DPYS	0.18	0.00	0.05	0.01
1808	DPYSL2	-0.09	-0.25	-0.05	0.15
1809	DPYSL3	0.00	0.00	0.00	NaN
1810	DR1	0.09	0.00	0.05	0.44
1811	SLC26A3	0.09	0.00	0.00	-0.16
1812	DRD1	0.00	0.00	0.00	NaN
1813	DRD2	0.00	0.00	0.00	NaN
1814	DRD3	0.00	0.00	0.00	NaN
1815	DRD4	-0.18	-0.13	0.00	0.28
1819	DRG2	0.00	0.00	0.00	NaN
1820	DRIL1	0.00	0.00	0.00	NaN
1822	DRPLA	0.00	0.00	0.00	0.47
1823	DSC1	0.00	0.00	-0.05	0.05
1824	DSC2	0.00	0.00	1.00	0.47
1826	DSCAM	0.00	0.00	0.00	NaN
1827	DSCR1	0.00	0.00	0.00	NaN
1828	DSG1	0.00	0.00	-0.10	0.54
1830	DSG3	0.00	0.00	-0.10	0.24
1832	DSP	0.00	0.00	0.00	NaN
1833	DSPG3	0.00	0.00	0.00	NaN
1836	SLC26A2	0.00	0.00	0.00	NaN
1837	DTNA	0.00	0.00	-0.20	0.18
1838	DTNB	0.09	0.00	0.00	0.73
1839	DTR	0.00	0.00	0.00	NaN

1841	DTYMK	0.00	0.00	-0.05	0.38
1842	ECM2	0.00	0.00	0.00	NaN
1843	DUSP1	0.00	0.00	0.00	NaN
1844	DUSP2	0.00	0.00	0.00	NaN
1845	DUSP3	0.00	-0.13	0.00	0.29
1846	DUSP4	-0.09	-0.25	-0.05	-0.02
1847	DUSP5	0.00	0.00	-0.05	-0.20
1848	DUSP6	0.09	0.00	0.00	0.56
1849	DUSP7	-0.09	0.00	-0.10	-0.20
1850	DUSP8	-0.09	-0.13	-0.05	-0.03
1854	DUT	0.00	0.00	0.00	NaN
1855	DVL1	0.00	0.00	0.00	NaN
1856	DVL2	0.00	0.00	0.00	NaN
1857	DVL3	0.09	0.00	0.00	0.29
1859	DYRK1A	0.00	0.00	0.00	NaN
1861	DYT1	0.00	0.00	0.00	NaN
1869	E2F1	0.00	0.00	0.00	0.36
1870	E2F2	-0.09	0.00	0.00	0.02
1871	E2F3	0.00	0.00	0.00	NaN
1874	E2F4	-0.09	0.00	0.00	-0.05
1875	E2F5	0.00	0.00	0.00	NaN
1876	E2F6	0.00	0.00	0.00	NaN
1877	E4F1	0.00	0.00	0.00	NaN
1880	EBI2	-0.09	-0.13	-0.05	0.05
1889	ECE1	-0.09	0.00	-0.05	0.13
1890	ECGF1	0.00	-0.25	0.00	-0.09
1891	ECH1	0.00	0.00	0.00	NaN
1892	ECHS1	0.00	0.00	-0.10	0.44
1893	ECM1	0.00	0.13	0.05	-0.20
1901	EDG1	0.09	0.00	0.00	0.12
1902	EDG2	0.00	0.00	0.00	0.22
1906	EDN1	0.00	0.00	0.05	0.25
1907	EDN2	0.00	0.00	0.00	NaN
1908	EDN3	0.18	0.25	0.00	0.13
1909	EDNRA	0.00	0.00	0.00	0.18
1910	EDNRB	-0.09	0.00	-0.10	0.11
1911	EDR1	-0.09	0.00	0.00	0.32
1912	EDR2	0.00	0.00	0.00	NaN
1915	EEF1A1	0.00	0.00	0.05	0.21
1917	EEF1A2	0.09	0.00	0.00	-0.14
1933	EEF1B2	0.00	0.00	0.00	NaN
1936	EEF1D	0.18	0.00	0.10	0.18
1937	EEF1G	0.00	0.00	0.00	NaN
1938	EEF2	0.00	0.00	0.00	NaN
1939	LGTN	0.00	0.13	0.00	0.37
1942	EFNA1	0.27	0.13	0.00	0.39
1943	EFNA2	0.00	0.00	0.00	NaN

1944	EFNA3	0.09	0.13	0.00	0.06
1945	EFNA4	0.09	0.13	0.00	0.29
1946	EFNA5	0.00	0.00	0.00	NaN
1948	EFNB2	-0.09	-0.13	0.00	0.33
1949	EFNB3	0.00	0.00	0.05	0.17
1950	EGF	0.00	0.00	0.00	NaN
1951	CELSR3	-0.09	0.00	-0.05	0.22
1952	CELSR2	0.09	0.00	0.05	0.14
1953	EGFL3	0.00	0.00	0.05	-0.01
1954	EGFL4	0.09	0.00	-0.05	0.23
1955	EGFL5	0.00	0.00	0.00	NaN
1956	EGFR	0.00	0.00	0.10	0.74
1958	EGR1	0.50	0.00	0.00	-0.12
1959	EGR2	0.00	0.00	0.00	NaN
1960	EGR3	-0.09	-0.25	-0.15	0.11
1961	EGR4	0.00	0.00	0.00	NaN
1962	EHHADH	0.50	0.00	0.00	0.23
1965	EIF2S1	0.00	0.00	0.50	0.29
1967	EIF2B1	0.00	0.00	0.00	NaN
1969	EPHA2	0.00	0.00	0.00	NaN
1973	EIF4A1	0.00	0.00	0.00	NaN
1974	EIF4A2	0.09	0.00	0.05	0.27
1975	EIF4B	0.00	0.00	0.00	NaN
1977	EIF4E	0.00	0.00	0.00	NaN
1978	EIF4EBP1	0.55	-0.13	-0.05	0.59
1979	EIF4EBP2	0.00	0.00	0.00	NaN
1981	EIF4G1	0.09	0.00	0.00	0.35
1982	EIF4G2	0.00	0.00	0.00	NaN
1983	EIF5	0.00	0.00	0.00	NaN
1984	EIF5A	0.00	0.00	0.00	NaN
1990	ELA1	0.00	0.00	0.00	NaN
1991	ELA2	0.00	0.00	0.05	-0.15
1992	SERPINB1	0.00	0.00	-0.10	0.11
1993	ELAVL2	0.00	-0.25	-0.10	0.32
1994	ELAVL1	0.00	0.00	0.00	NaN
1995	ELAVL3	0.00	0.00	0.00	NaN
1996	ELAVL4	0.00	0.00	0.00	NaN
1997	ELF1	0.00	0.00	-0.05	0.23
1998	ELF2	0.00	0.00	0.00	NaN
1999	ELF3	0.18	0.00	0.00	0.03
2001	ELF5	0.00	0.13	0.05	-0.10
2004	ELK3	0.00	0.13	0.00	-0.06
2005	ELK4	0.00	0.13	0.00	-0.05
2006	ELN	-0.09	0.00	0.00	-0.12
2009	EML1	0.00	0.00	0.00	NaN
2011	EMK1	0.00	0.00	0.00	NaN
2012	EMP1	-0.09	0.00	0.00	0.23

2013	EMP2	0.00	0.00	0.00	NaN
2014	EMP3	0.09	0.00	0.00	-0.12
2015	EMR1	0.00	0.00	0.00	NaN
2016	EMX1	0.00	0.00	0.00	NaN
2017	EMS1	0.45	0.25	0.00	0.87
2018	EMX2	0.00	0.00	-0.05	-0.09
2019	EN1	0.00	0.00	0.00	NaN
2020	EN2	0.00	0.00	0.00	NaN
2021	ENDOG	0.00	0.00	0.00	NaN
2022	ENG	-0.09	0.00	-0.05	0.07
2023	ENO1	0.00	0.00	0.00	NaN
2026	ENO2	-0.09	0.00	0.00	0.14
2027	ENO3	0.00	0.00	0.00	NaN
2028	ENPEP	0.00	0.00	0.00	NaN
2029	ENSA	0.00	0.13	0.05	0.23
2030	SLC29A1	0.00	0.00	0.00	NaN
2033	EP300	0.00	0.00	0.05	0.16
2034	EPAS1	0.00	0.50	0.00	-0.17
2035	EPB41	0.00	0.00	0.00	NaN
2037	EPB41L2	0.00	0.00	0.00	NaN
2038	EPB42	0.00	0.00	0.00	NaN
2039	EPB49	-0.09	-0.25	-0.10	0.19
2040	EPB72	0.00	0.00	-0.10	0.21
2041	EPHA1	0.00	0.00	-0.05	0.24
2042	EPHA3	-0.09	0.00	0.10	0.40
2043	EPHA4	0.00	0.00	0.00	NaN
2044	EPHA5	0.00	0.00	0.00	NaN
2045	EPHA7	-0.09	0.00	0.05	0.15
2047	EPHB1	0.00	0.00	0.00	NaN
2048	EPHB2	-0.09	0.00	0.00	0.10
2049	EPHB3	0.09	0.00	0.00	0.15
2050	EPHB4	0.18	0.00	0.00	0.29
2051	EPHB6	0.00	0.00	-0.05	0.16
2052	EPHX1	0.09	0.00	0.00	-0.05
2053	EPHX2	-0.09	-0.25	-0.10	0.17
2054	EPIM	0.00	0.00	0.00	NaN
2055	CLN8	-0.09	-0.25	-0.15	0.25
2056	EPO	0.18	0.00	0.00	-0.12
2057	EPOR	0.00	0.00	0.00	NaN
2058	EPRS	0.50	0.00	0.50	0.32
2059	EPS8	0.00	0.00	0.00	NaN
2060	EPS15	0.00	0.00	0.05	-0.38
2063	NR2F6	-0.09	0.00	0.00	0.46
2064	ERBB2	0.00	1.00	0.05	0.87
2065	ERBB3	0.00	0.00	0.00	NaN
2066	ERBB4	0.00	0.00	0.00	NaN
2067	ERCC1	0.09	0.00	0.00	0.33

2068	ERCC2	0.09	0.00	0.00	-0.10
2069	EREG	0.00	0.00	0.00	NaN
2070	EYA4	0.00	0.00	0.00	NaN
2071	ERCC3	0.00	0.00	-0.05	0.44
2072	ERCC4	0.00	0.00	0.50	0.19
2073	ERCC5	-0.09	-0.13	0.05	0.51
2074	ERCC6	0.00	0.00	0.00	NaN
2077	ERF	0.00	0.00	-0.05	0.26
2078	ERG	0.00	0.00	0.00	NaN
2079	ERH	0.00	0.00	0.00	NaN
2081	ERN1	0.27	0.13	0.00	0.27
2098	ESD	0.00	0.00	0.00	NaN
2099	ESR1	0.00	0.00	0.00	NaN
2100	ESR2	0.00	0.00	0.00	NaN
2101	ESRRA	0.00	0.00	0.00	NaN
2103	ESRRB	0.00	0.00	0.00	NaN
2104	ESRRG	0.09	0.00	0.00	0.09
2107	ETF1	0.00	0.00	0.00	NaN
2108	ETFA	0.00	0.00	0.00	NaN
2109	ETFB	0.09	0.13	-0.05	0.40
2110	ETFDH	0.00	0.00	0.00	0.43
2113	ETS1	0.00	0.00	-0.10	-0.06
2114	ETS2	0.00	0.00	0.00	NaN
2115	ETV1	0.00	0.13	0.05	0.36
2116	ETV2	0.00	0.00	0.00	NaN
2117	ETV3	0.00	0.00	0.00	NaN
2119	ETV5	0.09	0.00	0.05	-0.09
2120	ETV6	0.00	0.13	0.10	0.09
2121	EVC	0.00	-0.13	-0.10	0.11
2122	EVI1	0.50	0.00	0.00	-0.05
2123	EVI2A	0.00	0.00	0.00	NaN
2124	EVI2B	0.00	0.00	0.00	NaN
2125	EVPL	0.00	0.13	0.00	0.15
2128	EVX1	0.00	0.13	0.00	0.39
2130	EWSR1	-0.09	0.00	0.00	0.33
2131	EXT1	0.00	0.38	0.10	0.37
2132	EXT2	0.00	0.00	0.00	NaN
2134	EXTL1	0.00	0.00	0.00	NaN
2135	EXTL2	0.09	0.00	0.00	0.30
2137	EXTL3	-0.09	-0.25	-0.05	0.37
2138	EYA1	0.00	0.00	0.00	NaN
2139	EYA2	0.00	0.00	0.00	NaN
2140	EYA3	0.00	0.00	0.00	NaN
2145	EZH1	0.00	-0.13	0.00	0.12
2146	EZH2	0.00	0.00	-0.05	0.15
2147	F2	0.00	0.00	0.00	NaN
2149	F2R	0.00	0.00	0.00	NaN

2150	F2RL1	0.00	0.00	0.00	NaN
2151	F2RL2	0.00	0.00	0.00	NaN
2152	F3	0.00	0.00	0.00	NaN
2153	F5	0.00	0.00	0.00	NaN
2155	F7	-0.09	-0.13	-0.05	0.08
2159	F10	-0.09	-0.13	-0.05	0.04
2160	F11	0.00	0.00	0.00	-0.05
2161	F12	0.00	0.13	0.00	0.24
2162	F13A1	0.00	0.00	0.00	NaN
2165	F13B	0.00	0.13	0.00	0.06
2166	FAAH	0.00	0.00	0.00	NaN
2167	FABP4	0.00	0.13	0.05	0.06
2168	FABP1	0.00	0.00	-0.05	0.18
2169	FABP2	0.00	0.00	0.00	NaN
2170	FABP3	0.00	0.00	0.00	NaN
2171	FABP5	0.00	0.00	0.05	0.16
2172	FABP6	0.00	0.00	0.00	NaN
2173	FABP7	0.00	0.00	0.00	NaN
2175	FANCA	0.00	0.00	0.00	0.40
2176	FANCC	0.00	0.00	0.00	NaN
2178	FANCE	0.00	0.00	0.00	NaN
2180	FACL2	0.00	0.00	-0.05	0.18
2181	FACL3	0.00	0.00	0.00	NaN
2185	PTK2B	-0.09	-0.25	-0.10	0.32
2186	FALZ	0.18	0.38	0.00	0.73
2188	FANCF	0.00	0.00	0.00	NaN
2189	FANCG	-0.09	0.13	0.10	0.63
2192	FBLN1	0.00	0.00	0.00	NaN
2193	FARSL	0.00	0.00	0.00	NaN
2195	FAT	0.00	0.00	0.00	0.34
2196	FAT2	0.00	0.00	0.00	NaN
2197	FAU	-0.09	0.00	0.00	0.36
2199	FBLN2	0.00	0.00	-0.05	0.13
2200	FBN1	0.00	0.00	0.00	NaN
2201	FBN2	0.00	0.00	0.00	NaN
2202	EFEMP1	0.00	0.00	0.00	NaN
2203	FBP1	0.00	0.00	0.00	NaN
2204	FCAR	0.09	0.00	0.00	0.30
2205	FCER1A	0.00	0.00	0.00	NaN
2207	FCER1G	0.00	0.00	0.00	NaN
2208	FCER2	0.00	0.00	0.00	NaN
2209	FCGR1A	0.00	0.00	0.10	0.02
2212	FCGR2A	0.00	0.00	0.00	NaN
2213	FCGR2B	0.09	0.00	0.00	0.01
2214	FCGR3A	0.00	0.00	0.05	-0.10
2215	FCGR3B	0.09	0.00	0.00	-0.09
2217	FCGRT	0.00	0.00	0.00	NaN

2218	FCMD	0.00	0.00	0.00	NaN
2219	FCN1	0.00	0.00	0.00	NaN
2220	FCN2	0.00	0.00	0.00	NaN
2222	FDFT1	-0.09	-0.50	-0.15	0.59
2224	FDPS	0.09	0.13	0.00	0.31
2230	FDX1	-0.09	0.00	0.00	0.39
2232	FDXR	0.00	0.13	0.00	0.31
2235	FECH	-0.09	0.00	-0.20	0.47
2237	FEN1	-0.09	0.00	0.00	0.19
2241	FER	0.00	0.00	0.00	NaN
2242	FES	0.00	0.00	0.00	NaN
2243	FGA	0.00	0.00	0.00	0.20
2244	FGB	0.00	0.00	0.00	0.01
2247	FGF2	0.00	0.00	0.00	NaN
2248	FGF3	0.55	0.25	0.00	0.09
2250	FGF5	0.00	0.00	0.00	NaN
2251	FGF6	-0.09	0.00	0.05	0.04
2252	FGF7	0.00	0.00	0.00	NaN
2253	FGF8	0.00	-0.13	-0.05	0.17
2254	FGF9	0.00	0.00	-0.10	-0.18
2259	FGF14	-0.09	-0.13	0.05	0.25
2260	FGFR1	0.50	0.00	0.50	0.56
2261	FGFR3	0.00	-0.13	-0.20	0.04
2262	GPC5	0.00	0.13	-0.05	0.05
2263	FGFR2	0.09	0.00	-0.05	0.66
2264	FGFR4	0.00	0.13	0.00	0.09
2266	FGG	0.00	0.00	0.00	0.04
2267	FGL1	0.00	-0.13	-0.05	0.01
2268	FGR	-0.09	0.00	0.00	0.23
2271	FH	0.00	0.00	0.00	NaN
2272	FHIT	0.00	0.00	-0.10	0.20
2274	FHL2	0.00	0.00	0.00	NaN
2275	FHL3	0.00	0.00	0.00	NaN
2280	FKBP1A	0.00	0.00	0.00	NaN
2281	FKBP1B	0.09	0.00	-0.05	0.04
2286	FKBP2	0.00	0.00	0.00	NaN
2287	FKBP3	0.00	0.00	0.00	NaN
2288	FKBP4	0.00	0.00	0.05	0.19
2289	FKBP5	0.00	0.00	0.00	NaN
2290	FOXG1B	0.00	0.00	0.00	NaN
2294	FOXF1	-0.09	-0.13	-0.05	0.12
2295	FOXF2	0.00	-0.13	-0.10	0.13
2297	FOXD1	0.00	0.00	0.00	NaN
2299	FOXI1	0.00	0.00	0.00	NaN
2301	FOXE3	0.00	0.00	0.00	NaN
2302	FOXJ1	0.00	0.13	0.00	-0.08
2303	FOXC2	-0.09	-0.13	-0.05	-0.23

2304	FOXE1	0.50	0.00	0.50	0.08
2305	FOXM1	0.00	0.00	0.50	0.21
2306	FOXD2	0.00	0.00	0.00	NaN
2308	FOXO1A	0.00	0.00	-0.05	0.13
2309	FOXO3A	0.00	0.00	0.10	0.27
2313	FLI1	-0.09	0.00	-0.10	0.25
2314	FLII	0.00	0.00	0.00	NaN
2315	MLANA	-0.09	-0.13	-0.05	0.06
2317	FLNB	0.50	0.00	0.00	0.38
2318	FLNC	0.00	0.25	0.00	0.04
2319	FLOT2	0.00	0.38	0.00	0.59
2322	FLT3	0.00	0.00	-0.05	0.08
2323	FLT3LG	0.00	0.00	0.00	NaN
2324	FLT4	0.00	0.13	0.00	-0.18
2326	FMO1	0.00	0.00	0.00	NaN
2327	FMO2	0.00	0.00	0.00	NaN
2328	FMO3	0.00	0.00	0.00	NaN
2329	FMO4	0.00	0.00	0.00	NaN
2330	FMO5	0.00	0.00	0.05	0.01
2331	FMOD	0.00	0.13	0.00	-0.11
2335	FN1	0.00	0.00	0.00	NaN
2339	FNTA	-0.09	0.00	-0.05	0.60
2342	FNTB	0.00	0.00	0.00	NaN
2346	FOLH1	0.00	0.00	0.00	NaN
2348	FOLR1	0.00	0.00	0.00	NaN
2350	FOLR2	0.00	0.00	0.00	NaN
2352	FOLR3	0.00	0.00	0.00	NaN
2353	FOS	0.00	0.00	0.00	NaN
2354	FOSB	0.09	0.00	0.00	0.09
2355	FOSL2	0.00	0.00	0.00	NaN
2356	FPGS	-0.09	0.00	-0.05	0.45
2357	FPR1	0.00	0.00	-0.05	0.05
2358	FPRL1	0.00	0.00	-0.05	0.24
2359	FPRL2	0.00	0.00	-0.05	-0.02
2395	FRDA	0.00	0.00	-0.05	-0.13
2444	FRK	-0.09	0.00	0.10	0.17
2475	FRAP1	0.00	0.00	0.00	NaN
2483	FRG1	0.00	0.00	0.05	0.48
2487	FRZB	0.00	0.00	0.00	NaN
2488	FSHB	0.00	0.00	0.00	NaN
2492	FSHR	0.00	0.00	0.00	NaN
2494	NR5A2	0.09	0.00	0.00	0.34
2495	FTH1	-0.09	0.00	0.00	0.16
2512	FTL	0.09	0.00	0.00	0.09
2515	ADAM2	0.27	-0.13	-0.05	0.05
2516	NR5A1	0.00	0.00	0.00	NaN
2517	FUCA1	-0.09	0.00	0.00	-0.17

2520	GAS	0.00	0.13	0.00	-0.14
2521	FUS	0.00	0.00	0.00	NaN
2523	FUT1	0.09	0.00	0.00	0.26
2524	FUT2	0.09	0.00	0.00	-0.02
2525	FUT3	0.00	0.00	0.00	NaN
2526	FUT4	-0.09	0.13	-0.05	0.26
2527	FUT5	0.00	0.00	0.00	NaN
2528	FUT6	0.00	0.00	0.00	NaN
2529	FUT7	0.00	0.00	0.00	NaN
2530	FUT8	0.00	0.00	0.00	NaN
2531	FVT1	-0.09	0.00	-0.15	0.29
2532	FY	0.00	0.00	0.00	NaN
2533	FYB	0.00	0.00	0.05	0.51
2534	FYN	0.50	0.00	0.50	0.42
2535	FZD2	0.00	-0.13	0.00	0.11
2537	G1P3	-0.09	0.00	0.00	0.16
2538	G6PC	0.00	0.00	0.00	0.37
2542	G6PT1	0.00	0.00	0.00	NaN
2547	G22P1	0.00	0.00	0.10	0.27
2548	GAA	0.00	0.25	0.00	-0.11
2549	GAB1	0.00	0.00	0.05	0.17
2550	GABBR1	0.00	0.00	0.00	NaN
2551	GABPA	0.00	0.00	0.00	NaN
2553	GABPB2	0.50	0.00	0.50	0.63
2554	GABRA1	0.00	0.00	0.00	NaN
2555	GABRA2	0.00	0.00	0.00	NaN
2557	GABRA4	0.00	0.00	0.00	NaN
2558	GABRA5	0.00	0.00	0.00	0.25
2559	GABRA6	0.00	0.00	0.00	NaN
2560	GABRB1	0.00	0.00	0.00	NaN
2561	GABRB2	0.00	0.00	0.00	NaN
2562	GABRB3	0.00	-0.13	0.00	0.17
2563	GABRD	0.00	0.00	0.00	NaN
2566	GABRG2	0.00	0.00	0.00	NaN
2567	GABRG3	0.00	0.00	0.00	-0.01
2568	GABRP	0.00	0.00	0.00	NaN
2569	GABRR1	0.00	0.00	0.00	NaN
2570	GABRR2	0.00	0.00	0.00	NaN
2571	GAD1	0.00	0.00	0.00	NaN
2572	GAD2	-0.09	0.00	0.00	0.16
2580	GAK	0.00	-0.13	-0.10	0.31
2581	GALC	0.00	0.13	0.00	-0.06
2582	GALE	-0.09	0.00	0.00	0.09
2583	GALGT	0.00	0.00	0.00	NaN
2584	GALK1	0.18	0.13	0.00	0.34
2585	GALK2	0.00	0.00	0.00	NaN
2587	GALR1	-0.09	0.00	-0.25	-0.01

2588	GALNS	-0.09	-0.13	-0.05	0.40
2589	GALNT1	0.00	0.00	-0.25	0.49
2590	GALNT2	0.09	0.00	0.00	-0.02
2591	GALNT3	0.00	0.00	0.00	NaN
2592	GALT	-0.09	0.13	0.10	0.45
2593	GAMT	0.00	0.00	0.00	NaN
2596	GAP43	0.00	0.00	0.00	NaN
2597	GAPD	-0.09	0.00	0.00	0.46
2615	GARP	0.27	0.00	0.00	0.30
2617	GARS	0.00	0.50	0.00	0.33
2618	GART	0.00	0.00	0.00	NaN
2619	GAS1	0.00	0.00	0.00	NaN
2620	GAS2	0.00	0.00	0.00	NaN
2621	GAS6	-0.09	-0.13	-0.05	0.11
2622	GAS11	-0.09	-0.13	-0.05	-0.01
2624	GATA2	0.00	0.00	0.00	NaN
2625	GATA3	0.00	0.00	0.05	-0.01
2626	GATA4	-0.09	-0.50	-0.15	0.27
2627	GATA6	0.00	0.00	-0.05	0.02
2628	GATM	0.00	0.00	0.00	NaN
2629	GBA	0.27	0.13	0.00	0.28
2631	GBAS	0.00	0.00	0.10	0.37
2632	GBE1	-0.09	0.00	0.05	0.41
2633	GBP1	0.00	0.00	0.00	NaN
2634	GBP2	0.00	0.00	0.00	NaN
2637	GBX2	0.00	0.00	-0.05	-0.01
2638	GC	0.00	0.00	0.00	NaN
2639	GCDH	0.00	0.00	0.00	NaN
2641	GCG	0.00	0.00	-0.05	0.11
2643	GCH1	0.00	0.00	0.05	0.20
2644	GCHFR	0.00	0.00	0.00	NaN
2645	GCK	0.00	0.00	0.00	NaN
2646	GCKR	0.09	0.13	0.05	-0.07
2647	GCN5L1	0.00	0.00	0.00	NaN
2648	GCN5L2	0.00	0.13	0.00	0.48
2649	NR6A1	0.00	0.00	0.00	NaN
2650	GCNT1	0.00	0.00	-0.05	0.11
2651	GCNT2	0.00	0.00	0.05	0.62
2653	GCSH	-0.09	0.00	-0.05	0.35
2657	GDF1	0.00	0.00	0.00	NaN
2658	GDF2	0.00	0.00	0.00	NaN
2660	GDF8	0.00	0.00	0.00	NaN
2661	GDF9	0.00	-0.13	0.00	-0.11
2662	GDF10	0.00	0.00	0.00	NaN
2665	GDI2	0.00	0.00	0.00	NaN
2668	GDNF	0.00	0.00	0.05	0.01
2669	GEM	0.00	-0.13	0.00	0.11

2670	GFAP	0.00	-0.13	0.00	0.16
2671	GFER	0.00	0.00	0.00	NaN
2672	GFI1	0.00	0.00	0.05	-0.12
2673	GFPT1	0.00	0.00	0.00	NaN
2674	GFRA1	0.00	0.00	-0.05	0.08
2675	GFRA2	-0.09	-0.38	-0.10	0.00
2676	GFRA3	0.00	0.00	0.00	NaN
2677	GGCX	0.00	0.00	0.00	NaN
2678	GGT1	0.00	0.00	0.00	NaN
2679	GGT2	0.00	0.00	-0.10	0.09
2683	B4GALT1	-0.09	0.00	0.05	0.37
2687	GGTLA1	0.00	0.00	0.00	NaN
2688	GH1	0.27	0.13	0.00	0.30
2689	GH2	0.27	0.13	0.00	0.07
2690	GHR	0.00	0.00	0.05	-0.13
2691	GHRH	0.00	-0.13	0.00	-0.04
2692	GHRHR	0.00	0.13	0.00	0.13
2693	GHSR	0.09	0.00	0.00	0.07
2694	GIF	0.00	0.00	0.00	NaN
2695	GIP	0.00	0.38	0.00	-0.06
2696	GIPR	0.09	0.00	0.00	-0.14
2697	GJA1	0.00	0.00	0.05	-0.09
2700	GJA3	0.00	0.00	-0.10	0.01
2701	GJA4	0.00	0.00	0.00	NaN
2702	GJA5	0.00	0.00	0.05	-0.19
2703	GJA8	0.00	0.00	0.05	-0.02
2707	GJB3	0.00	0.00	0.05	0.19
2709	GJB5	0.00	0.00	0.00	NaN
2720	GLB1	0.00	0.00	0.00	NaN
2729	GCLC	0.00	0.00	0.00	NaN
2730	GCLM	0.09	0.00	0.00	0.37
2731	GLDC	-0.09	-0.13	-0.10	0.15
2733	GLE1L	0.00	0.13	0.10	0.12
2734	GLG1	0.00	0.00	0.05	0.24
2735	GLI	0.00	0.00	0.00	NaN
2736	GLI2	0.00	0.00	0.00	NaN
2737	GLI3	0.00	0.00	0.00	NaN
2739	GLO1	0.00	0.00	0.00	NaN
2740	GLP1R	0.00	0.00	0.00	NaN
2741	GLRA1	0.00	0.00	0.00	NaN
2743	GLRB	0.00	0.13	0.00	0.01
2744	GLS	0.00	0.00	0.00	NaN
2745	GLRX	0.00	0.00	0.00	NaN
2746	GLUD1	0.09	0.00	-0.10	0.55
2752	GLUL	0.00	0.00	0.00	NaN
2760	GM2A	0.00	0.00	0.00	NaN
2762	GMDS	0.00	-0.13	-0.10	0.33

2764	GMFB	0.00	0.00	0.05	0.40
2765	GML	0.18	0.13	0.05	0.19
2766	GMPR	0.00	0.00	0.00	NaN
2767	GNA11	0.00	0.00	0.00	NaN
2769	GNA15	0.00	0.00	0.00	NaN
2770	GNAI1	0.00	0.00	0.00	NaN
2771	GNAI2	-0.09	0.00	-0.05	0.23
2773	GNAI3	0.09	0.00	0.05	0.71
2774	GNAL	0.00	0.00	0.00	NaN
2775	GNAO1	0.00	0.00	0.00	NaN
2776	GNAQ	0.00	0.00	-0.10	0.58
2778	GNAS	0.18	0.50	0.00	0.36
2779	GNAT1	-0.09	0.00	-0.10	0.06
2780	GNAT2	0.09	0.00	0.05	0.09
2781	GNAZ	0.00	0.00	0.00	NaN
2782	GNB1	0.00	0.00	0.00	NaN
2783	GNB2	0.18	0.00	0.00	0.36
2784	GNB3	-0.09	0.00	0.00	-0.31
2785	GNG3	0.00	0.00	0.00	NaN
2786	GNG4	0.18	0.00	0.00	0.30
2787	GNG5	0.00	0.00	0.05	0.22
2788	GNG7	0.00	0.00	0.00	NaN
2790	GNG10	0.00	0.00	0.00	NaN
2791	GNG11	0.18	0.00	0.00	-0.16
2792	GNGT1	0.18	0.00	0.00	0.62
2794	GNL1	0.00	0.00	0.00	NaN
2796	GNRH1	-0.09	-0.25	-0.05	0.11
2797	GNRH2	0.00	0.00	0.00	NaN
2798	GNRHR	0.00	0.00	0.00	NaN
2799	GNS	0.09	0.00	0.00	0.00
2800	GOLGA1	0.00	0.00	0.00	NaN
2801	GOLGA2	0.00	0.00	0.00	NaN
2802	GOLGA3	0.00	0.00	0.00	NaN
2803	GOLGA4	0.00	0.00	0.00	NaN
2804	GOLGB1	0.00	0.00	0.00	NaN
2805	GOT1	0.00	-0.13	-0.05	0.10
2806	GOT2	0.00	0.00	0.00	NaN
2810	SFN	-0.09	-0.13	0.00	0.14
2811	GP1BA	0.00	0.00	0.00	NaN
2812	GP1BB	0.00	0.00	0.00	0.23
2813	GP2	0.00	0.00	0.00	NaN
2814	GP5	0.09	0.00	0.00	0.06
2815	GP9	0.00	0.00	0.00	NaN
2817	GPC1	0.00	0.00	-0.05	0.12
2819	GPD1	0.00	0.00	0.00	NaN
2820	GPD2	0.00	0.00	-0.05	0.16
2821	GPI	0.00	0.50	0.50	0.19

2822	GPLD1	0.00	0.00	0.05	0.06
2823	GPM6A	0.00	0.13	-0.10	0.05
2825	GPR1	0.00	0.00	0.00	NaN
2827	GPR3	-0.09	0.00	0.00	-0.23
2828	GPR4	0.09	0.00	0.00	0.34
2829	CCXCR1	-0.09	0.00	-0.05	0.22
2832	GPR8	0.00	0.00	0.00	NaN
2835	GPR12	0.00	0.00	-0.05	0.09
2838	GPR15	0.00	0.00	0.00	NaN
2840	GPR17	0.00	0.00	-0.05	-0.04
2841	GPR18	-0.09	-0.13	-0.05	0.06
2842	GPR19	-0.09	0.00	0.00	0.08
2843	GPR20	0.18	0.00	0.05	-0.11
2844	GPR21	0.00	0.00	0.00	NaN
2845	GPR22	0.09	0.00	0.00	0.43
2847	GPR24	0.00	0.00	0.05	-0.10
2848	GPR25	0.00	0.00	0.00	NaN
2850	GPR27	-0.09	0.00	-0.05	0.29
2852	GPR30	0.00	0.00	0.05	-0.05
2853	GPR31	0.00	0.00	0.00	NaN
2854	GPR32	0.00	0.00	0.00	NaN
2859	GPR35	0.00	0.00	-0.05	0.07
2861	GPR37	0.00	0.13	0.00	0.15
2862	GPR38	0.00	0.13	0.00	-0.08
2863	GPR39	0.00	0.00	-0.05	0.08
2867	GPR43	0.00	0.00	0.00	NaN
2868	GPRK2L	0.00	-0.13	-0.15	0.27
2869	GPRK5	0.00	0.00	-0.05	0.16
2870	GPRK6	0.00	0.13	0.00	0.22
2872	GPRK7	0.00	0.00	0.00	NaN
2873	GPS1	0.00	0.00	0.00	NaN
2874	GPS2	0.00	0.00	0.00	NaN
2876	GPX1	-0.09	0.00	-0.05	-0.04
2877	GPX2	0.00	0.00	0.00	NaN
2878	GPX3	0.00	0.00	0.00	NaN
2879	GPX4	0.00	0.00	0.00	NaN
2880	GPX5	0.00	0.00	0.00	NaN
2885	GRB2	0.18	0.13	0.00	0.72
2886	GRB7	0.00	1.00	0.05	0.88
2887	GRB10	0.00	0.00	0.00	NaN
2888	GRB14	0.00	0.00	0.00	NaN
2889	GRF2	0.00	0.00	0.00	NaN
2890	GRIA1	0.00	0.00	0.00	NaN
2891	GRIA2	0.00	0.13	0.00	0.13
2893	GRIA4	0.00	0.00	0.00	NaN
2895	GRID2	0.00	0.00	0.00	NaN
2896	GRN	0.09	-0.13	0.00	0.17

2897	GRIK1	0.00	0.00	0.00	NaN
2899	GRIK3	0.00	0.00	0.00	NaN
2900	GRIK4	-0.09	0.00	-0.10	-0.03
2901	GRIK5	0.00	0.00	-0.05	0.12
2902	GRIN1	0.00	0.00	0.00	NaN
2903	GRIN2A	0.00	0.00	0.00	NaN
2904	GRIN2B	-0.09	0.00	0.00	0.19
2905	GRIN2C	0.00	0.13	0.00	0.10
2906	GRIN2D	0.09	0.00	0.00	0.03
2908	NR3C1	0.00	0.00	0.00	NaN
2909	GRLF1	0.09	0.00	0.00	0.64
2911	GRM1	0.00	-0.13	0.00	0.25
2912	GRM2	-0.09	0.00	-0.10	-0.07
2913	GRM3	0.00	0.00	0.00	NaN
2914	GRM4	0.00	0.00	0.00	NaN
2915	GRM5	0.00	0.00	0.05	0.19
2916	GRM6	0.00	0.00	0.00	NaN
2917	GRM7	0.00	0.00	0.00	NaN
2918	GRM8	0.00	0.25	0.00	0.25
2919	GRO1	0.00	0.00	0.00	NaN
2920	GRO2	0.00	0.00	0.00	NaN
2921	GRO3	0.00	0.00	0.00	NaN
2922	GRP	-0.09	0.00	-0.20	-0.05
2923	GRP58	0.00	0.00	0.00	NaN
2926	GRSF1	0.00	0.00	0.00	NaN
2928	GSCL	-0.09	0.00	0.00	-0.21
2931	GSK3A	0.00	0.00	-0.05	0.40
2932	GSK3B	0.00	0.00	0.00	NaN
2934	GSN	0.00	0.00	-0.10	0.46
2935	GSPT1	0.00	0.00	0.00	NaN
2936	GSR	-0.09	-0.25	-0.05	0.16
2937	GSS	0.00	0.13	0.00	0.43
2939	GSTA2	0.00	0.00	0.00	NaN
2940	GSTA3	0.00	0.00	0.00	NaN
2941	GSTA4	0.00	0.00	0.00	NaN
2944	GSTM1	0.55	0.38	0.40	0.28
2946	GSTM2	0.09	0.00	0.10	0.10
2947	GSTM3	0.09	0.00	0.05	-0.03
2948	GSTM4	0.09	0.00	0.05	0.50
2949	GSTM5	0.09	0.00	0.05	0.23
2950	GSTP1	0.27	0.00	0.05	-0.16
2952	GSTT1	-0.18	-0.25	-0.50	0.77
2953	GSTT2	0.00	0.13	0.00	-0.15
2954	GSTZ1	0.00	0.00	0.00	NaN
2956	MSH6	0.00	0.00	0.00	NaN
2957	GTF2A1	0.00	0.00	0.00	NaN
2958	GTF2A2	0.00	0.00	0.05	0.06

2960	GTF2E1	0.00	0.00	0.00	NaN
2961	GTF2E2	-0.09	-0.25	-0.05	0.51
2962	GTF2F1	0.00	0.00	0.00	NaN
2963	GTF2F2	0.00	0.00	-0.05	-0.22
2965	GTF2H1	0.00	0.00	0.00	NaN
2967	GTF2H3	0.00	0.00	0.00	NaN
2968	GTF2H4	0.00	0.00	0.00	NaN
2969	GTF2I	-0.09	0.00	0.00	0.24
2971	GTF3A	0.00	0.00	-0.05	0.44
2974	GUCY1B2	0.00	0.13	0.00	0.86
2975	GTF3C1	0.00	0.00	0.00	NaN
2976	GTF3C2	0.09	0.00	0.05	0.52
2977	GUCY1A2	0.00	0.00	0.00	NaN
2979	GUCA1B	0.00	0.00	0.10	-0.07
2981	GUCA2B	0.00	0.00	0.00	NaN
2982	GUCY1A3	0.00	0.00	0.00	0.00
2983	GUCY1B3	0.00	0.00	0.00	0.03
2984	GUCY2C	0.50	0.00	0.00	0.04
2987	GUK1	0.09	0.00	0.00	0.40
2990	GUSB	0.00	0.00	0.00	NaN
2992	GYG	0.00	0.00	0.05	0.41
2993	GYPA	0.00	0.00	0.00	NaN
2994	GYPB	0.00	0.00	0.00	NaN
2995	GYPC	0.00	0.00	0.00	NaN
2996	GYPE	0.00	0.00	0.00	NaN
2997	GYS1	0.09	0.00	0.00	0.15
2998	GYS2	0.00	0.00	0.00	NaN
3000	GUCY2D	0.00	0.00	0.00	NaN
3001	GZMA	0.00	0.00	-0.05	0.01
3003	GZMK	0.00	0.00	-0.05	-0.07
3004	GZMM	0.00	0.00	-0.05	0.05
3005	H1F0	-0.09	0.00	0.05	-0.14
3006	H1F2	0.00	0.00	0.05	0.28
3007	H1F3	0.00	0.00	0.05	-0.06
3008	H1F4	0.00	0.00	-0.05	0.02
3009	H1F5	0.00	0.00	0.00	NaN
3010	H1FT	0.00	0.00	-0.05	0.06
3012	H2AFA	0.00	0.00	0.50	0.14
3013	H2AFG	0.00	0.00	0.00	0.03
3014	H2AFX	-0.09	0.00	-0.05	0.32
3015	H2AFZ	0.00	0.00	0.00	NaN
3017	H2BFB	0.00	0.00	-0.05	0.13
3018	H2BFF	0.00	0.00	0.05	0.51
3024	H1F1	0.00	0.00	0.05	0.26
3026	HABP2	0.00	0.00	-0.05	-0.25
3029	HAGH	0.00	0.00	0.00	NaN
3030	HADHA	0.50	0.00	0.50	0.71

3032	HADHB	0.09	0.00	0.05	0.59
3034	HAL	0.00	0.13	0.00	-0.20
3035	HARS	0.00	0.00	0.00	NaN
3036	HAS1	0.00	0.00	0.00	0.35
3037	HAS2	0.18	0.25	0.10	-0.01
3039	HBA1	0.00	0.00	-0.05	-0.05
3040	HBA2	0.00	0.00	-0.05	-0.07
3043	HBB	0.00	-0.13	0.00	0.06
3045	HBD	0.00	-0.13	0.00	0.10
3046	HBE1	0.00	-0.13	0.00	0.05
3047	HBG1	0.00	-0.13	0.00	0.07
3049	HBQ1	0.00	0.00	-0.05	-0.23
3050	HBZ	0.00	0.00	-0.05	0.08
3053	SERPIND1	-0.09	0.13	0.00	-0.09
3055	HCK	0.00	0.13	0.05	-0.27
3059	HCLS1	0.00	0.00	0.00	NaN
3060	HCRT	0.00	0.50	0.00	-0.01
3061	HCRTR1	0.00	0.00	0.00	NaN
3062	HCRTR2	0.00	0.00	0.00	NaN
3064	HD	0.00	-0.13	-0.15	0.36
3065	HDAC1	0.00	0.00	0.00	NaN
3066	HDAC2	-0.09	0.00	0.10	0.67
3067	HDC	0.18	0.00	0.00	0.19
3068	HDGF	0.00	0.13	0.00	-0.32
3069	HDLBP	0.00	0.00	-0.05	0.40
3071	HEM1	0.00	0.00	0.00	NaN
3073	HEXA	0.00	0.00	0.00	NaN
3074	HEXB	0.00	0.00	0.00	NaN
3077	HFE	0.50	0.50	0.50	0.14
3080	HFL3	0.00	0.13	0.00	-0.18
3081	HGD	0.00	0.00	0.00	NaN
3082	HGF	0.09	0.00	0.00	0.04
3083	HGFAC	0.00	-0.13	-0.15	0.05
3084	NRG1	0.50	0.00	0.50	0.21
3087	HHEX	0.00	0.00	0.00	NaN
3090	HIC1	0.00	-0.13	0.00	0.14
3091	HIF1A	0.00	0.00	0.00	NaN
3092	HIP1	-0.09	0.00	0.00	0.18
3093	HIP2	0.00	0.13	0.00	0.33
3094	HINT1	0.00	-0.13	0.00	0.30
3096	HIVEP1	0.00	0.00	0.05	0.28
3098	HK1	0.00	0.00	0.00	NaN
3099	HK2	0.00	0.00	0.00	NaN
3101	HK3	0.00	0.13	0.00	0.09
3104	HKR3	0.00	0.00	0.00	NaN
3105	HLA-A	0.00	0.00	0.00	NaN
3106	HLA-B	0.00	0.00	0.00	NaN

3109	HLA-DMB	0.09	0.00	0.00	-0.04
3110	HLXB9	0.00	0.00	-0.05	0.00
3111	HLA-DOA	0.09	0.00	0.00	0.09
3112	HLA-DOB	0.09	0.00	0.00	-0.13
3113	HLA-DPA1	0.09	0.00	0.00	0.07
3115	HLA-DPB1	0.09	0.00	0.00	-0.03
3117	HLA-DQA1	0.00	0.00	-0.05	0.14
3119	HLA-DQB1	0.55	0.25	0.15	0.24
3122	HLA-DRA	0.00	0.00	0.00	NaN
3123	HLA-DRB1	0.27	0.00	0.15	0.13
3127	HLA-DRB5	0.09	0.13	0.10	0.14
3134	HLA-F	0.00	0.00	0.00	NaN
3135	HLA-G	0.00	0.00	0.00	NaN
3140	HLALS	0.00	0.00	0.00	NaN
3141	HLCS	0.00	0.00	0.00	NaN
3145	HMBS	-0.09	0.00	-0.05	0.42
3146	HMG1	0.00	0.00	0.00	0.38
3148	HMG2	0.00	0.00	-0.05	0.38
3150	HMG14	0.00	0.00	0.00	NaN
3155	HMGCL	-0.09	0.00	0.00	0.07
3156	HMGCR	0.00	0.00	0.00	NaN
3157	HMGCS1	0.00	0.00	0.05	0.08
3158	HMGCS2	0.18	0.13	0.05	-0.10
3159	HMGIY	0.00	0.00	0.00	NaN
3161	HMMR	0.00	0.00	0.00	NaN
3162	HMOX1	0.00	0.00	0.00	NaN
3163	HMOX2	0.00	0.00	0.00	NaN
3164	NR4A1	0.00	0.00	0.00	NaN
3169	HNF3A	0.18	0.00	0.00	0.20
3170	HNF3B	0.00	0.00	0.00	NaN
3172	HNF4A	0.09	0.00	0.00	0.30
3174	HNF4G	0.00	0.25	0.00	-0.01
3175	ONECUT1	0.09	0.00	0.00	0.03
3176	HNMT	0.00	0.00	0.00	NaN
3177	SLC29A2	0.00	0.00	0.05	-0.29
3178	HNRPA1	0.00	0.00	0.00	NaN
3181	HNRPA2B1	0.00	0.13	0.00	-0.33
3182	HNRPAB	0.00	0.00	0.00	NaN
3183	HNRPC	0.00	0.00	0.00	NaN
3184	HNRPD	0.00	0.00	0.00	NaN
3185	HNRPF	0.00	0.00	0.00	NaN
3187	HNRPH1	0.00	0.00	0.05	0.40
3189	HNRPH3	0.00	0.00	0.00	NaN
3190	HNRPK	0.00	0.00	0.00	NaN
3191	HNRPL	0.00	0.00	0.00	NaN
3192	HNRPU	0.09	0.13	0.00	-0.01
3195	HOX11	0.00	-0.13	-0.05	-0.15

3198	HOXA1	0.00	0.13	0.00	-0.08
3199	HOXA2	0.00	0.13	0.00	-0.17
3200	HOXA3	0.00	0.13	0.00	0.47
3201	HOXA4	0.00	0.13	0.00	0.07
3202	HOXA5	0.00	0.13	0.00	-0.16
3203	HOXA6	0.00	0.13	0.00	0.48
3204	HOXA7	0.00	0.13	0.00	0.23
3205	HOXA9	0.00	0.13	0.00	-0.03
3206	HOXA10	0.00	0.13	0.00	-0.05
3207	HOXA11	0.00	0.13	0.00	0.41
3211	HOXB1	0.00	0.38	0.00	0.09
3212	HOXB2	0.00	0.38	0.00	0.34
3213	HOXB3	0.00	0.38	0.00	0.22
3215	HOXB5	0.00	0.38	0.00	0.37
3216	HOXB6	0.00	0.38	0.00	0.40
3217	HOXB7	0.00	0.38	0.00	0.48
3218	HOXB8	0.00	0.38	0.00	-0.19
3221	HOXC4	0.00	0.00	0.00	NaN
3222	HOXC5	0.00	0.00	0.00	NaN
3223	HOXC6	0.55	0.38	0.25	0.03
3224	HOXC8	0.09	0.00	0.00	-0.08
3226	HOXC10	0.09	0.00	0.00	0.15
3227	HOXC11	0.00	0.00	0.00	NaN
3229	HOXC13	0.00	0.00	0.00	NaN
3231	HOXD1	0.00	0.00	0.00	NaN
3232	HOXD3	0.00	0.00	0.00	NaN
3233	HOXD4	0.00	0.00	0.00	NaN
3235	HOXD9	0.00	0.00	0.00	NaN
3236	HOXD10	0.00	0.00	0.00	NaN
3237	HOXD11	0.00	0.00	0.00	NaN
3238	HOXD12	0.00	0.00	0.00	NaN
3239	HOXD13	0.00	0.00	0.00	NaN
3240	HP	0.00	0.00	0.05	-0.18
3241	HPCAL1	0.00	0.00	-0.05	0.32
3242	HPD	0.00	-0.13	0.00	-0.11
3248	HPGD	0.00	0.00	-0.05	0.07
3249	HPN	0.00	0.00	0.00	NaN
3250	HPR	0.00	0.00	0.05	-0.01
3257	HPS1	0.00	0.00	-0.05	0.15
3263	HPX	0.00	-0.13	0.00	-0.02
3265	HRAS	-0.27	-0.13	-0.05	0.25
3267	HRB	0.00	0.00	0.00	NaN
3270	HRC	0.09	0.00	0.00	-0.03
3275	HRMT1L1	0.00	0.00	0.00	NaN
3276	HRMT1L2	0.00	0.00	0.00	NaN
3280	HRY	0.18	0.00	0.00	0.35
3281	HSBP1	-0.09	0.00	-0.05	0.49

3283	HSD3B1	0.09	0.00	0.05	0.64
3284	HSD3B2	0.09	0.00	0.05	0.00
3290	HSD11B1	0.00	0.00	0.00	NaN
3291	HSD11B2	-0.09	0.00	0.05	0.02
3292	HSD17B1	0.00	-0.13	0.00	0.00
3293	HSD17B3	0.00	0.00	0.00	NaN
3294	HSD17B2	-0.09	0.00	-0.05	0.15
3295	HSD17B4	0.00	0.00	0.00	NaN
3297	HSF1	0.18	0.00	0.10	0.33
3298	HSF2	0.00	0.00	0.05	0.73
3299	HSF4	-0.09	0.00	0.00	-0.26
3300	DNAJB2	0.00	0.00	0.00	NaN
3301	DNAJA1	-0.09	0.00	0.05	0.33
3303	HSPA1A	0.00	0.50	0.50	0.18
3305	HSPA1L	0.00	0.00	0.00	NaN
3306	HSPA2	0.09	0.00	0.00	-0.10
3308	HSPA4	0.00	-0.13	0.00	0.29
3309	HSPA5	0.00	0.00	0.00	NaN
3310	HSPA6	0.00	0.00	0.00	NaN
3312	HSPA8	0.00	0.00	-0.05	0.11
3313	HSPA9B	0.00	0.00	0.00	NaN
3315	HSPB1	-0.09	0.00	0.00	0.39
3316	HSPB2	0.00	0.00	0.00	NaN
3320	HSPCA	0.00	-0.13	0.00	0.51
3321	IGSF3	0.09	0.00	0.10	0.14
3326	HSPCB	0.00	0.00	0.00	NaN
3329	HSPD1	0.00	0.00	0.00	NaN
3336	HSPE1	0.00	0.00	0.00	NaN
3337	DNAJB1	0.00	0.00	0.00	NaN
3338	DNAJC4	0.00	0.00	0.00	NaN
3339	HSPG2	-0.09	0.00	0.00	-0.14
3340	NDST1	0.00	0.00	0.00	NaN
3344	HTLF	0.00	0.00	0.00	NaN
3346	HTN1	0.00	0.00	0.00	NaN
3347	HTN3	0.00	0.00	0.00	NaN
3350	HTR1A	0.00	0.00	0.00	NaN
3351	HTR1B	0.00	0.00	0.00	NaN
3352	HTR1D	-0.09	0.00	0.00	0.06
3354	HTR1E	0.00	0.00	0.00	NaN
3355	HTR1F	-0.09	0.00	0.05	0.15
3356	HTR2A	0.09	0.00	0.00	-0.08
3357	HTR2B	0.00	0.00	0.00	NaN
3359	HTR3A	0.00	0.00	0.00	NaN
3360	HTR4	0.00	0.00	0.00	NaN
3361	HTR5A	0.00	0.00	0.00	NaN
3363	HTR7	0.09	0.00	0.00	0.10
3364	HUS1	0.00	0.13	0.00	-0.01

3371	HXB	0.00	0.00	0.00	NaN
3373	HYAL1	0.00	0.00	0.00	-0.04
3375	IAPP	0.00	0.00	0.00	NaN
3376	IARS	0.00	0.00	0.00	NaN
3381	IBSP	0.00	0.00	0.00	NaN
3382	ICA1	0.00	0.00	0.05	0.05
3383	ICAM1	0.00	0.00	0.05	0.44
3384	ICAM2	0.27	0.13	0.00	0.03
3385	ICAM3	0.00	0.00	0.05	0.36
3386	ICAM4	0.00	0.00	0.05	-0.22
3394	ICSBP1	-0.09	-0.13	-0.05	-0.05
3396	ICT1	0.00	0.13	0.00	0.30
3397	ID1	0.00	0.00	0.05	0.34
3398	ID2	0.00	0.00	-0.05	0.14
3399	ID3	-0.09	0.00	0.00	0.11
3400	ID4	0.00	0.00	0.05	0.20
3416	IDE	0.00	0.00	0.00	NaN
3417	IDH1	0.00	0.00	0.00	NaN
3418	IDH2	0.00	0.00	0.00	NaN
3419	IDH3A	0.00	0.00	0.00	NaN
3420	IDH3B	0.00	0.00	0.05	0.41
3422	IDI1	0.00	0.00	0.00	NaN
3425	IDUA	0.00	-0.13	-0.05	0.54
3426	IF	0.00	0.00	0.00	NaN
3428	IFI16	0.00	0.00	0.00	NaN
3429	IFI27	0.00	0.00	0.00	NaN
3430	IFI35	0.00	-0.13	0.00	0.17
3431	SP110	0.00	0.00	0.00	NaN
3434	IFIT1	0.09	0.00	0.00	-0.14
3437	IFIT4	0.09	0.00	0.00	-0.15
3439	IFNA1	0.00	0.00	1.00	0.17
3440	IFNA2	0.00	-0.13	-0.15	0.02
3441	IFNA4	0.00	-0.13	-0.15	-0.08
3442	IFNA5	0.00	-0.13	-0.15	0.12
3443	IFNA6	0.00	-0.13	-0.15	0.11
3444	IFNA7	0.00	-0.13	-0.15	0.01
3445	IFNA8	0.00	-0.13	-0.15	-0.22
3446	IFNA10	0.00	-0.13	-0.15	-0.19
3448	IFNA14	0.00	-0.13	-0.15	0.00
3449	IFNA16	0.00	-0.13	-0.15	-0.09
3451	IFNA17	0.00	-0.13	-0.15	0.04
3452	IFNA21	0.00	-0.13	-0.15	-0.09
3454	IFNAR1	0.00	0.00	0.00	NaN
3455	IFNAR2	0.00	0.00	0.00	NaN
3456	IFNB1	0.00	-0.25	-0.15	-0.09
3458	IFNG	0.09	0.00	-0.05	-0.32
3459	IFNGR1	0.00	0.00	0.00	NaN

3460	IFNGR2	0.00	0.00	0.00	NaN
3467	IFNW1	0.00	-0.13	-0.15	-0.13
3475	IFRD1	0.09	0.00	0.00	0.21
3479	IGF1	0.00	0.00	0.00	NaN
3480	IGF1R	0.00	0.00	0.00	NaN
3481	IGF2	0.00	-0.13	0.00	-0.06
3482	IGF2R	0.00	0.00	0.00	NaN
3483	IGFALS	0.00	0.00	0.00	NaN
3484	IGFBP1	0.00	0.00	0.00	NaN
3485	IGFBP2	0.00	0.00	0.00	NaN
3486	IGFBP3	0.00	0.00	0.00	NaN
3487	IGFBP4	0.00	0.38	0.00	-0.06
3488	IGFBP5	0.00	0.00	0.00	NaN
3489	IGFBP6	0.09	0.00	0.00	0.03
3490	IGFBP7	0.00	0.00	0.05	0.22
3491	CYR61	0.00	0.00	0.10	0.49
3508	IGHMBP2	0.45	0.00	0.00	0.56
3543	IGLL1	0.00	0.00	0.05	-0.12
3549	IHH	0.00	0.00	0.00	NaN
3550	IK	0.00	0.00	0.00	NaN
3551	IKBKB	0.36	-0.13	-0.05	0.50
3552	IL1A	0.00	0.00	0.00	NaN
3553	IL1B	0.00	0.00	0.00	NaN
3554	IL1R1	0.00	0.00	0.00	NaN
3556	IL1RAP	0.00	0.00	0.05	0.56
3557	IL1RN	0.00	0.00	0.00	NaN
3558	IL2	0.00	0.00	0.00	NaN
3559	IL2RA	0.00	0.00	0.00	NaN
3560	IL2RB	0.00	0.00	0.00	NaN
3562	IL3	0.00	-0.13	0.00	-0.51
3565	IL4	0.00	-0.13	0.00	-0.10
3566	IL4R	0.00	0.00	0.00	NaN
3567	IL5	0.00	-0.13	0.00	0.05
3568	IL5RA	0.00	-0.13	0.00	0.15
3569	IL6	0.00	0.13	0.00	-0.06
3570	IL6R	0.09	0.13	0.00	0.21
3572	IL6ST	0.00	0.00	0.00	NaN
3574	IL7	0.00	0.25	0.05	0.08
3575	IL7R	0.00	0.00	0.10	0.25
3576	IL8	0.00	0.00	0.00	NaN
3577	IL8RA	0.00	0.00	0.00	NaN
3578	IL9	0.00	-0.13	0.00	0.08
3579	IL8RB	0.00	0.00	0.00	NaN
3586	IL10	0.00	0.13	0.00	-0.09
3587	IL10RA	-0.09	0.00	0.00	0.10
3588	IL10RB	0.00	0.00	0.00	NaN
3589	IL11	0.09	0.00	0.00	0.01

3590	IL11RA	-0.09	0.13	0.10	0.29
3592	IL12A	0.00	0.00	0.00	NaN
3593	IL12B	0.00	0.00	0.00	NaN
3594	IL12RB1	0.00	0.00	0.00	NaN
3595	IL12RB2	0.00	0.00	0.00	NaN
3596	IL13	0.00	-0.13	0.00	-0.25
3600	IL15	0.00	0.00	0.00	NaN
3601	IL15RA	0.00	0.00	0.00	NaN
3603	IL16	0.00	0.00	0.00	NaN
3604	TNFRSF9	0.00	0.00	0.00	NaN
3605	IL17	0.00	0.00	0.00	NaN
3606	IL18	0.00	0.00	0.00	NaN
3607	ILF1	0.00	0.00	0.00	NaN
3608	ILF2	0.09	0.13	0.00	0.16
3609	ILF3	0.00	0.50	0.50	0.39
3611	ILK	0.00	-0.13	0.00	0.36
3612	IMPA1	0.00	0.00	0.05	0.38
3613	IMPA2	0.00	0.00	0.00	NaN
3614	IMPDH1	0.00	0.25	0.00	0.60
3615	IMPDH2	-0.09	0.00	-0.05	0.41
3617	IMPG1	0.00	0.00	0.05	-0.01
3619	INCENP	-0.09	0.00	0.00	0.28
3620	INDO	0.36	-0.13	-0.05	-0.02
3621	ING1	-0.09	-0.13	0.00	0.29
3622	ING1L	0.00	0.00	-0.05	0.38
3623	INHA	0.00	0.00	0.00	NaN
3624	INHBA	0.00	0.00	0.00	NaN
3625	INHBB	0.00	0.00	0.00	NaN
3627	SCYB10	0.00	0.00	0.00	NaN
3628	INPP1	0.00	0.00	0.00	NaN
3630	INS	0.00	-0.13	0.00	-0.05
3631	INPP4A	0.00	0.00	0.00	NaN
3632	INPP5A	0.00	0.00	-0.05	0.41
3633	INPP5B	0.00	0.00	0.00	NaN
3635	INPP5D	0.00	0.00	0.00	NaN
3636	INPPL1	0.00	0.00	0.00	NaN
3638	INSIG1	0.00	0.00	0.05	0.18
3640	INSL3	0.00	0.00	0.00	NaN
3641	INSL4	-0.09	-0.13	-0.05	0.36
3642	INSM1	0.00	0.00	0.00	NaN
3643	INSR	0.00	0.00	0.00	NaN
3645	INSRR	0.00	0.00	0.00	NaN
3646	EIF3S6	0.18	-0.13	0.00	0.58
3651	IPF1	0.00	0.00	-0.05	0.13
3652	IPP	0.00	0.00	0.00	NaN
3653	IPW	0.00	0.00	0.00	NaN
3655	ITGA6	0.00	0.00	0.00	NaN

3658	IREB2	0.00	0.00	0.00	NaN
3659	IRF1	0.00	-0.13	0.00	0.11
3660	IRF2	0.00	0.00	-0.05	0.10
3661	IRF3	0.00	0.00	0.00	NaN
3662	IRF4	0.00	0.00	0.00	0.02
3663	IRF5	0.00	0.25	0.00	0.17
3665	IRF7	-0.27	-0.13	-0.05	0.24
3667	IRS1	0.00	0.00	0.00	NaN
3669	ISG20	0.00	0.00	-0.05	0.16
3670	ISL1	0.00	0.00	0.00	NaN
3671	ISLR	0.00	0.00	0.00	NaN
3673	ITGA2	0.00	0.00	-0.05	0.03
3674	ITGA2B	0.00	-0.13	0.00	0.04
3675	ITGA3	0.00	0.25	0.00	0.25
3676	ITGA4	0.00	0.00	0.00	NaN
3678	ITGA5	0.00	0.00	0.00	NaN
3679	ITGA7	0.00	0.00	0.00	NaN
3680	ITGA9	0.00	0.00	0.00	NaN
3682	ITGAE	0.00	0.00	0.00	NaN
3684	ITGAM	0.00	0.00	0.00	NaN
3685	ITGAV	0.00	0.00	0.00	NaN
3687	ITGAX	0.00	0.00	0.00	NaN
3688	ITGB1	0.00	-0.13	0.05	0.38
3689	ITGB2	0.00	0.00	0.00	NaN
3690	ITGB3	0.00	-0.13	0.00	0.40
3691	ITGB4	0.18	0.13	0.00	0.19
3692	ITGB4BP	0.00	0.50	0.00	0.28
3693	ITGB5	0.00	0.00	0.00	NaN
3694	ITGB6	0.00	0.00	-0.10	0.03
3695	ITGB7	0.00	0.00	0.00	NaN
3696	ITGB8	0.00	0.13	0.00	-0.01
3698	ITIH2	0.00	0.00	0.00	NaN
3699	ITIH3	-0.09	0.00	-0.10	0.13
3700	ITIH4	-0.09	0.00	-0.10	0.17
3702	ITK	0.00	0.00	0.00	NaN
3703	ITM1	-0.09	0.00	-0.10	0.49
3704	ITPA	0.00	0.00	0.00	NaN
3705	ITPK1	0.00	0.00	0.00	NaN
3706	ITPKA	0.00	0.00	0.00	NaN
3707	ITPKB	0.09	0.00	0.00	0.44
3708	ITPR1	0.00	0.00	-0.05	0.03
3709	ITPR2	0.00	0.00	0.00	NaN
3710	ITPR3	0.00	0.00	0.00	NaN
3712	IVD	0.00	0.00	0.00	NaN
3713	IVL	0.00	0.00	0.00	NaN
3714	JAG2	0.00	0.00	0.00	NaN
3716	JAK1	0.00	0.00	0.05	-0.17

3717	JAK2	-0.09	-0.13	-0.10	0.59
3718	JAK3	0.00	0.00	0.00	NaN
3720	JMJ	0.00	0.00	0.05	0.12
3725	JUN	0.00	0.00	-0.05	0.19
3726	JUNB	0.00	0.00	0.00	NaN
3727	JUND	0.00	0.00	0.00	NaN
3728	JUP	0.00	0.13	0.00	0.18
3732	KAI1	0.00	0.00	0.00	NaN
3735	KARS	0.00	-0.13	0.05	0.37
3736	KCNA1	-0.09	0.00	0.05	0.06
3737	KCNA2	0.09	0.00	0.00	0.14
3738	KCNA3	0.09	0.00	0.00	0.11
3739	KCNA4	0.00	0.00	0.00	NaN
3741	KCNA5	-0.09	0.00	0.05	-0.05
3742	KCNA6	-0.09	0.00	0.05	0.24
3744	KCNA10	0.09	0.00	0.00	-0.08
3745	KCNB1	0.18	0.25	0.05	0.18
3746	KCNC1	0.00	0.00	0.00	NaN
3748	KCNC3	0.00	0.00	0.00	NaN
3749	KCNC4	0.09	0.00	0.00	-0.01
3751	KCND2	0.00	0.13	0.00	-0.04
3752	KCND3	0.09	0.00	0.00	0.04
3753	KCNE1	0.00	0.00	0.00	NaN
3754	KCNF1	0.00	0.00	-0.05	0.18
3755	KCNG1	0.09	0.13	0.05	-0.02
3756	KCNH1	0.00	0.00	0.00	NaN
3757	KCNH2	0.00	0.00	-0.05	0.23
3758	KCNJ1	-0.09	0.00	-0.10	0.06
3759	KCNJ2	0.00	0.13	0.00	-0.07
3760	KCNJ3	0.00	0.00	0.00	NaN
3761	KCNJ4	-0.09	0.00	0.05	-0.19
3762	KCNJ5	0.00	0.00	0.00	-0.01
3763	KCNJ6	0.00	0.00	0.00	NaN
3764	KCNJ8	0.00	0.00	0.00	NaN
3765	KCNJ9	0.00	0.00	0.00	NaN
3766	KCNJ10	0.00	0.00	0.00	NaN
3768	KCNJ12	0.00	-0.13	0.00	0.05
3769	KCNJ13	0.00	0.00	0.00	NaN
3770	KCNJ14	0.09	0.00	0.00	-0.04
3772	KCNJ15	0.00	0.00	0.00	NaN
3773	KCNJ16	0.00	0.13	0.00	0.02
3775	KCNK1	0.09	0.00	0.00	0.26
3776	KCNK2	0.09	0.00	0.00	0.00
3777	KCNK3	0.09	0.00	0.05	0.01
3778	KCNMA1	0.09	0.00	0.00	-0.09
3779	KCNMB1	0.00	0.00	0.00	NaN
3780	KCNN1	0.00	0.00	0.00	NaN

3781	KCNN2	0.00	0.00	0.00	NaN
3782	KCNN3	0.09	0.25	0.00	0.00
3783	KCNN4	0.09	0.00	-0.10	0.31
3784	KCNQ1	0.00	-0.13	0.00	-0.07
3785	KCNQ2	0.09	0.00	0.00	0.16
3786	KCNQ3	0.00	-0.25	0.05	-0.02
3787	KCNS1	0.09	0.13	0.00	0.24
3790	KCNS3	0.09	0.00	-0.05	0.57
3791	KDR	0.00	0.00	0.00	NaN
3792	KEL	0.00	0.00	-0.05	0.13
3795	KHK	0.09	0.00	0.05	0.47
3796	KIF2	0.00	0.00	0.00	NaN
3797	KIF3C	0.09	0.00	0.05	0.50
3798	KIF5A	0.00	0.00	0.00	NaN
3799	KIF5B	-0.09	-0.13	0.05	0.61
3800	KIF5C	0.00	0.00	0.00	NaN
3801	KIFC3	-0.09	0.00	0.00	0.01
3802	KIR2DL1	0.09	0.13	-0.05	0.01
3805	KIR2DL4	0.09	0.00	0.00	0.25
3809	KIR2DS4	0.09	0.00	0.00	0.07
3811	KIR3DL1	0.09	0.00	0.00	0.10
3812	KIR3DL2	0.09	0.00	0.00	0.16
3814	KISS1	0.00	0.13	0.00	-0.13
3815	KIT	0.00	0.00	0.00	NaN
3817	KLK2	0.00	0.00	0.00	NaN
3818	KLKB1	0.00	0.00	0.00	0.16
3820	KLRB1	-0.09	0.00	0.00	0.12
3822	KLRC2	-0.09	-0.50	-0.05	0.10
3823	KLRC3	-0.09	0.00	0.00	-0.06
3824	KLRD1	-0.09	0.00	0.00	0.18
3827	KNG	0.09	0.00	0.05	-0.09
3831	KNS2	0.00	0.00	0.00	NaN
3832	KNSL1	0.00	0.00	0.00	NaN
3833	KNSL2	0.09	0.00	0.05	0.28
3834	KNSL3	0.00	0.00	0.00	NaN
3836	KPNA1	0.00	0.00	0.00	NaN
3837	KPNB1	0.00	-0.13	0.00	0.22
3838	KPNA2	0.18	0.38	0.00	0.43
3839	KPNA3	0.00	0.00	0.00	NaN
3840	KPNA4	0.00	0.00	0.00	NaN
3841	KPNA5	-0.09	0.00	0.10	0.62
3842	KPNB2	0.00	0.00	0.00	NaN
3843	KPNB3	-0.09	0.00	-0.10	0.23
3845	KRAS2	0.00	0.00	0.00	NaN
3846	KRN1	0.00	0.00	0.00	-0.05
3848	KRT1	0.00	0.00	0.00	NaN
3849	KRT2A	0.00	0.00	0.00	NaN

3851	KRT4	0.00	0.00	0.00	NaN
3852	KRT5	0.00	0.00	0.00	NaN
3853	KRT6A	0.00	0.00	0.00	NaN
3854	KRT6B	0.00	0.00	0.00	NaN
3855	KRT7	0.00	0.00	0.00	NaN
3856	KRT8	0.00	0.00	-0.05	0.19
3857	KRT9	0.00	0.13	0.00	0.02
3858	KRT10	0.00	0.25	0.00	0.39
3859	KRT12	0.00	0.25	0.05	0.12
3860	KRT13	0.00	0.13	0.00	0.00
3861	KRT14	0.00	0.13	0.00	-0.08
3866	KRT15	0.00	0.13	0.00	0.08
3868	KRT16	0.00	0.13	0.00	-0.07
3872	KRT17	0.50	0.50	0.50	-0.08
3875	KRT18	0.00	0.00	0.00	NaN
3880	KRT19	0.00	0.13	0.05	0.18
3881	KRTHA1	0.00	0.13	0.00	0.02
3882	KRTHA2	0.00	0.13	0.00	-0.03
3883	KRTHA3A	0.00	0.25	0.00	0.01
3884	KRTHA3B	0.00	0.25	-0.05	0.30
3885	KRTHA4	0.00	0.13	0.00	0.07
3886	KRTHA5	0.00	0.13	0.00	-0.01
3887	KRTHB1	0.00	0.00	0.00	NaN
3889	KRTHB3	0.00	0.00	0.00	NaN
3891	KRTHB5	0.00	0.00	0.00	NaN
3892	KRTHB6	0.00	0.00	0.00	NaN
3895	KTN1	0.00	0.00	0.05	0.22
3898	LAD1	0.00	0.00	0.00	NaN
3899	LAF4	0.00	0.00	0.00	NaN
3902	LAG3	-0.09	0.00	0.00	-0.03
3903	LAIR1	0.09	0.00	-0.05	0.02
3904	LAIR2	0.09	0.00	0.00	-0.19
3906	LALBA	0.00	0.00	0.00	NaN
3908	LAMA2	0.00	0.00	0.05	-0.02
3909	LAMA3	0.00	0.00	-0.15	-0.04
3910	LAMA4	0.00	0.00	0.10	0.25
3911	LAMA5	0.09	0.13	0.00	0.16
3912	LAMB1	0.09	0.00	0.00	0.00
3913	LAMB2	-0.09	0.00	-0.05	0.14
3914	LAMB3	0.00	0.00	0.00	NaN
3915	LAMC1	0.00	0.00	0.00	NaN
3916	LAMP1	-0.09	-0.13	-0.05	0.41
3918	LAMC2	0.00	0.00	0.00	NaN
3925	STMN1	-0.09	0.00	0.00	0.13
3927	LASP1	0.00	0.38	0.00	0.68
3930	LBR	0.09	0.00	0.00	-0.15
3931	LCAT	-0.09	-0.13	0.00	0.11

3932	LCK	0.00	0.00	0.00	NaN
3933	LCN1	0.00	0.00	0.00	NaN
3934	LCN2	-0.09	0.00	0.00	0.11
3937	LCP2	0.00	0.00	0.05	0.12
3938	LCT	0.00	0.00	-0.05	-0.04
3939	LDHA	0.00	0.00	0.00	NaN
3945	LDHB	0.00	0.00	0.00	NaN
3948	LDHC	0.00	0.00	0.00	NaN
3949	LDLR	0.00	0.00	0.00	NaN
3950	LECT2	0.00	-0.13	0.00	-0.25
3952	LEP	0.00	0.25	0.00	-0.04
3953	LEPR	0.00	0.00	-0.05	0.31
3954	LETM1	0.00	-0.13	-0.20	0.44
3955	LFNG	0.00	0.13	0.10	-0.19
3956	LGALS1	-0.09	0.00	0.05	0.17
3957	LGALS2	-0.09	0.00	0.05	-0.03
3958	LGALS3	0.00	0.00	0.05	-0.07
3959	LGALS3BP	0.00	0.25	0.00	0.44
3960	LGALS4	0.00	0.00	0.00	NaN
3963	LGALS7	0.00	0.00	0.00	NaN
3964	LGALS8	0.09	0.13	0.00	0.17
3965	LGALS9	0.00	0.13	0.00	0.34
3972	LHB	0.09	0.00	0.00	-0.05
3973	LHCGR	0.00	0.00	0.00	NaN
3975	LHX1	0.00	-0.13	0.00	0.05
3976	LIF	0.00	0.00	0.00	NaN
3977	LIFR	0.00	0.00	0.05	0.20
3978	LIG1	0.09	0.00	0.00	0.16
3980	LIG3	0.00	-0.13	0.00	0.24
3981	LIG4	-0.09	-0.13	0.00	0.19
3982	LIM2	0.00	0.13	-0.05	0.10
3983	ABLIM	0.00	0.00	-0.05	0.21
3984	LIMK1	-0.09	0.00	0.00	0.27
3985	LIMK2	0.00	0.00	0.00	NaN
3987	LIMS1	0.00	0.00	0.00	NaN
3990	LIPC	1.00	0.00	0.50	0.09
3991	LIPE	0.09	0.00	-0.05	0.01
3992	FADS1	0.00	0.50	0.00	0.22
3993	LLGL2	0.18	0.13	0.05	0.38
3995	FADS3	-0.09	0.00	0.00	0.19
3996	LLGL1	0.00	0.00	0.00	NaN
3998	LMAN1	-0.09	0.00	-0.20	0.27
4000	LMNA	0.00	0.13	0.00	0.27
4001	LMNB1	0.00	0.00	0.00	NaN
4004	LMO1	0.00	0.00	-0.05	0.12
4005	LMO2	0.00	0.00	0.05	0.58
4008	LMO7	-0.09	0.13	-0.15	0.11

4010	LMX1B	0.09	0.00	-0.05	0.54
4012	LNPEP	0.00	0.00	0.00	NaN
4013	LOH11CR2A	0.00	0.00	-0.10	0.23
4014	LOR	0.00	0.00	0.00	NaN
4015	LOX	0.00	0.00	0.00	NaN
4016	LOXL1	0.00	0.00	0.00	NaN
4017	LOXL2	-0.09	-0.25	-0.15	0.27
4018	LPA	0.00	0.00	0.00	NaN
4025	LPO	0.09	-0.13	0.00	-0.20
4026	LPP	0.00	0.00	0.05	0.17
4033	LRMP	0.00	0.00	0.00	NaN
4034	LRRN1	0.33	0.00	0.00	0.39
4035	LRP1	0.00	0.00	0.00	NaN
4036	LRP2	0.00	0.00	0.00	NaN
4037	LRP3	0.00	0.00	0.05	0.07
4038	LRP4	0.00	0.00	0.00	NaN
4040	LRP6	-0.09	0.00	0.10	0.26
4041	LRP5	0.55	0.00	0.00	0.30
4043	LRPAP1	0.00	0.00	0.00	0.17
4045	LSAMP	0.00	0.00	0.00	NaN
4046	LSP1	0.00	-0.13	-0.05	0.11
4047	LSS	0.00	0.00	0.00	NaN
4048	LTA4H	0.00	0.13	0.00	0.13
4049	LTA	0.00	0.00	0.00	NaN
4050	LTB	0.00	0.00	0.00	NaN
4051	CYP4F3	0.00	0.00	0.00	NaN
4052	LTBP1	0.00	0.00	0.00	NaN
4053	LTBP2	0.00	0.00	0.50	0.05
4055	LTBR	-0.09	0.00	0.00	0.22
4056	LTC4S	0.00	0.00	0.00	NaN
4057	LTF	-0.09	0.00	-0.05	0.08
4058	LTK	0.00	0.00	0.00	NaN
4060	LUM	0.00	0.00	0.00	NaN
4061	LY6E	0.18	0.13	0.05	0.33
4062	LY6H	0.27	0.00	0.05	-0.08
4063	LY9	0.00	0.00	0.00	NaN
4064	LY64	0.00	0.00	0.00	NaN
4065	LY75	0.00	0.00	-0.10	0.06
4066	LYL1	0.00	0.00	0.00	NaN
4067	LYN	0.00	0.00	0.00	NaN
4070	TACSTD2	0.00	0.00	-0.05	0.02
4071	TM4SF1	0.00	0.00	0.05	0.21
4072	TACSTD1	0.00	0.00	0.00	NaN
4074	M6PR	-0.09	0.00	0.00	0.31
4076	M11S1	0.00	0.00	0.05	0.34
4077	M17S2	0.00	-0.13	0.00	0.30
4081	MAB21L1	0.00	0.00	0.00	NaN

4082	MARCKS	-0.09	0.00	0.10	0.34
4084	MAD	0.00	0.00	0.00	NaN
4085	MAD2L1	0.00	0.00	0.00	NaN
4087	MADH2	-0.09	0.00	-0.10	0.63
4088	MADH3	0.00	0.00	0.00	NaN
4089	MADH4	-0.09	-0.13	-0.25	0.52
4090	MADH5	0.00	-0.13	0.00	0.16
4091	MADH6	0.00	0.00	0.00	NaN
4092	MADH7	-0.09	-0.13	-0.15	0.45
4093	MADH9	0.00	0.00	0.00	NaN
4094	MAF	0.00	0.00	0.05	0.07
4097	MAFG	0.00	0.00	0.05	0.08
4099	MAG	0.00	0.00	0.00	NaN
4117	MAK	0.00	0.00	0.05	0.46
4121	MAN1A1	0.00	0.00	0.05	0.66
4122	MAN2A2	0.00	0.00	0.00	NaN
4123	MAN2C1	0.00	0.00	0.00	NaN
4124	MAN2A1	0.00	0.13	0.00	0.22
4125	MAN2B1	0.00	0.00	0.00	NaN
4126	MANBA	0.00	0.00	0.00	NaN
4130	MAP1A	0.00	0.00	0.00	NaN
4131	MAP1B	0.00	0.00	0.00	NaN
4133	MAP2	0.00	0.00	0.00	NaN
4134	MAP4	-0.09	0.00	-0.05	0.35
4137	MAPT	0.00	-0.13	0.00	-0.04
4139	MARK1	0.09	0.00	0.05	0.43
4140	MARK3	0.00	0.00	0.00	NaN
4141	MARS	0.00	0.00	0.00	NaN
4142	MAS1	0.00	0.00	0.00	NaN
4143	MAT1A	0.09	0.00	0.00	0.03
4144	MAT2A	0.00	0.00	0.00	NaN
4145	MATK	0.00	0.00	0.00	NaN
4146	MATN1	0.00	0.00	0.00	NaN
4147	MATN2	0.00	0.00	0.00	NaN
4148	MATN3	0.09	0.00	-0.05	0.67
4149	MAX	0.00	0.00	0.00	NaN
4150	MAZ	0.00	0.00	0.00	NaN
4151	MB	0.00	0.00	0.00	-0.01
4152	MBD1	0.00	-0.13	-0.05	0.07
4153	MBL2	0.00	0.00	0.00	-0.15
4154	MBNL	0.00	0.00	0.00	NaN
4157	MC1R	-0.09	-0.13	-0.05	-0.23
4158	MC2R	0.00	0.00	0.00	NaN
4159	MC3R	0.09	0.50	0.00	-0.16
4160	MC4R	0.00	0.00	-0.15	-0.23
4161	MC5R	0.00	0.00	0.00	NaN
4162	MCAM	-0.09	0.00	-0.10	0.30

4163	MCC	0.00	0.00	0.00	NaN
4166	CHST6	0.00	-0.13	0.05	0.29
4170	MCL1	0.00	0.13	0.05	0.35
4171	MCM2	0.00	0.00	0.00	NaN
4172	MCM3	0.00	0.00	0.00	NaN
4173	MCM4	0.00	0.00	0.00	NaN
4174	MCM5	0.00	0.00	0.00	NaN
4175	MCM6	0.00	0.00	-0.05	0.57
4176	MCM7	0.18	0.00	0.00	0.22
4179	MCP	0.09	0.13	0.00	0.30
4184	MCSP	0.00	0.00	0.00	NaN
4185	ADAM11	0.00	-0.13	0.00	-0.02
4188	MDFI	0.00	0.00	0.05	-0.10
4189	DNAJB9	0.09	0.00	0.00	0.00
4190	MDH1	0.00	0.00	0.00	NaN
4191	MDH2	-0.09	0.00	0.00	0.46
4192	MDK	0.00	0.00	0.00	NaN
4193	MDM2	0.09	0.13	0.00	0.28
4194	MDM4	0.00	0.13	0.00	0.41
4199	ME1	0.00	0.00	0.00	NaN
4200	ME2	-0.09	-0.13	-0.25	0.40
4201	MEA	0.00	0.00	0.00	NaN
4205	MEF2A	0.00	0.00	0.00	NaN
4207	MEF2B	0.00	0.00	0.00	NaN
4208	MEF2C	0.00	-0.13	0.00	0.10
4209	MEF2D	0.00	0.13	0.00	0.09
4210	MEFV	0.00	0.00	0.00	NaN
4211	MEIS1	0.00	0.00	0.00	NaN
4212	MEIS2	0.00	0.00	0.00	NaN
4214	MAP3K1	0.00	0.00	0.00	NaN
4215	MAP3K3	0.27	0.13	0.00	0.53
4216	MAP3K4	0.00	0.00	0.00	NaN
4217	MAP3K5	0.00	0.00	0.00	NaN
4218	MEL	0.00	0.13	0.00	-0.24
4221	MEN1	0.00	0.50	0.00	0.24
4222	MEOX1	0.00	-0.13	0.00	-0.32
4223	MEOX2	0.00	0.13	0.05	0.19
4224	MEP1A	0.00	0.00	0.00	NaN
4225	MEP1B	0.00	0.00	-0.15	-0.09
4232	MEST	0.00	0.25	0.05	0.02
4233	MET	0.00	0.25	0.00	0.11
4234	METTL1	0.00	0.00	0.00	NaN
4236	MFAP1	0.00	0.00	0.00	NaN
4237	MFAP2	-0.09	0.00	0.00	0.10
4238	MFAP3	0.00	0.00	0.00	NaN
4239	MFAP4	0.00	0.00	0.00	NaN
4240	MFGE8	0.00	0.00	-0.05	0.14

4241	MFI2	0.50	0.00	0.50	0.05
4242	MFNG	-0.09	0.00	0.05	0.08
4245	MGAT1	0.00	0.13	0.00	0.52
4246	SCGB2A1	-0.09	0.00	0.00	-0.51
4247	MGAT2	0.00	0.00	0.00	NaN
4248	MGAT3	-0.09	0.00	0.05	-0.08
4249	MGAT5	0.00	0.00	-0.05	-0.08
4250	SCGB2A2	-0.09	0.00	0.00	-0.35
4253	MGEA6	0.00	0.00	0.00	NaN
4254	KITLG	0.09	0.13	0.00	-0.09
4255	MGMT	0.00	0.00	-0.05	0.02
4256	MGP	-0.09	0.00	0.00	-0.22
4258	MGST2	0.00	0.00	0.00	NaN
4259	MGST3	0.00	0.00	0.00	NaN
4261	MHC2TA	0.00	0.00	0.00	NaN
4276	MICA	0.00	0.00	0.00	NaN
4277	MICB	0.00	0.00	0.00	NaN
4283	MIG	0.00	0.00	0.00	NaN
4285	MIPEP	0.00	0.00	-0.05	0.31
4286	MITF	-0.09	0.00	0.00	0.52
4287	MJD	0.00	0.00	0.00	NaN
4288	MKI67	0.00	0.00	-0.05	0.24
4289	MKLN1	0.00	0.25	0.00	0.23
4291	MLF1	0.00	0.00	0.00	NaN
4292	MLH1	0.00	0.00	0.00	NaN
4293	MAP3K9	0.00	0.00	0.00	NaN
4294	MAP3K10	0.00	0.00	0.00	NaN
4295	MLN	0.00	0.00	0.00	NaN
4296	MAP3K11	-0.09	0.00	0.05	0.33
4297	MLL	0.00	0.00	0.00	NaN
4298	MLLT1	0.00	0.00	0.00	NaN
4299	MLLT2	0.00	0.00	0.00	NaN
4300	MLLT3	0.00	-0.25	-0.15	0.09
4301	MLLT4	0.00	0.00	0.00	NaN
4306	NR3C2	0.00	0.00	0.00	0.06
4308	TRPM1	0.00	0.00	0.00	NaN
4311	MME	0.00	0.00	0.00	NaN
4312	MMP1	0.00	0.13	0.00	0.00
4313	MMP2	0.09	0.00	0.00	-0.05
4314	MMP3	0.00	0.13	0.00	0.04
4316	MMP7	0.00	0.13	0.00	-0.04
4317	MMP8	0.00	0.13	0.00	0.08
4318	MMP9	0.00	0.00	0.00	NaN
4319	MMP10	0.00	0.13	0.00	0.43
4320	MMP11	0.00	0.00	0.05	0.06
4321	MMP12	0.00	0.13	0.00	0.02
4322	MMP13	0.00	0.13	0.00	0.33

4323	MMP14	0.00	0.00	0.00	NaN
4324	MMP15	-0.09	0.00	0.00	-0.03
4325	MMP16	0.18	0.38	0.00	0.23
4326	MMP17	0.00	0.00	0.00	NaN
4327	MMP19	0.00	0.00	0.00	NaN
4329	ALDH6A1	0.00	0.00	0.00	NaN
4331	MNAT1	0.00	0.00	0.00	NaN
4332	MNDA	0.00	0.00	0.00	NaN
4335	MNT	0.00	-0.13	0.00	-0.07
4336	MOBP	0.00	0.00	-0.05	0.09
4338	MOCS2	0.00	0.00	-0.05	0.38
4340	MOG	0.00	0.00	0.00	NaN
4342	MOS	0.00	0.00	0.00	NaN
4345	MOX2	0.00	0.00	0.00	NaN
4350	MPG	0.00	0.00	-0.05	0.20
4351	MPI	0.00	0.00	0.00	NaN
4352	MPL	0.00	0.00	0.00	NaN
4353	MPO	0.09	0.00	0.00	0.20
4356	MPP3	0.00	-0.13	0.00	-0.24
4357	MPST	0.00	0.00	0.00	NaN
4358	MPV17	0.09	0.00	0.05	0.26
4359	MPZ	0.00	0.00	0.05	-0.10
4361	MRE11A	-0.09	0.13	-0.05	0.37
4363	ABCC1	0.00	0.00	0.00	NaN
4430	MYO1B	0.00	0.00	0.00	NaN
4436	MSH2	0.00	0.00	0.00	NaN
4437	MSH3	0.00	0.00	0.00	NaN
4438	MSH4	0.00	0.00	0.00	NaN
4439	MSH5	0.00	0.00	0.00	NaN
4440	MSI1	0.00	-0.13	0.00	-0.07
4477	MSMB	0.00	0.00	0.00	NaN
4481	MSR1	0.00	-0.13	-0.05	0.03
4482	MSRA	-0.09	-0.38	-0.15	0.24
4485	MST1	-0.09	0.00	-0.05	0.15
4486	MST1R	-0.09	0.00	-0.05	0.19
4487	MSX1	0.00	-0.13	-0.15	0.00
4488	MSX2	0.00	0.00	0.00	NaN
4495	MT1G	-0.09	0.00	0.00	0.08
4496	MT1H	-0.09	0.00	0.00	0.03
4501	MT1X	-0.09	0.00	0.00	0.05
4502	MT2A	-0.09	0.00	0.00	0.01
4504	MT3	-0.09	0.00	0.00	0.10
4507	MTAP	0.00	-0.13	-0.15	0.51
4521	NUDT1	0.00	0.13	0.05	0.41
4522	MTHFD1	0.00	0.00	0.05	0.01
4524	MTHFR	0.00	0.00	0.00	NaN
4528	MTIF2	0.09	0.00	0.00	-0.13

4542	MYO1F	0.00	0.00	0.00	NaN
4543	MTNR1A	0.00	0.00	0.00	0.08
4544	MTNR1B	0.00	0.00	-0.05	0.22
4547	MTP	0.00	0.00	0.05	-0.02
4548	MTR	0.09	0.00	0.00	0.41
4552	MTRR	0.00	0.00	0.05	0.64
4580	MTX1	0.27	0.13	0.00	0.66
4582	MUC1	0.50	0.50	0.00	0.43
4583	MUC2	-0.27	-0.13	-0.05	-0.08
4585	MUC4	0.18	0.00	0.00	0.46
4586	MUC5AC	-0.27	-0.13	-0.05	-0.27
4588	MUC6	-0.27	-0.13	-0.05	-0.08
4593	MUSK	0.00	0.00	0.00	NaN
4594	MUT	0.00	0.00	0.00	NaN
4595	MUTYH	0.00	0.00	0.00	NaN
4597	MVD	-0.09	-0.13	-0.05	0.24
4598	MVK	0.00	0.00	0.00	NaN
4599	MX1	0.00	0.00	0.00	NaN
4600	MX2	0.00	0.00	0.00	NaN
4601	MXI1	0.00	0.00	-0.05	0.18
4602	MYB	0.00	0.00	0.05	0.31
4603	MYBL1	0.00	0.00	0.05	0.41
4605	MYBL2	0.09	-0.13	0.00	-0.05
4606	MYBPC2	0.00	0.00	0.00	NaN
4608	MYBPH	0.00	0.13	0.00	-0.21
4609	MYC	0.18	0.38	0.30	0.00
4610	MYCL1	0.00	0.00	0.00	NaN
4613	MYCN	0.09	0.00	-0.10	0.26
4615	MYD88	0.00	0.25	0.00	0.76
4616	GADD45B	0.00	0.00	0.00	NaN
4617	MYF5	0.00	0.13	0.00	-0.04
4618	MYF6	0.00	0.13	0.00	0.04
4619	MYH1	0.00	0.00	0.00	NaN
4620	MYH2	0.00	0.00	0.00	NaN
4621	MYH3	0.00	0.00	0.00	NaN
4622	MYH4	0.00	0.00	0.00	NaN
4624	MYH6	0.00	0.00	0.00	NaN
4625	MYH7	0.00	0.00	0.00	NaN
4626	MYH8	0.00	0.00	0.00	NaN
4627	MYH9	-0.09	0.00	0.00	0.04
4629	MYH11	0.00	0.00	0.00	NaN
4632	MYL1	0.00	0.00	0.00	NaN
4633	MYL2	0.00	0.00	0.00	NaN
4634	MYL3	-0.09	0.00	-0.05	-0.08
4635	MYL4	0.00	-0.13	0.00	0.03
4636	MYL5	0.00	-0.13	-0.10	0.32
4637	MYL6	0.00	0.00	0.00	NaN

4638	MYLK	0.00	0.00	0.00	NaN
4640	MYO1A	0.00	0.00	0.00	NaN
4641	MYO1C	0.00	0.00	0.50	0.20
4642	MYO1D	0.00	0.00	0.00	NaN
4643	MYO1E	0.09	0.00	0.05	0.07
4644	MYO5A	0.09	0.00	0.00	0.37
4646	MYO6	0.00	0.00	0.05	-0.15
4647	MYO7A	0.27	0.00	0.00	0.11
4649	MYO9A	0.00	0.00	0.00	NaN
4650	MYO9B	0.00	0.00	0.00	NaN
4651	MYO10	0.00	0.00	0.10	0.32
4653	MYOC	0.00	0.00	0.00	NaN
4654	MYOD1	0.00	0.00	0.00	NaN
4656	MYOG	0.00	0.13	0.00	-0.20
4659	PPP1R12A	0.00	0.13	0.00	0.15
4660	PPP1R12B	0.09	0.13	0.00	0.04
4661	MYT1	0.00	0.00	0.00	NaN
4664	NAB1	0.00	0.00	0.00	NaN
4665	NAB2	0.00	0.00	0.00	NaN
4666	NACA	0.00	0.00	0.00	NaN
4668	NAGA	0.00	0.00	0.05	0.22
4669	NAGLU	0.00	-0.13	0.00	0.07
4670	HNRPM	0.00	0.00	0.00	NaN
4671	BIRC1	0.00	0.00	0.00	NaN
4673	NAP1L1	0.00	0.00	0.00	NaN
4676	NAP1L4	0.00	-0.13	-0.05	0.48
4677	NARS	-0.09	0.00	-0.20	0.50
4678	NASP	0.00	0.00	0.00	NaN
4680	CEACAM6	0.00	0.00	-0.05	0.15
4681	NBL1	-0.09	0.00	0.00	0.18
4682	NUBP1	0.00	0.00	0.00	NaN
4683	NBS1	0.18	0.25	0.05	0.70
4684	NCAM1	0.00	0.00	0.00	NaN
4686	NCBP1	0.00	0.00	0.00	NaN
4688	NCF2	0.00	0.00	0.00	NaN
4689	NCF4	-0.09	0.00	0.00	-0.06
4690	NCK1	0.00	0.00	0.00	NaN
4692	NDN	0.00	-0.13	0.00	0.04
4695	NDUFA2	0.00	0.00	0.00	NaN
4696	NDUFA3	0.09	0.00	0.00	0.30
4697	NDUFA4	0.00	0.13	0.05	0.37
4698	NDUFA5	0.00	0.13	0.00	0.24
4700	NDUFA6	0.00	0.00	0.05	0.16
4701	NDUFA7	0.00	0.13	0.00	0.01
4702	NDUFA8	0.00	0.00	0.00	NaN
4703	NEB	0.00	0.00	0.00	NaN
4704	NDUFA9	-0.09	0.00	0.05	0.44

4705	NDUFA10	0.00	0.00	-0.05	0.36
4706	NDUFAB1	0.00	0.00	-0.05	0.43
4707	NDUFB1	0.00	0.00	0.00	NaN
4708	NDUFB2	0.00	0.00	0.00	NaN
4709	NDUFB3	0.00	0.00	0.00	NaN
4710	NDUFB4	0.00	0.00	0.00	NaN
4711	NDUFB5	0.09	0.00	0.00	0.45
4712	NDUFB6	-0.09	0.25	0.00	0.25
4713	NDUFB7	0.00	-0.13	0.00	0.05
4714	NDUFB8	0.00	-0.13	-0.05	0.36
4717	NDUFC1	0.00	0.00	0.00	NaN
4718	NDUFC2	0.27	0.00	0.00	0.77
4719	NDUFS1	0.00	0.00	0.00	NaN
4720	NDUFS2	0.00	0.00	0.00	NaN
4722	NDUFS3	0.00	0.00	0.00	NaN
4724	NDUFS4	0.00	0.00	-0.05	0.60
4725	NDUFS5	0.00	0.00	0.00	NaN
4726	NDUFS6	0.00	0.13	0.10	0.47
4728	NDUFS8	0.18	0.00	0.05	0.59
4729	NDUFV2	0.00	0.00	0.00	NaN
4733	DRG1	0.00	0.13	0.00	0.07
4734	NEDD4	0.00	0.00	0.05	-0.05
4735	NEDD5	0.00	0.00	-0.05	0.37
4736	RPL10A	0.00	0.00	0.00	NaN
4738	NEDD8	0.00	0.00	0.00	NaN
4741	NEF3	0.00	-0.13	-0.05	0.26
4744	NEFH	-0.09	0.13	0.00	0.04
4745	NELL1	0.00	0.00	0.50	-0.02
4747	NEFL	0.00	-0.13	-0.05	0.02
4750	NEK1	0.00	0.00	-0.05	0.31
4751	NEK2	0.00	0.00	0.00	NaN
4752	NEK3	0.00	0.00	0.00	NaN
4753	NELL2	0.00	0.50	0.00	-0.01
4756	NEO1	0.00	0.00	0.00	NaN
4758	NEU1	0.00	0.00	0.00	NaN
4759	NEU2	0.00	0.00	0.00	NaN
4760	NEUROD1	0.00	0.00	0.00	NaN
4761	NEUROD2	0.00	1.00	0.00	0.35
4762	NEUROG1	0.00	-0.13	0.00	0.08
4763	NF1	0.00	0.00	0.00	NaN
4771	NF2	-0.09	0.00	0.00	0.24
4772	NFATC1	-0.09	0.00	-0.25	0.13
4775	NFATC3	-0.09	-0.13	0.00	0.24
4776	NFATC4	0.00	0.00	0.00	NaN
4778	NFE2	0.00	0.00	0.00	NaN
4779	NFE2L1	0.00	0.50	0.50	0.38
4780	NFE2L2	0.00	0.00	0.00	NaN

4781	NFIB	0.00	0.00	0.05	0.41
4782	NFIC	0.00	0.00	0.00	NaN
4783	NFIL3	0.00	0.00	0.00	NaN
4784	NFIX	0.00	0.00	0.00	NaN
4790	NFKB1	0.00	0.00	0.00	NaN
4791	NFKB2	0.00	-0.13	-0.05	0.22
4792	NFKBIA	0.00	0.00	0.00	NaN
4793	NFKBIB	0.00	0.00	0.00	NaN
4794	NFKBIE	0.00	0.00	0.00	NaN
4795	NFKBIL1	0.00	0.00	0.00	NaN
4798	NFRKB	-0.09	0.00	-0.10	0.35
4799	NFX1	-0.09	0.00	0.05	0.39
4800	NFYA	0.00	0.00	0.00	NaN
4801	NFYB	0.00	0.00	0.00	NaN
4802	NFYC	0.00	0.00	0.00	NaN
4803	NGFB	0.09	0.00	0.10	0.28
4804	NGFR	0.00	0.25	0.00	0.01
4808	NHLH2	0.09	0.00	0.10	-0.05
4809	NHP2L1	0.00	0.00	0.05	0.36
4811	NID	0.09	0.00	0.00	-0.18
4814	NINJ1	0.00	0.00	0.00	NaN
4815	NINJ2	0.00	0.00	0.05	0.29
4817	NIT1	0.00	0.00	0.00	NaN
4818	NKG7	0.00	0.13	-0.05	0.26
4820	NKTR	0.00	0.00	-0.05	0.31
4821	NKX2B	0.00	0.00	0.00	NaN
4824	NKX3A	-0.09	-0.25	-0.15	0.21
4825	NKX6A	0.00	0.00	0.00	NaN
4826	NNAT	0.00	-0.13	0.00	-0.33
4828	NMB	0.09	0.00	-0.05	0.17
4829	NMBR	-0.09	-0.13	0.00	0.10
4830	NME1	0.00	0.25	0.00	0.44
4831	NME2	0.00	0.25	0.00	0.55
4832	NME3	0.00	0.00	0.00	NaN
4833	NME4	0.00	0.00	-0.05	-0.01
4835	NQO2	0.00	0.00	-0.10	0.39
4836	NMT1	0.00	-0.13	0.00	0.41
4837	NNMT	-0.09	0.00	0.00	-0.09
4839	NOL1	-0.09	0.00	0.00	0.32
4842	NOS1	0.00	-0.13	0.00	0.07
4843	NOS2A	0.00	0.13	0.00	0.23
4846	NOS3	0.00	0.00	-0.05	0.04
4848	CNOT2	0.09	0.00	0.00	0.41
4849	CNOT3	0.09	0.00	0.00	0.03
4850	CNOT4	0.00	0.00	0.00	NaN
4852	NPY	0.00	0.13	0.00	0.07
4853	NOTCH2	0.18	0.13	0.05	0.33

4854	NOTCH3	0.00	0.00	0.05	0.48
4855	NOTCH4	0.00	0.00	0.00	NaN
4856	NOV	0.00	0.25	0.15	-0.01
4857	NOVA1	0.00	0.00	0.00	NaN
4858	NOVA2	0.09	0.00	0.00	0.07
4860	NP	0.00	0.00	0.00	NaN
4861	NPAS1	0.09	0.00	0.00	-0.10
4862	NPAS2	0.00	0.00	0.00	NaN
4863	NPAT	0.00	0.00	0.00	NaN
4864	NPC1	0.00	0.00	-0.15	0.41
4867	NPHP1	0.00	0.00	0.00	NaN
4868	NPHS1	0.00	0.00	0.00	NaN
4869	NPM1	0.00	0.00	0.00	NaN
4879	NPPB	0.00	0.00	0.00	NaN
4880	NPPC	0.00	0.00	0.00	NaN
4881	NPR1	0.09	0.13	0.00	-0.02
4882	NPR2	-0.09	0.00	0.10	0.05
4883	NPR3	0.00	0.00	0.15	0.25
4884	NPTX1	0.00	0.00	0.00	NaN
4885	NPTX2	0.18	0.00	0.00	-0.09
4886	NPY1R	0.00	0.00	0.00	NaN
4887	NPY2R	0.00	0.00	0.00	0.09
4888	NPY6R	0.00	-0.13	0.00	0.02
4889	NPY5R	0.00	0.00	0.00	NaN
4891	SLC11A2	0.00	0.00	0.00	NaN
4893	NRAS	0.18	0.00	0.10	0.48
4897	NRCAM	0.09	0.00	0.00	0.16
4898	NRD1	0.00	0.00	0.00	NaN
4900	NRGN	0.00	0.00	-0.10	0.18
4901	NRL	0.00	0.00	0.00	NaN
4902	NRTN	0.00	0.00	0.00	NaN
4904	NSEP1	0.00	0.00	0.00	NaN
4905	NSF	-0.18	-0.38	-0.15	0.34
4907	NT5E	0.00	0.00	0.00	NaN
4908	NTF3	-0.09	0.00	0.00	-0.34
4913	NTHL1	0.00	0.13	0.05	0.19
4914	NTRK1	0.00	0.00	0.00	NaN
4915	NTRK2	0.00	0.00	0.00	NaN
4916	NTRK3	0.00	0.00	-0.05	0.13
4917	NTN2L	0.00	0.00	0.00	NaN
4919	ROR1	0.00	0.00	-0.05	0.06
4920	ROR2	0.00	0.00	0.00	NaN
4921	DDR2	0.00	0.00	0.00	-0.18
4922	NTS	0.00	0.13	0.00	0.52
4923	NTSR1	0.09	0.13	0.00	0.03
4924	NUCB1	0.09	0.00	0.00	0.21
4925	NUCB2	0.00	0.00	0.00	NaN

4926	NUMA1	0.00	-0.13	0.00	0.03
4927	NUP88	0.00	0.00	0.00	NaN
4928	NUP98	0.00	-0.13	0.00	0.42
4929	NR4A2	0.00	0.00	-0.05	0.09
4931	NVL	0.09	0.00	0.00	0.36
4938	OAS1	0.00	0.00	0.00	NaN
4939	OAS2	0.00	0.00	0.00	NaN
4940	OAS3	0.00	0.00	0.00	NaN
4942	OAT	0.00	0.00	-0.05	0.13
4947	OAZ2	0.00	0.00	0.00	NaN
4948	OCA2	0.00	-0.13	0.00	0.04
4950	OCLN	0.00	0.00	0.00	NaN
4951	OCM	0.50	0.00	0.50	-0.05
4952	OCRL	0.00	0.00	-0.05	0.02
4953	ODC1	0.00	0.13	-0.05	0.04
4956	ODF1	0.50	0.50	0.00	0.30
4958	OMD	0.00	0.00	0.00	NaN
4967	OGDH	0.00	0.00	0.00	NaN
4968	OGG1	0.00	0.00	0.00	NaN
4969	OGN	0.00	0.00	0.00	NaN
4973	OLR1	-0.09	0.00	0.00	-0.09
4974	OMG	0.00	0.00	0.00	NaN
4975	OMP	0.27	0.00	0.00	0.09
4976	OPA1	0.18	0.00	0.05	0.59
4978	OPCML	-0.09	0.00	-0.15	-0.29
4985	OPRD1	0.00	0.00	0.00	NaN
4986	OPRK1	0.00	0.00	0.00	NaN
4987	OPRL1	0.00	0.00	0.00	NaN
4988	OPRM1	0.00	0.00	0.00	NaN
4990	SIX6	0.00	0.00	0.00	NaN
4991	OR1D2	0.00	0.00	0.00	NaN
4992	OR1F1	0.00	0.00	0.00	NaN
4993	OR2C1	0.00	0.00	0.00	NaN
4994	OR3A1	0.00	0.00	0.00	NaN
4995	OR3A2	0.00	0.00	0.00	NaN
4998	ORC1L	0.00	0.00	0.00	NaN
4999	ORC2L	0.00	0.00	0.00	NaN
5000	ORC4L	0.09	0.00	0.00	-0.14
5001	ORC5L	0.09	0.00	0.00	0.40
5002	SLC22A1L	0.00	-0.13	-0.05	0.13
5003	SLC22A1LS	0.00	-0.13	-0.05	0.12
5004	ORM1	0.09	0.00	0.00	0.06
5005	ORM2	0.09	0.00	0.00	-0.07
5007	OSBP	0.00	0.00	0.00	NaN
5008	OSM	0.00	0.00	0.00	NaN
5010	CLDN11	0.09	0.00	0.00	0.14
5016	OVGP1	0.09	0.00	0.00	0.13

5017	OVOL1	-0.09	0.13	0.05	0.24
5018	OXA1L	0.00	0.00	0.00	NaN
5019	OXCT	0.00	0.00	0.10	0.24
5020	OXT	0.00	0.00	0.00	NaN
5021	OXTR	0.00	0.00	0.00	NaN
5023	P2RX1	0.00	0.00	0.00	NaN
5024	P2RX3	0.00	0.00	0.00	NaN
5025	P2RX4	0.00	-0.13	0.00	0.12
5026	P2RX5	0.00	0.00	0.00	NaN
5027	P2RX7	0.00	-0.13	0.00	0.16
5028	P2RY1	0.00	0.00	0.00	NaN
5029	P2RY2	0.00	0.13	0.00	0.31
5031	P2RY6	0.00	0.13	0.00	-0.04
5032	P2RY11	0.00	0.00	0.05	0.00
5033	P4HA1	0.09	0.00	0.00	0.14
5034	P4HB	0.00	0.00	0.00	NaN
5037	PBP	0.00	-0.13	0.00	0.12
5042	PABPC3	0.00	-0.13	-0.15	0.10
5045	PACE	0.00	0.00	0.00	NaN
5046	PACE4	0.00	0.00	0.00	NaN
5047	PAEP	0.00	0.00	0.00	NaN
5048	PAFAH1B1	0.00	0.00	0.00	NaN
5049	PAFAH1B2	-0.09	0.13	0.05	0.00
5050	PAFAH1B3	0.09	0.00	-0.05	0.27
5051	PAFAH2	0.00	0.00	0.00	NaN
5052	PRDX1	0.00	0.00	0.00	NaN
5053	PAH	0.00	0.00	0.00	NaN
5054	SERPINE1	0.18	0.00	0.00	-0.06
5055	SERPINB2	-0.09	0.00	-0.10	-0.29
5058	PAK1	0.27	0.00	0.00	0.89
5062	PAK2	0.18	0.00	0.00	0.65
5064	PALM	0.00	0.00	0.00	NaN
5066	PAM	0.00	0.00	0.00	NaN
5068	PAP	0.00	0.00	0.00	NaN
5069	PAPPA	0.00	0.00	0.05	0.27
5071	PARK2	0.00	0.00	0.00	NaN
5073	PARN	0.00	0.00	0.00	NaN
5074	PAWR	0.00	0.50	0.00	0.23
5076	PAX2	0.00	-0.13	-0.05	0.04
5077	PAX3	0.00	0.00	0.00	NaN
5078	PAX4	0.00	0.25	0.00	0.19
5079	PAX5	-0.09	0.00	0.10	-0.14
5080	PAX6	0.00	0.00	0.00	NaN
5081	PAX7	-0.09	0.00	0.00	0.02
5082	PDCL	0.00	0.00	0.00	NaN
5083	PAX9	0.00	0.00	0.00	NaN
5087	PBX1	0.00	0.00	0.00	NaN

5090	PBX3	0.00	0.00	0.00	NaN
5092	PCBD	0.09	0.00	0.00	0.29
5093	PCBP1	0.00	0.00	-0.05	0.50
5094	PCBP2	0.00	0.00	0.00	NaN
5095	PCCA	-0.09	-0.13	0.00	0.40
5096	PCCB	0.00	0.00	0.00	NaN
5097	PCDH1	0.00	0.00	0.00	NaN
5098	PCDHGC3	0.00	0.00	0.00	NaN
5099	PCDH7	0.00	0.00	0.00	NaN
5100	PCDH8	0.00	0.00	0.00	NaN
5101	PCDH9	0.00	0.13	-0.15	0.12
5104	SERPINA5	0.00	0.00	0.00	NaN
5105	PCK1	0.18	0.63	0.00	-0.10
5106	PCK2	0.00	0.00	0.00	NaN
5110	PCMT1	0.00	0.00	0.00	NaN
5111	PCNA	0.00	0.00	0.00	NaN
5116	PCNT2	0.00	0.00	0.00	NaN
5118	PCOLCE	0.18	0.00	0.00	-0.03
5119	PCOLN3	-0.09	-0.13	0.00	0.22
5121	PCP4	0.00	0.00	0.00	NaN
5122	PCSK1	0.00	0.00	0.00	NaN
5125	PCSK5	0.00	0.00	-0.05	0.04
5126	PCSK2	0.00	0.00	0.00	NaN
5128	PCTK2	0.00	0.13	0.00	0.46
5129	PCTK3	0.00	0.13	0.05	0.17
5130	PCYT1A	0.18	0.00	0.00	0.46
5132	PDC	0.00	0.00	0.00	NaN
5133	PDCD1	0.09	0.00	-0.05	0.10
5134	PDCD2	0.00	0.00	0.00	NaN
5136	PDE1A	0.00	0.00	0.00	NaN
5137	PDE1C	0.00	0.13	0.00	0.35
5138	PDE2A	0.00	0.00	0.00	NaN
5139	PDE3A	0.00	0.00	0.00	NaN
5140	PDE3B	0.00	0.00	0.00	NaN
5141	PDE4A	0.00	0.00	0.05	-0.02
5142	PDE4B	0.00	0.00	0.00	NaN
5143	PDE4C	0.00	0.00	0.00	NaN
5144	PDE4D	0.00	0.00	0.00	NaN
5145	PDE6A	0.00	0.00	0.00	NaN
5146	PDE6C	0.00	0.00	0.00	NaN
5147	PDE6D	0.00	0.00	0.00	NaN
5148	PDE6G	0.00	0.00	0.00	NaN
5149	PDE6H	-0.09	0.00	0.00	0.28
5151	PDE8A	0.00	0.00	-0.05	0.15
5152	PDE9A	0.00	0.00	0.00	NaN
5153	PDE1B	0.00	0.00	0.00	NaN
5154	PDGFA	0.00	0.13	0.05	0.47

5155	PDGFB	-0.09	0.00	0.05	-0.05
5156	PDGFRA	0.00	0.00	0.00	NaN
5157	PDGFRL	0.00	-0.13	-0.05	0.04
5158	PDE6B	0.00	-0.13	-0.10	0.11
5159	PDGFRB	0.00	0.00	0.00	NaN
5161	PDHA2	0.00	0.00	0.00	NaN
5162	PDHB	0.00	-0.13	-0.10	0.27
5163	PDK1	0.00	0.00	0.00	NaN
5164	PDK2	0.00	0.25	0.00	0.61
5166	PDK4	0.18	0.00	0.00	0.15
5167	ENPP1	0.00	0.00	0.00	NaN
5168	ENPP2	0.00	0.25	0.15	-0.04
5169	ENPP3	0.00	0.00	0.00	NaN
5170	PDPK1	0.00	0.00	0.00	NaN
5172	SLC26A4	0.09	0.00	0.00	-0.14
5173	PDYN	0.00	0.00	0.05	-0.17
5174	PDZK1	0.00	0.00	0.05	-0.05
5175	PECAM1	0.27	0.13	0.00	0.15
5176	SERPINF1	0.00	-0.13	0.00	0.03
5178	PEG3	0.09	0.13	0.05	0.40
5179	PENK	0.00	0.00	0.00	NaN
5184	PEPD	0.00	0.00	0.05	0.09
5187	PER1	0.00	0.00	0.00	NaN
5188	PET112L	0.00	0.00	0.00	0.62
5189	PEX1	0.18	0.00	0.00	0.41
5190	PEX6	0.00	0.00	0.00	NaN
5191	PEX7	0.00	0.00	0.00	NaN
5192	PEX10	0.00	0.00	0.00	NaN
5193	PEX12	0.00	0.00	0.00	0.52
5194	PEX13	0.00	0.00	0.00	NaN
5195	PEX14	0.00	0.00	0.00	NaN
5196	PF4	0.00	0.00	0.00	NaN
5197	PF4V1	0.00	0.00	0.00	NaN
5198	PFAS	0.00	0.00	0.00	NaN
5201	PFDN1	0.00	0.00	0.50	-0.13
5202	PFDN2	0.00	0.00	0.00	NaN
5203	PFDN4	0.18	0.38	0.00	0.64
5204	PFDN5	0.00	0.00	0.00	NaN
5205	ATP8B1	-0.09	0.00	-0.20	-0.10
5208	PFKFB2	0.00	0.13	0.00	0.04
5209	PFKFB3	0.00	0.00	0.00	NaN
5210	PFKFB4	-0.09	0.00	-0.05	0.14
5211	PFKL	0.00	0.00	0.00	NaN
5213	PFKM	0.00	0.00	0.00	NaN
5214	PFKP	0.00	0.00	0.00	NaN
5216	PFN1	0.00	0.00	0.00	NaN
5217	PFN2	0.00	0.00	0.05	0.31

5218	PFTK1	0.09	0.00	0.00	0.48
5223	PGAM1	0.00	0.00	-0.05	0.17
5224	PGAM2	0.00	0.00	0.00	NaN
5225	PGC	0.00	0.00	0.05	-0.07
5226	PGD	0.00	0.00	0.00	NaN
5228	PGF	0.00	0.00	0.00	NaN
5229	PGGT1B	0.00	0.00	0.00	NaN
5236	PGM1	0.00	0.00	-0.05	-0.02
5239	PGM5	0.00	0.00	-0.05	-0.11
5241	PGR	0.00	0.00	-0.05	0.04
5243	ABCB1	0.00	0.00	0.00	NaN
5244	ABCB4	0.00	0.00	0.00	NaN
5245	PHB	0.00	0.38	0.00	0.61
5250	SLC25A3	0.00	0.00	0.00	0.62
5252	PHF1	0.00	0.00	0.00	NaN
5257	PHKB	-0.09	0.00	0.05	0.49
5260	PHKG1	0.00	0.00	0.10	0.15
5261	PHKG2	0.00	0.00	0.00	NaN
5264	PHYH	0.00	0.00	0.00	NaN
5265	SERPINA1	0.00	0.00	0.00	NaN
5266	PI3	0.00	0.13	0.00	-0.07
5267	SERPINA4	0.00	0.00	0.00	NaN
5268	SERPINB5	-0.09	0.00	-0.15	0.01
5271	SERPINB8	0.00	0.00	0.00	0.21
5272	SERPINB9	0.00	0.00	-0.10	0.04
5273	SERPINB10	-0.09	0.00	-0.10	-0.17
5274	SERPINI1	0.00	0.00	0.00	NaN
5275	SERPINB13	-0.09	0.00	-0.15	0.34
5276	SERPINI2	0.00	0.00	0.00	NaN
5279	PIGC	0.00	0.00	0.00	NaN
5281	PIGF	0.00	0.00	0.00	NaN
5283	PIGH	0.00	0.00	0.00	NaN
5284	PIGR	0.00	0.13	0.00	-0.13
5287	PIK3C2B	0.00	0.13	0.00	0.34
5288	PIK3C2G	0.00	0.00	0.00	NaN
5289	PIK3C3	0.00	-0.13	-0.05	0.55
5290	PIK3CA	0.09	0.00	0.05	0.65
5291	PIK3CB	0.00	0.00	0.00	NaN
5292	PIM1	0.00	0.00	0.00	NaN
5293	PIK3CD	0.00	0.00	0.00	NaN
5294	PIK3CG	0.09	0.00	0.00	0.07
5295	PIK3R1	0.00	0.00	0.00	NaN
5296	PIK3R2	0.00	0.00	0.00	NaN
5297	PIK4CA	0.00	0.13	0.05	-0.10
5298	PIK4CB	0.00	0.00	0.05	0.37
5300	PIN1	0.00	0.00	1.00	0.51
5301	PIN1L	0.00	0.00	0.00	NaN

5304	PIP	0.00	0.00	0.00	0.01
5305	PIP5K2A	-0.09	0.00	0.05	0.10
5306	PITPN	0.00	-0.13	0.00	0.03
5307	PITX1	0.00	-0.13	0.00	0.12
5308	PITX2	0.00	0.00	0.00	NaN
5309	PITX3	0.00	0.00	0.00	-0.16
5310	PKD1	0.00	0.13	0.00	0.05
5311	PKD2	0.00	0.00	0.00	NaN
5313	PKLR	0.09	0.13	0.00	-0.05
5315	PKM2	0.00	0.00	0.00	NaN
5316	PKNOX1	0.00	0.13	0.00	0.36
5317	PKP1	0.00	0.00	0.00	NaN
5318	PKP2	0.00	0.00	0.00	NaN
5319	PLA2G1B	0.00	-0.13	0.00	0.05
5320	PLA2G2A	-0.09	0.00	0.00	-0.27
5321	PLA2G4A	0.00	0.00	0.00	NaN
5322	PLA2G5	0.00	0.00	0.00	NaN
5324	PLAG1	0.00	0.00	0.00	NaN
5325	PLAGL1	0.00	0.00	0.00	NaN
5326	PLAGL2	0.00	0.00	0.05	0.14
5327	PLAT	0.36	-0.13	-0.05	-0.11
5328	PLAU	0.09	0.00	0.00	-0.13
5329	PLAUR	0.09	0.00	-0.10	0.14
5330	PLCB2	0.00	0.00	0.00	NaN
5332	PLCB4	0.00	0.00	0.00	NaN
5333	PLCD1	0.00	0.25	0.00	0.16
5334	PLCL1	0.00	0.00	0.00	NaN
5335	PLCG1	0.18	-0.13	0.00	0.26
5336	PLCG2	-0.09	0.00	-0.05	0.10
5337	PLD1	0.09	0.00	0.00	0.25
5338	PLD2	0.00	0.00	0.00	NaN
5339	PLEC1	0.18	0.00	0.10	0.10
5340	PLG	0.00	0.00	0.00	NaN
5341	PLEK	0.00	0.00	0.00	NaN
5342	PLGL	0.00	0.50	0.00	0.23
5345	SERPINF2	0.00	-0.13	0.00	-0.03
5346	PLIN	0.00	0.00	0.00	NaN
5347	PLK	0.00	0.00	-0.05	0.16
5348	FXYD1	0.00	0.00	0.00	NaN
5349	FXYD3	0.00	0.00	0.00	NaN
5350	PLN	-0.09	0.00	0.05	0.14
5351	PLOD	0.00	0.00	0.00	NaN
5352	PLOD2	0.00	0.00	-0.05	0.27
5357	PLS1	0.00	0.00	-0.05	0.09
5359	PLSCR1	0.00	0.00	-0.05	0.30
5360	PLTP	0.00	0.00	0.00	NaN
5361	PLXNA1	0.00	0.00	0.00	NaN

5362	PLXNA2	0.00	0.13	0.00	-0.11
5364	PLXNB1	-0.09	0.00	-0.05	0.24
5366	PMAIP1	0.00	0.00	-0.20	0.24
5367	PMCH	0.00	0.00	0.00	NaN
5368	PNOC	-0.09	-0.50	-0.10	-0.29
5371	PML	0.00	0.00	0.00	NaN
5372	PMM1	0.00	0.00	0.05	0.28
5373	PMM2	0.00	0.00	0.00	NaN
5375	PMP2	0.00	0.00	0.05	0.01
5376	PMP22	0.00	0.00	0.00	NaN
5378	PMS1	0.00	0.00	0.00	NaN
5379	PMS2L1	0.18	0.00	0.00	0.35
5380	PMS2L2	-0.09	0.00	0.00	-0.16
5382	PMS2L4	0.00	0.00	0.05	0.26
5387	PMS2L9	-0.09	0.00	0.00	-0.21
5393	PMSCL1	0.00	0.00	0.00	NaN
5394	PMSCL2	0.00	0.00	0.00	NaN
5395	PMS2	0.00	0.00	1.00	0.48
5396	PMX1	0.00	0.00	0.00	NaN
5406	PNLIP	0.00	0.00	-0.05	0.06
5407	PNLIPRP1	0.00	0.00	-0.05	0.01
5408	PNLIPRP2	0.00	0.00	-0.05	0.01
5409	PNMT	0.00	1.00	0.00	0.42
5411	PNN	0.00	0.00	0.00	NaN
5412	UBL3	0.00	0.00	-0.05	0.20
5413	PNUTL1	0.00	0.00	0.00	0.24
5414	PNUTL2	0.09	0.00	0.00	-0.08
5420	PODXL	0.00	0.25	0.00	-0.20
5423	POLB	0.36	0.13	0.00	0.62
5424	POLD1	0.00	0.00	0.00	NaN
5425	POLD2	0.00	0.00	0.00	NaN
5426	POLE	0.00	0.00	0.00	NaN
5427	POLE2	0.00	0.00	0.00	NaN
5428	POLG	0.00	0.00	-0.05	0.38
5429	POLH	0.00	0.00	0.00	NaN
5430	POLR2A	0.00	0.00	0.00	NaN
5431	POLR2B	0.00	0.00	0.00	NaN
5432	POLR2C	-0.09	0.00	0.00	0.40
5433	POLR2D	0.00	0.00	0.00	0.41
5434	POLR2E	0.00	0.00	0.00	NaN
5435	POLR2F	0.00	0.00	0.05	0.38
5436	POLR2G	0.00	0.00	0.00	NaN
5437	POLR2H	0.09	0.00	0.00	0.49
5438	POLR2I	0.00	0.00	0.00	NaN
5439	POLR2J	0.18	0.00	0.00	0.54
5440	POLR2K	0.09	0.00	0.05	0.24
5441	POLR2L	-0.18	-0.13	-0.05	0.51

5442	POLRMT	0.00	0.00	-0.05	0.24
5443	POMC	0.09	0.00	0.00	-0.01
5444	PON1	0.18	0.00	0.00	0.22
5445	PON2	0.18	0.00	0.00	0.34
5446	PON3	0.18	0.00	0.00	-0.01
5447	POR	-0.09	0.00	0.00	0.39
5449	POU1F1	-0.09	0.00	0.05	0.11
5450	POU2AF1	0.00	0.00	0.00	NaN
5451	POU2F1	0.00	0.00	0.00	NaN
5452	POU2F2	0.00	0.00	-0.05	0.13
5453	POU3F1	0.00	0.00	0.00	NaN
5454	POU3F2	0.00	0.00	0.00	NaN
5455	POU3F3	0.00	0.00	0.00	NaN
5457	POU4F1	-0.09	0.13	-0.10	0.09
5458	POU4F2	0.00	0.00	-0.05	0.19
5459	POU4F3	0.00	0.00	0.00	NaN
5460	POU5F1	0.00	0.00	0.00	NaN
5463	POU6F1	0.00	0.00	0.00	NaN
5464	PP	0.00	0.00	0.00	NaN
5465	PPARA	0.00	0.00	0.00	NaN
5467	PPARD	0.00	0.00	0.00	NaN
5468	PPARG	0.09	0.00	0.00	-0.13
5469	PPARBP	0.00	0.75	0.00	0.82
5470	PPEF2	0.00	0.00	0.00	NaN
5471	PPAT	0.00	0.00	0.00	NaN
5473	PPBP	0.00	0.00	0.00	NaN
5476	PPGB	0.00	0.00	0.00	NaN
5478	PPIA	0.00	0.00	0.00	NaN
5479	PPIB	0.00	0.00	0.00	NaN
5480	PPIC	0.00	0.00	0.00	NaN
5481	PPID	0.00	0.00	0.00	0.43
5493	PPL	0.00	0.00	-0.05	0.29
5494	PPM1A	0.00	0.00	0.00	NaN
5495	PPM1B	0.00	0.00	0.00	NaN
5496	PPM1G	0.09	0.00	0.05	0.42
5499	PPP1CA	0.36	0.00	0.05	0.68
5500	PPP1CB	0.00	0.00	0.00	NaN
5501	PPP1CC	0.00	0.00	0.00	NaN
5502	PPP1R1A	0.00	0.00	0.00	NaN
5504	PPP1R2	0.18	0.00	0.00	0.55
5506	PPP1R3A	0.09	0.00	0.00	-0.02
5507	PPP1R3C	0.00	0.00	0.00	NaN
5509	PPP1R3D	0.18	0.25	0.00	0.35
5510	PPP1R7	0.00	0.00	-0.05	0.44
5511	PPP1R8	0.00	0.00	0.00	NaN
5514	PPP1R10	0.00	0.00	0.00	NaN
5515	PPP2CA	0.00	-0.13	0.00	0.44

5516	PPP2CB	-0.09	-0.25	-0.05	0.55
5518	PPP2R1A	0.00	0.00	-0.05	0.33
5519	PPP2R1B	0.00	0.00	0.00	NaN
5520	PPP2R2A	-0.09	-0.25	-0.05	0.51
5521	PPP2R2B	0.00	0.00	0.00	NaN
5523	PPP2R3A	0.00	0.00	0.00	NaN
5524	PPP2R4	0.00	0.00	0.00	NaN
5525	PPP2R5A	0.00	0.00	0.00	NaN
5526	PPP2R5B	0.00	0.00	0.00	NaN
5527	PPP2R5C	0.00	0.00	0.00	NaN
5528	PPP2R5D	0.00	0.00	0.00	NaN
5529	PPP2R5E	0.00	0.00	-0.05	0.70
5530	PPP3CA	0.00	0.00	0.00	NaN
5531	PPP4C	0.00	0.00	0.00	NaN
5532	PPP3CB	0.09	0.00	0.00	0.56
5533	PPP3CC	-0.09	-0.25	-0.10	0.37
5534	PPP3R1	0.00	0.00	0.00	NaN
5536	PPP5C	0.09	0.00	0.00	0.17
5537	PPP6C	0.00	0.00	0.00	NaN
5538	PPT1	0.00	0.00	0.00	NaN
5539	PPY	0.00	-0.13	0.00	-0.25
5540	PPYR1	0.00	0.00	-0.05	0.05
5542	PRB1	0.00	0.00	0.10	0.12
5544	PRB3	-0.09	0.00	0.05	-0.22
5546	PRCC	0.00	0.13	0.00	-0.12
5547	PRCP	0.00	0.00	0.05	0.23
5549	PRELP	0.00	0.25	0.00	0.05
5550	PREP	0.00	0.00	0.10	0.64
5551	PRF1	0.00	0.00	0.00	NaN
5552	PRG1	0.50	0.00	0.00	0.03
5553	PRG2	0.00	0.00	0.00	NaN
5554	PRH1	-0.09	0.00	0.00	0.33
5557	PRIM1	0.00	0.00	0.00	NaN
5558	PRIM2A	0.00	0.13	0.00	-0.08
5563	PRKAA2	0.00	0.00	-0.05	-0.26
5564	PRKAB1	0.00	-0.13	0.00	0.17
5565	PRKAB2	0.00	0.00	0.05	-0.05
5566	PRKACA	0.00	0.00	0.00	NaN
5567	PRKACB	0.00	0.00	0.05	0.34
5568	PRKACG	0.00	0.00	-0.05	-0.11
5569	PKIA	0.00	0.25	0.05	0.17
5571	PRKAG1	0.00	0.00	0.00	NaN
5573	PRKAR1A	0.18	0.13	0.00	0.43
5575	PRKAR1B	0.00	0.13	0.05	0.14
5576	PRKAR2A	-0.09	0.00	-0.05	0.30
5577	PRKAR2B	0.09	0.00	0.00	0.02
5578	PRKCA	0.18	0.13	0.00	0.08

5579	PRKCB1	0.00	0.00	0.00	NaN
5580	PRKCD	0.00	0.00	-0.05	0.34
5581	PRKCE	0.00	0.00	0.00	NaN
5582	PRKCG	0.09	0.00	0.00	-0.07
5583	PRKCH	0.00	0.00	0.00	NaN
5584	PRKCI	0.09	0.00	0.00	0.23
5585	PRKCL1	0.00	0.00	0.00	NaN
5586	PRKCL2	0.00	0.00	0.00	NaN
5587	PRKCM	0.00	0.00	0.00	NaN
5588	PRKCQ	0.00	0.00	0.00	NaN
5589	PRKCSH	0.00	0.00	0.00	NaN
5590	PRKCZ	0.00	0.00	0.00	NaN
5591	PRKDC	0.00	0.00	0.00	NaN
5592	PRKG1	0.00	0.00	0.00	NaN
5593	PRKG2	0.00	0.00	0.00	NaN
5594	MAPK1	0.00	0.00	0.00	NaN
5595	MAPK3	0.00	0.00	0.00	NaN
5596	MAPK4	-0.09	-0.13	-0.25	0.07
5597	MAPK6	0.18	0.00	0.00	0.47
5598	MAPK7	0.00	0.00	0.00	NaN
5599	MAPK8	0.00	-0.13	0.00	0.18
5600	MAPK11	0.00	-0.25	0.00	0.09
5601	MAPK9	0.00	0.13	0.00	0.41
5602	MAPK10	0.00	0.00	0.00	NaN
5603	MAPK13	0.00	0.00	0.00	NaN
5604	MAP2K1	0.00	0.00	0.00	NaN
5605	MAP2K2	0.00	0.00	0.00	NaN
5606	MAP2K3	0.00	-0.13	0.00	0.20
5607	MAP2K5	0.00	0.00	0.00	NaN
5608	MAP2K6	0.00	0.13	0.00	0.56
5609	MAP2K7	0.00	0.00	0.00	NaN
5610	PRKR	0.00	0.00	0.50	0.37
5611	DNAJC3	-0.09	0.00	-0.05	-0.05
5612	PRKRIR	0.27	0.00	0.00	0.62
5617	PRL	0.00	0.00	0.00	NaN
5618	PRLR	0.00	0.00	0.15	-0.10
5619	PRM1	0.00	0.00	0.00	NaN
5620	PRM2	0.00	0.00	0.00	NaN
5621	PRNP	0.00	0.00	0.00	NaN
5623	PSPN	0.00	0.00	0.00	NaN
5624	PROC	0.00	0.00	-0.05	-0.06
5625	PRODH	0.50	0.00	0.50	-0.07
5626	PROP1	0.00	0.00	0.00	NaN
5627	PROS1	-0.09	0.00	0.00	0.12
5629	PROX1	0.00	0.00	0.00	NaN
5630	PRPH	0.00	0.00	0.00	NaN
5635	PRPSAP1	0.00	0.13	0.00	0.38

5636	PRPSAP2	0.00	0.00	0.00	NaN
5639	PRRG2	0.00	0.00	0.00	NaN
5641	LGMN	0.00	0.00	0.00	NaN
5644	PRSS1	0.00	0.00	-0.05	0.10
5645	PRSS2	0.00	0.00	-0.05	-0.45
5646	PRSS3	-0.09	0.00	0.10	0.13
5648	MASP1	0.00	0.00	0.10	0.08
5649	RELN	0.09	0.00	0.00	0.07
5650	KLK7	0.00	0.00	0.00	NaN
5651	PRSS7	0.00	-0.13	0.00	0.02
5652	PRSS8	0.00	0.00	0.00	NaN
5653	KLK6	0.00	0.00	0.00	NaN
5654	PRSS11	0.00	0.00	-0.05	0.10
5655	KLK10	0.00	0.00	0.00	NaN
5657	PRTN3	0.00	0.00	0.05	-0.02
5660	PSAP	0.09	0.00	0.00	0.08
5662	PSD	0.00	-0.13	-0.05	-0.23
5663	PSEN1	0.00	0.00	0.00	NaN
5664	PSEN2	0.09	0.00	0.00	0.22
5669	PSG1	0.50	0.00	0.00	0.07
5670	PSG2	0.09	-0.13	-0.05	0.03
5671	PSG3	0.09	0.00	-0.05	0.20
5672	PSG4	1.00	0.00	0.50	0.19
5673	PSG5	0.00	-0.13	-0.05	0.01
5675	PSG6	1.00	0.00	0.00	0.27
5676	PSG7	0.00	0.00	-0.05	0.14
5678	PSG9	0.00	-0.13	-0.05	0.06
5682	PSMA1	0.00	0.00	0.00	NaN
5683	PSMA2	0.00	0.00	0.00	NaN
5684	PSMA3	0.00	0.00	0.05	0.19
5685	PSMA4	0.00	0.00	0.00	NaN
5686	PSMA5	0.09	0.00	0.05	0.53
5687	PSMA6	0.00	0.00	0.00	NaN
5688	PSMA7	0.09	0.13	0.00	0.27
5689	PSMB1	0.00	0.00	0.00	NaN
5690	PSMB2	0.00	0.00	0.00	NaN
5691	PSMB3	0.00	0.38	0.00	0.76
5692	PSMB4	0.00	0.00	0.05	0.27
5693	PSMB5	0.00	0.00	0.00	NaN
5694	PSMB6	0.00	0.00	0.00	NaN
5695	PSMB7	0.00	0.00	0.00	NaN
5696	PSMB8	0.09	0.00	0.00	-0.04
5697	PYY	0.00	-0.13	0.00	0.15
5698	PSMB9	0.09	0.00	0.00	-0.07
5699	PSMB10	-0.09	-0.13	0.00	0.29
5700	PSMC1	0.00	0.13	0.00	0.17
5702	PSMC3	0.00	-0.13	0.00	0.24

5704	PSMC4	0.00	0.00	0.00	NaN
5705	PSMC5	0.27	0.13	0.00	0.66
5706	PSMC6	0.00	0.00	0.05	0.05
5707	PSMD1	0.00	0.00	0.00	NaN
5708	PSMD2	0.09	0.00	0.00	0.26
5709	PSMD3	0.00	0.63	0.00	0.79
5710	PSMD4	0.00	0.00	0.05	0.22
5711	PSMD5	0.00	0.00	0.00	NaN
5713	PSMD7	0.00	0.00	0.05	0.21
5714	PSMD8	0.00	0.00	0.00	NaN
5715	PSMD9	0.00	-0.13	0.00	0.33
5717	PSMD11	0.00	0.00	0.00	NaN
5718	PSMD12	0.18	0.13	0.00	0.51
5719	PSMD13	-0.27	-0.13	-0.10	0.48
5720	PSME1	0.00	0.00	0.00	NaN
5721	PSME2	0.00	0.00	0.00	NaN
5723	PSPH	0.00	0.00	0.10	0.83
5724	PTAFR	0.00	0.00	0.00	NaN
5725	PTBP1	0.00	0.00	0.00	NaN
5727	PTCH	0.00	0.00	0.00	NaN
5728	PTEN	0.50	0.00	0.50	0.42
5729	PTGDR	0.00	0.13	0.05	0.35
5730	PTGDS	0.00	0.00	0.00	NaN
5731	PTGER1	0.00	0.00	0.00	NaN
5732	PTGER2	0.00	0.13	0.05	-0.05
5733	PTGER3	0.00	0.00	0.00	NaN
5734	PTGER4	0.00	0.00	0.10	-0.10
5737	PTGFR	0.00	0.00	0.00	NaN
5739	PTGIR	0.09	0.00	0.00	0.18
5740	PTGIS	0.09	0.25	0.05	0.10
5741	PTH	0.00	0.00	0.00	NaN
5742	PTGS1	0.00	0.00	0.00	NaN
5743	PTGS2	0.00	0.00	0.00	NaN
5744	PTHLH	0.00	0.00	0.00	NaN
5745	PTHR1	-0.09	0.00	-0.05	-0.08
5746	PTHR2	0.00	0.00	0.00	NaN
5747	PTK2	0.18	-0.13	0.05	0.57
5753	PTK6	0.09	0.00	0.00	0.19
5754	PTK7	0.00	0.00	0.00	NaN
5756	PTK9	0.00	0.13	0.00	0.46
5757	PTMA	0.00	0.00	0.00	NaN
5763	PTMS	-0.09	0.00	0.00	0.26
5764	PTN	0.00	0.00	-0.05	0.13
5768	QSCN6	0.00	0.00	0.00	NaN
5770	PTPN1	0.27	0.25	0.05	0.41
5771	PTPN2	0.00	0.00	0.00	NaN
5774	PTPN3	0.00	0.00	0.00	NaN

5775	PTPN4	0.00	0.00	0.00	NaN
5777	PTPN6	0.00	0.00	0.00	0.38
5778	PTPN7	0.00	0.13	0.00	-0.26
5780	PTPN9	0.00	0.00	0.00	NaN
5781	PTPN11	0.00	0.00	0.00	NaN
5782	PTPN12	-0.09	0.00	0.00	0.32
5783	PTPN13	0.00	0.00	0.00	NaN
5784	PTPN14	0.00	0.00	0.00	NaN
5786	PTPRA	0.00	0.00	0.05	0.26
5787	PTPRB	0.09	0.00	0.00	-0.06
5788	PTPRC	0.09	0.00	0.00	0.02
5789	PTPRD	0.00	-0.13	0.00	-0.06
5790	PTPRCAP	0.36	0.00	0.05	0.25
5791	PTPRE	0.00	0.00	-0.05	0.03
5792	PTPRF	0.00	0.00	0.00	NaN
5793	PTPRG	-0.09	0.00	-0.10	0.17
5794	PTPRH	0.09	0.00	0.00	0.05
5795	PTPRJ	0.00	0.00	0.05	0.09
5796	PTPRK	0.00	0.00	0.05	0.36
5797	PTPRM	0.00	0.00	0.00	NaN
5798	PTPRN	0.00	0.00	0.00	NaN
5799	PTPRN2	0.00	0.00	-0.05	0.23
5801	PTPRR	0.50	0.50	0.00	-0.07
5802	PTPRS	0.00	0.00	0.00	NaN
5803	PTPRZ1	0.00	0.13	0.00	-0.05
5805	PTS	0.00	0.00	0.00	NaN
5813	PURA	0.00	0.00	0.00	NaN
5817	PVR	0.09	0.00	0.00	0.29
5818	PVRL1	-0.09	0.00	-0.10	0.29
5819	PVRL2	0.09	0.00	0.00	-0.22
5822	PWP2H	0.00	0.00	0.00	NaN
5824	PXF	0.00	0.00	0.00	NaN
5825	ABCD3	0.09	0.00	0.05	0.56
5826	ABCD4	0.00	0.00	0.00	NaN
5827	PXMP2	0.00	0.00	0.00	NaN
5828	PXMP3	0.00	0.50	0.00	0.42
5829	PXN	0.00	-0.13	0.00	0.29
5830	PXR1	0.00	0.00	0.00	0.35
5831	PYCR1	0.00	0.00	0.05	-0.06
5832	PYCS	0.00	0.00	-0.05	0.37
5833	PCYT2	0.00	0.00	0.00	NaN
5834	PYGB	0.00	0.00	-0.05	0.26
5836	PYGL	0.00	0.00	0.05	0.19
5837	PYGM	0.00	0.00	0.00	NaN
5858	PZP	-0.09	0.00	0.00	-0.17
5860	QDPR	0.00	0.00	-0.05	0.17
5861	RAB1A	0.00	0.00	0.00	NaN

5862	RAB2	0.00	0.00	0.00	NaN
5863	RAB2L	0.09	0.00	0.00	0.14
5864	RAB3A	0.00	0.00	0.00	NaN
5865	RAB3B	0.00	0.00	0.00	NaN
5866	RAB3IL1	-0.09	0.00	0.00	0.03
5867	RAB4A	0.09	0.00	0.00	0.33
5868	RAB5A	0.00	0.00	0.00	NaN
5869	RAB5B	0.00	0.00	0.00	NaN
5870	RAB6A	0.50	0.50	0.00	0.63
5871	MAP4K2	0.00	0.00	0.00	NaN
5872	RAB13	0.09	0.13	0.00	0.32
5873	RAB27A	0.00	0.00	0.05	0.61
5874	RAB27B	-0.09	0.00	-0.20	0.28
5875	RABGGTA	0.00	0.00	0.00	NaN
5876	RABGGTB	0.00	0.00	0.00	NaN
5877	RABIF	0.09	0.13	0.00	0.17
5878	RAB5C	0.00	0.13	0.00	0.51
5879	RAC1	0.00	0.00	0.10	0.57
5880	RAC2	0.00	0.00	0.00	NaN
5881	RAC3	0.00	0.00	0.05	-0.01
5883	RAD9	0.45	0.00	0.05	0.45
5884	RAD17	0.00	0.00	0.00	NaN
5885	RAD21	0.09	0.38	0.10	0.50
5886	RAD23A	0.00	0.00	0.00	NaN
5887	RAD23B	0.00	0.00	0.00	NaN
5888	RAD51	0.00	0.00	0.00	NaN
5889	RAD51C	0.18	0.13	0.00	0.76
5890	RAD51L1	0.00	0.00	-0.05	0.31
5892	RAD51L3	0.00	-0.13	0.00	0.33
5893	RAD52	0.00	0.00	0.05	0.10
5894	RAF1	0.00	0.00	0.00	NaN
5896	RAG1	0.00	0.13	0.05	0.39
5897	RAG2	0.00	0.13	0.05	-0.12
5898	RALA	0.00	0.00	0.00	NaN
5899	RALB	0.00	0.00	0.00	NaN
5900	RALGDS	0.00	0.00	0.00	NaN
5901	RAN	0.00	0.00	0.00	NaN
5902	RANBP1	-0.09	0.00	0.00	0.30
5903	RANBP2	0.00	0.00	0.00	NaN
5905	RANGAP1	0.00	0.00	0.05	0.23
5906	RAP1A	0.09	0.00	0.00	0.38
5909	RAP1GA1	-0.09	0.00	0.00	-0.28
5910	RAP1GDS1	0.00	0.00	0.00	NaN
5911	RAP2A	-0.09	0.00	-0.05	0.08
5912	RAP2B	0.00	0.00	0.00	NaN
5913	RAPSN	0.00	0.00	0.00	NaN
5914	RARA	0.00	0.38	0.00	0.65

5915	RARB	0.00	0.00	0.00	NaN
5916	RARG	0.00	0.00	0.00	NaN
5917	RARS	0.00	0.00	0.00	NaN
5918	RARRES1	0.00	0.00	0.00	NaN
5919	RARRES2	0.00	0.00	-0.05	0.19
5920	RARRES3	0.00	0.00	0.00	NaN
5921	RASA1	0.00	-0.13	0.00	0.26
5922	RASA2	0.00	0.00	0.00	NaN
5923	RASGRF1	0.00	0.00	0.00	NaN
5925	RB1	0.00	0.00	0.00	NaN
5926	RBBP1	0.00	0.00	0.50	0.31
5927	RBBP2	0.00	0.00	0.50	0.32
5928	RBBP4	0.00	0.00	0.00	NaN
5929	RBBP5	0.00	0.13	0.00	0.45
5930	RBBP6	0.00	0.00	0.00	NaN
5932	RBBP8	0.00	0.00	-0.10	0.32
5934	RBL2	0.18	0.00	0.00	0.56
5936	RBM4	0.00	0.00	0.05	-0.06
5937	RBMS1	0.00	0.00	-0.10	0.50
5939	RBMS2	0.00	0.00	0.00	NaN
5955	RCN2	0.00	0.00	0.00	NaN
5957	RCV1	0.00	0.00	0.00	NaN
5959	RDH5	0.00	0.00	0.00	NaN
5961	RDS	0.00	0.00	0.00	NaN
5962	RDX	-0.09	0.00	0.00	0.17
5965	RECQL	0.00	0.00	0.00	NaN
5966	REL	0.00	0.00	0.00	NaN
5967	REG1A	0.00	0.00	0.00	NaN
5968	REG1B	0.00	0.00	0.00	NaN
5969	REGL	0.00	0.00	0.00	NaN
5970	RELA	-0.09	0.00	0.05	0.34
5971	RELB	0.09	0.00	0.00	-0.15
5972	REN	0.00	0.13	0.00	-0.17
5976	RENT1	0.00	0.00	0.00	NaN
5977	REQ	-0.09	0.00	0.00	0.43
5978	REST	0.00	0.00	0.00	NaN
5979	RET	0.00	0.00	0.00	NaN
5980	REV3L	0.00	0.00	0.05	0.45
5981	RFC1	0.00	0.00	0.05	0.43
5982	RFC2	-0.09	0.00	0.00	0.41
5983	RFC3	0.00	0.00	0.05	-0.07
5984	RFC4	0.09	0.00	0.05	0.40
5985	RFC5	0.00	-0.13	0.00	0.38
5986	RFNG	0.00	0.00	0.00	NaN
5987	RFP	0.00	0.00	0.00	NaN
5988	RFPL1	0.00	0.00	0.00	NaN
5989	RFX1	0.00	0.00	0.00	NaN

5990	RFX2	0.00	0.00	0.00	NaN
5991	RFX3	0.00	-0.13	-0.10	0.28
5992	RFX4	0.00	0.00	0.00	NaN
5993	RFX5	0.00	0.00	0.05	0.34
5994	RFXAP	0.00	0.00	0.00	NaN
5995	RGR	0.09	0.00	-0.05	-0.05
5996	RGS1	0.00	0.00	0.00	NaN
5997	RGS2	0.00	0.00	0.00	NaN
5998	RGS3	0.00	0.00	0.00	NaN
5999	RGS4	0.00	0.00	0.00	NaN
6000	RGS7	0.09	0.00	0.00	0.01
6001	RGS10	0.00	0.00	-0.05	0.23
6002	RGS12	0.00	-0.13	-0.15	0.19
6003	RGS13	0.00	0.00	0.00	NaN
6004	RGS16	0.00	0.00	0.00	NaN
6005	RHAG	0.00	0.00	0.00	NaN
6006	RHCE	-0.09	0.00	0.00	0.17
6007	RHD	-0.36	-0.25	-0.30	0.23
6010	RHO	0.00	0.00	0.00	NaN
6011	RHOK	-0.09	-0.13	-0.05	-0.02
6013	RLN1	-0.09	-0.13	-0.05	0.28
6014	RIN	0.00	-0.25	0.00	0.05
6015	RING1	0.09	0.00	0.00	0.12
6016	RIT	0.09	0.13	0.00	0.25
6017	RLBP1	0.00	0.00	-0.05	0.09
6019	RLN2	-0.09	-0.13	-0.05	0.21
6036	RNASE2	0.00	0.00	0.00	NaN
6037	RNASE3	0.00	0.00	0.00	NaN
6039	RNASE6	0.00	0.00	0.00	NaN
6041	RNASEL	0.00	0.00	0.00	NaN
6045	RNF2	0.00	0.00	0.50	0.44
6046	BRD2	0.09	0.00	0.00	0.06
6047	RNF4	0.00	0.00	0.00	0.54
6048	RNF5	0.00	0.00	0.00	NaN
6049	RNF6	0.00	-0.13	-0.05	0.39
6050	RNH	-0.27	-0.13	-0.10	0.60
6051	RNPEP	0.00	0.00	0.00	NaN
6059	ABCE1	0.00	0.00	0.00	NaN
6091	ROBO1	-0.09	0.00	-0.05	0.26
6093	ROCK1	0.00	0.00	0.00	NaN
6094	ROM1	0.00	0.00	0.00	NaN
6095	RORA	0.00	0.00	0.00	0.42
6096	RORB	0.00	0.00	-0.05	0.09
6097	RORC	0.00	0.13	0.05	0.03
6098	ROS1	-0.09	0.00	0.10	-0.28
6117	RPA1	0.00	-0.13	0.00	0.03
6118	RPA2	0.00	0.00	0.00	NaN

6119	RPA3	0.00	0.00	0.05	0.24
6120	RPE	0.00	0.00	0.00	NaN
6121	RPE65	0.00	0.00	0.00	NaN
6122	RPL3	-0.09	0.00	0.05	0.16
6123	RPL3L	0.00	0.00	0.00	NaN
6124	RPL4	0.00	0.00	0.00	NaN
6125	RPL5	0.00	0.00	0.05	0.40
6128	RPL6	0.00	0.00	0.00	NaN
6129	RPL7	0.00	0.25	0.00	0.46
6130	RPL7A	0.00	0.00	0.00	NaN
6132	RPL8	0.18	0.00	0.10	0.37
6133	RPL9	0.00	0.00	0.00	NaN
6135	RPL11	-0.09	0.00	0.00	0.11
6136	RPL12	-0.09	0.00	-0.05	0.31
6137	RPL13	-0.09	-0.13	-0.05	0.40
6138	RPL15	0.00	0.00	0.00	NaN
6141	RPL18	0.09	0.00	0.00	0.42
6142	RPL18A	0.00	0.00	0.00	NaN
6143	RPL19	0.00	0.75	0.00	0.80
6144	RPL21	0.00	0.00	-0.05	0.21
6146	RPL22	0.00	0.00	0.00	NaN
6147	RPL23A	0.00	0.25	0.00	0.26
6150	MRPL23	0.00	0.50	0.00	0.31
6152	RPL24	0.09	0.00	0.00	0.11
6155	RPL27	0.00	-0.13	0.00	-0.01
6156	RPL30	0.00	0.00	0.00	NaN
6157	RPL27A	0.00	0.00	-0.05	0.17
6158	RPL28	0.09	0.00	0.00	0.34
6159	RPL29	-0.09	0.00	-0.10	0.52
6160	RPL31	0.00	0.00	0.00	NaN
6161	RPL32	0.00	0.00	0.00	NaN
6164	RPL34	0.00	0.00	0.00	NaN
6165	RPL35A	0.18	0.00	0.00	0.64
6166	RPL36AL	0.00	0.00	0.00	NaN
6167	RPL37	0.00	0.00	0.10	0.17
6168	RPL37A	0.00	0.00	0.00	NaN
6169	RPL38	0.00	0.13	0.00	0.26
6171	RPL41	0.00	0.00	0.00	NaN
6175	RPLP0	0.00	-0.13	0.00	0.12
6176	RPLP1	0.00	0.00	0.00	NaN
6181	RPLP2	-0.18	-0.13	-0.05	0.48
6182	MRPL12	0.00	0.00	0.00	NaN
6183	MRPS12	0.00	0.00	0.00	NaN
6184	RPN1	0.00	0.00	0.00	NaN
6185	RPN2	0.00	-0.13	0.00	0.26
6187	RPS2	0.00	0.00	0.00	NaN
6188	RPS3	0.18	0.00	0.00	0.49

6189	RPS3A	0.00	0.00	0.00	0.49
6193	RPS5	0.00	0.13	0.05	0.56
6194	RPS6	0.00	-0.25	-0.10	0.68
6195	RPS6KA1	-0.09	0.00	0.00	-0.03
6196	RPS6KA2	0.00	0.00	0.00	NaN
6198	RPS6KB1	0.18	0.13	0.00	0.80
6199	RPS6KB2	0.36	0.00	0.05	0.58
6201	RPS7	0.00	0.00	0.00	NaN
6202	RPS8	0.09	0.00	0.00	0.32
6203	RPS9	0.09	0.00	0.00	0.16
6204	RPS10	0.00	0.00	0.00	NaN
6205	RPS11	0.00	0.00	0.00	NaN
6207	RPS13	0.00	0.00	0.00	NaN
6208	RPS14	0.00	0.00	0.00	NaN
6209	RPS15	0.00	0.00	0.00	NaN
6210	RPS15A	0.00	0.00	0.00	NaN
6217	RPS16	0.00	0.00	0.00	NaN
6218	RPS17	0.00	0.00	0.00	NaN
6222	RPS18	0.09	0.00	0.00	0.19
6223	RPS19	0.00	0.00	-0.05	0.32
6224	RPS20	0.00	0.00	0.00	NaN
6227	RPS21	0.09	0.13	0.00	0.23
6228	RPS23	0.00	-0.13	0.00	0.35
6229	RPS24	0.09	0.00	0.05	0.18
6230	RPS25	0.00	0.00	0.00	NaN
6231	RPS26	0.00	0.00	0.00	NaN
6232	RPS27	0.09	0.13	0.00	0.19
6233	RPS27A	0.09	0.00	0.00	-0.13
6234	RPS28	0.00	0.00	0.00	NaN
6235	RPS29	0.00	0.00	0.00	NaN
6236	RRAD	-0.09	-0.13	0.00	-0.05
6237	RRAS	0.00	0.00	0.00	NaN
6238	RRBP1	0.00	0.00	0.00	NaN
6239	RREB1	0.00	-0.13	0.00	0.00
6240	RRM1	0.00	-0.13	0.00	0.56
6241	RRM2	0.00	0.00	-0.05	0.41
6248	RSC1A1	0.00	0.00	0.00	NaN
6249	RSN	0.00	-0.13	0.00	0.24
6251	RSU1	0.00	0.00	0.05	0.27
6252	RTN1	0.00	0.00	0.00	NaN
6253	RTN2	0.09	0.00	0.00	0.08
6256	RXRA	0.00	0.00	0.00	NaN
6257	RXRB	0.09	0.00	0.00	0.18
6258	RXRG	0.00	0.00	0.00	NaN
6259	RYK	0.00	0.00	0.00	NaN
6261	RYR1	0.00	0.00	0.00	NaN
6262	RYR2	0.00	0.00	0.00	NaN

6263	RYR3	0.00	0.00	0.00	NaN
6271	S100A1	0.09	0.13	0.00	0.18
6272	SORT1	0.09	0.00	0.05	0.40
6273	S100A2	0.09	0.00	0.00	-0.15
6274	S100A3	0.09	0.13	0.00	-0.18
6275	S100A4	0.00	0.00	0.00	NaN
6276	S100A5	0.00	0.00	0.00	NaN
6277	S100A6	0.00	0.00	0.00	NaN
6278	S100A7	0.00	0.00	0.00	NaN
6279	S100A8	0.00	0.00	0.00	NaN
6280	S100A9	0.00	0.00	0.00	NaN
6281	S100A10	0.00	0.00	0.50	0.10
6282	S100A11	0.00	0.00	0.05	-0.03
6283	S100A12	0.00	0.00	0.00	NaN
6284	S100A13	0.09	0.13	0.00	0.34
6285	S100B	0.00	0.00	0.00	NaN
6286	S100P	0.00	-0.13	-0.10	0.08
6293	SACM2L	0.09	0.00	0.00	0.12
6294	SAFB	0.00	0.00	0.00	NaN
6295	SAG	0.00	0.00	0.00	NaN
6296	SAH	0.00	0.00	0.00	NaN
6297	SALL2	0.00	0.00	0.00	NaN
6299	SALL1	0.00	0.00	-0.05	0.15
6301	SARS	0.09	0.00	0.05	0.58
6302	SAS	0.00	0.00	0.00	NaN
6304	SATB1	0.00	0.00	0.00	NaN
6305	SBF1	0.00	-0.25	0.00	0.13
6307	SC4MOL	0.00	0.00	0.00	NaN
6309	SC5DL	-0.09	0.00	-0.10	0.44
6310	SCA1	0.00	0.00	0.00	NaN
6311	SCA2	0.00	0.00	0.00	NaN
6314	SCA7	0.09	0.00	-0.05	0.45
6317	SERPINB3	-0.09	0.00	-0.10	0.11
6318	SERPINB4	-0.09	0.00	-0.15	0.08
6319	SCD	0.00	-0.13	-0.05	0.36
6320	SCGF	0.00	0.00	0.00	NaN
6323	SCN1A	0.00	0.00	0.00	NaN
6324	SCN1B	0.00	0.00	0.00	NaN
6326	SCN2A2	0.00	0.00	0.00	NaN
6327	SCN2B	-0.09	0.00	0.00	-0.01
6328	SCN3A	0.00	0.00	0.00	NaN
6329	SCN4A	0.27	0.13	0.00	0.09
6331	SCN5A	0.00	0.00	0.00	NaN
6332	SCN6A	0.00	0.00	0.00	NaN
6334	SCN8A	0.00	0.00	0.00	NaN
6335	SCN9A	0.00	0.00	0.00	NaN
6336	SCN10A	0.00	0.00	0.00	NaN

6337	SCNN1A	-0.09	0.00	0.00	0.01
6338	SCNN1B	0.00	0.00	-0.05	0.16
6339	SCNN1D	0.00	0.00	0.00	NaN
6340	SCNN1G	0.00	0.00	-0.05	0.12
6342	SCP2	0.00	0.00	0.00	NaN
6344	SCTR	0.00	0.00	0.00	NaN
6346	SCYA1	0.00	-0.13	0.00	-0.07
6347	SCYA2	0.00	-0.13	0.00	-0.07
6348	SCYA3	0.00	0.13	0.00	-0.04
6351	SCYA4	0.00	0.13	0.00	-0.10
6352	SCYA5	0.00	0.00	0.00	0.08
6355	SCYA8	0.00	-0.13	0.00	-0.28
6356	SCYA11	0.00	-0.13	0.00	-0.26
6357	SCYA13	0.00	0.00	0.50	0.07
6358	SCYA14	0.00	0.50	0.00	-0.16
6359	SCYA15	0.00	0.50	0.00	-0.03
6360	SCYA16	0.00	0.00	0.00	-0.28
6361	SCYA17	-0.09	0.00	0.00	0.28
6362	SCYA18	0.00	1.00	0.00	-0.07
6363	SCYA19	-0.09	0.13	0.10	-0.07
6364	SCYA20	0.00	0.00	0.00	NaN
6366	SCYA21	-0.09	0.13	0.10	-0.16
6367	SCYA22	-0.09	0.00	0.00	-0.46
6368	SCYA23	0.00	0.00	0.00	-0.01
6369	SCYA24	-0.09	0.00	0.00	-0.12
6370	SCYA25	0.00	0.00	0.00	NaN
6372	SCYB6	0.00	0.00	0.00	NaN
6373	SCYB11	0.00	0.00	0.00	NaN
6374	SCYB5	0.00	0.00	0.00	NaN
6375	SCYC1	0.00	0.00	0.00	NaN
6376	SCYD1	-0.09	0.00	0.00	0.05
6382	SDC1	0.09	0.00	-0.05	0.20
6383	SDC2	0.00	-0.25	0.00	0.22
6385	SDC4	0.00	0.00	0.00	NaN
6386	SDCBP	0.00	0.00	0.00	NaN
6387	SDF1	0.00	0.00	0.00	NaN
6388	SDF2	0.00	0.38	0.00	0.39
6389	SDHA	0.00	0.00	0.00	0.03
6390	SDHB	-0.09	0.00	0.00	0.44
6391	SDHC	0.00	0.00	0.05	0.36
6392	SDHD	0.00	0.00	0.00	NaN
6396	SEC13L1	0.00	0.00	0.00	NaN
6397	SEC14L1	0.00	0.13	0.00	0.39
6398	SECTM1	0.00	0.00	0.00	NaN
6400	SEL1L	0.00	0.00	0.00	NaN
6401	SELE	0.00	0.00	0.00	NaN
6402	SELL	0.00	0.00	0.00	NaN

6403	SELP	0.00	0.00	0.00	NaN
6404	SELPLG	0.00	-0.13	0.00	-0.01
6405	SEMA3F	-0.09	0.00	-0.05	0.19
6406	SEMG1	0.00	0.13	0.00	0.43
6414	SEPP1	0.00	0.00	0.05	-0.19
6415	SEPW1	0.09	0.00	0.00	0.25
6416	MAP2K4	0.00	0.00	0.00	NaN
6418	SET	0.00	0.00	0.00	NaN
6419	SETMAR	0.00	0.00	-0.05	0.18
6421	SFPQ	0.00	0.00	0.00	NaN
6422	SFRP1	0.45	-0.25	-0.05	-0.07
6424	SFRP4	0.00	0.00	0.00	NaN
6425	SFRP5	0.00	0.00	-0.05	0.14
6426	SFRS1	0.09	-0.13	0.00	0.34
6427	SFRS2	0.00	0.13	0.00	0.18
6428	SFRS3	0.00	0.00	0.05	-0.07
6429	SFRS4	0.00	0.00	0.00	NaN
6430	SFRS5	0.00	0.00	0.00	NaN
6431	SFRS6	0.00	-0.13	0.00	0.00
6432	SFRS7	0.00	0.13	0.00	0.09
6433	SFRS8	0.00	0.00	0.00	NaN
6434	SFRS10	0.09	0.00	0.05	0.39
6439	SFTPB	0.00	0.00	-0.05	0.24
6440	SFTPC	-0.09	0.00	0.00	0.28
6441	SFTPD	0.09	0.00	0.05	0.10
6442	SGCA	0.00	0.25	0.00	0.18
6443	SGCB	0.00	0.00	0.00	NaN
6444	SGCD	0.00	0.00	-0.05	0.15
6445	SGCG	0.50	0.00	0.00	0.76
6446	SGK	0.00	0.00	-0.05	0.19
6447	SGNE1	0.00	0.00	0.00	NaN
6448	SGSH	0.00	0.00	0.00	NaN
6449	SGT	0.00	0.00	0.00	NaN
6450	SH3BGR	0.00	0.00	0.00	NaN
6452	SH3BP2	0.00	-0.13	-0.15	0.36
6453	ITSN1	0.00	0.00	0.00	NaN
6455	SH3GL1	0.00	0.00	0.00	NaN
6456	SH3GL2	0.00	-0.13	-0.20	-0.04
6457	SH3GL3	0.00	0.00	-0.05	0.11
6461	SHB	-0.09	0.00	0.10	0.58
6462	SHBG	0.00	0.00	0.05	-0.01
6464	SHC1	0.09	0.13	0.00	0.08
6468	SHFM3	0.00	-0.13	-0.05	0.18
6469	SHH	0.00	0.00	0.00	NaN
6470	SHMT1	0.00	0.00	0.00	NaN
6472	SHMT2	0.00	0.00	0.00	NaN
6474	SHOX2	0.00	0.00	0.00	NaN

6476	SI	0.00	0.00	0.00	NaN
6477	SIAH1	-0.09	0.00	0.00	0.55
6478	SIAH2	0.09	0.00	0.00	-0.01
6480	SIAT1	0.09	0.00	0.05	0.10
6482	SIAT4A	0.00	0.00	0.05	0.37
6483	SIAT4B	-0.09	-0.13	0.05	0.02
6484	SIAT4C	-0.09	0.00	-0.15	0.34
6489	SIAT8A	0.00	0.00	0.00	NaN
6490	SILV	0.00	0.00	0.00	NaN
6491	SIL	0.00	0.00	0.00	NaN
6493	SIM2	0.00	0.00	0.00	NaN
6494	SIPA1	-0.09	0.00	0.05	0.13
6495	SIX1	0.00	0.00	0.00	NaN
6496	SIX3	0.00	0.00	0.00	NaN
6497	SKI	0.00	0.00	0.00	NaN
6498	SKIL	0.09	0.00	0.00	0.23
6499	SKIV2L	0.00	0.00	0.00	NaN
6500	SKP1A	0.00	-0.13	0.00	0.40
6502	SKP2	0.00	0.00	0.10	0.36
6503	SLA	0.00	0.00	0.05	-0.14
6504	SLAM	0.00	0.00	0.00	NaN
6505	SLC1A1	-0.09	-0.13	-0.15	0.34
6506	SLC1A2	0.00	0.13	0.05	-0.15
6507	SLC1A3	0.00	0.00	0.10	-0.11
6508	SLC4A3	0.00	0.00	0.00	NaN
6509	SLC1A4	0.00	0.00	0.00	NaN
6510	SLC1A5	0.09	0.00	0.00	0.47
6511	SLC1A6	0.00	0.00	0.00	NaN
6512	SLC1A7	0.00	0.00	0.00	NaN
6513	SLC2A1	0.00	0.00	0.00	NaN
6514	SLC2A2	0.09	0.00	0.00	0.21
6515	SLC2A3	-0.09	0.00	0.00	0.11
6517	SLC2A4	0.00	0.00	0.00	NaN
6518	SLC2A5	0.00	0.00	0.00	NaN
6519	SLC3A1	0.00	0.00	0.00	NaN
6520	SLC3A2	0.00	0.00	0.00	NaN
6521	SLC4A1	0.00	-0.13	0.00	0.14
6522	SLC4A2	0.00	0.00	-0.05	0.32
6523	SLC5A1	0.00	0.00	0.00	NaN
6524	SLC5A2	0.00	0.00	0.00	NaN
6525	SMTN	0.00	0.00	0.00	NaN
6527	SLC5A4	0.00	0.00	0.00	NaN
6528	SLC5A5	0.00	0.00	0.00	NaN
6529	SLC6A1	0.00	0.00	0.00	NaN
6530	SLC6A2	0.33	0.00	0.33	0.82
6531	SLC6A3	0.00	0.13	0.10	-0.12
6532	SLC6A4	0.00	0.38	0.00	0.25

6533	SLC6A6	0.00	0.00	0.00	NaN
6534	SLC6A7	0.00	0.00	0.00	NaN
6536	SLC6A9	0.00	0.00	0.00	NaN
6538	SLC6A11	0.00	0.00	0.00	NaN
6539	SLC6A12	0.00	0.00	0.00	0.10
6540	SLC6A13	0.00	0.00	0.05	0.22
6541	SLC7A1	0.00	0.00	-0.05	0.01
6542	SLC7A2	0.00	-0.13	-0.05	0.01
6543	SLC8A2	0.09	0.00	0.00	0.06
6545	SLC7A4	0.00	0.13	0.05	0.12
6546	SLC8A1	0.00	0.00	0.00	NaN
6548	SLC9A1	-0.09	-0.13	0.00	0.12
6549	SLC9A2	0.00	0.00	0.00	NaN
6550	SLC9A3	0.00	0.13	0.10	-0.01
6553	SLC9A5	-0.09	0.00	0.00	0.22
6554	SLC10A1	0.00	0.00	0.00	NaN
6555	SLC10A2	-0.09	-0.13	0.05	-0.03
6556	SLC11A1	0.00	0.00	0.00	NaN
6557	SLC12A1	0.00	0.00	0.00	NaN
6558	SLC12A2	0.00	0.00	0.00	NaN
6559	SLC12A3	-0.09	0.00	0.00	-0.07
6560	SLC12A4	-0.09	-0.13	0.00	-0.01
6561	SLC13A1	0.00	0.13	0.00	0.06
6563	SLC14A1	-0.09	-0.13	-0.20	-0.01
6564	SLC15A1	-0.09	-0.13	-0.05	0.38
6565	SLC15A2	0.00	0.00	0.05	0.43
6566	SLC16A1	0.00	0.00	0.05	0.25
6568	SLC17A1	0.00	0.00	0.05	0.19
6569	SLC34A1	0.00	0.50	0.50	0.12
6570	SLC18A1	0.00	0.00	0.00	-0.03
6571	SLC18A2	0.00	0.00	-0.05	0.02
6572	SLC18A3	0.00	0.00	0.00	NaN
6573	SLC19A1	0.00	0.00	0.00	NaN
6574	SLC20A1	0.00	0.00	0.00	NaN
6575	SLC20A2	0.36	0.00	-0.05	0.29
6576	SLC25A1	-0.09	0.00	0.00	0.12
6578	SLC21A2	0.00	0.00	0.00	NaN
6579	SLC21A3	0.00	0.00	0.00	NaN
6580	SLC22A1	0.00	0.00	0.00	NaN
6581	SLC22A3	0.00	0.00	-0.05	-0.06
6582	SLC22A2	0.00	0.00	0.00	NaN
6583	SLC22A4	0.00	-0.13	0.00	0.23
6584	SLC22A5	0.00	-0.13	0.00	-0.12
6585	SLIT1	0.00	0.00	0.00	0.03
6588	SLN	0.00	0.00	0.00	NaN
6590	SLPI	0.00	0.00	0.00	NaN
6591	SNAI2	0.00	0.13	0.00	-0.12

6595	SMARCA2	-0.09	-0.13	-0.05	0.48
6596	SMARCA3	0.00	0.00	0.05	0.46
6597	SMARCA4	0.00	0.00	0.00	NaN
6598	SMARCB1	0.00	0.00	0.05	0.37
6599	SMARCC1	-0.09	0.00	-0.05	0.52
6601	SMARCC2	0.00	0.00	0.00	NaN
6602	SMARCD1	0.00	0.00	0.00	NaN
6604	SMARCD3	0.00	0.00	-0.05	0.20
6605	SMARCE1	0.00	0.38	0.00	0.46
6606	SMN1	1.00	0.00	0.00	0.06
6608	SMOH	0.00	0.00	0.00	NaN
6609	SMPD1	0.00	-0.13	0.00	0.15
6610	SMPD2	0.00	0.00	0.05	0.44
6612	SMT3H1	0.00	0.00	0.00	NaN
6613	SMT3H2	0.00	0.13	0.00	0.45
6614	SN	0.00	0.00	0.00	NaN
6615	SNAI1	0.27	0.38	0.05	0.07
6616	SNAP25	0.00	0.00	0.05	0.00
6617	SNAPC1	0.00	0.00	0.00	NaN
6618	SNAPC2	0.00	0.00	0.00	NaN
6619	SNAPC3	0.00	-0.13	0.00	0.48
6620	SNCB	0.00	0.00	0.00	NaN
6621	SNAPC4	0.00	0.00	0.00	NaN
6622	SNCA	0.00	0.00	0.00	NaN
6623	SNCG	0.09	0.00	0.00	0.13
6624	SNL	0.00	0.00	0.10	0.48
6625	SNRP70	0.09	0.00	0.00	0.25
6626	SNRPA	0.00	0.00	0.00	NaN
6627	SNRPA1	0.00	0.00	0.00	NaN
6628	SNRPB	0.00	0.00	0.05	0.26
6629	SNRPB2	0.00	0.00	0.00	NaN
6632	SNRPD1	0.00	0.00	0.00	NaN
6633	SNRPD2	0.09	0.00	0.00	0.32
6634	SNRPD3	0.00	0.00	0.05	-0.17
6635	SNRPE	0.00	0.13	0.00	0.16
6636	SNRPF	0.00	0.13	0.00	0.22
6637	SNRPG	0.00	0.00	0.00	NaN
6638	SNRPN	0.00	0.00	0.00	NaN
6640	SNTA1	0.00	0.00	0.00	NaN
6641	SNTB1	0.00	0.38	0.15	0.36
6642	SNX1	0.00	0.00	0.00	NaN
6643	SNX2	0.00	0.00	0.00	NaN
6645	SNTB2	-0.09	-0.13	0.05	0.41
6646	SOAT1	0.00	0.00	0.00	NaN
6647	SOD1	0.00	0.00	0.00	NaN
6648	SOD2	0.00	0.00	0.00	NaN
6649	SOD3	0.00	0.00	-0.05	-0.10

6650	SOLH	0.00	0.00	-0.05	0.34
6651	SON	0.00	0.00	0.00	NaN
6652	SORD	0.00	0.00	0.00	NaN
6653	SORL1	-0.09	0.00	-0.10	0.26
6654	SOS1	0.00	0.13	0.00	-0.03
6655	SOS2	0.00	0.00	0.00	NaN
6656	SOX1	-0.09	-0.13	-0.05	-0.09
6657	SOX2	0.09	0.00	0.00	0.03
6659	SOX4	0.00	0.00	0.00	NaN
6660	SOX5	0.00	0.00	0.00	NaN
6662	SOX9	0.00	0.00	0.50	0.18
6663	SOX10	0.00	0.00	0.05	0.79
6664	SOX11	0.00	0.00	0.00	NaN
6665	SOX20	0.00	0.00	0.00	NaN
6666	SOX22	0.00	0.00	0.05	0.27
6668	SP2	0.00	0.00	0.00	0.38
6671	SP4	0.00	0.13	0.00	0.32
6672	SP100	0.00	0.00	0.00	NaN
6674	SPAG1	0.09	0.00	0.05	0.29
6675	UAP1	0.00	0.00	0.00	NaN
6676	SPAG4	0.00	0.00	0.00	NaN
6677	SPAM1	0.00	0.50	0.00	0.26
6678	SPARC	0.00	0.00	0.00	NaN
6683	SPG4	0.00	0.00	0.00	NaN
6687	SPG7	-0.09	-0.13	-0.05	0.67
6688	SPI1	0.00	0.00	0.00	NaN
6689	SPIB	0.00	0.00	0.00	NaN
6690	SPINK1	0.00	0.00	0.00	NaN
6691	SPINK2	0.00	0.00	0.00	NaN
6692	SPINT1	0.00	0.00	0.00	NaN
6693	SPN	0.00	0.00	0.00	NaN
6694	SPP2	0.00	0.00	0.00	NaN
6695	SPOCK	0.00	-0.13	0.00	0.07
6696	SPP1	0.00	0.00	0.00	NaN
6697	SPR	0.00	0.00	0.00	NaN
6698	SPRR1A	0.00	0.00	0.00	NaN
6699	SPRR1B	0.00	0.00	0.00	NaN
6701	SPRR2B	0.00	0.00	0.00	NaN
6702	SPRR2C	0.00	0.00	0.00	NaN
6707	SPRR3	0.00	0.00	0.00	NaN
6708	SPTA1	0.00	0.00	0.00	NaN
6709	SPTAN1	0.00	0.00	0.00	NaN
6710	SPTB	0.00	0.00	0.00	NaN
6711	SPTBN1	0.09	0.00	-0.05	0.19
6712	SPTBN2	0.00	0.00	0.05	-0.01
6713	SQLE	0.18	0.38	0.00	0.60
6714	SRC	0.00	-0.13	0.00	0.16

6715	SRD5A1	0.00	0.00	0.05	0.77
6716	SRD5A2	0.00	0.00	0.00	NaN
6717	SRI	0.00	0.00	0.00	NaN
6718	AKR1D1	0.00	0.00	0.00	NaN
6720	SREBF1	0.00	0.00	0.00	NaN
6721	SREBF2	0.00	0.00	0.05	0.04
6722	SRF	0.00	0.00	0.00	NaN
6723	SRM	0.00	0.00	0.05	-0.03
6726	SRP9	0.09	0.00	0.00	0.11
6727	SRP14	0.00	0.00	-0.05	0.49
6728	SRP19	0.00	0.00	0.00	NaN
6729	SRP54	0.00	0.00	0.00	NaN
6731	SRP72	0.00	0.00	0.00	NaN
6732	SRPK1	0.00	0.00	0.00	NaN
6733	SRPK2	0.09	0.00	0.00	0.35
6734	SRPR	-0.09	0.00	-0.15	0.51
6737	SSA1	0.00	-0.13	0.00	0.20
6738	SSA2	0.09	0.00	0.00	0.36
6741	SSB	0.00	0.00	0.00	NaN
6742	SSBP1	0.00	0.00	0.00	NaN
6744	SSFA2	0.00	0.00	0.00	NaN
6745	SSR1	0.00	-0.13	0.00	0.40
6746	SSR2	0.00	0.13	0.00	0.35
6747	SSR3	0.00	0.00	0.00	NaN
6749	SSRP1	0.00	0.00	0.00	NaN
6750	SST	0.00	0.00	0.10	-0.19
6751	SSTR1	0.00	0.00	0.00	NaN
6752	SSTR2	0.00	0.00	0.00	NaN
6753	SSTR3	0.00	0.00	0.00	NaN
6754	SSTR4	0.00	0.00	0.00	NaN
6755	SSTR5	0.00	0.00	-0.05	-0.17
6760	SS18	0.00	0.00	-0.10	0.35
6764	ST5	0.00	0.00	-0.05	0.22
6767	ST13	0.00	0.50	0.50	0.22
6768	ST14	-0.09	0.00	-0.10	0.19
6769	STAC	0.00	0.00	0.00	NaN
6772	STAT1	0.00	0.00	0.00	NaN
6773	STAT2	0.00	0.00	0.00	NaN
6774	STAT3	0.00	0.13	0.00	0.27
6775	STAT4	0.00	0.00	0.00	NaN
6776	STAT5A	0.00	0.13	0.00	0.05
6777	STAT5B	0.00	0.13	0.00	0.33
6778	STAT6	0.00	0.00	0.00	NaN
6779	STATH	0.00	0.00	0.00	NaN
6780	STAU	0.09	0.00	0.00	0.45
6781	STC1	-0.09	-0.25	-0.15	0.14
6782	STCH	0.00	-0.25	-0.05	0.44

6783	STE	0.00	0.00	0.00	NaN
6785	ELOVL4	0.00	0.00	0.00	NaN
6786	STIM1	0.00	-0.13	0.00	0.08
6787	NEK4	-0.09	0.00	-0.10	0.31
6788	STK3	0.00	0.00	0.00	NaN
6789	STK4	0.09	0.13	0.00	0.02
6790	STK6	0.09	0.50	0.00	0.38
6793	STK10	0.00	0.00	0.00	NaN
6794	STK11	0.00	0.00	0.00	NaN
6795	STK13	0.09	0.13	0.00	0.27
6799	SULT1A2	0.00	0.00	0.00	NaN
6801	STRN	0.00	0.00	0.00	NaN
6804	STX1A	-0.09	0.00	0.00	0.12
6809	STX3A	0.00	0.00	0.00	NaN
6810	STX4A	0.00	0.00	0.00	NaN
6811	STX5A	0.00	0.00	0.00	NaN
6812	STXBP1	-0.09	0.00	-0.05	0.19
6813	STXBP2	0.00	0.00	0.00	NaN
6814	STXBP3	0.00	0.00	0.05	0.53
6817	SULT1A1	0.00	0.00	0.00	NaN
6818	SULT1A3	0.00	0.00	0.00	NaN
6819	SULT1C1	0.00	0.00	0.00	NaN
6820	SULT2B1	0.09	0.00	0.00	0.22
6821	SUOX	0.00	0.00	0.00	NaN
6822	SULT2A1	0.09	0.00	0.00	0.09
6827	SUPT4H1	0.09	0.00	0.00	0.58
6829	SUPT5H	0.00	0.00	0.00	NaN
6830	SUPT6H	0.00	0.25	0.00	0.65
6832	SUPV3L1	0.00	0.00	0.00	NaN
6833	ABCC8	0.00	0.00	0.00	NaN
6834	SURF1	0.00	0.00	0.00	NaN
6835	SURF2	0.00	0.00	0.00	NaN
6837	SURF5	0.00	0.50	0.00	0.11
6840	SVIL	-0.09	0.00	0.05	0.49
6843	VAMP1	-0.09	0.00	0.00	0.13
6844	VAMP2	0.00	0.00	0.00	NaN
6846	SCYC2	0.00	0.00	0.00	NaN
6847	SYCP1	0.18	0.00	0.10	0.23
6850	SYK	0.00	0.00	0.00	NaN
6854	SYN2	0.09	0.00	0.00	0.16
6856	SYPL	0.09	0.00	0.00	0.25
6857	SYT1	0.00	0.00	0.00	NaN
6861	SYT5	0.09	0.00	0.00	0.70
6862	T	0.00	0.00	0.00	NaN
6863	TAC1	0.18	0.00	0.00	-0.03
6865	TACR2	0.00	0.00	0.00	NaN
6866	TAC3	0.00	0.00	0.00	NaN

6867	TACC1	0.27	-0.13	0.00	0.41
6868	ADAM17	0.50	0.00	0.00	0.31
6869	TACR1	0.00	0.00	0.00	NaN
6870	TACR3	0.00	0.00	0.00	NaN
6871	TADA2L	0.00	0.00	0.00	0.53
6874	TAF4	0.18	0.25	0.10	0.13
6875	TAF4B	0.00	0.00	-0.15	0.28
6876	TAGLN	-0.09	0.00	0.00	0.12
6877	TAF5	0.00	-0.13	-0.05	0.15
6878	TAF6	0.18	0.00	0.00	0.23
6879	TAF7	0.00	0.00	0.00	NaN
6880	TAF9	0.00	0.00	0.00	NaN
6881	TAF10	0.00	-0.13	0.00	0.27
6883	TAF12	0.00	0.00	0.00	NaN
6884	TAF13	0.09	0.00	0.05	0.41
6885	MAP3K7	0.00	0.00	0.00	NaN
6886	TAL1	0.00	0.00	0.00	NaN
6888	TALDO1	-0.18	-0.13	-0.05	0.39
6890	TAP1	0.09	0.00	0.00	0.05
6891	TAP2	0.09	0.00	0.00	0.05
6894	TARBP1	0.09	0.00	0.00	0.03
6895	TARBP2	0.00	0.00	0.00	NaN
6897	TARS	0.00	0.00	0.10	0.37
6898	TAT	0.00	0.13	0.05	0.53
6899	TBX1	-0.09	0.00	0.00	0.15
6900	CNTN2	0.00	0.13	0.00	-0.18
6902	TBCA	0.00	0.00	0.00	NaN
6903	TBCC	0.00	0.00	0.00	NaN
6904	TBCD	0.00	0.00	0.00	NaN
6905	TBCE	0.09	0.00	0.00	0.27
6908	TBP	0.00	0.00	0.00	NaN
6909	TBX2	0.27	0.13	0.00	0.70
6910	TBX5	0.00	0.00	0.00	NaN
6911	TBX6	0.00	0.00	0.00	NaN
6915	TBXA2R	0.00	0.00	0.00	NaN
6916	TBXAS1	0.00	0.00	0.00	NaN
6917	TCEA1	0.00	0.00	0.00	NaN
6919	TCEA2	0.00	0.00	0.00	NaN
6921	TCEB1	0.00	0.25	0.00	0.48
6923	TCEB2	0.00	0.13	0.00	0.32
6924	TCEB3	-0.09	0.00	0.00	0.31
6925	TCF4	-0.09	0.00	-0.20	0.08
6926	TBX3	0.00	0.00	0.00	NaN
6927	TCF1	0.00	-0.13	0.00	-0.21
6928	TCF2	0.00	0.13	0.00	0.42
6929	TCF3	0.00	0.00	0.00	NaN
6932	TCF7	0.00	-0.13	0.00	0.08

6934	TCF7L2	0.00	0.00	-0.05	0.21
6935	TCF8	-0.09	-0.13	0.05	0.07
6936	C2orf3	0.00	0.00	0.00	NaN
6938	TCF12	0.00	0.00	0.05	0.20
6939	TCF15	0.00	0.00	0.00	0.25
6940	ZNF354A	0.00	0.00	0.00	NaN
6942	TCF20	0.00	0.00	0.00	NaN
6943	TCF21	0.00	0.00	0.00	NaN
6944	TCFL1	0.00	0.00	0.05	0.50
6945	TCFL4	0.00	-0.13	0.00	0.17
6947	TCN1	0.00	0.00	0.00	NaN
6948	TCN2	0.00	0.00	0.00	NaN
6949	TCOF1	0.00	0.00	0.00	NaN
6950	TCP1	0.00	0.00	0.00	NaN
6953	TCP10	0.00	0.00	0.00	NaN
6954	TCP11	0.00	0.00	0.00	NaN
6988	TCTA	-0.09	0.00	-0.05	-0.06
6992	PPP1R11	0.00	0.00	0.00	NaN
6993	TCTEL1	0.00	0.00	0.00	NaN
6996	TDG	0.00	0.00	0.00	NaN
6997	TDGF1	-0.09	0.00	-0.05	-0.07
6999	TDO2	0.50	0.00	0.50	0.15
7001	PRDX2	0.00	0.00	0.00	NaN
7003	TEAD1	0.00	0.00	0.00	NaN
7004	TEAD4	0.00	0.00	0.05	0.14
7005	TEAD3	0.00	0.00	0.00	NaN
7006	TEC	0.00	0.00	0.00	NaN
7007	TECTA	-0.09	0.00	-0.10	0.24
7008	TEF	0.00	0.00	0.05	-0.11
7009	TEGT	0.00	0.00	0.00	NaN
7010	TEK	0.00	-0.13	-0.05	0.21
7013	TERF1	0.00	0.25	0.00	0.54
7014	TERF2	0.00	-0.13	0.05	0.28
7015	TERT	0.00	0.13	0.10	0.12
7016	TESK1	-0.09	0.00	0.10	0.56
7019	TFAM	0.00	0.00	0.00	NaN
7020	TFAP2A	0.00	0.00	0.05	0.14
7021	TFAP2B	0.00	0.00	0.00	NaN
7022	TFAP2C	0.18	0.50	0.00	0.50
7023	TFAP4	0.00	0.00	0.00	NaN
7024	TFCP2	0.00	0.00	0.00	NaN
7025	NR2F1	0.00	0.00	0.00	NaN
7027	TFDP1	-0.09	-0.13	-0.05	0.45
7029	TFDP2	0.00	0.00	0.00	NaN
7031	TFF1	0.00	0.00	0.00	NaN
7032	TFF2	0.00	0.00	0.00	NaN
7033	TFF3	0.00	0.00	0.00	NaN

7035	TFPI	0.00	0.00	0.00	NaN
7036	TFR2	0.18	0.00	0.00	0.11
7037	TFRC	0.18	0.00	0.00	0.34
7038	TG	0.09	0.00	0.10	0.07
7039	TGFA	0.00	0.13	0.00	0.15
7040	TGFB1	0.00	0.00	-0.05	-0.25
7042	TGFB2	0.09	0.00	0.05	0.10
7043	TGFB3	0.00	0.00	0.00	NaN
7044	EBAF	0.09	0.00	0.00	0.21
7045	TGFBI	0.00	-0.13	0.00	0.06
7046	TGFBR1	0.00	0.00	0.00	NaN
7047	TGM4	-0.09	0.00	-0.05	0.04
7048	TGFBR2	0.00	0.00	0.00	NaN
7049	TGFBR3	0.00	0.00	0.00	NaN
7050	TGIF	0.00	0.00	0.00	NaN
7051	TGM1	0.00	0.00	0.00	NaN
7052	TGM2	0.00	-0.13	0.00	0.02
7053	TGM3	0.00	0.00	0.05	-0.08
7054	TH	0.00	-0.13	0.00	-0.03
7056	THBD	0.00	0.00	0.00	NaN
7057	THBS1	0.00	0.00	-0.05	0.09
7058	THBS2	0.00	0.00	0.00	NaN
7059	THBS3	0.27	0.25	0.00	0.19
7060	THBS4	0.00	0.00	-0.05	0.09
7064	THOP1	0.00	0.00	0.00	NaN
7066	THPO	0.50	0.00	0.00	0.11
7067	THRA	0.00	0.50	0.00	0.29
7068	THRB	0.00	0.00	0.00	NaN
7070	THY1	-0.09	0.00	-0.10	0.10
7071	TIEG	0.18	0.00	0.00	-0.03
7072	TIA1	0.00	0.00	0.00	NaN
7073	TIAL1	0.00	0.00	-0.05	0.24
7074	TIAM1	0.00	0.00	0.00	NaN
7075	TIE	0.00	0.00	0.00	NaN
7077	TIMP2	0.00	0.25	0.00	0.26
7078	TIMP3	0.00	0.00	0.00	NaN
7079	TIMP4	0.09	0.00	0.00	-0.07
7080	TITF1	0.00	0.00	0.00	NaN
7082	TJP1	0.00	-0.13	0.00	0.34
7083	TK1	0.00	0.50	0.00	0.16
7087	ICAM5	0.00	0.00	0.05	-0.03
7088	TLE1	0.00	0.00	0.00	NaN
7089	TLE2	0.00	0.00	0.00	NaN
7090	TLE3	0.00	0.00	0.00	NaN
7091	TLE4	0.00	0.00	0.00	NaN
7092	TLL1	0.00	0.00	0.00	NaN
7093	TLL2	0.00	0.00	-0.05	-0.21

7095	TLOC1	0.09	0.00	0.00	0.30
7096	TLR1	0.00	0.00	0.05	-0.15
7097	TLR2	0.00	0.00	-0.05	0.12
7098	TLR3	0.00	0.00	-0.05	0.13
7099	TLR4	0.00	0.00	0.00	NaN
7100	TLR5	0.09	0.00	0.00	0.40
7101	NR2E1	0.00	0.00	0.10	-0.03
7103	TM4SF3	0.09	0.00	0.00	-0.07
7104	TM4SF4	0.00	0.00	0.05	0.16
7106	TM4SF7	-0.18	-0.13	-0.05	0.32
7107	TM7SF1	0.09	0.00	0.00	0.32
7108	TM7SF2	-0.09	0.00	0.00	0.06
7109	TMEM1	0.00	0.00	0.00	NaN
7110	TMF1	0.00	0.00	-0.05	0.13
7111	TMOD	0.00	0.00	0.00	NaN
7112	TMPO	0.00	0.13	0.00	0.28
7113	TMPRSS2	0.00	0.00	0.00	NaN
7122	CLDN5	-0.09	0.00	0.00	-0.15
7123	TNA	-0.09	0.00	-0.05	0.06
7124	TNF	0.00	0.00	0.00	NaN
7125	TNNC2	0.00	0.00	0.00	NaN
7126	TNFAIP1	0.00	0.25	0.00	0.39
7127	TNFAIP2	0.00	0.00	0.00	NaN
7128	TNFAIP3	0.00	0.00	0.00	NaN
7130	TNFAIP6	0.00	0.00	0.00	NaN
7132	TNFRSF1A	-0.09	0.00	0.00	0.43
7133	TNFRSF1B	0.00	0.00	0.00	NaN
7134	TNNC1	-0.09	-0.25	-0.10	0.15
7135	TNNI1	0.00	0.00	0.00	NaN
7136	TNNI2	0.00	-0.13	-0.05	0.09
7137	TNNI3	0.09	0.00	0.00	0.33
7139	TNNT2	0.00	0.00	0.00	NaN
7140	TNNT3	0.00	-0.13	-0.05	-0.60
7141	TNP1	0.00	0.00	0.00	NaN
7142	TNP2	0.00	0.00	0.00	NaN
7143	TNR	0.00	0.00	0.00	NaN
7145	TNS	0.00	0.00	0.00	NaN
7148	TNXB	0.00	0.00	0.00	NaN
7150	TOP1	0.18	-0.13	0.00	0.50
7153	TOP2A	0.00	0.38	0.00	0.40
7155	TOP2B	0.00	0.00	0.00	NaN
7156	TOP3A	0.00	0.00	0.00	NaN
7157	TP53	0.00	0.00	0.05	-0.13
7158	TP53BP1	0.00	0.00	0.00	NaN
7159	TP53BP2	0.09	0.00	0.00	0.25
7161	TP73	0.00	0.00	0.00	NaN
7162	TPBG	0.00	0.00	0.00	NaN

7163	TPD52	0.00	0.25	0.05	0.51
7164	TPD52L1	0.50	0.00	0.00	0.26
7167	TPI1	-0.09	0.00	0.00	0.39
7168	TPM1	0.00	0.00	0.00	NaN
7169	TPM2	-0.09	0.00	0.10	0.17
7171	TPM4	0.00	0.00	0.00	NaN
7172	TPMT	0.00	0.00	-0.05	0.06
7174	TPP2	-0.09	-0.13	0.05	0.48
7175	TPR	0.00	0.00	0.00	NaN
7177	TPSB1	0.00	0.00	-0.05	0.03
7178	TPT1	0.00	0.00	-0.05	0.15
7179	TPTE	-0.18	-0.13	-0.25	0.22
7180	TPX1	0.00	0.00	0.00	NaN
7181	NR2C1	0.00	0.13	0.00	0.34
7182	NR2C2	0.00	0.00	0.05	0.11
7184	TRA1	0.00	0.00	0.00	NaN
7185	TRAF1	0.00	0.00	-0.05	0.11
7186	TRAF2	0.00	0.00	0.00	NaN
7187	TRAF3	0.00	0.00	0.00	NaN
7188	TRAF5	0.00	0.00	0.00	NaN
7189	TRAF6	0.00	0.13	0.05	0.59
7200	TRH	0.00	0.00	0.00	NaN
7201	TRHR	0.18	0.00	0.00	0.15
7203	CCT3	0.00	0.13	0.00	0.16
7204	TRIO	0.00	0.00	0.20	0.45
7205	TRIP6	0.18	0.00	0.00	-0.09
7220	TRPC1	0.00	0.00	-0.05	0.12
7221	TRPC2	0.00	-0.13	0.00	0.11
7222	TRPC3	0.00	0.00	0.00	NaN
7223	TRPC4	0.00	0.00	0.00	NaN
7225	TRPC6	0.00	0.00	0.00	NaN
7226	TRPM2	0.00	-0.13	0.00	-0.17
7227	TRPS1	0.09	0.25	0.10	0.27
7247	TSN	0.00	0.00	0.00	NaN
7248	TSC1	0.00	0.00	0.00	NaN
7251	TSG101	0.00	0.00	0.00	NaN
7252	TSHB	0.18	0.00	0.10	0.03
7253	TSHR	0.00	0.00	0.00	NaN
7257	TSNAX	1.00	0.00	0.00	0.16
7259	TSPYL	-0.09	0.00	0.05	0.60
7260	TSSC1	0.00	0.00	0.00	NaN
7262	TSSC3	0.00	-0.13	-0.05	0.20
7263	TST	-0.09	0.00	0.00	0.18
7264	TSTA3	0.18	0.00	0.10	0.25
7265	TTC1	0.00	0.00	-0.05	-0.30
7266	DNAJC7	0.00	0.13	0.00	0.40
7267	TTC3	0.00	0.00	0.00	NaN

7268	TTC4	0.00	0.00	0.00	NaN
7272	TTK	0.00	0.00	0.00	NaN
7273	TTN	0.00	0.00	0.05	-0.11
7274	TTPA	0.00	0.00	0.00	NaN
7275	TUB	0.00	0.00	-0.05	0.25
7276	TTR	0.00	0.00	-0.10	-0.04
7277	TUBA1	0.00	0.00	0.00	NaN
7278	TUBA2	0.00	0.00	0.00	0.16
7280	TUBB	0.00	0.00	0.00	0.20
7283	TUBG1	0.00	-0.13	0.00	0.10
7284	TUFM	0.00	0.00	0.00	NaN
7286	TUFT1	0.00	0.00	0.05	0.11
7287	TULP1	0.00	0.00	0.00	NaN
7288	TULP2	0.09	0.00	0.00	-0.03
7289	TULP3	0.00	0.00	0.05	0.38
7290	HIRA	-0.09	0.00	0.00	0.36
7291	TWIST	0.00	0.13	0.05	0.15
7292	TNFSF4	0.00	0.00	0.00	NaN
7293	TNFRSF4	0.00	0.00	0.00	NaN
7294	TXK	0.00	0.00	0.00	NaN
7295	TXN	0.00	0.00	0.00	NaN
7296	TXNRD1	0.00	0.00	0.00	NaN
7297	TYK2	0.00	0.00	0.05	0.45
7298	TYMS	0.00	0.00	0.00	NaN
7299	TYR	0.00	0.00	0.05	-0.02
7301	TYRO3	0.00	0.00	0.00	NaN
7305	TYROBP	0.00	0.00	0.00	NaN
7306	TYRP1	0.00	0.00	0.05	0.10
7307	U2AF1	0.00	0.13	0.00	-0.11
7311	UBA52	0.00	0.00	0.00	NaN
7314	UBB	0.00	0.00	0.00	-0.18
7318	UBE1L	-0.09	0.00	-0.05	0.10
7320	UBE2B	0.00	-0.13	0.00	0.43
7321	UBE2D1	0.00	0.00	0.05	-0.08
7322	UBE2D2	0.00	0.00	0.00	NaN
7323	UBE2D3	0.00	0.00	-0.05	0.21
7324	UBE2E1	0.00	0.00	0.00	NaN
7326	UBE2G1	0.00	0.00	0.00	NaN
7327	UBE2G2	0.00	0.00	0.00	NaN
7328	UBE2H	0.00	0.25	0.05	0.26
7329	UBE2I	0.00	0.00	-0.05	0.52
7332	UBE2L3	0.00	0.00	0.00	NaN
7334	UBE2N	0.00	0.13	0.00	0.51
7336	UBE2V2	0.00	0.00	0.00	NaN
7337	UBE3A	0.00	0.00	0.00	NaN
7341	UBL1	0.50	0.00	0.50	0.13
7342	UBP1	0.00	0.00	0.00	NaN

7343	UBTF	0.00	-0.13	0.00	0.28
7345	UCHL1	0.00	0.00	0.05	0.39
7347	UCHL3	-0.09	0.13	-0.15	0.62
7348	UPK1B	0.00	0.00	0.00	NaN
7349	UCN	0.09	0.00	0.05	0.21
7350	UCP1	0.00	0.00	0.00	NaN
7351	UCP2	0.18	0.13	0.00	0.40
7352	UCP3	0.18	0.13	0.00	0.14
7353	UFD1L	-0.09	0.00	0.00	0.15
7356	SCGB1A1	0.00	0.00	0.00	NaN
7357	UGCG	0.00	0.00	0.00	NaN
7358	UGDH	0.00	0.00	0.00	NaN
7360	UGP2	0.00	0.00	0.00	NaN
7363	UGT2B4	0.00	0.00	0.00	NaN
7365	UGT2B10	0.00	0.00	0.05	-0.02
7366	UGT2B15	0.00	0.00	0.05	0.01
7367	UGT2B17	-0.18	-0.13	0.00	0.21
7368	UGT8	0.00	0.00	0.00	NaN
7369	UMOD	0.00	0.00	0.00	NaN
7371	UMPK	0.00	0.00	0.00	NaN
7372	UMPS	0.00	0.00	0.00	NaN
7373	COL14A1	0.00	0.25	0.15	0.14
7374	UNG	0.00	0.00	0.00	NaN
7375	USP4	-0.09	0.00	-0.05	0.43
7376	NR1H2	0.00	0.00	0.00	NaN
7378	UP	0.00	0.00	0.00	NaN
7379	UPK2	0.00	0.00	0.00	NaN
7380	UPK3	0.00	0.00	0.00	NaN
7381	UQCRB	0.00	-0.13	0.05	0.61
7384	UQCRC1	-0.09	0.00	-0.05	0.30
7385	UQCRC2	0.00	0.00	0.00	NaN
7386	UQCRFS1	0.00	0.13	0.10	0.70
7388	UQCRH	0.00	0.00	0.00	NaN
7389	UROD	0.00	0.00	0.00	NaN
7390	UROS	0.00	0.00	-0.05	0.26
7392	USF2	0.00	0.00	0.00	NaN
7398	USP1	0.00	0.00	0.00	NaN
7399	USH2A	0.09	0.00	0.00	0.28
7402	UTRN	0.00	0.00	0.00	NaN
7405	UVRAG	0.09	0.00	0.00	0.60
7407	VARS2	0.00	0.00	0.00	NaN
7408	VASP	0.09	0.00	0.00	-0.01
7409	VAV1	0.00	0.00	0.00	NaN
7410	VAV2	0.00	0.00	0.00	NaN
7412	VCAM1	0.09	0.00	0.00	-0.04
7414	VCL	0.09	0.00	0.00	0.14
7415	VCP	-0.09	0.13	0.10	0.67

7416	VDAC1	0.00	-0.13	0.00	0.28
7419	VDAC3	0.36	0.00	-0.05	0.77
7422	VEGF	0.00	0.00	0.00	NaN
7423	VEGFB	0.00	0.00	0.00	NaN
7424	VEGFC	0.00	0.13	-0.10	0.17
7425	VGF	0.18	0.00	0.00	0.47
7428	VHL	0.00	0.00	0.00	NaN
7429	VIL1	0.00	0.00	0.00	NaN
7430	VIL2	0.00	0.00	0.00	NaN
7431	VIM	0.00	0.00	0.05	0.00
7432	VIP	0.00	0.00	0.00	NaN
7433	VIPR1	0.00	0.00	-0.05	0.13
7434	VIPR2	0.00	0.00	-0.05	0.19
7436	VLDLR	0.00	1.00	1.00	0.34
7439	VMD2	-0.09	0.00	0.00	-0.21
7441	VPREB1	0.00	0.00	0.00	NaN
7442	TRPV1	0.00	0.00	0.00	NaN
7443	VRK1	0.00	0.00	0.00	NaN
7444	VRK2	0.00	0.00	0.00	NaN
7447	VSNL1	0.09	0.00	-0.10	-0.15
7448	VTN	0.00	0.25	0.00	0.45
7450	VWF	-0.09	0.00	0.00	-0.09
7453	WARS	0.00	0.00	0.00	NaN
7456	WASPIP	0.00	0.00	0.00	NaN
7458	WBSCR1	-0.09	0.00	0.00	0.37
7461	CYLN2	-0.09	0.00	0.00	0.09
7462	WBSCR5	-0.09	0.00	0.00	-0.12
7464	CORO2A	0.00	0.00	0.00	NaN
7465	WEE1	0.00	0.00	0.00	NaN
7466	WFS1	0.00	-0.13	-0.10	0.20
7468	WHSC1	0.00	-0.13	-0.15	0.46
7469	WHSC2	0.00	-0.13	-0.15	0.56
7471	WNT1	0.00	0.00	0.00	NaN
7472	WNT2	0.00	0.25	0.00	0.12
7473	WNT3	0.00	-0.13	0.00	-0.09
7474	WNT5A	0.00	0.00	-0.10	-0.05
7475	WNT6	0.00	0.00	0.00	NaN
7476	WNT7A	0.00	0.00	0.00	NaN
7477	WNT7B	0.00	0.00	0.00	NaN
7479	WNT8B	0.00	-0.13	-0.05	-0.09
7480	WNT10B	0.00	0.00	0.00	NaN
7481	WNT11	0.18	0.00	0.00	0.01
7482	WNT2B	0.09	0.00	0.05	0.08
7485	WRB	0.00	0.00	0.00	NaN
7486	WRN	-0.09	-0.25	-0.05	0.43
7490	WT1	0.00	0.00	0.00	NaN
7494	XBP1	-0.09	0.00	0.00	-0.19

7498	XDH	0.00	0.00	0.00	NaN
7507	XPA	0.00	0.00	0.00	NaN
7508	XPC	0.00	0.00	0.00	NaN
7511	XPNPEP1	0.00	0.00	-0.05	0.24
7514	XPO1	0.00	0.00	0.00	NaN
7515	XRCC1	0.09	0.00	-0.10	0.48
7516	XRCC2	0.00	0.00	-0.05	0.07
7518	XRCC4	0.00	-0.13	0.00	0.32
7520	XRCC5	0.00	0.00	0.00	NaN
7525	YES1	0.00	0.00	0.00	NaN
7528	YY1	0.00	0.00	0.00	NaN
7529	YWHAB	0.09	0.13	0.00	0.48
7531	YWHAE	0.00	-0.13	0.00	0.17
7533	YWHAH	0.00	0.00	0.00	NaN
7534	YWHAZ	0.18	0.00	0.00	0.25
7535	ZAP70	0.00	0.00	0.00	NaN
7538	ZFP36	0.00	0.00	0.00	NaN
7539	ZFP37	0.00	0.00	0.00	NaN
7541	ZFP161	0.00	0.00	0.00	NaN
7542	ZFPL1	-0.09	0.00	0.00	0.19
7545	ZIC1	0.00	0.00	0.00	NaN
7553	ZNF7	0.18	0.00	0.10	0.60
7554	ZNF8	0.00	0.13	0.05	0.68
7555	ZNF9	0.00	0.00	0.00	NaN
7556	ZNF10	0.00	0.00	0.00	NaN
7561	ZNF14	0.00	0.00	0.00	NaN
7564	ZNF16	0.18	0.00	0.10	0.31
7568	ZNF20	0.00	0.00	0.00	NaN
7570	ZNF22	0.00	0.00	0.00	NaN
7571	ZNF23	-0.09	0.13	0.05	0.47
7572	ZNF24	0.00	0.00	-0.20	0.59
7574	ZNF26	0.00	0.00	0.00	NaN
7584	ZNF35	-0.09	0.00	-0.05	0.15
7586	ZNF36	0.18	0.00	0.00	0.27
7587	ZNF37A	0.00	0.00	0.00	NaN
7589	ZNF38	0.18	0.00	0.00	0.38
7593	ZNF42	0.00	0.13	0.00	0.50
7594	ZNF43	0.00	-0.25	0.00	0.31
7596	ZNF45	0.09	0.00	-0.05	0.47
7597	ZNF46	0.09	0.00	0.00	-0.04
7625	ZNF74	0.00	0.13	0.05	0.31
7629	ZNF76	0.00	0.00	0.00	NaN
7633	ZNF79	0.00	0.00	-0.05	0.14
7634	ZNF80	0.00	0.00	0.00	NaN
7637	ZNF84	0.00	0.00	0.00	NaN
7638	ZNF221	0.09	0.00	0.00	0.36
7639	ZNF85	0.00	-0.25	0.00	0.28

7644	ZNF91	0.00	-0.25	0.00	0.30
7673	ZNF222	0.09	0.00	0.00	0.41
7678	ZNF124	0.00	0.13	0.00	0.12
7681	MKRN3	0.00	-0.13	0.00	-0.15
7690	ZNF131	0.00	0.00	0.05	0.23
7691	ZNF132	0.00	0.13	0.05	0.16
7692	ZNF133	0.00	0.00	0.00	NaN
7693	ZNF134	0.00	0.13	0.00	0.24
7695	ZNF136	0.00	0.00	0.00	NaN
7696	ZNF137	0.09	0.00	-0.05	0.16
7699	ZNF140	0.00	0.00	0.00	NaN
7700	ZNF141	0.00	-0.13	-0.10	0.28
7701	ZNF142	0.00	0.00	0.00	NaN
7702	ZNF143	0.00	0.00	0.00	NaN
7703	ZNF144	0.00	0.38	0.00	0.76
7704	ZNF145	-0.09	0.00	0.00	0.10
7705	ZNF146	0.00	0.00	0.00	NaN
7706	ZNF147	0.09	-0.13	0.00	0.27
7707	ZNF148	0.00	0.00	0.00	NaN
7709	ZNF151	0.00	0.00	0.00	NaN
7710	ZNF154	0.00	0.13	0.00	0.27
7711	ZNF155	0.09	0.00	0.00	0.44
7716	ZNF161	0.09	-0.13	0.00	0.36
7718	ZNF165	0.00	0.00	0.00	NaN
7726	TRIM26	0.00	0.00	0.00	NaN
7727	ZNF174	0.00	0.00	0.00	NaN
7728	ZNF175	0.00	0.13	-0.05	0.48
7730	ZNF177	0.50	0.50	0.50	0.19
7733	ZNF180	0.09	0.00	0.00	0.41
7738	ZNF184	0.00	0.00	0.00	NaN
7741	ZNF187	0.00	0.00	0.00	NaN
7743	ZNF189	0.00	0.00	0.00	NaN
7745	ZNF192	0.00	0.00	0.00	NaN
7746	ZNF193	0.00	0.00	0.00	NaN
7748	ZNF195	0.00	-0.13	0.00	0.12
7750	ZNF198	0.00	0.00	-0.05	0.35
7752	ZNF200	0.00	0.00	0.00	NaN
7753	ZNF202	0.00	0.00	-0.05	0.11
7755	ZNF205	0.00	0.00	0.00	NaN
7756	ZNF207	0.00	0.00	0.00	NaN
7757	ZNF208	0.00	-0.25	0.00	-0.16
7761	ZNF214	0.00	0.00	0.00	NaN
7762	ZNF215	0.00	0.00	0.00	NaN
7763	ZNF216	0.00	0.00	-0.05	0.06
7764	ZNF217	0.18	0.50	0.00	0.42
7766	ZNF223	0.09	0.00	0.00	0.06
7769	ZNF226	0.09	0.00	0.00	0.10

7773	ZNF230	0.09	0.00	0.00	0.47
7775	ZNF232	0.18	0.00	0.00	-0.26
7776	ZNF236	-0.09	0.00	-0.25	0.37
7781	SLC30A3	0.09	0.00	0.05	0.04
7782	SLC30A4	0.00	0.00	0.00	NaN
7783	ZP2	0.00	0.00	0.00	NaN
7786	MAP3K12	0.00	0.00	0.00	NaN
7791	ZYX	0.00	0.00	-0.05	0.21
7799	PRDM2	0.00	0.00	0.00	NaN
7802	P28	0.00	0.00	0.00	NaN
7803	PTP4A1	-0.09	0.13	0.00	0.25
7804	LRP8	0.00	0.00	0.00	NaN
7805	LAPTM5	0.00	0.00	0.00	NaN
7812	D1S155E	0.18	0.00	0.10	0.78
7813	EVI5	0.00	0.00	0.05	0.56
7818	DAP3	0.09	0.13	0.00	0.26
7827	NPHS2	0.00	0.00	0.00	NaN
7832	BTG2	0.00	0.13	0.00	0.49
7837	D2S448	0.00	0.00	0.00	NaN
7840	ALMS1	0.00	0.00	0.00	NaN
7841	GCS1	0.00	0.00	0.00	NaN
7844	ZFP103	0.00	0.00	-0.05	0.31
7846	TUBA3	0.00	0.00	0.00	NaN
7849	PAX8	0.00	0.00	0.00	NaN
7850	IL1R2	0.00	0.00	0.00	NaN
7851	BENE	0.09	0.00	0.00	-0.08
7852	CXCR4	0.00	0.00	0.00	0.12
7855	FZD5	0.00	0.00	0.00	NaN
7862	BRPF1	0.00	0.00	0.00	NaN
7866	IFRD2	-0.09	0.00	-0.05	0.39
7867	MAPKAPK3	-0.09	0.00	-0.05	0.32
7869	SEMA3B	-0.09	0.00	-0.05	0.17
7873	ARMET	-0.09	0.00	-0.10	-0.07
7874	USP7	0.00	0.00	0.00	NaN
7879	RAB7	0.00	0.00	0.00	NaN
7881	KCNAB1	0.00	0.00	0.00	NaN
7884	SLBP	0.00	-0.13	-0.15	0.41
7903	SIAT8D	0.00	0.00	0.00	NaN
7905	D5S346	0.00	0.00	0.05	0.09
7913	DEK	0.00	0.00	0.05	-0.35
7915	ALDH5A1	0.00	0.00	0.05	0.09
7916	BAT2	0.00	0.00	0.00	NaN
7917	BAT3	0.00	0.00	0.00	NaN
7919	BAT1	0.00	0.00	0.00	NaN
7922	HKE4	0.09	0.00	0.00	0.03
7932	OR2H3	0.00	0.00	0.00	NaN
7936	RDBP	0.00	0.00	0.00	NaN

7941	PLA2G7	0.00	0.00	0.00	NaN
7957	EPM2A	0.00	-0.13	0.05	0.67
7965	JTV1	0.00	0.00	0.10	0.35
7975	MAFK	0.00	0.13	0.05	0.35
7976	FZD3	-0.09	-0.25	-0.05	0.13
7978	MTERF	0.18	0.00	0.00	0.73
7979	DSS1	0.50	0.00	0.00	0.60
7980	TFPI2	0.18	0.00	0.00	-0.20
7982	ST7	0.00	0.25	0.00	0.63
7984	ARHGEF5	0.00	0.00	-0.05	-0.02
7988	ZNF212	0.00	0.00	-0.05	0.34
7991	N33	0.00	-0.13	-0.05	0.45
7993	D8S2298E	-0.09	-0.25	-0.05	0.32
7994	ZNF220	0.36	-0.13	-0.05	0.67
8000	PSCA	0.18	0.13	0.05	-0.17
8001	GLRA3	0.00	0.00	-0.05	0.18
8013	NR4A3	0.00	0.00	0.00	NaN
8019	BRD3	0.00	0.00	0.00	NaN
8021	NUP214	0.00	0.00	0.00	NaN
8022	LHX3	0.00	0.00	0.00	NaN
8027	STAM	0.00	0.00	0.05	0.27
8028	MLLT10	-0.09	0.00	0.05	0.55
8029	CUBN	0.00	0.00	0.05	-0.12
8030	D10S170	0.00	-0.13	0.05	0.05
8031	NCOA4	0.00	0.00	0.00	NaN
8034	SLC25A16	0.00	0.00	0.00	0.24
8036	SHOC2	0.00	0.00	-0.05	0.25
8038	ADAM12	0.00	0.00	-0.05	0.04
8045	C11orf13	-0.27	-0.13	-0.05	0.30
8048	CSRP3	0.00	0.00	0.50	0.00
8050	PDX1	0.00	0.13	0.05	0.53
8061	FOSL1	-0.09	0.00	0.05	0.27
8065	CUL5	0.00	0.00	0.00	NaN
8073	PTP4A2	0.00	0.00	0.00	NaN
8074	FGF23	-0.09	0.00	0.05	-0.09
8076	MAGP2	-0.09	0.00	0.00	0.14
8078	USP5	-0.09	0.00	0.00	0.22
8079	MLF2	0.00	0.50	0.00	0.41
8082	SSPN	0.00	0.00	0.00	NaN
8085	MLL2	0.00	0.00	0.00	NaN
8086	AAAS	0.00	0.00	0.00	NaN
8087	FXR1	0.09	0.00	0.00	0.32
8089	GAS41	0.09	0.00	0.00	0.37
8092	CART1	0.00	0.13	0.00	-0.04
8099	CDK2AP1	0.00	0.00	0.00	NaN
8100	TG737	0.00	0.00	-0.10	0.07
8106	PABPN1	0.00	0.00	0.00	NaN

8110	CERD4	0.00	0.00	0.00	NaN
8111	GPR68	0.00	0.13	0.00	0.12
8115	TCL1A	0.00	0.00	0.00	NaN
8120	AP3B2	0.00	0.00	-0.05	0.07
8123	D15S226E	0.00	0.00	0.00	NaN
8125	ANP32A	0.00	0.00	-0.05	0.57
8128	SIAT8B	0.00	0.00	0.00	NaN
8131	CGTHBA	0.00	0.00	0.00	0.10
8139	GAN	-0.09	0.00	-0.05	0.21
8140	SLC7A5	-0.09	-0.13	-0.05	0.25
8148	TAF15	0.00	0.00	0.00	0.34
8153	RHO7	0.00	-0.13	0.00	0.05
8161	COIL	0.09	-0.13	0.00	0.32
8165	AKAP1	0.09	-0.13	0.00	0.15
8170	SLC14A2	-0.09	-0.13	-0.20	-0.01
8174	MADCAM1	0.00	0.00	-0.05	-0.01
8175	SF3A2	0.00	0.00	0.00	NaN
8178	ELL	0.00	0.00	0.00	NaN
8187	ZNF239	0.00	0.00	0.00	NaN
8189	SPK	0.09	0.00	0.00	0.42
8190	MIA	0.00	0.00	0.00	NaN
8192	CLPP	0.00	0.00	0.00	NaN
8193	NEUD4	0.00	0.13	0.00	0.46
8195	MKKS	0.00	0.00	0.05	0.59
8200	GDF5	0.00	0.13	0.00	0.45
8202	NCOA3	0.18	0.13	0.00	0.92
8204	NRIP1	0.00	-0.13	-0.05	0.50
8208	CHAF1B	0.00	0.00	0.50	0.00
8209	C21orf33	0.00	0.00	0.00	NaN
8214	DGCR6	0.00	0.00	-0.05	-0.07
8216	LZTR1	0.00	0.13	0.00	0.22
8218	CLTCL1	-0.09	0.00	0.00	0.04
8220	DGSI	-0.09	0.00	0.00	0.43
8224	SYN3	0.00	0.00	0.00	NaN
8288	EPX	0.09	-0.13	0.00	-0.07
8289	SMARCF1	-0.09	0.00	0.00	0.16
8290	H3FT	0.09	0.00	0.00	-0.17
8291	DYSF	0.00	0.00	0.00	NaN
8292	COLQ	0.00	0.00	0.05	-0.20
8294	H4FM	0.00	0.00	0.15	0.19
8295	TRRAP	0.18	0.00	0.00	0.36
8301	PICALM	0.00	0.00	0.00	NaN
8302	KLRC4	-0.09	0.00	0.00	0.29
8303	SNN	0.00	0.00	-0.05	0.22
8309	ACOX2	0.00	-0.13	-0.15	0.14
8310	ACOX3	0.00	-0.13	-0.10	0.36
8312	AXIN1	0.00	0.00	-0.05	0.22

8315	BRAP	0.00	0.00	0.00	NaN
8317	CDC7L1	0.00	0.00	0.05	0.38
8318	CDC45L	-0.09	0.00	0.00	0.32
8321	FZD1	0.09	0.00	0.00	0.33
8322	FZD4	0.00	0.00	0.00	NaN
8323	FZD6	0.18	0.00	0.00	0.08
8324	FZD7	0.00	0.00	0.00	NaN
8326	FZD9	-0.09	0.00	0.00	-0.03
8328	GFI1B	0.00	0.00	0.00	NaN
8329	H2AFC	0.00	0.00	0.00	NaN
8330	H2AFD	0.00	0.00	0.00	NaN
8337	H2AFO	0.00	0.00	0.05	-0.05
8339	H2BFA	0.00	0.00	0.00	0.19
8340	H2BFC	0.00	0.00	0.00	NaN
8341	H2BFD	0.00	0.00	0.00	NaN
8342	H2BFE	0.00	0.00	0.00	NaN
8344	H2BFH	0.00	0.00	-0.05	-0.03
8345	H2BFJ	0.00	0.00	0.00	NaN
8347	H2BFL	0.00	0.00	-0.05	0.11
8348	H2BFN	0.00	0.00	0.00	NaN
8349	H2BFQ	0.00	0.00	0.10	-0.19
8350	H3FA	0.00	0.00	0.38	0.30
8369	H4FL	0.00	0.00	0.00	NaN
8379	MAD1L1	0.00	0.13	0.05	0.32
8382	NME5	0.00	0.00	0.00	NaN
8383	OR1A1	0.00	0.00	0.00	NaN
8385	OR1D4	0.00	0.00	0.00	NaN
8387	OR1E1	0.00	0.00	0.00	NaN
8388	OR1E2	0.00	0.00	0.00	NaN
8390	OR1G1	0.00	0.00	0.00	NaN
8392	OR3A3	0.00	0.00	0.00	NaN
8394	PIP5K1A	0.00	0.00	0.05	0.11
8395	PIP5K1B	0.00	0.00	-0.05	0.09
8396	PIP5K2B	0.00	0.38	0.00	0.76
8398	PLA2G6	-0.09	0.00	0.05	0.46
8399	PLA2G10	0.00	0.00	0.00	NaN
8402	SLC25A11	0.00	0.00	0.00	NaN
8403	SOX14	0.00	0.00	0.00	NaN
8404	SPARCL1	0.00	0.00	0.00	NaN
8405	SPOP	0.00	0.25	0.00	0.71
8408	ULK1	0.00	0.13	0.00	-0.05
8409	UXT	0.00	0.00	0.00	NaN
8411	EEA1	0.00	0.00	0.00	NaN
8412	BCAR3	0.09	0.00	0.00	0.38
8416	ANXA9	0.00	0.00	0.05	-0.14
8417	STX7	0.00	0.00	0.00	NaN
8418	CMAH	0.00	0.00	0.05	-0.06

8419	BFSP2	0.00	0.00	0.00	NaN
8424	BBOX1	0.00	0.00	0.00	NaN
8425	LTBP4	0.00	0.00	0.00	NaN
8427	ZNF282	0.00	0.00	-0.05	0.21
8428	STK24	-0.09	-0.13	-0.05	0.21
8431	NR0B2	-0.09	0.00	0.00	0.21
8433	UTF1	0.00	0.00	-0.10	-0.09
8434	RECK	-0.09	0.00	0.10	-0.08
8435	SOAT2	0.00	0.00	0.00	NaN
8436	SDPR	0.00	0.00	0.00	NaN
8437	RASAL1	0.00	0.00	0.00	NaN
8439	NSMAF	0.00	0.00	0.00	NaN
8440	NCK2	0.00	0.00	0.00	NaN
8443	GNPAT	0.09	0.00	0.00	0.38
8444	DYRK3	0.00	0.13	0.00	0.18
8445	DYRK2	0.09	0.00	-0.05	0.13
8446	DUSP11	0.00	0.00	0.00	NaN
8448	DOC2A	0.00	0.00	0.00	NaN
8449	DDX16	0.00	0.00	0.00	NaN
8451	CUL4A	-0.09	-0.13	-0.05	0.39
8452	CUL3	0.00	0.00	0.00	NaN
8453	CUL2	-0.09	0.00	0.05	0.63
8454	CUL1	0.00	0.00	-0.05	0.28
8455	ATRN	0.00	0.00	0.00	NaN
8456	WHN	0.00	0.38	0.00	-0.18
8459	TPST2	0.00	0.13	0.00	-0.03
8460	TPST1	-0.09	0.00	0.00	0.04
8462	TIEG2	0.00	0.00	-0.05	0.28
8464	SUPT3H	0.00	0.00	0.00	NaN
8467	SMARCA5	0.00	0.00	0.00	NaN
8468	FKBP6	-0.09	0.00	0.00	0.03
8470	ARGBP2	0.00	0.00	-0.05	-0.04
8476	PK428	0.09	0.00	0.00	0.02
8477	GPR65	0.00	0.13	0.00	0.12
8479	HIRIP3	0.00	0.00	0.00	NaN
8480	RAE1	0.18	0.50	0.00	0.67
8482	SEMA7A	0.00	0.00	0.00	NaN
8483	CILP	0.00	0.00	0.00	NaN
8484	GALR3	-0.09	0.00	0.05	0.02
8487	SIP1	0.00	0.00	0.00	NaN
8490	RGS5	0.00	0.00	0.00	NaN
8491	MAP4K3	0.00	0.00	0.00	NaN
8492	PRSS12	0.00	0.00	0.00	NaN
8493	PPM1D	0.18	0.13	0.00	0.81
8495	PPFIBP2	0.00	0.00	-0.05	0.16
8496	PPFIBP1	0.00	0.00	0.00	NaN
8498	RANBP3	0.00	0.00	0.00	NaN

8499	PPFIA2	0.00	0.13	0.00	0.09
8500	PPFIA1	0.55	0.25	0.00	0.76
8501	POV1	0.00	0.00	0.00	NaN
8502	PKP4	0.00	0.00	-0.05	0.10
8503	PIK3R3	0.00	0.00	0.00	NaN
8504	PEX3	0.00	0.00	0.00	NaN
8505	PARG	0.00	0.00	0.00	NaN
8506	CNTNAP1	0.00	-0.13	0.00	-0.01
8507	ENC1	0.33	0.00	0.33	0.11
8508	NIPSNAP1	-0.09	0.00	0.00	0.07
8509	NDST2	0.09	0.00	0.00	0.38
8511	MMP23A	0.00	0.00	-0.05	0.01
8513	LIPF	0.09	0.00	0.00	-0.04
8514	KCNAB2	0.00	0.00	0.00	NaN
8515	ITGA10	0.00	0.00	0.05	-0.06
8516	ITGA8	0.00	0.00	0.05	-0.22
8518	IKBKAP	0.00	0.00	0.00	NaN
8519	IFITM1	-0.27	-0.13	-0.15	0.37
8520	HAT1	0.00	0.00	0.00	NaN
8521	GCMA	0.00	0.00	0.00	NaN
8522	GAS7	0.00	0.00	0.00	NaN
8525	DGKZ	0.00	0.00	0.00	NaN
8526	DGKE	0.09	-0.13	0.00	0.13
8527	DGKD	0.00	0.00	0.00	NaN
8528	DDO	0.00	0.00	0.05	-0.06
8529	CYP4F2	0.00	0.00	0.00	NaN
8530	CST7	0.00	0.00	0.00	NaN
8531	CSDA	-0.09	0.00	0.00	0.17
8532	CPZ	0.00	-0.13	-0.10	0.00
8533	COPS3	0.00	0.00	0.00	NaN
8534	CHST1	0.00	0.00	0.00	NaN
8535	CBX4	0.00	0.25	0.00	0.55
8536	CAMK1	0.00	0.00	0.00	NaN
8537	BCAS1	0.18	0.50	0.00	0.56
8538	BARX2	-0.09	0.00	-0.10	0.07
8539	API5	0.00	0.00	0.00	NaN
8540	AGPS	0.00	0.00	0.00	NaN
8541	PPFIA3	0.09	0.00	0.00	0.23
8542	APOL1	0.00	0.00	0.00	NaN
8545	CGGBP1	-0.09	0.00	0.05	0.57
8546	AP3B1	0.00	0.00	0.00	NaN
8547	FCN3	-0.09	0.00	0.00	-0.04
8548	BLZF1	0.00	0.00	-0.05	0.19
8549	GPR49	0.09	0.00	0.00	-0.01
8550	MAPKAPK5	0.00	0.00	0.00	NaN
8553	BHLHB2	0.00	0.00	0.33	0.13
8554	PIAS1	0.00	0.00	0.00	NaN

8555	CDC14B	0.09	0.00	-0.05	0.10
8556	CDC14A	0.00	0.00	0.00	NaN
8557	TCAP	0.00	1.00	0.00	0.46
8558	CDK10	-0.09	-0.13	-0.05	0.54
8559	PRP18	0.00	0.00	0.00	NaN
8560	DEGS	0.09	0.00	0.00	0.25
8562	DENR	0.00	-0.13	0.00	0.29
8563	C22orf19	-0.09	0.00	0.00	0.15
8564	KMO	0.00	0.00	0.00	NaN
8565	YARS	0.00	0.00	0.00	NaN
8566	PDXK	0.00	0.00	0.00	NaN
8568	D21S2056E	0.00	0.00	0.00	NaN
8569	MKNK1	0.00	0.00	0.00	NaN
8570	KHSRP	0.00	0.00	0.00	NaN
8572	RIL	0.00	-0.13	0.00	0.12
8574	AKR7A2	-0.09	0.00	0.00	0.34
8575	PRKRA	0.00	0.00	0.50	0.53
8576	STK16	0.00	0.00	0.00	NaN
8577	TMEFF1	0.00	0.00	0.00	NaN
8578	SREC	0.00	-0.13	0.00	-0.03
8581	E48	0.18	0.13	0.05	0.28
8590	OR6A1	0.00	-0.13	0.00	0.00
8600	TNFSF11	0.00	0.00	-0.05	0.10
8601	RGS20	0.00	0.00	0.00	NaN
8602	RES4-25	0.00	-0.13	-0.15	0.22
8603	RES4-22	0.00	-0.13	-0.15	0.43
8604	SLC25A12	0.00	0.00	0.00	NaN
8605	PLA2G4C	0.09	0.00	0.00	-0.19
8607	RUVBL1	0.00	0.00	0.00	NaN
8608	RODH-4	0.00	0.00	0.00	NaN
8609	KLF7	0.00	0.00	0.00	NaN
8611	PPAP2A	0.00	0.00	-0.05	0.22
8612	PPAP2C	0.00	0.00	-0.05	-0.09
8613	PPAP2B	0.00	0.00	-0.05	0.24
8614	STC2	0.00	0.00	0.00	NaN
8615	P115	0.00	0.00	0.00	NaN
8618	CADPS	0.09	0.00	-0.05	0.24
8620	NPFF	0.00	0.00	0.00	NaN
8621	CDC2L5	0.00	0.00	0.00	NaN
8624	DSCR2	0.00	0.00	0.00	NaN
8625	RFXANK	0.00	0.00	0.00	NaN
8626	TP63	0.00	0.00	0.05	0.26
8629	JRK	0.18	0.13	0.05	0.31
8630	RODH	0.00	0.00	0.00	NaN
8631	SCAP1	0.00	0.00	0.00	-0.17
8633	UNC5C	0.00	0.00	0.00	NaN
8634	RTCD1	0.00	0.00	0.00	NaN

8635	RNASE6PL	0.00	0.00	0.00	NaN
8636	SSNA1	0.00	0.13	0.00	0.02
8638	OASL	0.00	-0.13	0.00	0.04
8639	AOC3	0.00	-0.13	0.00	-0.23
8642	PCDH16	0.00	-0.13	0.00	-0.05
8643	PTCH2	0.00	0.00	0.00	NaN
8644	AKR1C3	0.00	0.00	0.05	0.31
8645	KCNK5	0.00	0.00	0.00	NaN
8646	CHRD	0.09	0.00	0.00	0.07
8647	ABCB11	0.00	0.00	0.00	NaN
8648	NCOA1	0.09	0.00	0.00	0.56
8649	MAP2K1IP1	0.00	0.00	0.00	NaN
8650	NUMB	0.00	0.00	0.00	NaN
8651	SSI-1	0.00	0.00	0.00	NaN
8654	PDE5A	0.00	0.00	0.00	NaN
8655	PIN	0.00	0.00	0.00	-0.04
8658	TNKS	-0.09	-0.25	-0.10	0.14
8659	ALDH4A1	-0.09	0.00	0.00	0.15
8660	IRS2	-0.09	-0.13	0.00	0.21
8662	EIF3S9	0.00	0.13	0.05	0.57
8663	EIF3S8	0.00	0.00	0.00	NaN
8664	EIF3S7	-0.09	0.00	0.00	0.42
8665	EIF3S5	0.00	0.00	-0.05	0.18
8666	EIF3S4	0.00	0.00	0.05	0.45
8667	EIF3S3	0.00	0.38	0.10	0.78
8668	EIF3S2	0.00	0.00	0.00	NaN
8669	EIF3S1	0.00	0.00	0.00	NaN
8671	SLC4A4	0.00	0.00	0.00	NaN
8672	EIF4G3	-0.09	0.00	-0.05	0.44
8673	VAMP8	0.00	0.00	-0.05	0.08
8674	VAMP4	0.00	0.00	0.00	NaN
8676	STX11	0.00	0.00	0.00	NaN
8677	STX10	0.00	0.00	0.00	NaN
8678	BECN1	0.00	-0.13	0.00	0.41
8681	PLA2G4B	0.00	0.00	0.00	NaN
8682	PEA15	0.00	0.00	0.00	NaN
8683	SFRS9	0.00	-0.13	0.00	0.33
8685	MARCO	0.00	0.00	0.00	NaN
8687	KRTHA8	0.00	0.13	0.00	0.10
8688	KRTHA7	0.00	0.13	0.00	-0.02
8689	KRTHA6	0.00	0.13	0.00	0.02
8690	JRKL	0.00	0.00	0.05	0.05
8692	HYAL2	-0.09	0.00	-0.05	0.11
8693	GALNT4	0.09	0.00	0.00	0.36
8694	DGAT1	0.18	0.00	0.10	0.16
8697	CDC23	0.00	0.00	0.00	NaN
8698	EDG6	0.00	0.00	0.00	NaN

8702	B4GALT4	0.00	0.00	0.00	NaN
8703	B4GALT3	0.00	0.00	0.00	NaN
8704	B4GALT2	0.00	0.00	0.00	NaN
8705	B3GALT4	0.09	0.00	0.00	-0.02
8706	B3GALT3	0.00	0.00	0.00	NaN
8707	B3GALT2	0.09	0.00	0.00	0.18
8708	B3GALT1	0.00	0.00	0.00	NaN
8710	SERPINB7	-0.09	0.00	-0.15	-0.07
8711	TNK1	0.00	0.00	0.00	NaN
8714	ABCC3	0.00	0.25	0.00	0.57
8715	NOL4	0.00	0.00	-0.10	0.02
8717	TRADD	-0.09	0.00	0.00	0.21
8718	TNFRSF12	0.00	0.00	0.00	NaN
8720	MBTPS1	-0.09	-0.13	-0.05	0.32
8721	EDF1	0.00	0.00	0.00	NaN
8722	CTSF	0.00	0.00	0.05	-0.04
8723	SNX4	0.00	0.00	0.00	NaN
8724	SNX3	0.00	0.00	0.10	0.65
8725	RMP	0.00	0.13	0.15	0.69
8726	EED	0.00	0.00	0.00	NaN
8727	CTNNAL1	0.00	0.00	0.00	NaN
8728	ADAM19	0.00	0.00	0.00	NaN
8729	GBF1	0.00	-0.13	-0.05	0.12
8731	RNMT	0.00	0.00	0.00	NaN
8732	RNGTT	0.00	0.00	0.00	NaN
8733	GPAA1	0.18	0.00	0.10	0.36
8735	MYH13	0.00	0.00	0.00	NaN
8736	MYOM1	0.00	0.00	-0.05	0.05
8737	RIPK1	0.00	0.00	-0.10	0.16
8738	CRADD	0.00	0.13	0.00	0.21
8739	HRK	0.00	-0.13	0.00	0.12
8740	TNFSF14	0.00	0.00	0.00	NaN
8741	TNFSF13	0.00	0.00	0.00	NaN
8743	TNFSF10	0.09	0.00	0.00	-0.07
8744	TNFSF9	0.00	0.00	0.00	NaN
8745	ADAM23	0.00	0.00	0.00	NaN
8747	ADAM21	0.00	0.00	0.00	NaN
8748	ADAM20	0.00	0.00	0.00	NaN
8754	ADAM9	0.27	-0.13	0.00	0.38
8756	ADAM7	0.00	-0.13	-0.05	-0.06
8760	CDS2	0.00	0.00	0.00	NaN
8761	PABPC4	0.00	0.00	0.00	NaN
8763	CD164	0.00	0.00	0.05	0.22
8764	TNFRSF14	0.00	0.00	0.00	NaN
8766	RAB11A	0.09	0.00	0.00	0.27
8767	RIPK2	0.18	0.25	0.00	0.25
8771	TNFRSF6B	0.09	0.00	0.00	0.11

8772	FADD	0.55	0.25	0.00	0.77
8773	SNAP23	0.00	0.00	0.00	NaN
8774	NAPG	0.00	0.00	0.00	NaN
8775	NAPA	0.09	0.00	0.00	0.42
8777	MPDZ	0.00	0.00	0.05	0.63
8778	SIGLEC5	0.00	0.00	-0.05	0.16
8780	SUDD	0.00	0.00	-0.15	0.29
8785	MATN4	0.00	0.00	0.00	NaN
8786	RGS11	0.00	0.00	-0.05	-0.06
8787	RGS9	0.18	0.13	0.05	-0.24
8788	DLK1	0.00	0.00	0.00	NaN
8790	FPGT	0.00	0.00	0.00	NaN
8792	TNFRSF11A	-0.09	0.00	-0.15	0.18
8793	TNFRSF10D	-0.09	-0.25	-0.15	0.23
8794	TNFRSF10C	-0.09	-0.25	-0.15	0.25
8795	TNFRSF10B	-0.09	-0.25	-0.15	0.22
8796	SCEL	-0.09	0.13	-0.10	0.15
8798	DYRK4	-0.09	0.00	0.05	0.23
8799	PEX11B	0.00	0.00	0.05	0.04
8800	PEX11A	0.00	0.00	0.00	NaN
8801	SUCLG2	0.00	0.00	-0.05	0.58
8802	SUCLG1	0.00	0.00	0.00	NaN
8803	SUCLA2	0.00	0.00	0.00	NaN
8804	CREG	0.00	0.00	0.00	NaN
8805	TIF1	0.00	0.00	0.00	NaN
8807	IL18RAP	0.00	0.00	0.00	NaN
8808	IL1RL2	0.00	0.00	0.00	NaN
8809	IL18R1	0.00	0.00	0.00	NaN
8811	GALR2	0.00	0.13	0.00	-0.07
8813	DPM1	0.09	0.13	0.05	0.38
8814	CDKL1	0.00	0.00	0.00	NaN
8815	BCRP1	-0.09	0.00	0.05	0.53
8817	FGF18	0.00	0.00	0.00	NaN
8818	DPM2	-0.09	0.00	-0.05	0.24
8819	SAP30	0.00	0.00	-0.05	0.30
8820	HESX1	0.00	0.00	-0.10	0.37
8821	INPP4B	0.00	0.00	0.00	NaN
8822	FGF17	-0.09	-0.25	-0.10	0.11
8825	VELI1	0.00	0.13	0.00	0.38
8826	IQGAP1	0.00	0.00	0.00	NaN
8832	CD84	0.00	0.00	0.00	NaN
8833	GMPS	0.00	0.00	0.00	NaN
8834	PMI	0.00	-0.13	0.00	0.16
8835	STATI2	0.00	0.13	0.00	0.29
8836	GGH	0.00	0.00	0.00	NaN
8837	CFLAR	0.00	0.00	0.00	NaN
8838	WISP3	0.00	0.00	0.10	0.02

8839	WISP2	0.09	0.13	0.00	0.07
8840	WISP1	0.00	0.00	0.05	-0.13
8842	PROML1	0.00	0.00	-0.10	0.12
8844	KSR	0.00	0.13	0.00	0.29
8846	ABH	0.00	0.00	0.00	NaN
8850	PCAF	0.00	0.00	0.00	NaN
8851	CDK5R1	0.00	0.00	0.00	NaN
8853	DDEF2	0.09	0.00	-0.05	0.29
8854	ALDH1A2	0.00	0.00	0.00	NaN
8856	NR1I2	0.00	0.00	0.00	NaN
8857	FCGBP	0.00	0.00	0.00	NaN
8858	PROZ	-0.09	-0.13	-0.05	-0.08
8859	STK19	0.00	0.00	0.00	NaN
8861	LDB1	0.00	-0.13	-0.05	0.10
8863	PER3	0.00	0.00	0.00	NaN
8864	PER2	0.00	0.00	-0.05	0.17
8867	SYNJ1	0.00	0.00	0.00	NaN
8869	SIAT9	0.00	0.00	-0.05	0.12
8870	IER3	0.00	0.00	0.00	NaN
8871	SYNJ2	0.00	0.00	0.00	NaN
8872	D123	0.00	0.00	0.00	NaN
8874	ARHGEF7	-0.09	-0.13	0.00	0.34
8875	VNN2	0.00	0.00	0.00	NaN
8876	VNN1	0.00	0.00	0.00	NaN
8877	SPHK1	0.00	0.13	0.00	0.24
8878	SQSTM1	0.00	0.00	0.00	NaN
8879	SGPL1	0.09	0.00	0.00	0.42
8880	FUBP1	0.00	0.00	0.00	NaN
8881	CDC16	-0.09	-0.13	-0.05	0.35
8882	ZNF259	-0.09	0.00	0.00	0.30
8883	APPBP1	-0.09	-0.13	0.00	0.46
8884	SLC5A6	0.09	0.00	0.05	0.33
8886	DDX18	0.00	0.00	0.00	NaN
8887	TAX1BP1	0.00	0.13	0.00	-0.18
8888	MCM3AP	0.00	0.00	0.00	NaN
8891	EIF2B3	0.00	0.00	0.00	NaN
8892	EIF2B2	0.00	0.00	0.00	NaN
8893	EIF2B5	0.09	0.13	0.00	0.38
8894	EIF2S2	0.00	-0.13	0.00	0.45
8895	CPNE3	0.18	0.25	0.00	0.47
8896	G10	0.18	0.00	0.00	0.61
8897	MTMR3	0.00	0.00	0.00	NaN
8898	MTMR2	0.00	0.00	0.00	NaN
8899	PRP4	0.00	0.00	0.00	NaN
8900	CCNA1	0.00	0.00	0.00	NaN
8904	CPNE1	0.00	0.13	0.00	-0.02
8906	AP1G2	0.00	0.00	0.00	NaN

8910	SGCE	0.18	0.00	0.00	-0.21
8911	CACNA1I	-0.09	0.00	0.05	-0.10
8912	CACNA1H	0.00	0.13	-0.05	0.24
8913	CACNA1G	0.00	0.25	0.00	0.12
8914	TIMELESS	0.00	0.00	0.00	NaN
8915	BCL10	0.00	0.00	0.10	0.43
8916	HERC3	0.00	0.00	0.00	NaN
8924	HERC2	0.00	-0.13	0.00	0.20
8925	HERC1	0.00	0.00	0.00	NaN
8927	BSN	-0.09	0.00	-0.05	-0.07
8928	FOXH1	0.18	0.00	0.10	-0.03
8929	PMX2B	0.00	0.00	0.00	NaN
8930	MBD4	0.00	0.00	0.00	NaN
8932	MBD2	-0.09	0.00	-0.20	0.48
8933	CXX1	0.00	0.00	0.00	NaN
8934	RAB7L1	0.00	0.13	0.00	0.49
8935	SCAP2	0.00	0.13	0.00	-0.18
8936	WASF1	0.00	0.00	0.05	0.12
8938	BAIAP3	0.00	0.00	0.00	NaN
8939	FUBP3	0.00	0.00	0.00	NaN
8940	TOP3B	0.00	0.00	0.00	NaN
8941	CDK5R2	0.00	0.00	0.00	NaN
8942	KYNU	0.00	0.00	0.00	NaN
8943	AP3D1	0.00	0.00	0.00	NaN
8945	BTRC	0.00	-0.13	-0.05	0.06
8970	H2BFR	0.00	0.00	0.00	NaN
8971	H1FX	0.00	0.00	0.00	NaN
8972	MGAM	0.00	0.00	0.00	0.03
8973	CHRNA6	0.09	0.00	-0.05	0.10
8974	P4HA2	0.00	-0.13	0.00	0.28
8975	USP13	0.09	0.00	0.00	0.28
8976	WASL	0.00	0.13	0.00	0.40
8985	PLOD3	0.18	0.00	0.00	0.19
8986	RPS6KA4	0.00	0.00	0.00	NaN
8987	GENX-3414	0.00	0.00	0.00	NaN
8988	HSPB3	0.00	0.00	-0.05	0.01
8989	ANKTM1	0.00	0.25	0.00	-0.07
8991	SELENBP1	0.00	0.00	0.05	0.03
8992	ATP6V0E	0.00	0.00	0.00	NaN
8993	PGLYRP	0.09	0.00	0.00	-0.01
8994	LIMD1	-0.09	0.00	-0.05	0.18
8995	TNFSF18	0.00	0.00	0.00	NaN
8996	NOL3	0.50	0.50	0.50	0.42
8997	HAPIP	0.00	0.00	0.00	NaN
8999	CDKL2	0.00	0.00	0.00	NaN
9002	F2RL3	0.00	0.00	0.00	NaN
9013	TAF1C	-0.09	-0.13	-0.05	0.54

9014	TAF1B	0.00	0.00	-0.05	0.23
9015	TAF1A	0.09	0.00	0.00	-0.14
9019	MPZL1	0.00	0.00	0.00	NaN
9020	MAP3K14	0.00	-0.13	0.00	0.05
9021	SSI-3	0.00	0.13	0.00	0.01
9022	CLIC3	0.00	0.00	0.00	NaN
9023	CH25H	0.09	0.00	0.00	-0.03
9024	STK29	-0.27	-0.13	-0.05	-0.02
9025	RNF8	0.00	0.00	0.00	NaN
9026	HIP12	0.00	-0.13	0.00	0.06
9027	NAT8	0.00	0.00	0.00	NaN
9028	RHBDL	0.00	0.00	-0.05	0.12
9031	BAZ1B	0.09	0.00	0.00	-0.10
9032	TM4SF5	0.00	0.00	0.00	NaN
9033	PKD2L1	0.00	-0.13	-0.05	-0.13
9034	CCRL2	-0.09	0.00	-0.05	0.02
9037	SEMA5A	0.00	0.00	0.10	-0.09
9038	PNR	0.00	0.00	0.00	NaN
9039	UBE1C	0.00	0.00	-0.05	0.17
9040	UBE2M	0.00	0.13	0.10	0.38
9043	SPAG9	0.00	0.25	0.00	0.20
9044	BTAF1	0.00	0.00	0.00	NaN
9045	RPL14	0.00	0.00	-0.05	0.19
9046	DOK2	-0.09	-0.25	-0.10	0.03
9047	SH2D2A	0.00	0.00	0.00	0.23
9048	ARTN	0.00	0.00	0.00	NaN
9049	AIP	0.36	0.00	0.05	0.62
9050	PSTPIP2	-0.09	-0.13	-0.20	0.20
9051	PSTPIP1	0.00	0.00	0.00	NaN
9052	RAI3	-0.09	0.00	0.00	0.12
9053	MAP7	0.00	0.00	0.00	NaN
9054	NFS1	0.00	0.00	0.00	NaN
9055	PRC1	0.00	0.00	0.00	NaN
9056	SLC7A7	0.00	0.00	0.00	NaN
9057	SLC7A6	-0.09	-0.13	0.00	0.27
9058	SLC13A2	0.00	0.38	0.00	0.20
9060	PAPSS2	0.09	0.00	-0.15	0.26
9061	PAPSS1	0.00	0.00	0.00	NaN
9063	PIASX-BETA	-0.09	-0.13	-0.15	0.45
9064	MAP3K6	-0.09	0.00	0.00	0.09
9070	ASH2L	0.64	-0.13	-0.05	0.73
9071	CLDN10	-0.09	0.13	-0.05	0.01
9074	CLDN6	0.00	0.00	0.00	NaN
9076	CLDN1	0.00	0.00	0.05	0.56
9079	LDB2	0.00	0.00	-0.10	-0.14
9080	CLDN9	0.00	0.00	0.00	NaN
9088	PKMYT1	0.00	0.00	0.00	NaN

9091	PIGQ	0.00	0.00	-0.05	0.08
9092	SART1	-0.09	0.00	0.05	0.48
9093	DNAJA3	0.00	0.00	0.00	NaN
9094	UNC119	0.00	0.38	0.00	0.63
9095	TBX19	0.00	0.00	0.00	NaN
9097	USP14	0.00	0.00	0.00	NaN
9098	USP6	0.00	0.00	0.00	NaN
9099	USP2	-0.09	0.00	-0.10	0.16
9100	USP10	-0.09	0.00	-0.05	0.41
9101	USP8	0.18	0.00	0.00	0.28
9107	MTMR6	0.00	-0.13	-0.15	0.53
9108	MTMR7	0.00	-0.13	-0.05	0.01
9110	MTMR4	0.09	0.00	0.00	0.59
9112	MTA1	0.00	0.00	0.00	NaN
9113	LATS1	0.00	0.00	0.00	NaN
9114	ATP6V0D1	-0.09	0.00	0.05	0.68
9119	K6HF	0.00	0.00	0.00	NaN
9120	SLC16A6	0.18	0.13	0.00	0.19
9121	SLC16A5	0.00	0.13	0.00	0.67
9122	SLC16A4	0.09	0.00	0.00	-0.11
9123	SLC16A3	0.00	0.00	0.00	NaN
9124	PDLIM1	0.00	0.00	0.00	NaN
9125	RQCD1	0.00	0.00	0.00	NaN
9126	CSPG6	0.00	0.00	-0.05	0.22
9127	P2RXL1	0.00	0.13	0.00	0.24
9128	PRPF4	0.00	0.00	0.00	NaN
9129	HPRP3P	0.00	0.13	0.05	0.29
9132	KCNQ4	0.00	0.00	0.00	NaN
9133	CCNB2	0.09	0.00	0.05	0.25
9134	CCNE2	0.00	-0.13	0.00	0.41
9135	RAB5EP	0.00	0.00	0.00	NaN
9136	U3-55K	-0.09	0.00	-0.10	0.30
9138	ARHGEF1	0.00	0.00	-0.05	0.09
9139	CBFA2T2	-0.09	0.00	0.00	0.38
9140	APG12L	0.00	0.13	0.00	0.53
9141	PDCD5	0.00	0.00	0.05	0.04
9143	SYNGR3	0.00	0.00	0.00	NaN
9144	SYNGR2	0.00	0.13	0.00	0.30
9145	SYNGR1	-0.09	0.00	0.05	0.22
9146	HGS	0.00	0.00	0.00	NaN
9147	SDCCAG1	0.00	0.00	0.00	NaN
9148	NEURL	0.00	-0.13	-0.05	0.05
9149	DYRK1B	0.00	0.00	0.00	NaN
9150	CTDP1	-0.09	0.00	-0.25	0.51
9153	SLC28A2	0.00	0.00	0.00	NaN
9154	SLC28A1	0.00	0.00	-0.05	0.11
9156	EXO1	0.00	0.00	0.00	NaN

9158	FIBP	-0.09	0.00	0.05	0.58
9159	PCSK7	-0.09	0.00	0.00	-0.01
9162	DGKI	0.00	0.00	0.00	NaN
9166	EBAG9	0.18	0.00	0.05	0.34
9167	COX7A2L	0.00	0.00	0.00	NaN
9168	TMSB10	0.00	0.00	0.00	NaN
9169	SFRS2IP	0.00	0.00	0.00	NaN
9170	EDG4	0.00	0.00	0.00	NaN
9172	MYOM2	-0.09	-0.13	-0.15	-0.22
9173	IL1RL1	0.00	0.00	0.00	NaN
9175	MAP3K13	0.09	0.00	0.00	0.32
9177	HTR3B	0.00	0.00	0.00	NaN
9179	AP4M1	0.18	0.00	0.00	0.08
9180	OSMR	0.00	0.00	0.05	0.28
9181	ARHGEF2	0.00	0.13	0.00	0.14
9182	PAMCI	0.00	0.13	0.00	-0.08
9183	ZW10	0.00	0.00	0.00	NaN
9184	BUB3	0.09	0.00	-0.05	0.60
9187	SLC24A1	0.00	0.00	0.00	NaN
9188	DDX21	0.00	0.00	0.00	NaN
9191	DEDD	0.00	0.00	0.00	NaN
9194	SLC16A7	0.00	0.00	0.00	NaN
9196	KCNAB3	0.00	0.00	0.00	NaN
9197	ACATN	0.00	0.00	0.00	NaN
9200	PTPLA	0.00	0.00	0.05	-0.05
9201	DCAMKL1	0.00	0.00	0.00	NaN
9202	ZNF262	0.00	0.00	0.00	NaN
9204	ZNF258	0.00	0.00	0.00	NaN
9205	ZNF237	0.00	0.00	-0.05	0.34
9208	LRRFIP1	0.00	0.00	-0.05	0.36
9209	LRRFIP2	0.00	0.00	0.00	NaN
9211	LGI1	0.00	0.00	-0.05	-0.19
9212	STK12	0.00	0.00	0.00	NaN
9214	TOSO	0.00	0.13	0.00	0.32
9215	LARGE	0.00	0.00	0.00	NaN
9217	VAPB	0.18	0.50	0.00	0.63
9218	VAPA	0.00	0.00	0.00	NaN
9219	MTA1L1	0.00	0.00	0.00	NaN
9220	TIAF1	0.00	0.38	0.00	0.64
9221	NOLC1	0.00	-0.13	-0.05	0.17
9223	BAIAP1	-0.09	0.00	0.00	0.00
9227	LRAT	0.00	0.00	0.00	0.08
9228	DLGAP2	-0.09	-0.25	-0.15	0.01
9229	DLGAP1	0.00	0.00	0.00	NaN
9230	RAB11B	0.00	0.00	0.00	NaN
9231	DLG5	0.09	0.00	0.00	0.46
9232	PTTG1	0.00	0.00	0.00	NaN

9235	NK4	0.00	0.00	0.00	NaN
9236	CPR8	0.00	0.00	0.05	0.09
9238	CPR2	0.00	0.00	0.00	NaN
9240	PNMA1	0.00	0.00	0.00	NaN
9242	MSC	0.00	0.25	0.00	-0.04
9244	CRLF1	0.00	0.00	0.00	NaN
9245	GCNT3	0.00	0.00	0.05	0.09
9246	UBE2L6	0.00	0.00	0.00	NaN
9247	GCMB	0.00	0.00	0.05	-0.02
9249	SDR1	0.00	0.00	0.00	NaN
9252	RPS6KA5	0.00	0.13	0.00	0.55
9254	CACNA2D2	-0.09	0.00	-0.05	-0.04
9255	SCYE1	0.00	0.00	0.00	NaN
9256	PRAX-1	0.09	0.00	0.00	0.29
9258	MFHAS1	-0.09	-0.38	-0.15	0.25
9260	ENIGMA	0.00	0.00	0.00	NaN
9261	MAPKAPK2	0.00	0.13	0.00	0.46
9262	STK17B	0.00	0.00	0.00	NaN
9263	STK17A	0.00	0.00	0.00	NaN
9265	PSCD3	0.00	0.00	0.10	0.05
9266	PSCD2	0.09	0.00	0.00	0.26
9267	PSCD1	0.00	0.13	0.00	0.11
9270	ICAP-1A	0.09	0.00	-0.05	0.40
9271	PIWIL1	0.00	0.00	0.00	NaN
9274	BCL7C	0.00	0.00	0.00	NaN
9275	BCL7B	-0.09	0.00	0.00	0.25
9276	COPB2	0.00	0.00	0.00	NaN
9277	C6orf11	0.09	0.00	0.00	0.04
9278	ZNF297	0.09	0.00	0.00	-0.11
9283	ET(B)R-LP-2	0.00	0.00	0.00	NaN
9284	NPIP	0.00	0.00	0.00	NaN
9287	GPR58	0.00	0.00	0.00	NaN
9289	GPR56	-0.09	0.00	0.00	-0.07
9293	GPR52	0.00	0.00	0.05	-0.37
9294	EDG5	0.00	0.00	0.05	-0.15
9295	SFRS11	0.00	0.00	0.00	NaN
9296	ATP6V1F	0.00	0.25	0.00	0.54
9306	CIS4	0.00	0.00	0.50	0.17
9308	CD83	0.00	0.00	0.10	0.54
9310	ZFP93	0.09	0.00	0.00	0.10
9311	ACCN3	0.00	0.00	-0.05	0.20
9312	KCNB2	0.00	0.25	0.00	0.25
9313	MMP20	0.00	0.13	0.00	-0.01
9314	KLF4	0.00	0.00	0.00	NaN
9315	P311	0.00	0.13	0.00	0.01
9317	PTER	0.00	0.00	0.05	0.56
9318	TRIP15	0.00	0.00	0.00	NaN

9319	TRIP13	0.00	0.13	0.10	0.53
9320	TRIP12	0.00	0.00	0.00	NaN
9321	TRIP11	0.00	0.13	0.00	0.12
9322	TRIP10	0.00	0.00	0.00	NaN
9324	TRIP7	0.00	0.00	0.00	NaN
9325	TRIP4	0.00	0.00	-0.05	-0.07
9326	TRIP3	0.00	-0.13	0.00	0.33
9328	GTF3C5	0.00	0.00	0.00	NaN
9329	GTF3C4	0.00	0.00	0.00	NaN
9330	GTF3C3	0.00	0.00	0.00	NaN
9331	B4GALT6	0.00	0.00	-0.10	0.23
9332	CD163	-0.09	0.00	0.00	0.06
9333	TGM5	0.00	0.00	0.50	0.05
9334	B4GALT5	0.09	0.25	0.05	0.33
9337	CNOT8	0.00	0.00	0.00	NaN
9340	GLP2R	0.00	0.00	0.00	NaN
9341	VAMP3	0.00	0.00	0.00	NaN
9342	SNAP29	0.00	0.13	0.00	0.54
9344	TAO1	0.00	0.00	0.00	NaN
9348	NDST3	0.00	0.00	0.00	NaN
9350	CER1	0.00	0.00	0.05	-0.22
9351	SLC9A3R2	0.00	0.13	0.00	0.17
9352	TXNL	-0.09	0.00	-0.20	0.68
9353	SLIT2	0.00	0.00	-0.05	0.04
9354	UBE4A	0.00	0.00	0.00	NaN
9355	LHX2	0.00	0.00	0.00	NaN
9356	SLC22A6	0.00	0.00	0.00	NaN
9358	ITGBL1	-0.09	-0.13	0.05	0.08
9360	PPIG	0.00	0.00	0.00	NaN
9361	PRSS15	0.00	0.00	0.00	NaN
9362	CPNE6	0.00	0.00	0.00	NaN
9364	RAB28	0.00	0.00	-0.05	0.06
9365	KL	0.00	0.00	0.00	NaN
9368	SLC9A3R1	0.00	0.13	0.00	0.34
9369	NRXN3	0.00	0.00	0.00	NaN
9370	APM1	0.09	0.00	0.05	-0.09
9371	KIF3B	0.00	0.00	0.05	0.31
9372	MADHIP	0.00	0.00	0.00	NaN
9373	PLAA	0.00	-0.25	-0.05	0.70
9374	PPT2	0.00	0.00	0.00	NaN
9375	TM9SF2	-0.09	-0.13	-0.05	0.24
9376	SLC22A8	0.00	0.00	0.00	NaN
9377	COX5A	0.00	0.00	0.00	NaN
9378	NRXN1	0.00	0.00	0.00	NaN
9379	NRXN2	0.00	0.00	0.00	NaN
9380	GRHPR	-0.09	0.00	0.10	0.61
9381	OTOF	0.09	-0.13	0.05	-0.15

9388	LIPG	-0.09	-0.13	-0.20	0.12
9389	ORCTL4	0.00	0.00	0.00	NaN
9390	ORCTL3	0.00	0.25	0.00	0.22
9391	CIAO1	0.00	0.00	0.00	NaN
9392	TRAP-1	0.00	0.00	0.00	NaN
9394	HS6ST	0.00	0.00	-0.05	0.13
9397	NMT2	0.00	0.00	0.05	-0.05
9398	IGSF2	0.09	0.00	0.10	-0.01
9399	STOML1	0.00	0.00	0.00	NaN
9400	RECQL5	0.18	0.13	0.00	0.27
9401	RECQL4	0.18	0.00	0.10	0.33
9402	GRAP2	-0.09	0.00	0.05	0.05
9403	9/15/2010	0.00	0.00	0.05	0.46
9407	HAT	0.00	0.00	0.00	NaN
9409	PEX16	0.00	0.00	0.00	NaN
9410	HPRP8BP	0.00	0.00	0.00	NaN
9411	PARG1	0.09	0.00	0.00	0.31
9412	SURB7	0.00	0.00	0.00	NaN
9413	X123	0.00	0.00	0.00	NaN
9414	TJP2	0.00	0.00	0.05	-0.01
9415	FADS2	-0.09	0.00	0.00	0.18
9416	U5-100K	0.00	0.00	0.00	NaN
9419	CRIPT	0.00	0.00	0.00	NaN
9420	CYP7B1	0.00	0.00	0.00	NaN
9421	HAND1	0.00	0.00	0.00	NaN
9422	ZNF264	0.09	0.13	0.00	0.37
9423	NTN1	0.00	0.00	0.00	NaN
9425	CDYL	0.00	0.00	0.00	NaN
9427	ECEL1	0.00	0.00	0.00	NaN
9429	ABCG2	0.00	0.00	0.00	NaN
9435	CHST2	0.00	0.00	-0.05	0.14
9436	LY95	0.00	0.00	0.05	-0.03
9437	LY94	0.09	0.00	0.00	0.27
9439	CRSP3	0.00	0.00	0.00	NaN
9440	CRSP6	0.00	0.00	-0.10	0.13
9442	CRSP8	0.00	0.00	0.00	NaN
9443	CRSP9	0.00	0.00	0.00	NaN
9444	QKI	0.00	0.00	0.00	NaN
9445	ITM2B	0.00	0.00	0.00	NaN
9446	GSTTLp28	0.09	-0.13	-0.05	0.17
9447	AIM2	0.00	0.00	0.00	NaN
9448	MAP4K4	0.00	0.00	0.00	NaN
9450	MD-1	0.00	0.00	0.00	NaN
9451	EIF2AK3	0.00	0.00	-0.05	0.16
9453	GGPS1	0.09	0.00	0.00	0.43
9454	HOMER-3	0.00	0.00	0.00	NaN
9455	HOMER-2B	0.00	0.00	0.05	-0.20

9456	SYN47	0.00	0.00	-0.05	0.26
9457	ACT	0.00	0.00	0.00	NaN
9462	RASAL2	0.00	0.00	0.00	NaN
9463	PRKCABP	-0.09	0.00	0.05	-0.02
9464	HAND2	0.00	0.00	-0.05	-0.08
9465	AKAP7	0.00	0.00	0.00	NaN
9466	WSX1	0.00	0.00	0.00	NaN
9467	SH3BP5	0.00	0.00	0.05	0.14
9469	CHST3	0.09	0.00	0.00	0.10
9470	EIF4EL3	0.00	0.00	0.00	NaN
9472	AKAP6	0.00	0.00	0.00	NaN
9473	ICB-1	0.00	0.00	0.00	NaN
9474	APG5L	0.00	0.00	0.10	0.60
9475	ROCK2	0.00	0.00	0.00	NaN
9477	TRFP	0.00	0.00	0.05	-0.25
9479	MAPK8IP1	0.00	0.00	0.00	NaN
9480	ONECUT2	-0.09	0.00	-0.20	0.27
9482	STX8	0.00	0.00	0.00	NaN
9486	HNK-1ST	0.00	0.00	0.00	NaN
9487	PIGL	0.00	0.00	0.00	NaN
9488	PIGB	0.00	0.00	0.05	0.22
9489	PGS1	0.00	0.13	0.00	0.39
9491	PSMF1	0.00	0.00	0.00	NaN
9493	KNSL5	0.00	0.00	0.00	NaN
9495	AKAP5	0.00	0.00	0.00	NaN
9496	TBX4	0.27	0.13	0.00	-0.05
9497	SLC4A7	0.00	0.00	0.00	NaN
9498	SLC4A8	0.00	0.00	0.00	NaN
9499	TTID	0.00	-0.13	0.00	-0.09
9501	RPH3AL	0.00	-0.13	0.10	-0.09
9508	ADAMTS3	0.00	0.00	0.05	0.02
9509	ADAMTS2	0.00	0.00	0.00	NaN
9512	PMPCB	0.09	0.00	0.00	0.00
9513	FXR2	0.00	0.00	0.00	NaN
9514	CST	0.00	0.00	0.00	NaN
9515	STXBP-TOM	0.00	0.00	0.00	NaN
9516	PIG7	0.00	0.00	0.00	NaN
9517	SPTLC2	0.00	0.00	0.00	NaN
9518	PLAB	0.00	0.00	0.00	NaN
9519	TBPL1	0.00	0.00	0.00	NaN
9520	NPEPPS	0.00	-0.13	0.00	0.19
9521	EEF1E1	0.00	0.00	0.00	NaN
9522	SCAMP1	0.00	0.00	0.00	NaN
9524	GPSN2	0.00	0.00	0.00	NaN
9525	SKD1	-0.09	0.00	-0.15	0.33
9526	MPDU1	0.00	0.00	0.00	NaN
9527	GOSR1	0.00	0.38	0.00	0.08

9528	C1orf8	0.00	0.00	0.00	NaN
9529	BAG5	0.09	0.00	0.00	0.47
9530	BAG4	0.45	-0.13	-0.05	0.62
9531	BAG3	0.00	0.00	0.00	0.14
9532	BAG2	0.00	0.13	0.00	-0.13
9533	RPA40	0.00	0.00	0.00	NaN
9534	ZNF254	0.00	-0.25	0.00	0.23
9535	GMFG	0.00	0.00	0.00	NaN
9536	PTGES	0.00	0.00	0.00	NaN
9537	PIG11	0.00	0.00	0.00	NaN
9538	PIG8	-0.09	0.00	-0.10	0.58
9540	PIG3	0.09	0.00	-0.05	0.11
9542	NRG2	0.00	0.00	0.00	NaN
9545	RAB3D	0.00	0.00	0.00	NaN
9546	APBA3	0.00	0.00	0.00	NaN
9547	SCYB14	0.00	-0.13	0.00	-0.01
9550	ATP6V1G1	0.00	0.00	0.00	NaN
9551	ATP5J2	0.18	0.00	0.00	0.48
9552	SPAG7	0.00	0.00	0.00	NaN
9553	MRPL33	0.09	0.00	0.05	0.38
9554	SEC22L1	0.00	0.00	0.05	-0.12
9555	H2AFY	0.00	0.00	0.00	0.18
9556	C14orf2	0.00	0.00	0.00	NaN
9557	CHD1L	0.00	0.00	0.05	0.02
9559	VPS26	0.00	0.00	0.00	NaN
9562	MINPP1	0.09	0.00	-0.15	0.61
9563	H6PD	0.00	0.00	-0.05	0.11
9567	GTPBP1	-0.09	0.00	0.05	0.02
9568	GPR51	0.00	0.00	0.00	NaN
9569	GTF2IRD1	-0.09	0.00	0.00	0.13
9570	GOSR2	0.00	-0.13	0.00	0.20
9572	NR1D1	0.00	0.38	0.00	0.42
9573	GDF3	-0.09	0.00	0.00	-0.15
9575	CLOCK	0.00	0.00	0.00	NaN
9576	SPAG6	-0.09	0.00	0.05	-0.02
9577	BRE	0.09	0.00	0.05	0.11
9578	CDC42BPB	0.00	0.00	0.00	NaN
9580	SOX13	0.00	0.13	0.00	0.29
9581	KIAA0436	0.00	0.00	0.00	NaN
9582	DJ742C19.2	0.00	0.13	0.15	-0.04
9583	LYSAL1	-0.09	-0.25	-0.15	0.38
9584	RNPC2	0.00	0.00	0.00	NaN
9585	MPHOSPH1	0.09	0.00	0.00	0.64
9586	H_GS165L15.1	0.00	0.13	0.00	0.03
9587	KIAA0110	0.00	0.00	0.00	NaN
9588	AOP2	0.00	0.00	0.00	NaN
9589	WTAP	0.00	0.00	0.00	NaN

9590	AKAP12	0.00	0.00	0.00	NaN
9592	ETR101	0.00	0.00	0.00	NaN
9595	PSCDBP	0.00	0.00	-0.05	-0.03
9597	DAMS	0.00	-0.13	0.00	-0.11
9600	PITPNM	0.36	0.00	0.05	0.24
9601	ERP70	0.00	0.00	-0.05	-0.08
9603	NFE2L3	0.00	0.13	0.00	-0.12
9604	RNF14	0.00	0.00	0.00	NaN
9605	C16orf7	-0.09	-0.13	-0.05	0.19
9607	CART	0.00	0.00	0.00	NaN
9609	RAB36	0.00	0.00	0.00	NaN
9610	RIN1	0.00	0.00	0.05	0.43
9611	NCOR1	0.00	0.00	0.00	NaN
9612	NCOR2	-0.09	0.00	-0.05	0.61
9616	RNF7	0.00	0.00	0.00	NaN
9617	MTRF1	0.00	0.00	-0.05	0.06
9618	TRAF4	0.00	0.38	0.00	0.60
9619	ABCG1	0.00	0.00	0.00	NaN
9620	CELSR1	0.00	0.00	0.00	NaN
9623	TCL1B	0.00	0.00	0.00	NaN
9625	AATK	0.00	0.00	0.00	NaN
9627	SNCAIP	0.00	0.00	0.00	NaN
9628	RGS6	0.00	0.00	0.00	NaN
9629	CLCA3	0.00	0.00	0.10	0.33
9630	GNA14	0.00	0.00	-0.05	0.06
9631	NUP155	0.00	0.00	0.05	0.33
9632	SEC24C	0.09	0.00	0.00	0.30
9633	MTL5	0.45	0.00	0.00	0.33
9635	CLCA2	0.00	0.00	0.10	-0.13
9636	ISG15	0.00	0.00	0.00	NaN
9637	FEZ2	0.00	0.00	0.00	NaN
9638	FEZ1	-0.09	0.00	-0.10	-0.01
9639	ARHGEF10	-0.09	-0.25	-0.15	0.23
9640	KIAA0211	0.00	0.00	-0.05	0.26
9641	IKKE	0.00	0.13	0.00	0.39
9644	KIAA0418	0.00	-0.13	-0.05	0.31
9645	KIAA0750	0.00	0.00	0.00	NaN
9646	TSBP	0.00	0.00	0.00	NaN
9647	KIAA0015	-0.09	0.00	0.00	0.35
9648	KIAA0336	0.00	0.00	0.00	NaN
9649	RALGPS1A	0.09	0.00	-0.05	0.51
9650	KIAA0009	0.00	0.00	0.05	0.33
9651	KIAA0450	0.00	0.00	0.00	NaN
9652	KIAA0372	0.00	0.00	0.00	NaN
9653	HS2ST1	0.00	0.00	0.05	0.35
9654	KIAA0173	0.00	0.00	0.00	NaN
9655	SOCS5	0.00	0.00	0.00	NaN

9656	KIAA0170	0.00	0.00	0.00	NaN
9657	KIAA0036	0.00	0.00	0.05	0.68
9658	KIAA0222	-0.09	0.00	-0.25	0.04
9659	PDE4DIP	0.00	0.00	0.05	0.17
9662	KIAA0635	0.00	0.00	0.00	NaN
9663	LPIN2	-0.09	0.00	-0.05	0.36
9666	KIAA0675	0.00	0.00	0.00	NaN
9667	KIAA0138	0.00	0.00	0.00	NaN
9668	KIAA0798	0.00	0.00	-0.05	0.04
9669	IF2	0.00	0.00	0.00	NaN
9670	IMP13	0.00	0.00	0.00	NaN
9672	SDC3	0.00	0.00	0.00	NaN
9673	KIAA0446	0.00	0.13	0.00	-0.01
9674	KIAA0040	0.00	0.00	0.00	NaN
9675	KIAA0406	0.00	0.00	0.00	NaN
9677	KIAA0377	0.00	0.00	0.00	NaN
9678	KIAA0783	0.00	0.13	0.05	-0.20
9679	KIAA0140	0.00	0.00	-0.05	0.13
9681	KIAA0645	0.00	0.00	0.00	NaN
9682	KIAA0677	0.00	0.00	0.00	NaN
9683	N4BP1	-0.09	0.00	0.00	0.42
9684	KIAA0014	0.18	0.00	0.10	0.52
9685	KIAA0171	0.00	0.00	0.00	NaN
9686	KIAA0121	0.00	0.00	0.00	NaN
9687	KIAA0575	0.00	0.00	0.00	NaN
9688	KIAA0095	-0.09	0.00	0.00	0.39
9689	BZAP45	0.00	0.00	0.00	NaN
9690	KIAA0010	0.00	0.00	-0.05	0.04
9692	KIAA0391	0.00	0.00	0.00	NaN
9693	PDZ-GEF1	0.00	0.00	0.00	0.54
9694	KIAA0103	0.18	-0.13	0.00	0.58
9695	KIAA0212	0.00	0.00	0.05	0.12
9696	KIAA0445	0.00	0.00	0.00	NaN
9697	KIAA0057	0.00	0.00	0.00	NaN
9698	PUM1	0.00	0.00	0.00	NaN
9699	RAB3IP3	0.18	-0.25	0.00	0.08
9700	ESPL1	0.00	0.00	0.00	NaN
9701	KIAA0685	0.00	-0.25	-0.05	0.32
9702	KIAA0092	0.00	0.00	0.00	NaN
9703	KIAA0100	0.00	0.38	0.00	0.50
9704	DDX34	0.09	0.00	-0.05	0.15
9705	KIAA0535	0.00	0.00	0.00	NaN
9706	ULK2	0.00	-0.13	0.00	0.17
9708	PCDHGA8	0.00	0.00	0.00	NaN
9709	HERPUD1	-0.09	0.00	0.00	0.11
9710	KIAA0355	0.00	0.00	0.05	0.17
9711	KIAA0226	0.18	0.00	0.00	0.58

9712	RNTRE	0.00	0.00	0.00	NaN
9715	KIAA0773	0.00	0.00	-0.05	0.16
9716	KIAA0560	0.00	0.00	0.00	NaN
9717	KIAA0420	0.00	0.00	0.00	NaN
9718	ECE2	0.09	0.00	0.00	0.07
9719	KIAA0605	0.00	0.00	0.00	NaN
9720	KIAA0565	0.00	0.00	0.00	NaN
9721	KIAA0514	0.00	0.00	0.00	NaN
9722	CAPON	0.00	0.00	0.00	NaN
9723	SEMA3E	0.09	0.00	0.00	-0.11
9724	KIAA0266	0.00	0.00	0.00	NaN
9725	KIAA0792	0.09	0.00	0.00	0.03
9726	KIAA0296	0.00	0.00	0.00	NaN
9727	Rab11-FIP3	0.00	0.00	-0.05	0.10
9728	KIAA0256	0.00	0.00	0.00	NaN
9729	KIAA0408	0.00	0.00	0.05	0.15
9730	KIAA0800	-0.09	0.00	-0.10	0.30
9731	KIAA0562	0.00	0.00	0.00	NaN
9732	KIAA0716	0.09	0.00	0.00	-0.12
9733	SART3	0.00	-0.13	0.00	0.25
9734	HDAC9-PENDI	0.00	0.50	0.50	0.18
9735	KNTC1	0.00	-0.13	0.00	0.16
9736	KIAA0570	0.00	0.00	0.00	NaN
9738	KIAA0419	0.00	0.00	0.00	NaN
9739	KIAA0339	0.00	0.00	0.00	NaN
9741	LAPTM4A	0.09	0.00	-0.05	0.61
9742	KIAA0590	0.00	0.00	0.00	NaN
9743	KIAA0712	-0.09	0.00	-0.10	0.30
9744	CENTB1	0.00	0.00	0.00	NaN
9745	KIAA0390	0.00	0.13	0.05	-0.05
9746	CLSTN3	0.00	0.00	0.00	0.27
9747	KIAA0738	0.00	0.13	0.00	0.16
9749	KIAA0680	0.00	0.00	0.00	NaN
9750	C6orf32	0.00	0.00	0.05	-0.03
9751	SNPH	0.00	0.00	0.00	NaN
9752	PCDHA9	0.00	-0.25	0.00	0.09
9753	KIAA0426	0.00	0.00	0.00	NaN
9755	KIAA0775	0.00	-0.13	0.00	-0.19
9759	HDAC4	0.00	-0.13	-0.05	0.21
9760	TOX	0.00	0.00	0.00	NaN
9761	KIAA0152	0.00	-0.13	0.00	0.21
9762	KIAA0552	0.00	0.00	0.00	NaN
9764	KIAA0513	-0.09	0.00	-0.05	0.17
9765	ENDOFIN	0.00	0.00	-0.05	0.24
9766	KIAA0247	0.00	0.00	0.00	NaN
9768	KIAA0101	0.00	0.00	0.00	-0.18
9770	RASSF2	0.00	0.00	0.00	NaN

9771	GFR	0.00	0.13	0.05	0.13
9772	KIAA0195	0.18	0.13	0.00	0.39
9774	BTF	0.00	0.00	0.00	NaN
9775	KIAA0111	0.00	0.25	0.05	0.19
9776	KIAA0652	0.00	0.00	0.00	NaN
9777	KIAA0255	0.00	0.00	0.05	0.29
9778	KIAA0232	0.00	-0.13	-0.10	0.33
9779	KIAA0210	0.00	0.00	0.00	NaN
9780	KIAA0233	-0.09	-0.13	-0.05	0.35
9781	KIAA0161	0.00	0.00	-0.05	-0.02
9782	MATR3	0.00	0.00	0.00	NaN
9783	KIAA0237	0.00	0.00	0.00	NaN
9784	SNX17	0.09	0.00	0.05	0.50
9785	DDX38	0.00	0.00	0.05	0.29
9786	KIAA0586	0.00	0.00	0.05	0.45
9787	KIAA0008	0.00	0.00	0.05	0.32
9788	KIAA0429	0.18	0.38	-0.05	0.22
9789	KIAA0102	0.09	0.00	0.00	0.66
9790	KIAA0187	0.00	0.00	0.00	NaN
9791	PTDSS1	0.00	-0.13	0.05	0.45
9792	TRIP-Br2	0.00	0.00	0.00	NaN
9793	KIAA0097	0.00	0.00	0.00	NaN
9794	MAML1	0.00	0.00	0.05	0.22
9796	PHYHIP	-0.09	-0.25	-0.10	0.11
9797	KIAA0218	0.00	0.00	0.00	NaN
9798	KIAA0174	0.00	0.00	0.05	0.38
9801	MRPL19	0.00	0.00	0.00	NaN
9802	DAZAP2	0.00	0.00	0.00	NaN
9804	TOMM20-PEN	0.09	0.00	0.00	0.49
9805	KIAA0193	0.00	0.13	0.00	0.10
9806	KIAA0275	0.09	0.00	0.00	-0.04
9807	IHPK1	-0.09	0.00	-0.05	0.21
9808	KIAA0087	0.00	0.13	0.00	0.31
9810	RNF40	0.00	0.00	0.00	NaN
9811	KIAA0427	-0.09	-0.13	-0.20	0.40
9812	KIAA0141	0.00	0.00	0.00	NaN
9813	KIAA0494	0.00	0.00	0.00	NaN
9814	KIAA0542	0.00	0.00	0.00	NaN
9815	GIT2	0.00	0.00	0.00	NaN
9816	KIAA0133	0.09	0.00	0.00	0.03
9817	KEAP1	0.00	0.00	0.05	0.34
9818	KIAA0410	0.00	-0.13	-0.15	0.16
9819	KIAA0669	0.00	0.00	0.05	0.44
9820	KIAA0076	0.00	0.00	0.00	NaN
9821	RB1CC1	0.00	0.00	0.00	NaN
9824	ARHGAP11A	0.00	0.00	0.00	NaN
9825	SPATA2	0.27	0.25	0.10	0.70

9826	ARHGEF11	0.00	0.00	0.00	NaN
9827	KIAA0258	-0.09	0.00	0.10	0.74
9828	KIAA0337	0.00	0.13	0.00	0.30
9829	DNAJC6	0.00	0.00	-0.05	0.23
9830	TRIM14	0.00	0.00	0.00	NaN
9831	KIAA0628	0.18	0.00	0.10	0.68
9832	KIAA0555	0.00	0.00	0.00	NaN
9833	MELK	-0.09	0.00	0.10	0.52
9834	KIAA0125	0.91	0.63	0.65	0.03
9836	KIAA0547	0.00	0.00	0.00	NaN
9837	KIAA0186	0.00	0.00	-0.05	0.24
9839	ZFHX1B	0.00	0.00	0.00	NaN
9840	KIAA0748	0.00	0.00	0.00	NaN
9841	KIAA0441	0.00	0.00	0.05	0.53
9842	KIAA0356	0.00	-0.13	0.00	0.31
9844	ELMO1	0.00	0.00	0.00	NaN
9846	GAB2	0.27	0.00	0.00	0.23
9847	KIAA0528	0.00	0.00	0.00	NaN
9848	KIAA0626	0.00	0.00	-0.05	0.16
9849	KIAA0335	0.00	0.00	0.00	-0.04
9851	KIAA0753	0.00	0.00	0.00	NaN
9852	KIAA0766	0.00	0.00	0.00	NaN
9853	KIAA0375	-0.09	0.00	0.10	0.44
9854	KIAA0285	-0.09	0.00	-0.05	0.13
9855	KIAA0793	0.00	0.00	-0.05	0.21
9856	KIAA0319	0.00	0.00	0.05	-0.07
9857	CAP350	0.00	0.00	0.00	NaN
9858	KIAA0649	0.00	0.00	0.00	NaN
9859	KIAA0470	0.09	0.00	0.00	0.24
9860	KIAA0806	0.00	0.00	0.05	0.44
9861	KIAA0107	0.09	0.00	-0.05	0.70
9863	AIP1	-0.09	0.00	0.00	0.04
9865	KIAA0644	0.00	0.13	0.00	0.23
9866	KIAA0298	0.00	0.00	-0.05	-0.09
9867	KIAA0438	0.00	0.13	0.00	-0.03
9868	TOMM70A	0.00	0.00	0.00	NaN
9869	SETDB1	0.00	0.00	0.05	0.59
9870	KIAA0317	0.00	0.00	0.00	NaN
9871	SEC24D	0.00	0.00	0.00	NaN
9873	KIAA0769	0.00	0.00	0.00	NaN
9874	TLK1	0.00	0.00	0.00	NaN
9875	C21orf108	0.00	0.00	0.00	NaN
9878	KIAA0737	0.00	0.00	0.00	NaN
9879	KIAA0801	0.00	-0.13	0.00	0.15
9880	KIAA0352	0.00	0.00	0.00	NaN
9881	KIAA0342	0.00	0.00	0.00	NaN
9882	KIAA0603	-0.09	0.13	-0.15	0.58

9883	KIAA0618	-0.09	0.00	0.00	0.35
9884	KIAA0563	0.09	0.00	0.00	0.15
9885	OSBPL2	0.09	0.13	0.00	0.65
9886	KIAA0740	0.00	0.00	0.00	NaN
9887	C1orf16	0.00	0.00	0.00	NaN
9889	KIAA0637	0.00	-0.25	0.00	0.33
9890	KIAA0455	0.00	0.00	0.00	NaN
9891	KIAA0537	0.00	0.00	0.00	NaN
9892	SNAP91	0.00	0.00	0.00	NaN
9894	KIAA0683	0.00	0.00	0.00	NaN
9895	KIAA0329	0.00	0.00	0.00	NaN
9896	SAC3	0.00	0.00	0.05	0.27
9897	KIAA0196	0.18	0.38	0.00	0.66
9898	KIAA0144	0.09	0.13	0.00	0.41
9899	SV2B	0.00	0.00	0.00	NaN
9900	SV2	0.00	0.00	0.05	-0.06
9901	SRGAP3	0.50	0.50	0.00	0.27
9902	ENDO180	0.27	0.13	0.00	-0.10
9903	KIAA0469	0.00	0.00	0.00	NaN
9904	KIAA0682	0.00	0.00	0.00	NaN
9905	KIAA0397	0.00	-0.13	0.00	0.05
9906	KIAA0447	0.00	0.00	-0.05	0.26
9907	KIAA0415	0.00	0.00	0.10	0.32
9908	G3BP2	0.00	0.00	0.00	NaN
9909	KIAA0476	0.09	0.13	0.00	0.30
9910	KIAA0471	0.00	0.00	0.05	0.31
9911	KIAA0481	0.00	0.13	0.00	-0.13
9912	KIAA0672	0.00	0.00	0.00	NaN
9913	STAF65(gamm	0.09	0.00	0.05	0.62
9914	KIAA0703	-0.09	-0.13	0.00	-0.02
9915	ARNT2	0.00	0.00	0.00	NaN
9917	KIAA0475	0.00	0.00	0.00	NaN
9919	KIAA0310	0.00	0.00	0.00	NaN
9920	KIAA0711	-0.09	-0.13	-0.15	0.16
9921	RNF10	0.00	-0.13	0.00	0.15
9922	KIAA0763	0.00	0.00	0.00	NaN
9923	KIAA0478	-0.09	0.00	0.00	0.04
9924	KIAA0710	0.00	0.00	0.00	NaN
9925	KIAA0354	-0.09	0.00	0.05	0.64
9926	KIAA0205	0.00	0.00	0.00	NaN
9927	MFN2	0.00	0.00	0.00	NaN
9928	KIAA0042	0.00	0.00	0.00	NaN
9929	KIAA0063	-0.09	0.00	0.05	0.37
9931	HELZ	0.18	0.13	0.00	0.45
9933	KIAA0020	0.00	-0.13	-0.10	0.65
9934	GPR105	0.00	0.00	0.00	NaN
9935	MAFB	0.00	-0.13	0.00	-0.01

9936	KIAA0022	0.00	0.00	-0.10	0.17
9937	DCLRE1A	0.09	0.00	-0.05	0.39
9938	KIAA0053	0.00	0.00	0.00	NaN
9939	RBM8A	0.00	0.00	0.05	0.47
9940	DLEC1	0.00	0.25	0.00	0.71
9941	ENDOGL1	0.00	0.00	0.00	NaN
9942	XYLB	0.00	0.00	0.00	NaN
9943	OSR1	0.50	0.50	0.50	0.33
9945	GFPT2	0.00	0.13	0.00	-0.05
9946	CRYZL1	0.00	0.00	0.00	NaN
9948	WDR1	0.00	-0.13	-0.10	0.22
9950	GOLGA5	0.00	0.00	0.00	NaN
9953	HS3ST3B1	0.00	0.00	0.00	NaN
9955	HS3ST3A1	0.00	0.00	0.00	NaN
9956	HS3ST2	0.00	0.00	0.00	NaN
9957	HS3ST1	0.00	0.00	-0.05	-0.05
9958	USP15	0.00	0.00	0.00	NaN
9961	MVP	0.00	0.00	0.00	NaN
9962	SLC23A1	0.00	0.00	0.00	NaN
9966	TNFSF15	0.00	-0.13	0.00	-0.03
9967	TRAP150	0.00	0.00	0.00	NaN
9969	TRAP240	0.27	0.13	0.00	0.62
9970	NR1I3	0.00	0.00	0.00	NaN
9971	NR1H4	0.00	0.00	0.00	NaN
9972	NUP153	0.00	0.00	0.00	NaN
9973	CCS	0.00	0.00	0.05	0.23
9976	CLECSF2	-0.09	0.00	0.00	-0.16
9978	RBX1	0.00	0.00	0.05	0.37
9980	C21orf5	0.00	0.00	0.00	NaN
9982	HBP17	0.00	0.00	-0.10	0.16
9984	P84	0.00	0.00	0.00	NaN
9985	REC8	0.00	0.00	0.00	NaN
9986	RCE1	0.09	0.00	0.05	0.46
9987	HNRPDL	0.00	0.00	0.00	NaN
9988	DMTF1	0.00	0.00	0.00	NaN
9989	PPP4R1	0.00	0.00	0.00	NaN
9990	SLC12A6	0.00	0.00	0.00	NaN
9991	ROD1	0.00	0.00	0.00	NaN
9992	KCNE2	0.00	0.00	0.00	NaN
9994	CASP8AP2	0.00	0.00	0.00	NaN
9997	SCO2	0.00	-0.25	0.00	0.44
10000	AKT3	0.09	0.00	0.00	-0.22
10001	MED6	0.00	0.00	0.00	NaN
10002	NR2E3	0.00	0.00	0.00	NaN
10003	NAALAD2	0.00	0.00	0.00	NaN
10004	NAALADASEL	0.00	0.00	0.00	NaN
10005	PTE1	0.00	0.00	0.00	NaN

10006	SSH3BP1	-0.09	0.00	0.00	0.37
10007	GNPI	0.00	0.00	0.00	NaN
10010	TANK	0.00	0.00	-0.05	0.20
10014	HDAC5	0.00	-0.13	0.00	-0.07
10015	PDCD6IP	0.00	0.00	0.00	NaN
10016	PDCD6	0.00	0.13	0.10	0.25
10017	BCL2L10	0.18	0.00	0.00	-0.09
10018	BCL2L11	0.00	0.00	0.00	NaN
10019	LNK	0.00	0.00	0.00	NaN
10020	GNE	-0.09	0.00	0.10	0.42
10021	HCN4	0.00	0.00	0.00	NaN
10022	INSL5	0.00	0.00	0.00	NaN
10023	FRAT1	0.00	0.00	-0.05	0.14
10024	TROAP	0.00	0.00	0.00	NaN
10025	TRAP95	0.00	0.00	-0.05	0.52
10026	PIGK	0.00	0.00	0.00	NaN
10036	CHAF1A	0.18	0.00	0.00	0.10
10038	ADPRTL2	0.00	0.00	0.00	NaN
10039	ADPRTL3	-0.09	0.00	-0.10	0.21
10040	TOM1L1	0.00	0.00	0.00	0.43
10042	HMG2L1	0.00	0.00	0.00	NaN
10043	TOM1	0.00	0.00	0.00	NaN
10045	SH2D3A	0.00	0.00	0.00	NaN
10047	CST8	0.00	0.00	0.00	NaN
10048	RANBP9	0.00	0.00	0.10	0.50
10049	DNAJB6	0.00	0.00	-0.05	0.24
10050	SLC17A4	0.00	0.00	0.05	0.09
10051	SMC4L1	0.00	0.00	0.00	NaN
10052	GJA7	0.00	-0.13	0.00	0.11
10053	AP1M2	0.00	0.13	0.05	0.16
10054	UBA2	0.00	0.00	0.05	0.23
10057	ABCC5	0.09	0.00	0.00	0.21
10058	ABCB6	0.00	0.00	0.00	NaN
10059	DNM1L	0.00	0.00	0.00	NaN
10060	ABCC9	0.00	0.00	0.00	NaN
10061	ABCF2	0.00	0.00	-0.05	0.21
10062	NR1H3	0.00	0.00	0.00	NaN
10063	COX17	0.00	0.00	0.00	NaN
10066	SCAMP2	0.00	0.00	0.00	NaN
10067	SCAMP3	0.09	0.13	0.00	0.53
10068	IL18BP	0.00	-0.13	0.00	-0.03
10069	C21orf6	0.00	0.00	0.00	NaN
10072	DPP3	0.00	0.00	0.05	0.23
10073	RNUT1	0.00	0.00	0.00	NaN
10076	PTPRU	0.00	0.00	0.00	NaN
10077	PHEMX	0.00	-0.13	0.00	-0.10
10078	TSSC4	0.00	-0.13	0.00	0.18

10079	ATP9A	0.09	0.13	0.00	0.35
10083	PDZ-73/NY-CO	0.00	0.00	0.00	NaN
10086	HHLA1	0.00	-0.25	0.10	-0.20
10087	COL4A3BP	0.00	0.00	0.00	NaN
10089	KCNK7	-0.09	0.00	0.05	-0.19
10090	UST	0.00	0.00	0.00	NaN
10092	ARPC5	0.00	0.00	0.00	NaN
10093	ARPC4	0.00	0.00	0.00	NaN
10094	ARPC3	0.00	0.00	0.00	NaN
10095	ARPC1B	0.18	0.00	0.00	0.58
10096	ACTR3	0.00	0.00	0.00	NaN
10097	ACTR2	0.00	0.00	0.00	NaN
10098	TSPAN-5	0.00	0.00	0.00	NaN
10099	TSPAN-3	0.00	0.00	0.00	NaN
10100	TSPAN-2	0.18	0.00	0.10	0.08
10101	NUBP2	0.00	0.00	0.00	NaN
10102	TSFM	0.00	0.00	0.00	NaN
10103	TSPAN-1	0.00	0.00	0.00	NaN
10105	PPIF	0.09	0.00	0.00	0.41
10106	OS4	0.00	0.00	0.00	NaN
10107	TRIM10	0.00	0.00	0.00	NaN
10109	ARPC2	0.00	0.00	0.00	NaN
10110	SGK2	0.00	-0.13	0.00	0.10
10111	RAD50	0.00	-0.13	0.00	0.14
10112	RAB6KIFL	0.00	0.00	0.00	NaN
10113	PREB	0.09	0.00	0.05	0.55
10114	HIPK3	0.00	0.00	0.05	0.58
10116	FEM1B	0.00	0.00	0.00	NaN
10120	ACTR1B	0.00	0.00	0.00	NaN
10121	ACTR1A	0.00	-0.13	-0.05	0.49
10123	ARL7	0.00	0.00	0.00	NaN
10125	RASGRP1	0.00	0.00	-0.05	0.12
10126	DNAL4	-0.09	0.00	0.05	0.13
10127	ZNF263	0.00	0.00	0.00	NaN
10128	LRPPRC	0.00	0.00	0.00	NaN
10129	13CDNA73	0.00	0.00	0.00	NaN
10130	P5	0.00	0.00	-0.05	0.37
10133	OPTN	0.00	0.00	0.00	NaN
10135	PBEF	0.09	0.00	0.00	0.04
10136	ELA3A	-0.09	0.00	-0.10	0.06
10137	RBM12	0.00	0.00	0.00	NaN
10138	YAF2	0.00	0.13	-0.05	0.43
10140	TOB1	0.00	0.25	0.00	0.41
10141	C4orf6	0.00	-0.13	-0.15	0.00
10142	AKAP9	0.18	0.00	0.00	0.54
10144	KIAA0914	0.00	0.00	0.00	NaN
10146	G3BP	0.00	0.00	0.00	NaN

10147	KIAA0365	0.00	0.00	0.00	NaN
10148	EBI3	0.00	0.00	0.00	NaN
10150	MBLL	-0.09	-0.13	-0.05	0.27
10151	HNRPA3	0.00	0.00	0.00	NaN
10152	ABI-2	0.00	0.00	0.00	NaN
10154	PLXNC1	0.00	0.00	0.00	NaN
10155	TRIM28	0.00	0.13	0.00	0.58
10156	CAPRI	0.18	0.00	0.00	0.04
10157	AASS	0.00	0.13	0.00	-0.01
10158	DD96	0.00	0.00	0.00	NaN
10160	FARP1	-0.09	-0.13	-0.10	0.28
10161	P2Y5	0.00	0.13	-0.05	0.24
10162	C3F	0.00	0.00	0.00	0.52
10164	CHST4	0.00	0.13	0.05	0.19
10165	SLC25A13	0.18	0.00	0.00	0.53
10166	SLC25A15	0.00	0.00	-0.05	0.16
10168	ZNF197	-0.09	0.00	-0.05	-0.10
10169	SERF2	0.00	0.00	0.00	NaN
10170	RDHL	0.00	0.00	0.00	NaN
10171	RNAC	-0.09	-0.13	-0.10	0.64
10174	SCAM-1	-0.09	-0.25	-0.15	0.27
10175	CNIL	0.00	0.00	0.05	0.28
10179	RBM7	-0.09	0.00	0.00	0.24
10180	RBM6	-0.09	0.00	0.00	0.59
10181	RBM5	-0.09	0.00	-0.05	0.41
10184	LHFPL2	0.00	0.00	0.00	NaN
10186	LHFP	0.00	0.00	0.00	NaN
10188	ACK1	0.18	0.00	0.00	0.55
10190	APACD	0.00	0.00	0.00	NaN
10193	SBB103	0.00	0.00	0.00	NaN
10195	NOT56L	0.09	0.00	0.00	0.27
10196	PRMT3	0.00	0.00	0.00	NaN
10197	PSME3	0.00	-0.13	0.00	0.24
10198	MPHOSPH9	0.00	0.00	0.00	NaN
10199	MPHOSPH10	0.00	0.00	0.00	NaN
10200	MPHOSPH6	0.00	0.50	0.00	0.25
10201	NM23-H6	-0.09	0.00	-0.05	0.32
10202	DHRS2	0.00	0.00	0.00	NaN
10203	CALCRL	0.00	0.00	0.00	NaN
10204	NTF2	-0.09	-0.13	0.00	0.48
10205	EVA1	0.00	0.00	0.00	NaN
10206	RFP2	0.00	0.13	0.00	0.46
10207	INADL	0.00	0.00	0.00	NaN
10208	D13S106E	0.00	0.00	0.05	0.21
10209	SUI1	0.00	0.13	0.00	0.24
10210	TP53BPL	-0.09	0.00	0.00	0.70
10211	FLOT1	0.00	0.00	0.00	NaN

10212	DDX39	0.00	0.00	0.00	NaN
10213	POH1	0.00	0.00	-0.05	0.44
10215	OLIG2	0.00	0.00	0.00	NaN
10216	PRG4	0.00	0.00	0.00	NaN
10217	HYA22	0.00	0.00	0.00	NaN
10218	CDT6	0.00	0.00	0.00	NaN
10219	KLRG1	0.50	0.00	0.50	-0.09
10220	GDF11	0.00	0.00	0.00	NaN
10221	C8FW	0.18	0.38	0.10	0.31
10223	GPA33	0.00	0.00	0.00	NaN
10224	ZK1	-0.09	0.00	0.00	0.01
10225	TACTILE	0.00	0.00	0.00	NaN
10226	TIP47	0.00	0.00	0.00	NaN
10227	TETRAN	0.00	-0.13	-0.15	0.35
10228	STX6	0.00	0.00	0.00	NaN
10229	COQ7	0.00	0.00	0.00	NaN
10230	NBR2	0.00	-0.13	0.00	0.08
10231	DSCR1L1	0.00	0.00	0.00	NaN
10232	MSLN	0.00	0.00	-0.05	0.08
10233	B7	-0.09	0.00	0.00	0.04
10234	P37NB	0.09	0.00	0.00	-0.13
10235	RASGRP2	0.00	0.00	0.00	NaN
10236	HNRPR	-0.09	0.00	0.00	0.32
10237	UGTREL1	0.00	0.25	0.00	0.64
10238	HAN11	0.27	0.13	0.00	0.38
10239	AP3S2	0.00	0.00	0.00	NaN
10240	MRPS31	0.00	0.00	-0.05	0.22
10241	NDP52	0.00	0.38	0.00	0.70
10242	KCNMB2	0.09	0.00	0.05	0.05
10243	GPHN	0.00	0.00	0.05	0.20
10244	RAB9P40	0.00	0.00	0.00	NaN
10247	UK114	0.00	0.00	0.00	NaN
10248	RPP20	0.18	0.00	0.00	0.37
10249	GLYAT	0.00	0.00	0.00	NaN
10250	SRRM1	-0.09	0.00	0.00	0.20
10252	SPRY1	0.00	0.00	0.00	NaN
10253	SPRY2	-0.09	0.00	-0.05	0.22
10254	STAM2	0.00	0.00	0.00	NaN
10255	HCGIX	0.00	0.00	0.00	NaN
10256	CNK1	0.00	0.00	0.00	NaN
10257	ABCC4	-0.09	0.00	-0.05	0.14
10260	IRLB	0.00	0.00	0.00	NaN
10261	IGSF6	0.00	0.00	0.00	NaN
10262	SF3B4	0.00	0.00	0.05	0.36
10263	DOC-1R	0.27	0.00	0.05	0.21
10265	IRX5	0.09	0.00	0.00	0.33
10266	RAMP2	0.00	-0.13	0.00	0.08

10267	RAMP1	0.00	0.00	-0.05	0.14
10268	RAMP3	0.00	0.00	0.00	NaN
10269	ZMPSTE24	0.00	0.00	0.00	NaN
10270	AKAP8	0.00	0.00	0.00	NaN
10272	FSTL3	0.00	0.00	-0.05	0.18
10273	STUB1	0.00	0.00	-0.05	0.20
10274	STAG1	0.00	0.00	0.00	NaN
10277	UBE4B	0.00	0.00	0.00	NaN
10278	EFS2	0.00	0.00	0.00	NaN
10279	PRSS16	0.00	0.00	0.00	NaN
10280	SR-BP1	-0.09	0.13	0.10	0.56
10281	DSCR4	0.00	0.00	0.00	NaN
10282	BET1	0.18	0.00	0.00	0.50
10284	SAP18	0.00	0.00	-0.10	0.51
10285	SPF30	0.00	0.00	-0.05	0.09
10286	BCAS2	0.18	0.00	0.10	0.67
10287	RGS19	0.00	0.00	0.00	NaN
10288	LILRB2	1.00	0.00	0.50	0.09
10289	GC20	0.00	0.00	-0.05	0.16
10290	APEG1	0.00	0.00	0.00	NaN
10291	SF3A1	0.00	0.00	0.00	NaN
10293	TRIP	-0.09	0.00	-0.05	0.07
10294	DNAJA2	-0.09	0.00	-0.05	0.58
10295	BCKDK	0.00	0.00	0.00	NaN
10296	MAEA	-0.09	-0.13	-0.10	0.45
10297	APCL	0.00	0.00	0.00	NaN
10298	PAK4	0.00	0.00	0.00	NaN
10299	TEB4	0.00	0.00	0.10	0.21
10300	KATNB1	0.00	0.00	0.00	0.35
10301	DLEU1	0.00	0.13	0.00	0.43
10302	SNAPC5	0.00	0.00	0.00	NaN
10307	FE65L2	0.00	0.00	0.00	NaN
10308	ZNF267	0.00	0.00	0.00	NaN
10309	UNG2	0.00	0.00	-0.05	0.12
10311	DSCR3	0.00	0.00	0.00	NaN
10312	TCIRG1	0.18	0.00	0.05	0.30
10313	RTN3	0.00	0.00	0.00	NaN
10314	LANCL1	0.00	0.00	0.00	NaN
10316	GPR66	0.00	0.00	0.00	NaN
10317	B3GALT5	0.00	0.00	0.00	NaN
10318	NAF1	0.00	0.00	0.00	NaN
10319	LAMC3	0.00	0.00	0.00	NaN
10320	ZNFN1A1	0.00	0.00	0.00	NaN
10321	SGP28	0.00	0.00	0.00	NaN
10322	RAI15	0.00	0.00	0.00	NaN
10324	SARCOSIN	0.00	0.00	0.00	NaN
10326	SIRPB1	0.09	0.13	0.25	0.01

10327	AKR1A1	0.00	0.00	0.00	NaN
10328	NOC4	0.00	0.00	-0.05	0.44
10329	TMEM5	0.09	0.00	0.00	0.19
10330	TMEM4	0.00	0.00	0.00	NaN
10331	B3GNT3	0.00	0.00	0.00	NaN
10332	CD209L	0.00	0.00	0.00	NaN
10333	TLR6	0.00	0.00	0.05	-0.12
10336	RNF3	0.00	-0.13	-0.10	0.12
10342	TFG	0.00	0.00	0.00	NaN
10343	PKDREJ	0.00	0.00	0.00	NaN
10346	TRIM22	0.00	-0.13	-0.05	0.12
10347	ABCA7	0.00	0.00	0.00	NaN
10351	ABCA8	0.18	0.13	0.00	-0.17
10352	WARS2	0.09	0.00	0.05	0.51
10360	NPM3	0.00	0.50	0.00	0.12
10362	HMG20B	0.00	0.00	0.00	NaN
10363	HMG20A	0.00	0.00	0.00	NaN
10365	KLF2	0.00	0.00	0.05	-0.05
10367	CBARA1	0.09	0.00	0.00	0.39
10368	CACNG3	0.00	0.00	0.00	NaN
10369	CACNG2	-0.09	0.00	0.00	0.04
10370	CITED2	0.00	0.00	0.00	NaN
10371	SEMA3A	0.09	0.00	0.00	-0.01
10376	K-ALPHA-1	0.00	0.00	0.00	NaN
10379	ISGF3G	0.00	0.00	0.00	NaN
10380	BPNT1	0.09	0.00	0.05	0.42
10381	TUBB4	0.00	0.00	0.00	0.01
10383	TUBB2	0.00	0.00	0.00	NaN
10384	BTN3A3	0.00	0.00	0.00	NaN
10385	BTN2A2	0.00	0.00	0.00	NaN
10388	SYCP2	0.18	0.25	0.05	0.20
10390	CEPT1	0.09	0.00	0.00	0.32
10391	CORO2B	0.00	0.00	0.00	NaN
10392	CARD4	0.00	0.25	0.00	0.33
10393	APC10	0.00	0.00	0.00	NaN
10394	PRG3	0.00	0.00	0.00	NaN
10396	ATP8A1	0.00	0.00	0.00	NaN
10397	NDRG1	0.00	0.00	0.05	0.15
10398	MYL9	0.00	-0.13	0.00	0.15
10399	GNB2L1	0.00	0.13	0.00	0.59
10401	PIAS3	0.00	0.00	0.05	0.17
10402	ST3GALVI	0.00	0.00	0.00	NaN
10403	HEC	0.00	0.00	0.00	NaN
10404	PGCP	0.00	-0.25	0.00	0.22
10406	WFDC2	0.00	0.00	0.00	NaN
10407	SPAG11	-0.27	-0.25	-0.15	-0.02
10408	NCYM	0.09	0.00	-0.10	0.30

10409	BASP1	0.00	0.00	0.10	0.01
10410	IFITM3	-0.27	-0.13	-0.15	0.42
10411	EPAC	0.00	0.00	0.00	NaN
10412	YR-29	0.00	0.00	0.00	NaN
10413	YAP1	0.00	0.13	0.00	0.44
10417	SPON2	0.00	-0.13	-0.10	0.11
10418	SPON1	0.00	0.00	0.00	NaN
10419	SKB1	0.00	0.00	0.00	NaN
10420	TESK2	0.00	0.00	0.00	NaN
10421	CD2BP2	0.00	0.00	0.00	NaN
10422	GDBR1	0.00	0.00	0.00	NaN
10423	CDIPT	0.00	0.00	0.00	NaN
10424	PGRMC2	0.00	0.00	0.00	NaN
10425	ARIH2	-0.09	0.00	-0.05	0.43
10426	GCP3	-0.09	-0.13	-0.05	0.41
10427	SEC24B	0.00	0.00	0.00	NaN
10428	CFDP1	0.00	-0.13	0.05	0.31
10432	RBM14	0.00	0.00	0.05	-0.04
10434	LYPLA1	0.00	0.00	0.00	NaN
10435	CEP2	-0.09	0.00	0.00	-0.08
10436	C2F	0.00	0.00	0.00	0.29
10437	IFI30	0.00	0.00	0.00	NaN
10438	C1D	0.00	0.00	0.00	NaN
10439	OLFM1	0.00	0.00	0.00	NaN
10440	TIMM17A	0.00	0.00	0.00	NaN
10443	CG005	0.00	0.00	0.00	NaN
10444	ZYG	0.00	0.00	0.00	NaN
10445	MCRS1	0.00	0.00	0.00	NaN
10446	GAC1	0.00	0.13	0.00	0.30
10447	GS3786	0.00	0.13	0.00	0.24
10449	ACAA2	-0.09	-0.13	-0.15	0.31
10450	PPIE	0.00	0.00	0.00	NaN
10451	VAV3	0.09	0.00	0.00	-0.09
10452	TOMM40	0.09	0.00	0.00	0.16
10454	MAP3K7IP1	-0.09	0.00	0.05	0.20
10455	PECI	0.00	0.00	0.00	NaN
10456	HAX1	0.09	0.13	0.00	0.39
10457	GPNMB	0.00	0.13	0.00	-0.13
10458	BAIAP2	0.00	0.00	0.00	NaN
10460	TACC3	0.00	-0.13	-0.15	0.22
10461	MERTK	0.00	0.00	0.00	NaN
10462	HML2	0.00	0.00	0.00	NaN
10463	C4orf1	0.00	0.00	0.05	-0.39
10464	PIBF1	-0.09	0.13	-0.15	0.35
10465	PPIH	0.00	0.00	0.00	NaN
10466	COG5	0.09	0.00	0.00	0.11
10467	CG1I	0.18	0.00	0.00	0.40

10468	FST	0.00	0.00	-0.05	0.13
10469	TIMM44	0.00	0.00	0.00	NaN
10471	HKE2	0.09	0.00	0.00	0.15
10472	ZNF238	0.00	0.13	0.00	0.48
10473	HMG17L3	0.00	0.00	0.00	NaN
10474	TADA3L	0.00	0.00	0.00	NaN
10475	RNF15	0.00	0.00	0.05	-0.08
10477	UBE2E3	0.00	0.00	0.00	NaN
10478	SLC25A17	0.00	0.00	0.05	0.13
10480	GA17	0.09	0.00	0.00	0.32
10481	HOXB13	0.00	0.38	0.00	0.46
10482	NXF1	0.00	0.00	0.00	NaN
10483	SEC23B	0.00	0.00	0.00	NaN
10484	SEC23A	0.00	0.00	0.00	NaN
10485	CROC4	0.00	0.13	0.00	-0.03
10488	CREB3	-0.09	0.00	0.10	0.50
10489	MUF1	0.00	0.00	0.00	NaN
10490	VTI2	0.00	0.00	0.00	NaN
10491	CRTAP	0.00	0.00	0.00	NaN
10492	NSAP1	0.00	0.00	0.00	NaN
10494	STK25	0.00	0.00	-0.05	0.41
10497	UNC13	-0.09	0.13	0.10	0.62
10498	CARM1	0.00	0.00	-0.05	0.17
10499	NCOA2	0.50	0.00	0.00	0.19
10500	SEMA6C	0.00	0.00	0.05	0.16
10501	SEMA6B	0.00	0.00	0.00	NaN
10505	SEMA4F	0.00	0.00	0.00	NaN
10507	SEMA4D	0.00	0.00	0.00	NaN
10512	SEMA3C	0.09	0.00	0.00	0.20
10513	APPBP2	0.18	0.13	0.00	0.79
10514	MYBBP1A	0.00	0.00	0.00	NaN
10516	FBLN5	0.00	0.13	0.00	0.24
10517	C17orf1A	0.09	0.00	0.00	0.23
10518	KIP2	0.00	0.00	0.00	NaN
10519	CIB1	0.00	0.00	0.00	NaN
10520	ZNF211	0.00	0.13	0.00	-0.03
10521	DDX17	-0.09	0.00	0.05	0.10
10522	DEAF1	-0.18	-0.13	0.00	0.36
10523	CHERP	0.00	0.00	0.00	NaN
10524	HTATIP	0.00	0.00	0.05	0.16
10525	ORP150	0.00	0.00	-0.05	0.29
10526	RANBP8	0.00	0.00	0.00	NaN
10527	RANBP7	0.00	0.00	0.00	NaN
10528	NOL5A	0.00	0.00	0.05	0.17
10529	NEBL	-0.09	0.00	0.05	0.21
10531	MP1	0.00	0.00	0.00	NaN
10533	GSA7	0.00	0.00	0.00	NaN

10534	SSSCA1	-0.09	0.00	0.05	0.55
10535	RNASEHI	0.00	0.00	0.00	NaN
10536	HSU47926	-0.09	0.00	0.00	0.18
10537	UBD	0.00	0.00	0.00	NaN
10538	BATF	0.00	0.00	0.00	NaN
10539	TXNL2	0.00	0.00	-0.05	0.19
10540	DCTN2	0.00	0.00	0.00	NaN
10541	ANP32B	0.00	0.00	0.00	NaN
10542	HBXIP	0.09	0.00	0.00	0.42
10544	PROCR	0.00	0.13	0.00	-0.14
10548	TM9SF1	0.00	0.00	0.00	NaN
10550	JWA	0.00	0.00	-0.05	0.45
10551	AGR2	0.00	0.13	0.00	0.19
10552	ARPC1A	0.18	0.00	0.00	0.60
10553	HTATIP2	0.00	0.00	0.00	NaN
10554	AGPAT1	0.00	0.00	0.00	NaN
10555	AGPAT2	0.00	0.00	0.00	NaN
10556	RPP30	0.09	0.00	0.00	0.38
10557	RPP38	0.00	0.00	0.00	NaN
10558	SPTLC1	0.00	0.00	0.00	NaN
10559	SLC35A1	0.00	0.00	0.00	NaN
10560	SLC19A2	0.00	0.00	-0.05	0.28
10561	IFI44	0.00	0.00	0.00	NaN
10563	SCYB13	0.00	0.00	0.00	NaN
10564	ARFGEF2	0.27	0.00	0.00	0.21
10565	BIG1	0.00	0.00	0.00	NaN
10566	AKAP3	-0.09	0.00	0.05	0.08
10567	RABAC1	0.00	0.00	-0.05	0.08
10568	SLC34A2	0.00	0.00	-0.05	0.05
10570	DPYSL4	0.00	0.00	-0.10	0.18
10572	SIVA	0.00	0.00	0.00	NaN
10573	MAAT1	0.00	0.00	-0.05	0.30
10574	CCT7	0.00	0.00	0.00	NaN
10575	CCT4	0.00	0.00	0.00	NaN
10576	CCT2	0.09	0.00	0.00	0.22
10577	NPC2	0.00	0.00	0.00	NaN
10578	GNLY	0.00	0.00	-0.05	0.07
10579	TACC2	0.00	0.00	-0.05	0.16
10580	SH3D5	0.00	0.00	-0.05	-0.07
10581	IFITM2	-0.27	-0.13	-0.05	0.21
10584	COLEC10	0.00	0.25	0.10	0.26
10585	POMT1	0.00	0.00	0.00	NaN
10586	MAB21L2	0.00	0.00	0.00	-0.01
10587	TXNRD2	-0.09	0.00	0.00	0.15
10588	MTHFS	0.00	0.00	0.00	NaN
10589	DRAP1	-0.09	0.00	0.05	0.41
10590	SCGN	0.00	0.00	0.05	-0.02

10591	RCL	0.00	0.00	0.00	NaN
10592	SMC2L1	0.00	0.00	0.00	NaN
10594	PRPF8	0.00	-0.13	0.00	0.14
10595	ERN2	0.00	0.00	-0.05	0.25
10597	SEDLP	0.09	0.13	0.00	0.32
10598	C14orf3	0.00	0.00	0.00	NaN
10599	SLC21A6	0.00	0.00	0.00	NaN
10600	USP16	0.00	0.00	0.00	NaN
10602	CEP3	0.00	0.00	0.00	NaN
10603	APS	0.18	0.00	0.00	0.06
10605	PAIP1	0.00	0.00	0.05	0.02
10606	PAICS	0.00	0.00	0.00	NaN
10607	TBL3	0.00	0.00	0.00	NaN
10608	MAD4	0.00	-0.13	-0.20	0.31
10609	SC65	0.00	0.13	0.00	0.36
10610	STHM	0.00	0.13	0.00	0.43
10611	LIM	0.00	0.00	0.00	NaN
10612	TRIM3	0.00	-0.13	0.00	0.01
10613	KEO4	0.00	-0.13	-0.05	0.27
10614	HIS1	0.00	0.00	0.00	0.10
10615	DEEPEST	0.00	0.38	0.00	0.42
10616	C20orf18	0.00	0.00	0.05	0.34
10617	AMSH	0.00	0.00	0.00	NaN
10618	TGOLN2	0.00	0.00	0.00	NaN
10620	DRIL2	0.00	0.00	0.00	NaN
10621	POLR3F	0.00	0.00	0.00	NaN
10622	RPC32	0.00	0.00	0.00	NaN
10623	RPC62	0.00	0.00	0.05	0.19
10625	NS1-BP	0.00	0.00	0.00	NaN
10626	TRIM16	0.09	0.13	0.05	-0.09
10627	MLCB	0.09	0.00	-0.05	0.62
10628	TXNIP	0.00	0.00	0.05	-0.17
10629	TAF6L	0.00	0.00	0.00	NaN
10630	T1A-2	0.00	0.00	0.00	NaN
10631	OSF-2	0.00	0.00	0.00	NaN
10632	ATP5L	0.00	0.00	0.00	NaN
10633	RRP22	-0.09	0.00	0.05	-0.08
10634	GAS2L1	-0.09	0.00	0.05	0.32
10635	PIR51	-0.09	0.00	0.05	0.36
10636	RGS14	0.00	0.13	0.00	0.29
10637	LEFTB	0.09	0.00	0.00	-0.20
10638	SPHAR	0.09	0.00	0.00	0.30
10640	SEC10L1	0.00	0.00	0.05	0.19
10641	NPR2L	-0.09	0.00	-0.05	0.42
10643	KOC1	0.00	0.50	0.00	-0.12
10644	IMP-2	0.09	0.00	0.05	0.06
10645	CAMKK2	0.00	-0.13	0.00	0.12

10647	SCGB1D2	-0.09	0.00	0.00	-0.50
10648	SCGB1D1	-0.09	0.00	0.00	-0.20
10650	HFL-EDDG1	0.00	0.00	0.00	NaN
10651	MTX2	0.00	0.00	0.00	NaN
10652	YKT6	0.00	0.00	0.00	NaN
10653	SPINT2	0.00	0.13	0.00	0.21
10654	PMVK	0.09	0.13	0.00	0.39
10656	KHDRBS3	0.00	0.00	0.10	-0.09
10657	KHDRBS1	0.00	0.00	0.00	NaN
10658	CUGBP1	0.00	0.00	0.00	NaN
10659	CUGBP2	0.00	0.00	0.00	NaN
10660	LBX1	0.00	-0.13	-0.05	-0.06
10661	KLF1	0.00	0.00	0.00	NaN
10663	TYMSTR	-0.09	0.00	-0.05	-0.09
10664	CTCF	-0.09	0.00	0.00	0.35
10665	C6orf10	0.00	0.00	0.00	NaN
10666	DNAM-1	-0.09	0.00	-0.10	0.11
10667	FARS1	0.00	0.00	0.00	NaN
10668	CGR19	0.00	0.00	0.05	0.34
10669	CGR11	0.09	0.00	0.05	-0.03
10670	RAGA	0.00	-0.25	-0.10	0.55
10671	WS-3	-0.09	-0.25	-0.05	0.55
10672	GNA13	0.18	0.13	0.05	0.39
10675	CSPG5	-0.09	0.00	-0.05	0.18
10677	AVIL	0.00	0.00	0.00	NaN
10678	B3GNT1	0.00	0.00	0.50	-0.27
10681	GNB5	0.18	0.00	0.00	0.35
10683	DLL3	0.09	0.00	0.00	0.04
10686	CLDN16	0.00	0.00	0.05	0.07
10687	PNMA2	-0.09	-0.25	-0.05	0.15
10690	FUT9	-0.09	0.00	0.00	0.07
10691	GMEB1	0.00	0.00	0.00	NaN
10692	RRH	0.00	0.00	0.00	NaN
10693	CCT6B	0.00	-0.13	0.00	0.13
10694	CCT8	0.00	0.00	0.00	NaN
10695	TNRC5	0.00	0.00	0.00	NaN
10699	PRSC	0.00	0.00	0.00	NaN
10712	C1orf2	0.09	0.13	0.00	0.40
10713	SAD1	0.00	0.00	-0.05	0.25
10714	POLD3	0.09	0.00	0.00	0.45
10716	TBR1	0.00	0.00	-0.05	-0.09
10721	POLQ	0.00	0.00	0.00	NaN
10723	SLC12A7	0.00	0.13	0.10	-0.03
10724	MGEA5	0.00	-0.13	-0.05	0.25
10725	NFAT5	0.00	-0.13	0.05	0.36
10726	NUDC	-0.09	-0.13	0.00	0.44
10728	P23	0.00	0.00	0.00	NaN

10730	YME1L1	-0.09	0.00	0.00	0.24
10732	TCFL5	0.09	0.13	0.00	0.12
10733	STK18	0.00	0.00	0.00	NaN
10734	STAG3	0.18	0.00	0.00	0.10
10736	SIX2	0.00	0.00	0.00	NaN
10738	RFPL3	0.00	0.00	0.00	NaN
10739	RFPL2	0.00	0.00	0.00	NaN
10740	RFPL1S	0.00	0.00	0.00	NaN
10741	RBBP9	0.00	0.00	0.00	NaN
10743	RAI1	0.00	0.00	0.00	NaN
10744	PTTG2	0.00	0.00	0.00	NaN
10745	PHTF1	0.18	0.13	0.05	0.45
10747	MASP2	0.00	0.00	0.00	NaN
10748	KLRA1	-0.09	0.00	0.00	0.02
10749	KIF1C	0.00	0.00	0.00	NaN
10750	GRAP	0.00	0.00	0.05	0.01
10753	CAPN9	0.09	0.00	0.00	0.26
10755	RGS19IP1	0.00	0.00	0.00	NaN
10762	NUP50	0.00	0.00	0.00	NaN
10765	PLU-1	0.09	0.13	0.05	0.45
10766	TOB2	0.00	0.00	0.05	-0.21
10767	HBS1L	0.00	0.00	0.05	0.42
10768	AHCYL1	0.09	0.00	0.05	0.28
10769	SNK	0.00	0.00	0.00	NaN
10771	BS69	0.00	0.00	0.05	0.51
10772	FUSIP1	-0.09	0.00	0.00	0.15
10773	ZID	0.00	0.00	0.00	NaN
10775	POP4	0.00	0.13	0.15	0.75
10776	ARPP-19	0.09	0.00	0.00	0.50
10777	ARPP-21	0.00	0.00	0.00	NaN
10781	ZNF266	0.00	0.13	0.00	0.41
10782	ZNF274	0.00	0.13	0.05	0.28
10785	WDR4	0.00	0.00	0.00	NaN
10786	SLC17A3	0.00	0.00	0.05	-0.10
10787	NCKAP1	0.00	0.00	0.00	NaN
10788	IQGAP2	0.00	0.00	0.00	NaN
10791	VAMP5	0.00	0.00	-0.05	0.18
10793	ZNF273	0.09	0.00	0.00	-0.23
10794	ZNF272	0.09	0.13	0.00	0.49
10795	ZNF268	0.00	0.00	0.00	NaN
10797	MTHFD2	0.00	0.00	0.00	NaN
10798	OR5I1	0.09	0.00	0.00	0.13
10799	RPP40	0.00	0.00	0.00	NaN
10801	MSF	0.00	0.50	0.00	0.34
10802	SEC24A	0.00	-0.13	0.00	0.24
10807	SDCCAG3	0.00	0.00	0.00	NaN
10808	HSP105B	0.00	0.00	0.05	0.25

10810	WASF3	0.00	-0.13	-0.10	-0.08
10814	CPLX2	0.00	0.00	0.00	NaN
10817	SNT-2	0.00	0.00	0.05	-0.04
10818	SNT-1	0.09	0.00	0.00	0.35
10825	NEU3	0.09	0.13	0.00	0.11
10826	C5orf4	0.00	0.00	0.00	NaN
10827	C5orf3	0.00	0.00	0.00	NaN
10840	FTHFD	0.00	0.00	0.00	NaN
10841	FTCD	0.00	0.00	0.00	NaN
10842	GSBS	0.00	0.13	0.00	0.55
10844	GCP2	0.00	0.00	-0.10	0.47
10845	CLPX	0.00	0.00	0.00	NaN
10846	PDE10A	0.00	0.00	0.00	NaN
10847	SRCAP	0.00	0.00	0.50	0.40
10848	RAI	0.09	0.00	0.00	0.07
10849	ASE-1	0.09	0.00	0.00	0.05
10850	SCYA27	-0.09	0.13	0.10	-0.03
10855	HPSE	0.00	0.00	0.00	NaN
10856	RUVBL2	0.09	0.00	0.00	0.17
10858	CYP46	0.00	0.00	0.00	NaN
10859	LILRB1	0.09	0.00	0.00	0.04
10861	SLC26A1	0.00	-0.13	-0.05	0.05
10863	ADAM28	0.00	-0.13	-0.05	-0.02
10864	SLC22A7	0.00	0.00	0.00	NaN
10865	MRF-1	0.00	0.00	0.00	NaN
10866	P5-1	0.00	0.00	0.00	NaN
10867	NET-5	0.00	0.00	0.05	0.18
10868	USP20	0.00	0.00	0.00	NaN
10869	USP19	-0.09	0.00	-0.05	0.32
10871	CMRF35	0.00	0.13	0.00	0.04
10873	ME3	0.00	0.00	0.00	NaN
10874	NMU	0.00	0.00	0.00	NaN
10875	FGL2	-0.09	0.00	0.00	0.04
10876	HE3-ALPHA	0.00	0.00	0.00	NaN
10877	FHR-4	0.00	0.13	0.00	-0.05
10879	PROL3	0.00	0.00	0.00	NaN
10880	ACTL7B	0.00	0.00	0.05	0.61
10881	ACTL7A	0.00	0.00	0.00	NaN
10882	CRF	0.00	-0.13	0.00	-0.04
10884	MRPS30	0.00	0.00	0.10	-0.17
10885	WDR3	0.09	0.00	0.05	0.29
10891	PPARGC1	0.00	0.00	-0.05	0.05
10892	MALT1	-0.09	0.00	-0.15	0.40
10893	MMP24	0.00	0.50	0.00	0.17
10894	XLKD1	0.00	0.00	0.00	NaN
10895	SPBPBP	0.00	0.00	0.00	NaN
10896	OCLM	0.00	0.00	0.00	NaN

10897	54TM	0.09	0.00	0.05	0.35
10898	CPSF4	0.18	0.00	0.00	0.57
10899	JTB	0.09	0.13	0.00	0.34
10900	RPIP8	0.00	-0.13	0.00	0.02
10901	humNRDR	0.00	0.00	0.00	NaN
10902	SMAP	0.00	0.00	0.00	NaN
10903	CRA	0.00	0.00	0.05	0.46
10904	BLCAP	0.00	-0.13	0.00	0.29
10905	MAN1A2	0.09	0.00	0.05	0.06
10906	FLN29	0.00	0.00	0.00	NaN
10907	DIM1	-0.09	0.00	-0.25	0.67
10908	NTE	0.00	0.00	0.00	NaN
10911	UTS2	0.00	0.00	0.00	0.00
10912	GADD45G	0.00	0.00	0.00	NaN
10913	EDAR	0.00	0.00	0.00	NaN
10915	TCERG1	0.00	0.00	0.00	NaN
10919	BAT8	0.00	0.00	0.00	NaN
10920	COP9	0.00	0.00	-0.05	0.41
10921	RNPS1	0.00	0.00	0.00	NaN
10922	FASTK	0.00	0.00	-0.05	0.48
10923	PC4	0.00	0.00	0.15	0.34
10924	ASM3A	0.00	0.00	0.00	NaN
10926	ASK	0.00	0.00	0.00	NaN
10927	SPIN	0.00	0.00	0.00	NaN
10928	RALBP1	0.00	0.00	0.00	NaN
10929	SRP46	-0.09	0.13	-0.05	0.35
10930	APOBEC2	0.00	0.00	0.00	NaN
10933	MRG15	0.00	0.00	0.00	NaN
10934	MORF4	0.00	0.00	-0.05	-0.09
10935	PRDX3	0.00	0.00	-0.05	0.32
10936	GPR75	0.09	0.00	0.00	0.09
10938	EHD1	0.00	0.00	0.00	NaN
10939	AFG3L2	0.00	0.00	0.00	NaN
10942	PRSS21	0.00	0.00	0.00	NaN
10944	IMAGE145052	0.00	0.00	0.00	NaN
10945	KDELR1	0.09	0.00	0.00	0.32
10946	SF3A3	0.00	0.00	0.00	NaN
10947	AP3M2	0.36	-0.13	-0.05	0.59
10948	MLN64	0.00	1.00	0.00	0.87
10949	HNRPA0	0.00	-0.13	0.00	0.16
10950	BTG3	0.00	-0.13	0.00	0.25
10951	CBX1	0.00	0.00	0.00	0.41
10952	SEC61B	0.00	0.00	0.00	NaN
10953	TOMM34	0.09	0.13	0.00	0.39
10954	PDIR	0.00	0.00	0.00	NaN
10955	TDE1	0.09	0.13	0.00	0.59
10956	OS-9	0.00	0.00	0.00	NaN

10957	PROL2	0.00	0.00	0.00	NaN
10959	RNP24	0.00	0.00	0.00	NaN
10960	C5orf8	0.00	0.13	0.00	0.56
10961	C12orf8	0.00	0.13	0.00	0.34
10962	AF1Q	0.00	0.00	0.05	-0.03
10963	STIP1	0.00	0.00	0.00	NaN
10964	C1orf29	0.00	0.00	0.00	NaN
10965	ZAP128	0.00	0.00	0.00	0.27
10966	RAB40B	0.00	0.00	0.00	NaN
10969	EBNA1BP2	0.00	0.00	0.00	NaN
10970	CKAP4	0.00	0.00	0.00	NaN
10971	YWHAQ	0.00	0.00	-0.05	0.47
10972	TMP21	0.00	0.00	0.00	NaN
10973	RNAH	0.00	0.13	0.00	-0.14
10974	APM2	0.09	0.00	-0.10	0.35
10975	UQCR	0.00	0.00	0.00	NaN
10979	MIG2	0.00	0.00	0.05	0.30
10980	MOV34-34KD	0.18	0.00	0.00	0.52
10981	RAB32	0.00	-0.13	0.00	0.07
10982	MAPRE2	0.00	0.00	-0.20	0.35
10983	CCNI	0.00	0.00	0.00	NaN
10985	GCN1L1	0.00	-0.13	0.00	0.37
10987	COPS5	0.00	0.00	0.00	NaN
10988	METAP2	0.00	0.13	0.00	0.26
10989	IMMT	0.00	0.00	-0.05	0.40
10990	LILRB5	0.09	0.00	0.00	0.06
10991	SLC38A3	-0.09	0.00	-0.10	0.20
10992	SF3B2	-0.09	0.00	0.05	0.43
10993	SDS	0.00	0.00	0.00	NaN
10994	ILVBL	0.00	0.00	0.05	0.24
11004	KNSL6	0.00	0.00	0.00	NaN
11005	SPINK5	0.00	0.00	0.00	NaN
11006	LILRB4	0.09	0.00	0.00	0.20
11007	DIPA	-0.09	0.00	0.05	0.42
11009	IL24	0.00	0.13	0.00	0.31
11010	RTVP1	0.00	0.00	0.00	NaN
11011	TLK2	0.27	0.13	0.00	0.74
11012	KLK11	0.00	0.00	0.00	NaN
11014	KDELR2	0.00	0.00	0.10	0.38
11015	KDELR3	-0.09	0.00	0.05	0.27
11016	ATF7	0.00	0.00	0.00	NaN
11017	RY1	0.00	0.00	0.00	NaN
11018	IL1RL1LG	0.00	0.00	-0.05	0.05
11020	RABL4	-0.09	0.00	0.00	0.10
11021	RAB35	0.00	-0.13	0.00	0.13
11022	TDRKH	0.00	0.13	0.05	0.49
11024	LILRA1	0.09	0.00	0.00	0.03

11025	LILRB3	0.09	0.00	0.00	0.11
11026	LILRA3	0.09	0.13	0.05	-0.27
11027	LILRA2	0.09	0.00	0.00	0.04
11030	RBPMS	-0.09	-0.25	-0.05	0.13
11031	RAB31	0.00	0.00	0.00	NaN
11033	CENTA1	0.00	0.00	0.00	0.35
11034	DSTN	0.00	0.00	0.00	NaN
11037	SALF	0.00	0.00	0.00	NaN
11041	B3GNT6	0.27	0.00	0.00	-0.11
11044	POLS	0.00	0.00	0.50	0.68
11045	UPK1A	0.00	0.00	0.00	NaN
11046	SQV7L	0.00	0.00	0.00	NaN
11047	ADRM1	0.09	0.13	0.00	0.37
11051	CPSF5	0.00	0.00	0.00	NaN
11052	CPSF6	0.09	0.13	0.00	0.08
11054	OGFR	0.09	0.13	0.00	0.13
11055	ZPBP	0.00	0.00	0.00	NaN
11056	ROK1	0.00	0.00	0.00	0.35
11057	LABH2	0.00	0.00	-0.05	0.16
11059	WWP1	0.18	0.25	0.00	0.41
11060	WWP2	0.00	-0.13	0.05	0.42
11061	CHM-I	0.00	0.00	0.00	NaN
11062	PP35	0.09	0.00	0.00	0.54
11063	SOX30	0.00	0.00	0.00	NaN
11064	CEP1	0.00	0.00	-0.10	0.19
11065	UBE2C	0.00	0.00	0.00	NaN
11066	U1SNRNPBP	0.00	0.00	0.00	NaN
11067	DEPP	0.00	0.00	0.00	NaN
11068	101F6	-0.09	0.00	-0.05	0.22
11069	CAMP-GEFII	0.00	0.00	-0.05	0.02
11070	PL6	-0.09	0.00	-0.05	0.13
11072	DUSP14	0.00	0.00	0.00	0.32
11073	TOPBP1	0.00	0.00	0.00	NaN
11074	TRIM31	0.00	0.00	0.00	NaN
11075	STMN2	0.00	0.25	0.05	-0.03
11076	p25	0.00	0.13	0.10	0.18
11077	HSF2BP	0.00	0.00	0.00	NaN
11078	HRIHFB2122	-0.09	0.00	0.05	0.39
11079	RER1	0.00	0.00	0.00	NaN
11080	DNAJB4	0.00	0.00	0.00	NaN
11081	KERA	0.00	0.00	0.00	NaN
11082	ESM1	0.00	0.00	-0.05	0.08
11083	DATF1	0.09	0.13	0.00	0.43
11085	ADAM30	0.18	0.13	0.05	-0.10
11086	ADAM29	0.00	0.00	-0.05	0.01
11092	C9orf9	0.00	0.00	0.00	NaN
11094	C9orf7	0.00	0.00	0.00	NaN

11095	ADAMTS8	-0.09	0.00	-0.10	-0.05
11096	ADAMTS5	0.00	0.00	0.00	NaN
11097	NLP_1	0.00	0.13	0.00	-0.29
11098	SPUVE	0.00	0.00	0.00	NaN
11099	PTPN21	0.00	0.13	0.00	0.21
11100	E1B-AP5	0.00	0.00	-0.05	0.42
11102	RPP14	0.00	-0.13	-0.10	0.24
11103	HRB2	0.00	0.00	0.00	NaN
11104	KATNA1	0.00	0.00	0.00	NaN
11107	PRDM5	0.00	0.00	0.00	NaN
11108	PRDM4	0.00	-0.13	0.00	0.12
11113	CIT	0.00	-0.13	0.00	0.25
11116	FOP	0.00	0.00	0.00	NaN
11117	EMILIN	0.09	0.00	0.05	-0.05
11118	BTN3A2	0.00	0.00	0.00	NaN
11119	BTN3A1	0.00	0.00	0.00	NaN
11120	BTN2A1	0.00	0.00	0.00	NaN
11122	PTPRT	0.18	-0.13	0.00	0.18
11123	DSCR1L2	0.00	0.00	0.00	0.14
11124	FAF1	0.00	0.00	0.00	NaN
11126	BY55	0.00	0.00	0.05	0.01
11127	KIF3A	0.00	-0.13	0.00	0.10
11129	SWAP2	0.09	0.00	0.00	0.36
11130	ZWINT	0.00	0.13	0.00	0.32
11131	CAPN11	0.00	0.00	0.00	NaN
11132	CAPN10	0.00	0.00	-0.05	0.32
11133	KPTN	0.09	0.00	0.00	-0.03
11135	MSE55	-0.09	0.00	0.05	-0.17
11136	SLC7A9	0.00	0.13	0.05	-0.03
11137	PWP1	0.00	-0.13	0.00	0.24
11138	VRP	0.00	0.00	0.00	NaN
11140	CDC37	0.00	0.00	0.05	0.42
11142	PKIG	0.09	0.13	0.00	0.20
11143	HBOA	0.00	0.25	0.00	0.68
11144	DMC1	-0.09	0.00	0.05	0.06
11145	HRASLS3	0.00	0.00	0.00	NaN
11146	FAP48	0.00	0.00	0.05	0.59
11147	HHLA3	0.00	0.00	0.00	NaN
11148	HHLA2	0.00	0.00	0.00	NaN
11151	CORO1A	0.00	0.00	0.00	NaN
11153	HYPE	0.00	-0.13	0.00	0.07
11154	AP4S1	0.00	0.00	0.00	NaN
11155	LDB3	0.09	0.00	-0.05	-0.02
11156	PTP4A3	0.18	0.00	0.05	0.53
11157	LSM6	0.00	0.00	0.00	NaN
11158	RABL2B	0.09	-0.25	-0.05	0.26
11159	RABL2A	0.00	0.00	0.00	NaN

11160	C8orf2	0.55	-0.13	0.00	0.72
11161	C14orf1	0.00	0.00	0.00	NaN
11162	NUDT6	0.00	0.00	0.00	NaN
11163	NUDT4	0.00	0.13	0.00	0.35
11165	NUDT3	0.00	0.00	0.00	NaN
11166	SOX21	-0.09	-0.13	-0.05	0.26
11167	FSTL1	0.00	0.00	0.00	NaN
11168	PSIP2	0.00	-0.13	0.00	0.56
11169	AND-1	0.00	0.00	0.05	0.22
11170	TU3A	0.00	-0.13	-0.15	0.22
11171	UNRIP	0.00	0.00	0.00	NaN
11173	ADAMTS7	0.00	0.00	0.00	NaN
11174	ADAMTS6	0.00	0.00	0.00	NaN
11176	BAZ2A	0.00	0.00	0.00	NaN
11177	BAZ1A	0.00	0.00	0.00	NaN
11178	LZTS1	-0.09	-0.38	-0.10	0.11
11179	ZNF277	0.09	0.00	0.00	0.12
11180	WDR6	-0.09	0.00	-0.05	0.33
11181	TREH	0.00	0.00	0.00	NaN
11182	SLC2A6	0.00	0.00	0.00	NaN
11183	MAP4K5	0.00	0.00	0.00	NaN
11184	MAP4K1	0.00	0.00	0.00	NaN
11186	RASSF1	-0.09	0.00	-0.05	0.05
11187	PKP3	-0.27	-0.13	-0.15	0.24
11188	NISCH	-0.09	-0.25	-0.10	0.55
11189	TNRC4	0.00	0.13	0.05	-0.27
11190	CEP2	0.00	-0.13	0.00	0.04
11193	WBP4	0.00	0.00	-0.05	0.31
11194	ABCB8	0.00	0.00	-0.05	0.12
11196	P125	0.00	0.00	-0.05	0.14
11197	WIF1	0.09	0.00	0.00	-0.14
11198	FACTP140	0.00	0.00	0.00	NaN
11199	ANXA10	0.00	0.00	-0.05	0.04
11200	CHEK2	-0.09	0.00	0.00	0.24
11201	POLI	-0.09	0.00	-0.20	0.41
11202	KLK8	0.00	0.00	0.00	NaN
11211	FZD10	0.00	0.00	0.00	NaN
11212	PROSC	0.55	-0.13	0.00	0.81
11213	IRAK-M	0.09	0.00	0.00	-0.19
11215	AKAP11	0.00	0.00	-0.05	0.19
11216	AKAP10	0.00	-0.13	0.00	0.26
11217	AKAP2	0.00	0.00	0.00	NaN
11221	DUSP10	0.09	0.00	0.00	0.25
11222	MRPL3	0.00	0.00	0.00	NaN
11223	MSTP9	0.00	0.00	-0.05	0.10
11224	RPL35	0.00	0.00	0.00	NaN
11226	GALNT6	0.00	0.00	0.00	NaN

11228	C12orf2	0.00	0.00	0.00	NaN
11231	SEC63L	0.00	0.00	0.10	0.63
11232	POLG2	0.27	0.13	0.00	0.59
11234	AIBP63	0.00	0.00	0.00	NaN
11235	PDCD10	0.00	0.00	0.00	NaN
11236	TRC8	0.18	0.38	-0.05	0.70
11237	RNF24	0.00	0.00	0.00	NaN
11245	GPR	0.00	0.00	-0.05	-0.28
11247	NXPH4	0.00	0.00	0.00	NaN
11248	NXPH3	0.00	0.25	0.00	0.27
11250	GPR45	0.00	0.00	0.00	NaN
11251	GPR44	0.00	0.00	0.00	NaN
11252	PACSIN2	0.00	0.00	0.00	NaN
11253	MAN1B1	0.00	0.00	0.00	NaN
11255	HRH3	0.09	0.13	0.00	0.09
11257	TP53TG1	0.00	0.00	0.00	NaN
11258	DCTN3	-0.09	0.13	0.10	0.61
11259	DOC1	0.00	0.00	0.00	NaN
11260	XPOT	0.09	0.00	0.00	0.45
11261	CHP	0.00	0.00	0.00	NaN
11262	SP140	0.00	0.00	0.00	NaN
11264	PXMP4	-0.09	-0.13	0.00	0.13
11266	DUSP12	0.00	0.00	0.00	NaN
11267	EAP30	0.00	0.38	0.00	0.68
11269	DDX19	-0.09	-0.13	0.05	0.50
11272	PROL4	-0.09	0.00	0.00	0.19
11273	A2LP	0.00	0.00	0.00	NaN
11274	USP18	0.00	0.00	0.00	0.12
11275	KLHL2	0.00	0.00	0.00	NaN
11277	TREX1	-0.09	0.00	-0.05	0.01
11278	KLF12	-0.09	0.00	-0.15	0.11
11281	RPF-1	0.00	0.00	0.00	NaN
11282	MGAT4B	0.00	0.00	0.00	NaN
11283	CYP4F8	0.00	0.00	0.00	NaN
11284	PNKP	0.00	0.00	0.00	NaN
11285	B4GALT7	0.00	0.00	0.00	NaN
11309	SLC21A9	0.09	0.00	0.00	0.03
11311	VPS45A	0.00	0.00	0.05	0.48
11313	LYPLA2	-0.09	0.00	0.00	0.06
11314	CMRF-35H	0.00	0.13	0.00	0.11
11315	DJ-1	0.00	0.00	0.00	NaN
11316	COPE	0.00	0.13	0.00	-0.23
11317	RBPSUHL	0.00	0.00	0.00	NaN
11318	ADMR	0.00	0.00	0.00	NaN
11320	MGAT4A	0.00	0.00	0.00	NaN
11321	NTPBP	0.09	0.00	0.05	0.65
11322	LAK-4P	0.00	0.13	0.00	0.21

11325	RNAHP	0.27	0.13	0.00	0.53
11329	STK38	0.00	0.00	0.00	NaN
11330	CTRC	0.00	0.00	0.00	NaN
11331	REA	0.00	0.00	0.00	0.54
11332	BACH	0.00	0.00	0.00	NaN
11333	PDAP1	0.18	0.00	0.00	0.69
11334	FUS1	-0.09	0.00	-0.05	0.20
11335	CBX3	0.00	0.13	0.00	-0.32
11336	SEC6	0.00	0.13	0.10	0.60
11337	GABARAP	0.00	0.00	0.00	NaN
11338	U2AF65	0.09	0.13	0.00	0.23
11339	OIP5	0.00	0.00	0.00	NaN
11341	SCRG1	0.00	0.00	-0.05	0.00
11342	RNF13	0.00	0.00	0.05	0.24
11343	MGLL	0.00	0.00	0.00	NaN
11344	PTK9L	-0.09	0.00	-0.10	0.19
11345	GABARAPL2	0.00	-0.13	0.05	0.36
11346	KIAA1029	0.00	0.00	0.00	NaN
22794	MLN51	0.00	0.38	0.00	0.84
22795	NID2	0.00	0.13	0.05	0.26
22796	LDLC	0.09	0.00	0.00	0.48
22797	TFEC	0.00	0.25	0.00	-0.04
22798	LAMB4	0.09	0.00	0.00	0.10
22800	RRAS2	0.00	0.00	0.00	NaN
22802	CLCA4	0.00	0.00	0.10	-0.04
22806	ZNFN1A3	0.00	1.00	0.00	0.39
22807	ZNFN1A2	0.00	0.00	0.00	NaN
22808	MRAS	0.00	0.00	0.00	NaN
22809	ATF5	0.00	0.00	0.00	NaN
22821	GAP1IP4BP	-0.09	-0.13	-0.05	0.21
22822	PHLDA1	0.00	0.00	0.00	NaN
22823	M96	0.00	0.00	0.05	0.51
22824	APG-1	0.00	0.00	0.00	NaN
22826	DNAJC8	0.00	0.00	0.00	NaN
22827	SIAHBP1	0.18	0.00	0.10	0.49
22828	KIAA1116	0.00	0.00	0.00	NaN
22832	KIAA1009	0.00	0.00	0.00	NaN
22834	KIAA0924	0.00	0.38	0.00	0.55
22835	KIAA0961	0.00	0.13	0.00	0.15
22836	KIAA0878	0.00	0.00	0.00	NaN
22837	KIAA0977	0.00	0.00	0.00	NaN
22838	KIAA1100	0.00	0.00	0.00	NaN
22839	KIAA0964	0.00	0.00	0.00	NaN
22841	Rab11-FIP2	0.00	0.00	-0.05	0.21
22843	KIAA1072	0.18	0.13	0.00	0.20
22844	KIAA0967	-0.09	0.00	0.05	-0.13
22845	KIAA1094	0.00	0.00	0.00	NaN

22846	KIAA1036	0.00	0.00	0.00	NaN
22847	KIAA1084	0.00	0.00	0.05	-0.16
22848	KIAA1048	0.00	0.00	0.00	NaN
22849	KIAA0940	0.00	0.00	0.00	NaN
22850	KIAA0863	-0.09	0.00	-0.25	0.53
22852	KIAA1074	-0.09	0.00	0.00	0.26
22853	KIAA1079	0.18	0.00	0.00	0.27
22854	KIAA0976	0.18	0.00	0.00	0.20
22856	CHSY1	0.00	0.00	0.00	NaN
22858	ICK	0.00	0.00	0.00	NaN
22859	LEC2	0.00	0.00	0.00	NaN
22861	DEFCAP	0.00	-0.13	0.00	-0.02
22862	KIAA0970	0.00	0.13	0.00	0.40
22863	KIAA0831	0.00	0.00	0.05	0.15
22865	KIAA0848	0.00	0.00	0.00	NaN
22866	CNK2	0.00	-0.13	0.00	-0.23
22868	KIAA0971	0.00	0.00	0.00	NaN
22869	KIAA0972	0.00	0.00	-0.05	0.25
22870	KIAA1115	0.09	0.00	0.05	-0.11
22871	NLGN1	0.09	0.00	0.00	-0.01
22872	KIAA0905	0.00	0.00	0.00	NaN
22873	DZIP1	-0.09	0.13	-0.05	0.09
22874	PEPP3	0.00	0.13	0.00	0.11
22875	ENPP4	-0.09	0.00	-0.05	0.08
22877	MONDOA	0.00	-0.13	0.00	0.21
22878	KIAA1012	0.00	0.00	-0.10	0.55
22879	KIAA0872	0.00	0.13	0.00	0.01
22880	KIAA0852	0.00	0.00	0.00	NaN
22881	ANKRD6	0.00	0.00	0.00	NaN
22882	KIAA0854	0.18	0.25	0.05	0.44
22883	CLSTN1	0.00	0.00	0.00	NaN
22884	KIAA0982	0.00	0.00	0.00	NaN
22885	KIAA0843	0.00	0.00	0.00	NaN
22887	KIAA1041	0.00	0.00	0.00	NaN
22888	UBCE7IP5	0.00	0.00	0.00	NaN
22889	KIAA0907	0.09	0.13	0.00	0.32
22890	KIAA0997	0.09	0.00	0.00	0.38
22891	KIAA0844	0.00	0.00	0.00	NaN
22893	KIAA0945	0.00	0.00	0.00	NaN
22894	DIS3	-0.09	0.13	-0.15	0.51
22895	RPH3A	0.00	0.00	0.00	NaN
22897	KIAA1052	-0.09	0.00	0.00	0.25
22898	KIAA0870	0.18	0.00	0.05	0.31
22899	ARHGEF15	0.00	0.00	0.00	NaN
22900	TUCAN	0.09	0.00	0.00	0.10
22901	KIAA1001	0.18	0.13	0.00	-0.10
22902	KIAA0871	0.00	0.00	0.00	NaN

22903	BTBD3	0.00	0.13	0.00	-0.10
22904	KIAA0963	0.00	0.13	0.00	-0.13
22905	EPN2	0.00	0.00	0.00	NaN
22906	KIAA1042	0.00	0.00	-0.05	0.36
22907	DDX30	-0.09	0.00	-0.05	0.43
22908	SACM1L	-0.09	0.00	-0.05	0.42
22909	KIAA1018	0.00	0.00	0.00	NaN
22911	KIAA0893	0.09	0.00	0.05	0.51
22913	RALY	0.00	-0.13	0.00	0.32
22914	D12S2489E	-0.09	0.00	0.00	0.10
22915	MMRN	0.00	0.00	-0.05	-0.09
22916	NCBP2	0.18	0.00	0.00	0.62
22918	C1QR1	0.00	0.00	0.00	NaN
22919	MAPRE1	0.00	0.00	0.00	NaN
22920	KIFAP3	0.00	0.00	0.00	NaN
22921	PILB	-0.09	0.00	0.05	0.37
22924	MAPRE3	0.50	0.00	0.50	0.26
22925	PLA2R1	0.00	0.00	-0.10	0.07
22926	ATF6	0.00	0.00	0.00	NaN
22927	IHABP4	0.00	0.00	-0.05	-0.05
22928	SPS2	0.00	0.00	0.00	NaN
22929	SPS	0.00	0.00	0.00	NaN
22930	RAB3GAP	0.00	0.00	-0.05	0.19
22932	POMZP3	0.00	0.00	0.00	NaN
22933	SIRT2	0.00	0.00	0.00	NaN
22934	RPIA	0.00	0.00	-0.05	0.33
22936	ELL2	0.00	0.00	0.00	NaN
22938	SNW1	0.00	0.00	0.00	NaN
22941	SHANK2	0.36	0.13	0.00	0.41
22943	DKK1	0.00	0.00	0.00	NaN
22944	KIN	0.00	0.00	-0.05	0.12
22948	CCT5	0.00	0.00	0.10	0.35
22950	SLC4A1AP	0.09	0.00	0.05	0.56
22953	P2RX2	0.00	0.00	0.00	NaN
22954	TRIM32	0.00	0.00	0.00	NaN
22955	SCMH1	0.00	0.00	0.00	NaN
22974	C20orf1	0.00	0.00	0.05	0.30
22976	PAXIP1L	0.00	0.00	0.00	NaN
22977	AKR7A3	-0.09	0.00	0.00	-0.06
22978	NT5C2	0.00	-0.13	-0.05	0.19
22979	KIAA0953	0.09	0.00	0.00	0.15
22981	KIAA0980	0.00	0.00	-0.05	0.25
22982	KIAA0934	0.00	0.00	0.05	-0.29
22984	KIAA0185	0.00	-0.13	-0.05	0.30
22985	ACN	0.00	0.00	0.00	NaN
22986	SORCS3	0.00	-0.13	-0.05	-0.08
22987	KIAA1054	0.00	0.00	0.00	NaN

22989	KIAA1000	0.00	0.00	0.00	NaN
22990	KIAA0995	0.00	0.00	0.00	NaN
22992	FBXL11	0.45	0.00	0.05	0.60
22993	KIAA0194	0.00	0.00	0.00	NaN
22994	KIAA1118	0.00	0.00	0.00	NaN
22995	KIAA0912	0.00	0.00	0.00	NaN
22996	KIAA0452	0.00	0.00	0.00	NaN
22997	KIAA1030	-0.09	0.00	-0.15	-0.14
22998	KIAA1102	0.00	0.00	0.00	NaN
22999	RAB3IP2	0.00	0.00	0.05	0.04
23001	KIAA0993	0.00	0.00	0.00	NaN
23005	KIAA0596	0.00	0.00	0.00	NaN
23007	KIAA1069	0.00	0.00	0.00	NaN
23008	KIAA0265	0.00	0.25	0.05	0.44
23011	RAB21	0.09	0.00	0.00	0.31
23012	STK38L	0.00	0.00	0.00	NaN
23013	SHARP	0.00	0.00	0.00	NaN
23014	FBXO21	0.00	-0.13	0.00	0.22
23015	GOLGIN-67	-0.18	-0.13	-0.10	0.24
23016	KIAA0116	-0.09	0.00	-0.05	0.39
23017	LFG	0.00	0.00	0.00	NaN
23019	KIAA1007	0.00	0.00	0.00	NaN
23020	KIAA0788	0.00	0.00	0.00	NaN
23022	KIAA0992	0.00	0.00	-0.05	0.04
23023	KIAA0779	0.00	0.00	0.00	NaN
23024	KIAA1095	-0.09	0.00	-0.05	0.14
23025	KIAA1032	-0.09	0.00	0.00	0.26
23026	KIAA0865	-0.09	-0.13	0.00	0.02
23028	KIAA0601	-0.09	0.00	0.00	0.25
23029	KIAA0117	0.09	0.13	0.00	0.38
23030	KIAA0876	0.00	0.00	0.00	NaN
23031	KIAA0561	0.00	0.00	0.00	NaN
23032	VDU1	0.00	0.00	0.00	NaN
23033	KIAA1117	0.00	0.00	0.00	NaN
23034	KIAA1053	0.00	0.00	0.05	0.38
23035	KIAA0931	0.00	0.00	0.05	0.44
23036	KIAA0530	0.00	0.00	0.00	NaN
23037	PDZD2	0.00	0.00	0.15	0.02
23038	KIAA1037	-0.09	-0.13	0.00	-0.01
23039	RANBP16	-0.09	-0.25	-0.10	0.42
23040	MYT1L	0.00	0.00	0.00	NaN
23041	KIAA1040	0.00	0.00	0.00	NaN
23043	KIAA0551	0.09	0.00	0.00	0.62
23046	KIAA0449	0.00	0.00	0.00	NaN
23047	AS3	0.00	0.00	0.00	NaN
23048	FBP17	0.00	0.00	0.00	NaN
23049	SMG1	0.00	0.00	0.00	NaN

23051	TIX1	0.18	-0.13	0.00	0.29
23052	KIAA0830	-0.09	0.13	-0.05	0.46
23053	KIAA0913	0.09	0.00	0.00	0.34
23054	NCOA6	0.00	0.13	0.00	0.66
23057	C1orf15	0.00	0.00	0.00	NaN
23059	KIAA0643	0.00	0.00	0.00	NaN
23060	KIAA0295	0.00	0.00	-0.05	0.28
23061	KIAA0676	0.00	0.00	0.00	NaN
23062	GGA2	0.00	0.00	-0.05	0.33
23063	KIAA0261	0.09	0.00	-0.05	0.53
23064	KIAA0625	0.00	0.00	0.00	NaN
23065	KIAA0090	-0.09	0.00	0.00	0.29
23066	TIP120B	0.00	0.00	0.00	NaN
23067	KIAA1076	0.00	-0.13	0.00	0.12
23070	KIAA0082	0.00	0.00	0.00	NaN
23071	ERp44	0.00	0.00	0.00	NaN
23072	KIAA0322	0.00	0.00	0.00	NaN
23074	KIAA0701	0.00	0.00	0.00	NaN
23075	SWAP70	0.00	0.00	0.00	NaN
23076	KIAA0179	0.00	0.00	0.00	NaN
23077	KIAA0916	-0.09	0.13	-0.10	0.42
23078	KIAA0564	0.00	0.00	-0.05	0.21
23080	KIAA0241	0.00	0.13	0.00	0.10
23081	GASC1	-0.09	-0.13	-0.05	0.64
23082	KIAA0595	0.00	-0.13	-0.05	0.16
23085	ELKS	0.00	0.00	0.05	0.05
23086	SLAC2-B	0.00	0.00	0.00	NaN
23089	PEG10	0.50	0.00	0.00	-0.21
23090	OAZ	-0.09	0.00	-0.05	-0.13
23091	KIAA0853	0.00	0.00	-0.05	0.22
23092	GRAF	0.00	0.00	-0.05	0.27
23093	KIAA0998	0.00	0.00	0.00	NaN
23094	KIAA0545	0.00	0.13	0.00	0.56
23095	KIF1B	0.00	0.00	0.00	NaN
23097	KIAA1028	0.00	0.00	0.05	0.27
23098	SARM	0.00	0.25	0.00	0.21
23099	ZNF297B	0.09	0.00	-0.05	0.63
23101	KIAA0861	0.09	0.00	0.00	0.09
23102	KIAA1055	0.00	0.00	0.00	NaN
23105	KIAA1061	0.00	-0.13	0.00	-0.18
23107	MRPS27	0.00	0.00	0.00	NaN
23109	KIAA0749	0.00	0.00	0.00	NaN
23112	KIAA1093	-0.09	0.00	0.05	0.10
23113	KIAA0708	0.00	0.00	0.00	NaN
23114	NFASC	0.00	0.13	0.00	-0.18
23116	KIAA0423	0.00	0.00	0.00	NaN
23117	KIAA0220	0.00	0.00	-0.05	0.18

23118	MAP3K7IP2	0.00	0.00	0.00	NaN
23119	HIC2	0.00	0.00	0.00	NaN
23120	ATP10B	0.00	0.00	0.00	NaN
23122	CLASP2	0.00	0.00	0.00	NaN
23125	KIAA0909	0.00	0.00	0.00	NaN
23126	KIAA0461	0.00	0.00	0.05	0.49
23127	C1orf17	0.00	0.00	0.00	NaN
23129	KIAA0620	0.00	0.00	0.00	NaN
23130	KIAA0404	0.00	0.00	0.00	NaN
23131	KIAA0553	0.00	-0.13	0.00	0.38
23132	KIAA0809	-0.09	0.00	-0.10	0.48
23135	KIAA0346	0.00	0.00	0.00	NaN
23136	EPB41L3	0.00	0.00	0.00	NaN
23137	KIAA0594	0.00	0.00	0.00	NaN
23138	N4BP3	0.18	0.00	0.00	0.48
23139	MAST205	0.00	0.00	0.00	NaN
23140	KIAA0399	0.00	0.00	0.00	NaN
23141	KIAA0692	0.00	0.00	0.00	NaN
23142	KIAA0276	0.00	0.00	0.00	NaN
23143	KIAA1016	0.00	0.00	0.00	NaN
23144	KIAA0150	0.18	0.00	0.10	0.47
23145	KIAA0543	0.00	0.00	-0.05	0.27
23148	KIAA0363	0.00	0.00	0.00	NaN
23149	KIAA0290	-0.09	0.00	0.00	-0.12
23150	KIAA1013	0.00	0.00	0.00	0.08
23151	KIAA0767	0.00	-0.25	0.00	0.18
23152	CIC	0.09	0.00	-0.05	0.02
23154	KIAA0607	0.00	0.00	0.00	NaN
23155	MCLC	0.09	0.00	0.05	0.49
23158	KIAA0882	0.00	0.00	0.00	NaN
23160	KIAA0007	0.00	0.00	0.00	NaN
23161	SNX13	0.00	0.13	0.00	-0.09
23162	MAPK8IP3	0.00	0.00	0.00	NaN
23163	GGA3	0.00	0.13	0.00	0.46
23164	KIAA0864	0.00	0.00	0.00	NaN
23165	KIAA0225	0.00	0.00	0.00	NaN
23166	STAB1	-0.09	-0.25	-0.10	0.20
23167	KIAA0143	0.00	-0.25	0.10	0.66
23168	KIAA0252	0.00	0.00	0.00	NaN
23169	UGTREL7	0.00	0.00	0.00	NaN
23170	KIAA0153	0.00	0.00	0.05	0.34
23171	KIAA0089	0.00	0.00	0.00	NaN
23172	KIAA0157	0.00	0.00	-0.05	0.35
23173	METAP1	0.00	0.00	0.00	NaN
23174	BDG-29	-0.09	-0.13	-0.05	0.26
23175	LPIN1	0.00	0.00	0.00	NaN
23176	KIAA0202	0.00	-0.13	0.00	0.27

23177	KIAA0582	0.00	0.00	0.00	NaN
23178	PASK	0.00	0.00	-0.05	0.31
23179	RGL	0.00	0.00	0.00	NaN
23180	KIAA0084	0.00	0.00	0.05	-0.13
23181	KIAA0184	0.00	0.00	0.00	NaN
23185	KIAA0217	0.00	0.00	0.00	NaN
23186	RCOR	0.00	0.00	0.00	NaN
23187	KIAA0638	0.00	0.00	0.00	NaN
23189	KIAA0172	0.00	-0.25	-0.15	0.51
23190	UBXD2	0.00	0.00	-0.05	0.20
23191	CYFIP1	0.00	-0.13	0.05	0.28
23192	Apg4B	0.00	0.00	-0.05	0.35
23193	G2AN	0.00	0.00	0.00	NaN
23194	FBXL7	0.00	-0.25	0.15	-0.09
23195	MDN1	0.00	0.00	0.00	NaN
23196	C9orf10	0.00	0.00	0.05	0.05
23197	KIAA0887	0.00	0.00	0.00	NaN
23198	KIAA0077	0.09	0.00	0.00	0.02
23199	KIAA0182	0.00	0.00	-0.05	0.27
23200	ATP11B	0.09	0.00	0.00	-0.20
23201	KIAA0280	0.00	0.13	0.00	0.47
23203	INPP5E	0.00	0.00	0.00	NaN
23204	ARL6IP	0.00	0.00	0.00	NaN
23205	BG1	0.00	0.00	0.00	NaN
23207	KIAA0842	0.00	0.00	0.00	NaN
23208	KIAA0080	0.50	0.50	0.50	0.01
23210	PSR	0.00	0.13	0.00	0.51
23211	C19orf7	0.09	0.00	0.00	-0.12
23212	RRS1	0.00	0.00	0.05	0.35
23213	KIAA1077	0.00	0.00	0.00	NaN
23214	KIAA0370	0.00	0.00	0.00	NaN
23215	KIAA1096	0.00	0.00	0.00	NaN
23216	TBC1D1	0.00	0.00	0.00	NaN
23217	KIAA1086	0.00	0.00	0.00	NaN
23218	KIAA0540	-0.09	0.00	-0.05	0.29
23219	KIAA0483	0.09	0.00	0.00	0.40
23220	KIAA0937	0.00	0.00	0.00	NaN
23221	KIAA0717	-0.09	-0.25	-0.15	0.23
23224	SYNE-2	0.00	0.00	0.00	NaN
23225	KIAA0906	0.00	0.00	-0.05	0.24
23228	PLCL2	0.00	0.00	0.00	NaN
23230	CHAC	0.00	0.00	-0.05	0.31
23231	KIAA0746	0.00	0.00	-0.05	0.19
23232	KIAA0608	0.00	0.00	0.00	NaN
23233	SEC15B	0.00	0.00	0.00	NaN
23234	JDD1	0.09	0.00	0.00	0.45
23235	KIAA0781	0.00	0.00	0.00	NaN

23236	PLCB1	0.00	0.00	0.05	-0.08
23239	SCOP	-0.09	0.00	-0.15	0.36
23240	KIAA0922	0.00	0.00	-0.05	0.39
23241	KIAA0602	0.00	0.00	0.00	NaN
23242	KIAA0633	0.00	0.00	0.00	NaN
23243	KIAA0379	0.00	0.00	0.05	-0.02
23244	KIAA0648	0.00	0.00	0.00	NaN
23245	ASTN2	0.00	0.00	0.00	NaN
23246	BOP1	0.18	0.00	0.10	0.20
23247	KIAA0556	0.00	0.00	0.00	NaN
23250	ATP11A	-0.09	-0.13	-0.05	0.27
23253	KIAA0874	0.00	0.00	0.00	NaN
23255	KIAA0802	0.00	0.00	0.00	NaN
23256	RA410	0.00	0.00	0.00	NaN
23258	KIAA1091	0.00	0.00	0.00	NaN
23259	KIAA0725	0.45	-0.13	-0.05	0.54
23262	KIAA0433	0.00	0.00	0.00	NaN
23263	MCF2L	-0.09	-0.13	-0.05	0.14
23264	KIAA1031	0.00	0.00	0.05	0.26
23265	KIAA1067	0.00	0.50	0.00	0.38
23266	LPHH1	0.00	0.00	0.05	0.36
23268	KIAA1010	0.00	-0.13	-0.05	0.12
23271	KIAA1078	0.00	0.00	0.00	NaN
23272	RAP140	0.00	0.00	-0.10	0.23
23274	KIAA0350	0.00	0.00	0.00	NaN
23275	C21orf80	0.00	0.00	0.00	NaN
23276	KIAA0795	-0.09	0.00	-0.05	0.46
23277	KIAA0664	0.00	0.00	0.00	NaN
23279	NUP160	0.00	0.00	0.05	0.55
23281	KIAA0774	0.00	0.00	-0.05	-0.01
23283	CSTF2T	0.00	0.00	0.00	NaN
23284	LEC3	0.00	0.00	0.00	NaN
23285	KIAA1107	0.00	0.00	0.05	0.34
23286	KIAA0869	0.00	0.00	0.00	NaN
23287	AGTPBP1	0.00	0.00	0.00	NaN
23288	KIAA1023	0.00	0.13	0.10	0.22
23291	FBXW1B	0.00	0.00	0.00	NaN
23293	C17orf31	0.00	-0.13	0.00	0.10
23294	KIAA0229	0.00	0.00	0.00	NaN
23295	KIAA0544	0.00	0.00	-0.05	0.62
23299	BICD2	0.00	0.00	0.00	NaN
23300	KIAA0431	0.00	0.00	-0.05	0.29
23301	KIAA0903	0.00	0.00	0.00	NaN
23302	KIAA0523	0.00	0.00	0.00	NaN
23303	KIF13B	-0.09	-0.25	-0.05	0.11
23304	KIAA0349	0.00	0.00	0.00	NaN
23305	FACL6	0.00	-0.13	0.00	-0.28

23306	KIAA0286	0.00	0.00	0.00	NaN
23307	KIAA0674	0.00	0.00	0.00	NaN
23308	B7H2	0.00	0.00	0.00	NaN
23309	KIAA0700	0.00	0.00	0.00	NaN
23310	KIAA0056	-0.09	0.00	-0.15	0.28
23312	RC3	0.18	0.00	0.00	0.47
23313	KIAA0930	0.00	0.00	0.00	NaN
23314	KIAA1034	0.00	0.00	0.00	NaN
23316	KIAA0293	0.00	0.00	0.00	NaN
23317	KIAA0678	0.00	0.00	0.00	NaN
23318	KIAA0191	0.00	0.00	0.00	NaN
23321	TRIM2	0.00	0.00	-0.05	0.27
23322	KIAA1005	0.09	0.00	0.00	0.34
23325	KIAA1033	0.00	0.00	0.00	NaN
23326	USP22	0.00	-0.13	0.00	0.37
23327	NEDD4L	-0.09	0.00	-0.25	0.14
23328	KIAA0790	0.00	0.00	0.00	NaN
23331	KIAA1043	0.00	0.00	0.00	NaN
23332	CLASP1	0.00	0.00	0.00	NaN
23333	KIAA0877	0.00	0.00	0.00	NaN
23334	KIAA0467	0.00	0.00	0.00	NaN
23335	WDR7	-0.09	0.13	-0.20	0.33
23336	DMN	0.00	0.00	0.00	NaN
23338	KIAA0239	0.00	-0.13	0.00	0.34
23339	VPS39	0.00	0.00	0.00	NaN
23341	KIAA0962	0.00	0.00	0.00	NaN
23344	KIAA0747	0.00	0.00	0.00	NaN
23345	SYNE-1	0.00	0.00	0.00	NaN
23347	KIAA0650	0.00	0.00	-0.05	0.32
23348	KIAA1058	-0.09	-0.13	-0.05	0.17
23349	KIAA1045	-0.09	0.00	0.10	-0.06
23350	KIAA0332	0.00	0.00	-0.05	0.33
23351	KIAA0323	0.00	0.00	0.00	NaN
23352	RBAF600	-0.09	0.00	0.00	0.03
23353	KIAA0810	0.00	0.00	0.00	0.32
23354	KIAA0841	0.00	0.00	0.00	NaN
23355	KIAA0804	0.09	0.00	0.00	0.41
23357	KIAA0759	0.00	0.00	0.00	NaN
23358	USP24	0.00	0.00	0.00	NaN
23359	KIAA0574	0.00	-0.13	0.00	0.06
23360	KIAA1014	0.00	0.00	0.05	0.20
23361	KIAA0326	0.00	0.00	0.00	NaN
23362	KIAA0942	-0.09	-0.13	-0.05	0.18
23363	KIAA0657	0.00	0.00	0.00	NaN
23365	ARHGEF12	-0.09	0.00	-0.10	0.50
23366	KIAA0895	0.00	0.00	0.00	NaN
23367	KIAA0731	0.00	0.00	0.00	NaN

23369	PUM2	0.09	0.00	-0.05	0.54
23370	P114-RHO-GE	0.00	0.00	0.00	NaN
23371	KIAA1075	0.00	0.00	0.00	NaN
23373	KIAA0616	0.00	0.00	0.00	NaN
23378	KIAA0409	0.00	-0.13	0.00	0.39
23379	KIAA0947	0.00	0.00	0.05	0.26
23381	KIAA1089	0.00	0.13	0.00	0.54
23382	KIAA0828	0.00	0.00	0.00	NaN
23383	KIAA0892	0.00	0.00	0.00	NaN
23384	KIAA0376	0.00	0.00	0.00	NaN
23385	NCSTN	0.00	0.00	0.00	NaN
23386	KIAA1068	0.09	0.00	0.00	0.28
23387	KIAA0999	-0.09	0.00	0.00	0.28
23389	KIAA1025	0.00	-0.13	0.00	0.17
23390	HYPH	0.00	0.00	0.00	NaN
23392	KIAA0368	0.00	0.00	0.00	NaN
23394	ADNP	0.09	0.13	0.05	0.28
23395	LARS2	-0.09	0.00	-0.05	0.27
23396	PIP5K1C	0.00	0.00	0.00	NaN
23397	KIAA0074	0.00	0.00	0.00	NaN
23399	HSA011916	0.00	0.00	0.00	NaN
23400	HSA9947	-0.09	0.00	0.00	-0.03
23401	FRAT2	0.00	0.00	-0.05	0.18
23403	20D7-FC4	0.09	0.00	0.00	0.01
23404	RRP4	0.00	0.00	0.00	NaN
23405	DICER1	0.00	0.00	0.00	NaN
23408	SIRT5	0.00	0.00	0.10	0.59
23409	SIRT4	0.00	-0.13	0.00	-0.21
23410	SIRT3	-0.27	-0.13	-0.05	0.19
23411	SIRT1	0.00	0.00	0.00	NaN
23412	BUP	-0.09	0.00	0.05	0.18
23413	FREQ	0.00	0.00	0.00	NaN
23414	FOG2	0.18	-0.25	0.00	0.00
23415	KCNH4	0.00	0.13	0.00	-0.01
23417	MLYCD	-0.09	-0.13	-0.05	0.18
23418	CRB1	0.09	0.13	0.00	0.15
23420	PM5	0.00	0.00	0.00	NaN
23421	ITGB3BP	0.00	0.00	0.00	NaN
23423	P24B	0.00	0.00	0.00	NaN
23424	PCTAIRE2BP	0.00	0.00	0.00	NaN
23428	SLC7A8	0.00	0.00	0.00	NaN
23429	RYBP	-0.09	0.00	-0.05	0.43
23430	TPSD1	0.00	0.00	-0.05	-0.26
23431	AP4E1	0.18	0.00	0.00	0.01
23432	RE2	0.00	0.00	0.00	NaN
23433	TC10	0.00	0.00	0.00	NaN
23434	GR6	0.00	0.00	0.00	NaN

23435	TARDBP	0.00	0.00	0.00	NaN
23436	ELA3B	-0.09	0.00	0.05	0.00
23438	HARSL	0.00	0.00	0.00	NaN
23443	SLC35A3	0.00	0.00	0.00	NaN
23450	SF3B3	0.00	0.00	0.50	0.53
23451	SF3B1	0.00	0.00	0.00	NaN
23452	ANGPTL2	0.00	0.00	-0.05	0.03
23457	ABCB9	0.00	-0.13	0.00	-0.06
23460	ABCA6	0.18	0.13	0.00	0.20
23461	ABCA5	0.18	0.13	0.00	0.30
23462	HEY1	0.00	0.25	0.05	0.05
23463	ICMT	0.00	0.00	0.00	NaN
23464	GCAT	-0.09	0.00	0.05	0.52
23466	CBX6	-0.09	0.00	0.05	0.38
23468	CBX5	0.00	0.00	0.00	NaN
23469	PHF3	-0.09	0.00	0.00	0.24
23471	TRAM	0.00	0.00	0.00	NaN
23473	CAPN7	0.00	0.00	0.05	0.27
23474	YF13H12	0.09	0.00	-0.10	0.49
23475	QPRT	0.00	0.00	0.00	NaN
23476	BRD4	0.00	0.00	0.05	0.36
23478	SPC18	0.00	0.00	-0.05	0.56
23479	NIFU	0.00	-0.13	0.00	0.50
23480	SEC61G	0.00	0.00	0.10	0.78
23481	PES1	0.00	0.00	0.00	NaN
23483	TDPGD	0.00	0.13	-0.05	-0.10
23484	LEPROTL1	-0.09	-0.25	-0.05	0.29
23491	CES3	-0.09	-0.13	0.00	-0.18
23493	HEY2	0.00	0.00	0.00	NaN
23495	TACI	0.00	0.00	0.00	NaN
23498	HAAO	0.00	0.00	0.00	NaN
23499	MACF1	0.00	0.00	0.00	NaN
23500	DAAM2	0.00	0.00	0.00	NaN
23503	KIAA0321	0.00	0.00	0.00	NaN
23504	KIAA0318	0.00	0.00	0.00	NaN
23505	KIAA0257	0.00	0.00	0.00	NaN
23506	KIAA0240	0.00	0.00	0.00	NaN
23507	KIAA0231	0.00	0.00	0.00	NaN
23508	KIAA0227	0.00	0.00	0.00	NaN
23509	POFUT1	0.00	0.00	0.05	0.38
23510	KIAA0176	0.00	0.13	0.00	0.34
23512	JJAZ1	0.00	0.00	0.00	NaN
23513	SCRIB	0.18	0.00	0.10	0.49
23514	KIAA0146	0.00	0.00	0.00	NaN
23516	KIAA0062	-0.09	-0.25	-0.10	0.29
23517	KIAA0052	0.00	0.00	-0.05	0.41
23518	R3HDM	0.00	0.00	-0.05	0.38

23519	ANP32D	0.00	0.00	0.00	NaN
23520	ANP32C	0.00	0.00	0.00	NaN
23521	RPL13A	0.00	0.00	0.00	NaN
23522	MORF	0.09	0.00	0.00	0.48
23523	CABIN1	0.00	0.13	0.00	0.38
23524	SRRM2	0.00	0.13	0.00	0.39
23526	HA-1	0.00	0.00	0.00	NaN
23527	CENTB2	0.09	0.00	0.00	0.47
23528	ZNF281	0.09	0.00	0.00	-0.14
23529	CLC	0.45	0.00	0.05	0.07
23530	NNT	0.00	0.00	0.05	0.14
23531	MMD	0.00	0.00	0.00	0.25
23532	PRAME	0.09	0.00	0.00	-0.08
23533	P101-PI3K	0.00	0.00	0.00	NaN
23534	TRN-SR	0.00	0.00	0.00	NaN
23536	ADAT1	0.00	-0.13	0.05	0.38
23538	OR52A1	0.00	-0.13	0.00	0.13
23539	SLC16A8	-0.09	0.00	0.05	0.28
23541	SEC14L2	0.00	0.00	0.00	NaN
23542	MAPK8IP2	0.00	-0.25	0.00	-0.08
23543	RBM9	0.00	0.00	0.15	0.18
23544	SEZ6L	0.00	0.13	0.00	0.45
23545	ATP6V0A2	0.00	0.00	0.00	NaN
23546	SYNGR4	0.09	0.00	0.00	0.59
23547	ILT7	0.09	0.00	0.00	0.06
23548	OSRF	0.00	0.00	0.10	0.34
23549	DNPEP	0.00	0.00	0.00	NaN
23550	TIC	0.00	0.00	0.00	NaN
23552	CCRK	0.00	0.00	0.00	NaN
23553	HYAL4	0.00	0.13	0.00	0.23
23554	NET-2	0.00	0.13	0.00	-0.16
23555	NET-7	0.00	0.00	0.00	NaN
23556	PIGN	-0.09	0.00	-0.10	0.39
23558	WBP2	0.00	0.13	0.00	0.21
23560	CRFG	0.00	0.00	0.00	NaN
23562	CLDN14	0.00	0.00	0.00	NaN
23563	CHST5	1.00	0.00	1.00	-0.16
23564	DDAH2	0.00	0.00	0.00	NaN
23566	EDG7	0.00	0.00	0.05	-0.06
23568	BART1	-0.09	0.00	0.00	0.29
23569	PADI5	-0.09	0.00	0.00	-0.28
23580	CEP4	0.00	0.00	0.00	NaN
23583	SMUG1	0.00	0.00	0.00	NaN
23585	SMP1	-0.09	0.00	0.00	-0.05
23586	RIG-I	-0.09	0.00	0.00	0.41
23587	HSPC002	0.00	0.00	0.00	NaN
23588	LCP	0.00	0.13	0.00	0.23

23589	CRHSP-24	0.00	0.00	0.00	NaN
23590	TPT	-0.09	0.00	0.00	0.16
23591	4/2/2010	0.00	-0.13	0.00	0.00
23592	MAN1	0.09	0.00	0.00	0.44
23593	C6orf34	0.00	0.00	0.00	NaN
23594	ORC6L	-0.09	0.00	-0.05	0.02
23595	ORC3L	0.00	0.00	0.00	NaN
23596	OPN3	0.00	0.00	0.00	NaN
23598	ZNF278	0.00	0.00	0.00	NaN
23600	AMACR	0.00	0.00	0.10	0.06
23601	CLECSF5	0.00	0.00	0.00	NaN
23603	CORO1C	0.00	-0.13	0.00	0.36
23604	DAPK2	0.00	0.00	0.00	NaN
23607	CD2AP	0.00	0.00	0.00	NaN
23608	MKRN1	0.00	0.00	0.00	NaN
23609	MKRN2	0.00	0.00	0.00	NaN
23612	PHLDA3	0.00	0.00	0.00	NaN
23613	PRKCBP1	0.18	0.00	0.00	0.38
23614	PPY2	0.00	0.25	0.00	0.40
23615	PYY2	0.00	0.13	0.00	0.15
23616	SH3BP1	-0.09	0.00	0.05	-0.12
23617	STK22B	-0.09	0.00	0.00	0.18
23619	ZIM2	0.09	0.13	0.05	0.13
23620	NTSR2	0.00	0.00	0.00	NaN
23621	BACE	-0.09	0.00	0.00	0.15
23623	NESCA	0.09	0.13	0.00	0.46
23624	CBLC	0.09	0.00	0.00	0.01
23625	MTVR	-0.09	0.00	0.05	0.16
23629	BP75	-0.09	0.00	0.05	0.07
23632	CA14	0.00	0.13	0.05	-0.03
23633	KPNA6	0.50	0.00	0.00	-0.16
23635	SSBP2	0.00	0.00	0.00	NaN
23636	NUP62	0.00	0.00	-0.05	0.14
23637	GAPCENA	0.00	0.00	0.00	NaN
23639	TSLRP	0.00	0.00	0.05	-0.06
23640	HSPBP1	0.09	0.00	0.00	0.20
23641	LDOC1	0.00	0.00	-0.05	0.07
23643	MD-2	0.00	0.25	0.00	-0.06
23644	RCD-8	-0.09	-0.13	0.00	0.44
23645	PPP1R15A	0.09	0.00	0.00	-0.07
23646	HU-K4	0.00	0.00	0.00	NaN
23647	POR1	0.00	-0.13	0.00	-0.02
23649	POLA2	-0.09	0.00	0.00	0.19
23650	TRIM29	-0.09	0.00	-0.10	0.27
23654	PLXNB2	0.00	-0.25	0.05	-0.11
23657	SLC7A11	0.00	0.00	0.00	NaN
23658	LSM5	0.00	0.13	0.00	-0.25

23659	LYPLA3	-0.09	-0.13	0.00	0.10
23660	ZFP95	0.18	0.00	0.00	0.56
23670	TMEM2	0.00	0.00	-0.05	-0.18
23673	STX12	0.00	0.00	0.00	NaN
23677	SH3BP4	0.00	0.00	0.00	NaN
23678	SGKL	0.00	0.00	0.00	NaN
23682	RAB38	0.00	0.00	0.00	NaN
23683	PRKCN	0.00	0.00	0.00	NaN
23705	IGSF4	-0.09	0.00	0.00	0.12
23710	GABARAPL1	-0.09	0.00	0.00	0.09
23729	CARKL	0.00	0.00	0.00	NaN
23741	CRI1	0.00	0.00	0.00	NaN
23742	C15orf2	0.00	0.00	0.00	NaN
23743	BHMT2	0.00	0.00	0.00	NaN
23746	AIPL1	0.00	0.00	0.00	NaN
23753	SDF2L1	0.00	0.00	0.00	NaN
23759	PPIL2	0.00	0.00	0.00	NaN
23762	OSBP2	0.00	0.00	0.00	NaN
23764	MAFF	-0.09	0.00	0.05	0.57
23765	IL17R	0.00	0.00	0.05	0.40
23767	FLRT3	0.00	0.00	0.00	NaN
23768	FLRT2	0.00	0.13	0.00	-0.03
23769	FLRT1	0.00	0.00	0.00	NaN
23770	FKBP8	0.00	0.00	0.00	NaN
23774	BRD1	0.00	-0.13	0.00	0.20
23779	ARHGAP8	0.00	0.00	0.00	NaN
23780	APOL2	0.00	0.00	0.00	NaN
23786	MIL1	0.00	0.00	0.00	0.53
23787	MTCH1	0.00	0.00	0.00	NaN
23788	MTCH2	0.00	0.00	0.00	NaN
24138	RI58	0.09	0.00	0.00	-0.08
24139	EML2	0.09	0.00	0.00	0.39
24141	C20orf103	0.00	0.00	0.00	NaN
24144	TIP39	0.00	0.13	0.00	0.77
24145	PANX1	0.00	0.00	-0.10	0.11
24146	CLDN15	0.18	0.00	0.00	0.04
24147	FJX1	0.00	0.13	0.05	0.42
24148	C20orf14	0.00	0.00	0.00	NaN
24149	HRIHFB2436	0.00	0.00	0.00	NaN
24150	TP53TG3	0.00	0.13	-0.10	0.02
25758	G2	0.00	0.00	0.05	0.03
25759	SLI	0.00	0.00	-0.05	0.08
25764	HYPK	0.00	0.00	0.00	NaN
25769	SLC24A2	0.00	-0.25	-0.10	0.08
25770	HS747E2A	-0.09	0.00	0.00	-0.03
25771	C22orf4	0.00	-0.25	0.00	0.27
25774	HS322B1A	0.18	0.25	0.40	-0.20

25775	HSN44A4A	0.00	0.00	0.00	NaN
25776	C22orf2	-0.09	0.00	0.05	0.17
25778	KIAA0472	0.00	0.13	0.00	0.48
25780	GRP3	0.00	0.00	0.00	NaN
25782	RAB3-GAP150	0.09	0.00	0.05	0.62
25787	DGS-A	-0.09	0.00	-0.05	-0.24
25788	RAD54B	0.00	-0.13	0.00	0.47
25789	BSMAP	0.00	0.00	0.00	NaN
25790	NESG1	0.00	0.00	0.00	NaN
25792	CIZ1	-0.09	0.00	0.00	0.26
25793	FBXO7	0.00	0.00	0.00	NaN
25794	FSCN2	0.00	0.00	0.00	NaN
25796	PGLS	-0.09	0.00	0.00	0.13
25797	QPCT	0.00	0.00	0.00	NaN
25799	ZF5128	0.00	0.13	0.05	0.79
25800	LIV-1	0.00	-0.13	-0.25	0.43
25801	GCA	0.00	0.00	0.00	NaN
25802	LMOD1	0.00	0.00	0.00	NaN
25803	PDEF	0.00	0.00	0.00	NaN
25804	LSM4	0.00	0.00	0.00	NaN
25805	NMA	-0.09	0.00	0.05	-0.03
25806	VAX2	0.00	0.00	0.05	0.41
25807	C22orf3	-0.09	0.00	0.00	0.11
25809	TTLL1	0.00	0.00	0.00	NaN
25813	CGI-51	0.00	0.00	0.05	0.25
25814	E46L	-0.09	0.00	0.00	0.32
25816	GG2-1	0.00	0.00	0.00	NaN
25819	CCRN4L	0.00	0.00	0.00	NaN
25820	ARIH1	0.00	0.00	0.00	NaN
25821	MTO1	0.00	0.00	0.05	0.29
25822	DNAJB5	-0.09	0.00	0.10	-0.09
25823	TPSG1	0.00	0.00	-0.05	0.05
25825	BACE2	0.00	0.00	0.00	NaN
25827	FBXL2	0.00	0.00	0.00	NaN
25828	TXN2	-0.09	0.00	0.05	0.48
25829	C22orf5	-0.09	0.00	0.05	0.17
25830	SULT4A1	0.00	0.00	0.05	-0.04
25832	DJ328E19.C1.1	0.00	0.00	0.05	0.53
25833	10/11/2010	-0.09	0.00	-0.10	0.19
25834	HGNT-IV-H	0.00	0.13	0.00	0.01
25836	IDN3	0.00	0.00	0.05	0.57
25837	RAB26	0.00	0.00	0.00	NaN
25839	COG4	-0.09	-0.13	0.05	0.29
25840	DKFZP586A05	0.00	0.00	0.00	NaN
25841	DKFZP586C16	0.00	0.00	0.05	-0.15
25842	DKFZP547E21	-0.09	0.00	0.05	0.65
25843	PREI3	0.00	0.00	0.00	NaN

25844	DKFZP566C24	0.00	0.00	0.00	NaN
25847	DKFZP566D19	0.00	0.00	0.00	NaN
25849	DKFZP564O08	0.00	0.00	0.05	-0.01
25850	ZNF345	0.00	0.00	0.00	NaN
25852	DKFZP434A04	0.00	0.00	0.00	NaN
25854	DKFZP564J102	0.00	0.00	0.00	0.36
25855	BRMS1	0.00	0.00	0.05	0.20
25859	PART1	0.00	-0.13	-0.10	0.05
25861	DKFZP434N01	0.00	0.00	0.00	NaN
25862	DKFZP586D22	0.00	0.00	0.05	0.13
25864	DKFZP564O24	-0.09	0.00	-0.10	0.06
25865	PKD2	0.09	0.00	0.00	0.10
25870	DKFZP566I102	0.00	0.00	0.10	0.07
25873	RPL36	0.00	0.00	0.00	NaN
25874	DKFZP564B16	0.00	0.00	0.00	NaN
25875	DKFZP586A01	0.00	0.00	0.00	NaN
25879	DKFZP564O04	0.18	0.00	0.00	-0.10
25880	DKFZP564K20	0.00	0.00	0.00	NaN
25888	ZFP100	0.00	0.00	0.00	NaN
25891	DKFZP586H21	0.00	0.13	0.05	-0.07
25893	DKFZP434C09	0.00	0.13	0.00	-0.15
25895	DKFZP586D09	0.00	0.00	0.00	NaN
25896	DKFZP434B16	0.00	0.00	0.00	NaN
25897	DORFIN	0.09	0.00	0.10	0.11
25898	ZNF363	-0.09	0.00	0.00	0.02
25900	DKFZP586I222	-0.09	0.00	0.00	-0.04
25901	DKFZP586D06	0.00	0.00	0.00	NaN
25903	DKFZP586L151	0.00	0.00	0.00	NaN
25906	DKFZP564M08	0.00	0.00	0.00	NaN
25907	RIS1	-0.09	0.00	-0.05	-0.07
25909	DKFZP434N09	0.09	0.00	0.00	-0.16
25913	POT1	0.00	0.13	0.00	-0.11
25915	DKFZP564J012	-0.09	0.00	-0.05	0.40
25920	COBRA1	0.00	0.00	0.00	NaN
25924	DKFZP586F10	0.00	0.00	-0.05	0.13
25926	DKFZP586L07	0.18	0.13	0.00	0.51
25928	DKFZP564D20	0.00	0.13	0.00	-0.05
25932	CLIC4	-0.09	0.00	0.00	0.26
25936	DC8	0.00	0.00	0.00	NaN
25937	TAZ	0.00	0.00	0.05	0.35
25939	SAMHD1	0.00	-0.13	0.00	-0.03
25940	DKFZP564F05	0.00	0.00	0.00	NaN
25941	DKFZP586M15	0.00	-0.13	-0.25	0.44
25943	H-L(3)MBT	0.00	0.00	0.00	NaN
25945	DKFZP566B08	0.00	0.00	0.00	NaN
25949	P29	-0.09	0.00	0.00	0.04
25950	DKFZP566K02	0.09	0.00	0.05	0.63

25956	SEC31B-1	0.00	-0.13	-0.05	-0.13
25957	DKFZp564B07	0.00	0.00	0.00	NaN
25959	DKFZP434N16	0.00	0.00	0.00	NaN
25960	TEM5	0.55	-0.13	0.00	0.35
25961	DKFZP586P22	0.09	0.00	0.00	0.41
25963	DKFZP564G20	0.00	0.00	0.00	NaN
25970	SH2B	0.00	0.00	0.00	NaN
25972	DKFZP564G02	0.00	0.00	0.00	NaN
25974	DKFZP564I122	0.00	0.00	0.00	NaN
25976	DKFZP434J214	0.00	0.00	0.00	NaN
25977	DKFZP566B18	-0.09	0.00	0.00	0.29
25978	DKFZP564O12	-0.09	0.00	0.05	0.29
25979	DKFZp566O08	0.00	-0.13	0.00	-0.13
25980	C20orf4	0.00	0.00	0.05	0.14
25983	DKFZP564O09	0.00	0.00	0.00	NaN
25984	HAIK1	0.00	0.25	0.00	0.00
25987	E2IG4	0.27	0.00	0.00	0.73
25988	MIZF	0.00	0.00	-0.05	0.30
25992	DKFZP586B24	0.00	0.00	-0.05	0.18
25994	DKFZP564K24	0.00	0.00	-0.05	0.21
25996	DKFZP566E144	-0.09	0.00	0.00	0.13
25998	IBTK	0.00	0.00	0.00	NaN
25999	CLIPR-59	0.00	0.00	0.00	NaN
26000	DKFZP434P17	0.00	0.00	0.00	NaN
26001	DKFZP566H07	0.00	0.00	0.00	NaN
26002	DKFZP564G20	0.00	0.00	0.00	NaN
26003	GRASP55	0.00	0.00	0.00	NaN
26005	DKFZP586P01	0.18	0.13	0.00	0.65
26007	DKFZP586B16	-0.09	0.00	0.00	0.13
26009	DKFZP564I052	0.00	0.00	0.00	NaN
26011	DKFZP564O04	0.27	0.00	0.00	0.50
26012	DKFZP586J162	0.00	0.00	0.05	0.45
26015	DKFZP727M11	0.00	0.00	0.00	NaN
26017	DKFZP586O01	0.00	0.00	0.05	0.51
26018	LIG1	-0.09	0.00	0.00	0.08
26019	RENT2	0.00	0.00	0.00	NaN
26024	KIAA0632	0.18	0.00	0.00	0.38
26027	THEA	0.00	0.00	0.00	NaN
26030	KIAA0599	0.00	0.00	0.00	NaN
26031	OSBPL3	0.00	0.13	0.00	-0.09
26032	KIAA0527	0.00	0.00	0.00	NaN
26033	KIAA0534	0.00	0.00	0.00	NaN
26034	PIP3-E	0.00	0.00	0.00	NaN
26035	KIAA0836	0.00	0.00	0.00	NaN
26036	COASTER	0.00	0.13	0.00	-0.20
26037	KIAA0440	0.00	0.00	0.00	NaN
26039	SS18L1	0.09	0.13	0.00	0.36

26040	SETBP1	-0.09	-0.25	-0.05	0.19
26043	KIAA0794	0.18	0.00	0.00	0.49
26045	KIAA0416	0.00	0.00	-0.10	0.08
26047	CNTNAP2	0.00	0.00	0.00	NaN
26048	KIAA0557	0.00	0.00	-0.05	0.39
26049	KIAA0888	0.00	0.00	0.00	NaN
26050	KIAA0918	0.00	0.13	-0.05	-0.02
26051	PPP1R16B	0.00	-0.13	0.00	0.07
26052	KIAA0820	0.00	0.00	0.00	NaN
26054	SUSP1	0.00	0.00	0.05	-0.28
26056	KIAA0857	0.00	0.00	0.00	NaN
26057	KIAA0697	0.00	0.00	0.00	NaN
26058	TNRC15	0.00	0.00	0.00	NaN
26059	KIAA0378	0.00	0.00	-0.10	-0.01
26060	APPL	0.00	0.00	-0.10	0.41
26063	DECR2	0.00	0.00	-0.05	0.09
26064	RAI14	0.00	0.00	0.15	0.44
26065	DKFZP434D13	0.00	0.00	0.05	0.30
26073	DKFZP586F15	0.50	0.50	0.50	0.51
26082	DKFZP434L18	0.00	-0.13	-0.15	0.23
26083	DKFZP434O04	0.00	0.25	0.00	0.11
26084	DKFZP434D14	0.00	0.00	0.00	NaN
26085	KLK13	0.00	0.00	0.00	NaN
26088	GGA1	-0.09	0.00	0.05	0.34
26091	DKFZP564G09	0.00	0.00	0.00	NaN
26092	DKFZP586G01	0.00	0.00	0.00	NaN
26093	DKFZP586M1(	0.09	0.00	0.00	0.09
26094	DKFZp434K11	0.00	0.00	0.00	NaN
26095	DKFZP566K05	0.00	0.00	0.00	NaN
26097	DKFZP547E10	0.09	0.13	0.00	0.18
26098	DKFZp586F10	0.00	0.00	-0.05	0.15
26099	DKFZP566C04	0.00	0.00	0.00	NaN
26100	DKFZP434J154	0.00	0.00	0.10	0.52
26102	DKFZP434A06	0.00	0.00	0.00	NaN
26103	PAL	0.09	0.00	-0.05	0.14
26108	PYGO1	0.00	0.00	0.05	0.26
26112	DKFZP434C17	0.00	0.00	0.00	NaN
26115	DKFZP564D16	0.27	0.13	0.00	0.70
26118	WSB1	0.00	0.13	0.00	0.40
26119	ARH	-0.09	0.00	0.00	-0.01
26121	PRPF31	0.09	0.00	0.00	0.35
26123	DKFZP564D11	0.00	0.00	-0.05	0.38
26128	DKFZP586B09	0.00	0.00	0.00	NaN
26130	DKFZP434C21	0.00	0.00	0.00	NaN
26133	C20orf188	0.00	0.13	0.00	0.62
26135	PAI-RBP1	0.00	0.00	0.00	NaN
26136	TES	0.00	0.25	0.00	0.38

26137	ZNF288	0.00	0.00	0.00	NaN
26138	DKFZP434F12	0.00	0.00	0.00	NaN
26140	DKFZP434B10	0.00	0.00	0.00	NaN
26145	DKFZP434M15	0.09	0.00	0.00	0.13
26146	MIP-T3	0.00	0.00	-0.05	0.12
26148	DKFZP564P19	0.00	0.00	-0.05	0.23
26150	DKFZP566F05	0.00	0.00	0.00	NaN
26151	DKFZP564C10	0.00	0.13	0.00	0.48
26152	ZNF337	0.00	0.13	-0.05	0.24
26154	ABCA12	0.00	0.00	0.00	NaN
26155	DKFZP564C18	0.00	0.00	0.00	NaN
26165	DKFZP434B20	-0.18	0.00	0.00	0.01
26168	SENP3	0.00	0.00	0.00	NaN
26173	DKFZP586J06	0.00	0.00	0.05	0.29
26175	DKFZP564F11	0.00	0.00	0.00	NaN
26189	OR1A2	0.00	0.00	0.00	NaN
26190	FBXW2	0.00	0.00	0.00	NaN
26191	PTPN22	0.27	0.13	0.05	-0.05
26205	GMEB2	0.09	0.00	0.00	0.11
26206	SPAG8	-0.09	0.00	0.10	0.01
26207	RDGBB	0.18	0.13	0.00	0.29
26211	OR2F1	0.00	0.00	0.00	NaN
26220	DGCR5	-0.09	0.00	-0.05	0.02
26225	ARL5	0.00	0.00	0.00	NaN
26227	PHGDH	0.09	0.13	0.05	0.09
26229	B3GAT3	0.00	0.00	0.00	NaN
26230	TIAM2	0.00	0.00	0.00	NaN
26232	FBXO2	0.00	0.00	0.00	NaN
26233	FBXL6	0.18	0.00	0.15	0.16
26234	FBXL5	0.00	0.00	-0.10	0.29
26235	FBXL4	0.00	0.00	0.00	NaN
26238	HGC6.2	0.00	0.00	0.00	NaN
26239	XP5	0.00	0.00	0.00	NaN
26240	D6S2654E	0.00	0.00	0.00	NaN
26249	KLHL3	0.00	-0.13	0.00	-0.01
26251	KCNG2	0.09	0.00	-0.25	0.15
26253	CLECSF9	-0.09	0.00	0.00	-0.02
26255	PTTG3	0.09	0.25	0.05	0.06
26256	CABYR	0.00	0.00	-0.15	0.37
26257	NKX2.8	0.00	0.00	0.00	NaN
26261	FBXO24	0.18	0.00	0.00	0.22
26263	FBXO22	0.00	0.00	0.00	NaN
26266	SLC13A4	0.00	0.00	0.00	NaN
26268	FBXO9	0.00	0.00	0.00	NaN
26271	FBXO5	0.00	0.00	0.00	NaN
26273	FBXO3	0.00	0.00	0.05	0.49
26275	HIBCH	0.00	0.00	0.00	NaN

26276	VPS33B	0.00	0.00	0.00	NaN
26277	TINF2	0.00	0.00	0.00	NaN
26278	SACS	0.00	0.00	-0.05	-0.01
26279	PLA2G2D	0.00	0.00	0.00	NaN
26281	FGF20	0.09	-0.13	-0.05	0.08
26284	ERAL1	0.00	0.38	0.00	0.70
26285	CLDN17	0.00	0.00	0.00	NaN
26286	ARFGAP1	0.50	0.00	0.00	0.29
26287	ANKRD2	0.00	0.00	-0.05	-0.05
26289	AK5	0.00	0.00	0.00	NaN
26290	GALNT8	-0.09	0.00	0.05	-0.02
26291	FGF21	0.09	0.00	0.00	-0.04
26292	MYCBP	0.00	0.00	0.00	NaN
26297	DELGEF	0.00	0.00	0.00	NaN
26298	EHF	0.00	0.13	0.05	0.19
26330	GAPDS	0.00	0.00	0.00	NaN
26333	OR7A17	0.00	0.00	0.00	NaN
26353	H11	0.00	-0.13	0.00	0.13
26354	E2IG3	-0.09	0.00	-0.10	0.49
26355	E2IG5	0.00	0.00	0.00	NaN
26468	LHX6	0.00	0.00	0.00	NaN
26469	PTPN18	0.00	0.00	0.00	0.35
26470	PSK-1	0.00	0.00	0.00	NaN
26471	P8	0.00	0.00	0.00	NaN
26472	PPP1R14B	0.00	0.00	0.00	NaN
26476	OR10J1	0.00	0.00	0.00	NaN
26499	PLEK2	0.00	0.00	0.05	0.16
26502	NARF	0.00	0.00	0.00	NaN
26503	SLC17A5	0.00	0.00	0.05	0.14
26504	CNNM4	0.00	0.00	0.00	NaN
26505	CNNM3	0.00	0.00	0.00	NaN
26507	CNNM1	0.00	-0.13	-0.05	0.10
26508	HEYL	0.00	0.00	0.00	NaN
26509	FER1L3	0.00	0.00	0.00	NaN
26511	CHIC2	0.00	0.00	0.00	NaN
26512	DDX26	0.00	0.00	0.00	NaN
26515	FXC1	0.00	-0.13	0.00	0.32
26517	TIMM13	0.00	0.00	0.00	NaN
26519	TIMM10	0.00	0.00	0.00	NaN
26520	TIMM9	0.00	0.00	0.05	-0.02
26521	TIMM8B	0.00	0.00	0.00	NaN
26523	EIF2C1	0.00	0.00	0.00	NaN
26528	DAZAP1	0.00	0.00	0.00	NaN
26529	OR12D2	0.00	0.00	0.00	NaN
26531	OR11A1	0.00	0.00	0.00	NaN
26532	OR10H3	0.00	0.00	0.00	NaN
26538	OR10H2	0.00	0.00	0.00	NaN

26539	OR10H1	0.00	0.00	0.00	NaN
26574	DED	0.00	-0.13	0.00	0.16
26575	RGS17	0.00	0.00	0.00	NaN
26577	PCOLCE2	0.00	0.00	-0.05	0.08
26578	OSTF1	0.00	0.00	-0.05	-0.12
26585	CKTSF1B1	0.00	0.00	0.00	NaN
26586	CKAP2	0.00	0.00	0.00	NaN
26589	MRPL46	0.00	0.00	-0.05	0.30
26608	TBL2	-0.09	0.00	0.00	0.37
26648	OR7E24P	0.00	0.13	0.00	0.01
26658	OR7C2	0.00	0.00	0.00	NaN
26659	OR7C1	0.00	0.00	0.00	NaN
26692	OR2W1	0.00	0.00	0.00	NaN
26707	OR2J2	0.00	0.00	0.00	NaN
26747	NUFIP1	0.00	0.00	-0.05	0.32
26750	RPS6KC1	0.00	0.00	0.00	NaN
26751	DKFZP586F13	0.00	0.00	0.00	NaN
26762	HAVCR-1	0.00	0.00	0.00	NaN
26872	STEAP	0.09	0.00	0.00	0.23
26873	OPLAH	0.18	0.00	0.10	0.39
26952	PBI	0.00	0.00	0.00	NaN
26953	RANBP6	-0.09	-0.13	-0.05	0.70
26959	HBP1	0.09	0.00	0.00	-0.15
26960	NBEA	0.00	0.00	0.00	NaN
26973	CHORDC1	0.00	0.00	0.00	NaN
26984	SEC22A	0.00	0.00	0.00	NaN
26986	PABPC1	0.18	0.00	0.00	0.27
26993	NAKAP95	0.00	0.00	0.00	NaN
26994	RNF11	0.00	0.00	0.00	NaN
26998	FETUB	0.09	0.00	0.05	0.13
26999	CYFIP2	0.00	0.00	0.00	NaN
27000	ZRF1	0.09	0.00	0.00	0.52
27004	TCL6	0.00	0.00	0.00	NaN
27005	USP21	0.00	0.00	0.00	NaN
27006	FGF22	0.00	0.00	-0.05	0.02
27010	TPK1	0.00	0.00	0.00	NaN
27012	KV8.1	0.18	0.13	0.10	0.37
27013	CGI-57	0.00	0.00	0.00	NaN
27019	DNAI1	-0.09	0.13	0.10	0.02
27020	SDFR1	0.00	0.00	0.00	NaN
27022	FOXD3	0.00	0.00	0.00	NaN
27023	FOXB1	0.09	0.00	0.05	-0.25
27030	MLH3	0.00	0.00	0.00	NaN
27032	ATP2C1	0.00	0.00	0.00	NaN
27033	TZFP	0.00	0.00	0.00	NaN
27034	ACAD8	-0.09	0.00	-0.15	0.52
27036	SIGLEC7	0.00	0.13	0.00	0.45

27037	HTF9C	-0.09	0.00	0.00	-0.07
27039	PKD2L2	0.00	-0.13	0.00	-0.01
27042	DJ434O14.5	0.00	0.00	0.00	NaN
27043	PELP1	0.50	0.00	0.00	-0.14
27044	p100	0.00	0.25	0.00	0.75
27063	CARP	0.09	0.00	0.00	-0.03
27065	D4S234E	0.00	-0.13	-0.15	0.34
27067	STAU2	0.00	0.25	0.00	0.23
27068	SID6-306	0.00	0.00	0.00	NaN
27069	GHITM	0.09	0.00	-0.05	0.39
27071	DAPP1	0.00	0.00	0.00	NaN
27072	VPS41	0.18	0.00	0.00	0.32
27074	LAMP3	0.09	0.00	0.00	-0.02
27075	NET-6	0.00	0.13	0.00	0.15
27076	C4.4A	0.09	0.00	-0.10	0.33
27077	B9	0.00	0.00	0.00	NaN
27087	B3GAT1	-0.09	0.00	-0.10	0.26
27089	QP-C	0.00	-0.13	0.00	0.10
27090	SIAT7D	-0.09	0.00	-0.05	0.19
27091	CACNG5	0.18	0.13	0.00	-0.11
27094	KCNMB3	0.09	0.00	0.05	0.35
27095	BET3	0.00	0.00	0.00	NaN
27097	TAF5L	0.09	0.00	0.00	0.21
27098	CLUL1	0.00	0.00	0.00	NaN
27099	NSG-X	0.00	0.25	0.00	0.77
27101	SIP	0.00	0.00	0.05	0.29
27107	ZNF-U69274	0.00	0.00	0.00	NaN
27109	ATPW	0.00	0.00	0.00	NaN
27113	BBC3	0.09	0.00	0.00	-0.02
27115	PDE7B	0.00	0.00	0.05	0.06
27120	DKKL1-pendin	0.00	0.00	0.00	NaN
27121	DKK4	0.36	0.00	-0.05	0.02
27122	DKK3	0.00	0.00	0.00	NaN
27123	DKK2	0.00	0.00	0.00	NaN
27124	PIB5PA	0.00	0.00	0.00	NaN
27125	AF5Q31	0.00	-0.13	0.00	0.37
27128	PSCD4	0.00	0.00	0.05	0.10
27129	HSPB7	0.00	0.00	0.00	NaN
27130	INVS	0.00	0.00	0.00	NaN
27131	SNX5	0.00	0.00	0.00	NaN
27132	CPNE7	-0.09	-0.13	-0.05	-0.18
27134	TJP3	0.00	0.00	0.00	NaN
27136	MORC	0.00	0.00	0.00	NaN
27141	CIDEB	0.00	0.00	0.00	NaN
27147	KIAA1277	0.00	0.00	0.00	NaN
27156	RTDR1	0.00	0.00	0.00	NaN
27158	NR1	0.00	0.00	0.00	NaN

27159	TSA1902	0.09	0.00	0.00	0.09
27165	GA	0.00	0.00	0.00	NaN
27173	SLC39A1	0.09	0.13	0.00	0.36
27175	TUBG2	0.00	-0.13	0.00	-0.14
27178	FIL1(ZETA)	0.00	0.00	0.00	NaN
27179	FIL1(EPSILON)	0.00	0.00	0.00	NaN
27180	SIGLEC9	0.00	0.13	0.00	0.26
27181	SIGLEC8	0.00	0.13	-0.05	0.33
27183	VPS4A	0.00	-0.13	0.05	0.35
27190	IL17B	0.00	0.00	0.00	NaN
27229	76P	0.00	0.00	0.00	NaN
27230	SERP1	0.00	0.00	0.05	0.31
27231	MIBP	0.00	0.00	0.00	NaN
27232	GNMT	0.00	0.00	0.00	NaN
27233	SULT1C2	0.00	0.00	0.00	NaN
27235	CL640	0.00	0.00	0.00	NaN
27236	HSU52521	0.00	0.00	0.00	0.57
27237	ARHGEF16	0.00	0.00	0.00	NaN
27239	A	-0.09	0.00	0.00	0.02
27240	SIT	-0.09	0.00	0.10	-0.05
27241	B1	0.00	0.13	0.00	0.08
27242	TNFRSF21	0.00	0.00	0.00	NaN
27243	BC-2	0.00	0.13	0.00	0.57
27244	PA26	0.00	0.00	0.05	-0.04
27245	DJ159A19.3	-0.09	0.00	0.00	0.08
27246	ZNF364	0.00	0.00	0.05	-0.05
27247	HIRIP5	0.00	0.00	0.00	NaN
27249	CL25022	0.00	0.00	0.00	NaN
27250	PDCD4	0.00	0.00	-0.05	0.27
27252	AB026190	0.00	0.00	0.00	NaN
27253	PCDH17	0.00	0.00	0.00	NaN
27255	CNTN6	0.00	0.00	-0.05	0.15
27257	LSM1	0.45	-0.13	-0.05	0.75
27258	LSM3	0.00	0.00	0.00	NaN
27284	SULT1B1	0.00	0.00	0.00	NaN
27285	TEKT2	0.00	0.00	0.00	NaN
27287	VENTX2	0.00	0.00	-0.10	-0.05
27288	HNRNPG-T	0.00	0.00	0.00	NaN
27289	RHO6	0.00	0.00	0.00	NaN
27290	SPINK4	-0.09	0.00	0.05	-0.22
27291	PSORT	0.00	0.00	-0.05	-0.01
27292	HSA9761	0.00	0.00	0.00	NaN
27293	ASML3B	0.00	0.00	0.00	NaN
27295	ALP	0.00	0.00	-0.05	-0.03
27296	C20orf10	0.00	0.00	0.00	NaN
27297	CGRP-RCP	0.00	0.00	0.00	NaN
27299	ADAMDEC1	0.00	-0.13	-0.05	0.07

27300	AF020591	0.00	0.13	0.05	0.42
27302	BMP10	0.00	0.00	0.00	NaN
27303	RBMS3	0.00	0.00	0.00	NaN
27304	MOCS3	0.09	0.13	0.05	0.43
27309	HSA6591	0.00	0.00	0.00	NaN
27314	RAB30	0.00	0.00	0.05	-0.19
27315	FRAG1	0.00	-0.13	0.00	0.22
27324	TNRC9	0.00	0.00	-0.05	0.13
27329	ANGPTL3	0.00	0.00	0.00	NaN
27332	NP220	0.00	0.00	0.00	NaN
27333	GOLPH4	0.00	0.00	0.00	NaN
27335	M9	0.00	0.00	0.00	NaN
27338	E2-EPF	0.09	0.00	0.00	0.02
27339	NMP200	0.00	0.00	0.00	NaN
27340	DRIM	0.00	0.00	0.00	NaN
27341	CGI-96	0.00	0.00	0.10	0.45
27342	RABEX5	-0.09	0.00	0.00	0.36
27343	POLL	0.00	-0.13	-0.05	0.21
27345	KCNMB4	0.09	0.00	0.00	-0.16
27346	MAC30	0.00	0.25	0.00	0.21
27347	STK39	0.00	0.00	0.00	NaN
27350	APOBEC1L	-0.09	0.00	0.05	0.27
27352	DJ1042K10.2	-0.09	0.00	0.05	0.08
27429	PRSS25	0.00	0.00	0.00	NaN
27430	MAT2B	0.00	0.00	0.00	NaN
27434	POLM	0.00	0.00	0.00	NaN
27436	EML4	0.00	0.00	0.00	NaN
27440	CECR5	0.00	0.00	0.05	0.44
27445	PCLO	0.09	0.00	0.00	0.10
28231	SLC21A12	0.09	0.13	0.00	-0.11
28232	SLC21A11	0.00	0.00	0.00	NaN
28234	SLC21A8	0.00	0.00	0.00	NaN
28511	KBRAS2	0.00	0.13	0.00	0.41
28513	CDH19	-0.09	0.00	-0.10	0.02
28951	GS3955	0.00	0.00	0.00	NaN
28954	REM	0.00	0.00	0.00	NaN
28955	MYLE	0.00	0.00	0.00	NaN
28956	HSPC003	0.00	0.13	0.05	0.25
28957	MRPS28	0.00	0.50	0.50	0.60
28958	HSPC009	0.00	-0.13	0.00	0.28
28959	LR8	0.00	0.00	-0.05	0.04
28960	HSPC015	-0.09	0.00	-0.15	0.38
28962	HSPC019	0.00	0.00	0.10	0.37
28964	GIT1	0.00	0.38	0.00	0.45
28965	VLCS-H1	0.00	0.00	0.00	NaN
28966	SBBI31	0.00	0.00	0.00	NaN
28968	NTT5	0.00	0.00	0.00	NaN

28969	HSPC028	0.00	0.13	0.00	0.21
28971	PTD015	0.27	0.00	0.00	0.84
28972	SPC12	-0.09	0.00	-0.10	0.54
28973	MRPS18B	0.00	0.00	0.00	NaN
28974	HSPC023	0.00	0.00	0.00	NaN
28977	MRPL42	0.00	0.13	0.00	0.11
28978	PTD011	0.00	0.00	0.00	NaN
28981	CDV-1	0.00	0.00	0.00	NaN
28983	DESC1	0.00	0.00	0.05	0.01
28984	RGC32	0.00	0.00	-0.05	0.05
28990	HT001	0.00	0.00	0.00	NaN
28992	LRP16	0.00	0.00	0.00	NaN
28996	HIPK2	0.00	0.00	0.00	NaN
28997	PRO0611	0.00	0.00	0.05	-0.18
28998	MRPL13	0.00	0.38	0.15	0.63
28999	KLF15	0.00	0.00	0.00	NaN
29015	DKFZp762A22	0.00	0.00	0.00	NaN
29018	PRO1768	0.00	0.13	0.00	0.07
29028	PRO2000	0.27	0.25	0.05	0.59
29034	PRO0132	0.00	0.00	0.00	NaN
29035	PRO0149	0.00	0.00	0.00	NaN
29053	PRO0628	0.18	-0.13	0.00	0.04
29058	C20orf30	0.00	0.00	0.00	NaN
29062	HSPC049	0.00	0.00	0.00	NaN
29063	HSPC052	0.00	0.00	-0.05	0.20
29065	HSPC054	0.00	0.00	0.20	-0.46
29066	HSPC055	0.00	0.00	0.00	NaN
29068	HSPC063	-0.09	0.00	-0.10	0.28
29072	HYPB	-0.09	0.00	-0.05	0.58
29074	MRPL18	0.00	0.00	0.00	NaN
29075	HSPC072	0.00	0.00	0.00	NaN
29078	HSPC125	0.00	0.00	0.00	NaN
29079	HSPC126	0.00	0.00	0.00	NaN
29080	HSPC128	0.00	0.13	0.00	0.36
29081	HSPC133	0.00	0.00	0.00	NaN
29082	HSPC134	0.00	0.00	0.00	-0.07
29083	HSPC135	0.00	0.00	0.00	NaN
29086	HSPC142	-0.09	0.00	0.00	0.11
29087	HSPC144	-0.09	0.00	-0.15	0.49
29091	HSPC156	0.00	0.00	0.00	NaN
29092	HSPC157	-0.09	0.00	0.00	0.40
29093	MRPL22	0.00	0.00	0.00	NaN
29094	HSPC159	0.00	0.00	0.00	NaN
29095	HSPC160	0.00	0.00	0.00	NaN
29097	HSPC163	0.09	0.00	0.00	0.29
29098	MOG1	0.00	0.00	0.00	NaN
29099	HSPC166	0.00	0.13	0.05	0.65

29102	RNASE3L	0.09	0.25	0.40	0.31
29103	MCJ	0.00	0.00	-0.05	-0.12
29104	N6AMT1	0.00	0.00	0.00	NaN
29105	AF093680	-0.09	0.00	0.00	0.38
29107	NXT1	0.00	0.00	0.00	NaN
29108	ASC	0.00	0.00	0.00	NaN
29109	FHOD1	-0.09	0.00	0.00	0.20
29110	TBK1	0.09	0.00	0.00	0.32
29113	STG	0.00	0.00	0.00	NaN
29114	NP25	0.00	0.00	0.00	NaN
29115	HCNGP	0.18	0.13	0.00	0.49
29116	MIR	0.00	0.00	0.00	NaN
29117	BRD7	-0.09	0.00	-0.05	0.62
29118	GRTH	-0.09	0.00	-0.10	0.25
29119	VR22	0.00	0.00	0.00	NaN
29121	LLT1	-0.09	0.00	0.00	-0.04
29122	TSP50	-0.09	0.00	-0.05	0.19
29123	LZ16	-0.09	-0.13	-0.05	0.22
29124	PP13	0.00	0.00	0.00	NaN
29125	C11orf21	0.00	-0.13	0.00	-0.10
29127	RACGAP1	0.00	0.00	0.00	NaN
29760	BLNK	0.00	0.00	-0.05	0.13
29761	USP25	0.00	-0.13	-0.05	0.47
29763	PACSIN3	0.00	0.00	0.00	NaN
29766	TMOD3	0.18	0.00	0.00	0.29
29767	TMOD2	0.18	0.00	0.00	0.21
29774	DKFZP434P21	0.00	0.00	0.00	NaN
29775	CARD10	-0.09	0.00	0.10	0.02
29777	ABT1	0.00	0.00	0.00	NaN
29780	PARVB	0.00	0.00	0.05	0.01
29781	384D8-2	0.00	-0.25	0.00	0.14
29789	PTD004	0.00	0.00	0.00	NaN
29796	HSPC051	-0.09	0.00	0.00	0.30
29798	AF038169	0.00	0.00	-0.05	-0.08
29799	AF060862	0.00	0.00	0.00	NaN
29802	VPREB3	0.00	0.00	0.05	-0.03
29803	RIP60	0.00	0.00	-0.05	0.42
29842	LBP-9	0.00	0.00	0.00	NaN
29844	TFPT	0.09	0.00	0.00	-0.16
29855	UBN1	0.00	0.00	-0.05	0.62
29880	ALG5	0.00	0.00	0.00	NaN
29881	NPC1L1	0.00	0.00	0.00	NaN
29882	APC2	0.00	0.13	0.00	-0.02
29883	CNOT7	0.00	-0.13	-0.10	0.26
29887	SNX10	0.00	0.13	0.00	-0.10
29888	ZIN	0.09	0.00	0.00	0.19
29889	HUMAUANTIG	0.00	0.00	0.00	NaN

29890	HUMAGCGB	-0.09	0.00	-0.10	0.49
29893	HUMGT198A	0.00	-0.13	0.00	-0.10
29894	CPSF1	0.18	0.00	0.15	0.50
29895	HUMMLC2B	0.00	0.00	0.00	NaN
29896	HSU53209	0.00	0.13	0.00	0.45
29899	HSU54999	0.09	0.00	0.05	0.37
29901	HSU79266	0.00	0.00	0.00	NaN
29902	HSU79274	0.00	0.00	0.00	NaN
29903	HSU79303	1.00	1.00	0.00	0.32
29906	SIAT8E	-0.09	-0.13	-0.20	-0.04
29907	SNX15	0.00	0.00	0.00	NaN
29909	H963	0.00	0.00	0.00	NaN
29911	HOOK2	0.00	0.00	0.00	NaN
29914	TERE1	0.00	0.00	0.00	NaN
29915	HCF-2	0.00	0.00	0.00	NaN
29916	SNX11	0.00	0.00	0.00	0.27
29919	MIC1	0.00	0.00	-0.15	0.45
29922	NME7	0.00	0.00	-0.05	0.44
29923	HIG2	0.00	0.25	0.00	0.43
29924	EPSIN	0.09	0.13	0.00	0.33
29925	GMPPB	-0.09	0.00	-0.05	0.23
29926	GMPPA	0.00	0.00	0.00	NaN
29927	SEC61A1	0.00	0.00	0.00	NaN
29928	TIMM22	0.00	-0.13	0.00	0.06
29929	ALG6	0.00	0.00	0.00	NaN
29930	PCDHB1	0.00	0.00	0.00	NaN
29931	NAG-7	0.00	0.00	0.00	NaN
29933	G2A	-0.09	0.00	0.00	-0.01
29937	SPUF	0.00	0.00	0.00	NaN
29940	SART-2	-0.09	0.00	0.05	0.16
29942	PURG	-0.09	-0.25	-0.05	-0.08
29943	PADI1	-0.09	0.00	0.00	-0.32
29946	RBT1	0.00	0.00	0.00	NaN
29947	DNMT3L	0.00	0.00	0.00	NaN
29948	OKL38	-0.09	-0.13	-0.05	0.01
29949	IL19	0.00	0.13	0.00	-0.05
29953	TRHDE	0.09	0.00	0.00	0.28
29954	POMT2	0.00	0.00	0.00	NaN
29956	LASS2	0.00	0.00	0.05	0.26
29957	DKFZp586G01	0.09	0.00	0.00	0.18
29959	NRBP	0.09	0.00	0.05	0.65
29960	FTSJ2	0.00	0.13	0.05	0.54
29964	OBTP	0.00	0.00	1.00	0.18
29965	C16orf5	0.00	0.00	-0.05	0.21
29966	GS2NA	0.00	0.00	0.00	NaN
29967	ST7	0.18	-0.25	0.05	0.06
29968	PSA	0.00	0.00	-0.05	-0.30

29970	SCHIP1	0.00	0.00	0.00	NaN
29974	ACF	0.00	0.00	0.05	0.32
29980	DONSON	0.00	0.00	0.00	NaN
29982	NRBF-2	0.00	0.00	0.00	NaN
29984	ARHD	0.18	0.00	0.05	0.36
29986	SLC39A2	0.00	0.00	0.00	NaN
29988	SLC2A8	0.00	0.00	-0.05	0.64
29989	OBP2B	0.00	0.00	0.00	NaN
29990	PILR(BETA)	0.18	0.00	0.00	0.24
29991	OBP2A	0.00	0.00	0.10	-0.08
29992	PILR(ALPHA)	0.18	0.00	0.00	0.18
29994	BAZ2B	0.00	0.00	-0.05	0.15
29995	LMCD1	0.00	0.00	0.00	NaN
29997	GLTSCR2	0.09	0.00	0.00	0.32
29998	GLTSCR1	0.09	0.00	0.00	0.20
29999	FSCN3	0.00	0.25	0.00	0.12
30000	TRN2	0.00	0.00	0.00	NaN
30001	ERO1L	0.00	0.25	0.05	-0.16
30008	EFEMP2	-0.09	0.00	0.05	0.29
30009	TBX21	0.00	-0.13	0.00	-0.08
30012	HOX11L2	0.00	0.00	0.00	NaN
30811	HUNK	0.00	0.00	0.00	NaN
30813	VSX1	0.00	0.00	-0.05	0.01
30814	PLA2G2E	-0.09	0.00	0.00	-0.11
30817	EMR2	0.00	0.00	0.00	NaN
30819	KCNIP2	0.00	-0.13	-0.05	-0.18
30820	KCNIP1	0.00	0.00	0.00	NaN
30827	CGBP	-0.09	-0.13	-0.25	0.42
30835	CD209	0.00	0.00	0.00	NaN
30836	HSU15552	0.09	0.00	0.00	0.51
30837	NAP4	0.00	0.25	0.00	0.34
30844	EHD4	0.00	0.00	0.00	NaN
30845	EHD3	0.00	0.00	0.00	NaN
30846	EHD2	0.09	0.00	0.00	0.23
30849	PIK3R4	0.00	0.00	0.00	NaN
30850	HUMPPA	0.00	0.13	0.00	0.32
30851	TIP-1	0.00	0.00	0.00	NaN
43847	KLK14	0.00	0.00	0.00	NaN
49856	WDR8	0.00	0.00	0.00	NaN
49860	C1orf10	0.00	0.00	0.05	-0.13
50485	SMARCAL1	0.00	0.00	0.00	NaN
50486	G0S2	0.00	0.00	0.00	NaN
50487	PLA2G3	0.00	0.00	0.00	NaN
50488	MINK	0.00	0.00	0.00	NaN
50489	LANGERIN	0.00	0.00	0.05	0.28
50506	DUOX2	0.00	0.00	0.00	NaN
50507	NOX4	0.00	0.00	0.00	NaN

50508	NOX3	0.00	0.00	0.00	NaN
50509	COL5A3	0.00	0.00	0.05	-0.06
50512	PODLX2	0.00	0.00	0.00	NaN
50515	C4ST	0.00	0.00	0.00	NaN
50613	UBQLN3	0.00	-0.13	-0.05	-0.09
50615	IL21R	0.00	0.00	0.00	NaN
50616	IL22	0.50	0.00	0.00	0.24
50617	ATP6V0A4	0.00	0.00	0.00	NaN
50618	ITSN2	0.09	0.00	-0.05	0.55
50624	ERG-1	0.09	0.00	-0.05	0.45
50626	CYHR1	0.18	0.00	0.10	0.33
50632	CALCYON	0.00	0.00	-0.10	0.13
50649	ARHGEF4	0.00	0.00	-0.05	0.14
50650	ARHGEF3	0.00	0.00	-0.10	0.09
50674	NEUROG3	0.00	0.00	0.00	NaN
50700	RDH8	0.00	0.00	0.05	-0.09
50717	H326	0.00	0.00	0.00	NaN
50801	KCNK4	0.00	0.00	0.00	NaN
50804	MEF-2	0.00	0.00	0.00	NaN
50805	IRX4	0.00	0.13	0.10	-0.22
50807	DDEF1	0.00	0.00	0.20	0.28
50809	HP1-BP74	-0.09	0.00	-0.05	0.09
50810	CGI-142	0.00	0.00	0.00	0.19
50813	COPS7A	-0.09	0.00	0.00	0.25
50831	TAS2R3	0.00	0.00	0.05	0.14
50832	TAS2R4	0.00	0.00	0.00	NaN
50833	TAS2R16	0.00	0.13	0.00	0.08
50834	TAS2R1	0.00	0.00	0.10	-0.16
50835	TAS2R9	-0.09	0.00	0.00	0.03
50836	TAS2R8	-0.09	0.00	0.00	-0.02
50837	TAS2R7	-0.09	0.00	0.00	0.05
50838	TAS2R13	-0.09	0.00	0.00	0.33
50839	TAS2R10	-0.09	0.00	0.00	0.16
50840	TAS2R14	-0.09	0.00	0.00	-0.06
50848	JAM1	0.00	0.00	0.00	NaN
50852	TRIM	0.00	0.00	0.00	NaN
50853	VILL	0.00	0.25	0.00	-0.04
50855	PARD6A	-0.09	-0.13	0.00	-0.02
50856	CLECSF6	-0.09	0.00	0.00	0.00
50859	HSAJ1454	0.00	0.00	0.00	NaN
50861	STMN3	0.09	0.00	0.00	-0.15
50862	ZFP26	0.00	0.00	0.00	NaN
50863	HNT	-0.09	0.00	-0.10	0.10
50865	HEBP1	-0.09	0.00	0.00	0.29
50937	CDON	-0.09	0.00	-0.10	-0.11
50939	IMPG2	0.00	0.00	0.00	NaN
50940	PDE11A	0.00	0.00	0.00	NaN

50944	SHANK1	0.00	0.00	0.00	NaN
50999	LOC50999	0.00	0.00	0.05	0.30
51001	LOC51001	0.00	-0.13	0.05	0.56
51002	LOC51002	0.00	0.00	0.00	NaN
51003	LOC51003	0.00	0.00	0.00	NaN
51004	LOC51004	0.00	0.00	0.00	NaN
51005	LOC51005	0.00	0.00	0.00	NaN
51006	OVCOV1	0.00	0.00	0.00	NaN
51008	LOC51008	0.09	0.00	0.00	0.26
51009	F-LAN-1	0.00	-0.13	0.00	0.13
51011	LOC51011	0.00	0.00	0.00	NaN
51012	C20orf45	0.18	0.25	0.00	0.47
51013	CSL4	0.00	0.00	-0.05	0.03
51014	LOC51014	0.00	0.13	0.00	0.57
51015	LOC51015	0.00	0.00	0.00	NaN
51016	LOC51016	0.00	0.00	0.00	NaN
51018	LOC51018	0.09	0.00	0.00	-0.07
51019	LOC51019	0.00	0.00	0.00	NaN
51020	LOC51020	0.00	0.00	0.00	NaN
51021	MRPS16	0.09	0.00	0.00	0.43
51022	GLRX2	0.09	0.00	0.00	0.32
51023	MRPS18C	0.00	0.00	0.00	NaN
51024	LOC51024	0.18	0.00	0.00	0.56
51025	Magmas	0.00	0.00	0.00	NaN
51026	LOC51026	0.00	0.00	0.00	NaN
51027	LOC51027	0.00	0.00	0.05	0.27
51029	LOC51029	0.00	0.13	0.00	0.15
51030	LOC51030	0.00	0.00	0.00	NaN
51031	LOC51031	0.00	-0.13	0.00	-0.08
51032	LOC51032	0.00	0.00	0.00	NaN
51035	LOC51035	0.00	0.00	0.00	NaN
51042	LOC51042	0.00	0.00	0.00	NaN
51043	ZFP67	0.09	0.13	0.00	0.06
51046	SIAT8C	-0.09	0.00	-0.20	0.22
51050	PI15	0.00	0.25	0.00	-0.10
51052	LOC51052	0.00	0.00	-0.05	0.08
51053	LOC51053	0.00	0.00	0.05	0.15
51054	LOC51054	0.00	0.13	-0.05	0.37
51063	LOC51063	0.00	-0.13	-0.05	0.13
51065	RPS27L	0.00	0.00	0.00	NaN
51066	LOC51066	0.00	0.00	0.00	NaN
51067	LOC51067	0.00	0.00	0.00	NaN
51068	LOC51068	0.00	0.00	0.00	NaN
51069	MRPL2	0.00	0.00	0.00	NaN
51070	NOSIP	0.00	0.00	0.00	NaN
51071	LOC51071	0.00	0.00	0.00	NaN
51073	MRPL4	0.00	0.00	0.05	0.45

51074	LOC51074	0.00	0.13	0.05	0.39
51075	LOC51075	0.00	0.00	0.00	NaN
51076	LOC51076	0.00	-0.13	-0.05	0.30
51077	LOC51077	0.00	0.00	0.00	NaN
51078	LOC51078	0.00	0.00	-0.05	0.41
51079	GRIM19	0.00	0.00	0.00	NaN
51081	MRPS7	0.00	0.13	0.00	0.32
51082	LOC51082	0.00	0.00	-0.05	0.22
51083	LOC51083	0.45	0.00	0.00	-0.24
51084	CRYL1	0.00	0.00	-0.10	0.20
51086	LOC51086	0.00	0.00	0.00	NaN
51087	LOC51087	0.00	0.00	0.00	NaN
51088	LOC51088	0.00	0.00	0.05	-0.20
51090	PMLP	-0.09	0.00	0.00	-0.28
51092	LOC51092	-0.09	0.00	0.00	0.21
51093	LOC51093	0.00	0.13	0.00	0.00
51094	LOC51094	0.00	0.13	0.00	0.29
51096	LOC51096	0.00	0.13	0.00	0.25
51097	LOC51097	0.00	0.00	0.00	NaN
51098	C20orf9	0.09	-0.13	0.00	0.57
51099	CGI-58	0.00	0.00	-0.05	0.28
51100	SH3GLB1	0.00	0.00	0.10	0.55
51101	LOC51101	0.00	0.25	0.05	0.68
51102	LOC51102	0.00	0.00	0.00	NaN
51103	LOC51103	0.00	0.00	0.00	NaN
51104	LOC51104	0.00	0.00	-0.05	0.11
51105	CGI-72	0.00	0.00	0.05	0.26
51106	TFB1M	0.00	0.00	0.00	NaN
51107	LOC51107	0.00	0.13	0.05	0.52
51108	DREV1	0.00	0.00	0.00	NaN
51109	ARSDR1	0.00	0.00	0.00	NaN
51110	LOC51110	0.00	0.25	0.00	0.71
51112	LOC51112	0.00	0.00	0.00	NaN
51115	LOC51115	0.18	0.25	0.00	0.45
51116	MRPS2	0.00	0.00	0.00	NaN
51117	COQ4	0.00	0.00	0.00	NaN
51118	CGI-94	0.00	0.00	0.00	NaN
51121	RPL26L1	0.00	0.00	0.00	NaN
51123	LOC51123	0.18	0.00	0.00	0.15
51125	LOC51125	0.45	-0.25	-0.05	0.78
51127	TRIM17	0.09	0.00	0.00	-0.09
51128	LOC51128	0.00	-0.13	0.00	0.40
51129	ANGPTL4	0.00	0.00	0.00	NaN
51131	LOC51131	0.00	0.13	0.00	0.31
51133	LOC51133	0.09	0.00	0.00	0.31
51134	LOC51134	0.00	0.00	0.00	NaN
51135	IRAK4	0.00	0.13	0.00	0.50

51136	LOC51136	0.18	0.13	0.00	0.45
51138	COPS4	0.00	0.00	0.00	NaN
51142	LOC51142	0.00	0.00	0.10	0.59
51143	LOC51143	0.00	-0.13	0.00	0.36
51144	HSD17B12	0.00	0.00	0.00	NaN
51146	LOC51146	0.00	0.00	0.00	NaN
51147	ING4	-0.09	0.00	0.00	0.12
51149	LOC51149	0.00	0.00	0.00	NaN
51150	Cab45	0.00	0.00	0.00	NaN
51151	MATP	0.00	0.00	0.10	0.05
51154	C1orf33	-0.09	0.00	0.00	0.20
51155	HN1	0.00	0.13	0.00	0.00
51156	SERPINA10	0.00	0.00	0.00	NaN
51157	LOC51157	0.09	0.13	0.00	0.28
51160	VPS28	0.18	0.00	0.15	0.42
51161	LOC51161	-0.09	0.00	-0.05	0.11
51162	LOC51162	0.00	0.00	0.00	NaN
51163	DBR1	0.00	0.00	0.00	NaN
51164	DCTN4	0.00	0.00	0.00	NaN
51167	b5&b5R	0.00	0.00	0.00	NaN
51168	MYO15A	0.00	0.00	0.00	NaN
51170	LOC51170	0.00	0.00	0.00	NaN
51171	LOC51171	0.09	0.00	0.00	-0.11
51172	LOC51172	0.00	0.00	0.00	NaN
51174	LOC51174	0.18	0.13	0.00	0.80
51176	LEF1	0.00	0.00	0.00	NaN
51177	LOC51177	0.00	0.00	0.05	0.76
51179	HAO2	0.09	0.00	0.05	0.28
51181	DCXR	0.00	0.00	0.00	NaN
51182	LOC51182	0.00	0.00	0.00	NaN
51184	LOC51184	0.00	0.00	0.00	NaN
51185	LOC51185	0.00	-0.13	0.00	0.31
51187	C15orf15	0.00	0.00	0.05	0.27
51188	SS18L2	0.00	0.00	-0.05	0.29
51191	LOC51191	0.00	0.00	0.00	NaN
51193	LOC51193	0.09	0.00	0.05	0.34
51195	LOC51195	0.00	0.38	0.00	0.54
51196	PLCE1	0.00	0.00	0.00	NaN
51198	LOC51198	-0.18	-0.13	-0.25	-0.08
51199	NIN	0.00	0.00	0.05	0.14
51200	CPA4	0.00	0.25	0.05	0.10
51202	LOC51202	-0.09	0.00	0.00	0.40
51203	ANKT	0.00	0.00	0.00	NaN
51204	LOC51204	0.27	0.13	0.00	0.66
51205	LOC51205	0.00	0.00	0.05	0.10
51206	GP6	0.09	0.00	0.00	-0.14
51207	LOC51207	0.09	0.00	0.00	0.12

51208	CLDN18	0.00	0.00	0.00	NaN
51214	IGF2AS	0.00	-0.13	0.00	0.04
51218	LOC51218	0.00	0.00	0.00	NaN
51222	ZNF219	0.00	0.00	0.00	NaN
51224	TCEB3L	-0.09	-0.13	-0.15	-0.09
51226	LOC51226	0.00	0.00	0.00	0.11
51227	DSCR5	0.00	0.00	0.00	NaN
51230	C20orf104	0.00	0.00	0.00	NaN
51231	LOC51231	0.00	0.00	0.00	NaN
51232	CRIM1	0.00	0.00	0.00	NaN
51233	LOC51233	0.00	0.00	0.05	0.14
51236	LOC51236	0.18	0.00	0.10	0.36
51237	FLJ32987	0.00	0.00	0.00	NaN
51241	LOC51241	0.00	0.00	0.00	NaN
51265	CDKL3	0.00	-0.13	0.00	0.07
51266	LOC51266	-0.09	0.00	0.00	-0.04
51267	LOC51267	-0.09	0.00	0.00	-0.02
51268	PIPOX	0.00	0.38	0.00	0.46
51271	UBAP	-0.09	0.13	0.10	0.76
51272	LOC51272	-0.27	-0.13	-0.15	0.40
51274	KLF3	0.00	0.00	0.05	0.01
51275	LOC51275	0.00	0.00	0.00	NaN
51276	HSPC059	0.00	0.13	0.00	-0.03
51278	IER5	0.00	0.00	0.05	0.02
51279	LOC51279	0.00	0.00	0.00	0.23
51280	GOLPH2	0.00	0.00	0.00	NaN
51281	FLJ20499	0.00	0.00	-0.05	0.21
51282	SCAND1	0.00	0.00	0.00	NaN
51283	LOC51283	0.00	0.00	0.00	NaN
51285	LOC51285	0.00	0.00	0.00	NaN
51286	BM88	-0.18	-0.13	-0.05	0.09
51287	E2IG2	0.09	0.13	0.00	0.32
51289	LOC51289	0.00	0.00	0.10	-0.16
51290	LOC51290	0.00	0.00	0.00	NaN
51291	LOC51291	0.00	0.00	0.00	NaN
51292	LOC51292	0.00	0.00	0.00	NaN
51293	8D6A	0.00	0.13	0.00	-0.19
51294	PCDH12	0.00	0.00	0.00	NaN
51295	LOC51295	0.00	0.00	0.00	NaN
51296	PHT2	0.00	0.00	0.00	NaN
51297	PLUNC	0.00	0.00	0.00	NaN
51298	THEG	0.00	0.00	-0.05	0.03
51299	LOC51299	0.00	0.00	0.00	NaN
51301	LOC51301	0.00	0.00	0.00	NaN
51302	CYP39A1	0.00	0.00	0.00	NaN
51303	LOC51303	0.00	0.00	0.00	NaN
51304	ZDHHC3	-0.09	0.00	-0.05	0.28

51306	C5orf5	0.00	-0.13	0.00	0.28
51307	C5orf6	0.00	0.00	0.00	NaN
51308	LOC51308	0.00	0.00	0.00	NaN
51310	LOC51310	0.00	0.00	0.00	NaN
51312	LOC51312	-0.09	-0.25	-0.15	0.23
51313	LOC51313	0.00	0.00	0.00	0.05
51314	LOC51314	0.00	0.00	0.00	NaN
51315	LOC51315	0.00	0.00	-0.05	0.34
51317	BHC80	0.00	0.00	0.00	NaN
51318	MRPL35	0.00	0.00	-0.05	0.06
51319	LOC51319	0.00	0.00	0.00	NaN
51320	LOC51320	-0.09	-0.13	-0.20	0.39
51321	LOC51321	0.18	0.38	0.00	0.32
51322	WAC	-0.09	0.00	0.05	0.37
51324	ACP33	0.00	0.00	0.00	NaN
51327	ERAF	0.00	0.00	0.00	NaN
51329	LOC51329	0.00	0.00	0.00	NaN
51330	FN14	0.00	0.00	0.00	NaN
51332	SPTBN5	0.00	0.00	0.00	NaN
51333	LOC51333	0.00	0.00	0.00	NaN
51334	LOC51334	0.00	0.00	0.00	NaN
51335	NEUGRIN	0.00	0.00	0.00	NaN
51337	LOC51337	0.18	0.13	0.05	0.20
51338	MS4A4A	0.00	0.00	0.00	NaN
51339	LOC51339	0.00	0.00	0.00	NaN
51340	CRNKL1	0.00	0.00	0.00	NaN
51341	FBI1	0.00	0.00	0.05	0.04
51343	FZR1	0.00	0.00	0.00	NaN
51347	JIK	-0.09	-0.13	0.00	0.18
51348	KLRF1	-0.09	0.00	0.00	-0.07
51350	HUMCYT2A	0.00	0.00	0.00	NaN
51351	H-plk	0.00	0.00	0.00	-0.20
51352	WIT-1	0.00	0.00	0.00	NaN
51361	HOOK1	0.00	0.00	-0.05	0.09
51362	PRP17	0.00	0.00	0.05	0.35
51363	GALNAC4S-6S	0.00	0.00	-0.05	0.09
51364	BLu	-0.09	0.00	-0.05	-0.09
51365	PS-PLA1	0.00	0.00	0.00	NaN
51366	DD5	0.18	0.00	0.00	0.31
51367	POP5	0.00	-0.13	0.00	0.31
51368	SIG11	-0.09	0.00	-0.10	0.45
51371	HSPC014	0.00	0.00	-0.05	0.05
51372	HSPC016	-0.09	0.00	-0.05	0.28
51373	MRPS17	0.00	0.00	0.10	0.55
51374	4/3/2010	0.09	0.00	0.05	0.57
51375	SNX7	0.00	0.00	0.00	NaN
51377	UCH37	0.09	0.00	0.00	0.32

51378	ANGPT4	0.00	0.00	0.00	NaN
51379	CRLF3	0.00	0.13	0.00	0.29
51380	CSAD	0.00	0.00	0.00	NaN
51382	ATP6V1D	0.00	0.00	0.05	0.32
51384	WNT16	0.00	0.13	0.00	0.11
51385	SZF1	-0.09	0.00	-0.05	0.17
51386	EIF3S6IP	-0.09	0.00	0.05	0.33
51388	HSPC031	0.00	-0.13	0.05	0.26
51397	PTD002	0.00	0.13	0.00	0.47
51398	PTD008	0.00	0.00	0.05	-0.04
51399	PTD009	0.00	0.00	0.00	NaN
51400	PME-1	0.09	0.00	0.00	0.43
51406	RARG-1	0.00	0.00	0.10	0.52
51409	HEMK	-0.09	0.00	-0.05	0.28
51411	BIN2	0.00	0.00	0.00	NaN
51412	ACTL6	0.18	0.00	0.00	0.15
51421	AMOTL2	0.00	0.00	0.00	NaN
51422	PRKAG2	0.00	0.00	-0.05	0.03
51427	ZFD25	-0.09	0.00	0.00	0.17
51428	ABS	0.00	0.00	0.00	NaN
51430	C1orf9	0.00	0.00	0.00	NaN
51433	ANAPC5	0.00	-0.13	0.00	0.28
51435	CSR1	-0.09	-0.25	-0.10	0.27
51439	FAM8A1	0.00	0.00	0.00	NaN
51440	HPCAL4	0.00	0.00	0.00	NaN
51441	HGRG8	0.00	0.00	0.00	NaN
51444	STRIN	0.00	0.00	-0.15	0.38
51447	IHPK2	-0.09	0.00	-0.05	0.35
51449	PCL1	0.00	0.00	0.00	NaN
51450	PRX2	0.00	0.00	0.00	NaN
51451	LCMT	0.00	0.00	0.00	NaN
51454	CED-6	0.00	0.00	0.00	NaN
51455	REV1L	0.00	0.00	0.00	NaN
51458	RHCG	0.00	0.00	-0.05	0.01
51463	SH120	0.00	0.00	0.05	0.39
51465	NCUBE1	0.00	0.00	0.00	NaN
51466	RNB6	0.00	0.00	0.00	NaN
51471	CML2	0.00	0.00	0.00	NaN
51474	EPLIN	0.00	0.00	0.00	NaN
51475	CABP2	0.27	0.00	0.05	0.12
51477	ISYNA1	0.00	0.00	0.00	NaN
51478	HSD17B7	0.00	0.00	0.00	NaN
51479	ANKHZN	0.00	0.00	0.00	NaN
51490	HSPC109	0.00	0.00	0.00	NaN
51491	HSPC111	0.00	0.00	0.00	NaN
51493	HSPC117	0.00	0.00	0.00	NaN
51495	HSPC121	0.00	0.00	0.00	NaN

51497	TH1L	0.18	0.25	0.00	0.59
51499	HSPC132	0.00	-0.13	0.00	0.27
51501	HSPC138	0.00	0.00	0.00	NaN
51504	HSPC152	0.00	0.00	0.00	NaN
51506	HSPC155	0.00	0.00	0.00	NaN
51507	C20orf43	0.09	0.50	0.00	0.55
51510	HSPC177	-0.09	0.00	0.05	0.52
51512	GTSE1	0.00	0.00	0.00	NaN
51513	TEL2	0.00	0.00	0.00	NaN
51517	AF3P21	-0.09	0.00	-0.05	0.50
51526	C20orf111	0.09	0.00	0.00	0.15
51531	HSPC219	0.00	0.00	0.00	NaN
51533	HSPC226	-0.09	-0.25	-0.10	0.19
51540	SCLY	0.00	0.00	-0.05	0.23
51542	HCC8	0.00	0.00	0.00	NaN
51547	SIRT7	0.00	0.00	0.00	NaN
51548	SIRT6	0.00	0.00	0.00	NaN
51550	CINP	0.00	0.00	0.00	NaN
51552	RAB14	0.00	0.00	-0.10	0.56
51555	PXR2b	0.09	0.00	0.00	0.11
51557	LGS	-0.09	0.00	0.05	0.26
51559	TU12B1-TY	0.00	0.00	0.00	NaN
51561	IL23A	0.00	0.00	0.00	NaN
51562	MBIP	0.00	0.00	0.00	NaN
51567	AD022	0.00	0.00	0.05	0.41
51569	BM-002	0.00	0.00	0.00	NaN
51571	BM-009	0.00	0.13	0.20	0.38
51573	MIR16	0.00	0.00	0.00	NaN
51574	HDCMA18P	0.00	0.00	0.00	NaN
51575	C20orf6	0.00	0.00	0.00	NaN
51582	OAZIN	0.18	0.00	0.00	0.19
51585	PCF11	0.00	0.00	0.05	-0.13
51586	PCQAP	0.00	0.13	0.05	0.65
51588	PIASY	0.00	0.00	0.00	NaN
51592	TRIM33	0.18	0.00	0.10	0.87
51593	ARS2	0.18	0.00	0.00	0.29
51594	NAG	0.09	0.00	-0.05	0.46
51596	LOC51596	0.00	0.00	0.00	NaN
51599	LISCH7	0.00	0.00	0.00	NaN
51601	LOC51601	0.00	0.00	0.00	NaN
51603	CGI-01	0.00	0.00	0.00	NaN
51604	LOC51604	0.00	0.00	0.00	NaN
51606	ATP6V1H	0.00	0.00	0.00	NaN
51611	LOC51611	0.09	0.00	0.00	0.41
51614	SDBCAG84	0.00	0.13	0.00	-0.02
51617	LOC51617	0.00	0.00	0.00	NaN
51619	LOC51619	0.00	0.00	0.00	NaN

51621	KLF13	0.00	0.00	0.00	NaN
51622	LOC51622	0.00	0.00	0.10	0.26
51626	LOC51626	0.00	0.00	0.00	NaN
51631	LOC51631	0.00	0.00	0.00	NaN
51635	LOC51635	0.00	0.00	0.00	NaN
51637	LOC51637	0.00	0.13	0.05	0.32
51642	MRPL48	0.09	0.13	0.00	0.46
51643	LOC51643	0.09	0.00	0.00	0.47
51646	LOC51646	0.00	0.00	0.00	NaN
51647	LOC51647	-0.09	-0.13	0.00	0.43
51650	MRPS33	0.00	0.00	0.00	NaN
51651	LOC51651	0.18	0.13	0.00	0.54
51652	LOC51652	0.00	0.00	-0.05	0.38
51654	C20orf34	0.00	0.00	0.00	NaN
51657	LOC51657	-0.09	0.00	0.00	0.30
51659	LOC51659	0.00	0.00	-0.05	0.47
51660	BRP44l	0.00	0.00	0.00	NaN
51663	ZFR	0.00	0.00	0.15	0.36
51666	ASB4	0.18	0.00	0.00	-0.18
51668	LOC51668	0.00	0.00	0.00	NaN
51669	MGC8721	-0.09	-0.25	-0.05	0.10
51673	LOC51673	-0.09	0.00	0.05	-0.11
51690	LOC51690	0.00	0.00	0.00	NaN
51691	LOC51691	0.00	0.13	0.00	0.32
51693	LOC51693	-0.09	-0.13	-0.05	0.50
51696	LOC51696	0.00	0.00	0.00	NaN
51700	LOC51700	0.00	0.00	-0.05	-0.04
51702	PADI3	-0.09	0.00	0.00	0.11
51703	FACL5	0.00	0.00	-0.05	0.04
51704	GPRC5B	0.00	0.00	0.00	NaN
51705	LOC51705	0.00	0.00	0.00	NaN
51706	LOC51706	0.00	0.13	0.00	0.21
51710	GIOT-2	0.00	0.00	0.00	NaN
51714	LOC51714	0.00	0.00	0.05	0.38
51715	RAB23	0.00	0.13	0.00	-0.10
51716	LOC51716	0.09	-0.38	0.05	-0.18
51719	MO25	0.00	0.00	0.00	NaN
51720	LOC51720	0.00	0.13	0.00	0.48
51725	LOC51725	0.00	0.00	0.00	NaN
51727	UMP-CMPK	0.00	0.00	0.00	NaN
51728	POLR3K	0.00	0.00	-0.05	0.39
51729	WBP11	-0.09	0.00	0.00	0.31
51733	UPB1	0.00	0.00	0.00	NaN
51734	SEPX1	0.00	0.00	0.00	NaN
51735	RA-GEF-2	0.00	-0.13	0.00	0.03
51741	WWOX	0.00	0.00	0.00	NaN
51742	BCAA	0.09	0.00	0.00	0.34

51744	CD244	0.00	0.00	0.00	NaN
51747	LUC7A	0.00	0.38	0.00	0.36
51751	CLST11240	0.00	-0.13	0.00	-0.18
51752	ARTS-1	0.00	0.00	0.00	NaN
51754	LOC51754	-0.09	0.00	0.10	0.43
51755	CrkRS	0.09	0.75	0.00	0.64
51759	LOC51759	0.00	0.00	0.00	NaN
51760	LOC51760	0.00	0.00	0.00	NaN
51761	ATP8A2	0.00	-0.13	-0.15	0.11
51762	LOC51762	0.00	0.00	0.00	NaN
51763	SKIP	0.00	-0.13	0.00	0.22
51764	GNG13	0.00	0.00	-0.05	0.11
51768	TM7SF3	0.00	0.00	0.00	NaN
51773	HBXAP	0.27	0.00	0.00	0.64
51776	ZAK	0.00	0.00	0.05	0.14
51778	MYOZ2	0.00	0.00	0.00	NaN
51780	C5orf7	0.00	0.00	0.00	NaN
51806	CLSP	0.00	0.00	0.05	0.24
51807	TUBA8	0.00	0.00	0.00	-0.13
51809	GALNT7	0.00	0.00	-0.05	0.29
51816	CECR1	0.00	0.00	0.05	-0.05
53335	BCL11A	0.00	0.00	0.00	NaN
53339	BTBD1	0.00	0.00	-0.05	0.46
53340	SPA17	0.00	0.00	-0.10	0.11
53343	NUDT9	0.00	0.00	0.00	NaN
53346	TM6SF1	0.00	0.00	-0.05	0.05
53347	UBASH3A	0.00	0.00	0.00	NaN
53353	LRP1B	-0.09	0.00	0.00	0.14
53358	SHC3	0.00	0.00	0.00	NaN
53371	NUP54	0.00	0.00	0.00	NaN
53373	KIAA1169	0.00	0.00	0.00	NaN
53405	CLIC5	0.00	0.00	0.00	NaN
53407	STX18	0.00	-0.13	-0.15	0.30
53615	MBD3	0.00	0.00	0.00	NaN
53616	ADAM22	0.00	0.00	0.00	NaN
53630	BCDO	-0.09	0.00	-0.05	0.21
53635	PTOV1	0.00	0.00	0.00	NaN
53637	EDG8	0.00	0.00	0.05	0.05
53820	DSCR6	0.00	0.00	0.00	NaN
53822	FXYD7	0.00	0.00	0.00	NaN
53826	FXYD6	-0.09	0.00	0.00	-0.12
53827	FXYD5	0.00	0.00	0.00	NaN
53829	GPR86	0.00	0.00	0.00	NaN
53832	IL20RA	0.00	0.00	0.00	NaN
53836	GPR87	0.00	0.00	0.00	NaN
53838	C11orf24	0.55	0.00	0.00	0.48
53840	TRIM34	0.00	-0.13	-0.05	0.12

53841	MUCDHL	-0.27	-0.13	-0.05	0.15
53904	MYO3A	-0.09	0.00	0.00	0.09
53905	DUOX1	0.00	0.00	0.00	NaN
53916	RAB4B	0.00	0.00	0.00	NaN
53918	PELO	0.00	0.00	-0.05	0.45
53919	SLC21A14	0.00	0.00	0.00	NaN
53942	CNTN5	0.00	0.00	0.00	NaN
53944	CSNK1G1	0.09	0.00	0.00	0.25
53947	A4GALT	0.00	0.00	0.00	NaN
54014	WDR9	0.00	0.00	0.00	NaN
54020	SLC37A1	0.00	0.00	0.00	NaN
54039	PCBP3	0.00	0.00	0.00	NaN
54069	C21orf45	0.00	0.00	0.00	NaN
54093	C21orf18	0.00	0.00	0.00	NaN
54101	ANKRD3	0.00	0.00	0.00	NaN
54103	LOC54103	-0.09	0.00	0.00	0.03
54107	POLE3	0.00	0.00	0.00	NaN
54112	GPR88	0.09	0.00	0.00	0.16
54148	MRPL39	0.00	0.00	0.00	NaN
54149	C21orf91	0.00	-0.13	0.00	0.00
54165	RP42	0.09	0.00	0.00	0.15
54187	SAS	0.00	0.00	0.00	NaN
54205	HCS	0.00	0.13	0.00	0.00
54207	KCNK10	0.00	0.13	0.00	-0.13
54209	TREM2	0.00	0.00	0.00	NaN
54210	TREM1	0.00	0.00	0.00	NaN
54212	SNTG1	0.00	0.00	0.00	NaN
54221	SNTG2	0.00	0.00	0.00	NaN
54329	GPR85	0.09	0.00	0.00	-0.32
54332	GDAP1	0.00	0.25	0.00	-0.10
54344	DPM3	0.27	0.13	0.00	0.39
54345	SOX18	0.00	0.00	0.00	NaN
54360	C17	0.00	-0.13	-0.15	0.01
54361	WNT4	-0.09	0.00	0.00	-0.05
54363	HAO1	0.00	0.00	0.05	0.04
54386	RAP1	0.00	-0.13	0.05	0.35
54407	SLC38A2	0.00	0.00	0.50	-0.06
54431	DKFZP434J181	0.00	0.00	0.00	NaN
54434	SSH1	0.00	-0.13	0.00	0.31
54435	HCGIV.9	0.00	0.00	0.00	NaN
54436	FLJ20356	0.00	-0.13	-0.10	0.11
54438	FLJ20330	0.00	0.00	0.05	0.46
54441	DKFZP434A01	0.00	0.00	0.00	0.20
54442	FLJ20040	0.00	0.13	0.00	0.23
54453	RIN2	0.00	0.00	0.00	NaN
54454	KIAA1240	0.09	0.00	-0.05	0.40
54455	KIAA1332	0.09	0.13	0.10	0.11

54458	DKFZP564J157	0.00	0.00	0.00	NaN
54462	KIAA1128	0.09	0.00	-0.05	0.49
54463	FLJ20152	0.00	0.00	0.10	-0.08
54465	ETAA16	0.00	0.00	0.00	NaN
54468	FLJ20323	0.00	0.00	0.05	0.22
54469	AWP1	0.00	0.00	0.00	NaN
54471	FLJ20232	-0.09	0.00	0.05	0.46
54472	TOLLIP	-0.27	-0.13	-0.05	0.42
54474	KRT20	0.00	0.25	0.00	0.29
54475	FLJ10458	0.00	-0.13	0.00	0.10
54476	TRIAD3	0.00	0.00	0.10	0.43
54477	PEPP2	0.00	0.00	0.00	NaN
54478	FLJ10156	0.00	0.00	0.00	NaN
54480	KIAA1402	0.00	0.00	-0.05	0.31
54482	FLJ10287	0.00	0.00	0.00	NaN
54487	DGCR8	-0.09	0.00	0.00	-0.12
54490	UGT2B28	-0.09	0.00	-0.15	0.00
54491	FLJ11127	0.00	-0.25	0.20	0.38
54494	FLJ20010	-0.09	0.00	0.00	0.26
54496	FLJ10640	-0.09	-0.13	0.00	0.33
54498	C20orf16	0.00	0.00	0.00	NaN
54499	LOC54499	0.00	0.00	0.00	NaN
54502	FLJ20273	0.00	0.00	0.00	NaN
54503	FLJ10852	0.00	0.00	0.00	NaN
54504	CPVL	0.00	0.13	0.00	-0.10
54505	LOC54505	0.00	0.00	-0.05	0.44
54509	ARHF	0.00	-0.13	0.00	0.03
54512	FLJ20591	0.18	0.00	0.10	0.25
54514	VASA	0.00	0.00	0.00	NaN
54517	FLJ20485	0.09	0.00	0.00	0.31
54518	LOC54518	-0.09	0.00	0.00	0.12
54520	FLJ10996	0.00	0.00	0.00	NaN
54529	FLJ20752	0.00	0.00	0.00	NaN
54531	KIAA1193	0.00	0.00	-0.05	0.19
54535	HCR	0.00	0.00	0.00	NaN
54537	MGC5560	0.09	0.00	-0.10	0.73
54540	FLJ10404	0.00	0.00	0.00	NaN
54541	RTP801	0.09	0.00	0.00	0.01
54542	MNAB	0.00	0.00	0.00	NaN
54543	LOC54543	0.00	0.13	0.00	-0.01
54544	NICE-1	0.00	0.00	0.00	NaN
54545	3PAP	0.00	0.00	0.15	0.26
54546	FLJ20225	-0.09	0.00	0.00	-0.10
54549	FLJ10832	0.00	0.00	0.00	NaN
54550	LOC54550	-0.09	-0.13	-0.05	-0.06
54551	MAGEL2	0.00	-0.13	0.00	-0.02
54554	WDR5B	0.00	0.00	0.00	NaN

54555	FLJ10432	0.00	0.00	0.00	NaN
54556	ING3	0.00	0.13	0.00	0.27
54558	SPATA6	0.00	0.00	0.00	NaN
54566	EHM2	0.00	0.00	0.00	NaN
54576	UGT1A8	0.00	0.00	0.00	NaN
54578	UGT1A6	0.00	0.00	0.00	NaN
54581	SCAND2	0.00	0.00	-0.05	0.14
54583	EGLN1	0.09	0.00	0.00	0.29
54585	LZTFL1	-0.09	0.00	-0.05	0.38
54596	FLJ10884	0.00	0.00	0.00	NaN
54600	UGT1A9	0.00	0.00	0.00	NaN
54606	NOH61	0.00	0.00	0.00	NaN
54619	FLJ10895	0.00	0.00	-0.05	0.14
54621	FLJ20674	0.00	-0.13	0.00	0.05
54622	FLJ20051	0.00	0.00	0.00	0.00
54626	HES2	0.00	0.00	0.00	NaN
54629	KIAA1164	0.09	0.00	0.05	0.05
54657	UGT1A4	0.00	0.00	0.00	NaN
54658	UGT1A1	0.00	0.00	0.00	NaN
54659	UGT1A3	0.00	0.00	0.00	NaN
54662	FLJ10743	0.00	0.00	0.00	NaN
54663	FLJ10439	0.00	0.00	0.00	NaN
54664	FLJ11273	0.00	0.13	0.10	0.13
54665	FLJ11220	0.27	0.00	0.05	0.48
54674	LRRN3	0.09	0.00	0.00	0.08
54676	GTPBP2	0.00	0.00	0.00	NaN
54677	CROT	0.00	0.00	0.00	NaN
54680	FLJ20729	0.00	0.00	0.10	0.54
54682	FLJ10298	-0.09	0.00	0.10	-0.11
54704	PDP	0.00	-0.13	0.00	0.43
54707	FLJ10349	-0.09	-0.13	0.00	0.40
54708	FLJ20445	0.00	0.00	0.00	NaN
54714	CNGB3	0.18	0.25	0.00	0.23
54715	A2BP1	0.00	0.00	0.00	NaN
54716	XT3	-0.09	0.00	-0.05	0.07
54718	BTN2A3	0.00	0.00	0.00	NaN
54726	HSHIN1	0.00	0.00	0.00	NaN
54732	HSGP25L2G	0.00	0.00	0.00	NaN
54733	HSNOV1	0.00	0.00	0.00	NaN
54738	HSRNAFEV	0.00	0.00	0.00	NaN
54739	HSXIAPAF1	0.00	0.00	0.00	NaN
54741	HSOBRGRP	0.00	0.00	-0.05	0.31
54752	DKFZp434H22	0.00	-0.13	0.00	-0.43
54758	DKFZp434G05	-0.09	-0.13	-0.05	0.49
54762	DKFZp434C03	0.00	0.00	0.00	NaN
54763	DKFZp434B12	0.00	0.00	0.00	NaN
54765	HSA249128	0.00	0.13	0.05	0.68

54766	BTG4	0.00	0.00	0.00	NaN
54769	DKFZp761C07	0.00	0.00	0.00	NaN
54777	DKFZp434A17	0.00	0.00	-0.05	0.49
54778	DKFZp761D08	0.09	0.00	0.05	0.32
54780	FLJ20003	0.00	0.00	-0.05	0.29
54784	FLJ20013	0.18	0.00	0.00	0.37
54785	FLJ20014	0.00	0.00	0.00	NaN
54788	DNAJB12	0.09	0.00	0.00	0.55
54793	FLJ20038	-0.09	-0.25	-0.05	0.29
54795	TRPM4	0.09	0.00	0.00	-0.06
54796	FLJ20043	0.00	-0.13	-0.15	0.08
54797	FLJ20045	0.00	0.00	0.00	NaN
54798	FLJ20047	0.00	0.00	0.00	0.12
54799	FLJ20055	0.00	0.25	0.00	0.01
54800	FLJ20059	0.09	0.00	0.00	0.12
54801	FLJ20060	0.00	-0.25	-0.10	0.29
54802	IPT	0.00	0.00	0.00	NaN
54805	CNNM2	0.00	-0.13	-0.05	-0.05
54806	FLJ20069	0.00	0.00	0.05	0.04
54807	FLJ20070	0.00	0.13	0.00	0.37
54808	FLJ20071	-0.09	-0.13	-0.15	-0.06
54809	FLJ20073	0.18	0.00	0.00	-0.04
54810	GIPC2	0.00	0.00	0.00	NaN
54811	FLJ20079	0.00	0.00	0.00	NaN
54812	FLJ20080	0.00	0.00	0.00	NaN
54813	FLJ20081	0.00	0.00	0.00	NaN
54814	FLJ20084	0.09	0.00	0.00	0.12
54815	FLJ20085	0.00	0.00	0.00	NaN
54816	FLJ20086	0.00	0.00	0.05	-0.17
54819	FLJ20094	0.00	-0.13	0.00	0.18
54820	NUDE1	0.00	0.00	0.00	NaN
54825	PC-LKC	0.00	0.00	0.00	NaN
54826	FLJ20125	0.00	0.00	0.00	NaN
54827	FLJ20127	-0.09	0.00	0.00	0.04
54828	FLJ20128	0.27	0.13	0.00	0.70
54829	ASPN	0.00	0.00	0.00	NaN
54831	FLJ20132	0.00	0.00	0.00	NaN
54832	FLJ20136	0.00	0.00	0.00	NaN
54834	GDAP2	0.18	0.00	0.05	0.39
54836	FLJ20150	0.00	0.00	0.00	NaN
54838	FLJ20154	0.00	-0.13	-0.05	0.31
54839	FLJ20156	0.00	0.00	0.00	NaN
54840	APTX	-0.09	0.00	0.00	0.56
54842	FLJ20160	0.00	0.00	0.00	NaN
54845	FLJ20171	0.00	-0.13	0.00	0.30
54847	FLJ20174	0.00	0.00	0.00	NaN
54848	FLJ20184	0.00	0.00	0.00	NaN

54849	FLJ20186	-0.09	-0.13	-0.05	0.29
54850	FLJ20188	0.00	0.00	0.05	0.60
54851	FLJ20189	-0.09	0.13	-0.05	0.42
54852	FLJ20190	0.00	0.00	-0.05	-0.09
54853	FLJ20195	0.00	0.00	0.00	NaN
54854	FLJ20200	0.09	0.00	0.00	-0.07
54855	FLJ20202	0.09	0.00	0.05	0.03
54856	FLJ20203	0.09	0.13	0.00	0.37
54858	FLJ20208	0.00	0.00	0.00	NaN
54859	FLJ20211	-0.09	0.00	-0.05	0.07
54860	MS4A12	0.00	0.00	0.00	NaN
54861	FLJ20224	0.00	0.00	-0.05	0.02
54862	FLJ20241	0.00	0.00	0.00	NaN
54863	FLJ20245	0.00	0.00	0.00	NaN
54866	PPP1R14D	0.00	0.00	0.00	NaN
54867	FLJ20254	0.09	0.00	0.05	0.60
54868	FLJ20255	0.00	0.13	0.00	0.07
54869	EPS8R1	0.09	0.00	0.00	0.39
54870	FLJ20259	-0.09	0.00	-0.05	0.50
54872	FLJ20265	0.00	-0.13	-0.10	0.39
54873	PALMD	0.00	0.00	0.00	NaN
54874	FLJ20275	0.09	0.00	0.05	0.38
54875	FLJ20276	0.00	0.00	-0.20	0.31
54876	FLJ20280	0.00	0.00	-0.05	0.03
54877	FLJ20281	-0.09	0.00	-0.15	0.32
54878	DPP8	0.00	0.00	0.00	NaN
54879	ST7L	0.09	0.00	0.05	0.11
54881	FLJ20287	0.00	0.00	0.00	NaN
54882	FLJ20288	0.00	0.00	0.00	NaN
54883	FLJ20291	0.00	0.38	0.00	0.66
54884	FLJ20296	0.00	0.00	0.00	NaN
54886	FLJ20300	0.00	0.00	0.00	NaN
54891	FLJ20309	0.00	0.00	0.00	NaN
54892	FLJ20311	0.00	0.00	-0.05	0.05
54893	FLJ20313	0.00	0.00	0.00	NaN
54894	FLJ20315	0.09	0.00	0.00	0.49
54896	FLJ20320	-0.09	0.00	0.00	0.12
54897	FLJ20321	0.00	0.00	0.00	NaN
54898	ELOVL2	0.00	0.00	0.05	-0.04
54900	FLJ20340	0.00	0.13	0.00	0.02
54901	FLJ20342	0.00	0.00	0.00	NaN
54902	FLJ20343	0.00	0.00	0.00	NaN
54903	FLJ20345	0.09	-0.13	0.00	0.23
54904	WHSC1L1	0.45	-0.13	0.00	0.62
54905	FLJ20359	0.00	0.00	0.00	-0.24
54906	FLJ20360	0.00	0.00	0.05	0.20
54908	FLJ20364	0.00	0.00	0.00	NaN

54910	SEMA4C	0.00	0.00	0.00	NaN
54913	FLJ20374	0.00	0.00	0.00	NaN
54914	FLJ20375	0.00	-0.25	-0.05	0.37
54916	FLJ20392	0.00	0.00	0.05	-0.02
54918	FLJ20396	0.00	-0.13	0.00	0.44
54919	FLJ20397	0.00	0.13	0.00	0.53
54920	FLJ20399	-0.09	-0.13	0.00	0.31
54922	FLJ20401	0.09	0.00	0.00	0.03
54923	FLJ20406	0.09	0.00	0.00	0.11
54925	FLJ20417	0.00	0.00	0.00	NaN
54927	FLJ20420	0.00	-0.13	0.00	0.28
54928	FLJ20421	0.00	0.00	0.00	NaN
54929	FLJ20422	0.00	0.00	0.00	NaN
54930	FLJ20424	0.00	0.00	0.00	NaN
54932	FLJ20433	0.00	0.00	0.00	NaN
54933	FLJ20435	0.00	0.00	0.00	NaN
54934	FLJ20436	0.00	0.00	0.00	NaN
54937	FLJ20449	0.00	0.00	0.00	NaN
54938	FLJ20450	0.00	0.00	0.00	NaN
54939	FLJ20452	0.00	0.00	0.00	NaN
54941	FLJ20456	0.00	0.00	-0.15	0.26
54942	FLJ20457	0.00	0.00	0.00	NaN
54943	C21orf55	0.00	0.00	0.00	NaN
54946	FLJ20473	0.00	0.00	0.00	NaN
54948	MRPL16	0.00	0.00	0.00	NaN
54951	FLJ20502	0.00	0.00	0.00	NaN
54952	SECP43	0.00	0.00	0.00	NaN
54953	C1orf27	0.00	0.00	0.00	NaN
54955	FLJ20508	0.00	0.00	0.00	NaN
54956	FLJ20509	0.00	0.00	0.00	NaN
54957	FLJ20511	0.00	0.00	0.05	0.25
54958	FLJ20512	0.09	0.00	0.00	0.23
54959	FLJ20513	0.00	0.00	0.00	NaN
54961	SSH-3	0.45	0.00	0.05	0.56
54962	FLJ20516	0.00	0.00	0.00	NaN
54963	URKL1	0.00	0.13	0.00	-0.45
54964	FLJ20519	0.00	0.00	0.05	0.20
54968	FLJ20533	0.00	0.25	0.00	0.46
54969	FLJ20534	0.00	0.13	-0.05	0.06
54970	FLJ20535	0.00	0.00	0.00	NaN
54971	BANP	-0.09	-0.13	-0.05	0.30
54972	FLJ20539	0.00	0.00	0.00	NaN
54974	FLJ20546	0.00	0.00	0.00	NaN
54976	C20orf27	0.00	0.00	0.00	NaN
54977	FLJ20551	0.00	0.00	-0.05	0.18
54978	FLJ20555	0.09	0.00	0.05	0.28
54979	HRASLS2	0.00	0.00	0.00	NaN

54980	FLJ20558	0.00	0.00	0.00	NaN
54981	FLJ20559	0.00	0.00	-0.05	0.04
54982	CLN6	0.00	0.00	0.00	NaN
54985	FLJ20568	0.00	0.00	0.00	NaN
54986	FLJ20574	0.00	0.00	-0.05	0.17
54987	FLJ20580	0.00	0.00	0.00	NaN
54988	FLJ20581	0.00	0.00	0.00	NaN
54991	FLJ20584	0.00	0.00	0.00	NaN
54993	FLJ20595	0.00	0.00	-0.05	0.04
54994	C20orf11	0.09	0.13	0.00	0.17
54995	FLJ20604	0.00	0.00	0.00	NaN
54996	FLJ20605	0.09	0.13	0.05	0.22
54997	TSC	0.00	-0.13	0.00	-0.25
54998	FLJ20608	0.00	0.00	0.00	NaN
55001	FLJ20619	0.00	0.00	0.00	NaN
55002	FLJ20623	-0.18	-0.13	-0.05	0.19
55003	FLJ20624	0.00	0.00	0.05	0.28
55005	FLJ20627	0.00	0.00	0.00	NaN
55007	FLJ20635	0.00	0.00	0.00	NaN
55008	FLJ20637	0.00	0.00	0.00	NaN
55009	FLJ20640	0.00	0.00	0.00	NaN
55010	FLJ20641	0.00	0.00	0.00	NaN
55011	FLJ20643	0.00	0.00	0.00	NaN
55012	FLJ20644	0.00	0.00	0.00	NaN
55013	FLJ20647	0.00	0.00	0.00	NaN
55014	FLJ20651	0.00	0.00	0.00	NaN
55015	FLJ20666	0.00	0.00	0.00	NaN
55016	FLJ20668	0.00	0.00	0.00	NaN
55018	FLJ20694	0.00	0.38	0.00	0.17
55020	FLJ20699	0.00	0.00	0.00	NaN
55022	FLJ20701	0.00	0.00	0.00	NaN
55023	PHIP	0.00	0.00	0.00	NaN
55024	BANK	0.00	0.00	0.00	NaN
55027	FLJ20718	0.00	0.00	-0.05	0.31
55028	FLJ20721	0.00	0.00	0.00	NaN
55030	FLJ20725	0.00	0.00	0.05	0.28
55031	FLJ20727	0.00	0.00	0.00	NaN
55032	FLJ20730	0.00	0.00	0.00	NaN
55033	FLJ20731	0.00	0.13	0.00	-0.27
55034	HMCS	0.00	-0.13	-0.25	0.22
55035	FLJ20736	0.00	0.00	0.00	NaN
55036	FLJ20753	0.00	0.25	0.00	0.27
55037	FLJ20758	0.00	0.00	-0.05	0.26
55038	CDCA4	0.00	0.00	0.00	NaN
55039	FLJ20772	0.18	0.38	-0.05	0.65
55040	EPN3	0.00	0.25	0.00	0.55
55041	FLJ20783	0.00	0.00	-0.05	0.10

55048	FLJ20847	0.00	0.00	0.00	NaN
55049	FLJ20850	0.00	0.00	0.00	NaN
55051	FLJ10008	0.00	0.13	0.00	0.03
55052	MRPL20	0.00	0.00	0.00	NaN
55054	FLJ10035	0.00	0.00	0.00	NaN
55055	FLJ10036	0.00	0.00	0.00	NaN
55056	FLJ10038	0.18	0.00	0.00	0.56
55057	FLJ10040	-0.09	0.00	0.00	0.14
55061	FLJ10052	0.09	0.00	0.00	0.27
55062	FLJ10055	0.18	0.13	0.00	-0.03
55063	FLJ10057	0.18	0.00	0.00	0.31
55064	FLJ10058	-0.09	-0.13	-0.15	-0.21
55065	FLJ10060	0.00	0.00	0.00	-0.17
55066	FLJ10079	-0.09	-0.13	0.05	0.09
55068	FLJ10094	0.00	0.00	-0.05	-0.04
55069	FLJ10099	-0.09	0.00	0.00	0.47
55070	FLJ10103	0.00	0.00	-0.05	0.39
55071	FLJ10110	0.00	0.00	-0.05	-0.12
55072	FLJ10111	0.00	0.00	0.00	NaN
55073	FLJ10120	-0.09	0.25	0.05	0.02
55074	OXR1	0.18	-0.13	0.00	0.43
55076	FLJ10134	0.00	0.00	0.00	NaN
55079	FLJ10142	0.09	0.00	-0.05	-0.29
55080	FLJ10143	-0.09	0.00	0.00	0.18
55081	ESRRBL1	0.00	0.00	0.00	NaN
55082	FLJ10154	-0.09	-0.13	0.00	0.41
55083	FLJ10157	0.00	0.13	0.00	-0.12
55084	FLJ10159	0.00	0.00	0.10	-0.08
55088	FLJ10188	0.00	0.00	-0.05	0.16
55089	SLC38A4	0.00	0.00	0.00	NaN
55090	FLJ10193	0.00	0.00	0.00	NaN
55092	FLJ10199	0.00	0.00	0.00	NaN
55093	FLJ10204	0.27	0.25	0.05	0.47
55094	FLJ10206	0.00	0.00	0.05	0.28
55095	FLJ10211	0.00	0.00	0.00	NaN
55096	FLJ10213	-0.09	0.00	-0.05	0.23
55100	FLJ10233	0.00	0.00	0.05	0.32
55101	FLJ10241	0.00	0.00	-0.05	0.34
55102	FLJ10242	0.00	0.00	0.00	NaN
55103	FLJ10244	0.00	0.00	0.00	NaN
55105	FLJ10252	0.09	0.00	0.00	-0.15
55106	FLJ10260	0.00	0.00	0.00	-0.13
55107	FLJ10261	0.55	0.25	0.00	0.39
55108	FLJ10276	0.00	0.00	0.00	NaN
55109	FLJ10283	0.00	0.00	0.00	NaN
55110	FLJ10292	-0.18	0.00	0.00	0.30
55111	FLJ10297	-0.09	0.00	0.00	-0.04

55112	FLJ10300	0.00	0.00	-0.05	0.31
55113	FLJ10307	0.00	0.00	0.00	NaN
55114	RICH1	0.00	0.00	0.00	NaN
55116	FLJ10315	0.00	0.00	0.00	NaN
55117	NTT73	0.00	0.13	0.00	-0.08
55118	CRTAC1	0.00	0.00	-0.05	0.02
55119	FLJ10330	0.00	0.00	0.00	NaN
55120	FLJ10335	0.00	0.00	0.00	NaN
55122	FLJ10342	0.00	0.00	0.00	NaN
55124	PIWIL2	-0.09	-0.25	-0.10	-0.10
55127	FLJ10359	0.09	0.13	0.00	0.35
55128	SS-56	0.00	-0.13	0.00	0.01
55129	FLJ10375	0.00	0.00	-0.05	0.31
55130	FLJ10376	-0.09	0.00	0.00	-0.10
55131	FLJ10377	0.00	0.25	0.00	0.51
55133	FLJ10379	0.00	0.00	0.00	NaN
55135	FLJ10385	0.00	0.00	0.05	0.39
55138	FLJ10408	-0.09	0.00	0.00	-0.14
55139	FLJ10415	0.00	0.00	0.00	NaN
55140	FLJ10422	-0.09	-0.50	-0.10	0.49
55142	FLJ10460	0.00	0.00	0.00	NaN
55143	FLJ10468	0.00	0.00	0.00	NaN
55144	LRRC5	0.00	0.00	0.05	0.16
55145	FLJ10477	0.09	0.00	-0.05	0.39
55146	ZDHHC4	0.00	0.00	0.10	0.39
55147	FLJ10482	0.00	0.00	0.00	NaN
55148	FLJ10483	0.00	0.00	0.00	NaN
55149	FLJ10486	-0.09	-0.13	0.05	0.54
55150	FLJ10490	0.09	0.00	0.00	0.07
55151	FLJ10493	0.00	0.00	0.00	NaN
55152	FLJ10496	-0.09	0.00	-0.05	0.09
55153	FLJ10498	0.00	0.00	0.00	NaN
55154	FLJ10504	0.09	0.13	0.00	0.32
55156	FLJ10511	0.00	0.00	0.05	0.30
55157	FLJ10514	0.00	0.00	0.00	NaN
55159	FLJ10520	0.00	0.00	0.05	0.46
55160	FLJ10521	-0.09	0.00	0.00	0.03
55161	FLJ10525	0.00	0.00	0.00	NaN
55163	FLJ10535	0.00	0.00	0.00	0.19
55164	FLJ10539	-0.09	0.00	-0.05	0.30
55165	FLJ10540	0.00	0.00	0.00	NaN
55166	FLJ10545	0.00	0.00	0.00	NaN
55167	FLJ10546	0.00	0.00	0.00	NaN
55168	MRPS18A	0.00	0.00	0.00	NaN
55171	FLJ10560	0.09	0.00	0.05	0.62
55172	FLJ10563	0.00	0.00	0.00	NaN
55173	MRPS10	0.00	0.00	0.05	0.26

55175	FLJ10572	0.00	0.13	0.00	0.18
55176	FLJ10578	0.00	0.00	0.00	NaN
55177	FLJ10579	0.00	0.00	0.00	NaN
55178	FLJ10581	0.00	-0.13	0.00	0.07
55179	FLJ10582	0.00	0.00	0.00	NaN
55180	FLJ10583	0.00	0.00	0.00	NaN
55181	FLJ10587	0.18	0.13	0.00	0.68
55182	FLJ10597	0.00	0.00	0.00	NaN
55184	C20orf12	0.00	0.00	0.00	NaN
55186	FLJ10618	0.00	0.00	0.00	NaN
55187	FLJ10619	0.00	0.00	0.00	NaN
55188	FLJ10620	0.00	0.00	0.00	NaN
55191	FLJ10631	0.00	0.00	0.00	0.46
55192	FLJ10634	0.00	0.00	0.00	NaN
55193	PB1	-0.09	-0.25	-0.10	0.47
55194	FLJ10647	0.00	0.00	0.00	NaN
55195	FLJ10650	0.00	0.00	0.00	NaN
55196	FLJ10652	0.00	0.00	0.00	NaN
55197	FLJ10656	0.09	-0.13	-0.25	0.42
55200	FLJ10665	-0.09	0.00	0.00	-0.01
55201	C19orf5	-0.09	0.00	0.00	0.15
55203	FLJ10675	0.00	0.00	-0.05	-0.13
55204	FLJ10687	0.00	0.13	0.05	0.51
55205	FLJ10697	-0.09	0.00	-0.15	0.43
55206	FLJ10701	0.00	0.00	0.00	NaN
55207	FLJ10702	0.00	0.00	0.05	0.06
55208	FLJ10704	-0.09	-0.13	-0.05	0.21
55209	FLJ10707	0.00	0.00	0.00	NaN
55210	FLJ10709	0.00	0.13	0.00	-0.29
55211	FLJ10713	0.00	0.00	0.00	NaN
55212	FLJ10715	0.00	0.00	0.00	NaN
55213	FLJ10716	0.00	0.13	0.00	-0.16
55214	FLJ10718	0.00	0.00	0.05	0.25
55215	FLJ10719	0.00	0.00	-0.05	0.51
55216	FLJ10726	0.00	0.00	0.00	NaN
55218	FLJ10738	0.00	0.00	0.00	NaN
55219	FLJ10747	-0.09	0.00	0.00	-0.06
55220	FLJ10748	0.00	0.13	0.00	-0.04
55222	FLJ10751	0.00	0.00	0.00	NaN
55223	FLJ10759	0.00	0.00	0.00	NaN
55224	FLJ10761	0.00	0.13	0.00	0.30
55225	KIAA1579	0.00	0.00	-0.05	0.18
55226	FLJ10774	0.00	0.00	0.05	0.48
55227	LANO	0.00	0.00	0.00	NaN
55228	FLJ10781	0.09	0.13	0.00	0.03
55229	FLJ10782	0.09	0.00	0.00	-0.07
55231	FLJ10786	0.00	0.00	0.05	0.26

55233	C2orf6	0.00	0.00	0.00	NaN
55234	SMU-1	-0.09	0.00	0.05	0.22
55236	FLJ10808	0.00	0.00	0.00	NaN
55237	FLJ10811	0.00	0.00	0.00	NaN
55238	FLJ10815	0.00	0.00	0.00	NaN
55239	FLJ10826	0.00	0.00	0.00	NaN
55240	FLJ10829	0.00	0.00	0.00	NaN
55243	KIRREL	0.00	0.00	0.00	NaN
55244	FLJ10847	0.00	-0.13	0.00	0.11
55245	C20orf44	0.00	0.13	0.00	0.35
55246	FLJ10853	-0.09	-0.25	-0.10	0.42
55247	FLJ10858	0.00	0.00	-0.10	0.19
55248	FLJ10874	0.00	0.00	0.00	NaN
55249	YAP	0.50	0.50	0.00	0.38
55252	FLJ10898	0.09	0.00	0.00	0.49
55253	FLJ10900	-0.09	0.00	0.00	0.29
55254	FLJ10902	0.00	0.00	0.00	NaN
55255	FLJ10904	0.00	0.00	0.00	NaN
55256	SIPL	0.00	0.00	0.00	NaN
55257	C20orf20	0.09	0.13	0.00	0.00
55258	FLJ10916	0.00	0.00	-0.05	0.27
55259	FLJ10921	0.00	0.00	0.00	NaN
55260	FLJ10922	0.09	0.00	0.00	0.23
55262	FLJ10925	0.18	0.00	0.00	0.29
55266	FLJ10936	0.09	0.00	0.00	0.12
55267	FLJ10945	0.00	0.00	0.00	NaN
55268	FLJ10948	0.00	0.00	0.00	NaN
55269	PSP1	0.00	0.00	-0.15	0.46
55270	FLJ10956	0.00	0.00	0.00	NaN
55273	FLJ10970	0.00	0.00	0.00	-0.33
55274	XAP135	0.00	0.00	0.00	NaN
55275	FLJ10979	0.00	-0.13	0.00	0.15
55277	FLJ10986	0.00	0.00	-0.05	0.03
55278	FLJ10989	0.00	0.00	0.10	0.64
55279	FLJ10997	-0.09	0.00	0.05	0.36
55280	FLJ10998	0.00	-0.13	-0.05	0.40
55281	FLJ11000	0.00	0.00	0.00	NaN
55282	FLJ11004	-0.09	0.00	0.05	0.47
55283	MCOLN3	0.00	0.00	0.05	-0.04
55284	FLJ11011	0.00	0.25	0.00	0.35
55286	FLJ11017	0.00	0.13	0.00	0.16
55287	FLJ11036	0.00	0.00	0.00	NaN
55288	FLJ11040	0.00	0.00	0.00	NaN
55289	FLJ11042	0.00	0.00	0.00	NaN
55291	C11orf23	0.36	0.00	0.00	0.60
55293	FLJ11068	0.00	0.00	0.00	NaN
55294	FBXW7	0.00	0.00	0.50	0.29

55295	FLJ11078	0.00	0.00	0.00	NaN
55296	FLJ11082	0.00	0.00	-0.05	0.11
55297	FLJ11088	0.00	0.00	-0.05	0.25
55298	FLJ11099	0.00	-0.13	0.00	0.05
55299	BRIX	0.00	0.00	0.15	0.40
55301	FLJ11106	0.00	0.00	0.00	NaN
55303	HIMAP4	0.00	0.00	-0.05	-0.05
55304	C20orf38	0.00	0.00	0.00	NaN
55308	FLJ11126	-0.09	-0.13	0.05	0.53
55311	FLJ11137	0.09	0.13	0.00	0.35
55312	FLJ11149	0.00	0.00	-0.05	0.15
55313	FLJ11151	0.00	0.00	0.00	NaN
55314	FLJ11155	0.00	0.00	0.00	0.00
55315	FLJ11160	0.09	0.00	0.00	-0.01
55316	FLJ11164	0.00	0.25	0.00	0.43
55317	C20orf29	0.00	0.00	-0.05	0.22
55319	FLJ11184	0.00	0.00	0.00	NaN
55320	FLJ11186	0.00	0.00	0.00	NaN
55321	C20orf46	0.00	0.00	0.00	NaN
55322	FLJ11193	0.00	0.00	0.15	0.43
55323	FLJ11196	0.00	0.00	0.00	NaN
55324	FLJ11198	0.09	0.00	0.00	0.47
55325	FLJ11200	0.00	0.00	-0.05	0.26
55326	FLJ11210	-0.09	-0.38	-0.10	0.40
55327	LIN-7-C	0.00	0.00	0.00	NaN
55328	FLJ11218	0.09	0.00	-0.05	0.17
55329	FLJ11222	0.00	0.00	0.05	0.02
55332	FLJ11259	0.00	0.00	0.00	NaN
55333	FLJ11271	0.00	0.00	0.00	NaN
55334	FLJ11274	0.00	0.00	0.00	NaN
55335	FLJ11275	0.00	0.00	0.00	NaN
55336	FBXL8	-0.09	0.00	0.00	0.30
55337	FLJ11286	0.00	0.00	0.05	0.62
55339	FLJ11294	0.00	0.00	-0.05	0.28
55340	IAN4L1	0.00	0.00	-0.05	0.19
55341	FLJ11301	0.09	0.00	0.00	0.44
55343	FLJ11320	0.09	0.00	0.00	0.17
55346	FLJ11336	0.00	0.00	0.00	NaN
55347	FLJ11342	0.00	0.00	0.00	NaN
55350	VNN3	0.00	0.00	0.00	NaN
55351	HSA250839	0.00	-0.13	-0.15	-0.27
55353	LC27	0.00	0.00	0.00	NaN
55355	DKFZp762E13	0.00	0.00	0.00	NaN
55357	TBC1D2	0.00	0.00	0.00	NaN
55359	DKFZp761P10	-0.09	0.00	0.00	0.11
55361	PI4KII	0.00	0.00	-0.05	0.06
55364	IMPACT	0.00	0.00	-0.15	0.42

55365	HCA112	0.00	0.00	-0.05	0.10
55366	GPR48	0.00	0.00	0.00	NaN
55367	LRDD	-0.18	-0.13	-0.05	0.14
55374	PRO1580	0.00	0.00	0.00	NaN
55384	MEG3	0.00	0.00	0.00	NaN
55388	MCM10	0.00	0.00	0.00	NaN
55389	PRO2266	0.00	0.00	0.00	NaN
55421	HSA277841	0.00	0.00	0.00	NaN
55422	ZNF361	0.09	0.00	-0.05	0.52
55423	SIRPB2	0.00	0.00	0.50	-0.23
55425	LSR7	0.00	0.00	-0.05	0.17
55435	PRO0971	0.00	0.00	0.00	NaN
55450	PRO1489	-0.09	0.00	0.00	0.01
55454	PRO0082	0.00	0.00	0.00	NaN
55466	DNAJA4	0.00	0.00	0.00	NaN
55471	PRO1853	0.00	0.00	0.00	NaN
55472	PRO1905	0.00	-0.13	0.00	-0.22
55486	PARL	0.09	0.00	0.00	0.42
55500	EKI1	0.00	0.00	0.00	NaN
55501	C4S-2	0.00	0.13	0.05	0.37
55505	NOLA3	0.00	0.00	0.00	NaN
55506	H2AFY2	0.00	0.00	0.00	NaN
55507	GPRC5D	-0.09	0.00	0.00	-0.18
55508	BLOV1	0.09	0.13	0.00	0.57
55509	SNFT	0.00	0.00	0.00	NaN
55510	HAGE	0.00	0.00	0.05	0.27
55515	ASIC4	0.00	0.00	0.00	NaN
55520	ELAC1	-0.09	-0.13	-0.25	0.12
55521	TRIM36	0.00	0.00	0.00	NaN
55526	KIAA1630	0.00	0.00	0.00	NaN
55532	DKFZp547M23	0.09	0.00	0.05	0.05
55539	HSA404617	0.00	-0.13	-0.05	0.03
55540	IL17BR	0.00	0.00	-0.10	0.21
55554	KLK15	0.00	-0.13	0.00	-0.59
55556	HSRTSBETA	0.00	0.00	0.00	NaN
55565	LOC55565	0.00	0.00	0.05	0.01
55568	DKFZp586H06	0.00	0.00	0.00	NaN
55573	H41	0.00	0.00	0.00	NaN
55576	STAB2	0.00	0.00	0.00	NaN
55577	NAGK	0.00	0.00	0.00	NaN
55578	P38IP	0.00	0.00	0.00	NaN
55584	CHRNA9	0.00	0.00	0.00	NaN
55585	HSA243666	0.09	0.25	0.00	0.38
55589	BIKE	0.00	0.00	0.00	NaN
55591	VEZATIN	0.00	0.13	0.05	-0.10
55592	DKFZp434M03	0.00	0.00	0.00	NaN
55596	DKFZp434E22	0.00	-0.13	0.00	0.31

55601	FLJ20035	0.00	0.00	-0.05	0.09
55602	FLJ20036	0.00	0.00	-0.05	0.18
55603	FLJ20037	0.00	0.00	0.00	NaN
55604	FLJ20048	0.00	0.00	0.05	-0.09
55607	PPP1R9A	0.18	0.00	0.00	0.22
55608	FLJ20093	-0.09	-0.13	0.00	0.21
55610	FLJ20097	0.18	0.00	0.00	0.30
55611	FLJ20113	0.00	0.00	0.00	NaN
55612	C20orf42	0.00	0.00	0.00	NaN
55616	UPLC1	-0.09	0.00	0.00	0.13
55617	C20orf13	0.00	0.00	0.00	NaN
55619	FLJ20220	0.00	0.00	0.00	NaN
55620	FLJ20234	0.00	0.00	0.00	NaN
55621	FLJ20244	0.00	0.00	0.00	NaN
55622	FLJ20272	0.00	0.00	0.00	NaN
55623	FLJ20274	0.00	0.00	0.00	NaN
55624	FLJ20277	0.00	0.00	0.00	NaN
55625	ZDHHC7	-0.09	0.00	-0.05	0.41
55626	FLJ20294	0.00	0.00	0.00	NaN
55627	FLJ20297	0.00	0.00	-0.05	0.55
55628	FLJ20307	-0.09	0.00	-0.25	0.34
55629	FLJ20312	-0.09	0.00	0.00	0.21
55630	SLC39A4	0.18	0.13	0.15	0.35
55631	FLJ20331	0.00	0.00	0.00	NaN
55635	FLJ20354	0.00	0.00	0.00	NaN
55638	FLJ20366	0.18	0.00	0.05	0.19
55640	FLJ20371	0.00	0.00	0.00	NaN
55643	BTBD2	0.00	0.00	0.00	NaN
55644	OSGEP	0.00	0.00	0.00	NaN
55647	RAB20	-0.09	-0.13	0.00	0.02
55650	FLJ20477	-0.09	-0.13	0.05	0.06
55651	NOLA2	0.18	0.00	0.00	0.46
55652	FLJ20489	0.00	0.00	0.00	NaN
55653	BCAS4	0.18	0.13	0.00	0.43
55654	FLJ20507	0.00	0.00	0.00	NaN
55655	NALP2	0.09	0.00	0.00	0.08
55656	FLJ20530	0.00	-0.13	0.00	0.62
55657	FLJ20531	0.00	0.00	0.00	NaN
55658	FLJ20552	0.00	0.00	-0.05	0.16
55660	FNBP3	0.00	0.00	0.00	NaN
55661	DDX27	0.09	0.00	0.05	0.05
55662	HIF1AN	0.00	-0.13	-0.05	0.17
55663	FLJ20626	0.00	0.13	0.10	0.32
55664	HARC	-0.09	-0.13	-0.15	0.63
55665	URG4	0.00	0.00	0.00	NaN
55666	NPL4	0.00	0.00	0.00	NaN
55667	FLJ20686	0.00	-0.25	-0.10	0.25

55668	FLJ20689	0.00	0.00	0.00	NaN
55669	MFN1	0.09	0.00	0.05	0.43
55670	FLJ20695	0.09	0.00	0.00	0.41
55671	FLJ20707	0.00	0.13	0.00	0.07
55672	FLJ20719	0.09	0.00	-0.05	0.43
55679	FLJ10044	0.00	0.00	-0.05	0.00
55680	Rabip4R	0.00	0.00	0.00	NaN
55683	FLJ10081	0.00	0.00	0.00	NaN
55684	FLJ10101	0.00	0.00	0.00	NaN
55686	FLJ10116	0.00	0.00	0.00	NaN
55689	FLJ10201	0.09	0.00	0.00	-0.08
55691	FLJ10210	0.00	0.00	0.00	NaN
55692	LUC7L	0.00	0.00	-0.05	0.10
55693	FLJ10251	-0.09	0.13	-0.10	0.23
55695	NOL1R	-0.09	0.00	0.00	0.28
55696	FLJ10290	0.00	0.00	0.00	NaN
55697	FLJ10305	0.09	-0.13	0.05	0.41
55699	FLJ10326	0.09	0.00	0.05	0.47
55700	FLJ10350	0.00	0.00	0.00	NaN
55701	FLJ10357	0.00	0.00	0.00	NaN
55702	FLJ10374	0.00	0.00	0.00	NaN
55703	FLJ10388	0.00	0.00	0.00	NaN
55704	FLJ10392	0.09	0.00	0.00	-0.26
55705	FLJ10402	0.00	0.00	0.00	NaN
55706	FLJ10407	0.00	0.00	0.00	NaN
55707	FLJ10420	0.00	0.00	0.00	NaN
55709	FLJ10450	0.00	0.00	0.00	NaN
55711	FLJ10462	0.00	0.00	0.00	NaN
55713	ZNF334	0.00	0.00	0.00	NaN
55715	DOK4	-0.09	0.00	0.00	0.12
55716	LIMR	0.00	0.00	0.00	NaN
55718	FLJ10509	0.00	0.00	0.00	NaN
55719	FLJ10512	0.00	-0.13	-0.05	0.26
55720	FLJ10534	0.00	-0.13	0.00	0.13
55721	FLJ10547	0.00	0.00	0.00	NaN
55722	FLJ10565	0.00	0.13	0.10	0.54
55723	FLJ10604	0.00	0.00	0.00	NaN
55726	FLJ10637	0.00	0.00	0.00	NaN
55727	FLJ10648	0.00	0.00	0.00	NaN
55729	FLJ10688	-0.09	0.00	0.00	0.09
55731	FLJ10700	0.00	0.38	0.00	0.57
55732	FLJ10706	0.00	0.00	0.00	NaN
55733	FLJ10724	0.00	0.00	0.00	NaN
55734	ZFP64	0.00	0.13	0.05	0.36
55735	FLJ10737	0.00	0.00	0.00	NaN
55737	VPS35	-0.09	0.00	-0.05	0.29
55739	FLJ10769	-0.09	-0.13	0.00	0.46

55740	FLJ10773	0.09	0.00	0.00	0.14
55741	C20orf31	0.00	0.13	0.00	0.43
55742	PARVA	0.00	0.00	0.00	NaN
55743	CHFR	0.00	0.00	0.00	NaN
55744	FLJ10803	0.00	0.00	0.00	NaN
55745	FLJ10813	0.00	0.00	0.05	0.16
55746	NUP133	0.09	0.00	0.00	0.27
55748	FLJ10830	-0.09	0.00	-0.25	0.61
55750	FLJ10842	0.00	0.00	0.00	NaN
55751	FLJ10846	0.00	0.13	0.00	0.55
55752	FLJ10849	0.00	0.00	0.00	NaN
55753	FLJ10851	0.00	0.00	0.00	NaN
55754	FLJ10856	0.00	0.00	0.05	0.19
55755	C48	0.00	0.00	0.00	NaN
55756	FLJ10871	-0.09	-0.25	-0.05	0.40
55757	UGCGL2	-0.09	0.00	-0.05	0.21
55758	FLJ10876	0.00	0.00	0.00	NaN
55759	WDR12	0.00	0.00	0.00	NaN
55760	DDX32	0.00	0.00	-0.05	0.26
55761	FLJ10890	0.00	0.00	0.00	NaN
55762	FLJ10891	0.09	0.00	-0.05	0.27
55764	WDR10	0.00	0.00	0.00	NaN
55765	FLJ10901	0.00	0.00	0.00	NaN
55768	FLJ11005	0.00	0.00	0.00	NaN
55769	ZNF83	0.09	0.00	-0.05	0.36
55770	FLJ11026	0.00	-0.13	-0.10	0.39
55771	FLJ11029	0.18	0.13	0.00	0.48
55775	TDP1	0.00	0.13	0.00	0.27
55776	FLJ11101	0.00	0.00	0.00	NaN
55777	FLJ11113	0.00	0.00	0.00	NaN
55778	FLJ11132	0.00	0.00	0.00	NaN
55779	FLJ11142	0.00	0.00	0.00	NaN
55781	FLJ11159	0.00	0.00	0.00	NaN
55784	FLJ11175	0.00	0.00	0.00	NaN
55785	FLJ11183	0.00	0.13	0.00	0.09
55786	FLJ11191	0.09	0.00	-0.05	0.35
55788	FLJ11240	0.00	0.00	0.05	0.14
55790	ChGn	0.00	-0.13	-0.05	0.01
55791	FLJ11269	0.09	0.00	0.00	0.05
55793	FLJ11280	0.00	0.00	0.05	-0.11
55794	DDX28	-0.09	-0.13	0.00	0.33
55795	FLJ11305	-0.09	-0.13	-0.05	0.40
55798	METL	0.50	0.50	0.00	0.09
55799	CACNA2D3	0.00	0.00	-0.15	0.17
55800	HSA243396	0.00	0.00	-0.05	0.13
55801	IL26	0.09	0.00	-0.05	0.03
55802	HSA275986	0.00	0.00	-0.10	0.40

55803	CENTA2	0.00	0.00	0.00	NaN
55805	DKFZp761O01	0.00	0.00	-0.05	0.04
55806	HR	-0.09	-0.25	-0.10	0.25
55810	FHX	-0.09	0.00	0.00	0.37
55811	SAC	0.00	0.00	0.00	NaN
55812	HSD-3.1	0.00	0.13	0.00	-0.17
55813	HCA66	0.00	0.00	0.00	NaN
55816	C20orf180	0.18	0.50	0.00	0.33
55818	TSGA	0.00	0.00	-0.05	0.18
55819	GP	0.00	0.00	0.00	NaN
55821	ALLC	0.00	0.00	0.00	NaN
55823	VPS11	0.00	0.00	-0.05	0.28
55825	HSA250303	0.00	0.00	0.00	NaN
55827	PC326	0.00	0.00	0.00	NaN
55830	AD-017	-0.09	0.00	-0.10	0.45
55831	LOC55831	0.00	0.00	0.00	NaN
55832	TIP120A	0.09	0.00	0.00	0.49
55833	LOC55833	-0.09	0.13	0.10	0.67
55835	CENPJ	0.00	-0.13	-0.15	0.44
55837	BM036	0.00	0.00	0.00	NaN
55839	BM039	0.00	0.00	-0.05	0.15
55840	BM040	0.00	0.00	0.05	0.16
55843	BM046	0.00	0.00	0.00	NaN
55844	MDS026	0.00	0.00	-0.05	0.29
55846	MDS028	0.00	0.00	0.05	0.27
55847	MDS029	0.00	0.00	0.00	NaN
55848	MDS030	-0.09	-0.13	-0.05	0.57
55850	MDS032	-0.09	0.00	0.00	0.21
55851	MDS033	0.00	0.00	0.00	NaN
55852	HT008	0.27	0.13	0.00	0.71
55853	HT009	0.00	0.00	0.00	NaN
55854	HT010	0.00	0.00	0.00	NaN
55855	HT011	0.00	0.00	-0.05	0.31
55856	HT012	0.00	0.00	0.05	0.60
55857	C20orf19	0.00	0.00	0.00	NaN
55858	TPARL	0.00	0.00	0.00	NaN
55861	C20orf35	0.00	0.00	0.00	NaN
55862	LOC55862	0.00	0.00	0.05	0.14
55863	HT007	0.00	0.13	0.00	0.18
55867	SLC22A11	0.00	0.00	0.00	NaN
55870	ASH1	0.09	0.13	0.00	0.26
55872	TOPK	-0.09	-0.25	-0.10	0.61
55876	PRO2521	0.00	0.88	0.00	0.71
55884	LOC55884	0.00	-0.13	0.00	0.39
55888	ZFP	-0.09	0.00	-0.05	-0.06
55889	GLP	0.00	0.00	0.00	NaN
55890	GPRC5C	0.00	0.13	0.00	0.35

55891	LENEP	0.09	0.13	0.00	0.05
55892	MYNN	0.09	0.00	0.00	0.32
55893	LOC55893	-0.09	-0.50	-0.10	0.36
55898	IRO039700	0.00	0.00	0.50	0.00
55900	ZNF302	0.00	0.00	0.05	0.16
55901	LOC55901	0.00	0.00	0.00	NaN
55905	ZNF313	0.27	0.25	0.10	0.48
55907	CMAS	0.00	0.00	0.00	NaN
55908	LOC55908	0.09	0.00	0.00	-0.17
55909	BIN3	-0.09	-0.25	-0.15	0.38
55911	APOB48R	0.00	0.00	0.00	NaN
55914	ERBB2IP	0.00	0.00	-0.05	0.04
55915	LANCL2	0.00	0.00	0.10	0.80
55917	DKFZp547A02	0.09	0.00	0.05	0.04
55924	LOC55924	0.09	0.00	0.00	0.26
55930	MYO5C	0.18	0.00	0.00	0.37
55937	G3A	0.00	0.00	0.00	NaN
55954	LOC55954	-0.09	0.00	0.00	0.06
55957	F25965	0.00	0.00	0.00	NaN
55958	KIAA1354	0.00	-0.13	-0.15	0.66
55966	MOT8	0.00	0.00	0.00	NaN
55968	NSFL1C	0.00	0.00	0.00	NaN
55969	C20orf24	0.00	-0.13	0.00	0.47
55972	MCFP	0.00	0.00	0.00	NaN
55973	BAP29	0.09	0.00	0.00	0.13
55974	LOC55974	0.27	0.13	0.00	0.67
55975	SBBI26	0.50	0.50	0.00	-0.05
56005	IL27	0.00	0.00	0.00	NaN
56006	FLJ12886	0.09	0.00	-0.10	0.39
56033	BARX1	0.00	0.00	0.00	NaN
56034	PDGFC	0.00	0.00	0.00	0.06
56052	HMT-1	0.00	0.00	0.00	NaN
56061	UBPH	0.00	0.00	-0.05	0.03
56100	PCDHGB6	0.00	0.00	0.00	NaN
56101	PCDHGB5	0.00	0.00	0.00	NaN
56105	PCDHGA11	0.00	0.00	0.00	NaN
56106	PCDHGA10	0.00	0.00	0.00	NaN
56107	PCDHGA9	0.00	0.00	0.00	NaN
56112	PCDHGA3	0.00	0.00	0.00	NaN
56114	PCDHGA1	0.00	0.00	0.00	NaN
56123	PCDHB13	0.00	0.00	0.00	NaN
56124	PCDHB12	0.00	0.00	0.00	NaN
56125	PCDHB11	0.00	0.00	0.00	NaN
56128	PCDHB8	0.00	0.00	0.00	NaN
56130	PCDHB6	0.00	0.00	0.00	NaN
56132	PCDHB3	0.00	0.00	0.05	-0.18
56137	PCDHA12	0.00	0.00	0.00	NaN

56139	PCDHA10	0.00	0.25	0.00	0.02
56142	PCDHA6	0.00	0.00	0.00	NaN
56143	PCDHA5	0.00	0.00	0.00	NaN
56145	PCDHA3	0.00	0.00	0.00	NaN
56146	PCDHA2	0.00	0.00	0.00	NaN
56154	TEX15	-0.09	-0.25	-0.05	-0.12
56155	TEX14	0.18	0.00	0.00	0.18
56158	TEX12	0.00	0.00	0.00	NaN
56163	RNF17	0.00	-0.13	-0.15	0.08
56165	TDRD1	0.00	0.00	-0.05	0.06
56171	DNAH7	0.00	0.00	0.00	NaN
56172	ANKH	0.00	-0.25	0.15	0.15
56242	ZNF253	0.00	0.00	0.00	NaN
56244	BTNL2	0.00	0.00	0.00	NaN
56245	C21orf62	0.00	0.00	0.00	NaN
56252	ZAP3	0.00	0.00	0.00	NaN
56253	CRTAM	0.00	0.00	-0.05	-0.08
56255	DJ971N18.2	0.00	0.00	0.05	0.29
56257	FLJ20257	0.18	0.00	0.00	0.36
56259	C20orf33	0.00	0.00	0.00	NaN
56260	FLJ11267	0.00	0.00	0.05	0.22
56267	LOC56267	0.00	0.00	-0.05	0.10
56269	R30953_1	0.09	0.00	-0.10	0.09
56270	LOC56270	0.00	0.00	0.00	NaN
56287	LOC56287	0.00	0.00	0.00	NaN
56288	PARD3	-0.09	0.00	0.05	0.46
56300	IL-1H1	0.00	0.00	0.00	NaN
56302	TRPV5	0.00	0.00	-0.05	0.14
56311	LOC56311	0.00	0.13	0.00	-0.05
56339	M6A	0.00	0.00	0.00	NaN
56341	HRMT1L3	0.00	0.00	0.05	0.04
56344	CABP5	0.09	0.00	0.00	0.06
56413	BLTR2	0.00	0.00	0.00	NaN
56475	REPRIMO	0.00	0.00	0.00	NaN
56478	4E-T	0.00	0.00	0.00	NaN
56521	JDP1	0.00	0.00	0.00	NaN
56547	MMP26	0.00	-0.13	0.00	-0.04
56603	P450RAI-2	0.00	0.00	0.00	NaN
56604	TUBB4Q	0.00	0.00	0.05	0.03
56605	ERO1-L(BETA)	0.09	0.13	0.00	0.24
56606	SLC2A9	0.00	-0.13	-0.10	-0.09
56616	SMAC	0.00	-0.13	0.00	0.45
56647	BCCIP	0.00	0.00	-0.05	0.19
56648	EIF5A2	0.09	0.00	0.00	-0.03
56652	C10orf2	0.00	-0.13	-0.05	0.07
56654	NPDC1	0.00	0.00	0.00	NaN
56656	OR2S2	-0.09	0.00	0.10	-0.21

56659	KCNK13	0.00	0.13	0.00	0.10
56660	KCNK12	0.00	0.00	0.00	NaN
56672	C11orf17	0.00	0.00	-0.05	0.31
56673	C11orf16	0.00	0.00	-0.05	0.04
56674	C11orf15	0.00	0.00	-0.05	0.05
56675	C11orf14	0.00	0.00	-0.05	0.07
56676	ASCL3	0.00	0.00	-0.05	0.00
56683	C21orf59	0.00	0.00	0.00	NaN
56729	FIZZ3	0.00	0.00	0.00	NaN
56731	SLC2A4RG	0.09	0.00	0.00	0.06
56829	FLB6421	0.00	0.00	0.00	NaN
56833	BLAME	0.00	0.00	0.00	NaN
56834	LOC56834	0.00	0.00	0.00	NaN
56848	SPHK2	0.09	0.00	0.00	0.16
56851	LOC56851	0.00	0.00	0.00	NaN
56882	SPEC1	0.00	0.00	0.05	0.04
56886	UGCGL1	0.00	0.00	-0.05	0.18
56888	PCMF	0.00	0.00	0.00	NaN
56889	SMBP	0.00	0.00	-0.10	0.24
56890	MDM1	0.09	0.00	-0.05	0.65
56891	LOC56891	0.00	0.00	0.00	NaN
56892	C8orf4	0.36	-0.25	-0.05	0.22
56893	C1orf6	0.00	0.13	0.00	-0.18
56894	AGPAT3	0.00	0.00	0.00	NaN
56895	LPAAT-delta	0.00	0.00	0.00	NaN
56897	WHIP	0.00	0.00	-0.10	0.02
56898	LOC56898	0.00	0.00	0.00	NaN
56899	LOC56899	0.50	0.50	0.00	0.10
56901	LOC56901	0.00	0.00	0.00	NaN
56902	LOC56902	0.00	0.00	0.00	NaN
56903	PAPOLB	0.00	0.00	0.10	-0.03
56904	SH3GLB2	0.00	0.00	0.00	NaN
56906	LOC56906	0.00	0.00	0.00	NaN
56910	STARD7	0.00	0.00	0.00	NaN
56911	C21orf7	0.00	0.00	0.00	NaN
56912	LOC56912	0.00	0.00	0.00	NaN
56913	C1GALT1	0.00	0.00	0.05	0.12
56914	OTOR	0.00	0.00	0.00	NaN
56915	RRP46	0.00	0.00	-0.05	0.30
56918	DKFZp547H02	0.00	0.00	-0.05	0.33
56920	LOC56920	-0.09	-0.25	-0.10	0.10
56922	MCCC1	0.09	0.00	0.00	0.38
56924	PAK6	0.00	0.00	0.00	NaN
56925	LXN	0.00	0.00	0.00	NaN
56926	LOC56926	0.00	0.00	0.00	NaN
56929	FEM1A	0.00	0.13	0.00	0.59
56934	CARPX	0.00	0.13	0.00	-0.05

56935	FN5	0.00	0.00	-0.05	0.00
56936	PLPL	0.00	0.00	0.00	NaN
56937	TMEPAI	0.18	0.63	0.00	0.46
56938	ARNTL2	0.00	0.00	0.00	NaN
56940	MKPX	0.00	0.00	-0.05	0.17
56941	DC12	0.00	0.00	0.00	NaN
56942	DC13	0.00	0.00	-0.05	0.11
56943	DC6	0.18	0.00	0.05	0.25
56944	HNOEL-iso	0.27	0.13	0.10	0.27
56945	MRPS22	0.00	0.00	0.00	NaN
56946	C11ORF30	0.27	0.00	0.00	0.75
56947	GL004	0.00	0.00	0.00	NaN
56948	HCDI	0.00	0.00	0.00	NaN
56949	HCNP	0.00	0.00	0.00	NaN
56950	HSKM-B	0.00	0.00	0.00	NaN
56951	HTGN29	0.00	-0.13	0.00	0.29
56953	NT5M	0.00	0.00	0.00	NaN
56954	NIT2	0.00	0.00	0.00	NaN
56955	MEPE	0.00	0.00	0.00	NaN
56957	CEZANNE	0.00	0.00	0.05	-0.16
56965	LOC56965	0.00	0.00	0.00	NaN
56967	DKFZp761F20	0.00	0.00	0.00	NaN
56969	DKFZp547I014	0.09	0.00	-0.05	0.10
56978	PRDM8	0.00	0.00	0.00	NaN
56979	PRDM9	0.00	0.00	0.10	-0.08
56980	PRDM10	-0.09	0.00	-0.10	0.24
56981	PRDM11	0.00	0.00	0.00	NaN
56983	MDS010	0.00	0.00	0.00	NaN
56984	MDS003	0.00	0.00	0.00	NaN
56985	MDS006	0.00	0.00	0.00	NaN
56986	MDS009	0.18	0.00	0.00	0.29
56992	KNSL7	-0.09	0.00	-0.05	0.20
56993	TOMM22	-0.09	0.00	0.05	0.56
56994	CHPT1	0.00	0.00	0.00	NaN
56995	TUSP	0.00	0.00	0.00	NaN
56996	LOC56996	0.18	0.00	0.00	0.36
56997	CABC1	0.09	0.00	0.00	0.18
56998	CTNNBIP1	0.00	0.00	0.00	NaN
56999	ADAMTS9	0.09	0.00	-0.05	0.18
57001	DC11	0.18	0.00	0.00	0.31
57003	GK001	0.27	0.13	0.00	0.61
57007	RDC1	0.00	0.00	-0.05	0.09
57016	AKR1B10	0.00	0.00	0.00	NaN
57019	LOC57019	-0.09	0.00	0.00	0.53
57020	MGC16824	0.00	0.00	0.00	NaN
57030	SLC17A7	0.00	0.00	0.00	NaN
57035	DJ465N24.2.1	-0.09	0.00	0.00	0.21

57045	TSG	0.00	0.13	0.05	-0.04
57047	PLSCR2	0.00	0.00	-0.05	0.05
57048	PLSCR3	0.00	0.00	0.00	NaN
57050	SAS10	0.00	0.00	0.00	NaN
57053	CHRNA10	0.00	-0.13	0.00	0.09
57060	PCBP4	-0.09	0.00	-0.10	0.28
57062	DDX24	0.00	0.00	0.00	NaN
57082	AF15Q14	0.00	0.00	0.00	NaN
57084	SLC17A6	0.00	0.00	0.00	NaN
57088	PLSCR4	0.00	0.00	-0.05	0.07
57089	LALP1	0.00	-0.13	-0.05	0.06
57091	C20orf32	0.09	0.50	0.00	0.09
57092	pcnp	0.00	0.00	0.00	NaN
57093	RNF18	0.00	0.13	0.10	0.20
57096	RPGRIP1	0.00	0.00	0.00	NaN
57097	C12orf6	-0.09	0.00	0.05	-0.13
57099	LOC57099	0.00	0.00	0.00	NaN
57101	C12orf3	-0.09	0.00	0.00	0.05
57102	C12orf4	-0.09	0.00	0.05	0.34
57103	C12orf5	-0.09	0.00	0.05	0.37
57104	TTS-2.2	-0.18	-0.13	-0.05	0.09
57105	LOC57105	0.00	0.13	0.00	0.03
57107	LOC57107	0.00	0.00	0.10	0.70
57109	LOC57109	0.00	0.00	0.00	NaN
57110	HRASLS	0.18	0.00	0.05	-0.07
57111	RAB25	0.00	0.13	0.00	0.15
57115	LOC57115	0.00	0.00	0.00	NaN
57116	LOC57116	0.09	0.00	0.00	-0.11
57117	LOC57117	0.00	0.00	0.00	NaN
57118	LOC57118	0.00	0.00	0.00	NaN
57119	SPINLW1	0.00	0.00	0.00	NaN
57122	NUP107	0.09	0.13	0.00	0.37
57124	TEM1	0.00	0.00	0.05	0.23
57125	TEM7	0.00	0.38	0.00	0.04
57126	PRV1	0.09	0.00	-0.10	0.19
57127	RHBG	0.00	0.13	0.00	-0.15
57128	CGI-203	0.00	0.00	0.00	NaN
57130	CGI-152	0.00	0.00	0.00	NaN
57132	CHMP1.5	0.00	0.00	0.00	NaN
57134	HMIC	-0.09	0.00	0.00	-0.19
57139	RGL3	0.00	0.00	0.00	NaN
57140	RNPEPL1	0.00	0.00	-0.05	0.37
57142	RTN4	0.09	0.00	-0.05	0.40
57144	PAK7	0.00	0.00	0.00	NaN
57148	KIAA1219	0.00	-0.13	0.00	0.26
57149	LOC57149	0.00	0.00	0.00	NaN
57150	DKFZP586E19	0.00	0.00	0.00	NaN

57151	LOC57151	0.00	0.00	0.00	-0.01
57152	ARS	0.18	0.13	0.05	0.02
57154	SMURF1	0.18	0.00	0.00	0.40
57157	DKFZP564F01	-0.09	0.00	0.00	0.29
57158	JPH2	0.09	0.00	0.00	0.00
57161	PELI2	0.00	0.00	0.05	0.05
57162	PELI1	0.00	0.00	0.00	NaN
57165	CX46.6	0.09	0.00	0.00	0.05
57171	LSFR2	0.00	0.00	0.00	NaN
57172	CAMK1G	0.00	0.00	0.00	NaN
57175	DKFZP762I166	0.36	0.00	0.05	0.56
57180	ARP3BETA	0.00	0.00	-0.05	0.28
57184	FLJ00005	0.00	0.00	0.00	NaN
57185	DJ462O23.2	-0.09	0.00	0.00	-0.05
57188	KIAA1233	0.00	0.00	-0.05	0.04
57191	V1RL1	0.00	0.13	0.00	0.25
57192	MCOLN1	0.00	0.00	0.00	NaN
57198	ATP8B2	0.09	0.13	0.00	0.28
57205	ATP10D	0.00	0.00	0.00	NaN
57209	LOC57209	0.00	0.00	0.00	NaN
57212	KIAA0495	0.00	0.00	0.00	NaN
57213	C13orf1	0.00	0.00	0.00	NaN
57214	KIAA1199	0.00	0.00	0.00	NaN
57215	HRIHFB2206	-0.09	-0.13	0.00	0.29
57226	DJ122O8.2	0.00	0.00	0.00	NaN
57228	LOC57228	0.00	0.00	0.00	NaN
57231	SNX14	0.00	0.00	0.00	NaN
57282	SLC4A10	0.00	0.00	-0.05	0.05
57326	HPIP	0.09	0.13	0.00	0.34
57332	CBX8	0.00	0.25	0.00	0.38
57335	ZNF286	0.00	0.00	0.05	-0.11
57336	ZNF287	0.00	0.00	0.00	NaN
57337	SENP7	0.00	0.00	0.00	NaN
57338	JPH3	-0.09	-0.13	-0.05	-0.22
57343	ZNF304	0.09	0.13	0.00	0.33
57348	TTYH1	0.09	0.00	0.00	0.12
57369	CX36	0.00	0.00	0.00	NaN
57379	AICDA	-0.09	0.00	0.00	-0.10
57380	MRS2L	0.00	0.00	0.00	NaN
57402	LOC57402	0.09	0.13	0.00	0.25
57403	RAB22A	0.18	0.50	0.00	0.60
57404	CYP-M	0.00	0.00	0.00	NaN
57405	AD024	0.00	0.00	0.00	NaN
57406	LOC57406	0.00	-0.13	-0.10	0.26
57408	HT017	0.00	0.00	-0.15	0.10
57415	HT021	0.09	0.00	-0.05	0.37
57418	WDR18	0.00	0.00	0.00	NaN

57419	SLC24A3	0.00	0.00	0.00	NaN
57446	NDRG3	0.00	-0.13	0.00	0.15
57447	NDRG2	0.00	0.00	0.00	NaN
57460	KIAA1157	0.00	0.00	0.00	NaN
57466	KIAA1172	0.00	0.00	0.00	NaN
57468	SLC12A5	0.00	0.00	0.00	NaN
57470	KIAA1185	0.00	0.00	0.00	NaN
57472	KIAA1194	0.00	0.13	0.00	0.50
57473	KIAA1196	0.00	0.00	0.00	NaN
57488	KIAA1228	0.00	0.00	-0.05	0.13
57493	KIAA1237	0.00	0.00	0.00	NaN
57496	KIAA1243	0.00	0.00	0.00	NaN
57498	KIDINS220	0.00	0.00	-0.05	0.27
57509	ATIP1	0.00	-0.13	-0.05	0.13
57513	CASKIN2	0.18	0.13	0.00	0.73
57515	KIAA1253	0.00	0.00	0.05	0.47
57523	KIAA1305	0.00	0.00	0.00	NaN
57535	KIAA1324	0.00	0.00	0.05	0.01
57538	MIDORI	0.00	0.00	-0.05	0.18
57551	KIAA1361	0.00	0.38	0.00	0.30
57556	SEMA6A	0.00	0.13	0.00	-0.14
57570	KIAA1393	0.00	0.00	0.00	NaN
57573	KIAA1396	0.00	0.13	0.05	-0.02
57576	KIAA1405	-0.09	0.00	0.00	0.03
57579	KIAA1411	0.00	0.00	0.05	0.14
57586	SYT13	0.00	0.00	0.00	NaN
57591	MKL1	-0.09	0.00	0.05	0.00
57596	KIAA1446	0.00	0.00	0.00	NaN
57599	KIAA1449	0.00	0.00	0.00	NaN
57602	KIAA1453	0.00	0.38	0.00	0.29
57604	KIAA1456	0.00	-0.25	-0.10	0.02
57610	KIAA1464	-0.09	-0.13	0.00	0.21
57613	KIAA1467	-0.09	0.00	0.00	0.18
57615	KIAA1473	0.00	-0.13	-0.05	0.18
57634	EP400	0.00	0.00	0.00	NaN
57645	KIAA1513	0.00	0.00	0.00	NaN
57657	KIAA1535	0.09	0.13	0.00	-0.19
57658	KIAA1536	0.00	0.00	0.00	NaN
57663	USP29	0.09	0.13	0.00	0.23
57664	PLEKHA4	0.09	0.00	0.00	-0.08
57669	KIAA1548	0.00	0.00	0.00	NaN
57698	KIAA1598	0.00	0.00	-0.05	0.14
57706	KIAA1608	0.00	0.00	0.00	NaN
57707	KIAA1609	-0.09	-0.13	-0.05	0.28
57710	KIAA1614	0.00	0.00	0.00	NaN
57715	SEMA4G	0.00	-0.13	-0.05	-0.12
57718	KIAA1622	0.00	0.00	0.00	NaN

57720	GPR107	0.00	0.00	0.00	NaN
57728	KIAA1638	0.00	0.00	0.05	0.57
57730	KIAA1641	0.27	0.38	0.30	0.06
57731	SPTBN4	0.00	0.00	0.00	NaN
57733	GLUC	0.00	0.00	-0.05	-0.02
57758	CEGP1	0.00	0.00	0.05	-0.08
57761	C20orf97	0.00	0.00	0.05	-0.17
57763	ANKRA2	0.00	0.00	0.00	NaN
57786	RBAK	0.00	0.00	0.10	-0.11
57787	MARKL1	0.09	0.00	0.00	0.05
57794	DKFZP434E22	0.00	0.00	0.00	NaN
57795	LOC57795	0.00	0.00	0.00	NaN
57798	RG083M05.2	0.18	0.00	0.00	0.68
57799	RAB40C	0.00	0.00	-0.05	0.11
57805	LOC57805	-0.09	-0.25	-0.15	0.07
57817	HAMP	0.00	0.00	0.00	NaN
57818	IGRP	0.00	0.00	0.00	NaN
57819	C6orf28	0.00	0.00	0.00	NaN
57820	HEI10	0.00	0.00	0.00	NaN
57821	LOC57821	0.00	0.00	-0.05	-0.06
57823	CRACC	0.00	0.00	0.00	NaN
57824	HB-1	0.00	0.00	0.00	NaN
57827	G4	0.00	0.00	0.00	NaN
57828	DKFZP434A10	0.00	0.13	0.00	0.03
57830	LOC57830	0.00	0.50	0.00	-0.19
57834	CYP4F11	0.00	0.00	0.00	NaN
57862	LOC57862	0.00	0.00	0.00	NaN
57863	LOC57863	0.00	0.00	0.00	NaN
58155	PTBP2	0.09	0.00	0.05	0.66
58158	NEUROD4	0.00	0.00	0.00	NaN
58189	WFDC1	-0.09	-0.13	0.00	-0.19
58190	NLI-IF	0.00	0.00	0.00	NaN
58472	SQRDL	0.00	0.00	0.00	NaN
58473	PHRET1	0.09	0.13	0.00	0.53
58477	APMCF1	0.09	0.00	0.00	-0.11
58478	MASA	0.00	0.00	0.00	NaN
58483	EST-YD1	0.00	0.00	0.00	NaN
58486	LOC58486	0.00	0.00	0.00	NaN
58487	ZF	0.00	0.00	0.00	NaN
58488	PCTP	0.00	0.00	0.00	0.44
58494	JAM2	0.00	0.00	0.00	NaN
58495	ZNF339	0.00	0.00	0.00	NaN
58497	HTCD37	0.00	0.00	0.05	0.47
58498	LOC58498	0.00	0.00	0.00	NaN
58500	LOC58500	0.18	0.00	0.10	0.51
58503	PROL1	0.00	0.00	0.00	NaN
58504	LOC58504	0.00	-0.13	0.00	0.08

58509	LOC58509	0.00	0.00	0.00	NaN
58511	DLAD	0.00	0.00	0.05	0.31
58513	EPS15R	0.00	0.00	0.05	0.51
58516	TERA	0.00	0.00	0.00	NaN
58517	S164	0.00	0.00	0.00	NaN
58525	LOC58525	0.00	0.00	0.00	NaN
58528	RAGD	0.00	0.00	-0.05	0.08
58529	MYOZ1	0.09	0.00	0.00	0.01
58530	C6orf23	0.00	0.00	0.00	NaN
58533	SNX6	0.00	0.00	0.00	NaN
58985	IL22R	-0.09	0.00	0.00	0.03
58986	TMEM8	0.00	0.00	-0.05	0.32
59269	HIVEP3	0.00	0.00	0.00	NaN
59307	SIGIRR	-0.27	-0.13	-0.15	0.40
59335	PRDM12	0.00	0.00	0.00	NaN
59336	PRDM13	0.00	0.00	0.00	NaN
59338	PLEKHA1	0.00	0.00	-0.05	0.18
59340	HRH4	0.00	0.00	-0.15	-0.07
59341	TRPV4	0.00	0.00	0.05	-0.22
59342	RISC	0.09	-0.13	0.00	0.11
59344	ALOXE3	0.00	0.00	0.00	NaN
59347	FKSG2	0.27	-0.13	-0.05	0.11
59348	ZBRK1	0.00	0.00	-0.05	0.15
59351	UC28	0.00	0.00	0.00	NaN
60312	AFAP	0.00	-0.13	-0.10	0.25
60313	SP192	0.00	0.00	0.00	NaN
60314	C12orf10	0.00	0.00	0.00	NaN
60370	PP5395	0.00	0.00	-0.05	0.06
60385	TSKS	0.00	0.00	0.00	NaN
60436	TGIF2	0.00	-0.13	0.00	0.09
60467	BPESC1	0.00	0.00	0.00	NaN
60468	BACH2	0.00	0.00	0.00	NaN
60481	HELO1	0.00	0.00	0.00	NaN
60482	SLC5A7	0.00	0.00	0.00	NaN
60484	BRAL1	0.00	0.13	0.00	-0.29
60485	WW45	0.00	0.00	0.00	NaN
60487	MDS024	0.00	0.00	0.00	NaN
60489	MDS019	-0.09	0.00	0.05	0.42
60490	MDS018	0.00	0.00	0.00	NaN
60491	NIF3L1	0.00	0.00	0.00	NaN
60492	MDS025	0.00	0.00	0.05	0.23
60493	FLJ13149	0.00	0.00	0.00	NaN
60494	FLJ23514	0.00	0.00	0.00	NaN
60495	HPA2	0.00	0.00	-0.05	0.06
60496	AASDHPPT	0.00	0.00	0.00	NaN
60509	FLJ21839	0.09	0.00	0.05	0.33
60526	FLJ21820	0.09	0.00	-0.05	0.35

60528	ELAC2	0.00	0.00	0.00	NaN
60529	ALX4	0.00	0.00	0.00	NaN
60558	FLJ13220	0.00	0.00	0.00	NaN
60559	FLJ22649	0.00	0.13	-0.10	0.39
60560	FLJ21613	0.00	0.00	0.00	NaN
60561	FLJ11785	0.09	0.00	0.00	0.33
60598	KCNK15	0.09	0.13	0.00	-0.10
60625	DDX35	0.00	-0.13	0.00	0.27
60626	RIC-8	-0.27	-0.13	-0.15	0.79
60672	FLJ12438	0.00	0.00	0.00	NaN
60673	FLJ11773	0.00	0.00	0.00	NaN
60676	PLAC3	0.00	0.00	0.00	NaN
60681	FKBP10	0.00	0.13	0.00	0.12
60684	FLJ12716	0.00	0.00	-0.05	0.44
60685	TEX27	0.00	0.00	0.00	NaN
60686	FLJ12154	0.00	0.00	0.00	NaN
63826	SRR	0.00	-0.13	0.00	0.10
63827	BCAN	0.00	0.13	0.00	0.02
63874	FLJ12816	0.00	0.00	0.00	NaN
63875	MRPL17	0.00	0.00	0.00	0.49
63876	PKNOX2	-0.09	0.00	-0.10	0.10
63877	FLJ13188	0.00	0.00	-0.05	0.03
63891	FLJ12565	-0.09	0.00	-0.05	0.36
63892	FLJ21877	0.00	0.00	-0.05	0.35
63893	E2-230K	0.00	0.13	0.00	0.35
63894	FLJ12707	0.00	0.00	0.00	NaN
63895	FLJ23403	0.00	0.00	0.00	NaN
63897	FLJ22087	0.18	0.13	0.00	0.76
63898	FLJ20967	-0.09	-0.13	-0.05	0.03
63899	FLJ22609	-0.09	0.00	0.00	0.24
63901	FLJ22794	0.00	0.00	0.00	NaN
63906	FLJ12455	-0.09	-0.13	0.00	-0.03
63910	C20orf59	0.09	0.13	0.00	-0.11
63916	ELMO2	0.00	0.00	0.00	NaN
63917	FLJ21634	0.00	0.00	-0.05	0.27
63920	LOC63920	0.00	0.00	0.00	NaN
63923	LOC63923	0.00	0.00	0.05	-0.25
63924	FLJ20871	0.00	0.00	0.00	NaN
63925	ZNF335	0.00	0.00	0.00	NaN
63926	ANKRD5	0.00	0.00	0.00	NaN
63928	LOC63928	0.00	0.00	0.00	NaN
63929	LOC63929	0.00	0.00	0.05	-0.07
63931	MRPS14	0.00	0.00	0.05	0.35
63933	FLJ20958	0.00	0.00	0.10	0.48
63934	FLJ14011	0.00	0.13	0.05	0.16
63935	C20orf67	0.00	0.00	0.00	NaN
63940	C6orf9	0.00	0.00	0.00	NaN

63941	APBA2BP	-0.09	-0.13	0.00	0.38
63943	FKBPL	0.00	0.00	0.00	NaN
63967	CLASPIN	0.00	0.00	0.00	NaN
63970	P53AIP1	-0.09	0.00	-0.10	0.07
63971	KIF13A	0.00	0.00	-0.05	0.11
63974	NEUROD6	0.00	0.13	0.00	0.10
63976	PRDM16	0.00	0.00	0.00	NaN
63978	PRDM14	0.00	0.00	0.00	NaN
63982	C11orf25	0.00	0.00	0.00	NaN
64062	SE70-2	-0.09	0.13	-0.10	0.26
64063	PRSS22	0.00	0.00	0.00	NaN
64064	OXCT2	0.00	0.00	0.00	NaN
64065	PIGPC1	0.00	0.00	0.00	NaN
64066	MMP27	0.00	0.13	0.00	0.26
64067	NPAS3	0.00	0.00	0.00	NaN
64077	LHPP	0.00	0.00	-0.05	0.10
64078	SLC28A3	0.00	0.00	0.00	NaN
64080	RBSK	0.09	0.00	0.05	0.33
64081	MAWBP	0.00	0.00	0.00	NaN
64083	GOLPH3	0.00	0.00	0.15	0.26
64084	CLSTN2	0.00	0.00	0.00	NaN
64087	MCCC2	0.00	0.00	0.00	NaN
64089	SNX16	0.00	0.00	0.05	-0.15
64091	POP2	0.00	0.00	0.00	NaN
64092	SAMSN1	0.00	-0.25	-0.05	-0.02
64096	GFRA4	0.00	0.00	0.00	NaN
64097	EPB41L4	0.00	0.00	0.00	NaN
64100	ELSPBP1	0.09	0.00	0.00	0.11
64106	OT7T022	0.00	0.00	0.00	NaN
64108	IFRG28	0.00	0.00	0.10	0.46
64110	MAGEF1	0.09	0.00	0.00	0.23
64111	RFRP	0.00	0.13	0.00	-0.06
64112	MAP-1	0.00	0.00	0.00	NaN
64114	PP1201	0.00	0.00	0.00	NaN
64116	LOC64116	0.00	0.00	0.00	NaN
64118	PP3111	0.00	0.00	0.00	NaN
64121	GTR2	0.00	0.00	0.00	NaN
64122	FN3K	0.00	0.00	0.00	NaN
64123	ETL	0.00	0.00	0.00	NaN
64127	CARD15	-0.09	0.00	-0.05	0.02
64129	LIECG3	0.00	0.00	0.00	NaN
64130	LIN-7B	0.09	0.00	0.00	0.32
64132	XYLT2	0.00	0.25	0.00	0.59
64135	MDA5	0.00	0.00	-0.05	0.13
64137	ABCG4	-0.09	0.00	-0.05	-0.10
64149	NJMU-R1	0.00	0.00	0.00	NaN
64151	HCAP-G	0.00	0.00	-0.05	0.22

64167	LOC64167	0.00	0.00	0.00	NaN
64170	CARD9	0.00	0.00	0.00	NaN
64172	LOC64172	0.00	0.00	0.00	NaN
64173	SPATA1	0.00	0.00	0.05	0.01
64174	LOC64174	-0.09	-0.13	0.00	0.06
64175	GROS1	0.00	0.00	0.00	NaN
64180	LOC64180	-0.09	-0.13	0.00	-0.01
64184	HE3-BETA	0.00	0.00	0.00	NaN
64208	POP3	0.00	0.00	0.10	0.19
64210	MMS19L	0.00	0.00	-0.05	0.30
64211	LHX5	0.00	0.00	0.00	NaN
64215	DNAJL1	-0.09	0.00	0.05	0.12
64216	TFB2M	0.00	0.00	0.00	NaN
64218	FLJ12287	0.00	0.13	0.00	0.04
64220	FLJ12541	0.00	0.00	0.00	NaN
64221	FLJ21044	0.00	0.00	-0.10	-0.32
64222	ADIR	0.00	0.00	0.00	NaN
64223	GBL	0.00	0.00	0.00	NaN
64231	MS4A6A	0.00	0.00	0.00	NaN
64232	MS4A5	0.00	0.00	0.00	NaN
64240	ABCG5	0.00	0.00	0.00	NaN
64283	FLJ21817	0.00	0.00	0.00	NaN
64284	RAB17	0.00	0.00	-0.05	0.17
64285	FLJ22357	0.00	0.00	-0.05	0.25
64318	AD24	0.00	0.00	0.00	NaN
64319	FLJ11618	0.00	0.00	0.00	NaN
64320	RNF25	0.00	0.00	0.00	NaN
64321	SOX17	0.00	0.00	0.00	NaN
64324	NSD1	0.00	0.13	0.00	0.44
64328	XPO4	0.00	0.00	-0.10	0.35
64342	FLJ14249	0.09	0.00	0.05	0.24
64343	FLJ21939	-0.09	0.00	-0.05	-0.12
64344	HIF-3A	0.09	0.00	0.00	0.16
64374	SIL1	0.00	0.00	-0.05	0.44
64375	ZNFN1A4	0.00	0.00	0.00	NaN
64376	PEGASUS	0.09	0.00	-0.05	0.70
64377	CHST8	0.00	0.00	0.05	-0.15
64388	FLJ21195	0.00	0.00	0.00	NaN
64393	WIG1	0.09	0.00	0.05	0.35
64395	FLJ13057	0.00	0.00	0.00	NaN
64397	ZFP106	0.00	0.00	0.00	NaN
64398	MPP5	0.00	0.00	0.05	0.22
64400	FTS	0.09	0.00	0.00	0.45
64405	CDH22	0.00	0.00	0.00	NaN
64410	FLJ12587	0.00	0.00	-0.05	0.18
64411	ARAP3	0.00	0.00	0.00	NaN
64417	FLJ21657	0.00	0.00	0.05	-0.15

64418	FLJ13576	0.09	0.00	0.00	-0.07
64421	SCIDA	0.00	0.00	0.00	NaN
64422	APG3	0.00	0.00	0.00	NaN
64423	FLJ22056	0.00	0.00	0.00	NaN
64425	FLJ13390	-0.09	0.00	0.05	0.33
64427	FLJ12788	0.00	0.00	0.00	NaN
64428	FLJ21988	0.00	0.00	-0.05	0.27
64429	FLJ21952	0.00	0.00	-0.05	0.30
64430	FLJ12799	0.00	0.00	0.00	NaN
64431	FLJ13433	0.00	0.00	0.00	NaN
64446	DNAI2	0.00	0.13	0.00	0.14
64499	TPSB2	0.00	0.00	-0.05	0.09
64506	CPEB1	0.00	0.00	0.00	NaN
64577	ALDH8A1	0.00	0.00	0.05	-0.10
64579	NDST4	0.00	0.00	0.00	NaN
64581	CLECSF12	-0.09	0.00	0.00	0.06
64582	HUMNPIIY20	0.00	0.00	0.00	NaN
64598	AF053356_CD	0.18	0.00	0.00	0.00
64600	PLA2G2F	-0.09	0.00	0.00	0.03
64641	FLJ11500	-0.09	-0.25	-0.05	0.00
64682	MCPR	0.00	0.00	0.00	NaN
64693	CTAGE-1	0.00	0.00	-0.05	0.11
64708	COPS7B	0.00	0.00	0.00	NaN
64710	NUCKS	0.00	0.13	0.00	0.14
64714	PDIP	0.00	0.00	-0.05	0.02
64745	FLJ20859	0.00	0.13	0.05	0.02
64746	GOLPH1	0.09	0.00	0.00	0.37
64747	FLJ14153	0.00	0.00	0.05	-0.03
64748	FLJ13055	0.00	0.00	0.00	NaN
64750	SMURF2	0.27	0.13	0.00	0.21
64754	FLJ21080	0.09	0.00	0.00	0.01
64755	FLJ13868	0.00	0.00	0.00	NaN
64757	FLJ22390	0.09	0.00	0.00	0.12
64759	TEM6	0.00	0.00	0.00	NaN
64760	RAI16	-0.09	-0.25	-0.10	0.32
64761	FLJ22693	0.00	0.00	0.00	NaN
64762	FLJ21610	0.00	0.00	-0.15	0.10
64763	FLJ22059	0.00	0.00	-0.05	0.32
64764	DKFZP586F24	0.00	0.00	0.00	NaN
64766	FLJ12903	0.00	0.00	0.00	NaN
64768	C9orf12	0.00	0.00	0.00	NaN
64769	FLJ11730	0.00	0.00	0.00	NaN
64771	FLJ22195	0.00	0.00	0.00	NaN
64772	FLJ21865	0.00	0.25	0.00	0.54
64776	FLJ23499	0.00	0.00	0.00	NaN
64777	FLJ22318	0.18	0.00	0.00	0.53
64778	FLJ23399	0.09	0.00	0.00	0.11

64779	FLJ12998	-0.09	-0.13	-0.05	0.49
64780	MICAL	0.00	0.00	0.05	0.04
64781	LK4	0.00	-0.25	0.00	0.41
64782	FLJ12484	0.00	0.00	-0.05	0.30
64783	RBM15	0.09	0.00	0.00	0.00
64784	FLJ21868	0.00	0.00	0.00	NaN
64785	FLJ13912	-0.09	0.00	0.00	0.16
64786	FLJ12085	0.09	0.00	0.00	0.31
64787	EPS8R2	-0.18	-0.13	0.00	0.08
64788	FLJ12681	0.00	0.00	-0.05	0.12
64789	FLJ21144	0.00	0.00	0.00	NaN
64792	FLJ14117	0.18	0.00	0.00	0.47
64793	DKFZP434L01	-0.09	0.00	0.00	-0.10
64794	DDX31	0.00	0.00	0.00	NaN
64795	FLJ13910	0.00	0.00	0.00	0.36
64798	FLJ12428	0.00	0.25	0.15	-0.13
64799	FLJ12476	0.00	0.00	0.00	NaN
64800	FLJ23588	0.00	0.00	0.05	0.01
64816	CYP3A43	0.18	0.00	0.00	0.43
64834	ELOVL1	0.00	0.00	0.00	NaN
64837	FLJ12387	-0.09	0.00	0.05	0.09
64838	FLJ22362	0.09	0.13	0.05	-0.15
64847	FLJ21347	0.00	0.25	0.00	0.52
64848	FLJ21940	0.00	0.13	0.00	0.33
64849	SLC13A3	0.00	0.00	0.00	NaN
64850	AGXT2L1	0.00	0.00	0.00	NaN
64852	FLJ22347	0.00	0.00	0.00	NaN
64854	FLJ12552	0.00	0.00	0.00	NaN
64856	FLJ22215	0.00	0.13	0.00	0.01
64858	DCLRE1B	0.27	0.13	0.05	0.82
64859	FLJ22833	0.00	0.00	0.00	NaN
64863	FLJ23017	0.00	0.00	0.00	NaN
64864	FLJ12994	0.00	0.00	0.05	-0.07
64866	FLJ22969	-0.09	0.00	-0.05	0.23
64895	PAPOLG	0.00	0.00	0.00	NaN
64897	FLJ12448	0.00	0.00	0.00	0.09
64901	RANBP17	0.00	0.00	0.05	-0.01
64919	BCL11B	0.00	0.00	0.00	NaN
64921	FLJ21213	0.18	0.00	0.00	0.37
64922	FLJ21302	0.00	-0.13	-0.05	0.07
64924	ZTL1	0.00	0.00	0.00	NaN
64925	FLJ12800	-0.09	0.00	-0.05	-0.01
64927	FLJ12572	0.00	0.00	0.00	NaN
64940	FLJ13195	0.00	0.00	0.05	0.32
64943	FLJ12442	-0.09	-0.25	-0.10	0.14
64960	MRPS15	0.00	0.00	0.00	NaN
64963	MRPS11	0.00	0.00	-0.05	0.39

64981	MRPL34	-0.09	0.00	0.00	0.24
65003	MRPL11	0.00	0.00	0.05	0.40
65005	MRPL9	0.00	0.13	0.05	0.41
65009	NDRG4	-0.09	0.00	0.00	0.05
65010	SLC26A6	-0.09	0.00	0.00	0.04
65012	SLC26A10	0.00	0.00	0.00	NaN
65018	PINK1	-0.09	0.00	0.00	0.19
65055	FLJ13110	0.00	0.00	-0.05	0.18
65057		-0.09	-0.13	0.00	0.40
65080	MRPL44	0.00	0.00	0.00	NaN
65082	VPS33A	0.00	-0.13	0.00	-0.25
65083	Nrap	-0.09	0.00	0.10	0.49
65094	FLJ12517	0.09	0.00	0.00	0.09
65095	FLJ12949	0.00	0.13	0.05	0.39
65108	MACMARCKS	0.00	0.00	0.00	NaN
65110	UPF3A	-0.09	-0.13	-0.05	0.38
65117	FLJ11021	0.00	-0.13	0.00	0.36
65121	LOC65121	0.00	0.00	0.00	NaN
65123	FLJ21919	0.09	0.13	0.00	0.34
65124	FLJ21870	0.00	0.00	0.00	NaN
65125	PRKWNK1	0.00	0.00	1.00	0.60
65220	FLJ13052	0.00	0.00	0.20	0.02
65243	LOC65243	0.00	0.00	0.00	NaN
65244	FLJ13117	0.00	0.00	0.00	NaN
65250	FLJ13231	0.00	0.00	0.05	0.18
65258	MPPE1	0.00	0.00	0.00	NaN
65260	FLJ12439	0.00	0.00	0.00	NaN
65263	FLJ13852	0.18	0.00	0.10	0.50
65264	FLJ13855	0.00	0.38	0.00	0.71
65265	FLJ20989	0.18	0.00	0.15	0.41
65979	FLJ13171	0.00	0.00	0.00	NaN
65980	FLJ13441	0.00	0.13	0.10	0.53
65981	FLJ22569	0.00	0.00	0.00	NaN
65982	FLJ12895	0.00	0.13	0.00	-0.09
65983	FLJ21313	0.00	0.00	0.00	NaN
65985	FLJ12389	-0.09	0.00	0.00	0.47
65986	RINZF	0.00	0.25	0.05	0.48
65987	MGC2376	0.27	0.00	0.00	-0.01
65988	MGC2474	0.00	0.00	0.00	NaN
65989	MGC2487	0.00	0.00	0.00	NaN
65990	MGC2494	0.00	0.00	-0.05	0.36
65992	C20orf116	0.00	0.00	0.00	NaN
65993	MRPS34	0.00	0.00	0.00	NaN
65997	MGC2827	0.00	0.00	0.00	NaN
65999	MGC3036	0.00	0.00	-0.05	0.12
66002	CYP4F12	0.00	0.00	0.05	-0.08
66008	ALS2CR3	0.00	0.00	0.00	NaN

66035	SLC2A11	0.00	0.00	0.05	-0.11
66036	MTMR8	-0.09	-0.50	-0.15	0.37
78986	MGC1136	0.09	-0.25	-0.05	-0.03
78988	MRP63	0.00	0.00	-0.10	0.44
78989	MGC3279	0.00	0.00	0.00	NaN
78990	FLJ21916	0.00	0.00	0.00	NaN
78991	MGC3265	0.00	0.00	0.00	NaN
78992	MGC3262	0.00	0.00	-0.05	0.22
78994	MGC3121	0.00	0.00	0.00	NaN
78995	MGC3130	0.00	-0.13	0.00	-0.31
78996	MGC5242	0.00	0.00	0.00	NaN
78997	MGC3129	0.09	0.13	0.00	0.03
78998	MGC3113	0.18	0.00	0.05	0.27
78999	MGC3103	0.09	0.00	0.05	-0.02
79000	MGC2603	-0.09	0.00	0.00	0.26
79001	IMAGE345520	0.00	0.00	0.00	NaN
79003	MGC2488	0.00	-0.13	0.00	0.41
79004	MGC2491	0.00	-0.13	-0.05	0.42
79005	MGC3180	0.00	0.00	0.05	0.58
79006	MGC2601	0.00	0.00	-0.05	0.11
79007	MGC3101	-0.09	-0.13	-0.05	0.18
79008	MGC5178	0.00	0.00	0.00	NaN
79009	GU2	0.00	0.00	0.00	NaN
79012	MGC8407	-0.09	0.00	-0.05	0.07
79016	MGC2594	-0.09	0.00	0.00	0.19
79018	MGC3048	0.00	0.00	0.00	NaN
79019	MGC861	0.00	0.00	0.05	0.45
79020	MGC2821	0.00	0.00	-0.05	0.03
79022	MGC5576	0.00	0.00	0.00	NaN
79023	MGC5585	0.00	0.00	0.00	NaN
79025	MGC5356	0.09	0.00	0.00	0.35
79026	MGC5395	0.00	0.00	0.00	NaN
79029	MGC5347	0.00	0.00	0.00	NaN
79031	MGC3062	0.00	0.00	0.00	NaN
79033	PRNPIP	0.00	0.00	0.00	NaN
79034	MGC2718	0.00	0.00	0.10	0.07
79035	MGC2731	0.00	0.00	0.00	NaN
79036	MGC2749	0.00	0.00	0.00	NaN
79037	MGC2463	0.18	0.00	0.00	0.32
79038	MGC2550	0.00	0.00	0.00	NaN
79039	MGC2835	0.00	0.00	0.00	NaN
79042	LENG5	0.09	0.00	0.00	0.14
79047	MGC2628	0.00	0.00	0.05	-0.04
79048	SBP2	0.00	0.00	0.00	NaN
79050	MGC3162	0.00	0.00	0.00	NaN
79053	MGC2840	0.27	0.00	0.00	0.68
79054	TRPM8	0.00	0.00	0.00	NaN

79056	TMG4	0.09	0.00	0.00	0.20
79058	ASPSCR1	0.00	0.00	0.00	NaN
79064	MGC3196	0.00	0.00	0.00	NaN
79065	FLJ22169	0.00	0.00	0.00	NaN
79066	MGC3329	0.00	0.00	0.00	NaN
79068	MGC5149	0.09	0.00	0.00	0.54
79070	MGC5302	-0.09	-0.13	0.05	0.43
79071	LCE	0.00	0.00	0.00	NaN
79072	MGC5297	0.00	0.00	0.05	0.30
79073	MGC5508	0.00	0.00	0.00	NaN
79074	MGC5509	0.00	0.00	0.00	NaN
79075	MGC5528	0.00	0.25	0.15	0.57
79077	MGC5627	0.00	0.00	0.00	NaN
79078	MGC955	0.00	0.00	0.00	NaN
79080	MGC2574	0.00	0.00	0.00	NaN
79081	MGC2477	0.00	0.00	0.00	NaN
79083	MLPH	0.00	0.00	-0.05	0.24
79084	MEP50	0.09	0.00	0.00	0.43
79085	MGC2615	0.00	0.00	0.00	NaN
79086	MGC2747	0.00	0.00	0.00	NaN
79087	MGC3136	0.00	-0.25	0.00	0.37
79088	MGC2663	0.00	0.00	0.00	NaN
79089	MGC3123	0.00	-0.13	0.00	0.07
79090	MGC2650	0.09	0.00	0.05	0.28
79091	MGC2654	0.00	0.00	0.00	NaN
79092	CARD14	0.00	0.25	0.00	0.22
79094	MGC4504	0.00	0.00	0.00	NaN
79095	C9orf16	-0.09	0.00	0.00	0.34
79096	MGC4707	0.00	0.00	0.00	NaN
79097	MGC4827	0.00	0.00	0.00	NaN
79098	MGC4309	0.00	0.13	0.00	-0.09
79101	MGC5306	0.00	0.00	-0.10	0.16
79109	MGC2745	0.00	0.00	0.00	NaN
79132	LGP2	0.00	0.13	0.00	0.09
79133	C20orf7	0.00	0.00	0.00	NaN
79134	FLJ20979	0.00	0.00	0.00	NaN
79136	C6orf22	0.00	0.00	0.00	NaN
79137	MGC3035	0.00	0.00	0.00	NaN
79139	MGC3067	0.18	0.25	0.05	0.59
79140	MGC1203	0.00	0.00	0.00	NaN
79143	LENG4	0.09	0.00	0.00	0.11
79144	C20orf149	0.09	0.00	0.05	0.04
79145	MGC2217	0.00	0.00	0.00	NaN
79147	FKRP	0.09	0.00	0.00	0.29
79148	MMP28	0.00	0.00	0.00	0.00
79149	MGC4161	0.09	0.13	0.00	-0.25
79152	FAAH	0.00	0.00	0.05	-0.08

79153	MGC4171	0.00	0.00	0.00	NaN
79154	MGC4172	0.00	-0.13	0.00	-0.03
79155	ABIN-2	0.00	-0.13	-0.15	0.38
79156	MGC4090	0.00	0.13	0.15	0.61
79157	ET	0.00	0.13	0.00	0.16
79158	MGC4170	0.00	0.00	0.00	NaN
79159	MGC3731	-0.09	0.00	0.05	0.52
79161	MGC4175	0.00	0.00	0.00	NaN
79166	ILT10	1.00	0.00	0.00	-0.25
79169	MGC4174	0.09	0.00	0.00	0.54
79170	MGC11242	0.00	0.00	0.00	0.18
79171	MGC10433	0.00	0.00	0.00	NaN
79172	MGC11266	0.09	0.00	0.00	0.00
79173	MGC11271	0.00	0.00	0.00	NaN
79174	MGC11256	0.00	-0.25	0.00	0.30
79175	MGC10715	0.00	0.00	0.05	-0.04
79176	MGC11279	0.00	-0.13	-0.05	0.26
79177	MGC2508	0.09	0.00	-0.10	0.56
79178	THTP	0.00	0.00	0.00	NaN
79180	MGC4342	0.00	0.00	0.00	NaN
79183	C20orf121	0.09	0.13	0.00	0.35
79187	FSD1	0.00	0.00	0.00	NaN
79188	MGC3222	0.00	0.00	0.00	NaN
79228	MGC2655	0.00	0.00	0.00	NaN
79230	MGC4054	0.00	0.00	0.00	NaN
79269	MGC10765	-0.09	0.00	0.10	0.30
79363	MGC10731	0.09	0.00	0.00	0.20
79364	MGC11349	0.00	0.00	0.00	NaN
79365	BHLHB3	0.00	0.00	0.00	NaN
79368	SPAP1	0.00	0.00	0.00	NaN
79369	B3GNT4	0.00	-0.13	0.00	-0.15
79370	BCLG	-0.09	0.00	0.10	0.10
79400	NOX5	0.00	0.00	0.00	NaN
79411	MGC10771	0.00	0.00	0.00	NaN
79412	MGC10791	0.00	0.00	0.00	NaN
79413	MGC10796	0.00	0.00	0.00	NaN
79414	MGC2656	0.00	0.00	0.00	NaN
79415	MGC4368	0.00	0.00	0.00	NaN
79441	MGC4701	0.00	-0.13	-0.20	0.42
79442	LRRC2	-0.09	0.00	-0.05	-0.28
79443	FYCO1	-0.09	0.00	-0.05	0.41
79444	BIRC7	0.09	0.00	0.00	-0.11
79446	MGC4645	0.00	0.00	0.00	NaN
79447	MGC4606	0.00	0.00	0.00	NaN
79469	BCMSUNL	0.00	0.00	0.00	NaN
79567	FLJ13725	-0.09	0.00	0.10	0.35
79568	FLJ22555	0.00	0.00	0.00	NaN

79570	FLJ12650	0.00	0.00	0.00	NaN
79571	FLJ22035	0.00	0.25	0.00	0.63
79572	FLJ20986	0.09	0.00	0.00	0.56
79573	FLJ22584	0.09	0.00	0.00	0.30
79574	EPS8R3	0.09	0.00	0.05	0.16
79575	FLJ11743	-0.09	0.00	0.00	-0.15
79577	C1orf28	0.09	0.00	0.00	0.31
79581	FLJ11856	0.18	0.00	0.15	0.23
79582	FLJ22724	0.00	0.00	0.00	NaN
79585	FLJ22021	0.00	0.00	0.00	NaN
79586	FLJ22678	0.00	0.00	0.00	NaN
79587	FLJ12118	-0.09	-0.13	0.00	0.35
79590	MRPL24	0.00	0.13	0.00	-0.03
79591	FLJ13114	0.00	-0.13	-0.05	0.24
79594	FLJ12875	-0.09	0.00	0.00	0.20
79596	FLJ13449	-0.09	0.13	-0.10	0.50
79598	FLJ23047	0.00	0.00	0.00	NaN
79600	FLJ21127	0.00	0.00	0.00	NaN
79602	FLJ21432	0.00	0.00	0.05	0.39
79603	FLJ12089	0.00	0.00	0.00	NaN
79605	FLJ11413	0.09	0.00	0.00	-0.02
79608	FLJ11608	0.00	0.00	-0.05	0.13
79609	FLJ13920	0.00	0.00	0.00	NaN
79611	FLJ21963	0.00	0.13	0.00	-0.05
79612	FLJ22054	0.00	0.00	-0.05	0.36
79614	FLJ14054	0.00	0.00	0.15	0.07
79616	FLJ14166	0.00	0.00	0.00	NaN
79618	FLJ21616	-0.09	-0.25	-0.05	0.33
79621	FLJ11712	0.00	0.13	0.00	0.54
79622	FLJ22940	0.00	0.00	-0.05	0.39
79623	FLJ12691	0.00	0.00	0.00	NaN
79624	FLJ12910	0.00	0.00	0.00	NaN
79625	FLJ23191	0.00	0.00	0.00	NaN
79627	FLJ21079	0.00	0.00	0.05	0.05
79628	KIAA1985	0.00	0.00	0.00	NaN
79629	FLJ22709	-0.09	0.00	0.00	0.12
79630	FLJ23221	0.00	0.13	0.05	0.25
79631	FLJ13119	0.00	0.00	0.00	NaN
79632	FLJ13942	-0.09	0.00	0.05	-0.13
79633	FLJ23056	0.00	0.00	0.00	NaN
79634	FLJ23142	0.00	0.00	0.00	NaN
79635	FLJ13646	0.09	0.00	0.05	0.49
79637	FLJ22160	0.00	0.13	0.00	0.17
79639	FLJ22353	0.00	0.00	0.00	NaN
79640	FLJ23584	0.00	0.00	0.05	0.05
79641	FLJ22386	0.00	0.00	-0.05	0.42
79642	FLJ23548	0.00	0.00	0.00	NaN

79643	FLJ11749	0.00	0.00	-0.05	0.37
79644	FLJ13352	0.00	0.00	0.00	NaN
79645	FLJ11767	0.00	0.13	0.00	0.24
79646	FLJ12899	0.00	0.00	0.00	NaN
79647	FLJ12666	0.00	0.00	0.05	-0.05
79648	FLJ12847	-0.09	-0.25	-0.05	0.38
79650	FLJ13154	-0.09	0.00	0.00	0.20
79651	FLJ22341	0.00	0.13	0.00	0.12
79652	FLJ20898	0.00	0.00	0.00	NaN
79654	FLJ21156	0.00	0.00	0.00	NaN
79656	FLJ11588	0.00	0.00	0.00	NaN
79657	FLJ21908	0.00	0.00	0.00	NaN
79659	FLJ11756	0.00	0.13	0.00	-0.08
79661	NEIL1	0.00	0.00	0.00	NaN
79663	FLJ22623	0.00	0.00	0.00	NaN
79664	FLJ11896	0.00	0.00	0.00	0.44
79665	FLJ22060	0.18	0.13	0.00	0.67
79666	FLJ13187	0.00	-0.13	0.00	0.17
79667	FLJ13197	0.00	0.00	0.05	0.13
79668	FLJ21308	0.00	0.00	0.00	NaN
79669	FLJ23186	0.00	0.00	0.00	NaN
79670	FLJ13409	0.00	0.00	0.00	NaN
79671	FLJ21478	-0.09	0.00	-0.05	0.04
79672	FLJ12171	0.00	0.00	0.00	NaN
79673	FLJ12586	0.00	0.13	0.00	0.34
79675	FLJ21901	0.00	0.00	0.00	NaN
79676	FLJ13491	0.00	0.00	0.00	NaN
79677	FLJ22116	0.09	0.00	-0.10	0.34
79679	FLJ22418	0.09	0.00	0.10	-0.16
79680	FLJ21125	-0.09	0.00	0.00	0.00
79682	FLJ23468	0.00	0.00	-0.05	0.40
79683	FLJ20984	0.00	0.00	0.00	NaN
79684	FLJ23342	0.00	0.00	-0.10	0.25
79685	FLJ11526	0.00	0.00	0.00	NaN
79686	FLJ21276	0.00	0.00	0.00	NaN
79689	FLJ23153	0.09	0.00	0.00	-0.06
79690	GAL3ST-4	0.18	0.00	0.00	-0.03
79691	FLJ12960	0.00	0.00	0.00	NaN
79692	FLJ23393	0.00	0.00	0.00	NaN
79693	FLJ23476	0.00	0.00	0.00	NaN
79694	FLJ12838	-0.09	0.00	0.00	0.01
79695	FLJ21212	0.09	0.00	0.00	0.13
79696	FLJ23093	0.00	0.00	0.00	NaN
79697	FLJ21802	0.00	0.00	0.00	NaN
79698	FLJ13842	0.55	0.00	-0.05	0.54
79701	FLJ22222	0.00	0.00	0.00	NaN
79703	FLJ22531	0.00	0.00	0.05	0.02

79705	FLJ23119	0.00	0.00	0.00	NaN
79706	FLJ13902	0.18	0.00	0.00	0.48
79707	FLJ23323	0.00	0.00	0.00	NaN
79709	FLJ22329	-0.09	0.00	0.00	0.15
79711	FLJ23338	0.00	0.00	0.00	NaN
79712	FLJ11753	0.00	0.00	0.00	NaN
79713	FLJ22573	0.00	0.00	0.00	NaN
79714	FLJ12436	-0.09	0.00	-0.05	0.30
79716	NPEPL1	0.18	0.63	0.00	0.56
79717	FLJ11838	0.00	0.00	0.00	NaN
79719	FLJ11506	0.00	0.00	0.00	NaN
79720	FLJ12750	0.00	-0.13	0.00	0.19
79722	FLJ11795	0.00	0.00	0.00	NaN
79723	SUV39H2	0.00	0.00	0.00	NaN
79724	FLJ23436	0.00	0.00	0.00	NaN
79725	FLJ23320	0.00	0.00	0.00	NaN
79726	FLJ12270	0.00	0.00	0.05	0.27
79727	FLJ12457	-0.09	0.00	0.00	0.03
79728	FLJ21816	0.00	0.00	-0.05	0.20
79729	FLJ22938	0.00	0.00	0.00	NaN
79730	FLJ14001	0.00	0.00	0.05	0.32
79731	FLJ23441	0.27	0.00	0.00	0.53
79733	FLJ23311	0.00	0.00	-0.05	0.33
79734	FLJ12242	0.00	0.00	0.00	NaN
79735	FLJ12168	0.00	0.00	0.00	NaN
79736	FLJ22729	0.00	0.00	0.00	NaN
79738	FLJ23560	0.00	0.00	0.00	NaN
79739	FLJ23033	0.00	0.00	0.05	0.03
79740	FLJ23049	0.00	0.00	0.00	NaN
79741	FLJ13031	0.00	-0.13	0.05	-0.22
79744	FLJ23233	0.00	0.13	0.00	0.48
79746	FLJ20909	0.00	0.00	0.00	NaN
79747	FLJ23121	0.00	-0.13	0.00	0.16
79748	FLJ13993	0.00	0.00	0.00	NaN
79750	FLJ22419	0.00	0.00	0.00	NaN
79751	FLJ13044	-0.18	-0.13	-0.05	-0.04
79752	FLJ14007	0.00	0.00	0.05	0.22
79753	SNIP1	0.00	0.00	0.00	NaN
79754	ASB13	0.00	0.00	0.05	0.09
79755	FLJ13841	0.00	0.00	0.00	NaN
79758	FLJ13639	0.00	0.00	0.00	NaN
79759	FLJ13479	0.00	0.00	0.00	NaN
79760	FLJ13956	0.09	0.00	0.00	0.13
79762	FLJ14146	0.09	0.00	0.05	0.33
79763	FLJ23469	0.09	0.00	0.05	0.28
79767	ELMO3	-0.09	0.00	0.00	0.29
79768	FLJ22557	0.00	0.00	0.00	NaN

79770	FLJ22625	0.00	-0.13	0.00	0.33
79772	FLJ22344	0.00	0.00	0.00	NaN
79774	FLJ22474	-0.09	-0.13	-0.05	0.01
79776	FLJ20980	0.00	0.25	0.00	-0.09
79777	FLJ13322	0.00	-0.13	0.00	-0.23
79778	FLJ23471	0.00	0.00	0.05	0.16
79781	FLJ22527	0.00	0.00	-0.05	0.03
79782	FLJ23259	0.09	0.00	0.00	-0.04
79783	C7orf10	0.00	0.00	0.00	NaN
79785	FLJ22655	0.00	0.00	0.00	NaN
79786	C16orf44	-0.09	0.00	-0.05	0.41
79788	FLJ14345	0.09	0.00	-0.05	0.09
79789	calmin	0.00	0.00	0.00	NaN
79791	FLJ22477	-0.09	-0.13	-0.05	-0.01
79792	FLJ12150	0.18	0.00	0.10	0.05
79794	FLJ21415	0.00	-0.13	0.00	0.23
79796	DIBD1	0.00	0.00	0.00	NaN
79797	FLJ12827	0.00	0.00	0.00	NaN
79799	FLJ21934	0.00	0.00	0.05	-0.08
79800	ALS2CR8	0.00	0.00	0.00	NaN
79801	FLJ22009	-0.09	0.00	-0.05	0.19
79802	FLJ13840	0.09	0.00	0.00	-0.07
79803	FLJ22501	0.00	-0.13	-0.05	0.10
79804	FLJ11539	0.00	0.00	-0.05	0.21
79805	FLJ12505	0.00	0.00	0.00	NaN
79807	FLJ13273	0.00	0.00	0.00	NaN
79809	FLJ11457	0.00	0.00	0.00	NaN
79810	FLJ12598	0.00	0.00	0.00	NaN
79811	FLJ13213	0.09	0.00	0.05	0.16
79812	ENDOGLYX1	0.50	0.00	0.00	0.03
79813	Eu-HMTase1	0.00	0.00	0.05	0.02
79814	AGMAT	0.00	0.00	0.00	NaN
79815	FLJ13955	0.00	0.00	0.00	NaN
79817	FLJ13204	0.00	-0.13	-0.10	0.02
79818	FLJ21603	0.00	0.13	-0.05	0.30
79819	FLJ23129	0.00	0.00	0.00	NaN
79820	FLJ14298	0.00	0.13	0.00	0.28
79822	FLJ10312	0.00	0.00	0.00	NaN
79823	FLJ23451	0.00	0.00	0.00	NaN
79825	FLJ12057	0.00	0.00	0.00	NaN
79828	FLJ13984	0.00	0.00	0.00	NaN
79829	FLJ13848	0.00	0.00	0.00	NaN
79830	FLJ23151	0.00	0.00	0.00	NaN
79831	FLJ13798	0.00	0.00	0.00	NaN
79832	FLJ21924	0.00	0.00	0.00	NaN
79833	GEMIN6	0.00	0.13	0.00	-0.13
79834	FLJ21140	0.00	0.00	0.00	NaN

79837	FLJ22055	0.00	0.00	0.00	NaN
79838	FLJ13593	0.00	0.00	0.00	NaN
79839	FLJ23594	-0.09	0.00	-0.10	0.08
79840	FLJ12610	0.00	0.00	0.00	NaN
79841	FLJ23598	0.00	0.00	0.05	-0.13
79842	FLJ23392	0.00	0.00	0.00	NaN
79843	FLJ22746	0.00	0.00	0.00	NaN
79844	FLJ13153	0.09	0.13	0.30	0.50
79845	FLJ12526	0.09	-0.25	-0.05	-0.16
79846	FLJ21062	0.09	0.00	0.00	0.44
79847	FLJ22529	0.00	-0.13	-0.05	0.15
79848	FLJ22490	0.00	0.00	0.00	NaN
79849	FLJ22756	-0.09	0.00	-0.10	-0.11
79850	FLJ22282	0.00	-0.13	0.00	-0.07
79852	FLJ22408	0.00	0.00	0.05	-0.15
79853	FLJ22800	0.00	0.00	0.00	NaN
79854	FLJ22639	0.00	0.00	0.05	0.33
79857	FLJ13224	0.00	0.00	0.00	NaN
79858	NEK11	0.00	0.00	0.00	NaN
79861	FLJ21665	0.00	0.00	0.05	-0.09
79862	FLJ12606	0.00	0.13	0.00	-0.01
79863	FLJ21172	-0.09	0.00	-0.25	0.54
79864	FLJ23554	0.00	0.00	-0.05	0.08
79865	FLJ13693	0.00	0.00	0.00	NaN
79866	FLJ22624	-0.09	0.13	-0.15	0.37
79867	FLJ12975	0.00	0.00	0.00	NaN
79869	FLJ12529	-0.09	0.00	0.00	0.21
79870	BAALC	0.18	0.00	0.00	0.04
79871	FLJ13150	0.00	0.00	0.05	0.04
79872	HAKAI	0.09	0.00	0.00	-0.08
79873	FLJ22494	-0.09	-0.25	-0.10	0.25
79874	FLJ23282	0.00	0.00	0.00	NaN
79875	FLJ13710	0.00	0.00	0.00	NaN
79876	FLJ23251	0.00	0.00	0.00	NaN
79877	FLJ22955	0.00	-0.13	0.00	0.15
79879	FLJ22349	0.00	0.00	0.05	0.08
79882	FLJ11806	0.00	0.13	0.00	0.00
79883	FLJ23447	0.00	0.00	0.00	NaN
79884	FLJ21159	0.00	0.00	0.00	0.16
79885	FLJ22237	0.00	0.00	-0.05	0.29
79886	FLJ13657	0.00	-0.25	-0.05	0.63
79887	FLJ22662	-0.09	0.00	0.00	0.08
79888	FLJ12443	0.00	0.13	0.10	0.43
79890	RIN3	0.00	0.00	0.00	NaN
79891	FLJ23506	0.00	0.13	0.00	0.48
79892	FLJ13081	0.00	0.00	-0.05	0.18
79893	LZK1	0.00	-0.13	0.00	0.18

79894	FLJ22301	0.00	0.00	0.00	NaN
79895	KIAA1939	0.18	0.00	0.00	0.16
79896	FLJ22002	-0.09	0.00	0.05	0.45
79897	FLJ22638	0.00	0.00	0.00	NaN
79898	FLJ13590	0.00	0.00	-0.05	0.22
79899	FLJ14213	0.00	0.13	0.05	-0.14
79901	FLJ23462	0.00	0.00	0.00	NaN
79902	FLJ12549	0.00	0.13	0.00	0.11
79903	FLJ14154	0.00	0.00	0.00	NaN
79905	FLJ21240	0.00	0.00	0.00	NaN
79906	FLJ13941	0.00	0.00	0.00	NaN
79908	FLJ21458	0.00	0.13	-0.05	0.02
79912	FLJ22028	0.00	0.00	0.00	NaN
79913	FLJ12785	0.00	-0.13	0.00	0.08
79915	FLJ12735	0.00	0.13	0.00	-0.27
79918	FLJ21148	0.00	0.00	0.00	NaN
79919	FLJ22671	0.00	0.00	-0.05	-0.04
79922	FLJ22578	0.00	-0.13	0.00	0.03
79923	FLJ12581	-0.09	0.00	0.00	-0.15
79924	FLJ21135	0.00	-0.25	0.00	-0.30
79927	FLJ14050	0.00	0.00	0.00	NaN
79929	FLJ12748	0.09	0.00	0.00	0.71
79930	FLJ22570	0.00	0.00	0.00	NaN
79931	FLJ21162	0.00	0.00	0.00	NaN
79932	FLJ14225	0.00	0.00	0.00	NaN
79933	FLJ12921	0.09	0.00	0.00	-0.08
79934	FLJ12229	0.00	0.00	0.00	NaN
79935	FLJ13265	0.00	0.00	0.00	NaN
79937	CASPR3	0.00	0.00	0.05	-0.10
79939	FLJ14251	0.00	0.00	0.00	NaN
79940	FLJ13189	0.00	0.00	0.05	0.11
79943	FLJ14129	0.18	0.00	0.10	0.10
79944	FLJ12618	0.00	0.00	0.00	NaN
79946	FLJ14280	0.00	-0.13	-0.05	-0.24
79947	FLJ13102	-0.09	0.00	0.00	0.19
79949	FLJ23537	0.09	-0.13	-0.05	0.37
79953	C20orf39	0.00	0.00	0.00	NaN
79954	FLJ14075	0.00	0.00	-0.05	0.01
79955	FLJ23209	0.00	-0.13	-0.05	0.12
79956	FLJ23309	-0.09	-0.13	0.00	0.34
79957	FLJ22672	0.00	0.13	0.00	-0.07
79958	FLJ22757	0.00	0.00	0.00	NaN
79959	FLJ12542	0.00	0.00	0.00	NaN
79960	FLJ22479	0.00	0.00	0.00	NaN
79961	FLJ22457	0.09	0.00	0.00	0.34
79962	FLJ13236	0.00	0.00	0.00	NaN
79963	FLJ14297	0.00	-0.13	-0.10	0.12

79966	FLJ21032	0.00	0.00	0.00	NaN
79968	FLJ12973	0.00	0.00	0.00	NaN
79969	FLJ13158	0.00	0.00	0.00	NaN
79970	FLJ12700	0.00	0.00	-0.05	0.32
79971	FLJ23091	0.00	0.00	0.00	NaN
79973	FLJ14356	0.00	0.00	0.00	NaN
79974	FLJ21986	0.00	0.13	0.00	0.15
79977	FLJ13782	0.18	0.00	0.00	0.21
79980	C20orf172	0.00	-0.13	0.00	0.20
79981	FLJ22615	0.00	0.00	0.05	-0.21
79982	FLJ14281	0.00	0.00	0.00	NaN
79986	FLJ12985	0.09	0.00	-0.05	0.33
79987	POLYDOM	0.00	0.00	0.00	NaN
79989	FLJ12571	0.00	0.00	0.00	NaN
79990	FLJ21019	0.00	-0.13	0.00	-0.11
79991	FLJ22559	0.00	-0.13	-0.05	0.23
79998	FLJ12056	0.00	0.00	0.00	NaN
80000	FLJ13687	0.00	0.00	0.00	NaN
80003	FLJ11383	0.09	0.00	0.00	-0.10
80004	FLJ21918	-0.09	-0.13	0.00	-0.10
80005	FLJ21034	-0.09	-0.25	-0.05	0.12
80006	FLJ13611	0.00	0.00	0.00	NaN
80007	FLJ13490	0.09	0.00	-0.05	0.70
80008	FLJ23235	0.00	0.00	0.05	-0.16
80010	FLJ12888	0.00	0.00	0.00	NaN
80011	NIP30	-0.09	0.00	0.00	0.28
80012	FLJ12729	0.09	0.00	0.00	0.25
80013	FLJ13397	0.00	0.00	0.05	-0.10
80014	FLJ22029	0.00	0.00	-0.05	0.26
80017	FLJ20950	0.00	0.13	0.00	0.13
80019	FLJ11807	0.00	0.00	-0.05	-0.04
80020	FLJ23322	-0.09	0.00	0.00	0.07
80021	FLJ23375	0.00	0.00	0.00	NaN
80022	FLJ22686	0.18	0.13	0.00	0.17
80023	C20orf98	0.00	0.00	0.05	0.33
80024	FLJ22233	0.00	0.00	0.00	NaN
80025	C20orf48	0.00	0.00	-0.05	0.30
80028	FLJ11467	0.00	0.00	0.10	0.28
80031	FLJ11598	0.00	0.00	0.00	NaN
80032	FLJ11637	0.00	0.00	0.00	NaN
80034	FLJ11703	0.00	0.00	0.00	NaN
80035	FLJ11722	0.00	0.00	-0.05	-0.11
80036	FLJ11726	0.00	0.00	0.00	NaN
80039	FLJ11800	-0.27	0.13	-0.35	0.02
80045	FLJ12132	0.00	0.00	0.00	NaN
80054	FLJ12355	0.00	0.00	0.05	-0.21
80055	FLJ12377	0.00	0.00	0.00	NaN

80059	FLJ12568	0.00	0.00	0.00	NaN
80063	FLJ12668	0.00	0.00	0.00	NaN
80067	FLJ13096	0.00	0.00	0.00	NaN
80069	FLJ13162	0.00	0.00	0.00	NaN
80070	FLJ13166	0.00	0.13	0.00	0.05
80071	FLJ13215	0.00	0.00	-0.10	0.15
80072	FLJ13315	0.00	0.00	0.00	NaN
80086	TUBA4	0.00	0.00	0.00	NaN
80095	FLJ14260	0.00	0.13	0.00	0.33
80097	FLJ14346	0.00	0.00	-0.05	-0.07
80099	FLJ21075	0.00	0.00	0.00	NaN
80108	FLJ21628	0.00	0.00	0.00	NaN
80110	FLJ21941	0.00	0.00	-0.05	0.32
80111	FLJ22173	0.00	0.00	0.00	NaN
80114	FLJ22476	0.00	0.00	0.00	NaN
80115	FLJ22582	-0.09	0.00	0.05	0.23
80117	FLJ22595	0.00	0.00	0.00	NaN
80122	FLJ23074	0.00	0.00	-0.05	0.03
80124	FLJ23132	0.00	0.00	0.05	0.31
80125	FLJ23168	0.00	0.00	0.00	NaN
80127	FLJ23189	0.00	0.00	0.00	NaN
80128	FLJ23229	0.27	0.13	0.00	0.34
80129	FLJ23305	0.00	0.00	0.00	NaN
80131	FLJ23420	0.00	0.00	0.00	NaN
80133	FLJ23550	0.00	0.00	0.00	NaN
80135	RPF1	0.00	0.00	0.05	0.55
80142	C9orf15	-0.09	0.00	0.00	0.24
80143	FLJ21168	0.18	0.00	0.10	0.72
80144	FLJ22031	0.00	0.00	0.00	NaN
80145	FLJ23445	0.09	0.00	-0.05	0.53
80146	UXS1	0.00	0.00	0.00	NaN
80148	FLJ22378	-0.09	0.00	-0.25	0.36
80149	FLJ23231	0.00	0.00	0.00	NaN
80150	FLJ22316	-0.09	0.00	0.00	-0.19
80152	FLJ13111	-0.09	-0.13	0.00	0.11
80153	FLJ21128	0.00	0.00	0.00	NaN
80154	FLJ22795	0.00	0.00	0.00	NaN
80155	FLJ13340	0.00	0.00	0.00	NaN
80157	FLJ21511	0.00	0.00	0.00	NaN
80162	FLJ22635	-0.27	-0.13	-0.15	0.28
80167	FLJ21106	0.00	0.00	0.00	NaN
80168	FLJ22644	0.18	0.00	0.05	0.17
80169	FLJ22170	0.00	0.00	0.00	NaN
80173	CMG1	0.00	-0.25	-0.05	0.51
80174	ASKL1	0.00	-0.13	0.00	-0.11
80176	FLJ22393	0.00	0.00	0.00	NaN
80177	FLJ21269	0.00	0.00	0.00	NaN

80178	FLJ13909	0.00	0.00	0.00	NaN
80179	FLJ22865	0.00	-0.13	0.00	-0.04
80183	FLJ21562	0.00	0.00	-0.05	0.07
80184	FLJ13615	0.09	0.13	0.00	0.23
80185	FLJ23263	0.18	-0.25	-0.05	0.57
80194	FLJ21749	0.36	0.00	0.05	0.54
80195	FLJ13263	0.09	0.00	0.00	0.23
80196	RNF34	0.00	-0.13	0.00	0.41
80198	MUS81	-0.09	0.00	0.05	0.37
80199	FLJ22688	0.00	0.00	0.00	NaN
80201	FLJ22761	0.00	0.00	0.00	NaN
80204	FLJ12673	0.00	0.00	0.00	NaN
80205	FLJ12178	0.00	0.00	0.00	NaN
80206	KIAA1695	0.00	-0.13	-0.30	0.14
80207	OPA3	0.09	0.00	0.00	0.26
80208	FLJ21439	0.00	0.00	0.00	NaN
80209	FLJ12661	0.00	0.00	0.00	NaN
80210	FLJ12584	0.00	0.00	0.00	NaN
80212	FLJ22471	0.00	0.00	0.00	NaN
80213	BLP2	0.00	0.00	0.00	NaN
80215	FLJ20856	0.00	0.00	-0.05	-0.08
80216	FLJ22670	0.00	0.00	0.00	NaN
80217	FLJ22944	0.09	-0.13	-0.05	0.21
80218	FLJ13194	0.00	0.00	0.00	NaN
80219	FLJ13448	0.00	0.00	0.00	NaN
80221	FLJ20920	0.00	0.25	0.00	0.50
80222	FLJ12528	0.00	0.13	0.05	0.10
80223	RCP	0.55	-0.13	0.00	0.58
80224	FLJ12660	0.00	0.00	0.00	NaN
80227	FLJ11848	0.09	0.13	0.00	0.58
80228	CBCIP2	0.18	0.00	0.00	0.34
80230	FLJ22251	0.00	0.00	0.05	0.23
80232	FLJ21016	0.09	0.00	0.00	0.43
80233	FLJ22175	0.00	0.00	0.00	NaN
80235	FLJ12768	0.18	0.00	0.00	-0.13
80237	FLJ22637	0.00	0.00	0.00	NaN
80243	FLJ12987	0.00	0.00	0.00	NaN
80254	FLJ13386	0.00	0.00	0.00	NaN
80255	FLJ22004	0.00	0.00	0.00	NaN
80256	FLJ11560	-0.09	0.13	0.10	0.41
80262	FLJ12076	-0.09	0.00	0.00	-0.22
80263	FLJ13181	0.09	0.00	0.10	0.23
80264	FLJ13659	0.00	-0.25	0.00	0.38
80267	C1orf22	0.00	0.00	0.05	0.50
80271	ITPKC	0.00	0.00	0.00	NaN
80273	HMGE	0.00	-0.13	-0.10	0.06
80279	C53	0.00	0.00	0.00	0.28

80301	PP1628	0.00	0.00	0.00	NaN
80303	FLJ13612	0.00	0.00	0.00	NaN
80304	FLJ21945	0.09	0.00	-0.05	0.21
80305	PP2447	0.00	-0.25	0.00	0.24
80306	EG1	0.00	0.00	-0.05	0.28
80307	FER1L4	0.00	0.00	0.00	NaN
80308	PP591	0.09	0.13	0.00	0.32
80310	SCDGF-B	0.00	0.13	0.00	0.06
80317	ZNF306	0.00	0.00	0.00	NaN
80319	IDAX	0.00	0.00	0.00	NaN
80321	BITE	0.00	0.00	0.00	NaN
80323	SE57-1	-0.09	0.00	-0.20	0.34
80324	PUS1	0.00	0.00	0.00	NaN
80328	ULBP2	0.00	0.00	-0.05	0.06
80329	ULBP1	0.00	0.00	-0.05	0.25
80335	PRO2730	-0.09	0.00	-0.10	0.35
80339	C22orf20	0.00	0.00	0.05	-0.08
80342	DJ434O14.3	0.00	0.00	0.00	NaN
80344	PRO2389	0.00	0.00	0.00	NaN
80345	FLJ22191	0.00	0.00	0.00	NaN
80346	FLJ22246	-0.09	-0.25	-0.10	0.26
80347	NBP	0.00	-0.13	0.00	0.30
80349	REC14	0.00	0.00	0.00	NaN
80350	APOARGC	0.00	0.00	-0.05	-0.05
80351	TNKS2	0.00	0.00	0.00	NaN
80352	HZFW1	0.00	0.00	0.00	NaN
80380	PDL2	-0.09	-0.13	-0.05	0.18
80700	UBXD1	0.00	0.00	0.00	NaN
80704	SLC19A3	0.00	0.00	0.00	NaN
80705	TSGA10	0.00	0.00	0.00	NaN
80723	MGC3295	0.00	0.00	-0.05	0.12
80724	MGC5601	0.00	0.00	0.00	NaN
80736	C6orf29	0.00	0.00	0.00	NaN
80737	C6orf27	0.00	0.00	0.00	NaN
80739	C6orf25	0.00	0.00	0.00	NaN
80740	C6orf24	0.00	0.00	0.00	NaN
80741	C6orf20	0.00	0.00	0.00	NaN
80745	MGC2454	0.00	0.00	0.00	NaN
80746	MGC2776	0.00	0.00	0.00	NaN
80755	MGC2744	0.00	-0.13	0.00	0.20
80757	MGC4659	0.00	0.00	0.00	NaN
80758	MGC10772	0.00	0.13	0.00	0.02
80759	MGC10818	0.00	0.00	0.05	-0.13
80760	MGC10848	0.00	0.00	0.00	NaN
80761	MGC10902	0.00	0.00	0.00	NaN
80762	MGC10924	0.00	0.00	0.00	NaN
80763	MGC10946	0.00	0.00	0.00	NaN

80764	MGC10963	0.00	0.13	0.00	0.46
80765	STARD5	0.00	0.00	0.00	NaN
80772	MGC10334	0.00	0.00	0.05	0.00
80774	MGC10986	0.27	0.13	0.00	0.04
80775	MGC10993	0.00	0.00	0.00	NaN
80776	MGC4093	0.00	0.00	-0.05	-0.05
80777	CYB5-M	0.00	-0.13	0.05	0.46
80778	MGC10520	0.18	0.00	0.10	0.45
80781	COL18A1	0.00	0.00	0.00	NaN
80789	KIAA1698	0.00	0.00	0.00	NaN
80830	APOL6	0.00	0.00	0.00	NaN
80831	APOL5	0.00	0.00	0.00	NaN
80853	KIAA1718	0.00	0.00	0.00	NaN
80864	NG3	0.00	0.00	0.00	NaN
80868	HCGIV-6	0.00	-0.13	-0.15	0.07
80895	ILKAP	0.00	0.00	-0.05	0.46
80896	C1orf13	0.00	0.00	0.00	NaN
80975	TMPRSS5	0.00	0.00	0.00	NaN
81025	GJA10	0.00	0.00	0.00	NaN
81027	TUBB1	0.18	0.25	0.00	-0.07
81029	WNT5B	0.00	0.00	0.05	-0.01
81031	SLC2A10	0.00	0.00	0.00	NaN
81033	ERG2	0.27	0.13	0.00	0.24
81034	LOC81034	0.18	0.00	0.05	0.18
81035	COLEC12	0.00	0.00	0.00	NaN
81285	OR51E2	0.00	-0.13	0.00	0.08
81490	PTDSS2	-0.27	-0.13	-0.10	0.42
81491	GPR63	0.00	0.00	0.00	NaN
81492	RSHL1	0.09	0.00	0.00	0.03
81493	SYNCOILIN	0.00	0.00	0.00	NaN
81494	FHR5	0.00	0.13	0.00	0.02
81501	LOC81501	0.18	-0.25	0.05	-0.14
81533	CDA08	-0.09	0.00	0.00	0.33
81537	LOC81537	0.00	0.00	-0.05	0.20
81539	SLC38A1	0.00	0.00	0.00	NaN
81542	TXNDC	0.00	0.00	0.05	0.18
81543	LRRC3	0.00	0.00	0.00	NaN
81544	PP1665	0.18	0.00	0.00	0.31
81545	SP329	0.00	0.00	0.00	NaN
81550	FLJ21007	0.00	0.00	-0.10	0.14
81551	STMN4	-0.09	-0.25	-0.10	-0.29
81552	DKFZP564K08	0.00	0.00	0.10	0.59
81553	DKFZP566A15	0.09	0.00	-0.10	0.32
81554	DKFZP434D04	-0.09	0.00	0.00	0.22
81555	AF140225	0.00	0.00	0.00	NaN
81556	DKFZP564O16	0.00	0.00	0.00	NaN
81558	LOC81558	0.00	0.25	0.00	0.58

81562	DKFZP564L242	0.00	0.00	0.00	NaN
81563	C1orf21	0.00	0.00	0.05	0.00
81565	NUDEL	0.00	0.00	0.00	NaN
81566	C12orf22	0.00	0.00	0.00	NaN
81567	MGC3178	0.00	0.00	0.00	NaN
81569	LOC81569	-0.09	0.00	0.00	-0.15
81570	SKD3	0.00	0.00	0.00	NaN
81571	GL012	0.00	0.00	0.00	NaN
81575	DKFZP434F031	-0.09	0.00	0.00	0.16
81576	MGC10471	0.00	0.00	0.00	NaN
81577	MGC11335	-0.09	-0.13	0.00	0.17
81578	COL21A1	0.00	0.13	0.00	-0.03
81579	PLA2G12	0.00	0.00	0.00	NaN
81602	NYD-SP15	0.00	0.13	0.00	0.39
81603	RNF27	0.00	-0.13	-0.05	0.26
81605	MGC2668	0.00	0.00	0.00	NaN
81606	DKFZP566J091	0.00	0.00	0.00	NaN
81608	DKFZP586K07	0.00	0.00	0.00	NaN
81609	MY014	0.00	0.00	0.05	0.34
81611	LANP-L	0.00	0.13	0.05	0.41
81616	PRTD-NY3	0.00	0.00	0.00	NaN
81617	FLJ12577	0.00	0.13	0.00	0.14
81618	ITM3	0.00	0.00	0.00	NaN
81619	MGC11352	0.09	0.00	0.00	0.22
81620	CDT1	-0.09	-0.13	-0.05	0.33
81621	FKSG28	0.00	-0.13	-0.05	0.12
81622	UNC93B1	0.18	0.00	0.05	0.33
81623	DEFB126	0.00	0.00	0.05	-0.01
81624	DIAPH3	0.00	0.00	-0.10	-0.08
81626	C1orf14	0.00	0.00	0.00	NaN
81627	C1orf25	0.00	0.00	0.10	0.43
81628	THG-1	0.18	0.00	0.00	0.21
81631	MAP1A/1BLC3	-0.09	-0.13	-0.05	0.51
81669	HCLA-ISO	0.00	0.00	0.00	NaN
81671	VMP1	0.18	0.13	0.00	0.71
81689	MGC4276	0.00	0.00	0.00	NaN
81691	LOC81691	0.00	0.00	0.00	NaN
81696	OR5V1	0.00	0.00	0.00	NaN
81698	C15orf5	0.00	0.00	0.00	NaN
81788	DKFZP434J037	0.00	0.13	0.00	0.35
81789	DKFZP761E21	0.00	0.00	0.00	NaN
81790	DKFZP564A02	0.09	0.00	-0.05	0.40
81792	ADAMTS12	0.00	0.00	0.10	0.14
81796	OATPRP4	0.00	0.00	0.00	NaN
81797	OR12D3	0.00	0.00	0.00	NaN
81831	NETO2	-0.09	0.00	-0.05	0.17
81833	SPACA1	0.00	0.00	0.00	NaN

81847	DKFZP434O14	0.00	0.00	0.05	0.66
81849	MGC3184	0.00	0.00	0.00	NaN
81850	KRTAP1.3	0.00	0.25	0.00	0.24
81851	KRTAP1.1	0.00	0.25	0.00	-0.08
81853	MGC1223	0.00	0.00	0.05	0.50
81854	MGC3771	0.00	0.00	0.00	NaN
81855	BA108L7.2	0.00	-0.13	-0.05	0.30
81856	MGC5384	0.09	0.00	-0.05	0.03
81858	DKFZP434N19	0.18	0.00	0.10	0.34
81870	KRTAP9.9	0.00	0.38	-0.05	0.00
81873	MGC3038	0.00	0.00	0.00	NaN
81875	FLJ12671	0.00	0.13	0.00	0.28
81876	RAB1B	-0.09	0.00	0.05	0.11
81888	HT036	0.00	0.00	0.00	NaN
81890	TGT	0.00	-0.13	0.00	0.06
81892	DC50	0.00	0.00	0.00	NaN
81893	LAT1-3TM	0.00	0.00	0.00	NaN
81894	MRS3/4	0.00	-0.13	-0.05	0.39
81926	MGC:5244	0.00	0.00	0.00	NaN
81929	SEC13L	0.00	0.00	0.00	NaN
81930	DKFZP434G22	0.00	0.00	0.00	NaN
81931	FLJ12488	0.00	0.00	0.00	NaN
81932	MGC12904	0.00	0.00	0.00	NaN
83394	NIR1	0.00	0.00	0.00	NaN
83439	TCF-3	0.00	0.00	0.00	NaN
83440	DKFZP434B19	0.00	0.00	0.00	NaN
83442	SH3BGRL3	-0.09	0.00	0.00	0.22
83443	MGC3133	0.00	0.00	0.00	NaN
83444	PAPA-1	0.00	0.00	0.00	NaN
83445	MGC3146	-0.09	0.00	0.00	-0.24
83446	DKFZP434K11	0.00	0.00	0.00	NaN
83447	DKFZP434N12	0.00	0.00	0.05	0.07
83448	DKFZP434G14	0.00	0.13	0.00	0.12
83449	DKFZP434G13	0.00	0.00	0.05	-0.19
83450	DKFZP586M11	0.00	0.00	0.05	-0.06
83451	PP1226	-0.09	0.00	0.00	0.16
83452	RAB33B	0.00	0.00	0.00	NaN
83460	MGC2963	0.00	0.00	0.00	NaN
83461	MGC2577	-0.09	0.00	0.00	0.17
83463	MAD3	0.00	0.13	0.00	-0.10
83464	DKFZP564D03	0.00	0.00	0.00	NaN
83468	LOC83468	0.00	0.00	0.00	NaN
83475	MGC4293	0.00	0.00	0.00	NaN
83478	DKFZP564B11	0.00	0.00	0.00	NaN
83480	FKSG32	-0.09	0.00	-0.10	0.37
83481	EPPK1	0.18	0.00	0.10	0.25
83482	SCRT1	0.18	0.00	0.10	0.00

83483	PLVAP	-0.09	0.00	0.00	-0.07
83637	DKFZP761I212	0.00	0.00	0.00	NaN
83638	P5326	-0.09	0.00	0.05	0.26
83660	TLN2	0.00	0.00	0.00	NaN
83696	MGC4737	0.18	0.00	0.05	0.30
83714	DKFZP761G19	0.00	0.00	0.05	0.03
83716	DKFZP434B04	-0.09	0.00	-0.05	0.19
83729	MGC4638	0.00	0.00	0.00	NaN
83737	ITCH	0.00	0.13	0.00	0.35
83743	GRWD	0.09	0.00	0.00	0.15
83744	BA526D8.4	0.00	0.00	0.00	NaN
83759	MGC10871	0.00	0.00	0.05	0.06
83941	BBP	0.00	0.00	0.00	NaN
83988	NCALD	0.18	0.13	0.00	-0.19
83989	DKFZP564D17	0.00	0.00	0.00	NaN
84059	VLGR1	0.00	0.00	0.00	NaN
84060	DKFZP564O05	0.18	0.00	0.00	0.55
84065	DKFZP564D04	-0.09	0.00	0.00	0.23
84079	DKFZP434L07	0.00	0.00	0.05	0.13
84084	RAB6C	0.00	0.00	-0.05	-0.02
84099	ID2B	0.09	0.00	-0.05	-0.03
84107	ZIC4	0.00	0.00	0.00	NaN
84124	FLJ12298	0.18	0.00	0.00	0.41
84148	MYST1	0.00	0.00	0.00	NaN
84168	TEM8	0.00	0.00	0.00	NaN
84172	Rpo1-2	0.00	0.00	0.00	NaN
84179	FLJ22269	0.00	-0.13	-0.10	-0.21
84193	FLJ23027	0.00	0.00	0.00	NaN
84196	FLJ23277	-0.09	0.00	0.00	0.15
84218	DKFZp434P22	0.00	1.00	0.00	0.68
84220	RANBP2L1	0.00	0.00	-0.05	0.07
84243	DKFZp667O24	-0.09	-0.13	0.00	0.30
84263	MGC10940	0.00	0.00	0.00	NaN
84271	KIAA1649	0.00	0.00	0.00	NaN
84272	MGC11061	0.00	0.00	0.00	NaN
84296	MGC14799	0.45	-0.13	-0.05	0.22
84298	MGC14817	0.09	0.00	0.00	-0.03
84364	ZNF289	0.00	0.00	0.00	NaN
84436	KIAA1827	0.09	0.00	-0.05	0.16
84516	MGC3248	0.00	0.00	-0.05	0.55
84525	SMAP31	0.00	0.00	0.00	NaN
84549	LOC84549	0.09	-0.25	-0.05	0.61
84561	SLC12A8	0.00	0.00	0.00	NaN
84612	PARD6B	0.18	0.13	0.00	0.04
84617	TUBB-5	0.00	0.00	0.00	NaN
84658	EMR3	0.00	-0.13	0.00	-0.25
84669	NY-REN-60	0.18	0.13	0.00	0.61

84705	GTPBG3	-0.09	0.00	0.00	0.08
84720	MGC3079	-0.09	0.13	0.10	0.60
84722	DDA3	0.09	0.00	0.05	0.43
84747	MGC5139	0.00	-0.13	0.00	0.19
84752	MGC4655	-0.09	0.00	0.00	0.08
84759	MGC10882	0.00	0.00	0.00	NaN
84779	MGC10646	0.00	0.00	0.00	NaN
84789	MGC2889	0.18	0.00	0.05	0.11
84790	TUBA6	0.00	0.00	0.00	NaN
84809	MGC12760	0.09	0.00	-0.10	0.52
84818	IL17RL	0.00	0.00	0.00	NaN
84820	MGC13098	0.00	0.00	0.00	NaN
84859	MGC4126	0.18	0.00	0.00	0.35
84861	FLJ14360	0.00	0.13	0.05	0.23
84864	FLJ14393	0.00	0.00	0.00	NaN
84890	FLJ14547	0.00	0.00	0.00	NaN
84901	FLJ14639	0.00	0.00	0.00	NaN
84928	FLJ14803	0.00	0.25	0.05	0.36
84934	FLJ14827	0.00	0.00	0.00	NaN
84951	FLJ14950	0.00	0.38	0.00	0.04
84975	MGC11308	0.00	0.00	0.00	NaN
84986	MGC14258	0.00	0.00	-0.05	0.12
85002	MGC16279	0.00	-0.25	-0.15	0.14
85236	H2B/S	0.00	0.00	0.00	NaN
85352	KIAA1644	0.00	0.00	0.05	-0.01
85359	DGCR6L	0.00	0.00	0.05	0.13
85360	7h3	0.00	0.00	0.05	0.43
85363	TRIM5	0.00	-0.13	-0.05	0.13
85377	KIAA1668	0.00	0.00	0.05	0.14
85452	KIAA1751	0.00	0.00	0.00	NaN
85453	KIAA1750	0.00	-0.25	0.00	0.14
85459	KIAA1731	0.00	0.00	-0.10	0.12
85476	EFG1	0.00	0.00	0.00	NaN
85477	SCIN	0.00	0.13	0.05	0.00
89781	HPS4	0.00	0.13	0.00	0.67
89845	FLJ00002	0.00	0.00	0.00	NaN
89853	FLJ00001	0.00	0.00	-0.05	0.12
89870	TRIM15	0.00	0.00	0.00	NaN
89874	SLC25A21	0.00	0.00	0.00	NaN
89910	UBE3B	0.00	0.00	0.00	NaN
89927	BC008967	0.00	0.00	0.00	NaN
89941	LOC89941	0.00	0.00	-0.05	0.32
89944	LOC89944	-0.09	0.00	-0.10	-0.15
90011	FLJ00060	0.09	0.00	0.00	-0.09
90233	LOC90233	0.00	0.13	0.00	-0.05
90273	R29124_1	0.00	0.00	-0.05	0.15
90326	LOC90326	0.00	0.00	0.00	NaN

90355	LOC90355	0.00	0.00	0.00	NaN
90379	LOC90379	0.00	0.00	0.00	NaN
90480	PLINP-1	0.00	0.00	0.00	NaN
90627	GT650	0.00	0.00	0.00	NaN
90634	CG018	0.00	0.00	0.00	NaN
90864	SSB-3	0.00	0.00	0.00	NaN
90865	DVS27	-0.09	-0.13	-0.05	0.39
90956	MGC20727	0.00	0.00	0.00	NaN
90993	OASIS	0.00	0.00	0.00	NaN
91137	LOC91137	0.00	0.00	0.00	NaN
91227	GGTL4	0.09	0.00	0.00	0.16
91289	BC002942	0.00	-0.25	0.00	0.17
91300	LOC91300	0.00	0.00	0.00	NaN
91369	MGC15396	0.00	0.38	0.00	0.42
91543	cig5	0.00	0.00	-0.05	0.05
91647	ATP12	0.00	0.00	0.05	-0.04
91746	KIAA1966	0.00	0.00	0.00	NaN
91754	NEK9	0.00	0.00	0.00	NaN
91860	MGC4809	0.00	0.00	0.00	NaN
91949	COG7	0.00	0.00	-0.05	0.35
91977	MYOZ3	0.00	0.00	0.00	NaN
92086	GGTLA4	0.00	0.00	0.05	-0.01
92170	LOC92170	0.00	0.00	-0.10	0.52
92211	KIAA1775	0.09	0.00	-0.05	-0.16
92342	MGC9084	0.00	0.00	0.00	NaN
92483	LDHL	0.00	0.00	0.05	-0.34
92579	LOC92579	0.00	-0.13	0.00	0.21
92595	MGC13138	0.00	0.00	0.00	NaN
92609	LOC92609	0.09	0.00	0.00	-0.29
92815	MGC3165	0.09	0.00	0.00	0.34
93081	LOC93081	-0.09	-0.13	0.05	0.55
93210	MGC9753	0.00	1.00	0.00	0.87
93349	LOC93349	0.00	0.00	0.00	NaN
93408	LOC93408	0.18	0.00	0.00	0.18
93436	MGC19595	0.00	0.00	0.00	NaN
93643	TJP4	0.00	0.00	0.00	NaN
94009	SERHL	0.00	0.00	0.00	NaN
94025	MUC16	0.00	0.00	0.00	NaN
94104	C21orf66	0.00	0.00	0.00	NaN
94239	H2AV	0.00	0.00	0.00	NaN
96764	NCOA6IP	0.00	0.00	0.00	NaN
103910	MLC-B	0.09	0.00	0.00	0.34
112399	EGLN3	0.00	0.00	0.00	NaN
112869	LOC112869	0.00	0.00	0.00	NaN
112950	MGC17544	0.00	0.00	0.00	NaN
113146	LOC113146	0.00	0.00	0.00	NaN
113177	IMAGE:42153	0.00	0.00	0.00	NaN

113178	SCAMP-4	0.00	0.00	0.00	NaN
113251	LOC113251	0.00	0.00	0.00	NaN
113791	MGC17330	0.00	0.00	0.00	NaN
113878	DTX2	0.00	0.00	0.00	NaN
114049	WBSCR22	-0.09	0.00	0.00	0.23
114088	TRIM9	0.00	0.00	0.05	0.05
114548	CIAS1	0.00	0.13	0.00	0.32
114625	ERMAP	0.00	0.00	0.00	NaN
114785	KIAA1887	0.00	0.00	0.00	NaN
114791	KIAA1899	0.00	0.00	0.05	0.12
114819	KIAA1922	0.00	0.00	0.00	NaN
114876	OSBPL1A	0.00	0.00	-0.15	0.50
114881	OSBPL7	0.00	0.00	0.00	0.12
114882	OSBPL8	0.00	0.00	0.00	NaN
114883	OSBPL9	0.00	0.00	0.00	NaN
114884	OSBPL10	0.00	0.00	0.00	NaN
114885	OSBPL11	0.00	0.00	0.00	NaN
114897	C1QTNF1	0.00	0.25	0.00	-0.04
114899	C1QTNF3	0.00	0.00	0.10	-0.05
115207	LOC115207	-0.09	0.13	-0.10	0.37
115290	FBG4	0.00	0.13	0.00	0.06
116039	OSR2	0.09	0.13	0.10	0.53
116138	PEAS	0.00	0.00	0.00	NaN
116150	LOC116150	-0.09	0.00	0.05	0.56
116496	C1orf24	0.00	0.00	0.10	0.34
116832	RPL39L	0.09	0.00	0.05	0.25
116984	CENTD1	0.00	0.00	0.00	NaN
116985	CENTD2	0.00	0.00	0.00	NaN
116986	CENTG1	0.00	0.00	0.00	NaN
116987	CENTG2	0.00	0.00	-0.05	0.58
117246	FTSJ3	0.27	0.13	0.00	0.61
117247	SLC16A10	0.00	0.00	0.05	-0.13
118433	RPL23AP7	0.00	0.00	0.00	NaN
119016	MRIP2	0.18	0.25	0.20	-0.02
124583	LOC124583	0.00	0.25	0.00	0.28
131566	ESDN	0.00	0.00	0.00	NaN
131601	TPRA40	0.00	0.00	0.00	NaN
133619	LOC133619	0.00	0.00	0.00	NaN
140465	MLC1SA	0.00	0.00	0.00	NaN
140467	ZNF358	0.00	0.00	0.00	NaN
140545	RNF32	0.00	0.00	-0.05	0.06
140801	RPL10L	0.00	0.00	0.00	NaN
140803	TRPM6	0.00	0.00	-0.05	-0.09
140885	PTPNS1	0.00	0.00	0.05	0.15
150726	KIAA1940	0.00	0.00	0.00	NaN
154661	MGC26655	0.00	0.00	0.00	NaN
169522	Kv11.1	0.00	-0.13	-0.10	0.04

171017	TNRC1	-0.09	0.00	0.00	0.11
171392	TIZ	0.00	-0.38	0.00	0.48
171546	MGC24447	0.00	0.00	0.00	NaN
171558	PTCRA	0.00	0.00	0.00	NaN
171586	LABH3	0.00	0.00	-0.05	0.26
192286	MGC2198	0.00	0.00	0.00	NaN
192683	SCAMP5	0.00	0.00	0.00	NaN
