# Supplementary material for: A network-based, integrative study to identify core biological pathways that drive breast cancer clinical subtypes
Source: Br J Cancer. 2012 Feb 16;106(6):1107–16. doi: 10.1038/bjc.2011.584 (PMC3304402; doi:10.1038/bjc.2011.584)
Supplement: Supplementary Table S3 [file bjc2011584x6.pdf]

Chin et al. ER+

Information on driver network genes			
Gene Name	Median expression in ER+ group - Median gene expression in ER- group	Amplification and expression status (Amplified = 1, Differentially expressed = 2, Both amplified and differentially expressed = 12, Neither amplified nor differentially expressed = 0)	Frequency of appearance in the network from resampling (min = 0, max = 1, cutoff = 0.5)
AP1G1	2.09	2	1.00
AR	1.30	2	1.00
CCND1	0.43	1	1.00
BCL2	0.85	2	1.00
CCNG2	0.54	2	0.68
CLTC	0.07	1	1.00
DDX5	0.07	1	1.00
ERBB3	0.55	2	1.00
ESR1	2.44	2	1.00
FOXA1	2.10	2	1.00
IGF1R	0.96	2	1.00
IL6ST	0.65	2	0.83
IRS1	0.69	2	0.72
ITGB5	0.80	2	0.66
KRT18	0.70	2	1.00
MAPT	1.23	2	1.00
MYB	1.64	2	1.00
PHB	-0.13	1	1.00
PRKAR1A	0.35	1	0.89
PRKDC	-0.41	1	1.00
PSMC5	0.11	1	1.00
PTK2	0.12	1	1.00
RARA	0.46	2	0.98

Information on the driver network			
Source gene	Target gene	Source of interaction (HPRD = 1, KEGG = 2, TF = 3)	Differential expression correlation between genes in ER+ and ER- groups
AR	CCND1	1	0.04
AR	ESR1	1	-0.12
AR	FOXA1	1	-0.52
AR	IL6ST	1	-0.30
AR	SPDEF	1	-0.99
CCND1	ESR1	1	-0.30
BCL2	IRS1	1	0.64
BCL2	TMBIM6	1	0.17
BCL2	BECN1	1	1.19
BCL2	BCLAF1	1	1.30
BCL2	MOAP1	1	1.22
CLTC	AP1G1	1	0.12
DDX5	ESR1	1	-0.20
ERBB3	IL6ST	1	-0.73
ESR1	CCNG2	3	0.93
ESR1	IGF1R	1	-0.16
ESR1	RARA	1	-0.35
ESR1	SMARCA2	1	1.28
ESR1	TFF1	3	-0.54
ESR1	XBP1	1	0.11
ESR1	SLC9A3R1	3	-0.39
ESR1	CELSR1	1	-0.02
FOXA1	TFF1	3	-0.26

RET	0.66	2	0.76
RPS6KB1	-0.05	1	1.00
SLC22A5	0.76	2	0.64
SMARCA2	0.35	0	1.00
TMBIM6	0.33	2	0.81
TFF1	2.98	2	1.00
XBP1	0.95	2	1.00
XPA	0.27	2	0.80
YWHAZ	-0.36	1	1.00
SPOP	0.03	1	1.00
BECN1	0.30	0	1.00
GPAA1	-0.16	1	1.00
ASH2L	0.09	1	1.00
DEDD	0.04	1	1.00
SLC9A3R1	0.71	2	0.64
BAG4	0.03	1	1.00
CELSR1	1.09	2	1.00
BCLAF1	-0.04	0	1.00
VAV3	0.89	2	1.00
APPBP2	0.33	1	1.00
NPRL2	0.46	2	0.85
SPDEF	1.22	2	1.00
PIGT	0.38	2	0.94
PRR13	0.27	2	0.95
SSH3	0.42	2	0.90
EPN3	0.18	1	1.00
MOAP1	0.33	0	1.00

FOXA1	XBP1	1	-0.07
IGF1R	IRS1	1	0.05
IGF1R	VAV3	1	0.36
PHB	AR	1	-0.69
PRKAR1A	NPRL2	1	0.72
PRKDC	AR	1	0.78
PRKDC	XPA	1	-0.31
PRKDC	BECN1	1	-0.04
PSMC5	ESR1	1	-0.66
PTK2	ERBB3	1	0.21
PTK2	IGF1R	1	0.16
PTK2	IRS1	1	0.17
PTK2	ITGB5	1	0.14
PTK2	RET	1	0.33
RPS6KB1	MAPT	1	-0.37
SLC22A5	SLC9A3R1	1	-0.29
TFF1	ESR1	1	-0.54
TFF1	FOXA1	1	-0.26
YWHAZ	IGF1R	1	-0.12
YWHAZ	IRS1	1	-0.56
YWHAZ	KRT18	1	0.72
YWHAZ	MAPT	1	0.37
YWHAZ	SSH3	1	0.09
SPOP	AR	1	-0.35
GPAA1	PIGT	1	0.04
GPAA1	PRR13	1	0.24
ASH2L	MYB	1	-0.50
DEDD	KRT18	1	0.49
BAG4	BCL2	1	0.00
APPBP2	AR	1	-0.26
EPN3	ERBB3	2	0.20
EPN3	IGF1R	2	-0.06
EPN3	RET	2	-0.40

Chin et al. HER2+

Information on driver network genes			
Gene Name	Median expression in HER2+ group - Median gene expression in HER2- group	Amplification and expression status (Amplified = 1, Differentially expressed = 2, Both amplified and differentially expressed = 12, Neither amplified nor differentially expressed = 0)	Frequency of appearance in the network from resampling (min = 0, max = 1, cutoff = 0.5)
AANAT	0.05	2	0.65
AKT2	-0.16	2	0.53
CCND1	-0.34	1	1.00
CDH2	0.26	2	0.60
LYST	0.42	2	0.53
CSNK2A1	-0.37	0	0.70
ERBB2	1.90	12	1.00
FGFR4	1.07	2	1.00
FUS	-0.39	0	1.00
GRB2	0.12	1	1.00
GRB7	1.96	12	1.00
HIF1A	0.47	2	0.72
HSP90AA1	0.37	2	0.99
IFI27	0.76	2	0.70
KRT6B	1.59	2	0.51
KRT7	0.46	2	0.64
KRT15	0.82	2	0.51
KRT17	0.91	2	1.00
MAP3K3	-0.06	1	0.99
PDE3B	0.14	2	0.60
MED1	0.10	1	1.00
PRKAR1A	-0.38	1	0.89
MAPK11	0.35	2	0.59

Information on the driver network			
Source gene	Target gene	Source of interaction (HPRD = 1, KEGG = 2, TF = 3)	Differential expression correlation between genes in HER2+ and HER2- groups
AKT2	HSP90AA1	1	-0.67
AKT2	PDE3B	2	-0.65
CCND1	IFI27	1	-1.04
CCND1	THRA	1	0.39
CDH2	FGFR4	1	0.16
LYST	YWHAZ	1	-0.17
ERBB2	GRB2	2	0.30
ERBB2	GRB7	1	-0.90
ERBB2	HSP90AA1	1	0.41
GRB2	FGFR4	2	0.01
GRB2	GRB7	1	-1.07
GRB2	KRT7	1	0.55
GRB2	KRT17	1	0.36
GRB2	CCL5	1	-0.48
GRB2	TFAP2B	1	0.45
GRB2	TP63	1	0.43
GRB2	EPPK1	1	0.38
HIF1A	HSP90AA1	1	-0.69
KRT6B	KRT15	1	0.31
KRT7	KRT17	1	0.21
KRT15	PSMC5	1	0.26
MAP3K3	HSP90AA1	1	-0.75
MAP3K3	MAP2K3	2	0.62

MAP2K3	0.24	1	0.70
PSMC5	0.04	1	0.87
PSMD11	-0.15	1	0.82
PTH1R	0.24	2	0.63
PTN	0.54	2	0.71
CCL5	0.51	2	0.62
SLC9A3	0.24	2	0.58
TFAP2B	2.38	2	1.00
THRA	0.35	2	1.00
YWHAZ	-0.57	1	1.00
TP63	0.29	2	0.68
OASL	0.53	2	0.68
SLC9A3R1	-0.01	1	0.94
MED24	1.25	2	1.00
TOM1L1	-0.35	1	0.61
GLYAT	0.19	2	0.60
KIF1C	0.23	2	0.70
EPN3	-0.14	1	1.00
EPPK1	0.51	2	0.70

MAP3K3	YWHAZ	1	0.80
MED1	THRA	1	0.53
MED1	MED24	1	0.08
PRKAR1A	GRB2	1	0.80
PRKAR1A	PDE3B	2	0.97
MAP2K3	MAPK11	2	-1.01
PSMD11	PTN	1	-0.43
PTN	GLYAT	1	-0.94
THRA	FUS	1	1.33
THRA	OASL	1	-0.67
THRA	MED24	1	0.44
YWHAZ	AANAT	1	-0.89
YWHAZ	KIF1C	1	-0.46
SLC9A3R1	PTH1R	1	-0.13
SLC9A3R1	SLC9A3	1	0.39
TOM1L1	GRB2	1	0.48
KIF1C	CSNK2A1	1	1.37
EPN3	FGFR4	2	0.19

Chin et al. TN

Information on driver network genes			
Gene Name	Median expression in TNBC group - Median gene expression in non-TNBC group	Amplification and expression status (Amplified = 1, Differentially expressed = 2, Both amplified and differentially expressed = 12, Neither amplified nor differentially expressed = 0)	Frequency of appearance in the network from resampling (min = 0, max = 1, cutoff = 0.5)
ARNT	0.02	1	0.86
ATP1B3	0.41	2	0.76
KLF5	0.94	2	0.99
CDKN2A	0.79	2	0.58
CSF2RB	0.74	2	1.00
EGFR	1.17	2	1.00
ENO1	0.75	2	0.82
ESR1	-2.34	0	1.00
FANCA	0.39	2	0.54
FOLR1	1.05	2	0.92
GPM6B	1.13	2	1.00
GRB2	0.03	0	0.77
NR3C1	0.09	0	0.94
HSF1	0.11	1	0.94
HSPA1A	0.66	2	0.94
ICAM1	0.71	2	0.67
IGHM	0.75	2	0.95
IL7R	0.68	2	0.84
IMPA2	0.90	2	0.95
EIF3E	0.18	1	0.95
KRT17	1.60	2	1.00
LYN	0.79	2	1.00
MCM3	0.39	2	0.59

Information on the driver network			
Source gene	Target gene	Source of interaction (HPRD = 1, KEGG = 2, TF = 3)	Differential expression correlation between genes in TNBC and non-TNBC groups
ARNT	ENO1	3	0.96
CDKN2A	MCM5	1	0.83
CDKN2A	CDC7	1	-0.92
CSF2RB	LYN	1	0.22
CSF2RB	SYK	1	-0.06
CSF2RB	YWHAZ	1	-0.70
EGFR	ESR1	1	1.18
EGFR	GPM6B	1	-1.08
EGFR	HSPA1A	1	-0.98
EGFR	ICAM1	1	0.06
EGFR	KRT17	1	-0.99
EGFR	LYN	1	-0.95
EGFR	MET	1	0.19
EGFR	NCK1	2	-0.14
EGFR	PLCG2	2	-0.73
EGFR	PLD1	1	1.31
EGFR	TGFA	1	0.41
EGFR	UCHL1	1	-0.05
EGFR	YWHAZ	1	-0.04
ENO1	FANCA	1	0.74
ENO1	YWHAZ	1	0.05
FOLR1	LYN	1	-0.13
HSF1	HSPA1A	3	-0.04

MCM5	0.51	2	0.61
MET	0.68	2	0.62
MSN	0.51	2	0.91
NCK1	0.48	2	0.73
PLA2G4A	0.64	2	1.00
PLCB4	0.92	2	1.00
PLCG2	0.69	2	0.99
PLD1	0.14	0	1.00
PLEC	-0.10	1	1.00
POU2AF1	1.19	2	0.77
PRKDC	0.27	1	1.00
PTK2	0.08	1	1.00
PTPRC	0.73	2	0.56
S100A1	1.33	2	0.94
S100B	0.51	2	1.00
SEL1L	-0.09	0	0.95
SFRP1	1.94	2	1.00
SYK	0.44	2	0.77
TGFA	0.82	2	0.86
UCHL1	0.97	2	0.69
VCAM1	0.64	2	0.78
YES1	0.51	2	0.66
YWHAZ	0.39	1	1.00
CDC7	0.72	2	0.55
FZD6	0.45	1	1.00
BAG4	-0.04	1	0.94
NDRG1	0.90	2	0.94
IGF2BP3	0.29	2	1.00
IGF2BP2	0.74	2	0.65
WWP1	-0.74	1	1.00
PTP4A3	0.23	1	0.90
STK38	0.40	2	0.98
PUF60	0.18	1	1.00
WWTR1	0.50	2	0.94

HSF1	PRKDC	1	0.01
HSPA1A	NR3C1	1	1.21
HSPA1A	YWHAZ	1	-0.12
ICAM1	MSN	1	-0.31
ICAM1	PLCG2	2	0.23
IGHM	SEL1L	1	1.17
IL7R	LYN	1	0.01
EIF3E	NDRG1	1	0.38
KRT17	UCHL1	1	0.17
LYN	PLCG2	1	0.12
LYN	PRKDC	1	0.24
LYN	PTPRC	1	0.00
LYN	SYK	2	0.12
MCM3	MCM5	1	0.38
MCM3	CDC7	1	-0.04
MCM5	CDC7	1	-0.04
MSN	SYK	1	0.16
MSN	VCAM1	1	0.09
PLCB4	IMPA2	2	-0.11
PLCB4	PLCG2	2	-0.17
PLCB4	CALML5	2	-0.35
PLCG2	IMPA2	2	-0.23
PLCG2	PLA2G4A	2	-0.06
PLCG2	SYK	1	0.18
PLCG2	CALML5	2	-0.07
PLEC	EGFR	1	0.58
PLEC	MSN	1	-0.13
POU2AF1	SYK	1	0.18
PTK2	EGFR	1	0.16
PTK2	IGHM	1	0.75
PTK2	LYN	1	0.27
PTK2	NCK1	1	0.29
PTK2	SYK	1	-0.46
PTK2	YES1	1	0.23

PABPC1	0.11	1	0.95
USP21	-0.19	1	0.69
LSM1	0.14	1	0.67
CALML5	0.93	2	1.00

S100A1	S100B	1	0.30
S100B	NDRG1	1	0.04
S100B	STK38	1	-0.19
SYK	GRB2	1	1.15
SYK	PLD1	2	-0.56
YWHAZ	STK38	1	-0.35
YWHAZ	WWTR1	1	0.11
FZD6	PLCB4	2	-0.16
FZD6	SFRP1	1	0.57
BAG4	HSPA1A	1	-0.20
WWP1	KLF5	1	0.55
WWP1	EGFR	2	-0.31
WWP1	MET	2	-0.34
PTP4A3	ATP1B3	1	0.20
PTP4A3	MCM3	1	0.23
PUF60	IGF2BP3	1	0.18
PUF60	IGF2BP2	1	0.32
PABPC1	NDRG1	1	0.10
USP21	UCHL1	1	0.34
LSM1	PTPRC	1	-0.45

Andre et al. ER+

Information on driver network genes			
Gene Name	Median expression in ER+ group - Median gene expression in ER- group	Amplification and expression status (Amplified = 1, Differentially expressed = 2, Both amplified and differentially expressed = 12, Neither amplified nor differentially expressed = 0)	Frequency of appearance in the network from resampling (min = 0, max = 1, cutoff = 0.5)
AP1G1	1.38	2	1.00
AR	0.79	2	1.00
ARL3	0.44	2	0.80
CCND1	0.76	12	1.00
BCL2	0.56	2	0.58
BCL2L1	0.86	2	1.00
CLTC	0.10	1	1.00
DDX5	0.13	1	1.00
NQO1	0.69	2	0.96
ERBB3	0.57	2	0.98
ESR1	1.69	2	1.00
FGFR2	0.93	2	0.54
FGR	0.68	2	0.82
BLOC1S1	0.16	2	0.72
GFRA1	0.62	2	0.99
FOXA1	0.96	2	1.00
HSP90AA1	-0.07	0	1.00
IGF1R	0.78	2	1.00
IKBKB	0.78	2	0.86
IL6ST	0.72	2	1.00
INSM1	0.64	2	0.54
IRS1	0.56	2	0.99
ITGB5	0.58	2	0.70

Information on the driver network			
Source gene	Target gene	Source of interaction (HPRD = 1, KEGG = 2, TF = 3)	Differential expression correlation between genes in ER+ and ER- groups
AP1G1	RABEP1	1	-0.01
AR	CCND1	1	-0.27
AR	ESR1	1	-0.24
AR	FOXA1	1	-0.37
AR	IL6ST	1	-0.08
AR	PHB	1	-0.45
AR	TP53	1	0.43
AR	SPOP	1	0.08
AR	APPBP2	1	-0.13
AR	SPDEF	1	-0.29
ARL3	TP53	1	0.23
CCND1	ESR1	1	-0.02
CCND1	INSM1	1	-0.25
CCND1	NPDC1	1	0.12
BCL2	BCL2L1	1	0.67
BCL2	IRS1	1	0.14
BCL2	PPP2R5A	1	0.71
BCL2	TMBIM6	1	0.03
BCL2	TP53	2	-0.04
BCL2	BECN1	1	0.90
BCL2L1	IRS1	1	-0.12
BCL2L1	TMBIM6	1	-0.17
BCL2L1	TP53	1	0.28

KRT18	0.64	2	0.81
MAPT	1.21	2	1.00
MAP3K3	-0.01	1	0.86
SCGB2A2	1.77	2	0.82
MUC1	0.94	2	0.93
MYB	0.68	2	0.79
PGR	0.86	2	0.97
PHB	0.21	1	1.00
PPP2R5A	0.19	2	0.92
PRKAR1A	0.28	1	0.71
PRKDC	-0.17	1	1.00
PSMC5	0.06	1	1.00
PTK2	-0.09	1	1.00
RARA	0.50	2	0.76
RET	0.82	2	1.00
RPS6KB1	0.07	1	1.00
SMARCD3	0.39	2	0.54
TMBIM6	0.34	2	1.00
TFF1	2.09	2	1.00
TP53	1.04	2	0.82
UBC	0.15	0	1.00
XBP1	0.90	2	1.00
YWHAZ	-0.16	1	1.00
ZNF24	0.31	2	0.51
AKAP1	0.14	1	0.65
SPOP	0.25	12	1.00
BECN1	0.37	2	1.00
FADD	0.12	1	0.83
NOL3	0.90	2	0.68
ASH2L	0.14	1	0.79
RABEP1	0.44	2	1.00
DEDD	0.05	1	0.81
BAG4	0.13	1	0.58
CELSR1	0.57	2	0.98

BCL2L1	BECN1	1	0.18
CLTC	AP1G1	1	-0.30
DDX5	ESR1	1	0.67
DDX5	TP53	1	0.07
NQO1	TP53	1	0.03
ERBB3	IL6ST	1	0.22
ERBB3	MUC1	1	-0.12
ESR1	BCL2L1	3	-0.25
ESR1	NQO1	3	-0.68
ESR1	BLOC1S1	1	0.16
ESR1	HSP90AA1	1	1.10
ESR1	IGF1R	1	-0.05
ESR1	MUC1	3	-0.04
ESR1	PGR	3	0.16
ESR1	RARA	1	-0.19
ESR1	SMARCD3	1	0.61
ESR1	TFF1	3	-0.10
ESR1	TP53	3	0.56
ESR1	UBC	1	1.11
ESR1	XBP1	1	0.14
ESR1	CELSR1	1	0.04
ESR1	MED16	1	1.12
ESR1	HEXIM1	1	0.96
GFRA1	RET	1	-0.11
FOXA1	TFF1	3	-0.32
FOXA1	XBP1	1	0.07
IGF1R	IRS1	1	-0.31
IGF1R	VAV3	1	0.06
IKBKB	IRS1	2	0.13
MAPT	PPP2R5A	1	-0.11
MAP3K3	IKBKB	1	-0.38
SCGB2A2	SCGB1D2	1	0.12
MUC1	ESR1	1	-0.04
MUC1	TP53	1	0.06

KIAA0232	0.20	2	1.00
MED16	0.20	0	1.00
VAV3	0.57	2	1.00
APPBP2	0.35	1	1.00
HEXIM1	0.28	2	0.54
SCGB1D2	2.04	2	0.82
SPDEF	1.19	2	0.99
SSH3	0.75	2	0.86
EPN3	0.70	12	1.00
NPDC1	0.52	2	0.57
KCNK15	1.24	2	1.00

PGR	ESR1	1	0.16
PHB	TP53	1	-0.08
PRKDC	AR	1	0.41
PRKDC	IKBKB	1	0.06
PRKDC	TP53	2	-0.46
PRKDC	BECN1	1	-0.30
PSMC5	ESR1	1	0.98
PTK2	ERBB3	1	0.17
PTK2	FGR	1	0.04
PTK2	IGF1R	1	-0.12
PTK2	IRS1	1	-0.08
PTK2	ITGB5	1	-0.18
PTK2	RET	1	0.96
PTK2	TP53	1	-0.49
RPS6KB1	MAPT	1	0.01
TFF1	ESR1	1	-0.10
TFF1	FOXA1	1	-0.32
TP53	ESR1	1	0.56
TP53	ZNF24	1	-0.01
TP53	SCGB1D2	3	-0.02
YWHAZ	IGF1R	1	0.09
YWHAZ	IRS1	1	-0.08
YWHAZ	KRT18	1	-0.12
YWHAZ	MAPT	1	0.22
YWHAZ	TP53	1	-0.66
YWHAZ	KIAA0232	1	0.22
YWHAZ	SSH3	1	-0.67
YWHAZ	KCNK15	1	0.11
AKAP1	PRKAR1A	1	-0.06
FADD	NOL3	1	-0.15
ASH2L	MYB	1	-0.62
DEDD	KRT18	1	0.15
DEDD	FADD	1	-0.38
BAG4	BCL2	1	0.01

EPN3	ERBB3	2	-0.18
EPN3	FGFR2	2	0.07
EPN3	IGF1R	2	-0.04
EPN3	RET	2	-0.09

Andre et al. HER2+

Information on driver network genes			
Gene Name	Median expression in HER2+ group - Median gene expression in HER2- group	Amplification and expression status (Amplified = 1, Differentially expressed = 2, Both amplified and differentially expressed = 12, Neither amplified nor differentially expressed = 0)	Frequency of appearance in the network from resampling (min = 0, max = 1, cutoff = 0.5)
CDC6	-0.19	1	0.83
CLTC	0.33	1	0.99
MAPK14	0.03	0	0.72
ERBB2	1.52	12	1.00
FGFR4	0.64	2	0.53
GRK6	0.34	2	0.55
GRB2	0.14	1	1.00
GRB7	1.07	12	1.00
UBE2K	-0.13	0	0.75
HSPA1A	0.87	2	1.00
KRT7	0.71	2	0.71
SMAD2	-0.10	0	0.72
MAP3K10	0.19	2	0.94
NUMA1	0.70	2	0.56
ORC5L	0.69	2	0.79
PHB	-0.05	1	0.96
PML	0.69	2	0.72
MED1	0.50	12	0.99
RAD21	-0.05	1	0.59
RB1	0.20	0	0.72
TFAP2B	1.57	2	1.00
TFF1	0.52	2	0.51
UBE2I	-0.50	2	0.75

Information on the driver network			
Source gene	Target gene	Source of interaction (HPRD = 1, KEGG = 2, TF = 3)	Differential expression correlation between genes in HER2+ and HER2- groups
CDC6	ORC5L	2	0.13
CLTC	MAP3K10	1	0.20
CLTC	EHD1	1	0.02
ERBB2	GRB2	2	0.82
ERBB2	GRB7	1	0.36
GRK6	SLC9A3R1	1	0.15
GRB2	FGFR4	2	0.69
GRB2	GRB7	1	0.29
GRB2	KRT7	1	0.88
GRB2	TFAP2B	1	0.54
GRB2	BLNK	1	-0.42
HSPA1A	KRT7	1	-0.17
MAP3K10	PHB	1	-0.22
PML	MAPK14	1	1.31
PML	SMAD2	1	1.29
PML	RB1	1	1.29
PML	UBE2I	1	0.37
MED1	TFF1	1	0.15
MED1	MED24	1	-0.50
RAD21	NUMA1	1	-0.20
TFAP2B	UBE2I	1	-0.34
UBE2I	UBE2K	1	1.31
UBE2I	PRPF40A	1	1.31

YWHAZ	0.12	1	1.00
SLC9A3R1	0.13	1	0.82
MED24	0.34	2	0.85
EHD1	1.01	2	0.83
BLNK	0.26	2	0.66
EPN3	0.24	1	0.57
PRPF40A	0.04	0	0.75

YWHAZ	HSPA1A	1	-1.21
EPN3	FGFR4	2	0.35

Andre et al. TN

Information on driver network genes			
Gene Name	Median expression in TNBC group - Median gene expression in non-TNBC group	Amplification and expression status (Amplified = 1, Differentially expressed = 2, Both amplified and differentially expressed = 12, Neither amplified nor differentially expressed = 0)	Frequency of appearance in the network from resampling (min = 0, max = 1, cutoff = 0.5)
ANXA1	0.49	2	0.56
ARNT	0.17	1	0.88
COL9A3	1.68	2	0.99
CP	0.74	2	0.82
EGFR	0.79	2	1.00
EPB41L2	0.69	2	0.76
GPM6B	0.99	2	1.00
ICAM1	0.37	2	0.66
IGHM	0.55	2	0.73
EIF3E	0.16	1	0.99
KRT17	0.75	2	1.00
MET	0.80	2	0.99
MSN	0.54	2	0.59
PIK3CG	0.54	2	0.61
PLEC	-0.10	1	1.00
PTK2	0.19	1	1.00
S100A7	0.67	2	0.51
SFRP1	1.47	2	1.00
YWHAZ	0.14	1	1.00
FZD6	0.30	1	1.00
NDRG1	0.78	2	0.99
IGF2BP2	1.13	2	0.98
WWP1	-0.56	1	1.00

Information on the driver network			
Source gene	Target gene	Source of interaction (HPRD = 1, KEGG = 2, TF = 3)	Differential expression correlation between genes in TNBC and non-TNBC groups
ANXA1	EGFR	1	-0.69
ARNT	CP	3	0.47
COL9A3	EGFR	1	0.48
EGFR	GPM6B	1	-0.08
EGFR	ICAM1	1	-0.10
EGFR	KRT17	1	0.02
EGFR	MET	1	0.08
EGFR	PIK3CG	2	-0.03
EGFR	S100A7	1	-0.85
ICAM1	MSN	1	0.03
EIF3E	NDRG1	1	0.38
MET	PIK3CG	2	-0.02
PLEC	EGFR	1	-0.44
PLEC	MSN	1	0.19
PTK2	EGFR	1	-0.33
PTK2	IGHM	1	-0.09
PTK2	PIK3CG	2	0.45
YWHAZ	EGFR	1	-0.24
YWHAZ	EPB41L2	1	0.70
YWHAZ	WWTR1	1	-0.05
FZD6	SFRP1	1	0.55
NDRG1	PABPC1	1	0.24
WWP1	EGFR	2	-0.22

PUF60	0.16	1	0.99
WWTR1	1.12	2	1.00
PABPC1	0.14	1	0.99

WWP1	MET	2	-0.88
PUF60	IGF2BP2	1	0.13
PUF60	PABPC1	1	-0.02

Neve et al. ER+

Information on driver network genes			
Gene Name	Median expression in ER+ group - Median gene expression in ER- group	Amplification and expression status (Amplified = 1, Differentially expressed = 2, Both amplified and differentially expressed = 12, Neither amplified nor differentially expressed = 0)	Frequency of appearance in the network from resampling (min = 0, max = 1, cutoff = 0.5)
KLF5	-0.44114	0	1.00
CDH1	1.233511	2	1.00
CTSD	0.673943	2	0.80
CTTN	0.412132	12	1.00
ERBB3	0.841504	2	0.76
ESR1	1.262475	2	1.00
FOXO1	-0.127276	0	1.00
GNAI1	-0.290558	0	1.00
FOXA1	1.202347	2	1.00
ID2	0.94878	2	0.65
IGFBP4	0.798117	2	0.56
MUC1	1.255907	2	1.00
MYB	0.661227	2	0.78
NACA	0.283102	2	0.52
PAK1	-0.068565	1	1.00
PIK3R1	0.113519	0	1.00
PPP1CA	0.427083	1	1.00
PSMC5	0.271763	1	1.00
TFF1	3.94927	2	1.00
XBP1	1.436549	2	1.00
FADD	0.385548	1	0.64
ARHGEF7	0.181931	0	1.00
ASH2L	0.532299	12	0.98

Information on the driver network			
Source gene	Target gene	Source of interaction (HPRD = 1, KEGG = 2, TF = 3)	Differential expression correlation between genes in ER+ and ER- groups
CDH1	PPP1CA	1	-0.87
CTSD	IGFBP4	1	0.55
CTTN	ARPC3	1	0.32
CTTN	SHANK2	1	0.13
ERBB3	MUC1	1	0.08
ERBB3	PIK3R1	2	0.51
ESR1	KLF5	1	1.26
ESR1	CTSD	3	0.12
ESR1	FOXO1	1	1.37
ESR1	GNAI1	1	1.41
ESR1	MUC1	3	-0.77
ESR1	PAK1	1	1.12
ESR1	PIK3R1	1	1.27
ESR1	PSMC5	1	-0.98
ESR1	TFF1	3	-0.55
ESR1	XBP1	1	-1.08
ESR1	SLC9A3R1	3	-1.02
ESR1	MED13	1	-0.98
FOXA1	TFF1	3	-0.51
FOXA1	XBP1	1	-0.62
ID2	PPP1CA	1	-0.15
MUC1	ESR1	1	-0.77
MYB	ASH2L	1	0.47

SLC9A3R1	1.06108	2	1.00
MED13	0.564292	12	1.00
TOM1L1	0.541347	2	0.55
ARPC3	0.360683	2	0.74
LPHN1	0.049523	0	1.00
SHANK2	0.7572	2	1.00
MED13L	0.483418	2	0.93
CCDC47	0.481158	1	0.63

NACA	FADD	1	-0.83
PIK3R1	TOM1L1	1	-0.75
TFF1	ESR1	1	-0.55
TFF1	FOXA1	1	-0.51
MED13	MED13L	1	-0.09
TOM1L1	CCDC47	1	0.05
SHANK2	ARHGEF7	1	1.25
SHANK2	LPHN1	1	1.25

Neve et al. HER2+

Information on driver network genes			
Gene Name	Median expression in HER2+ group - Median gene expression in HER2- group	Amplification and expression status (Amplified = 1, Differentially expressed = 2, Both amplified and differentially expressed = 12, Neither amplified nor differentially expressed = 0)	Frequency of appearance in the network from resampling (min = 0, max = 1, cutoff = 0.5)
ALDH2	1.73	2	1.00
CDH1	1.29	2	0.87
CHGB	0.19	2	0.54
CLN3	0.79	2	1.00
CTSD	0.77	2	0.60
ERBB2	2.31	12	1.00
ERBB3	0.80	2	0.52
GRB7	1.56	12	1.00
FOXA1	1.03	2	0.90
MUC1	1.01	2	0.64
PHB	0.49	1	1.00
MED1	0.67	12	1.00
PRKCD	0.58	2	0.58
PTPRF	0.58	2	0.69
RARA	0.49	12	0.99
RPN2	0.72	2	0.73
S100A8	1.11	2	0.97
S100A9	1.28	2	0.99
SAT1	0.84	2	0.57
TFF1	1.60	2	1.00
XBP1	1.15	2	0.92
PCGF2	0.25	1	0.99
UNC119	0.18	1	1.00

Information on the driver network			
Source gene	Target gene	Source of interaction (HPRD = 1, KEGG = 2, TF = 3)	Differential expression correlation between genes in HER2+ and HER2- groups
ALDH2	UNC119	1	-0.50
CDH1	PTPRF	1	-1.02
CDH1	NDRG1	1	-0.05
CHGB	S100A8	1	0.17
CLN3	PHB	1	0.80
CLN3	RPN2	1	0.33
CTSD	MED1	1	0.51
ERBB2	ERBB3	1	-0.50
ERBB2	GRB7	1	-0.11
ERBB2	MUC1	1	-0.26
ERBB2	TOB1	1	-0.98
ERBB3	GRB7	1	-0.94
ERBB3	MUC1	1	-0.36
FOXA1	TFF1	3	-0.40
FOXA1	XBP1	1	-0.19
MUC1	PRKCD	1	0.06
MED1	RARA	1	0.19
MED1	TFF1	1	0.33
MED1	MED24	1	0.30
MED1	MED9	1	0.15
RPN2	NDRG1	1	0.15
S100A8	S100A9	1	0.07
S100A8	UNC119	1	0.63

MED24	0.14	2	0.63
TOB1	0.71	2	0.69
NDRG1	1.16	2	0.86
SPDEF	1.65	2	0.99
MED9	0.17	2	0.98

SAT1	UNC119	1	0.05
TFF1	FOXA1	1	-0.40
PCGF2	SPDEF	1	-0.22
MED24	MED9	1	0.26

Neve et al. TN

Information on driver network genes			
Gene Name	Median expression in TNBC group - Median gene expression in non-TNBC group	Amplification and expression status (Amplified = 1, Differentially expressed = 2, Both amplified and differentially expressed = 12, Neither amplified nor differentially expressed = 0)	Frequency of appearance in the network from resampling (min = 0, max = 1, cutoff = 0.5)
ANXA1	2.77	2	0.64
CAV1	2.84	2	0.64
CAV2	2.28	2	0.64
CD44	1.64	2	0.64
COL6A2	0.16	0	0.64
CTSK	-0.11	1	0.62
EGFR	1.40	2	0.64
EPHA2	0.90	2	0.62
EPS15	-0.05	0	0.64
ESR1	-1.10	0	0.64
FGFR1	-0.13	1	0.98
FN1	1.47	2	0.64
GTF2F2	0.25	2	0.99
TNC	0.82	2	0.53
IGFBP3	1.15	2	0.64
LYN	0.87	2	0.61
MET	1.14	2	0.64
MSN	2.68	2	0.64
PAK2	-0.20	0	0.61
PRNP	1.18	2	0.64
SOS2	-0.09	0	0.64
SPARC	1.31	2	0.62
TGFB1	0.94	2	0.64

Information on the driver network			
Source gene	Target gene	Source of interaction (HPRD = 1, KEGG = 2, TF = 3)	Differential expression correlation between genes in TNBC and non-TNBC groups
ANXA1	EGFR	1	-0.50
CAV1	CAV2	1	0.10
CAV1	EGFR	1	-0.87
CAV1	IGFBP3	1	-0.85
CAV1	PRNP	1	0.06
CAV2	EGFR	1	-0.28
CD44	EGFR	1	-0.15
CD44	FN1	1	0.47
CD44	IGFBP3	1	-0.45
CD44	MSN	1	-0.14
CD44	TGFBR2	1	-0.16
CTSK	SPARC	1	-0.15
EGFR	EPHA2	1	-0.39
EGFR	EPS15	1	1.19
EGFR	ESR1	1	1.38
EGFR	FN1	1	-1.14
EGFR	TNC	1	-0.07
EGFR	LYN	1	-1.03
EGFR	MET	1	-0.29
EGFR	SOS2	2	1.48
EGFR	UROD	1	0.03
EGFR	TUBA1A	1	-1.46
FGFR1	NRP1	1	1.28

TGFBI	1.55	2	0.64
TGFBR2	1.13	2	0.64
UROD	0.51	2	0.63
TUBA1A	1.49	2	0.55
C19orf2	0.18	1	0.99
NRP1	0.91	2	0.96
TRIP10	0.37	2	0.61
TUBB6	1.55	2	0.56

FN1	COL6A2	1	1.20
FN1	TNC	1	0.07
FN1	IGFBP3	1	-0.56
FN1	SPARC	1	0.58
FN1	TGFBI	1	0.07
GTF2F2	C19orf2	1	-0.49
LYN	PAK2	1	1.25
LYN	TRIP10	1	-0.21
SPARC	TGFB1	1	0.44
TGFB1	TGFBR2	2	0.96
TUBA1A	TUBB6	2	0.12
