# Supplementary material for: A network-based, integrative study to identify core biological pathways that drive breast cancer clinical subtypes
Source: Br J Cancer. 2012 Feb 16;106(6):1107–16. doi: 10.1038/bjc.2011.584 (PMC3304402; doi:10.1038/bjc.2011.584)
Supplement: Supplementary Table S4 [file bjc2011584x7.pdf]

**siRNA screen data for 10 "differential expression member" genes**

| Gene name | Entrez.Gene.Id | BT20 TNBC | BT20 TNBC | BT20 TNBC | BT549 TNBC | BT549 TNBC | BT549 TNBC | HBL100 TNBC | HBL100 TNBC | HBL100 TNBC |
|---|---|---|---|---|---|---|---|---|---|---|
| B3GNT9 | 84752 | -0.01 | 0.02 | 0.56 | -2.60 | -0.41 | -0.18 | 0.04 | 0.11 | 1.71 |
| B3GNT9 | 84752 | -0.23 | -1.28 | 1.88 | 0.28 | -1.58 | 0.52 | -1.00 | -0.79 | -1.41 |
| B3GNT9 | 84752 | 0.90 | -1.13 | -1.23 | -0.23 | 0.15 | 0.13 | 3.05 | 3.46 | 4.08 |
| B3GNT9 | 84752 | 0.10 | -0.39 | -0.25 | 1.21 | 0.87 | 0.54 | 0.25 | 2.45 | 1.91 |
| BTG3 | 10950 | -0.18 | 1.41 | 1.23 | 2.56 | 0.53 | 1.13 | 0.22 | -1.00 | 0.57 |
| BTG3 | 10950 | -0.56 | -0.15 | -0.70 | -2.24 | -2.57 | -2.13 | -4.51 | -4.22 | -3.87 |
| BTG3 | 10950 | 0.29 | 0.67 | -0.67 | 1.09 | -0.32 | 0.81 | 0.25 | 2.21 | 1.32 |
| BTG3 | 10950 | 1.09 | 1.65 | -0.10 | 1.00 | 0.02 | 0.79 | -0.20 | -0.62 | -0.51 |
| CHST3 | 9469 | -0.72 | -0.34 | 0.15 | -0.67 | -0.13 | 0.20 | 0.12 | 5.23 | 0.19 |
| CHST3 | 9469 | -1.90 | 0.33 | -2.24 | -0.95 | -0.34 | -1.20 | -1.74 | -2.60 | -2.06 |
| CHST3 | 9469 | 1.23 | 0.66 | 1.12 | 0.87 | 1.24 | 1.16 | -0.59 | -0.36 | 0.21 |
| CHST3 | 9469 | 0.38 | 1.13 | 0.40 | -0.44 | -0.47 | 1.74 | -0.17 | -0.60 | -0.57 |
| CSRP2 | 1466 | 0.97 | 4.14 | 1.16 | 0.70 | 1.21 | 2.18 | 3.83 | 6.37 | 3.44 |
| CSRP2 | 1466 | -0.24 | 0.14 | -0.94 | -2.16 | -0.44 | -0.67 | -1.23 | -2.01 | -2.53 |
| CSRP2 | 1466 | 1.70 | 0.83 | -0.03 | 0.43 | 1.22 | -0.80 | 1.18 | 0.81 | 0.37 |
| CSRP2 | 1466 | 0.65 | -0.43 | 0.41 | -0.19 | 0.40 | 1.42 | -0.15 | -0.69 | -0.27 |
| GSTP1 | 2950 | -2.23 | -1.24 | -0.18 | -0.64 | -0.54 | -2.40 | -0.22 | -1.35 | 0.13 |
| GSTP1 | 2950 | 0.08 | -1.44 | -1.31 | -0.58 | -1.00 | -0.07 | 0.54 | -0.80 | -1.45 |
| GSTP1 | 2950 | 0.22 | -0.70 | -0.86 | -0.26 | -0.55 | -0.37 | -0.59 | -0.44 | -1.72 |
| GSTP1 | 2950 | -0.62 | -0.90 | 0.56 | -0.62 | -0.35 | 0.58 | 0.42 | -0.03 | -0.05 |
| ID4 | 3400 | 0.25 | 0.08 | -0.24 | -0.05 | -0.52 | -0.11 | 0.83 | 0.09 | 0.08 |
| ID4 | 3400 | -0.28 | -1.64 | -2.59 | -1.23 | -0.91 | 0.38 | -1.88 | -1.49 | -2.35 |
| ID4 | 3400 | 0.42 | 0.11 | 0.58 | 0.30 | 1.02 | 0.16 | -0.19 | -0.28 | 1.40 |
| ID4 | 3400 | -2.01 | -3.65 | -0.45 | 0.08 | -0.07 | -0.47 | 3.69 | 3.30 | 2.96 |
| MCAM | 4162 | -0.38 | 0.46 | -0.32 | 1.11 | 0.06 | 0.48 | 3.93 | 1.57 | 1.22 |
| MCAM | 4162 | -0.76 | -1.16 | 1.88 | -1.90 | 0.06 | -0.45 | 1.96 | 1.87 | 0.77 |
| MCAM | 4162 | -0.16 | -0.41 | -0.50 | -2.24 | -0.62 | -0.62 | -0.65 | -2.00 | -1.62 |
| MCAM | 4162 | -1.18 | -0.39 | 0.61 | -0.60 | -0.60 | -1.75 | -3.62 | -3.50 | -2.64 |
| PSAT1 | 29968 | 0.03 | 1.42 | 0.30 | -0.19 | 0.31 | -0.16 | -0.28 | 0.78 | 1.25 |
| PSAT1 | 29968 | -1.97 | -1.47 | -1.56 | -1.57 | -0.66 | -1.16 | -1.24 | -0.66 | -0.34 |
| PSAT1 | 29968 | -0.77 | -0.41 | 0.55 | -0.93 | 1.45 | 0.69 | 1.06 | 1.46 | 4.91 |

| | | | | | | | | | | |
|---|---|---|---|---|---|---|---|---|---|---|
| PSAT1 | 29968 | 1.55 | 0.01 | 0.55 | 1.42 | -0.10 | 0.24 | 2.62 | 1.19 | 0.58 |
| TM4SF1 | 4071 | 1.13 | 0.90 | 1.60 | 0.29 | -0.73 | -1.96 | -0.28 | -1.05 | -0.96 |
| TM4SF1 | 4071 | 0.10 | 1.26 | 1.18 | 0.66 | -0.30 | -0.13 | -0.55 | -0.34 | -0.35 |
| TM4SF1 | 4071 | 0.45 | 0.08 | 1.04 | -0.57 | -0.95 | -0.29 | -0.64 | -1.43 | -1.47 |
| TM4SF1 | 4071 | -0.74 | 0.47 | -0.47 | 0.41 | 0.17 | 0.01 | -0.34 | -0.18 | 0.25 |
| ZIC1 | 7545 | 0.67 | 0.52 | 0.43 | -0.37 | -0.21 | -1.79 | 4.03 | 6.14 | 1.23 |
| ZIC1 | 7545 | -2.25 | -1.56 | -0.88 | 2.24 | 1.13 | 1.19 | 0.03 | 0.13 | -0.05 |
| ZIC1 | 7545 | -0.43 | -0.14 | 0.06 | 1.88 | 0.46 | 1.87 | 1.06 | 0.92 | 1.17 |
| ZIC1 | 7545 | -0.71 | -0.05 | -0.10 | -1.56 | -1.84 | -1.16 | -3.31 | -4.51 | -4.58 |

## siRNA screen data for 10 "differential expression member" genes

| Gene name | Entrez.Gene.Id | HS578T TNBC | HS578T TNBC | HS578T TNBC | MDAMB157 TNBC | MDAMB157 TNBC | MDAMB157 TNBC | MDAMB231 TNBC | MDAMB231 TNBC | MDAMB231 TNBC |
|---|---|---|---|---|---|---|---|---|---|---|
| B3GNT9 | 84752 | 1.40 | 3.38 | 2.59 | 3.24 | 3.93 | 2.90 | -0.29 | -0.28 | 0.21 |
| B3GNT9 | 84752 | 2.25 | 1.01 | 2.47 | -0.11 | -0.23 | 0.40 | -0.28 | 0.64 | -0.54 |
| B3GNT9 | 84752 | 0.69 | 0.90 | -0.20 | 0.09 | -0.06 | 2.82 | 0.19 | -1.05 | -0.29 |
| B3GNT9 | 84752 | -0.58 | 0.49 | 0.71 | -0.30 | 0.13 | 0.30 | -0.68 | -0.61 | 0.16 |
| BTG3 | 10950 | 1.43 | 1.00 | 2.28 | 2.45 | 2.77 | 2.73 | 0.27 | 0.30 | 0.23 |
| BTG3 | 10950 | -1.67 | -2.60 | -1.97 | -2.49 | -3.68 | -3.20 | 0.01 | -0.79 | -5.04 |
| BTG3 | 10950 | 2.72 | 3.22 | 1.81 | 1.41 | 1.67 | 4.60 | 0.16 | -0.27 | -0.48 |
| BTG3 | 10950 | 0.36 | 0.32 | 1.15 | 0.62 | 0.94 | 0.67 | -1.38 | -0.75 | -0.57 |
| CHST3 | 9469 | -1.11 | -0.20 | -0.18 | -1.98 | -1.83 | -1.96 | 1.65 | 0.15 | 1.26 |
| CHST3 | 9469 | -0.69 | -0.68 | -0.42 | -1.38 | -1.64 | -2.10 | -0.39 | -0.99 | -0.79 |
| CHST3 | 9469 | -0.02 | 0.64 | 0.55 | 0.90 | 0.54 | 2.98 | 0.83 | 0.48 | -0.17 |
| CHST3 | 9469 | -0.09 | -0.27 | -0.55 | -2.09 | -0.32 | 1.35 | 0.15 | -0.40 | 0.37 |
| CSRP2 | 1466 | 0.93 | 1.07 | 0.31 | 5.06 | 3.55 | 3.03 | 0.73 | 0.13 | 0.50 |
| CSRP2 | 1466 | -1.23 | -0.88 | -0.63 | -4.68 | -5.67 | -5.56 | -1.32 | -1.83 | -0.95 |
| CSRP2 | 1466 | 0.56 | -0.16 | 0.73 | 5.01 | 3.49 | 1.36 | 0.88 | 1.41 | 1.09 |
| CSRP2 | 1466 | -0.49 | -0.09 | 0.49 | 0.97 | 2.48 | 1.15 | 0.12 | -0.92 | 1.78 |
| GSTP1 | 2950 | 0.60 | 1.22 | 0.51 | -2.13 | -3.10 | -3.21 | 0.40 | 0.14 | -0.81 |
| GSTP1 | 2950 | -0.21 | -0.58 | 1.61 | -0.28 | 0.35 | 0.15 | 0.11 | -0.13 | 0.05 |
| GSTP1 | 2950 | 1.11 | 0.44 | 0.99 | 0.71 | 0.45 | 0.65 | 0.42 | -2.51 | -0.03 |
| GSTP1 | 2950 | 0.48 | 0.20 | -0.17 | 0.81 | 2.18 | 1.15 | -0.74 | -3.05 | -0.53 |
| ID4 | 3400 | 0.50 | 0.60 | 0.29 | 3.16 | 1.22 | 2.57 | -0.69 | -1.07 | -0.15 |
| ID4 | 3400 | -0.85 | -0.65 | -0.39 | 0.18 | 0.63 | 0.37 | -1.32 | -1.10 | -2.75 |
| ID4 | 3400 | -0.70 | -0.08 | -1.21 | -1.59 | -1.83 | -2.12 | 0.18 | -0.13 | 0.70 |
| ID4 | 3400 | -0.41 | 0.19 | 0.06 | -3.34 | -3.51 | -2.55 | -0.06 | -0.85 | -0.50 |
| MCAM | 4162 | -0.46 | -1.28 | -0.25 | -1.45 | -3.08 | -2.35 | -0.43 | -1.08 | -0.72 |
| MCAM | 4162 | -0.43 | 0.22 | 0.14 | 1.07 | 1.37 | 0.66 | -0.22 | -1.36 | -0.56 |
| MCAM | 4162 | -2.24 | -3.33 | -2.24 | -0.45 | -0.14 | -0.07 | -0.03 | -0.19 | -0.51 |
| MCAM | 4162 | 0.22 | -0.80 | -0.64 | -2.85 | -1.96 | -2.58 | -1.99 | -2.44 | 0.28 |
| PSAT1 | 29968 | -0.21 | 1.18 | 0.01 | 0.67 | 0.11 | 0.07 | 1.24 | 0.92 | -0.40 |
| PSAT1 | 29968 | -0.46 | -0.10 | 0.25 | -1.95 | -1.69 | -1.70 | -1.21 | -1.29 | -1.23 |
| PSAT1 | 29968 | 0.15 | 1.53 | 0.61 | 0.65 | -0.04 | 0.58 | 1.56 | -0.12 | 0.68 |

| | | | | | | | | | | |
|---|---|---|---|---|---|---|---|---|---|---|
| PSAT1 | 29968 | 0.25 | 0.15 | -0.19 | 0.91 | 0.63 | 0.84 | 0.26 | 0.00 | -0.49 |
| TM4SF1 | 4071 | -0.43 | -0.35 | -0.39 | 2.03 | 1.93 | 2.24 | -0.81 | -0.28 | -0.48 |
| TM4SF1 | 4071 | -0.18 | 0.04 | 0.59 | 0.31 | 0.55 | 1.64 | 0.14 | 0.35 | -0.16 |
| TM4SF1 | 4071 | -0.81 | -1.26 | -1.08 | -5.50 | -8.01 | -4.11 | -0.62 | -1.26 | -0.61 |
| TM4SF1 | 4071 | 0.08 | -0.25 | -1.17 | -0.70 | -0.85 | -0.61 | -0.15 | -0.42 | -1.25 |
| ZIC1 | 7545 | -0.27 | 0.51 | 0.00 | 1.30 | -0.04 | 0.00 | 0.30 | -0.68 | 0.30 |
| ZIC1 | 7545 | 0.62 | -0.39 | -1.27 | -1.09 | -2.09 | -0.39 | -0.68 | 1.24 | -0.90 |
| ZIC1 | 7545 | 0.94 | -0.41 | 2.94 | 0.93 | 0.20 | 0.29 | -0.24 | -0.89 | 0.04 |
| ZIC1 | 7545 | -0.98 | -2.82 | -1.34 | -2.87 | -4.67 | -2.12 | 0.04 | -1.28 | -0.61 |

## siRNA screen data for 10 "differential expression member" genes

| Gene name | Entrez.Gene.Id | MDAMB436 TNBC | MDAMB436 TNBC | MDAMB436 TNBC | MDAMB453 TNBC | MDAMB453 TNBC | MDAMB453 TNBC | MDAMB468 TNBC | MDAMB468 TNBC | MDAMB468 TNBC |
|---|---|---|---|---|---|---|---|---|---|---|
| B3GNT9 | 84752 | -0.89 | 2.30 | -1.65 | -0.99 | -0.53 | -1.42 | 1.47 | 2.40 | 2.51 |
| B3GNT9 | 84752 | 1.03 | 1.53 | 0.78 | -0.81 | -0.20 | -0.48 | -0.32 | -2.89 | 0.04 |
| B3GNT9 | 84752 | 0.16 | -0.74 | 0.35 | -1.33 | -0.44 | 0.04 | -4.15 | -4.22 | -4.75 |
| B3GNT9 | 84752 | 1.34 | -0.06 | 0.09 | -1.33 | 1.47 | 0.55 | 0.44 | -0.71 | -0.83 |
| BTG3 | 10950 | 0.82 | 1.57 | 1.03 | -0.02 | 0.67 | 1.15 | 0.14 | 0.16 | -0.50 |
| BTG3 | 10950 | 0.87 | -0.36 | -1.94 | -1.07 | 0.81 | -0.12 | -8.61 | -11.99 | -6.43 |
| BTG3 | 10950 | 0.36 | -0.64 | -0.43 | -1.82 | -0.54 | 0.99 | 0.33 | 0.06 | 0.59 |
| BTG3 | 10950 | -0.95 | 1.19 | 0.10 | -1.39 | 1.23 | -0.88 | 0.36 | -2.40 | -1.59 |
| CHST3 | 9469 | -0.78 | -2.63 | -4.56 | -0.71 | 0.41 | -1.00 | -0.83 | -0.80 | -0.74 |
| CHST3 | 9469 | -1.47 | 0.78 | 0.00 | -1.64 | -1.71 | -2.38 | -0.57 | 0.51 | -0.51 |
| CHST3 | 9469 | -0.09 | 0.78 | 1.19 | 1.10 | 0.76 | 0.71 | -0.18 | -0.36 | -0.26 |
| CHST3 | 9469 | -1.07 | 0.29 | -0.63 | -1.69 | -1.15 | -0.56 | 0.80 | -0.78 | -0.04 |
| CSRP2 | 1466 | 0.60 | 1.19 | -0.12 | 0.50 | 1.44 | -0.03 | 1.04 | 0.63 | 1.11 |
| CSRP2 | 1466 | -0.23 | -2.01 | -1.93 | 0.08 | -0.62 | 1.67 | -3.62 | -3.54 | -6.67 |
| CSRP2 | 1466 | 0.04 | 2.07 | 0.47 | 2.12 | 0.29 | 0.40 | -0.21 | 0.25 | 0.46 |
| CSRP2 | 1466 | -0.49 | 1.38 | -2.22 | -0.83 | 2.53 | 0.49 | -0.11 | -0.11 | 0.89 |
| GSTP1 | 2950 | -0.34 | -1.70 | -1.98 | 0.22 | -0.22 | -0.39 | -0.08 | -0.70 | -2.38 |
| GSTP1 | 2950 | -0.45 | -0.24 | -0.22 | 0.22 | -1.37 | 1.67 | -0.72 | -0.49 | -0.22 |
| GSTP1 | 2950 | 1.09 | -0.02 | -0.26 | -1.56 | -0.29 | -0.21 | -0.27 | -0.15 | 1.34 |
| GSTP1 | 2950 | -0.89 | -0.42 | -0.33 | 0.99 | -0.13 | 0.15 | 1.28 | 2.27 | 3.05 |
| ID4 | 3400 | 1.27 | 0.07 | 0.92 | -1.19 | -0.10 | -0.43 | 0.33 | -0.27 | -0.49 |
| ID4 | 3400 | -0.22 | -1.49 | -3.13 | -0.19 | -1.40 | -2.80 | -0.61 | -0.87 | -2.78 |
| ID4 | 3400 | -0.64 | -0.57 | -0.81 | 0.19 | 0.78 | 0.79 | -0.53 | 0.04 | 4.36 |
| ID4 | 3400 | -1.14 | 0.91 | 1.21 | -0.37 | -1.19 | -0.32 | -4.77 | -4.70 | -5.97 |
| MCAM | 4162 | 0.25 | -0.63 | -3.00 | -0.49 | 0.37 | 1.19 | 0.44 | -1.45 | -0.40 |
| MCAM | 4162 | -0.12 | 0.27 | -0.23 | -1.41 | -0.15 | -0.76 | -0.55 | -0.87 | -0.42 |
| MCAM | 4162 | -0.40 | -0.83 | -0.45 | -1.42 | -1.98 | -2.27 | -0.71 | -1.60 | 2.50 |
| MCAM | 4162 | 0.86 | 0.41 | 0.09 | -0.81 | -0.41 | -0.79 | 1.34 | 1.20 | 0.74 |
| PSAT1 | 29968 | -1.33 | -2.07 | -1.19 | -0.50 | -0.02 | -1.45 | -1.11 | -0.70 | -1.68 |
| PSAT1 | 29968 | 0.12 | -1.57 | -3.14 | 0.27 | -0.81 | -2.44 | -3.71 | -3.14 | -3.05 |
| PSAT1 | 29968 | -1.20 | -0.38 | -0.29 | -0.31 | -0.33 | 0.38 | 0.74 | 1.01 | 1.78 |

| | | | | | | | | | | |
|---|---|---|---|---|---|---|---|---|---|---|
| PSAT1 | 29968 | 0.53 | 1.07 | 2.58 | -0.07 | 0.56 | 1.77 | 0.21 | 1.00 | 0.18 |
| TM4SF1 | 4071 | 1.06 | 1.57 | 2.54 | -0.64 | -1.51 | -2.00 | -0.20 | 0.65 | -0.95 |
| TM4SF1 | 4071 | 0.64 | -0.13 | 1.25 | -1.69 | 0.20 | -0.31 | 2.03 | 0.94 | 0.48 |
| TM4SF1 | 4071 | -0.17 | 0.06 | 0.53 | 0.03 | 1.20 | 0.87 | -2.85 | -4.28 | -3.09 |
| TM4SF1 | 4071 | -0.03 | 0.33 | 0.51 | -0.38 | -0.40 | 0.86 | 0.69 | 0.17 | -0.96 |
| ZIC1 | 7545 | 0.32 | 1.29 | 1.50 | -0.49 | 2.04 | -0.11 | -0.16 | 0.82 | 1.03 |
| ZIC1 | 7545 | -1.16 | -1.20 | -0.97 | -0.71 | -2.43 | 0.16 | -0.82 | -2.74 | -0.43 |
| ZIC1 | 7545 | -0.44 | -1.69 | -0.55 | 0.62 | 0.05 | -1.66 | 0.36 | 0.01 | 0.02 |
| ZIC1 | 7545 | -0.31 | -1.39 | -1.44 | -0.43 | -1.21 | 0.01 | -1.89 | -2.74 | -4.18 |

## siRNA screen data for 10 "differential expression member" genes

| Gene name | Entrez.Gene.Id | HER2_AU565 HER2+ | HER2_AU565 HER2+ | HER2_AU565 HER2+ | ER_BT483 ER+ | ER_BT483 ER+ | ER_BT483 ER+ | HER2_SKBr3 HER2+ | HER2_SKBr3 HER2+ | HER2_SKBr3 HER2+ |
|---|---|---|---|---|---|---|---|---|---|---|
| B3GNT9 | 84752 | -0.20 | -0.66 | -0.06 | 0.42 | -0.06 | 0.27 | 0.30 | 0.22 | 0.94 |
| B3GNT9 | 84752 | 1.58 | -0.03 | 1.16 | 0.19 | -0.36 | -0.11 | -0.58 | 0.84 | -0.31 |
| B3GNT9 | 84752 | 0.03 | -2.68 | -1.61 | -1.85 | -0.20 | -0.06 | 0.41 | 0.84 | 0.40 |
| B3GNT9 | 84752 | -1.40 | -0.49 | 0.24 | 0.95 | 1.14 | 1.00 | -0.60 | -0.22 | -1.85 |
| BTG3 | 10950 | 1.62 | 3.17 | 2.67 | 0.85 | 1.79 | 1.54 | 2.95 | 0.82 | 1.38 |
| BTG3 | 10950 | -2.61 | -3.25 | -3.20 | -0.20 | -1.41 | -1.12 | -2.37 | -0.96 | -1.67 |
| BTG3 | 10950 | 1.33 | -0.28 | 0.10 | 0.59 | 0.10 | 0.30 | 0.22 | 2.70 | 1.13 |
| BTG3 | 10950 | 0.29 | 0.65 | 0.81 | -3.73 | 0.10 | -1.48 | -3.17 | 0.67 | 0.79 |
| CHST3 | 9469 | 0.08 | 1.04 | -1.03 | -1.26 | -2.41 | -1.01 | -0.02 | 0.09 | 1.02 |
| CHST3 | 9469 | -2.70 | -1.57 | -2.31 | 0.23 | -1.19 | -0.13 | 0.60 | 0.41 | -0.38 |
| CHST3 | 9469 | 0.52 | 3.32 | 1.54 | 0.68 | 0.10 | 0.00 | -0.22 | 0.96 | 0.15 |
| CHST3 | 9469 | -0.79 | 0.54 | -0.07 | 1.04 | 0.25 | -1.72 | 0.03 | 0.34 | 0.58 |
| CSRP2 | 1466 | 4.55 | 3.76 | 1.56 | 0.88 | -0.29 | -0.63 | -0.72 | 0.30 | 1.02 |
| CSRP2 | 1466 | -3.29 | -2.12 | -1.82 | -0.25 | 1.19 | 0.13 | -1.32 | -0.80 | -1.21 |
| CSRP2 | 1466 | 1.30 | 2.13 | 1.71 | 0.17 | -0.03 | 0.33 | 2.97 | 0.74 | 0.38 |
| CSRP2 | 1466 | -1.95 | -0.21 | -1.33 | -0.23 | -0.27 | -0.80 | -0.40 | 0.07 | 0.22 |
| GSTP1 | 2950 | -0.05 | 0.52 | -0.44 | 0.49 | 0.39 | 0.66 | 0.31 | -0.03 | 0.00 |
| GSTP1 | 2950 | -1.93 | -0.14 | 0.48 | -0.83 | -1.81 | -0.19 | 0.04 | -0.12 | -0.91 |
| GSTP1 | 2950 | 0.49 | -0.34 | 0.22 | -0.06 | -0.74 | -5.42 | -0.29 | 0.36 | 0.47 |
| GSTP1 | 2950 | 0.16 | -0.08 | 1.00 | 0.02 | -0.27 | 1.06 | 2.07 | -0.01 | 1.40 |
| ID4 | 3400 | -2.37 | -1.94 | -0.52 | 0.23 | -0.29 | -0.23 | -0.38 | -1.07 | 1.48 |
| ID4 | 3400 | -2.03 | -1.63 | -2.52 | -1.76 | -1.13 | -2.50 | -3.13 | -1.42 | -1.47 |
| ID4 | 3400 | -2.56 | -1.17 | -2.23 | -0.87 | 0.78 | -0.27 | -0.90 | -1.08 | -0.82 |
| ID4 | 3400 | 1.74 | -1.77 | 0.93 | -1.61 | -1.44 | -1.51 | 0.82 | 0.30 | -0.24 |
| MCAM | 4162 | -0.13 | 0.67 | -0.19 | 0.47 | 1.07 | 0.60 | -0.01 | 0.23 | 0.52 |
| MCAM | 4162 | -0.42 | 0.63 | 0.68 | 0.32 | 0.98 | 0.23 | 1.14 | 1.66 | 1.75 |
| MCAM | 4162 | -1.86 | -1.08 | -2.16 | -0.70 | -0.56 | -0.18 | -0.75 | -0.20 | -1.51 |
| MCAM | 4162 | 0.19 | -0.20 | 0.65 | 0.31 | 1.62 | 0.94 | -0.62 | 0.30 | 0.88 |
| PSAT1 | 29968 | -1.27 | -2.34 | -1.73 | -1.66 | -3.99 | -0.15 | -3.04 | -0.94 | -2.43 |
| PSAT1 | 29968 | -0.06 | -0.16 | -1.59 | -1.41 | -3.11 | -1.06 | 3.28 | 0.76 | 2.09 |
| PSAT1 | 29968 | -1.54 | -1.69 | -0.50 | -0.25 | 0.48 | -0.30 | 0.30 | -0.52 | 0.14 |

| | | | | | | | | | | |
|---|---|---|---|---|---|---|---|---|---|---|
| PSAT1 | 29968 | 0.06 | 0.28 | 2.58 | -0.48 | 0.21 | 0.22 | -0.07 | -0.83 | 0.82 |
| TM4SF1 | 4071 | -1.63 | -1.11 | -0.96 | -1.08 | -0.29 | -0.36 | 0.44 | 0.03 | 0.09 |
| TM4SF1 | 4071 | 1.12 | 1.02 | 1.28 | 0.55 | -0.04 | 0.21 | 2.65 | 1.19 | 1.79 |
| TM4SF1 | 4071 | 0.61 | -0.82 | 1.07 | -2.57 | -1.71 | -1.17 | -0.30 | 0.35 | 0.32 |
| TM4SF1 | 4071 | -0.61 | -0.03 | -0.51 | 0.28 | 0.38 | 0.19 | -0.45 | 0.07 | -0.90 |
| ZIC1 | 7545 | 0.75 | 1.38 | 0.09 | 1.24 | 0.66 | 0.27 | 0.90 | 1.98 | 2.21 |
| ZIC1 | 7545 | -0.05 | -0.50 | -1.20 | -1.47 | -3.15 | -1.59 | 0.50 | 1.11 | -0.16 |
| ZIC1 | 7545 | 1.77 | -0.52 | 0.61 | 0.32 | 0.29 | 1.03 | 1.23 | 0.22 | -0.29 |
| ZIC1 | 7545 | -2.65 | -3.09 | -0.41 | 0.26 | 0.92 | 0.40 | 0.44 | -0.40 | -0.56 |

## siRNA screen data for 10 "differential expression member" genes

| Gene name | Entrez.Gene.Id | ER_ZR751<br>ER+ | ER_ZR751<br>ER+ | ER_ZR751<br>ER+ |
|---|---|---|---|---|
| B3GNT9 | 84752 | 0.29 | 0.35 | 0.49 |
| B3GNT9 | 84752 | 0.58 | -0.51 | 0.56 |
| B3GNT9 | 84752 | 1.21 | 1.48 | 1.51 |
| B3GNT9 | 84752 | 2.47 | 0.18 | 1.36 |
| BTG3 | 10950 | 1.58 | -1.12 | -0.10 |
| BTG3 | 10950 | 0.37 | -0.33 | -1.09 |
| BTG3 | 10950 | 1.70 | 0.55 | 1.61 |
| BTG3 | 10950 | -1.02 | -0.18 | 0.10 |
| CHST3 | 9469 | 0.22 | 2.16 | 0.01 |
| CHST3 | 9469 | 0.87 | 0.44 | -1.20 |
| CHST3 | 9469 | 0.07 | 1.35 | 0.09 |
| CHST3 | 9469 | -0.03 | -1.64 | 2.38 |
| CSRP2 | 1466 | 2.19 | 1.12 | 1.26 |
| CSRP2 | 1466 | -2.74 | -2.25 | -0.44 |
| CSRP2 | 1466 | 1.74 | -0.44 | -0.09 |
| CSRP2 | 1466 | 0.12 | -1.44 | -0.23 |
| GSTP1 | 2950 | 0.84 | 1.43 | 0.33 |
| GSTP1 | 2950 | 1.20 | 0.06 | 0.14 |
| GSTP1 | 2950 | 1.41 | 0.16 | 1.17 |
| GSTP1 | 2950 | -1.18 | -0.46 | -1.21 |
| ID4 | 3400 | 1.58 | 0.81 | -0.28 |
| ID4 | 3400 | -2.67 | -0.67 | -1.44 |
| ID4 | 3400 | -0.08 | -1.97 | 1.60 |
| ID4 | 3400 | -2.91 | 0.50 | 1.78 |
| MCAM | 4162 | 1.08 | 0.92 | 1.29 |
| MCAM | 4162 | 0.50 | 1.20 | 0.09 |
| MCAM | 4162 | -0.16 | -0.05 | -1.76 |
| MCAM | 4162 | 0.86 | -2.29 | 1.09 |
| PSAT1 | 29968 | 0.42 | 1.30 | 0.28 |
| PSAT1 | 29968 | -0.70 | -1.19 | 0.18 |
| PSAT1 | 29968 | 2.11 | 1.42 | 0.44 |

| | | | | |
|---|---|---|---|---|
| PSAT1 | 29968 | 0.66 | -0.64 | 0.82 |
| TM4SF1 | 4071 | -1.42 | 0.13 | -1.35 |
| TM4SF1 | 4071 | 0.62 | 0.78 | 1.30 |
| TM4SF1 | 4071 | 0.93 | 1.18 | 2.56 |
| TM4SF1 | 4071 | -0.50 | 0.32 | 0.00 |
| ZIC1 | 7545 | 0.10 | 0.31 | -0.37 |
| ZIC1 | 7545 | -0.21 | 1.03 | 0.68 |
| ZIC1 | 7545 | 0.67 | -1.02 | 0.22 |
| ZIC1 | 7545 | 1.23 | 0.35 | -0.51 |

## siRNA screen data for 40 "driver network member" genes from driver networks

| Gene name | Entrez.Gene.Id | BT20 TNBC | BT20 TNBC | BT20 TNBC | BT549 TNBC | BT549 TNBC | BT549 TNBC | HBL100 TNBC | HBL100 TNBC | HBL100 TNBC |
|---|---|---|---|---|---|---|---|---|---|---|
| ANXA1 | 301 | 0.00 | -0.15 | -0.05 | 0.20 | 0.05 | -1.31 | -0.77 | -0.16 | -1.02 |
| ANXA1 | 301 | 0.59 | -0.32 | 0.28 | 0.96 | 0.64 | 0.52 | 1.42 | 2.12 | 0.59 |
| ANXA1 | 301 | -1.42 | -1.85 | -1.12 | -2.45 | 0.00 | -1.18 | 0.09 | 0.17 | -0.21 |
| ANXA1 | 301 | 0.36 | -0.42 | 0.22 | -1.12 | -1.15 | -1.65 | -0.20 | -0.37 | -0.94 |
| ATP1B3 | 483 | -0.58 | 0.34 | -0.37 | -0.95 | 0.06 | 0.40 | -1.86 | -1.78 | -0.85 |
| ATP1B3 | 483 | 1.38 | 0.79 | 1.60 | 1.01 | 0.07 | -0.06 | -1.52 | -2.95 | -2.08 |
| ATP1B3 | 483 | -1.06 | -0.73 | -0.98 | -0.91 | -1.15 | -0.50 | -1.31 | -1.99 | -3.23 |
| ATP1B3 | 483 | -0.16 | -0.52 | 0.70 | 0.50 | 0.34 | -0.50 | 0.21 | 1.74 | 1.55 |
| CAV2 | 858 | -1.06 | -0.07 | -0.47 | -0.32 | -0.02 | -1.48 | -1.35 | -1.44 | -0.76 |
| CAV2 | 858 | -0.31 | -0.76 | -0.59 | 0.33 | -0.09 | -1.10 | 0.10 | -0.19 | -0.44 |
| CAV2 | 858 | 0.12 | -0.23 | 1.28 | 0.88 | 0.47 | 1.26 | -0.06 | -0.32 | -0.30 |
| CAV2 | 858 | 1.30 | 1.40 | -0.54 | 1.08 | 1.10 | 0.31 | 1.75 | 2.56 | 2.72 |
| CD44 | 960 | 1.15 | -0.25 | 0.65 | -0.02 | 1.61 | 0.21 | 3.66 | 2.76 | 1.67 |
| CD44 | 960 | -0.42 | -0.16 | -1.20 | -0.55 | -0.06 | -0.12 | -0.73 | -0.69 | -0.38 |
| CD44 | 960 | -0.13 | 0.31 | -0.04 | -0.80 | -0.49 | -2.07 | 0.19 | -0.32 | -0.52 |
| CD44 | 960 | 0.22 | 0.32 | 0.54 | -1.00 | 0.56 | 1.28 | 0.92 | -0.23 | -0.23 |
| CDKN2A | 1029 | 0.17 | 1.31 | 1.05 | -0.04 | -0.51 | -0.33 | -0.56 | -0.86 | 0.32 |
| CDKN2A | 1029 | 1.75 | 1.51 | -1.94 | 0.07 | 0.06 | -0.30 | -1.20 | -0.41 | -0.34 |
| CDKN2A | 1029 | -2.08 | -0.21 | -1.24 | 0.71 | 0.11 | -0.34 | 0.38 | 1.10 | 0.05 |
| CDKN2A | 1029 | -0.24 | 0.03 | 0.20 | -0.12 | 0.40 | 0.59 | -0.60 | -0.24 | -0.47 |
| ENO1 | 2023 | -1.23 | 0.68 | -0.93 | 0.23 | 0.01 | -0.23 | 0.64 | 0.37 | -0.33 |
| ENO1 | 2023 | -0.24 | 0.09 | 0.33 | -1.49 | 0.37 | -0.53 | -0.64 | -0.24 | -0.43 |
| ENO1 | 2023 | -0.50 | -2.16 | 1.17 | 0.48 | 1.05 | -2.33 | 0.17 | 0.55 | -0.33 |
| ENO1 | 2023 | 0.60 | 0.26 | -0.66 | 0.26 | 0.51 | 1.59 | 1.28 | 1.39 | 0.78 |
| EPB41L2 | 2037 | 1.48 | 0.04 | 2.01 | 0.45 | 0.41 | 0.15 | -0.11 | -1.15 | -1.17 |
| EPB41L2 | 2037 | 2.00 | 0.52 | -0.26 | -0.02 | -0.65 | -0.19 | -0.02 | -0.07 | -0.44 |
| EPB41L2 | 2037 | -1.15 | 0.00 | -0.13 | -2.07 | -0.33 | -1.99 | -1.91 | -3.25 | -3.96 |
| EPB41L2 | 2037 | 0.36 | 1.23 | 1.48 | -0.06 | 0.63 | 0.44 | -0.08 | 0.68 | 0.03 |
| FOLR1 | 2348 | 1.06 | 1.39 | 1.90 | 0.04 | 0.45 | 0.19 | -0.05 | 0.16 | 0.36 |
| FOLR1 | 2348 | 0.01 | -0.73 | 0.57 | 0.17 | 0.05 | 0.08 | -2.35 | -2.53 | -2.01 |
| FOLR1 | 2348 | 1.07 | 0.79 | 1.71 | 0.19 | 0.88 | 0.78 | 2.72 | 0.94 | 1.59 |

| | | | | | | | | | | |
|---|---|---|---|---|---|---|---|---|---|---|
| FOLR1 | 2348 | 0.28 | -0.47 | -0.42 | -0.59 | 0.50 | 1.02 | -2.31 | -2.57 | -0.51 |
| ICAM1 | 3383 | 0.31 | 0.22 | 0.05 | -0.37 | 0.06 | -0.31 | -0.44 | 0.71 | -0.06 |
| ICAM1 | 3383 | 0.36 | -1.03 | 0.30 | 0.35 | -0.43 | -1.27 | -0.99 | -1.49 | -1.66 |
| ICAM1 | 3383 | -1.18 | -1.90 | 0.23 | 0.04 | 0.54 | 0.61 | -0.54 | -0.37 | -0.40 |
| ICAM1 | 3383 | 1.31 | 0.08 | 2.30 | 0.68 | 0.80 | 1.48 | 0.76 | 0.56 | 0.82 |
| IGF2BP2 | 10644 | 1.54 | -3.25 | 0.27 | 0.11 | 0.07 | 0.02 | 0.16 | 0.71 | 0.44 |
| IGF2BP2 | 10644 | 0.04 | 0.65 | 0.88 | 1.01 | 0.05 | -0.03 | 3.38 | 4.47 | 0.94 |
| IGF2BP2 | 10644 | -1.00 | -1.17 | -1.00 | -0.35 | -0.14 | -0.10 | -0.60 | -1.14 | -1.10 |
| IGF2BP2 | 10644 | -0.50 | -0.13 | -0.16 | -1.09 | -0.05 | -0.05 | -0.68 | 0.15 | -1.30 |
| IGF2BP3 | 10643 | -0.55 | -0.32 | -0.05 | -1.46 | 0.24 | 0.10 | -0.48 | -0.57 | 0.27 |
| IGF2BP3 | 10643 | -0.19 | -0.69 | 0.42 | 1.20 | -0.31 | 1.08 | 0.17 | -0.13 | -1.15 |
| IGF2BP3 | 10643 | -0.82 | -0.12 | -0.68 | -2.05 | 0.40 | -0.87 | 3.32 | 0.40 | 0.01 |
| IGF2BP3 | 10643 | 0.49 | -1.14 | 1.28 | -0.36 | 1.01 | -0.33 | 4.34 | 3.04 | 0.82 |
| IMPA2 | 3613 | 0.55 | -4.08 | -0.81 | -0.42 | 0.36 | 1.29 | 0.78 | 2.24 | 1.08 |
| IMPA2 | 3613 | 0.05 | 0.11 | 0.46 | -0.38 | -0.75 | 0.35 | 0.29 | -0.46 | 0.06 |
| IMPA2 | 3613 | 0.24 | -3.20 | -1.44 | 0.26 | 0.42 | 0.09 | -0.28 | -0.23 | -0.38 |
| IMPA2 | 3613 | -1.19 | -2.31 | -1.86 | -0.42 | -0.22 | 0.08 | -0.21 | -0.65 | -0.57 |
| KLF5 | 688 | -2.49 | 0.80 | 0.88 | 0.77 | 1.31 | 1.68 | -0.49 | 0.45 | 0.41 |
| KLF5 | 688 | -1.49 | -2.31 | 1.58 | 2.07 | -0.96 | 1.08 | -0.19 | -0.36 | -1.61 |
| KLF5 | 688 | -0.49 | -0.69 | -0.37 | -0.41 | 0.66 | -1.79 | -1.55 | -2.23 | -2.11 |
| KLF5 | 688 | -0.11 | 1.07 | 1.57 | -0.11 | 0.36 | -0.15 | 0.18 | 0.75 | -0.04 |
| LYN | 4067 | -0.30 | 1.95 | 2.50 | 0.89 | 0.85 | 0.34 | 0.09 | 1.08 | -0.25 |
| LYN | 4067 | -0.20 | -1.34 | -0.32 | -0.85 | -0.08 | -0.94 | -2.84 | -2.83 | -2.36 |
| LYN | 4067 | 0.57 | 1.71 | 0.40 | 0.37 | -0.09 | 0.73 | -1.88 | -1.82 | -1.11 |
| LYN | 4067 | 0.00 | -0.41 | 1.01 | 0.80 | 1.09 | 1.68 | 0.43 | 0.14 | 0.51 |
| MCM3 | 4172 | -2.99 | -1.03 | -1.33 | -0.38 | -1.01 | -0.18 | -3.25 | -2.57 | -1.95 |
| MCM3 | 4172 | -0.25 | -0.31 | -1.15 | -0.22 | 0.03 | -1.09 | -0.48 | -0.71 | -0.75 |
| MCM3 | 4172 | -3.06 | -2.40 | -2.28 | -5.19 | -2.39 | -1.90 | -7.11 | -5.54 | -4.42 |
| MCM3 | 4172 | 2.11 | 1.12 | 1.49 | 1.23 | -0.06 | 1.63 | 1.58 | 0.29 | 0.52 |
| MCM5 | 4174 | 1.12 | -1.30 | -0.43 | -0.38 | 0.22 | 1.57 | -0.28 | -0.14 | -0.66 |
| MCM5 | 4174 | -0.07 | 0.14 | 0.34 | 0.00 | -0.02 | 0.11 | 4.04 | 2.12 | 2.22 |
| MCM5 | 4174 | 0.48 | 1.14 | 1.16 | 2.71 | 0.03 | 1.69 | 0.02 | 0.38 | 0.04 |
| MCM5 | 4174 | -1.88 | -2.58 | -0.19 | -0.33 | -0.28 | 0.04 | 0.04 | -0.65 | -0.69 |
| MET | 4233 | 0.82 | 0.34 | 0.84 | -0.43 | 1.35 | 1.70 | 0.47 | 6.39 | 0.80 |

| | | | | | | | | | | |
|---|---|---|---|---|---|---|---|---|---|---|
| MET | 4233 | -0.43 | -1.03 | -2.19 | 0.04 | -0.88 | -0.57 | -0.86 | -0.37 | -0.74 |
| MET | 4233 | -1.08 | -2.10 | -0.36 | -3.66 | -1.60 | -2.57 | -5.50 | -6.83 | -5.55 |
| MET | 4233 | -0.58 | -1.54 | -1.43 | -0.09 | 1.31 | 0.58 | 0.86 | 2.60 | 0.53 |
| MSN | 4478 | 0.78 | 0.90 | -0.01 | -0.15 | -0.47 | -0.67 | 2.25 | 1.26 | 2.89 |
| MSN | 4478 | 0.21 | -0.86 | 1.45 | 0.96 | -0.28 | -0.48 | 0.38 | 0.17 | 0.57 |
| MSN | 4478 | -0.39 | -0.21 | 0.28 | 0.59 | 1.24 | -1.34 | 0.30 | 0.29 | 0.04 |
| MSN | 4478 | -1.52 | -1.05 | -0.96 | -0.79 | -0.67 | -0.44 | -0.79 | -1.85 | -2.99 |
| NCK1 | 4690 | -1.44 | 0.16 | 0.48 | -0.12 | 0.77 | 0.26 | -0.09 | 0.28 | 1.17 |
| NCK1 | 4690 | 0.62 | -0.49 | 0.40 | 0.00 | 0.22 | -0.35 | 0.16 | -0.27 | -0.96 |
| NCK1 | 4690 | 2.07 | 2.53 | 1.96 | 0.60 | 0.24 | 0.05 | -0.11 | 2.21 | 0.07 |
| NCK1 | 4690 | 0.19 | -1.87 | 0.18 | -1.42 | -0.53 | -0.64 | 0.28 | -0.47 | -0.87 |
| NDRG1 | 10397 | -0.59 | 0.20 | 0.00 | 0.19 | -0.35 | -0.10 | -0.70 | -1.41 | -0.87 |
| NDRG1 | 10397 | -0.36 | -0.30 | -0.59 | 0.04 | 0.25 | -0.05 | 0.04 | -0.05 | 0.03 |
| NDRG1 | 10397 | 0.05 | 0.67 | -0.38 | -0.11 | 0.03 | 0.95 | -1.07 | 0.22 | 0.64 |
| NDRG1 | 10397 | 0.40 | 0.80 | 0.91 | 2.83 | 0.72 | 1.01 | -0.57 | 0.48 | -0.13 |
| PLCG2 | 5336 | 0.93 | 0.32 | 1.35 | -0.65 | -1.18 | -1.58 | -1.48 | -4.61 | -2.51 |
| PLCG2 | 5336 | -0.03 | 1.30 | 0.27 | 2.75 | 0.13 | -0.32 | 3.69 | 4.11 | 2.09 |
| PLCG2 | 5336 | 1.11 | -0.22 | -0.03 | -0.20 | -0.39 | 1.02 | 1.19 | 1.09 | 0.49 |
| PLCG2 | 5336 | 1.84 | 1.82 | 0.90 | -0.31 | -0.66 | 0.06 | -0.39 | -1.58 | -1.05 |
| PRKDC | 5591 | -1.47 | -0.20 | -0.22 | -1.43 | -1.27 | -1.69 | -0.14 | -0.75 | -0.48 |
| PRKDC | 5591 | -2.47 | 0.15 | 0.30 | 0.22 | -0.26 | 0.08 | -0.11 | 0.00 | 0.88 |
| PRKDC | 5591 | 0.76 | -0.20 | 0.43 | 1.49 | 0.72 | 0.44 | 0.75 | 1.40 | 0.23 |
| PRKDC | 5591 | 1.74 | 2.54 | 1.59 | -0.09 | 0.31 | -0.57 | 0.96 | -0.15 | -0.68 |
| PRNP | 5621 | -0.54 | 0.43 | 0.27 | 0.23 | -0.81 | -1.23 | -1.39 | -1.60 | -1.48 |
| PRNP | 5621 | -2.33 | -1.59 | -2.86 | -0.48 | -1.26 | -0.93 | 0.23 | -1.47 | -0.89 |
| PRNP | 5621 | 0.75 | -1.39 | 1.28 | 0.36 | 0.87 | 0.19 | -0.02 | 2.23 | 0.49 |
| PRNP | 5621 | 1.00 | 0.62 | 1.12 | 0.13 | 0.23 | 0.68 | 0.89 | 1.55 | 1.77 |
| S100A1 | 6271 | 2.27 | 1.00 | 1.54 | 0.45 | -0.10 | 0.33 | 0.93 | 0.21 | 0.05 |
| S100A1 | 6271 | -0.12 | 0.54 | 1.43 | 0.41 | -1.02 | 0.60 | 0.44 | 0.10 | -0.43 |
| S100A1 | 6271 | 0.11 | 0.46 | 0.63 | -0.10 | 0.22 | -0.37 | -1.06 | -1.98 | -0.32 |
| S100A1 | 6271 | -0.03 | 0.10 | -0.30 | 0.26 | 0.48 | 0.37 | -0.33 | -0.83 | -0.81 |
| S100B | 6285 | -1.05 | -0.81 | 0.13 | -0.52 | -1.00 | -0.63 | -2.13 | -2.06 | -2.81 |
| S100B | 6285 | -0.11 | -0.91 | -0.61 | 0.76 | 0.81 | 2.20 | 0.35 | 0.63 | 0.91 |
| S100B | 6285 | 1.56 | 0.19 | -0.12 | 0.37 | 0.78 | 1.29 | 1.21 | 1.45 | 1.88 |

| | | | | | | | | | | |
|---|---|---|---|---|---|---|---|---|---|---|
| S100B | 6285 | 1.11 | 0.05 | 0.87 | 1.09 | 0.75 | -0.56 | 0.19 | 1.33 | -0.65 |
| STK38 | 11329 | 2.07 | 3.02 | 3.05 | 0.40 | -0.58 | 0.00 | 0.98 | 2.36 | 0.59 |
| STK38 | 11329 | -1.28 | -0.28 | -1.03 | -1.73 | -1.27 | -0.87 | -0.61 | -1.22 | -1.80 |
| STK38 | 11329 | 0.51 | -0.54 | 2.13 | 0.25 | 0.40 | 2.41 | -0.02 | 0.07 | -0.50 |
| STK38 | 11329 | 0.31 | -0.08 | 0.46 | -0.48 | -0.81 | 1.35 | 0.70 | 1.49 | 0.70 |
| TGFBI | 7045 | -1.34 | -0.90 | -2.06 | -0.84 | -0.26 | -3.00 | -0.66 | -0.58 | 0.18 |
| TGFBI | 7045 | -0.66 | 1.01 | 0.08 | -0.19 | -0.15 | -1.78 | -0.98 | -0.14 | -0.57 |
| TGFBI | 7045 | 1.18 | -0.85 | 2.79 | 0.05 | 0.98 | -1.09 | 3.09 | 3.63 | 2.71 |
| TGFBI | 7045 | -0.17 | 1.84 | 0.51 | 1.86 | 1.67 | 1.24 | 1.88 | 0.32 | 0.04 |
| UCHL1 | 7345 | 0.12 | 0.13 | 0.60 | 1.50 | -0.12 | -0.42 | -0.28 | -0.36 | -0.90 |
| UCHL1 | 7345 | -1.96 | -3.27 | -2.30 | -3.19 | -1.44 | -2.36 | -3.24 | -4.56 | -3.93 |
| UCHL1 | 7345 | 0.47 | 0.91 | 2.10 | -0.14 | 0.34 | 0.02 | -0.35 | -1.10 | -0.78 |
| UCHL1 | 7345 | 0.28 | -0.06 | -1.00 | -0.98 | -1.31 | -1.07 | -1.33 | -2.22 | -2.91 |
| VCAM1 | 7412 | 2.68 | 0.22 | 1.74 | -0.91 | 0.07 | -2.67 | -0.01 | -1.00 | 0.02 |
| VCAM1 | 7412 | 0.39 | 0.72 | 1.25 | -1.95 | -0.44 | -0.28 | 0.24 | 1.08 | 0.69 |
| VCAM1 | 7412 | 0.46 | 1.29 | 1.42 | 1.20 | 0.22 | 0.65 | 0.47 | 0.21 | 1.41 |
| VCAM1 | 7412 | 0.68 | 0.84 | 2.53 | -0.06 | -0.31 | 0.36 | 0.60 | 0.02 | 0.77 |
| WWTR1 | 25937 | 0.21 | 1.54 | 0.98 | -0.19 | -0.40 | -0.39 | -3.50 | -2.99 | -2.42 |
| WWTR1 | 25937 | 0.06 | -0.47 | 0.09 | -1.10 | -0.78 | -1.25 | -1.40 | 0.06 | -0.89 |
| WWTR1 | 25937 | 0.29 | -2.52 | 0.52 | 0.04 | -0.28 | -0.02 | 0.60 | 0.09 | 0.83 |
| WWTR1 | 25937 | -0.31 | -0.05 | -1.48 | -0.38 | -0.88 | 0.08 | -1.02 | -1.35 | -3.05 |

## siRNA screen data for 40 "driver network member" genes from driver networks

| Gene name | Entrez.Gene.Id | HS578T TNBC | HS578T TNBC | HS578T TNBC | MDAMB157 TNBC | MDAMB157 TNBC | MDAMB157 TNBC | MDAMB231 TNBC | MDAMB231 TNBC | MDAMB231 TNBC |
|---|---|---|---|---|---|---|---|---|---|---|
| ANXA1 | 301 | -0.77 | -0.05 | 0.06 | 2.77 | 1.93 | 2.34 | -0.23 | 0.33 | -0.51 |
| ANXA1 | 301 | -0.54 | -1.25 | 0.04 | 0.85 | 0.59 | 0.61 | 0.06 | 0.11 | -3.22 |
| ANXA1 | 301 | -0.96 | -2.17 | -1.12 | -0.79 | 15.70 | -0.84 | 0.13 | -1.51 | -1.07 |
| ANXA1 | 301 | 0.92 | -0.74 | 0.53 | 1.68 | 1.73 | 1.04 | -0.95 | -3.28 | -0.83 |
| ATP1B3 | 483 | -0.53 | -1.03 | -0.51 | -1.47 | -0.46 | -1.49 | 0.20 | -0.26 | 0.24 |
| ATP1B3 | 483 | -0.96 | 0.24 | -1.78 | -1.45 | -1.82 | -1.52 | 0.21 | 1.00 | -0.59 |
| ATP1B3 | 483 | -0.77 | -2.50 | -1.68 | -2.27 | -3.93 | -2.92 | -0.67 | -1.44 | -0.87 |
| ATP1B3 | 483 | 1.30 | 0.88 | 0.47 | 0.45 | 1.26 | 1.51 | -0.72 | 1.13 | -1.06 |
| CAV2 | 858 | 0.05 | 0.98 | 0.64 | -0.03 | 0.03 | -0.49 | 0.28 | -0.15 | -0.99 |
| CAV2 | 858 | -0.45 | -0.10 | 1.09 | -0.01 | 0.20 | -0.13 | -0.37 | -0.55 | -0.59 |
| CAV2 | 858 | 0.26 | 2.55 | 0.71 | 1.20 | 1.81 | 2.60 | 0.49 | 1.58 | 0.11 |
| CAV2 | 858 | 1.20 | -0.17 | 2.00 | 5.56 | 4.82 | 2.09 | -0.03 | -0.16 | 1.45 |
| CD44 | 960 | 0.17 | 0.36 | 0.24 | 1.86 | 2.63 | 1.34 | 0.95 | 1.26 | 2.84 |
| CD44 | 960 | -0.46 | 0.30 | 0.14 | -0.27 | -0.58 | -0.49 | 0.61 | 0.54 | -1.06 |
| CD44 | 960 | -3.28 | -2.80 | -2.44 | -1.43 | -1.30 | -2.10 | -1.23 | -1.49 | -1.98 |
| CD44 | 960 | 0.24 | 0.64 | -0.43 | 5.68 | 0.93 | 0.27 | -0.11 | 0.13 | 0.28 |
| CDKN2A | 1029 | -0.42 | 1.18 | 2.80 | 2.31 | 2.78 | 4.26 | 0.41 | 0.60 | 0.13 |
| CDKN2A | 1029 | -0.50 | -1.08 | -0.42 | -0.19 | 0.10 | -0.84 | -0.92 | 0.71 | 1.76 |
| CDKN2A | 1029 | -0.39 | 1.42 | 0.74 | 1.75 | 2.27 | 1.82 | -0.02 | 0.22 | -0.29 |
| CDKN2A | 1029 | -0.66 | -0.11 | -0.23 | 0.15 | 0.26 | 0.87 | -0.84 | -2.53 | -1.86 |
| ENO1 | 2023 | -0.40 | 0.26 | 1.50 | 1.68 | 0.68 | 0.57 | 0.87 | 2.13 | -0.34 |
| ENO1 | 2023 | -0.76 | -1.26 | -0.43 | 0.25 | 0.69 | -0.32 | -1.64 | -1.18 | -2.91 |
| ENO1 | 2023 | 1.38 | 2.67 | 0.98 | 0.63 | 1.00 | 1.96 | 0.69 | 0.00 | -0.96 |
| ENO1 | 2023 | 0.63 | 0.31 | 2.12 | 1.25 | 0.29 | 1.11 | -0.25 | 0.21 | -0.26 |
| EPB41L2 | 2037 | 1.32 | -0.45 | -0.62 | 1.51 | 0.50 | 0.83 | 0.14 | 1.43 | -2.72 |
| EPB41L2 | 2037 | -0.66 | -1.34 | 0.00 | 4.53 | 1.83 | 1.07 | 0.55 | -0.07 | -2.01 |
| EPB41L2 | 2037 | -3.09 | -1.42 | -2.86 | -2.63 | -2.85 | -3.70 | -0.83 | -1.28 | -0.52 |
| EPB41L2 | 2037 | 2.08 | 0.81 | 2.02 | 0.65 | 0.46 | 0.40 | 0.13 | 0.79 | 1.53 |
| FOLR1 | 2348 | 0.27 | -0.02 | 0.43 | 3.39 | 0.55 | 2.69 | -0.47 | -0.17 | 0.71 |
| FOLR1 | 2348 | -0.09 | 0.32 | 0.32 | 0.17 | -0.82 | 0.04 | -1.43 | 0.14 | -0.59 |
| FOLR1 | 2348 | 0.18 | -1.09 | -1.11 | 1.99 | 1.68 | 1.94 | 0.10 | 0.69 | 1.11 |

| | | | | | | | | | | |
|---|---|---|---|---|---|---|---|---|---|---|
| FOLR1 | 2348 | -1.36 | -1.58 | -0.40 | 2.33 | 2.85 | 0.37 | 0.30 | -0.37 | -0.10 |
| ICAM1 | 3383 | -0.93 | -0.91 | 0.01 | -0.29 | 0.06 | 0.01 | -1.68 | 0.20 | -0.15 |
| ICAM1 | 3383 | -0.63 | 1.08 | -0.12 | 2.46 | 2.81 | 3.72 | 0.26 | 1.00 | 0.43 |
| ICAM1 | 3383 | 0.33 | 1.17 | 1.91 | -0.54 | -2.28 | 1.32 | 0.16 | -1.03 | 0.00 |
| ICAM1 | 3383 | 0.88 | 1.65 | 0.80 | 3.86 | 5.31 | 1.34 | -0.75 | 3.15 | 1.21 |
| IGF2BP2 | 10644 | 0.05 | 1.57 | 0.19 | 2.21 | 2.31 | 1.13 | 0.13 | -0.46 | -1.30 |
| IGF2BP2 | 10644 | 0.38 | -1.41 | -0.14 | 3.13 | 2.66 | 1.78 | -0.69 | -0.28 | -1.18 |
| IGF2BP2 | 10644 | -0.60 | -0.50 | -0.26 | -0.57 | -1.65 | -1.12 | -0.90 | -0.46 | -0.65 |
| IGF2BP2 | 10644 | 8.70 | -1.34 | -0.69 | -2.84 | -1.93 | -1.49 | -1.62 | -0.16 | -0.82 |
| IGF2BP3 | 10643 | -0.96 | -1.35 | -0.23 | 0.94 | -0.31 | -0.12 | -0.79 | -0.42 | -2.87 |
| IGF2BP3 | 10643 | 0.63 | -0.09 | 0.18 | -0.97 | -1.57 | -0.72 | 0.18 | -0.06 | -1.12 |
| IGF2BP3 | 10643 | 1.47 | 0.08 | -0.30 | 0.62 | 1.05 | 0.46 | -0.96 | 1.05 | -0.12 |
| IGF2BP3 | 10643 | 0.23 | 0.01 | 1.36 | 4.95 | 2.40 | 2.02 | -0.12 | -0.01 | -1.17 |
| IMPA2 | 3613 | 1.22 | 0.80 | 0.02 | -0.97 | 1.11 | 0.41 | 0.05 | 0.97 | -0.05 |
| IMPA2 | 3613 | 1.13 | 0.88 | 0.93 | -2.12 | -2.52 | -0.72 | -0.45 | -2.79 | -0.90 |
| IMPA2 | 3613 | -0.74 | 0.28 | -0.35 | -0.52 | -0.94 | -1.59 | -0.99 | -2.23 | -0.91 |
| IMPA2 | 3613 | 0.60 | 0.48 | -0.07 | -0.75 | 0.39 | 0.59 | -0.84 | -0.28 | 0.64 |
| KLF5 | 688 | -0.44 | -0.27 | -0.28 | 0.15 | 0.11 | 0.20 | -0.57 | 0.57 | -1.80 |
| KLF5 | 688 | 1.02 | 2.78 | 1.62 | 0.55 | 0.50 | 0.55 | 0.21 | -0.72 | -1.35 |
| KLF5 | 688 | -0.29 | -1.07 | -1.89 | -5.26 | -4.55 | -3.30 | 0.03 | 0.21 | -1.22 |
| KLF5 | 688 | 0.28 | -0.63 | 1.55 | 0.90 | 2.62 | 1.20 | 0.52 | 1.60 | -2.19 |
| LYN | 4067 | 2.05 | 4.46 | 2.76 | 4.43 | 1.33 | 2.08 | 0.79 | -0.23 | -1.76 |
| LYN | 4067 | -1.08 | -1.54 | -0.97 | -0.98 | -0.60 | -1.87 | 0.50 | -0.83 | 1.39 |
| LYN | 4067 | -0.16 | 0.40 | 0.97 | 0.29 | -0.18 | -1.15 | 1.15 | -0.36 | 0.87 |
| LYN | 4067 | 0.49 | 3.86 | 3.55 | 4.92 | 3.83 | 3.35 | 0.13 | 3.74 | 2.32 |
| MCM3 | 4172 | -0.66 | 0.09 | 0.04 | 1.11 | 2.09 | 1.53 | -0.14 | 0.10 | -1.26 |
| MCM3 | 4172 | -1.01 | -0.69 | -0.14 | -0.70 | -1.21 | -2.07 | -0.30 | -0.26 | -1.43 |
| MCM3 | 4172 | -2.49 | -2.30 | -1.89 | -5.62 | -6.86 | -5.54 | -1.26 | -2.21 | -4.48 |
| MCM3 | 4172 | 4.21 | 1.23 | 4.44 | 5.47 | 7.04 | 2.05 | 1.62 | 3.90 | 1.57 |
| MCM5 | 4174 | -0.48 | 0.37 | -1.57 | 0.54 | 0.81 | 0.80 | 0.27 | 0.37 | -0.32 |
| MCM5 | 4174 | -0.33 | 1.57 | 0.22 | 1.52 | 2.64 | 0.93 | -0.46 | 0.23 | -1.30 |
| MCM5 | 4174 | 1.63 | -0.11 | 1.96 | 0.27 | 1.63 | 0.64 | -0.43 | -0.35 | -0.54 |
| MCM5 | 4174 | -0.51 | -0.04 | 1.09 | 0.30 | 1.40 | 0.76 | -1.41 | -1.37 | -0.71 |
| MET | 4233 | -0.14 | 24.44 | 0.07 | -2.49 | -1.75 | -1.87 | -0.07 | -0.76 | -2.29 |

| | | | | | | | | | | |
|---|---|---|---|---|---|---|---|---|---|---|
| MET | 4233 | 0.02 | 1.15 | 0.09 | -0.64 | 1.32 | 0.47 | -0.31 | -1.06 | -2.44 |
| MET | 4233 | -2.07 | -2.45 | -3.48 | -8.19 | -6.11 | -5.86 | -0.56 | -6.59 | -1.49 |
| MET | 4233 | 2.50 | 1.01 | 1.99 | 1.21 | 2.11 | 1.27 | -0.18 | -2.36 | 0.31 |
| MSN | 4478 | 0.73 | -0.07 | 0.68 | 0.92 | 0.48 | -0.82 | 1.03 | 0.46 | -0.37 |
| MSN | 4478 | 0.65 | 0.50 | 1.64 | 2.00 | 1.09 | 1.10 | -0.47 | -0.43 | -0.69 |
| MSN | 4478 | 1.15 | 0.66 | 1.16 | 0.69 | 1.21 | 1.20 | 1.28 | -0.47 | -0.22 |
| MSN | 4478 | -2.82 | -2.50 | -1.48 | -3.45 | -4.98 | -3.95 | -1.07 | -1.45 | -1.28 |
| NCK1 | 4690 | -0.53 | -0.90 | -0.24 | -2.54 | -2.28 | -1.71 | -0.91 | 0.43 | -0.75 |
| NCK1 | 4690 | -1.63 | -1.08 | -0.86 | 0.84 | -0.54 | 0.43 | 0.17 | -0.07 | 0.43 |
| NCK1 | 4690 | 0.18 | 1.59 | 0.28 | 5.83 | 5.33 | 1.97 | -0.08 | 0.22 | 2.62 |
| NCK1 | 4690 | -0.82 | -1.74 | -1.89 | 1.09 | 1.34 | 1.24 | -1.89 | -1.51 | -1.72 |
| NDRG1 | 10397 | -0.75 | -1.74 | -0.02 | -2.58 | -0.94 | -2.00 | -0.64 | -0.62 | -1.49 |
| NDRG1 | 10397 | 0.10 | -0.05 | 0.77 | -1.03 | 0.16 | -1.92 | 0.36 | -0.33 | 0.11 |
| NDRG1 | 10397 | -0.97 | -0.20 | 1.09 | 1.61 | 0.16 | 0.21 | -1.27 | -0.15 | -1.09 |
| NDRG1 | 10397 | 0.25 | -0.03 | -0.12 | -0.16 | -0.07 | 0.16 | -1.41 | -1.14 | -2.11 |
| PLCG2 | 5336 | -2.29 | -3.39 | -2.24 | -4.04 | -2.21 | -3.09 | -0.08 | -0.63 | -1.02 |
| PLCG2 | 5336 | -0.44 | 1.88 | 1.63 | 0.68 | 1.49 | 0.62 | 1.24 | -0.17 | -0.24 |
| PLCG2 | 5336 | -0.71 | 0.13 | 0.07 | -0.60 | -0.91 | 0.18 | -0.70 | -1.08 | -2.00 |
| PLCG2 | 5336 | 1.23 | -0.29 | -0.03 | 2.28 | 1.54 | 1.54 | -0.82 | 2.01 | 0.14 |
| PRKDC | 5591 | -0.47 | -0.29 | -1.88 | -0.09 | 0.04 | 0.43 | -0.47 | -2.18 | -2.45 |
| PRKDC | 5591 | -0.90 | 0.10 | -0.70 | 1.10 | 1.13 | 0.85 | 0.26 | -0.28 | -0.45 |
| PRKDC | 5591 | 0.88 | -0.22 | 0.47 | 2.66 | 1.07 | 0.37 | -0.37 | 2.29 | -0.20 |
| PRKDC | 5591 | 0.00 | -0.13 | 1.18 | -1.31 | -0.56 | 0.36 | 0.96 | 0.73 | 0.63 |
| PRNP | 5621 | -1.06 | -0.64 | -0.73 | -0.64 | -0.13 | -0.04 | -0.73 | -1.45 | -3.31 |
| PRNP | 5621 | -0.70 | -2.04 | -1.98 | -1.78 | -2.41 | -1.39 | -2.04 | -1.43 | -1.95 |
| PRNP | 5621 | -0.38 | -0.81 | 0.22 | -0.35 | -0.95 | -0.31 | 0.09 | 1.10 | 0.63 |
| PRNP | 5621 | 2.56 | 1.33 | 0.86 | 2.13 | 5.33 | 3.78 | -0.57 | 0.28 | 0.07 |
| S100A1 | 6271 | 5.02 | 6.21 | 3.37 | 2.52 | 2.29 | 3.57 | -0.27 | 2.31 | 0.94 |
| S100A1 | 6271 | 1.10 | 2.99 | 1.44 | -0.22 | 0.12 | 0.45 | 0.11 | 0.56 | 0.69 |
| S100A1 | 6271 | 0.30 | -0.43 | 0.35 | 5.71 | 2.44 | 3.85 | -0.72 | -3.21 | -0.99 |
| S100A1 | 6271 | 0.45 | -0.47 | -0.45 | -2.07 | -1.49 | -1.68 | -1.49 | -1.74 | -2.68 |
| S100B | 6285 | -2.29 | -2.97 | 0.31 | -1.82 | 0.44 | -0.49 | -1.37 | -2.05 | -1.16 |
| S100B | 6285 | 0.88 | 0.92 | 1.99 | 1.35 | 2.24 | 1.25 | 0.00 | 1.13 | 1.74 |
| S100B | 6285 | 3.51 | 2.10 | 2.17 | 4.18 | 6.91 | 3.67 | 1.19 | -2.25 | 0.18 |

| | | | | | | | | | | |
|---|---|---|---|---|---|---|---|---|---|---|
| S100B | 6285 | 1.68 | -0.38 | 0.99 | -1.14 | -0.73 | -0.60 | -0.64 | 0.69 | 0.16 |
| STK38 | 11329 | 1.49 | 5.29 | 3.09 | 3.60 | 2.30 | 2.82 | 2.02 | 0.73 | 1.13 |
| STK38 | 11329 | -0.52 | -0.40 | -1.50 | -0.98 | -1.20 | -1.06 | -0.12 | -1.25 | -0.96 |
| STK38 | 11329 | -1.22 | -3.45 | -4.06 | -1.76 | -1.26 | -1.72 | -0.46 | -1.38 | -1.25 |
| STK38 | 11329 | 1.81 | 0.46 | -0.32 | 1.38 | 2.17 | 1.27 | -0.41 | -1.89 | 0.87 |
| TGFBI | 7045 | 0.37 | 0.40 | 0.94 | -0.13 | -0.12 | -0.52 | -2.14 | -1.02 | -1.61 |
| TGFBI | 7045 | -0.37 | -0.65 | -0.89 | -1.27 | -0.32 | -0.12 | -1.00 | 0.46 | -0.56 |
| TGFBI | 7045 | 0.06 | -1.53 | 0.45 | -0.88 | 0.38 | 0.23 | 1.74 | -1.17 | 0.86 |
| TGFBI | 7045 | 2.37 | 2.70 | 2.87 | 3.64 | 3.99 | 2.00 | 1.25 | 0.71 | 1.33 |
| UCHL1 | 7345 | -1.49 | -0.56 | 0.03 | -0.55 | -0.56 | -1.12 | 0.21 | -0.65 | -1.40 |
| UCHL1 | 7345 | -1.57 | -4.90 | -2.65 | -3.42 | -4.87 | -3.03 | -0.30 | -0.09 | -0.89 |
| UCHL1 | 7345 | 0.05 | -1.98 | -0.05 | 1.48 | 0.95 | 1.42 | 1.89 | 0.33 | 0.51 |
| UCHL1 | 7345 | -3.18 | -2.90 | -2.16 | -3.01 | -4.87 | -3.54 | -1.30 | -2.15 | -1.23 |
| VCAM1 | 7412 | -0.46 | -0.28 | 0.28 | -0.79 | -0.08 | -0.79 | -0.33 | -1.14 | -0.06 |
| VCAM1 | 7412 | -0.25 | -0.04 | -0.21 | 0.42 | 0.86 | 0.79 | -0.56 | -1.52 | -0.56 |
| VCAM1 | 7412 | 0.83 | 2.08 | 2.68 | 2.26 | 2.66 | 3.13 | 0.21 | 1.19 | 0.48 |
| VCAM1 | 7412 | 2.83 | 2.11 | 0.80 | 2.79 | 2.71 | 1.30 | -0.89 | 2.08 | 1.32 |
| WWTR1 | 25937 | -0.63 | -0.47 | -0.49 | 0.68 | 1.40 | 0.72 | -0.14 | -0.91 | -0.26 |
| WWTR1 | 25937 | -0.46 | -1.71 | -3.15 | -4.62 | -1.81 | -2.37 | 0.54 | 0.79 | 1.27 |
| WWTR1 | 25937 | 0.92 | 0.82 | 0.83 | 2.03 | 2.25 | 2.94 | -0.42 | 0.34 | 0.71 |
| WWTR1 | 25937 | -2.70 | -2.88 | -1.77 | -3.42 | -6.68 | -5.64 | -1.60 | -1.73 | -2.70 |

## siRNA screen data for 40 "driver network member" genes from driver networks

| Gene name | Entrez.Gene.Id | MDAMB436 TNBC | MDAMB436 TNBC | MDAMB436 TNBC | MDAMB453 TNBC | MDAMB453 TNBC | MDAMB453 TNBC | MDAMB468 TNBC | MDAMB468 TNBC | MDAMB468 TNBC |
|---|---|---|---|---|---|---|---|---|---|---|
| ANXA1 | 301 | 1.09 | 1.65 | 1.19 | 0.05 | -0.67 | -0.98 | 1.07 | 3.28 | 1.26 |
| ANXA1 | 301 | 2.06 | 0.06 | 4.05 | -1.26 | 0.51 | 1.68 | -0.25 | -3.36 | -1.10 |
| ANXA1 | 301 | -1.15 | -0.77 | -2.01 | -1.88 | -2.25 | -2.20 | -0.37 | 2.42 | -1.24 |
| ANXA1 | 301 | -0.63 | -0.14 | -0.23 | -0.58 | -0.81 | -1.20 | 2.34 | 1.63 | 1.35 |
| ATP1B3 | 483 | -0.02 | -0.68 | -3.10 | -0.96 | 0.25 | -0.67 | -1.38 | -2.21 | -0.42 |
| ATP1B3 | 483 | -1.41 | -0.66 | -1.25 | 1.31 | -1.71 | 2.04 | -0.71 | 0.14 | -0.24 |
| ATP1B3 | 483 | -3.67 | -2.81 | -2.87 | -1.60 | -0.29 | 0.59 | -2.22 | -1.37 | -2.51 |
| ATP1B3 | 483 | -2.99 | 0.29 | -0.57 | -0.81 | 0.31 | 0.79 | -0.27 | -0.14 | -0.38 |
| CAV2 | 858 | 0.90 | 0.78 | 2.43 | -1.06 | -2.17 | -2.31 | -1.99 | -1.64 | -1.50 |
| CAV2 | 858 | -0.11 | -0.56 | -0.78 | -1.56 | -1.25 | -1.00 | -0.54 | -1.56 | -0.77 |
| CAV2 | 858 | 1.02 | -0.33 | 3.35 | -0.61 | 2.51 | -2.36 | -1.04 | 2.72 | 0.16 |
| CAV2 | 858 | 1.65 | 1.70 | 1.96 | -0.77 | -0.15 | -0.53 | 1.85 | 3.08 | 2.78 |
| CD44 | 960 | -0.14 | 1.82 | 2.20 | -0.67 | 0.20 | 0.12 | 0.08 | 0.62 | -0.33 |
| CD44 | 960 | 0.72 | -2.31 | -1.86 | -1.54 | -0.22 | -0.68 | -0.49 | -1.46 | 0.06 |
| CD44 | 960 | -2.33 | -2.20 | -3.62 | 0.09 | 1.06 | 1.97 | -0.29 | -0.62 | -0.17 |
| CD44 | 960 | 0.77 | 2.39 | 1.75 | -0.06 | 0.77 | -0.35 | 0.80 | -1.25 | -1.27 |
| CDKN2A | 1029 | 0.21 | -0.25 | 0.01 | -0.15 | 0.72 | 1.51 | 0.05 | 1.89 | 0.16 |
| CDKN2A | 1029 | -0.82 | -0.42 | -0.10 | -1.63 | -0.24 | -1.02 | 0.54 | -1.00 | -0.08 |
| CDKN2A | 1029 | -0.06 | -0.65 | 0.76 | -1.36 | -0.77 | -0.53 | -0.61 | -2.59 | -1.54 |
| CDKN2A | 1029 | 3.87 | 0.28 | -0.24 | -3.22 | -0.09 | 0.37 | 0.18 | -0.87 | -0.30 |
| ENO1 | 2023 | 0.70 | -0.60 | -0.41 | -1.17 | -0.81 | 0.08 | 1.80 | 1.04 | 2.77 |
| ENO1 | 2023 | -1.15 | -0.52 | -0.87 | -1.35 | -0.39 | -1.95 | 0.06 | -1.28 | -1.04 |
| ENO1 | 2023 | 1.54 | -1.03 | -3.04 | -2.98 | -0.21 | -0.02 | 0.36 | -0.37 | -1.26 |
| ENO1 | 2023 | 0.49 | 0.01 | 0.96 | 1.32 | 0.67 | 1.29 | 0.94 | 0.01 | 0.63 |
| EPB41L2 | 2037 | 1.01 | 2.07 | 0.46 | -0.80 | -0.55 | -1.89 | 0.06 | -1.68 | -1.24 |
| EPB41L2 | 2037 | 0.77 | -0.11 | 1.26 | -0.81 | 1.37 | -1.38 | -0.91 | -0.08 | 0.01 |
| EPB41L2 | 2037 | -0.96 | -1.60 | -3.72 | -0.55 | -0.52 | -1.34 | -2.77 | -1.69 | -10.15 |
| EPB41L2 | 2037 | 2.98 | 2.76 | 3.43 | 1.38 | 2.32 | 1.27 | 0.28 | 2.57 | 1.82 |
| FOLR1 | 2348 | -1.21 | -1.13 | -0.33 | -0.58 | -1.19 | -0.76 | -2.57 | -0.54 | -0.63 |
| FOLR1 | 2348 | 0.25 | -0.78 | -0.09 | 0.47 | -1.22 | 0.65 | -2.00 | -4.08 | -3.20 |
| FOLR1 | 2348 | 0.62 | 1.28 | 1.50 | 0.58 | -0.07 | -0.52 | 0.19 | 0.45 | 0.00 |

| | | | | | | | | | | |
|---|---|---|---|---|---|---|---|---|---|---|
| FOLR1 | 2348 | -2.84 | -1.67 | -3.66 | -0.91 | -1.09 | 0.15 | -2.27 | -0.99 | -0.72 |
| ICAM1 | 3383 | -1.48 | 0.85 | -1.78 | 0.59 | 0.26 | -0.99 | -2.00 | -0.69 | -0.49 |
| ICAM1 | 3383 | 0.98 | -0.22 | -0.33 | -0.29 | 1.35 | -0.01 | 0.00 | 2.02 | 2.49 |
| ICAM1 | 3383 | -1.19 | -0.40 | -0.27 | -1.80 | -0.65 | -0.57 | 0.96 | 0.46 | 1.12 |
| ICAM1 | 3383 | 3.69 | -2.45 | 1.52 | -2.01 | 1.31 | -0.53 | 1.63 | 1.90 | 2.29 |
| IGF2BP2 | 10644 | 0.15 | 2.03 | 0.63 | -0.54 | 0.03 | -1.49 | 2.80 | 1.92 | 1.96 |
| IGF2BP2 | 10644 | -0.11 | -0.39 | -1.03 | -0.62 | 0.36 | 0.36 | 0.06 | -0.20 | -1.27 |
| IGF2BP2 | 10644 | -1.38 | -0.66 | -3.08 | -1.52 | -1.93 | -0.68 | -2.49 | -1.31 | -0.86 |
| IGF2BP2 | 10644 | -1.32 | -0.88 | -1.20 | -0.44 | -0.19 | 0.94 | -0.96 | -0.71 | -1.57 |
| IGF2BP3 | 10643 | -0.10 | -2.10 | -2.45 | -1.64 | 0.81 | -2.27 | -1.42 | -3.77 | -2.42 |
| IGF2BP3 | 10643 | 0.00 | -0.20 | -1.58 | -0.48 | -0.52 | -0.32 | -2.14 | -1.56 | -3.32 |
| IGF2BP3 | 10643 | -0.03 | 0.43 | -3.77 | -1.55 | -2.62 | 0.08 | -1.50 | -1.80 | -2.19 |
| IGF2BP3 | 10643 | 2.44 | 4.00 | -1.94 | 1.16 | -0.50 | -0.18 | 0.51 | 0.50 | -0.28 |
| IMPA2 | 3613 | -0.80 | -1.69 | -0.14 | 3.20 | 0.29 | 1.78 | -3.88 | -1.37 | -3.77 |
| IMPA2 | 3613 | 1.98 | 0.53 | 0.40 | 0.71 | 1.24 | 1.77 | -0.87 | -1.79 | -1.71 |
| IMPA2 | 3613 | -1.05 | 0.36 | -2.91 | -0.72 | -0.40 | 1.11 | -0.27 | 0.45 | -0.55 |
| IMPA2 | 3613 | -1.21 | -1.30 | -2.04 | -2.20 | 0.16 | 0.34 | -1.84 | -0.93 | -2.41 |
| KLF5 | 688 | -1.85 | -0.83 | -3.44 | -1.04 | -0.34 | -0.66 | -1.95 | -6.08 | -2.79 |
| KLF5 | 688 | 0.54 | -0.67 | 1.11 | 0.92 | -0.81 | -0.02 | -0.73 | 0.27 | -0.28 |
| KLF5 | 688 | -0.39 | -0.83 | 1.40 | -1.46 | 0.30 | 2.46 | -1.78 | -0.11 | -0.15 |
| KLF5 | 688 | 0.35 | -2.58 | 0.74 | 0.98 | -1.46 | 1.91 | 0.47 | 0.91 | 1.33 |
| LYN | 4067 | 0.98 | 2.76 | 0.86 | 0.20 | -0.23 | 0.07 | 0.40 | 0.45 | 0.31 |
| LYN | 4067 | -0.76 | -0.79 | -2.87 | -2.45 | -0.52 | -1.53 | 2.30 | 2.43 | 1.26 |
| LYN | 4067 | 2.50 | 0.73 | 0.97 | 0.09 | -0.30 | 0.79 | -0.03 | 0.47 | 3.09 |
| LYN | 4067 | 1.87 | 1.59 | 1.33 | -1.15 | -0.07 | -0.10 | 1.36 | 2.02 | 2.66 |
| MCM3 | 4172 | 0.40 | -1.10 | -0.89 | -4.01 | -2.52 | -3.94 | -1.55 | -3.13 | -1.32 |
| MCM3 | 4172 | -0.60 | -0.58 | -2.85 | -3.02 | -2.15 | -1.31 | 0.40 | -0.91 | -0.03 |
| MCM3 | 4172 | -3.48 | -6.21 | -10.33 | -2.15 | -1.19 | -3.85 | -2.02 | -1.11 | -0.80 |
| MCM3 | 4172 | 2.71 | 2.58 | 2.33 | 2.06 | -0.72 | 0.41 | 2.15 | 2.51 | 2.65 |
| MCM5 | 4174 | 0.09 | -0.62 | 0.58 | -0.95 | -0.43 | -1.47 | -1.38 | -0.68 | -1.13 |
| MCM5 | 4174 | 1.25 | -0.16 | -0.95 | 0.67 | 0.29 | 0.99 | 2.12 | -0.85 | 0.91 |
| MCM5 | 4174 | -0.18 | -0.83 | -0.23 | -0.67 | 1.55 | 0.96 | 0.44 | -0.08 | -0.08 |
| MCM5 | 4174 | 0.98 | 0.53 | -0.72 | -3.12 | -0.41 | 1.37 | 1.16 | -0.34 | 0.00 |
| MET | 4233 | 1.54 | 0.69 | -0.24 | -0.21 | -0.03 | -0.55 | -0.42 | -1.77 | -1.57 |

| | | | | | | | | | | |
|---|---|---|---|---|---|---|---|---|---|---|
| MET | 4233 | -0.28 | -1.29 | -4.61 | -1.55 | 0.06 | -0.18 | 0.09 | 5.16 | 2.28 |
| MET | 4233 | -2.66 | -2.58 | -2.81 | -2.81 | -1.64 | -0.96 | -4.62 | -2.83 | -5.30 |
| MET | 4233 | 0.51 | 0.90 | 0.46 | -3.22 | -1.95 | -1.03 | 0.10 | 1.77 | 0.69 |
| MSN | 4478 | 0.75 | -1.76 | 0.25 | 1.50 | -1.71 | -0.16 | 0.82 | -1.72 | 1.21 |
| MSN | 4478 | 0.95 | 0.03 | 0.64 | -1.67 | -0.01 | -0.64 | -0.88 | -0.85 | -1.96 |
| MSN | 4478 | 0.67 | -0.21 | -0.29 | -1.26 | -1.54 | 1.51 | 0.21 | 0.72 | 0.69 |
| MSN | 4478 | -2.15 | -0.38 | -1.34 | -2.11 | -0.54 | -0.33 | -3.79 | -3.72 | -4.55 |
| NCK1 | 4690 | 1.63 | 0.34 | 2.55 | -0.35 | -0.34 | -0.85 | -1.25 | -4.23 | -1.33 |
| NCK1 | 4690 | -1.04 | -0.62 | -1.48 | -0.79 | -2.25 | -0.44 | -0.89 | -0.72 | -0.25 |
| NCK1 | 4690 | -0.51 | 0.26 | 0.82 | 0.04 | 0.28 | -0.25 | -1.43 | -0.17 | 0.86 |
| NCK1 | 4690 | -1.59 | -1.11 | -2.03 | -1.42 | -1.60 | -1.93 | 0.15 | 0.25 | 0.41 |
| NDRG1 | 10397 | -0.22 | -1.15 | -1.46 | -0.37 | -1.10 | -0.62 | -0.14 | -0.53 | -0.25 |
| NDRG1 | 10397 | 0.20 | -2.17 | -1.35 | 0.48 | -2.06 | -0.08 | 0.10 | -1.61 | -0.12 |
| NDRG1 | 10397 | 0.08 | 0.63 | 0.18 | -1.41 | -0.34 | -0.83 | 0.30 | -0.05 | -0.37 |
| NDRG1 | 10397 | 0.14 | -0.99 | 1.11 | -0.45 | 0.75 | -0.60 | -1.99 | -0.93 | -2.12 |
| PLCG2 | 5336 | -1.59 | -1.77 | -0.91 | -0.93 | -2.10 | -0.51 | -4.98 | -6.57 | -6.68 |
| PLCG2 | 5336 | 0.66 | 1.24 | 0.59 | -0.64 | 1.04 | -0.48 | 0.16 | -0.36 | 1.43 |
| PLCG2 | 5336 | 0.71 | 1.19 | 2.32 | -0.52 | -0.25 | 0.32 | -1.52 | 0.02 | 0.23 |
| PLCG2 | 5336 | 0.32 | 0.25 | 0.83 | 0.50 | -0.17 | 0.38 | -3.20 | -3.86 | -5.46 |
| PRKDC | 5591 | -0.33 | -2.08 | -2.54 | -0.15 | -0.37 | -1.89 | -0.27 | 0.06 | -0.65 |
| PRKDC | 5591 | 0.67 | -3.69 | -1.22 | -0.20 | -0.16 | -1.25 | -1.62 | -0.68 | -1.54 |
| PRKDC | 5591 | 0.63 | 2.07 | 1.29 | -0.56 | 1.02 | 1.00 | 2.35 | 1.76 | 0.68 |
| PRKDC | 5591 | 0.72 | 1.67 | 0.74 | -0.59 | -0.29 | -0.55 | -2.32 | -2.61 | -5.50 |
| PRNP | 5621 | -1.09 | -0.27 | -1.97 | -0.46 | -1.31 | -0.75 | 0.14 | 0.43 | 0.22 |
| PRNP | 5621 | -1.12 | -0.77 | -2.97 | -0.16 | -1.26 | -0.15 | -3.84 | -4.35 | -5.37 |
| PRNP | 5621 | -2.11 | -0.45 | -0.66 | -0.81 | -0.33 | -1.51 | -0.69 | -0.13 | 0.20 |
| PRNP | 5621 | -1.79 | -2.86 | -0.47 | 1.36 | -0.96 | 3.52 | -0.02 | 0.22 | -0.24 |
| S100A1 | 6271 | 1.86 | 1.21 | 2.30 | -0.40 | 0.05 | -0.26 | 1.54 | 1.77 | 1.96 |
| S100A1 | 6271 | 0.01 | 0.59 | 1.33 | 0.45 | 0.38 | -1.12 | -0.97 | -0.62 | -0.24 |
| S100A1 | 6271 | -1.66 | -2.07 | -1.97 | -0.76 | -0.95 | 0.51 | -2.06 | -0.07 | -0.17 |
| S100A1 | 6271 | 0.11 | -1.66 | -1.25 | -1.79 | 0.76 | -0.95 | -1.94 | -0.71 | -1.22 |
| S100B | 6285 | 1.67 | 1.07 | 1.06 | -1.80 | -0.41 | 0.15 | 0.74 | 0.44 | -7.13 |
| S100B | 6285 | 0.63 | 0.06 | 1.13 | -2.05 | -0.26 | -1.53 | 1.91 | 2.01 | 2.95 |
| S100B | 6285 | -0.78 | 1.86 | 0.73 | 0.07 | 0.00 | 0.02 | 1.47 | 0.98 | 2.25 |

| | | | | | | | | | | |
|---|---|---|---|---|---|---|---|---|---|---|
| S100B | 6285 | -0.94 | -0.11 | -0.36 | -1.28 | -2.53 | -2.86 | -0.17 | 0.10 | -1.19 |
| STK38 | 11329 | 1.83 | 1.83 | 2.36 | 0.19 | -0.52 | 1.74 | 1.11 | 0.16 | 0.33 |
| STK38 | 11329 | 0.74 | -1.19 | -3.92 | -0.12 | -0.20 | -0.50 | -1.66 | -0.79 | -1.66 |
| STK38 | 11329 | -0.30 | 0.16 | -1.43 | -1.20 | 0.14 | -1.43 | -4.68 | -3.37 | -4.84 |
| STK38 | 11329 | 0.66 | 0.64 | 0.39 | -2.33 | 1.64 | 1.64 | 1.11 | 0.11 | 0.82 |
| TGFBI | 7045 | -0.04 | 0.47 | 1.17 | -0.56 | -0.34 | -0.30 | 0.09 | -0.56 | -1.12 |
| TGFBI | 7045 | -1.06 | -0.64 | -4.66 | -1.47 | -0.28 | -1.73 | -0.73 | -0.93 | 0.26 |
| TGFBI | 7045 | 0.12 | -0.17 | -2.15 | -0.45 | -0.98 | -1.22 | -7.21 | -5.96 | -8.54 |
| TGFBI | 7045 | 0.00 | 0.09 | 0.59 | 0.60 | 1.89 | 0.47 | -0.17 | -0.55 | -0.27 |
| UCHL1 | 7345 | -2.01 | -0.52 | -0.61 | -0.62 | -0.02 | -1.12 | -2.42 | -1.90 | -1.06 |
| UCHL1 | 7345 | -1.68 | -1.80 | -2.89 | -2.89 | -3.71 | -3.60 | -2.24 | -2.71 | -0.89 |
| UCHL1 | 7345 | -0.12 | -1.60 | -0.44 | 0.74 | 2.33 | 1.53 | -0.08 | -2.72 | -2.41 |
| UCHL1 | 7345 | -4.07 | -0.41 | -1.55 | -3.54 | -1.15 | -0.10 | -1.88 | -2.15 | -3.19 |
| VCAM1 | 7412 | -0.90 | 0.49 | -3.13 | -0.85 | -0.58 | -1.32 | -1.53 | -2.59 | -2.44 |
| VCAM1 | 7412 | -0.79 | -0.61 | 0.73 | 0.02 | -2.08 | 0.53 | -0.45 | 0.25 | 0.21 |
| VCAM1 | 7412 | 0.94 | 1.88 | 0.57 | 1.49 | 1.10 | 0.69 | 0.70 | 1.29 | 1.34 |
| VCAM1 | 7412 | 2.17 | 2.37 | 2.69 | 1.49 | -0.07 | 0.89 | 2.42 | 2.32 | 2.99 |
| WWTR1 | 25937 | -0.63 | -1.21 | -1.95 | -0.08 | -0.09 | 0.42 | 1.36 | 0.86 | -0.07 |
| WWTR1 | 25937 | -1.32 | -1.42 | -0.19 | -3.27 | -0.20 | -1.58 | 0.32 | -0.85 | 0.71 |
| WWTR1 | 25937 | 4.03 | 1.32 | 1.54 | -0.11 | 0.59 | -0.02 | 1.53 | 1.56 | 1.70 |
| WWTR1 | 25937 | -0.92 | -2.01 | -0.40 | -3.20 | -1.61 | -0.13 | -1.00 | -0.24 | -1.58 |

**siRNA screen data for 40 "driver network member" genes from driver networks**

| Gene name | Entrez.Gene.Id | HER2_AU565 HER2+ | HER2_AU565 HER2+ | HER2_AU565 HER2+ | ER_BT483 ER+ | ER_BT483 ER+ | ER_BT483 ER+ | HER2_SKBr3 HER2+ | HER2_SKBr3 HER2+ | HER2_SKBr3 HER2+ |
|---|---|---|---|---|---|---|---|---|---|---|
| ANXA1 | 301 | -0.10 | 0.15 | -1.43 | -0.24 | -0.16 | -0.23 | -0.89 | 0.07 | 0.07 |
| ANXA1 | 301 | 1.61 | -0.18 | 2.30 | 0.69 | 0.21 | 0.73 | -0.02 | 0.32 | 2.45 |
| ANXA1 | 301 | -1.49 | -0.07 | -1.95 | -0.96 | -0.91 | -1.40 | -1.23 | 3.57 | -2.64 |
| ANXA1 | 301 | -0.55 | 0.37 | -0.69 | -2.67 | -0.32 | -1.69 | 0.34 | -0.27 | 0.21 |
| ATP1B3 | 483 | 1.05 | -0.80 | 1.23 | 0.17 | 0.07 | -0.48 | 2.44 | 0.52 | 0.76 |
| ATP1B3 | 483 | -0.25 | 0.65 | 0.31 | 1.64 | 0.28 | 1.02 | -0.25 | -0.82 | 0.10 |
| ATP1B3 | 483 | -2.02 | -0.92 | -1.30 | -0.37 | -0.43 | 0.74 | -0.53 | -0.06 | -0.85 |
| ATP1B3 | 483 | 2.06 | 0.49 | 2.44 | 1.41 | 0.89 | 1.48 | -0.60 | -0.15 | -1.18 |
| CAV2 | 858 | 0.71 | -0.39 | -0.32 | -2.49 | -1.72 | -2.20 | 1.32 | 2.75 | 1.15 |
| CAV2 | 858 | 0.47 | -0.38 | -0.13 | -0.32 | -1.31 | -0.68 | -0.49 | -0.91 | 0.72 |
| CAV2 | 858 | 2.46 | 0.03 | 2.73 | 1.51 | 0.25 | 0.25 | 0.49 | 2.62 | 1.54 |
| CAV2 | 858 | 0.52 | 0.33 | 1.82 | -0.08 | -0.96 | 0.95 | -1.19 | -0.73 | 0.36 |
| CD44 | 960 | 0.43 | 2.00 | 0.97 | 1.38 | 1.58 | 0.65 | -0.73 | 1.33 | 0.79 |
| CD44 | 960 | -2.33 | -0.08 | -0.22 | -0.89 | -0.81 | -0.12 | 1.92 | 1.02 | 0.27 |
| CD44 | 960 | -0.18 | -0.51 | -0.45 | -0.45 | -0.05 | -0.20 | -0.14 | -0.34 | -0.55 |
| CD44 | 960 | -0.32 | 0.52 | -0.22 | -0.44 | 1.16 | 0.08 | 0.45 | 0.22 | -0.27 |
| CDKN2A | 1029 | 1.07 | 3.10 | 1.98 | -1.25 | -0.05 | 0.79 | 1.18 | 1.67 | 0.23 |
| CDKN2A | 1029 | -1.30 | -0.07 | -2.10 | 0.05 | -0.09 | 0.23 | 0.13 | -0.27 | -0.50 |
| CDKN2A | 1029 | -0.30 | -0.57 | -0.35 | 0.62 | 1.72 | 0.81 | 0.40 | 0.04 | 0.43 |
| CDKN2A | 1029 | -2.01 | -1.13 | -2.24 | -1.12 | -0.36 | 0.25 | 0.50 | 0.43 | 1.91 |
| ENO1 | 2023 | 0.35 | -1.03 | -0.47 | -3.03 | -3.04 | -2.05 | -0.80 | -0.41 | -0.08 |
| ENO1 | 2023 | -0.38 | -0.30 | -0.05 | 0.15 | 0.09 | -0.06 | 1.12 | 0.13 | 1.61 |
| ENO1 | 2023 | 0.21 | -0.70 | -2.17 | -3.12 | -1.88 | -1.96 | -0.40 | -0.61 | 0.18 |
| ENO1 | 2023 | 0.25 | 0.15 | -0.03 | -0.59 | -0.08 | 0.18 | 0.63 | 0.22 | 0.78 |
| EPB41L2 | 2037 | -0.04 | 0.76 | 0.20 | 0.19 | -1.42 | -0.33 | -0.69 | -0.40 | 0.34 |
| EPB41L2 | 2037 | -0.08 | 0.44 | 0.24 | 0.19 | -0.14 | -0.21 | -0.06 | -0.33 | 0.27 |
| EPB41L2 | 2037 | -4.35 | -4.34 | -4.18 | -0.16 | -1.37 | 0.49 | -2.64 | -3.56 | -2.91 |
| EPB41L2 | 2037 | 2.86 | 1.97 | 4.26 | 1.15 | 2.11 | 0.94 | -0.09 | 0.92 | 1.76 |
| FOLR1 | 2348 | -0.23 | -0.37 | -0.06 | 0.92 | -0.32 | 0.71 | -1.80 | -0.14 | -0.42 |
| FOLR1 | 2348 | 0.31 | -1.50 | -0.44 | 0.42 | -1.43 | -0.29 | -2.04 | 0.94 | 0.01 |
| FOLR1 | 2348 | 1.33 | 0.25 | 1.60 | 0.80 | 0.03 | 0.13 | 1.58 | 2.07 | 1.19 |

| | | | | | | | | | | |
|---|---|---|---|---|---|---|---|---|---|---|
| FOLR1 | 2348 | -1.54 | -2.05 | -4.08 | -0.43 | -0.79 | -1.14 | -0.10 | -0.90 | -0.97 |
| ICAM1 | 3383 | 0.41 | -0.43 | -1.14 | -0.16 | 0.59 | -0.28 | 1.29 | 2.86 | 1.56 |
| ICAM1 | 3383 | -1.26 | -0.25 | -1.53 | -0.53 | -2.35 | -1.29 | 0.99 | 0.99 | -3.60 |
| ICAM1 | 3383 | -1.28 | -2.32 | -0.99 | 0.05 | 0.27 | -0.13 | 0.89 | -0.06 | 0.49 |
| ICAM1 | 3383 | 3.05 | 3.51 | 1.58 | 1.11 | 0.79 | -1.05 | 0.07 | 0.29 | 2.13 |
| IGF2BP2 | 10644 | -1.28 | 0.31 | 0.16 | -0.28 | -0.57 | -1.39 | 1.08 | -0.06 | 0.63 |
| IGF2BP2 | 10644 | -3.34 | -2.71 | -4.71 | -0.03 | -0.88 | -0.26 | 0.60 | -0.13 | 1.02 |
| IGF2BP2 | 10644 | -0.99 | 0.08 | -1.46 | 0.29 | -0.35 | -0.09 | 0.62 | 0.06 | 0.35 |
| IGF2BP2 | 10644 | 0.50 | -1.30 | -1.24 | -0.17 | 0.33 | -0.17 | 0.52 | 0.69 | 0.39 |
| IGF2BP3 | 10643 | -1.16 | 0.78 | -0.33 | 0.09 | -0.20 | 0.31 | 1.43 | 0.18 | 1.56 |
| IGF2BP3 | 10643 | 0.43 | 0.16 | 1.94 | -0.89 | 0.13 | 0.80 | -0.50 | -1.62 | -1.05 |
| IGF2BP3 | 10643 | 0.19 | -1.81 | -0.64 | 0.04 | 0.89 | 0.59 | 0.54 | -1.36 | 0.19 |
| IGF2BP3 | 10643 | 1.02 | 0.05 | 1.70 | 0.51 | 0.68 | -7.51 | 0.73 | 0.20 | 1.23 |
| IMPA2 | 3613 | 0.34 | 0.46 | 0.11 | -1.39 | -0.57 | -0.06 | 0.47 | -0.03 | 1.48 |
| IMPA2 | 3613 | -1.14 | -2.79 | -0.34 | 0.23 | 0.18 | 0.25 | -0.31 | -0.56 | -0.48 |
| IMPA2 | 3613 | -0.24 | -0.93 | -0.97 | 0.53 | 0.93 | 0.53 | -0.36 | -0.21 | -1.21 |
| IMPA2 | 3613 | -1.50 | -1.39 | -1.49 | -1.50 | -0.59 | -1.39 | 1.59 | -0.32 | 0.13 |
| KLF5 | 688 | -2.53 | -2.26 | -4.65 | 0.31 | -1.02 | -0.05 | 0.10 | -1.31 | -0.67 |
| KLF5 | 688 | 0.83 | 0.44 | 3.79 | 0.23 | -2.21 | 0.46 | 0.88 | 0.92 | 1.06 |
| KLF5 | 688 | -0.40 | -1.14 | 0.10 | -1.19 | -0.97 | -0.94 | 0.40 | 0.12 | 0.13 |
| KLF5 | 688 | 0.16 | 0.92 | 0.17 | -0.33 | 0.05 | 1.11 | 1.39 | 2.21 | 1.16 |
| LYN | 4067 | 1.22 | 1.80 | 0.19 | -0.30 | 0.46 | 0.61 | -0.70 | -0.09 | 0.24 |
| LYN | 4067 | -1.67 | -0.72 | -2.52 | 0.37 | 1.21 | 0.49 | 0.60 | -0.59 | 0.02 |
| LYN | 4067 | 0.45 | 0.58 | 0.35 | 0.06 | -1.80 | 0.24 | -0.22 | -1.21 | -0.04 |
| LYN | 4067 | 2.87 | 1.98 | 2.13 | 0.62 | 0.67 | 1.84 | 0.06 | 1.74 | 1.67 |
| MCM3 | 4172 | -2.22 | -1.28 | -0.35 | -0.72 | -1.70 | -0.98 | -0.09 | -0.67 | 0.24 |
| MCM3 | 4172 | -2.07 | -0.46 | -2.75 | -0.66 | -2.62 | -0.55 | -0.79 | -1.12 | -2.31 |
| MCM3 | 4172 | -3.34 | -1.80 | -4.82 | -0.12 | -0.15 | -0.09 | -3.30 | -1.75 | -2.60 |
| MCM3 | 4172 | 4.10 | 4.77 | 4.25 | 1.28 | -0.55 | 0.99 | 1.36 | 0.07 | 1.84 |
| MCM5 | 4174 | -0.40 | 0.46 | -1.05 | -1.83 | 0.54 | -0.54 | -2.01 | 1.76 | 0.48 |
| MCM5 | 4174 | 0.88 | 0.88 | 3.39 | 1.76 | 1.71 | 1.23 | 1.65 | 2.14 | 0.28 |
| MCM5 | 4174 | 1.12 | 1.50 | 0.85 | 2.32 | 1.27 | 0.41 | 0.54 | 1.21 | 1.89 |
| MCM5 | 4174 | -0.46 | -2.00 | -0.11 | -0.05 | 0.30 | -0.02 | 0.50 | 1.00 | 0.57 |
| MET | 4233 | -0.72 | -1.31 | 0.23 | 0.29 | -0.21 | -0.31 | -2.83 | -1.48 | -0.58 |

| | | | | | | | | | | |
|---|---|---|---|---|---|---|---|---|---|---|
| MET | 4233 | 0.36 | 1.74 | -0.87 | 1.24 | -0.06 | 0.59 | 0.76 | 0.74 | 0.35 |
| MET | 4233 | -2.92 | -1.30 | -3.64 | -2.10 | -1.81 | -0.94 | -4.27 | -3.79 | -2.26 |
| MET | 4233 | -0.92 | 1.08 | -2.13 | -0.55 | -0.10 | -1.61 | -0.45 | -0.15 | -0.13 |
| MSN | 4478 | -1.53 | 0.34 | 0.45 | 0.15 | 0.48 | -0.02 | -0.11 | 0.64 | 0.64 |
| MSN | 4478 | 0.65 | 0.47 | 1.92 | -0.21 | 0.90 | 1.24 | 1.40 | 1.49 | 0.60 |
| MSN | 4478 | -2.89 | -2.06 | -0.71 | 0.09 | 0.63 | -1.32 | 0.43 | 0.58 | 0.82 |
| MSN | 4478 | -0.15 | -0.12 | -1.04 | -0.08 | -0.17 | -0.95 | 1.25 | 1.51 | -0.31 |
| NCK1 | 4690 | -0.65 | -0.32 | -1.66 | 0.50 | -0.56 | -0.30 | -0.16 | 0.39 | -0.65 |
| NCK1 | 4690 | -0.37 | -0.41 | 0.54 | -1.93 | -2.50 | -1.62 | -0.45 | 0.22 | 0.31 |
| NCK1 | 4690 | -1.61 | 2.80 | 3.92 | -0.02 | 1.82 | -1.29 | -0.05 | -0.54 | 0.06 |
| NCK1 | 4690 | -2.06 | -0.42 | -1.45 | -0.01 | 0.28 | 0.26 | -1.43 | -0.47 | -1.97 |
| NDRG1 | 10397 | 0.16 | 0.52 | 0.33 | -0.81 | -0.28 | 0.48 | -0.46 | 0.17 | 0.06 |
| NDRG1 | 10397 | 0.24 | -0.70 | 0.07 | 0.36 | 0.94 | 0.36 | -0.41 | -0.36 | -0.16 |
| NDRG1 | 10397 | -0.09 | 0.40 | -0.15 | 1.37 | 1.47 | 0.22 | 0.58 | 0.63 | -0.80 |
| NDRG1 | 10397 | -2.55 | -2.66 | -3.81 | 0.53 | 1.12 | -0.09 | -1.32 | -1.05 | -0.21 |
| PLCG2 | 5336 | 0.03 | 0.28 | 0.20 | -0.41 | -0.67 | -1.29 | -0.18 | -0.13 | 1.06 |
| PLCG2 | 5336 | 0.59 | 3.00 | -0.33 | 1.02 | 2.08 | 1.74 | -0.38 | 0.32 | 0.30 |
| PLCG2 | 5336 | -0.54 | 1.64 | -0.47 | -1.51 | -1.45 | -1.38 | 1.13 | 0.00 | 0.94 |
| PLCG2 | 5336 | 1.78 | 0.57 | 2.54 | -0.60 | 0.47 | 0.87 | 0.23 | -0.18 | 0.84 |
| PRKDC | 5591 | 0.41 | -0.04 | -0.33 | -1.22 | -1.79 | -0.85 | 0.43 | -0.34 | 0.08 |
| PRKDC | 5591 | 0.42 | -3.52 | -0.41 | 0.36 | 0.13 | 0.96 | 2.20 | 1.88 | 1.78 |
| PRKDC | 5591 | 0.31 | 1.26 | 0.37 | -0.81 | 1.57 | 0.94 | 3.39 | 1.30 | 0.93 |
| PRKDC | 5591 | -0.76 | 0.05 | -1.64 | 0.96 | 1.93 | 1.23 | 2.53 | 7.48 | 1.10 |
| PRNP | 5621 | -0.62 | -0.59 | -1.90 | -1.32 | -1.51 | 0.02 | 1.34 | 0.23 | 0.86 |
| PRNP | 5621 | -1.44 | 0.11 | -3.75 | -1.73 | -2.10 | -2.39 | 1.10 | 0.69 | 0.56 |
| PRNP | 5621 | -2.33 | 0.18 | -0.95 | 0.03 | -1.62 | -0.83 | -1.17 | -0.02 | -0.42 |
| PRNP | 5621 | 0.21 | 2.69 | 1.98 | -0.12 | 0.68 | 0.27 | 0.89 | 0.87 | 0.30 |
| S100A1 | 6271 | 3.43 | 2.02 | 4.07 | -0.32 | -0.51 | 0.18 | 1.97 | 3.29 | 1.71 |
| S100A1 | 6271 | 0.98 | 0.41 | 1.00 | -0.06 | 0.00 | 0.08 | -0.23 | 0.38 | -0.09 |
| S100A1 | 6271 | 0.70 | 1.82 | -0.76 | -0.17 | 0.61 | 1.20 | -0.12 | -0.39 | 0.66 |
| S100A1 | 6271 | -0.66 | -0.66 | -1.16 | -0.55 | -0.07 | -0.22 | -1.50 | -0.69 | -1.17 |
| S100B | 6285 | -4.97 | -2.69 | -1.93 | 0.02 | 0.07 | 0.44 | 0.42 | 0.44 | 2.06 |
| S100B | 6285 | 1.21 | 1.01 | 1.41 | 0.04 | -1.43 | 0.74 | 0.44 | -0.95 | 0.28 |
| S100B | 6285 | 0.53 | 3.14 | 0.79 | 0.08 | -0.88 | -0.49 | 0.33 | -1.36 | -0.29 |

| | | | | | | | | | | |
|---|---|---|---|---|---|---|---|---|---|---|
| S100B | 6285 | 0.53 | -0.50 | 2.10 | -0.30 | -0.01 | -0.06 | -0.53 | 0.66 | 1.18 |
| STK38 | 11329 | 4.99 | 3.17 | 2.00 | 0.82 | 1.06 | 0.90 | 1.93 | 2.29 | 2.00 |
| STK38 | 11329 | -0.03 | -1.60 | -0.35 | -0.13 | -1.01 | -1.41 | 1.68 | 1.45 | 0.51 |
| STK38 | 11329 | -2.83 | -1.39 | -4.23 | -3.10 | -1.69 | -1.72 | 0.46 | -0.85 | -0.28 |
| STK38 | 11329 | 0.63 | 3.10 | 3.50 | -1.65 | -0.37 | -0.03 | -0.09 | 0.50 | -0.33 |
| TGFBI | 7045 | -0.09 | -0.42 | 0.72 | -0.91 | -1.47 | -1.75 | 0.28 | 2.96 | 1.73 |
| TGFBI | 7045 | -0.79 | -2.38 | -0.57 | -0.03 | -0.54 | -1.12 | 1.87 | 0.02 | 2.36 |
| TGFBI | 7045 | 1.92 | 1.65 | 1.88 | 0.59 | -2.64 | -0.68 | 0.52 | 4.55 | -0.78 |
| TGFBI | 7045 | 2.21 | 1.82 | 1.06 | 0.44 | 0.38 | 0.74 | 1.57 | 1.35 | 1.94 |
| UCHL1 | 7345 | 0.04 | -0.22 | -0.81 | -0.71 | -0.67 | -1.08 | -0.35 | -0.65 | -0.52 |
| UCHL1 | 7345 | -4.73 | -3.53 | -6.77 | -4.39 | -1.70 | -4.29 | -1.48 | -1.60 | -1.39 |
| UCHL1 | 7345 | 0.18 | 0.84 | 0.80 | -1.11 | -1.66 | -2.26 | 0.01 | 0.29 | -0.39 |
| UCHL1 | 7345 | -0.48 | 0.39 | -0.75 | -0.82 | 0.55 | 0.13 | -2.27 | -2.05 | -2.35 |
| VCAM1 | 7412 | -2.32 | -1.43 | -2.38 | -0.97 | -0.30 | -0.69 | -1.73 | -1.25 | -1.77 |
| VCAM1 | 7412 | 0.21 | -0.05 | 0.55 | 0.80 | 1.01 | 0.82 | 0.40 | 1.43 | -0.47 |
| VCAM1 | 7412 | 2.19 | 2.13 | 3.58 | 1.24 | 0.47 | 0.50 | 2.52 | 2.72 | 1.24 |
| VCAM1 | 7412 | 1.37 | 2.72 | 4.79 | 0.67 | 1.32 | 0.78 | 1.01 | 1.76 | 2.01 |
| WWTR1 | 25937 | 1.34 | 1.23 | 3.25 | 1.57 | 0.66 | 1.30 | 1.60 | 0.58 | 2.20 |
| WWTR1 | 25937 | -0.91 | 0.21 | -0.90 | -0.15 | 0.03 | -0.19 | -1.64 | -1.58 | -0.74 |
| WWTR1 | 25937 | 0.31 | 2.96 | 2.90 | -2.73 | 0.40 | 1.30 | 0.73 | 1.66 | 0.88 |
| WWTR1 | 25937 | 0.00 | -0.81 | -1.28 | -0.61 | -2.91 | -2.49 | -1.03 | -0.75 | -1.43 |

## siRNA screen data for 40 "driver network member" genes from driver networks

| Gene name | Entrez.Gene.Id | ER_ZR751 ER+ | ER_ZR751 ER+ | ER_ZR751 ER+ |
|---|---|---|---|---|
| ANXA1 | 301 | -0.15 | 0.32 | 0.03 |
| ANXA1 | 301 | 1.07 | 0.19 | 0.61 |
| ANXA1 | 301 | 0.08 | -1.87 | -1.85 |
| ANXA1 | 301 | -0.26 | 0.55 | 0.22 |
| ATP1B3 | 483 | 0.86 | -0.05 | -0.05 |
| ATP1B3 | 483 | 1.73 | -0.81 | -0.32 |
| ATP1B3 | 483 | -0.12 | -1.00 | 0.17 |
| ATP1B3 | 483 | 1.82 | 0.63 | 1.58 |
| CAV2 | 858 | -0.08 | 0.49 | -0.89 |
| CAV2 | 858 | -3.60 | -1.30 | -0.03 |
| CAV2 | 858 | 0.41 | 0.13 | 0.47 |
| CAV2 | 858 | 1.50 | 0.44 | 1.47 |
| CD44 | 960 | 0.50 | 1.11 | -0.55 |
| CD44 | 960 | 0.17 | 0.18 | 0.38 |
| CD44 | 960 | 1.36 | 1.61 | 1.52 |
| CD44 | 960 | -0.14 | 1.21 | 0.69 |
| CDKN2A | 1029 | 0.20 | 1.80 | -0.69 |
| CDKN2A | 1029 | -0.65 | -0.21 | -0.93 |
| CDKN2A | 1029 | 0.93 | 1.15 | 1.13 |
| CDKN2A | 1029 | -0.83 | 0.35 | 0.70 |
| ENO1 | 2023 | 0.45 | -0.72 | -1.06 |
| ENO1 | 2023 | 0.04 | 0.40 | -0.56 |
| ENO1 | 2023 | -0.39 | 0.23 | 0.13 |
| ENO1 | 2023 | 0.50 | -0.32 | 1.24 |
| EPB41L2 | 2037 | 1.60 | -2.48 | 0.69 |
| EPB41L2 | 2037 | -0.49 | -1.25 | 1.07 |
| EPB41L2 | 2037 | 0.18 | -0.78 | -0.43 |
| EPB41L2 | 2037 | 2.50 | 2.41 | 2.66 |
| FOLR1 | 2348 | -0.26 | 0.88 | -0.31 |
| FOLR1 | 2348 | 0.32 | 0.21 | 0.69 |
| FOLR1 | 2348 | 1.49 | 0.96 | 0.57 |

| | | | | |
|---|---|---|---|---|
| FOLR1 | 2348 | -1.20 | -0.20 | -0.13 |
| ICAM1 | 3383 | -0.96 | 1.12 | 0.75 |
| ICAM1 | 3383 | 0.45 | 0.92 | -0.21 |
| ICAM1 | 3383 | -0.93 | 0.38 | 0.64 |
| ICAM1 | 3383 | 2.44 | 1.67 | 2.09 |
| IGF2BP2 | 10644 | -0.57 | 1.79 | -1.16 |
| IGF2BP2 | 10644 | 0.90 | 1.11 | 1.35 |
| IGF2BP2 | 10644 | 0.21 | 1.42 | -0.31 |
| IGF2BP2 | 10644 | 0.47 | -0.06 | -0.10 |
| IGF2BP3 | 10643 | -0.19 | -0.94 | -0.93 |
| IGF2BP3 | 10643 | -0.39 | -1.06 | -0.02 |
| IGF2BP3 | 10643 | -0.67 | -1.74 | 1.10 |
| IGF2BP3 | 10643 | 0.64 | -0.42 | 1.95 |
| IMPA2 | 3613 | -0.05 | -0.42 | 1.50 |
| IMPA2 | 3613 | 0.88 | 0.53 | 0.96 |
| IMPA2 | 3613 | 1.03 | 0.85 | 1.12 |
| IMPA2 | 3613 | 0.53 | -0.43 | 0.37 |
| KLF5 | 688 | 1.23 | 0.21 | -0.08 |
| KLF5 | 688 | 0.72 | 1.26 | -1.23 |
| KLF5 | 688 | -1.34 | -0.65 | 0.97 |
| KLF5 | 688 | 1.20 | 2.03 | 0.27 |
| LYN | 4067 | -0.53 | 1.00 | 0.27 |
| LYN | 4067 | -0.12 | 0.29 | 0.05 |
| LYN | 4067 | 2.42 | 0.32 | 1.83 |
| LYN | 4067 | 1.01 | -1.88 | 1.04 |
| MCM3 | 4172 | 0.73 | -0.96 | 0.15 |
| MCM3 | 4172 | -0.45 | 1.36 | -0.38 |
| MCM3 | 4172 | -1.33 | -0.71 | -2.17 |
| MCM3 | 4172 | 1.09 | 1.20 | 2.47 |
| MCM5 | 4174 | -1.12 | 0.44 | -0.52 |
| MCM5 | 4174 | -1.05 | 0.64 | -0.36 |
| MCM5 | 4174 | 0.49 | 0.04 | 0.53 |
| MCM5 | 4174 | 0.59 | 1.33 | 1.47 |
| MET | 4233 | 0.22 | -0.91 | 0.23 |

| | | | | |
|---|---|---|---|---|
| MET | 4233 | 0.66 | 1.46 | -0.68 |
| MET | 4233 | -2.07 | 0.17 | -0.86 |
| MET | 4233 | 1.59 | 0.29 | 0.35 |
| MSN | 4478 | 0.51 | -0.82 | 0.20 |
| MSN | 4478 | -0.42 | -1.40 | -0.14 |
| MSN | 4478 | 0.06 | 0.23 | 1.22 |
| MSN | 4478 | -0.29 | -0.26 | 1.05 |
| NCK1 | 4690 | 0.29 | -0.67 | -1.38 |
| NCK1 | 4690 | -0.18 | 0.16 | -0.02 |
| NCK1 | 4690 | -0.94 | -0.12 | 0.97 |
| NCK1 | 4690 | -0.89 | -0.46 | -0.36 |
| NDRG1 | 10397 | -0.94 | -2.85 | -0.74 |
| NDRG1 | 10397 | -1.94 | -2.93 | 0.37 |
| NDRG1 | 10397 | 0.60 | -0.14 | 0.82 |
| NDRG1 | 10397 | -0.02 | -0.77 | 0.49 |
| PLCG2 | 5336 | 0.08 | -1.59 | -0.45 |
| PLCG2 | 5336 | 0.43 | 1.17 | -0.15 |
| PLCG2 | 5336 | 0.51 | 1.18 | 0.35 |
| PLCG2 | 5336 | 1.40 | -0.85 | 0.58 |
| PRKDC | 5591 | 0.42 | -1.05 | -0.16 |
| PRKDC | 5591 | 1.23 | 0.04 | 1.47 |
| PRKDC | 5591 | 0.81 | 1.32 | 1.15 |
| PRKDC | 5591 | 0.98 | 0.99 | 0.26 |
| PRNP | 5621 | 0.72 | 0.35 | -0.40 |
| PRNP | 5621 | -0.32 | -0.62 | -0.63 |
| PRNP | 5621 | 0.73 | -0.33 | -0.02 |
| PRNP | 5621 | 0.18 | 1.60 | 0.20 |
| S100A1 | 6271 | 2.04 | 1.49 | 1.84 |
| S100A1 | 6271 | 1.33 | 0.49 | 0.06 |
| S100A1 | 6271 | -0.27 | 1.06 | -0.38 |
| S100A1 | 6271 | 0.63 | -1.00 | -0.28 |
| S100B | 6285 | 0.09 | -1.49 | 1.35 |
| S100B | 6285 | 1.79 | 1.18 | -0.03 |
| S100B | 6285 | -0.27 | 0.34 | -0.03 |

| | | | | |
|---|---|---|---|---|
| S100B | 6285 | 0.29 | 0.80 | 0.47 |
| STK38 | 11329 | 3.68 | 0.28 | 0.20 |
| STK38 | 11329 | 0.49 | -0.67 | 0.02 |
| STK38 | 11329 | -0.68 | 1.12 | -0.24 |
| STK38 | 11329 | -0.73 | 1.85 | 0.74 |
| TGFBI | 7045 | -0.45 | 0.10 | -0.83 |
| TGFBI | 7045 | 0.09 | -0.16 | -0.06 |
| TGFBI | 7045 | -0.55 | 0.83 | -0.63 |
| TGFBI | 7045 | 1.30 | 0.32 | 0.44 |
| UCHL1 | 7345 | 0.86 | -0.17 | 0.98 |
| UCHL1 | 7345 | -2.37 | -1.65 | -1.79 |
| UCHL1 | 7345 | -0.29 | -0.29 | -0.58 |
| UCHL1 | 7345 | -1.16 | -0.94 | -0.13 |
| VCAM1 | 7412 | 0.20 | -1.66 | -0.72 |
| VCAM1 | 7412 | -0.22 | 0.31 | -0.12 |
| VCAM1 | 7412 | 1.18 | 1.88 | 0.52 |
| VCAM1 | 7412 | 1.29 | 0.79 | 2.70 |
| WWTR1 | 25937 | 0.52 | -1.31 | 1.43 |
| WWTR1 | 25937 | -0.28 | -1.91 | -0.68 |
| WWTR1 | 25937 | 1.14 | -0.12 | 1.31 |
| WWTR1 | 25937 | -1.16 | 0.04 | -0.01 |
