# Supplementary material for: A network-based, integrative study to identify core biological pathways that drive breast cancer clinical subtypes
Source: Br J Cancer. 2012 Feb 16;106(6):1107–16. doi: 10.1038/bjc.2011.584 (PMC3304402; doi:10.1038/bjc.2011.584)
Supplement: Supplementary Table S5 [file bjc2011584x8.pdf]

Differentially expressed genes for ER+ subtype from Chin et al.	
Gene ID	Frequency of appearance in the differentially expressed gene list from resampling (min = 0, max = 1, cutoff = 0.5)
9	1.00
12	0.99
36	0.80
115	1.00
164	1.00
210	0.78
222	0.63
323	0.91
367	1.00
374	0.93
377	1.00
388	1.00
403	0.64
517	0.93
563	0.87
576	0.71
585	0.51
596	1.00
644	0.64
689	1.00
720	1.00
721	1.00
771	1.00
776	1.00
901	0.68
987	1.00
1101	0.69
1287	0.53
1311	0.79
1359	0.80
1360	1.00
1382	0.52
1396	1.00
1471	0.76
1528	0.99
1555	1.00
1556	1.00
1580	0.57
1602	1.00
1725	0.50

1846	1.00
1917	1.00
1955	0.74
2065	1.00
2099	1.00
2166	0.87
2203	1.00
2593	0.82
2625	1.00
2752	0.52
2800	0.51
2922	0.77
2947	1.00
3029	0.98
3169	1.00
3212	1.00
3226	0.71
3249	0.99
3295	0.53
3315	0.76
3480	1.00
3485	0.98
3572	0.83
3667	0.72
3693	0.66
3800	0.87
3875	1.00
4059	0.90
4137	1.00
4250	1.00
4602	1.00
4680	0.99
4832	1.00
4886	1.00
5046	0.78
5104	0.97
5167	0.93
5174	0.56
5304	0.91
5327	0.94
5507	1.00
5914	0.98
5979	0.76
6337	1.00
6451	0.81
6505	0.79
6584	1.00

6659	0.96
6697	0.53
6922	1.00
6926	1.00
7009	0.81
7021	0.71
7031	1.00
7033	1.00
7078	0.68
7162	1.00
7357	0.79
7466	1.00
7494	1.00
7507	0.80
7802	1.00
7832	0.91
7844	1.00
7869	0.99
8309	1.00
8382	0.85
8416	1.00
8576	0.78
8614	1.00
8786	0.96
8821	1.00
9052	0.82
9338	1.00
9368	0.64
9501	0.60
9518	0.98
9620	1.00
9633	1.00
9649	1.00
9658	0.99
9687	1.00
9742	0.99
9743	0.55
10040	0.87
10103	1.00
10229	0.55
10234	1.00
10439	0.77
10451	1.00
10512	0.89
10529	0.55
10548	0.55
10551	1.00

10560	0.97
10641	0.85
10647	0.98
10916	1.00
10935	0.51
10974	0.87
11042	0.90
11059	1.00
11145	1.00
11226	1.00
22796	0.77
22836	0.70
22977	0.85
22996	1.00
23030	1.00
23116	0.61
23158	1.00
23171	0.66
23263	0.69
23362	0.97
23428	1.00
23639	0.60
25800	1.00
25803	1.00
25837	0.98
27134	1.00
27324	0.95
29956	0.98
50810	0.61
51097	1.00
51133	0.98
51322	0.79
51340	0.94
51363	0.55
51380	0.63
51466	0.95
51477	0.77
51604	0.94
51760	0.81
51809	1.00
54458	0.95
54463	0.99
54502	1.00
54585	0.58
54837	1.00
54847	0.99
54898	1.00

54961	0.90
55358	0.80
55450	1.00
55638	0.99
55686	1.00
55733	1.00
55793	1.00
55837	0.70
55861	0.59
56521	1.00
56904	0.94
57416	1.00
57496	1.00
57535	0.89
57613	1.00
57728	0.63
57758	1.00
57763	0.82
60481	0.93
63941	0.99
64084	1.00
64087	1.00
64284	0.82
64847	0.62
65055	0.74
79006	0.90
79083	1.00
79178	0.92
79600	1.00
79603	1.00
79624	1.00
79641	0.77
79702	1.00
79838	1.00
79846	0.65
79921	0.96
80127	0.99
80129	0.56
80736	1.00
81031	0.97
84745	0.91
89927	0.88
90355	1.00

Differentially expressed genes for HER2+ subtype from Chin et al.	
Gene ID	Frequency of appearance in the differentially expressed gene list from resampling (min = 0, max = 1, cutoff = 0.5)
15	0.65
43	0.59
133	0.72
183	0.71
208	0.53
244	1.00
247	0.70
489	0.58
667	0.65
677	0.55
785	0.54
909	0.62
917	0.50
1000	0.60
1001	0.95
1004	0.58
1043	0.64
1087	0.60
1130	0.53
1159	1.00
1191	0.70
1285	0.82
1305	0.71
1384	0.97
1474	1.00
1525	0.51
1672	1.00
1755	0.53
1824	0.50
1846	1.00
1848	0.77
1951	0.97
1953	0.75
2064	1.00
2201	0.72
2264	1.00
2334	0.67
2525	0.58
2537	0.72

2568	0.86
2591	0.71
2675	0.63
2742	0.98
2867	0.53
2886	1.00
3091	0.72
3126	0.62
3216	0.57
3237	0.53
3263	0.96
3269	0.51
3320	0.99
3429	0.70
3434	0.51
3447	0.52
3622	0.73
3669	0.62
3783	0.66
3846	0.70
3854	0.73
3855	0.64
3861	0.67
3866	0.51
3872	1.00
3887	0.71
3929	0.77
4082	0.99
4199	0.77
4246	0.95
4249	0.61
4250	1.00
4281	0.68
4312	0.99
4316	1.00
4586	0.71
4587	0.72
4588	0.53
4680	0.96
4761	0.71
4938	0.69
4974	0.73
4988	0.60
5140	0.60
5178	0.72
5304	0.70
5307	0.57

5409	1.00
5460	0.53
5502	0.82
5600	0.59
5625	1.00
5691	0.52
5697	0.72
5709	1.00
5745	0.63
5764	0.71
5790	0.72
5898	0.73
5918	0.81
6196	0.54
6238	0.91
6273	0.72
6278	1.00
6279	1.00
6280	1.00
6286	0.62
6332	0.54
6352	0.62
6372	0.66
6550	0.58
6558	0.89
6590	0.95
6599	0.69
6664	1.00
6752	0.68
6965	0.70
7007	0.54
7021	1.00
7033	1.00
7067	1.00
7258	0.72
7262	0.55
7264	0.79
7703	0.59
7782	0.62
8309	0.69
8396	0.55
8496	0.54
8537	0.70
8557	0.97
8564	1.00
8626	0.68
8638	0.68

8842	0.97
8909	0.54
8997	0.61
9022	0.72
9060	1.00
9168	0.63
9210	0.70
9220	0.71
9248	1.00
9334	0.70
9415	1.00
9480	0.62
9572	0.69
9635	1.00
9636	0.72
9669	0.54
9749	0.89
9751	0.71
9862	1.00
10125	0.60
10200	0.62
10249	0.84
10321	0.77
10487	0.99
10587	0.73
10647	1.00
10686	0.70
10749	0.70
10948	1.00
11013	0.79
11042	0.51
22794	0.73
22943	0.71
23017	0.85
23034	0.70
23037	0.73
23075	0.84
23293	0.81
23326	0.50
23475	0.98
23591	0.63
23650	0.78
25803	0.81
26045	0.51
26118	0.71
26136	0.66
26499	0.95

26576	0.72
27074	0.57
29053	0.58
29091	0.73
30850	0.70
51685	0.58
51806	1.00
51809	0.69
54531	0.50
54544	0.63
54798	0.65
54869	0.56
54883	0.57
54972	0.61
55008	0.81
55287	0.64
55568	0.76
55738	0.62
55852	0.55
55876	1.00
57490	0.54
64816	0.56
64922	0.55
65055	0.71
79152	1.00
79412	0.94
79615	0.72
79762	0.62
79802	0.71
79820	0.60
79919	0.95
80156	0.77
83481	0.70
84227	0.66
91543	0.70
93210	1.00
94009	1.00
112399	0.55
154796	0.55

Differentially expressed genes for TN subtype from Chin et al.	
Gene ID	Frequency of appearance in the differentially expressed gene list from resampling (min = 0, max = 1, cutoff = 0.5)
244	1.00
262	1.00
330	0.72
419	0.89
483	0.76
688	0.99
864	1.00
898	0.74
991	1.00
1001	1.00
1029	0.93
1054	0.94
1116	1.00
1117	1.00
1381	1.00
1410	1.00
1439	1.00
1466	0.71
1475	0.98
1503	0.56
1515	0.81
1825	0.97
1830	0.63
1871	0.76
1956	1.00
2001	1.00
2019	1.00
2023	0.82
2049	0.97
2051	0.76
2120	1.00
2173	1.00
2175	0.65
2178	0.63
2348	0.92
2564	0.55
2568	1.00

2633	0.88
2824	1.00
2921	0.56
2950	0.79
3112	0.61
3117	1.00
3126	0.57
3159	1.00
3303	0.94
3383	0.67
3400	1.00
3455	1.00
3502	1.00
3507	0.95
3514	1.00
3575	0.84
3595	0.74
3613	0.95
3620	0.78
3627	0.84
3755	0.81
3783	0.97
3852	1.00
3854	1.00
3861	0.99
3866	1.00
3868	1.00
3872	1.00
3914	0.59
3930	0.86
3934	0.82
3945	1.00
4050	0.71
4065	0.71
4067	1.00
4071	0.85
4162	1.00
4166	0.99
4172	0.59
4174	1.00
4233	0.62
4240	0.67
4281	1.00
4283	1.00
4312	1.00
4316	1.00
4318	0.97

4321	0.98
4478	0.91
4603	1.00
4687	0.59
4690	0.73
4771	0.84
4781	1.00
4794	0.88
4904	0.96
4953	1.00
5268	1.00
5321	1.00
5332	1.00
5336	0.99
5450	1.00
5613	1.00
5621	0.65
5788	0.56
5806	0.96
5918	1.00
5971	0.85
6271	0.94
6273	0.62
6285	1.00
6289	0.67
6305	0.93
6352	1.00
6362	0.94
6363	0.92
6422	1.00
6489	0.76
6590	0.97
6648	1.00
6663	1.00
6850	0.77
7039	0.86
7345	0.69
7368	1.00
7412	0.84
7525	0.66
7545	1.00
7913	0.76
8140	0.90
8190	1.00
8317	0.86
8543	1.00
8566	0.79

8645	1.00
8685	0.73
8690	0.98
8836	0.69
8842	1.00
9061	0.71
9095	1.00
9200	0.60
9235	0.92
9319	0.76
9435	1.00
9466	0.53
9469	1.00
9568	0.57
9582	1.00
9654	0.89
9688	0.64
9806	1.00
10155	1.00
10232	0.98
10397	0.94
10402	0.71
10479	0.70
10537	1.00
10563	1.00
10568	0.63
10625	0.61
10643	1.00
10644	0.65
10659	0.54
10892	0.84
10950	0.99
11013	0.83
11135	0.80
11329	0.98
11341	0.98
22808	0.99
22822	0.77
23175	1.00
23229	0.91
23231	1.00
23321	1.00
23424	0.90
23650	1.00
23683	0.99
25907	0.98
25937	0.94

25984	1.00
26031	0.90
26227	0.99
27074	1.00
27299	1.00
28831	0.97
29015	1.00
29842	0.69
29968	0.99
29970	0.82
51026	0.96
51237	0.51
51312	0.86
51442	1.00
51704	0.93
51806	1.00
53335	1.00
54149	0.99
54535	0.96
54763	1.00
54781	0.89
55003	0.90
55765	0.55
55816	0.73
55872	0.58
56603	0.74
56833	0.88
56935	1.00
57110	0.53
57348	1.00
58473	0.64
58528	0.89
60683	0.92
64771	0.91
79098	0.99
79627	1.00
79817	0.97
80183	0.54
81553	0.99
81611	0.99
81618	0.69
83439	1.00
84535	0.90
84752	0.66
85377	1.00
85453	0.86
94025	1.00

140885	0.68

Differentially expressed genes for ER+ subtype from Andre et al.	
Gene ID	Frequency of appearance in the differentially expressed gene list from resampling (min = 0, max = 1, cutoff = 0.5)
9	1.00
12	0.94
18	0.84
36	0.95
115	0.97
164	1.00
214	0.98
222	0.98
323	1.00
367	1.00
373	0.66
374	1.00
377	0.97
388	1.00
403	0.97
585	0.60
595	1.00
596	0.58
598	1.00
689	0.53
720	0.99
721	0.98
771	1.00
819	0.93
987	0.77
1101	0.86
1311	0.91
1345	0.99
1360	0.60
1363	0.66
1396	1.00
1471	0.88
1528	0.96
1555	1.00
1556	1.00
1580	1.00
1602	1.00
1728	0.96
1846	0.89
1917	0.99

1952	0.89
1955	0.90
2065	0.98
2099	1.00
2166	1.00
2263	0.54
2268	0.82
2593	1.00
2625	1.00
2647	0.72
2674	0.99
2697	0.94
2743	1.00
2752	0.92
2804	0.60
2952	0.96
3081	1.00
3169	1.00
3480	1.00
3485	0.95
3487	1.00
3551	0.86
3572	1.00
3642	0.54
3667	0.99
3693	0.70
3875	0.81
3899	1.00
3909	0.70
3964	1.00
4059	0.99
4137	1.00
4179	1.00
4246	1.00
4250	1.00
4254	0.57
4582	0.93
4602	0.79
4646	0.98
4680	0.80
4886	0.99
5002	0.86
5025	0.99
5104	1.00
5167	1.00
5174	0.95
5241	0.97

5304	1.00
5327	0.94
5364	0.99
5441	0.56
5525	0.92
5538	0.99
5733	0.65
5914	0.76
5979	1.00
6038	0.75
6319	0.98
6337	1.00
6405	0.97
6500	0.99
6542	1.00
6604	0.54
6659	0.61
6922	1.00
6926	0.99
7009	1.00
7021	0.81
7031	1.00
7033	1.00
7090	0.84
7157	0.82
7162	1.00
7257	0.58
7320	1.00
7357	1.00
7494	1.00
7572	0.62
7593	0.93
7802	1.00
7844	0.78
7869	1.00
7905	0.97
8073	0.62
8228	0.70
8363	0.84
8405	0.66
8416	1.00
8553	0.50
8614	1.00
8678	1.00
8722	0.96
8794	0.53
8821	0.98

8996	0.68
9052	0.84
9120	1.00
9135	1.00
9187	0.60
9254	0.75
9338	0.98
9498	0.58
9518	0.96
9620	0.98
9674	1.00
9687	1.00
9778	1.00
9802	0.76
9915	0.76
10103	1.00
10202	0.63
10324	0.75
10439	1.00
10451	1.00
10512	0.80
10551	1.00
10560	0.62
10610	0.57
10614	0.54
10647	1.00
10677	0.86
10847	0.52
10916	1.00
10965	0.63
10974	1.00
11030	0.85
11042	1.00
11059	0.94
11160	0.52
11226	0.74
22885	0.99
22906	0.75
22924	0.84
22977	0.99
22996	1.00
23027	0.51
23030	0.94
23158	1.00
23389	1.00
23428	1.00
23528	0.97

25800	1.00
25803	0.99
25992	0.65
26018	0.58
26234	0.95
27075	1.00
27134	1.00
27324	0.71
28958	0.87
29956	1.00
51097	1.00
51133	0.97
51313	0.99
51322	0.74
51340	0.96
51363	0.57
51364	1.00
51380	0.99
51409	0.55
51466	0.93
51643	1.00
51706	1.00
51809	0.90
54361	0.97
54502	0.98
54795	0.72
54837	1.00
54898	1.00
54961	0.86
55040	0.67
55107	0.52
55194	0.98
55450	0.97
55638	0.71
55673	0.99
55686	0.99
55712	0.91
55764	0.99
55930	0.52
56521	1.00
56654	0.57
57045	0.67
57241	0.55
57416	1.00
57419	0.74
57496	1.00
57535	0.96

57613	1.00
57728	0.65
57758	1.00
57804	0.51
60481	0.87
60598	1.00
63931	0.99
64084	1.00
64769	0.51
64788	0.81
65055	0.59
79006	1.00
79025	0.51
79083	1.00
79137	0.69
79269	0.75
79570	0.91
79624	1.00
79702	1.00
80129	0.81
80736	1.00
84179	0.77
90355	1.00
92579	0.59

Differentially expressed genes for HER2+ subtype from Andre et al.	
Gene ID	Frequency of appearance in the differentially expressed gene list from resampling (min = 0, max = 1, cutoff = 0.5)
220	0.65
222	1.00
229	0.87
412	0.75
463	0.53
489	0.70
825	0.51
1056	0.62
1066	0.82
1123	0.50
1384	1.00
1442	0.83
1443	0.85
1444	0.51
1773	0.54
1805	0.88
1848	1.00
1910	0.59
1951	0.67
2064	1.00
2108	0.61
2264	0.53
2688	0.81
2870	0.55
2886	1.00
3117	1.00
3119	0.57
3263	0.56
3303	1.00
3357	0.56
3399	0.76
3418	0.59
3419	0.63
3502	1.00
3507	0.78
3514	1.00
3669	0.72
3755	0.56
3768	0.92
3784	0.52

3855	0.71
3887	1.00
3927	1.00
3949	0.75
4250	0.57
4294	0.94
4324	0.88
4613	1.00
4680	0.89
4849	0.88
4926	0.56
5001	0.79
5029	0.78
5130	0.90
5277	0.79
5304	0.69
5346	0.74
5371	0.78
5408	0.56
5409	1.00
5469	1.00
5514	0.95
5691	0.99
5709	1.00
5717	0.94
5741	0.84
5918	0.85
5988	0.75
6278	0.86
6319	0.50
6440	0.61
6522	0.74
6549	1.00
6636	0.88
6664	0.81
6857	0.82
7021	1.00
7031	0.51
7123	1.00
7177	0.58
7204	0.69
7329	0.75
7703	0.64
8273	0.53
8358	0.55
8396	0.96
8418	0.72

8557	0.74
8564	0.77
8659	0.97
8714	0.97
8773	0.52
8776	0.88
8996	0.64
9031	0.64
9220	0.86
9235	0.68
9517	0.57
9635	1.00
9749	0.82
9751	0.58
9862	0.85
10093	0.85
10211	0.55
10321	0.89
10563	0.99
10612	0.51
10647	0.51
10677	0.78
10863	0.53
10938	0.83
10948	1.00
11063	0.84
11283	0.91
11322	0.84
22794	0.65
22904	0.60
23037	0.85
23075	0.62
23089	0.76
23106	1.00
23129	0.61
23289	0.96
23359	0.73
23462	0.53
23508	1.00
23600	0.87
26047	0.94
26051	0.52
26207	0.54
26236	0.73
27318	0.92
27324	0.97
28831	0.99

29760	0.66
29844	0.53
30844	0.72
30850	0.93
50512	0.73
51237	1.00
51284	0.63
51755	0.95
51760	0.98
51806	0.94
53353	0.53
54187	0.74
54490	0.96
54795	0.82
55223	0.73
55673	0.62
55794	0.51
55876	1.00
55890	0.94
57099	0.63
57175	0.64
57485	0.69
79095	0.57
79152	0.80
79695	0.53
79838	0.98
79901	0.56
80237	0.70
93210	1.00
94009	0.95
114884	1.00

Differentially expressed genes for TN subtype from Andre et al.	
Gene ID	Frequency of appearance in the differentially expressed gene list from resampling (min = 0, max = 1, cutoff = 0.5)
119	0.72
123	0.73
204	0.79
301	0.56
419	0.91
445	1.00
800	0.52
827	0.61
871	0.95
1001	1.00
1029	1.00
1116	0.92
1117	1.00
1284	0.95
1299	0.99
1356	0.82
1381	0.95
1410	0.99
1466	1.00
1515	0.88
1612	1.00
1824	0.96
1956	1.00
2001	1.00
2019	1.00
2037	0.76
2139	0.56
2171	0.82
2173	0.95
2346	0.63
2558	0.81
2568	1.00
2619	1.00
2633	0.99
2707	1.00
2824	1.00
2950	1.00
3112	1.00
3126	0.52
3383	0.66

3400	1.00
3502	0.60
3507	0.73
3595	0.81
3627	0.93
3852	1.00
3854	1.00
3861	0.93
3868	1.00
3872	1.00
3928	1.00
4057	0.53
4068	0.63
4071	0.51
4162	0.99
4174	1.00
4233	0.99
4240	0.57
4281	0.99
4288	0.83
4316	1.00
4439	0.83
4478	0.59
4626	0.72
4781	1.00
4919	1.00
5214	0.58
5268	1.00
5294	0.61
5325	0.93
5329	0.55
5358	0.92
5613	1.00
5621	0.77
5653	0.85
5806	0.54
5918	1.00
5962	0.92
6273	0.53
6278	0.51
6289	0.99
6347	0.99
6368	0.64
6373	0.95
6422	1.00
6489	0.59
6491	0.97

6518	0.61
6564	0.56
6566	0.51
6624	0.94
6663	0.99
6664	0.79
6732	0.61
6829	1.00
7368	1.00
7545	1.00
7804	0.68
7850	0.73
7851	1.00
8061	0.59
8190	1.00
8208	0.99
8317	0.98
8581	0.90
8645	1.00
8690	0.93
8767	0.77
8796	0.61
8842	1.00
9235	0.82
9401	0.56
9466	0.93
9469	0.99
9603	0.54
9654	1.00
9688	0.78
9833	0.55
9933	0.96
10155	0.58
10397	0.99
10469	0.68
10516	0.58
10537	0.80
10563	0.74
10644	0.98
10656	0.91
10855	0.83
10874	1.00
10876	0.55
10950	0.92
10964	0.71
11004	0.93
11013	1.00

11182	0.63
11254	1.00
11341	0.99
22873	0.82
22974	0.97
23007	0.63
23175	1.00
23216	0.51
23266	0.78
23287	0.75
23321	0.97
23397	0.95
23432	0.69
23507	0.86
23650	1.00
23683	0.98
24149	0.88
25870	1.00
25907	1.00
25928	0.68
25937	1.00
25984	0.90
26227	0.93
27074	1.00
27234	0.97
28831	0.69
29015	1.00
29899	0.52
29967	0.56
29968	0.98
51029	0.70
51442	1.00
51806	1.00
53335	1.00
54763	0.87
54933	0.96
54954	0.99
55121	0.52
55765	0.64
55839	0.91
55975	0.90
56935	1.00
57820	0.54
63934	0.96
64778	0.60
65987	0.88
79156	0.96

79605	0.97
79627	0.52
79709	1.00
79890	0.64
81557	0.59
81611	0.62
81618	0.94
83439	0.83
84752	0.85
85377	1.00
94025	0.64

**Differentially expressed genes for ER+ subtype from Neve et al.**	
Gene ID	Frequency of appearance in the differentially expressed gene list from resampling (min = 0, max = 1, cutoff = 0.5)
18	0.83
21	1.00
164	1.00
217	0.62
222	0.92
347	0.93
377	0.90
388	1.00
489	0.79
517	0.68
684	0.98
771	1.00
845	0.59
999	1.00
1041	0.74
1159	0.97
1191	0.91
1350	0.97
1363	1.00
1365	1.00
1374	0.98
1382	0.96
1396	1.00
1428	0.53
1509	0.80
1528	0.69
1534	0.99
1602	0.85
1917	0.90
1975	0.70
2013	0.75
2017	0.97
2029	0.99
2065	0.76
2099	1.00
2203	1.00
2264	0.86
2625	1.00
2690	0.51
2947	0.83

3017	0.84
3131	0.50
3169	1.00
3315	0.65
3382	0.85
3398	0.65
3485	1.00
3487	0.68
3625	0.92
3856	0.83
3875	0.82
3880	0.79
3964	0.78
4137	0.72
4147	0.66
4250	0.98
4329	1.00
4488	0.89
4582	1.00
4602	0.78
4666	0.52
4671	0.80
4925	0.76
5025	0.54
5053	0.58
5087	0.97
5094	0.99
5204	0.68
5265	0.56
5367	0.52
5379	0.58
5412	1.00
5571	0.58
5603	0.57
5625	0.89
5710	0.55
6199	1.00
6302	0.68
6414	1.00
6478	0.92
6506	0.62
6509	0.76
6833	0.67
7009	0.68
7031	1.00
7033	1.00
7057	0.50

7108	0.86
7137	0.89
7227	0.98
7466	0.59
7494	1.00
7905	1.00
8161	0.64
8416	0.98
8500	0.95
8932	0.93
8991	1.00
9022	0.82
9060	0.55
9368	1.00
9482	0.75
9517	0.78
9529	0.68
9619	0.97
9674	0.61
9687	1.00
9716	0.60
9848	0.87
9865	0.75
9887	0.64
9919	0.59
10023	0.72
10040	0.55
10094	0.74
10102	1.00
10106	1.00
10140	1.00
10265	0.61
10388	0.60
10439	0.93
10512	0.99
10529	1.00
10551	1.00
10610	1.00
10647	0.56
10677	0.98
10899	0.99
10956	0.79
10974	1.00
11122	0.50
11153	0.89
11160	0.81
11212	0.64

11226	1.00
22941	1.00
22992	1.00
22996	1.00
23041	1.00
23171	0.62
23185	0.66
23199	0.97
23242	0.76
23329	0.51
23339	0.67
23389	0.93
23522	0.94
23623	0.71
25800	0.99
25803	1.00
25924	0.66
26353	1.00
26471	0.50
27075	0.69
27134	0.56
27250	0.99
27324	0.87
29108	1.00
29760	0.59
29905	0.70
29956	0.68
29984	0.99
51015	0.60
51322	0.74
51644	0.72
54716	0.51
54794	0.88
54837	1.00
54869	0.74
54898	0.56
54961	0.92
55040	0.71
55291	0.98
55793	1.00
55930	0.93
56654	0.99
56967	0.53
57135	0.68
57402	0.95
57415	0.89
57416	1.00

57419	0.75
57535	0.50
57663	0.58
64084	0.71
64757	1.00
79083	0.98
79170	0.57
79624	0.99
79666	1.00
79762	0.53
79767	0.59
79961	1.00
79962	0.94
80129	0.61
80134	0.65
80194	0.99
80223	1.00
80237	0.90
80303	0.90
80319	0.54
81031	1.00
84861	0.89
92595	0.73

Differentially expressed genes for HER2+ subtype from Neve et al.	
Gene ID	Frequency of appearance in the differentially expressed gene list from resampling (min = 0, max = 1, cutoff = 0.5)
217	1.00
222	1.00
226	0.65
230	1.00
388	0.83
397	0.51
489	1.00
563	1.00
934	1.00
999	1.00
1081	0.93
1114	0.56
1191	0.85
1201	1.00
1237	1.00
1298	0.97
1362	1.00
1363	1.00
1365	1.00
1397	0.58
1509	0.60
1543	0.76
1846	1.00
1848	0.73
1917	0.58
1942	0.54
1958	0.61
2052	0.56
2064	1.00
2065	0.52
2139	0.59
2179	0.62
2180	0.91
2203	0.93
2264	0.52
2539	0.99
2567	0.74
2678	0.88
2679	0.74
2752	0.51

2760	0.54
2778	0.68
2878	0.74
2886	1.00
2939	0.53
2940	0.62
2947	0.87
3169	0.90
3485	0.59
3625	0.80
3689	0.70
3851	0.74
3855	0.98
3856	0.77
3866	0.99
3875	0.72
3887	0.77
3934	0.73
3985	0.54
4102	0.72
4118	0.83
4151	0.89
4250	1.00
4256	0.99
4282	0.65
4301	0.91
4324	0.50
4477	0.68
4488	0.96
4582	0.64
4680	1.00
4689	0.59
4781	0.54
4891	0.81
5087	0.57
5127	0.75
5349	0.52
5447	0.97
5469	0.98
5569	0.88
5580	0.71
5625	0.74
5652	0.84
5709	0.69
5792	0.79
5909	0.88
5914	0.63

5920	0.72
6143	1.00
6185	0.73
6279	0.97
6280	1.00
6286	0.97
6303	0.57
6337	0.94
6376	0.77
6388	0.96
6414	0.53
6480	0.96
6576	0.52
6653	1.00
6830	1.00
6965	0.74
7031	1.00
7033	0.90
7074	0.51
7108	0.69
7126	1.00
7163	0.72
7351	0.98
7422	0.64
7442	0.70
7494	0.97
7728	0.57
7869	0.61
7904	0.96
7975	0.57
8418	0.62
8495	0.99
8557	0.77
8564	0.94
8635	0.99
8714	0.69
8800	0.63
8991	0.80
8997	0.68
9022	1.00
9121	0.82
9123	0.91
9145	0.51
9220	1.00
9518	1.00
9618	1.00
9681	0.98

9703	1.00
9709	0.70
9848	0.75
9862	0.63
9865	0.83
9907	0.79
10140	0.69
10202	1.00
10214	0.58
10221	0.51
10397	0.95
10454	0.92
10457	0.94
10529	0.72
10551	1.00
10647	0.74
10948	1.00
10974	0.98
11013	0.61
11322	0.62
22834	0.72
22996	0.94
22998	0.71
23089	0.77
23106	1.00
23130	0.76
23171	0.58
23242	1.00
23381	0.66
23475	0.93
23650	0.72
23705	0.74
25803	0.99
25805	0.50
25984	0.99
26073	1.00
26088	0.66
26118	0.87
26173	0.78
26268	1.00
26284	1.00
27102	0.92
27156	0.63
27346	0.71
28957	0.54
28992	0.63
29796	0.55

29923	0.57
50617	0.74
51314	0.57
51442	0.75
51755	1.00
51760	0.89
53840	1.00
54490	0.54
54795	0.63
54972	0.74
55090	0.98
55107	1.00
55187	0.85
55223	0.96
55412	1.00
55450	0.77
55611	0.55
55655	0.71
55731	0.95
55876	1.00
55911	0.81
55977	1.00
56834	0.95
57235	0.63
63970	0.77
64063	1.00
64788	0.56
64795	0.74
64856	0.74
65986	0.59
79170	0.71
79679	0.96
79820	0.95
79885	0.73
79888	0.93
79919	0.80
79932	0.68
80024	0.87
80221	0.94
80303	0.97
80344	0.73
80352	0.58
81031	0.92
81554	0.65
83451	0.69
91227	0.95
92086	0.59

93210	1.00
112399	0.99

Differentially expressed genes for TN subtype from Neve et al.	
Gene ID	Frequency of appearance in the differentially expressed gene list from resampling (min = 0, max = 1, cutoff = 0.5)
136	0.94
182	1.00
220	0.66
231	1.00
244	0.89
301	1.00
558	0.92
800	1.00
822	0.89
857	1.00
858	1.00
960	1.00
991	0.72
1052	1.00
1075	0.89
1466	0.95
1490	0.58
1645	1.00
1646	1.00
1647	0.65
1806	1.00
1948	0.76
1956	1.00
1969	0.92
1984	1.00
2004	1.00
2012	1.00
2014	1.00
2119	0.51
2131	0.87
2171	1.00
2182	0.96
2202	0.66
2335	0.99
2512	0.59
2618	0.80
2744	1.00
2791	0.51
2876	0.93
2908	0.87

2950	1.00
2963	0.99
3105	0.61
3189	0.93
3371	0.81
3400	0.57
3428	0.92
3486	1.00
3490	1.00
3628	0.67
3685	0.58
3852	0.69
3945	1.00
3956	1.00
3992	0.63
4017	1.00
4067	0.96
4071	1.00
4082	0.97
4102	0.81
4154	0.82
4162	1.00
4166	1.00
4233	1.00
4277	0.53
4478	1.00
4501	0.78
4502	0.59
4664	0.78
4678	1.00
4837	1.00
4863	0.73
4907	0.70
5328	0.66
5358	1.00
5366	0.62
5378	0.78
5533	0.69
5621	1.00
5698	0.81
5793	0.91
5965	0.71
6273	1.00
6443	0.98
6446	1.00
6566	1.00
6590	0.73

6591	0.93
6624	0.81
6646	0.79
6648	0.70
6672	1.00
6678	0.62
6732	0.70
6764	0.79
7040	0.99
7045	1.00
7048	1.00
7091	0.84
7169	1.00
7204	0.99
7378	1.00
7389	0.97
7431	1.00
7545	0.92
7846	0.79
7980	0.69
8061	1.00
8519	0.93
8540	1.00
8572	0.79
8644	0.93
8727	0.88
8781	0.66
8829	0.96
8898	1.00
9260	0.57
9263	0.86
9322	1.00
9448	0.97
9469	0.94
9532	1.00
9636	0.86
9702	0.73
9859	0.91
10019	0.66
10123	0.96
10147	0.93
10179	1.00
10186	0.52
10253	0.78
10398	0.63
10403	0.98
10409	0.72

10410	0.72
10473	0.97
10602	0.94
10643	1.00
10644	1.00
10728	1.00
10946	1.00
10950	0.89
10979	0.98
11050	0.91
11080	0.60
11118	0.54
11167	1.00
11217	1.00
22800	0.89
22822	1.00
22873	0.86
22939	1.00
22943	0.85
23157	1.00
23393	0.87
23516	0.71
23530	0.73
23543	1.00
23548	0.65
23657	1.00
23764	0.61
25870	1.00
25932	1.00
25996	0.71
26009	0.61
26064	0.91
26872	0.90
27347	0.67
29015	0.86
29940	1.00
29968	1.00
29980	0.82
51170	0.56
51186	1.00
51232	1.00
53827	0.99
54933	0.58
55035	0.94
55071	0.74
55076	1.00
55214	1.00

55326	0.66
55355	0.55
55589	0.62
55621	0.73
55700	0.54
55862	1.00
55975	0.86
56034	1.00
56912	1.00
64208	0.92
64778	0.84
79791	0.54
79890	1.00
79912	0.57
79971	0.64
84168	1.00
84617	1.00
84752	1.00
85453	1.00
113146	1.00
116496	0.98
140597	0.52
