# Supplementary material for: A network-based, integrative study to identify core biological pathways that drive breast cancer clinical subtypes
Source: Br J Cancer. 2012 Feb 16;106(6):1107–16. doi: 10.1038/bjc.2011.584 (PMC3304402; doi:10.1038/bjc.2011.584)
Supplement: Supplementary Table S6 [file bjc2011584x9.pdf]

Supplemental Table 6: Comparison of driver networks from Chin et al. seed genes. Overlap of the driver networks created from Chin et al. gene expression dataset using seed genes from Chin et al. and Andre et al.

	**ER+**	**HER2+**	**TN**
Chin et al. seed and Chin et al. expression	33	11	40
Overlap with Andre et al. seed and Chin et al. expression	30	8	36
