# Supplementary material for: A network-based, integrative study to identify core biological pathways that drive breast cancer clinical subtypes
Source: Br J Cancer. 2012 Feb 16;106(6):1107–16. doi: 10.1038/bjc.2011.584 (PMC3304402; doi:10.1038/bjc.2011.584)
Supplement: Supplementary Data [file bjc2011584x10.doc]

**Supplementary Information**

**A network-based, integrative study to identify core biological pathways that drive breast cancer clinical subtypes**

Bhaskar Dutta^1^, Lajos Pusztai^2*^, Yuan Qi^3^, Fabrice Andre^4^, Vladimir Lazar^4^, Giampaolo Bianchini^2^, Naoto Ueno^2^, Roshan Agarwal^5^, Bailiang Wang^2^, Christine Y Shiang^2^, Gabriel N Hortobagyi^2^, Gordon B Mills^1^, W Fraser Symmans^5^, Gábor Balázsi^1*^

Departments of ^1^Systems Biology, ^2^Breast Medical Oncology, ^3^Biostatistics and ^5^Pathology of The University of Texas MD Anderson Cancer Center, Houston, TX the ^4^Institute Gustave Roussy, Villejuif, France and the ^5^Department of Medical Oncology, Imperial College, London, UK

^*^Corresponding Authors

**Analysis of DNA copy number data:** Copy number data were analyzed to identify the segments on the chromosome that were significantly amplified or deleted for each patient. Gene copy number aberrations were defined after circular binary segmentation using the DNACopy software in Bioconductor. We defined amplifications (deletions) as values greater (smaller) than 3 standard deviations (SD) of the mean of the middle 50% of the segmented log2 values, respectively. Random probes that did not map to any chromosome and low quality probes which generated less than twice the background signal were filtered out. Amplification, deletion and normal status of each gene was represented by +1,-1 and 0, respectively. Averages of these representative numbers were calculated for each gene from each subtype. Genes with variable copy number changes between patients or cell lines (amplification in some while deletion in others) within a particular subgroup will have an averages close to 0. Genes were ranked based on their averages and only genes at the top and bottom of the list were selected as amplified or deleted genes for a subgroup. These genes were used as seed genes for network growth. Due to its higher resolution, the Andre *et al.* CGH data was used for defining seed genes and network building in both patient data sets (Supplemental Table S2). Genes with significant copy number changes from the Neve *et al.* dataset (Supplemental Table S2) was used as seed genes for the cell line data set.

**Analysis of gene expression data:** Microarray data were median-polished and log-transformed for further analysis. Each of these studies used the same Affymetrix U133A gene chip platform for expression analysis. Probe annotation for the microarray data sets were obtained from the Affymetrix web site.

**siRNA validation experiments:** All cell lines were tested for Mycoplasma contamination and growth curves under standard culture conditions were established. We optimized the transfection conditions for each cell line separately using 3 different types of negative controls including cells grown in regular OptiMEM media (Invitrogen Inc), cells grown in the presence of transfection reagents, and cells transfected with control siRNA including 5 different siGENOME-1,-2,-3,-4 constructs and 4 different ON-TARGETplus siRNA constructs (Dharmacon). Three different siRNA positive controls (40 nM final concentration) were used that targeted the PLK1 (polo-like kinase 1), KIFF11 (kinesine family member 11), COPB2 (coatomer protein beta2) genes and siCONTROL TOX to assess transfection efficiency. We assessed 8 different transfection reagents for efficacy and toxicity (DharmaFect-1,-2-3-4, Xtreme Gene, HiPerect, RNAiMax and siPORT NeoFX). Cell viability was determined 96 hours after transfection using the Cell Titer Blue cell viability assay (Promega, Madison, WI). The optimal transfection conditions were defined as the most effective transfection reagent and the best negative and positive siRNA controls that were established for each cell line separately. The most effective transfection condition for each cell line was defined as the experimental conditions that maximized the Z-factor that is computed as Z=1-(3 x SSD/R) where R is the dynamic range of the assay (i.e. the absolute difference between mean cell viability for a given negative control and positive control) and SSD is the sum of the standard deviations for the positive and negative control assays. A Z-factor closest to 1.0 was considered the best and the corresponding experimental variables were defined as the optimal transfection condition for a given cell line. After finding the optimal conditions for each cell line, the full siRNA screen was performed for all 44 target genes. Cells were added to 384-well plates that contained the target siRNAs and the cell line-specific negative and positive control siRNA constructs and incubated at 37^o^C for 96 hours. The final concentration of all siRNA constructs is 40 nM per well in 50μl total volume. Cell seeding density varied from cell line to cell line depending on population doubling time, the goal was to seed cells in a density that yielded about 70-90% confluence in the control wells at the time of read out at 96 hours. Each cell line screen was performed in 3 parallel replicates. A control plate, including only replicates of positive and negative siRNA controls and cells grown in OptiMEM alone, was inserted after every 10 test plates to assess stability of assay read out. Cell viability was calculated as follows; median value of the absorbance measured in empty wells that contain media alone was subtracted from absorbance readings of all other wells and individual readings of each test wells were divided by the median value of negative siRNA controls in a given plate and multiplied by 100 to derive percent viability. Average percent viability across the 3 replicate plates is reported for each siRNA construct. Unequal variance t-test was used to assess significant decrease in viability compared to controls.

**Driver networks with seed genes from Chin et al. CNA data:** We used seed genes from Andre et al. for identifying driver-networks for both of the patient datasets (Chin et al. and Andre et al.). We took this approach because, Andre et al. dataset was from a higher resolution array and had shown increased expression-CNA correlation compared to Chin et al. However, we were curious to test, to what extent the driver networks will be different if seed genes were used from Chin et al. Hence, we followed the same steps and used the same criteria of differential expression and correlation, as mentioned in the materials and methods section, to identify the seed genes from Chin et al. dataset. The distribution of average CNA score and CNA-expression correlation are provided in the Supplemental Table 2. The driver networks created based on both CNA and expression data from Chin et al. dataset is provided in the Supplemental Fig 2. Overlaps of these driver-networks with the ones explained in the main text (Fig 2) are also highlighted. The sizes of the driver networks and the numbers of overlapping genes are listed in Supplemental Table 3. Corresponding to all three subtypes, we observed a significant overlap between the two driver-networks (between Supplemental Fig 2A-C and Fig 2A-C). We also found that most of the biological conclusions, as discussed in the main text (Fig 2), holds true for these driver-networks as well. For example, in case of ER+ subtype, *ESR1* gene was found as the central gene of the driver-network (Supplemental Fig 2A). Apoptosis and autophagy pathway genes were also members of the driver network. In TN driver-network, *EGFR* gene was the central node (Supplemental Fig 2C). Genes associated with EMT were also enriched in TN subnetwork.
